# Supplementary material for: Inequalities in healthcare disruptions during the COVID-19 pandemic: evidence from 12 UK population-based longitudinal studies
Source: BMJ Open. 2022 Oct 13;12(10):e064981. doi: 10.1136/bmjopen-2022-064981 (PMC9561494; doi:10.1136/bmjopen-2022-064981)
Supplement: Supplementary data [file bmjopen-2022-064981supp001.pdf]

Supplementary File 1: Meta-analysis summary restricted to representative studies

Note: ALSPAC, GS, TwinsUK and BiB

excluded. **Summary of results**

		Any healthcare disruption				Appointments				Prescription/Medication				Procedures/surgery			
		OR	Lower CI	Upper CI	I^2%	OR	Lower CI	Upper CI	I^2%	OR	Lower CI	Upper CI	I^2%	OR	Lower CI	Upper CI	I^2%
**Sex**																	
Female vs. Male	Unadjusted[ǂ]	1.27	1.19	1.36	0	1.29	1.18	1.42	5.66	1.39	0.90	2.14	73.3	1.24	1.13	1.37	0
	**Basic adjustment**	**1.34**	**1.15**	**1.57**	**65.33**	**1.36**	**1.25**	**1.47**	**0**	**1.37**	**0.86**	**2.16**	**74.9**	**1.27**	**1.12**	**1.43**	**11.85**
	Full adjustment	1.34	1.15	1.56	61.89	1.34	0.94	1.91		1.99	0.77	5.12		1.21	1.01	1.44	
**Age**																	
16-24y vs 45-54y	Unadjusted	0.50	0.41	0.62		0.43	0.34	0.54		0.65	0.42	1.02		0.48	0.34	0.68	
	**Basic adjustment**	**0.49**	**0.39**	**0.60**		**0.42**	**0.33**	**0.52**		**0.62**	**0.39**	**0.97**		**0.47**	**0.33**	**0.66**	
	Full adjustment	0.47	0.37	0.61		no information				no information				no information			
25-34y vs 45-54y	Unadjusted	0.71	0.58	0.86		0.65	0.53	0.80		0.97	0.66	1.44		0.78	0.57	1.07	
	**Basic adjustment**	**0.70**	**0.58**	**0.85**		**0.64**	**0.52**	**0.79**		**0.97**	**0.65**	**1.43**		**0.77**	**0.56**	**1.06**	
	Full adjustment	0.77	0.63	0.94		no information				no information				no information			
35-44y vs 45-54y	Unadjusted	0.74	0.63	0.88		0.70	0.58	0.83		0.83	0.58	1.18		0.88	0.69	1.12	
	**Basic adjustment**	**0.74**	**0.63**	**0.87**		**0.69**	**0.58**	**0.82**		**0.83**	**0.58**	**1.18**		**0.87**	**0.68**	**1.11**	
	Full adjustment	0.86	0.73	1.03		no information				no information				no information			
55-64y vs 45-54y	Unadjusted	1.40	1.23	1.59	0	1.37	1.19	1.58	0	0.75	0.27	2.07	67.12	1.51	1.26	1.80	0
	**Basic adjustment**	**1.42**	**1.25**	**1.61**	**0**	**1.39**	**1.21**	**1.60**	**0**	**0.80**	**0.30**	**2.09**	**64.1**	**1.52**	**1.28**	**1.80**	**0**
	Full adjustment	1.21	1.06	1.40	0	1.04	0.48	2.25		0.52	0.16	1.68		1.37	0.93	2.01	

65-74y vs 45-54y	Unadjusted	1.72	1.51	1.96	0	1.57	1.21	2.04	16.91	0.76	0.31	1.86	59.63	1.93	1.63	2.30	0
	**Basic adjustment**	**1.78**	**1.56**	**2.02**	**0**	**1.67**	**1.42**	**1.97**	**2.81**	**0.85**	**0.38**	**1.91**	**52.96**	**1.98**	**1.67**	**2.34**	**0**
	Full adjustment	1.35	1.14	1.58	0	1.01	0.42	2.43		1.41	0.34	5.89		1.55	1.05	2.30	
75y+ vs 45-54y	Unadjusted	1.97	1.68	2.31	1.58	1.87	1.56	2.24	0	0.89	0.59	1.35	6.53	2.10	1.46	3.02	66.45
	**Basic adjustment**	**2.06**	**1.76**	**2.41**	**0**	**1.97**	**1.64**	**2.35**	**0**	**0.98**	**0.68**	**1.42**	**0**	**2.14**	**1.57**	**2.91**	**55.32**
	Full adjustment	1.38	1.13	1.70	0.00	1.07	0.44	2.61		1.26	0.39	4.02		1.75	1.17	2.62	
**Ethnicity**																	
Non-White vs White*	Unadjusted	0.96	0.82	1.12	0	1.02	0.72	1.46	44.43	1.02	0.39	2.67	84.87	0.90	0.71	1.14	0
	**Basic adjustment**	**1.23**	**1.05**	**1.44**	**0**	**1.25**	**0.87**	**1.81**	**48.35**	**1.06**	**0.42**	**2.67**	**83.18**	**1.16**	**0.91**	**1.47**	**0**
	Full adjustment	1.10	0.94	1.29	0	1.39	0.61	3.20		2.04	0.70	5.98		0.96	0.63	1.48	
Black vs White	Unadjusted	1.22	0.91	1.65	0	1.02	0.53	1.94	48.54	0.49	0.07	3.52	85.41	0.87	0.58	1.31	0
	**Basic adjustment**	**1.47**	**1.08**	**1.98**	**0**	**1.18**	**0.57**	**2.44**	**59.36**	**0.50**	**0.08**	**3.36**	**84**	**1.03**	**0.68**	**1.55**	**0**
	Full adjustment	1.20	0.92	1.58	0	0.88	0.18	4.22		0.37	0.04	3.11		0.87	0.41	1.82	
East Asian vs White	Unadjusted	0.82	0.38	1.73		0.79	0.35	1.80		0.97	0.47	1.97		1.38	0.47	4.02	
	**Basic adjustment**	**1.04**	**0.53**	**2.06**		**1.03**	**0.49**	**2.16**		**1.04**	**0.52**	**2.09**		**1.80**	**0.65**	**4.99**	
	Full adjustment	1.01	0.60	1.68		no information				no information				no information			
Mixed vs White	Unadjusted	1.13	0.82	1.57	0	1.27	0.49	3.29	77.57	1.53	0.90	2.60	0	1.12	0.70	1.80	0
	**Basic adjustment**	**1.38**	**0.88**	**2.17**	**34.69**	**1.47**	**0.59**	**3.67**	**75.14**	**1.67**	**0.98**	**2.86**	**0**	**no information**			
	Full adjustment	1.36	0.88	2.11	24.01	no information				0.93	0.10	8.48		0.85	0.32	2.21	
South Asian vs White	Unadjusted	0.76	0.58	1.01	29.78	0.84	0.56	1.25	38.69	0.80	0.17	3.77	93.25	0.70	0.45	1.09	28.01
	**Basic adjustment**	**1.02**	**0.84**	**1.24**	**0**	**1.05**	**0.84**	**1.31**	**0**	**0.83**	**0.18**	**3.76**	**92.41**	**0.90**	**0.64**	**1.26**	**0**
	Full adjustment	0.95	0.72	1.25	21.29	2.65	1.03	6.82		4.47	1.38	14.50		1.11	0.62	1.99	

Other Ethnicity vs White	Unadjusted	0.56	0.25	1.25	40.34	0.82	0.45	1.49	0	0.70	0.14	3.54	63.29	0.81	0.11	6.21	67.17
	**Basic adjustment**	**0.72**	**0.25**	**2.07**	**64.12**	**1.02**	**0.41**	**2.51**	**48.38**	**0.96**	**0.17**	**5.25**	**66.68**	**0.82**	**0.08**	**8.51**	**74.76**
	Full adjustment	0.72	0.25	2.02	64.08	no information				no information				no information			
**Education**																	
A-level/equivalent vs Higher education/Degree	Unadjusted	1.02	0.85	1.22	53.07	1.02	0.84	1.25	39.4	0.94	0.68	1.30	26.88	0.68	0.37	1.28	90.63
	**Basic adjustment**	**1.11**	**0.99**	**1.25**	**8.05**	**1.13**	**0.99**	**1.29**	**1.31**	**0.92**	**0.71**	**1.19**	**0**	**0.73**	**0.38**	**1.39**	**91.07**
	Full adjustment	0.98	0.85	1.12	21.18	0.98	0.59	1.63		3.39	1.04	11.09		1.05	0.83	1.32	
GCSE/equivalent vs Higher education/Degree	Unadjusted	0.96	0.84	1.10	36.2	0.96	0.84	1.11	19.4	0.95	0.69	1.30	39.93	1.01	0.89	1.14	0
	**Basic adjustment**	**0.94**	**0.79**	**1.12**	**55.76**	**0.91**	**0.73**	**1.13**	**53.46**	**0.96**	**0.68**	**1.35**	**45.45**	**1.04**	**0.92**	**1.19**	**0**
	Full adjustment	0.84	0.73	0.95	24.18	0.63	0.36	1.10		1.96	0.59	6.47		0.81	0.62	1.04	
<GCSE/equivalent vs Higher education/Degree	Unadjusted	1.13	0.89	1.43	72.27	1.06	0.83	1.36	61.17	1.22	0.77	1.94	58.91	1.38	1.21	1.58	0
	**Basic adjustment**	**1.12**	**0.96**	**1.30**	**33.28**	**1.04**	**0.85**	**1.27**	**34.32**	**1.25**	**0.88**	**1.78**	**27.88**	**1.20**	**1.04**	**1.38**	**0**
	Full adjustment	0.85	0.76	0.96	3.24	0.70	0.42	1.17		3.22	1.01	10.27		0.86	0.66	1.12	
**Occupational class**																	
Intermediate vs Managerial/Admin/Professional	Unadjusted	1.07	0.97	1.18	0	1.04	0.93	1.17	0	0.99	0.74	1.35	17.14	1.15	1.00	1.32	0
	**Basic adjustment**	**1.04**	**0.94**	**1.15**	**0**	**1.02**	**0.91**	**1.15**	**0**	**0.96**	**0.68**	**1.37**	**29.88**	**1.12**	**0.98**	**1.28**	**0**
	Full adjustment	0.97	0.88	1.08	0	1.13	0.67	1.90		0.74	0.21	2.59		1.05	0.84	1.31	
Manual/Routine vs Managerial/Admin/Professional	Unadjusted	1.13	0.99	1.29	29.12	1.06	0.94	1.18	0	1.30	1.00	1.68	9.9	1.13	0.91	1.41	33.75
	**Basic adjustment**	**1.20**	**1.09**	**1.32**	**0**	**1.15**	**1.03**	**1.30**	**0**	**1.35**	**1.01**	**1.81**	**16.85**	**1.20**	**1.05**	**1.37**	**0**
	Full adjustment	1.03	0.93	1.15	0	1.29	0.81	2.06		0.75	0.27	2.13		1.07	0.85	1.35	
Other social class vs	Unadjusted	1.36	0.90	2.06	92.46	1.40	1.01	1.94	79.32	2.03	1.25	3.29	73.7	1.55	0.90	2.68	87.81

Managerial/ Admin/Professional	**Basic adjustment**	**1.48**	**1.10**	**2.00**	**81.34**	**1.51**	**1.18**	**1.93**	**56.45**	**2.44**	**1.71**	**3.49**	**45.49**	**1.64**	**1.10**	**2.46**	**72.22**
	Full adjustment	1.19	0.99	1.42	44.84	1.39	0.80	2.42		4.12	1.43	11.82		0.94	0.69	1.27	

Basic adjustment: sex, age, and ethnicity (where available)
Full adjustment: sex, age, and ethnicity (where available) , education, occupational class, UK Nation (where appropriate), household composition, and pre-pandemic self-reported health.

Empty I^2% column indicates only one study included

*Binary variable including Black, East Asian, Mixed, South Asian, and other ethnicity in 'non-White'

Summary of stratified results

		Any healthcare disruption			
**Sex**		OR	Lower CI	Upper CI	I2%
Female vs. Male	Overall	1.34	1.15	1.57	65.33
	Not shielding	1.32	1.09	1.61	75.25
	Shielding	1.48	1.20	1.83	0
	16-24y	2.21	1.61	3.03	3.99
	25-34y	1.45	0.86	2.43	63.72
	35-44y	1.48	1.14	1.92	
	45-54	1.97	1.61	2.42	0
	55-64	1.16	1.02	1.32	0
	75+	1.03	0.80	1.32	42.24
**Age**		OR	Lower CI	Upper CI	I2%
16-24y vs 45-54y	Overall	0.49	0.39	0.60	
	Not shielding	0.50	0.40	0.62	
	Shielding	0.64	0.23	1.78	
25-34y vs 45-54y	Overall	0.70	0.58	0.85	
	Not shielding	0.71	0.58	0.87	
	Shielding	0.86	0.34	2.16	
35-44y vs 45-54y	Overall	0.74	0.63	0.87	
	Not shielding	0.76	0.64	0.90	
	Shielding	0.48	0.24	0.96	
55-64y vs 45-54y	Overall	1.42	1.25	1.61	0
	Not shielding	1.37	1.20	1.57	0
	Shielding	1.32	0.80	2.17	0
65-74y vs 45-54y	Overall	1.78	1.56	2.02	0
	Not shielding	1.67	1.46	1.91	0
	Shielding	1.33	0.82	2.15	0
75y+ vs 45-54y	Overall	2.06	1.76	2.41	0
	Not shielding	1.96	1.66	2.33	0
	Shielding	1.07	0.65	1.78	0
**Ethnicity**		OR	Lower CI	Upper CI	I2%
Non-White vs White*	Overall	1.23	1.05	1.44	0

	Not shielding	0.96	0.62	1.48	73.47
	Shielding	1.56	0.97	2.49	0
	16-24y	1.24	0.84	1.82	0
	25-34y	0.70	0.47	1.04	0
	35-44y	1.42	0.94	2.12	
	45-54	1.71	1.20	2.44	0
	55-64	1.20	0.87	1.66	0
	75+	1.28	0.67	2.45	0
Black vs White	Overall	1.47	1.08	1.98	0
	Not shielding	0.84	0.38	1.83	72.85
	Shielding	1.49	0.59	3.78	0
	16-24y	1.15	0.51	2.59	0
	25-34y	0.74	0.30	1.86	16.69
	35-44y	2.11	0.87	5.12	
	45-54	1.99	0.93	4.25	15.25
	55-64	1.74	1.03	2.95	0
	75+	1.23	0.42	3.56	0
East Asian vs White	Overall	1.04	0.53	2.06	
	Not shielding	1.04	0.52	2.11	
	Shielding				
	16-24y	0.01	0.00	0.05	
	25-34y	0.57	0.12	2.62	
	35-44y	1.55	0.69	3.48	
	45-54	1.62	0.42	6.18	
	55-64	0.90	0.36	2.21	
	75+				
Mixed vs White	Overall	1.38	0.88	2.17	34.69
	Not shielding	1.28	0.88	1.86	0
	Shielding	1.89	0.64	5.55	0
	16-24y	2.50	1.25	5.02	0
	25-34y	1.09	0.61	1.95	0
	35-44y	2.47	0.88	6.95	
	45-54	1.01	0.48	2.14	
	55-64	1.19	0.56	2.51	0
	75+	1.47	0.34	6.42	22.46
South Asian vs White	Overall	1.02	0.84	1.24	0
	Not shielding	0.92	0.64	1.34	42.86
	Shielding	1.30	0.72	2.36	0
	16-24y	0.98	0.62	1.53	13.95

	25-34y	0.43	0.26	0.72	2.58
	35-44y	0.91	0.58	1.42	
	45-54	2.55	0.59	10.92	86.27
	55-64	0.90	0.47	1.74	19
	75+	1.11	0.40	3.12	0
Other Ethnicity vs White	Overall	0.72	0.25	2.07	64.12
	Not shielding	0.63	0.20	1.95	62.21
	Shielding	0.19	0.01	4.52	
	16-24y	0.18	0.00	15.35	88.56
	25-34y	0.57	0.10	3.20	70.09
	35-44y	1.52	0.36	6.41	
	45-54	1.12	0.37	3.38	
	55-64	0.49	0.12	1.96	
	75+	4.18	0.35	50.04	
**Education**		OR	Lower CI	Upper CI	I2%
A-level/equivalent vs Higher education/Degree	Overall	1.11	0.99	1.25	8.05
	Not shielding	1.02	0.85	1.23	47.74
	Shielding	0.92	0.66	1.30	0
	16-24y	1.39	0.96	2.01	0
	25-34y	0.97	0.55	1.71	52.33
	35-44y	1.48	1.00	2.18	
	45-54	1.10	0.86	1.40	0
	55-64	0.99	0.76	1.29	44.12
	75+	0.77	0.57	1.05	0
GCSE/equivalent vs Higher education/Degree	Overall	0.94	0.79	1.12	55.76
	Not shielding	0.93	0.79	1.10	47.54
	Shielding	0.80	0.60	1.06	0
	16-24y	0.93	0.36	2.40	83.45
	25-34y	1.05	0.53	2.07	70.84
	35-44y	1.19	0.86	1.64	
	45-54	1.00	0.70	1.44	60.4
	55-64	1.06	0.91	1.24	0
	75+	0.88	0.59	1.31	54.52
<GCSE/equivalent vs Higher education/Degree	Overall	1.12	0.96	1.30	33.28
	Not shielding	1.01	0.83	1.23	50.08
	Shielding	0.86	0.63	1.18	8.77
	16-24y	0.79	0.38	1.61	46.71
	25-34y	1.31	0.61	2.81	62.99

	35-44y	0.87	0.56	1.36	
	45-54	1.32	0.85	2.06	61.45
	55-64	1.18	0.97	1.43	0
	75+	0.98	0.78	1.24	0
**Occupational class**		OR	Lower CI	Upper CI	I2%
Intermediate vs Managerial/Admin/Professional	Overall	1.04	0.94	1.15	0
	Not shielding	1.04	0.94	1.15	0
	Shielding	0.86	0.59	1.25	13.43
	16-24y	0.88	0.55	1.41	0
	25-34y	1.25	0.86	1.81	0
	35-44y	1.13	0.81	1.58	
	45-54	1.13	0.92	1.39	0
	55-64	0.92	0.77	1.11	0
	75+	1.02	0.76	1.37	0
Manual/Routine vs Managerial/Admin/Professional	Overall	1.20	1.09	1.32	0
	Not shielding	1.20	1.08	1.33	0
	Shielding	0.94	0.71	1.24	0
	16-24y	1.14	0.74	1.75	0
	25-34y	1.55	0.97	2.48	36.45
	35-44y	1.23	0.88	1.71	
	45-54	1.04	0.85	1.27	0
	55-64	1.14	0.95	1.37	0
	75+	1.29	0.98	1.70	0
Other social class vs Managerial/Admin/Professional	Overall	1.48	1.10	2.00	81.34
	Not shielding	1.44	1.10	1.89	73.49
	Shielding	0.92	0.38	2.22	82.67
	16-24y	1.01	0.34	2.95	79.64
	25-34y	2.09	1.40	3.13	0
	35-44y	2.16	1.34	3.48	
	45-54	2.05	0.98	4.29	85.15
	55-64	1.73	1.28	2.33	64.79
	75+	1.02	0.62	1.69	0

Adjusted for sex, age, and ethnicity (where available)

Empty I^2% column indicates only one study included

*Binary variable including Black, East Asian, Mixed, South Asian, and other ethnicity in 'non-White'
