# Supplementary material for: Inequalities in healthcare disruptions during the COVID-19 pandemic: evidence from 12 UK population-based longitudinal studies
Source: BMJ Open. 2022 Oct 13;12(10):e064981. doi: 10.1136/bmjopen-2022-064981 (PMC9561494; doi:10.1136/bmjopen-2022-064981)
Supplement: Supplementary data [file bmjopen-2022-064981supp002.pdf]

Supplementary File 2: Variable coding

Contents

Healthcare disruptions 2

Covariates 6

A note about shielding 11

Maddock J, *et al. BMJ Open* 2022; 12:e064981. doi: 10.1136/bmjopen-2022-064981

Healthcare disruptions

Study	*Question (exact wording)*	*Possible Answers*	*Recoding if needed*
*** PRESCRIPTION or MEDICATION ACCESS ***			
**MCS**<br>**NS**<br>**BCS 70**<br>**NCDS**<br>**NSHD**	Since the Coronavirus outbreak in March, have you had any difficulty obtaining any of your prescribed medication?	1=Yes; 2=No/Not applicable	= 1
**ALSPAC**	Not Available		
**USOC**	Q1: Still thinking about your situation now, have you been able to access the NHS services you need: Prescription medicine?<br>Q2: Still thinking about your situation now, have you been able to access the community health and social care services and support you need... Over the counter medications?	For both Q1 and Q2:<br>1=Yes; 2=No; 3=Not required	Q1=2 OR Q2=2
**ELSA**	Since the coronavirus outbreak, have you been able to get access to your regular medications?	1=Yes; 2=No; 3=No need	= 2
**GS**	How strongly do you agree with the following statements: Accessing and remembering to take my medication has become more difficult during the COVID-19 pandemic	From 1 (do not agree at all)<br>to 10 (agree very strongly)	=6/10
**TWINS UK**	Have you experienced any of the following as a result of COVID-19? Unable to access required medication	0= No; 1 = Yes	= 1
**BIB**	Q1: Have you or a member of your household needed to access pharmacy services since lockdown began?<br>Q2: If yes, did you receive the support you needed?	Q1. 0=No; 1=Yes<br>Q2. 0= No; 1=Yes; 2=Haven't tried	Q1=1 & Q2=0
*** PROCEDURES or SURGERIES ***			
**MCS**<br>**NS**<br>**BCS 70**<br>**NCDS**<br>**NSHD**	Q1: At the time of the Coronavirus outbreak in March, did you have an in-patient or out-patient appointment booked at a hospital for a consultation, investigation, treatment or surgery?<br>Q2: Have you now had your surgery?<br>Q3: Did your (last) surgery take place on the planned date or was it delayed?<br>Q4: Why has your surgery not taken place?	Q1. 1=Yes - for a consultation investigation or treatment; 2=Yes - for surgery; 3=No.<br>Q2. 1=Yes; 2=No.<br>Q3. 1=Surgery took place on the planned date; 2=Surgery was delayed.<br>Q4. 1=My surgery was postponed and	Q1=2 & Q2=1 & Q3=2<br>*OR*<br>Q1=2 & Q2=2 &<br>Q4=(1 OR 3)

		has not yet happened; 2=My surgery was not postponed, but it hasn't happened yet; 3=My surgery was cancelled	
**ALSPAC GS**	Q1: Have you had any medical treatments or appointments that have had to be cancelled or postponed during the COVID-19 pandemic? For example, hospital referral, non-emergency surgery, cancer, treatment, etc.<br>Q2: What types of medical treatments or appointments were cancelled or postponed?	Q1. 1=Yes; 2=No.<br>Q2. a -- surgery: 1=Yes; -9=Not applicable<br>b -- cancer treatment: 1=Yes; -9=Not applicable<br>c -- dialysis: 1=Yes; -9=Not applicable	Q1=1 & Q2 (a OR b OR c)=1
**USOC**	Q1: [since previous survey] have you had or been waiting for NHS treatment? Please select all that apply.<br>Q2: Has your treatment plan(s) been changed in any way?	Q1. 1=Yes, tests/consultations planned or in progress; 2=Yes, operation or procedure planned; 3=Yes, targeted therapy, chemotherapy or radiotherapy planned or in progress; 4=Yes, other treatment planned; 5=No<br>Q2. 1=Yes, consultations/treatments cancelled or postponed by NHS; 2=Yes, alternative treatment provided; 3=Yes, I cancelled or postponed treatment; 4=No, treatment continuing as planned	Q1=2/4 & Q2=1/3
**ELSA**	Since the coronavirus outbreak, have you had a hospital operation or treatment cancelled?	1.Yes; 2.No	=1
**TWINS UK**	Not Available		
**BIB**	Not Available		
*** APPOINTMENTS ***			
**MCS NS BCS 70 NCDS NSHD**	Q1: At the time of the Coronavirus outbreak in March, did you have an in-patient or out-patient appointment booked at a hospital for a consultation, investigation, treatment or surgery?<br>Q2: Have you now had your in/ out-patient hospital appointment for a consultation, investigation or treatment?<br>Q3: Did your (last) appointment take place on the planned date or was it delayed?<br>Q4: Why has your in-/out-patient hospital appointment for a consultation, investigation or treatment not taken place?	Q1. 1=Yes - for a consultation investigation or treatment; 2=Yes - for surgery; 3=No.<br>Q2. 1=Yes; 2=No.<br>Q3. 1=Appointment took place on the planned date; 2=Appointment was delayed.<br>Q4. 1=My appointment was postponed	Q1=1 & Q2=1 & Q3=2 OR<br>Q1=1 & Q2=2 & Q4=(1 OR 3)

Maddock J, *et al. BMJ Open* 2022; 12:e064981. doi: 10.1136/bmjopen-2022-064981

		and has not yet happened; 2=My appointment was not postponed, but it hasn't happened yet; 3=My appointment was cancelled	
**ALSPAC GS**	Q1. Have you had any medical treatments or appointments that have had to be cancelled or postponed during the COVID-19 pandemic? For example, hospital referral, non-emergency surgery, cancer, treatment, etc.<br>Q2. What types of medical treatments or appointments were cancelled or postponed?	Q1. 1=Yes; 2=No.<br>Q2= d -- GP referral: 1=Yes; -9=Not applicable<br>e -- Hospital referral: 1=Yes; -9=Not applicable<br>f -- Routine clinical appointment: 1=Yes; -9=Not applicable<br>g -- Cancer testing: 1=Yes; -9=Not applicable<br>h -- Cancer screening: 1=Yes; -9=Not applicable	Q1=1 & Q2(d OR e OR f OR g OR h)=1
**USOC**	Thinking about your situation now, have you been able to access the NHS services you need to help manage your condition(s) over the last 4 weeks?<br>Q1: GP or primary care practice staff?<br>Q2: Hospital or clinic outpatient?<br>Q3: Hospital or clinic inpatient?<br>Q4: [since previous survey] have you had or been waiting for NHS treatment? Please select all that apply.<br>Q5: Has your treatment plan(s) been changed in any way?	Q1-3. 1=Yes, in person; 2=(Q1 & Q2 only) Yes, online or by phone only; 3=No, not able to access; 4=No, decided not to seek help at this time/cancelled; 5=Alternative treatment provided; 6=Not required<br>Q4. 1=Yes, tests/consultations planned or in progress; 2=Yes, operation or procedure planned; 3=Yes, targeted therapy, chemotherapy or radiotherapy planned or in progress; 4=Yes, other treatment planned; 5=No<br>Q5. 1=Yes, consultations/treatments cancelled or postponed by NHS; 2=Yes, alternative treatment provided; 3=Yes, I cancelled or postponed treatment; 4=No, treatment continuing as planned	Q1|Q2|Q3=(3 OR 4 OR 5)<br>OR<br>Q4=1 AND Q5=(1 OR 2 OR3)
**ELSA**	Q1: Since the coronavirus outbreak, have you wanted to see or talk to a GP?<br>Q2: Have you been able to see or talk to a GP?	Q1: 1=Yes; 2=No<br>Q2: 1=Yes; 2=No; 3=I did not attempt to contact them 4.I did not need to contact them	Q1= & Q2=2
**TWINS UK**	Not Available		

Maddock J, *et al. BMJ Open* 2022; 12:e064981. doi: 10.1136/bmjopen-2022-064981

**BIB**	Q1: Have you or a member of your household needed to access<br>-- (1) your doctor (GP) or nurse<br>-- (2) NHS111<br>-- (3) Health emergency services (A&E)<br>-- (4) A specialist (consultant) doctor or specialist clinic (hospital outpatient) appointment since lockdown began?<br>Q2: If yes, were you able to access (1, 2, 3, or 4)?	Q1. 0=No; 1=Yes<br>Q2. 0= No; 1=Yes; 2=Haven't tried	Q1= & Q2=0

Covariates

Variables	*Study*	*Options*	*Recoding if needed*
*** Sex * 0=Male; 1=Female**			
	**All**	0=Male; 1=Female	
*** Ethnicity * 0=White; 1=South East Asian; 2=Other Asian; 3=Black; 4=Mixed; 5=Other Non-White**			
	**MCS**	1=White; 2=Mixed; 3=Indian; 4=Pakistani; 5=Bangladeshi; 6=Other Asian; 7=Black Caribbean; 8=Black African; 9=Other Black; 10=Chinese; 11=Other ethnic group	1=0, 2=4, 3-5=1, 6 & 10=2, 7-9=3, 11=5
	**NS**	1=White; 2=Mixed; 3=Indian; 4=Pakistani; 5=Bangladeshi; 6=Black Caribbean; 7=Black African; 8=Other	1=0, 2=4, 3-5=1, 6-7=3, 8=5
	**BCS70**	Not Available	
	**NCDS**	Not Available	
	**NSHD**	Not Available	
	**ALSPAC**	G0 (Parents) 1=White; 2=Black Caribbean; 3=Black African; 4=Other black; 5=Indian; 6=Pakistani; 7=Bangladeshi; 8=Chinese; 9=Other<br>G1 (Children) 1=White; 2=Mixed/Multiple Ethnic group; 3=Asian; 4=Black/African/Caribbean/Black British; 5=Arab or Other	G0: 1=0; 5/7=1, 8=2, 2/4=3, 9=5<br>G1: 1=0; 3=2, 4=3, 2=4
	**USOC**	1=White British; 2=Irish (White); 3=Gypsy or Irish Traveller (white); 4=Any other white background; 5=White and black Caribbean (mixed); 6=White and black African (mixed); 7=White and Asian (mixed); 8=Any other mixed background; 9=Indian (Asian or Asian British); 10=Pakistani (Asian or Asian British); 11=Bangladeshi (Asian or Asian British); 12=Chinese (Asian or Asian British); 13=Any other Asian background (Asian or Asian British); 14=Caribbean (Black or Black British); 15=African (Black or Black British); 16=Any other Black background (Black or Black British); 17=Arab (other Ethnic group); 97=Any other ethnic group	1-4=0, 5-8=4, 9-11=1, 12-13=2, 14-16=3, 17-97=5
	**ELSA**	1.White; 2=Mixed ethnic group; 3=Black; 4=Black British; 5=Asian; 6=Asian British	1=0; 2=4; 3/4=3; 5/6=1
	**GS**	1=White Scottish; 2=White English; 3=White Welsh; 4=White N. Irish; 5=White Irish; 6=White Gypsy/Irish traveller; 7=White Polish; 8=Any other white; 9=Asian/British Asian - Indian; 10=Asian/British Asian - Pakistani; 11=Asian/British Asian - Bangladeshi; 12=Asian/British Asian - Chinese; 13=Any other Asian background; 14=Black or Black British - African; 15=Black or Black British - Caribbean; 16=Any other Black/African/Caribbean background; 17=Arab or Arab British; 18=Mixed - White and Black Caribbean; 19=Mixed - White and Black African; 20=Mixed - White and Asian; 21=Any other Mixed/Multiple ethnic background; 22=Any other ethnic group	1/8=0, 9/11=1, 12/13=2, 14/16=3, 18/21=4, 17&22=5

Maddock J, *et al. BMJ Open* 2022; 12:e064981. doi: 10.1136/bmjopen-2022-064981

**TWINS UK**	1=White- English, Welsh, Scottish, Northern Irish, Irish; 2=White- Other white background; 3=Mixed/multiple ethnic groups - White and Black Caribbean; 4=Mixed/multiple ethnic groups - White and Black African; 5=Mixed/multiple ethnic groups - White and Asian; 6=Mixed/multiple ethnic groups - Other mixed/ multiple ethnic background; 7=Asian/Asian British- Indian; 8=Asian/Asian British - Pakistani; 9=Asian/Asian British - Bangladeshi; 10=Asian/Asian British - Chinese; 11=Asian/Asian British - Other Asian background; 12=Black/Black British - African; 13=Black/Black British - Caribbean; 14=Black/Black British - Other Black Background; 15=Middle-Eastern; 16=Other ethnic group	1/2=0; 10=1; 7/9|11=2; 12/14=3; 3/6=4; 15/16=5
**BIB**	BiB: 1=White British; 2=White other; 3=Mixed-White and Black; 4=Mixed-White and South Asian; 5=Black; 6=Indian; 7=Pakistani; 8=Bangladeshi; 9=Other<br>BIBBS: 1=White British; 2=White Irish; 3=Pakistani; 4=Indian; 5=Bangladeshi; 6=White Polish; 7=White Slovakian; 8=White Romanian; 9=White Czech; 10=Other White; 11=White Gypsy/Roma/Irish traveller; 12=Chinese; 13=African; 14=Caribbean; 15=Mixed White/Black Caribbean; 16=Mixed White/Black African; 17=Mixed White/Asian; 18=Do not wish to answer; 19=Other	BiB: 1/2=0; 6/8=1; 5=3; 3/4=4; 9=5<br>BiBBs: 1/2=0; 6/11=0; 3/5=1; 13/14=3; 15/18=4; all other options=5
*** Education * 0= Degree; 1=A-Level; 2=GCSE; 3=Low or None**		
**MCS<br>NS<br>BCS 70<br>NCDS**	0=None; 1=Nvq1; 2=Nvq2; 3=Nvq3; 4=Nvq4; 5=Nvq5<br>*parent's education for MCS	0/1 = 0 2=1 3=2 4/5=3
**NSHD**	0=None attempted; 1.=Vocational course, proficiency only; 2=Sub GCE or sub Burnham C; 3=GCE 'O' level or Burnham C; 4=GCE 'A' Level or Burnham B; 5=Burnham A2; 6= 1st Degree or graduate equivalent; 7= Higher degree, Masters; 8= Higher degree, doctorate; 9=Unknown	6|7|8=0; 4|5=1; 3=2; 0|1|2|9=3
**ALSPAC**	1=Degree; 2=A levels/AS levels or equivalent; 3=O levels; 4=Vocational; 5=CSE<br>*parent's education for G1 (Children)	1=0; 2=1; 3=2; 4/5=3
**USOC**	1.Higher degree 2. 1st degree or equivalent 3. Diploma in Higher Education 4. Teaching qualification (not PGCE) 5. Nursing or other medical qualification 6. Other higher degree 7. A-Level 8. Welsh baccalaureate 9. International baccalaureate 10. AS Level 11. Scottish Highers 12. Certificate of 6th year studies 13. GCSE/O-Level 14. Certificate of secondary education 15. Standard or lower 16. Other school certificate 96. No qualifications	1-6=0, 7-12=1, 13-16=2, 96=3
**ELSA**	1=Nvq4/nvq5/degree or equivalent; 2=Higher Education below degree; 3=Nvq3/GCE A level equivalent; 4=Nvq2/GCE O level equivalent; 5=Nvq1/CSE other grade equivalent; 6=Foreign/other; 7=No qualification	1=0; 2/3=1; 4=2; 5/7=3
**GS**	1=No qualifications; 2=Other (please specify); 3=School leavers certificate; 4=CSEs or equivalent; 5=Standard grade, National 4 or 5, O levels, GCSEs or equivalent; 6=Higher grade, A levels, AS levels or equivalent; 7=NVQ or HND or HNC or equivalent; 8=Other professional or technical qualification; 9=Undergraduate degree; 10=Postgraduate degree	9|10=0; 6|7|8 =1; 5=2; <5=3

	**TWINS UK**	1=No qualification; 2=NVQ1/SVQ1; 3=O-level/GCSE/NVQ2/SVQ2/Scottish intermediate; 4=Scottish Higher, NVQ3, City and Guilds, Pitman; 5=A-level, Scottish Advanced Higher; 6=Higher vocational training (e.g. Diploma, NVQ4, SVQ4); 7=Undergraduate degree; 8=Postgraduate degree (e.g. Masters or PhD), NVQ5, SVQ5	6/8=0; 4/5=1; 3=2; 1/2=3
	**BIB**	1=<5 GCSE equivalent; 2=5 GCSE equivalent; 3=A-level equivalent; 4=Higher than A-level; 5=Other; 6=Don't know; 7=Foreign unknown	4=0; 3=1; 5/7=2; 1=3; missing=1
*** Occupational Social Class * 1=Managerial/Admin/Professional; 2=Intermediate; 3=Manual/routine; 4=Other**			
NS-SEC: National Statistics Socioeconomic Classification. RGSC: Registrat General's Social Class. ONS SOC: Office of National Statistics Standard Occupational Classification			
	**MCS**<br>**NS**<br>**BCS 70**<br>**NCDS**	[NS-SEC] 1=Higher managerial and professional; 2=Lower managerial and professional; 3=Intermediate occupations; 4=Small employers and own account workers; 5=Lower supervisory and technical; 6=Semi-routine occupations; 7=Routine occupations; 8=Never worked and long-term unemployed<br>*parent's occupational social class for MCS	2=1; 3-4=2; 5-7=3; 8=4
	**NSHD**	[RGSC] 1=I Professional; 2=II Managerial and Technical; 3=IIINM Skilled non-manual; 4=IIIM Skilled manual; 5=IV Partly skilled; 6=V Unskilled;	2=1; 3/5=2; 6=3;
	**ALSPAC**	[RGSC] 1=I Professional; 2=II Managerial and Technical; 3=IIINM Skilled non-manual; 4=IIIM Skilled manual; 5=IV Partly skilled; 6=V Unskilled; 7=Armed Forces<br>*parent's occupational social class for G1 (Children)	2=1; 3/5=2; 6=3; 7=4
	**USOC**	[NS-SEC] 1=Higher managerial and professional; 2=Lower managerial and professional; 3=Intermediate occupations; 4=Small employers and own account workers; 5=Lower supervisory and technical; 6=Semi-routine occupations; 7=Routine occupations; 8=Never worked and long-term unemployed	2=1; 3-4=2; 5-7=3; 8=4
	**ELSA**	[NS-SEC] -3=Incomplete/No job info; 1=Higher and Lower managerial/ professional; 2=Intermediate occupations; 3=Routine and manual occupations; 99=Other	99=4; -3=4
	**GS**	[ONS SOC] 1=Managers, directors, senior officials; 2=Associate professional and technical occupations; 3=Administrative and secretarial occupations; 4=Skilled trades occupations; 5=Sales and customer service occupations; 6=Process, plant and machine operatives; 7=Elementary (unskilled) occupations; 8=Never worked	1/3=1; 4/5=2; 6/7=3; 8=4
	**TWINS UK**	Not Available	
	**BIB**	1=Modern professional occupations; 2=Clerical and intermediate occupations; 3=Senior managers or administrators; 4=Technical and craft occupations; 5=Semi-routine manual and service occupations; 6=Routine manual and service occupations; 7=Middle or junior managers; 8=Traditional professional occupations;<br>9=Self-employed; 10=Student/in training; 11=Does not work-long term unemployed/sick; 12=Don't know<br>*Based on either own class (80.7%) or partner's (19.3%)	3=1; 8=1; 4=2; 7=2; 5/6=3; all other options=4

*** Living Arrangement * 1=Alone; 2=With partner/spouse only; 3=With partner/spouse and child(ren); 4=With child(ren), without partner/spouse; 5=Any other living arrangement<br><br>OR * Partnership Status * 1=Married/Partnered; 0=Not married/partnered**			
	**MCS<br>NS<br>BCS 70<br>NCDS**	Who do you currently live with? 1. Husband/Wife/Cohabiting Partner2. Children (including adult children, step-children, adopted children, foster children or any other children you consider yourself parent to) 3. Parent or Parent-in-law (including step-parent or adoptive parent) 4. Grandparent 5. Grandchild 6. Sibling 7. Other relative 8. Friend / unrelated sharer 9. Other	1 = Husband/Wife/Cohabit ing Partner; 0 = Other
	**NSHD**	Who do you currently live with? (Options include Husband/Wife/Cohabiting Partner)	1= Partner in HH 0= No partner in household
	**ALSPAC**	NA	NA
	**USOC**	Derived from Household Grid	0=partner present; 1=Single
	**ELSA**	IF respondents live with other people, they are asked for each person "what is this person's relationship to you". Options include "1. Husband/wife/partner"	1=Partner in HH 0=No partner in HH
	**GS**	1. Married/ Civil partnership 2. In a relationship, living together 3. In a relationship, not living together 4. Single 5. Separated 6. Divorced 7. Widowed 8. Other	1-3=1 4-8 = 0
	**TWINS UK**	Single, never married (1); Single, divorced or widowed (2); In a relationship/married but living apart (3); In a relationship/married and cohabiting (4)	1, 2 = 0; 3, 4 = 1
	**BIB**	What is your current relationship status? 0=do not wish to answer; 1=single; 2=married; 3=not married but in a relationship	1=0; 2/3=1
*** Shielding Status * 1=Advised to Shield; 0=Not advised to shield**			
	**MCS<br>NS<br>BCS 70<br>NCDS<br>NSHD**	Did you at any time receive a letter or text message from the NHS or Chief Medical Officer saying that you have been identified as someone at risk of severe illness if you catch Coronavirus, because you have an underlying disease or health condition? 1=Yes; 2=No	2=0
	**ALSPAC**	Not Available	

**USOC**	Have you received a letter, text or email from the NHS or Chief Medical Officer saying that you have been identified as someone at risk of severe illness if you catch coronavirus, because you have an underlying disease or health condition? 1=Yes; 2=No	2=0
**ELSA**	Have you been contacted by the NHS or your GP and advised that you are vulnerable and at risk of severe illness if you catch coronavirus (Covid-19), and should stay at home at all times and avoid any face-to-face contact? 1=Yes; 2=No	2=0
**GS**	Have you been contacted by letter or text message to say you are at sever risk from COVID-19 due to and underlying health condition and should be shielding? 1=Yes; 2=No	2=0
**TWINS UK**	Have you received a letter or text message over the past few months to say you are at high risk from COVID-19 due to an underlying health condition, and should be 'shielding'? 1=Yes; 2=No	2=0
**BIB**	Have you been advised by a health professional that you are high risk or vulnerable and should self-isolate for 12 weeks to protect yourself from coronavirus? 0=No; 1=Yes	

*** Pre-Pandemic Self-Assessed Health * 1=Good/Very Good/Excellent; 0=Fair/Poor**		
**MCS**<br>**NS**<br>**BCS 70**<br>**NCDS**<br>**NSHD**	In general, in the 3 months before the Coronavirus outbreak would you say your health was … 1=Excellent; 2=Very Good; 3=Good; 4=Fair; 5=Poor	1/3=1; 4/5=0
**ALSPAC (G0 & G1)**	(2020) Do you have a history of diabetes (A), obesity (B) or asthma (C)?	1 if A & B & C==0<br>0 if A | B | C==1
**USOC**	(2018/19) In general, would you say your health is… 1=Excellent; 2=Very Good; 3=Good; 4=Fair; 5=Poor	1/3=1; 4/5=0
**ELSA**	(2018/19) Would you say your health is… 1=Excellent; 2=Very Good; 3=Good; 4=Fair; 5=Poor	1/3=1; 4/5=0
**GS**	NA	
**TWINS UK**	(2020) In general, would you say your health is… 1=Excellent; 2=Very Good; 3=Good; 4=Fair; 5=Poor	1/3=1; 4/5=0
**BIB**	(2016 - 2020) In general, would you say your health is… 1=Excellent; 2=Very Good; 3=Good; 4=Fair; 5=Poor	1/3=1; 4/5=0

A note about shielding

Who had to shield?

Initially 1.5 million, increasing to 2.2 million, people in the UK were identified as clinically extremely vulnerable (CEV) by their GP. They were sent a letter asking them to shield – not go out – for at least 12 weeks until the end of June. This timeframe was extended, and on 1st August, CEV individuals in England, Scotland and Northern Ireland were told that shielding had been paused. In Wales shielding continued until 16th August.

Who was classed as clinically extremely vulnerable?

People falling into the clinically extremely vulnerable group include:

- Solid organ transplant recipients
- People with cancer who are undergoing active chemotherapy or radical radiotherapy for lung cancer
- People with cancers of the blood or bone marrow such as leukaemia, lymphoma or myeloma who are at any stage of treatment
- People having immunotherapy or other continuing antibody treatments for cancer
- People having other targeted cancer treatments which can affect the immune system, such as protein kinase inhibitors or PARP inhibitors (which prevent cancer cells from repairing)
- People who have had bone marrow or stem cell transplants in the last 6 months, or who are still taking immunosuppression drugs
- People with severe respiratory conditions including all cystic fibrosis, severe asthma and severe chronic obstructive pulmonary disease (COPD)
- People with rare diseases and inborn errors of metabolism that significantly increase the risk of infections such as Severe combined immunodeficiency (SCID) or homozygous sickle cell)
- People on immunosuppression therapies sufficient to significantly increase risk of infection
- Women who are pregnant with significant heart disease, congenital or acquired.

Source:
https://web.archive.org/web/20200330181117/https://www.gov.uk/government/publications/covid-19-guidance-on-social-distancing-and-for-vulnerable-people/guidance-on-social-distancing-for-everyone-in-the-uk-and-protecting-older-people-and-vulnerable-adults
