# Supplementary material for: Inequalities in healthcare disruptions during the COVID-19 pandemic: evidence from 12 UK population-based longitudinal studies
Source: BMJ Open. 2022 Oct 13;12(10):e064981. doi: 10.1136/bmjopen-2022-064981 (PMC9561494; doi:10.1136/bmjopen-2022-064981)
Supplement: Supplementary data [file bmjopen-2022-064981supp003.pdf]

Supplementary File 3: Meta-analysis results

Contents

Summary of results 3
Summary of stratified results 6
Any healthcare disruption 10
Sex 10
Unadjusted 10
Basic adjustment 11
Full adjustment 12
Occupational class 13
Unadjusted 13
Basic adjustment 15
Full adjustment 17
Education 19
Unadjusted 19
Basic adjustment 21
Full adjustment 23
Age 25
Unadjusted 25
Basic adjustment 27
Full adjustment 29
Ethnicity 31
Unadjusted 31
Basic adjustment 34
Full adjustment 38
Appointments 41
Sex 41
Unadjusted 41
Basic adjustment 42
Full adjustment 43
Occupational class 44
Unadjusted 44
Basic adjustment 46
Full adjustment 48
Education 50
Unadjusted 50
Basic adjustment 52
Full Adjustment 54
Age 56
Unadjusted 56
Basic adjustment 58
Full adjustment 60
Ethnicity 62
Unadjusted 62
Basic adjustment 65
Full adjustment 68
Prescription/Medication access 71
Sex 71
Unadjusted 71
Basic adjustment 72
Full adjustment 73
Occupational class 74

Maddock J, *et al. BMJ Open* 2022; 12:e064981. doi: 10.1136/bmjopen-2022-064981

Unadjusted 74
Basic adjustment 76
Full adjustment 78
Education 80
Unadjusted 80
Basic adjustment 82
Full adjustment 84
Age 86
Unadjusted 86
Basic adjustment 89
Full adjustment 91
Ethnicity 93
Unadjusted 93
Basic adjustment 96
Full adjustment 99
Procedures/surgery 102
Sex 102
Unadjusted 102
Basic adjustment 103
Full adjustment 104
Occupational class 105
Unadjusted 105
Basic adjustment 107
Full adjustment 109
Education 111
unadjusted 111
Basic adjustment 113
Full adjustment 115
Age 117
Unadjusted 117
Basic adjustment 120
Full adjustment 122
Ethnicity 123
Unadjusted 123
Basic adjustment 126
Full adjustment 129
Any healthcare disruption stratified by shielding status 131
Sex 131
Occupational class 132
Education 136
Age 140
Ethnicity 143
Any healthcare disruption stratified by age 148
Sex 148
Occupational class 149
Education 152
Ethnicity 155

Summary of results

		Any healthcare disruption				Appointments				Prescription/Medication				Procedures/surgery			
		OR	Lower CI	Upper CI	I²%	OR	Lower CI	Upper CI	I²%	OR	Lower CI	Upper CI	I²%	OR	Lower CI	Upper CI	I²%
**Sex**																	
Female vs. Male	Unadjusted	1.26	1.14	1.39	58.51	1.30	1.12	1.52	73.54	1.33	1.00	1.77	75.15	1.12	0.93	1.36	60.28
	**Basic adjustment**	**1.27**	**1.15**	**1.40**	**53.11**	**1.33**	**1.17**	**1.52**	**60**	**1.27**	**0.94**	**1.74**	**77.98**	**1.15**	**0.97**	**1.37**	**47.79**
	Full adjustment	1.30	1.15	1.46	44.89	1.41	1.10	1.82	0.00	1.18	0.50	2.77	59.02	1.09	0.77	1.55	18.15
**Age**																	
16-24y vs 45-54y	Unadjusted	0.77	0.41	1.47	71.12	0.55	0.26	1.20	55.5	1.17	0.34	4.05	86.43	0.48	0.34	0.68	
	**Basic adjustment**	**0.76**	**0.39**	**1.46**	**71.95**	**0.55**	**0.24**	**1.23**	**58.53**	**1.09**	**0.33**	**3.67**	**85.53**	**0.47**	**0.33**	**0.66**	
	Full adjustment	0.85	0.32	2.24	70.28	0.89	0.18	4.36		4.71	1.40	15.86		no information			
25-34y vs 45-54y	Unadjusted	0.87	0.71	1.07	51.9	0.71	0.53	0.96	72.25	1.23	0.75	2.03	74.54	0.78	0.59	1.02	0
	**Basic adjustment**	**0.85**	**0.70**	**1.04**	**47.62**	**0.67**	**0.57**	**0.77**	**0**	**1.16**	**0.68**	**1.96**	**76.95**	**0.77**	**0.58**	**1.03**	**0**
	Full adjustment	0.92	0.74	1.15	43.99	1.21	0.66	2.22		1.18	0.52	2.67	0	no information			
35-44y vs 45-54y	Unadjusted	0.93	0.74	1.16	67.57	0.77	0.66	0.89	23.09	1.06	0.75	1.51	57.05	0.99	0.71	1.36	42.96
	**Basic adjustment**	**0.92**	**0.74**	**1.15**	**68.24**	**0.78**	**0.65**	**0.92**	**35.96**	**1.04**	**0.74**	**1.47**	**56.72**	**1.03**	**0.67**	**1.58**	**61.87**
	Full adjustment	1.03	0.81	1.29	59.88	1.03	0.59	1.78		1.25	0.62	2.52	0	1.36	0.93	2.00	0
55-64y vs 45-54y	Unadjusted	1.16	0.98	1.38	63.54	1.33	1.21	1.47	0	0.79	0.57	1.08	61.08	1.17	0.65	2.10	91.47
	**Basic adjustment**	**1.18**	**0.99**	**1.39**	**64.04**	**1.35**	**1.22**	**1.49**	**0**	**0.79**	**0.57**	**1.10**	**65.97**	**1.18**	**0.66**	**2.10**	**89.83**
	Full adjustment	1.17	1.05	1.29	0	1.55	0.62	3.91	49.89	0.85	0.48	1.52	0	no information			
65-74y vs 45-54y	Unadjusted	1.36	1.11	1.67	75.24	1.61	1.46	1.78	0	0.73	0.48	1.11	79.59	1.93	1.67	2.23	0
	**Basic adjustment**	**1.39**	**1.13**	**1.72**	**77.16**	**1.65**	**1.49**	**1.82**	**0**	**0.75**	**0.49**	**1.16**	**80.63**	**1.95**	**1.68**	**2.26**	**0**
	Full adjustment	1.33	1.19	1.49	0	1.98	0.48	8.10	73.94	1.15	0.65	2.04	0	1.57	1.07	2.31	0
75y+ vs 45-54y	Unadjusted	1.45	0.92	2.29	90.02	1.83	1.59	2.12	0	0.66	0.46	0.94	47.94	2.05	1.59	2.64	28.83
	**Basic adjustment**	**1.50**	**0.93**	**2.39**	**91.23**	**1.89**	**1.65**	**2.17**	**0**	**0.69**	**0.47**	**1.01**	**53.79**	**2.07**	**1.66**	**2.59**	**17.97**
	Full adjustment	1.16	0.86	1.58	62.75	1.07	0.44	2.61		0.78	0.39	1.57	1.68	1.75	1.17	2.62	
**Ethnicity**																	

3

Non-White vs White*	Unadjusted	1.02	0.89	1.18	10.29	0.95	0.72	1.25	56.69	1.36	0.79	2.33	79.57	0.89	0.71	1.12	0
	**Basic adjustment**	**1.19**	**1.05**	**1.35**	**0**	**1.10**	**0.86**	**1.39**	**42.54**	**1.32**	**0.82**	**2.12**	**71.12**	**1.14**	**0.91**	**1.44**	**0**
	Full adjustment	1.09	0.96	1.25	0	1.15	0.83	1.61	0	1.97	1.08	3.62	0	1.68	0.36	7.76	62.98
Black vs White	Unadjusted	1.16	0.87	1.55	0	0.95	0.54	1.64	33.95	0.63	0.13	3.06	81.71	0.87	0.58	1.29	0
	**Basic adjustment**	**1.38**	**1.03**	**1.84**	**0**	**1.01**	**0.54**	**1.92**	**49.83**	**0.64**	**0.14**	**2.87**	**80**	**1.03**	**0.68**	**1.55**	**0**
	Full adjustment	1.15	0.86	1.53	4.01	0.81	0.23	2.83	0	0.37	0.04	3.11		0.87	0.41	1.82	
East Asian vs White	Unadjusted	0.97	0.56	1.68	0	0.61	0.28	1.30	12.51	2.34	0.39	14.15	88.19	1.23	0.48	3.15	0
	**Basic adjustment**	**1.13**	**0.67**	**1.90**	**0**	**0.80**	**0.39**	**1.64**	**17.46**	**1.95**	**0.53**	**7.24**	**79.39**	**1.61**	**0.61**	**4.22**	**0**
	Full adjustment	0.96	0.60	1.51	0	1.19	0.26	5.51		no information				no information			
Mixed vs White	Unadjusted	1.05	0.79	1.38	0	1.02	0.55	1.89	64.07	1.51	0.99	2.30	0	1.05	0.66	1.67	0
	**Basic adjustment**	**1.24**	**0.86**	**1.78**	**27.61**	**1.22**	**0.69**	**2.15**	**56.82**	**1.34**	**0.82**	**2.18**	**16.29**	**1.34**	**0.76**	**2.36**	**20.19**
	Full adjustment	1.25	0.88	1.77	15.86	1.61	0.80	3.22	51.21	0.93	0.10	8.48		0.85	0.32	2.21	
South Asian vs White	Unadjusted	0.85	0.61	1.18	64.69	0.92	0.59	1.42	67.42	0.99	0.36	2.72	89.3	0.68	0.45	1.04	18
	**Basic adjustment**	**1.05**	**0.84**	**1.32**	**28.25**	**1.11**	**0.88**	**1.39**	**12.68**	**0.98**	**0.38**	**2.54**	**87.94**	**0.89**	**0.64**	**1.24**	**0**
	Full adjustment	0.93	0.67	1.30	57.84	1.03	0.29	3.63		2.81	1.19	6.63	11.86	1.11	0.62	1.99	
Other Ethnicity vs White	Unadjusted	0.79	0.46	1.34	28.89	0.91	0.58	1.45	0	1.23	0.56	2.67	25.72	0.84	0.20	3.48	44.62
	**Basic adjustment**	**0.90**	**0.49**	**1.63**	**44.27**	**1.07**	**0.68**	**1.68**	**0**	**1.45**	**0.81**	**2.60**	**0**	**0.95**	**0.23**	**4.03**	**52.56**
	Full adjustment	0.82	0.45	1.50	35.18	1.97	1.08	3.62	0.00	3.74	0.39	35.91		no information			
**Education**																	
A-level/equivalent vs Higher education/Degree	Unadjusted	1.04	0.91	1.17	58.16	1.03	0.91	1.17	42.88	0.94	0.77	1.15	21.54	0.84	0.55	1.29	88.56
	**Basic adjustment**	**1.08**	**0.97**	**1.20**	**38.7**	**1.07**	**0.99**	**1.16**	**0**	**1.02**	**0.82**	**1.28**	**27.13**	**0.87**	**0.58**	**1.30**	**85.92**
	Full adjustment	1.01	0.92	1.11	0	0.97	0.76	1.25	0	1.61	0.63	4.12	59.22	1.03	0.82	1.29	0
GCSE/equivalent vs Higher education/Degree	Unadjusted	0.99	0.87	1.14	62.23	1.03	0.95	1.12	0	0.98	0.75	1.27	53.81	1.03	0.91	1.16	0
	**Basic adjustment**	**1.00**	**0.87**	**1.14**	**59.18**	**1.01**	**0.91**	**1.12**	**15.22**	**1.04**	**0.77**	**1.39**	**62.34**	**1.05**	**0.93**	**1.18**	**0**
	Full adjustment	0.91	0.81	1.02	48.6	0.86	0.66	1.12	0	1.01	0.63	1.61	1.57	0.82	0.64	1.05	0
<GCSE/equivalent vs Higher education/Degree	Unadjusted	1.06	0.88	1.28	76.55	1.09	0.92	1.29	52.88	1.07	0.74	1.54	69.78	1.45	1.28	1.64	0
	**Basic adjustment**	**1.05**	**0.91**	**1.21**	**53.17**	**1.01**	**0.86**	**1.18**	**42.42**	**1.17**	**0.82**	**1.67**	**63.42**	**1.26**	**1.11**	**1.44**	**0**
	Full adjustment	0.87	0.75	1.00	34.17	0.90	0.54	1.50	58.54	1.17	0.39	3.49	60.69	1.53	0.34	6.85	71.25
**Occupational class**																	

Intermediate vs Managerial/Admin/Professional	Unadjusted	1.08	1.00	1.16	0	1.01	0.91	1.14	22.79	1.09	0.83	1.42	41.19	1.19	1.05	1.34	0
	**Basic adjustment**	**1.07**	**0.99**	**1.15**	**0**	**1.01**	**0.93**	**1.10**	**0**	**1.10**	**0.88**	**1.38**	**26.19**	**1.16**	**1.03**	**1.31**	**0**
	Full adjustment	1.00	0.92	1.08	0	1.01	0.78	1.30	0	0.73	0.34	1.61	0	0.78	0.42	1.47	48.08
Manual/Routine vs Managerial/Admin/Professional	Unadjusted	1.13	1.03	1.23	12.12	1.04	0.90	1.20	36.16	1.38	1.16	1.64	0	1.11	0.91	1.35	25.25
	**Basic adjustment**	**1.17**	**1.08**	**1.27**	**0**	**1.07**	**0.93**	**1.23**	**28.46**	**1.36**	**1.11**	**1.67**	**8.93**	**1.17**	**1.03**	**1.33**	**0**
	Full adjustment	1.02	0.93	1.12	0	1.10	0.84	1.44	6.05	0.51	0.18	1.43	23.27	0.92	0.56	1.50	20.63
Other social class vs Managerial/Admin/Professional	Unadjusted	1.47	1.02	2.13	89.12	1.41	1.08	1.84	66.23	2.16	1.30	3.57	76.1	1.71	0.94	3.10	87.16
	**Basic adjustment**	**1.51**	**1.12**	**2.04**	**79.69**	**1.46**	**1.16**	**1.84**	**47.81**	**2.45**	**1.72**	**3.50**	**45.5**	**1.81**	**1.17**	**2.80**	**73.85**
	Full adjustment	1.19	1.00	1.43	39.12	1.30	0.85	1.99	0.00	1.42	0.13	15.78	76.18	0.94	0.69	1.27	

Basic adjustment: sex, age, and ethnicity (where available)

Full adjustment: sex, age, and ethnicity (where available) education, occupational class, UK Nation (where appropriate), household composition, and pre-pandemic self-reported health.

Empty I^2% column indicates only one study included

*Binary variable including Black, East Asian, Mixed, South Asian, and other ethnicity in 'non-White'

Summary of stratified results

		Any healthcare disruption			
**Sex**		OR	Lower CI	Upper CI	I2%
Female vs. Male	Overall	1.27	1.15	1.40	53.11
	Not shielding	1.26	1.12	1.43	61.12
	Shielding	1.37	1.15	1.63	0
	16-24y	2.22	1.63	3.02	0
	25-34y	1.56	1.30	1.87	0
	35-44y	1.51	1.23	1.86	0
	45-54	1.72	1.35	2.18	36.61
	55-64	1.09	0.92	1.30	59.58
	75+	1.08	0.90	1.30	20
**Age**		OR	Lower CI	Upper CI	I2%
16-24y vs 45-54y	Overall	0.76	0.39	1.46	71.95
	Not shielding	0.79	0.40	1.56	70.32
	Shielding	0.64	0.23	1.78	
25-34y vs 45-54y	Overall	0.85	0.70	1.04	47.62
	Not shielding	0.86	0.70	1.06	43.4
	Shielding	1.09	0.61	1.95	0
35-44y vs 45-54y	Overall	0.92	0.74	1.15	68.24
	Not shielding	0.95	0.74	1.21	68.26
	Shielding	0.68	0.34	1.34	47.41
55-64y vs 45-54y	Overall	1.18	0.99	1.39	64.04
	Not shielding	1.21	1.02	1.43	53.82
	Shielding	1.24	0.87	1.77	0
65-74y vs 45-54y	Overall	1.39	1.13	1.72	77.16
	Not shielding	1.44	1.20	1.72	64.1
	Shielding	1.11	0.79	1.56	0
75y+ vs 45-54y	Overall	1.50	0.93	2.39	91.23
	Not shielding	1.61	1.17	2.22	79.38
	Shielding	0.83	0.51	1.37	32.84
**Ethnicity**		OR	Lower CI	Upper CI	I2%
Non-White vs White*	Overall	1.19	1.05	1.35	0
	Not shielding	1.06	0.86	1.31	41.46
	Shielding	1.62	1.08	2.43	0
	16-24y	1.30	0.89	1.89	0
	25-34y	0.92	0.65	1.29	36.48
	35-44y	1.31	1.01	1.71	0
	45-54	1.61	1.16	2.22	0
	55-64	1.13	0.85	1.50	0

	75+	1.28	0.67	2.45	0
Black vs White	Overall	1.38	1.03	1.84	0
	Not shielding	0.80	0.43	1.49	58.06
	Shielding	1.60	0.67	3.83	0
	16-24y	1.15	0.51	2.59	0
	25-34y	0.82	0.40	1.68	0
	35-44y	1.91	0.81	4.48	0
	45-54	1.99	0.93	4.25	15.25
	55-64	1.69	1.00	2.84	0
	75+	1.23	0.42	3.56	0
East Asian vs White	Overall	1.13	0.67	1.90	0
	Not shielding	0.95	0.54	1.68	0
	Shielding		no information		
	16-24y	0.01	0.00	0.05	
	25-34y	0.62	0.20	1.92	0
	35-44y	1.63	0.80	3.32	0
	45-54	1.75	0.54	5.64	0
	55-64	0.96	0.43	2.15	0
	75+		no information		
Mixed vs White	Overall	1.24	0.86	1.78	27.61
	Not shielding	1.18	0.85	1.62	0
	Shielding	1.85	0.71	4.77	0
	16-24y	2.50	1.25	5.02	0
	25-34y	1.26	0.79	2.02	0
	35-44y	1.15	0.23	5.69	73.12
	45-54	0.92	0.46	1.87	0
	55-64	1.06	0.53	2.11	0
	75+	1.47	0.34	6.42	22.46
South Asian vs White	Overall	1.05	0.84	1.32	28.25
	Not shielding	0.98	0.75	1.28	35.03
	Shielding	1.44	0.87	2.38	0
	16-24y	0.98	0.62	1.53	13.95
	25-34y	0.80	0.38	1.71	74.73
	35-44y	1.11	0.80	1.55	10.1
	45-54	1.67	0.43	6.48	82
	55-64	0.82	0.44	1.56	14.81
	75+	1.11	0.40	3.12	0
Other Ethnicity vs White	Overall	0.90	0.49	1.63	44.27
	Not shielding	0.85	0.45	1.62	43.11
	Shielding	0.75	0.11	4.96	10.15
	16-24y	0.18	0.00	15.35	88.56
	25-34y	0.80	0.31	2.08	49.28

	35-44y	1.41	0.58	3.40	0
	45-54	1.74	0.56	5.45	29.75
	55-64	0.77	0.27	2.22	0
	75+	4.18	0.35	50.04	
**Education**		OR	Lower CI	Upper CI	I2%
A-level/equivalent vs Higher education/Degree	Overall	1.08	0.97	1.20	38.7
	Not shielding	1.09	0.96	1.23	39.28
	Shielding	0.95	0.74	1.22	0
	16-24y	1.33	0.93	1.90	0
	25-34y	0.99	0.69	1.42	62.16
	35-44y	1.62	1.28	2.05	0
	45-54	1.13	0.96	1.34	0
	55-64	1.01	0.89	1.14	0
	75+	0.96	0.65	1.40	57.49
GCSE/equivalent vs Higher education/Degree	Overall	1.00	0.87	1.14	59.18
	Not shielding	0.99	0.84	1.17	64.95
	Shielding	0.80	0.62	1.04	0
	16-24y	0.94	0.49	1.81	64.06
	25-34y	1.24	0.80	1.94	69.1
	35-44y	1.26	0.97	1.63	0
	45-54	1.16	0.83	1.62	62.52
	55-64	1.03	0.91	1.17	0
	75+	0.92	0.65	1.30	35.17
<GCSE/equivalent vs Higher education/Degree	Overall	1.05	0.91	1.21	53.17
	Not shielding	1.02	0.88	1.19	46.14
	Shielding	0.87	0.68	1.11	0
	16-24y	0.77	0.47	1.28	11.51
	25-34y	0.99	0.67	1.45	42.2
	35-44y	1.03	0.74	1.43	0
	45-54	1.48	1.08	2.04	34.96
	55-64	1.20	1.03	1.41	0
	75+	0.96	0.78	1.20	0
**Occupational class**		OR	Lower CI	Upper CI	I2%
Intermediate vs Managerial/Admin/Professional	Overall	1.07	0.99	1.15	0
	Not shielding	1.07	0.98	1.16	0
	Shielding	0.87	0.65	1.16	7.88
	16-24y	0.92	0.60	1.41	0
	25-34y	1.04	0.84	1.29	10.79
	35-44y	1.28	0.92	1.78	46.81
	45-54	1.12	0.94	1.33	0
	55-64	1.01	0.86	1.19	22.02

Maddock J, *et al. BMJ Open* 2022; 12:e064981. doi: 10.1136/bmjopen-2022-064981

	75+	1.00	0.76	1.33	0
Manual/Routine vs Managerial/Admin/Professional	Overall	1.17	1.08	1.27	0
	Not shielding	1.18	1.07	1.29	0
	Shielding	0.93	0.71	1.21	0
	16-24y	1.15	0.77	1.71	0
	25-34y	1.11	0.80	1.55	50.55
	35-44y	1.24	0.95	1.63	0
	45-54	1.08	0.90	1.30	0
	55-64	1.16	1.00	1.35	0
	75+	1.27	0.96	1.67	0
Other social class vs Managerial/Admin/Professional	Overall	1.51	1.12	2.04	79.69
	Not shielding	1.48	1.04	2.09	83.37
	Shielding	0.89	0.39	2.07	78.87
	16-24y	1.02	0.46	2.26	58.35
	25-34y	1.85	1.29	2.64	0
	35-44y	1.44	0.55	3.80	68.27
	45-54	2.05	0.98	4.29	85.15
	55-64	1.65	1.21	2.27	60.63
	75+	1.02	0.62	1.69	0

Adjusted for sex, age, and ethnicity (where available)
Empty I^2% column indicates only one study included
*Binary variable including Black, East Asian, Mixed, South Asian, and other ethnicity in 'non-White'

Any healthcare disruption

Sex

Unadjusted

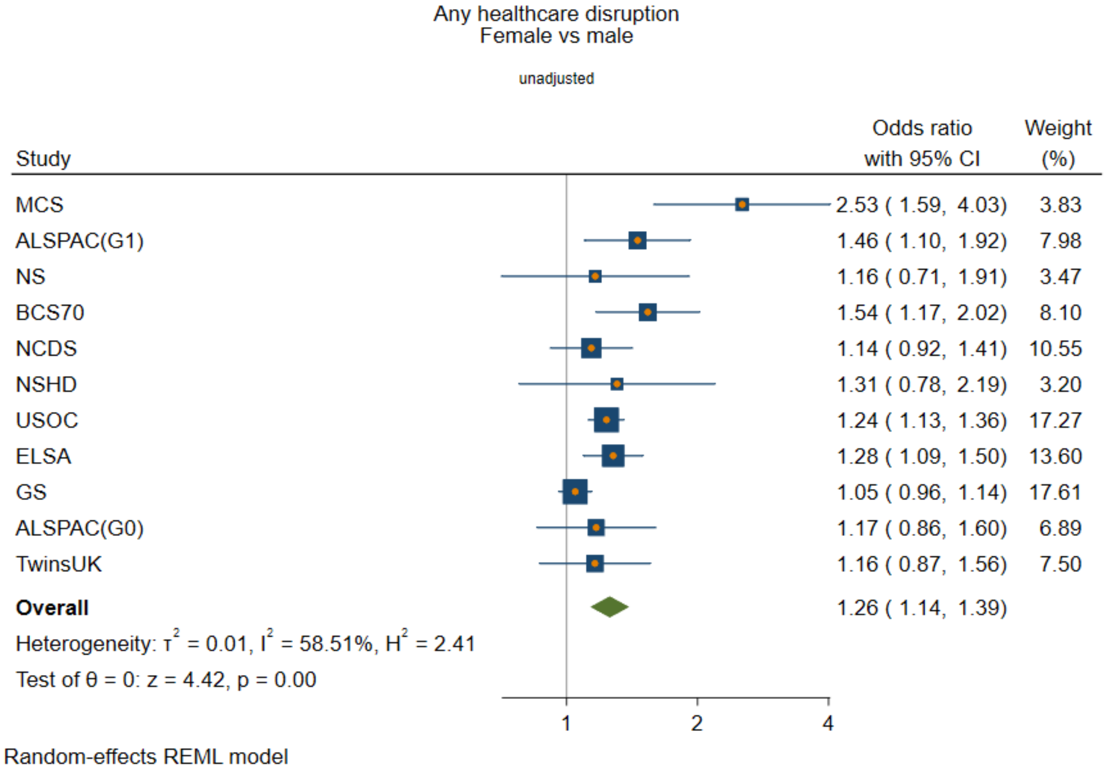

Basic adjustment

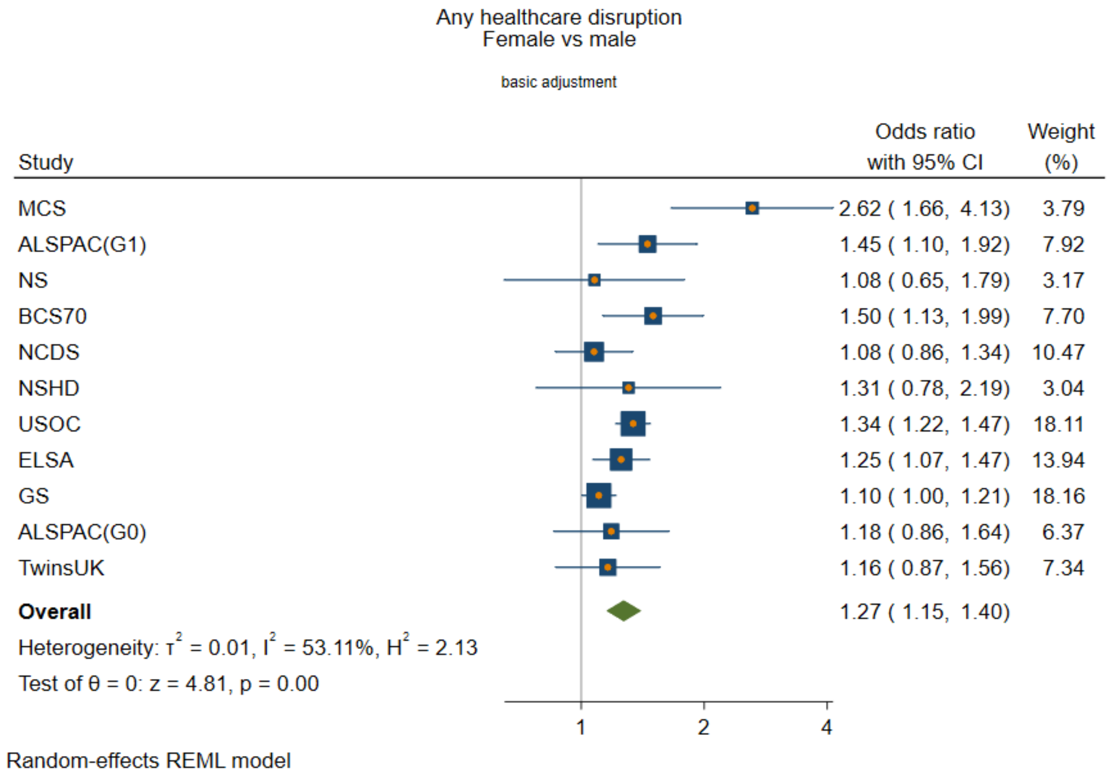

<u>Full adjustment</u>

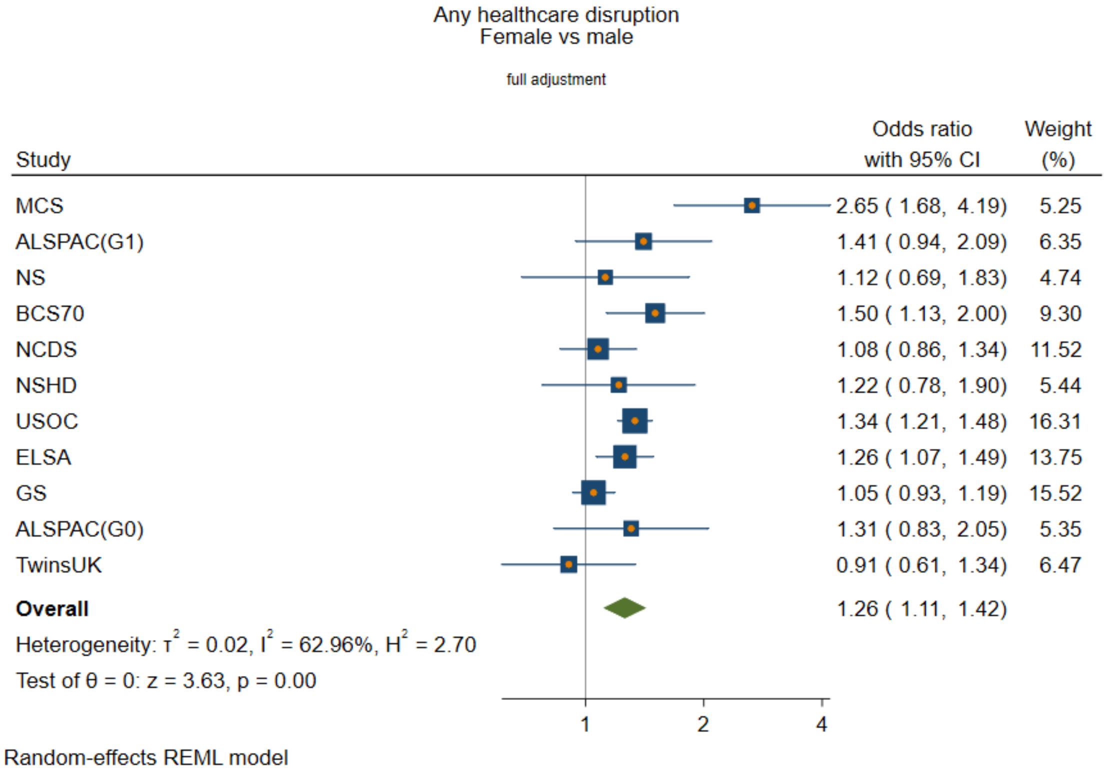

Occupational class
<u>Unadjusted</u>

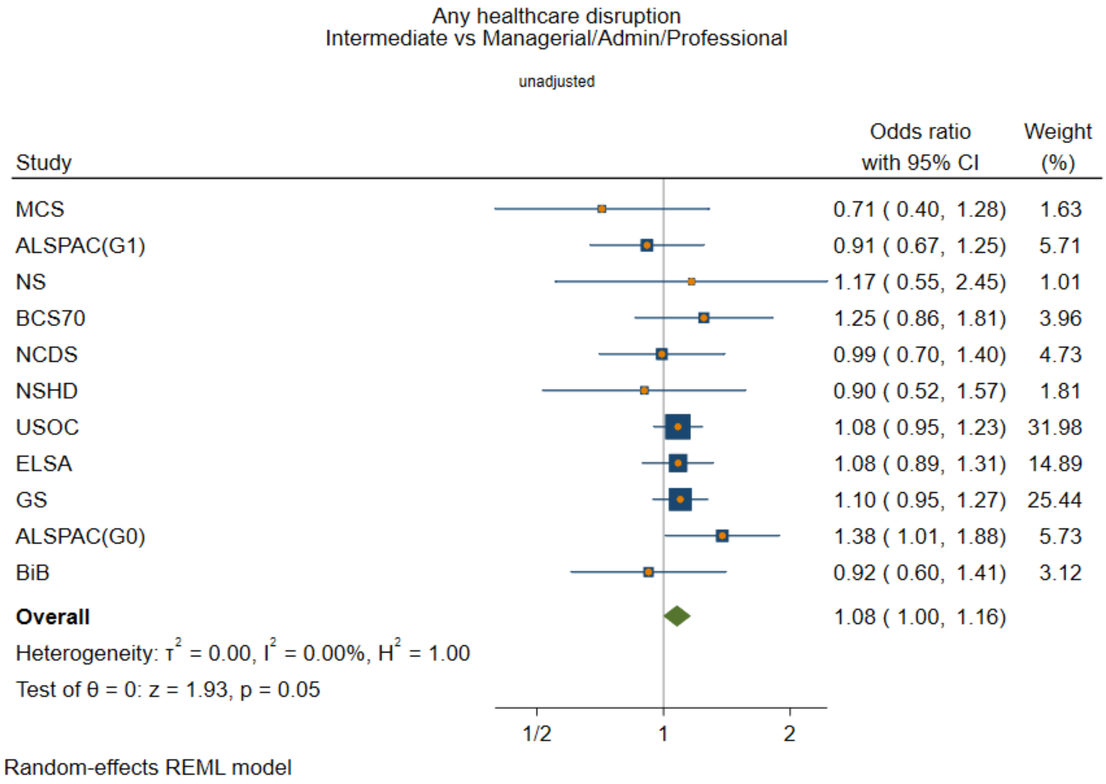

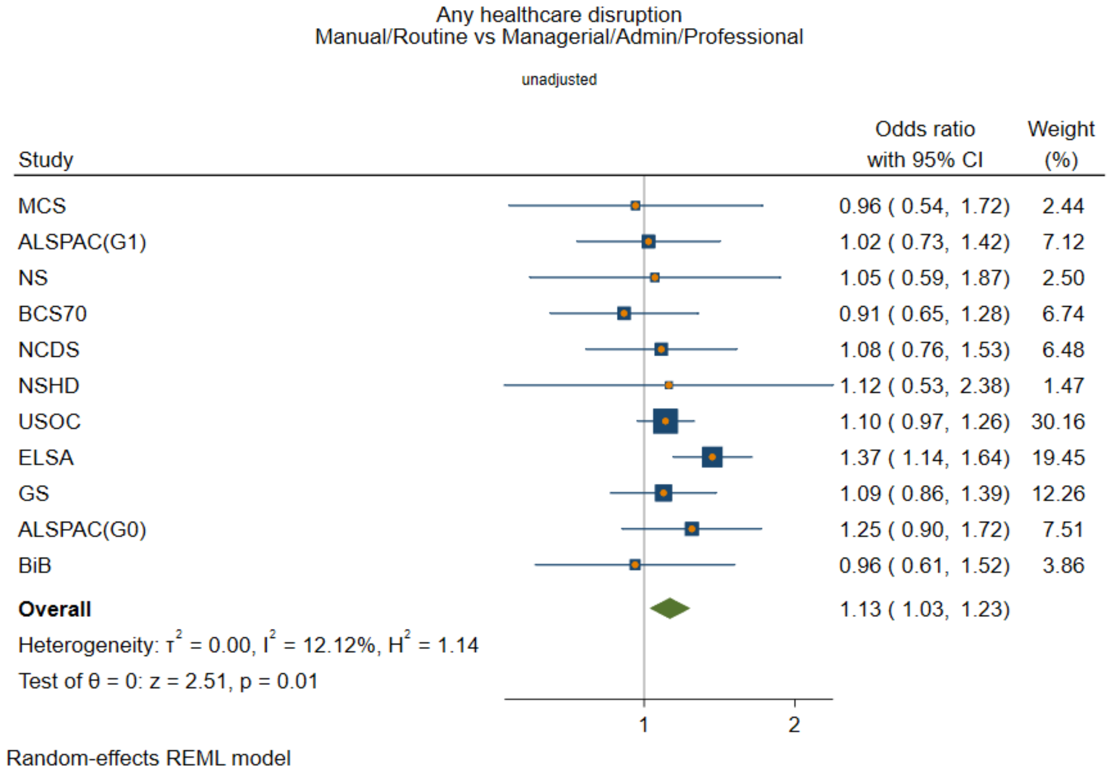

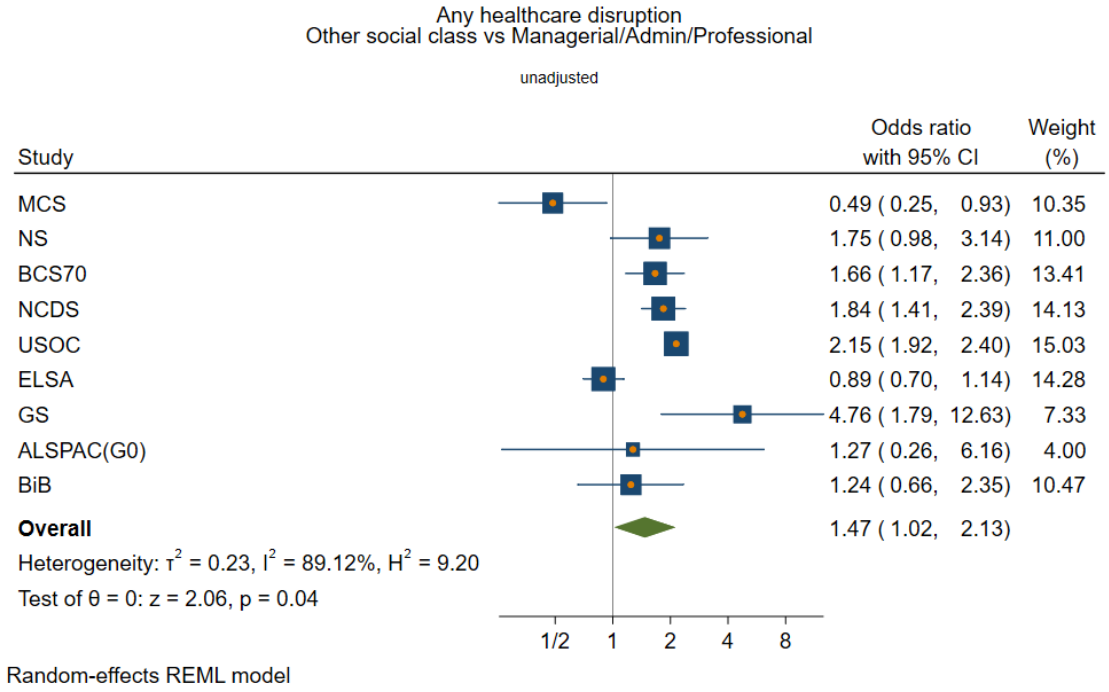

Maddock J, *et al. BMJ Open* 2022; 12:e064981. doi: 10.1136/bmjopen-2022-064981

Basic adjustment

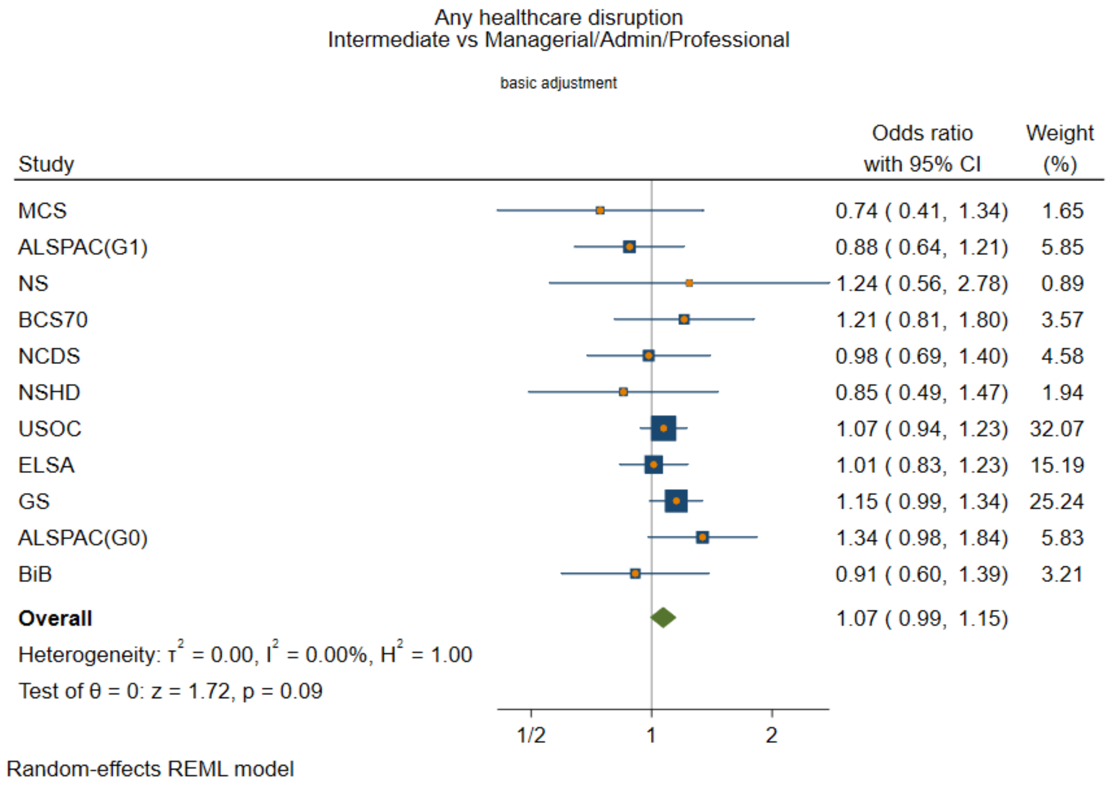

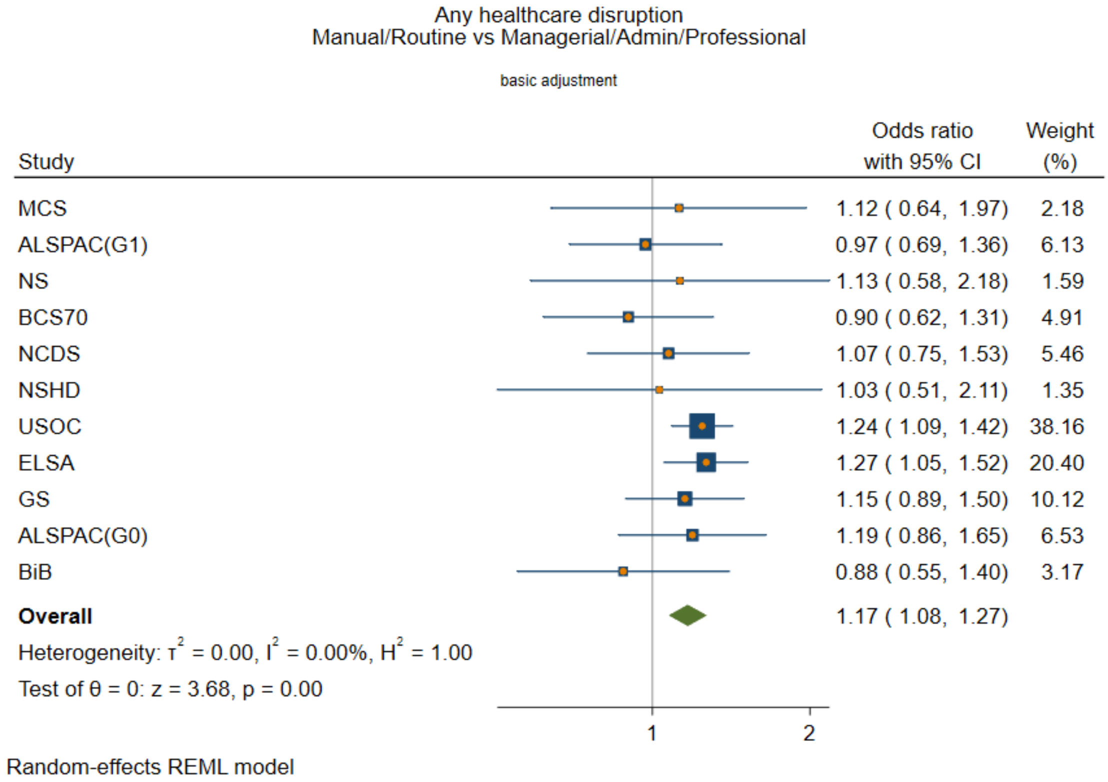

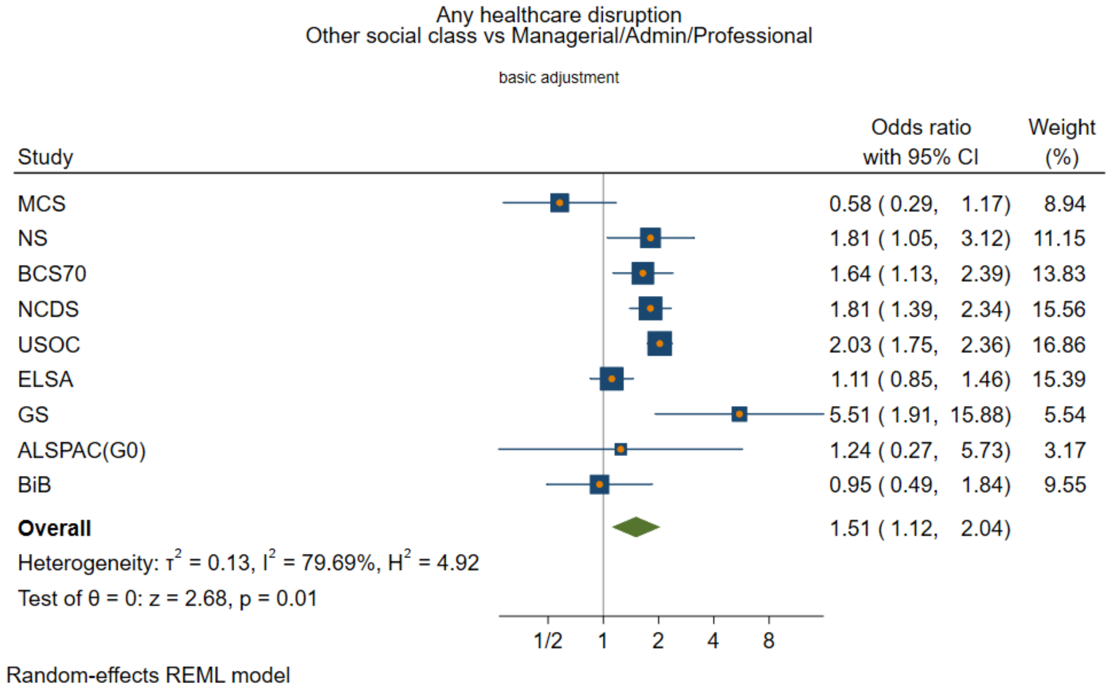

Maddock J, *et al. BMJ Open* 2022; 12:e064981. doi: 10.1136/bmjopen-2022-064981

Full adjustment

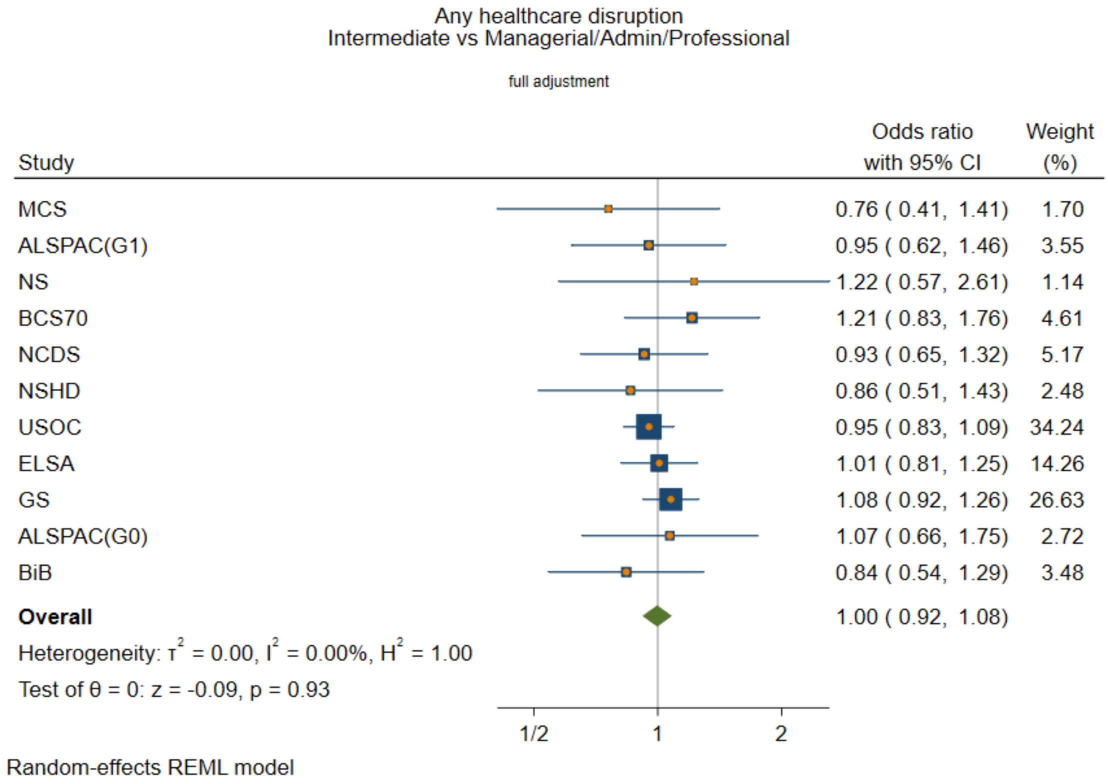

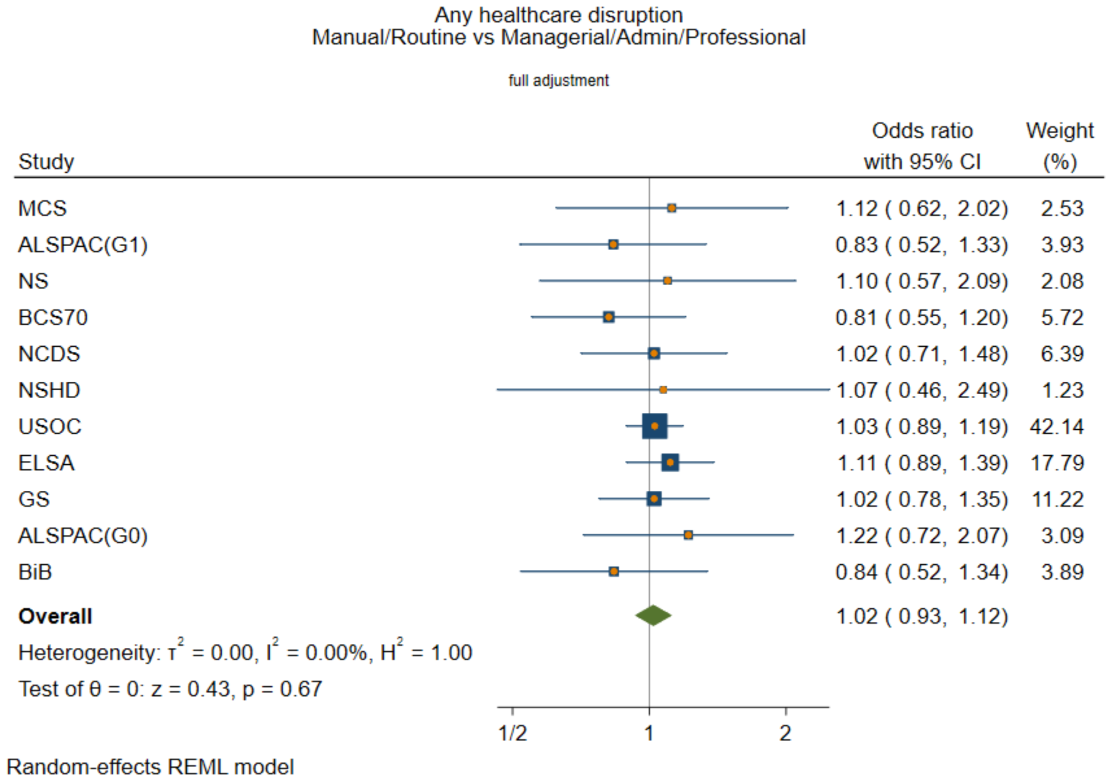

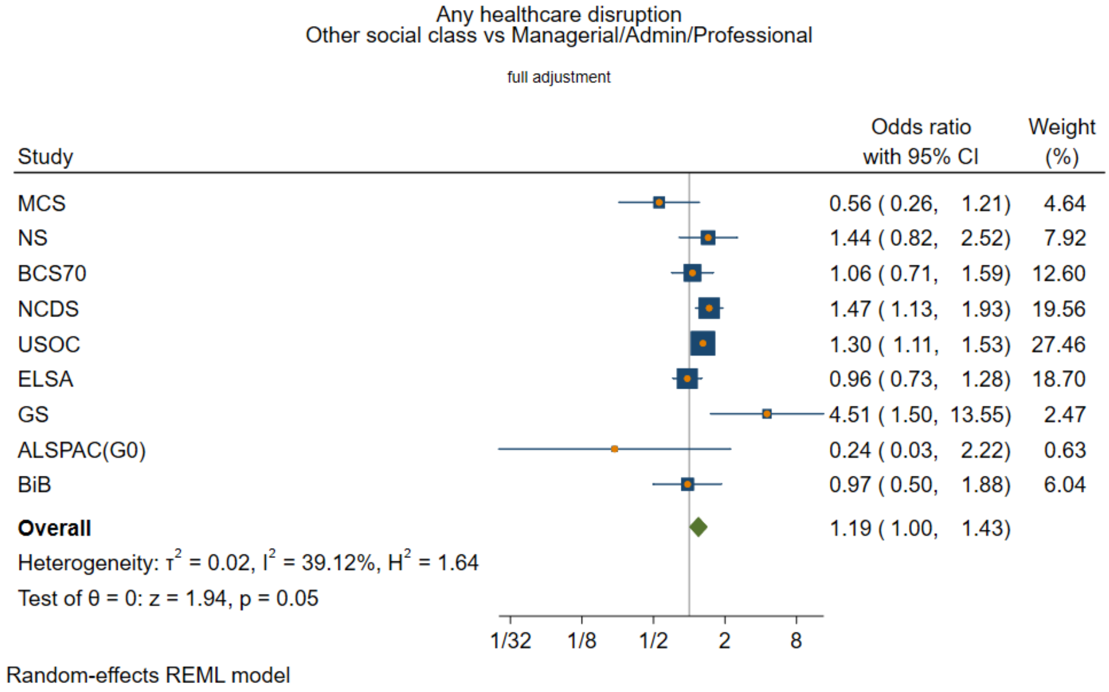

Maddock J, *et al. BMJ Open* 2022; 12:e064981. doi: 10.1136/bmjopen-2022-064981

Education
<u>Unadjusted</u>

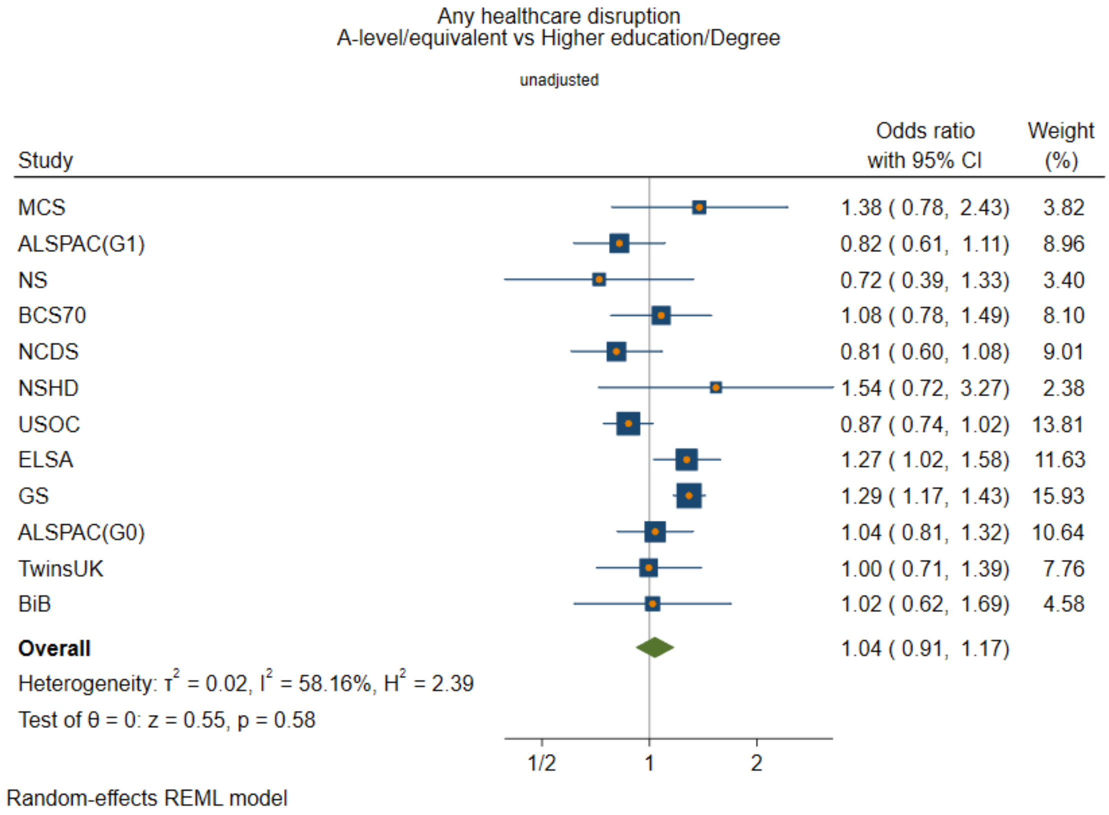

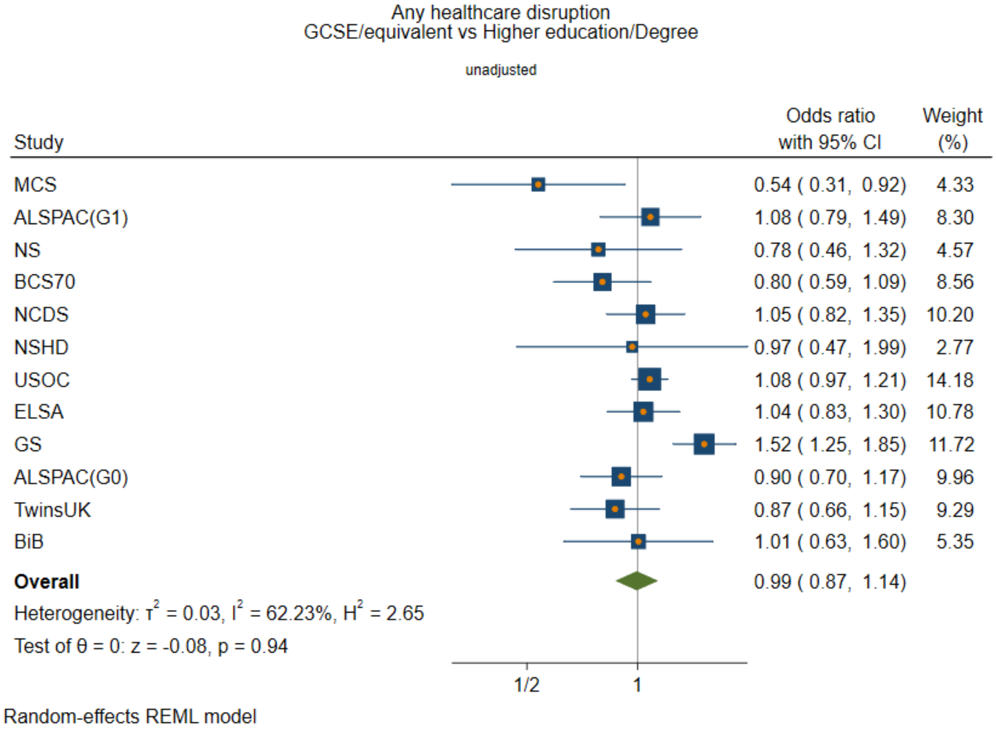
Any healthcare disruption
GCSE/equivalent vs Higher education/Degree
unadjusted
Study
Odds ratio with 95% CI
Weight (%)
MCS 0.54 (0.31, 0.92) 4.33
ALSPAC(G1) 1.08 (0.79, 1.49) 8.30
NS 0.78 (0.46, 1.32) 4.57
BCS70 0.80 (0.59, 1.09) 8.56
NCDS 1.05 (0.82, 1.35) 10.20
NSHD 0.97 (0.47, 1.99) 2.77
USOC 1.08 (0.97, 1.21) 14.18
ELSA 1.04 (0.83, 1.30) 10.78
GS 1.52 (1.25, 1.85) 11.72
ALSPAC(G0) 0.90 (0.70, 1.17) 9.96
TwinsUK 0.87 (0.66, 1.15) 9.29
BiB 1.01 (0.63, 1.60) 5.35
Overall 0.99 (0.87, 1.14)
Heterogeneity: τ² = 0.03, I² = 62.23%, H² = 2.65
Test of θ = 0: z = -0.08, p = 0.94
1/2
1
Random-effects REML model

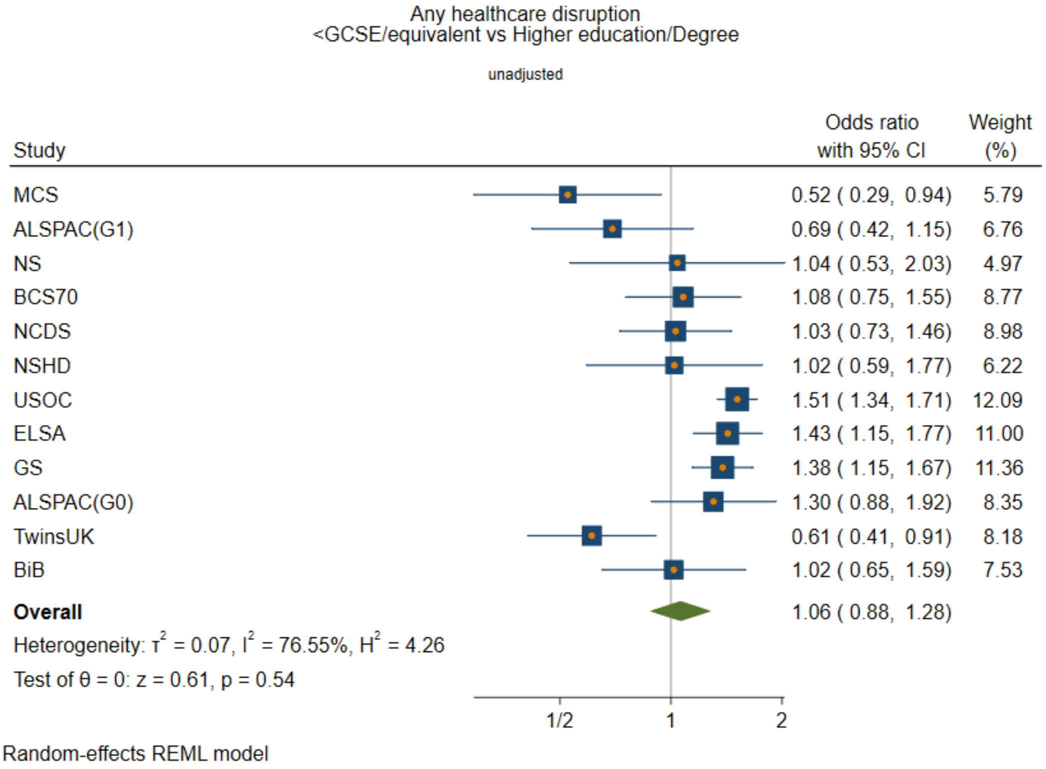
Any healthcare disruption
<GCSE/equivalent vs Higher education/Degree
unadjusted
Study
Odds ratio with 95% CI
Weight (%)
MCS 0.52 (0.29, 0.94) 5.79
ALSPAC(G1) 0.69 (0.42, 1.15) 6.76
NS 1.04 (0.53, 2.03) 4.97
BCS70 1.08 (0.75, 1.55) 8.77
NCDS 1.03 (0.73, 1.46) 8.98
NSHD 1.02 (0.59, 1.77) 6.22
USOC 1.51 (1.34, 1.71) 12.09
ELSA 1.43 (1.15, 1.77) 11.00
GS 1.38 (1.15, 1.67) 11.36
ALSPAC(G0) 1.30 (0.88, 1.92) 8.35
TwinsUK 0.61 (0.41, 0.91) 8.18
BiB 1.02 (0.65, 1.59) 7.53
Overall 1.06 (0.88, 1.28)
Heterogeneity: τ² = 0.07, I² = 76.55%, H² = 4.26
Test of θ = 0: z = 0.61, p = 0.54
1/2
1
2
Random-effects REML model

Basic adjustment

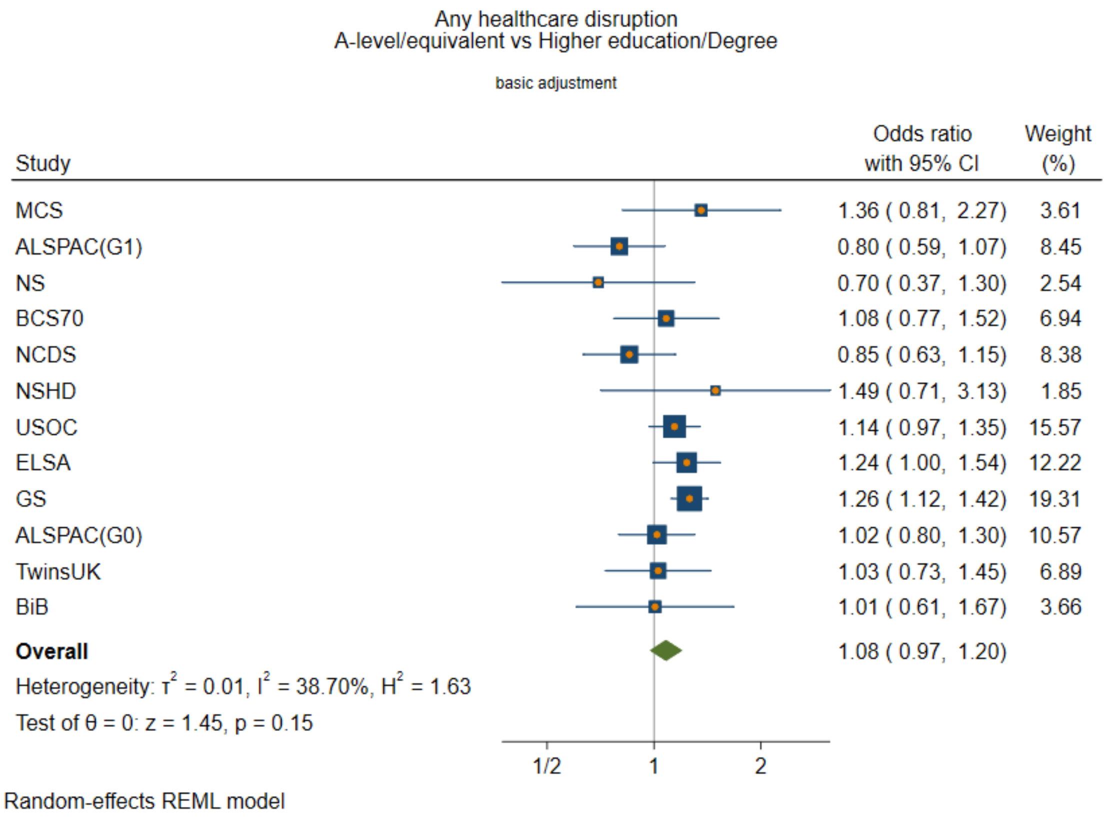

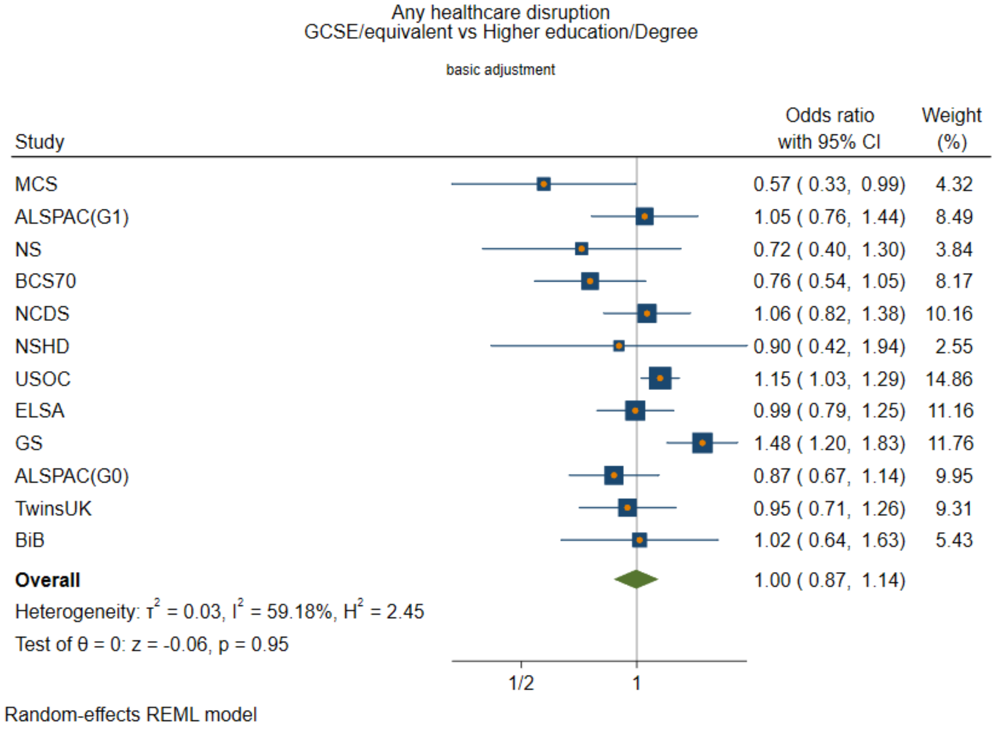

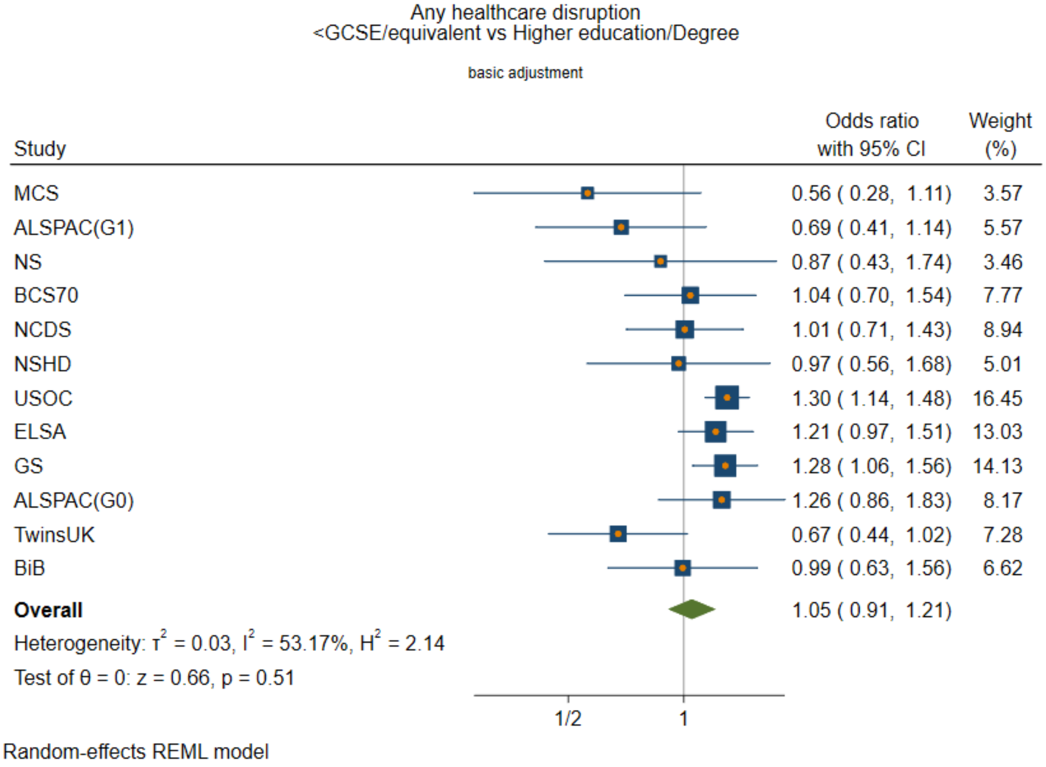

Maddock J, *et al. BMJ Open* 2022; 12:e064981. doi: 10.1136/bmjopen-2022-064981

Full adjustment

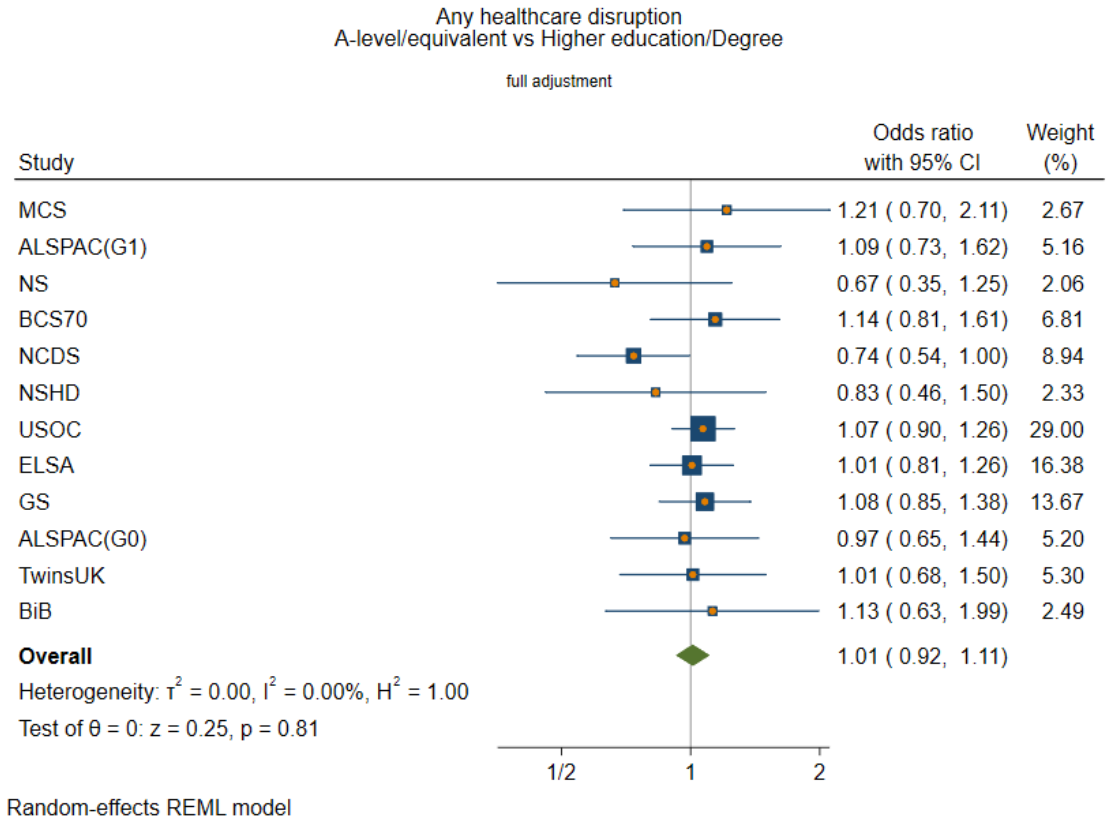

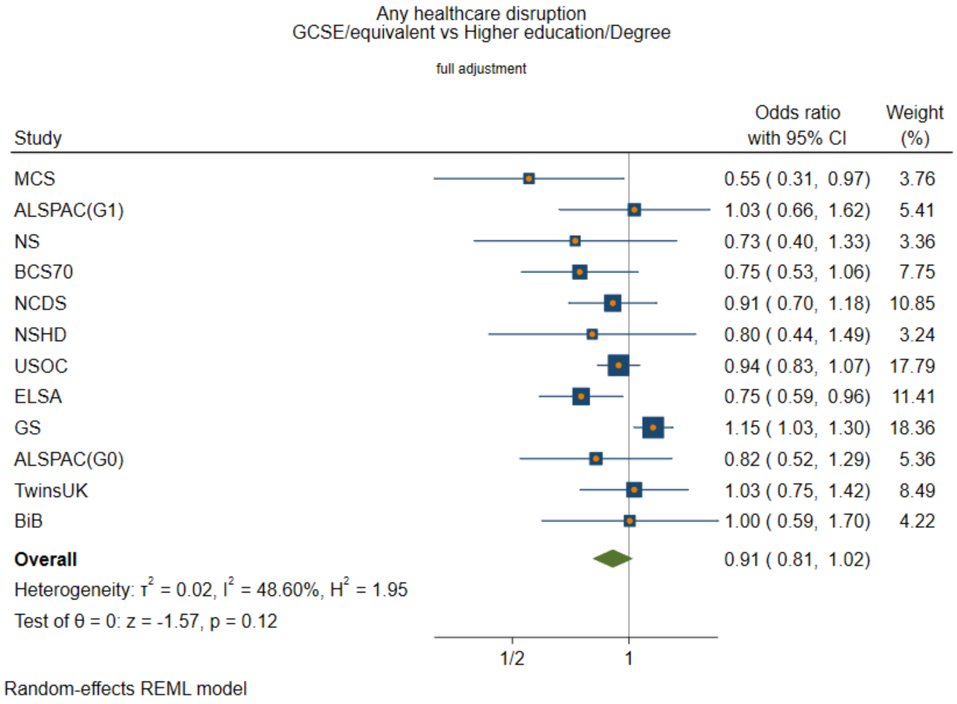

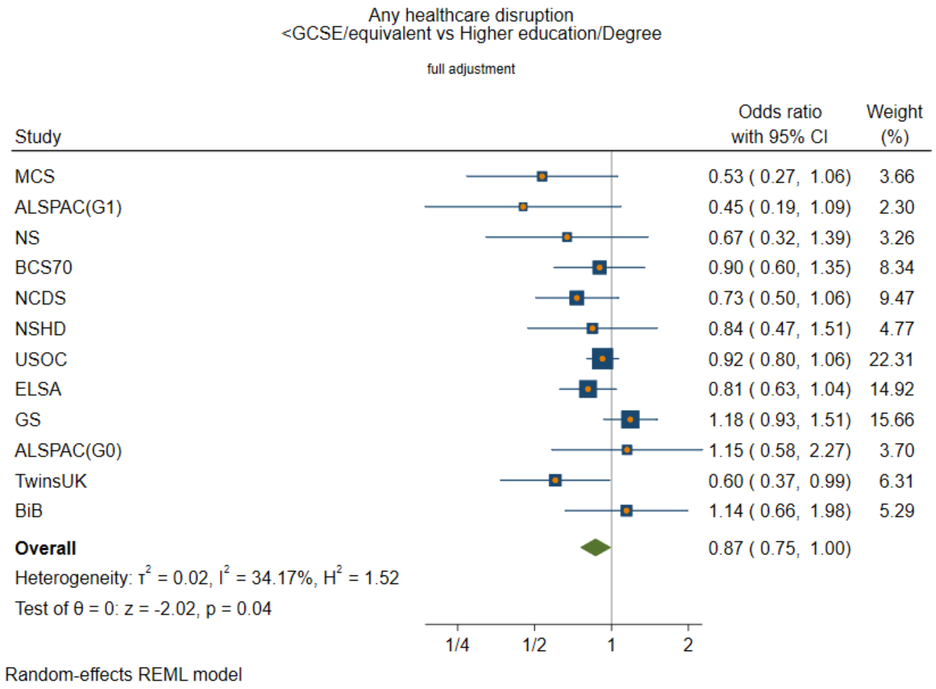

Maddock J, *et al. BMJ Open* 2022; 12:e064981. doi: 10.1136/bmjopen-2022-064981

Age

<u>Unadjusted</u>

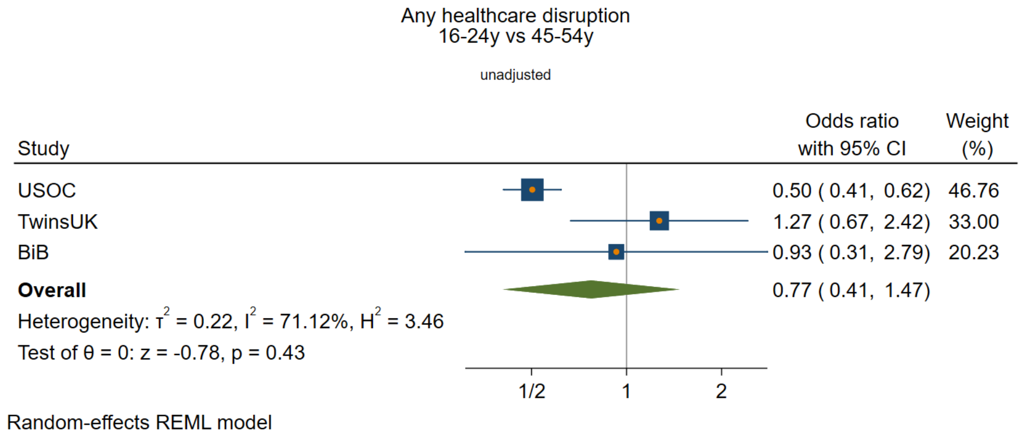

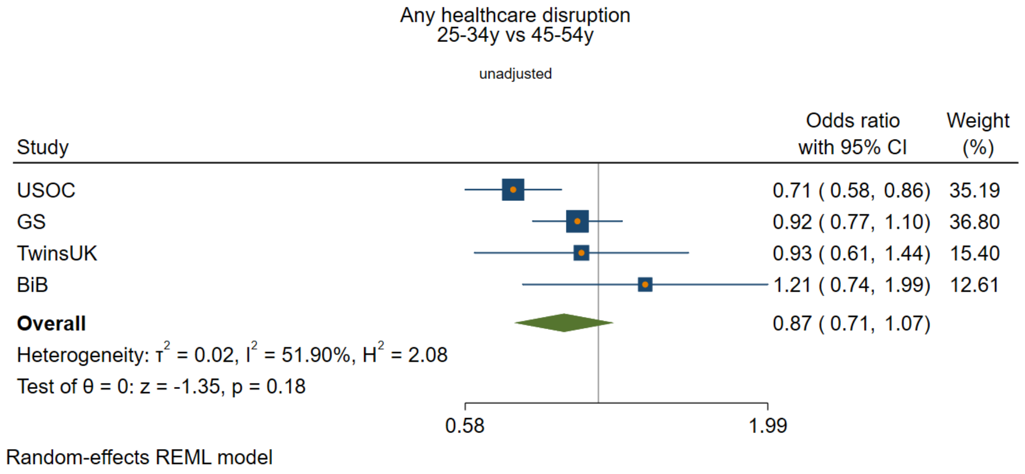

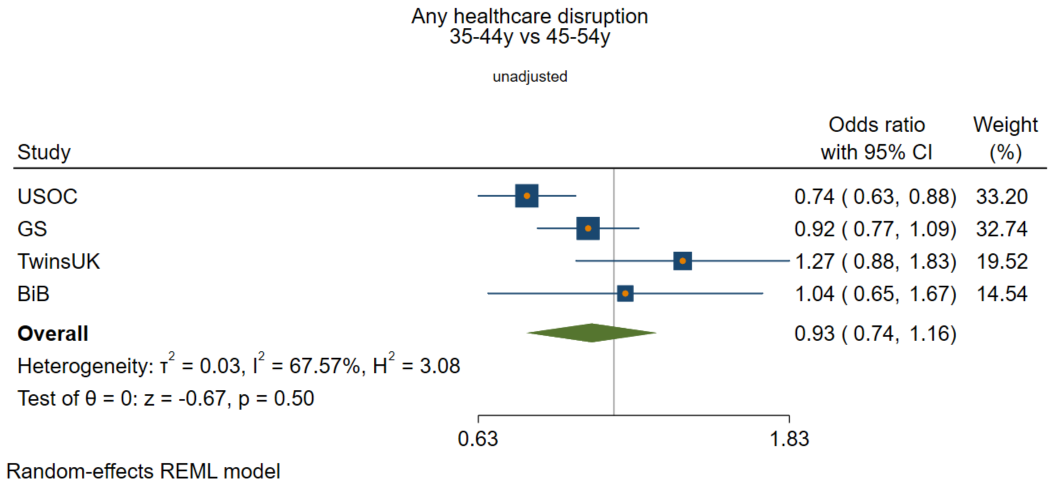

Maddock J, *et al. BMJ Open* 2022; 12:e064981. doi: 10.1136/bmjopen-2022-064981

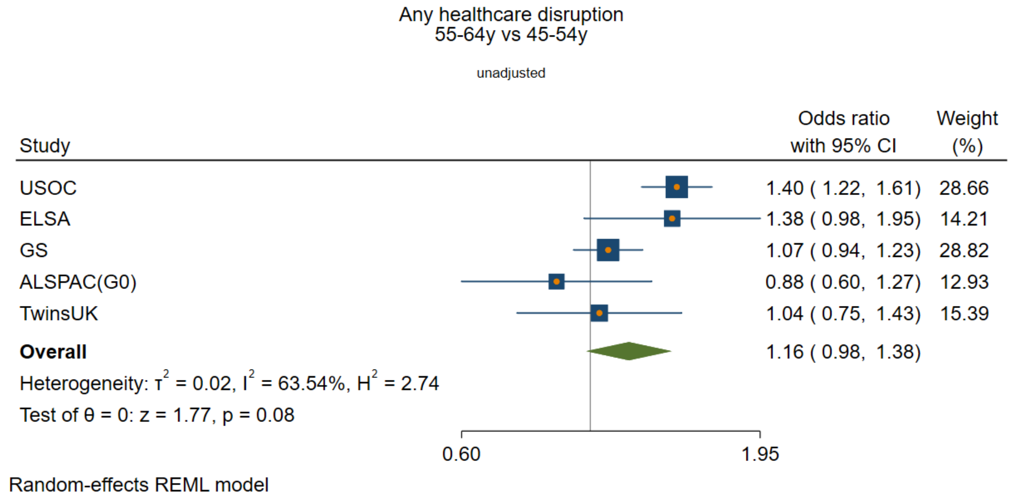
Any healthcare disruption
55-64y vs 45-54y
unadjusted
Study
Odds ratio with 95% CI
Weight (%)
USOC 1.40 (1.22, 1.61) 28.66
ELSA 1.38 (0.98, 1.95) 14.21
GS 1.07 (0.94, 1.23) 28.82
ALSPAC(G0) 0.88 (0.60, 1.27) 12.93
TwinsUK 1.04 (0.75, 1.43) 15.39
Overall 1.16 (0.98, 1.38)
Heterogeneity: τ² = 0.02, I² = 63.54%, H² = 2.74
Test of θ = 0: z = 1.77, p = 0.08
0.60
1.95
Random-effects REML model

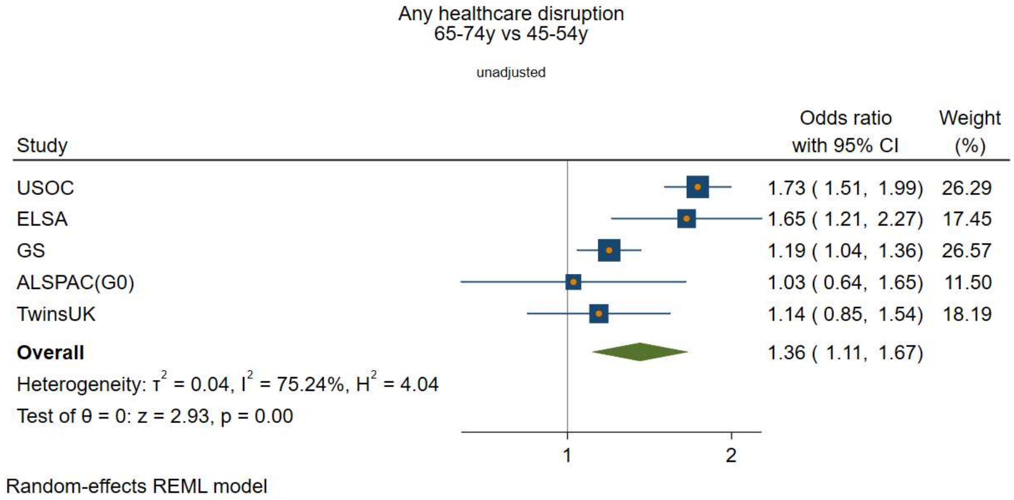
Any healthcare disruption
65-74y vs 45-54y
unadjusted
Study
Odds ratio with 95% CI
Weight (%)
USOC 1.73 (1.51, 1.99) 26.29
ELSA 1.65 (1.21, 2.27) 17.45
GS 1.19 (1.04, 1.36) 26.57
ALSPAC(G0) 1.03 (0.64, 1.65) 11.50
TwinsUK 1.14 (0.85, 1.54) 18.19
Overall 1.36 (1.11, 1.67)
Heterogeneity: τ² = 0.04, I² = 75.24%, H² = 4.04
Test of θ = 0: z = 2.93, p = 0.00
1
2
Random-effects REML model

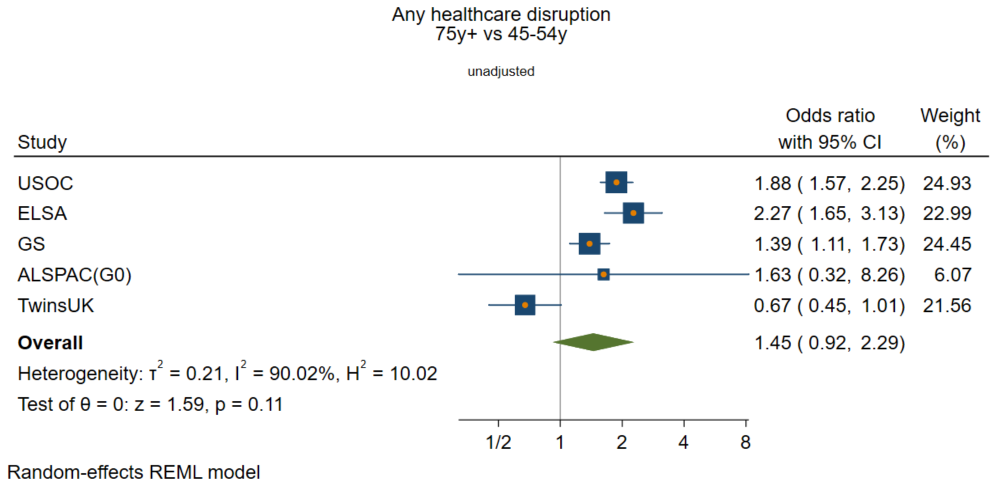
Any healthcare disruption
75y+ vs 45-54y
unadjusted
Study
Odds ratio with 95% CI
Weight (%)
USOC 1.88 (1.57, 2.25) 24.93
ELSA 2.27 (1.65, 3.13) 22.99
GS 1.39 (1.11, 1.73) 24.45
ALSPAC(G0) 1.63 (0.32, 8.26) 6.07
TwinsUK 0.67 (0.45, 1.01) 21.56
Overall 1.45 (0.92, 2.29)
Heterogeneity: τ² = 0.21, I² = 90.02%, H² = 10.02
Test of θ = 0: z = 1.59, p = 0.11
1/2
1
2
4
8
Random-effects REML model

26

Basic adjustment

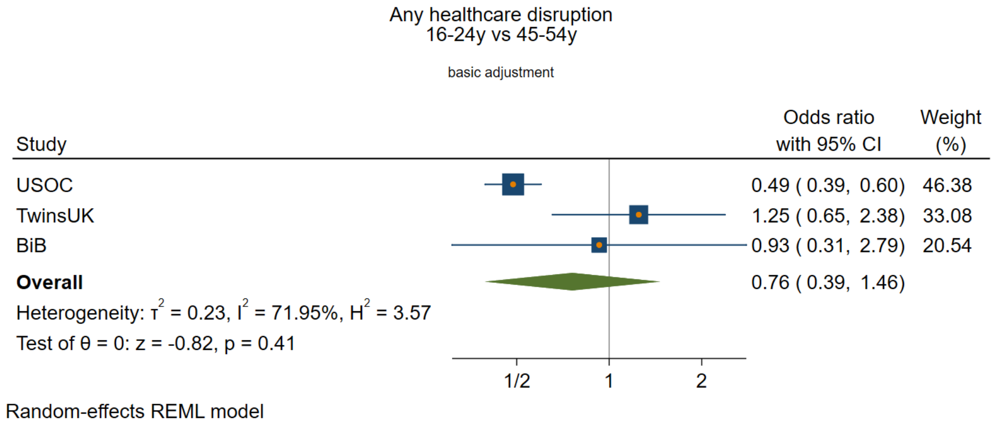

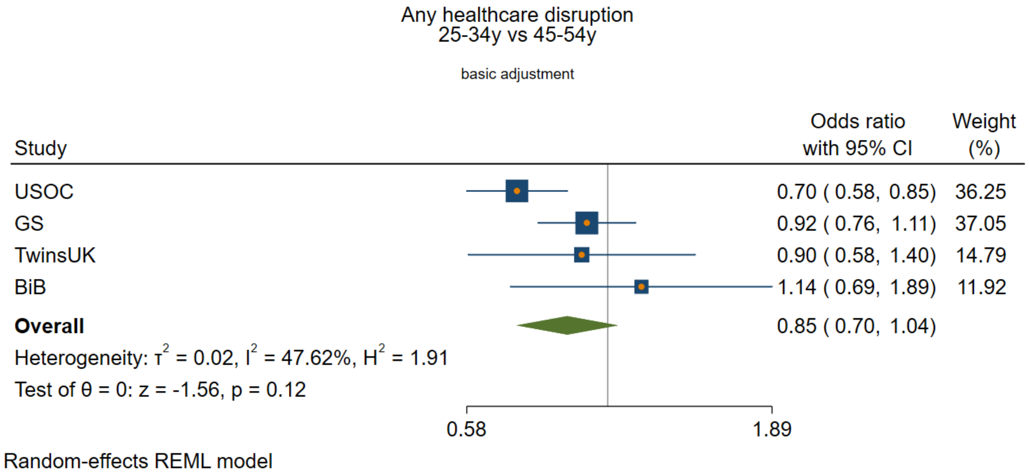

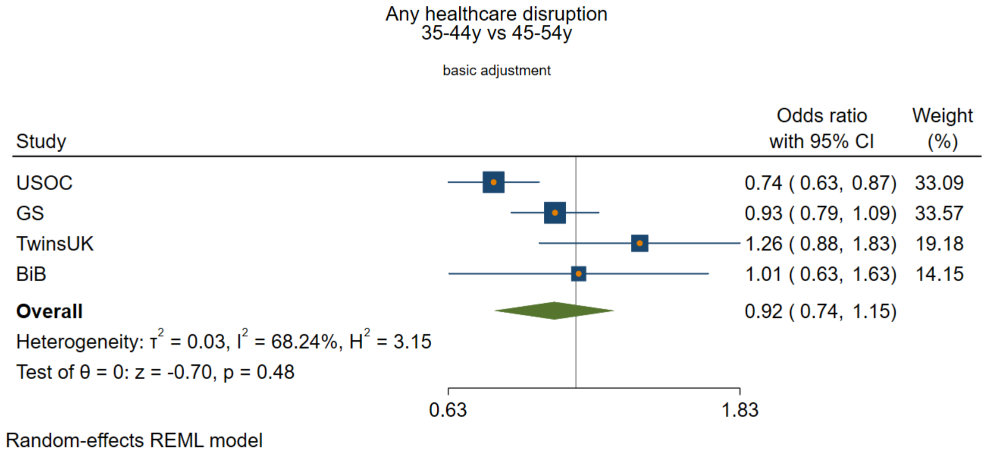

Maddock J, *et al. BMJ Open* 2022; 12:e064981. doi: 10.1136/bmjopen-2022-064981

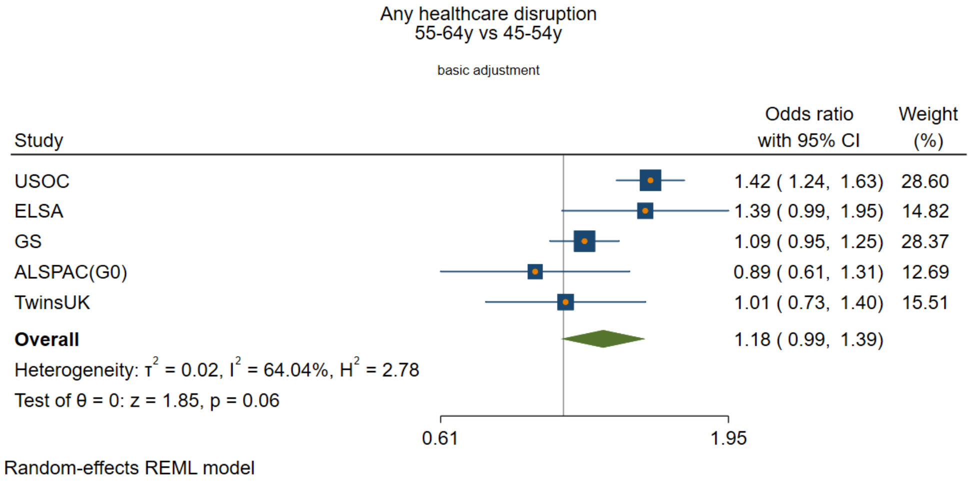
Any healthcare disruption
55-64y vs 45-54y
basic adjustment
Study
Odds ratio with 95% CI
Weight (%)
USOC 1.42 (1.24, 1.63) 28.60
ELSA 1.39 (0.99, 1.95) 14.82
GS 1.09 (0.95, 1.25) 28.37
ALSPAC(G0) 0.89 (0.61, 1.31) 12.69
TwinsUK 1.01 (0.73, 1.40) 15.51
Overall 1.18 (0.99, 1.39)
Heterogeneity: τ² = 0.02, I² = 64.04%, H² = 2.78
Test of θ = 0: z = 1.85, p = 0.06
0.61
1.95
Random-effects REML model

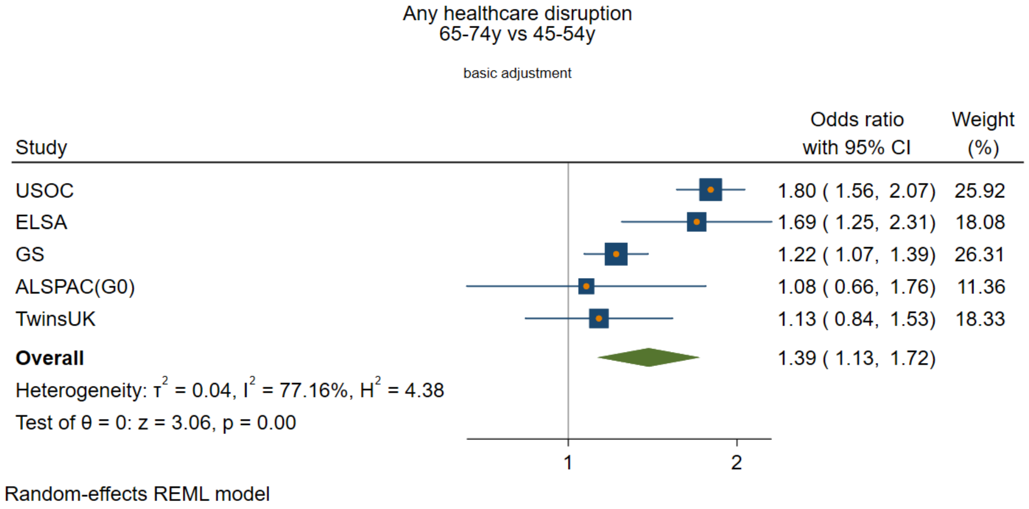
Any healthcare disruption
65-74y vs 45-54y
basic adjustment
Study
Odds ratio with 95% CI
Weight (%)
USOC 1.80 (1.56, 2.07) 25.92
ELSA 1.69 (1.25, 2.31) 18.08
GS 1.22 (1.07, 1.39) 26.31
ALSPAC(G0) 1.08 (0.66, 1.76) 11.36
TwinsUK 1.13 (0.84, 1.53) 18.33
Overall 1.39 (1.13, 1.72)
Heterogeneity: τ² = 0.04, I² = 77.16%, H² = 4.38
Test of θ = 0: z = 3.06, p = 0.00
1
2
Random-effects REML model

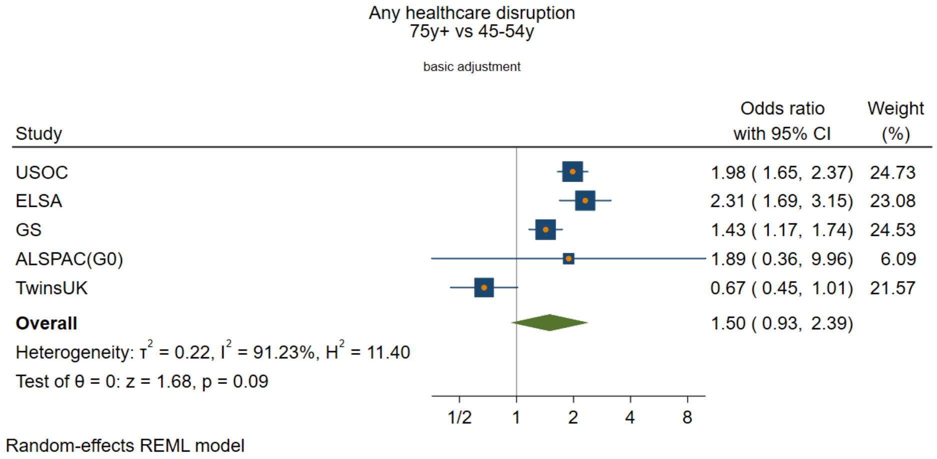
Any healthcare disruption
75y+ vs 45-54y
basic adjustment
Study
Odds ratio with 95% CI
Weight (%)
USOC 1.98 (1.65, 2.37) 24.73
ELSA 2.31 (1.69, 3.15) 23.08
GS 1.43 (1.17, 1.74) 24.53
ALSPAC(G0) 1.89 (0.36, 9.96) 6.09
TwinsUK 0.67 (0.45, 1.01) 21.57
Overall 1.50 (0.93, 2.39)
Heterogeneity: τ² = 0.22, I² = 91.23%, H² = 11.40
Test of θ = 0: z = 1.68, p = 0.09
1/2
1
2
4
8
Random-effects REML model

Full adjustment

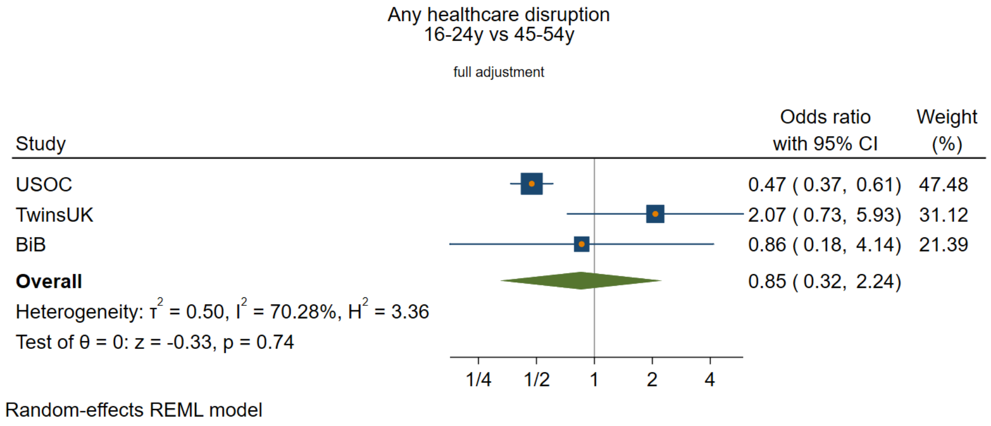

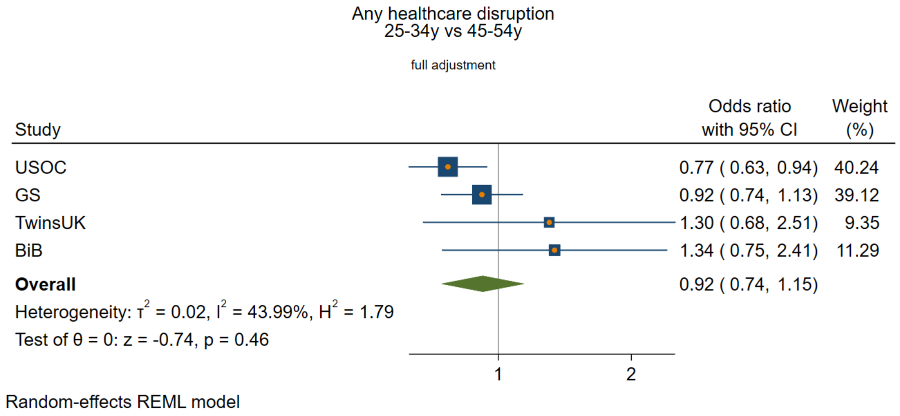

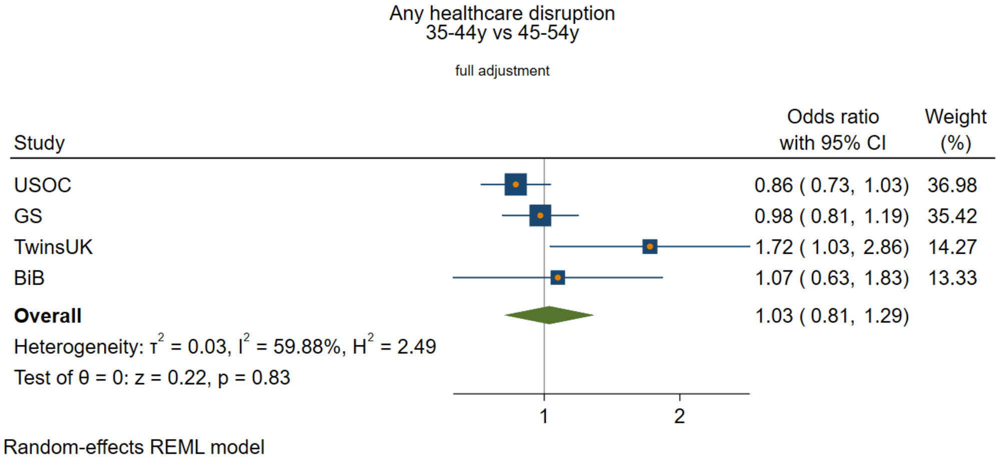

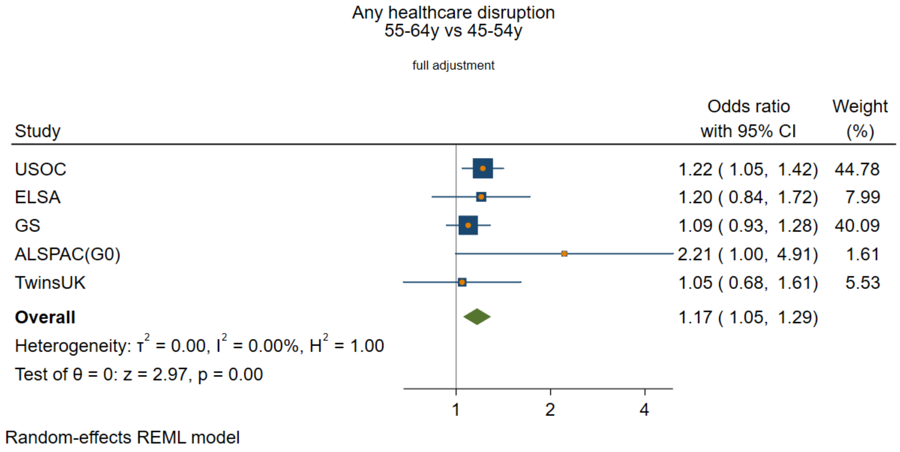
Any healthcare disruption
55-64y vs 45-54y
full adjustment
Study
Odds ratio with 95% CI
Weight (%)
USOC 1.22 (1.05, 1.42) 44.78
ELSA 1.20 (0.84, 1.72) 7.99
GS 1.09 (0.93, 1.28) 40.09
ALSPAC(G0) 2.21 (1.00, 4.91) 1.61
TwinsUK 1.05 (0.68, 1.61) 5.53
Overall 1.17 (1.05, 1.29)
Heterogeneity: τ² = 0.00, I² = 0.00%, H² = 1.00
Test of θ = 0: z = 2.97, p = 0.00
1
2
4
Random-effects REML model

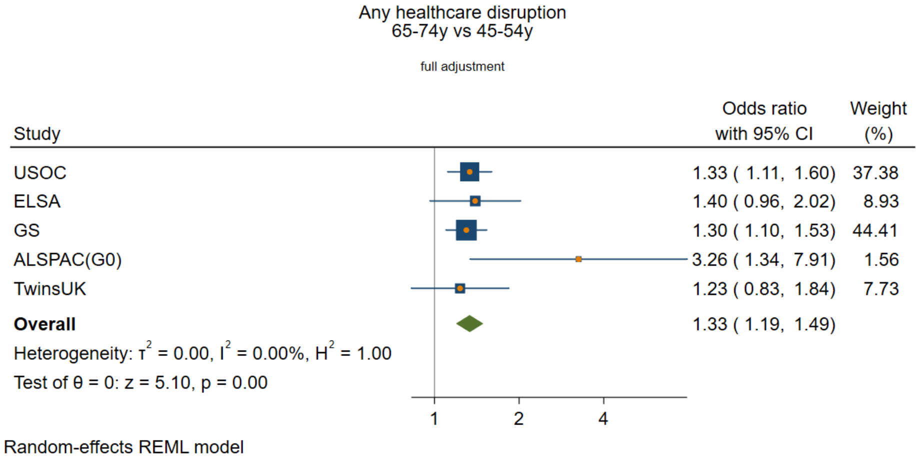
Any healthcare disruption
65-74y vs 45-54y
full adjustment
Study
Odds ratio with 95% CI
Weight (%)
USOC 1.33 (1.11, 1.60) 37.38
ELSA 1.40 (0.96, 2.02) 8.93
GS 1.30 (1.10, 1.53) 44.41
ALSPAC(G0) 3.26 (1.34, 7.91) 1.56
TwinsUK 1.23 (0.83, 1.84) 7.73
Overall 1.33 (1.19, 1.49)
Heterogeneity: τ² = 0.00, I² = 0.00%, H² = 1.00
Test of θ = 0: z = 5.10, p = 0.00
1
2
4
Random-effects REML model

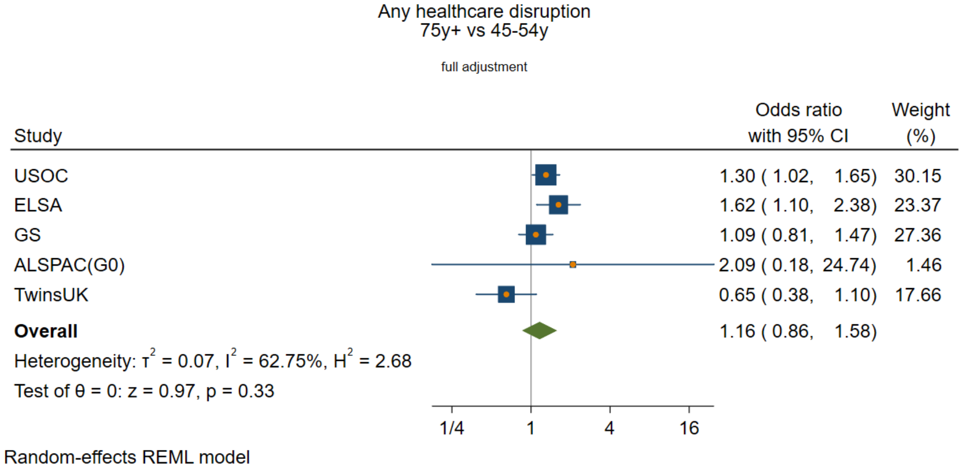
Any healthcare disruption
75y+ vs 45-54y
full adjustment
Study
Odds ratio with 95% CI
Weight (%)
USOC 1.30 (1.02, 1.65) 30.15
ELSA 1.62 (1.10, 2.38) 23.37
GS 1.09 (0.81, 1.47) 27.36
ALSPAC(G0) 2.09 (0.18, 24.74) 1.46
TwinsUK 0.65 (0.38, 1.10) 17.66
Overall 1.16 (0.86, 1.58)
Heterogeneity: τ² = 0.07, I² = 62.75%, H² = 2.68
Test of θ = 0: z = 0.97, p = 0.33
1/4
1
4
16
Random-effects REML model

Ethnicity

Unadjusted

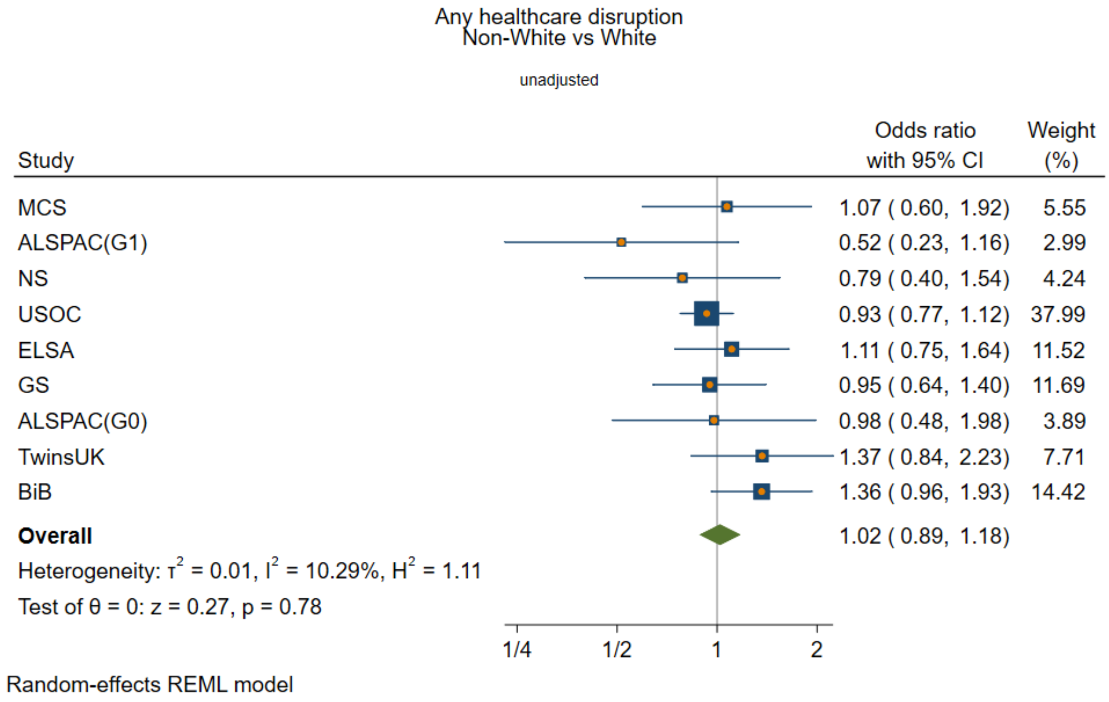

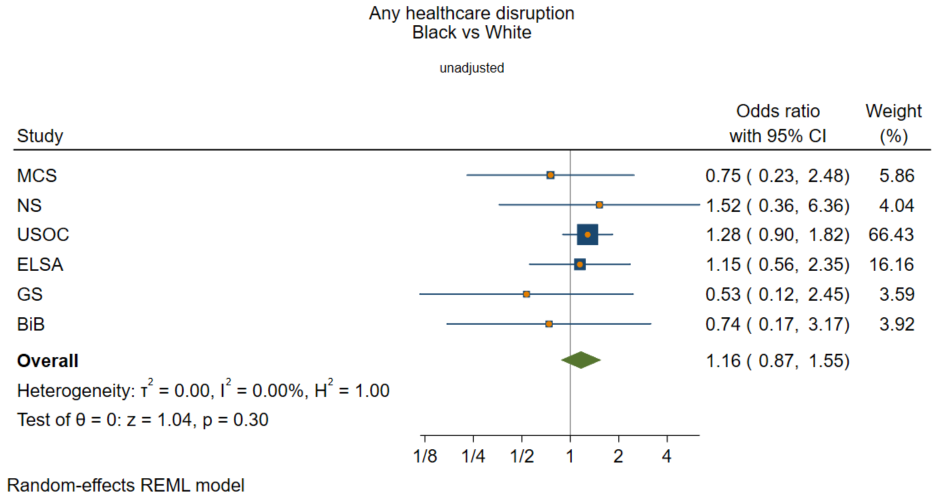

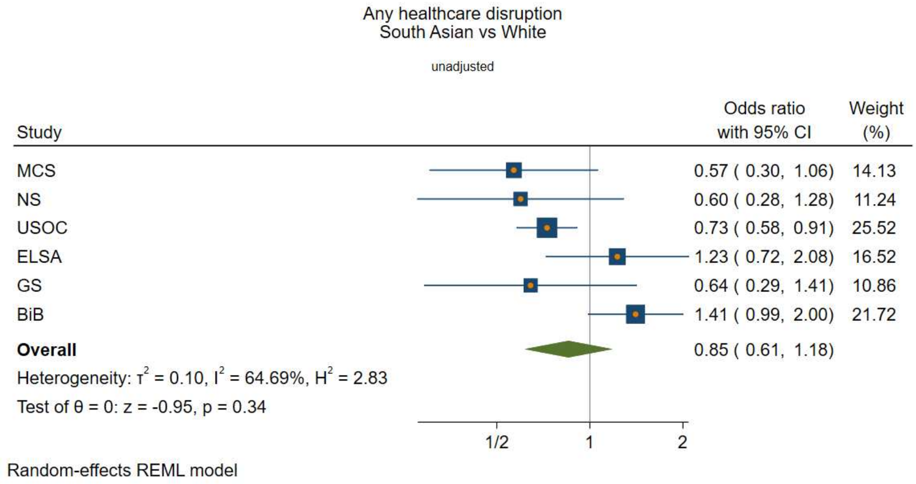

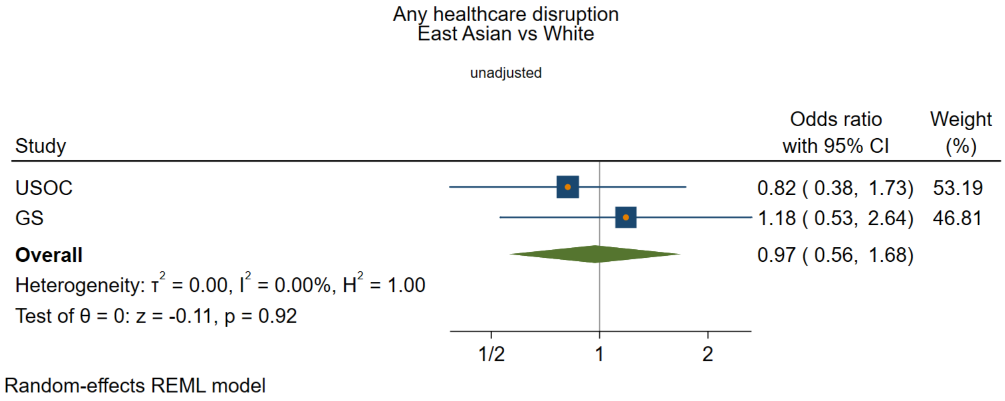

Maddock J, *et al. BMJ Open* 2022; 12:e064981. doi: 10.1136/bmjopen-2022-064981

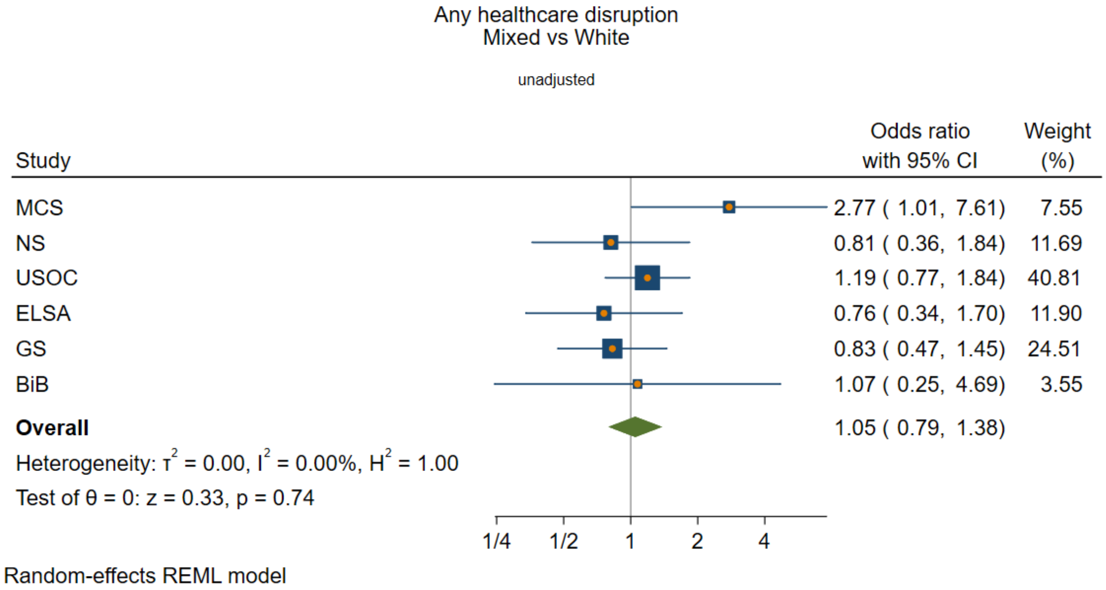
Any healthcare disruption
Mixed vs White
unadjusted
Study
Odds ratio with 95% CI
Weight (%)
MCS 2.77 (1.01, 7.61) 7.55
NS 0.81 (0.36, 1.84) 11.69
USOC 1.19 (0.77, 1.84) 40.81
ELSA 0.76 (0.34, 1.70) 11.90
GS 0.83 (0.47, 1.45) 24.51
BiB 1.07 (0.25, 4.69) 3.55
Overall 1.05 (0.79, 1.38)
Heterogeneity: τ² = 0.00, I² = 0.00%, H² = 1.00
Test of θ = 0: z = 0.33, p = 0.74
1/4 1/2 1 2 4
Random-effects REML model

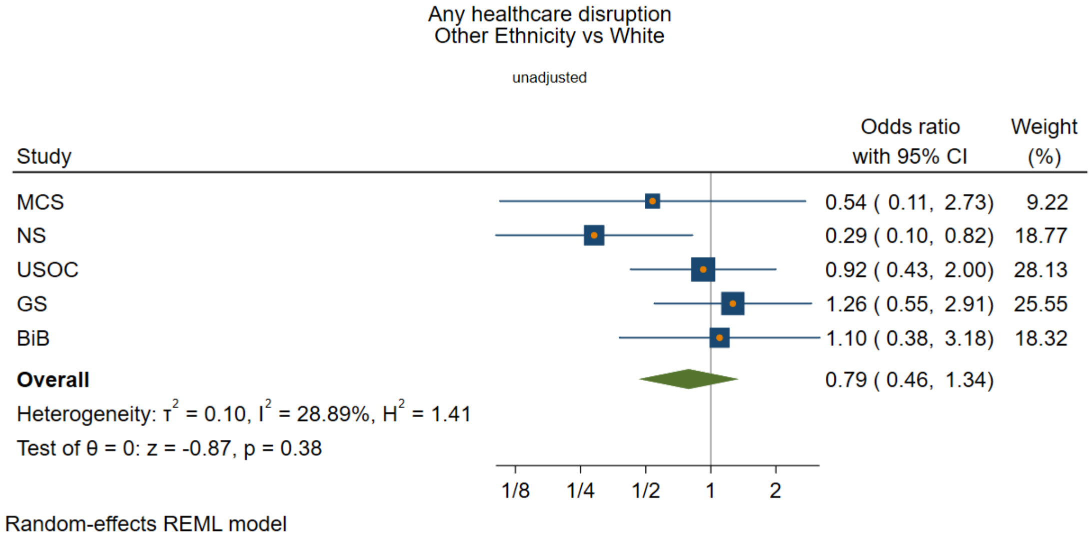
Any healthcare disruption
Other Ethnicity vs White
unadjusted
Study
Odds ratio with 95% CI
Weight (%)
MCS 0.54 (0.11, 2.73) 9.22
NS 0.29 (0.10, 0.82) 18.77
USOC 0.92 (0.43, 2.00) 28.13
GS 1.26 (0.55, 2.91) 25.55
BiB 1.10 (0.38, 3.18) 18.32
Overall 0.79 (0.46, 1.34)
Heterogeneity: τ² = 0.10, I² = 28.89%, H² = 1.41
Test of θ = 0: z = -0.87, p = 0.38
1/8 1/4 1/2 1 2
Random-effects REML model

Basic adjustment

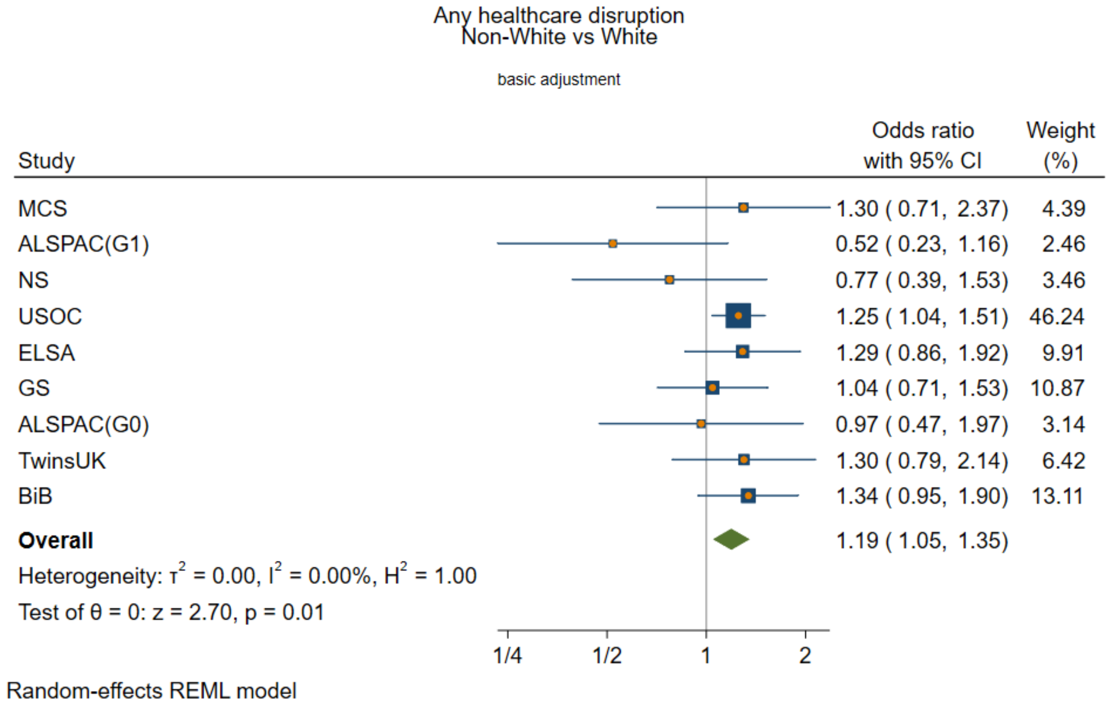

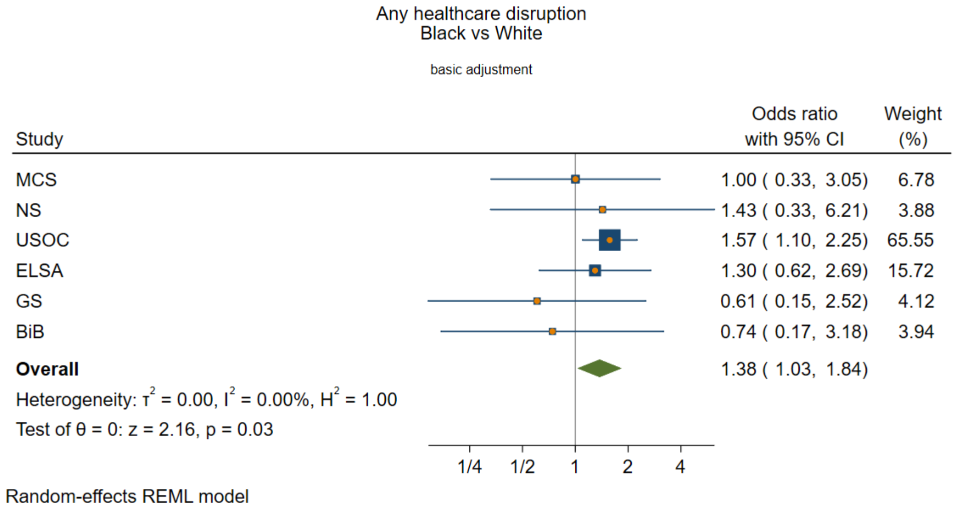
Any healthcare disruption
Black vs White
basic adjustment
Study
Odds ratio with 95% CI
Weight (%)
MCS 1.00 (0.33, 3.05) 6.78
NS 1.43 (0.33, 6.21) 3.88
USOC 1.57 (1.10, 2.25) 65.55
ELSA 1.30 (0.62, 2.69) 15.72
GS 0.61 (0.15, 2.52) 4.12
BiB 0.74 (0.17, 3.18) 3.94
Overall 1.38 (1.03, 1.84)
Heterogeneity: τ² = 0.00, I² = 0.00%, H² = 1.00
Test of θ = 0: z = 2.16, p = 0.03
1/4
1/2
1
2
4
Random-effects REML model

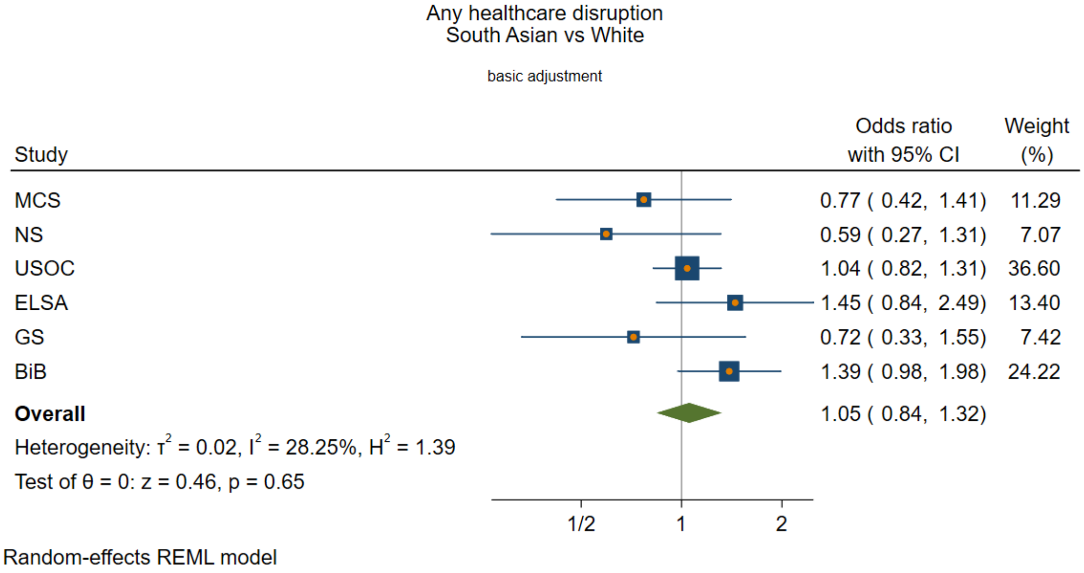
Any healthcare disruption
South Asian vs White
basic adjustment
Study
Odds ratio with 95% CI
Weight (%)
MCS 0.77 (0.42, 1.41) 11.29
NS 0.59 (0.27, 1.31) 7.07
USOC 1.04 (0.82, 1.31) 36.60
ELSA 1.45 (0.84, 2.49) 13.40
GS 0.72 (0.33, 1.55) 7.42
BiB 1.39 (0.98, 1.98) 24.22
Overall 1.05 (0.84, 1.32)
Heterogeneity: τ² = 0.02, I² = 28.25%, H² = 1.39
Test of θ = 0: z = 0.46, p = 0.65
1/2
1
2
Random-effects REML model

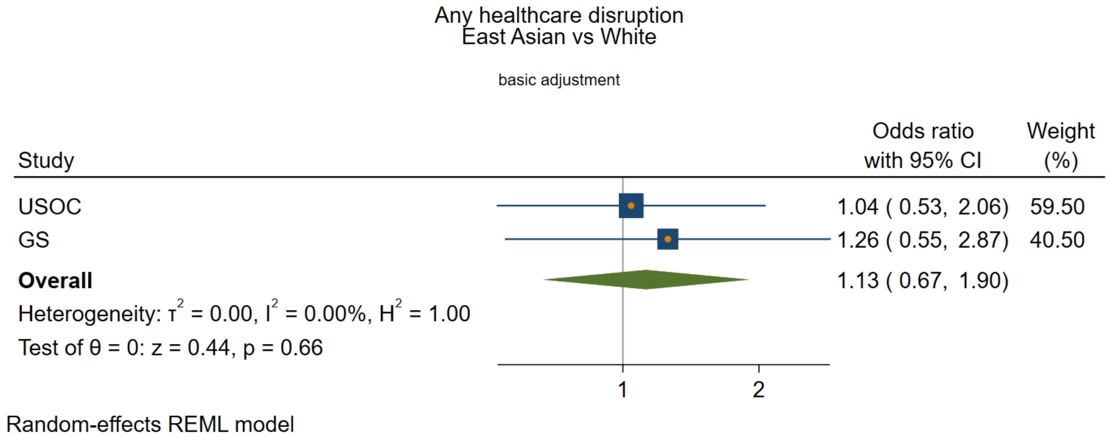

Maddock J, *et al. BMJ Open* 2022; 12:e064981. doi: 10.1136/bmjopen-2022-064981

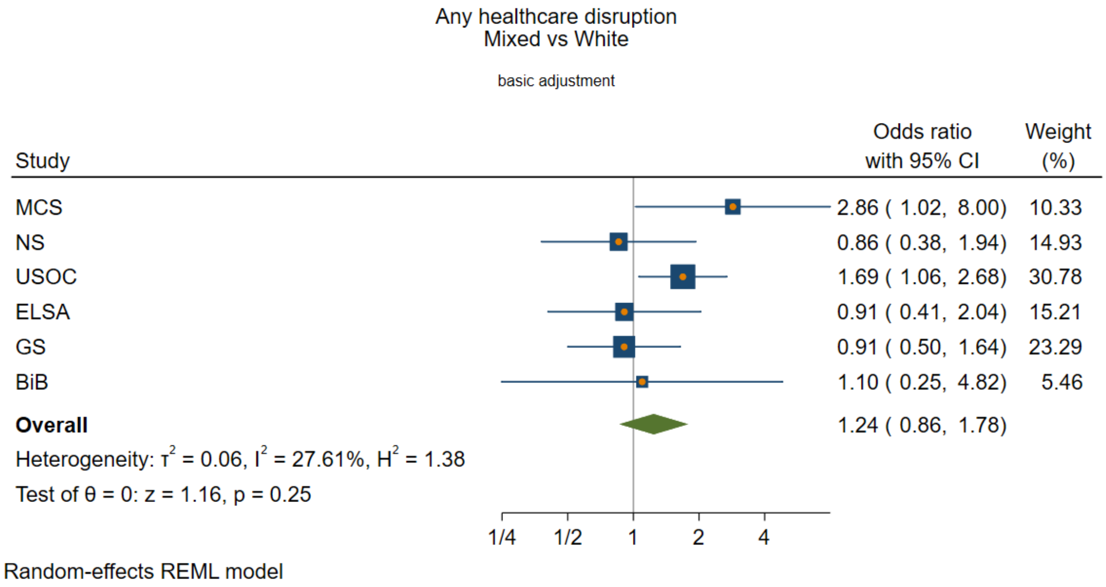
Any healthcare disruption
Mixed vs White
basic adjustment
Study
Odds ratio with 95% CI
Weight (%)
MCS 2.86 (1.02, 8.00) 10.33
NS 0.86 (0.38, 1.94) 14.93
USOC 1.69 (1.06, 2.68) 30.78
ELSA 0.91 (0.41, 2.04) 15.21
GS 0.91 (0.50, 1.64) 23.29
BiB 1.10 (0.25, 4.82) 5.46
Overall 1.24 (0.86, 1.78)
Heterogeneity: τ² = 0.06, I² = 27.61%, H² = 1.38
Test of θ = 0: z = 1.16, p = 0.25
1/4
1/2
1
2
4
Random-effects REML model

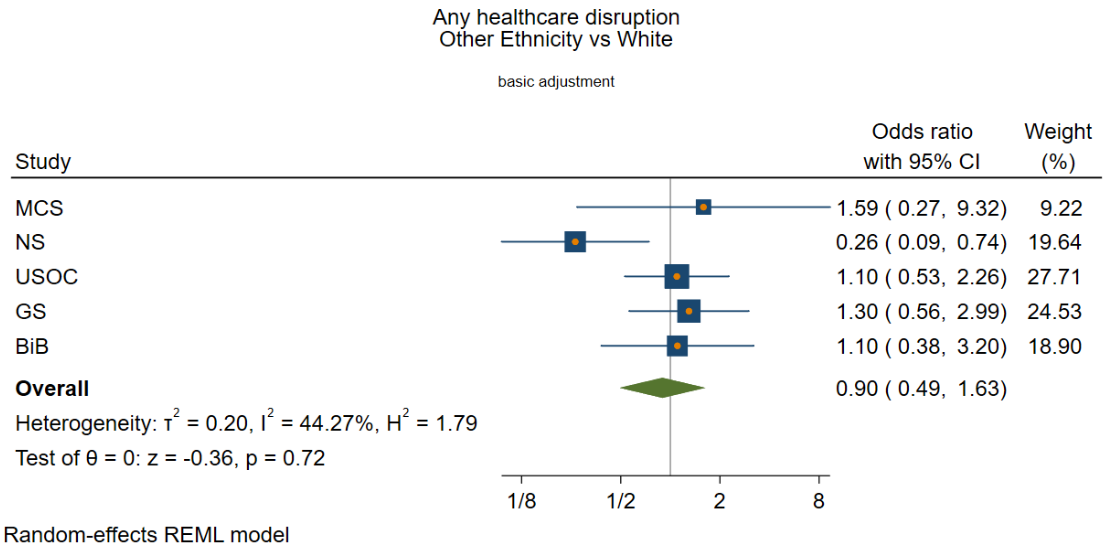
Any healthcare disruption
Other Ethnicity vs White
basic adjustment
Study
Odds ratio with 95% CI
Weight (%)
MCS 1.59 (0.27, 9.32) 9.22
NS 0.26 (0.09, 0.74) 19.64
USOC 1.10 (0.53, 2.26) 27.71
GS 1.30 (0.56, 2.99) 24.53
BiB 1.10 (0.38, 3.20) 18.90
Overall 0.90 (0.49, 1.63)
Heterogeneity: τ² = 0.20, I² = 44.27%, H² = 1.79
Test of θ = 0: z = -0.36, p = 0.72
1/8
1/2
2
8
Random-effects REML model

Full adjustment

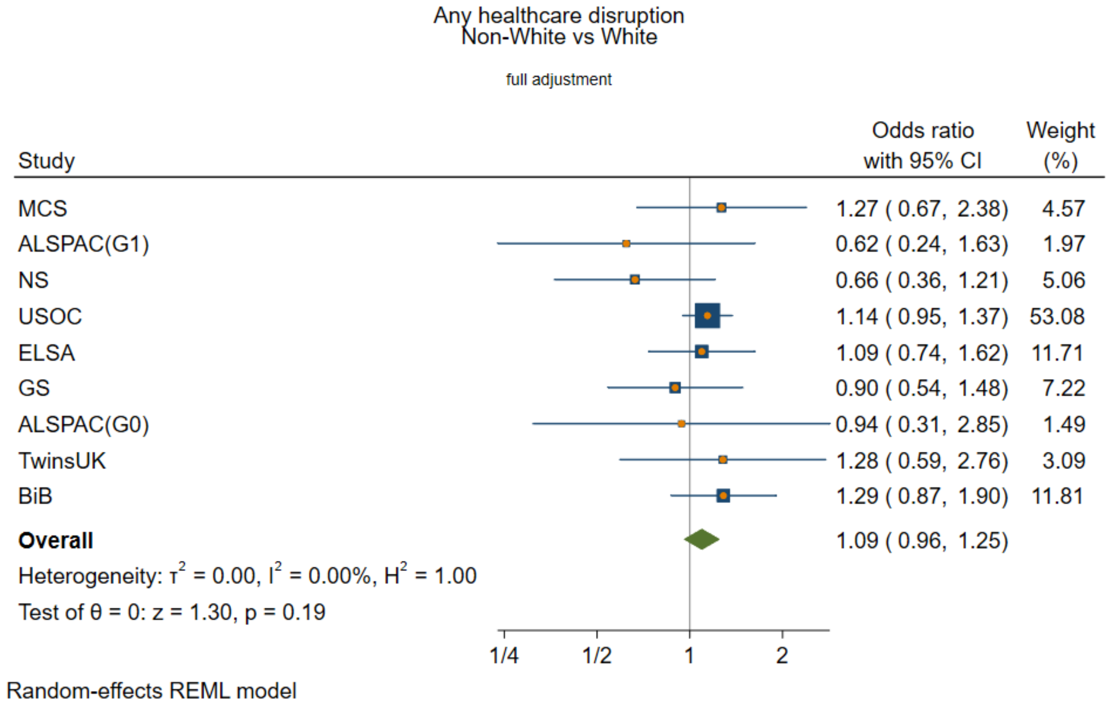

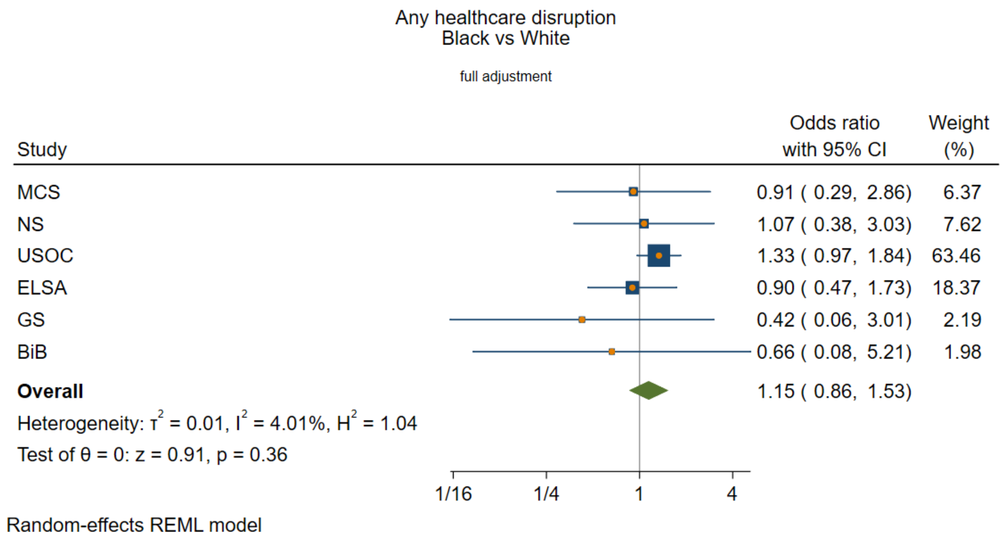
Any healthcare disruption
Black vs White
full adjustment
Study
Odds ratio with 95% CI
Weight (%)
MCS 0.91 (0.29, 2.86) 6.37
NS 1.07 (0.38, 3.03) 7.62
USOC 1.33 (0.97, 1.84) 63.46
ELSA 0.90 (0.47, 1.73) 18.37
GS 0.42 (0.06, 3.01) 2.19
BiB 0.66 (0.08, 5.21) 1.98
Overall 1.15 (0.86, 1.53)
Heterogeneity: τ² = 0.01, I² = 4.01%, H² = 1.04
Test of θ = 0: z = 0.91, p = 0.36
1/16
1/4
1
4
Random-effects REML model

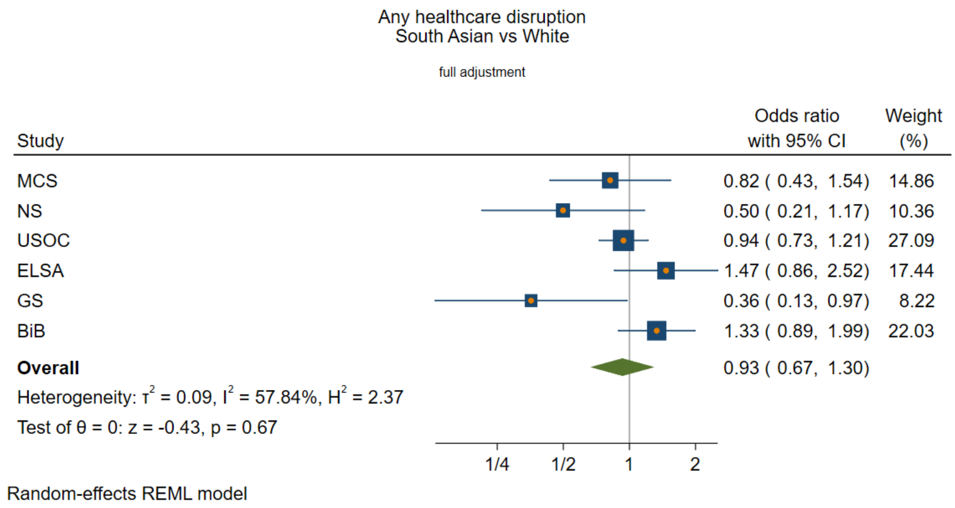
Any healthcare disruption
South Asian vs White
full adjustment
Study
Odds ratio with 95% CI
Weight (%)
MCS 0.82 (0.43, 1.54) 14.86
NS 0.50 (0.21, 1.17) 10.36
USOC 0.94 (0.73, 1.21) 27.09
ELSA 1.47 (0.86, 2.52) 17.44
GS 0.36 (0.13, 0.97) 8.22
BiB 1.33 (0.89, 1.99) 22.03
Overall 0.93 (0.67, 1.30)
Heterogeneity: τ² = 0.09, I² = 57.84%, H² = 2.37
Test of θ = 0: z = -0.43, p = 0.67
1/4
1/2
1
2
Random-effects REML model

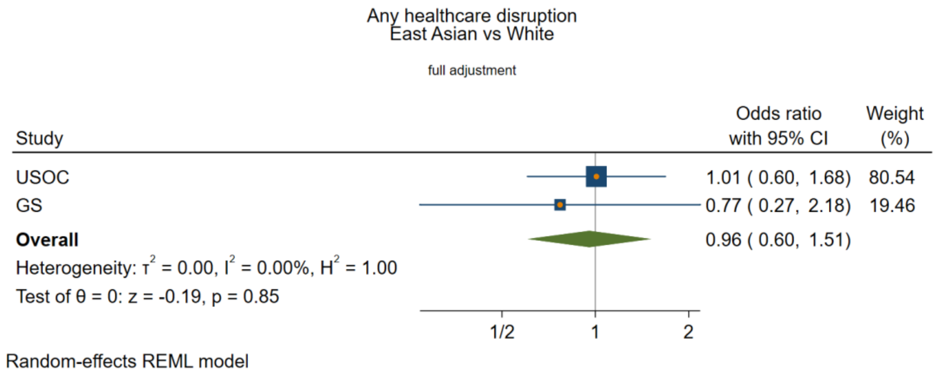
Any healthcare disruption
East Asian vs White
full adjustment
Study
Odds ratio with 95% CI
Weight (%)
USOC 1.01 (0.60, 1.68) 80.54
GS 0.77 (0.27, 2.18) 19.46
Overall 0.96 (0.60, 1.51)
Heterogeneity: τ² = 0.00, I² = 0.00%, H² = 1.00
Test of θ = 0: z = -0.19, p = 0.85
1/2
1
2
Random-effects REML model

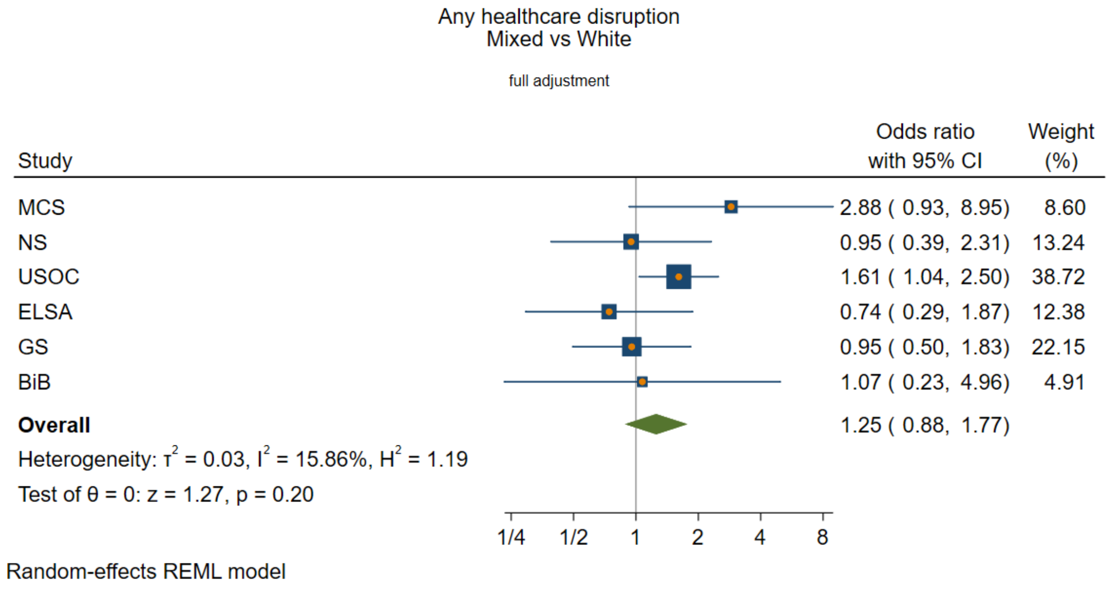
Any healthcare disruption
Mixed vs White
full adjustment
Study
Odds ratio with 95% CI
Weight (%)
MCS
2.88 (0.93, 8.95)
8.60
NS
0.95 (0.39, 2.31)
13.24
USOC
1.61 (1.04, 2.50)
38.72
ELSA
0.74 (0.29, 1.87)
12.38
GS
0.95 (0.50, 1.83)
22.15
BiB
1.07 (0.23, 4.96)
4.91
Overall
1.25 (0.88, 1.77)
Heterogeneity: τ² = 0.03, I² = 15.86%, H² = 1.19
Test of θ = 0: z = 1.27, p = 0.20
1/4
1/2
1
2
4
8
Random-effects REML model

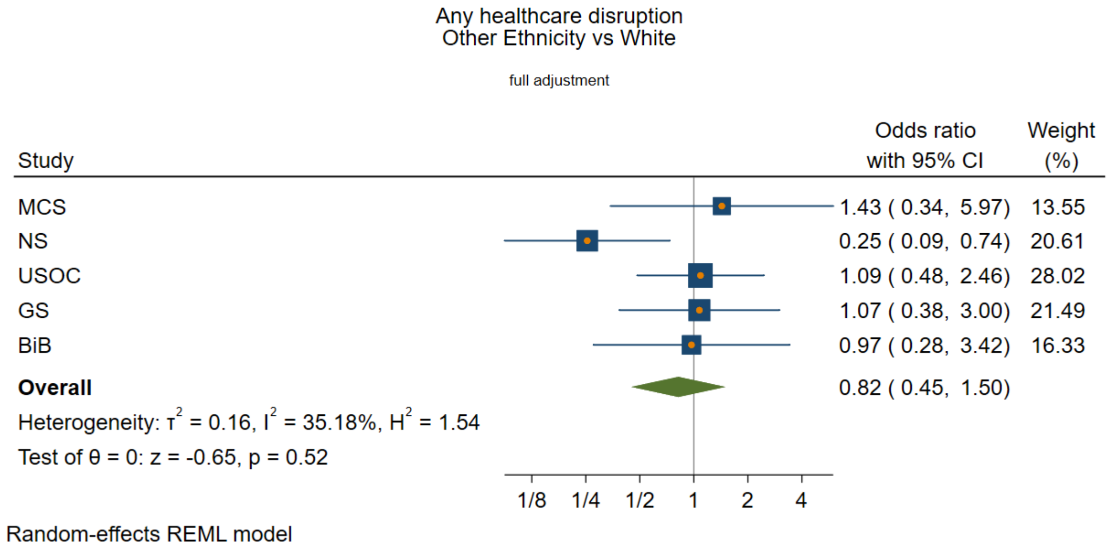
Any healthcare disruption
Other Ethnicity vs White
full adjustment
Study
Odds ratio with 95% CI
Weight (%)
MCS
1.43 (0.34, 5.97)
13.55
NS
0.25 (0.09, 0.74)
20.61
USOC
1.09 (0.48, 2.46)
28.02
GS
1.07 (0.38, 3.00)
21.49
BiB
0.97 (0.28, 3.42)
16.33
Overall
0.82 (0.45, 1.50)
Heterogeneity: τ² = 0.16, I² = 35.18%, H² = 1.54
Test of θ = 0: z = -0.65, p = 0.52
1/8
1/4
1/2
1
2
4
Random-effects REML model

Appointments

Sex

Unadjusted

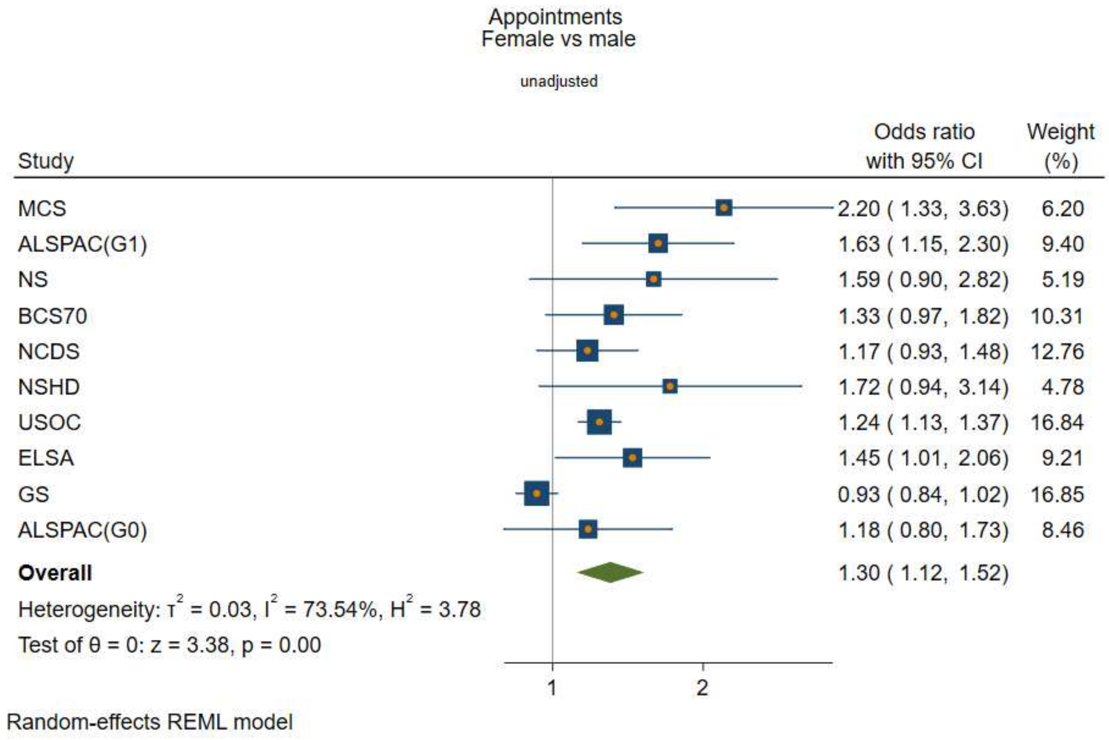

Basic adjustment

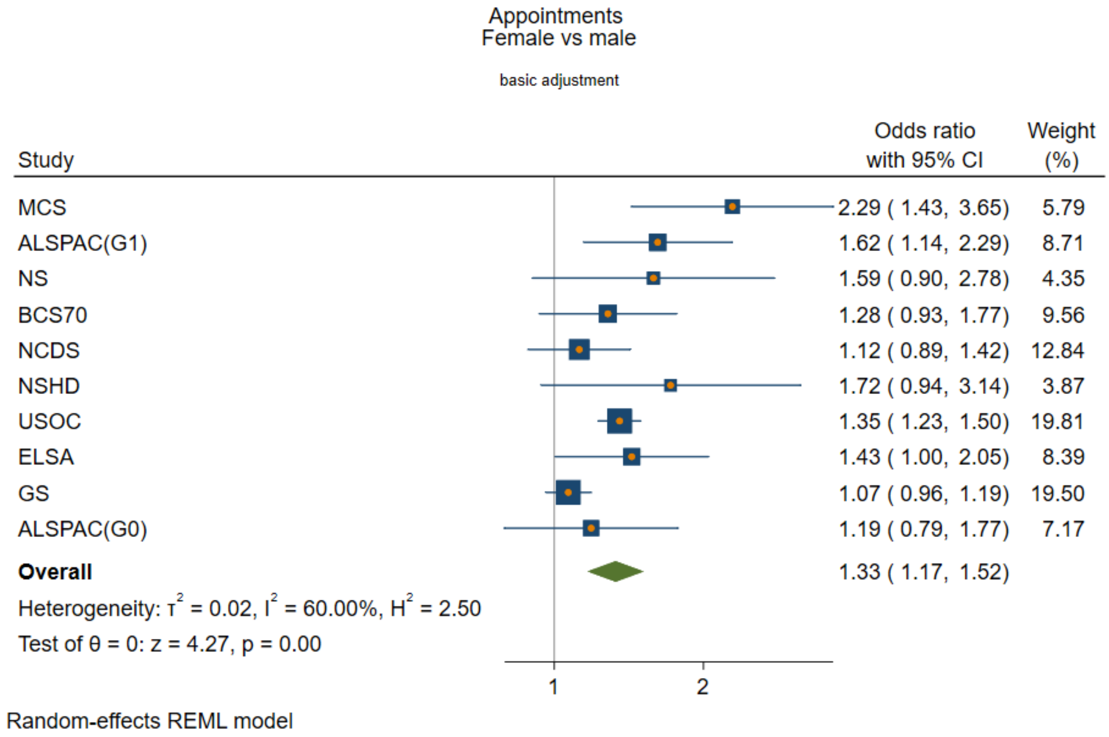

Maddock J, *et al. BMJ Open* 2022; 12:e064981. doi: 10.1136/bmjopen-2022-064981

Full adjustment

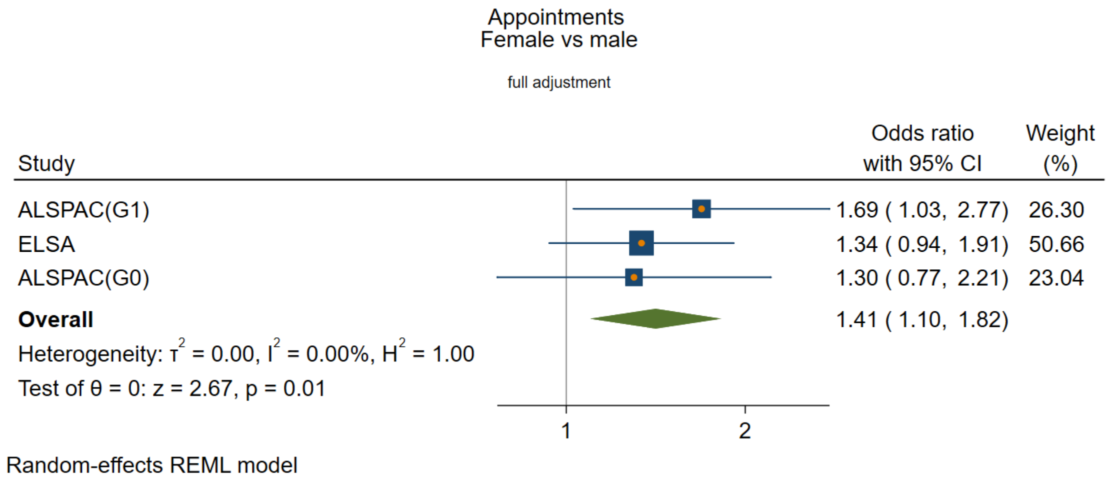

Occupational class

Unadjusted

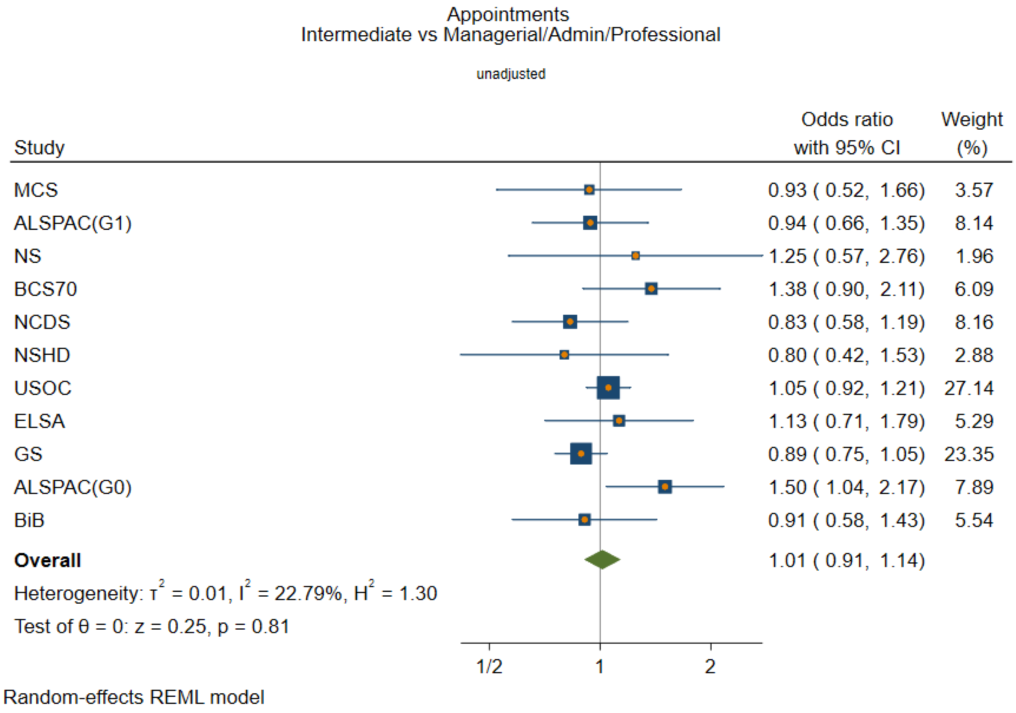

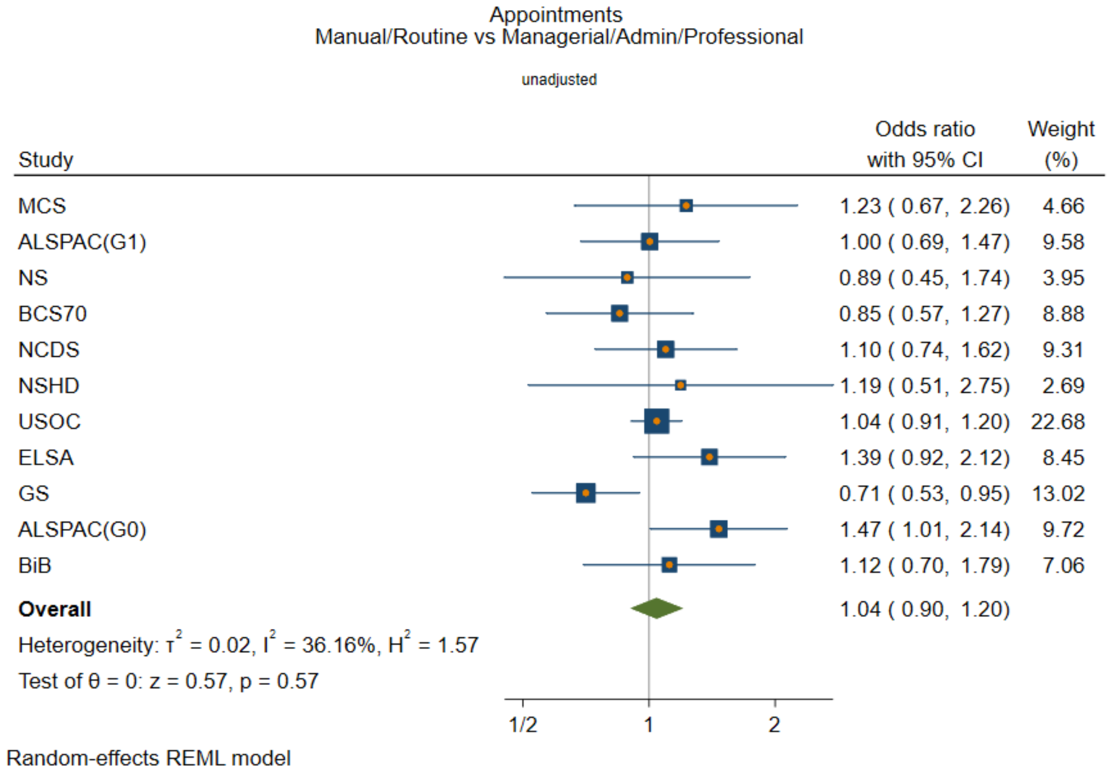

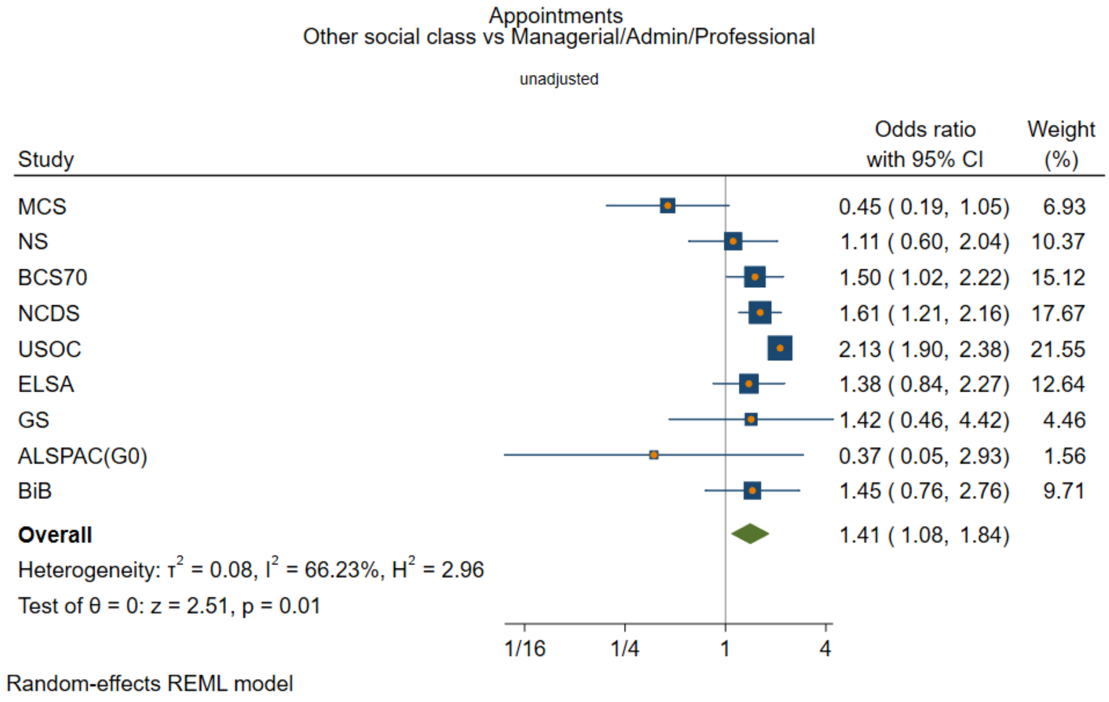

Maddock J, *et al. BMJ Open* 2022; 12:e064981. doi: 10.1136/bmjopen-2022-064981

Basic adjustment

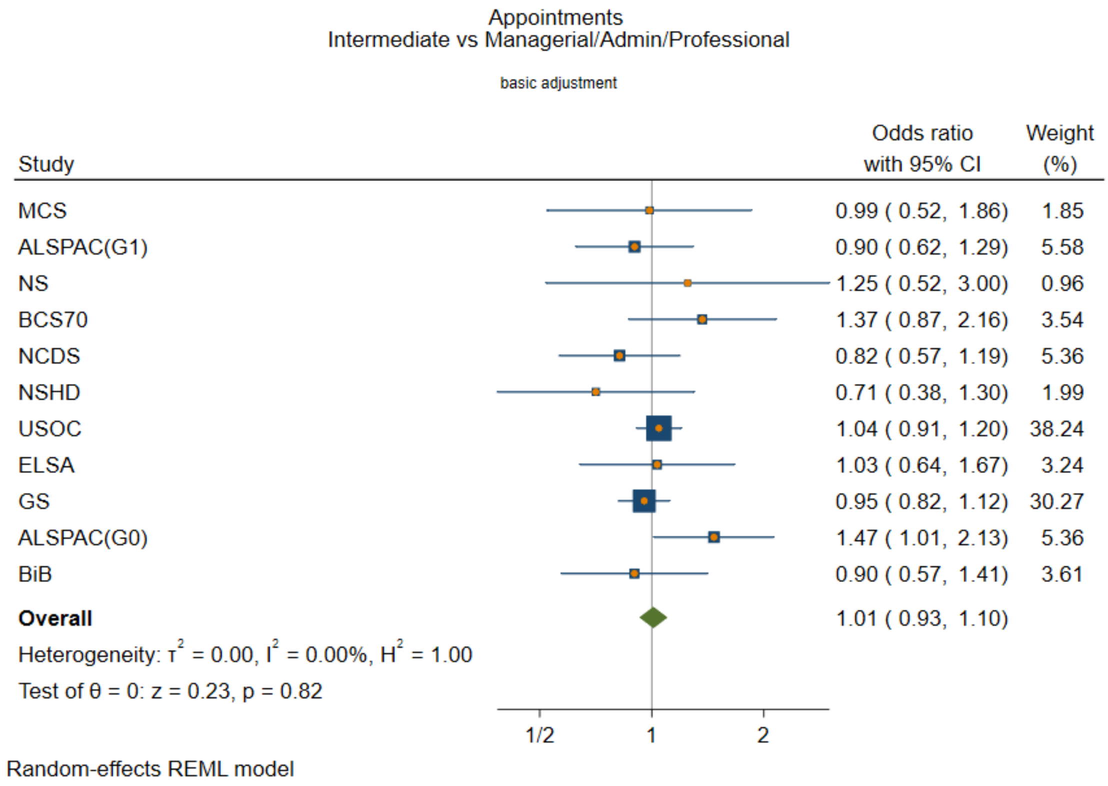

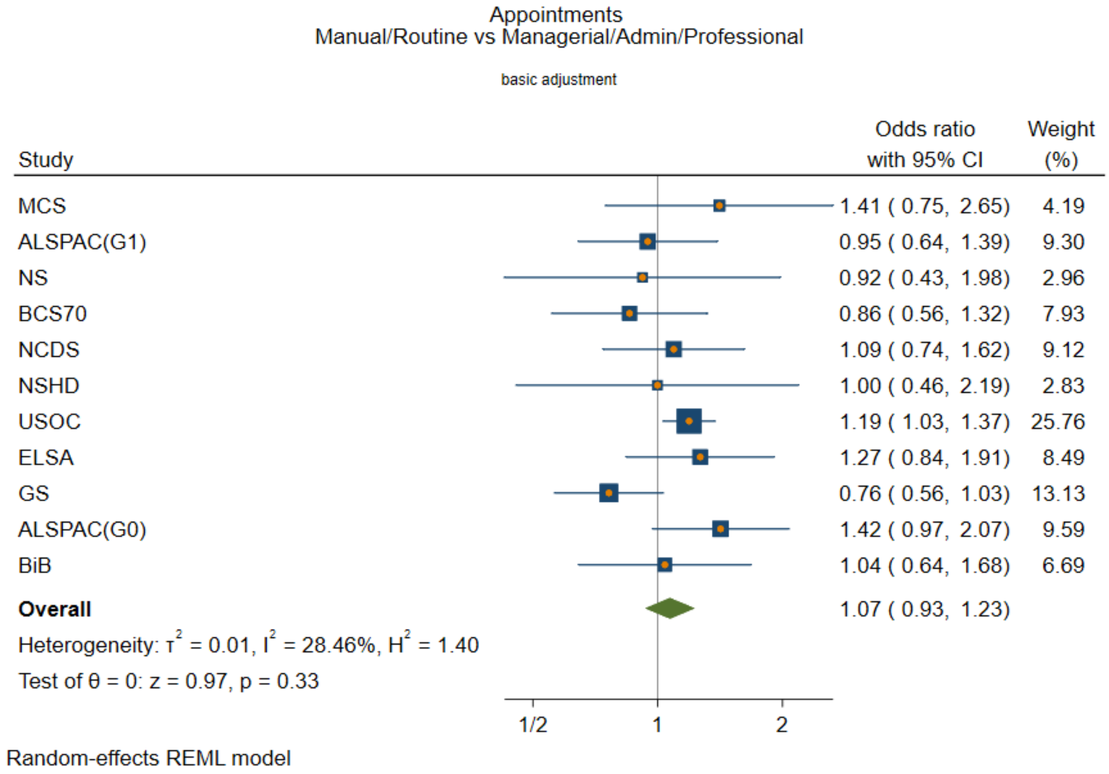

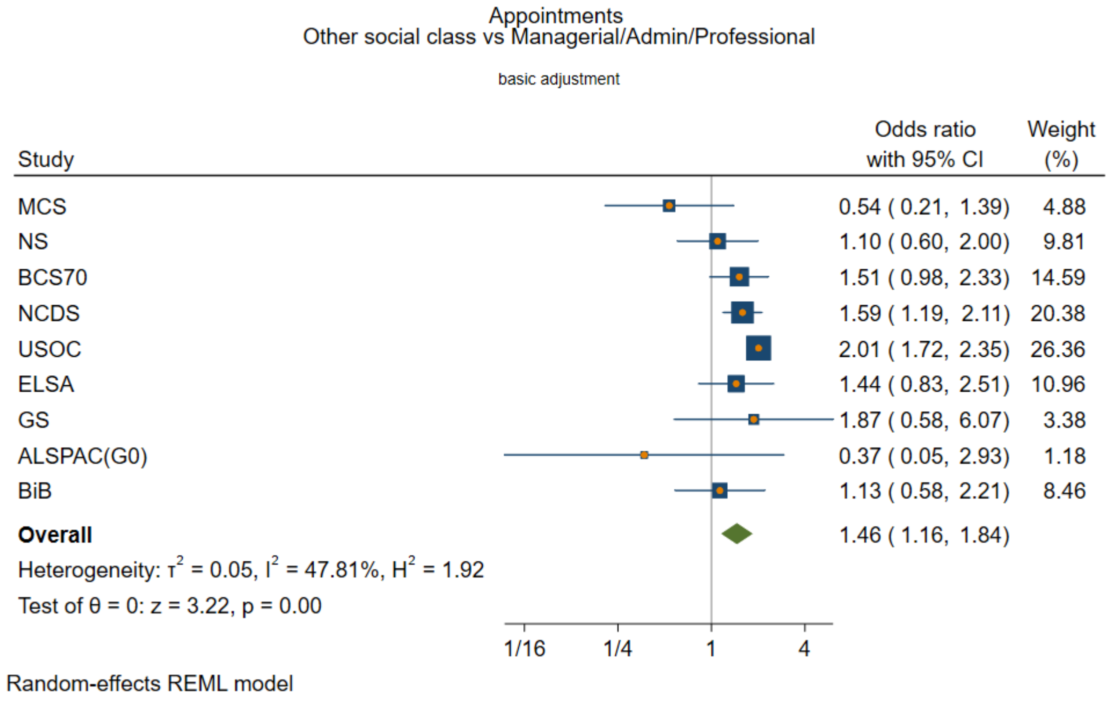

Maddock J, *et al. BMJ Open* 2022; 12:e064981. doi: 10.1136/bmjopen-2022-064981

Full adjustment

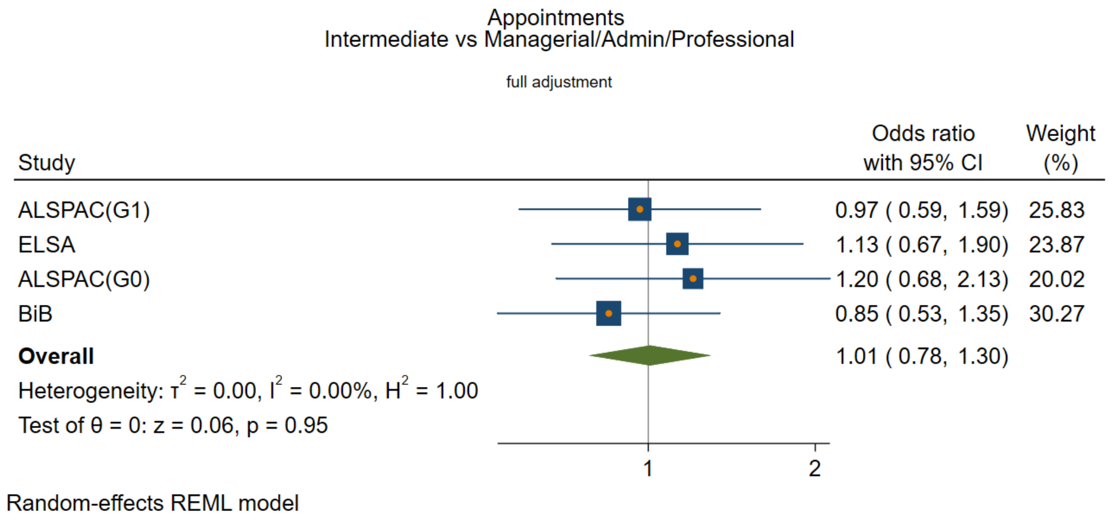

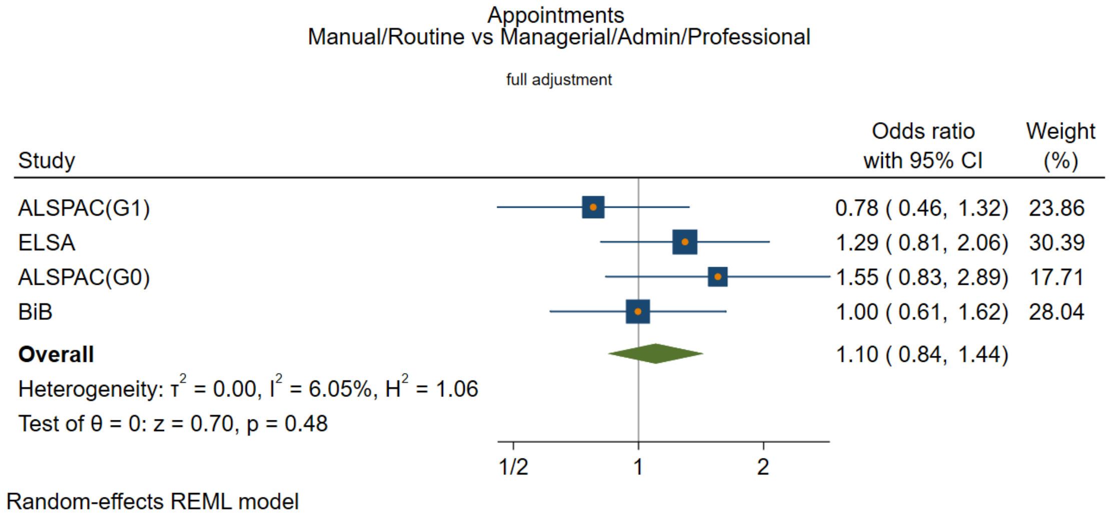
Appointments
Manual/Routine vs Managerial/Admin/Professional
full adjustment
Study
Odds ratio with 95% CI
Weight (%)
ALSPAC(G1) 0.78 (0.46, 1.32) 23.86
ELSA 1.29 (0.81, 2.06) 30.39
ALSPAC(G0) 1.55 (0.83, 2.89) 17.71
BiB 1.00 (0.61, 1.62) 28.04
Overall 1.10 (0.84, 1.44)
Heterogeneity: τ² = 0.00, I² = 6.05%, H² = 1.06
Test of θ = 0: z = 0.70, p = 0.48
1/2
1
2
Random-effects REML model

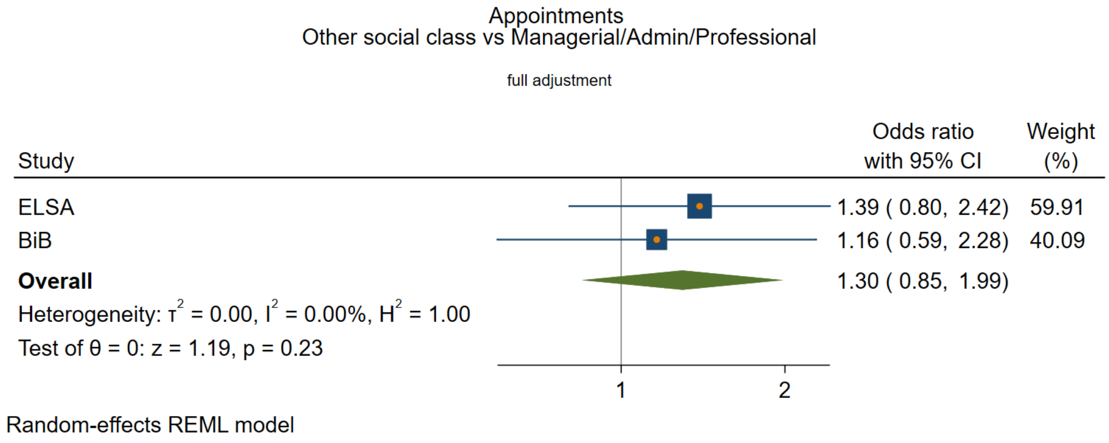
Appointments
Other social class vs Managerial/Admin/Professional
full adjustment
Study
Odds ratio with 95% CI
Weight (%)
ELSA 1.39 (0.80, 2.42) 59.91
BiB 1.16 (0.59, 2.28) 40.09
Overall 1.30 (0.85, 1.99)
Heterogeneity: τ² = 0.00, I² = 0.00%, H² = 1.00
Test of θ = 0: z = 1.19, p = 0.23
1
2
Random-effects REML model

Education
<u>Unadjusted</u>

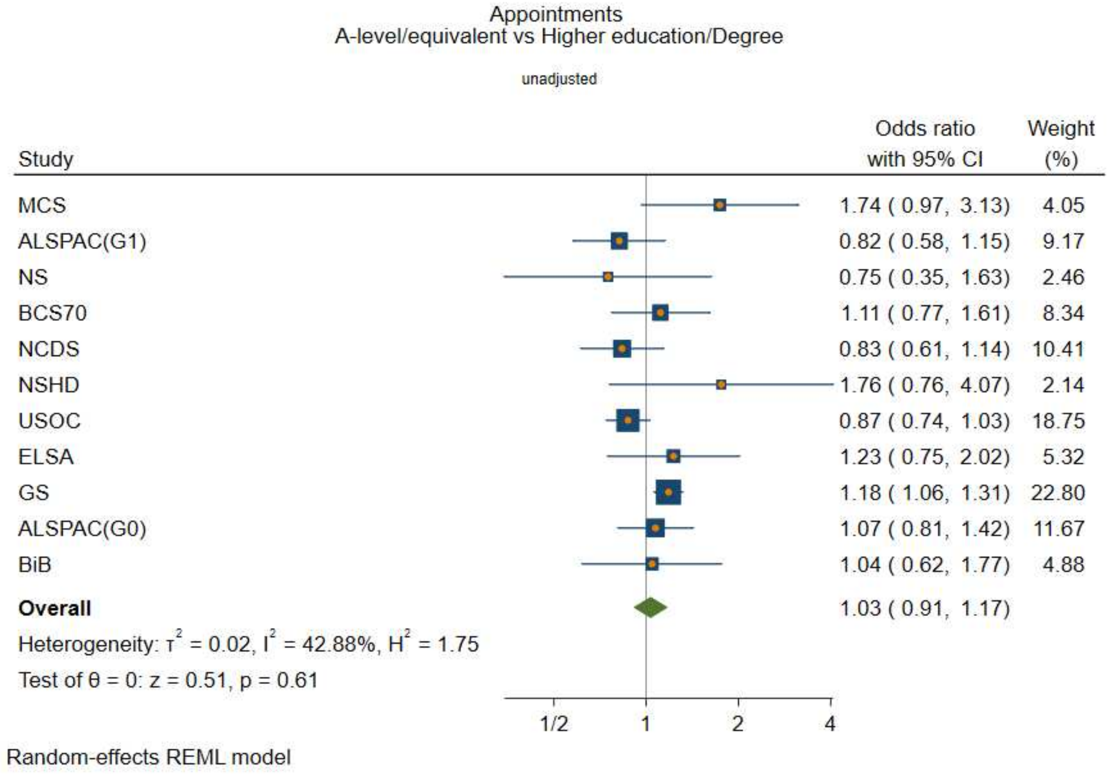

Appointments
GCSE/equivalent vs Higher education/Degree

unadjusted

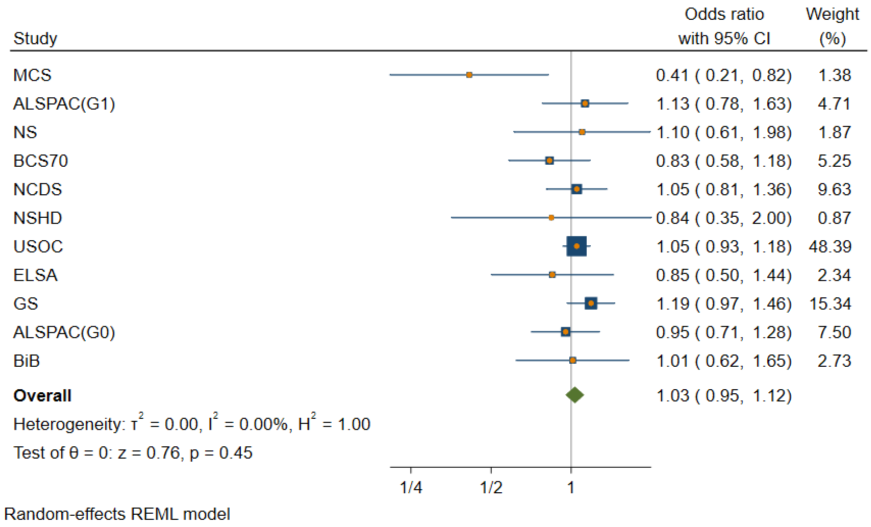

Appointments
<GCSE/equivalent vs Higher education/Degree

unadjusted

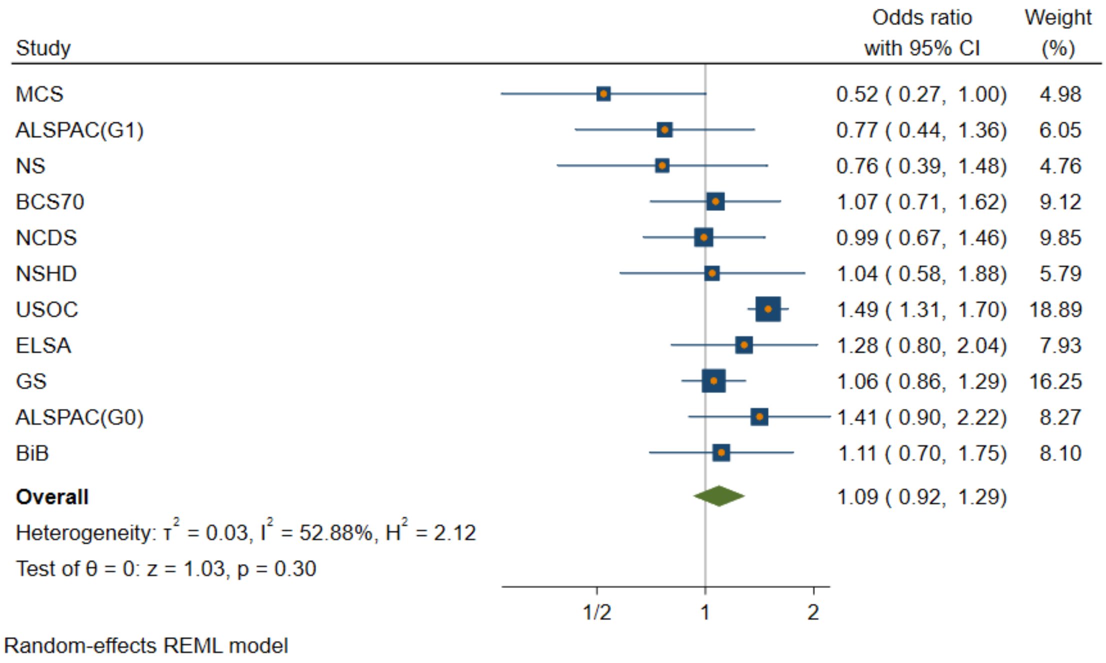

Basic adjustment

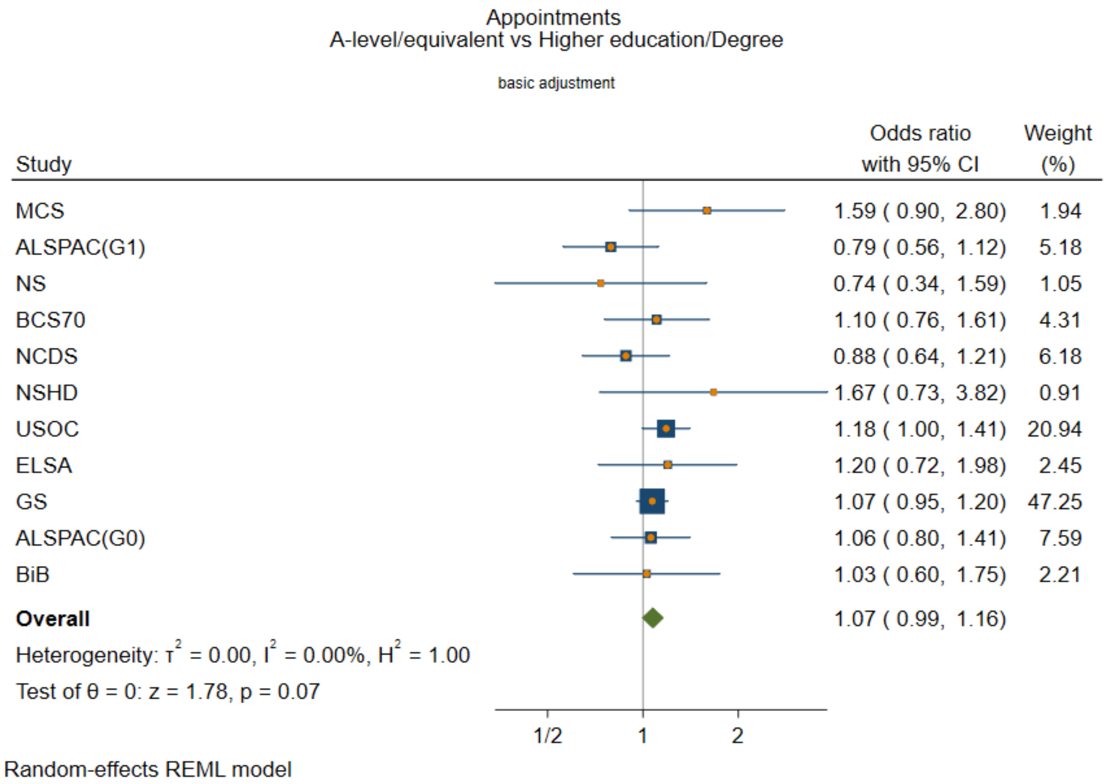

Appointments
GCSE/equivalent vs Higher education/Degree

basic adjustment

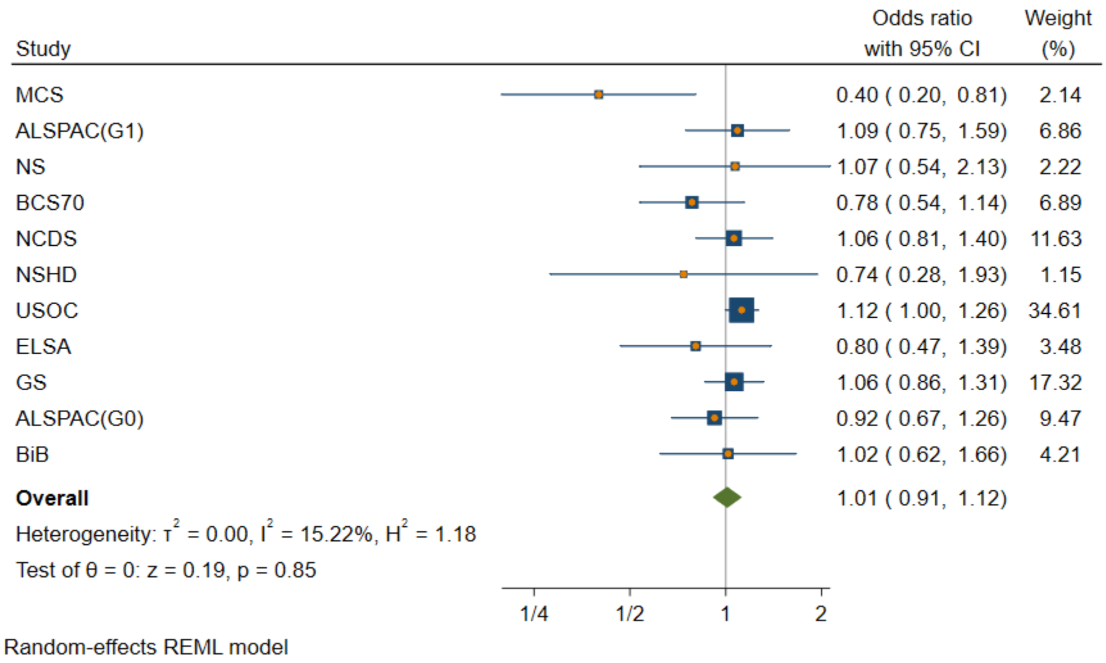

Appointments
<GCSE/equivalent vs Higher education/Degree

basic adjustment

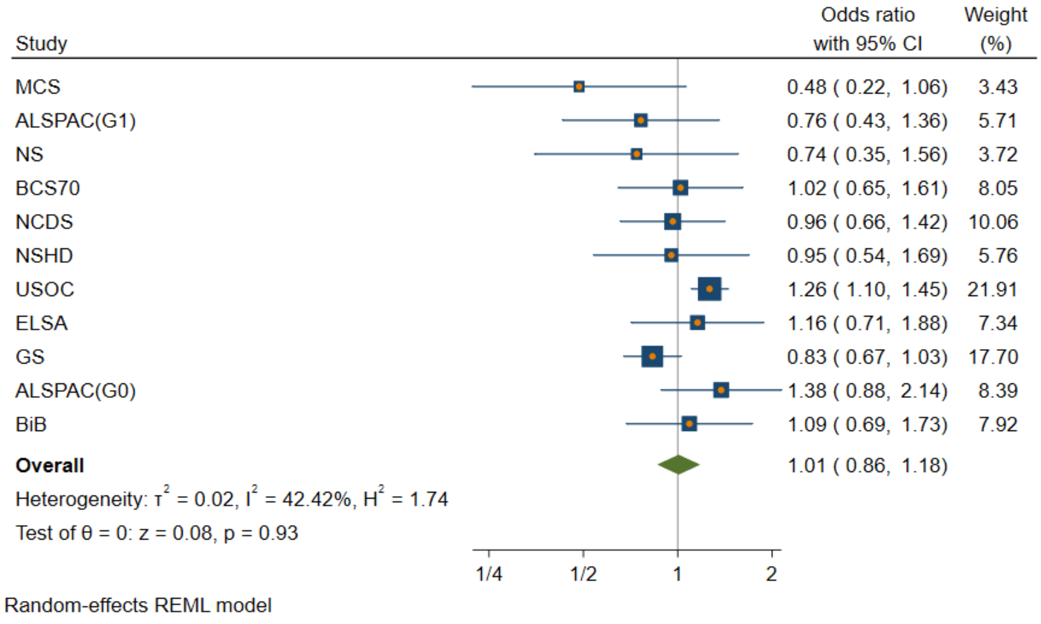

Maddock J, *et al. BMJ Open* 2022; 12:e064981. doi: 10.1136/bmjopen-2022-064981

Full Adjustment

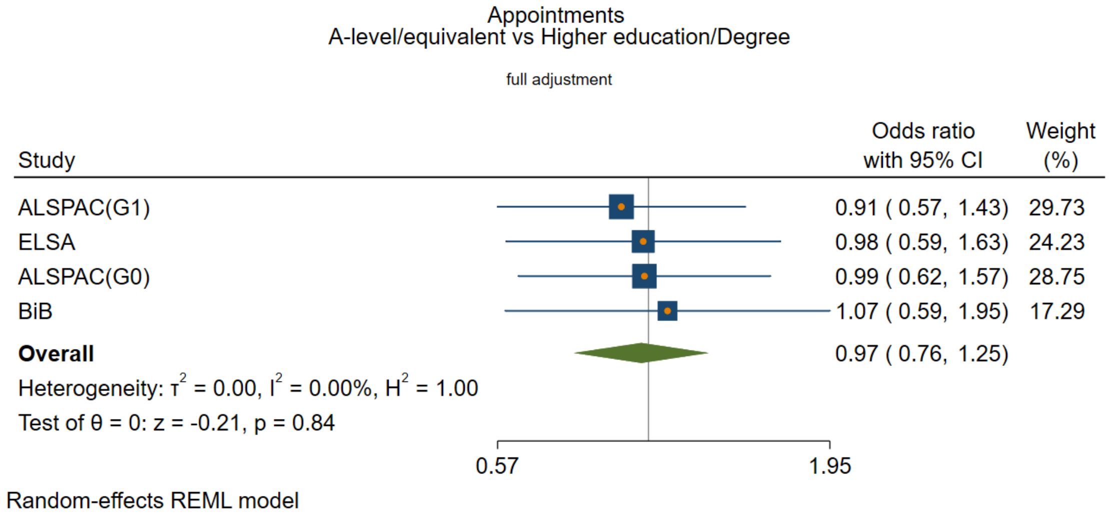

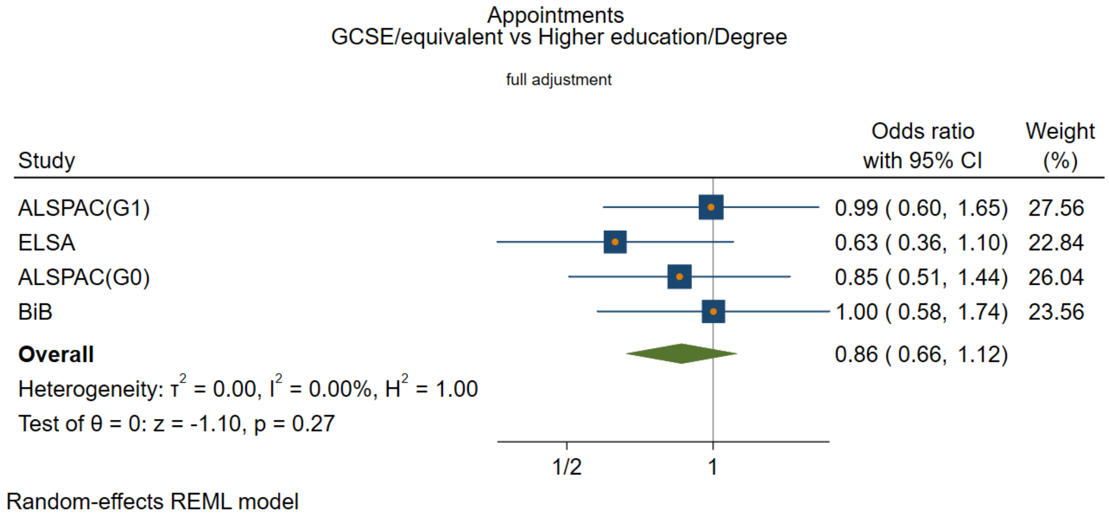

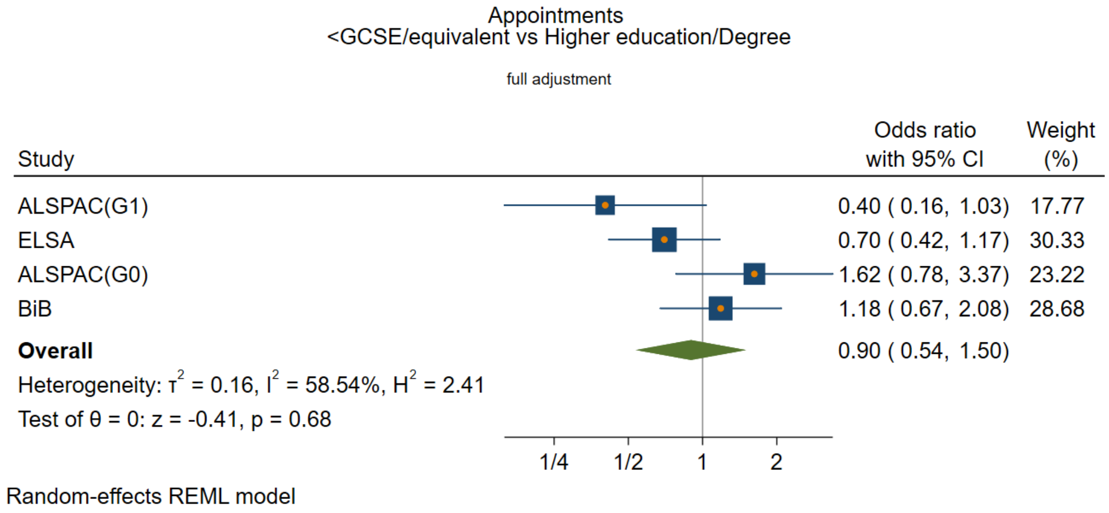

Maddock J, *et al. BMJ Open* 2022; 12:e064981. doi: 10.1136/bmjopen-2022-064981

Age

<u>Unadjusted</u>

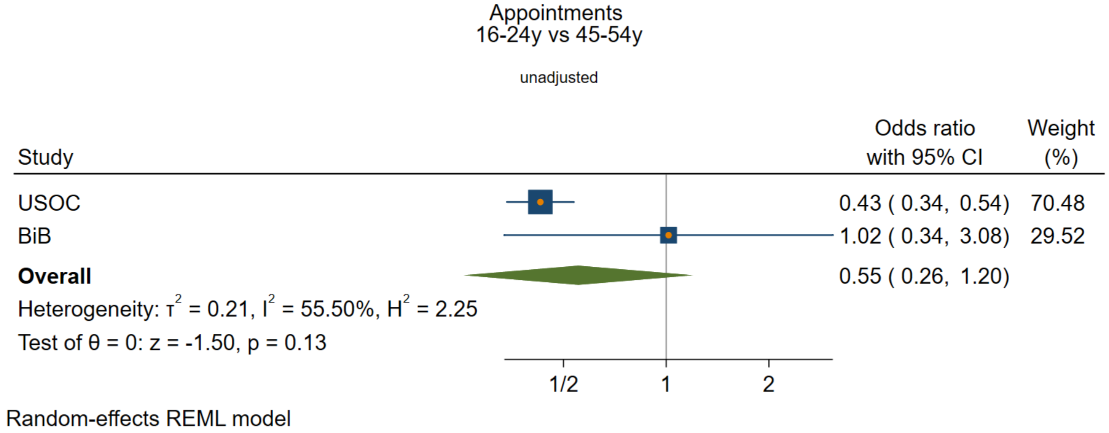

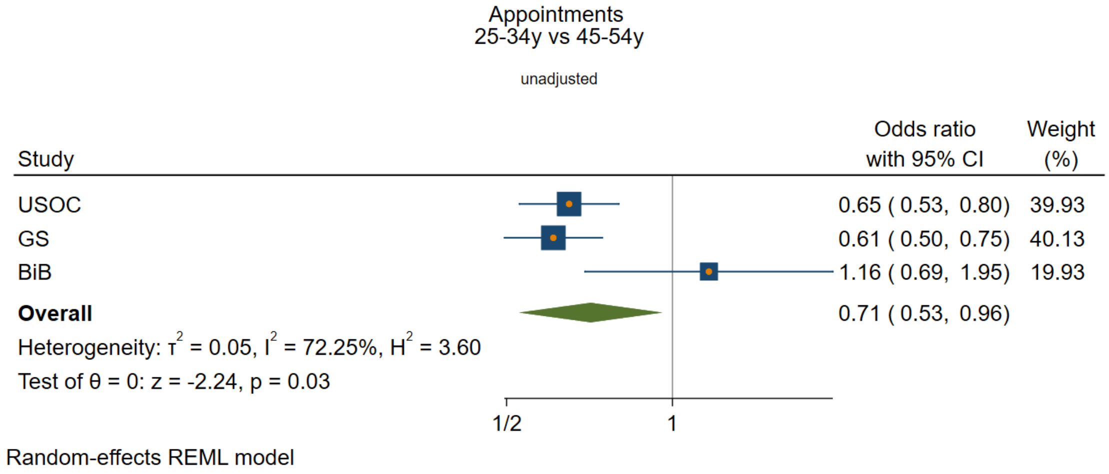

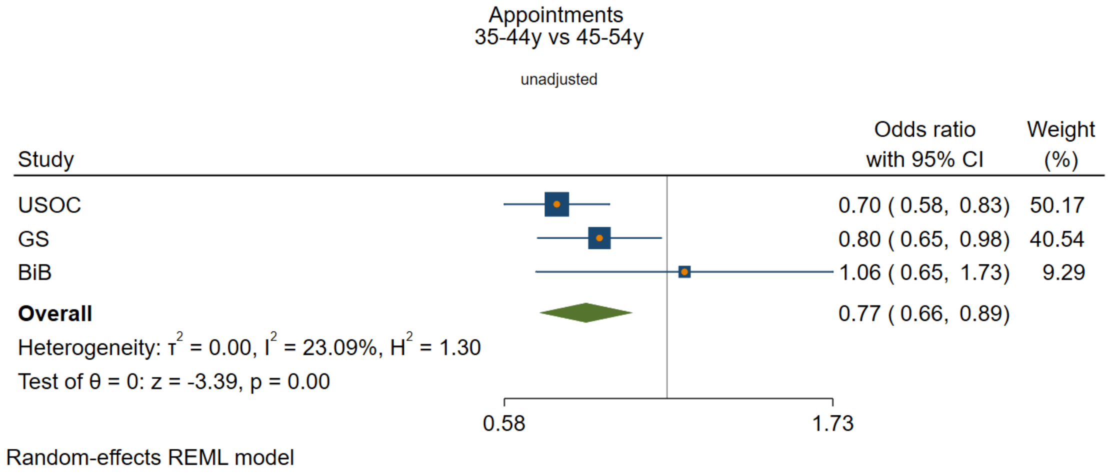

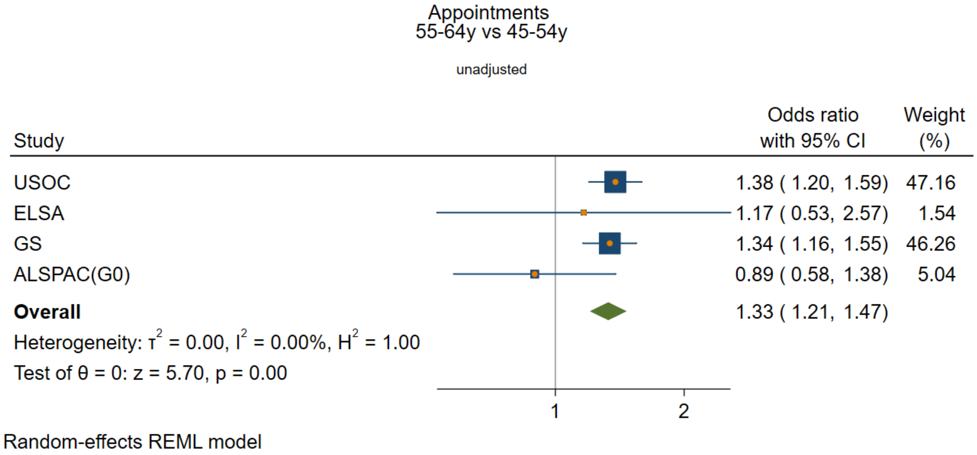

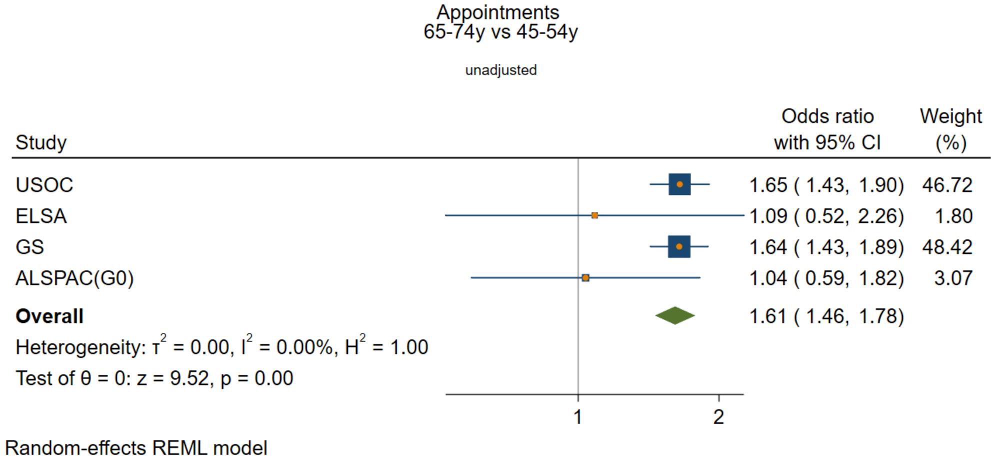

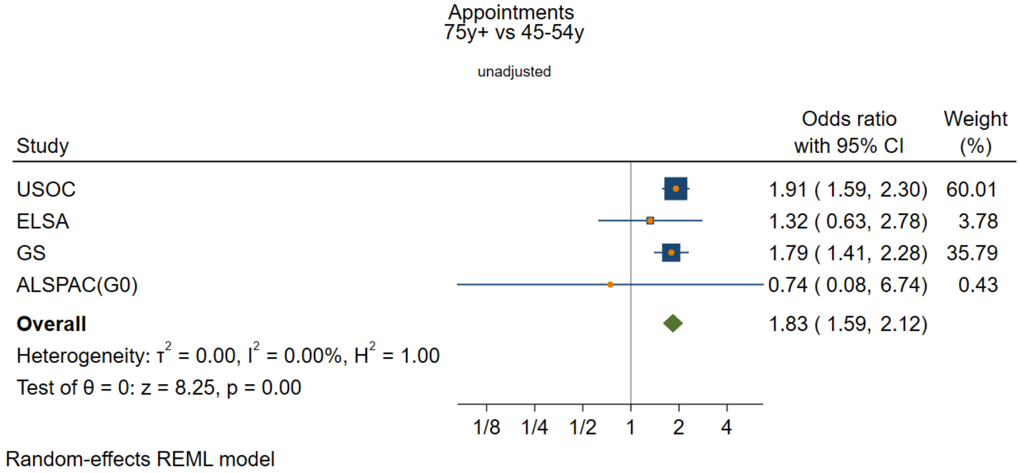

Maddock J, *et al. BMJ Open* 2022; 12:e064981. doi: 10.1136/bmjopen-2022-064981

Basic adjustment

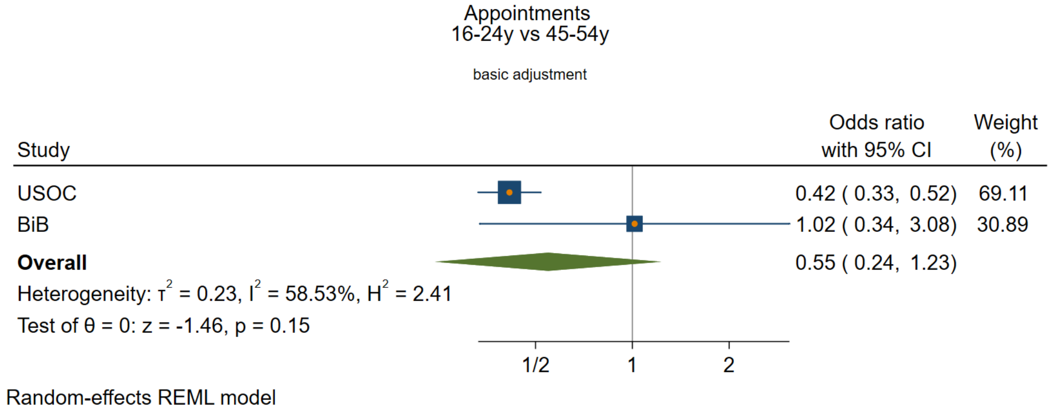

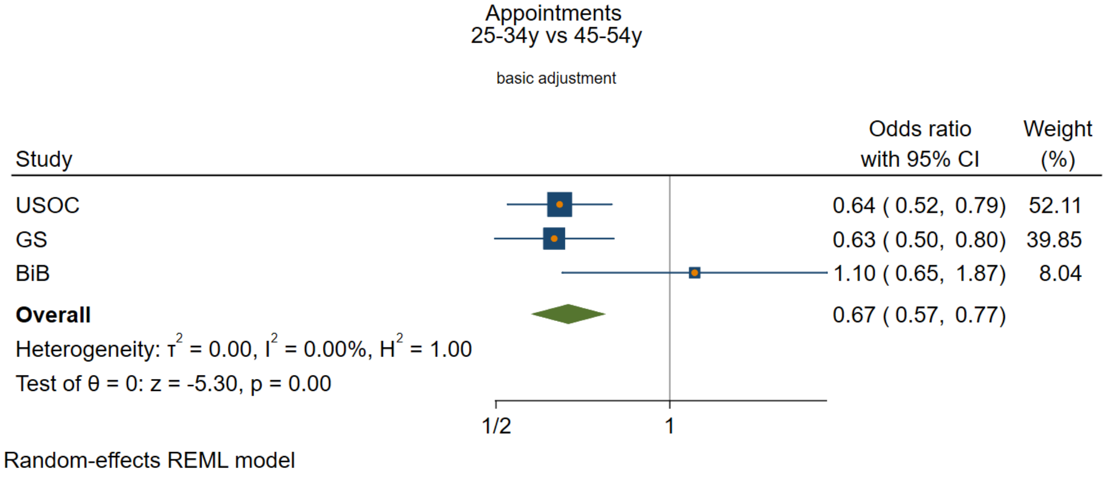

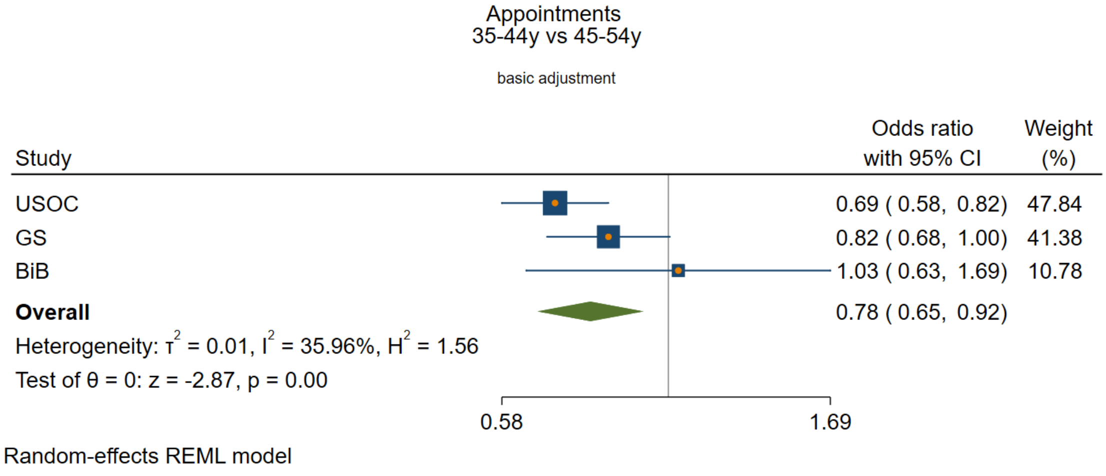

Maddock J, *et al. BMJ Open* 2022; 12:e064981. doi: 10.1136/bmjopen-2022-064981

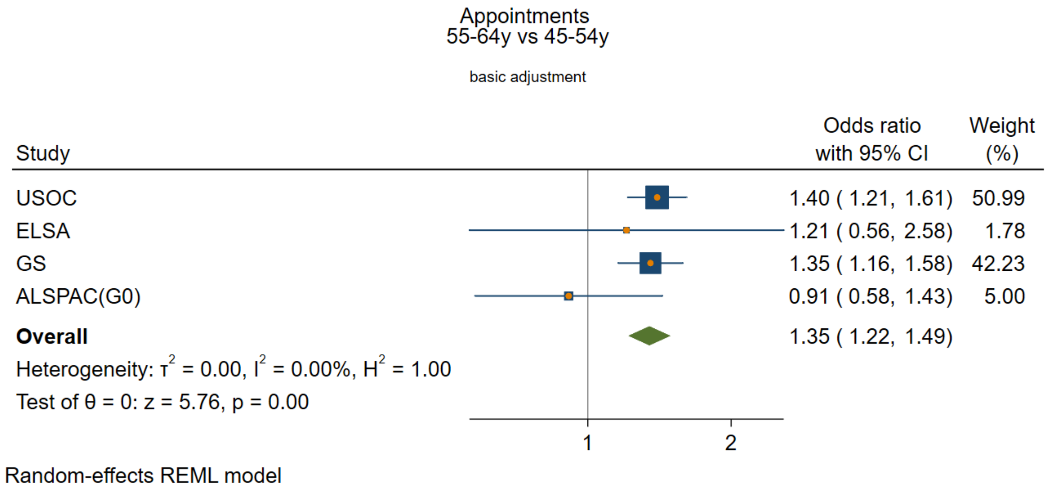

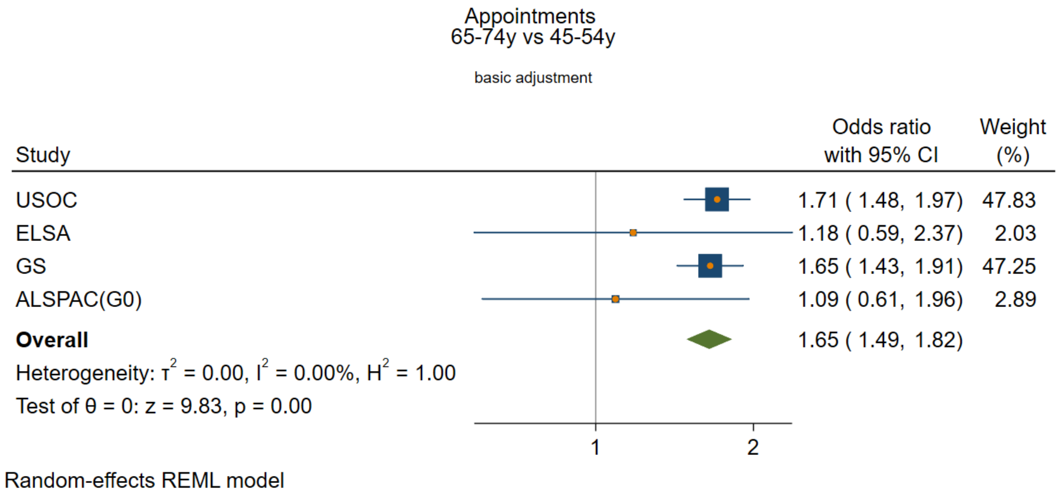

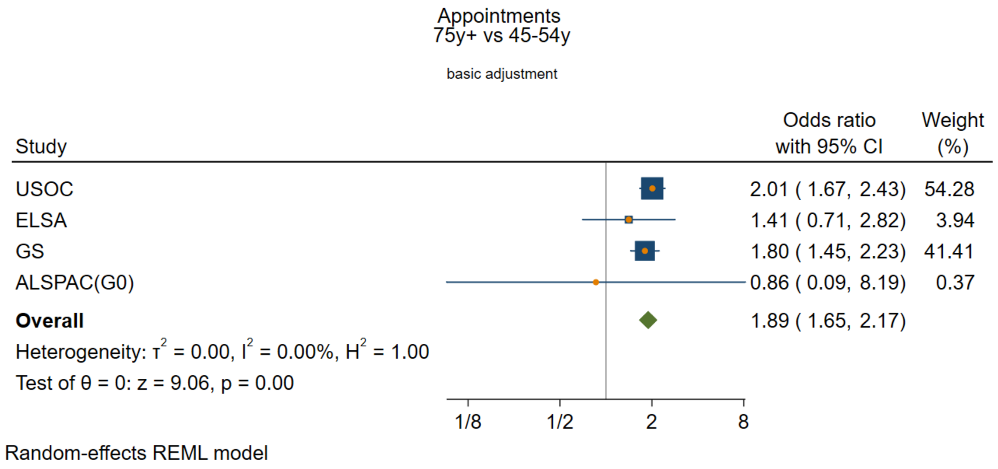

Maddock J, *et al. BMJ Open* 2022; 12:e064981. doi: 10.1136/bmjopen-2022-064981

Full adjustment

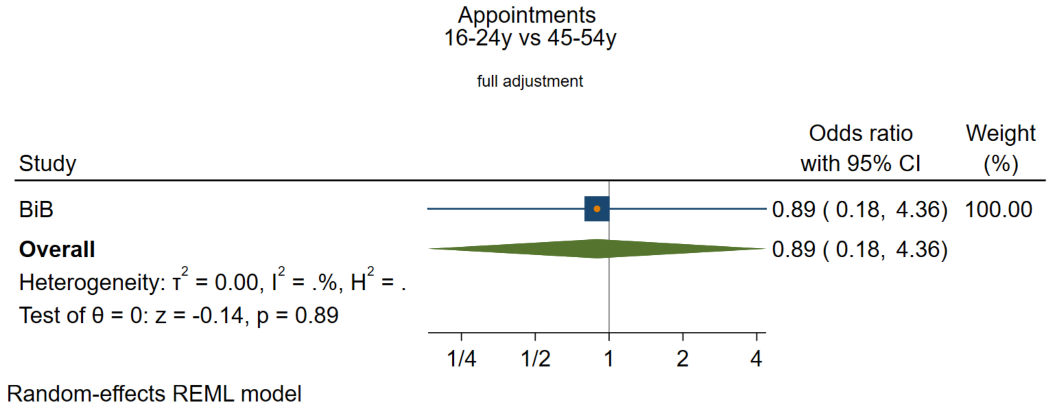

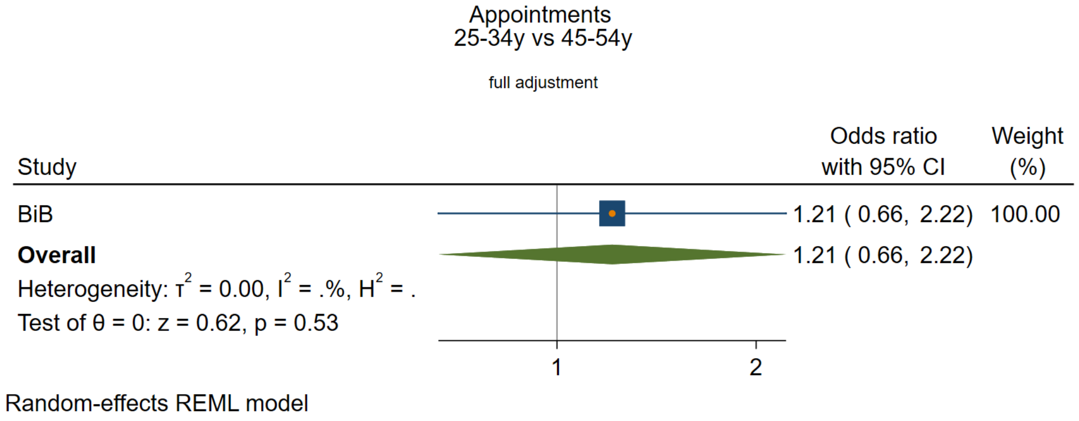

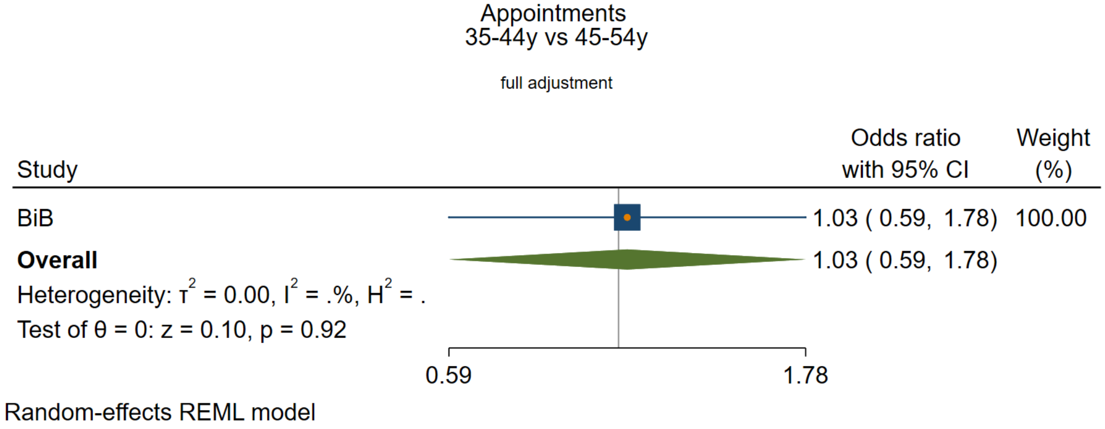

Maddock J, *et al. BMJ Open* 2022; 12:e064981. doi: 10.1136/bmjopen-2022-064981

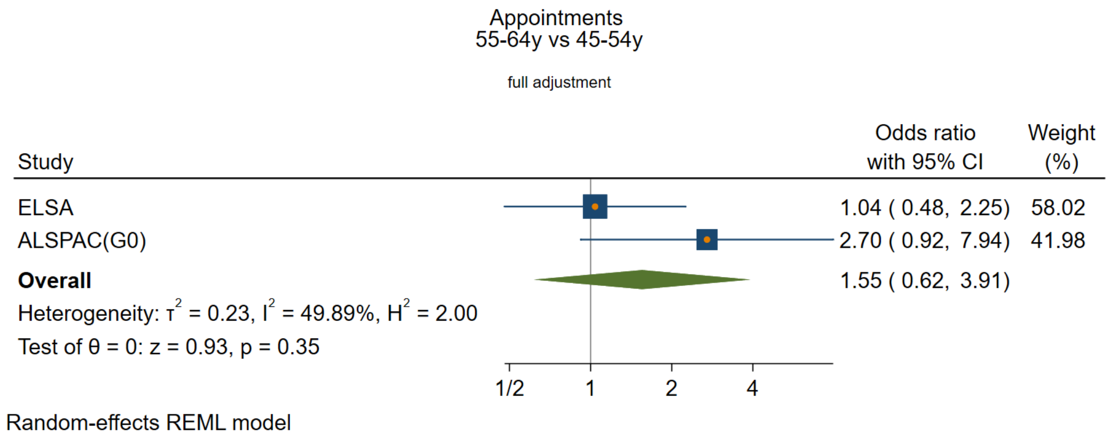
Appointments
55-64y vs 45-54y
full adjustment
Study
Odds ratio with 95% CI
Weight (%)
ELSA 1.04 (0.48, 2.25) 58.02
ALSPAC(G0) 2.70 (0.92, 7.94) 41.98
Overall 1.55 (0.62, 3.91)
Heterogeneity: τ² = 0.23, I² = 49.89%, H² = 2.00
Test of θ = 0: z = 0.93, p = 0.35
1/2
1
2
4
Random-effects REML model

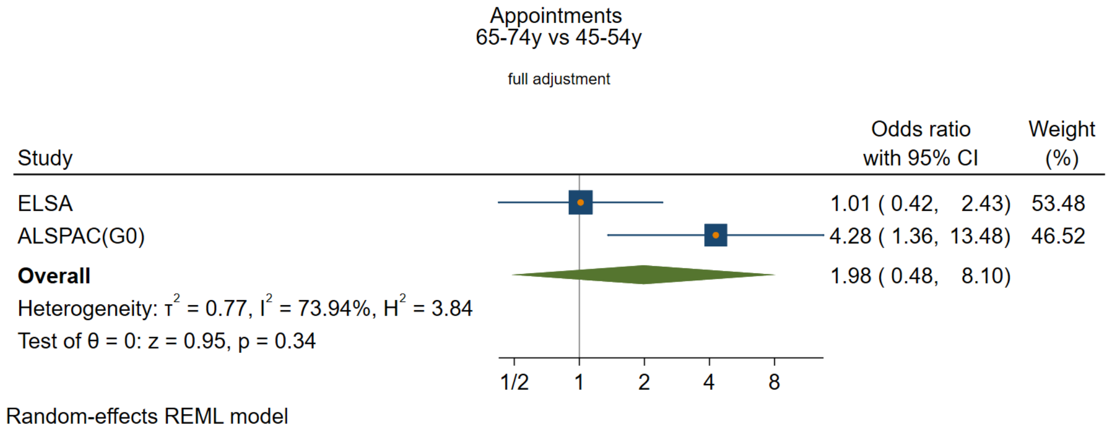
Appointments
65-74y vs 45-54y
full adjustment
Study
Odds ratio with 95% CI
Weight (%)
ELSA 1.01 (0.42, 2.43) 53.48
ALSPAC(G0) 4.28 (1.36, 13.48) 46.52
Overall 1.98 (0.48, 8.10)
Heterogeneity: τ² = 0.77, I² = 73.94%, H² = 3.84
Test of θ = 0: z = 0.95, p = 0.34
1/2
1
2
4
8
Random-effects REML model

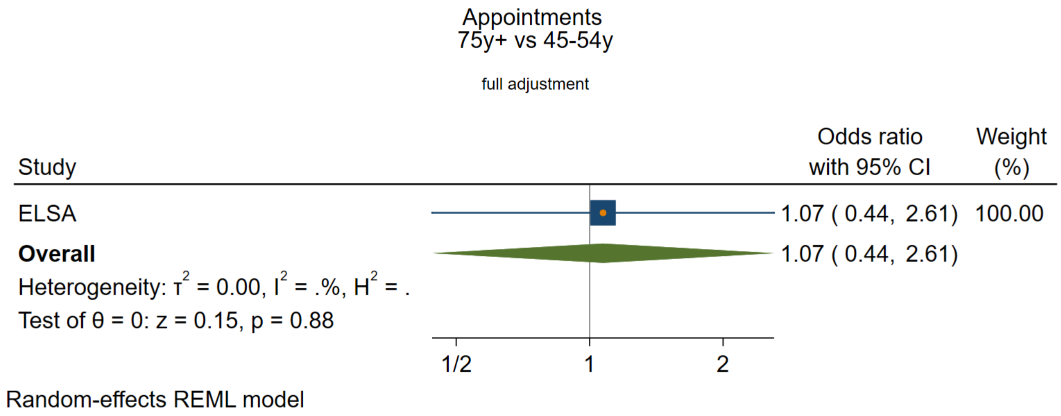
Appointments
75y+ vs 45-54y
full adjustment
Study
Odds ratio with 95% CI
Weight (%)
ELSA 1.07 (0.44, 2.61) 100.00
Overall 1.07 (0.44, 2.61)
Heterogeneity: τ² = 0.00, I² = .%, H² = .
Test of θ = 0: z = 0.15, p = 0.88
1/2
1
2
Random-effects REML model

Ethnicity
Unadjusted

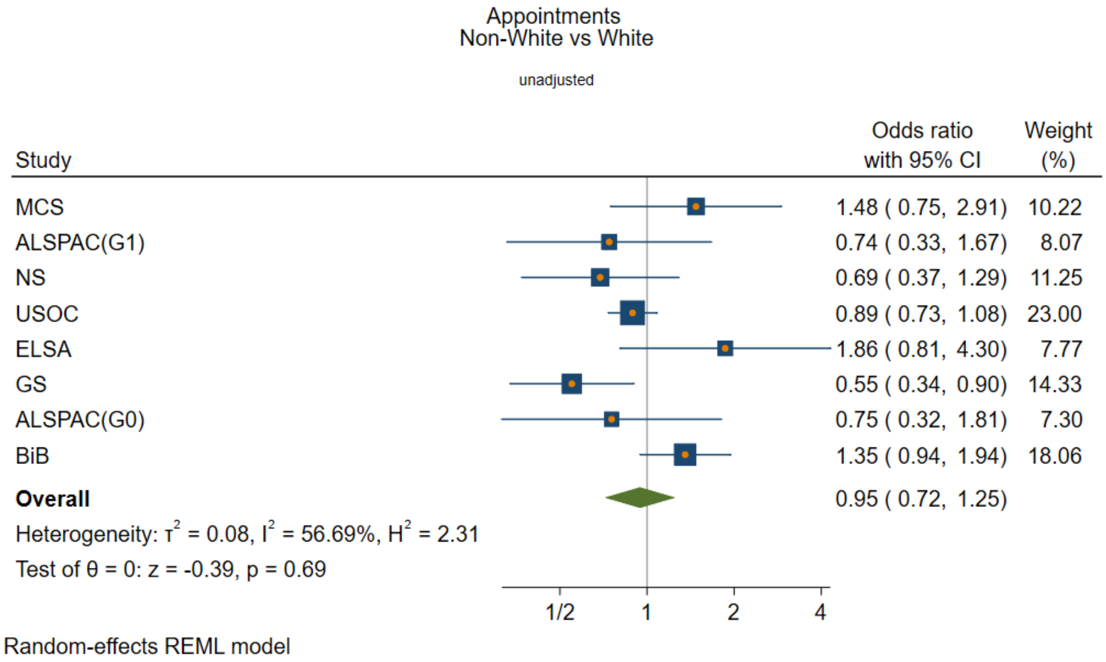

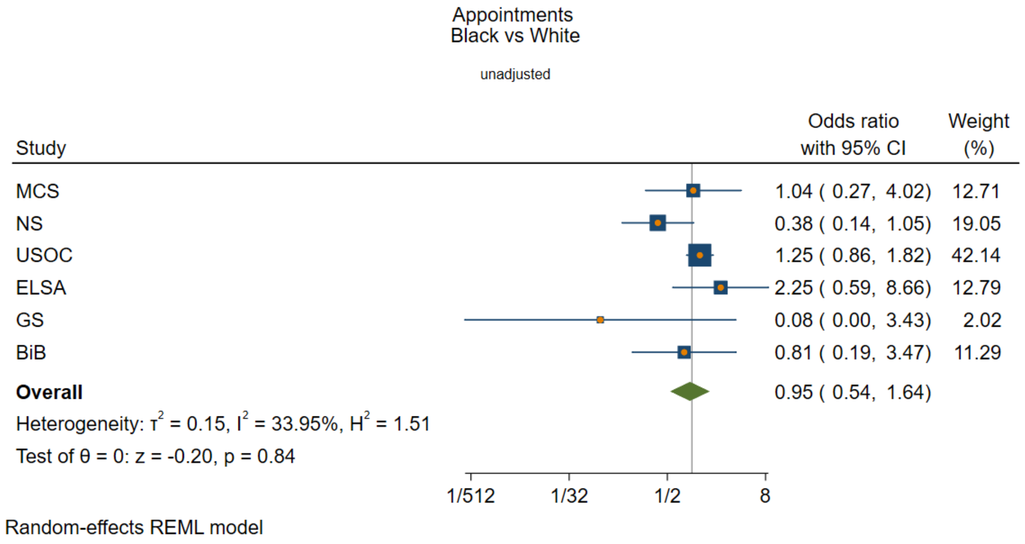

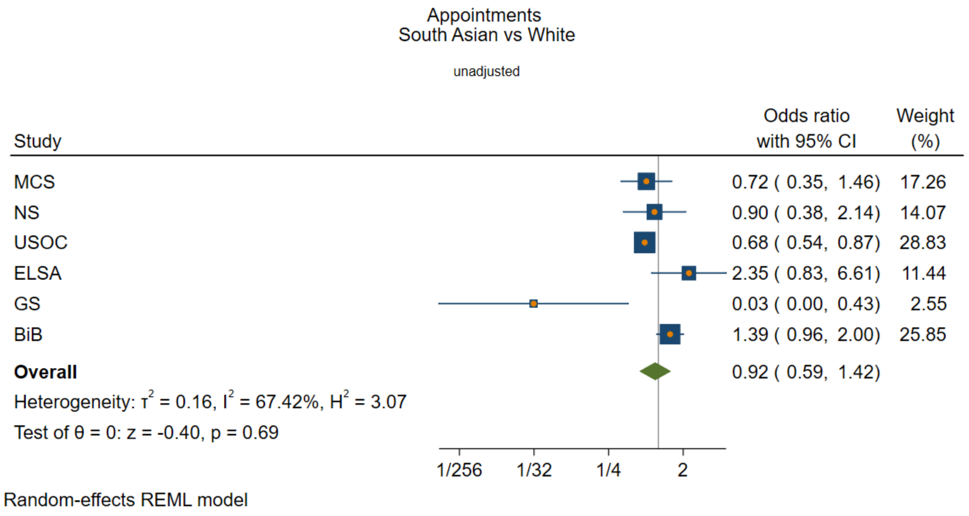

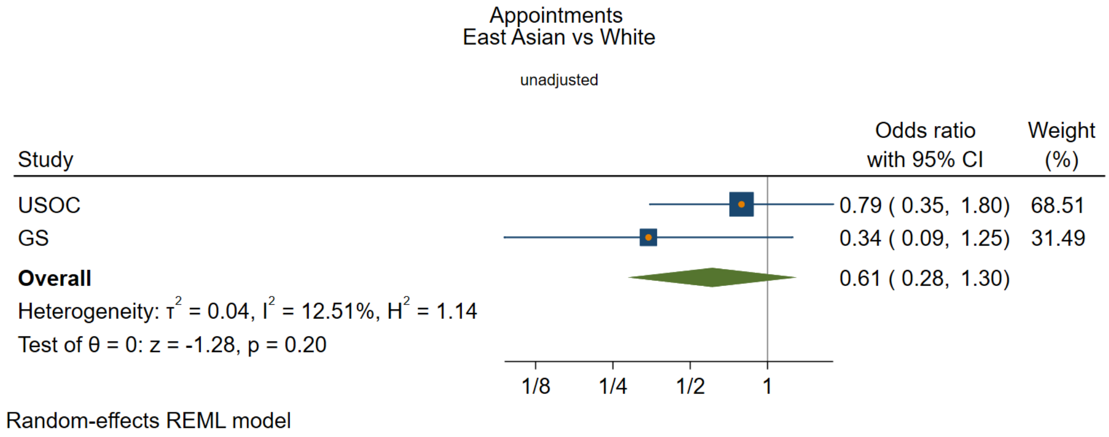

Maddock J, *et al. BMJ Open* 2022; 12:e064981. doi: 10.1136/bmjopen-2022-064981

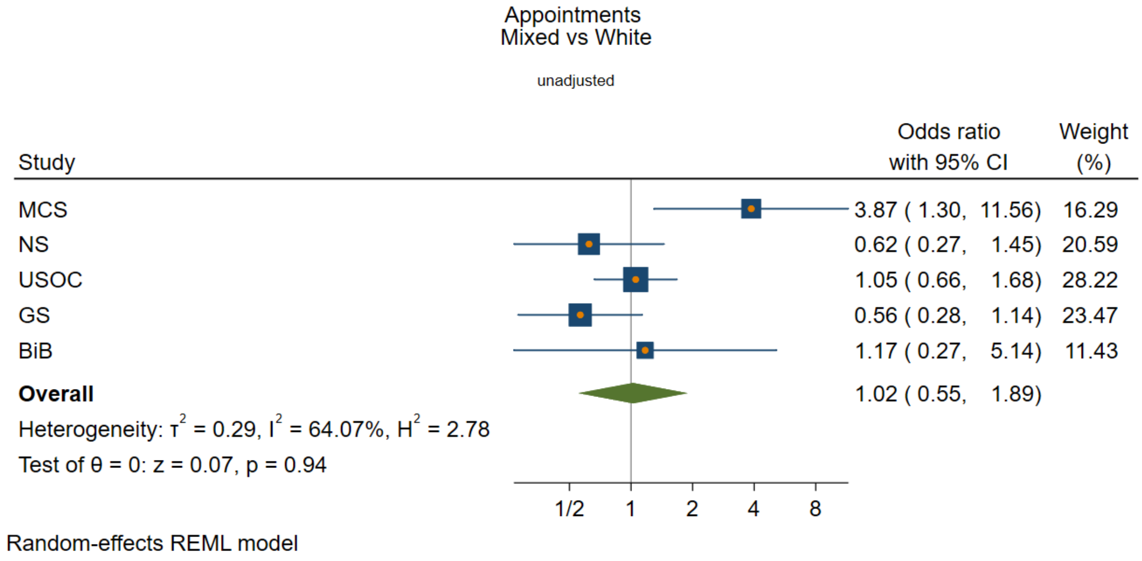
Appointments
Mixed vs White
unadjusted
Study
Odds ratio with 95% CI
Weight (%)
MCS 3.87 (1.30, 11.56) 16.29
NS 0.62 (0.27, 1.45) 20.59
USOC 1.05 (0.66, 1.68) 28.22
GS 0.56 (0.28, 1.14) 23.47
BiB 1.17 (0.27, 5.14) 11.43
Overall 1.02 (0.55, 1.89)
Heterogeneity: τ² = 0.29, I² = 64.07%, H² = 2.78
Test of θ = 0: z = 0.07, p = 0.94
1/2
1
2
4
8
Random-effects REML model

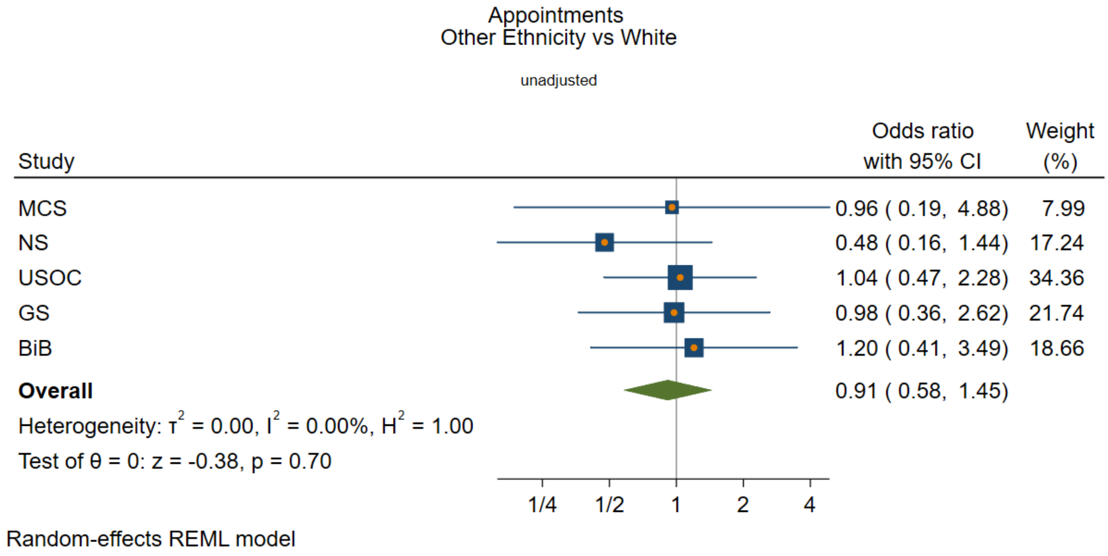
Appointments
Other Ethnicity vs White
unadjusted
Study
Odds ratio with 95% CI
Weight (%)
MCS 0.96 (0.19, 4.88) 7.99
NS 0.48 (0.16, 1.44) 17.24
USOC 1.04 (0.47, 2.28) 34.36
GS 0.98 (0.36, 2.62) 21.74
BiB 1.20 (0.41, 3.49) 18.66
Overall 0.91 (0.58, 1.45)
Heterogeneity: τ² = 0.00, I² = 0.00%, H² = 1.00
Test of θ = 0: z = -0.38, p = 0.70
1/4
1/2
1
2
4
Random-effects REML model

Basic adjustment

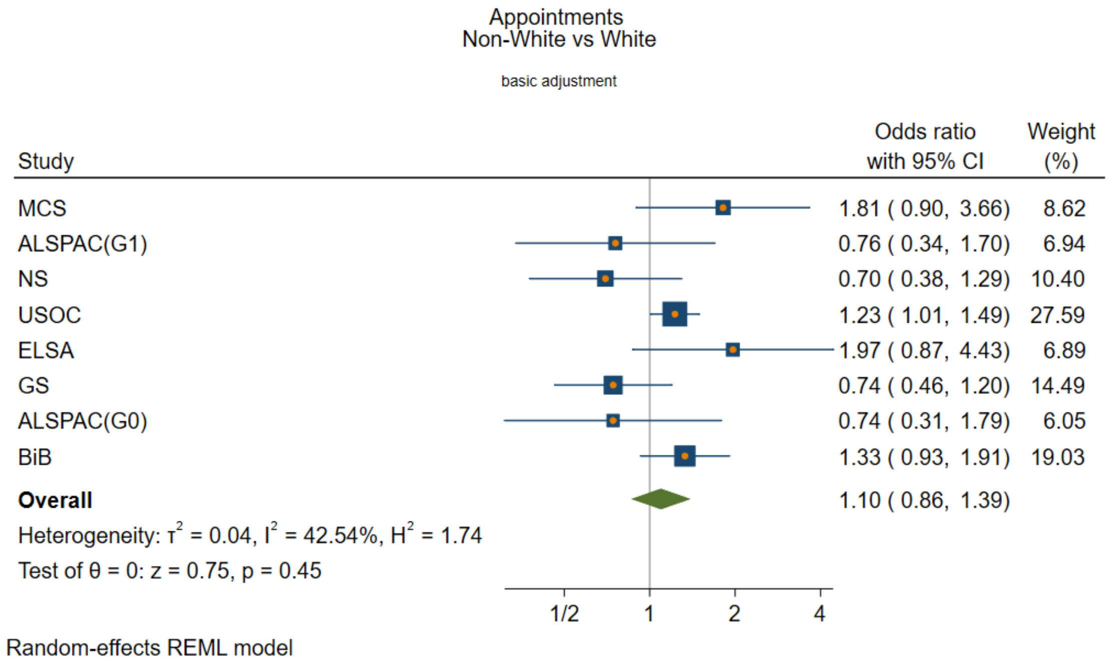

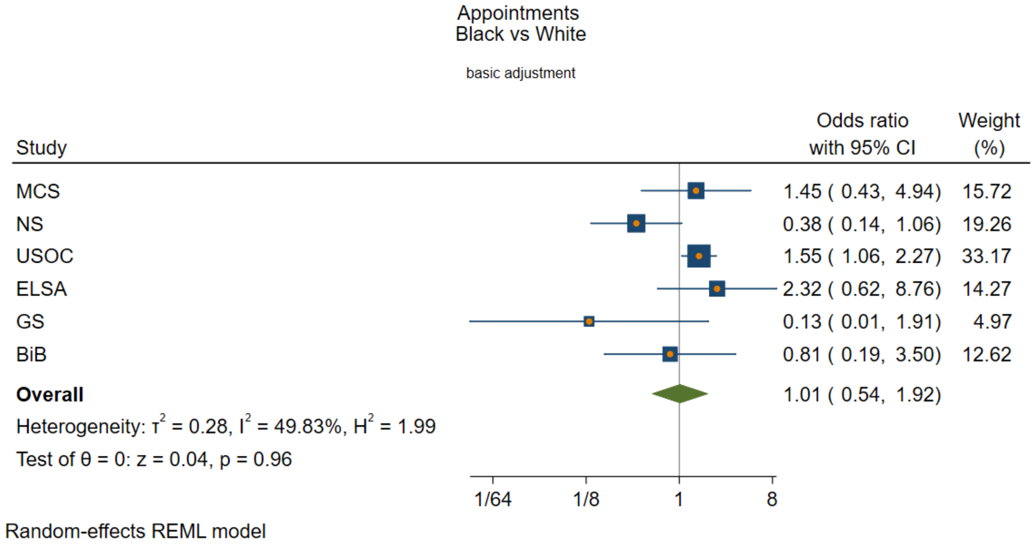
Appointments
Black vs White
basic adjustment
Study
Odds ratio with 95% CI
Weight (%)
MCS
1.45 (0.43, 4.94)
15.72
NS
0.38 (0.14, 1.06)
19.26
USOC
1.55 (1.06, 2.27)
33.17
ELSA
2.32 (0.62, 8.76)
14.27
GS
0.13 (0.01, 1.91)
4.97
BiB
0.81 (0.19, 3.50)
12.62
Overall
1.01 (0.54, 1.92)
Heterogeneity: τ² = 0.28, I² = 49.83%, H² = 1.99
Test of θ = 0: z = 0.04, p = 0.96
1/64
1/8
1
8
Random-effects REML model

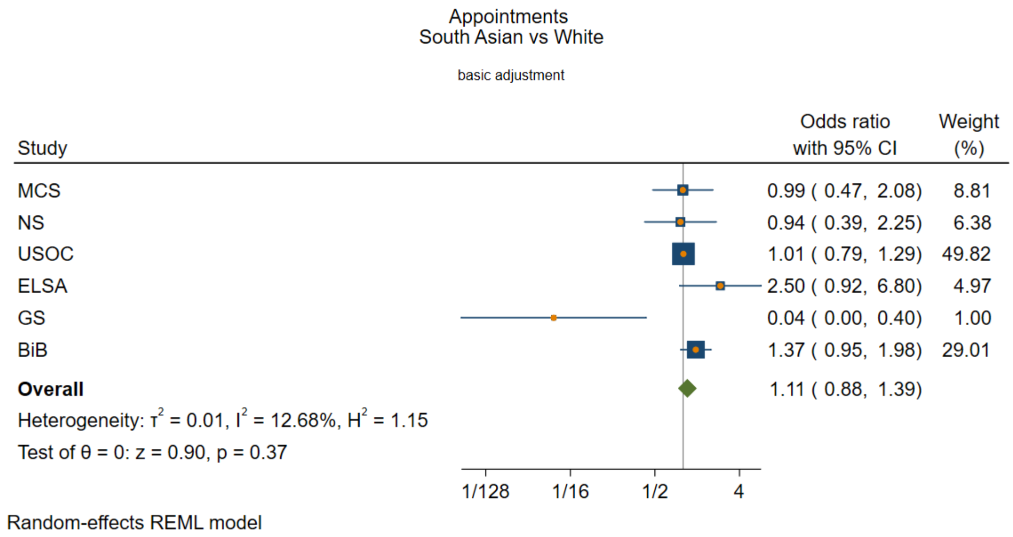
Appointments
South Asian vs White
basic adjustment
Study
Odds ratio with 95% CI
Weight (%)
MCS
0.99 (0.47, 2.08)
8.81
NS
0.94 (0.39, 2.25)
6.38
USOC
1.01 (0.79, 1.29)
49.82
ELSA
2.50 (0.92, 6.80)
4.97
GS
0.04 (0.00, 0.40)
1.00
BiB
1.37 (0.95, 1.98)
29.01
Overall
1.11 (0.88, 1.39)
Heterogeneity: τ² = 0.01, I² = 12.68%, H² = 1.15
Test of θ = 0: z = 0.90, p = 0.37
1/128
1/16
1/2
4
Random-effects REML model

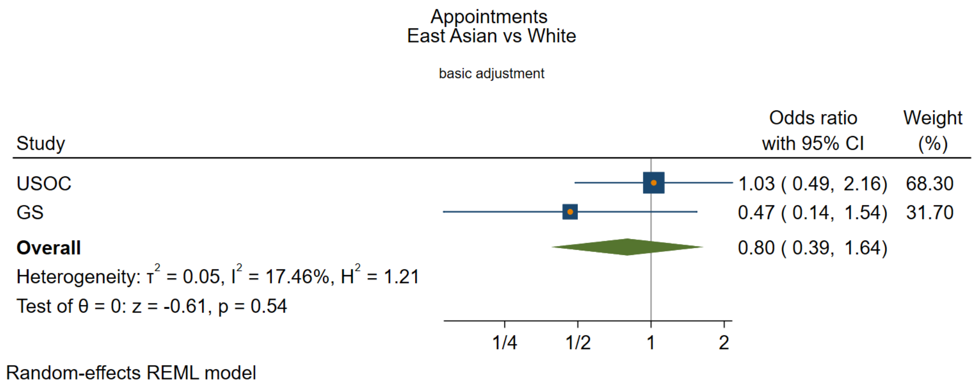
Appointments
East Asian vs White
basic adjustment
Study
Odds ratio with 95% CI
Weight (%)
USOC
1.03 (0.49, 2.16)
68.30
GS
0.47 (0.14, 1.54)
31.70
Overall
0.80 (0.39, 1.64)
Heterogeneity: τ² = 0.05, I² = 17.46%, H² = 1.21
Test of θ = 0: z = -0.61, p = 0.54
1/4
1/2
1
2
Random-effects REML model

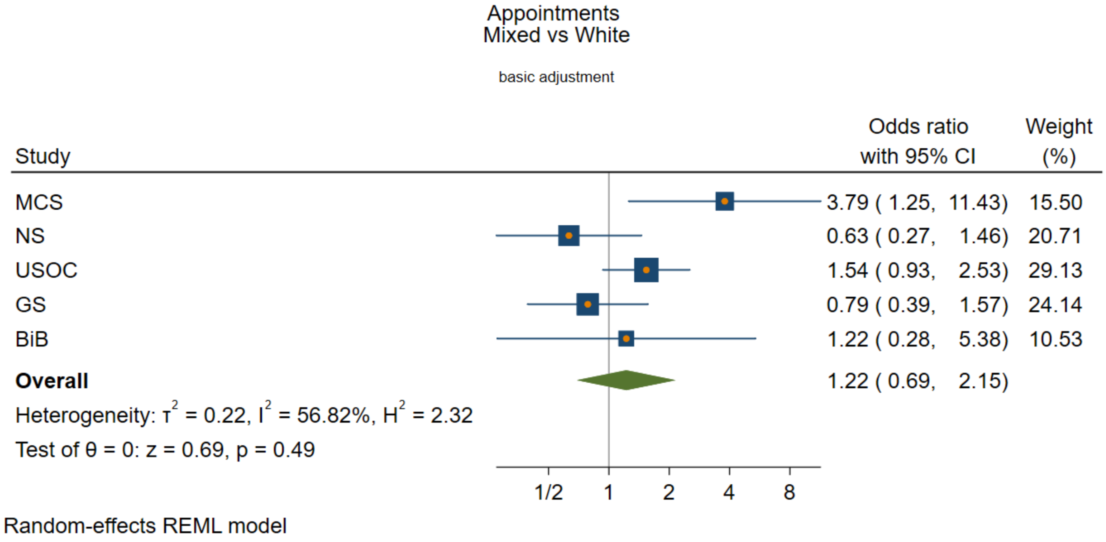
Appointments
Mixed vs White
basic adjustment
Study
Odds ratio with 95% CI
Weight (%)
MCS 3.79 (1.25, 11.43) 15.50
NS 0.63 (0.27, 1.46) 20.71
USOC 1.54 (0.93, 2.53) 29.13
GS 0.79 (0.39, 1.57) 24.14
BiB 1.22 (0.28, 5.38) 10.53
Overall 1.22 (0.69, 2.15)
Heterogeneity: τ² = 0.22, I² = 56.82%, H² = 2.32
Test of θ = 0: z = 0.69, p = 0.49
1/2
1
2
4
8
Random-effects REML model

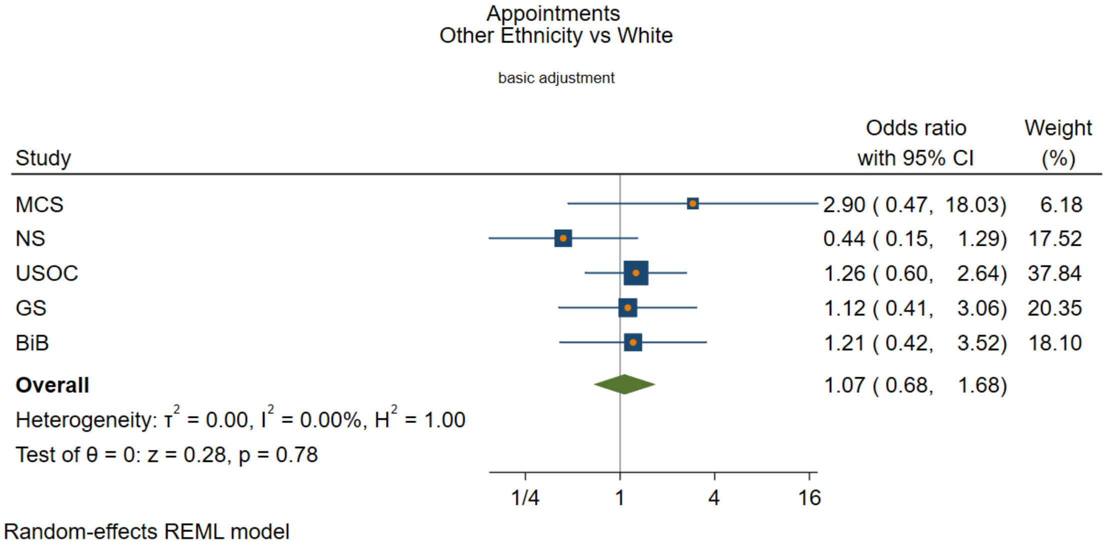
Appointments
Other Ethnicity vs White
basic adjustment
Study
Odds ratio with 95% CI
Weight (%)
MCS 2.90 (0.47, 18.03) 6.18
NS 0.44 (0.15, 1.29) 17.52
USOC 1.26 (0.60, 2.64) 37.84
GS 1.12 (0.41, 3.06) 20.35
BiB 1.21 (0.42, 3.52) 18.10
Overall 1.07 (0.68, 1.68)
Heterogeneity: τ² = 0.00, I² = 0.00%, H² = 1.00
Test of θ = 0: z = 0.28, p = 0.78
1/4
1
4
16
Random-effects REML model

Full adjustment

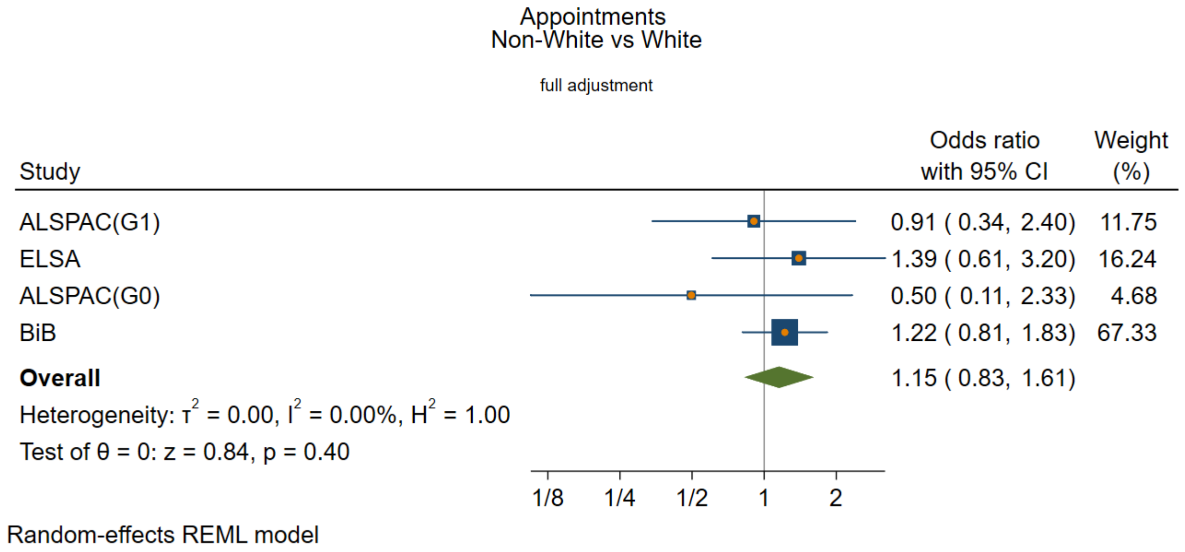

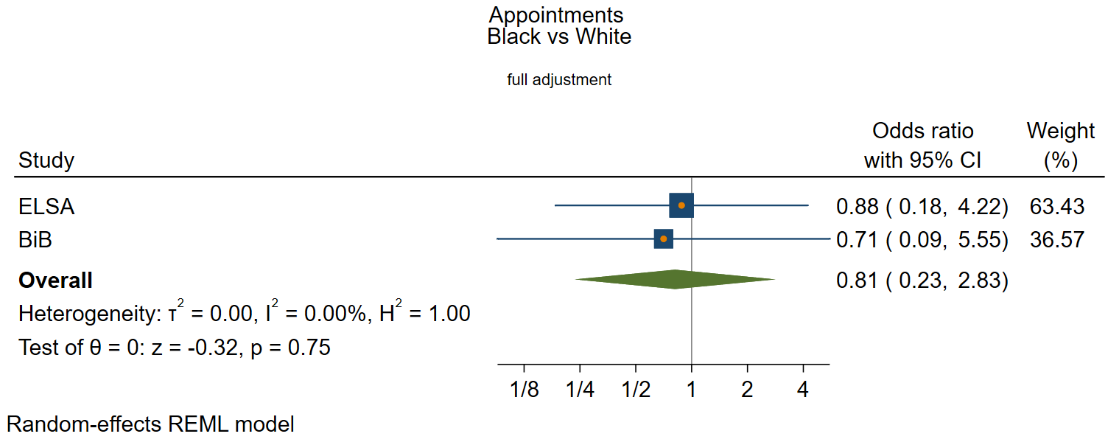

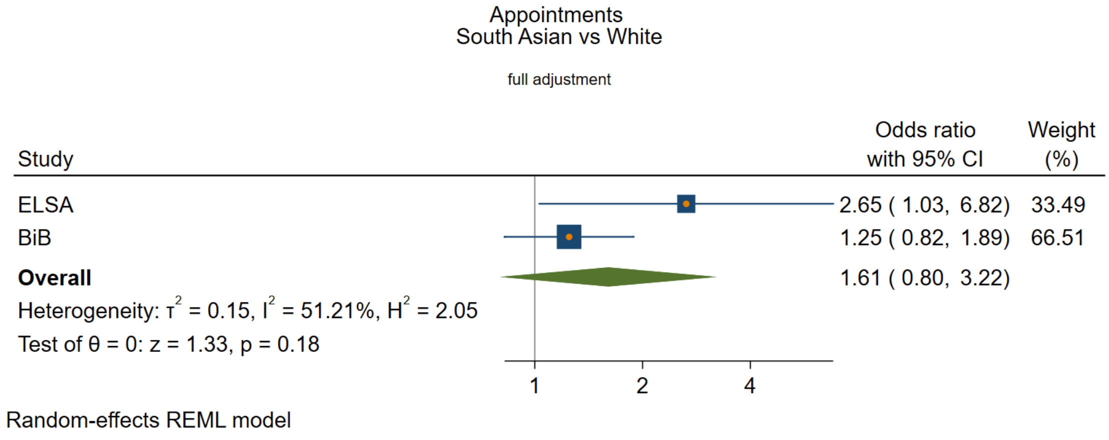

Maddock J, et al. BMJ Open 2022; 12:e064981. doi: 10.1136/bmjopen-2022-064981

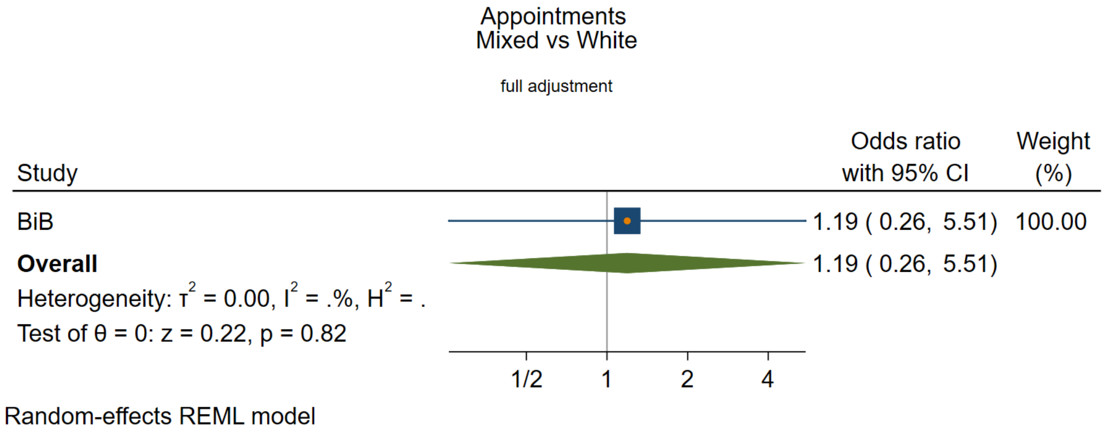

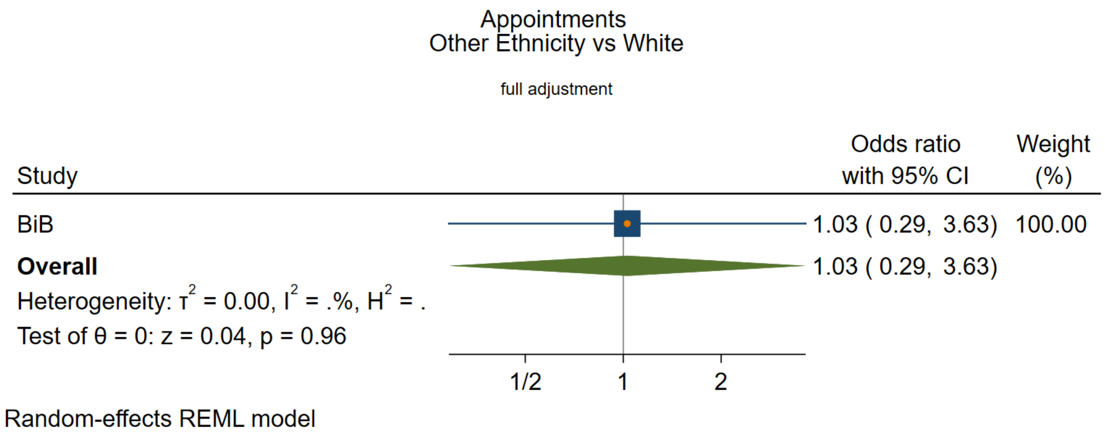

Maddock J, *et al. BMJ Open* 2022; 12:e064981. doi: 10.1136/bmjopen-2022-064981

Prescription/Medication access

Sex

Unadjusted

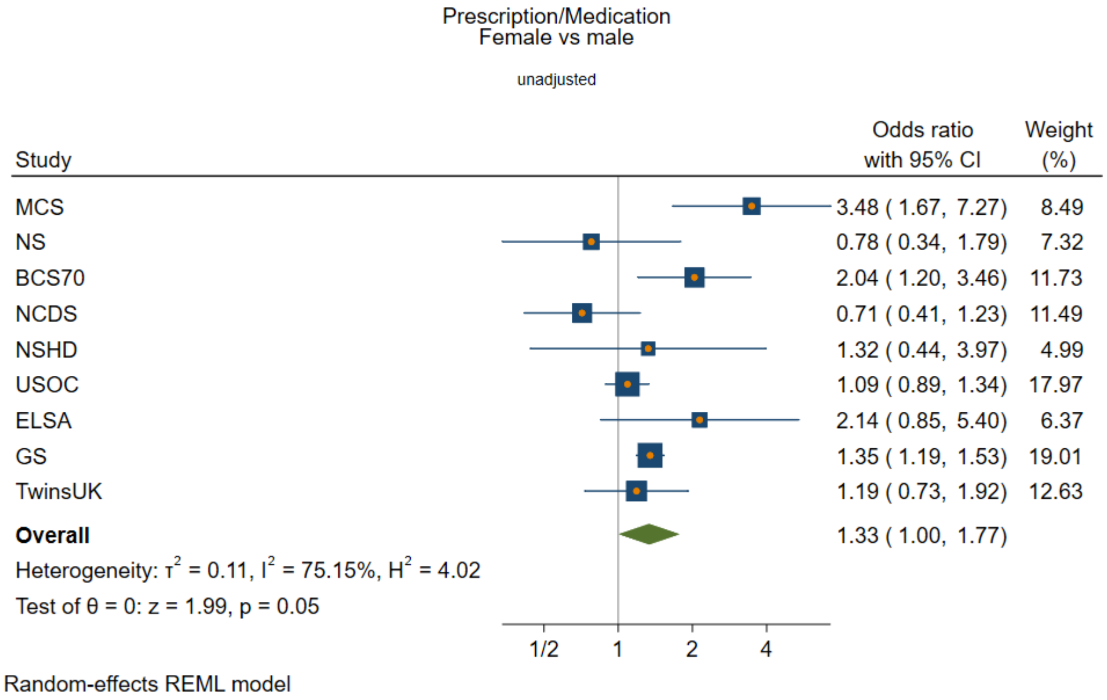

Basic adjustment

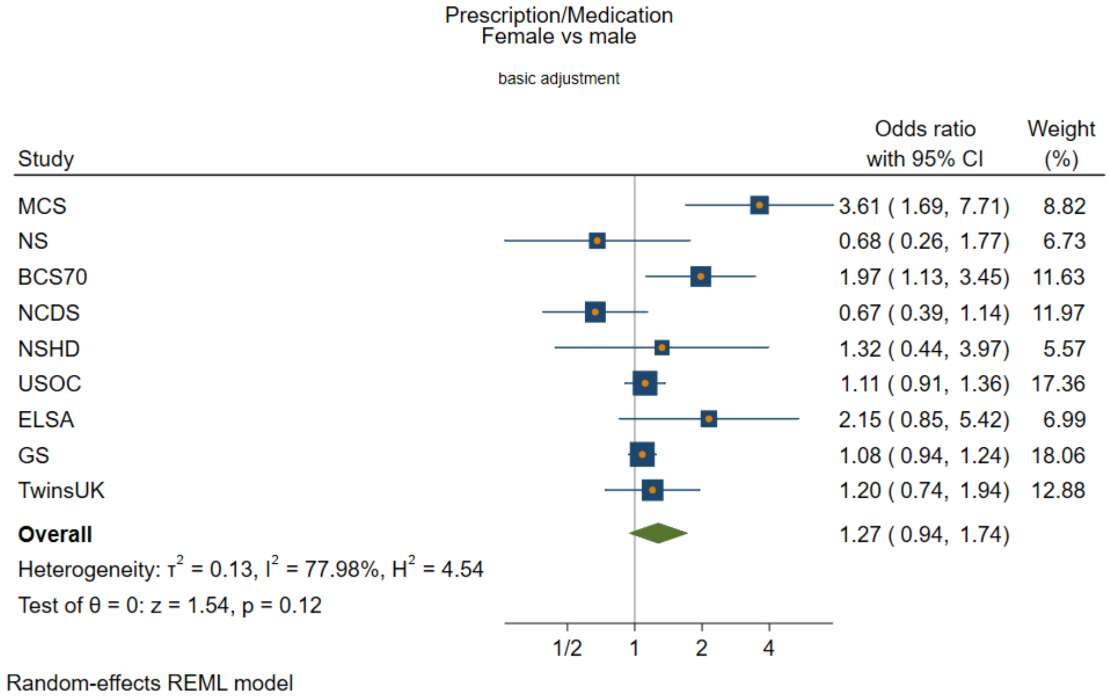

Maddock J, *et al. BMJ Open* 2022; 12:e064981. doi: 10.1136/bmjopen-2022-064981

Full adjustment

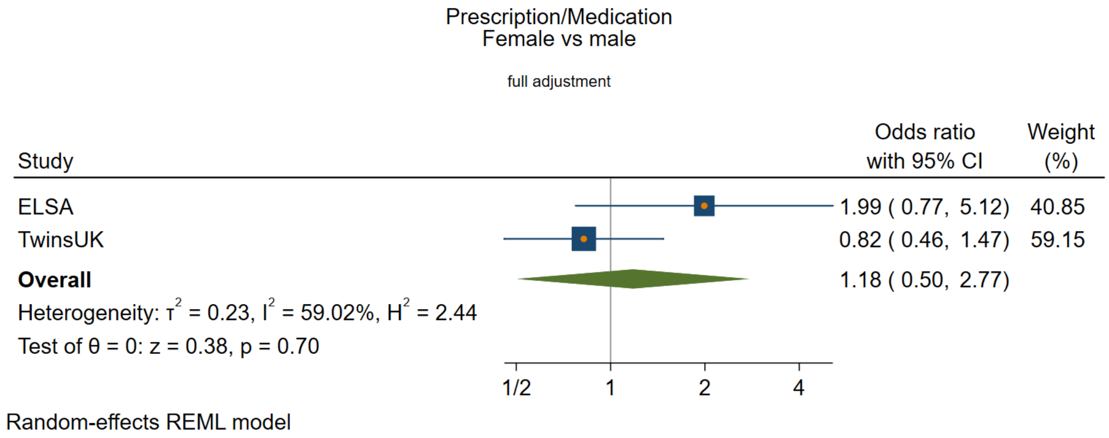

Occupational class

Unadjusted

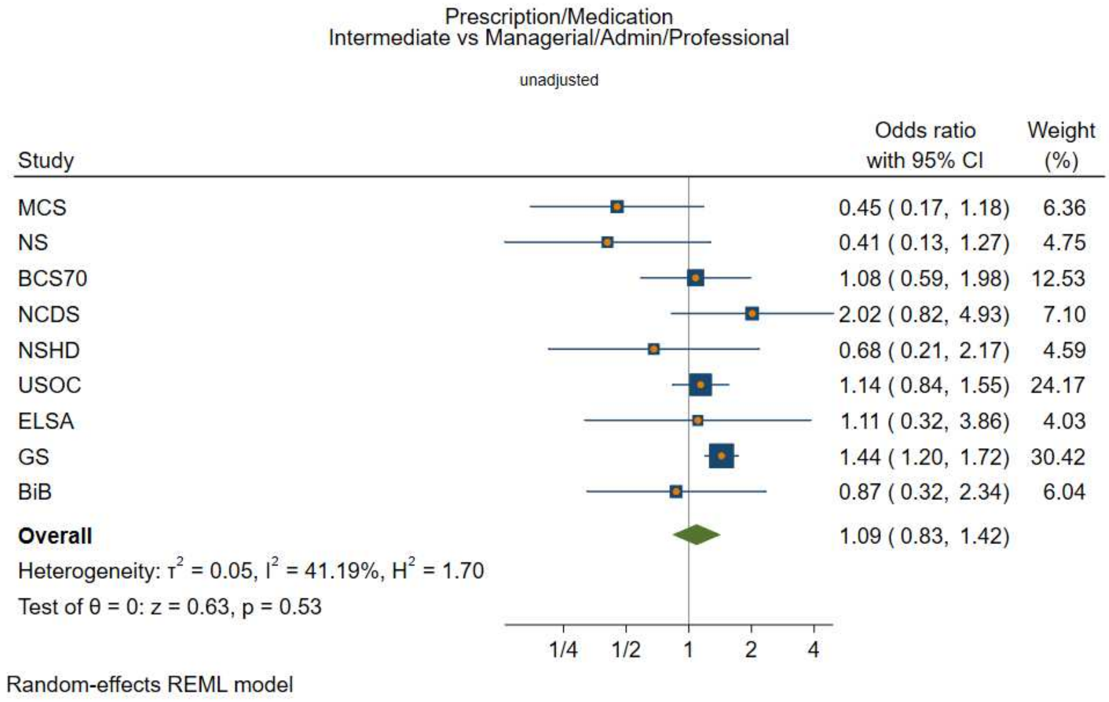

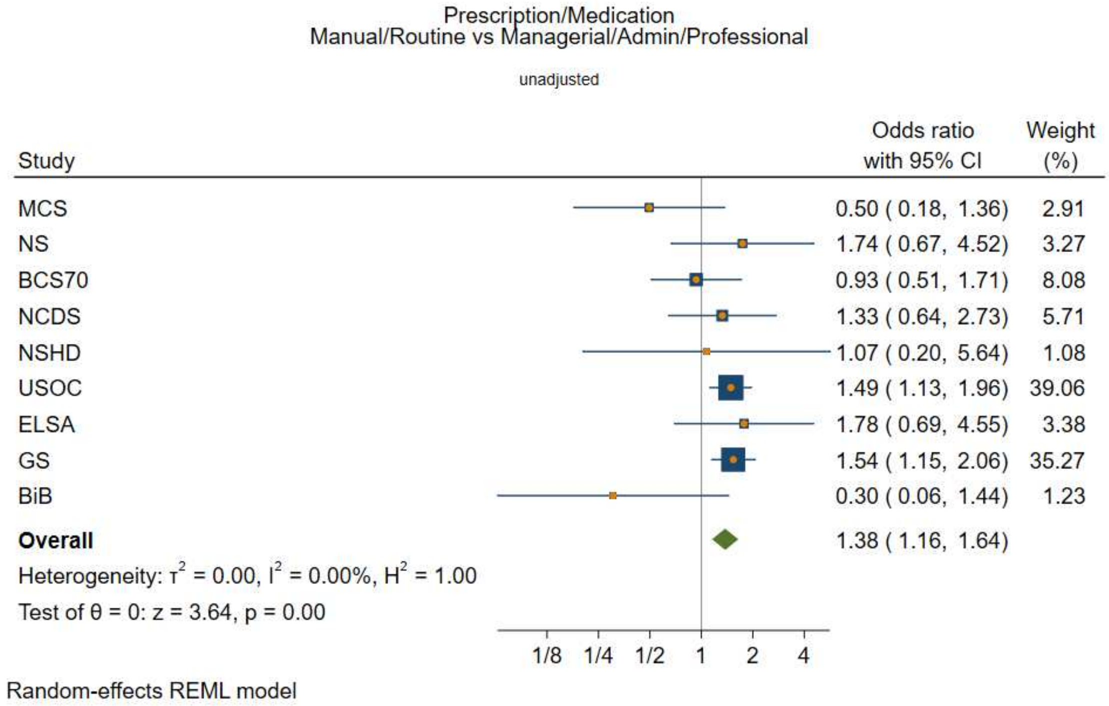

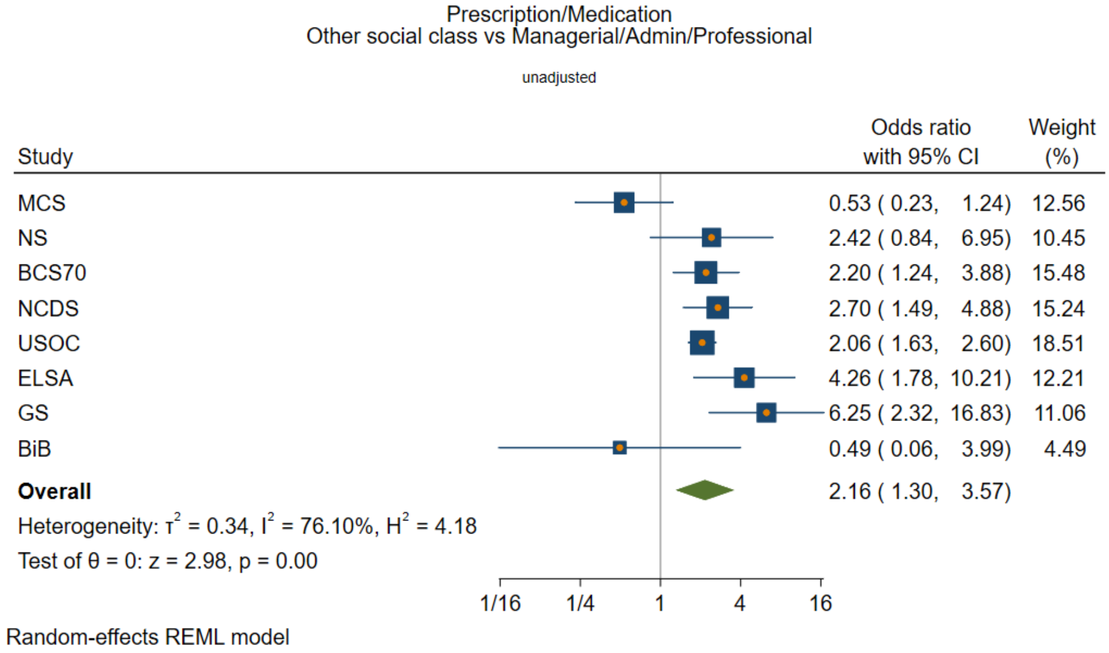

Maddock J, et al. BMJ Open 2022; 12:e064981. doi: 10.1136/bmjopen-2022-064981

Basic adjustment

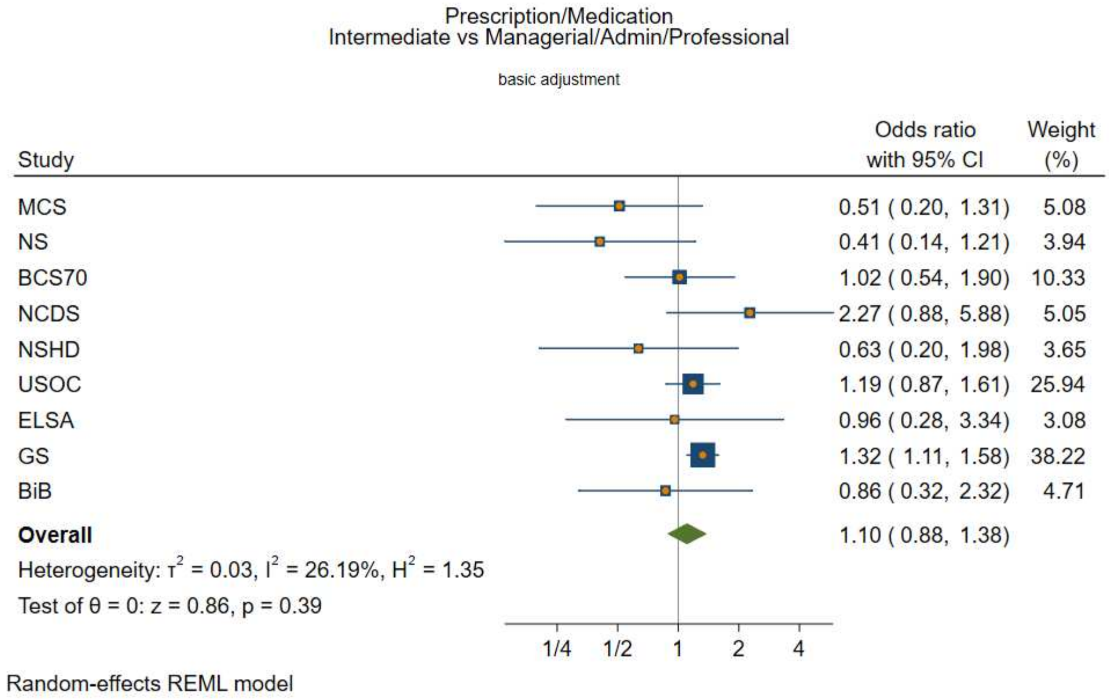

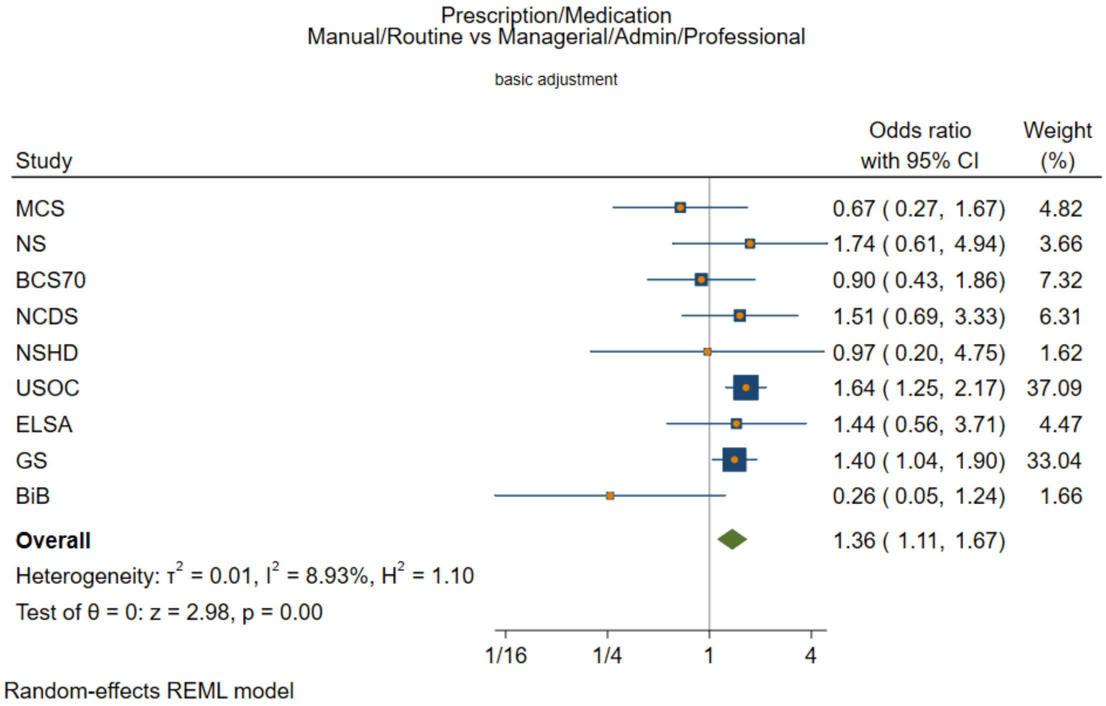
Prescription/Medication
Manual/Routine vs Managerial/Admin/Professional
basic adjustment
Study
Odds ratio with 95% CI
Weight (%)
MCS 0.67 (0.27, 1.67) 4.82
NS 1.74 (0.61, 4.94) 3.66
BCS70 0.90 (0.43, 1.86) 7.32
NCDS 1.51 (0.69, 3.33) 6.31
NSHD 0.97 (0.20, 4.75) 1.62
USOC 1.64 (1.25, 2.17) 37.09
ELSA 1.44 (0.56, 3.71) 4.47
GS 1.40 (1.04, 1.90) 33.04
BiB 0.26 (0.05, 1.24) 1.66
Overall 1.36 (1.11, 1.67)
Heterogeneity: τ² = 0.01, I² = 8.93%, H² = 1.10
Test of θ = 0: z = 2.98, p = 0.00
1/16
1/4
1
4
Random-effects REML model

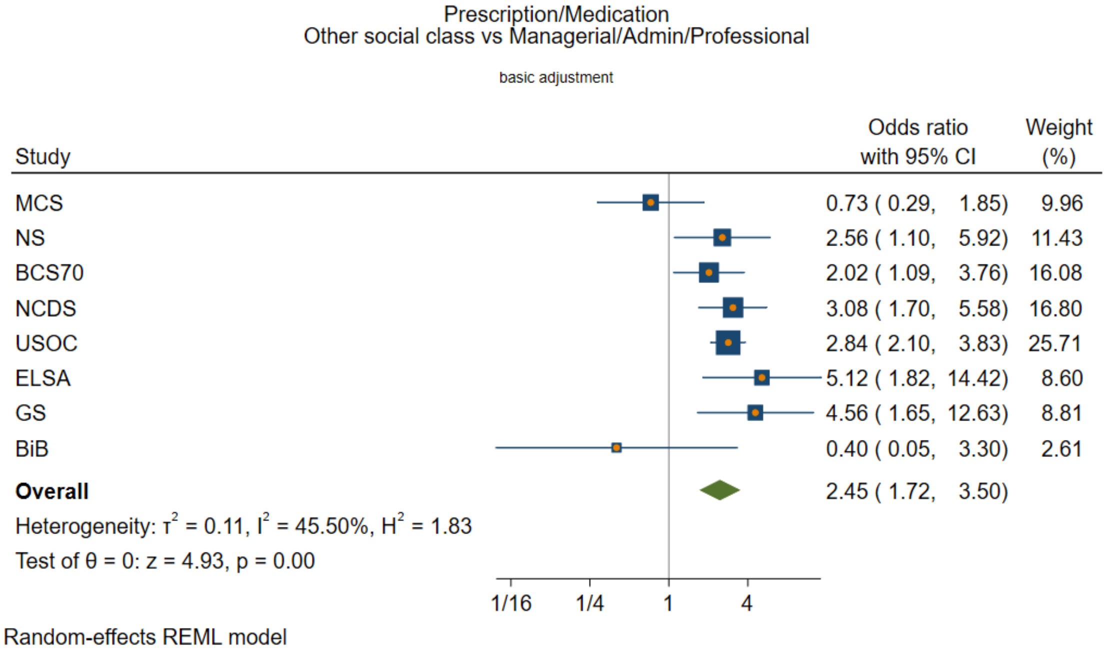
Prescription/Medication
Other social class vs Managerial/Admin/Professional
basic adjustment
Study
Odds ratio with 95% CI
Weight (%)
MCS 0.73 (0.29, 1.85) 9.96
NS 2.56 (1.10, 5.92) 11.43
BCS70 2.02 (1.09, 3.76) 16.08
NCDS 3.08 (1.70, 5.58) 16.80
USOC 2.84 (2.10, 3.83) 25.71
ELSA 5.12 (1.82, 14.42) 8.60
GS 4.56 (1.65, 12.63) 8.81
BiB 0.40 (0.05, 3.30) 2.61
Overall 2.45 (1.72, 3.50)
Heterogeneity: τ² = 0.11, I² = 45.50%, H² = 1.83
Test of θ = 0: z = 4.93, p = 0.00
1/16
1/4
1
4
Random-effects REML model

Full adjustment

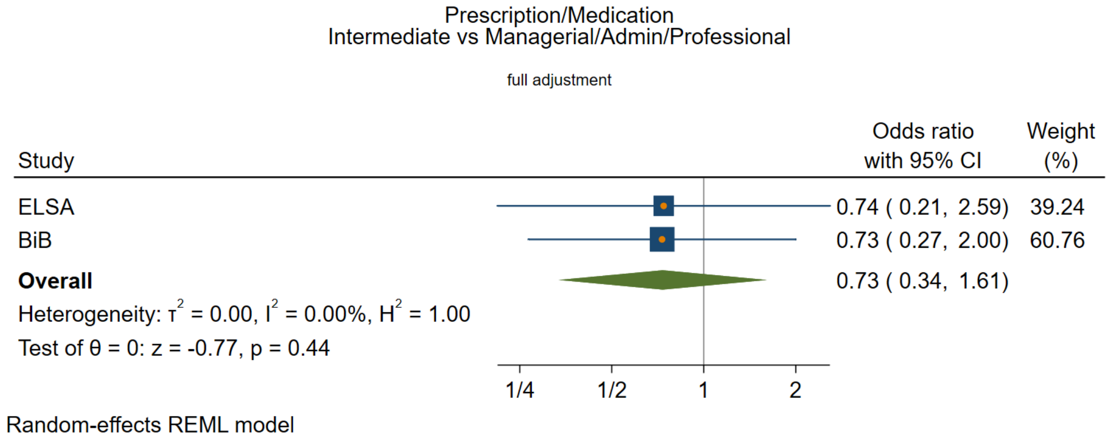

78

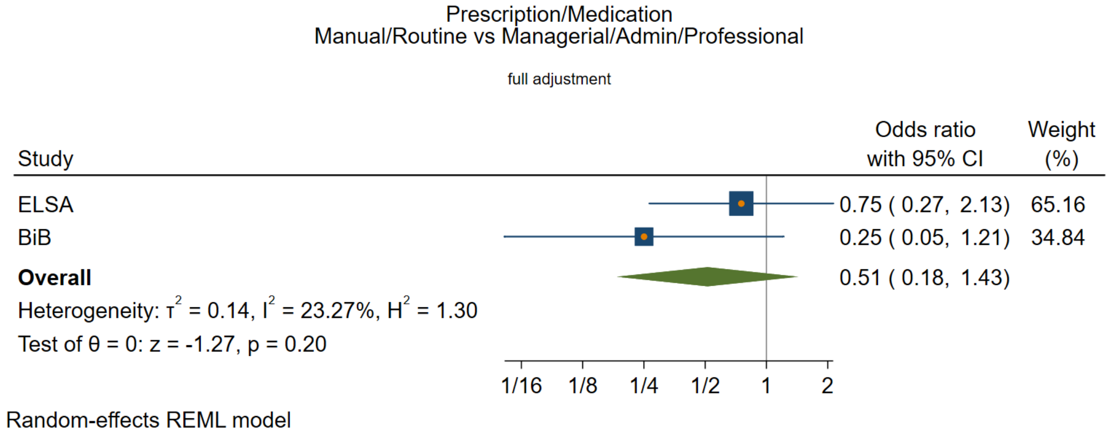
Prescription/Medication
Manual/Routine vs Managerial/Admin/Professional
full adjustment
Study
Odds ratio with 95% CI
Weight (%)
ELSA
0.75 (0.27, 2.13)
65.16
BiB
0.25 (0.05, 1.21)
34.84
Overall
0.51 (0.18, 1.43)
Heterogeneity: τ2 = 0.14, I2 = 23.27%, H2 = 1.30
Test of θ = 0: z = -1.27, p = 0.20
1/16
1/8
1/4
1/2
1
2
Random-effects REML model

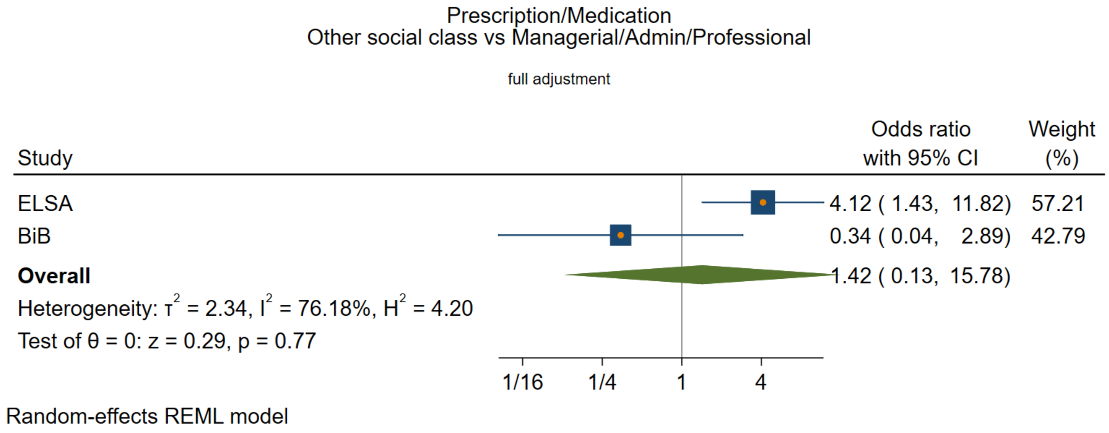
Prescription/Medication
Other social class vs Managerial/Admin/Professional
full adjustment
Study
Odds ratio with 95% CI
Weight (%)
ELSA
4.12 (1.43, 11.82)
57.21
BiB
0.34 (0.04, 2.89)
42.79
Overall
1.42 (0.13, 15.78)
Heterogeneity: τ2 = 2.34, I2 = 76.18%, H2 = 4.20
Test of θ = 0: z = 0.29, p = 0.77
1/16
1/4
1
4
Random-effects REML model

Education
<u>Unadjusted</u>

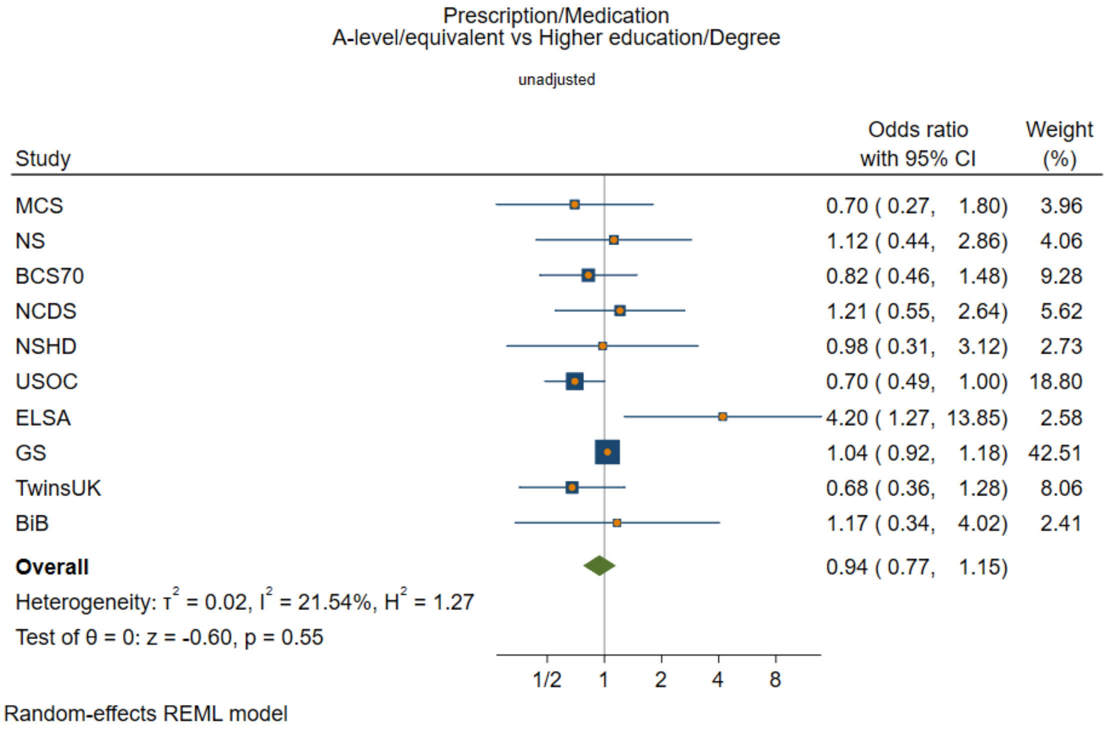

Prescription/Medication
GCSE/equivalent vs Higher education/Degree

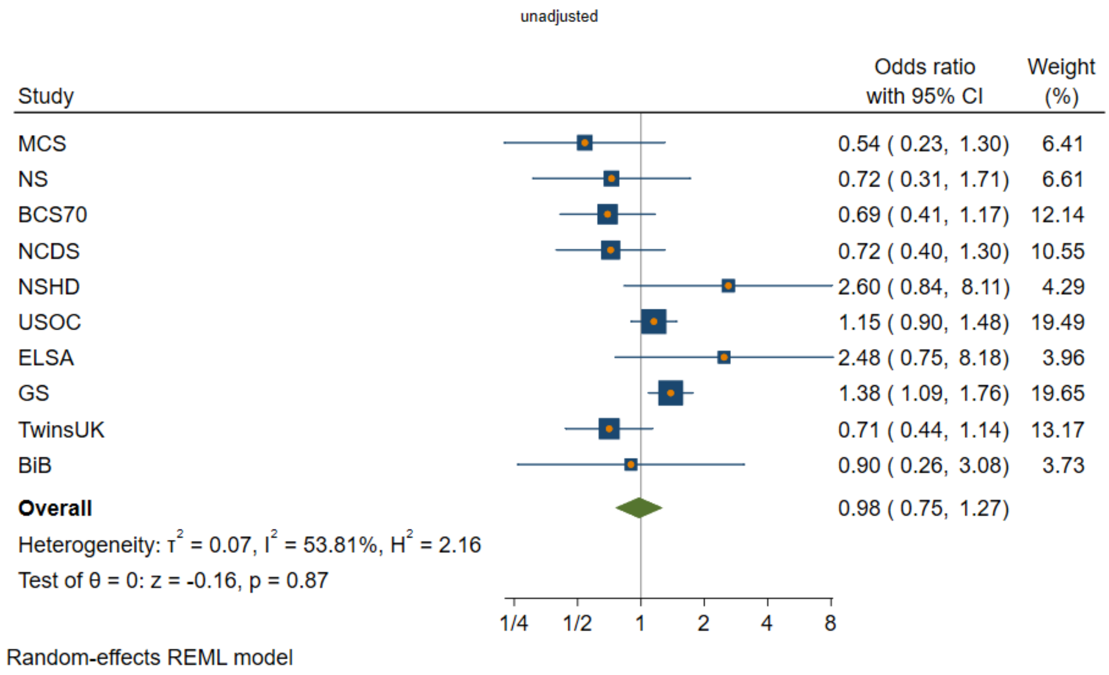

Prescription/Medication
<GCSE/equivalent vs Higher education/Degree

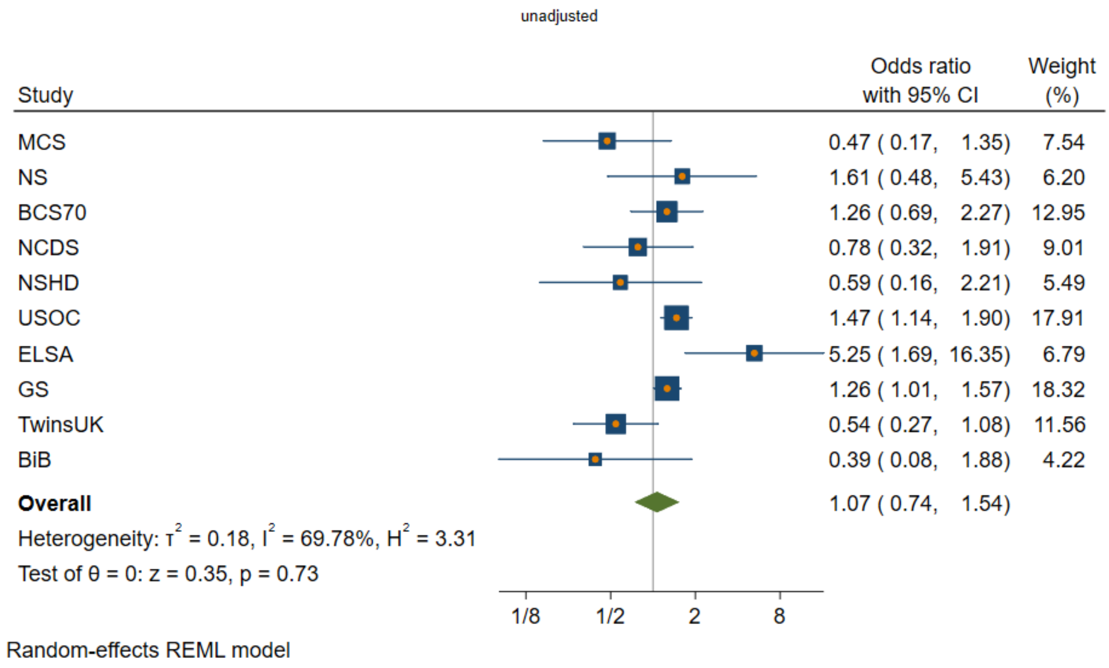

Basic adjustment

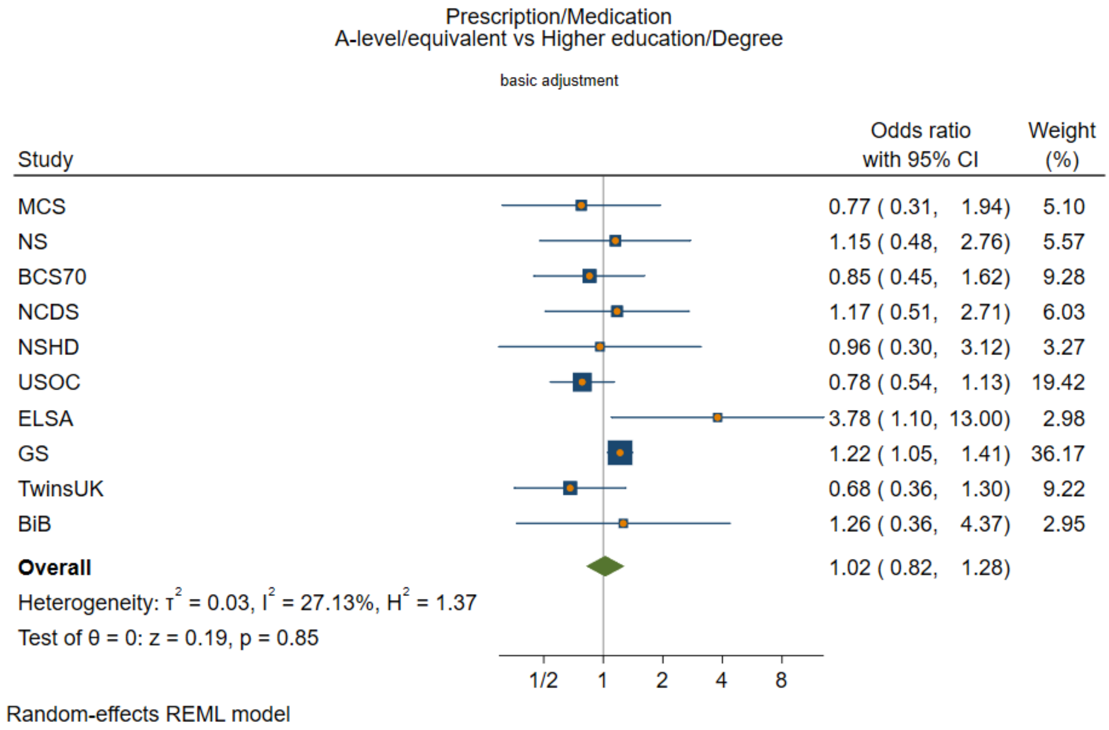

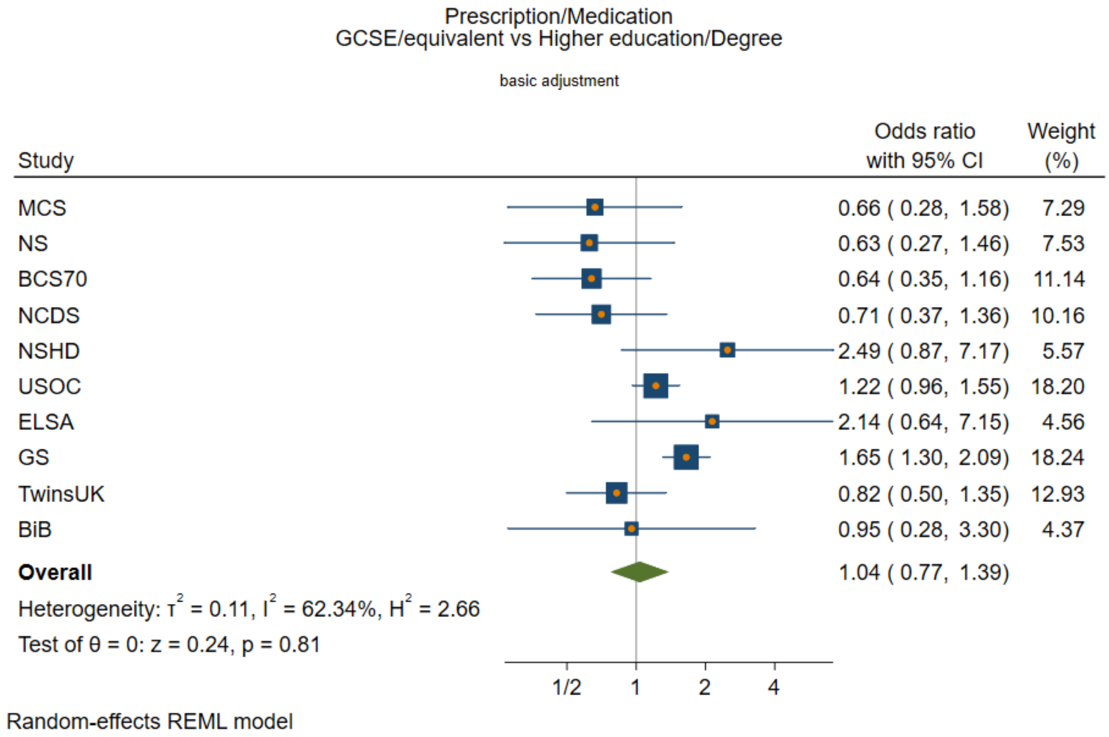

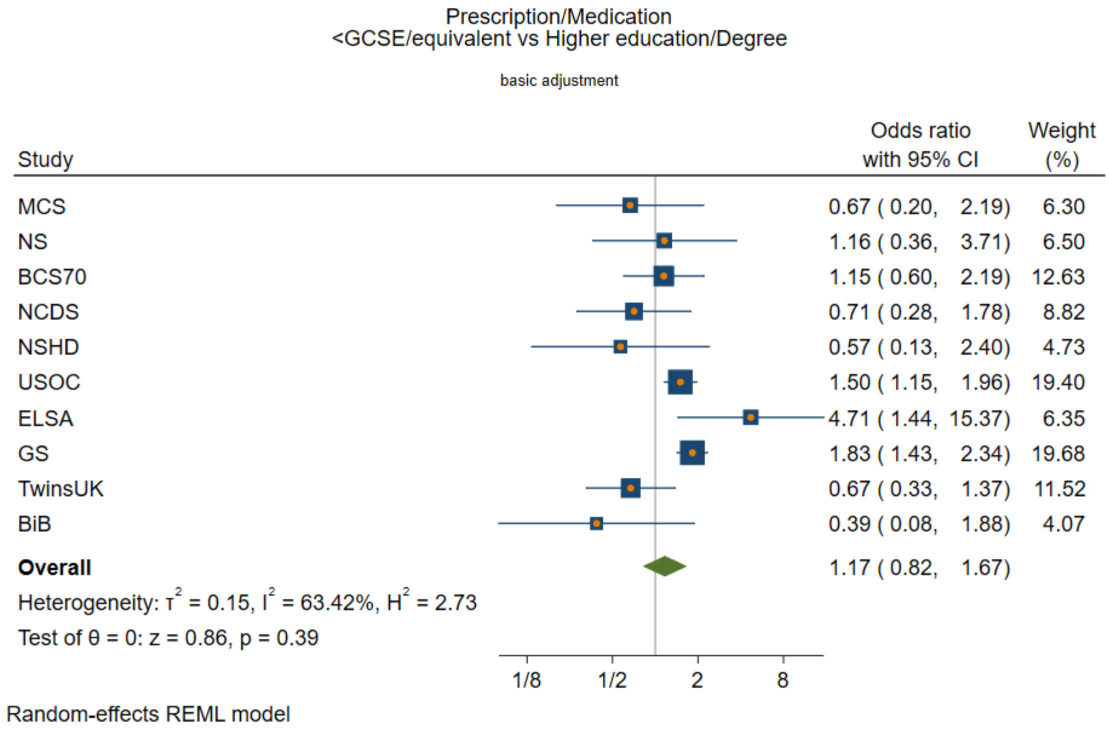

Maddock J, et al. BMJ Open 2022; 12:e064981. doi: 10.1136/bmjopen-2022-064981

Full adjustment

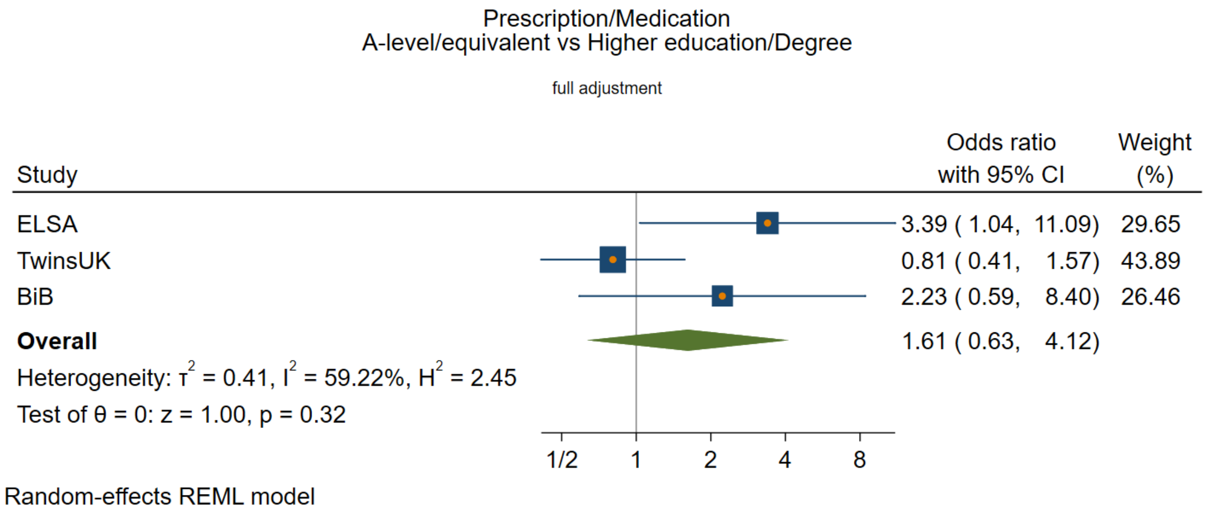

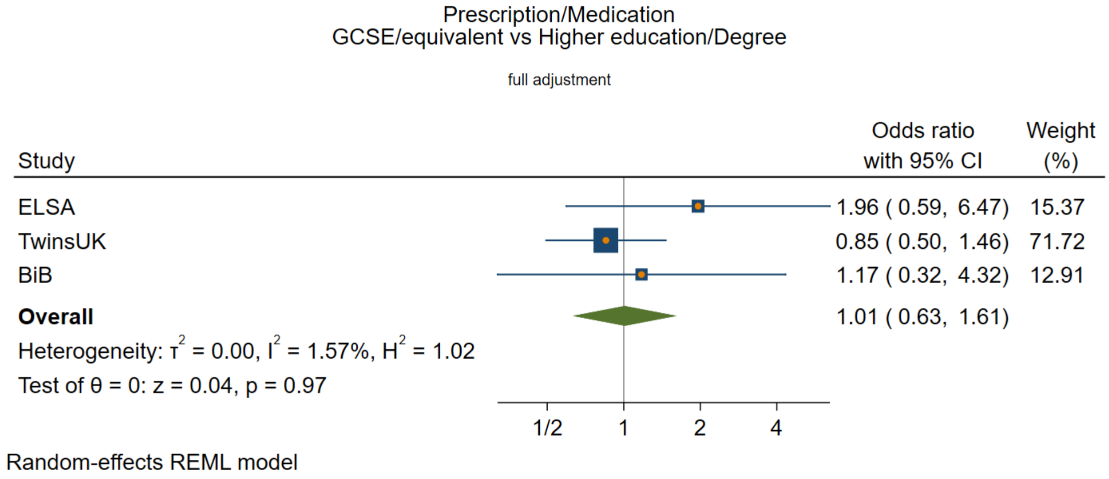
Prescription/Medication
GCSE/equivalent vs Higher education/Degree
full adjustment
Study
Odds ratio with 95% CI
Weight (%)
ELSA
1.96 (0.59, 6.47)
15.37
TwinsUK
0.85 (0.50, 1.46)
71.72
BiB
1.17 (0.32, 4.32)
12.91
Overall
1.01 (0.63, 1.61)
Heterogeneity: τ² = 0.00, I² = 1.57%, H² = 1.02
Test of θ = 0: z = 0.04, p = 0.97
1/2
1
2
4
Random-effects REML model

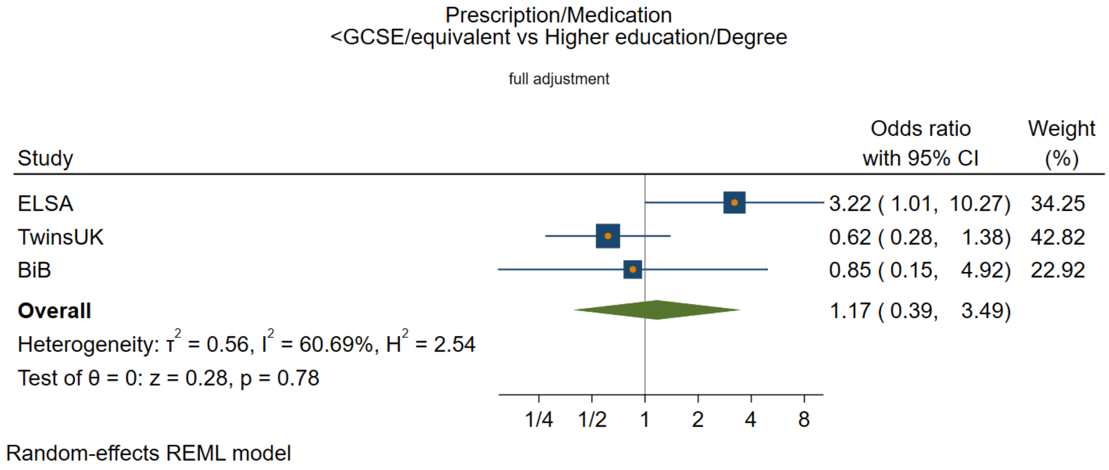
Prescription/Medication
<GCSE/equivalent vs Higher education/Degree
full adjustment
Study
Odds ratio with 95% CI
Weight (%)
ELSA
3.22 (1.01, 10.27)
34.25
TwinsUK
0.62 (0.28, 1.38)
42.82
BiB
0.85 (0.15, 4.92)
22.92
Overall
1.17 (0.39, 3.49)
Heterogeneity: τ² = 0.56, I² = 60.69%, H² = 2.54
Test of θ = 0: z = 0.28, p = 0.78
1/4
1/2
1
2
4
8
Random-effects REML model

Age

Unadjusted

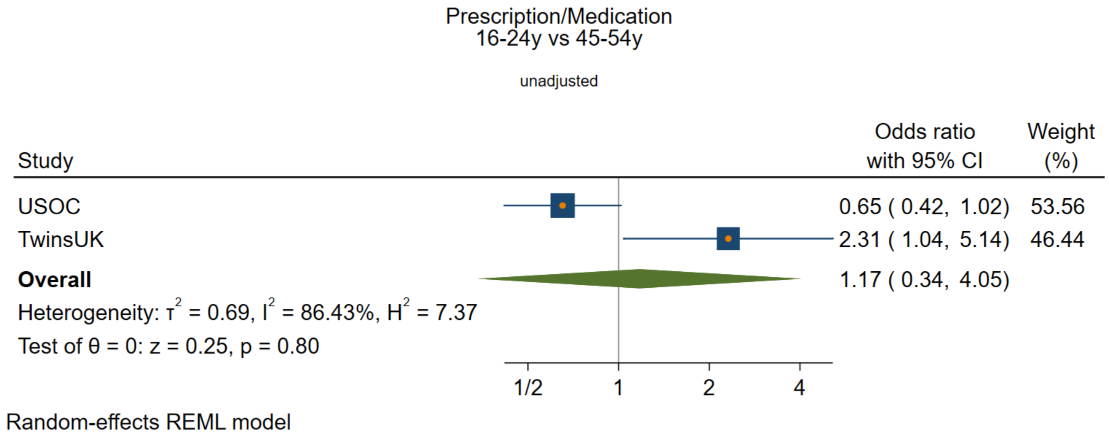

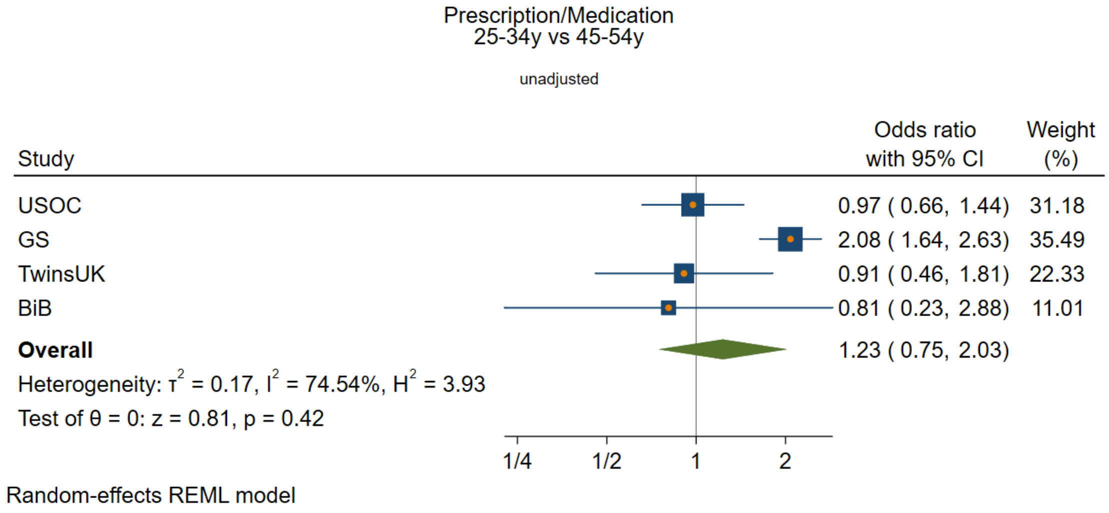

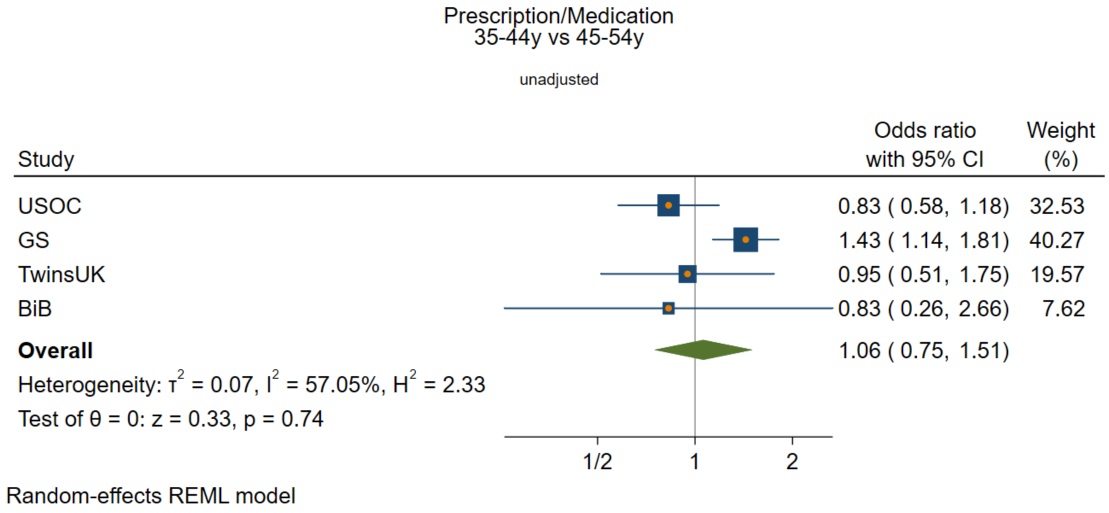

Maddock J, *et al. BMJ Open* 2022; 12:e064981. doi: 10.1136/bmjopen-2022-064981

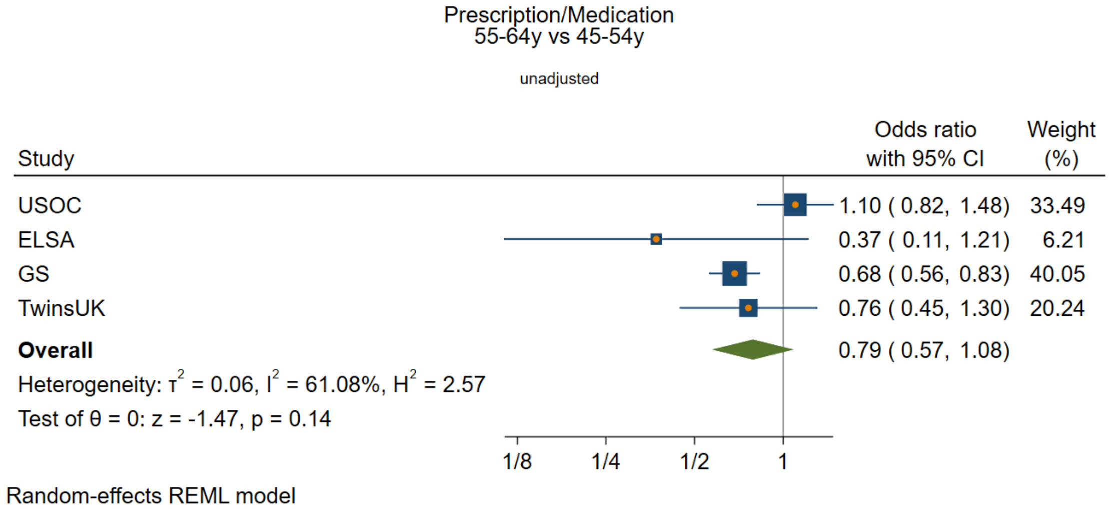

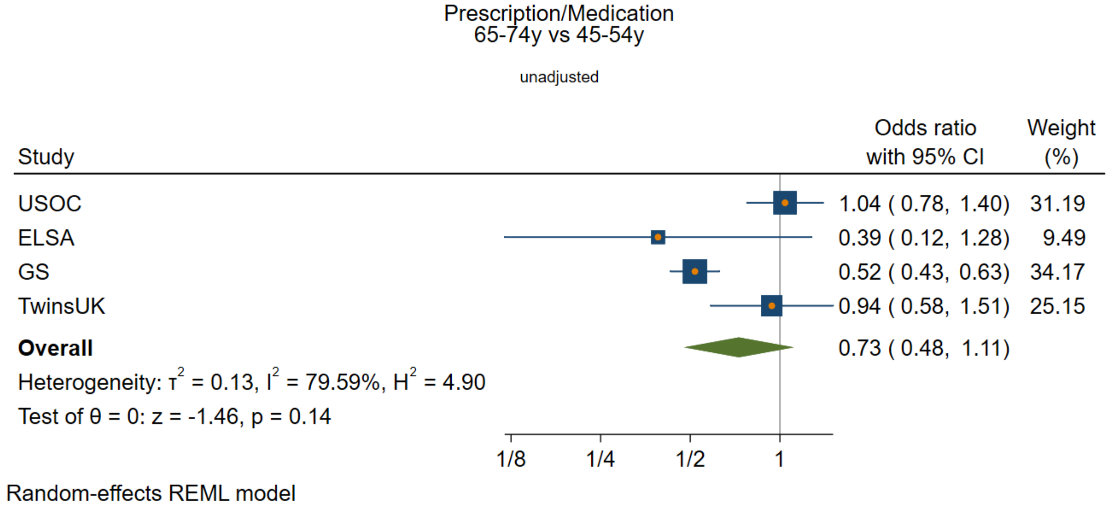

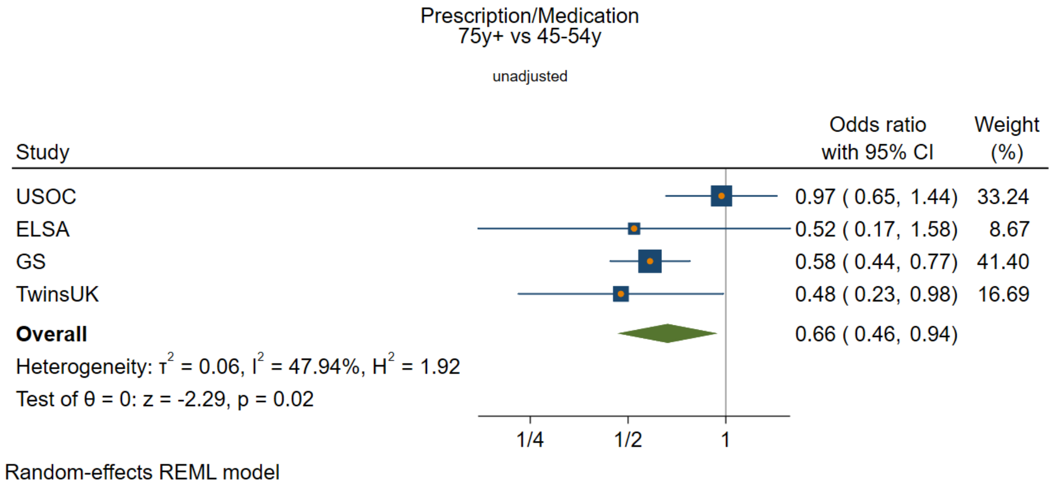

Maddock J, *et al. BMJ Open* 2022; 12:e064981. doi: 10.1136/bmjopen-2022-064981

Basic adjustment

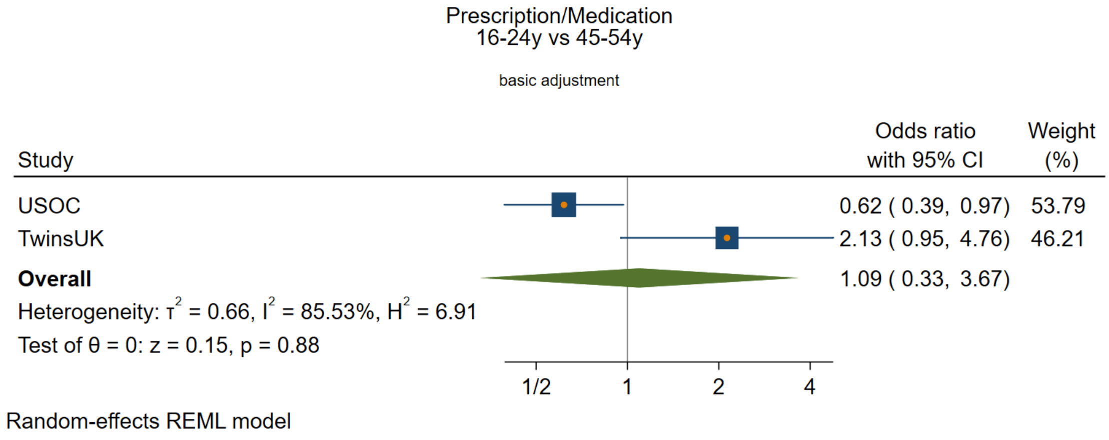

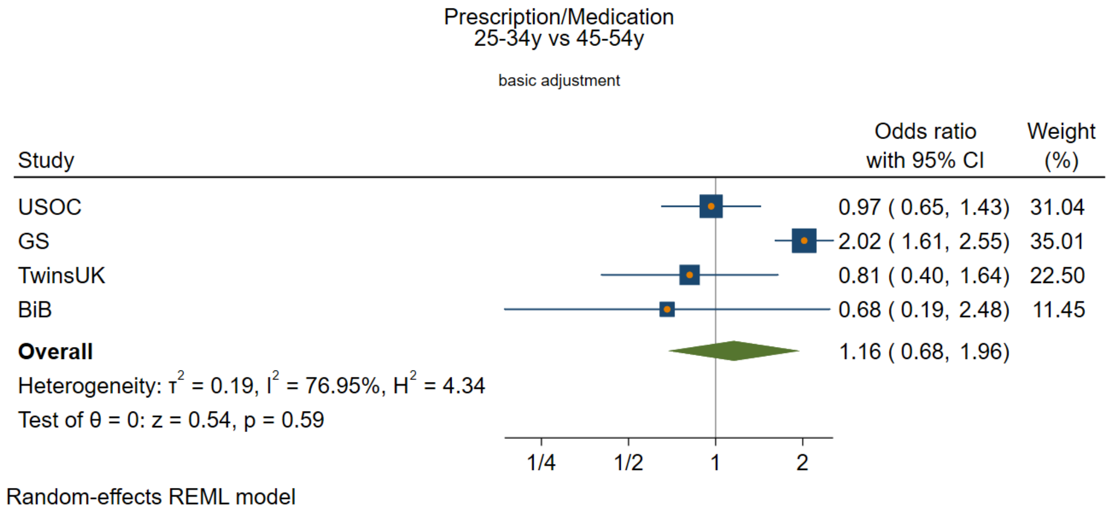

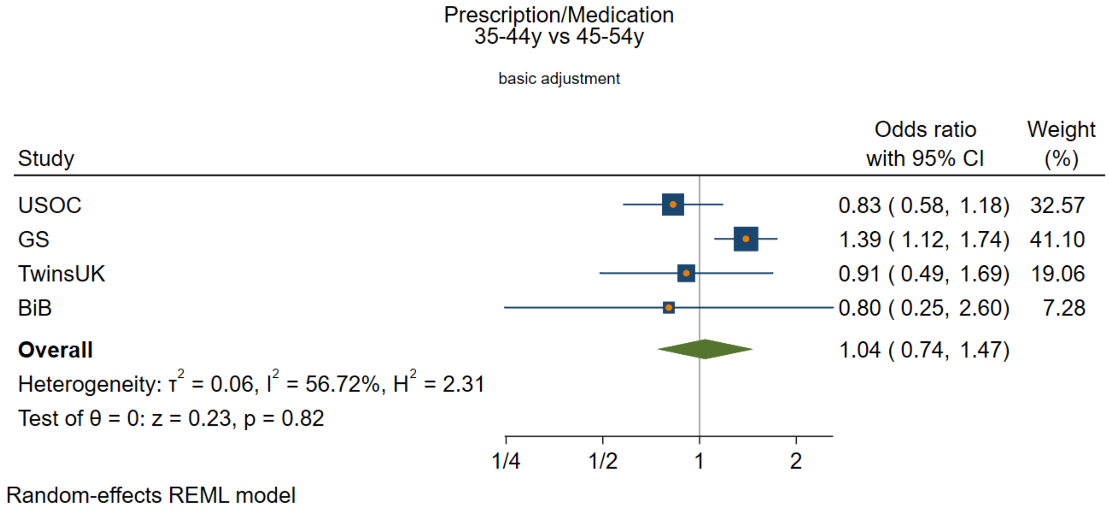

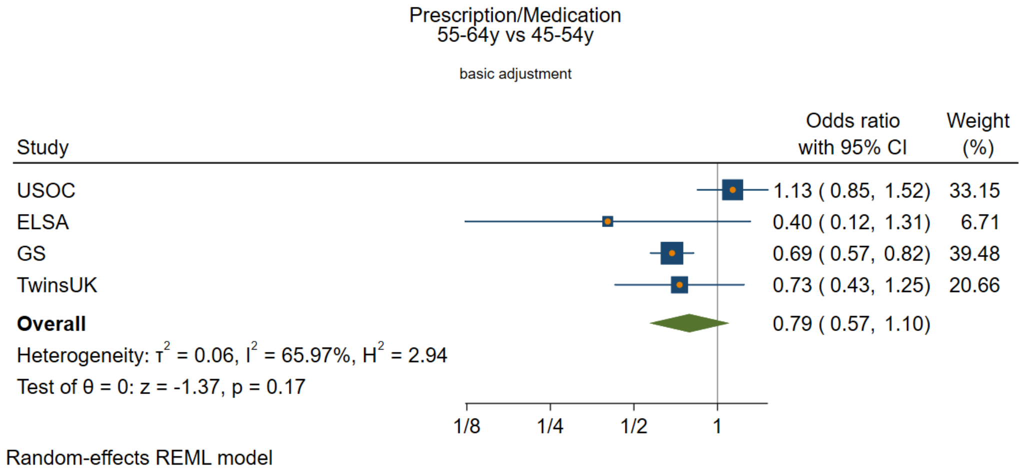

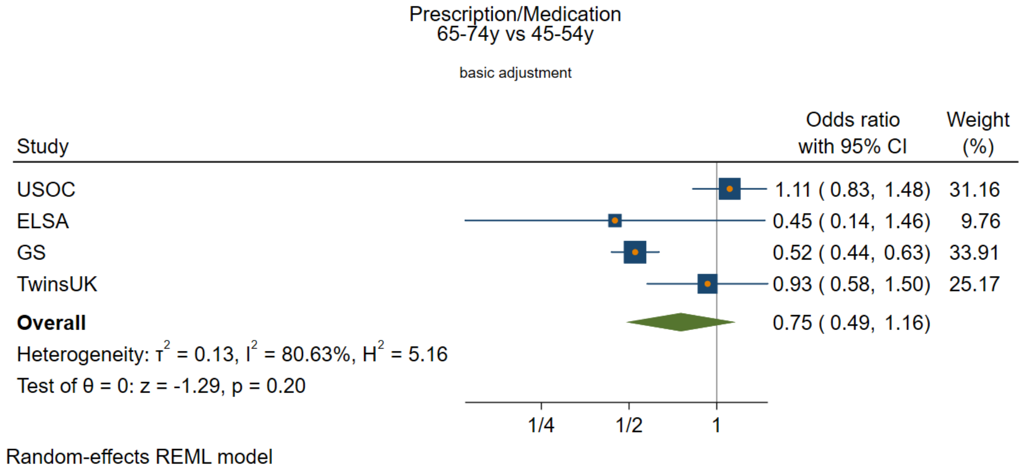

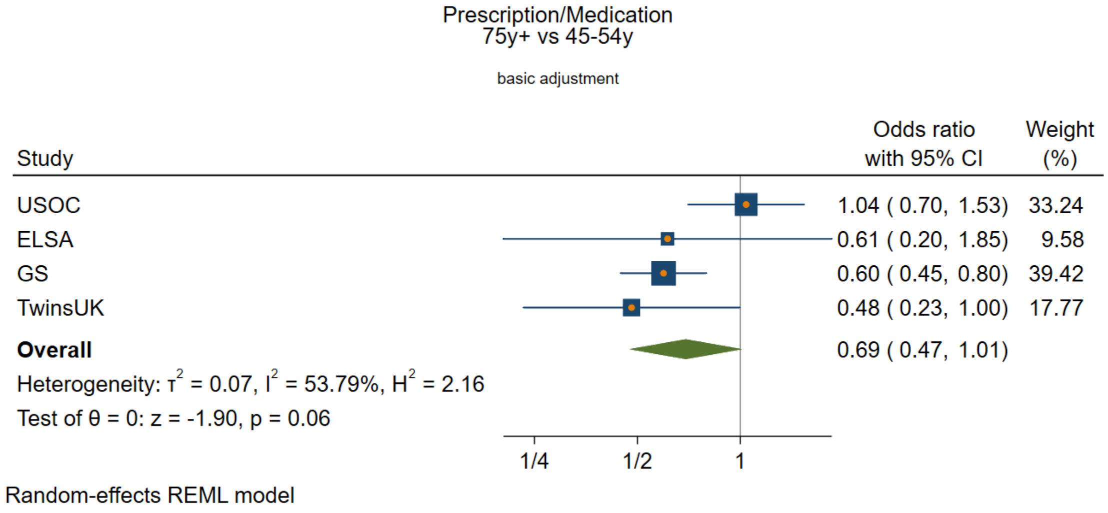

Maddock J, *et al. BMJ Open* 2022; 12:e064981. doi: 10.1136/bmjopen-2022-064981

Full adjustment

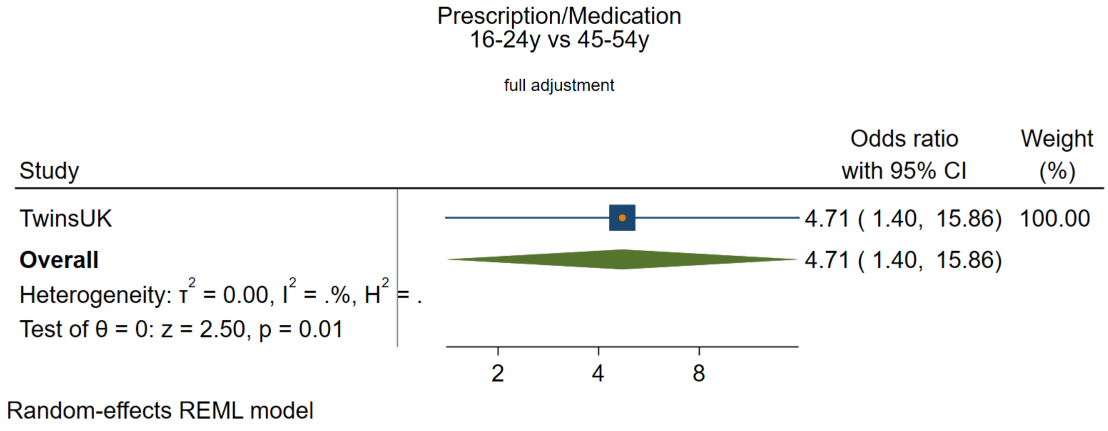

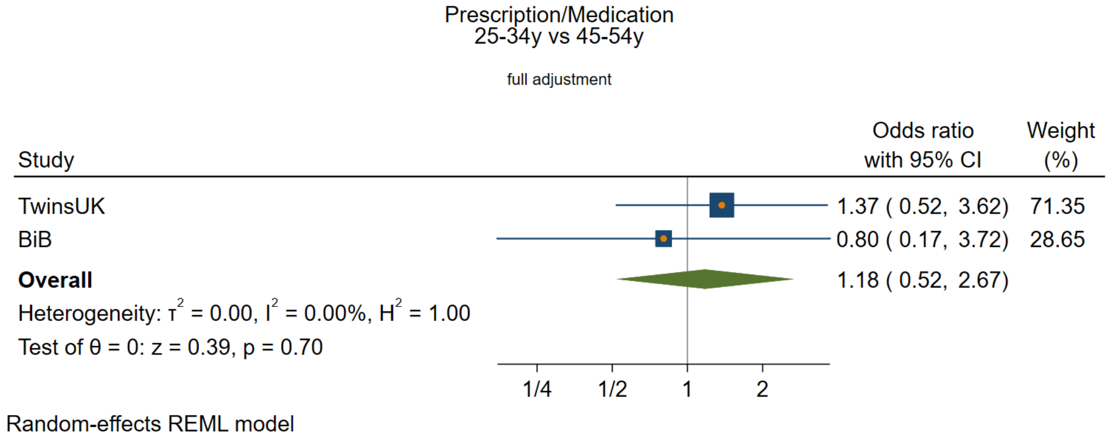

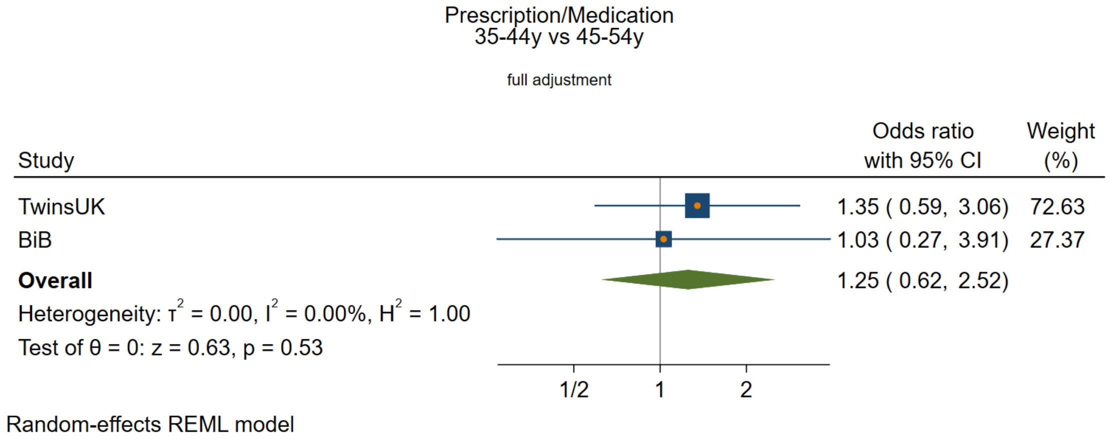

Maddock J, *et al. BMJ Open* 2022; 12:e064981. doi: 10.1136/bmjopen-2022-064981

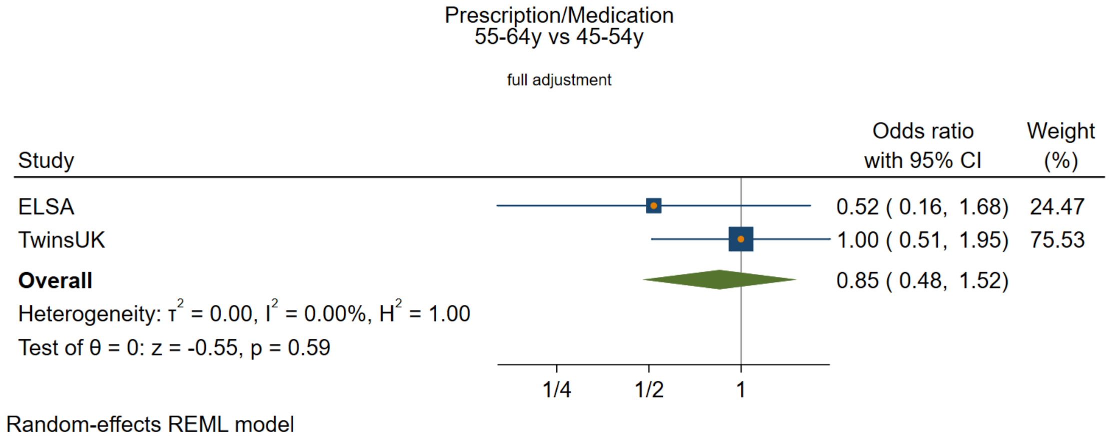
Prescription/Medication
55-64y vs 45-54y
full adjustment
Study
Odds ratio with 95% CI
Weight (%)
ELSA
0.52 (0.16, 1.68)
24.47
TwinsUK
1.00 (0.51, 1.95)
75.53
Overall
0.85 (0.48, 1.52)
Heterogeneity: τ² = 0.00, I² = 0.00%, H² = 1.00
Test of θ = 0: z = -0.55, p = 0.59
1/4
1/2
1
Random-effects REML model

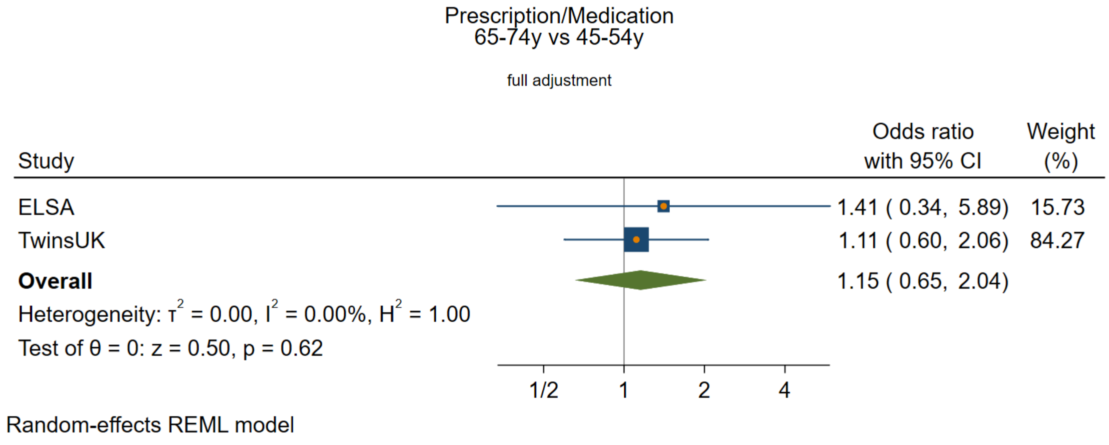
Prescription/Medication
65-74y vs 45-54y
full adjustment
Study
Odds ratio with 95% CI
Weight (%)
ELSA
1.41 (0.34, 5.89)
15.73
TwinsUK
1.11 (0.60, 2.06)
84.27
Overall
1.15 (0.65, 2.04)
Heterogeneity: τ² = 0.00, I² = 0.00%, H² = 1.00
Test of θ = 0: z = 0.50, p = 0.62
1/2
1
2
4
Random-effects REML model

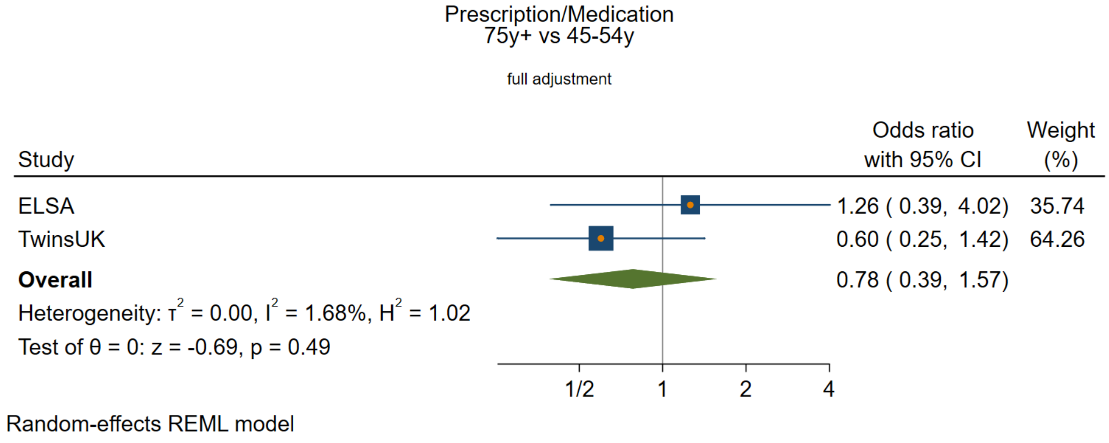
Prescription/Medication
75y+ vs 45-54y
full adjustment
Study
Odds ratio with 95% CI
Weight (%)
ELSA
1.26 (0.39, 4.02)
35.74
TwinsUK
0.60 (0.25, 1.42)
64.26
Overall
0.78 (0.39, 1.57)
Heterogeneity: τ² = 0.00, I² = 1.68%, H² = 1.02
Test of θ = 0: z = -0.69, p = 0.49
1/2
1
2
4
Random-effects REML model

Ethnicity

Unadjusted

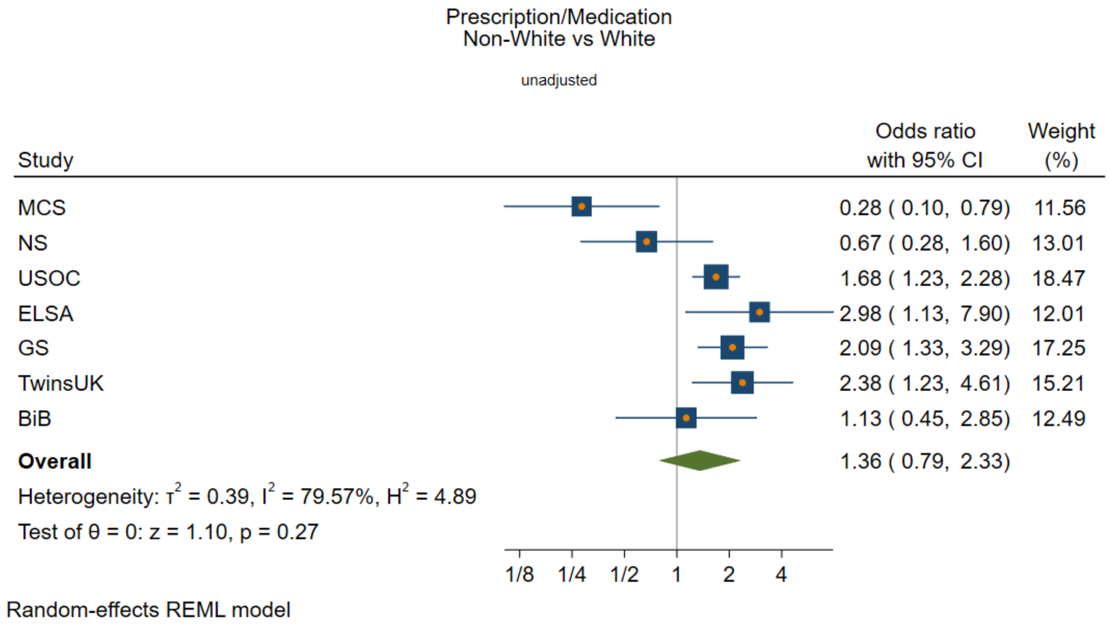

Maddock J, *et al. BMJ Open* 2022; 12:e064981. doi: 10.1136/bmjopen-2022-064981

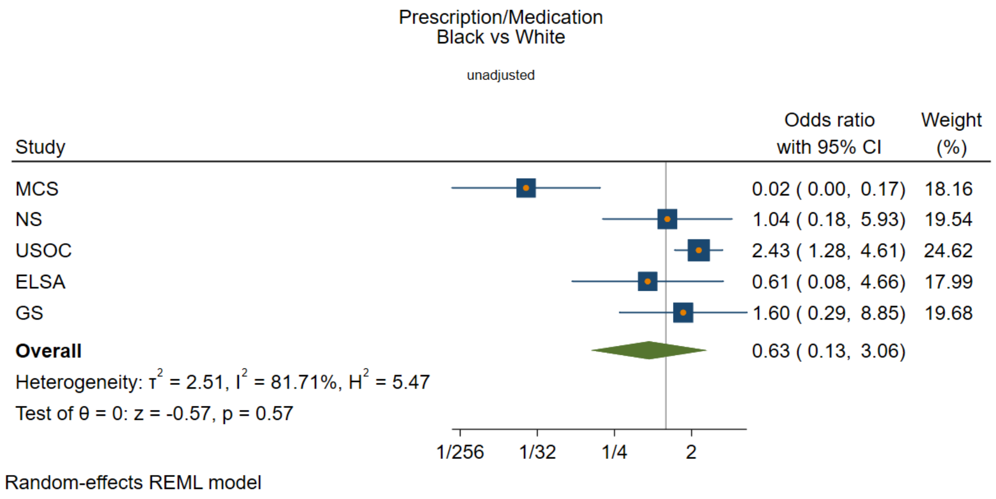
Prescription/Medication
Black vs White
unadjusted
Study
Odds ratio with 95% CI
Weight (%)
MCS 0.02 (0.00, 0.17) 18.16
NS 1.04 (0.18, 5.93) 19.54
USOC 2.43 (1.28, 4.61) 24.62
ELSA 0.61 (0.08, 4.66) 17.99
GS 1.60 (0.29, 8.85) 19.68
Overall 0.63 (0.13, 3.06)
Heterogeneity: τ² = 2.51, I² = 81.71%, H² = 5.47
Test of θ = 0: z = -0.57, p = 0.57
1/256
1/32
1/4
2
Random-effects REML model

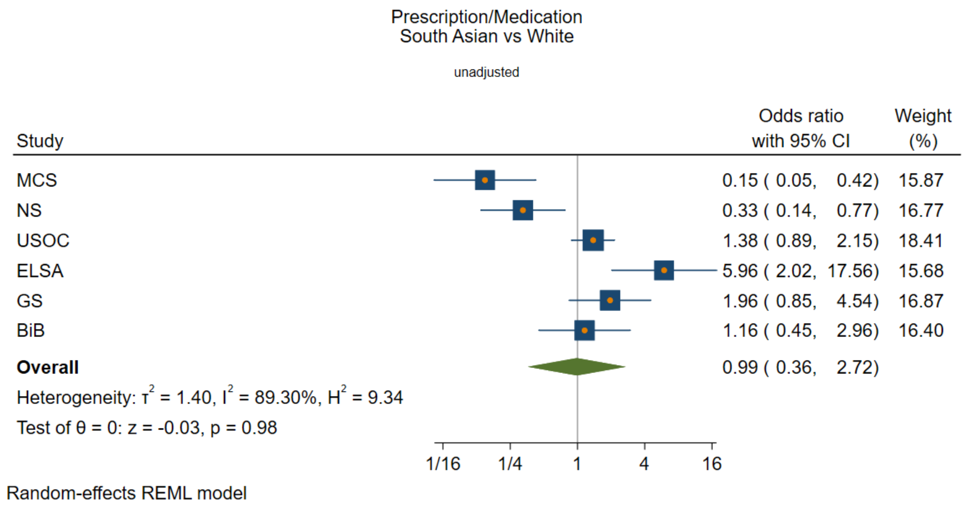
Prescription/Medication
South Asian vs White
unadjusted
Study
Odds ratio with 95% CI
Weight (%)
MCS 0.15 (0.05, 0.42) 15.87
NS 0.33 (0.14, 0.77) 16.77
USOC 1.38 (0.89, 2.15) 18.41
ELSA 5.96 (2.02, 17.56) 15.68
GS 1.96 (0.85, 4.54) 16.87
BiB 1.16 (0.45, 2.96) 16.40
Overall 0.99 (0.36, 2.72)
Heterogeneity: τ² = 1.40, I² = 89.30%, H² = 9.34
Test of θ = 0: z = -0.03, p = 0.98
1/16
1/4
1
4
16
Random-effects REML model

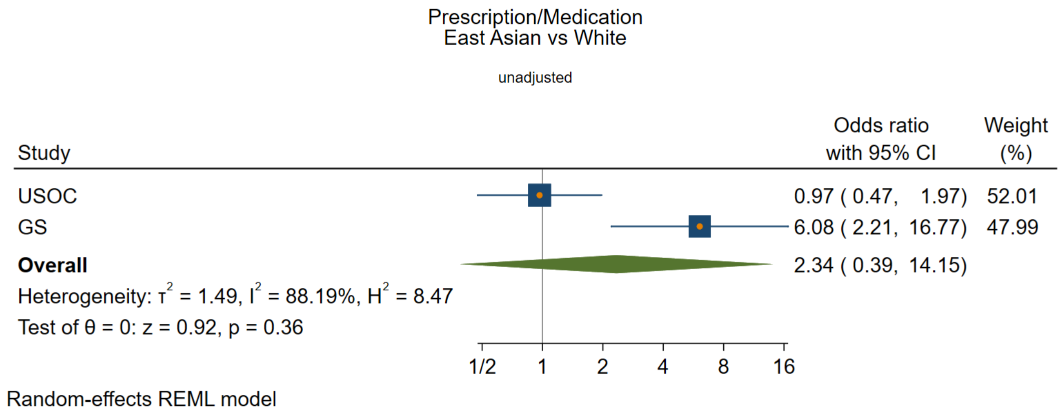
Prescription/Medication
East Asian vs White
unadjusted
Study
Odds ratio with 95% CI
Weight (%)
USOC 0.97 (0.47, 1.97) 52.01
GS 6.08 (2.21, 16.77) 47.99
Overall 2.34 (0.39, 14.15)
Heterogeneity: τ² = 1.49, I² = 88.19%, H² = 8.47
Test of θ = 0: z = 0.92, p = 0.36
1/2
1
2
4
8
16
Random-effects REML model

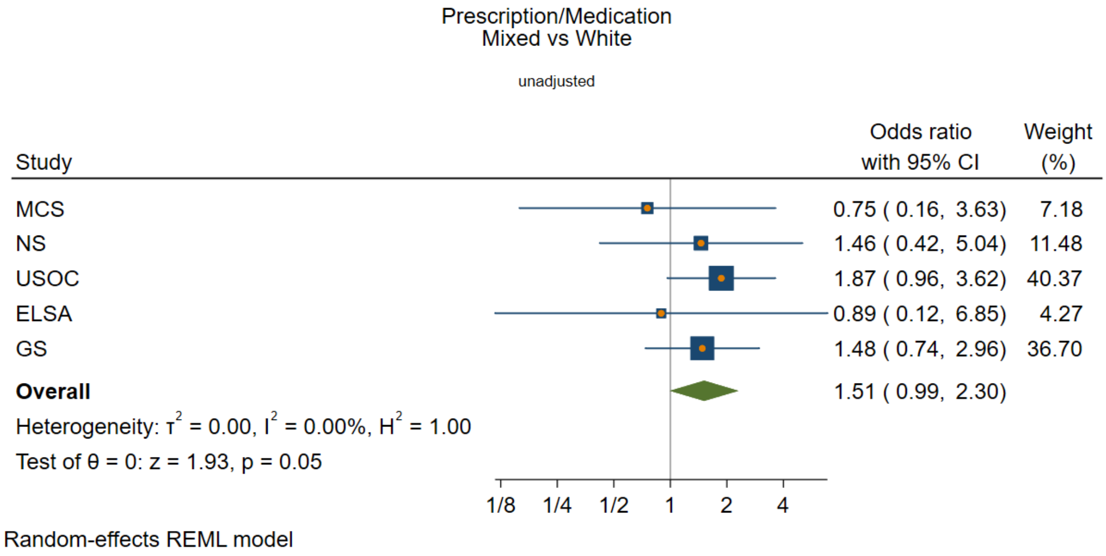

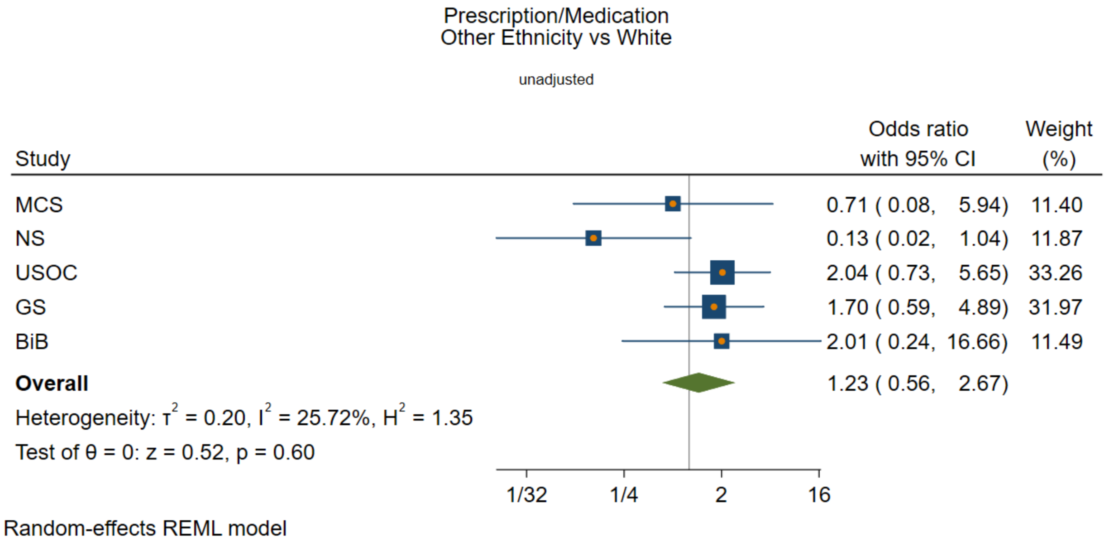

Maddock J, *et al. BMJ Open* 2022; 12:e064981. doi: 10.1136/bmjopen-2022-064981

Basic adjustment

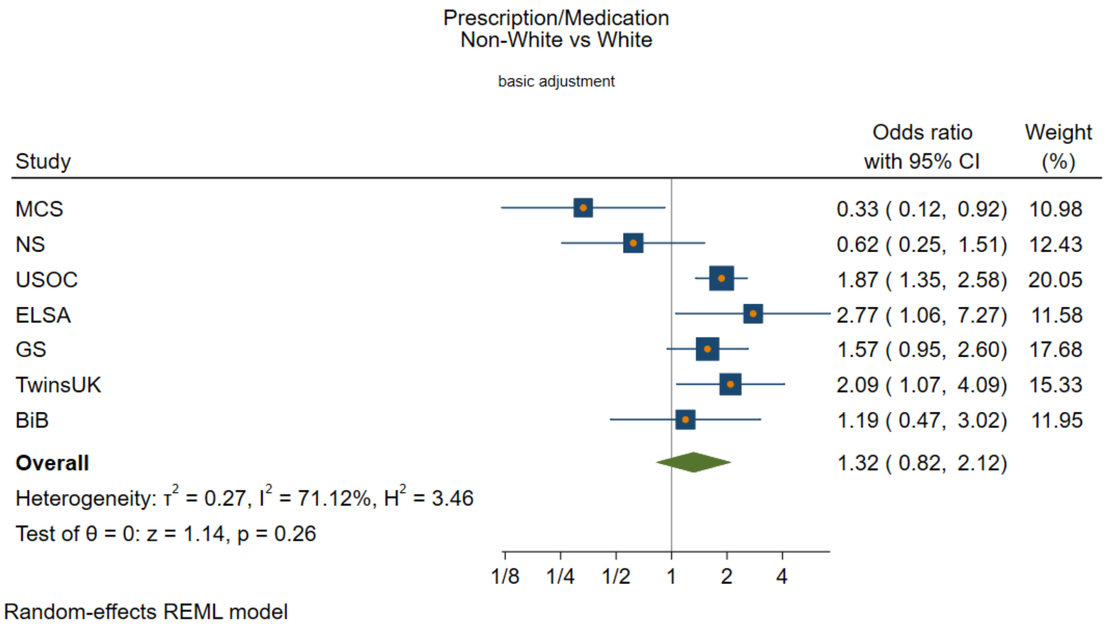

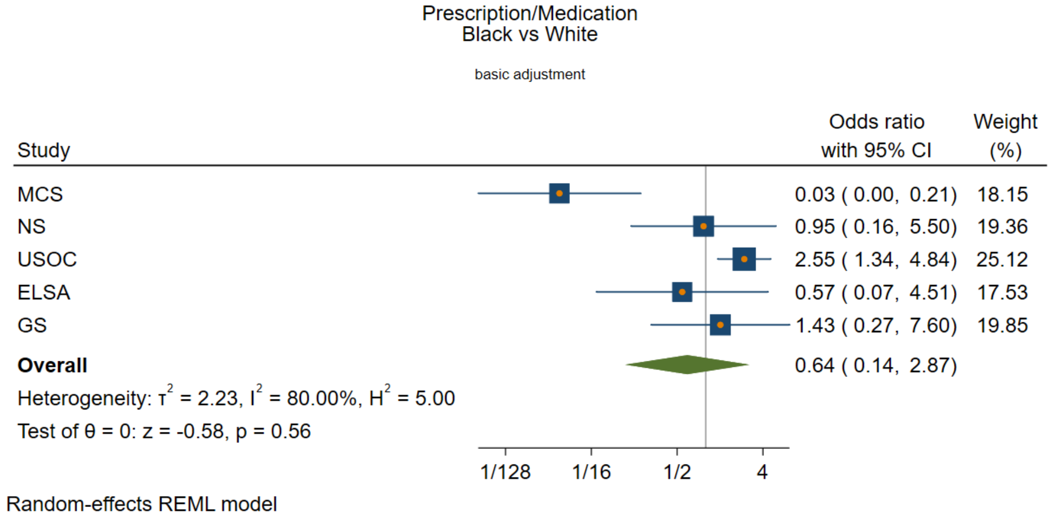

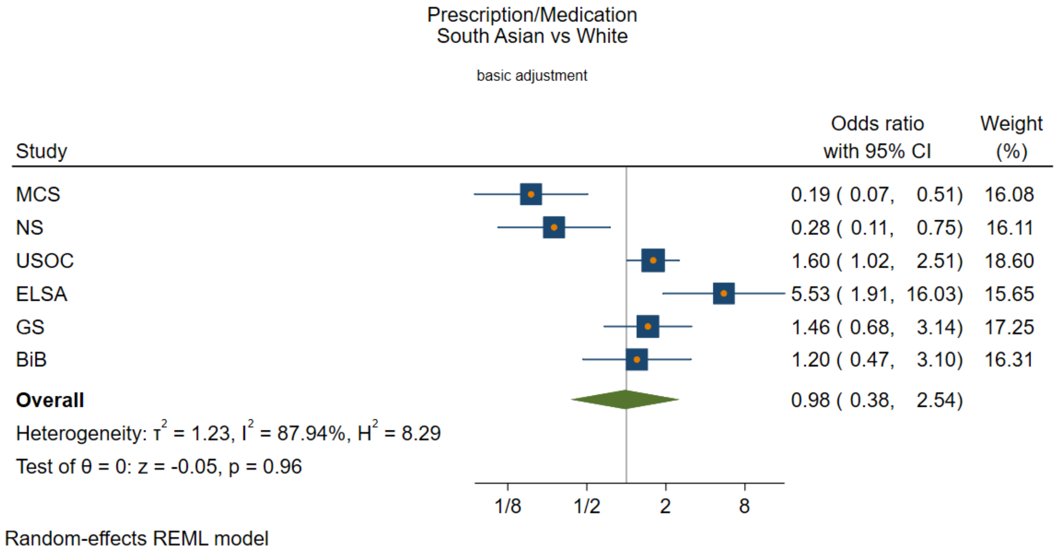

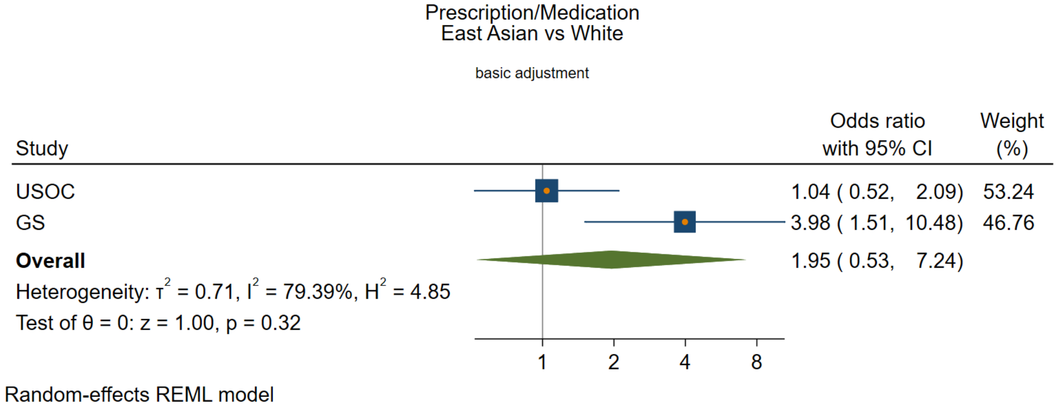

Maddock J, *et al. BMJ Open* 2022; 12:e064981. doi: 10.1136/bmjopen-2022-064981

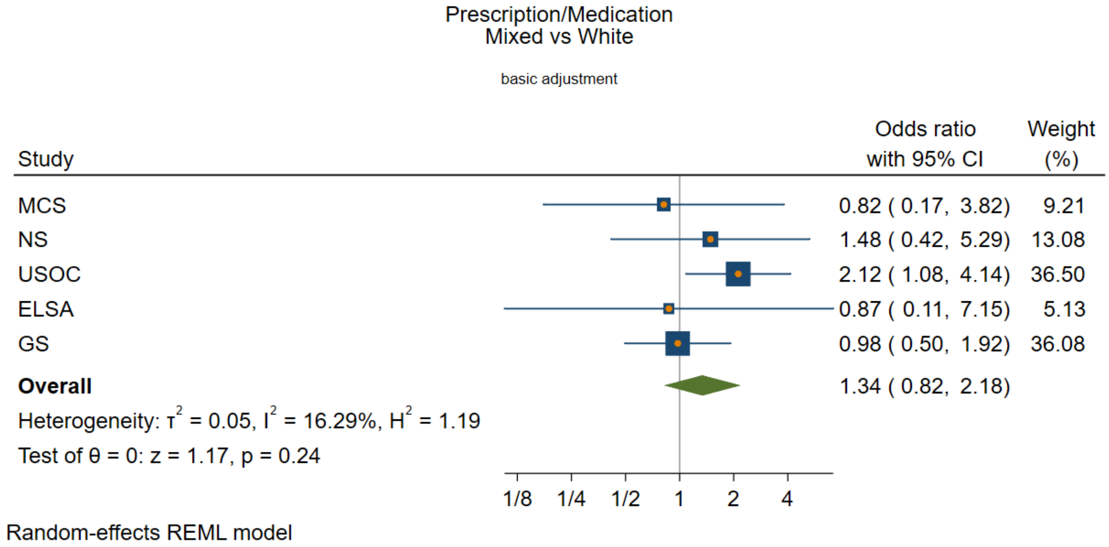
Prescription/Medication
Mixed vs White
basic adjustment
Study
Odds ratio with 95% CI
Weight (%)
MCS 0.82 (0.17, 3.82) 9.21
NS 1.48 (0.42, 5.29) 13.08
USOC 2.12 (1.08, 4.14) 36.50
ELSA 0.87 (0.11, 7.15) 5.13
GS 0.98 (0.50, 1.92) 36.08
Overall 1.34 (0.82, 2.18)
Heterogeneity: τ² = 0.05, I² = 16.29%, H² = 1.19
Test of θ = 0: z = 1.17, p = 0.24
1/8
1/4
1/2
1
2
4
Random-effects REML model

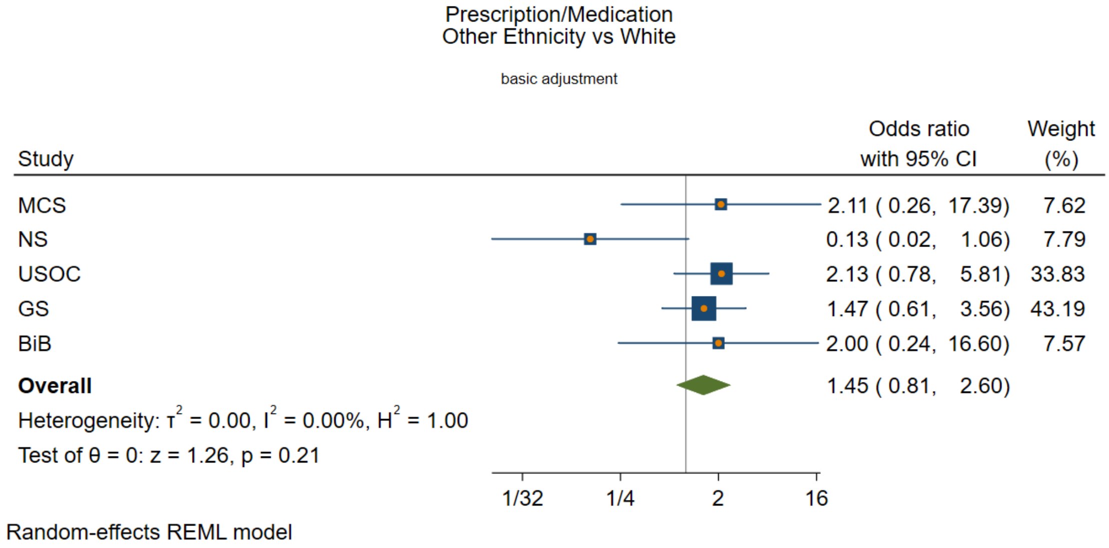
Prescription/Medication
Other Ethnicity vs White
basic adjustment
Study
Odds ratio with 95% CI
Weight (%)
MCS 2.11 (0.26, 17.39) 7.62
NS 0.13 (0.02, 1.06) 7.79
USOC 2.13 (0.78, 5.81) 33.83
GS 1.47 (0.61, 3.56) 43.19
BiB 2.00 (0.24, 16.60) 7.57
Overall 1.45 (0.81, 2.60)
Heterogeneity: τ² = 0.00, I² = 0.00%, H² = 1.00
Test of θ = 0: z = 1.26, p = 0.21
1/32
1/4
2
16
Random-effects REML model

Full adjustment

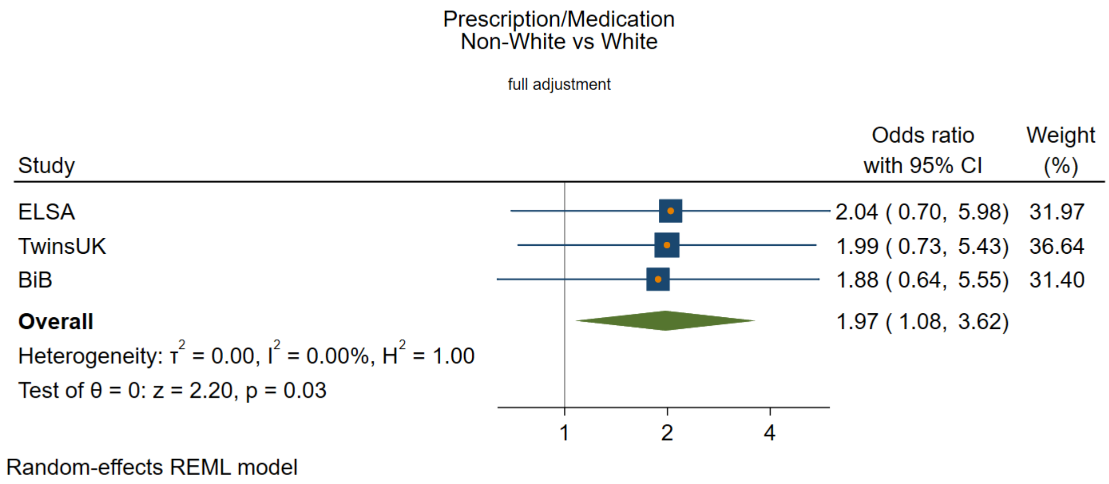

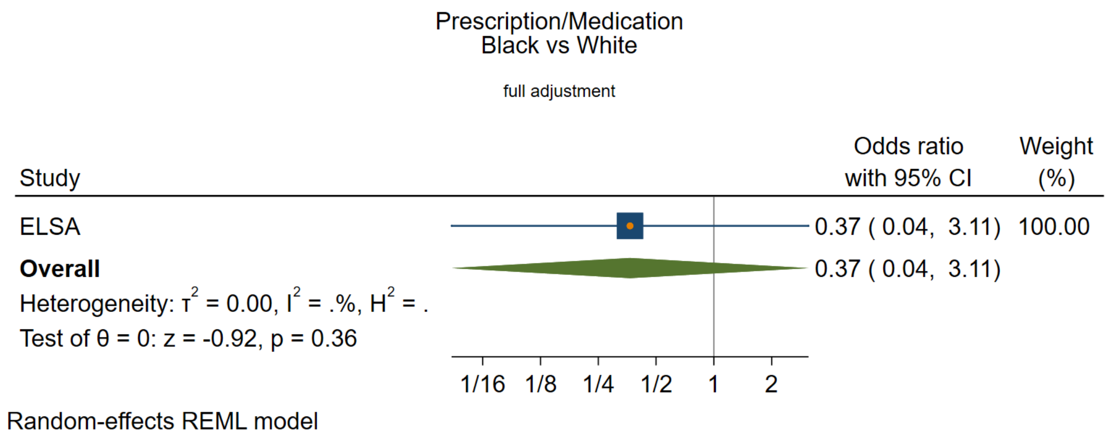
Prescription/Medication
Black vs White
full adjustment
Study
Odds ratio with 95% CI
Weight (%)
ELSA
0.37 (0.04, 3.11)
100.00
Overall
0.37 (0.04, 3.11)
Heterogeneity: τ² = 0.00, I² = .%, H² = .
Test of θ = 0: z = -0.92, p = 0.36
1/16
1/8
1/4
1/2
1
2
Random-effects REML model

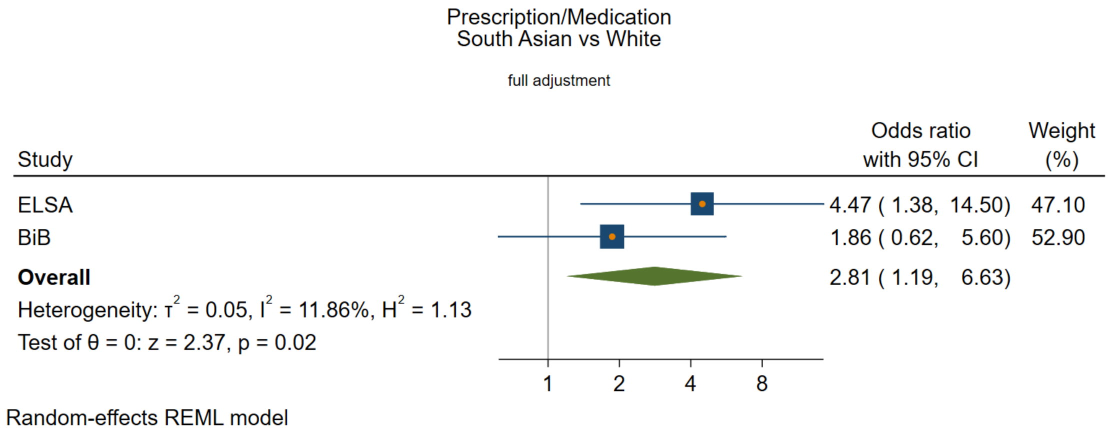
Prescription/Medication
South Asian vs White
full adjustment
Study
Odds ratio with 95% CI
Weight (%)
ELSA
4.47 (1.38, 14.50)
47.10
BiB
1.86 (0.62, 5.60)
52.90
Overall
2.81 (1.19, 6.63)
Heterogeneity: τ² = 0.05, I² = 11.86%, H² = 1.13
Test of θ = 0: z = 2.37, p = 0.02
1
2
4
8
Random-effects REML model

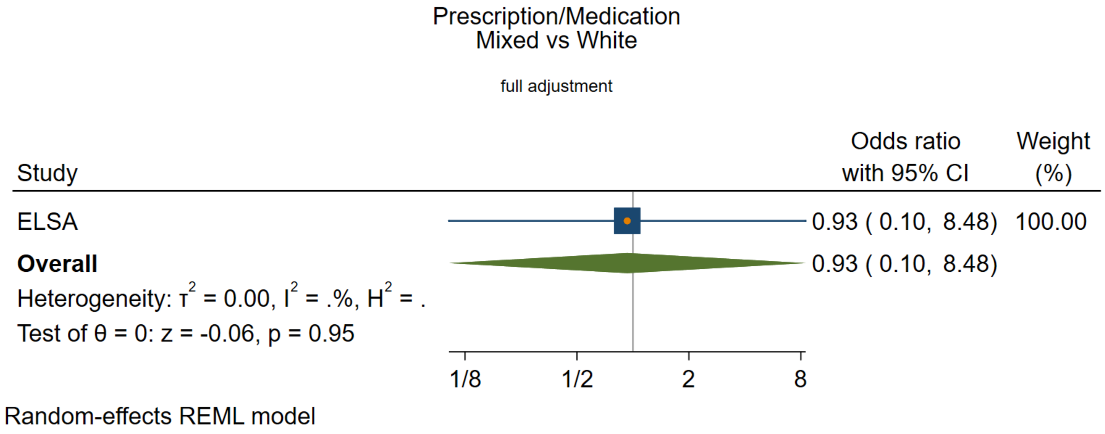
Prescription/Medication
Mixed vs White
full adjustment
Study
Odds ratio with 95% CI
Weight (%)
ELSA
0.93 (0.10, 8.48)
100.00
Overall
0.93 (0.10, 8.48)
Heterogeneity: τ² = 0.00, I² = .%, H² = .
Test of θ = 0: z = -0.06, p = 0.95
1/8
1/2
2
8
Random-effects REML model

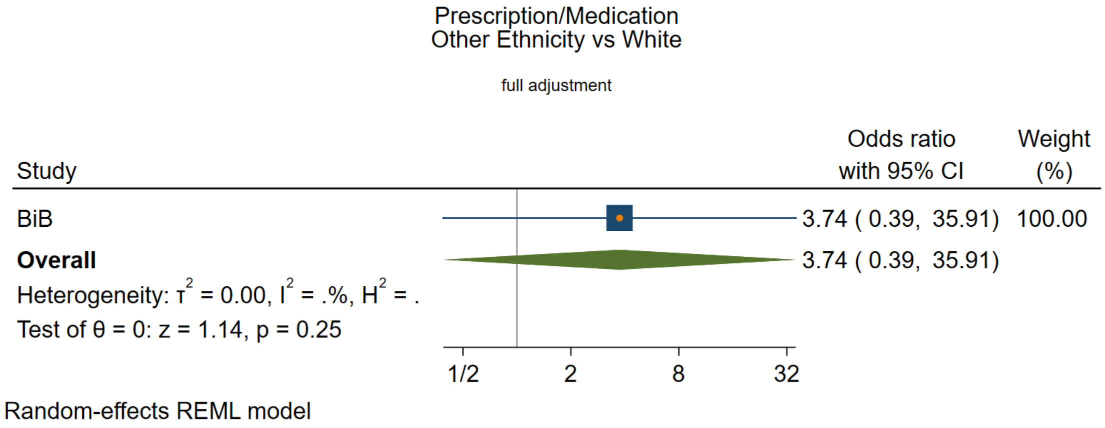
Prescription/Medication
Other Ethnicity vs White
full adjustment
Study
Odds ratio with 95% CI
Weight (%)
BiB
3.74 (0.39, 35.91)
100.00
Overall
3.74 (0.39, 35.91)
Heterogeneity: τ² = 0.00, I² = .%, H² = .
Test of θ = 0: z = 1.14, p = 0.25
1/2
2
8
32
Random-effects REML model

Procedures/surgery

Sex
Unadjusted

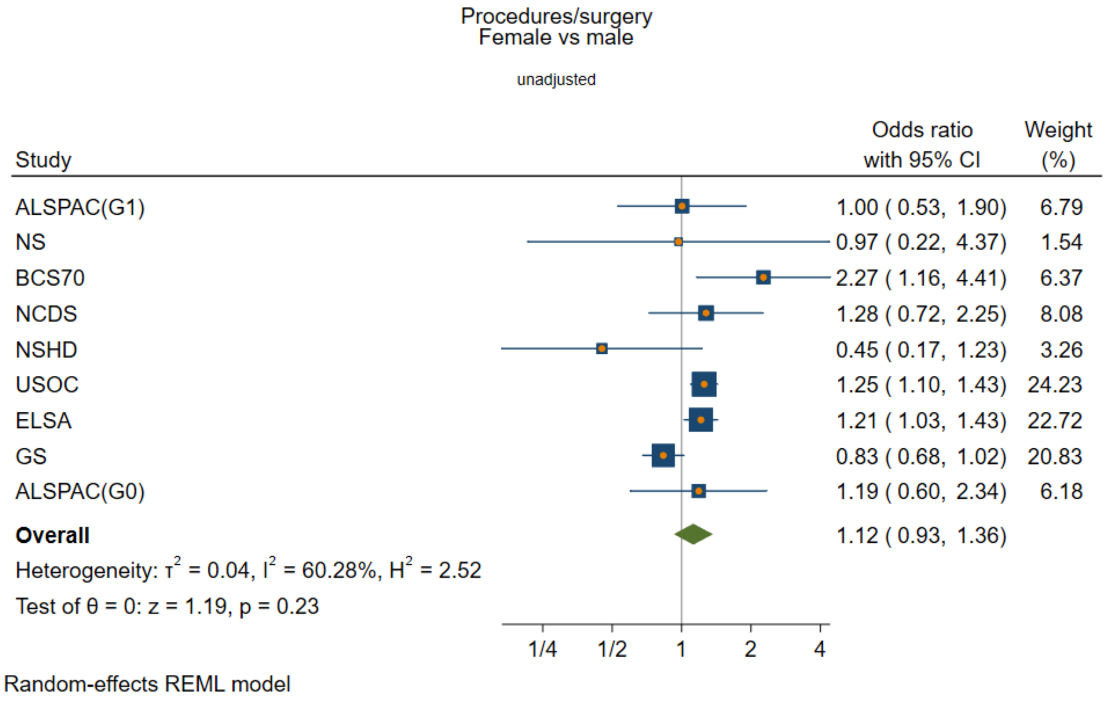

Maddock J, *et al. BMJ Open* 2022; 12:e064981. doi: 10.1136/bmjopen-2022-064981

Basic adjustment

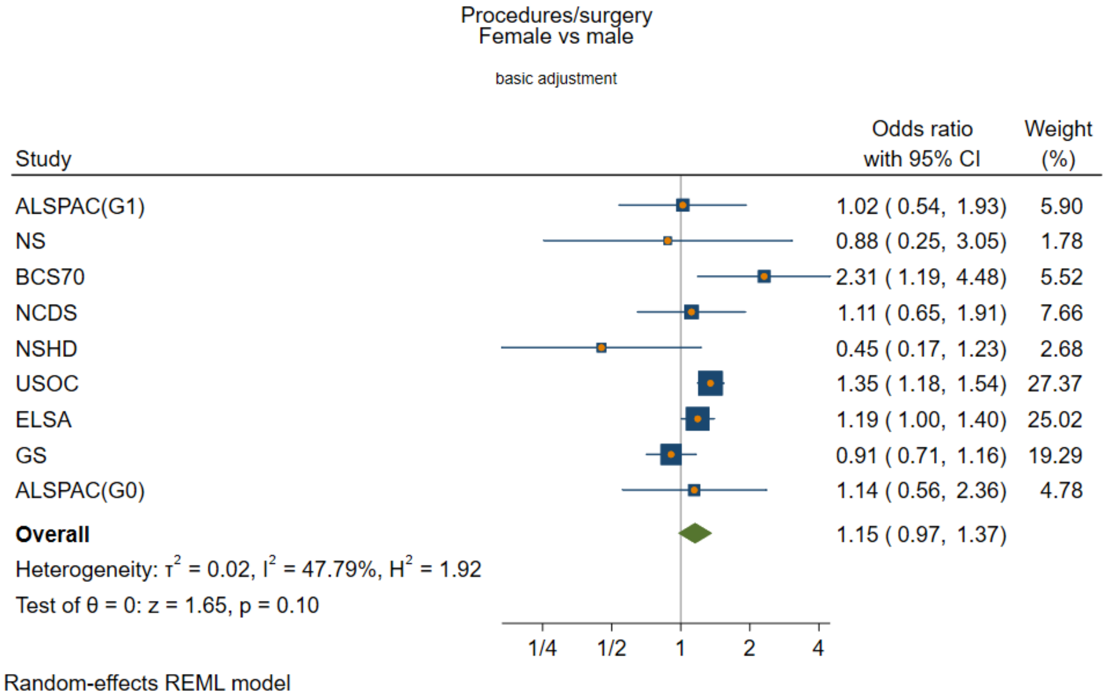

Full adjustment

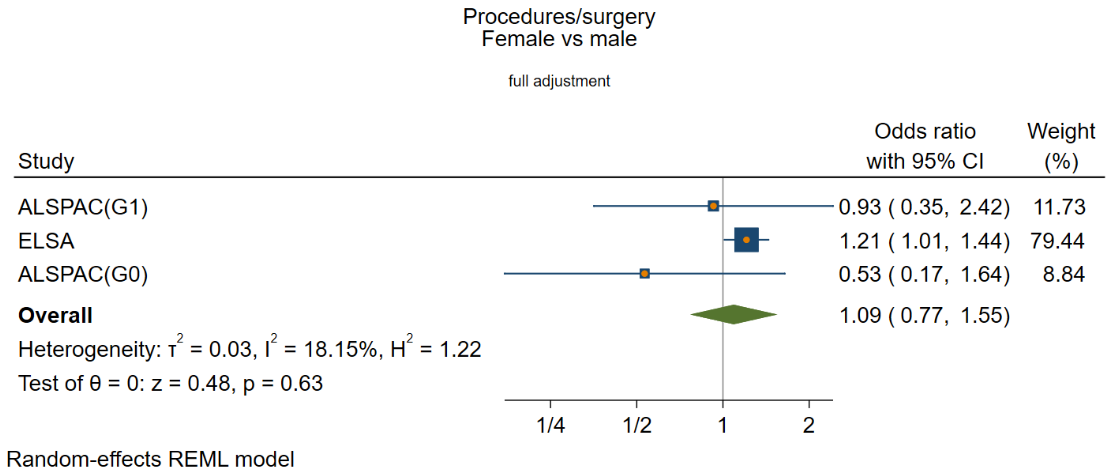

Occupational class
<u>Unadjusted</u>

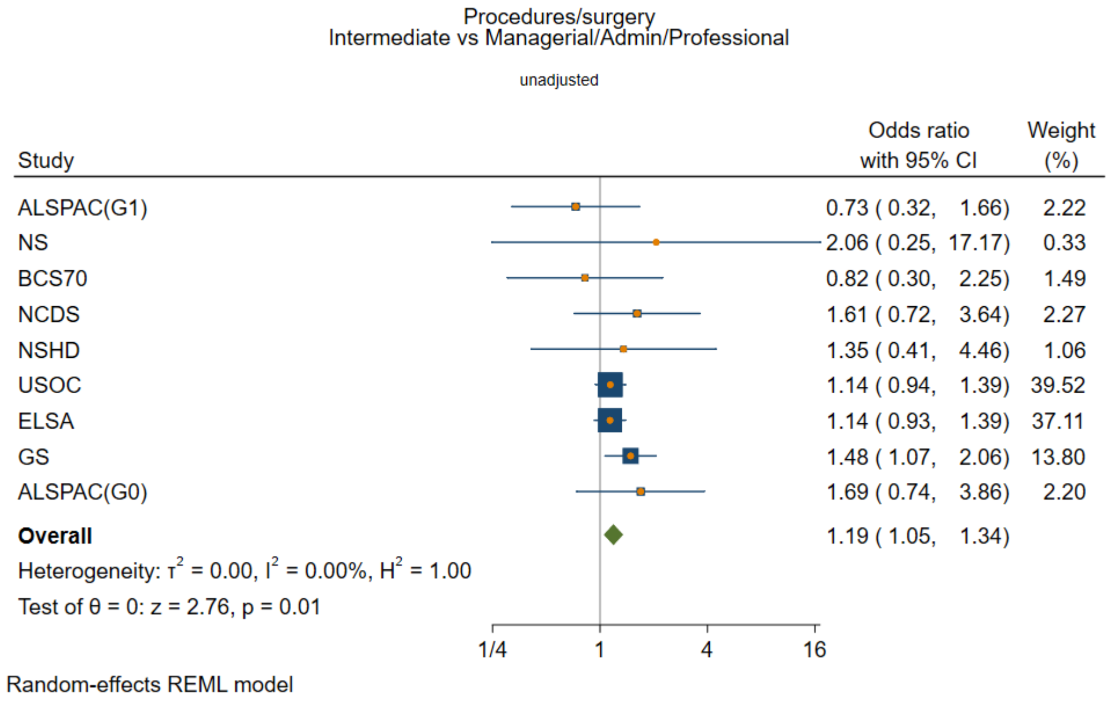

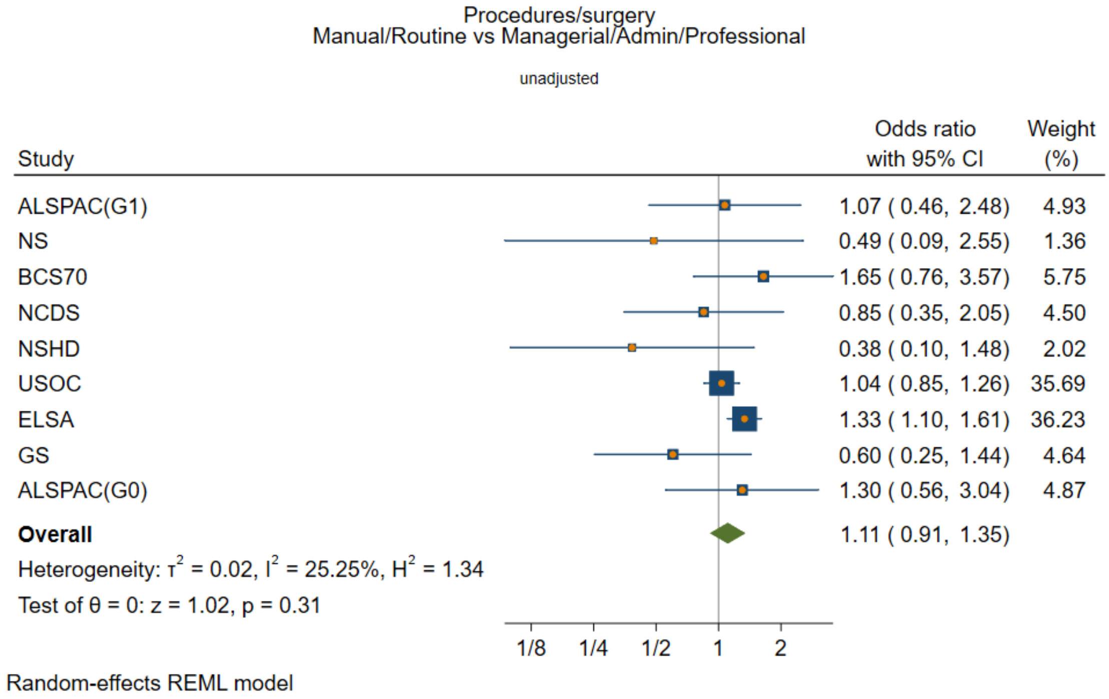

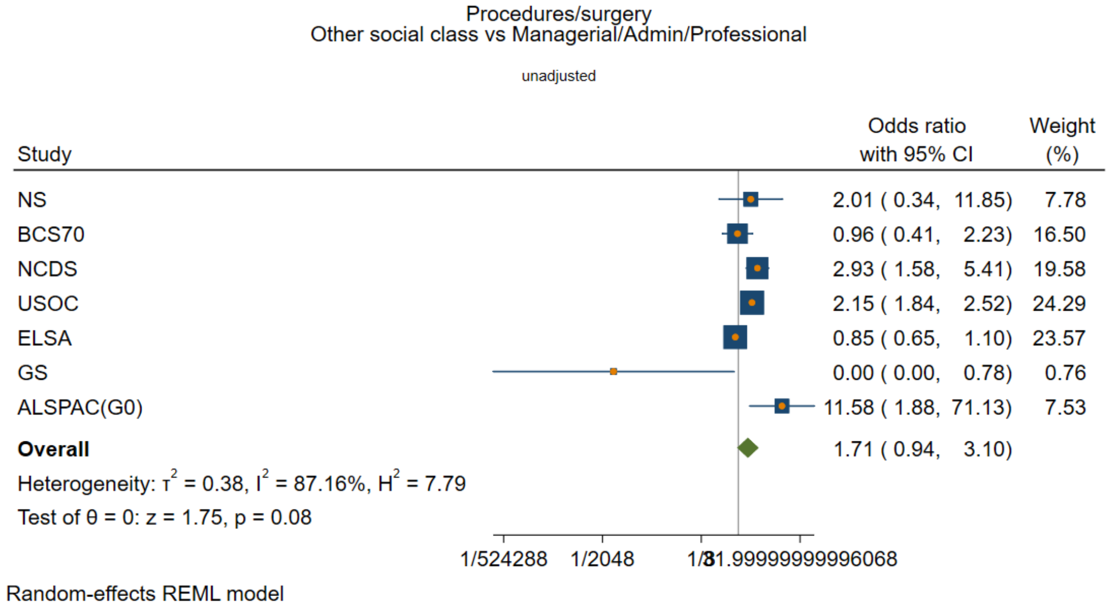

Maddock J, *et al. BMJ Open* 2022; 12:e064981. doi: 10.1136/bmjopen-2022-064981

Basic adjustment

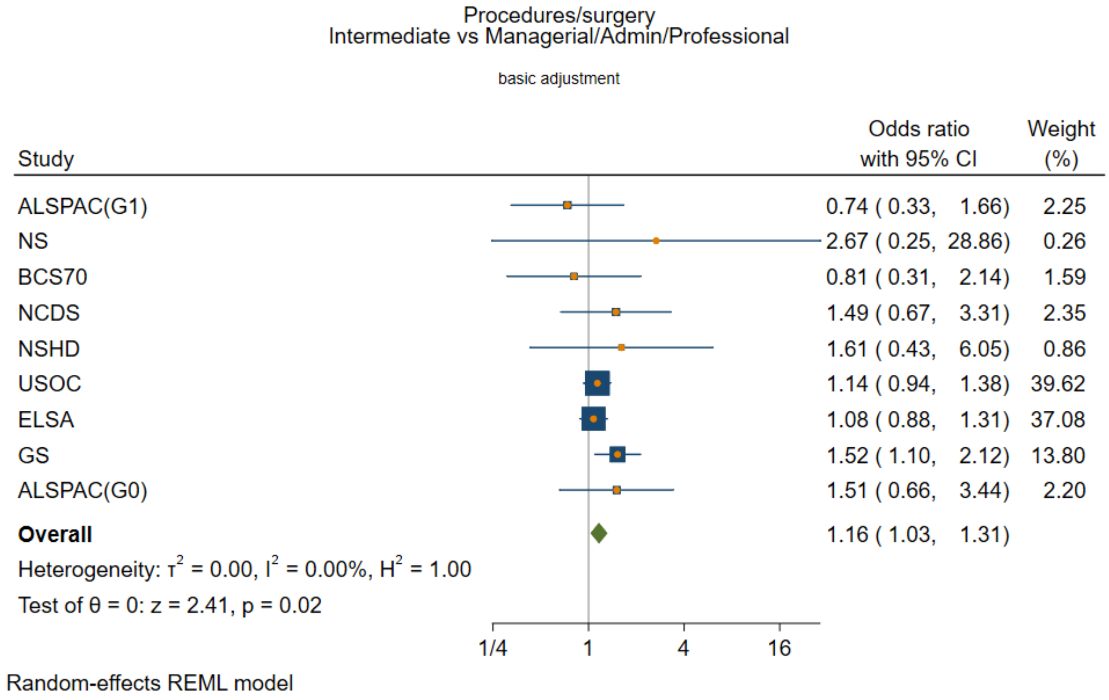

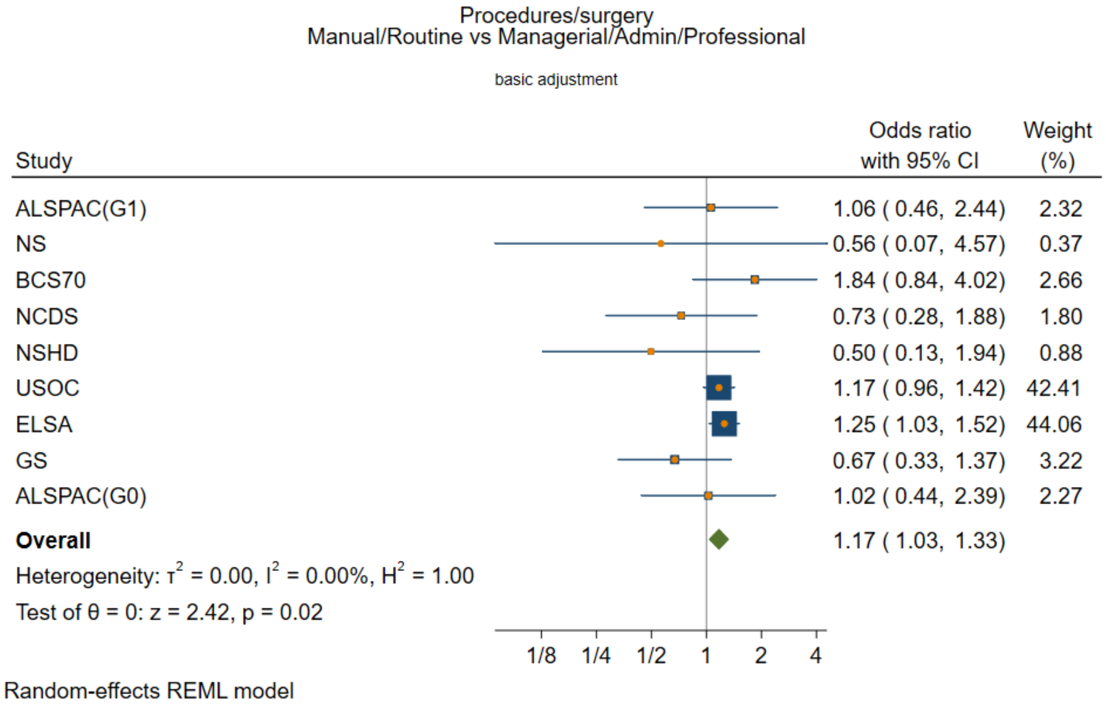
Procedures/surgery
Manual/Routine vs Managerial/Admin/Professional
basic adjustment
Study
Odds ratio with 95% CI
Weight (%)
ALSPAC(G1) 1.06 (0.46, 2.44) 2.32
NS 0.56 (0.07, 4.57) 0.37
BCS70 1.84 (0.84, 4.02) 2.66
NCDS 0.73 (0.28, 1.88) 1.80
NSHD 0.50 (0.13, 1.94) 0.88
USOC 1.17 (0.96, 1.42) 42.41
ELSA 1.25 (1.03, 1.52) 44.06
GS 0.67 (0.33, 1.37) 3.22
ALSPAC(G0) 1.02 (0.44, 2.39) 2.27
Overall 1.17 (1.03, 1.33)
Heterogeneity: τ² = 0.00, I² = 0.00%, H² = 1.00
Test of θ = 0: z = 2.42, p = 0.02
1/8 1/4 1/2 1 2 4
Random-effects REML model

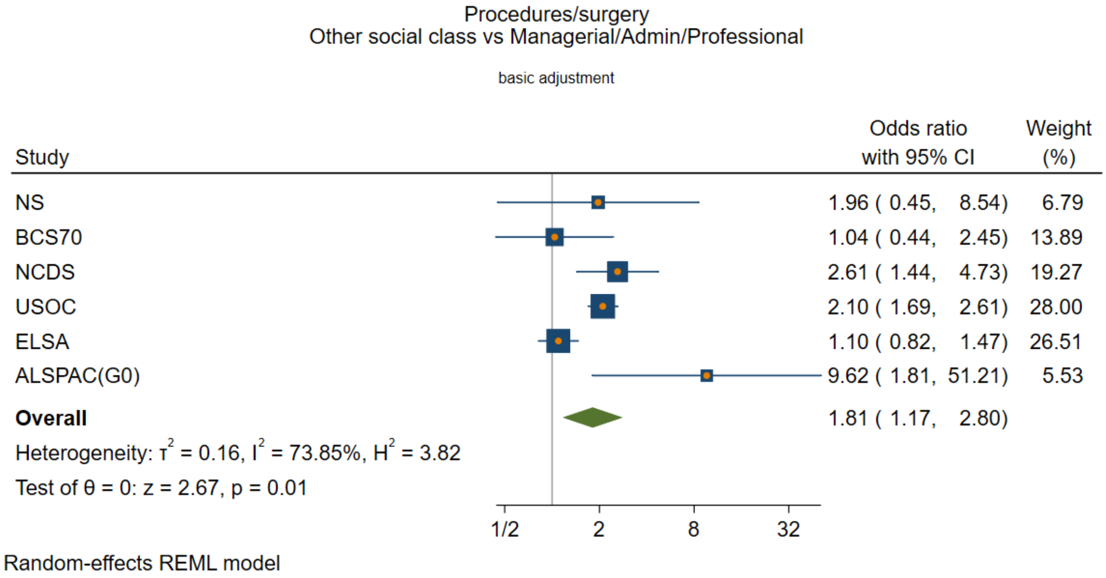
Procedures/surgery
Other social class vs Managerial/Admin/Professional
basic adjustment
Study
Odds ratio with 95% CI
Weight (%)
NS 1.96 (0.45, 8.54) 6.79
BCS70 1.04 (0.44, 2.45) 13.89
NCDS 2.61 (1.44, 4.73) 19.27
USOC 2.10 (1.69, 2.61) 28.00
ELSA 1.10 (0.82, 1.47) 26.51
ALSPAC(G0) 9.62 (1.81, 51.21) 5.53
Overall 1.81 (1.17, 2.80)
Heterogeneity: τ² = 0.16, I² = 73.85%, H² = 3.82
Test of θ = 0: z = 2.67, p = 0.01
1/2 2 8 32
Random-effects REML model

Full adjustment

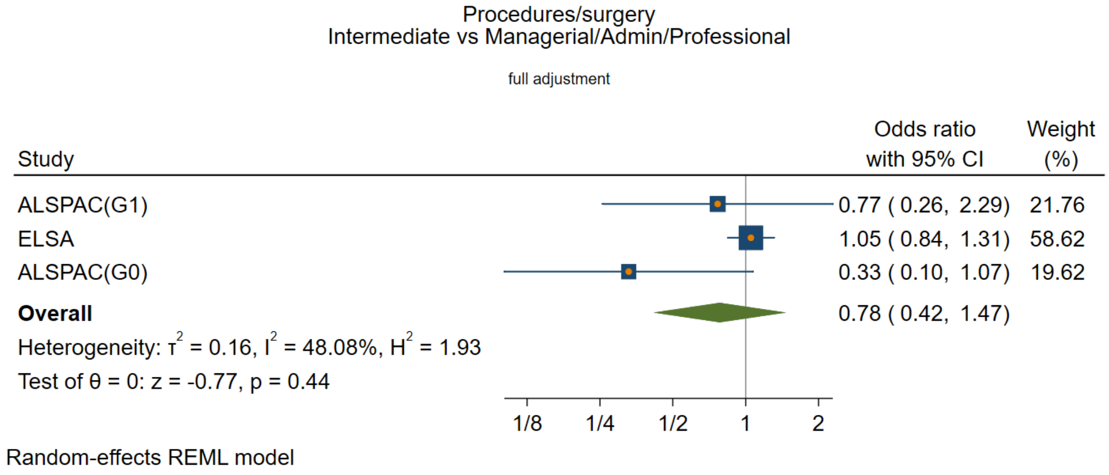

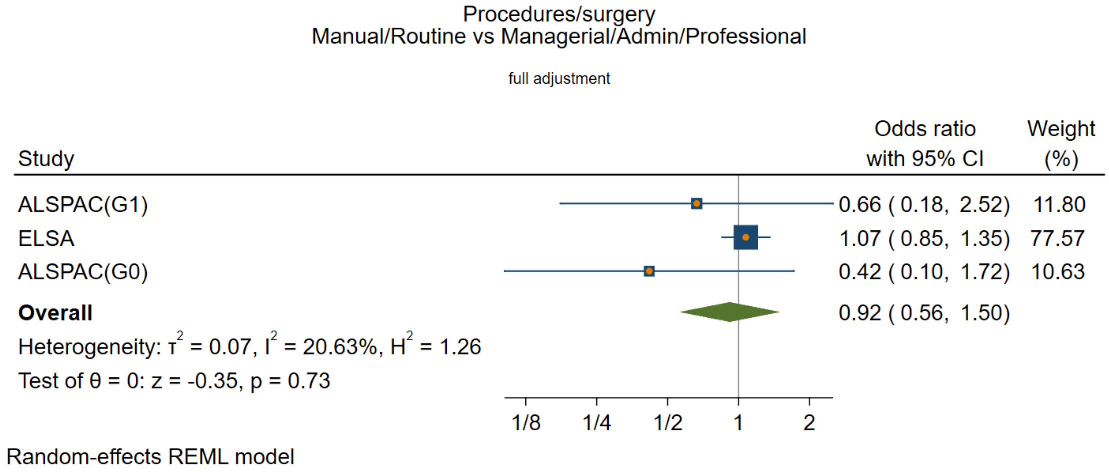

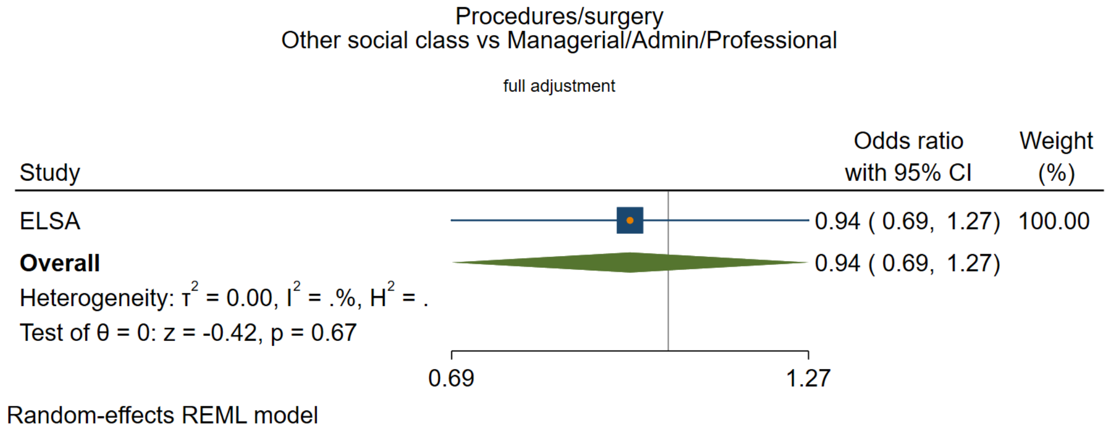

Maddock J, *et al. BMJ Open* 2022; 12:e064981. doi: 10.1136/bmjopen-2022-064981

Education
<u>unadjusted</u>

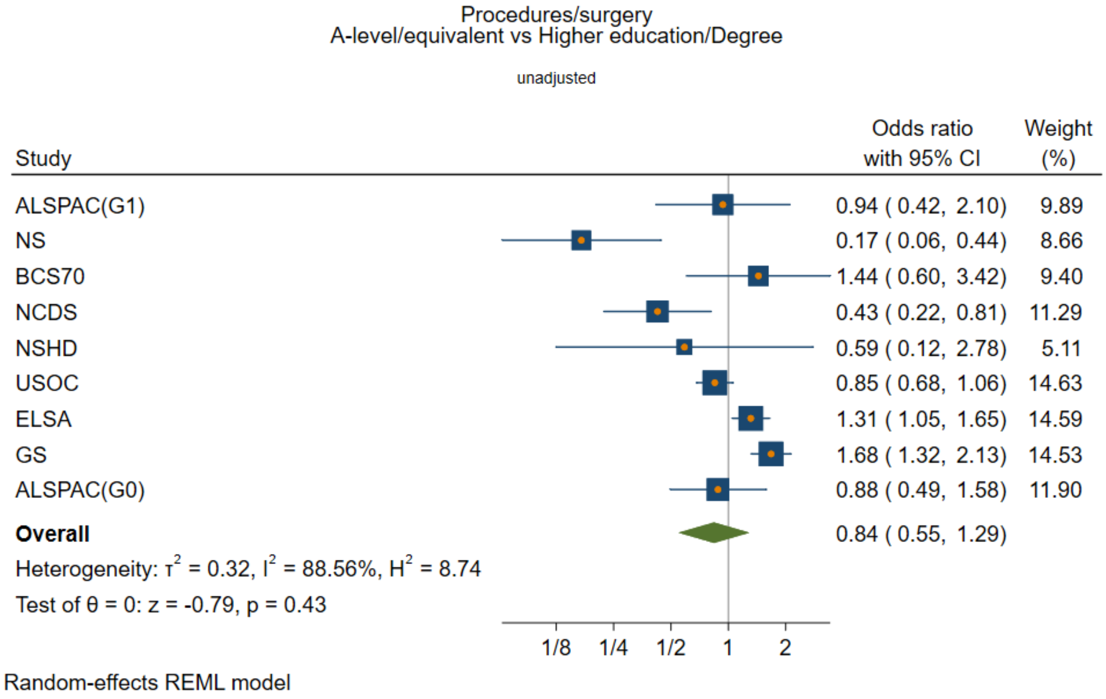

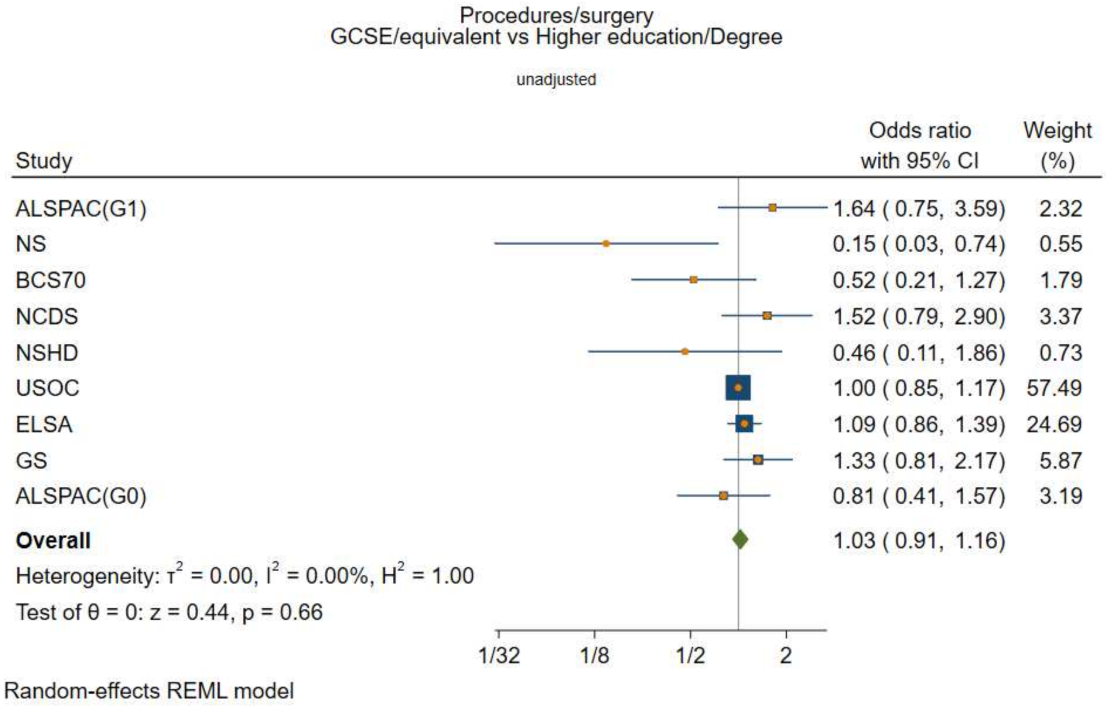

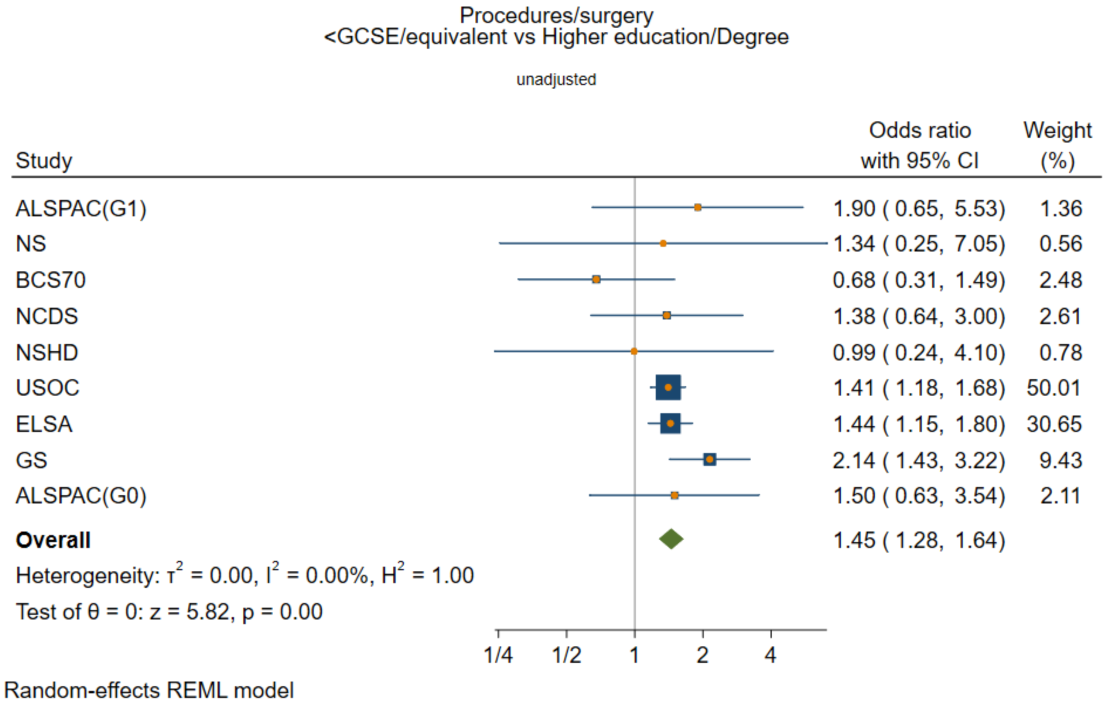

Maddock J, *et al. BMJ Open* 2022; 12:e064981. doi: 10.1136/bmjopen-2022-064981

Basic adjustment

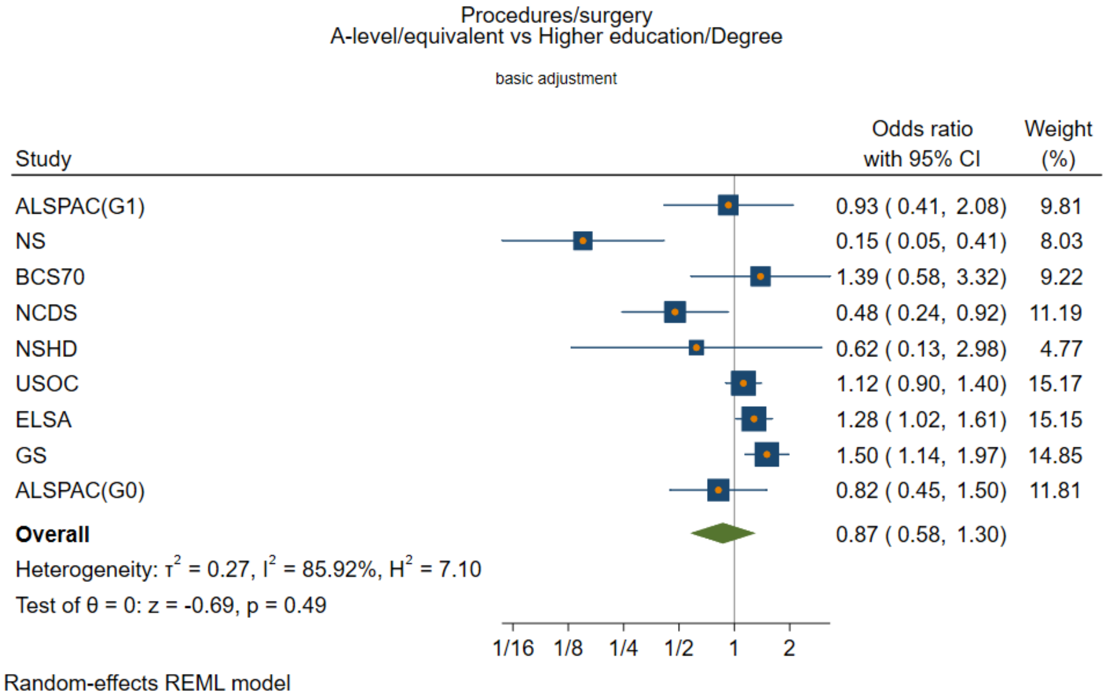

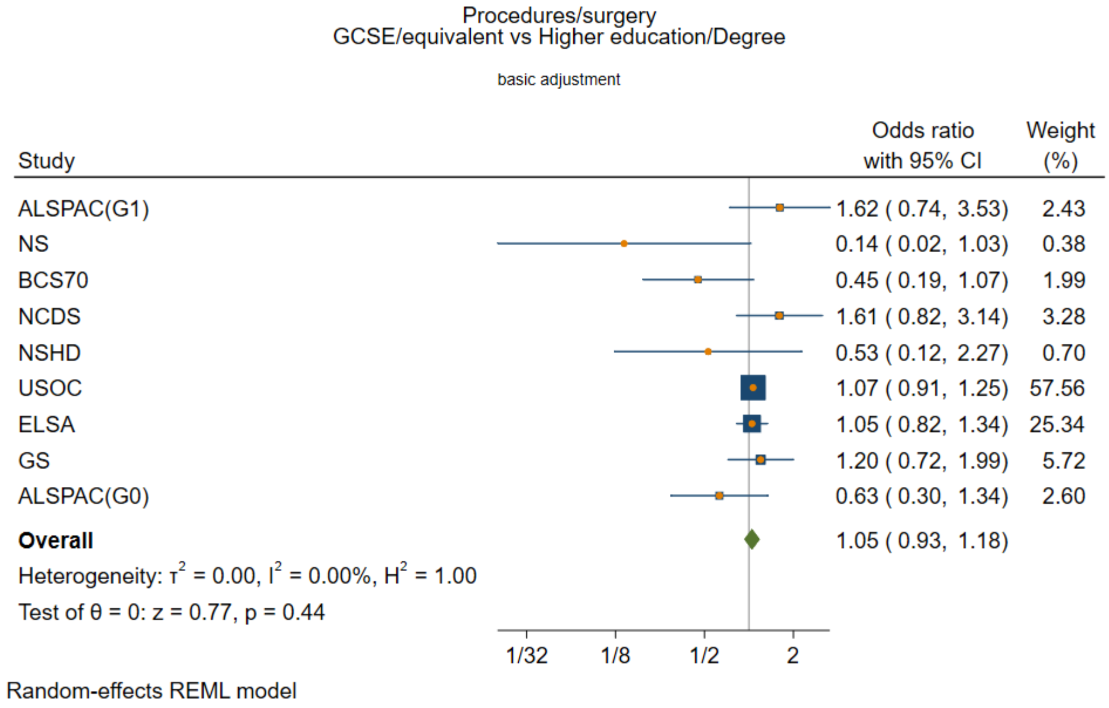
Procedures/surgery
GCSE/equivalent vs Higher education/Degree
basic adjustment
Study
Odds ratio with 95% CI
Weight (%)
ALSPAC(G1) 1.62 (0.74, 3.53) 2.43
NS 0.14 (0.02, 1.03) 0.38
BCS70 0.45 (0.19, 1.07) 1.99
NCDS 1.61 (0.82, 3.14) 3.28
NSHD 0.53 (0.12, 2.27) 0.70
USOC 1.07 (0.91, 1.25) 57.56
ELSA 1.05 (0.82, 1.34) 25.34
GS 1.20 (0.72, 1.99) 5.72
ALSPAC(G0) 0.63 (0.30, 1.34) 2.60
Overall 1.05 (0.93, 1.18)
Heterogeneity: τ^2 = 0.00, I^2 = 0.00%, H^2 = 1.00
Test of θ = 0: z = 0.77, p = 0.44
1/32
1/8
1/2
2
Random-effects REML model

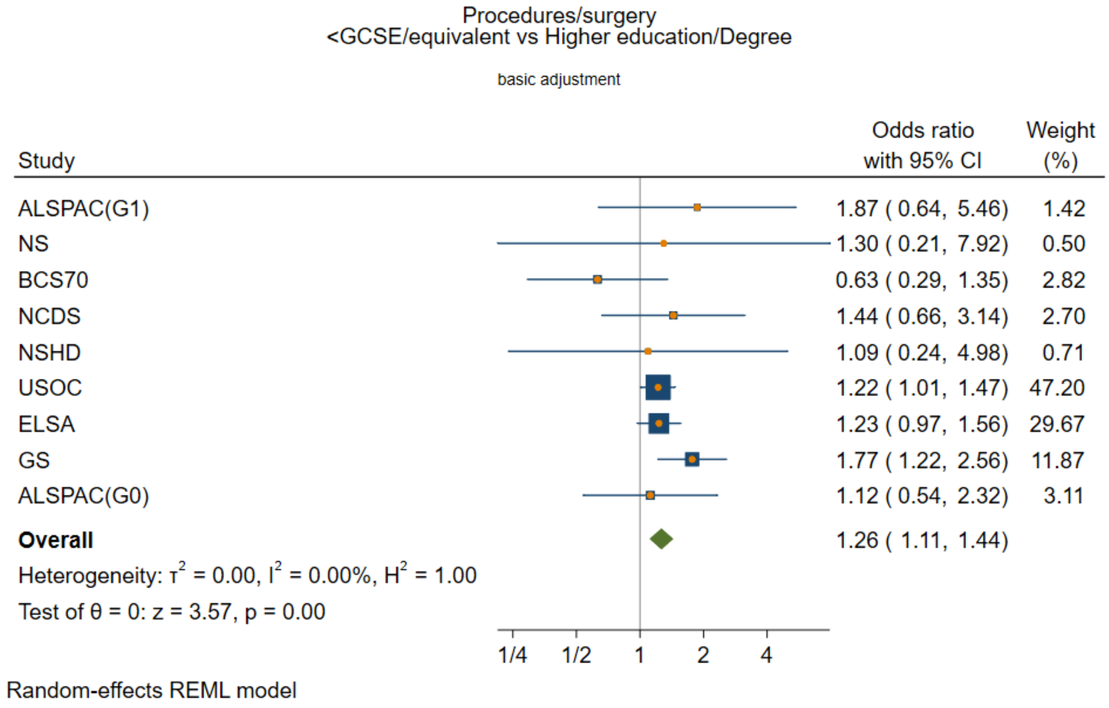
Procedures/surgery
<GCSE/equivalent vs Higher education/Degree
basic adjustment
Study
Odds ratio with 95% CI
Weight (%)
ALSPAC(G1) 1.87 (0.64, 5.46) 1.42
NS 1.30 (0.21, 7.92) 0.50
BCS70 0.63 (0.29, 1.35) 2.82
NCDS 1.44 (0.66, 3.14) 2.70
NSHD 1.09 (0.24, 4.98) 0.71
USOC 1.22 (1.01, 1.47) 47.20
ELSA 1.23 (0.97, 1.56) 29.67
GS 1.77 (1.22, 2.56) 11.87
ALSPAC(G0) 1.12 (0.54, 2.32) 3.11
Overall 1.26 (1.11, 1.44)
Heterogeneity: τ^2 = 0.00, I^2 = 0.00%, H^2 = 1.00
Test of θ = 0: z = 3.57, p = 0.00
1/4
1/2
1
2
4
Random-effects REML model

Full adjustment

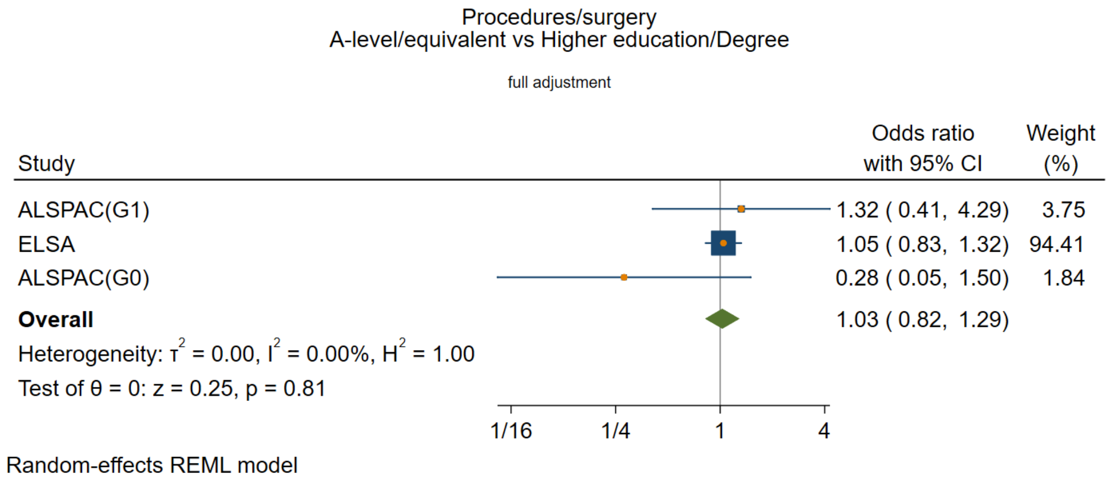

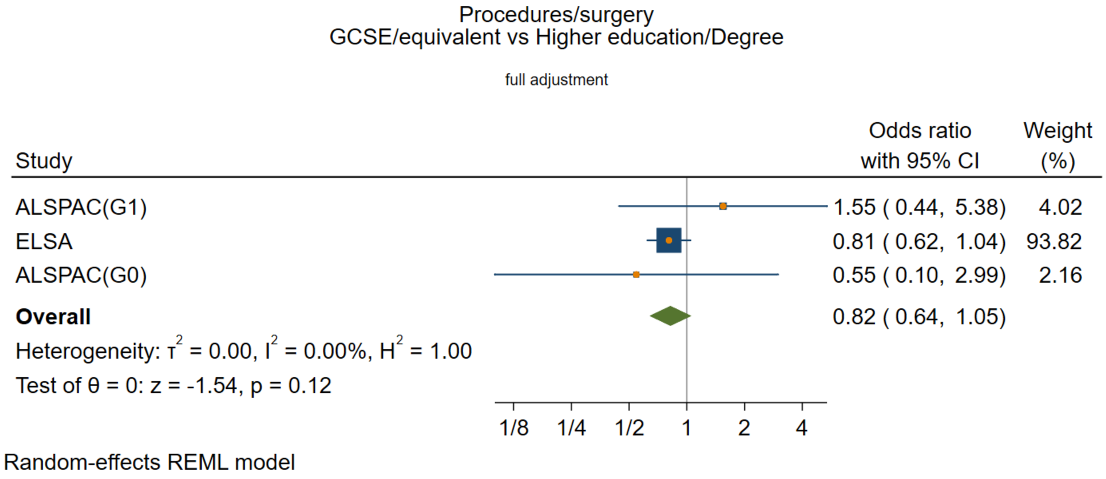
Procedures/surgery
GCSE/equivalent vs Higher education/Degree
full adjustment
Study
Odds ratio with 95% CI
Weight (%)
ALSPAC(G1) 1.55 (0.44, 5.38) 4.02
ELSA 0.81 (0.62, 1.04) 93.82
ALSPAC(G0) 0.55 (0.10, 2.99) 2.16
Overall 0.82 (0.64, 1.05)
Heterogeneity: τ² = 0.00, I² = 0.00%, H² = 1.00
Test of θ = 0: z = -1.54, p = 0.12
1/8 1/4 1/2 1 2 4
Random-effects REML model

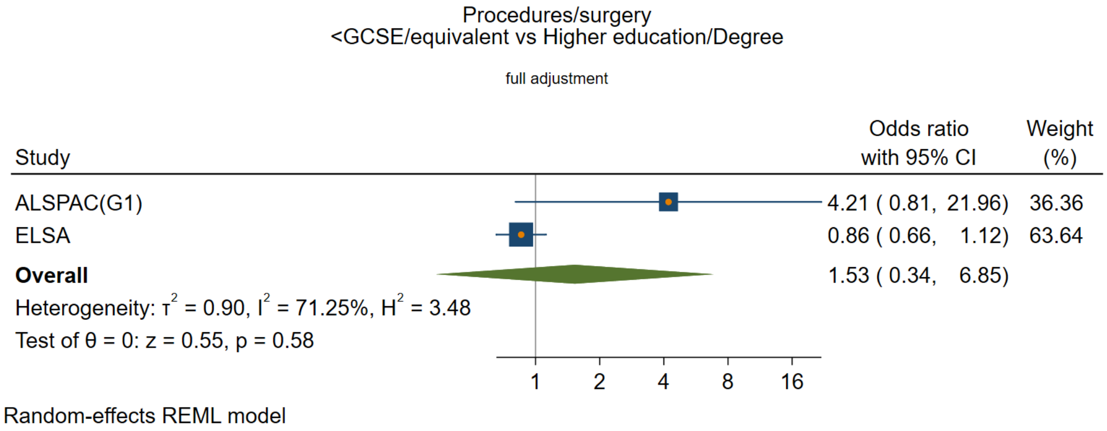
Procedures/surgery
<GCSE/equivalent vs Higher education/Degree
full adjustment
Study
Odds ratio with 95% CI
Weight (%)
ALSPAC(G1) 4.21 (0.81, 21.96) 36.36
ELSA 0.86 (0.66, 1.12) 63.64
Overall 1.53 (0.34, 6.85)
Heterogeneity: τ² = 0.90, I² = 71.25%, H² = 3.48
Test of θ = 0: z = 0.55, p = 0.58
1 2 4 8 16
Random-effects REML model

Age

<u>Unadjusted</u>

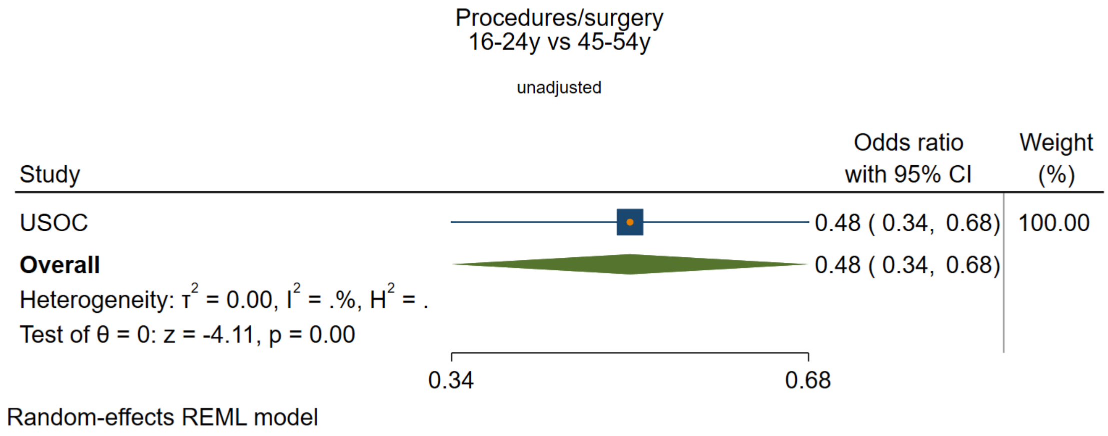

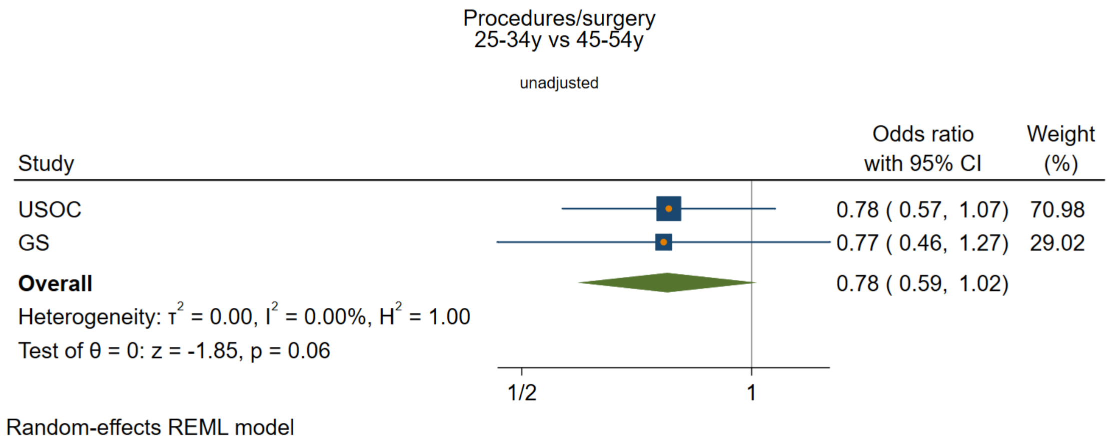

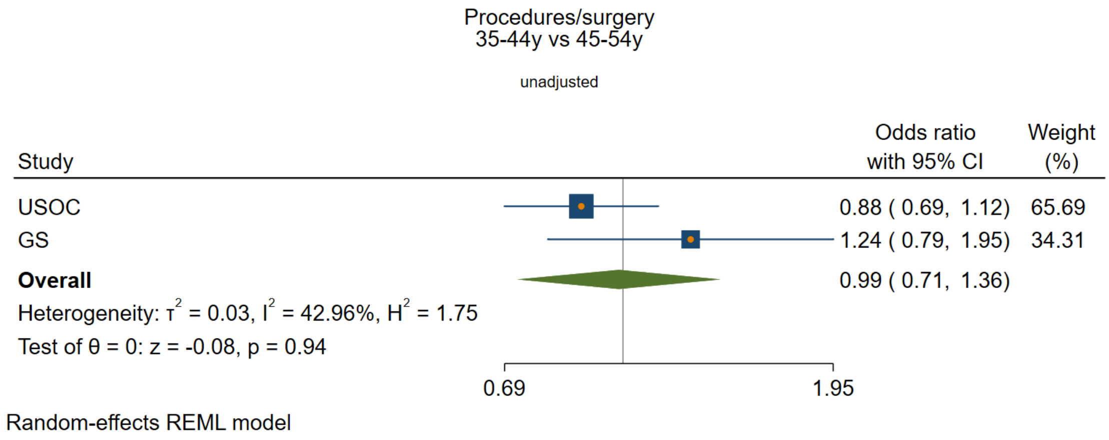

Maddock J, *et al. BMJ Open* 2022; 12:e064981. doi: 10.1136/bmjopen-2022-064981

Procedures/surgery
55-64y vs 45-54y

unadjusted

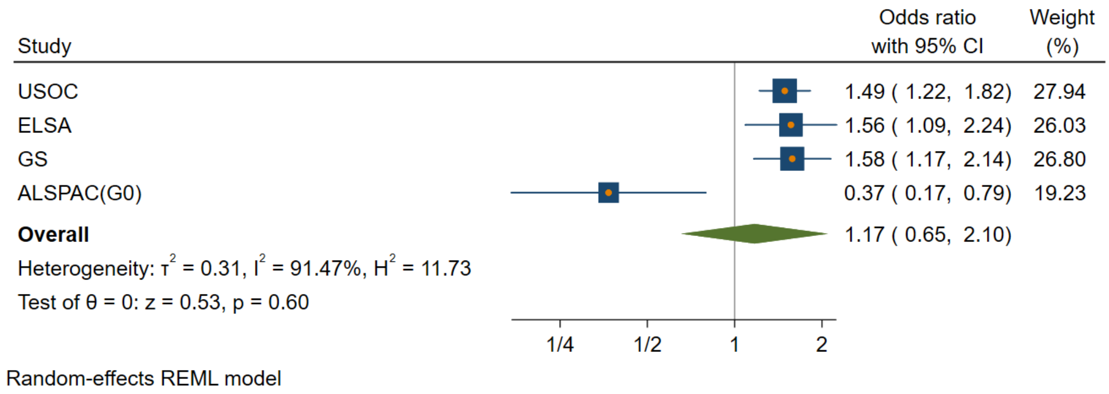

Procedures/surgery
65-74y vs 45-54y

unadjusted

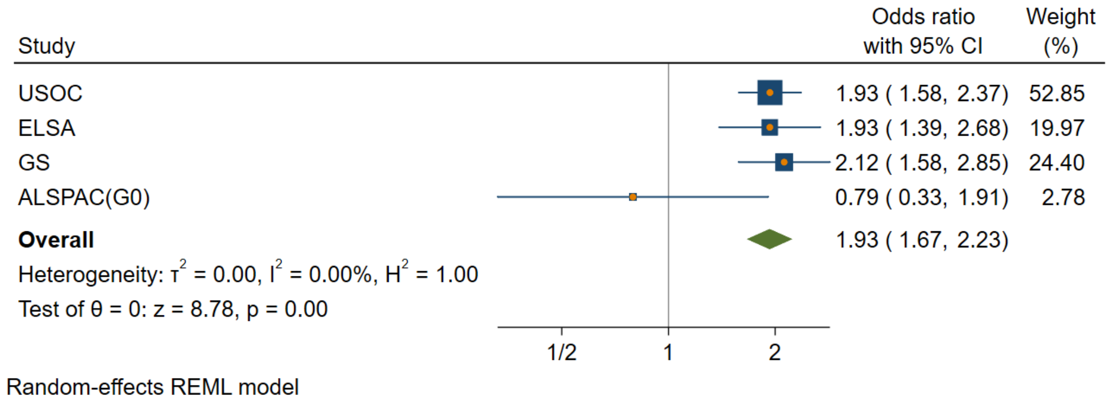

Maddock J, *et al. BMJ Open* 2022; 12:e064981. doi: 10.1136/bmjopen-2022-064981

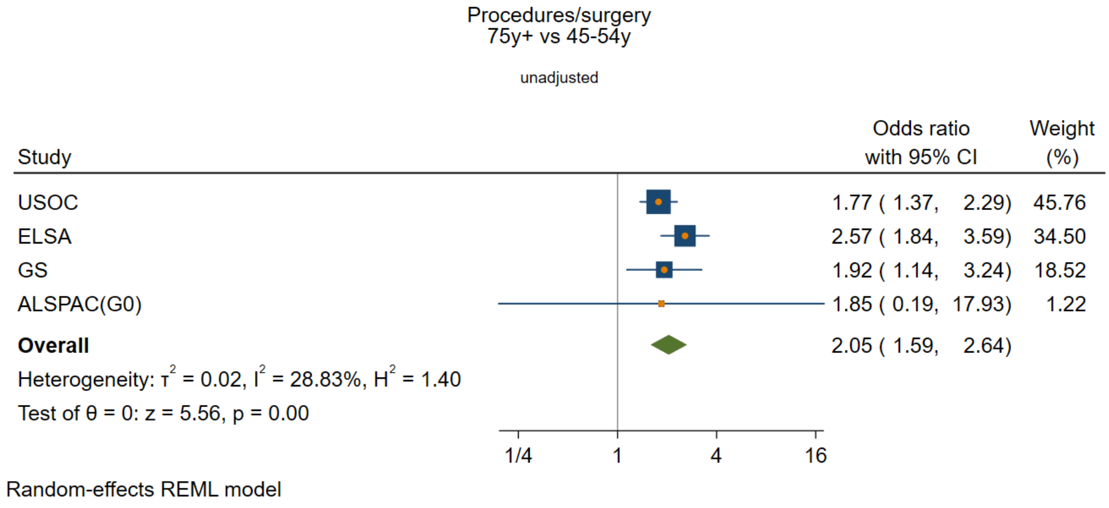
Procedures/surgery
75y+ vs 45-54y
unadjusted
Study
Odds ratio with 95% CI
Weight (%)
USOC 1.77 (1.37, 2.29) 45.76
ELSA 2.57 (1.84, 3.59) 34.50
GS 1.92 (1.14, 3.24) 18.52
ALSPAC(G0) 1.85 (0.19, 17.93) 1.22
Overall 2.05 (1.59, 2.64)
Heterogeneity: τ² = 0.02, I² = 28.83%, H² = 1.40
Test of θ = 0: z = 5.56, p = 0.00
1/4
1
4
16
Random-effects REML model

Basic adjustment

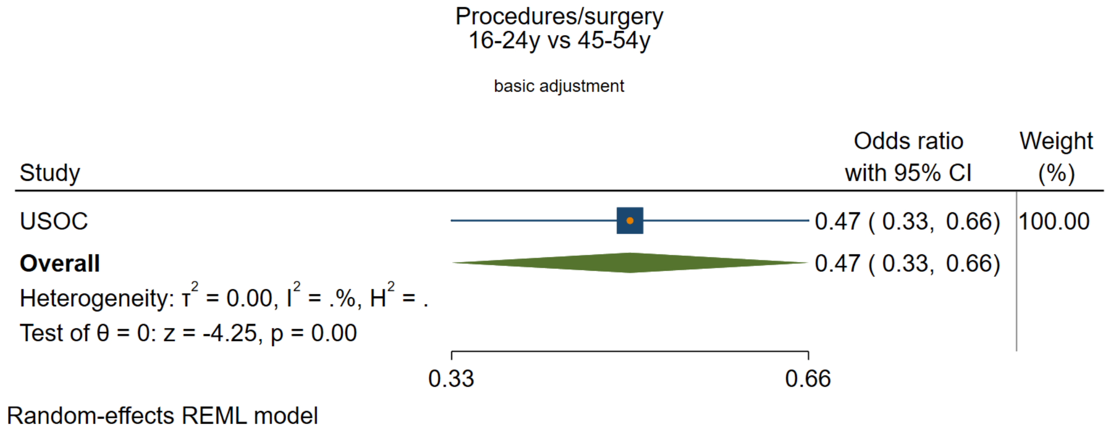

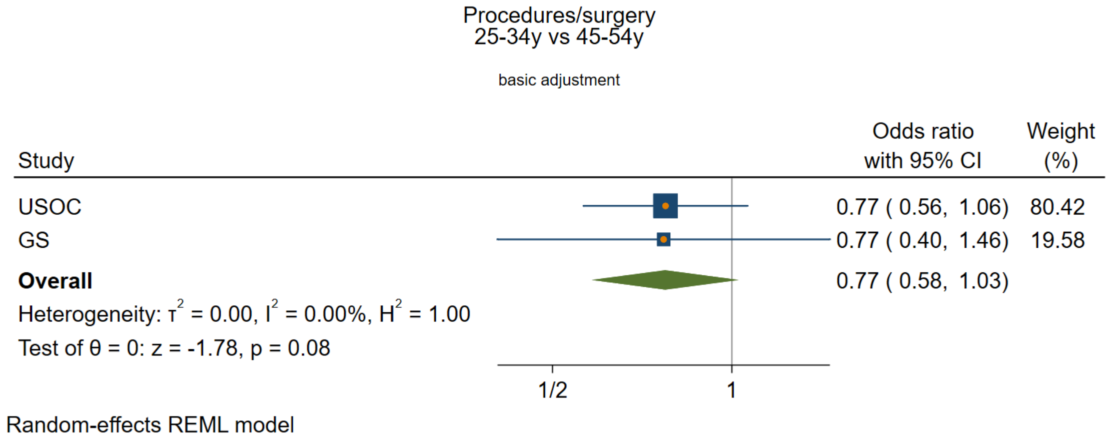

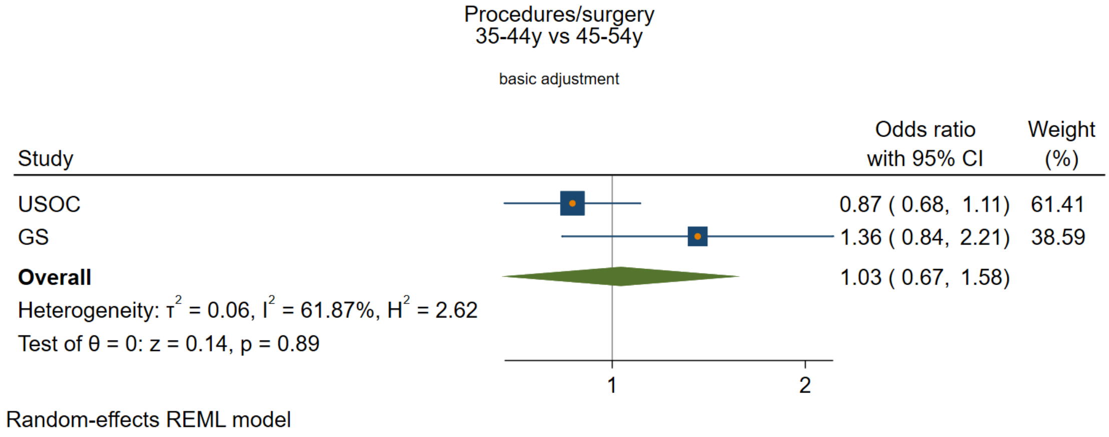

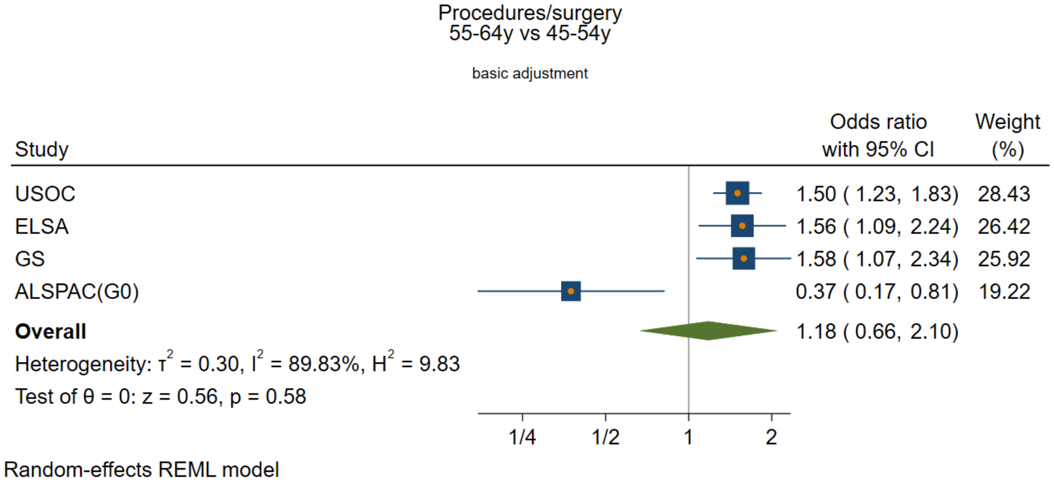

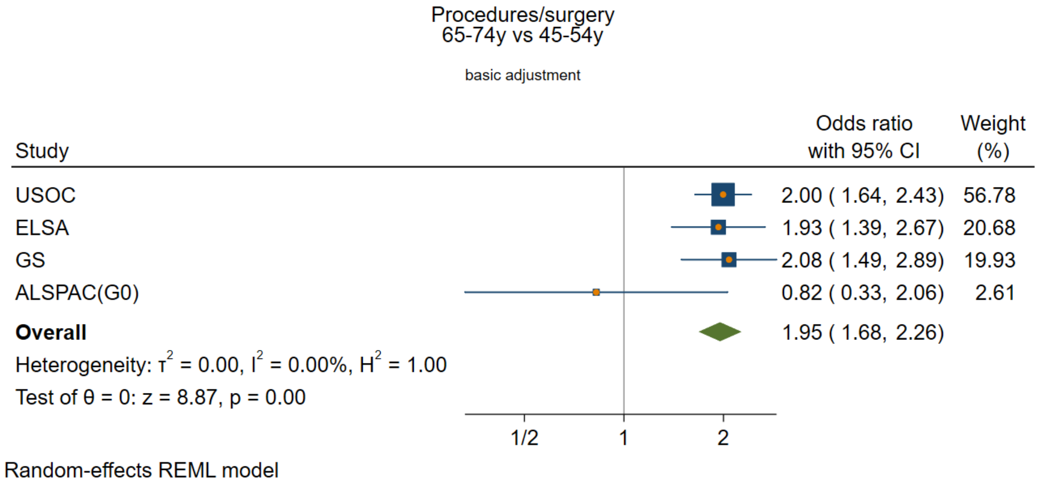

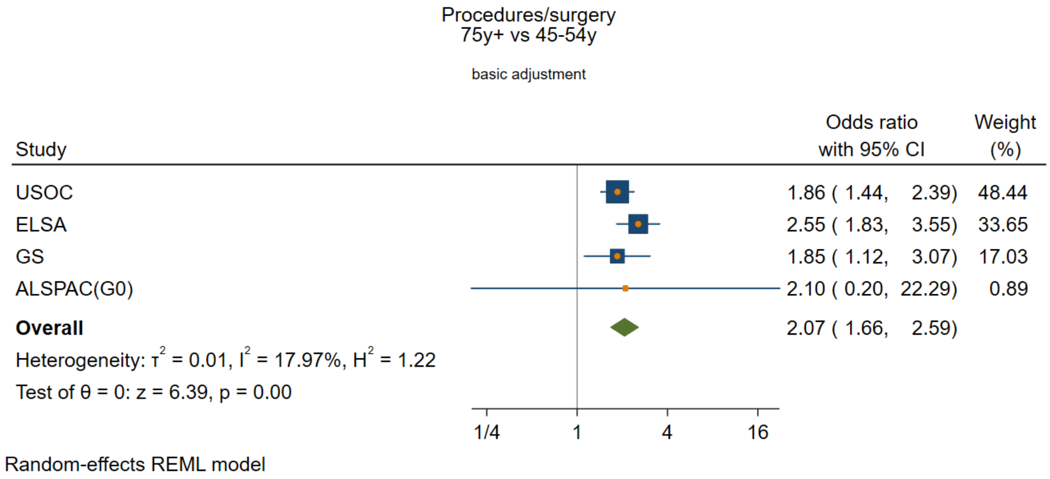

Maddock J, *et al. BMJ Open* 2022; 12:e064981. doi: 10.1136/bmjopen-2022-064981

Full adjustment

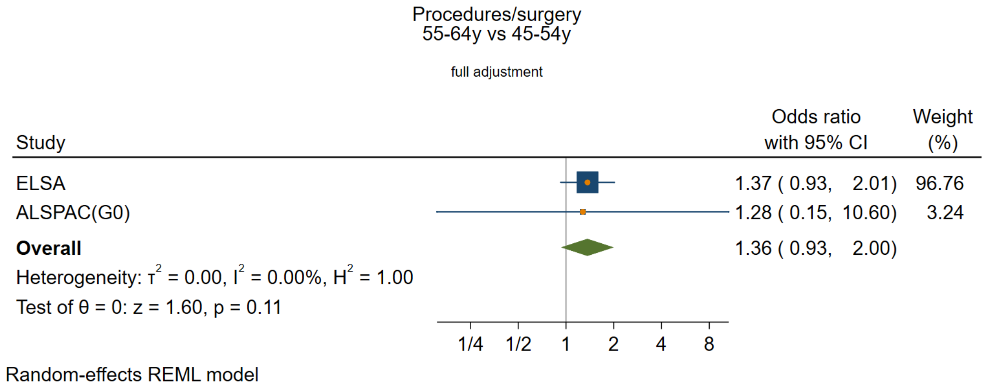

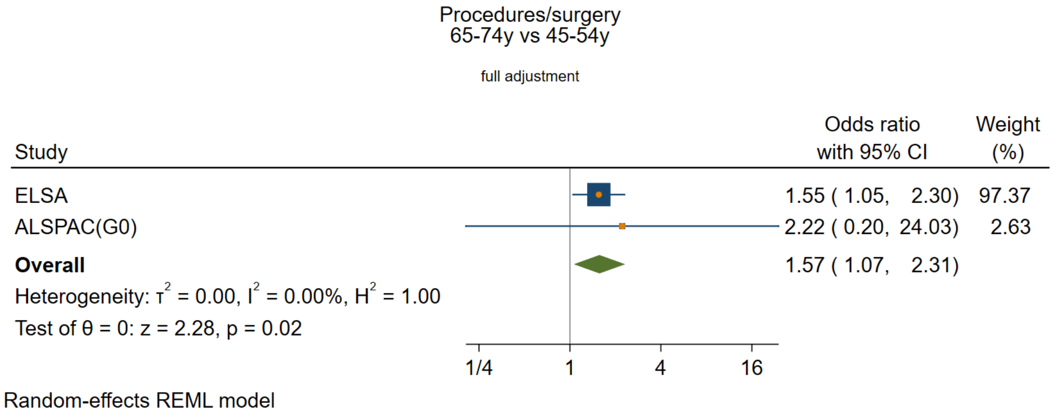

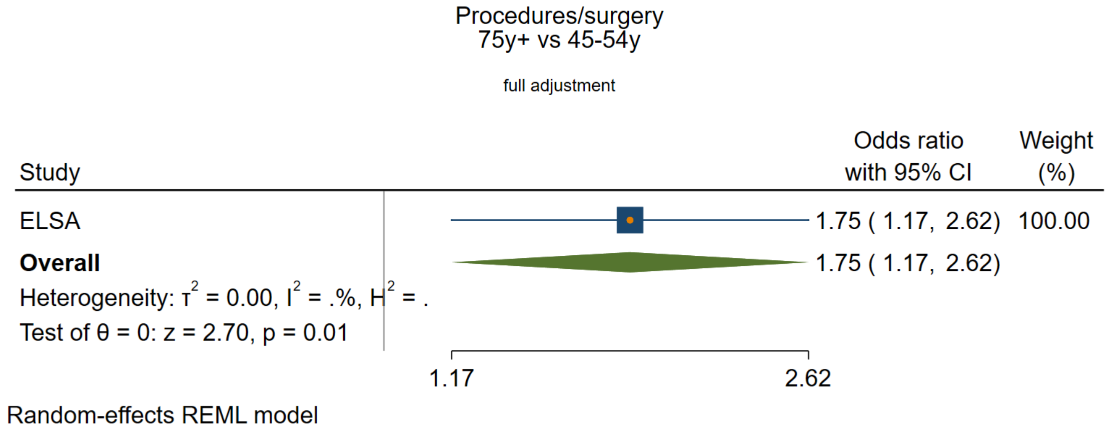

Maddock J, *et al. BMJ Open* 2022; 12:e064981. doi: 10.1136/bmjopen-2022-064981

Ethnicity
<u>Unadjusted</u>

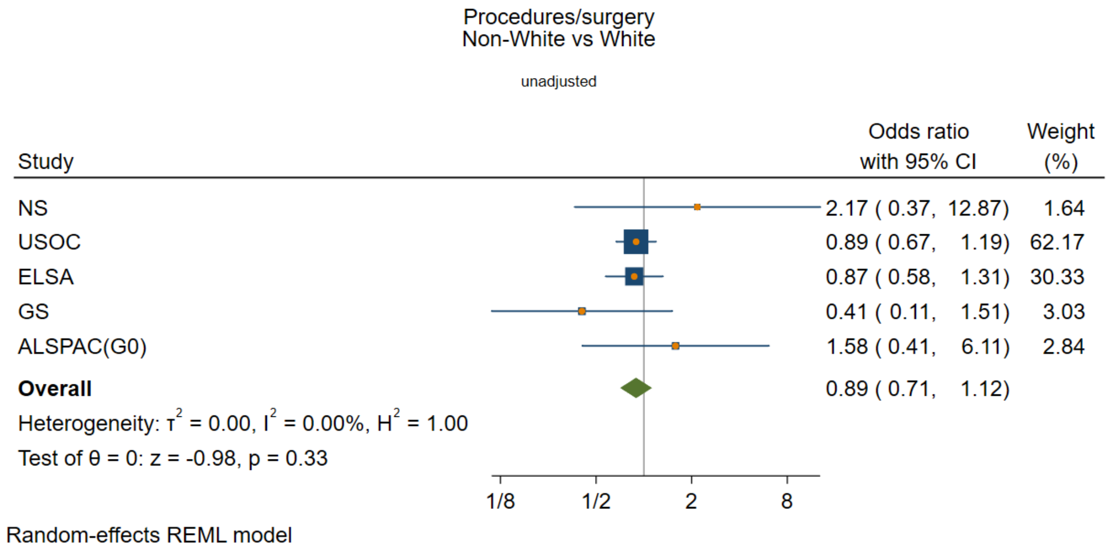

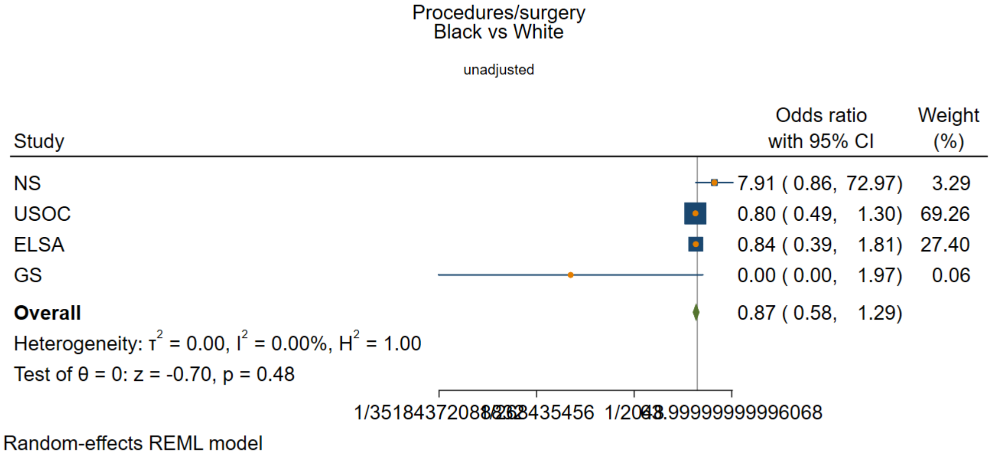
Procedures/surgery
Black vs White
unadjusted
Study
Odds ratio with 95% CI
Weight (%)
NS 7.91 (0.86, 72.97) 3.29
USOC 0.80 (0.49, 1.30) 69.26
ELSA 0.84 (0.39, 1.81) 27.40
GS 0.00 (0.00, 1.97) 0.06
Overall 0.87 (0.58, 1.29)
Heterogeneity: τ² = 0.00, I² = 0.00%, H² = 1.00
Test of θ = 0: z = -0.70, p = 0.48
1/3518437208882688435456 1/2048 3.99999999996068
Random-effects REML model

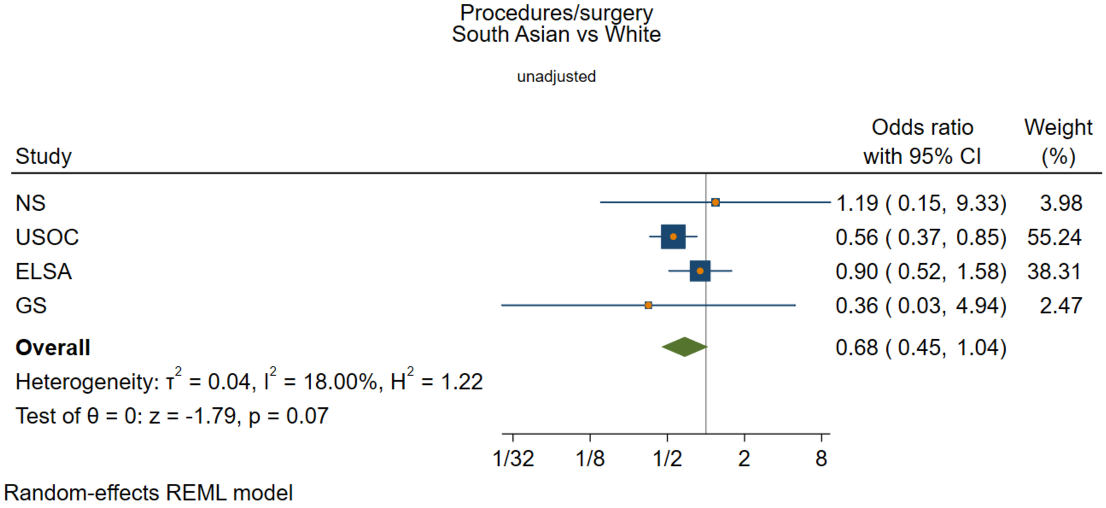
Procedures/surgery
South Asian vs White
unadjusted
Study
Odds ratio with 95% CI
Weight (%)
NS 1.19 (0.15, 9.33) 3.98
USOC 0.56 (0.37, 0.85) 55.24
ELSA 0.90 (0.52, 1.58) 38.31
GS 0.36 (0.03, 4.94) 2.47
Overall 0.68 (0.45, 1.04)
Heterogeneity: τ² = 0.04, I² = 18.00%, H² = 1.22
Test of θ = 0: z = -1.79, p = 0.07
1/32 1/8 1/2 2 8
Random-effects REML model

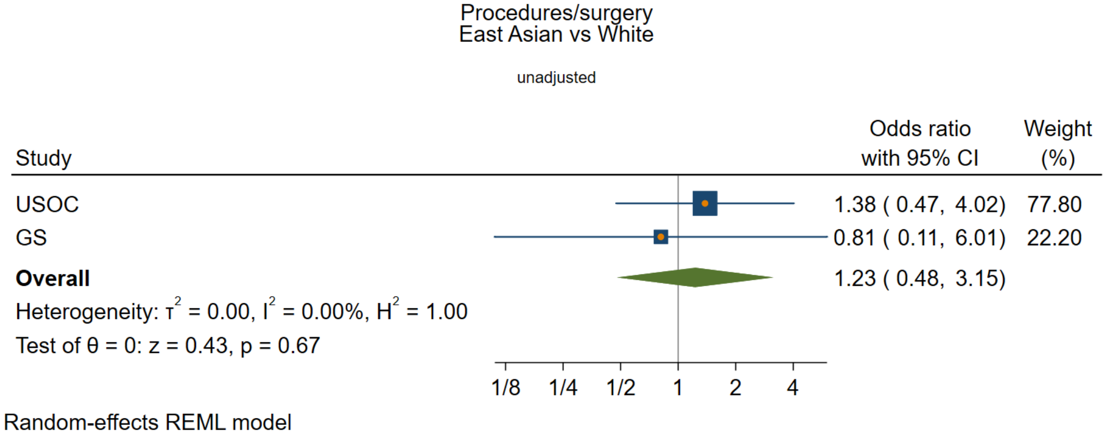
Procedures/surgery
East Asian vs White
unadjusted
Study
Odds ratio with 95% CI
Weight (%)
USOC 1.38 (0.47, 4.02) 77.80
GS 0.81 (0.11, 6.01) 22.20
Overall 1.23 (0.48, 3.15)
Heterogeneity: τ² = 0.00, I² = 0.00%, H² = 1.00
Test of θ = 0: z = 0.43, p = 0.67
1/8 1/4 1/2 1 2 4
Random-effects REML model

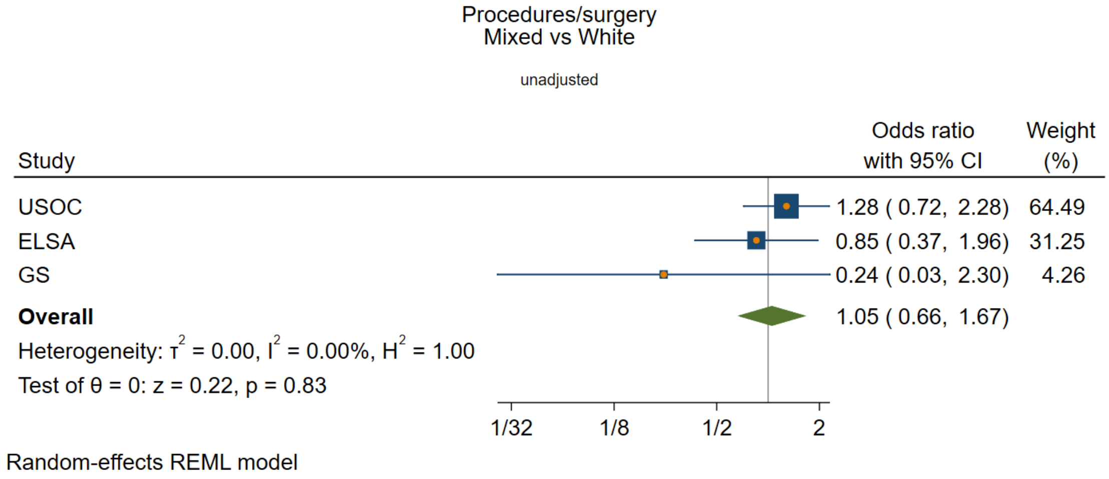
Procedures/surgery
Mixed vs White
unadjusted
Study
Odds ratio with 95% CI
Weight (%)
USOC 1.28 (0.72, 2.28) 64.49
ELSA 0.85 (0.37, 1.96) 31.25
GS 0.24 (0.03, 2.30) 4.26
Overall 1.05 (0.66, 1.67)
Heterogeneity: τ2 = 0.00, I2 = 0.00%, H2 = 1.00
Test of θ = 0: z = 0.22, p = 0.83
1/32
1/8
1/2
2
Random-effects REML model

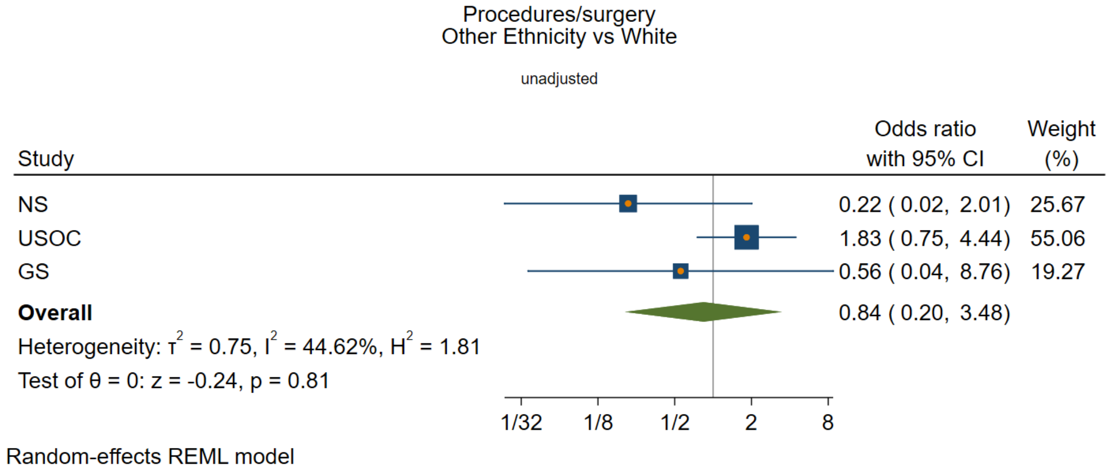
Procedures/surgery
Other Ethnicity vs White
unadjusted
Study
Odds ratio with 95% CI
Weight (%)
NS 0.22 (0.02, 2.01) 25.67
USOC 1.83 (0.75, 4.44) 55.06
GS 0.56 (0.04, 8.76) 19.27
Overall 0.84 (0.20, 3.48)
Heterogeneity: τ2 = 0.75, I2 = 44.62%, H2 = 1.81
Test of θ = 0: z = -0.24, p = 0.81
1/32
1/8
1/2
2
8
Random-effects REML model

Basic adjustment

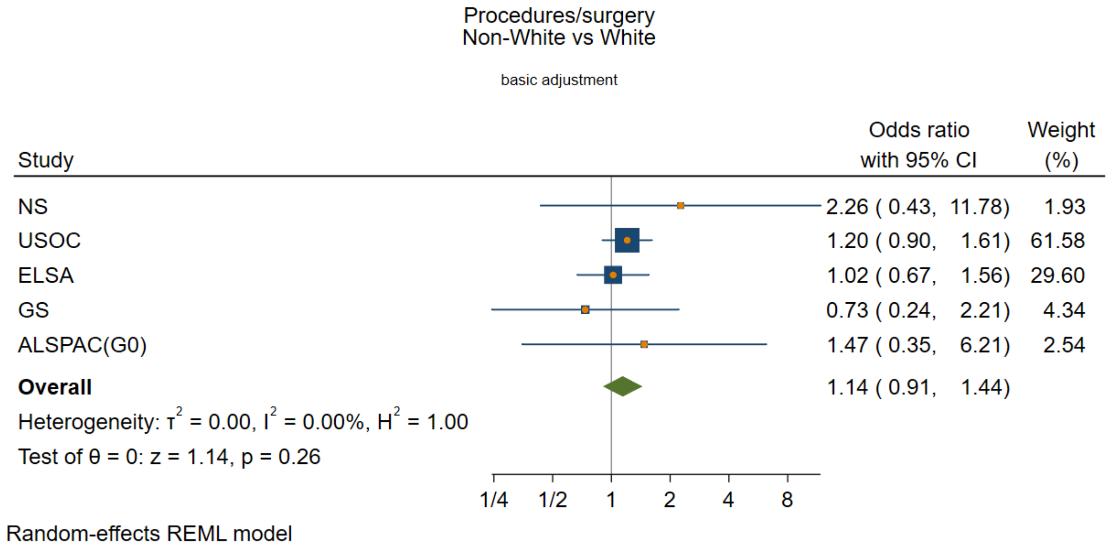

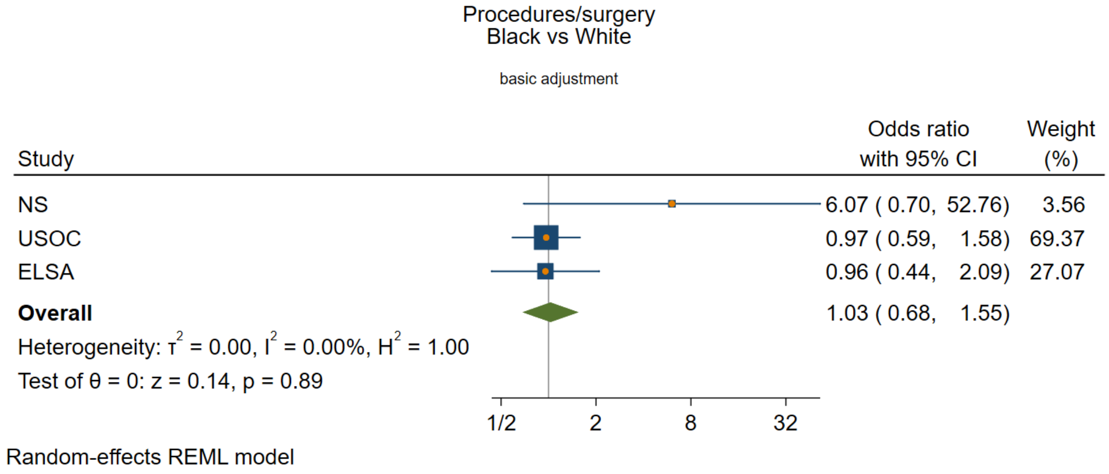

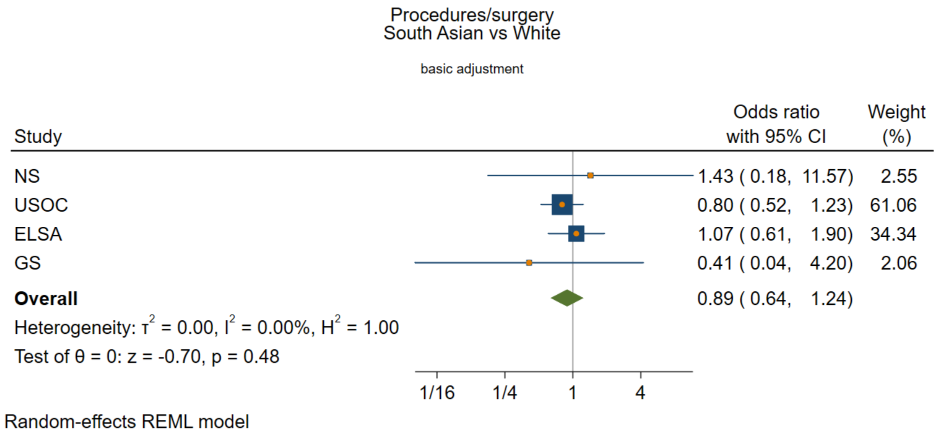

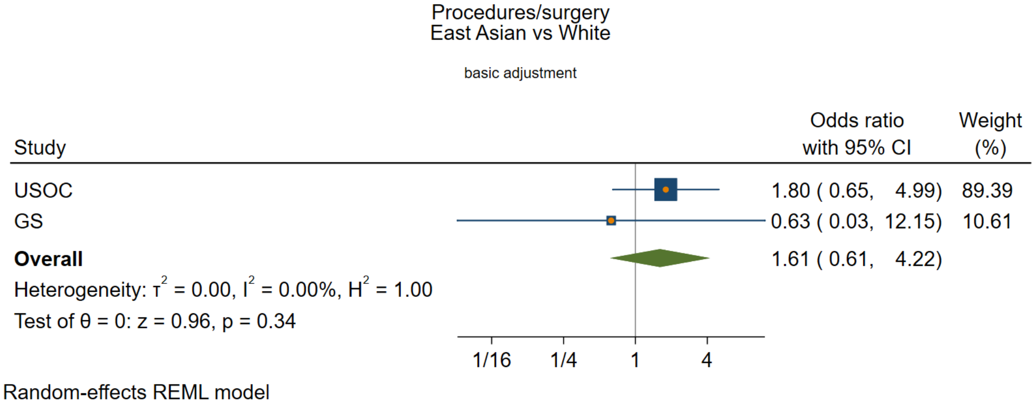

Maddock J, *et al. BMJ Open* 2022; 12:e064981. doi: 10.1136/bmjopen-2022-064981

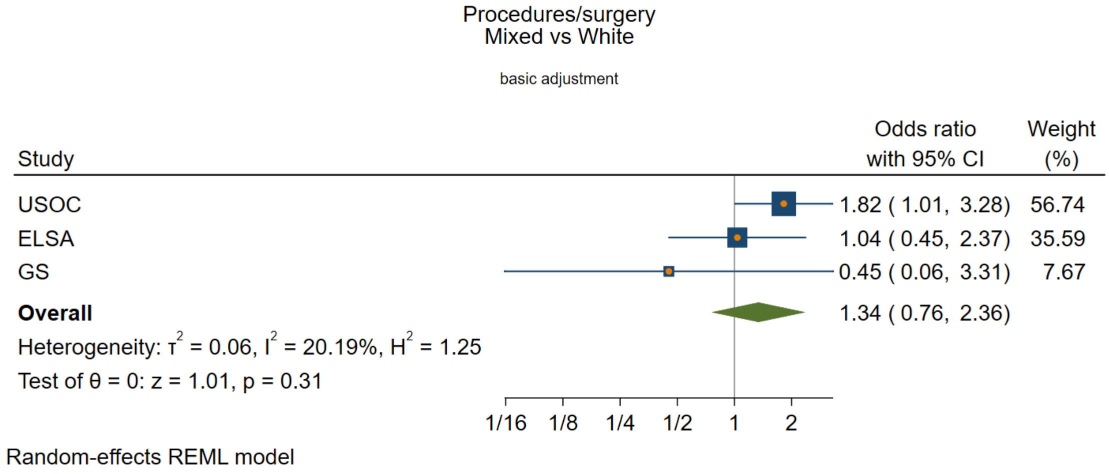

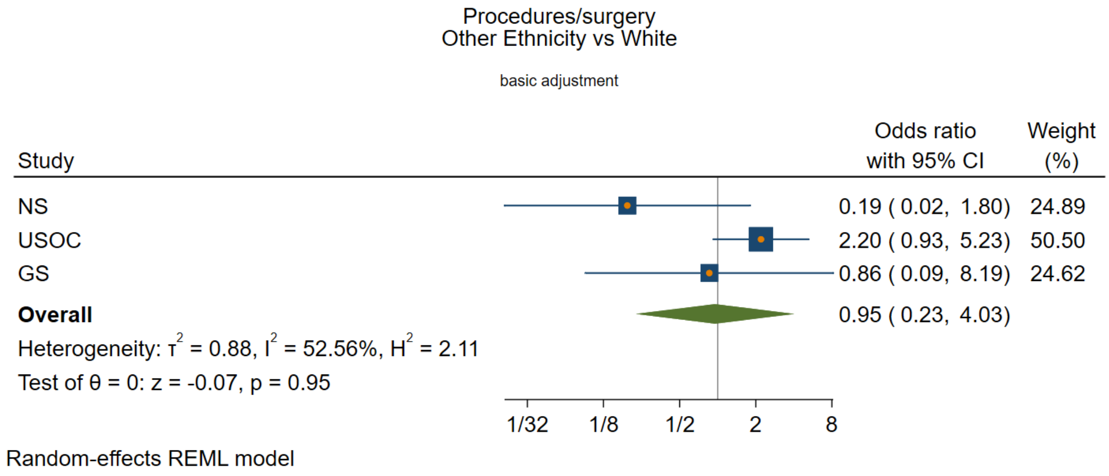

Maddock J, *et al. BMJ Open* 2022; 12:e064981. doi: 10.1136/bmjopen-2022-064981

Full adjustment

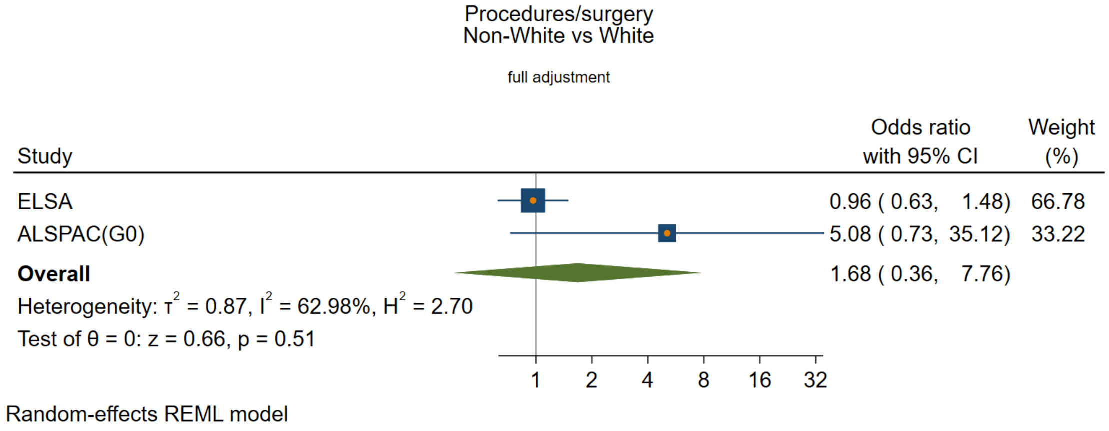

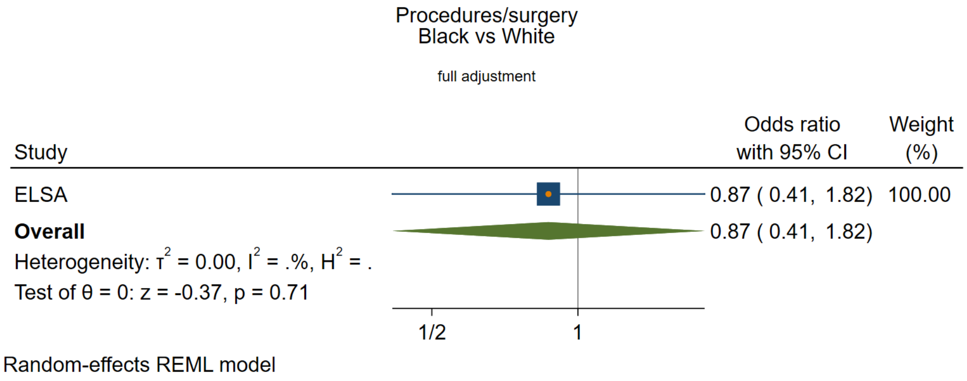

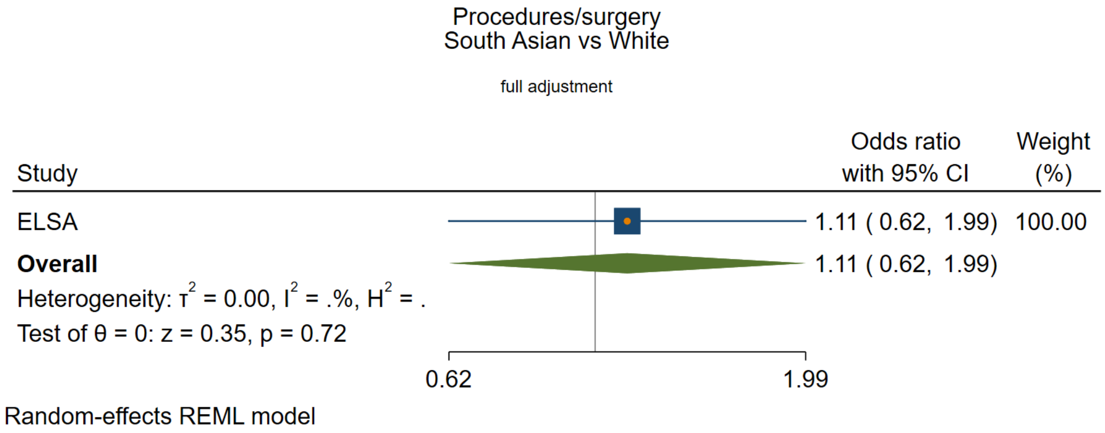

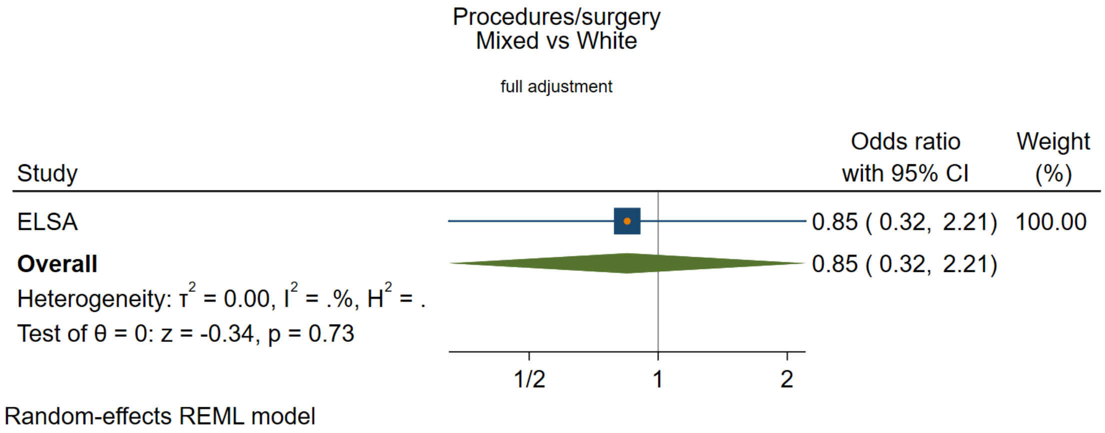

Maddock J, *et al. BMJ Open* 2022; 12:e064981. doi: 10.1136/bmjopen-2022-064981

Any healthcare disruption stratified by shielding status

Sex

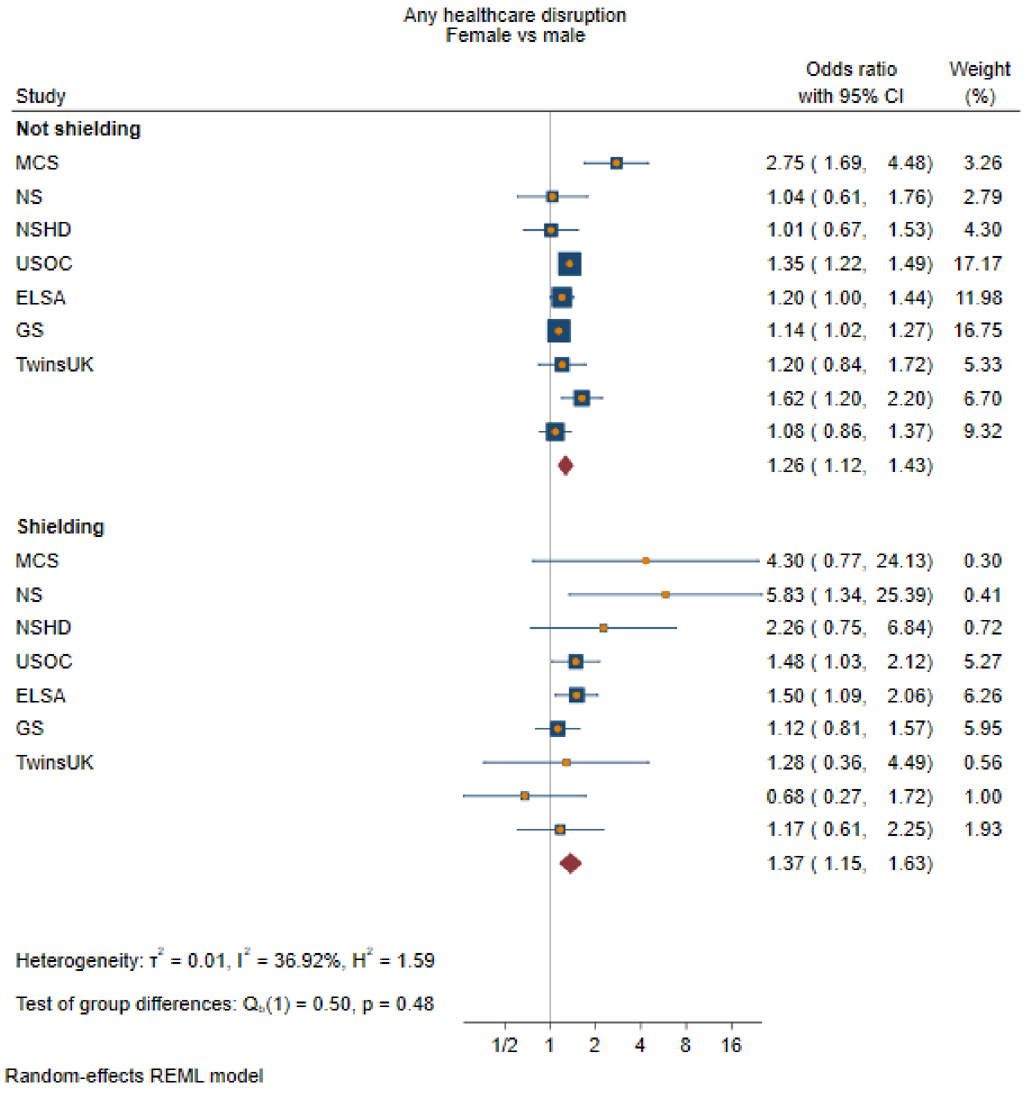

Maddock J, *et al. BMJ Open* 2022; 12:e064981. doi: 10.1136/bmjopen-2022-064981

Occupational class

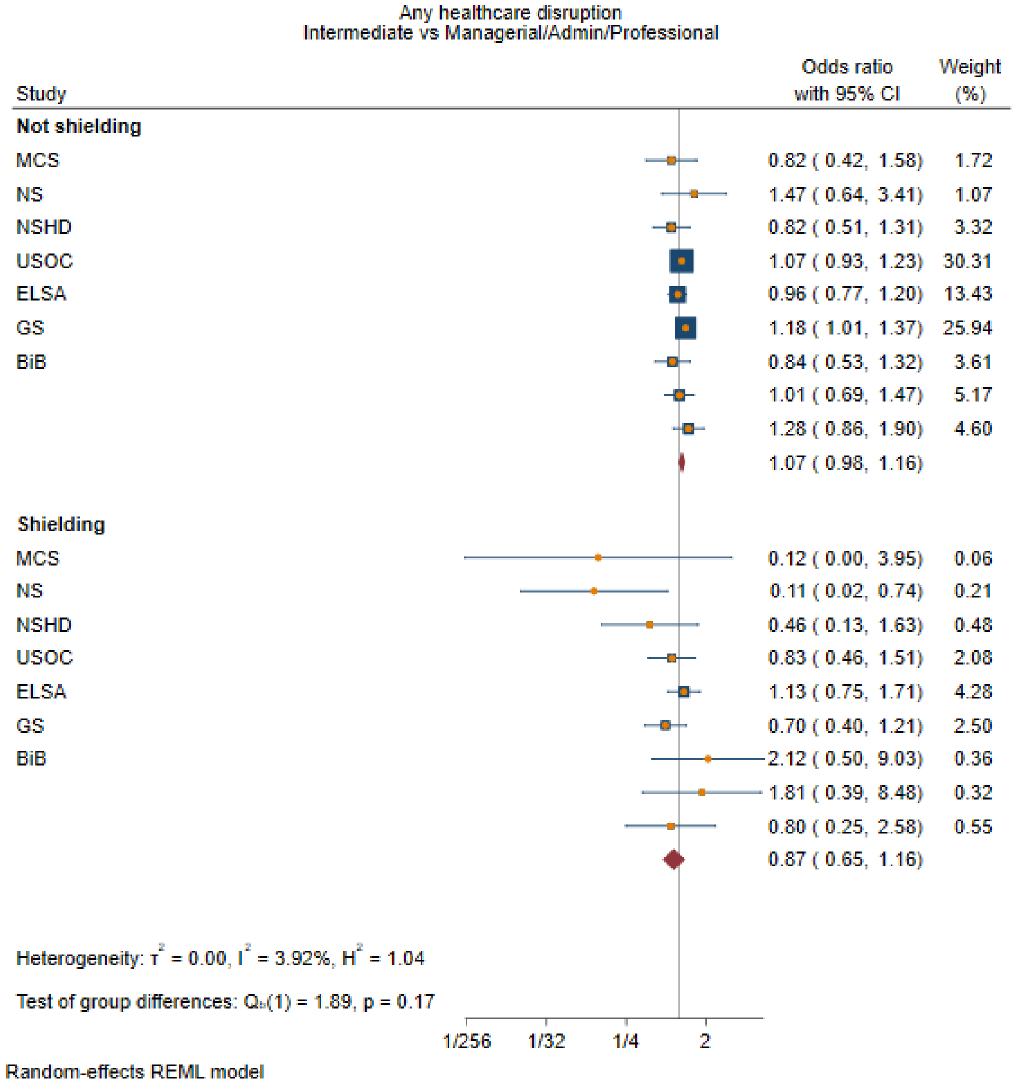
Any healthcare disruption
Intermediate vs Managerial/Admin/Professional
Study
Odds ratio with 95% CI
Weight (%)
Not shielding
MCS 0.82 (0.42, 1.58) 1.72
NS 1.47 (0.64, 3.41) 1.07
NSHD 0.82 (0.51, 1.31) 3.32
USOC 1.07 (0.93, 1.23) 30.31
ELSA 0.96 (0.77, 1.20) 13.43
GS 1.18 (1.01, 1.37) 25.94
BiB 0.84 (0.53, 1.32) 3.61
1.01 (0.69, 1.47) 5.17
1.28 (0.86, 1.90) 4.60
1.07 (0.98, 1.16)
Shielding
MCS 0.12 (0.00, 3.95) 0.06
NS 0.11 (0.02, 0.74) 0.21
NSHD 0.46 (0.13, 1.63) 0.48
USOC 0.83 (0.46, 1.51) 2.08
ELSA 1.13 (0.75, 1.71) 4.28
GS 0.70 (0.40, 1.21) 2.50
BiB 2.12 (0.50, 9.03) 0.36
1.81 (0.39, 8.48) 0.32
0.80 (0.25, 2.58) 0.55
0.87 (0.65, 1.16)
Heterogeneity: τ² = 0.00, I² = 3.92%, H² = 1.04
Test of group differences: Qb(1) = 1.89, p = 0.17
1/256
1/32
1/4
2
Random-effects REML model

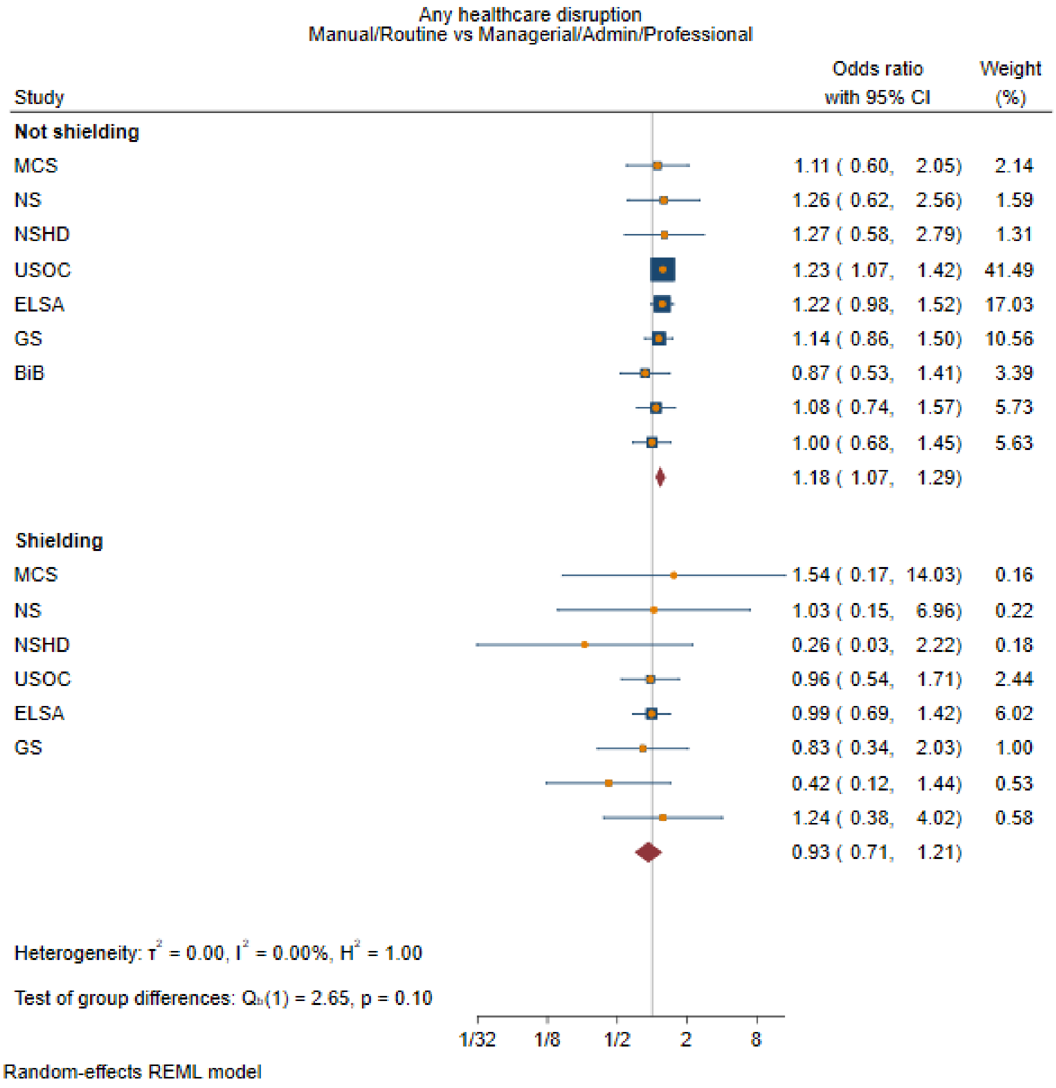
Any healthcare disruption
Manual/Routine vs Managerial/Admin/Professional
Study
Odds ratio with 95% CI
Weight (%)
Not shielding
MCS 1.11 (0.60, 2.05) 2.14
NS 1.26 (0.62, 2.56) 1.59
NSHD 1.27 (0.58, 2.79) 1.31
USOC 1.23 (1.07, 1.42) 41.49
ELSA 1.22 (0.98, 1.52) 17.03
GS 1.14 (0.86, 1.50) 10.56
BiB 0.87 (0.53, 1.41) 3.39
1.08 (0.74, 1.57) 5.73
1.00 (0.68, 1.45) 5.63
1.18 (1.07, 1.29)
Shielding
MCS 1.54 (0.17, 14.03) 0.16
NS 1.03 (0.15, 6.96) 0.22
NSHD 0.26 (0.03, 2.22) 0.18
USOC 0.96 (0.54, 1.71) 2.44
ELSA 0.99 (0.69, 1.42) 6.02
GS 0.83 (0.34, 2.03) 1.00
0.42 (0.12, 1.44) 0.53
1.24 (0.38, 4.02) 0.58
0.93 (0.71, 1.21)
Heterogeneity: τ² = 0.00, I² = 0.00%, H² = 1.00
Test of group differences: Qb(1) = 2.65, p = 0.10
1/32 1/8 1/2 2 8
Random-effects REML model

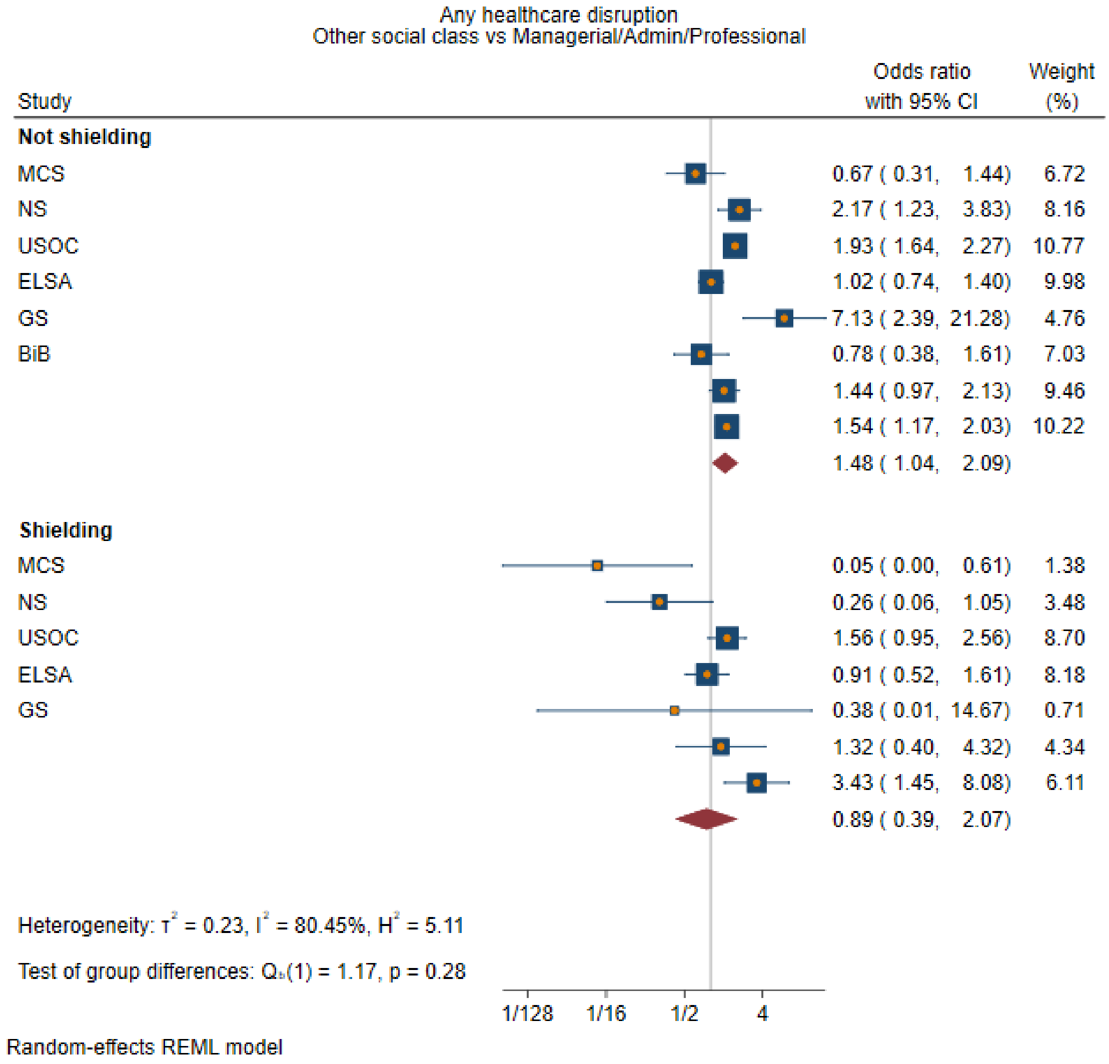
Any healthcare disruption
Other social class vs Managerial/Admin/Professional
Study
Odds ratio with 95% CI
Weight (%)
Not shielding
MCS 0.67 (0.31, 1.44) 6.72
NS 2.17 (1.23, 3.83) 8.16
USOC 1.93 (1.64, 2.27) 10.77
ELSA 1.02 (0.74, 1.40) 9.98
GS 7.13 (2.39, 21.28) 4.76
BiB 0.78 (0.38, 1.61) 7.03
1.44 (0.97, 2.13) 9.46
1.54 (1.17, 2.03) 10.22
1.48 (1.04, 2.09)
Shielding
MCS 0.05 (0.00, 0.61) 1.38
NS 0.26 (0.06, 1.05) 3.48
USOC 1.56 (0.95, 2.56) 8.70
ELSA 0.91 (0.52, 1.61) 8.18
GS 0.38 (0.01, 14.67) 0.71
1.32 (0.40, 4.32) 4.34
3.43 (1.45, 8.08) 6.11
0.89 (0.39, 2.07)
Heterogeneity: τ² = 0.23, I² = 80.45%, H² = 5.11
Test of group differences: Qb(1) = 1.17, p = 0.28
1/128
1/16
1/2
4
Random-effects REML model

Education

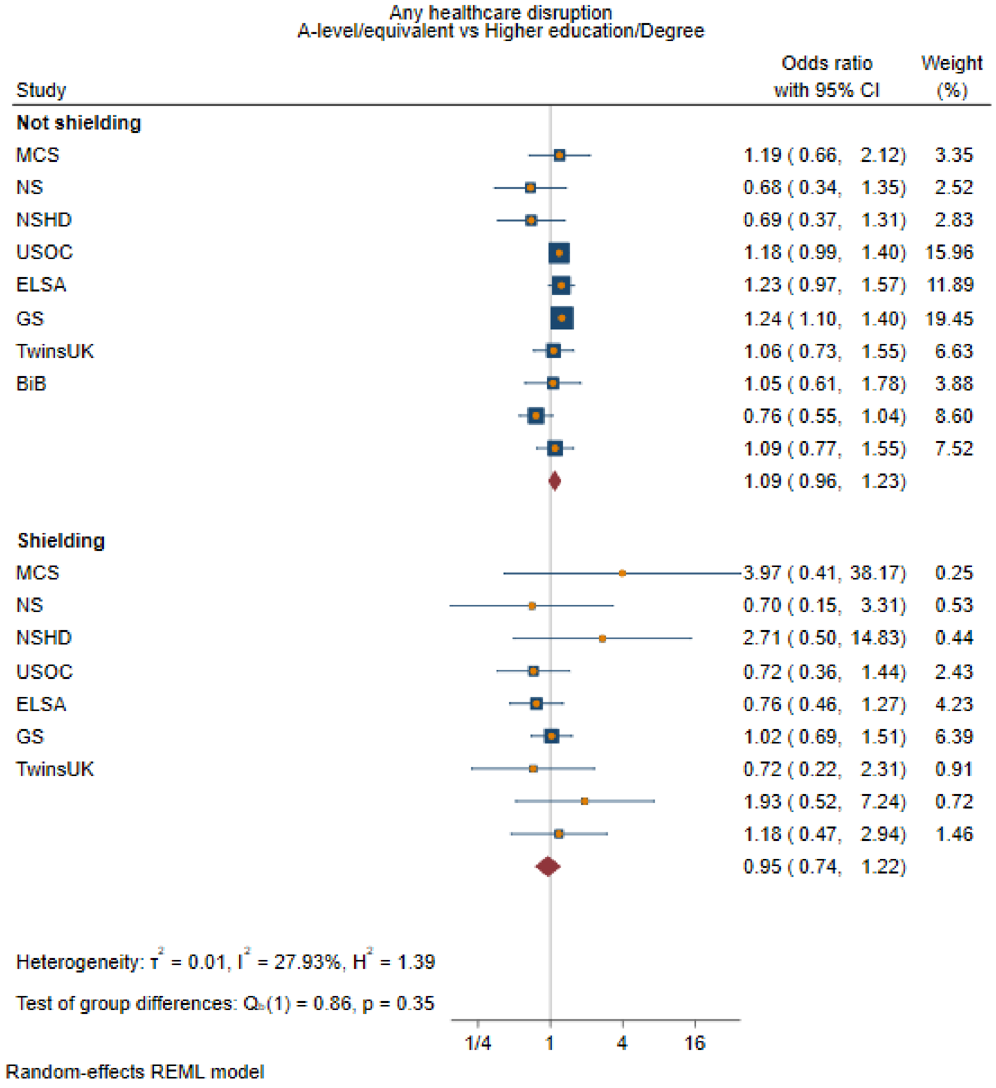

Maddock J, *et al. BMJ Open* 2022; 12:e064981. doi: 10.1136/bmjopen-2022-064981

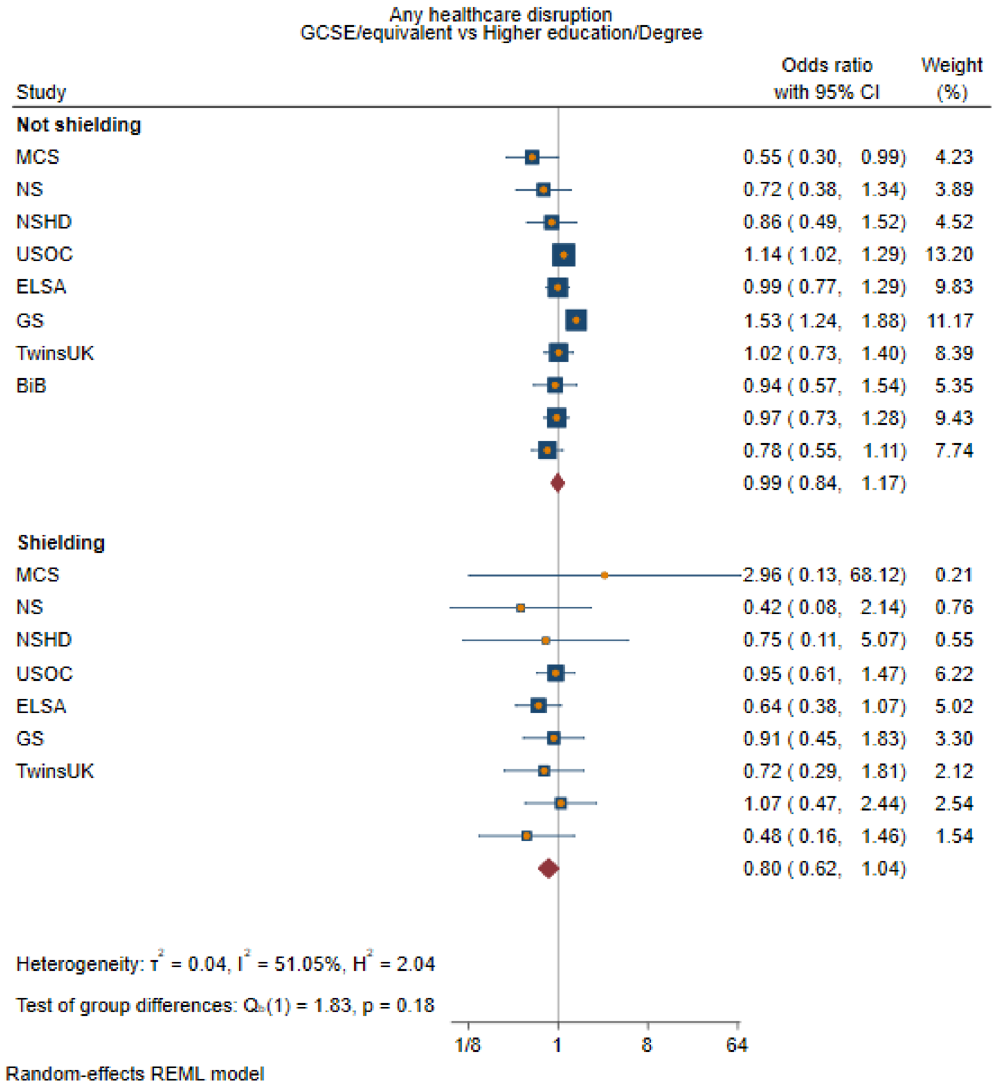
Any healthcare disruption
GCSE/equivalent vs Higher education/Degree
Study
Odds ratio with 95% CI
Weight (%)
Not shielding
MCS 0.55 (0.30, 0.99) 4.23
NS 0.72 (0.38, 1.34) 3.89
NSHD 0.86 (0.49, 1.52) 4.52
USOC 1.14 (1.02, 1.29) 13.20
ELSA 0.99 (0.77, 1.29) 9.83
GS 1.53 (1.24, 1.88) 11.17
TwinsUK 1.02 (0.73, 1.40) 8.39
BiB 0.94 (0.57, 1.54) 5.35
0.97 (0.73, 1.28) 9.43
0.78 (0.55, 1.11) 7.74
0.99 (0.84, 1.17)
Shielding
MCS 2.96 (0.13, 68.12) 0.21
NS 0.42 (0.08, 2.14) 0.76
NSHD 0.75 (0.11, 5.07) 0.55
USOC 0.95 (0.61, 1.47) 6.22
ELSA 0.64 (0.38, 1.07) 5.02
GS 0.91 (0.45, 1.83) 3.30
TwinsUK 0.72 (0.29, 1.81) 2.12
1.07 (0.47, 2.44) 2.54
0.48 (0.16, 1.46) 1.54
0.80 (0.62, 1.04)
Heterogeneity: τ² = 0.04, I² = 51.05%, H² = 2.04
Test of group differences: Qb(1) = 1.83, p = 0.18
1/8
1
8
64
Random-effects REML model

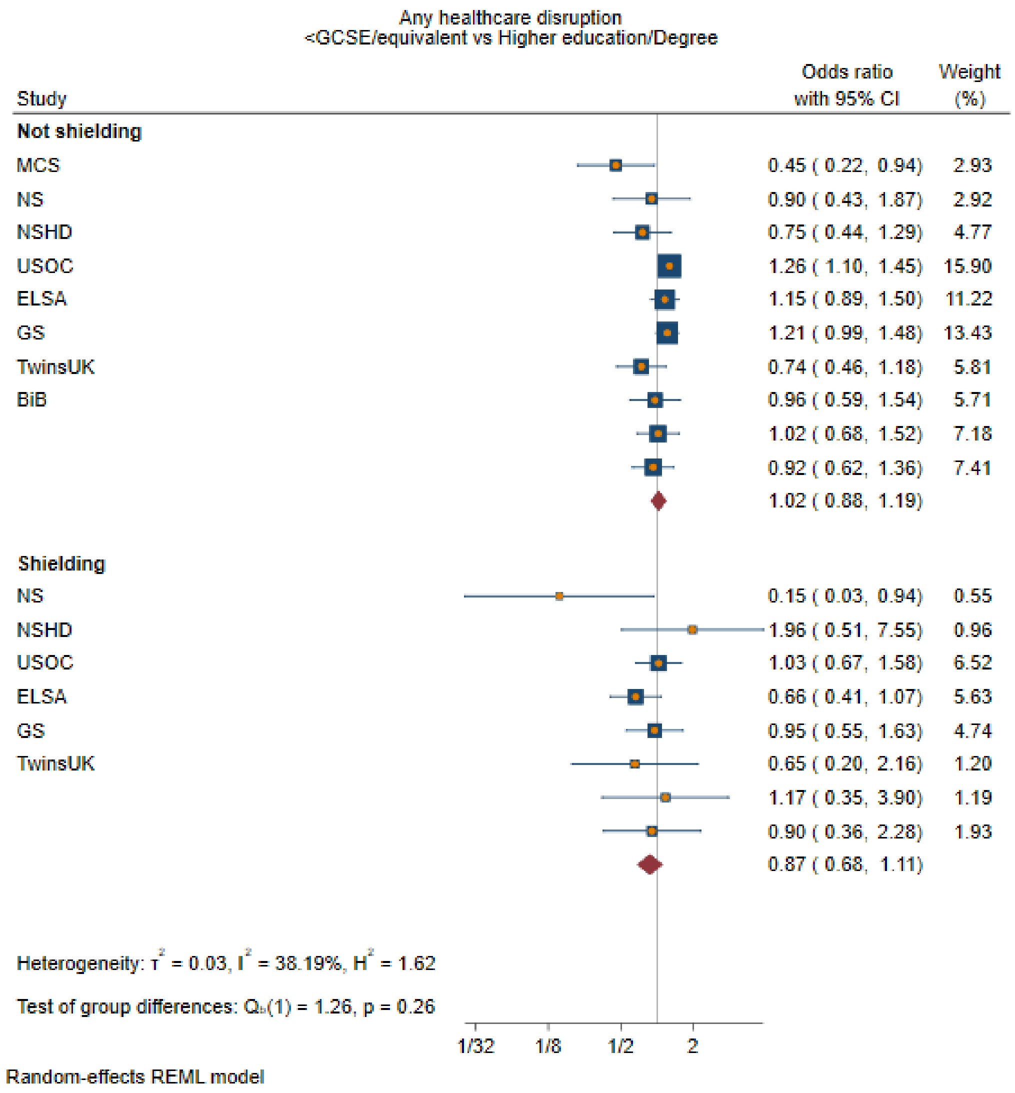
Any healthcare disruption
<GCSE/equivalent vs Higher education/Degree
Study
Odds ratio with 95% CI
Weight (%)
Not shielding
MCS
0.45 (0.22, 0.94) 2.93
NS
0.90 (0.43, 1.87) 2.92
NSHD
0.75 (0.44, 1.29) 4.77
USOC
1.26 (1.10, 1.45) 15.90
ELSA
1.15 (0.89, 1.50) 11.22
GS
1.21 (0.99, 1.48) 13.43
TwinsUK
0.74 (0.46, 1.18) 5.81
BiB
0.96 (0.59, 1.54) 5.71
1.02 (0.68, 1.52) 7.18
0.92 (0.62, 1.36) 7.41
1.02 (0.88, 1.19)
Shielding
NS
0.15 (0.03, 0.94) 0.55
NSHD
1.96 (0.51, 7.55) 0.96
USOC
1.03 (0.67, 1.58) 6.52
ELSA
0.66 (0.41, 1.07) 5.63
GS
0.95 (0.55, 1.63) 4.74
TwinsUK
0.65 (0.20, 2.16) 1.20
1.17 (0.35, 3.90) 1.19
0.90 (0.36, 2.28) 1.93
0.87 (0.68, 1.11)
Heterogeneity: τ² = 0.03, I² = 38.19%, H² = 1.62
Test of group differences: Qb(1) = 1.26, p = 0.26
1/32
1/8
1/2
2
Random-effects REML model

Age

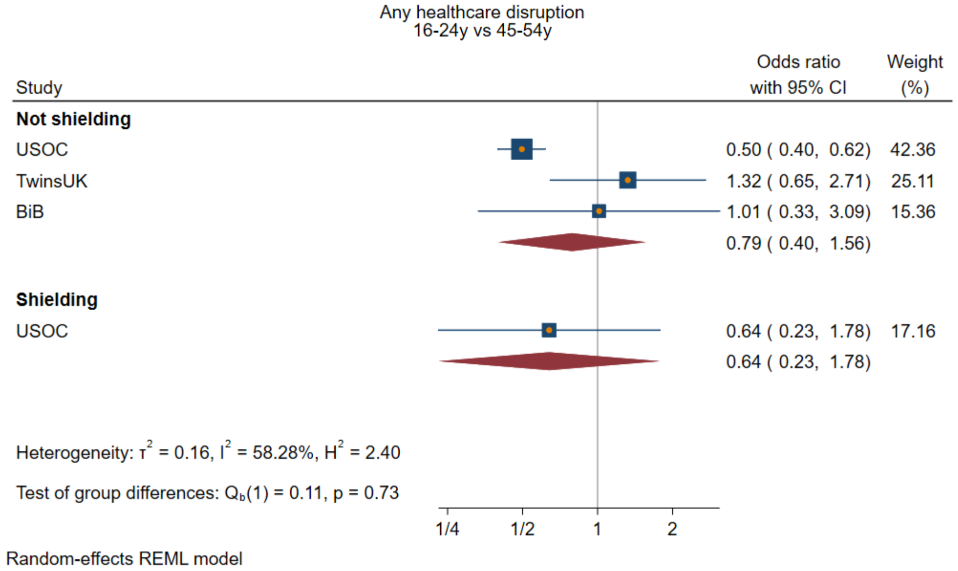

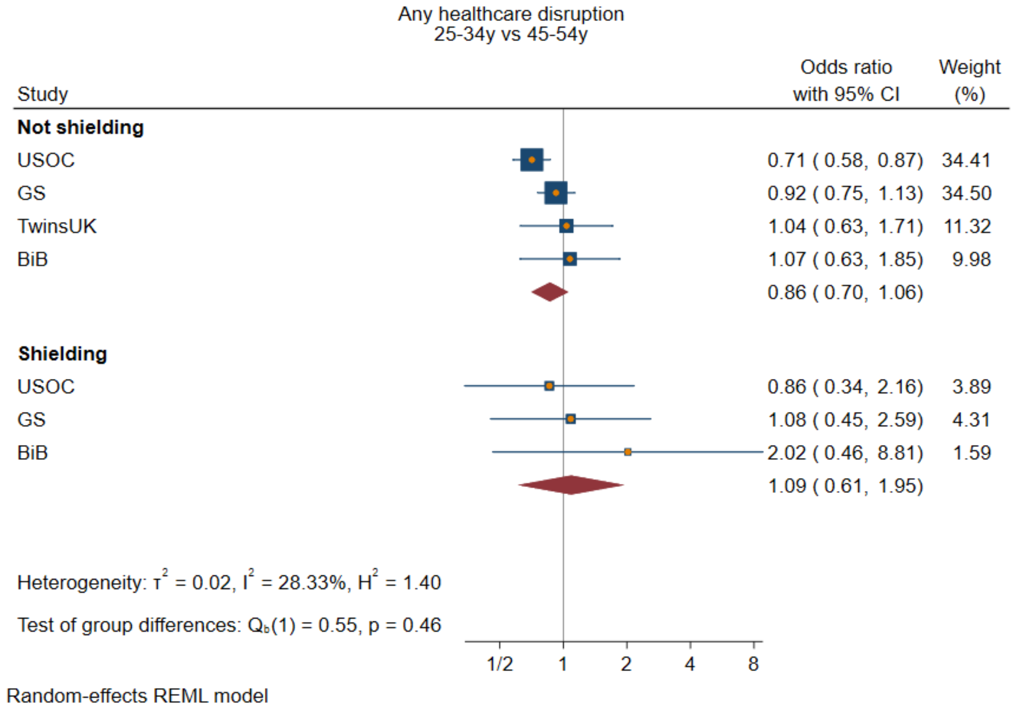

Maddock J, *et al. BMJ Open* 2022; 12:e064981. doi: 10.1136/bmjopen-2022-064981

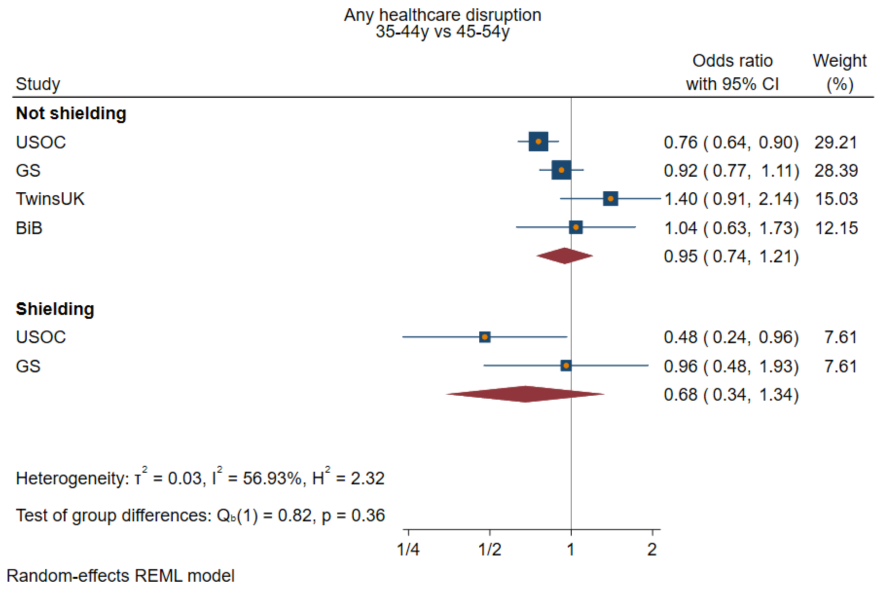
Any healthcare disruption
35-44y vs 45-54y
Study
Odds ratio with 95% CI
Weight (%)
Not shielding
USOC
0.76 (0.64, 0.90) 29.21
GS
0.92 (0.77, 1.11) 28.39
TwinsUK
1.40 (0.91, 2.14) 15.03
BiB
1.04 (0.63, 1.73) 12.15
0.95 (0.74, 1.21)
Shielding
USOC
0.48 (0.24, 0.96) 7.61
GS
0.96 (0.48, 1.93) 7.61
0.68 (0.34, 1.34)
Heterogeneity: τ² = 0.03, I² = 56.93%, H² = 2.32
Test of group differences: Qb(1) = 0.82, p = 0.36
1/4
1/2
1
2
Random-effects REML model

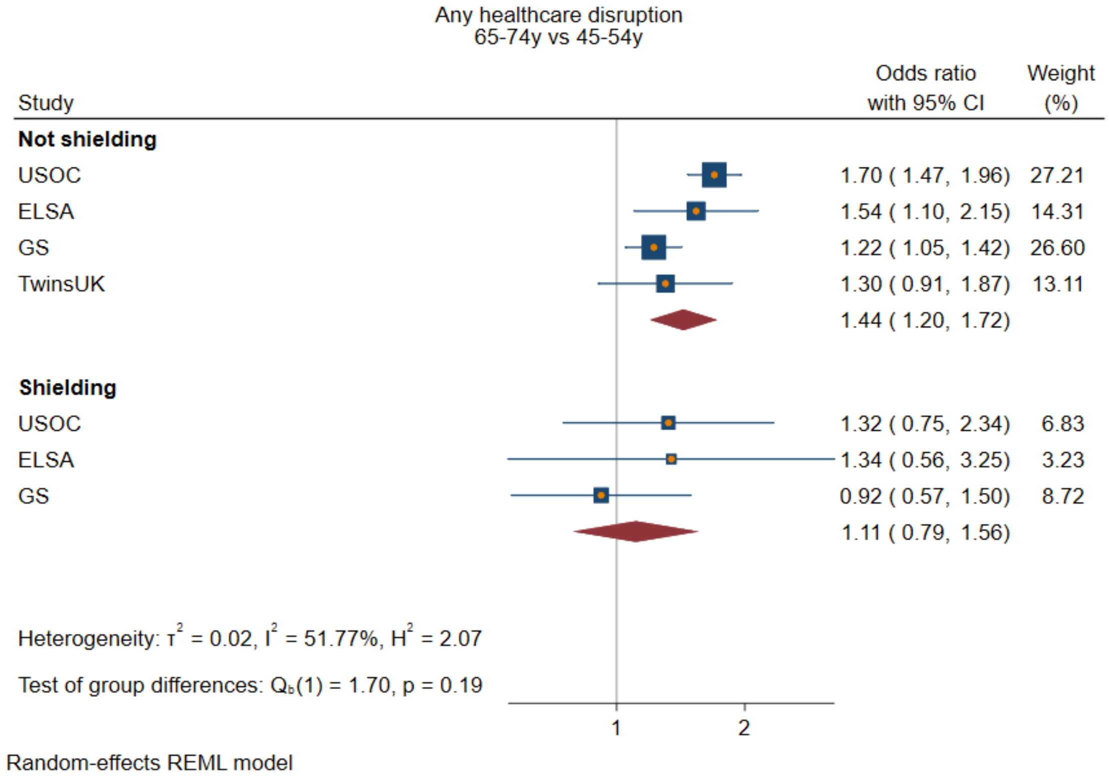
Any healthcare disruption
65-74y vs 45-54y
Study
Odds ratio with 95% CI
Weight (%)
Not shielding
USOC
1.70 (1.47, 1.96) 27.21
ELSA
1.54 (1.10, 2.15) 14.31
GS
1.22 (1.05, 1.42) 26.60
TwinsUK
1.30 (0.91, 1.87) 13.11
1.44 (1.20, 1.72)
Shielding
USOC
1.32 (0.75, 2.34) 6.83
ELSA
1.34 (0.56, 3.25) 3.23
GS
0.92 (0.57, 1.50) 8.72
1.11 (0.79, 1.56)
Heterogeneity: τ² = 0.02, I² = 51.77%, H² = 2.07
Test of group differences: Qb(1) = 1.70, p = 0.19
1
2
Random-effects REML model

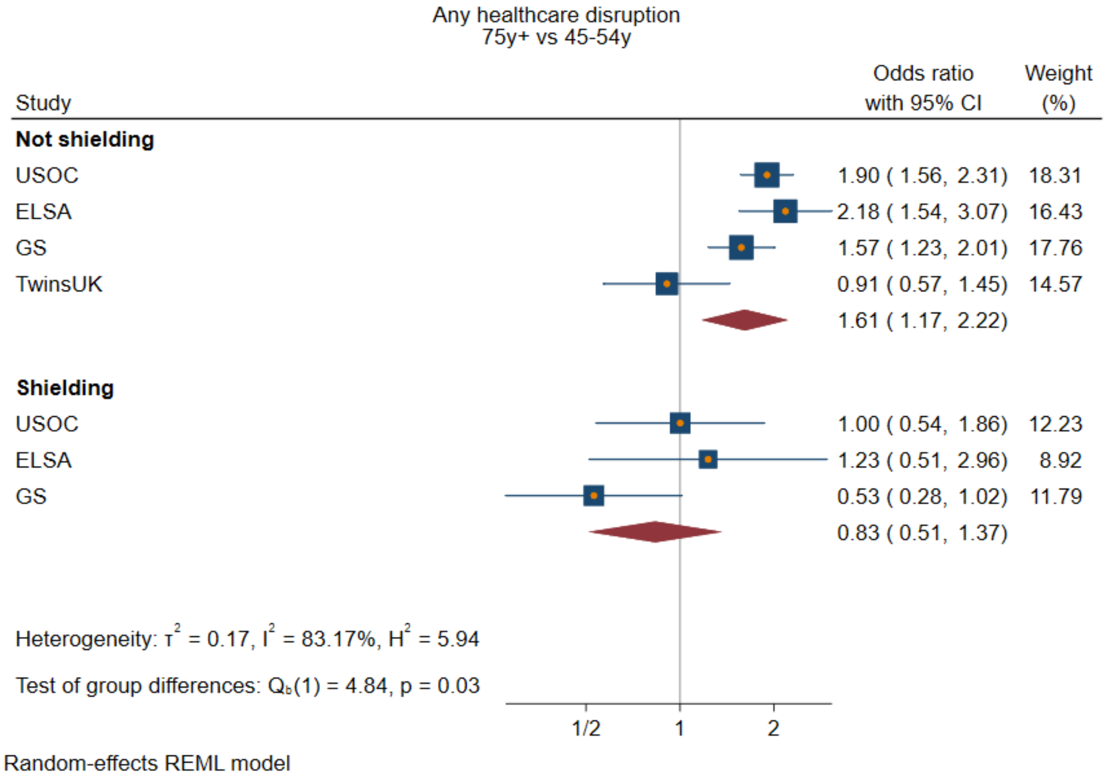
Any healthcare disruption
75y+ vs 45-54y
Study
Odds ratio with 95% CI
Weight (%)
Not shielding
USOC 1.90 (1.56, 2.31) 18.31
ELSA 2.18 (1.54, 3.07) 16.43
GS 1.57 (1.23, 2.01) 17.76
TwinsUK 0.91 (0.57, 1.45) 14.57
1.61 (1.17, 2.22)
Shielding
USOC 1.00 (0.54, 1.86) 12.23
ELSA 1.23 (0.51, 2.96) 8.92
GS 0.53 (0.28, 1.02) 11.79
0.83 (0.51, 1.37)
Heterogeneity: τ² = 0.17, I² = 83.17%, H² = 5.94
Test of group differences: Qb(1) = 4.84, p = 0.03
1/2
1
2
Random-effects REML model

Ethnicity

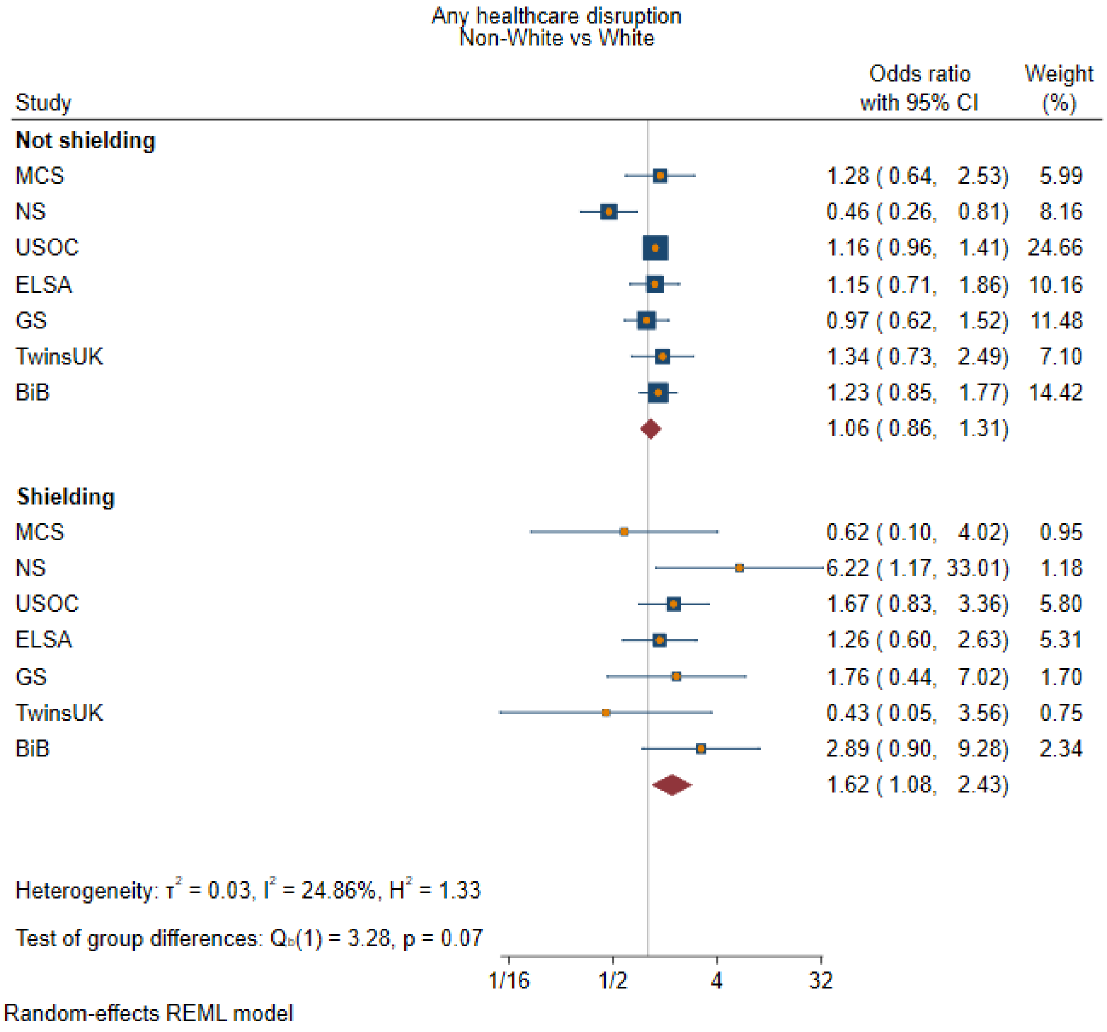

Maddock J, et al. BMJ Open 2022; 12:e064981. doi: 10.1136/bmjopen-2022-064981

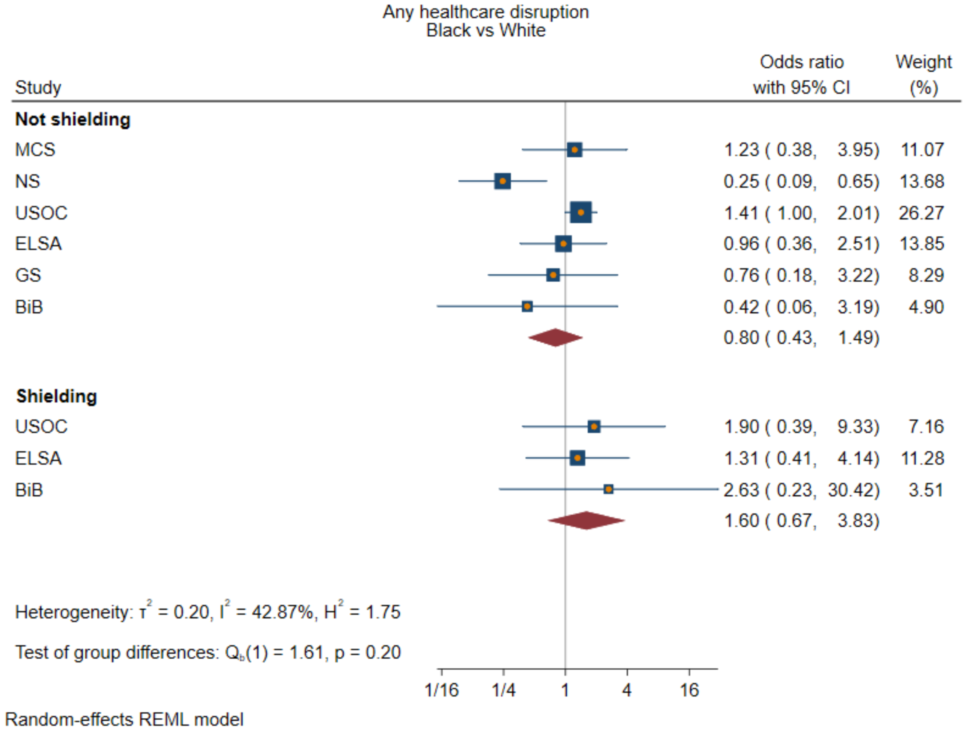

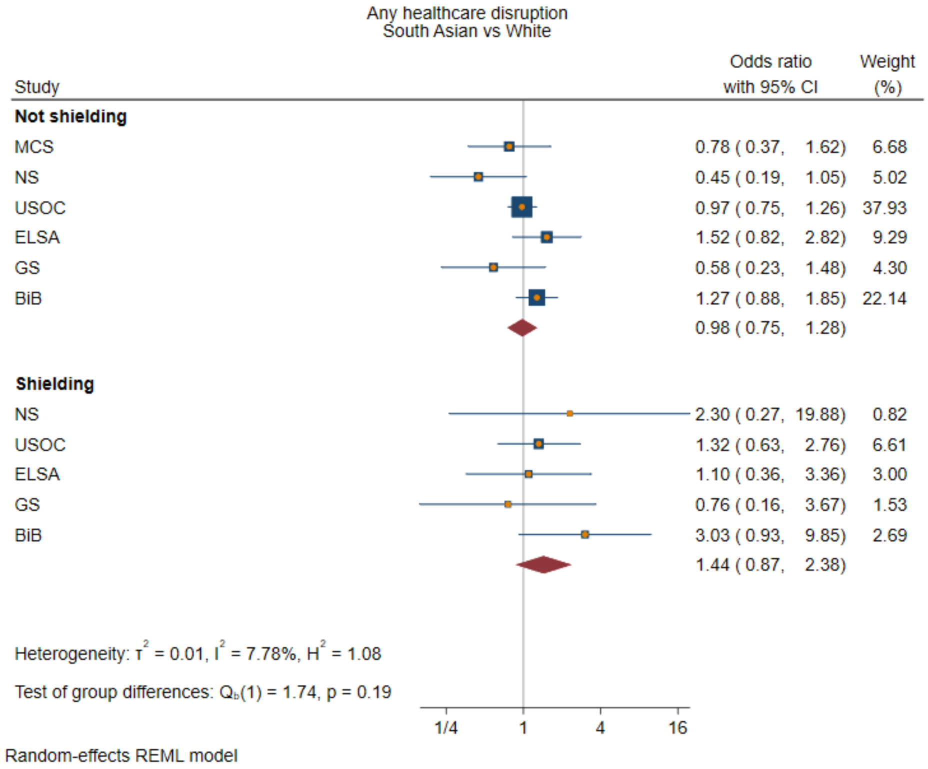

Maddock J, *et al. BMJ Open* 2022; 12:e064981. doi: 10.1136/bmjopen-2022-064981

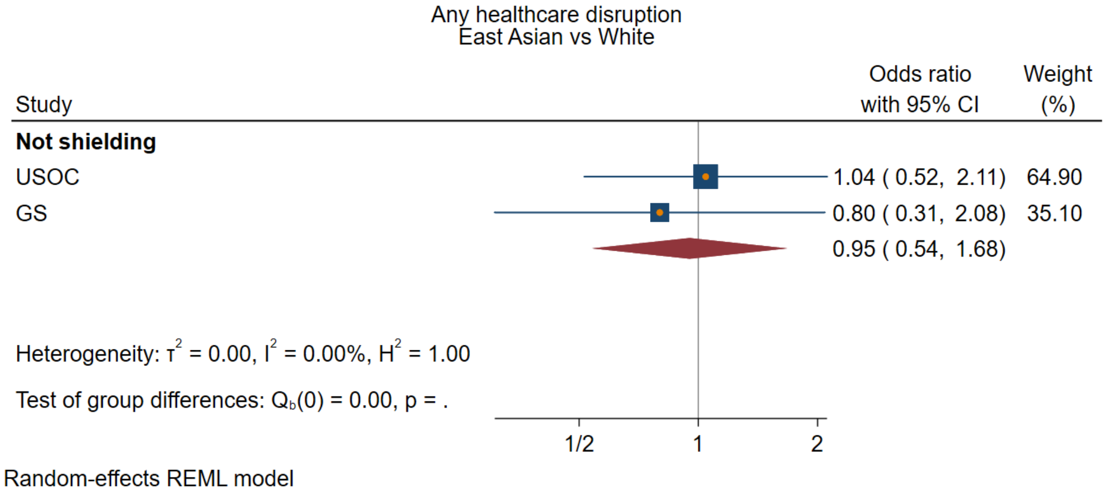

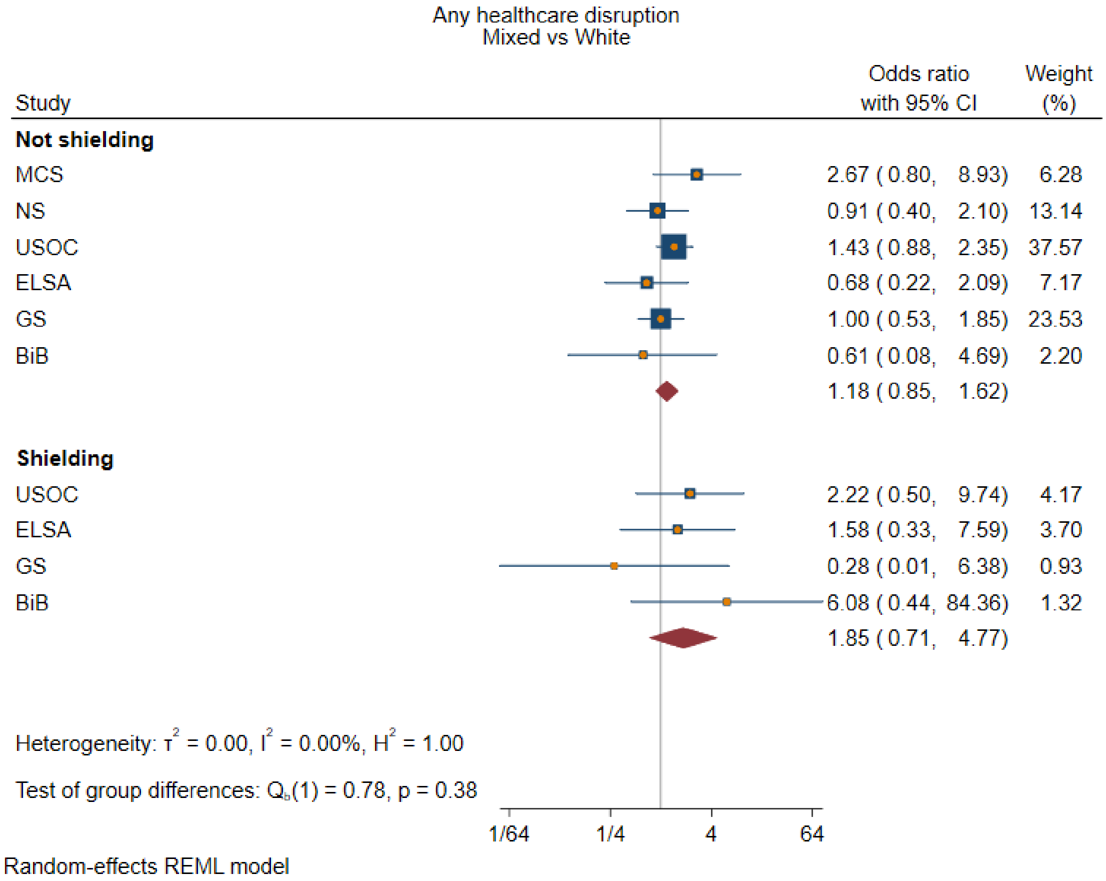

Maddock J, *et al. BMJ Open* 2022; 12:e064981. doi: 10.1136/bmjopen-2022-064981

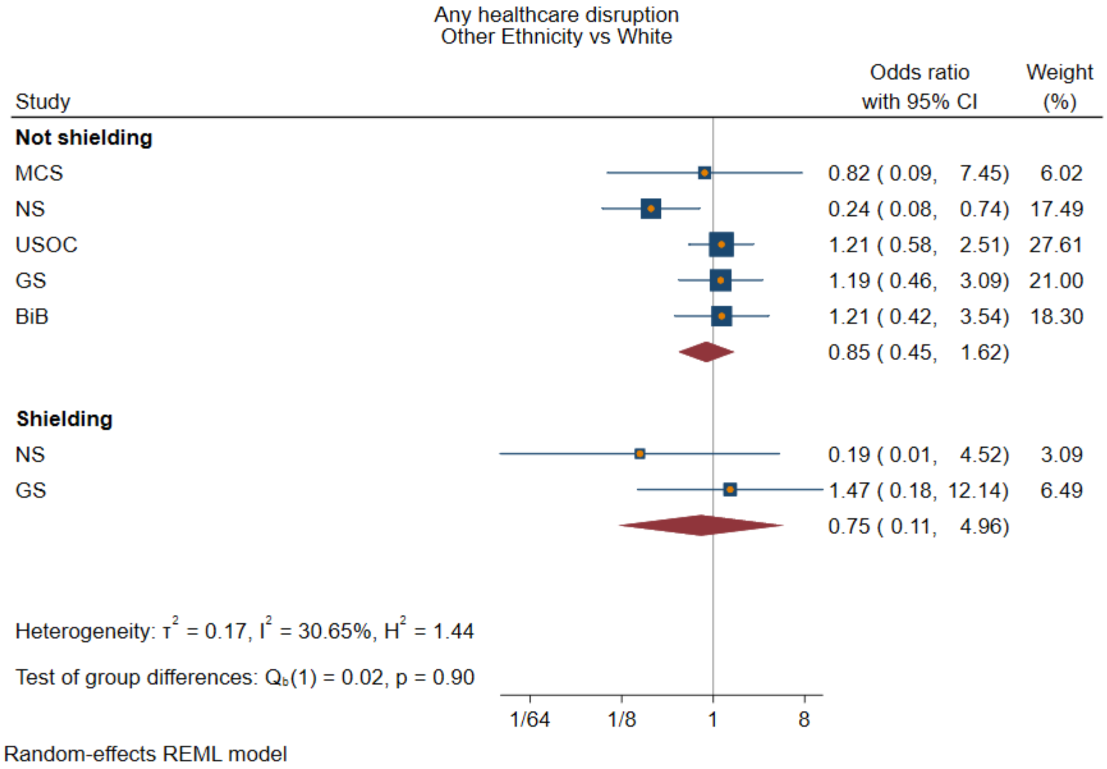
Any healthcare disruption
Other Ethnicity vs White
Study
Odds ratio with 95% CI
Weight (%)
Not shielding
MCS
0.82 (0.09, 7.45) 6.02
NS
0.24 (0.08, 0.74) 17.49
USOC
1.21 (0.58, 2.51) 27.61
GS
1.19 (0.46, 3.09) 21.00
BiB
1.21 (0.42, 3.54) 18.30
0.85 (0.45, 1.62)
Shielding
NS
0.19 (0.01, 4.52) 3.09
GS
1.47 (0.18, 12.14) 6.49
0.75 (0.11, 4.96)
Heterogeneity: τ² = 0.17, I² = 30.65%, H² = 1.44
Test of group differences: Q_b(1) = 0.02, p = 0.90
1/64
1/8
1
8
Random-effects REML model

Any healthcare disruption stratified by age

Sex

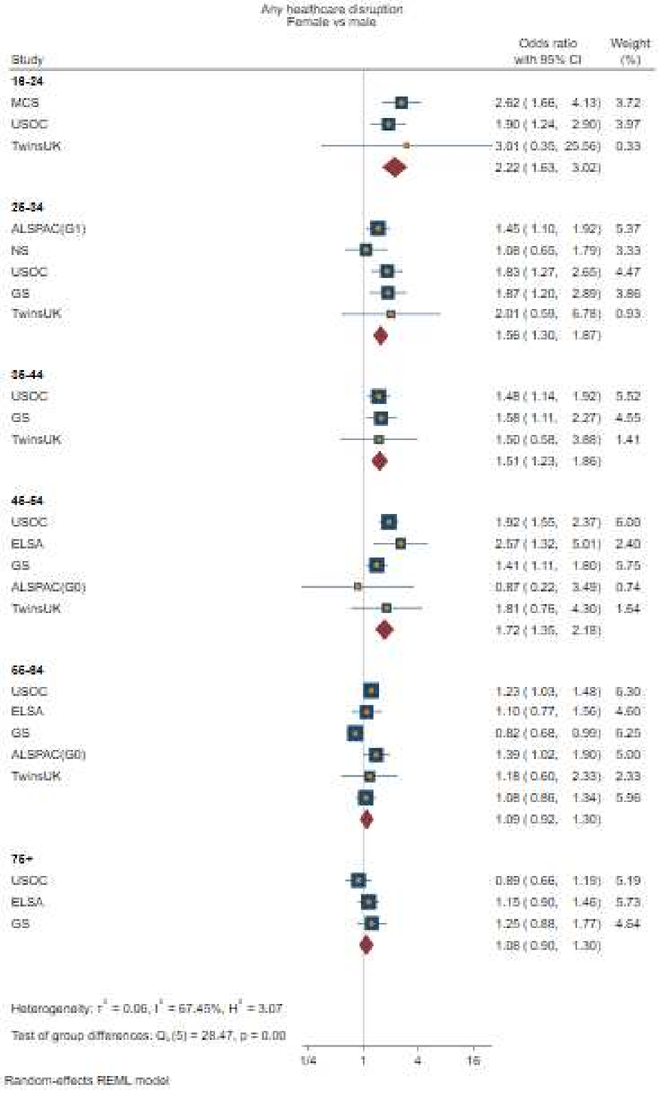

Occupational class

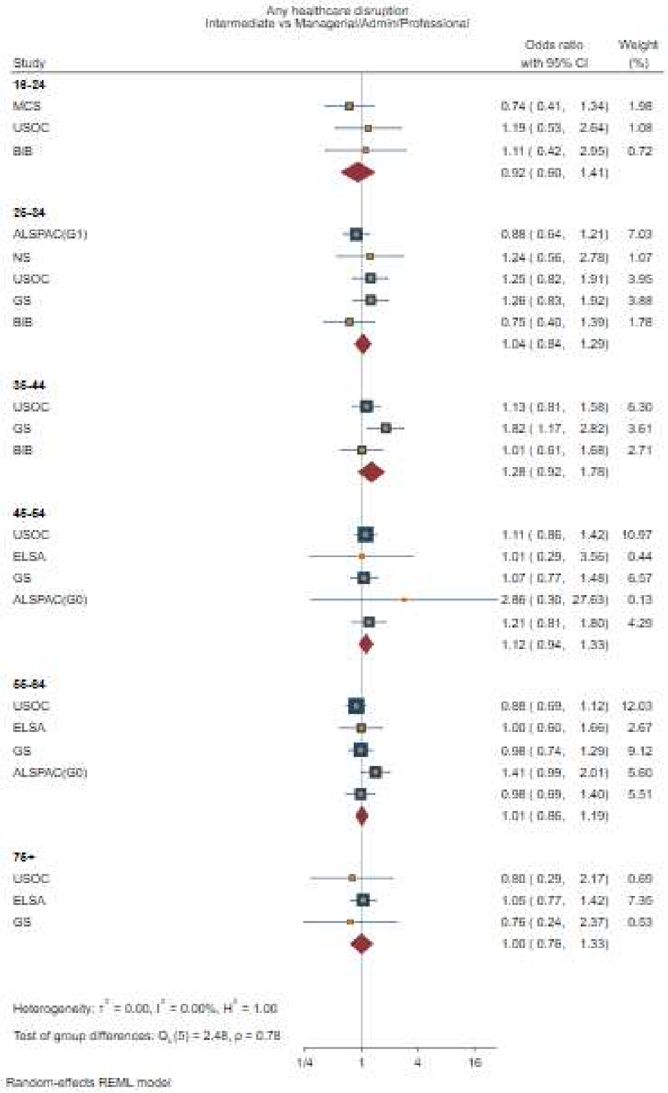

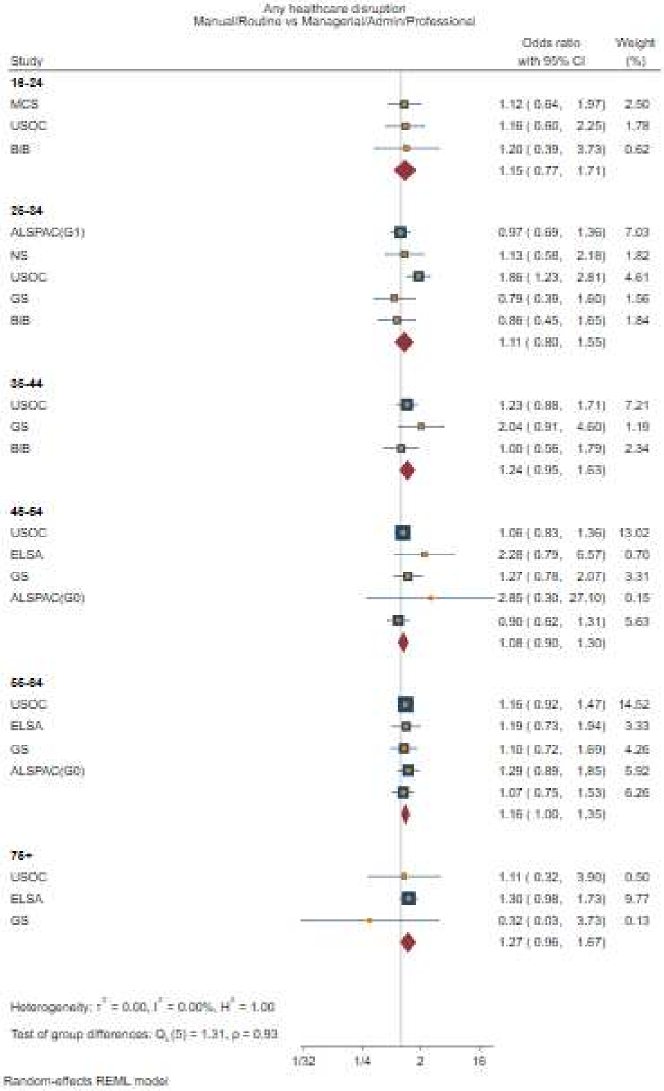
Any healthcare disruption
Manual/Routine vs Managerial/Admin/Professional
Study
Odds ratio with 95% CI
Weight (%)
16-24
MCS
1.12 (0.64, 1.97) 2.50
USOC
1.16 (0.60, 2.25) 1.78
BIB
1.20 (0.39, 3.73) 0.62
1.15 (0.77, 1.71)
25-34
ALSPAC(G1)
0.97 (0.69, 1.36) 7.03
NS
1.13 (0.58, 2.18) 1.82
USOC
1.86 (1.23, 2.81) 4.61
GS
0.79 (0.39, 1.60) 1.56
BIB
0.86 (0.45, 1.65) 1.84
1.11 (0.80, 1.55)
35-44
USOC
1.23 (0.88, 1.71) 7.21
GS
2.04 (0.91, 4.60) 1.19
BIB
1.00 (0.56, 1.79) 2.34
1.24 (0.95, 1.63)
45-54
USOC
1.06 (0.83, 1.36) 13.02
ELSA
2.28 (0.79, 6.57) 0.70
GS
1.27 (0.78, 2.07) 3.31
ALSPAC(G0)
2.85 (0.30, 27.10) 0.15
0.90 (0.62, 1.31) 5.63
1.08 (0.90, 1.30)
55-64
USOC
1.16 (0.92, 1.47) 14.62
ELSA
1.19 (0.73, 1.94) 3.33
GS
1.10 (0.72, 1.69) 4.26
ALSPAC(G0)
1.29 (0.89, 1.85) 5.92
1.07 (0.75, 1.53) 6.26
1.16 (1.00, 1.35)
75+
USOC
1.11 (0.32, 3.90) 0.50
ELSA
1.30 (0.98, 1.73) 9.77
GS
0.32 (0.03, 3.73) 0.13
1.27 (0.96, 1.67)
Heterogeneity: τ² = 0.00, I² = 0.00%, H² = 1.00
Test of group differences: Q_b(5) = 1.31, p = 0.93
1/32
1/4
2
16
Random-effects REML model

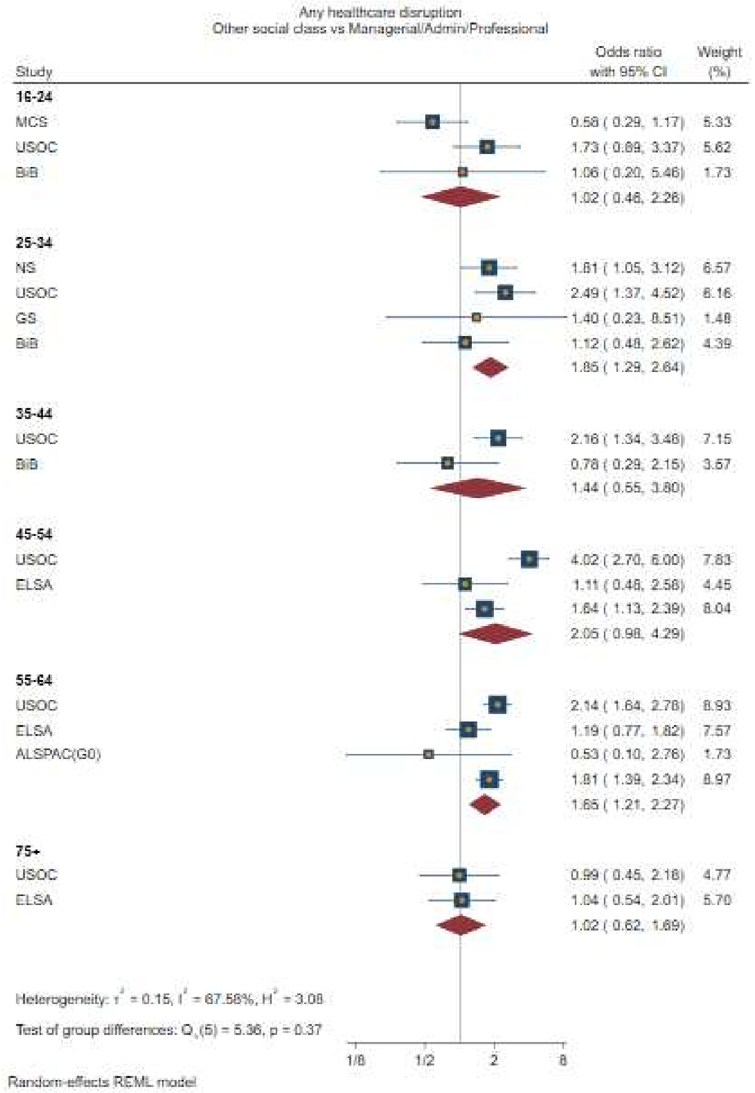
Any healthcare disruption
Other social class vs Managerial/Admin/Professional
Study
Odds ratio with 95% CI
Weight (%)
16-24
MCS 0.58 (0.29, 1.17) 5.33
USOC 1.73 (0.89, 3.37) 5.62
BiB 1.06 (0.20, 5.46) 1.73
1.02 (0.46, 2.26)
25-34
NS 1.81 (1.05, 3.12) 6.57
USOC 2.49 (1.37, 4.52) 6.16
GS 1.40 (0.23, 8.51) 1.48
BiB 1.12 (0.48, 2.62) 4.39
1.85 (1.29, 2.64)
35-44
USOC 2.16 (1.34, 3.48) 7.15
BiB 0.78 (0.29, 2.15) 3.57
1.44 (0.55, 3.80)
45-54
USOC 4.02 (2.70, 6.00) 7.83
ELSA 1.11 (0.48, 2.58) 4.45
1.64 (1.13, 2.39) 8.04
2.05 (0.98, 4.29)
55-64
USOC 2.14 (1.64, 2.78) 8.93
ELSA 1.19 (0.77, 1.82) 7.57
ALSPAC(G0) 0.53 (0.10, 2.76) 1.73
1.81 (1.39, 2.34) 8.97
1.65 (1.21, 2.27)
75+
USOC 0.99 (0.45, 2.18) 4.77
ELSA 1.04 (0.54, 2.01) 5.70
1.02 (0.62, 1.69)
Heterogeneity: τ² = 0.15, I² = 67.58%, H² = 3.08
Test of group differences: Q_b(5) = 5.36, p = 0.37
1/8
1/2
2
8
Random-effects REML model

151

Education

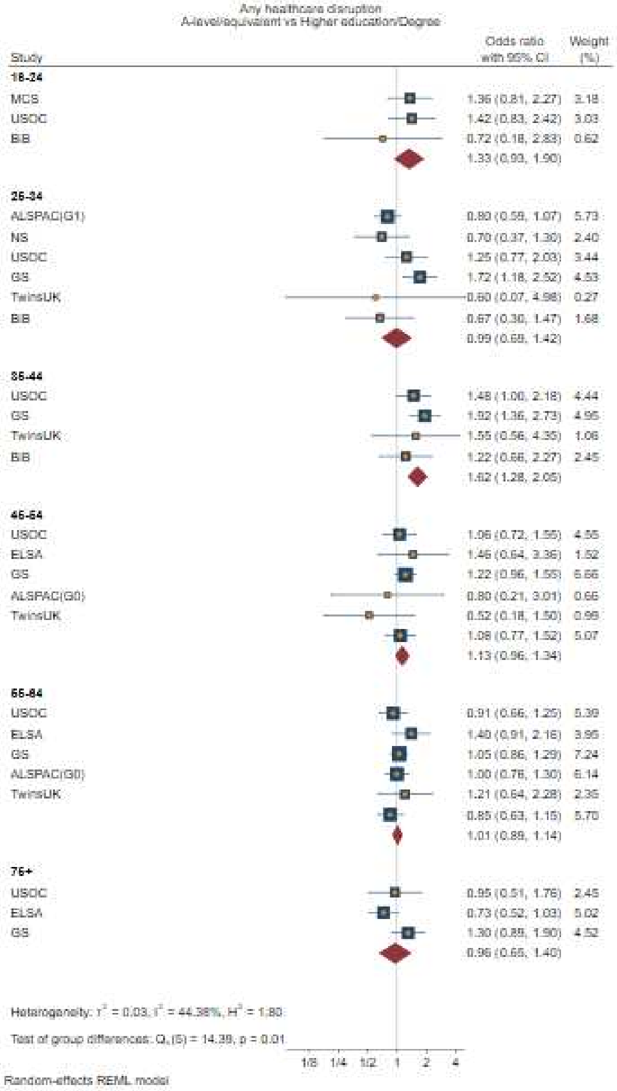
Any healthcare disruption
A-level/equivalent vs Higher education/Degree
Study
Odds ratio with 95% CI
Weight (%)
18-24
MCS 1.36 (0.81, 2.27) 3.18
USOC 1.42 (0.83, 2.42) 3.03
BiB 0.72 (0.18, 2.83) 0.62
1.33 (0.93, 1.90)
25-34
ALSPAC(G1) 0.80 (0.59, 1.07) 5.73
NS 0.70 (0.37, 1.30) 2.40
USOC 1.25 (0.77, 2.03) 3.44
GS 1.72 (1.18, 2.52) 4.53
TwinsUK 0.60 (0.07, 4.98) 0.27
BiB 0.67 (0.30, 1.47) 1.68
0.99 (0.69, 1.42)
35-44
USOC 1.48 (1.00, 2.18) 4.44
GS 1.92 (1.36, 2.73) 4.95
TwinsUK 1.55 (0.56, 4.35) 1.06
BiB 1.22 (0.66, 2.27) 2.45
1.62 (1.28, 2.05)
45-64
USOC 1.06 (0.72, 1.55) 4.55
ELSA 1.46 (0.64, 3.36) 1.52
GS 1.22 (0.96, 1.55) 6.66
ALSPAC(G0) 0.80 (0.21, 3.01) 0.66
TwinsUK 0.52 (0.18, 1.50) 0.99
1.08 (0.77, 1.52) 5.07
1.13 (0.96, 1.34)
65-74
USOC 0.91 (0.66, 1.25) 5.39
ELSA 1.40 (0.91, 2.16) 3.95
GS 1.05 (0.86, 1.29) 7.24
ALSPAC(G0) 1.00 (0.76, 1.30) 6.14
TwinsUK 1.21 (0.64, 2.28) 2.35
0.85 (0.63, 1.15) 5.70
1.01 (0.89, 1.14)
75+
USOC 0.95 (0.51, 1.76) 2.45
ELSA 0.73 (0.52, 1.03) 5.02
GS 1.30 (0.89, 1.90) 4.52
0.96 (0.65, 1.40)
Heterogeneity: τ² = 0.03, I² = 44.38%, H² = 1.80
Test of group differences: Q_b(5) = 14.39, p = 0.01
1/8 1/4 1/2 1 2 4
Random-effects REML model

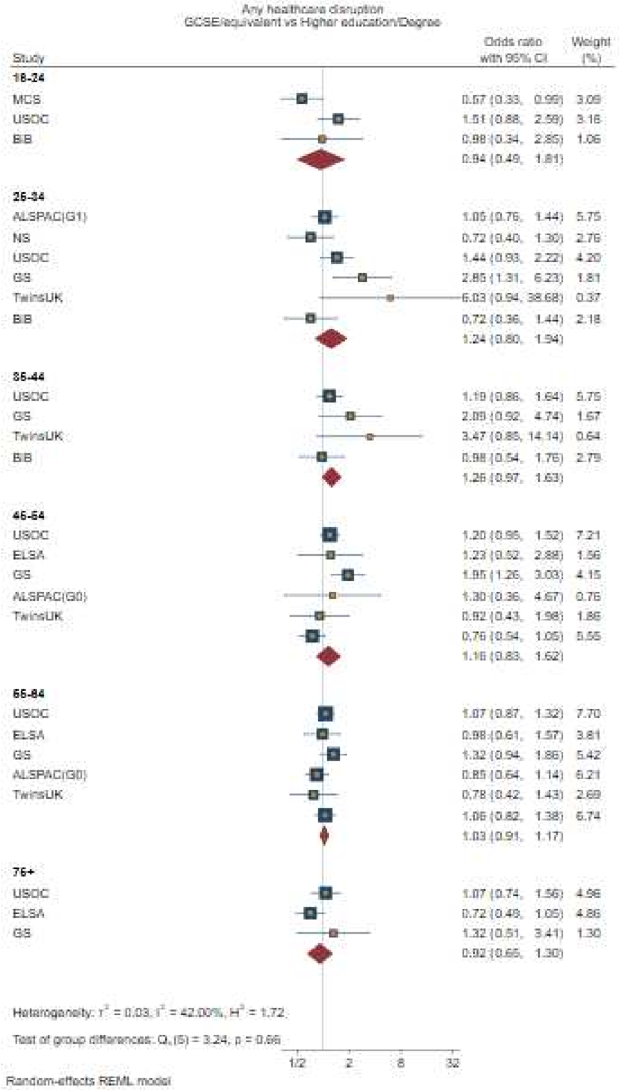
Any healthcare disruption
GCSE/equivalent vs Higher education/Degree
Study
Odds ratio with 95% CI
Weight (%)
18-24
MCS
USOC
BIB
0.57 (0.33, 0.99) 3.09
1.51 (0.88, 2.59) 3.16
0.98 (0.34, 2.85) 1.06
0.94 (0.49, 1.81)
25-34
ALSPAC(G1)
NS
USOC
GS
TwinsUK
BIB
1.05 (0.76, 1.44) 5.75
0.72 (0.40, 1.30) 2.76
1.44 (0.93, 2.22) 4.20
2.85 (1.31, 6.23) 1.81
6.03 (0.94, 38.68) 0.37
0.72 (0.36, 1.44) 2.18
1.24 (0.80, 1.94)
35-44
USOC
GS
TwinsUK
BIB
1.19 (0.86, 1.64) 5.75
2.09 (0.92, 4.74) 1.67
3.47 (0.85, 14.14) 0.64
0.98 (0.54, 1.76) 2.79
1.26 (0.97, 1.63)
45-64
USOC
ELSA
GS
ALSPAC(G0)
TwinsUK
1.20 (0.95, 1.52) 7.21
1.23 (0.52, 2.88) 1.56
1.95 (1.26, 3.03) 4.15
1.30 (0.36, 4.67) 0.76
0.92 (0.43, 1.98) 1.86
0.76 (0.54, 1.05) 5.55
1.16 (0.83, 1.62)
65-84
USOC
ELSA
GS
ALSPAC(G0)
TwinsUK
1.07 (0.87, 1.32) 7.70
0.98 (0.61, 1.57) 3.81
1.32 (0.94, 1.86) 5.42
0.85 (0.64, 1.14) 6.21
0.78 (0.42, 1.43) 2.69
1.06 (0.82, 1.38) 6.74
1.03 (0.91, 1.17)
75+
USOC
ELSA
GS
1.07 (0.74, 1.56) 4.96
0.72 (0.49, 1.05) 4.86
1.32 (0.51, 3.41) 1.30
0.92 (0.65, 1.30)
Heterogeneity: τ^2 = 0.03, I^2 = 42.00%, H^2 = 1.72
Test of group differences: $Q_b(5)$ = 3.24, p = 0.66
1/2
2
8
32
Random-effects REML model

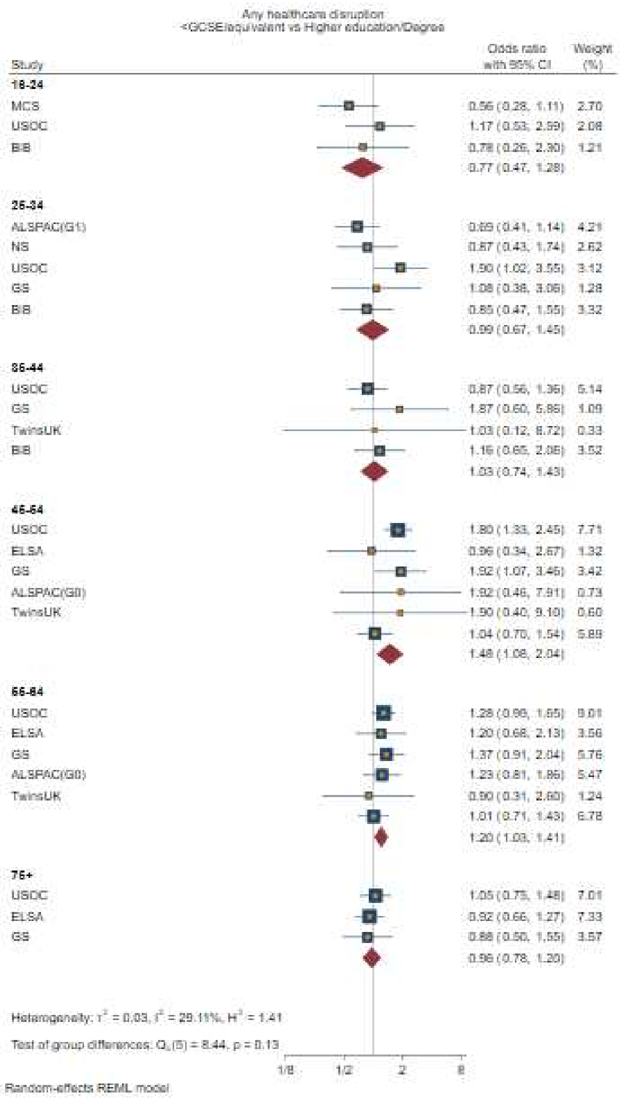
Any healthcare disruption
<GCSE/equivalent vs Higher education/Degree
Study
Odds ratio with 95% CI
Weight (%)
18-24
MCS
0.56 (0.28, 1.11) 2.70
USOC
1.17 (0.53, 2.59) 2.08
BIB
0.78 (0.26, 2.30) 1.21
0.77 (0.47, 1.28)
25-34
ALSPAC(G1)
0.69 (0.41, 1.14) 4.21
NS
0.87 (0.43, 1.74) 2.62
USOC
1.90 (1.02, 3.55) 3.12
GS
1.08 (0.38, 3.06) 1.28
BIB
0.85 (0.47, 1.55) 3.32
0.99 (0.67, 1.45)
35-44
USOC
0.87 (0.56, 1.36) 5.14
GS
1.87 (0.60, 5.86) 1.09
TwinsUK
1.03 (0.12, 8.72) 0.33
BIB
1.16 (0.65, 2.06) 3.52
1.03 (0.74, 1.43)
45-64
USOC
1.80 (1.33, 2.45) 7.71
ELSA
0.96 (0.34, 2.67) 1.32
GS
1.92 (1.07, 3.46) 3.42
ALSPAC(G0)
1.92 (0.46, 7.91) 0.73
TwinsUK
1.90 (0.40, 9.10) 0.60
1.04 (0.70, 1.54) 5.89
1.48 (1.08, 2.04)
65-74
USOC
1.28 (0.99, 1.65) 9.01
ELSA
1.20 (0.68, 2.13) 3.56
GS
1.37 (0.91, 2.04) 5.76
ALSPAC(G0)
1.23 (0.81, 1.86) 5.47
TwinsUK
0.90 (0.31, 2.60) 1.24
1.01 (0.71, 1.43) 6.78
1.20 (1.03, 1.41)
75+
USOC
1.05 (0.75, 1.48) 7.01
ELSA
0.92 (0.66, 1.27) 7.33
GS
0.88 (0.50, 1.55) 3.57
0.96 (0.78, 1.20)
Heterogeneity: τ^2 = 0.03, I^2 = 29.11%, H^2 = 1.41
Test of group differences: $Q_b(5)$ = 8.44, p = 0.13
1/8
1/2
2
8
Random-effects REML model

Ethnicity

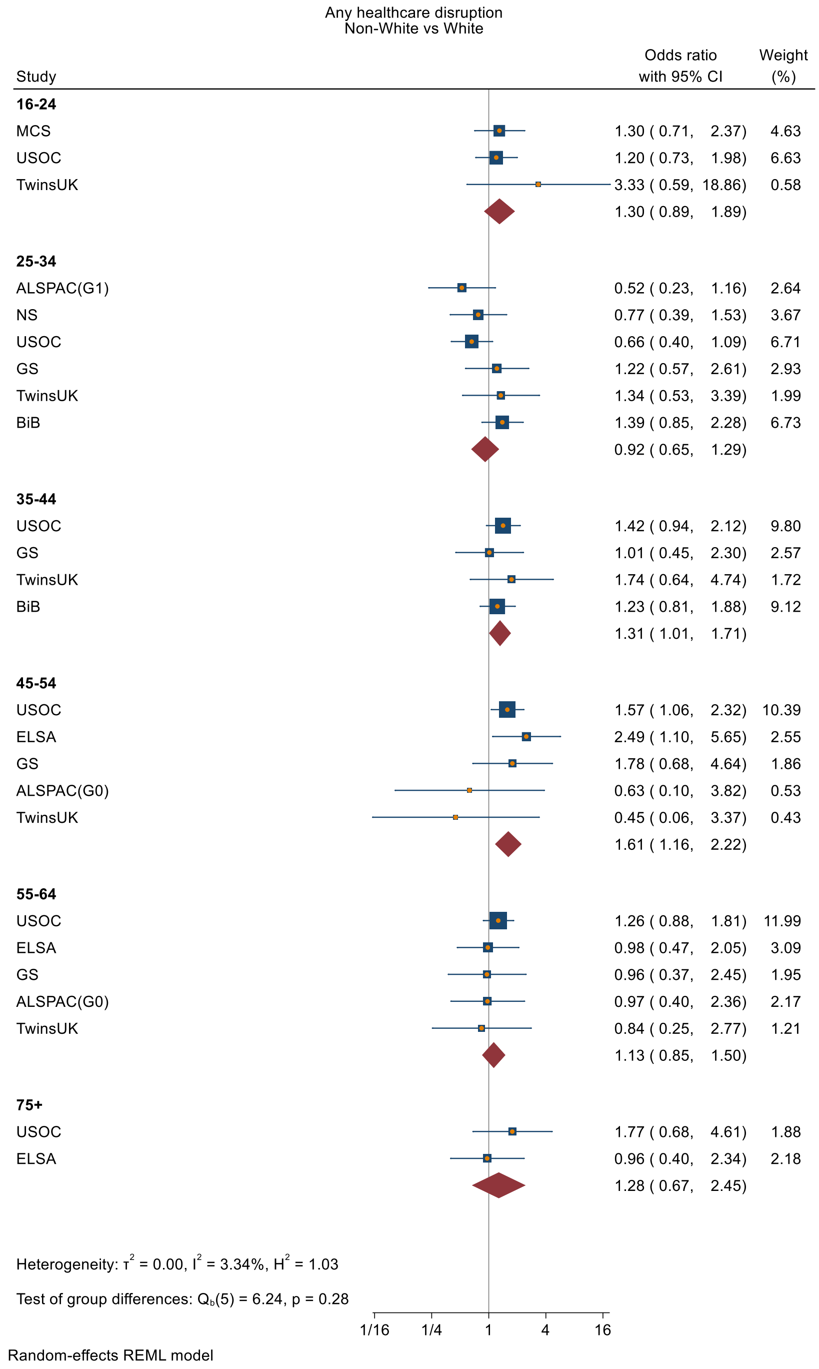
Any healthcare disruption
Non-White vs White
Study
Odds ratio with 95% CI
Weight (%)
16-24
MCS 1.30 (0.71, 2.37) 4.63
USOC 1.20 (0.73, 1.98) 6.63
TwinsUK 3.33 (0.59, 18.86) 0.58
1.30 (0.89, 1.89)
25-34
ALSPAC(G1) 0.52 (0.23, 1.16) 2.64
NS 0.77 (0.39, 1.53) 3.67
USOC 0.66 (0.40, 1.09) 6.71
GS 1.22 (0.57, 2.61) 2.93
TwinsUK 1.34 (0.53, 3.39) 1.99
BiB 1.39 (0.85, 2.28) 6.73
0.92 (0.65, 1.29)
35-44
USOC 1.42 (0.94, 2.12) 9.80
GS 1.01 (0.45, 2.30) 2.57
TwinsUK 1.74 (0.64, 4.74) 1.72
BiB 1.23 (0.81, 1.88) 9.12
1.31 (1.01, 1.71)
45-54
USOC 1.57 (1.06, 2.32) 10.39
ELSA 2.49 (1.10, 5.65) 2.55
GS 1.78 (0.68, 4.64) 1.86
ALSPAC(G0) 0.63 (0.10, 3.82) 0.53
TwinsUK 0.45 (0.06, 3.37) 0.43
1.61 (1.16, 2.22)
55-64
USOC 1.26 (0.88, 1.81) 11.99
ELSA 0.98 (0.47, 2.05) 3.09
GS 0.96 (0.37, 2.45) 1.95
ALSPAC(G0) 0.97 (0.40, 2.36) 2.17
TwinsUK 0.84 (0.25, 2.77) 1.21
1.13 (0.85, 1.50)
75+
USOC 1.77 (0.68, 4.61) 1.88
ELSA 0.96 (0.40, 2.34) 2.18
1.28 (0.67, 2.45)
Heterogeneity: τ² = 0.00, I² = 3.34%, H² = 1.03
Test of group differences: Qb(5) = 6.24, p = 0.28
1/16 1/4 1 4 16
Random-effects REML model

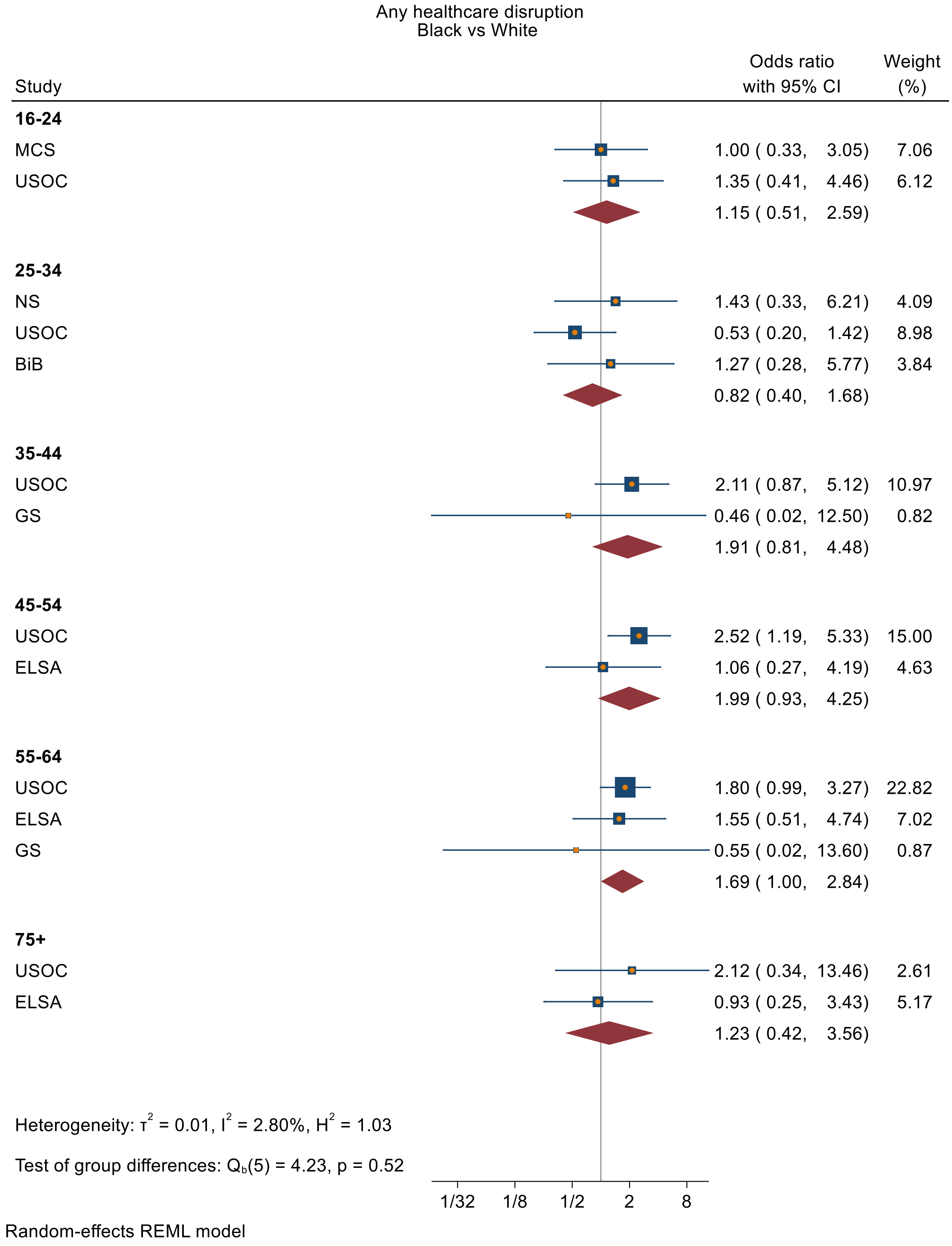
Any healthcare disruption
Black vs White
Study
Odds ratio with 95% CI
Weight (%)
16-24
MCS 1.00 (0.33, 3.05) 7.06
USOC 1.35 (0.41, 4.46) 6.12
1.15 (0.51, 2.59)
25-34
NS 1.43 (0.33, 6.21) 4.09
USOC 0.53 (0.20, 1.42) 8.98
BiB 1.27 (0.28, 5.77) 3.84
0.82 (0.40, 1.68)
35-44
USOC 2.11 (0.87, 5.12) 10.97
GS 0.46 (0.02, 12.50) 0.82
1.91 (0.81, 4.48)
45-54
USOC 2.52 (1.19, 5.33) 15.00
ELSA 1.06 (0.27, 4.19) 4.63
1.99 (0.93, 4.25)
55-64
USOC 1.80 (0.99, 3.27) 22.82
ELSA 1.55 (0.51, 4.74) 7.02
GS 0.55 (0.02, 13.60) 0.87
1.69 (1.00, 2.84)
75+
USOC 2.12 (0.34, 13.46) 2.61
ELSA 0.93 (0.25, 3.43) 5.17
1.23 (0.42, 3.56)
Heterogeneity: τ² = 0.01, I² = 2.80%, H² = 1.03
Test of group differences: Qb(5) = 4.23, p = 0.52
1/32
1/8
1/2
2
8
Random-effects REML model

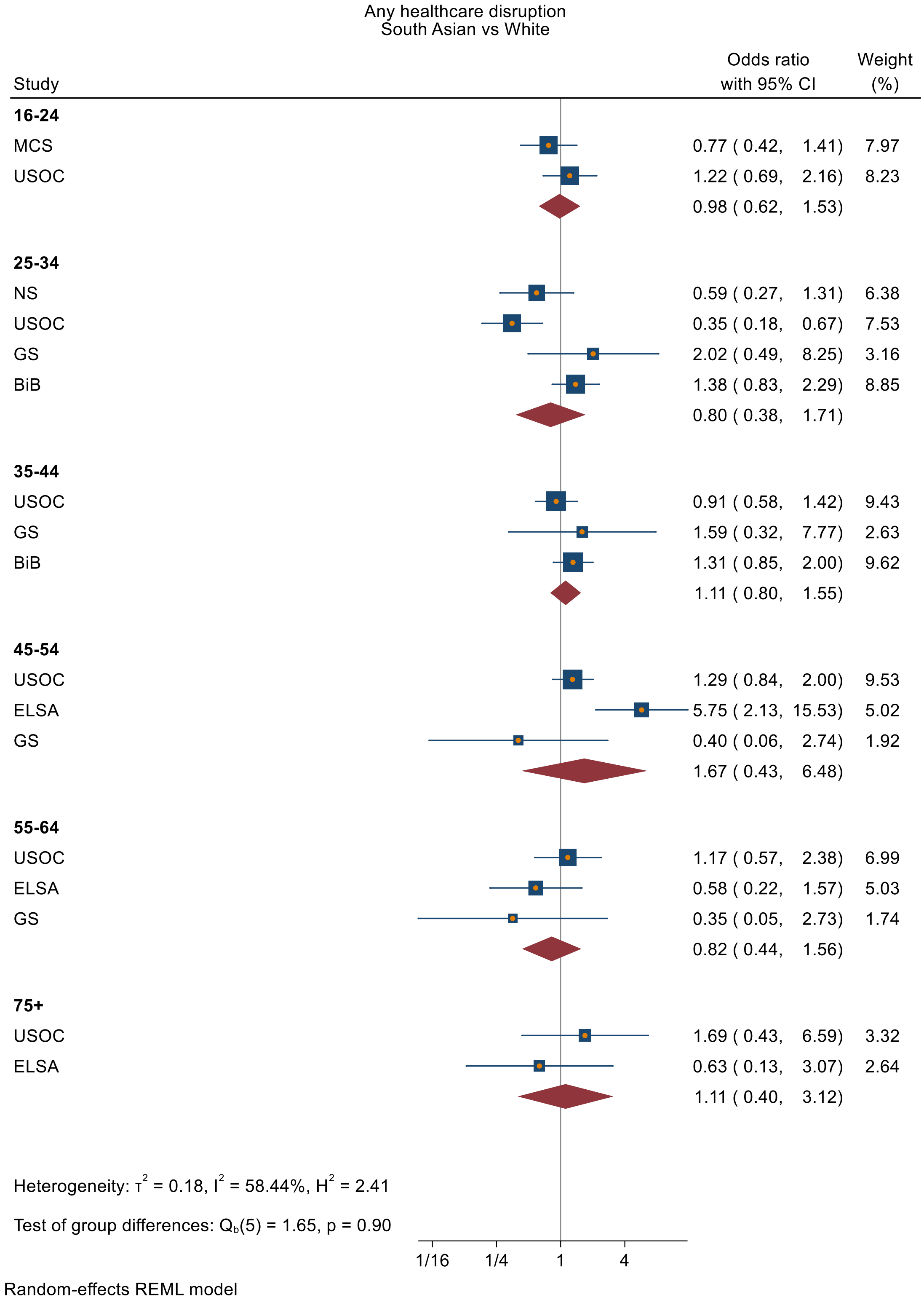
Any healthcare disruption
South Asian vs White
Study
Odds ratio with 95% CI
Weight (%)
16-24
MCS 0.77 (0.42, 1.41) 7.97
USOC 1.22 (0.69, 2.16) 8.23
0.98 (0.62, 1.53)
25-34
NS 0.59 (0.27, 1.31) 6.38
USOC 0.35 (0.18, 0.67) 7.53
GS 2.02 (0.49, 8.25) 3.16
BiB 1.38 (0.83, 2.29) 8.85
0.80 (0.38, 1.71)
35-44
USOC 0.91 (0.58, 1.42) 9.43
GS 1.59 (0.32, 7.77) 2.63
BiB 1.31 (0.85, 2.00) 9.62
1.11 (0.80, 1.55)
45-54
USOC 1.29 (0.84, 2.00) 9.53
ELSA 5.75 (2.13, 15.53) 5.02
GS 0.40 (0.06, 2.74) 1.92
1.67 (0.43, 6.48)
55-64
USOC 1.17 (0.57, 2.38) 6.99
ELSA 0.58 (0.22, 1.57) 5.03
GS 0.35 (0.05, 2.73) 1.74
0.82 (0.44, 1.56)
75+
USOC 1.69 (0.43, 6.59) 3.32
ELSA 0.63 (0.13, 3.07) 2.64
1.11 (0.40, 3.12)
Heterogeneity: τ² = 0.18, I² = 58.44%, H² = 2.41
Test of group differences: Q_b(5) = 1.65, p = 0.90
1/16
1/4
1
4
Random-effects REML model

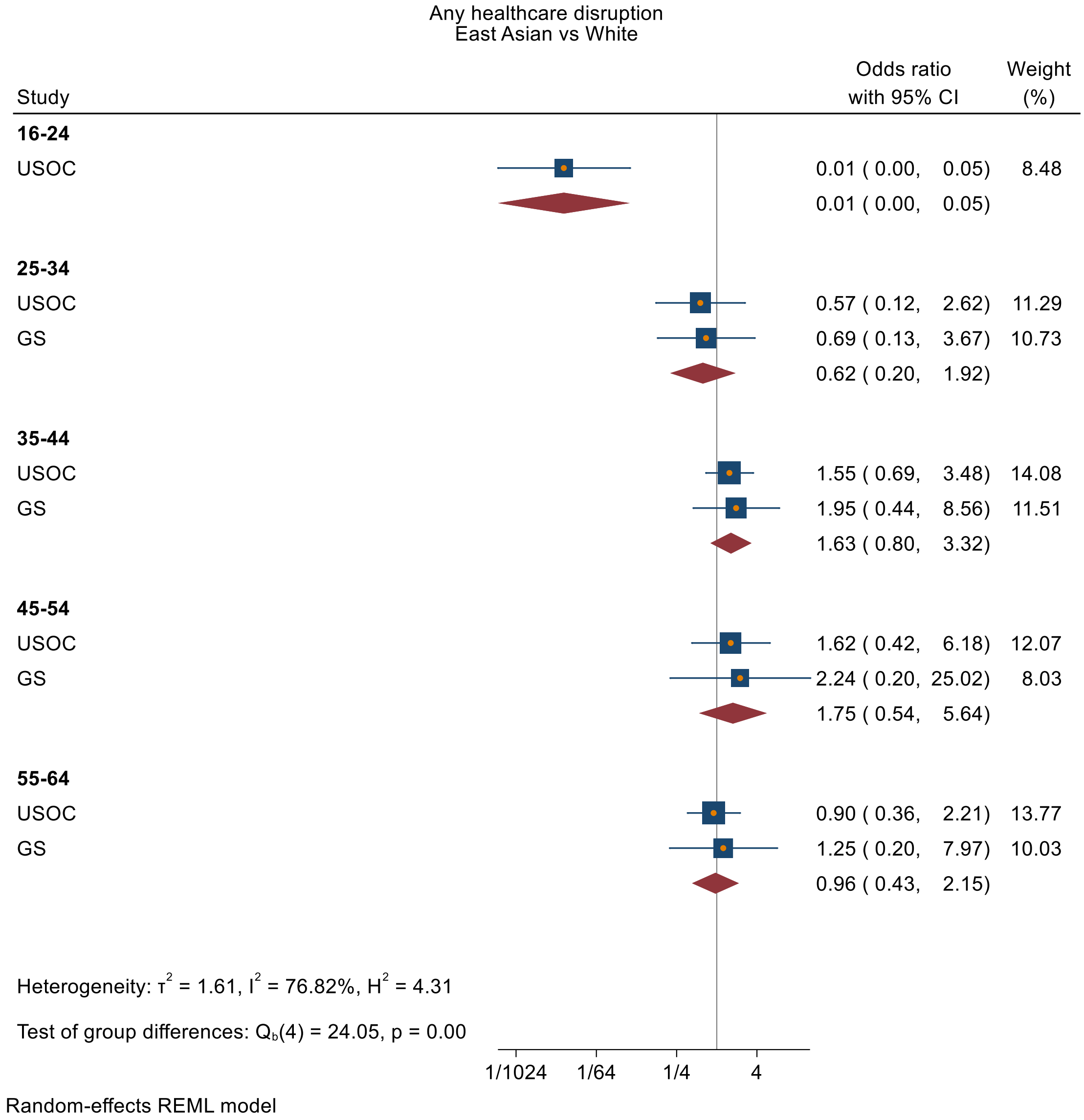

Maddock J, *et al. BMJ Open* 2022; 12:e064981. doi: 10.1136/bmjopen-2022-064981

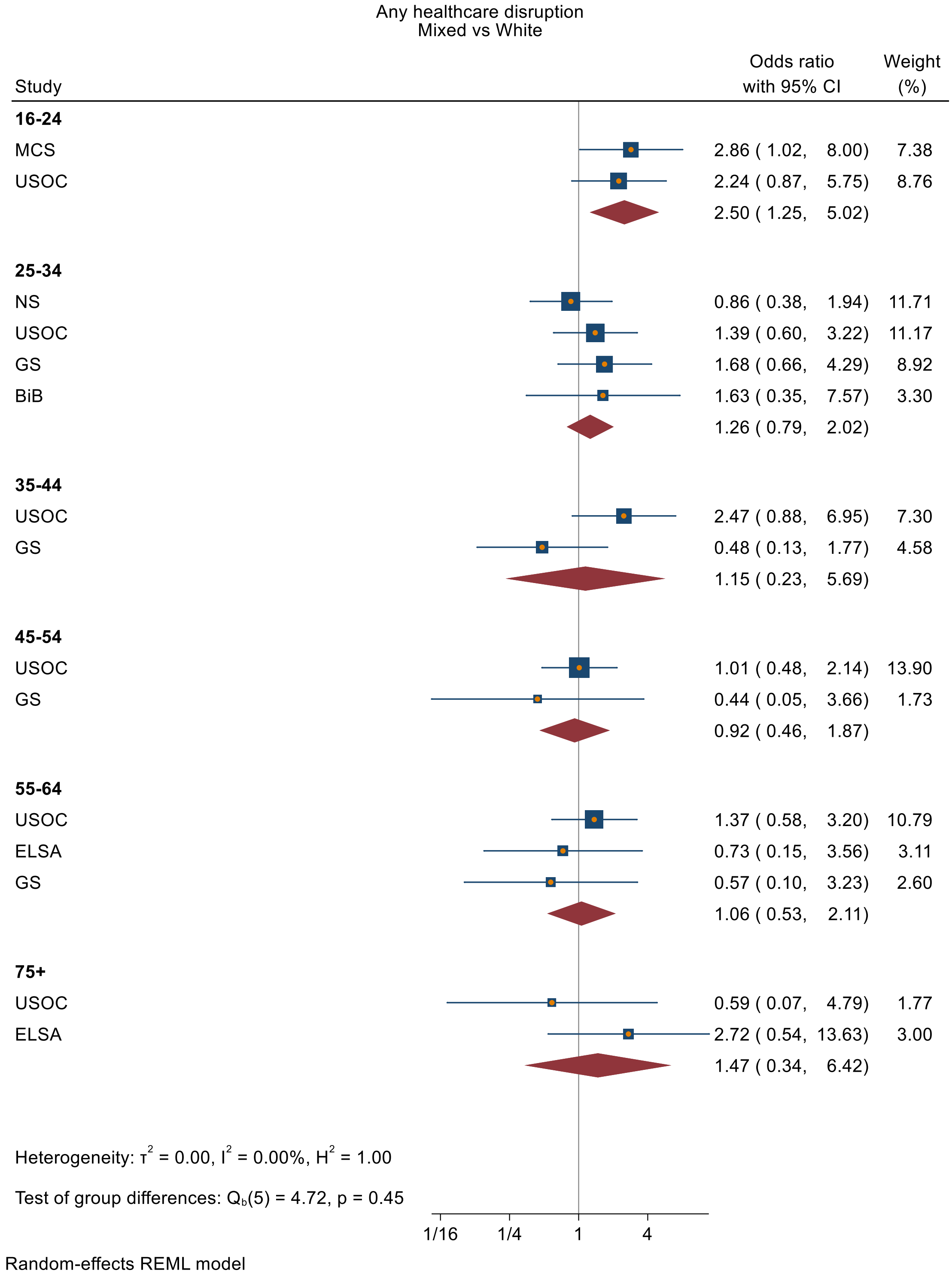
Any healthcare disruption
Mixed vs White
Study
Odds ratio with 95% CI
Weight (%)
16-24
MCS 2.86 (1.02, 8.00) 7.38
USOC 2.24 (0.87, 5.75) 8.76
2.50 (1.25, 5.02)
25-34
NS 0.86 (0.38, 1.94) 11.71
USOC 1.39 (0.60, 3.22) 11.17
GS 1.68 (0.66, 4.29) 8.92
BiB 1.63 (0.35, 7.57) 3.30
1.26 (0.79, 2.02)
35-44
USOC 2.47 (0.88, 6.95) 7.30
GS 0.48 (0.13, 1.77) 4.58
1.15 (0.23, 5.69)
45-54
USOC 1.01 (0.48, 2.14) 13.90
GS 0.44 (0.05, 3.66) 1.73
0.92 (0.46, 1.87)
55-64
USOC 1.37 (0.58, 3.20) 10.79
ELSA 0.73 (0.15, 3.56) 3.11
GS 0.57 (0.10, 3.23) 2.60
1.06 (0.53, 2.11)
75+
USOC 0.59 (0.07, 4.79) 1.77
ELSA 2.72 (0.54, 13.63) 3.00
1.47 (0.34, 6.42)
Heterogeneity: τ² = 0.00, I² = 0.00%, H² = 1.00
Test of group differences: Q_b(5) = 4.72, p = 0.45
1/16
1/4
1
4
Random-effects REML model

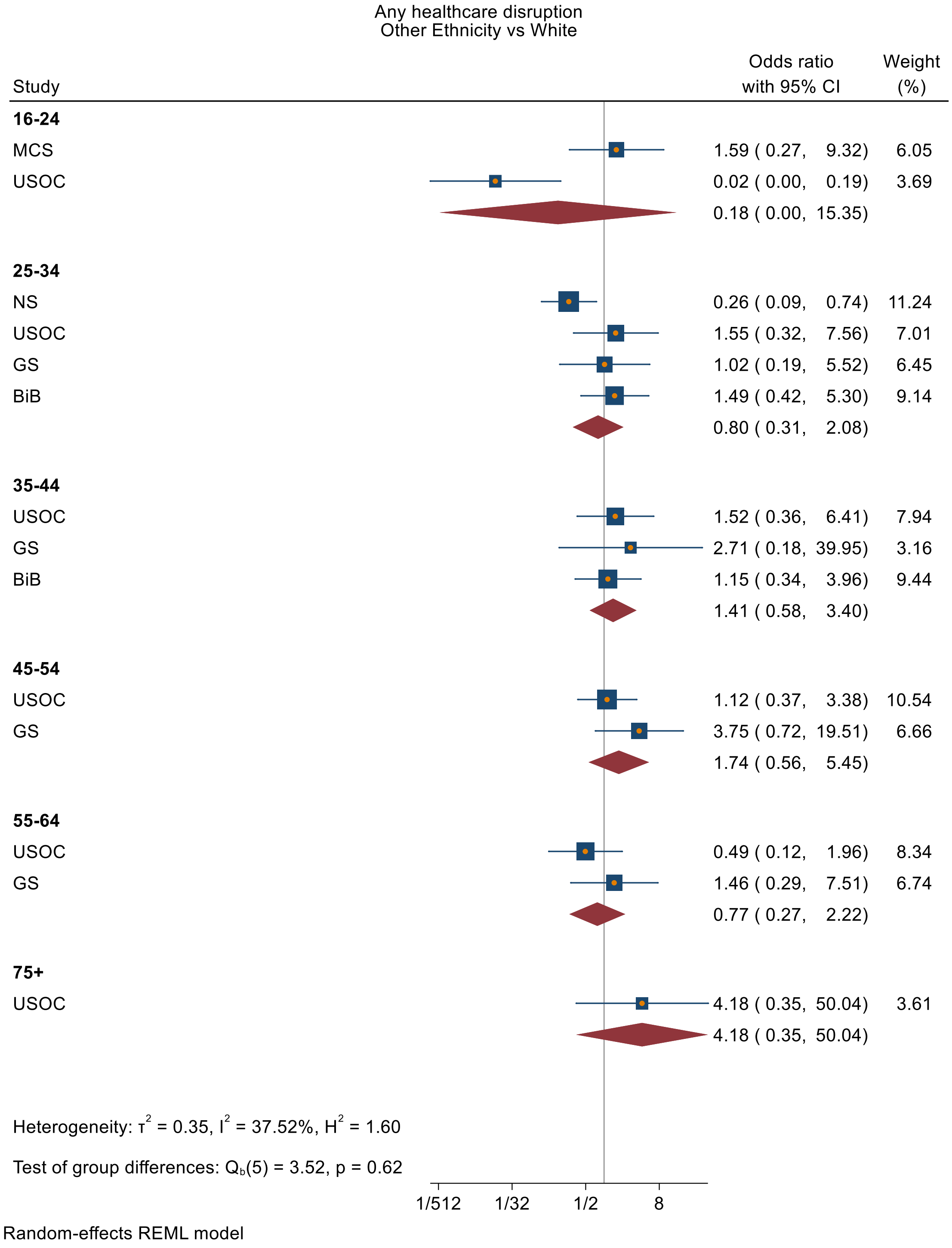
Any healthcare disruption
Other Ethnicity vs White
Study
Odds ratio with 95% CI
Weight (%)
16-24
MCS 1.59 (0.27, 9.32) 6.05
USOC 0.02 (0.00, 0.19) 3.69
0.18 (0.00, 15.35)
25-34
NS 0.26 (0.09, 0.74) 11.24
USOC 1.55 (0.32, 7.56) 7.01
GS 1.02 (0.19, 5.52) 6.45
BiB 1.49 (0.42, 5.30) 9.14
0.80 (0.31, 2.08)
35-44
USOC 1.52 (0.36, 6.41) 7.94
GS 2.71 (0.18, 39.95) 3.16
BiB 1.15 (0.34, 3.96) 9.44
1.41 (0.58, 3.40)
45-54
USOC 1.12 (0.37, 3.38) 10.54
GS 3.75 (0.72, 19.51) 6.66
1.74 (0.56, 5.45)
55-64
USOC 0.49 (0.12, 1.96) 8.34
GS 1.46 (0.29, 7.51) 6.74
0.77 (0.27, 2.22)
75+
USOC 4.18 (0.35, 50.04) 3.61
4.18 (0.35, 50.04)
Heterogeneity: τ^2 = 0.35, I^2 = 37.52%, H^2 = 1.60
Test of group differences: $Q_b(5)$ = 3.52, p = 0.62
1/512
1/32
1/2
8
Random-effects REML model
