# Supplementary material for: Inequalities in healthcare disruptions during the COVID-19 pandemic: evidence from 12 UK population-based longitudinal studies
Source: BMJ Open. 2022 Oct 13;12(10):e064981. doi: 10.1136/bmjopen-2022-064981 (PMC9561494; doi:10.1136/bmjopen-2022-064981)
Supplement: Author's manuscript [file bmjopen-2022-064981.draft_revisions.pdf]

BMJ Open is committed to open peer review. As part of this commitment we make the peer review history of every article we publish publicly available.

When an article is published we post the peer reviewers' comments and the authors' responses online. We also post the versions of the paper that were used during peer review. These are the versions that the peer review comments apply to.

The versions of the paper that follow are the versions that were submitted during the peer review process. They are not the versions of record or the final published versions. They should not be cited or distributed as the published version of this manuscript.

BMJ Open is an open access journal and the full, final, typeset and author-corrected version of record of the manuscript is available on our site with no access controls, subscription charges or pay-per-view fees (http://bmjopen.bmj.com).

If you have any questions on BMJ Open's open peer review process please email info.bmjopen@bmj.com

BMJ Open

Inequalities in healthcare disruptions during the COVID-19 pandemic: Evidence from 12 UK population-based longitudinal studies

Journal:	*BMJ Open*
Manuscript ID	bmjopen-2022-064981
Article Type:	Original research
Date Submitted by the Author:	20-May-2022
Complete List of Authors:	Maddock, Jane ; University College London Faculty of Population Health Sciences, Cardiovascular Science<br>Parsons, Sam; UCL IOE, Centre for Longitudinal Studies<br>Di Gessa, Giorgio; UCL, Institute of Epidemiology and Health Care<br>Green, Michael; University of Glasgow MRC/CSO Social and Public Health Sciences Unit<br>Thompson, Ellen J.; King's College London Department of Twin Research and Genetic Epidemiology<br>Stevenson, Anna J.; The University of Edinburgh Centre for Genomic and Experimental Medicine<br>Kwong, Alex; MRC Integrative Epidemiology Unit; The University of Edinburgh Division of Psychiatry<br>McElroy, Eoin; University of Leicester, Department of Neuroscience, Psychology and Behaviour<br>Santorelli, Gillian; Bradford Teaching Hospitals NHS Foundation Trust<br>Silverwood, Richard; UCL Institute of Education Centre for Longitudinal Studies<br>Captur, Gaby; MRC Unit for Lifelong Health and Ageing, UCL<br>Chaturvedi, Nishi; University College London<br>Steves, Claire J.; King's College London Department of Twin Research and Genetic Epidemiology<br>Steptoe, Andrew; University College London, Department of Behavioural Science and Health<br>Patalay, Praveetha; University College London, Population Health and Experimental Medicine<br>Ploubidis, George; UCL Institute of Education Centre for Longitudinal Studies<br>Katikireddi, Srinivasa; University of Glasgow
Keywords:	COVID-19, EPIDEMIOLOGY, Health policy < HEALTH SERVICES ADMINISTRATION & MANAGEMENT, Quality in health care < HEALTH SERVICES ADMINISTRATION & MANAGEMENT, PUBLIC HEALTH, SOCIAL MEDICINE

For peer review only - http://bmjopen.bmj.com/site/about/guidelines.xhtml

I, the Submitting Author has the right to grant and does grant on behalf of all authors of the Work (as defined in the below author licence), an exclusive licence and/or a non-exclusive licence for contributions from authors who are: i) UK Crown employees; ii) where BMJ has agreed a CC-BY licence shall apply, and/or iii) in accordance with the terms applicable for US Federal Government officers or employees acting as part of their official duties; on a worldwide, perpetual, irrevocable, royalty-free basis to BMJ Publishing Group Ltd ("BMJ") its licensees and where the relevant Journal is co-owned by BMJ to the co-owners of the Journal, to publish the Work in this journal and any other BMJ products and to exploit all rights, as set out in our licence.

The Submitting Author accepts and understands that any supply made under these terms is made by BMJ to the Submitting Author unless you are acting as an employee on behalf of your employer or a postgraduate student of an affiliated institution which is paying any applicable article publishing charge ("APC") for Open Access articles. Where the Submitting Author wishes to make the Work available on an Open Access basis (and intends to pay the relevant APC), the terms of reuse of such Open Access shall be governed by a Creative Commons licence – details of these licences and which Creative Commons licence will apply to this Work are set out in our licence referred to above.

Other than as permitted in any relevant BMJ Author's Self Archiving Policies, I confirm this Work has not been accepted for publication elsewhere, is not being considered for publication elsewhere and does not duplicate material already published. I confirm all authors consent to publication of this Work and authorise the granting of this licence.

For peer review only

Inequalities in healthcare disruptions during the COVID-19 pandemic: Evidence from 12 UK population-based longitudinal studies

Authors:

Jane Maddock*[1^], Sam Parsons[2^] Giorgio Di Gessa[3^], Michael J. Green[4^], Ellen J. Thompson[5^], Anna J. Stevenson[6], Alex S.F. Kwong[6,7], Eoin McElroy[8], Gillian Santorelli[9], Richard J. Silverwood[2], Gabriella Captur[1], Nishi Chaturvedi[1], Claire J. Steves[5], Andrew Steptoe[3], Praveetha Patalay[1,2], George B. Ploubidis[2], Srinivasa Vittal Katikireddi[4]

^Joint first authors

1 MRC Unit for Lifelong Health and Ageing, University College London

2 Centre for Longitudinal Studies, UCL Social Research Institute, University College London

3 Institute of Epidemiology and Health Care, University College London

4 MRC/CSO Social & Public Health Sciences Unit, University of Glasgow

5 Department of Twin Research and Genetic Epidemiology, School of Life Course Sciences, King's College London

6 Division of Psychiatry, University of Edinburgh

7 MRC Integrative Epidemiology Unit, University of Bristol

8 Department of Neuroscience, Psychology and Behaviour, University of Leicester

9 Born in Bradford, Bradford Institute for Health Research, Temple Bank House, Bradford Royal Infirmary

*Correspondence to: Dr Jane Maddock jane.maddock@ucl.ac.uk

For peer review only - http://bmjopen.bmj.com/site/about/guidelines.xhtml

Abstract

Objectives: We investigated associations between multiple socio-demographic characteristics (sex, age, occupational social class, education, and ethnicity) and self-reported healthcare disruptions during the early stages of the COVID-19 pandemic.

Setting: Community-dwelling participants in the United Kingdom between April 2020 up to January 2021.

Participants: Over 68,000 participants from 12 longitudinal studies.

Outcomes: Self-reported healthcare disruption to medication access, procedures, appointments.

Results: Prevalence of healthcare disruption varied substantially across studies: between 6% and 32% reported any disruption, with 1% to 10% experiencing disruptions in medication, 1% to 17% experiencing disruption in procedures and 4% to 28% experiencing disruption in clinical appointments. Females (Odd Ratio (OR): 1.27 [95%CI: 1.15,1.40]; I^2=53%), older persons (e.g., OR: 1.39 [1.13,1.72]; I^2=77% for 65-75y vs 45-54y), and Ethnic minorities (excluding White minorities) (OR: 1.19 [1.05,1.35]; I^2=0% vs White) were more likely to report healthcare disruptions. Those in a more disadvantaged social class were also more likely to report healthcare disruptions (e.g., OR: 1.17 [1.08, 1.27]; I^2=0% for manual/routine vs managerial/professional), but no clear differences were observed by education. We did not find evidence that these associations differed by shielding status or age.

Conclusions: Healthcare disruptions during the COVID-19 pandemic could contribute to the maintenance or widening of existing health inequalities.

Keywords: Healthcare Disparities; Health Services Accessibility; Longitudinal Studies; United Kingdom; Socioeconomic Factors; Ethnicity; Social Class

For peer review only

Introduction

The coronavirus disease 2019 (COVID-19) pandemic is affecting all aspects of society. Health systems world-wide have faced major disruption as they respond to large increases in demand arising from the COVID-19 disease.[1–5] Furthermore, healthcare access has been reduced by governmental control measures and the public's fear of contracting infection.[6] Disruptions may have both short and long-term health consequences as preventive treatments are foregone, disease surveillance is interrupted and disease diagnoses are delayed. While the disruption of health systems can impact the entire population, it has become apparent that not all groups have been affected equally. For example, recent evidence has demonstrated that both elective and emergency hospital admissions vary by socioeconomic deprivation and ethnic minority quintiles, with the more deprived areas showing a large fall in elective admissions, and areas with high ethnic minority populations showing larger falls in emergency admissions.[5] Understanding the impacts of the pandemic on health systems and on equity of healthcare access is therefore a major policy priority.

In the UK, the National Health Service (NHS) provides free healthcare and prioritises equity of delivery. However, the UK's relatively high COVID-19 burden and associated repeated lockdown measures have raised concerns that the health system may not be providing accessible care to those who need it most. Recent reports from NHS Digital indicate a large increase in those waiting 12 months or more for elective treatments in February 2021 compared to March 2020.[7] Furthermore, despite decreases in attendance at accident and emergency services,[4] the number of patients waiting over 12 hours for admission was 34% higher in January 2021 than January 2020. Disruption to pharmacological treatments has also been reported with delays in accessing medication.[8,9] However, a comprehensive assessment of inequalities in healthcare disruption in the community is lacking.

It is well known that health systems do not meet the needs of all social groups equitably, with marked health inequalities by sex, ethnicity, and socioeconomic position.[10,11] For example, the inverse care law demonstrates that health service provision is often not allocated according to need, with more socioeconomically deprived areas relatively under-served.[12] Given the barriers that some social groups

For peer review only - http://bmjopen.bmj.com/site/about/guidelines.xhtml

face in accessing high quality healthcare, there is considerable concern that disadvantaged groups (e.g., ethnic minorities) will be disproportionately impacted by healthcare disruption during the COVID-19 pandemic, as some emerging evidence suggests.[13,14]

Harnessing multiple longitudinal studies allows inequalities to be studied in detail by improving statistical power and allows consistency of findings to be investigated. We therefore aimed to investigate inequalities in healthcare disruption during the COVID-19 pandemic in 12 population-based longitudinal studies, to help inform targeting of policy responses as we move out of the acute phase of the pandemic. We investigate healthcare disruptions (including prescription or medication access, procedures or surgery, clinical appointments) by sex, age, ethnicity, education, and occupational social class and we explore whether associations differ by age, or for those who have been recommended to 'shield' due to clinical vulnerability.

Methods

Design

The UK National Core Studies – Longitudinal Health and Wellbeing programme aims to draw together data from multiple UK population-based longitudinal studies to answer questions relevant to the pandemic response. By coordinating analyses within each study and statistically pooling results in a meta-analysis, we can provide robust evidence to understand how the pandemic has impacted population health and support efforts to mitigate its health effects going forward.

Participants

Data were from 12 UK population studies which had conducted surveys both before and during the COVID-19 pandemic. Details of the design, sample frames, current age range, timing of the COVID-19 surveys, response rates, and analytical sample size are available in Supplementary Table S1.

Our population of interest is the current UK population aged 16 years or older. The following studies are considered to be nationally representative samples of their target age-groups: the Millennium

For peer review only - http://bmjopen.bmj.com/site/about/guidelines.xhtml

Cohort Study (MCS);[15] Next Steps (NS);[16] the 1970 British Cohort Study (BCS);[17] the National Child Development Study (NCDS);[18] the National Survey of Health and Development (NSHD); [19,20] Understanding Society (USOC);[21] and the English Longitudinal Study of Ageing (ELSA).[22] We also included the Avon Longitudinal Study of Parents and Children (ALSPAC-G1);[23] the parents of the ALSPAC-G1 cohort which we refer to as ALSPAC-G0;[24] the Born in Bradford study (BIB);[25,26] Generation Scotland: the Scottish Family Health Study (GS);[27] and the UK Adult Twin Registry (TwinsUK).[28,29] We present results from all 12 studies in the main manuscript and results restricted to representative samples in Supplementary File 3.

We can further categorise these studies into age homogenous birth cohorts (where all individuals were of similar age within each cohort) and age heterogeneous studies (each covering a range of age groups). The age homogenous studies include: MCS; ALSPAC-G1; NS; BCS; NCDS; and NSHD. The age heterogenous studies include: BIB; USOC; GS; ALSPAC-G0; TwinsUK; and ELSA. Analytical samples were defined within each study based on respondents who had no missing data on at least one healthcare disruption outcome in a COVID-19 survey and on a minimum set of covariates (sex, ethnicity, and age where relevant). Most studies were weighted to be representative of their target populations accounting for differential non-response.[20,30,31] Weights were not available for BIB or TwinsUK. Studies were ordered for presentation by age of sample (youngest to oldest), with the age homogenous cohorts first, followed by the age heterogenous studies.

Patient and public involvement

Participants were not involved in the design or conduct of our research.

Ethical approval

Ethics statement and data access details for each study can be found in Supplementary Table S2.

Measures

Below we describe the overall approach to measuring each variable in the analysis.

For peer review only - http://bmjopen.bmj.com/site/about/guidelines.xhtml

Outcomes

We assessed self-reported disruptions to prescriptions or medication access; procedures or surgery; and appointments (e.g., with a GP or outpatient services); and a combined variable indicating disruptions to any of the afore mentioned. Any deviation from planned/existing treatment was coded as a disruption, regardless of the reason for the disruption. The wording of the questions was the same for MCS, NS, BCS, NCDS and NSHD. There was variation in how the questions were asked in the other studies. Full details of the questions and coding used within each study are available in Supplementary File 1. ALSPAC did not have information about prescriptions or medication access. BIB did not have information about procedures or surgery. TwinsUK did not have information about procedures or surgery or appointments. Where multiple pandemic survey waves had been included, we coded for any disruptions reported up to and including the most recent. This meant at least 7 months of follow-up for most studies (GS had five and ELSA four, while ALSPAC had the longest follow-up period at nine months).

Indicators of Inequality

We assessed inequalities associated with key sociodemographic characteristics i.e., sex, age, ethnicity, education, and occupational social class. For age, we considered age-groups categorised as: 16-24; 25-34; 35-44; 45-54; 55-64; 65-74; and 75+ years. Depending on the level of detail of ethnicity available, we examined both a binary (White [including White minorities] vs Ethnic minorities [excluding White minorities]) and a finer categorisation of ethnicity (White, South Asian, Black, Mixed, Other Asian, Other Ethnic Minority). For education we distinguished between degree or equivalent; A-level or equivalent (i.e., post-compulsory schooling qualifications); GCSE or equivalent (i.e., qualifications for completing compulsory schooling); and fewer or no qualifications. We also examined occupational class with the following categories (based on different coding schemes in different studies): Professional/Managerial; Intermediate; Routine/Manual; and Other (which included never/long-term non-employed and, in some studies, respondents who could not be classified elsewhere). Where respondents' education and occupational class were not available, we considered parental education or household social class. For full details, see Supplementary File 1.

For peer review only - http://bmjopen.bmj.com/site/about/guidelines.xhtml

Moderators

We decided *a priori* to examine modification by age and clinical vulnerability to COVID-19 to see whether inequalities varied by life-stage or were particularly acute for those with higher healthcare needs and at higher risk from COVID-19 harms. For moderation by age, the age-heterogeneous studies split their samples into the age-bands covered, while age homogeneous cohorts were included within the appropriate age bands (see above for banding). In the UK, clinically extremely vulnerable people were advised to stay at home ('shield') during the pandemic. In this paper, shielding status was based on whether respondents reported having received a letter from the NHS advising them to stay at home to protect themselves. More information about shielding can be found in Supplementary File 1.

Other variables

The following covariates were also included where relevant and available within each study: UK Nation (i.e., England, Scotland, Wales, or Northern Ireland); household composition (based on partnership status and whether there were children in the household); and pre-pandemic self-reported health (good vs poor).

Analysis

Within each study, distributions of sociodemographic characteristics and healthcare disruption were examined. Then, each healthcare disruption outcome was regressed on each indicator of inequality (i.e., sex, age, ethnicity, education, and occupational class). Unadjusted associations are included in Supplementary File 2. Since our aim was primarily to describe inequalities, we focus on presenting associations with minimal adjustment only for sex, age, and ethnicity when applicable. To assess whether associations were independent of other related factors, we also provide results in Supplementary File 2 for any healthcare disruption which additionally adjust for: education, occupational class, UK Nation (where appropriate), household composition, and pre-pandemic self-reported health. Moderation by age and shielding status was assessed using stratified models.

For peer review only - http://bmjopen.bmj.com/site/about/guidelines.xhtml

For peer review only

Results were then meta-analysed for each outcome for the full sample, and within age and shielding strata. We used a random effects meta-analysis with restricted maximum likelihood. For stratified results, a test of group differences was performed using the subgroup meta-analysis command. We report heterogeneity using the I^2 statistic (0% indicates low variation between estimates across studies, while values closer to 100% indicate greater heterogeneity).

Finally, in sensitivity analyses we restricted meta-analyses to representative studies (MCS, NS, BCS, NCDS and NSHD, USOC and ELSA). Meta-analyses were conducted in Stata 16 (V.16).[32]

For peer review only

For peer review only - http://bmjopen.bmj.com/site/about/guidelines.xhtml

Results

Descriptive Statistics

The distribution of demographic and socio-economic characteristics within each study is presented in Table 1. A total of 68,912 participants were included in the coordinated analysis. Due to study design, participants from BIB were all female, as were the vast majority (89.4%) from TwinsUK. The age ranged from 16 years in BIB and USOC to 90+ years in TwinsUK and ELSA.

Overall, the prevalence of any healthcare disruption ranged from 6.4% in TwinsUK to 31.8% in USOC (Figure 1). Table 2 shows that disruptions to medical appointments were most common, ranging from 3.5% (ELSA) to 28.4% (USOC). Disruptions in prescriptions or medication access varied from 1.2% (BIB) to 10.4% (GS). Disruptions to procedures or surgery were least common ranging from 0.7% (MCS) to 16.8% (ELSA).

For peer review only

For peer review only - http://bmjopen.bmj.com/site/about/guidelines.xhtml

Table 1. Percent (and N) distribution of demographic and socio-economic characteristics by study

		MCS	*ALSPAC G1*	*NS*	*BCS70*	*NCDS*	*NSHD*	*BIB*	*USOC*	*GS*	*ALSPAC G0*	*TWINS UK*	*ELSA*
**Total analytic N**		3,147	3,430	3,311	5,175	5,747	1,569	1,726	13,253	17,139	3,625	4,282	6,508
**Female**		65.0 (2,045)	65.3 (2,240)	64.8 (2,145)	57.9 (2,994)	53.7 (3,086)	52.6 (825)	100.0 (1,726)	57.9 (7,668)	67.0 (11,476)	73.1 (2,651)	89.4 (3,830)	56.3 (3,663)
**Mean Age in 2020 (range)**		19.5 (18.7-20.1)	28.4 (27-29)	30.6 (29.9-31.4)	50.5 (50.4-50.6)	62.6 (62.5-62.7)	74	37.5 (16-54)	51.1 (16-96.2)	57.0 (18-100)	59.4 (45-89)	61.2 (22-96)	69.3 (52-90+)
**Ethnicity**	*White*	86.1 (2,708)	98.4 (3,330)	74.6 (2,470)	NA	NA	NA	37.8 (653)	98.3 (16,843)	87.2 (11,561)	98.4 (3,567)	97.1 (4,156)	95.9 (6,239)
	South Asian	7.6 (240)	NA	15.0 (496)	NA	NA	NA	56.1 (968)	0.4 (70)	6.7 (885)	NA	0.7 (28)	2.1 (135)
	East Asian	1.0 (30)	NA	NA	NA	NA	NA	NA	0 .3 (51)	1.2 (155)	NA	0.1 (3)	NA
	Black	2.6 (83)	NA	3.8 (127)	NA	NA	NA	2.0 (34)	0.1 (21)	2.5 (334)	NA	1.1 (45)	1.2 (75)
	Mixed	2.4 (76)	NA	4.6 (152)	NA	NA	NA	1.4 (24)	0.6 (105)	1.8 (241)	NA	0.9 (38)	0.9 (59)
	Other	0.3 (10)	NA	2.0 (66)	NA	NA	NA	2.7 (47)	0.3 (49)	0.6 (77)	NA	0.3 (12)	NA
	All ethnic minorities	13.9 (439)	2.9 (100)	25.4 (841)	NA	NA	NA	62.2 (1,073)	1.3 (226)	12.8 (1692)	1.6 (58)	2.9 (126)	4.1 (269)
**Education**	*Higher Education or Degree*	55.9 (1,758)	29.0 (994)	48.9 (1,620)	46.6 (2,411)	46.0 (2,646)	29.0 (994)	35.1 (556)	50.7 (8,602)	47.1 (6,238)	29.7 (1,075)	55.7 (2,386)	25.6 (1,666)
	A-level or equivalent	15.0 (473)	35.1 (1,203)	23.4 (773)	14.2 (733)	18.0 (1,034)	35.1 (1203)	17.2 (273)	35.9 (6,096)	11.6 (1,543)	29.7 (1,078)	11.6 (498)	27.6 (1,798)
	GCSE or equivalent	19.5 (615)	26.1 (896)	19.0 (628)	23.4 (1,209)	22.8 (1,311)	26.1 (896)	22.3 (354)	6.2 (1046)	25.2 (3,341)	30.3 (1,098)	20.5 (877)	22.3 (1,452)
	<GCSE or none	9.6 (301)	9.83 (337)	8.8 (290)	15.9 (822)	13.2 (756)	9.8 (337)	25.5 (405)	7.2 (1,214)	16.1 (2,131)	10.3 (374)	12.2 (521)	24.5 (1,592)
**Social Class**	*Managerial, Admin, Professional*	51.3 (1,614)	18.0 (616)	47.6 (1,575)	42.7 (2,209)	23.0 (1,319)	18 (616)	31.2 (475)	81.0 (10,716)	35.0 (4,639)	13.4 (486)	NA	32.4 (2,111)
	Intermediate	15.4 (484)	46.2 (1,583)	18.9 (625)	21.1 (1,091)	14.9 (856)	46.1 (1,583)	35.7 (545)	14.4 (1,906)	17.1 (2,264)	41.2 (1,492)	NA	23.0 (1,497)
	Manual/Routine	18.9 (595)	35.3 (1,212)	15.0 (495)	19.5 (1,009)	16.5 (948)	35.3 (1,212)	25.3 (386)	4.4 (581)	20.1 (2,663)	44.6 (1,617)	NA	28.2 (1,834)
	Other	14.4 (454)	0.6 (19)	18.6 (616)	16.7 (866)	45.7 (2,624)	0.6 (19)	7.8 (119)	0.2 (27)	27.8 (3687)	0.8 (30)	NA	16.4 (1,066)
**Instructed to Shield**		2.5 (79)	NA	3.3 (110)	5.2 (267)	6.9 (393)	8.8 (101)	7.6 (131)	6.2 (825)	7.8 (1,332)	NA	5.9 (252)	16.3 (1,062)

For peer review only - http://bmjopen.bmj.com/site/about/guidelines.xhtml

Sources: MCS (Millennium Cohort Study); ALSPAC G1 (Children of the Avon Longitudinal Study of Parents and Children); NS (Next Steps); BCS 70 (1970 British Cohort Study), NCDS (National Child Development Study); NSHD (National Survey of Health and Development); BIB (Born in Bradford); USOC (Understanding Society); GS (Generation Scotland: the Scottish Family Health Study); ALSPAC G0 (parents of ALSPAC); TWINS UK (UK Adult Twin Registry); ELSA (English Longitudinal Study of Ageing). Notes: Studies are ordered by age homogeneity/heterogeneity and mean age of respondents at the time of the interview. Samples for each study restricted to respondents with non-missing information on healthcare disruptions and valid information on sex, social class, education and (where applicable) age and ethnicity. All information about how data were collected and variables were coded is available in Supplementary File 1. NA = Not available/Info not collected. Unweighted data.

For peer review only

For peer review only - http://bmjopen.bmj.com/site/about/guidelines.xhtml

Table 2. Percent prevalence (and 95% confidence intervals) of healthcare disruptions during the pandemic, by study

	MCS	*ALSPAC (G1)*	*NS*	*BCS70*	*NCDS*	*NSHD*	*BIB*	*USOC*	*GS*	*ALSPAC (G0)*	*TWINS UK*	*ELSA*
**Prescription/ medication access**	4.0 (2.3-5.5)	NA	3.8 (2.3-5.3)	3.4 (2.7-4.2)	2.4 (1.8-3.0)	2.2 (1.3-3.8)	1.2 (0.7-1.7)	5.5 (5.0-6.1)	10.4 (9.9-10.9)	NA	2.9 (2.5-3.3)	0.8 (0.6-1.2)
**Procedures or surgery**	0.7 (0.0-1.2)	1.6 (1.2-2.1)	2.1 (0.0-3.8)	1.0 (0.7-1.2)	2.8 (2.0-3.5)	2.5 (1.4-4.4)	NA	12.3 (11.6-13.0)	2.1 (1.9-2.4)	2.9 (2.1-3.9)	NA	16.8 (15.7-17.9)
**Appointments**	6.2 (4.9-7.6)	11.7 (10.3-13.2)	7.3 (5.6-9.0)	10.6 (9.2-12.1)	12.1 (10.9-13.3)	12.0 (9.3-15.6)	8.6 (7.4-10.1)	28.4 (27.4-29.4)	16.6 (16.0-17.1)	14.4 (12.8-16.2)	NA	3.5 (2.9-4.1)
**Any healthcare disruption**	10.1 (8.1-12.1)	15.9 (14.3-17.6)	12.8 (10.3-15.4)	14.3 (12.7-15.9)	16.7 (15.2-18.2)	16.4 (13.2-20.2)	9.4 (8.1-10.9)	31.8 (30.8-32.8)	25.3 (24.6-25.9)	19.9 (18.1-21.9)	6.35 (5.9-7.2)	19.5 (18.3-20.8)

Sources: MCS (Millennium Cohort Study); ALSPAC G1 (Children of the Avon Longitudinal Study of Parents and Children); NS (Next Steps); BCS 70 (1970 British Cohort Study), NCDS (National Child Development Study); NSHD (National Survey of Health and Development); BIB (Born in Bradford); USOC (Understanding Society); GS (Generation Scotland: the Scottish Family Health Study); ALSPAC G0 (parents of ALSPAC); TWINS UK (UK Adult Twin Registry); ELSA (English Longitudinal Study of Ageing). Notes: Studies are ordered by age homogeneity/heterogeneity and mean age of respondents at the time of the interview. Samples for each study restricted to respondents with non-missing information on healthcare disruptions and valid information on sex, social class, education and (where applicable) age and ethnicity. All information about how data were collected and variables were coded is available in Supplementary File 1. TWINSUK had an additional question: "Have you experienced healthcare disruption as a result of the COVID-19 pandemic?" This data was also used to derive the 'any healthcare disruption' variable for TWINSUK. NA = Not available/Info not collected. Weighted data where applicable

For peer review only - http://bmjopen.bmj.com/site/about/guidelines.xhtml

The following sections describe results adjusted for sex, age, and ethnicity when applicable. Unadjusted results and results adjusted for education, occupational class, UK Nation (where appropriate), household composition, and pre-pandemic self-reported health can be found in Supplementary File 2. The associations were largely robust to further adjustment.

Sex and healthcare disruptions

Across all studies females were generally more likely to report any healthcare disruptions than males (Supplementary Table S3 for details).

Pooled results from the meta-analysis demonstrate that females had increased odds of any healthcare disruption compared with males (OR: 1.27 [95%CI: 1.15,1.40]; I^2=54%, figure 2, supplementary file 2). Similar associations were observed for disruptions to appointments (OR: 1.33 [95%CI: 1.17,1.52]; I^2=60%). The association between sex and the less prevalent disruptions to procedures and medications crossed the null (Supplementary File 2 and Figure 2).

There were differences in the association between sex and healthcare disruption when stratified by age (p<0.001. Supplementary File 2). The odds of having any healthcare disruption for females was highest among 16–24-year-olds (OR: 2.22 [95% CI 1.63, 3.02]; I^2=0%, and Supplementary File 2). An association between sex and healthcare disruption was observed up to age 54 years but there were no clear associations among those aged 55 years and above. There was no evidence that the association between sex and healthcare disruption differed by shielding and non-shielding groups (Supplementary File 2).

<Figure 1 and 2 about here>

Age and healthcare disruptions

A higher prevalence of having any healthcare disruption was observed among older participants of the national birth cohorts where the same questionnaire was used (Figure 1). This age difference was also observed among the ALSPAC studies and for other age-heterogenous studies as seen in Supplementary Table S3.

For peer review only

For peer review only - http://bmjopen.bmj.com/site/about/guidelines.xhtml

The meta-analysis including age-heterogenous studies were supportive of age differences for any healthcare disruptions e.g., OR: 1.39 [1.13,1.72]; I^2=77% for 65-75y vs 45-54y (Figure 3, Supplementary File 2). Disruptions seemed less likely in younger age groups and more likely among older age groups, though some estimates cross the null and had high heterogeneity, which may be because of few studies in specific age categories (Figure 3, Supplementary File 2). Associations for disruptions to medical appointments and procedures or surgery showed these age differences more clearly (Figure 3, Supplementary File 2).

There were no clear differences in the association with age and any healthcare disruption by shielding status. However, for those who were shielding, confidence intervals were wide (Supplementary File 2). The magnitude for the association of healthcare disruption among 75-year-olds and above vs 45–54-year-olds was higher among the non-shielding group (OR: 1.61 [95%CI: 1.17,2.22]; I^2=79%) compared with the shielding group (OR: 0.83 [95%CI:0.51,1.37]; I^2=83%, Supplementary File 2).

<Figure 3 about here>

Ethnicity and healthcare disruptions

Among the studies that had data on ethnicity, between 7.8% (BIB) and 31.9% (USOC) of the White groups reported healthcare disruption. Between 8.3% (TWINSUK) and 23.6% (GS) of Ethnic minority groups reported having any healthcare disruption (Supplementary Table S3).

In meta-analysis, Ethnic minorities compared to White groups had increased odds of any healthcare disruption (OR: 1.19 [1.05,1.35]; I^2=0%, Figure 4 and Supplementary File 2). This association was less clear for specific domains of healthcare disruption (Figure 4, Supplementary File 2). Among the studies that had a finer categorisation of ethnicity, only the Black ethnic groups had clearly raised odds for any healthcare disruption compared with White groups (OR: 1.38 [1.03,1.84]; I^2=0%). Associations with healthcare disruption were less evident for other ethnic groups but were imprecisely estimated (Figure 4, Supplementary File 2).

For peer review only

For peer review only - http://bmjopen.bmj.com/site/about/guidelines.xhtml

There were no major differences in associations between ethnicity and any healthcare disruption by age, though this may simply be due to low power as confidence intervals were wide (Supplementary File 2). The clearest associations with Ethnic minority groups were within the 35-44- and 45-74-year age ranges (OR:1.31 [1.01,1.71]; I^2=0% and OR:1.61 [1.16,2.22]; I^2=0%). The mixed ethnicity group were also at particular risk for disruption in the 16–24-year age range too (OR:2.50 [1.25,5.02]; I^2=0%). The magnitude for the association between any healthcare disruption among Ethnic minority groups vs. White groups was higher among those who were shielding (OR: 1.56[1.01 to 2.39]; compared to OR: 1.06[0.86 to 1.31] for non-shielding). This observation was consistent across more granular ethnicity categories, but confidence intervals were wide (Supplementary File 2).

<Figure 4 about here>

Education and healthcare disruptions

There was no clear pattern in the prevalence of healthcare disruption across education levels. For example, in USOC 29.7% of those with any healthcare disruption had a degree or equivalent and 39% had no school-leaving qualifications. In TWINSUK 9.9% of those with any healthcare disruption had a degree or equivalent and 6.1% had no school-leaving (Supplementary Table S3).

In meta-analysis, we did not observe clear associations between education level and healthcare disruption, other than that those without school-leaving qualifications had raised odds of disruptions to procedures or surgery (OR: 1.26 [1.11,1.44]; I^2=0%; Supplementary File 2 and Figure 5). We did not observe differences by age or shielding status (Supplementary File 2).

<Figure 5 about here>

Occupational class and healthcare disruptions

The prevalence of any healthcare disruption ranged between 9.7% (BIB) and 25.7% (USOC) among the Professional/Managerial social class and between 9.3% (BIB) and 27.6% (USOC) for the Manual/Routine social class (Supplementary Table S3).

For peer review only - http://bmjopen.bmj.com/site/about/guidelines.xhtml

Results from meta-analysis show that those in a more disadvantaged occupational class were more likely to report any healthcare disruptions (e.g., OR: 1.17 [95%CI: 1.08, 1.27]; I^2=0% for Manual/Routine compared with Professional/Managerial, Figure 6, Supplementary File 2). The OR was greatest for the non-employed occupational class category (OR: 1.51 [95%CI: 1.12,2.04]), however the I^2 was also large (80%). This implies considerable between study heterogeneity, though two of the four individual studies (MCS and ELSA) that did not show clear associations for this category were at the extremes of the age range considered. Similar associations were seen for domains of healthcare disruption, with the largest inequalities seen for access to medications. We did not observe differences by age or shielding status (Supplementary File 2).

<Figure 6 about here>

Sensitivity analysis

There were no major differences in the results after restricting to representative samples (Supplementary File 3).

For peer review only - http://bmjopen.bmj.com/site/about/guidelines.xhtml

Discussion

Our study demonstrates marked inequalities in healthcare disruption during the COVID-19 pandemic by harnessing data from 12 UK longitudinal studies. Females were more likely to report healthcare disruptions than males, especially at younger ages (<55 years). This inequality was observed for each healthcare disruption type including prescription medication, procedures or surgery, and appointments as well as a combined measure for any of these disruptions. Older adults were especially likely to report disruptions to medical appointments and procedures and surgeries compared to their younger counterparts. Ethnic minority (excluding White minorities) groups were more likely to report healthcare disruption compared to White (including White minorities) groups. Furthermore, when stratifying results by shielding status, the magnitude for the association between any healthcare disruption among Ethnic minority groups (compared to White groups) was higher among those who were shielding. In studies where a finer breakdown of ethnicity was possible, Black ethnic minority groups had the most clearly increased odds of disruption compared to White ethnic groups. Occupational class was also found to be associated with healthcare disruption with those in a routine/manual occupation or other (which included never/long-term non-employed) being more likely to experience healthcare disruption than those in a managerial/professional occupation. No clear association between education and healthcare disruption was found in the main, age or shielding status stratified analyses.

The direct burden of COVID-19 on health services across the globe has been colossal and remains so in some countries, with prioritisation of COVID-19 patients, leaving less capacity and resources for non-COVID-19 healthcare. Furthermore, associated repeated lockdown measures are also likely to decrease healthcare access and availability with a decrease in the number of people attending A&E services,[4] and reports of difficulties accessing medication.[9]

Our findings are consistent with current evidence from a smaller sub-set of the studies examined here suggesting that females are more likely to experience disruption to planned surgery, medical procedures, or other medical appointments during lockdown.[13] Furthermore, our results show

For peer review only - http://bmjopen.bmj.com/site/about/guidelines.xhtml

that older adults were more likely to report healthcare disruption as compared to their younger counterparts, especially disruptions to medical appointments and planned procedures or surgeries. This finding is consistent with current UK evidence indicating that older adults experience more delays and disruption to health services.[33–36] Black ethnic minority groups were also found to be at increased risk of healthcare disruption compared to white ethnic groups – an issue of particular concern given pre-pandemic ethnic inequalities in healthcare.[37] The inequalities by occupational class we found are consistent with prior evidence of socioeconomic healthcare inequalities reported in the UK in the past decade,[38] and highlight that these have still been present in the COVID-19 pandemic. Associations with occupational class were clearer than those for education, which is also an indicator of socioeconomic position but may have been a more distal influence.

Strengths and limitations

The analysis brings together data from 12 longitudinal studies with rich and sensitive information on healthcare disruption. This study is strengthened by the coordinated investigation in multiple longitudinal studies with differing study designs, different target populations, and varying selection and attrition processes. Our combined approach provides the largest sample size available to prospectively investigate differences between ethnic groups, within representative population-based samples. What's more, though utilising non-response weights available, the proportion of ethnic minority groups within some of the studies is representative of the UK population. Moreover, the use of multiple studies increased statistical power to look at subpopulations such as ethnic minority groups across cohorts and allowed for greater examination of how inequalities were patterned by age. While not all 12 studies were representative of the population of interest, removing them in sensitivity analyses did not change our conclusions. Our novel approach to coordinated analyses harnessing multiple datasets therefore allowed research questions to be addressed which would not otherwise be possible.

Differences between studies in a range of factors including measurement of healthcare disruption, timing of surveys, design, response rates, and differential selection into the COVID-19 sweeps are potentially responsible for heterogeneity in estimates. However, despite this heterogeneity,

For peer review only - http://bmjopen.bmj.com/site/about/guidelines.xhtml

the key findings were consistent across most datasets. Furthermore, this heterogeneity can be informative, for example, by virtue of mixing age-specific and age range studies, we identified that sex inequalities were stronger at younger ages, and inequalities by occupational class were concentrated within working ages. The definition of healthcare disruption used may also have contained a range of disruptions of greater or lesser severity, and there may have been further inequalities in the severity of disruptions experienced, however we were not able to assess this using the available data.

We have focused on our aim of identifying who experienced greater disruptions in healthcare, rather than on adjustment for confounders to estimate causal effects of the exposures in question.[39] Nevertheless, many of the associations we observed were robust to adjustment for a wider range of related variables, but bias due to residual confounding cannot be ruled out. Importantly, we did not condition our analyses on healthcare need. Many of the inequalities we observed for healthcare disruptions may be due to inequalities in health, with those who have greater health needs being more likely to require healthcare that could be disrupted. Accounting for differences in need could have masked inequalities in healthcare disruptions that are caused by inequalities in health and could have made it less clear which groups have been more likely to experience disruption during the pandemic. Restricting analyses to those who needed care could also induce bias if there were unmeasured determinants of both need and disruption.[40] Nevertheless, another study of the USOC data analysed here that did restrict analyses to those needing care still found income-related inequalities in healthcare disruption, and most of the associations we observed were robust to adjustment for pre-pandemic self-assessed health.[41]

Impact of healthcare disruption

Disadvantaged groups such as females, older adults, Black ethnic minority groups, and those in routine/manual occupations have had elevated odds of healthcare disruption in the first 8-10 months of the COVID-19 pandemic. Delays and disruptions to treatment could have ongoing implications for patients' physical and mental health.[42] Action is needed to remedy these inequalities, and efforts to ensure continuity of care during pandemic-related disruptions may need to be more clearly targeted to those who most need that care. As healthcare access resumes, given the forgone delays in treatments

For peer review only - http://bmjopen.bmj.com/site/about/guidelines.xhtml

and the subsequent backlog of postponed surgeries,[43] these groups may require prioritised support to address unmet needs experienced during the pandemic.

Conclusion

There have been clear inequalities in disruptions to healthcare during the COVID-19 pandemic in the UK. Females (especially at younger ages), older adults, ethnic minorities, and those in disadvantaged occupational classes have been more likely to experience healthcare disruptions. These are groups who usually experience worse health, so disruptions related to COVID-19 have clear potential to maintain or even exacerbate existing health inequalities.

For peer review only

Author contribution statement:

Katikireddi, Ploubidis, Maddock, Parsons, Di Gessa, Green and Thompson conceptualised the study and design. Katikireddi, Ploubidis, Maddock Parsons, Di Gessa, Green, Thompson, Silverwood designed the methodology, Maddock, Parsons, Di Gessa, Green, Thompson, Stevenson, Kwong, McElroy and Santorelli conducted the formal analysis. Maddock, Parsons, Di Gessa, Green, Thompson drafted the manuscript. All authors contributed to critical revision and provided final approval of the manuscript. The project was supervised by Ploubidis and Katikireddi. Funding was acquired by Patalay, Katikireddi, Ploubidis, Silverwood, and Chaturvedi.

Competing interests:

No conflicts of interest were declared by JM, SP, GDG, MJG, EJT, AJS, GS, RJS, GC, NC, AS, ASFK, CJS, PP, GBP. SVK is a member of the Scientific Advisory Group on Emergencies subgroup on ethnicity and COVID-19 and is co-chair of the Scottish Government's Ethnicity Reference Group on COVID-19. NC serves on a data safety monitoring board for trials sponsored by Astra-Zeneca.

Funding:

This work was supported by the National Core Studies, an initiative funded by UKRI, NIHR and the Health and Safety Executive. The COVID-19 Longitudinal Health and Wellbeing National Core Study was funded by the Medical Research Council (MC_PC_20030).

Understanding Society is an initiative funded by the Economic and Social Research Council and various Government Departments, with scientific leadership by the Institute for Social and Economic Research, University of Essex, and survey delivery by NatCen Social Research and Kantar Public. The Understanding Society COVID-19 study is funded by the Economic and Social Research Council (ES/K005146/1) and the Health Foundation (2076161). The research data are distributed by the UK Data Service.

The Millennium Cohort Study, Next Steps, British Cohort Study 1970 and National Child Development Study 1958 are supported by the Centre for Longitudinal Studies, Resource Centre 2015-20 grant (ES/M001660/1) and a host of other co-funders. The 1946 NSHD cohort is hosted by the MRC Unit for Lifelong Health and Ageing funded by the Medical Research Council (MC_UU_00019/1 Theme 1: Cohorts and Data Collection). The COVID-19 data collections in these five cohorts were funded by the UKRI grant Understanding the economic, social and health impacts of COVID-19 using lifetime data: evidence from 5 nationally representative UK cohorts (ES/V012789/1).

The English Longitudinal Study of Ageing was developed by a team of researchers based at University College London, NatCen Social Research, the Institute for Fiscal Studies, the University of Manchester and the University of East Anglia. The data were collected by NatCen Social Research. The funding is

For peer review only

currently provided by the National Institute on Aging in the US, and a consortium of UK government departments coordinated by the National Institute for Health Research. Funding has also been received by the Economic and Social Research Council. The English Longitudinal Study of Ageing Covid-19 Sub-study was supported by the UK Economic and Social Research Grant (ESRC) ES/V003941/1.

The UK Medical Research Council and Wellcome (Grant Ref: 217065/Z/19/Z) and the University of Bristol provide core support for ALSPAC. A comprehensive list of grants funding is available on the ALSPAC website (http://www.bristol.ac.uk/alspac/external/documents/grant-acknowledgements.pdf). We are extremely grateful to all the families who took part in this study, the midwives for their help in recruiting them, and the whole ALSPAC team, which includes interviewers, computer and laboratory technicians, clerical workers, research scientists, volunteers, managers, receptionists and nurses. Please note that the study website contains details of all the data that is available through a fully searchable data dictionary and variable search tool" and reference the following webpage: http://www.bristol.ac.uk/alspac/researchers/our-data/. Ethical approval for the study was obtained from the ALSPAC Ethics and Law Committee and the Local Research Ethics Committees

TwinsUK receives funding from the Wellcome Trust (WT212904/Z/18/Z), the National Institute for Health Research (NIHR) Biomedical Research Centre based at Guy's and St Thomas' NHS Foundation Trust and King's College London. TwinsUK is also supported by the Chronic Disease Research Foundation and Zoe Global Ltd. The funders had no role in study design, data collection and analysis, decision to publish, or preparation of the manuscript.

Generation Scotland received core support from the Chief Scientist Office of the Scottish Government Health Directorates [CZD/16/6] and the Scottish Funding Council [HR03006]. Genotyping of the GS:SFHS samples was carried out by the Genetics Core Laboratory at the Wellcome Trust Clinical Research Facility, Edinburgh, Scotland and was funded by the Medical Research Council UK and the Wellcome Trust (Wellcome Trust Strategic Award "STratifying Resilience and Depression Longitudinally" (STRADL) Reference 104036/Z/14/Z). Generation Scotland is funded by the Wellcome Trust (216767/Z/19/Z).

Born in Bradford (BiB) receives core infrastructure funding from the Wellcome Trust (WT101597MA), and a joint grant from the UK Medical Research Council (MRC) and UK Economic and Social Science Research Council (ESRC) (MR/N024397/1) and one from the British Heart Foundation (BHF) (CS/16/4/32482). The National Institute for Health Research Yorkshire and Humber ARC, and Clinical Research Network both provide support for BiB research.

SVK acknowledges funding from a NRS Senior Clinical Fellowship (SCAF/15/02), the Medical Research Council (MC_UU_00022/2) and the Scottish Government Chief Scientist Office (SPHSU17). ASFK acknowledges funding from the ESRC (ES/V011650/1). DJP acknowledges funding from the Wellcome Trust (216767/Z/19/Z and 221574/Z/20/Z). EJT acknowledges funding

For peer review only - http://bmjopen.bmj.com/site/about/guidelines.xhtml

from the Wellcome Trust (WT212904/Z/18/Z). GBP acknowledges funding from the Economic and Social Research Council (ES/V012789/1).

Acknowledgments

The contributing studies have been made possible because of the tireless dedication, commitment and enthusiasm of the many people who have taken part. We would like to thank the participants and the numerous team members involved in the studies including interviewers, technicians, researchers, administrators, managers, health professionals and volunteers including:

Generation Scotland: Drew Altschul, Chloe Fawns-Ritchie, Archie Campbell, Robin Flaig.
ALSPAC: Daniel J Smith, Nicholas J Timpson, Kate Northstone.
Understanding Society: Michaela Benzeval
TwinsUK: Deborah Hart, María Paz García, Rachel Horsfall, Ruth C.E. Bowyer.
Centre for Longitudinal Studies: Matt Brown, Lisa Calderwood, Emla Fitzsimons, Alissa Goodman, Aida Sanchez
NSHD: Andrew Wong, Maria Popham, Karen MacKinnon, Imran Shah, Philip Curran

We are additionally grateful to our funders for their financial input and support in making this research happen.

For peer review only

Ethics and data access statement

**NSHD, NCDS, BCS70, NS and MCS**	The most recent sweeps of the **NSHD, NCDS, BCS70**, **Next Steps** and **MCS** have all been granted ethical approval by the National Health Service (NHS) Research Ethics Committee and all participants have given informed consent. Data for NCDS (SN 6137), BCS70 (SN 8547), Next Steps (SN 5545), MCS (SN 8682) and all four COVID-19 surveys (SN 8658) are available through the UK Data Service. NSHD data are available on request to the NSHD Data Sharing Committee. Interested researchers can apply to access the NSHD data via a standard application procedure. Data requests should be submitted to mrclha.swiftinfo@ucl.ac.uk; further details can be found at http://www.nshd.mrc.ac.uk/data.aspx. doi:10.5522/NSHD/Q101; doi:10.5522/NSHD/Q10.
**ALSPAC**	Ethical approval was obtained from the **ALSPAC** Ethics and Law Committee and the Local Research Ethics Committees. The study website contains details of all the data that is available through a fully searchable data dictionary and variable search tool: http://www.bristol.ac.uk/alspac/researchers/our-data. ALSPAC data is available to researchers through an online proposal system. Information regarding access can be found on the ALSPAC website (http://www.bristol.ac.uk/media-library/sites/alspac/documents/researchers/data-access/ALSPAC_Access_Policy.pdf).
**BIB**	Ethical approval for **Born in Bradford** was granted by the National Health Service Health Research Authority Yorkshire and the Humber (Bradford Leeds) Research Ethics Committee (reference: 16/YH/0320). Data from the various BiB family studies are available to researchers; see the study website for information on how to access data (https://borninbradford.nhs.uk/research/how-to-access-data/).
**USOC**	The University of Essex Ethics Committee has approved all data collection for the **Understanding Society** main study and COVID-19 waves. No additional ethical approval was necessary for this secondary data analysis. All data are available through the UK Data Service (SN 6614 and SN 8644).
**ELSA**	Waves 1-9 of **ELSA** were approved through the National Research Ethics Service, while the COVID-19 Sub-study was approved by the UCL Research Ethics Committee. All participants provided informed consent. All data are available through the UK Data Service (SN 8688 and 5050).
**GS**	**Generation Scotland** obtained ethical approval from the East of Scotland Committee on Medical Research Ethics (on behalf of the National Health Service). Reference number 20/ES/0021. Access to data is approved by the Generation Scotland Access Committee. See https://www.ed.ac.uk/generation-scotland/for-researchers/access or email access@generationscotland.org for further details.
**TWINSUK**	All wave of **TwinsUK** have received ethical approval associated with TwinsUK Biobank (19/NW/0187), TwinsUK (EC04/015) or Healthy Ageing Twin Study (H.A.T.S) (07/H0802/84) studies from NHS Research Ethics Committees at the Department of Twin Research and Genetic Epidemiology, King's College London. The TwinsUK Resource Executive Committee (TREC) oversees management, data sharing and collaborations involving the TwinsUK registry (for further details see https://twinsuk.ac.uk/resources-for-researchers/access-our-data/).

For peer review only - http://bmjopen.bmj.com/site/about/guidelines.xhtml

References

1. Boserup B, Mckenney M, Elkbuli A. The impact of the COVID-19 pandemic on emergency department visits and patient safety in the United States. *Am J Emerg Med*. 2020;38(January):1732-1736.

2. Vuma CD, Manganyi J, Wilson K, Rees D. The Effect on Fit of Multiple Consecutive Donning and Doffing of N95 Filtering Facepiece Respirators. *Ann Work Expo Heal*. 2019;63(8):930-936. doi:10.1093/annweh/wxz060

3. Salerno R, Conti CB, De Silvestri A, Campbell Davies SE, Mezzina N, Ardizzone S. The impact of covid-19 pandemic on urgent endoscopy in Italy: a nation-wide multicenter study. *Scand J Gastroenterol*. 2020;55(7):870-876. doi:10.1080/00365521.2020.1782466

4. Kelly E, Firth Z. How is COVID-19 changing the use of emergency care by region? The Health Foundation. Published 2020. https://www.health.org.uk/news-and-comment/charts-and-infographics/how-is-covid-19-changing-the-use-of-emergency-care-by-region

5. Warner M, Burn S, Stoye G, Aylin PP, Bottle A, Propper C. Socioeconomic deprivation and ethnicity inequalities in disruption to NHS hospital admissions during the COVID-19 pandemic: a national observational study. *BMJ Qual Saf*. Published online 2021:bmjqs-2021-013942. doi:10.1136/bmjqs-2021-013942

6. Lazzerini M, Barbi E, Apicella A, Marchetti F, Cardinale F, Trobia G. Delayed access or provision of care in Italy resulting from fear of COVID-19. *Lancet Child Adolesc Heal*. 2020;4(5):e10-e11. doi:10.1016/S2352-4642(20)30108-5

7. Association BM. Pressure points in the NHS. British Medical Association. Published 2021. https://www.bma.org.uk/advice-and-support/nhs-delivery-and-workforce/pressures/pressure-points-in-the-nhs

8. Cheong JLY, Goh ZHK, Marras C, Tanner CM, Kasten M, Noyce AJ. The Impact of COVID-19 on Access to Parkinson's Disease Medication. *Mov Disord*. 2020;35(12):2129-2133. doi:10.1002/mds.28293

9. Maldonado D, Tu E, Mahmood S, et al. Medication access difficulty and COVID-related distress are associated with disease flares in rheumatology patients during the COVID-19 pandemic. *Arthritis Care Res (Hoboken)*. Published online 2020:0-3. doi:10.1002/acr.24531

10. Bleich SN, Jarlenski MP, Bell CN, Laveist TA. Health inequalities: Trends, progress, and policy. *Annu Rev Public Health*. 2012;33:7-40. doi:10.1146/annurev-publhealth-031811-124658

11. Immergut EM, Schneider SM. Is it unfair for the affluent to be able to purchase "better" healthcare? Existential standards and institutional norms in healthcare attitudes across 28 countries. *Soc Sci Med*. 2020;267(June):113146. doi:10.1016/j.socscimed.2020.113146

12. Tudor Hart J. the Inverse Care Law. *Lancet*. 1971;297(7696):405-412. doi:10.1016/S0140-6736(71)92410-X

13. Topriceanu CC, Wong A, Moon JC, et al. Evaluating access to health and care services during lockdown by the COVID-19 survey in five UK national longitudinal studies. *BMJ Open*. 2021;11(3). doi:10.1136/bmjopen-2020-045813

14. lnstitute for Fiscal Studies. COVID-19 and disruptions to the health and social care of older people in England. Published online 2020:1-21.

15. Joshi HE, Fitzsimons E. The UK Millennium Cohort Study: the making of a multi- purpose

For peer review only - http://bmjopen.bmj.com/site/about/guidelines.xhtml

resource for social science and policy in the UK. *Longit Life Course Stud*. 2016;7(4):409-430. doi:10.14301/llcs.v7i4.416

16. Calderwood L, Sanchez C. Next Steps (formerly known as the Longitudinal Study of Young People in England). Published online 2016:2-4.

17. Elliott J, Shepherd P. Cohort profile: 1970 British Birth Cohort (BCS70). *Int J Epidemiol*. 2006;35(4):836-843. doi:10.1093/ije/dyl174

18. Power C, Elliott J. Cohort profile: 1958 British birth cohort (National Child Development Study). *Int J Epidemiol*. 2006;35(1):34-41. doi:10.1093/ije/dyi183

19. Wadsworth M, Kuh D, Richards M, Hardy R. Cohort profile: The 1946 National Birth Cohort (MRC National Survey of Health and Development). *Int J Epidemiol*. 2006;35(1):49-54. doi:10.1093/ije/dyi201

20. Brown M, Goodman A, Peters A, et al. COVID-19 Survey in Five National Longitudinal Studies: Wave 1 User Guide (Version 1). *UCL Cent Longitud Stud MRC Unit Lifelong Heal Ageing London, UK*. 2020;(December):1-62. https://cls.ucl.ac.uk/wp-content/uploads/2021/01/UCL-Cohorts-COVID-19-Survey-user-guide.pdf

21. University of Essex, Institute for Social and Economic Research, NatCen Social Research KP. Understanding Society: Waves 1-9, 2009-2019 and Harmonised BHPS: Waves 1-18, 1991-2009. [data collection].

22. Steptoe A, Breeze E, Banks J, Nazroo J. Cohort profile: The English Longitudinal Study of Ageing. *Int J Epidemiol*. 2013;42(6):1640-1648. doi:10.1093/ije/dys168

23. Boyd A, Golding J, Macleod J, et al. Cohort profile: The 'Children of the 90s'-The index offspring of the avon longitudinal study of parents and children. *Int J Epidemiol*. 2013;42(1):111-127. doi:10.1093/ije/dys064

24. Fraser A, Macdonald-wallis C, Tilling K, et al. Cohort Profile : The Avon Longitudinal Study of Parents and Children : ALSPAC mothers cohort. 2013;(April 2012):97-110. doi:10.1093/ije/dys066

25. Wright J, Small N, Raynor P, et al. Cohort profile: The born in bradford multi-ethnic family cohort study. *Int J Epidemiol*. 2013;42(4):978-991. doi:10.1093/ije/dys112

26. Dickerson J, Bird PK, McEachan RRC, et al. Born in Bradford's Better Start: An experimental birth cohort study to evaluate the impact of early life interventions. *BMC Public Health*. 2016;16(1):1-14. doi:10.1186/s12889-016-3318-0

27. Smith BH, Campbell A, Linksted P, et al. Cohort profile: Generation scotland: Scottish family health study (GS: SFHS). The study, its participants and their potential for genetic research on health and illness. *Int J Epidemiol*. 2013;42(3):689-700. doi:10.1093/ije/dys084

28. Suthahar A, Sharma P, Hart D, et al. TwinsUK COVID-19 personal experience questionnaire (CoPE): wave 1 data capture April-May 2020 [version 1 ; peer review : awaiting peer review]. 2021;(May 2020):1-10.

29. Verdi S, Abbasian G, Bowyer RCE, et al. TwinsUK: The UK Adult Twin Registry Update. *Twin Res Hum Genet*. 2019;(May 2007):1-7. doi:10.1017/thg.2019.65

30. Addario G, Dangerfield P, Hussey D, Pacchiotti B, Wood M. *Adapting Fieldwork during the COVID-19 Outbreak A Methodological Overview of the ELSA COVID-19 Substudy (Wave 1).*; 2020.

31. *Institute for Social and Economic Research. Understanding Society COVID-19 User Guide.*; 2021. doi:10.1007/978-1-349-15392-3

For peer review only - http://bmjopen.bmj.com/site/about/guidelines.xhtml

32. Press S. Stata Statistical Software: Release 16. StataCorp LLC.

33. Mann DM, Chen J, Chunara R, Testa PA, Nov O. COVID-19 transforms health care through telemedicine: Evidence from the field. *J Am Med Informatics Assoc*. 2020;27(7):1132-1135. doi:10.1093/jamia/ocaa072

34. Beaunoyer E, Dupéré S, Guitton MJ. COVID-19 and digital inequalities: Reciprocal impacts and mitigation strategies. *Comput Human Behav*. 2020;111(April). doi:10.1016/j.chb.2020.106424

35. Crawford A, Serhal E. Digital health equity and COVID-19: The innovation curve cannot reinforce the social gradient of health. *J Med Internet Res*. 2020;22(6):1-5. doi:10.2196/19361

36. Kojima G, Liljas AEM, Iliffe S. Frailty syndrome: Implications and challenges for health care policy. *Risk Manag Healthc Policy*. 2019;12:23-30. doi:10.2147/RMHP.S168750

37. Katikireddi SV, Cezard G, Bhopal RS, et al. Assessment of health care, hospital admissions, and mortality by ethnicity: population-based cohort study of health-system performance in Scotland. *Lancet Public Heal*. 2018;3(5):e226-e236. doi:10.1016/S2468-2667(18)30068-9

38. Cookson R, Propper C, Asaria M, Raine R. Socio-Economic Inequalities in Health Care in England. *Fisc Stud*. 2016;37(3-4):371-403. doi:10.1111/j.1475-5890.2016.12109

39. Conroy S, Murray EJ. Let the question determine the methods: descriptive epidemiology done right. *Br J Cancer*. 2020;123(9):1351-1352. doi:10.1038/s41416-020-1019-z

40. Cole SR, Platt RW, Schisterman EF, et al. Illustrating bias due to conditioning on a collider. *Int J Epidemiol*. 2010;39(2):417-420. doi:10.1093/ije/dyp334

41. Davillas A, Jones AM. Unmet health care need and income-Related horizontal equity in use ofhealth care during the COVID-19 pandemic. *Health Econ*. 2021;(December 2020):1-6. doi:10.1002/hec.4282

42. Barach P, Fisher SD. Disruption of healthcare: Will the COVID pandemic worsen non-COVID outcomes and disease outbreaks? *Prog Pediatr Cardiol*. 2020;(January).

43. Nepogodiev D, Omar OM, Glasbey JC, et al. Elective surgery cancellations due to the COVID-19 pandemic: global predictive modelling to inform surgical recovery plans. *Br J Surg*. 2020;107(11):1440-1449. doi:10.1002/bjs.11746

For peer review only - http://bmjopen.bmj.com/site/about/guidelines.xhtml

Figure Legends:

Figure 1. Prevalence (and 95% CIs) of any healthcare disruption by study
Sources: MCS (Millennium Cohort Study); ALSPAC G1 (Children of the Avon Longitudinal Study of Parents and Children); NS (Next Steps); BCS 70 (1970 British Cohort Study), NCDS (National Child Development Study); NSHD (National Survey of Health and Development); BIB (Born in Bradford); USOC (Understanding Society); GS (Generation Scotland: the Scottish Family Health Study); ALSPAC G0 (parents of ALSPAC); TWINS UK (UK Adult Twin Registry); ELSA (English Longitudinal Study of Ageing). Notes: Studies are ordered by age homogeneity/heterogeneity and mean age of respondents at the time of the interview. Samples for each study restricted to respondents with non-missing information on healthcare disruptions and valid information on sex, social class, education and (where applicable) age and ethnicity. All information about how data were collected and variables were coded is available in Supplementary File 1.

Figure 2: Associations between female (compared to male) sex and healthcare disruption. Notes: Adjusted for age and ethnicity where applicable.

Figure 3: Associations between age (compared to 45-54 year olds) and healthcare disruption. Notes: Adjusted for sex and ethnicity where applicable.

Figure 4: Associations between Ethnicity (compared to White groups) and healthcare disruption. Notes: Panels illustrate findings for some larger ethnic groups separately and the final panel presents results for all non-White ethnic minorities combined. Adjusted for age and sex where applicable.

Figure 5: Associations between education (compared to degree level) and healthcare disruption Notes: Adjusted for age, sex and ethnicity where applicable.

Figure 6: Associations between occupational social class (compared to Professional/Managerial) and healthcare disruption. Notes: Adjusted for age, sex and ethnicity where applicable.

For peer review only

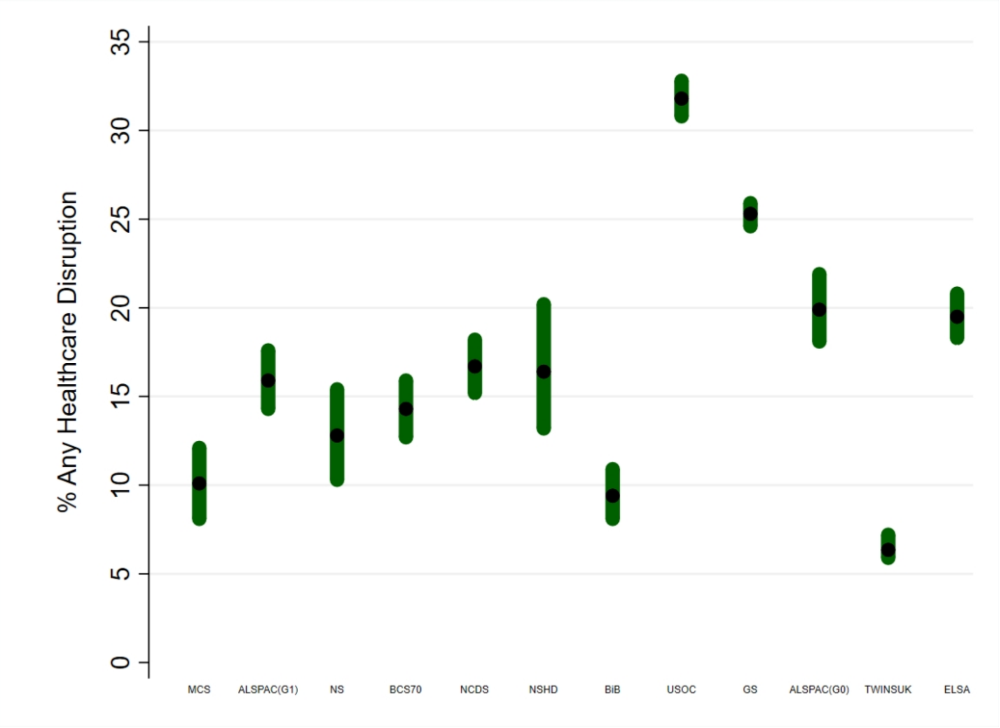

Figure 1. Prevalence (and 95% CIs) of any healthcare disruption by study
Sources: MCS (Millennium Cohort Study); ALSPAC G1 (Children of the Avon Longitudinal Study of Parents and Children); NS (Next Steps); BCS 70 (1970 British Cohort Study), NCDS (National Child Development Study); NSHD (National Survey of Health and Development); BIB (Born in Bradford); USOC (Understanding Society); GS (Generation Scotland: the Scottish Family Health Study); ALSPAC G0 (parents of ALSPAC); TWINS UK (UK Adult Twin Registry); ELSA (English Longitudinal Study of Ageing). Notes: Studies are ordered by age homogeneity/heterogeneity and mean age of respondents at the time of the interview. Samples for each study restricted to respondents with non-missing information on healthcare disruptions and valid information on sex, social class, education and (where applicable) age and ethnicity. All information about how data were collected and variables were coded is available in Supplementary File 1.

416x303mm (72 x 72 DPI)

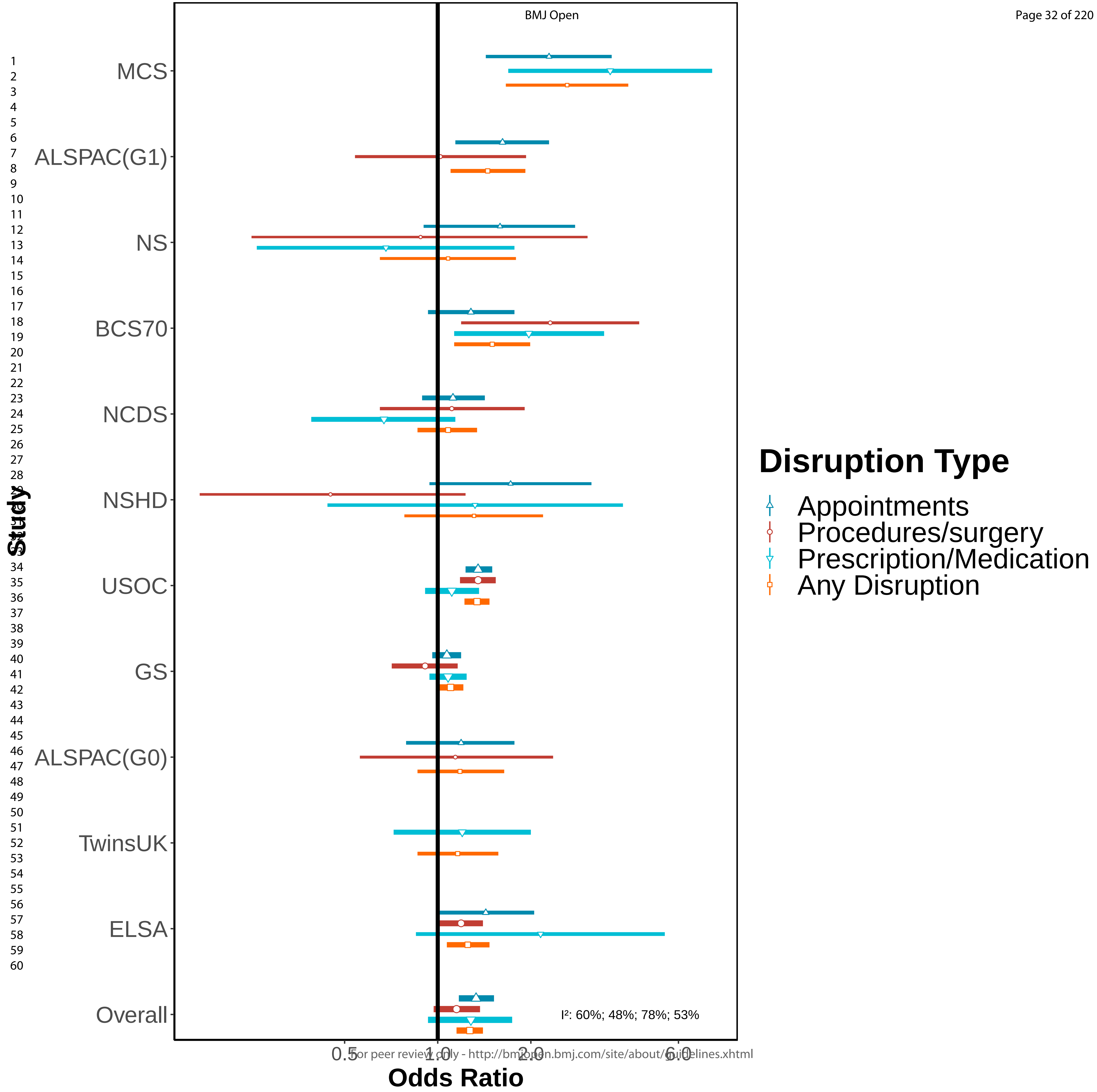
Study
MCS
ALSPAC(G1)
NS
BCS70
NCDS
NSHD
USOC
GS
ALSPAC(G0)
TwinsUK
ELSA
Overall
I²: 60%; 48%; 78%; 53%
0.5
1.0
2.0
6.0
Odds Ratio
Disruption Type
Appointments
Procedures/surgery
Prescription/Medication
Any Disruption

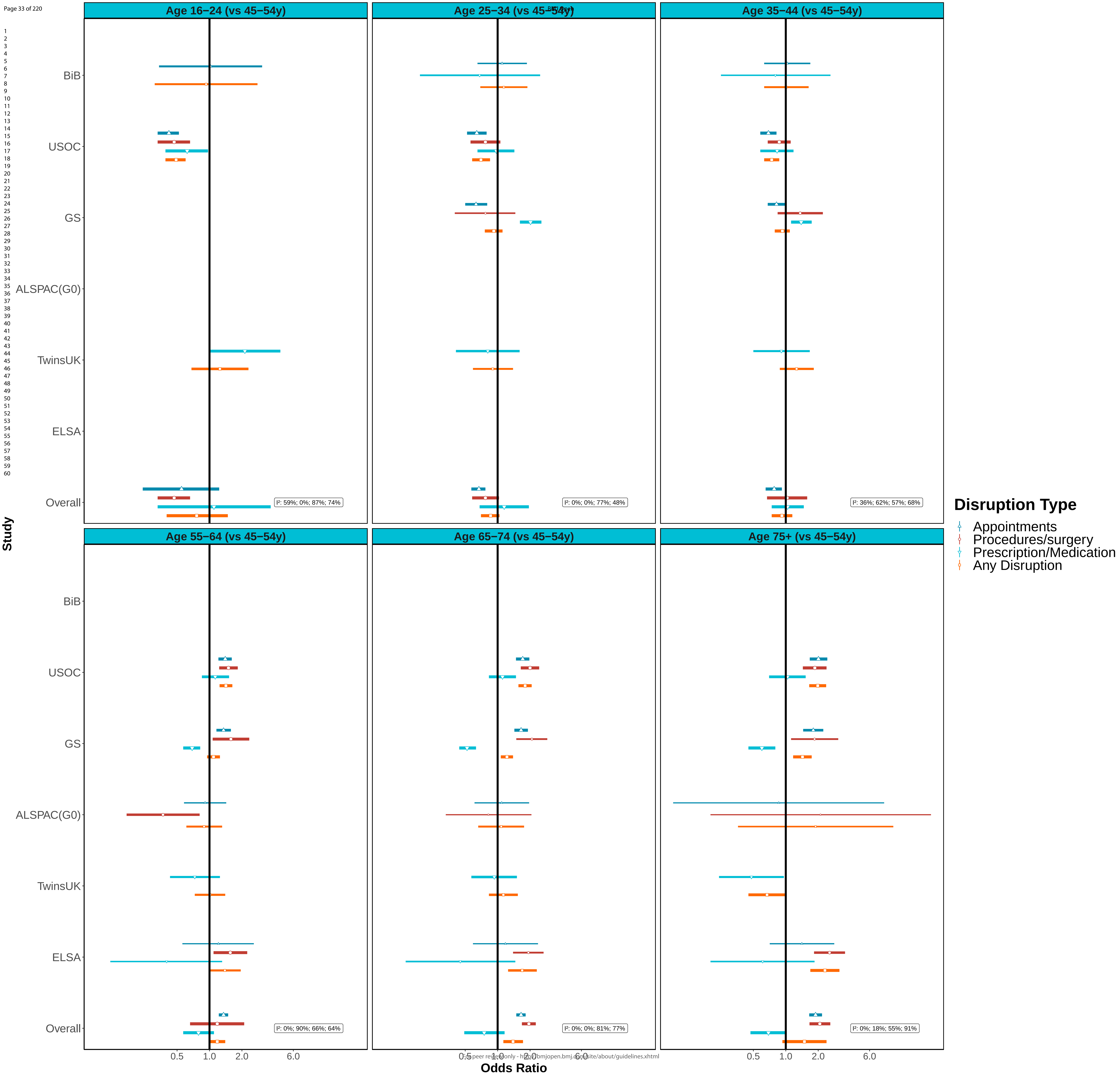

Age 16–24 (vs 45–54y)
Age 25–34 (vs 45–54y)
Age 35–44 (vs 45–54y)
Age 55–64 (vs 45–54y)
Age 65–74 (vs 45–54y)
Age 75+ (vs 45–54y)
BiB
USOC
GS
ALSPAC(G0)
TwinsUK
ELSA
Overall
I²: 59%; 0%; 87%; 74%
I²: 0%; 0%; 77%; 48%
I²: 36%; 62%; 57%; 68%
I²: 0%; 90%; 66%; 64%
I²: 0%; 0%; 81%; 77%
I²: 0%; 18%; 55%; 91%
Study
0.5
1.0
2.0
6.0
Odds Ratio
Disruption Type
Appointments
Procedures/surgery
Prescription/Medication
Any Disruption

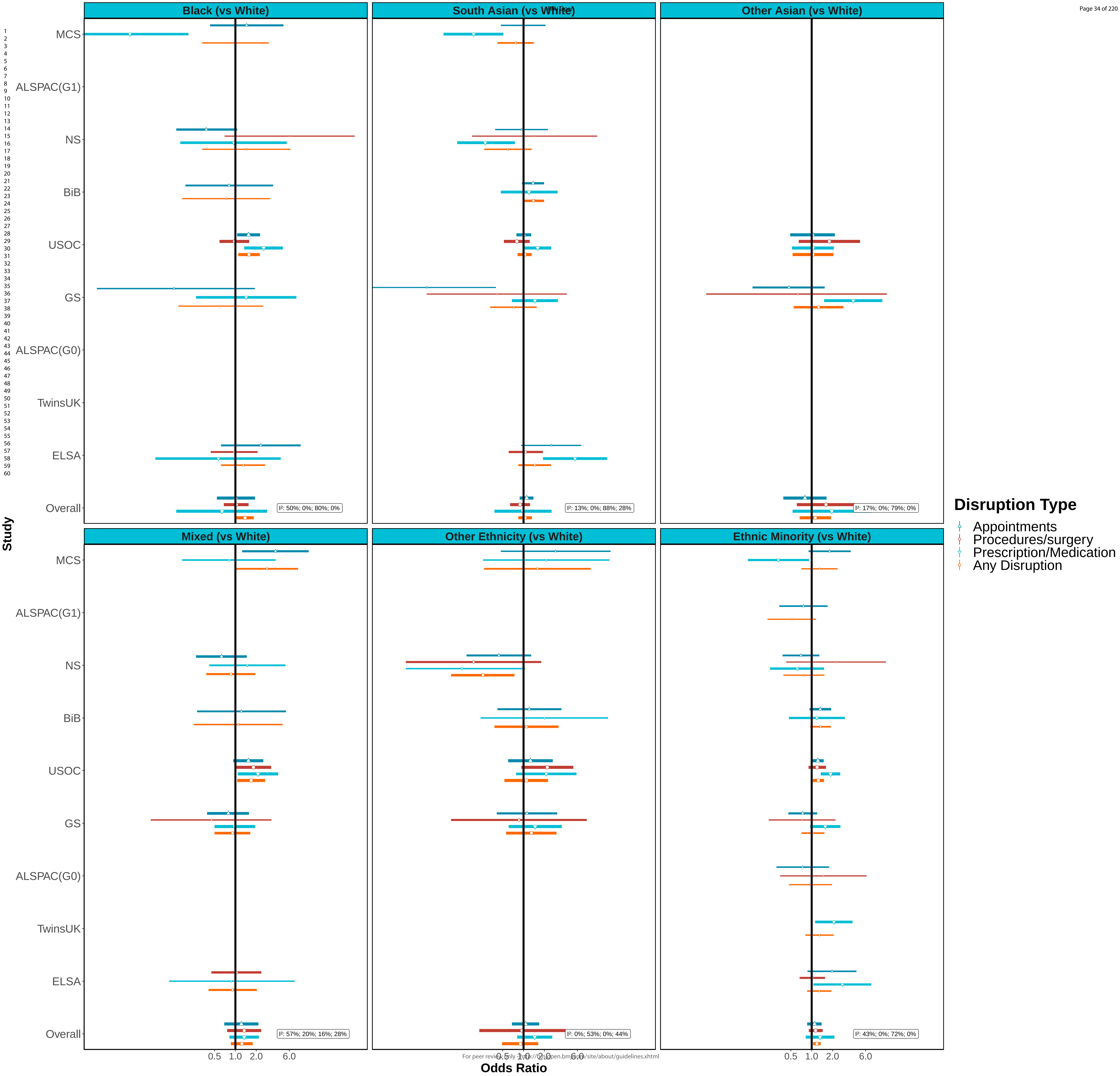
Black (vs White)
South Asian (vs White)
Other Asian (vs White)
Mixed (vs White)
Other Ethnicity (vs White)
Ethnic Minority (vs White)
MCS
ALSPAC(G1)
NS
BiB
USOC
GS
ALSPAC(G0)
TwinsUK
ELSA
Overall
Study
I²: 50%; 0%; 80%; 0%
I²: 13%; 0%; 88%; 28%
I²: 17%; 0%; 79%; 0%
I²: 57%; 20%; 16%; 28%
I²: 0%; 53%; 0%; 44%
I²: 43%; 0%; 72%; 0%
0.5
1.0
2.0
6.0
Odds Ratio
Disruption Type
Appointments
Procedures/surgery
Prescription/Medication
Any Disruption

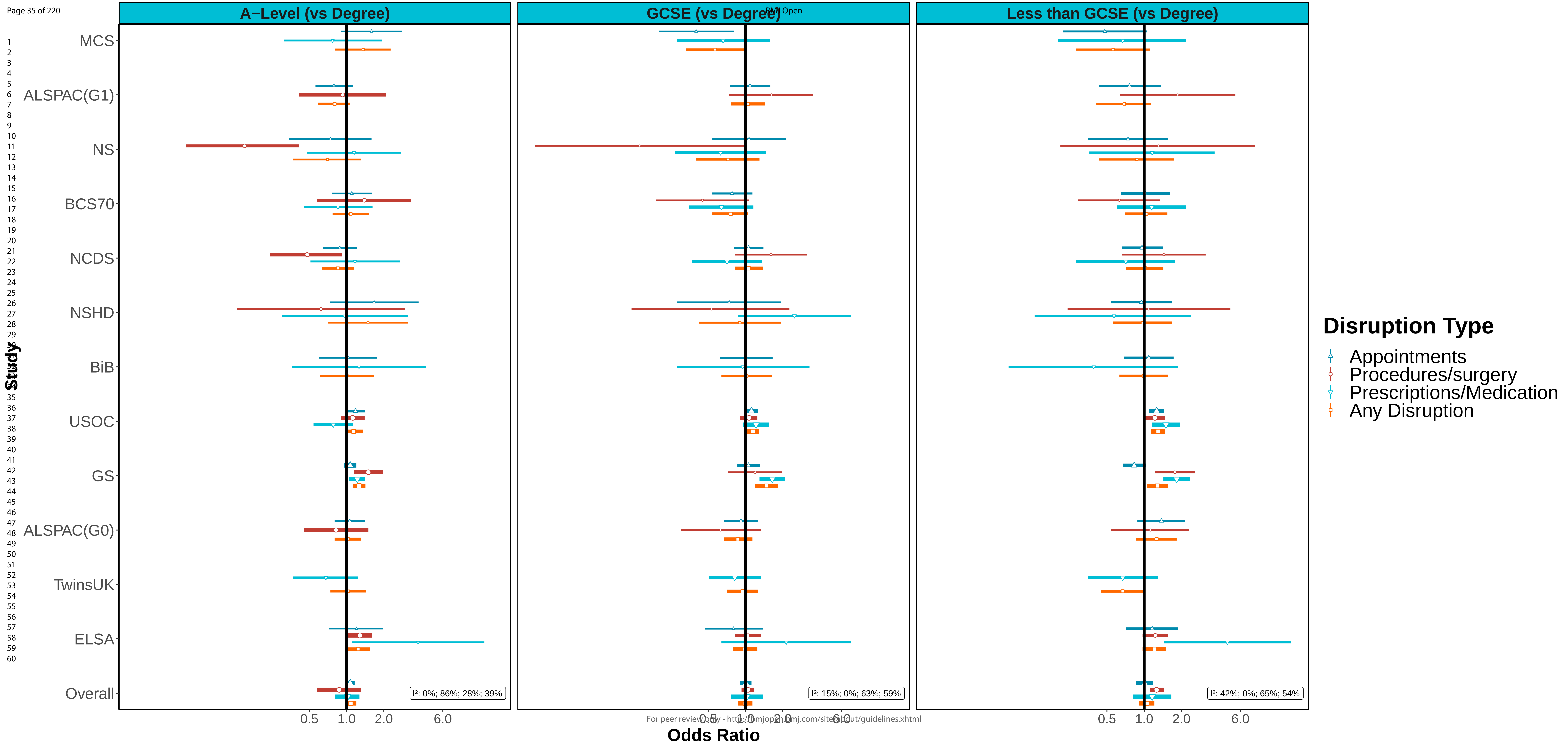
A−Level (vs Degree)
GCSE (vs Degree)
Less than GCSE (vs Degree)
MCS
ALSPAC(G1)
NS
BCS70
NCDS
NSHD
BiB
USOC
GS
ALSPAC(G0)
TwinsUK
ELSA
Overall
Study
I²: 0%; 86%; 28%; 39%
I²: 15%; 0%; 63%; 59%
I²: 42%; 0%; 65%; 54%
0.5
1.0
2.0
6.0
Odds Ratio
Disruption Type
Appointments
Procedures/surgery
Prescriptions/Medication
Any Disruption

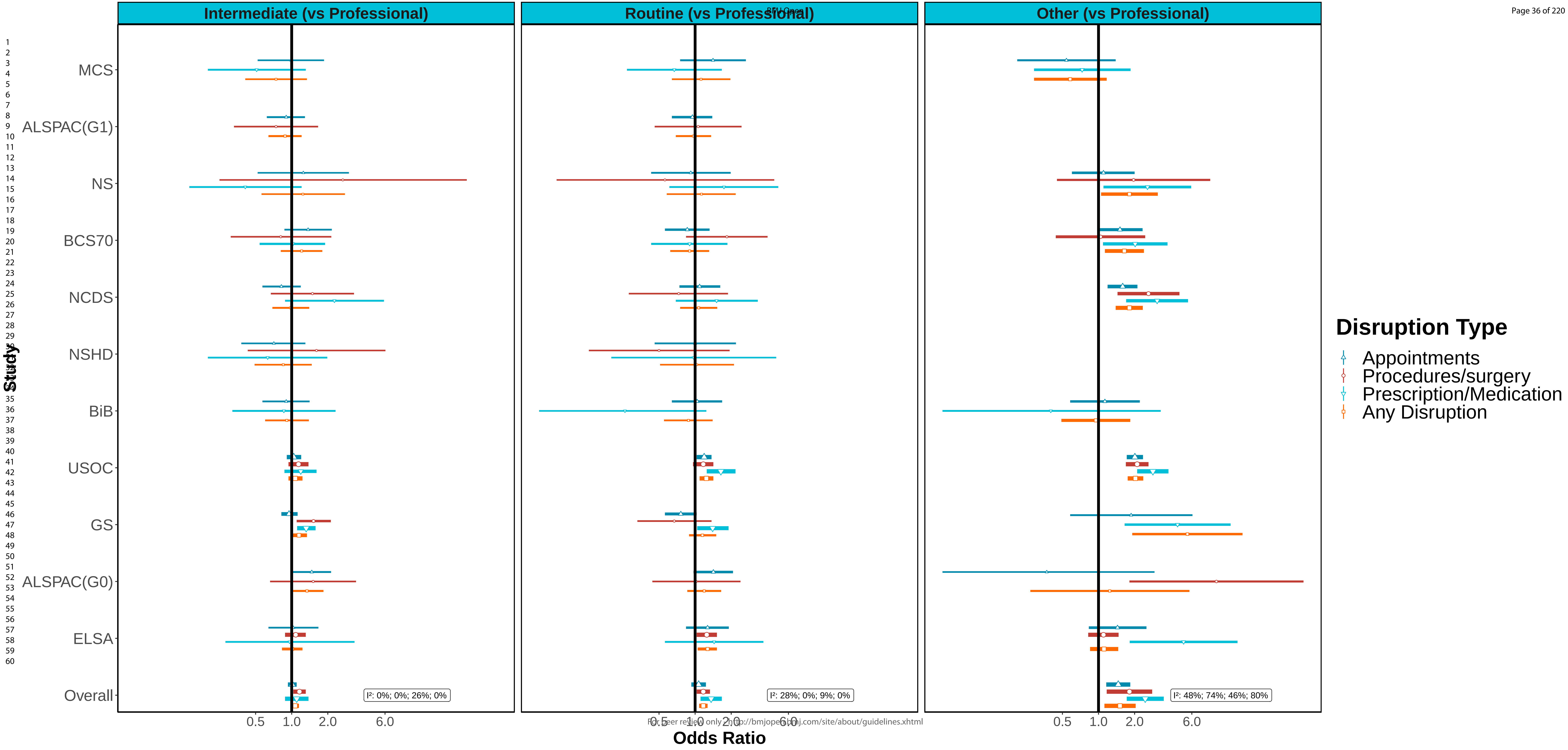
Intermediate (vs Professional)
Routine (vs Professional)
Other (vs Professional)
Study
MCS
ALSPAC(G1)
NS
BCS70
NCDS
NSHD
BiB
USOC
GS
ALSPAC(G0)
ELSA
Overall
I²: 0%; 0%; 26%; 0%
I²: 28%; 0%; 9%; 0%
I²: 48%; 74%; 46%; 80%
0.5
1.0
2.0
6.0
Odds Ratio
Disruption Type
Appointments
Procedures/surgery
Prescription/Medication
Any Disruption

Inequalities in healthcare disruptions during Covid-19 in the UK: Evidence from 12 population-based longitudinal studies

List of Supplementary Tables

Supplementary Table S1. Details of each study

Supplementary Table S2. Ethics and data access statements for each study

Supplementary Table S3. Percent prevalence of any healthcare disruptions by selected characteristics and study

For peer review only

Supplementary Table S1. Details of each study

Study Population	Design and Sample Frame	2020 Age Range	Pre-pandemic Survey	Details of Covid surveys (response rate)	Analytic N
Age Homogenous Cohorts					
MCS: Millennium Cohort Study	Cohort of UK children born between Sept 2000 and Jan 2002 with regular follow-up surveys from birth.	18-20	2018	Two surveys: May (26.6%) & Sep-Oct (24.2%)	3147
ALSPAC (G1): Avon Longitudinal Study of Parents and Children- Generation 1	Cohort of children born in the South-West of England between April 1991 and Dec 1992, with regular follow-up surveys from birth. (original young people)	27-29	2017-2018	Three questionnaires: April (19%), June (17.4%), December (26.4%)	3430
NS: Next Steps, formerly known as Longitudinal Study of Young People in England	Sample recruited via secondary schools in England at around age 13 with regular follow-up surveys thereafter.	29-31	2015	Two surveys: May (20.3%) & Sep-Oct (31.8%)	3311
BCS70: British Cohort Study 1970	Cohort of all children born in Great Britain (i.e. England, Wales & Scotland) in one week in 1970, with regular follow-up surveys from birth.	50	2016	Two surveys: May (40.4%) & Sep-Oct (43.9%)	5175
NCDS: National Child Development Study	Cohort of all children born in Great Britain (i.e. England, Wales & Scotland) in one week in 1958, with regular follow-up surveys from birth.	62	2013	Two surveys: May (57.9%) & Sep-Oct (53.9%)	5747
NSHD: National Survey of Health and Development	Cohort of all children born in Great Britain (i.e. England, Wales & Scotland) in one week in 1946, with regular follow-up surveys from birth.	74	2015	Two surveys: May (68.2%) & Sep-Oct (61.5%)	1569
Age Heterogeneous Studies					
BIB: Born in Bradford	Birth cohort recruiting pregnant women and their children between 2007 and 2010; and pregnant women and their children in three deprived areas of Bradford between 2016 and 2020	17-54	2016-2020	Two surveys: April-Jun (28%) & Oct-Nov (24%)	1726
USOC: Understanding Society: the UK Household Longitudinal Survey	A nationally representative longitudinal household panel study, based on a clustered-stratified probability sample of UK households, with all adults aged 16+ in chosen households surveyed annually.	16-96	2018-2019	Six: surveys: April (40.3%); May (33.6%); Jun (32.0%); July (31.2%); Sep (29.2%) & Nov (27.3%)	13253
ELSA: English Longitudinal Study of Aging	A nationally-representative population study of individuals aged 50+ living in England, with biennial surveys since 2002/03.	52-90+	2018-2019	First Covid-19 sub-study: Jun-July (75%)	6508
GS: Generation Scotland: the Scottish Family Health Study	A family-structured, population-based Scottish cohort, with participants aged 18-99 recruited between 2006-2011	27-100	2006-2011	Two surveys: April-Jun (21.6%) & Jul-Aug (15.6%)	17139
ALSPAC(G0): Avon Longitudinal Study of Parents and Children- Generation 0	Parents of the ALSPAC(G1) cohort described above, treated as a separate age-heterogenous study population. (original parents)	45-81	2011-2013	Three questionnaires: April (12.4%), June (12.2%), December (14.3%)	3625
TWINSUK: the UK Adult Twin Registry	A cohort of UK volunteer adult twins (55% monozygotic and 43% dizygotic) who were sampled between 18-101 years of age.	22-96	2017-2018	Three surveys: April (64.3%), July (77.6%) & November (76.1%)	4282

For peer review only

Supplementary Table S2. Ethics and data access statements for each study

**NSHD, NCDS, BCS70, NS and MCS**	The most recent sweeps of the **NSHD, NCDS, BCS70**, **Next Steps** and **MCS** have all been granted ethical approval by the National Health Service (NHS) Research Ethics Committee and all participants have given informed consent. Data for NCDS (SN 6137), BCS70 (SN 8547), Next Steps (SN 5545), MCS (SN 8682) and all four COVID-19 surveys (SN 8658) are available through the UK Data Service. NSHD data are available on request to the NSHD Data Sharing Committee. Interested researchers can apply to access the NSHD data via a standard application procedure. Data requests should be submitted to mrclha.swiftinfo@ucl.ac.uk; further details can be found at http://www.nshd.mrc.ac.uk/data.aspx. doi:10.5522/NSHD/Q101; doi:10.5522/NSHD/Q10.
**ALSPAC**	Ethical approval was obtained from the **ALSPAC** Ethics and Law Committee and the Local Research Ethics Committees. The study website contains details of all the data that is available through a fully searchable data dictionary and variable search tool: http://www.bristol.ac.uk/alspac/researchers/our-data. ALSPAC data is available to researchers through an online proposal system. Information regarding access can be found on the ALSPAC website (http://www.bristol.ac.uk/media-library/sites/alspac/documents/researchers/data-access/ALSPAC_Access_Policy.pdf).
**BIB**	Ethical approval for **Born in Bradford** was granted by the National Health Service Health Research Authority Yorkshire and the Humber (Bradford Leeds) Research Ethics Committee (reference: 16/YH/0320). Data from the various BiB family studies are available to researchers; see the study website for information on how to access data (https://borninbradford.nhs.uk/research/how-to-access-data/).
**USOC**	The University of Essex Ethics Committee has approved all data collection for the **Understanding Society** main study and COVID-19 waves. No additional ethical approval was necessary for this secondary data analysis. All data are available through the UK Data Service (SN 6614 and SN 8644).
**ELSA**	Waves 1-9 of **ELSA** were approved through the National Research Ethics Service, while the COVID-19 Sub-study was approved by the UCL Research Ethics Committee. All participants provided informed consent. All data are available through the UK Data Service (SN 8688 and 5050).
**GS**	**Generation Scotland** obtained ethical approval from the East of Scotland Committee on Medical Research Ethics (on behalf of the National Health Service). Reference number 20/ES/0021. Access to data is approved by the Generation Scotland Access Committee. See https://www.ed.ac.uk/generation-scotland/for-researchers/access or email access@generationscotland.org for further details.
**TWINSUK**	All wave of **TwinsUK** have received ethical approval associated with TwinsUK Biobank (19/NW/0187), TwinsUK (EC04/015) or Healthy Ageing Twin Study (H.A.T.S) (07/H0802/84) studies from NHS Research Ethics Committees at the Department of Twin Research and Genetic Epidemiology, King's College London. The TwinsUK Resource Executive Committee (TREC) oversees management, data sharing and collaborations involving the TwinsUK registry (for further details see https://twinsuk.ac.uk/resources-for-researchers/access-our-data/).

For peer review only

Supplementary Table S3. Percent prevalence of any healthcare disruptions by selected characteristics and study

		MCS	*ALSPAC (G1)*	*NS*	*BCS70*	*NCDS*	*NSHD*	*BIB*	*USOC*	*GS*	*ALSPAC (G0)*	*TWINS UK*	*ELSA*
**Sex**	*Male*	6.1	12.8	12.1	11.7	15.6	14.5	NA	29.4	24.9	18.1	7.4	17.5
	Female	14.1	17.5	13.8	16.9	17.4	18.2	9.4	34.0	25.5	20.5	8.5	21.3
**Age**	*16-24*	10.1						8.2	18.3	NA	NA	10	NA
	25-34		15.9	12.8				10.4	24.0	22.9	NA	7.7	NA
	35-44							9.1	24.9	23.0	NA	13.2	NA
	45-54				14.3			8.7	30.9	24.2	21.3	13.9	13.0
	55-64					16.7		--	38.6	25.2	19.2	21.6	17.2
	65-74						16.4	--	43.6	26.8	21.8	31.4	20.0
	75+							--	45.6	29.2	30.6	9.2	25.5
**Ethnicity**	*White*	10.0	16.1	13.3	--	--	--	7.8	31.9	25.4	19.9	8.3	19.5
	South Asian	6.6	NA	8.4	--	--	--	10.6	25.4	20.0	NA	5.1	22.9
	OtherAsian	NA	NA	NA	--	--	--	NA	37.5	27.4	NA	11.1	NA
	Black	7.7	NA	18.8	--	--	--	5.9	35.8	19.0	NA	11.5	21.7
	Mixed	23.5	NA	11.1	--	--	--	8.3	27.7	22.9	NA	10	15.5
	Other	11.1	NA	4.2	--	--	--	8.5	30.2	28.6	NA	9.1	NA
	All ethnic Minorities	10.6	9.0	10.7	--	--	--	10.3	30.4	23.6	19.6	8.3	21.1
**Education**	*Higher Ed*	11.2	16.9	14.0	14.5	16.8	16.03	9.0	29.7	23.3	19.4	9.9	16.9
	A-level	14.8	14.4	10.5	15.5	14.0	22.67	9.2	27.0	26.7	20.0	10.3	20.5
	GCSE	6.3	18.1	11.3	12.0	17.6	15.6	9.0	31.3	29.3	17.8	9.2	17.4
	<GCSE/ None	6.2	12.4	14.5	15.5	17.2	16.3	9.1	39.0	27.8	23.9	6.1	22.4
**Social Class**	*Managerial/ Admin/ Professional*	11.6	16.4	11.1	12.6	12.7	17.0	9.7	25.7	24.3	16.4	-	18.3
	Intermediate	8.5	15.2	12.7	15.3	12.6	15.5	9.0	27.2	25.7	21.3	-	19.5
	Manual/Routine	11.2	16.7	11.6	11.6	13.6	18.6	9.3	27.6	25.6	19.6	-	23.4
	Other	6.0	0	18.0	19.3	21.1	0	11.8	42.6	51.9	20.0	-	16.6
**Not Instructed to Shield**		9.0	--	12.0	12.4	14.6	16.7	--	29.6	23.9	--	8.9	16.2
**Instructed to Shield**		47.5	--	44.3	49.4	41.9	28.4	--	61.0	42.0	--	15.3	35.5

Sources: MCS (Millennium Cohort Study); ALSPAC G1 (Children of the Avon Longitudinal Study of Parents and Children); NS (Next Steps); BCS 70 (1970 British Cohort Study), NCDS (National Child Development Study); NSHD (National Survey of Health and Development); BIB (Born in Bradford); ; USOC (Understanding Society); GS (Generation Scotland: the Scottish Family Health Study); ALSPAC G0 (parents of ALSPAC); TWINS UK (UK Adult Twin Registry); ELSA (English Longitudinal Study of Ageing). Notes: Samples for each study restricted to respondents with non-missing information on healthcare disruptions and valid information on sex, social class, education and (where applicable) age and ethnicity. All information about how information was collected and variables were coded is available in Supplementary File 1. NA= Not available; (--)= Info not collected. Weighted data where applicable

For peer review only

For peer review only

Supplementary File 1: Variable coding

Contents

Healthcare disruptions 2

Covariates 6

A note about shielding 11

For peer review only

Healthcare disruptions

Study	*Question (exact wording)*	*Possible Answers*	*Recoding if needed*
*** PRESCRIPTION or MEDICATION ACCESS ***			
**MCS<br>NS<br>BCS 70<br>NCDS<br>NSHD**	Since the Coronavirus outbreak in March, have you had any difficulty obtaining any of your prescribed medication?	1=Yes; 2=No/Not applicable	= 1
**ALSPAC**	Not Available		
**USOC**	Q1: Still thinking about your situation now, have you been able to access the NHS services you need: Prescription medicine?<br>Q2: Still thinking about your situation now, have you been able to access the community health and social care services and support you need... Over the counter medications?	For both Q1 and Q2:<br>1=Yes; 2=No; 3=Not required	Q1=2 OR Q2=2
**ELSA**	Since the coronavirus outbreak, have you been able to get access to your regular medications?	1=Yes; 2=No; 3=No need	= 2
**GS**	How strongly do you agree with the following statements: Accessing and remembering to take my medication has become more difficult during the COVID-19 pandemic	From 1 (do not agree at all) to 10 (agree very strongly)	=6/10
**TWINS UK**	Have you experienced any of the following as a result of COVID-19? Unable to access required medication	0= No; 1 = Yes	= 1
**BIB**	Q1: Have you or a member of your household needed to access pharmacy services since lockdown began?<br>Q2: If yes, did you receive the support you needed?	Q1. 0=No; 1=Yes<br>Q2. 0= No; 1=Yes; 2=Haven't tried	Q1=1 & Q2=0
*** PROCEDURES or SURGERIES ***			
**MCS<br>NS<br>BCS 70<br>NCDS<br>NSHD**	Q1: At the time of the Coronavirus outbreak in March, did you have an in-patient or out-patient appointment booked at a hospital for a consultation, investigation, treatment or surgery?<br>Q2: Have you now had your surgery?<br>Q3: Did your (last) surgery take place on the planned date or was it delayed?<br>Q4: Why has your surgery not taken place?	Q1. 1=Yes - for a consultation investigation or treatment; 2=Yes - for surgery; 3=No.<br>Q2. 1=Yes; 2=No.<br>Q3. 1=Surgery took place on the planned date; 2=Surgery was delayed.<br>Q4. 1=My surgery was postponed and	Q1=2 & Q2=1 & Q3=2<br>*OR*<br>Q1=2 & Q2=2 & Q4=(1 OR 3)

For peer review only - http://bmjopen.bmj.com/site/about/guidelines.xhtml

		has not yet happened; 2=My surgery was not postponed, but it hasn't happened yet; 3=My surgery was cancelled	
**ALSPAC GS**	Q1: Have you had any medical treatments or appointments that have had to be cancelled or postponed during the COVID-19 pandemic? For example, hospital referral, non-emergency surgery, cancer, treatment, etc.<br>Q2: What types of medical treatments or appointments were cancelled or postponed?	Q1. 1=Yes; 2=No.<br>Q2. a -- surgery: 1=Yes; -9=Not applicable<br>b -- cancer treatment: 1=Yes; -9=Not applicable<br>c -- dialysis: 1=Yes; -9=Not applicable	Q1=1 & Q2 (a OR b OR c)=1
**USOC**	Q1: [since previous survey] have you had or been waiting for NHS treatment? Please select all that apply.<br>Q2: Has your treatment plan(s) been changed in any way?	Q1. 1=Yes, tests/consultations planned or in progress; 2=Yes, operation or procedure planned; 3=Yes, targeted therapy, chemotherapy or radiotherapy planned or in progress; 4=Yes, other treatment planned; 5=No<br>Q2. 1=Yes, consultations/treatments cancelled or postponed by NHS; 2=Yes, alternative treatment provided; 3=Yes, I cancelled or postponed treatment; 4=No, treatment continuing as planned	Q1=2/4 & Q2=1/3
**ELSA**	Since the coronavirus outbreak, have you had a hospital operation or treatment cancelled?	1.Yes; 2.No	=1
**TWINS UK**	Not Available		
**BIB**	Not Available		

*** APPOINTMENTS ***			
**MCS NS BCS 70 NCDS NSHD**	Q1: At the time of the Coronavirus outbreak in March, did you have an in-patient or out-patient appointment booked at a hospital for a consultation, investigation, treatment or surgery?<br>Q2: Have you now had your in/ out-patient hospital appointment for a consultation, investigation or treatment?<br>Q3: Did your (last) appointment take place on the planned date or was it delayed?<br>Q4: Why has your in-/out-patient hospital appointment for a consultation, investigation or treatment not taken place?	Q1. 1=Yes - for a consultation investigation or treatment; 2=Yes - for surgery; 3=No.<br>Q2. 1=Yes; 2=No.<br>Q3. 1=Appointment took place on the planned date; 2=Appointment was delayed.<br>Q4. 1=My appointment was postponed	Q1=1 & Q2=1 & Q3=2 OR Q1=1 & Q2=2 & Q4=(1 OR 3)

For peer review only - http://bmjopen.bmj.com/site/about/guidelines.xhtml

		and has not yet happened; 2=My appointment was not postponed, but it hasn't happened yet; 3=My appointment was cancelled	
**ALSPAC GS**	Q1. Have you had any medical treatments or appointments that have had to be cancelled or postponed during the COVID-19 pandemic? For example, hospital referral, non-emergency surgery, cancer, treatment, etc.<br>Q2. What types of medical treatments or appointments were cancelled or postponed?	Q1. 1=Yes; 2=No.<br>Q2= d -- GP referral: 1=Yes; -9=Not applicable<br>e -- Hospital referral: 1=Yes; -9=Not applicable<br>f -- Routine clinical appointment: 1=Yes; -9=Not applicable<br>g -- Cancer testing: 1=Yes; -9=Not applicable<br>h -- Cancer screening: 1=Yes; -9=Not applicable	Q1=1 & Q2(d OR e OR f OR g OR h)=1
**USOC**	Thinking about your situation now, have you been able to access the NHS services you need to help manage your condition(s) over the last 4 weeks?<br>Q1: GP or primary care practice staff?<br>Q2: Hospital or clinic outpatient?<br>Q3: Hospital or clinic inpatient?<br>Q4: [since previous survey] have you had or been waiting for NHS treatment? Please select all that apply.<br>Q5: Has your treatment plan(s) been changed in any way?	Q1-3. 1=Yes, in person; 2=(Q1 & Q2 only) Yes, online or by phone only; 3=No, not able to access; 4=No, decided not to seek help at this time/cancelled; 5=Alternative treatment provided; 6=Not required<br>Q4. 1=Yes, tests/consultations planned or in progress; 2=Yes, operation or procedure planned; 3=Yes, targeted therapy, chemotherapy or radiotherapy planned or in progress; 4=Yes, other treatment planned; 5=No<br>Q5. 1=Yes, consultations/treatments cancelled or postponed by NHS; 2=Yes, alternative treatment provided; 3=Yes, I cancelled or postponed treatment; 4=No, treatment continuing as planned	Q1|Q2|Q3=(3 OR 4 OR 5)<br>OR<br>Q4=1 AND Q5=(1 OR 2 OR3)
**ELSA**	Q1: Since the coronavirus outbreak, have you wanted to see or talk to a GP?<br>Q2: Have you been able to see or talk to a GP?	Q1: 1=Yes; 2=No<br>Q2: 1=Yes; 2=No; 3=I did not attempt to contact them 4.I did not need to contact them	Q1= & Q2=2
**TWINS UK**	Not Available		

For peer review only - http://bmjopen.bmj.com/site/about/guidelines.xhtml

**BIB**	Q1: Have you or a member of your household needed to access<br>-- (1) your doctor (GP) or nurse<br>-- (2) NHS111<br>-- (3) Health emergency services (A&E)<br>-- (4) A specialist (consultant) doctor or specialist clinic (hospital outpatient) appointment since lockdown began?<br>Q2: If yes, were you able to access (1, 2, 3, or 4)?	Q1. 0=No; 1=Yes<br>Q2. 0= No; 1=Yes; 2=Haven't tried	Q1= & Q2=0

For peer review only

Covariates

Variables	*Study*	*Options*	*Recoding if needed*
*** Sex * 0=Male; 1=Female**			
	**All**	0=Male; 1=Female	
*** Ethnicity * 0=White; 1=South East Asian; 2=Other Asian; 3=Black; 4=Mixed; 5=Other Non-White**			
	**MCS**	1=White; 2=Mixed; 3=Indian; 4=Pakistani; 5=Bangladeshi; 6=Other Asian; 7=Black Caribbean; 8=Black African; 9=Other Black; 10=Chinese; 11=Other ethnic group	1=0, 2=4, 3-5=1, 6 & 10=2, 7-9=3, 11=5
	**NS**	1=White; 2=Mixed; 3=Indian; 4=Pakistani; 5=Bangladeshi; 6=Black Caribbean; 7=Black African; 8=Other	1=0, 2=4, 3-5=1, 6-7=3, 8=5
	**BCS70**	Not Available	
	**NCDS**	Not Available	
	**NSHD**	Not Available	
	**ALSPAC**	G0 (Parents) 1=White; 2=Black Caribbean; 3=Black African; 4=Other black; 5=Indian; 6=Pakistani; 7=Bangladeshi; 8=Chinese; 9=Other<br>G1 (Children) 1=White; 2=Mixed/Multiple Ethnic group; 3=Asian; 4=Black/African/Caribbean/Black British; 5=Arab or Other	G0: 1=0; 5/7=1, 8=2, 2/4=3, 9=5<br>G1: 1=0; 3=2, 4=3, 2=4
	**USOC**	1=White British; 2=Irish (White); 3=Gypsy or Irish Traveller (white); 4=Any other white background; 5=White and black Caribbean (mixed); 6=White and black African (mixed); 7=White and Asian (mixed); 8=Any other mixed background; 9=Indian (Asian or Asian British); 10=Pakistani (Asian or Asian British); 11=Bangladeshi (Asian or Asian British); 12=Chinese (Asian or Asian British); 13=Any other Asian background (Asian or Asian British); 14=Caribbean (Black or Black British); 15=African (Black or Black British); 16=Any other Black background (Black or Black British); 17=Arab (other Ethnic group); 97=Any other ethnic group	1-4=0, 5-8=4, 9-11=1, 12-13=2, 14-16=3, 17-97=5
	**ELSA**	1.White; 2=Mixed ethnic group; 3=Black; 4=Black British; 5=Asian; 6=Asian British	1=0; 2=4; 3/4=3; 5/6=1
	**GS**	1=White Scottish; 2=White English; 3=White Welsh; 4=White N. Irish; 5=White Irish; 6=White Gypsy/Irish traveller; 7=White Polish; 8=Any other white; 9=Asian/British Asian - Indian; 10=Asian/British Asian - Pakistani; 11=Asian/British Asian - Bangladeshi; 12=Asian/British Asian - Chinese; 13=Any other Asian background; 14=Black or Black British - African; 15=Black or Black British - Caribbean; 16=Any other Black/African/Caribbean background; 17=Arab or Arab British; 18=Mixed - White and Black Caribbean; 19=Mixed - White and Black African; 20=Mixed - White and Asian; 21=Any other Mixed/Multiple ethnic background; 22=Any other ethnic group	1/8=0, 9/11=1, 12/13=2, 14/16=3, 18/21=4, 17&22=5

**TWINS UK**	1=White- English, Welsh, Scottish, Northern Irish, Irish; 2=White- Other white background; 3=Mixed/multiple ethnic groups - White and Black Caribbean; 4=Mixed/multiple ethnic groups - White and Black African; 5=Mixed/multiple ethnic groups - White and Asian; 6=Mixed/multiple ethnic groups - Other mixed/ multiple ethnic background; 7=Asian/Asian British- Indian; 8=Asian/Asian British - Pakistani; 9=Asian/Asian British - Bangladeshi; 10=Asian/Asian British - Chinese; 11=Asian/Asian British - Other Asian background; 12=Black/Black British - African; 13=Black/Black British - Caribbean; 14=Black/Black British - Other Black Background; 15=Middle-Eastern; 16=Other ethnic group	1/2=0; 10=1; 7/9|11=2; 12/14=3; 3/6=4; 15/16=5
**BIB**	BiB: 1=White British; 2=White other; 3=Mixed-White and Black; 4=Mixed-White and South Asian; 5=Black; 6=Indian; 7=Pakistani; 8=Bangladeshi; 9=Other<br>BIBBS: 1=White British; 2=White Irish; 3=Pakistani; 4=Indian; 5=Bangladeshi; 6=White Polish; 7=White Slovakian; 8=White Romanian; 9=White Czech; 10=Other White; 11=White Gypsy/Roma/Irish traveller; 12=Chinese; 13=African; 14=Caribbean; 15=Mixed White/Black Caribbean; 16=Mixed White/Black African; 17=Mixed White/Asian; 18=Do not wish to answer; 19=Other	BiB: 1/2=0; 6/8=1; 5=3; 3/4=4; 9=5<br>BiBBs: 1/2=0; 6/11=0; 3/5=1; 13/14=3; 15/18=4; all other options=5
*** Education * 0= Degree; 1=A-Level; 2=GCSE; 3=Low or None**		
**MCS**<br>**NS**<br>**BCS 70**<br>**NCDS**	0=None; 1=Nvq1; 2=Nvq2; 3=Nvq3; 4=Nvq4; 5=Nvq5<br>*parent's education for MCS	0/1 = 0 2=1 3=2 4/5=3
**NSHD**	0=None attempted; 1.=Vocational course, proficiency only; 2=Sub GCE or sub Burnham C; 3=GCE 'O' level or Burnham C; 4=GCE 'A' Level or Burnham B; 5=Burnham A2; 6= 1st Degree or graduate equivalent; 7= Higher degree, Masters; 8= Higher degree, doctorate; 9=Unknown	6|7|8=0; 4|5=1; 3=2; 0|1|2|9=3
**ALSPAC**	1=Degree; 2=A levels/AS levels or equivalent; 3=O levels; 4=Vocational; 5=CSE<br>*parent's education for G1 (Children)	1=0; 2=1; 3=2; 4/5=3
**USOC**	1.Higher degree 2. 1st degree or equivalent 3. Diploma in Higher Education 4. Teaching qualification (not PGCE) 5. Nursing or other medical qualification 6. Other higher degree 7. A-Level 8. Welsh baccalaureate 9. International baccalaureate 10. AS Level 11. Scottish Highers 12. Certificate of 6th year studies 13. GCSE/O-Level 14. Certificate of secondary education 15. Standard or lower 16. Other school certificate 96. No qualifications	1-6=0, 7-12=1, 13-16=2, 96=3
**ELSA**	1=Nvq4/nvq5/degree or equivalent; 2=Higher Education below degree; 3=Nvq3/GCE A level equivalent; 4=Nvq2/GCE O level equivalent; 5=Nvq1/CSE other grade equivalent; 6=Foreign/other; 7=No qualification	1=0; 2/3=1; 4=2; 5/7=3
**GS**	1=No qualifications; 2=Other (please specify); 3=School leavers certificate; 4=CSEs or equivalent; 5=Standard grade, National 4 or 5, O levels, GCSEs or equivalent; 6=Higher grade, A levels, AS levels or equivalent; 7=NVQ or HND or HNC or equivalent; 8=Other professional or technical qualification; 9=Undergraduate degree; 10=Postgraduate degree	9|10=0; 6|7|8 =1; 5=2; <5=3

For peer review only - http://bmjopen.bmj.com/site/about/guidelines.xhtml

**TWINS UK**	1=No qualification; 2=NVQ1/SVQ1; 3=O-level/GCSE/NVQ2/SVQ2/Scottish intermediate; 4=Scottish Higher, NVQ3, City and Guilds, Pitman; 5=A-level, Scottish Advanced Higher; 6=Higher vocational training (e.g. Diploma, NVQ4, SVQ4); 7=Undergraduate degree; 8=Postgraduate degree (e.g. Masters or PhD), NVQ5, SVQ5	6/8=0; 4/5=1; 3=2; 1/2=3
**BIB**	1=<5 GCSE equivalent; 2=5 GCSE equivalent; 3=A-level equivalent; 4=Higher than A-level; 5=Other; 6=Don't know; 7=Foreign unknown	4=0; 3=1; 5/7=2; 1=3; missing=1
*** Occupational Social Class * 1=Managerial/Admin/Professional; 2=Intermediate; 3=Manual/routine; 4=Other**		
NS-SEC: National Statistics Socioeconomic Classification. RGSC: Registrat General's Social Class. ONS SOC: Office of National Statistics Standard Occupational Classification		
**MCS**<br>**NS**<br>**BCS 70**<br>**NCDS**	[NS-SEC] 1=Higher managerial and professional; 2=Lower managerial and professional; 3=Intermediate occupations; 4=Small employers and own account workers; 5=Lower supervisory and technical; 6=Semi-routine occupations; 7=Routine occupations; 8=Never worked and long-term unemployed<br>*parent's occupational social class for MCS	2=1; 3-4=2; 5-7=3; 8=4
**NSHD**	[RGSC] 1=I Professional; 2=II Managerial and Technical; 3=IIINM Skilled non-manual; 4=IIIM Skilled manual; 5=IV Partly skilled; 6=V Unskilled;	2=1; 3/5=2; 6=3;
**ALSPAC**	[RGSC] 1=I Professional; 2=II Managerial and Technical; 3=IIINM Skilled non-manual; 4=IIIM Skilled manual; 5=IV Partly skilled; 6=V Unskilled; 7=Armed Forces<br>*parent's occupational social class for G1 (Children)	2=1; 3/5=2; 6=3; 7=4
**USOC**	[NS-SEC] 1=Higher managerial and professional; 2=Lower managerial and professional; 3=Intermediate occupations; 4=Small employers and own account workers; 5=Lower supervisory and technical; 6=Semi-routine occupations; 7=Routine occupations; 8=Never worked and long-term unemployed	2=1; 3-4=2; 5-7=3; 8=4
**ELSA**	[NS-SEC] -3=Incomplete/No job info; 1=Higher and Lower managerial/ professional; 2=Intermediate occupations; 3=Routine and manual occupations; 99=Other	99=4; -3=4
**GS**	[ONS SOC] 1=Managers, directors, senior officials; 2=Associate professional and technical occupations; 3=Administrative and secretarial occupations; 4=Skilled trades occupations; 5=Sales and customer service occupations; 6=Process, plant and machine operatives; 7=Elementary (unskilled) occupations; 8=Never worked	1/3=1; 4/5=2; 6/7=3; 8=4
**TWINS UK**	Not Available	
**BIB**	1=Modern professional occupations; 2=Clerical and intermediate occupations; 3=Senior managers or administrators; 4=Technical and craft occupations; 5=Semi-routine manual and service occupations; 6=Routine manual and service occupations; 7=Middle or junior managers; 8=Traditional professional occupations;<br>9=Self-employed; 10=Student/in training; 11=Does not work-long term unemployed/sick; 12=Don't know<br>*Based on either own class (80.7%) or partner's (19.3%)	3=1; 8=1; 4=2; 7=2; 5/6=3; all other options=4

For peer review only - http://bmjopen.bmj.com/site/about/guidelines.xhtml

*** Living Arrangement * 1=Alone; 2=With partner/spouse only; 3=With partner/spouse and child(ren); 4=With child(ren), without partner/spouse; 5=Any other living arrangement<br><br>OR * Partnership Status * 1=Married/Partnered; 0=Not married/partnered**		
**MCS<br>NS<br>BCS 70<br>NCDS**	Who do you currently live with? 1. Husband/Wife/Cohabiting Partner2. Children (including adult children, step-children, adopted children, foster children or any other children you consider yourself parent to) 3. Parent or Parent-in-law (including step-parent or adoptive parent) 4. Grandparent 5. Grandchild 6. Sibling 7. Other relative 8. Friend / unrelated sharer 9. Other	1 = Husband/Wife/Cohabiting Partner; 0 = Other
**NSHD**	Who do you currently live with? (Options include Husband/Wife/Cohabiting Partner)	1= Partner in HH 0= No partner in household
**ALSPAC**	NA	NA
**USOC**	Derived from Household Grid	0=partner present; 1=Single
**ELSA**	IF respondents live with other people, they are asked for each person "what is this person's relationship to you". Options include "1. Husband/wife/partner"	1=Partner in HH 0=No partner in HH
**GS**	1. Married/ Civil partnership 2. In a relationship, living together 3. In a relationship, not living together 4. Single 5. Separated 6. Divorced 7. Widowed 8. Other	1-3=1 4-8 = 0
**TWINS UK**	Single, never married (1); Single, divorced or widowed (2); In a relationship/married but living apart (3); In a relationship/married and cohabiting (4)	1, 2 = 0; 3, 4 = 1
**BIB**	What is your current relationship status? 0=do not wish to answer; 1=single; 2=married; 3=not married but in a relationship	1=0; 2/3=1
*** Shielding Status * 1=Advised to Shield; 0=Not advised to shield**		
**MCS<br>NS<br>BCS 70<br>NCDS<br>NSHD**	Did you at any time receive a letter or text message from the NHS or Chief Medical Officer saying that you have been identified as someone at risk of severe illness if you catch Coronavirus, because you have an underlying disease or health condition? 1=Yes; 2=No	2=0
**ALSPAC**	Not Available	

	**USOC**	Have you received a letter, text or email from the NHS or Chief Medical Officer saying that you have been identified as someone at risk of severe illness if you catch coronavirus, because you have an underlying disease or health condition? 1=Yes; 2=No	2=0
	**ELSA**	Have you been contacted by the NHS or your GP and advised that you are vulnerable and at risk of severe illness if you catch coronavirus (Covid-19), and should stay at home at all times and avoid any face-to-face contact? 1=Yes; 2=No	2=0
	**GS**	Have you been contacted by letter or text message to say you are at sever risk from COVID-19 due to and underlying health condition and should be shielding? 1=Yes; 2=No	2=0
	**TWINS UK**	Have you received a letter or text message over the past few months to say you are at high risk from COVID-19 due to an underlying health condition, and should be ‘shielding’? 1=Yes; 2=No	2=0
	**BIB**	Have you been advised by a health professional that you are high risk or vulnerable and should self-isolate for 12 weeks to protect yourself from coronavirus? 0=No; 1=Yes	
*** Pre-Pandemic Self-Assessed Health * 1=Good/Very Good/Excellent; 0=Fair/Poor**			
	**MCS**<br>**NS**<br>**BCS 70**<br>**NCDS**<br>**NSHD**	In general, in the 3 months before the Coronavirus outbreak would you say your health was … 1=Excellent; 2=Very Good; 3=Good; 4=Fair; 5=Poor	1/3=1; 4/5=0
	**ALSPAC (G0 & G1)**	(2020) Do you have a history of diabetes (A), obesity (B) or asthma (C)?	1 if A & B & C==0<br>0 if A | B | C==1
	**USOC**	(2018/19) In general, would you say your health is… 1=Excellent; 2=Very Good; 3=Good; 4=Fair; 5=Poor	1/3=1; 4/5=0
	**ELSA**	(2018/19) Would you say your health is… 1=Excellent; 2=Very Good; 3=Good; 4=Fair; 5=Poor	1/3=1; 4/5=0
	**GS**	NA	
	**TWINS UK**	(2020) In general, would you say your health is… 1=Excellent; 2=Very Good; 3=Good; 4=Fair; 5=Poor	1/3=1; 4/5=0
	**BIB**	(2016 - 2020) In general, would you say your health is… 1=Excellent; 2=Very Good; 3=Good; 4=Fair; 5=Poor	1/3=1; 4/5=0

For peer review only - http://bmjopen.bmj.com/site/about/guidelines.xhtml

A note about shielding

Who had to shield?

Initially 1.5 million, increasing to 2.2 million, people in the UK were identified as clinically extremely vulnerable (CEV) by their GP. They were sent a letter asking them to shield – not go out – for at least 12 weeks until the end of June. This timeframe was extended, and on 1st August, CEV individuals in England, Scotland and Northern Ireland were told that shielding had been paused. In Wales shielding continued until 16th August.

Who was classed as clinically extremely vulnerable?

People falling into the clinically extremely vulnerable group include:

- Solid organ transplant recipients
- People with cancer who are undergoing active chemotherapy or radical radiotherapy for lung cancer
- People with cancers of the blood or bone marrow such as leukaemia, lymphoma or myeloma who are at any stage of treatment
- People having immunotherapy or other continuing antibody treatments for cancer
- People having other targeted cancer treatments which can affect the immune system, such as protein kinase inhibitors or PARP inhibitors (which prevent cancer cells from repairing)
- People who have had bone marrow or stem cell transplants in the last 6 months, or who are still taking immunosuppression drugs
- People with severe respiratory conditions including all cystic fibrosis, severe asthma and severe chronic obstructive pulmonary disease (COPD)
- People with rare diseases and inborn errors of metabolism that significantly increase the risk of infections such as Severe combined immunodeficiency (SCID) or homozygous sickle cell)
- People on immunosuppression therapies sufficient to significantly increase risk of infection
- Women who are pregnant with significant heart disease, congenital or acquired.

Source: https://web.archive.org/web/20200330181117/https://www.gov.uk/government/publications/covid-19-guidance-on-social-distancing-and-for-vulnerable-people/guidance-on-social-distancing-for-everyone-in-the-uk-and-protecting-older-people-and-vulnerable-adults

For peer review only - http://bmjopen.bmj.com/site/about/guidelines.xhtml

Supplementary file 2: Meta-analysis results

Contents

Summary of results ... 3
Summary of stratified results ... 6
Any healthcare disruption ... 10
  Sex ... 10
    Unadjusted ... 10
    Basic adjustment ... 11
    Full adjustment ... 12
  Occupational class ... 13
    Unadjusted ... 13
    Basic adjustment ... 15
    Full adjustment ... 17
  Education ... 19
    Unadjusted ... 19
    Basic adjustment ... 21
    Full adjustment ... 23
  Age ... 25
    Unadjusted ... 25
    Basic adjustment ... 27
    Full adjustment ... 29
  Ethnicity ... 31
    Unadjusted ... 31
    Basic adjustment ... 34
    Full adjustment ... 38
Appointments ... 41
  Sex ... 41
    Unadjusted ... 41
    Basic adjustment ... 42
    Full adjustment ... 43
  Occupational class ... 44
    Unadjusted ... 44
    Basic adjustment ... 46
    Full adjustment ... 48
  Education ... 50
    Unadjusted ... 50
    Basic adjustment ... 52
    Full Adjustment ... 54
  Age ... 56
    Unadjusted ... 56
    Basic adjustment ... 58
    Full adjustment ... 60
  Ethnicity ... 62
    Unadjusted ... 62
    Basic adjustment ... 65
    Full adjustment ... 68
Prescription/Medication access ... 71
  Sex ... 71
    Unadjusted ... 71
    Basic adjustment ... 72
    Full adjustment ... 73
  Occupational class ... 74

For peer review only

For peer review only - http://bmjopen.bmj.com/site/about/guidelines.xhtml

Unadjusted 74
Basic adjustment 76
Full adjustment 78
Education 80
Unadjusted 80
Basic adjustment 82
Full adjustment 84
Age 86
Unadjusted 86
Basic adjustment 89
Full adjustment 91
Ethnicity 93
Unadjusted 93
Basic adjustment 96
Full adjustment 99
Procedures/surgery 102
Sex 102
Unadjusted 102
Basic adjustment 103
Full adjustment 104
Occupational class 105
Unadjusted 105
Basic adjustment 107
Full adjustment 109
Education 111
unadjusted 111
Basic adjustment 113
Full adjustment 115
Age 117
Unadjusted 117
Basic adjustment 120
Full adjustment 122
Ethnicity 123
Unadjusted 123
Basic adjustment 126
Full adjustment 129
Any healthcare disruption stratified by shielding status 131
Sex 131
Occupational class 132
Education 136
Age 140
Ethnicity 143
Any healthcare disruption stratified by age 148
Sex 148
Occupational class 149
Education 152
Ethnicity 155

For peer review only - http://bmjopen.bmj.com/site/about/guidelines.xhtml

Summary of results

		Any healthcare disruption				Appointments				Prescription/Medication				Procedures/surgery			
		OR	Lower CI	Upper CI	I²%	OR	Lower CI	Upper CI	I²%	OR	Lower CI	Upper CI	I²%	OR	Lower CI	Upper CI	I²%
**Sex**																	
Female vs. Male	Unadjusted	1.26	1.14	1.39	58.51	1.30	1.12	1.52	73.54	1.33	1.00	1.77	75.15	1.12	0.93	1.36	60.28
	**Basic adjustment**	**1.27**	**1.15**	**1.40**	**53.11**	**1.33**	**1.17**	**1.52**	**60**	**1.27**	**0.94**	**1.74**	**77.98**	**1.15**	**0.97**	**1.37**	**47.79**
	Full adjustment	1.30	1.15	1.46	44.89	1.41	1.10	1.82	0.00	1.18	0.50	2.77	59.02	1.09	0.77	1.55	18.15
**Age**																	
16-24y vs 45-54y	Unadjusted	0.77	0.41	1.47	71.12	0.55	0.26	1.20	55.5	1.17	0.34	4.05	86.43	0.48	0.34	0.68	
	**Basic adjustment**	**0.76**	**0.39**	**1.46**	**71.95**	**0.55**	**0.24**	**1.23**	**58.53**	**1.09**	**0.33**	**3.67**	**85.53**	**0.47**	**0.33**	**0.66**	
	Full adjustment	0.85	0.32	2.24	70.28	0.89	0.18	4.36		4.71	1.40	15.86		no information			
25-34y vs 45-54y	Unadjusted	0.87	0.71	1.07	51.9	0.71	0.53	0.96	72.25	1.23	0.75	2.03	74.54	0.78	0.59	1.02	0
	**Basic adjustment**	**0.85**	**0.70**	**1.04**	**47.62**	**0.67**	**0.57**	**0.77**	**0**	**1.16**	**0.68**	**1.96**	**76.95**	**0.77**	**0.58**	**1.03**	**0**
	Full adjustment	0.92	0.74	1.15	43.99	1.21	0.66	2.22		1.18	0.52	2.67	0	no information			
35-44y vs 45-54y	Unadjusted	0.93	0.74	1.16	67.57	0.77	0.66	0.89	23.09	1.06	0.75	1.51	57.05	0.99	0.71	1.36	42.96
	**Basic adjustment**	**0.92**	**0.74**	**1.15**	**68.24**	**0.78**	**0.65**	**0.92**	**35.96**	**1.04**	**0.74**	**1.47**	**56.72**	**1.03**	**0.67**	**1.58**	**61.87**
	Full adjustment	1.03	0.81	1.29	59.88	1.03	0.59	1.78		1.25	0.62	2.52	0	1.36	0.93	2.00	0
55-64y vs 45-54y	Unadjusted	1.16	0.98	1.38	63.54	1.33	1.21	1.47	0	0.79	0.57	1.08	61.08	1.17	0.65	2.10	91.47
	**Basic adjustment**	**1.18**	**0.99**	**1.39**	**64.04**	**1.35**	**1.22**	**1.49**	**0**	**0.79**	**0.57**	**1.10**	**65.97**	**1.18**	**0.66**	**2.10**	**89.83**
	Full adjustment	1.17	1.05	1.29	0	1.55	0.62	3.91	49.89	0.85	0.48	1.52	0	no information			
65-74y vs 45-54y	Unadjusted	1.36	1.11	1.67	75.24	1.61	1.46	1.78	0	0.73	0.48	1.11	79.59	1.93	1.67	2.23	0
	**Basic adjustment**	**1.39**	**1.13**	**1.72**	**77.16**	**1.65**	**1.49**	**1.82**	**0**	**0.75**	**0.49**	**1.16**	**80.63**	**1.95**	**1.68**	**2.26**	**0**
	Full adjustment	1.33	1.19	1.49	0	1.98	0.48	8.10	73.94	1.15	0.65	2.04	0	1.57	1.07	2.31	0
75y+ vs 45-54y	Unadjusted	1.45	0.92	2.29	90.02	1.83	1.59	2.12	0	0.66	0.46	0.94	47.94	2.05	1.59	2.64	28.83
	**Basic adjustment**	**1.50**	**0.93**	**2.39**	**91.23**	**1.89**	**1.65**	**2.17**	**0**	**0.69**	**0.47**	**1.01**	**53.79**	**2.07**	**1.66**	**2.59**	**17.97**
	Full adjustment	1.16	0.86	1.58	62.75	1.07	0.44	2.61		0.78	0.39	1.57	1.68	1.75	1.17	2.62	
**Ethnicity**																	

For peer review only - http://bmjopen.bmj.com/site/about/guidelines.xhtml

Non-White vs White*	Unadjusted	1.02	0.89	1.18	10.29	0.95	0.72	1.25	56.69	1.36	0.79	2.33	79.57	0.89	0.71	1.12	0
	**Basic adjustment**	**1.19**	**1.05**	**1.35**	**0**	**1.10**	**0.86**	**1.39**	**42.54**	**1.32**	**0.82**	**2.12**	**71.12**	**1.14**	**0.91**	**1.44**	**0**
	Full adjustment	1.09	0.96	1.25	0	1.15	0.83	1.61	0	1.97	1.08	3.62	0	1.68	0.36	7.76	62.98
Black vs White	Unadjusted	1.16	0.87	1.55	0	0.95	0.54	1.64	33.95	0.63	0.13	3.06	81.71	0.87	0.58	1.29	0
	**Basic adjustment**	**1.38**	**1.03**	**1.84**	**0**	**1.01**	**0.54**	**1.92**	**49.83**	**0.64**	**0.14**	**2.87**	**80**	**1.03**	**0.68**	**1.55**	**0**
	Full adjustment	1.15	0.86	1.53	4.01	0.81	0.23	2.83	0	0.37	0.04	3.11		0.87	0.41	1.82	
East Asian vs White	Unadjusted	0.97	0.56	1.68	0	0.61	0.28	1.30	12.51	2.34	0.39	14.15	88.19	1.23	0.48	3.15	0
	**Basic adjustment**	**1.13**	**0.67**	**1.90**	**0**	**0.80**	**0.39**	**1.64**	**17.46**	**1.95**	**0.53**	**7.24**	**79.39**	**1.61**	**0.61**	**4.22**	**0**
	Full adjustment	0.96	0.60	1.51	0	1.19	0.26	5.51			no information				no information		
Mixed vs White	Unadjusted	1.05	0.79	1.38	0	1.02	0.55	1.89	64.07	1.51	0.99	2.30	0	1.05	0.66	1.67	0
	**Basic adjustment**	**1.24**	**0.86**	**1.78**	**27.61**	**1.22**	**0.69**	**2.15**	**56.82**	**1.34**	**0.82**	**2.18**	**16.29**	**1.34**	**0.76**	**2.36**	**20.19**
	Full adjustment	1.25	0.88	1.77	15.86	1.61	0.80	3.22	51.21	0.93	0.10	8.48		0.85	0.32	2.21	
South Asian vs White	Unadjusted	0.85	0.61	1.18	64.69	0.92	0.59	1.42	67.42	0.99	0.36	2.72	89.3	0.68	0.45	1.04	18
	**Basic adjustment**	**1.05**	**0.84**	**1.32**	**28.25**	**1.11**	**0.88**	**1.39**	**12.68**	**0.98**	**0.38**	**2.54**	**87.94**	**0.89**	**0.64**	**1.24**	**0**
	Full adjustment	0.93	0.67	1.30	57.84	1.03	0.29	3.63		2.81	1.19	6.63	11.86	1.11	0.62	1.99	
Other Ethnicity vs White	Unadjusted	0.79	0.46	1.34	28.89	0.91	0.58	1.45	0	1.23	0.56	2.67	25.72	0.84	0.20	3.48	44.62
	**Basic adjustment**	**0.90**	**0.49**	**1.63**	**44.27**	**1.07**	**0.68**	**1.68**	**0**	**1.45**	**0.81**	**2.60**	**0**	**0.95**	**0.23**	**4.03**	**52.56**
	Full adjustment	0.82	0.45	1.50	35.18	1.97	1.08	3.62	0.00	3.74	0.39	35.91			no information		
**Education**																	
A-level/equivalent vs Higher education/Degree	Unadjusted	1.04	0.91	1.17	58.16	1.03	0.91	1.17	42.88	0.94	0.77	1.15	21.54	0.84	0.55	1.29	88.56
	**Basic adjustment**	**1.08**	**0.97**	**1.20**	**38.7**	**1.07**	**0.99**	**1.16**	**0**	**1.02**	**0.82**	**1.28**	**27.13**	**0.87**	**0.58**	**1.30**	**85.92**
	Full adjustment	1.01	0.92	1.11	0	0.97	0.76	1.25	0	1.61	0.63	4.12	59.22	1.03	0.82	1.29	0
GCSE/equivalent vs Higher education/Degree	Unadjusted	0.99	0.87	1.14	62.23	1.03	0.95	1.12	0	0.98	0.75	1.27	53.81	1.03	0.91	1.16	0
	**Basic adjustment**	**1.00**	**0.87**	**1.14**	**59.18**	**1.01**	**0.91**	**1.12**	**15.22**	**1.04**	**0.77**	**1.39**	**62.34**	**1.05**	**0.93**	**1.18**	**0**
	Full adjustment	0.91	0.81	1.02	48.6	0.86	0.66	1.12	0	1.01	0.63	1.61	1.57	0.82	0.64	1.05	0
<GCSE/equivalent vs Higher education/Degree	Unadjusted	1.06	0.88	1.28	76.55	1.09	0.92	1.29	52.88	1.07	0.74	1.54	69.78	1.45	1.28	1.64	0
	**Basic adjustment**	**1.05**	**0.91**	**1.21**	**53.17**	**1.01**	**0.86**	**1.18**	**42.42**	**1.17**	**0.82**	**1.67**	**63.42**	**1.26**	**1.11**	**1.44**	**0**
	Full adjustment	0.87	0.75	1.00	34.17	0.90	0.54	1.50	58.54	1.17	0.39	3.49	60.69	1.53	0.34	6.85	71.25
**Occupational class**																	

For peer review only - http://bmjopen.bmj.com/site/about/guidelines.xhtml

Intermediate vs Managerial/Admin/Professional	Unadjusted	1.08	1.00	1.16	0	1.01	0.91	1.14	22.79	1.09	0.83	1.42	41.19	1.19	1.05	1.34	0
	**Basic adjustment**	**1.07**	**0.99**	**1.15**	**0**	**1.01**	**0.93**	**1.10**	**0**	**1.10**	**0.88**	**1.38**	**26.19**	**1.16**	**1.03**	**1.31**	**0**
	Full adjustment	1.00	0.92	1.08	0	1.01	0.78	1.30	0	0.73	0.34	1.61	0	0.78	0.42	1.47	48.08
Manual/Routine vs Managerial/Admin/Professional	Unadjusted	1.13	1.03	1.23	12.12	1.04	0.90	1.20	36.16	1.38	1.16	1.64	0	1.11	0.91	1.35	25.25
	**Basic adjustment**	**1.17**	**1.08**	**1.27**	**0**	**1.07**	**0.93**	**1.23**	**28.46**	**1.36**	**1.11**	**1.67**	**8.93**	**1.17**	**1.03**	**1.33**	**0**
	Full adjustment	1.02	0.93	1.12	0	1.10	0.84	1.44	6.05	0.51	0.18	1.43	23.27	0.92	0.56	1.50	20.63
Other social class vs Managerial/Admin/Professional	Unadjusted	1.47	1.02	2.13	89.12	1.41	1.08	1.84	66.23	2.16	1.30	3.57	76.1	1.71	0.94	3.10	87.16
	**Basic adjustment**	**1.51**	**1.12**	**2.04**	**79.69**	**1.46**	**1.16**	**1.84**	**47.81**	**2.45**	**1.72**	**3.50**	**45.5**	**1.81**	**1.17**	**2.80**	**73.85**
	Full adjustment	1.19	1.00	1.43	39.12	1.30	0.85	1.99	0.00	1.42	0.13	15.78	76.18	0.94	0.69	1.27	

Basic adjustment: sex, age, and ethnicity (where available)

Full adjustment: sex, age, and ethnicity (where available) education, occupational class, UK Nation (where appropriate), household composition, and pre-pandemic self-reported health.

Empty I^2% column indicates only one study included

*Binary variable including Black, East Asian, Mixed, South Asian, and other ethnicity in 'non-White'

For peer review only

Summary of stratified results

		Any healthcare disruption			
**Sex**		OR	Lower CI	Upper CI	I2%
Female vs. Male	Overall	1.27	1.15	1.40	53.11
	Not shielding	1.26	1.12	1.43	61.12
	Shielding	1.37	1.15	1.63	0
	16-24y	2.22	1.63	3.02	0
	25-34y	1.56	1.30	1.87	0
	35-44y	1.51	1.23	1.86	0
	45-54	1.72	1.35	2.18	36.61
	55-64	1.09	0.92	1.30	59.58
	75+	1.08	0.90	1.30	20
**Age**		OR	Lower CI	Upper CI	I2%
16-24y vs 45-54y	Overall	0.76	0.39	1.46	71.95
	Not shielding	0.79	0.40	1.56	70.32
	Shielding	0.64	0.23	1.78	
25-34y vs 45-54y	Overall	0.85	0.70	1.04	47.62
	Not shielding	0.86	0.70	1.06	43.4
	Shielding	1.09	0.61	1.95	0
35-44y vs 45-54y	Overall	0.92	0.74	1.15	68.24
	Not shielding	0.95	0.74	1.21	68.26
	Shielding	0.68	0.34	1.34	47.41
55-64y vs 45-54y	Overall	1.18	0.99	1.39	64.04
	Not shielding	1.21	1.02	1.43	53.82
	Shielding	1.24	0.87	1.77	0
65-74y vs 45-54y	Overall	1.39	1.13	1.72	77.16
	Not shielding	1.44	1.20	1.72	64.1
	Shielding	1.11	0.79	1.56	0
75y+ vs 45-54y	Overall	1.50	0.93	2.39	91.23
	Not shielding	1.61	1.17	2.22	79.38
	Shielding	0.83	0.51	1.37	32.84
**Ethnicity**		OR	Lower CI	Upper CI	I2%
Non-White vs White*	Overall	1.19	1.05	1.35	0
	Not shielding	1.06	0.86	1.31	41.46
	Shielding	1.62	1.08	2.43	0
	16-24y	1.30	0.89	1.89	0
	25-34y	0.92	0.65	1.29	36.48
	35-44y	1.31	1.01	1.71	0
	45-54	1.61	1.16	2.22	0
	55-64	1.13	0.85	1.50	0

For peer review only
For peer review only - http://bmjopen.bmj.com/site/about/guidelines.xhtml

	75+	1.28	0.67	2.45	0
Black vs White	Overall	1.38	1.03	1.84	0
	Not shielding	0.80	0.43	1.49	58.06
	Shielding	1.60	0.67	3.83	0
	16-24y	1.15	0.51	2.59	0
	25-34y	0.82	0.40	1.68	0
	35-44y	1.91	0.81	4.48	0
	45-54	1.99	0.93	4.25	15.25
	55-64	1.69	1.00	2.84	0
	75+	1.23	0.42	3.56	0
East Asian vs White	Overall	1.13	0.67	1.90	0
	Not shielding	0.95	0.54	1.68	0
	Shielding		no information		
	16-24y	0.01	0.00	0.05	
	25-34y	0.62	0.20	1.92	0
	35-44y	1.63	0.80	3.32	0
	45-54	1.75	0.54	5.64	0
	55-64	0.96	0.43	2.15	0
	75+		no information		
Mixed vs White	Overall	1.24	0.86	1.78	27.61
	Not shielding	1.18	0.85	1.62	0
	Shielding	1.85	0.71	4.77	0
	16-24y	2.50	1.25	5.02	0
	25-34y	1.26	0.79	2.02	0
	35-44y	1.15	0.23	5.69	73.12
	45-54	0.92	0.46	1.87	0
	55-64	1.06	0.53	2.11	0
	75+	1.47	0.34	6.42	22.46
South Asian vs White	Overall	1.05	0.84	1.32	28.25
	Not shielding	0.98	0.75	1.28	35.03
	Shielding	1.44	0.87	2.38	0
	16-24y	0.98	0.62	1.53	13.95
	25-34y	0.80	0.38	1.71	74.73
	35-44y	1.11	0.80	1.55	10.1
	45-54	1.67	0.43	6.48	82
	55-64	0.82	0.44	1.56	14.81
	75+	1.11	0.40	3.12	0
Other Ethnicity vs White	Overall	0.90	0.49	1.63	44.27
	Not shielding	0.85	0.45	1.62	43.11
	Shielding	0.75	0.11	4.96	10.15
	16-24y	0.18	0.00	15.35	88.56
	25-34y	0.80	0.31	2.08	49.28

For peer review only - http://bmjopen.bmj.com/site/about/guidelines.xhtml

		OR	Lower CI	Upper CI	I2%
	35-44y	1.41	0.58	3.40	0
	45-54	1.74	0.56	5.45	29.75
	55-64	0.77	0.27	2.22	0
	75+	4.18	0.35	50.04	
**Education**		OR	Lower CI	Upper CI	I2%
A-level/equivalent vs Higher education/Degree	Overall	1.08	0.97	1.20	38.7
	Not shielding	1.09	0.96	1.23	39.28
	Shielding	0.95	0.74	1.22	0
	16-24y	1.33	0.93	1.90	0
	25-34y	0.99	0.69	1.42	62.16
	35-44y	1.62	1.28	2.05	0
	45-54	1.13	0.96	1.34	0
	55-64	1.01	0.89	1.14	0
	75+	0.96	0.65	1.40	57.49
GCSE/equivalent vs Higher education/Degree	Overall	1.00	0.87	1.14	59.18
	Not shielding	0.99	0.84	1.17	64.95
	Shielding	0.80	0.62	1.04	0
	16-24y	0.94	0.49	1.81	64.06
	25-34y	1.24	0.80	1.94	69.1
	35-44y	1.26	0.97	1.63	0
	45-54	1.16	0.83	1.62	62.52
	55-64	1.03	0.91	1.17	0
	75+	0.92	0.65	1.30	35.17
<GCSE/equivalent vs Higher education/Degree	Overall	1.05	0.91	1.21	53.17
	Not shielding	1.02	0.88	1.19	46.14
	Shielding	0.87	0.68	1.11	0
	16-24y	0.77	0.47	1.28	11.51
	25-34y	0.99	0.67	1.45	42.2
	35-44y	1.03	0.74	1.43	0
	45-54	1.48	1.08	2.04	34.96
	55-64	1.20	1.03	1.41	0
	75+	0.96	0.78	1.20	0
**Occupational class**		OR	Lower CI	Upper CI	I2%
Intermediate vs Managerial/Admin/Professional	Overall	1.07	0.99	1.15	0
	Not shielding	1.07	0.98	1.16	0
	Shielding	0.87	0.65	1.16	7.88
	16-24y	0.92	0.60	1.41	0
	25-34y	1.04	0.84	1.29	10.79
	35-44y	1.28	0.92	1.78	46.81
	45-54	1.12	0.94	1.33	0
	55-64	1.01	0.86	1.19	22.02

For peer review only - http://bmjopen.bmj.com/site/about/guidelines.xhtml

	75+	1.00	0.76	1.33	0
Manual/Routine vs Managerial/Admin/Professional	Overall	1.17	1.08	1.27	0
	Not shielding	1.18	1.07	1.29	0
	Shielding	0.93	0.71	1.21	0
	16-24y	1.15	0.77	1.71	0
	25-34y	1.11	0.80	1.55	50.55
	35-44y	1.24	0.95	1.63	0
	45-54	1.08	0.90	1.30	0
	55-64	1.16	1.00	1.35	0
	75+	1.27	0.96	1.67	0
Other social class vs Managerial/Admin/Professional	Overall	1.51	1.12	2.04	79.69
	Not shielding	1.48	1.04	2.09	83.37
	Shielding	0.89	0.39	2.07	78.87
	16-24y	1.02	0.46	2.26	58.35
	25-34y	1.85	1.29	2.64	0
	35-44y	1.44	0.55	3.80	68.27
	45-54	2.05	0.98	4.29	85.15
	55-64	1.65	1.21	2.27	60.63
	75+	1.02	0.62	1.69	0

Adjusted for sex, age, and ethnicity (where available)

Empty I^2% column indicates only one study included

*Binary variable including Black, East Asian, Mixed, South Asian, and other ethnicity in 'non-White'

For peer review only - http://bmjopen.bmj.com/site/about/guidelines.xhtml

Any healthcare disruption

Sex

Unadjusted

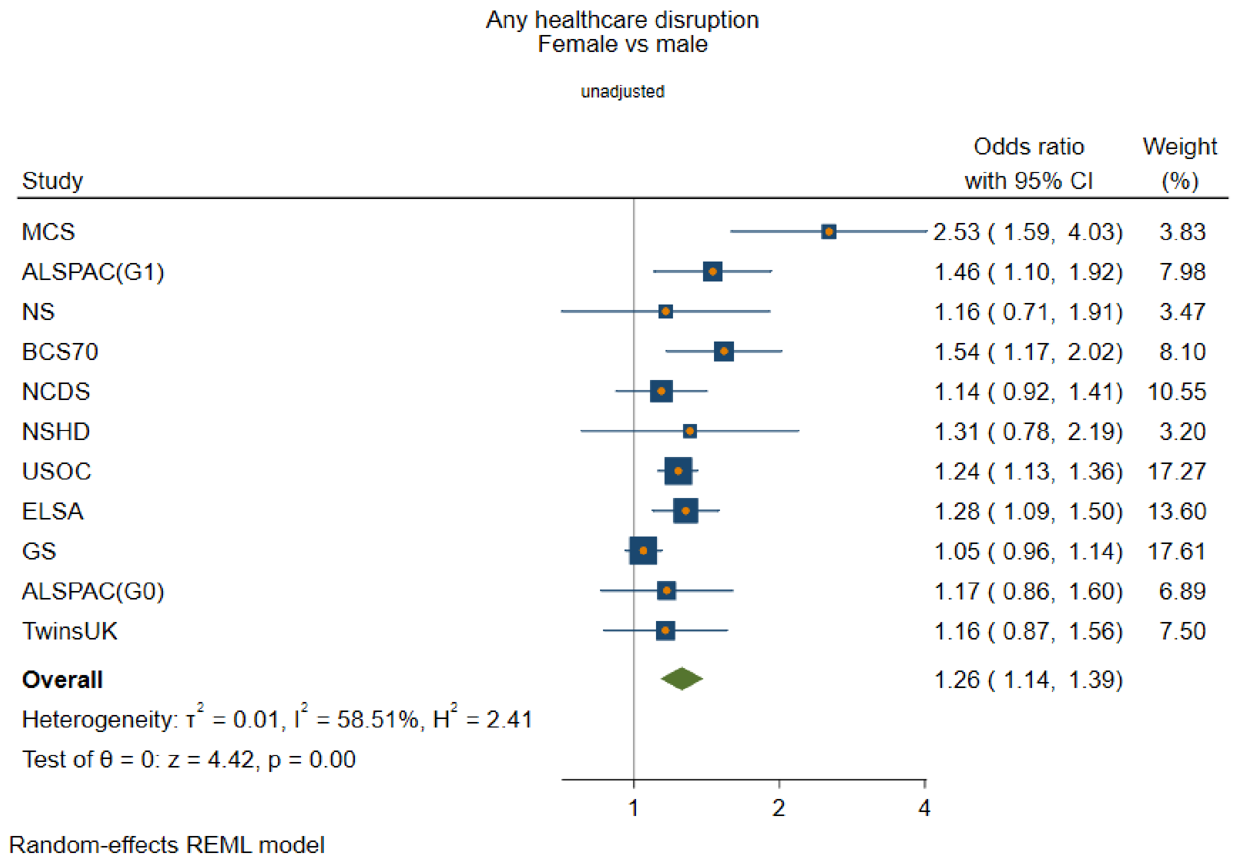

only

For peer review only - http://bmjopen.bmj.com/site/about/guidelines.xhtml

Basic adjustment

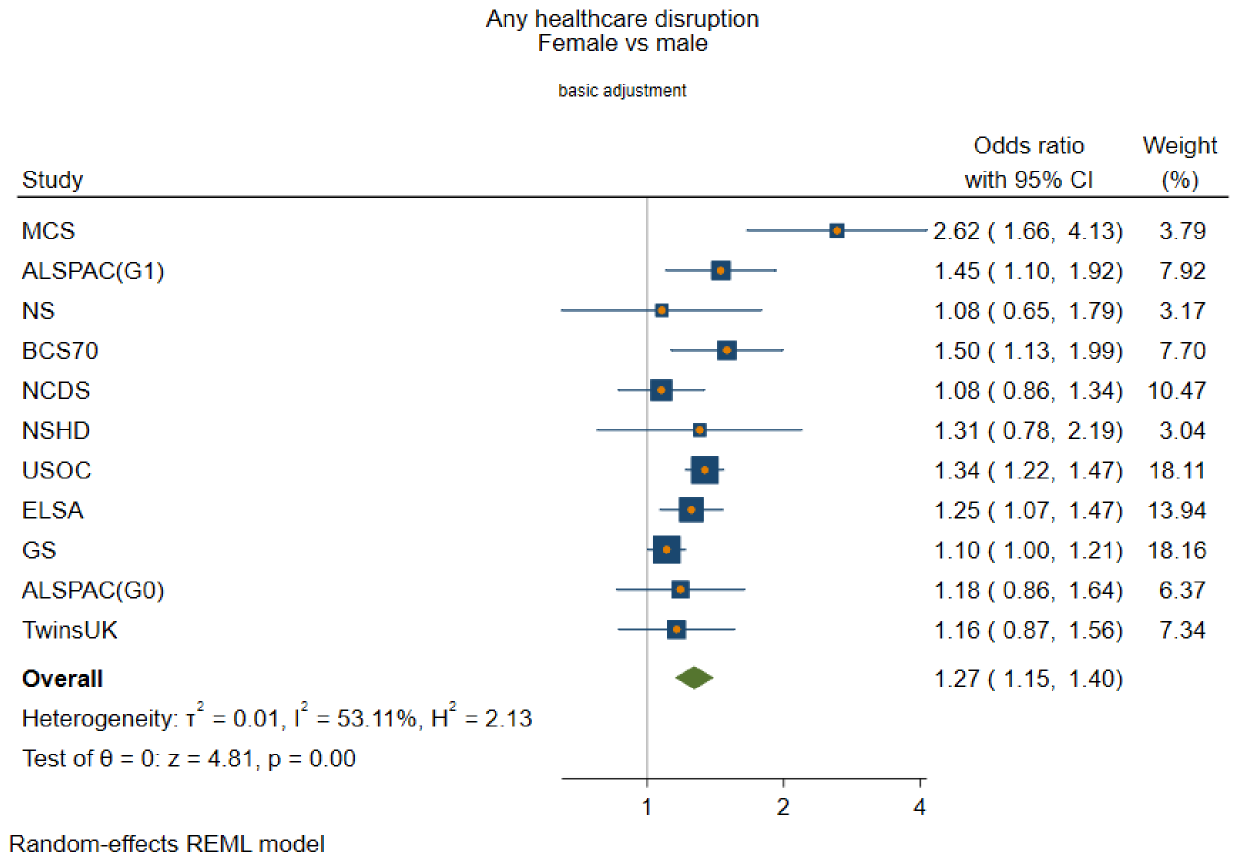

For peer review only - http://bmjopen.bmj.com/site/about/guidelines.xhtml

Full adjustment

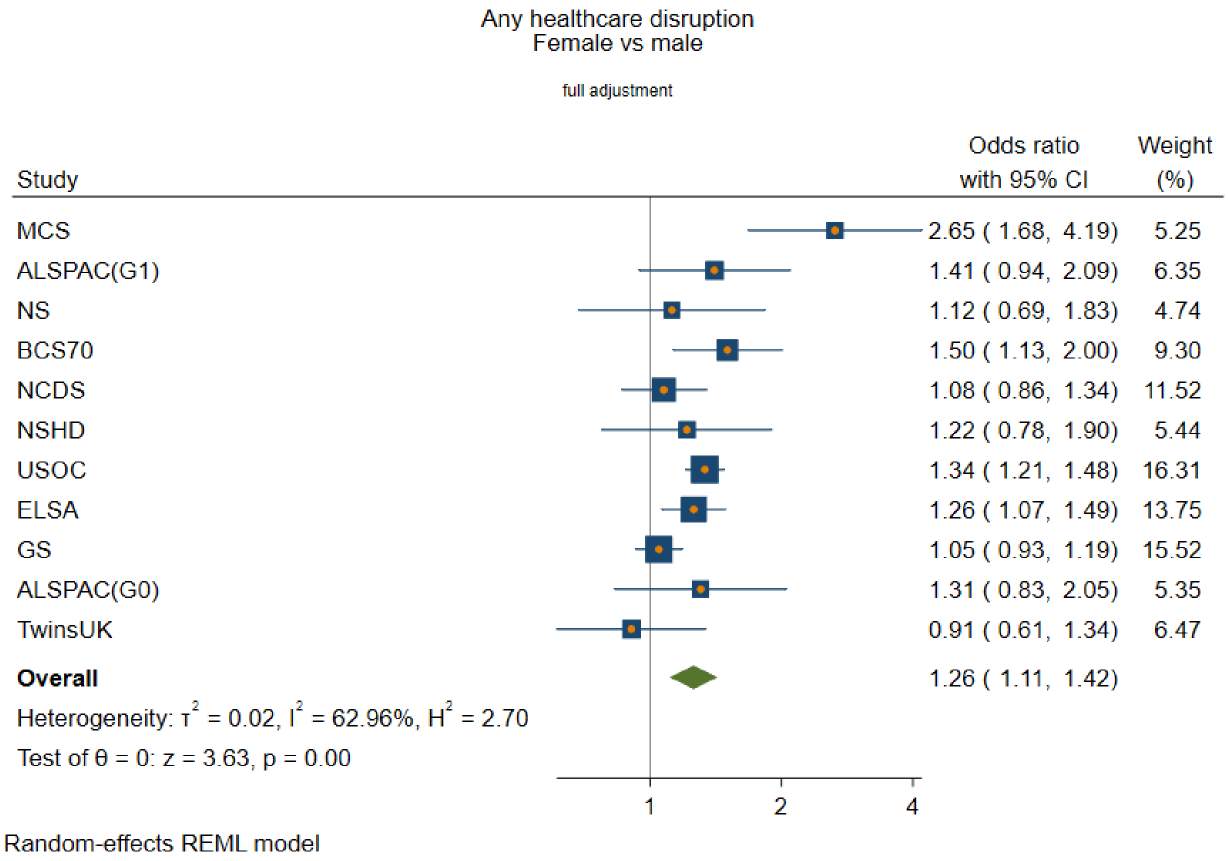

For peer review only - http://bmjopen.bmj.com/site/about/guidelines.xhtml

Occupational class
Unadjusted

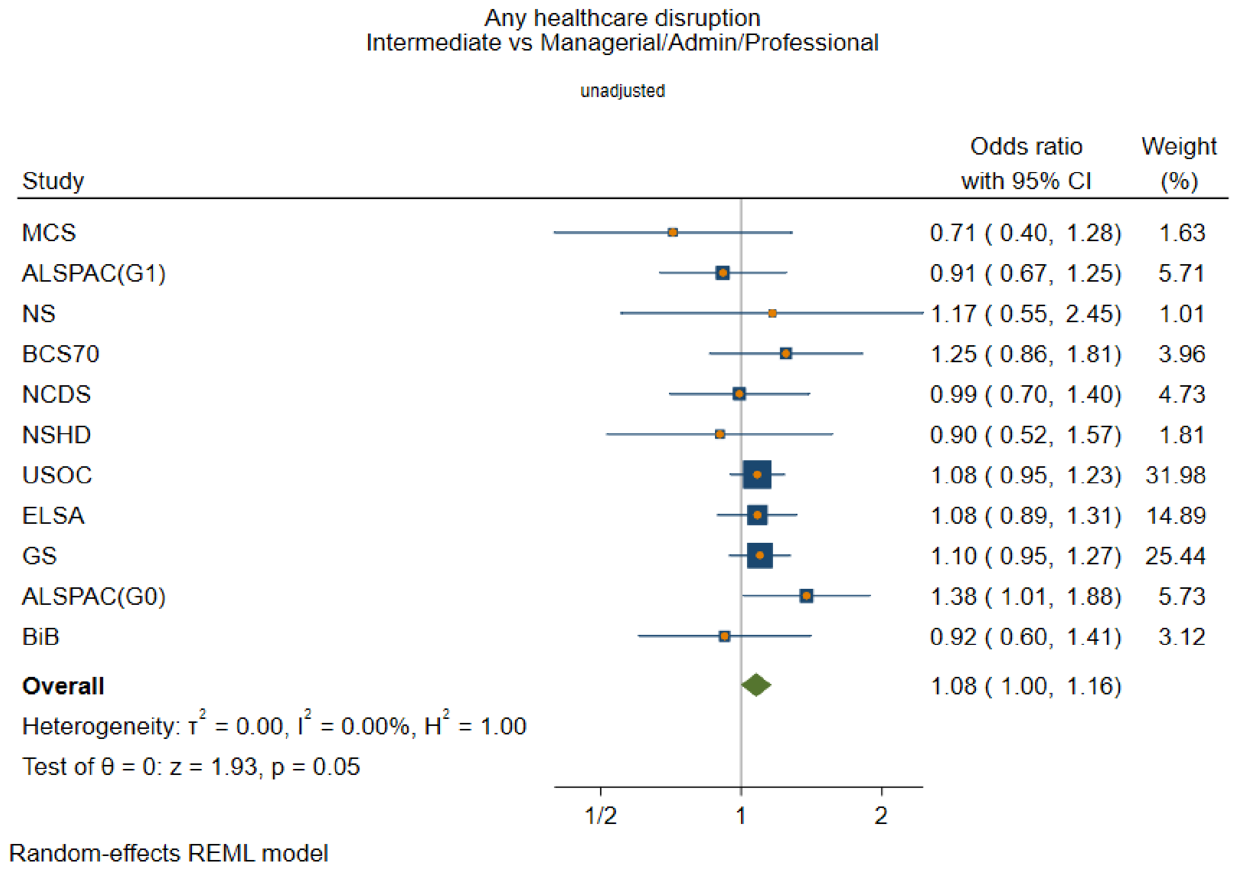

only

For peer review only - http://bmjopen.bmj.com/site/about/guidelines.xhtml

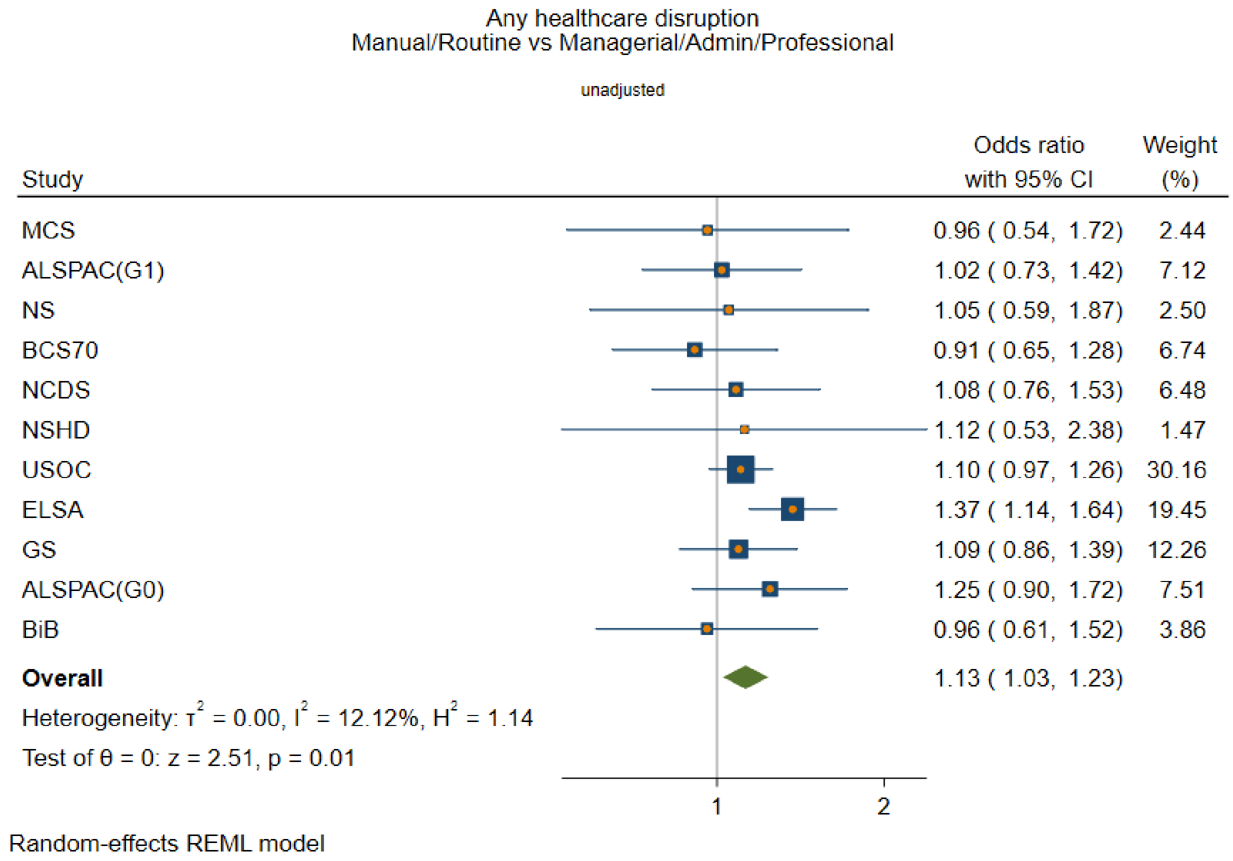
Any healthcare disruption
Manual/Routine vs Managerial/Admin/Professional
unadjusted
Study
Odds ratio with 95% CI
Weight (%)
MCS 0.96 (0.54, 1.72) 2.44
ALSPAC(G1) 1.02 (0.73, 1.42) 7.12
NS 1.05 (0.59, 1.87) 2.50
BCS70 0.91 (0.65, 1.28) 6.74
NCDS 1.08 (0.76, 1.53) 6.48
NSHD 1.12 (0.53, 2.38) 1.47
USOC 1.10 (0.97, 1.26) 30.16
ELSA 1.37 (1.14, 1.64) 19.45
GS 1.09 (0.86, 1.39) 12.26
ALSPAC(G0) 1.25 (0.90, 1.72) 7.51
BiB 0.96 (0.61, 1.52) 3.86
Overall 1.13 (1.03, 1.23)
Heterogeneity: τ² = 0.00, I² = 12.12%, H² = 1.14
Test of θ = 0: z = 2.51, p = 0.01
1
2
Random-effects REML model

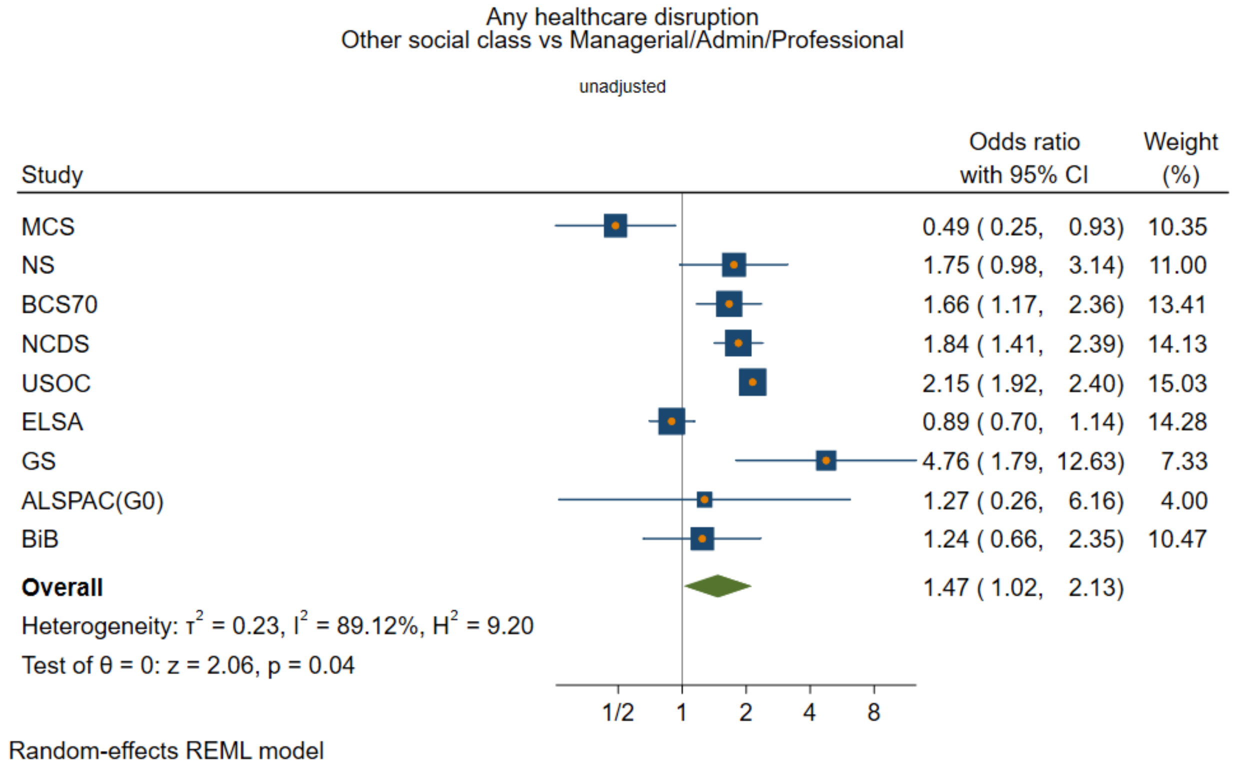
Any healthcare disruption
Other social class vs Managerial/Admin/Professional
unadjusted
Study
Odds ratio with 95% CI
Weight (%)
MCS 0.49 (0.25, 0.93) 10.35
NS 1.75 (0.98, 3.14) 11.00
BCS70 1.66 (1.17, 2.36) 13.41
NCDS 1.84 (1.41, 2.39) 14.13
USOC 2.15 (1.92, 2.40) 15.03
ELSA 0.89 (0.70, 1.14) 14.28
GS 4.76 (1.79, 12.63) 7.33
ALSPAC(G0) 1.27 (0.26, 6.16) 4.00
BiB 1.24 (0.66, 2.35) 10.47
Overall 1.47 (1.02, 2.13)
Heterogeneity: τ² = 0.23, I² = 89.12%, H² = 9.20
Test of θ = 0: z = 2.06, p = 0.04
1/2
1
2
4
8
Random-effects REML model

Basic adjustment

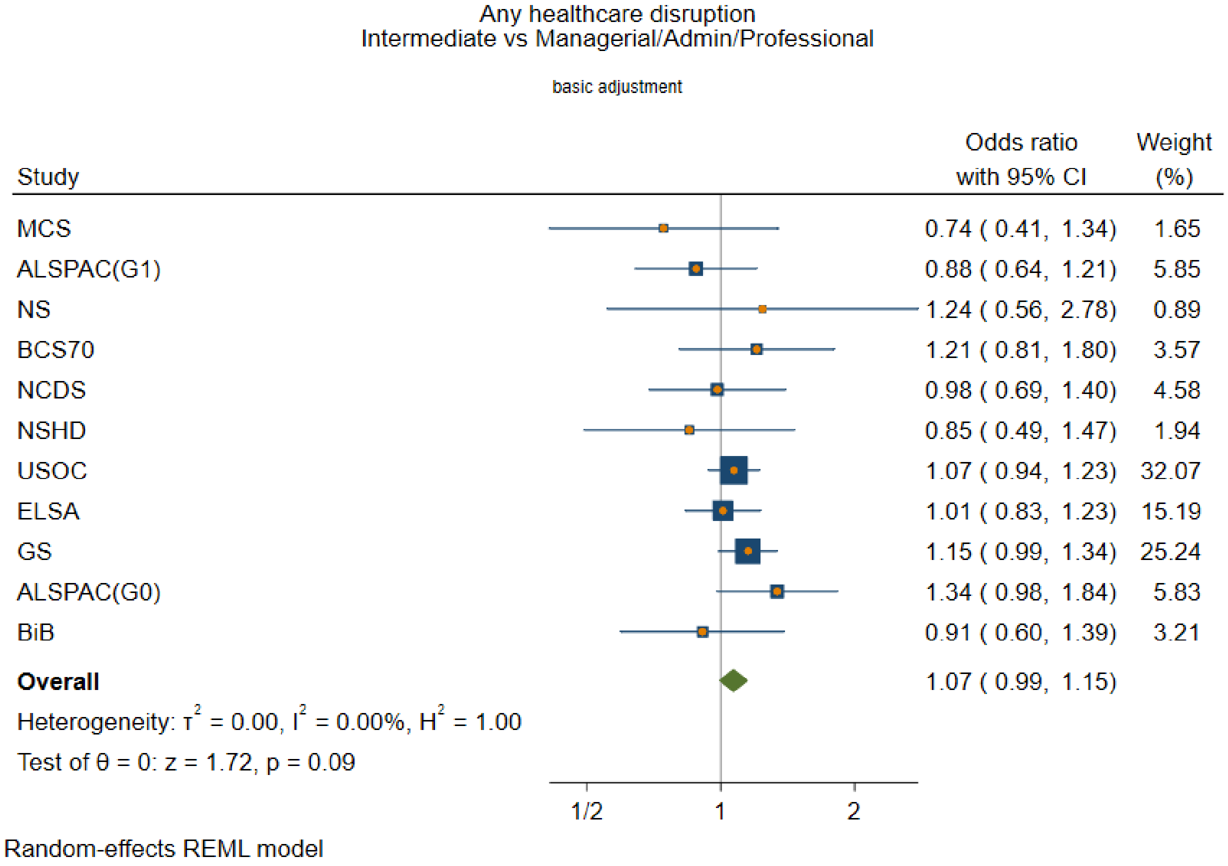

For peer review only - http://bmjopen.bmj.com/site/about/guidelines.xhtml

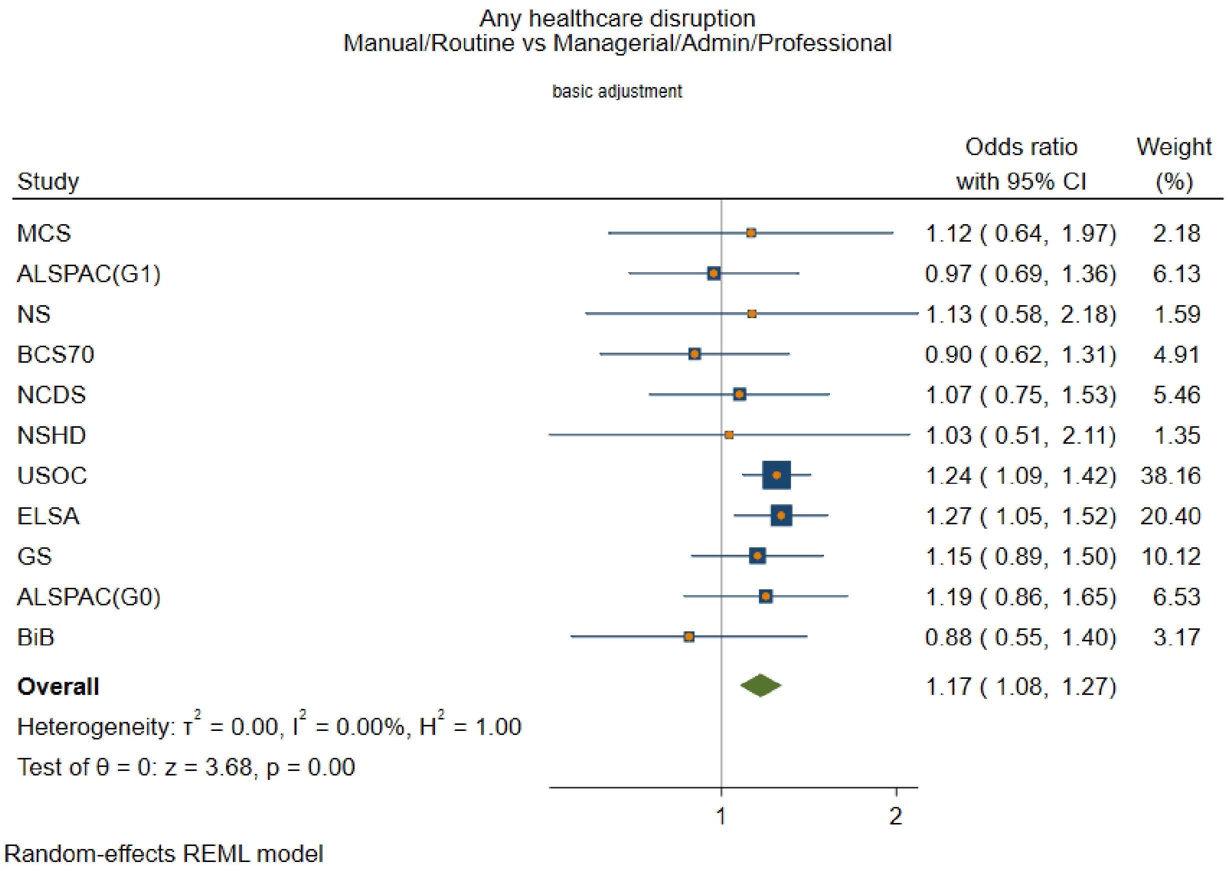
Any healthcare disruption
Manual/Routine vs Managerial/Admin/Professional
basic adjustment
Study
Odds ratio with 95% CI
Weight (%)
MCS 1.12 (0.64, 1.97) 2.18
ALSPAC(G1) 0.97 (0.69, 1.36) 6.13
NS 1.13 (0.58, 2.18) 1.59
BCS70 0.90 (0.62, 1.31) 4.91
NCDS 1.07 (0.75, 1.53) 5.46
NSHD 1.03 (0.51, 2.11) 1.35
USOC 1.24 (1.09, 1.42) 38.16
ELSA 1.27 (1.05, 1.52) 20.40
GS 1.15 (0.89, 1.50) 10.12
ALSPAC(G0) 1.19 (0.86, 1.65) 6.53
BiB 0.88 (0.55, 1.40) 3.17
Overall 1.17 (1.08, 1.27)
Heterogeneity: τ² = 0.00, I² = 0.00%, H² = 1.00
Test of θ = 0: z = 3.68, p = 0.00
1
2
Random-effects REML model

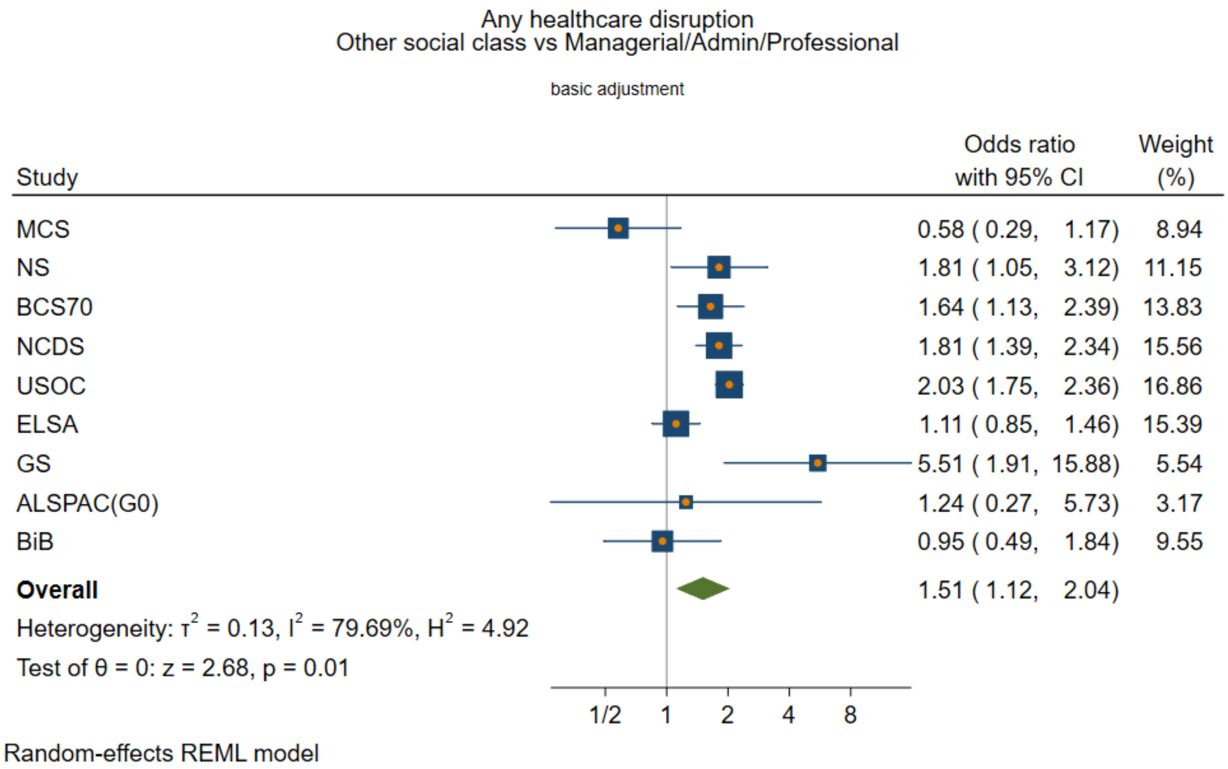
Any healthcare disruption
Other social class vs Managerial/Admin/Professional
basic adjustment
Study
Odds ratio with 95% CI
Weight (%)
MCS 0.58 (0.29, 1.17) 8.94
NS 1.81 (1.05, 3.12) 11.15
BCS70 1.64 (1.13, 2.39) 13.83
NCDS 1.81 (1.39, 2.34) 15.56
USOC 2.03 (1.75, 2.36) 16.86
ELSA 1.11 (0.85, 1.46) 15.39
GS 5.51 (1.91, 15.88) 5.54
ALSPAC(G0) 1.24 (0.27, 5.73) 3.17
BiB 0.95 (0.49, 1.84) 9.55
Overall 1.51 (1.12, 2.04)
Heterogeneity: τ² = 0.13, I² = 79.69%, H² = 4.92
Test of θ = 0: z = 2.68, p = 0.01
1/2
1
2
4
8
Random-effects REML model

Full adjustment

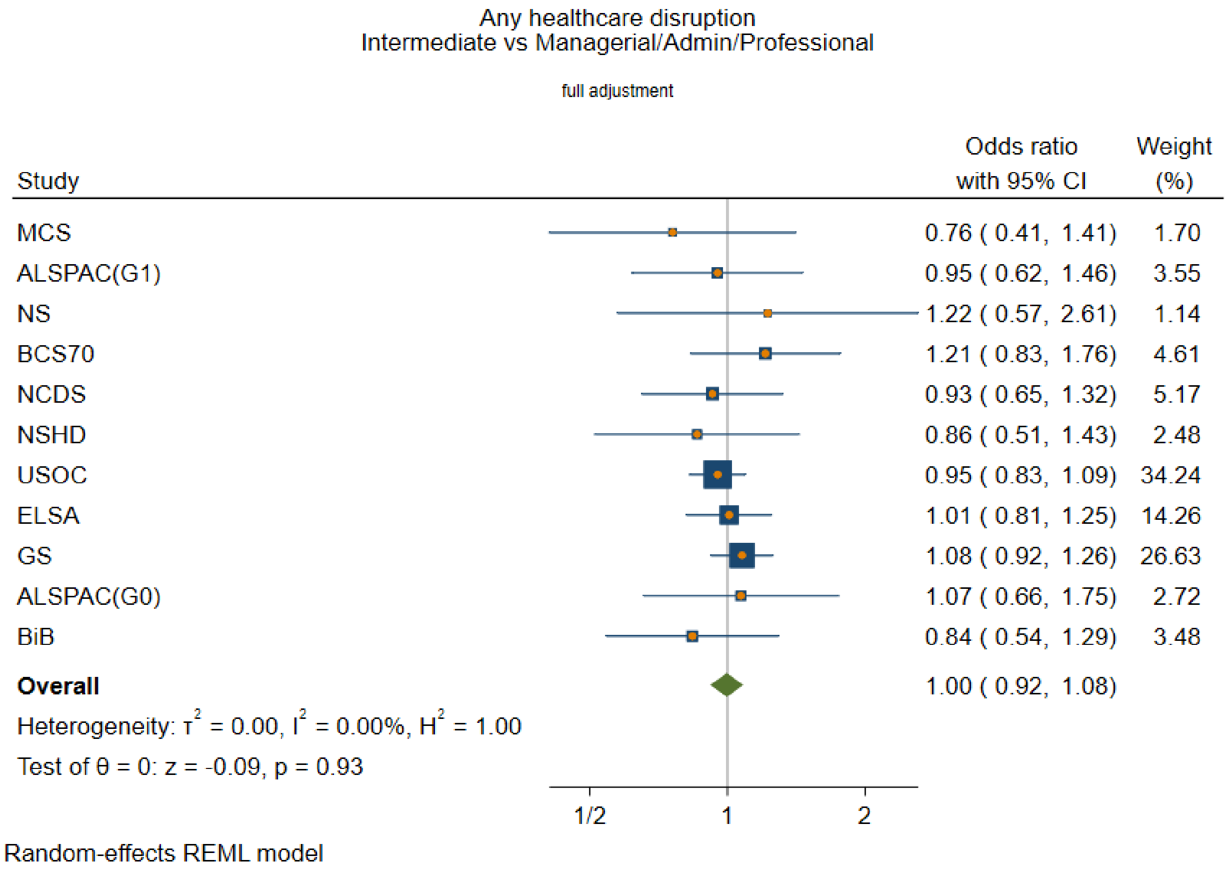

w only

For peer review only - http://bmjopen.bmj.com/site/about/guidelines.xhtml

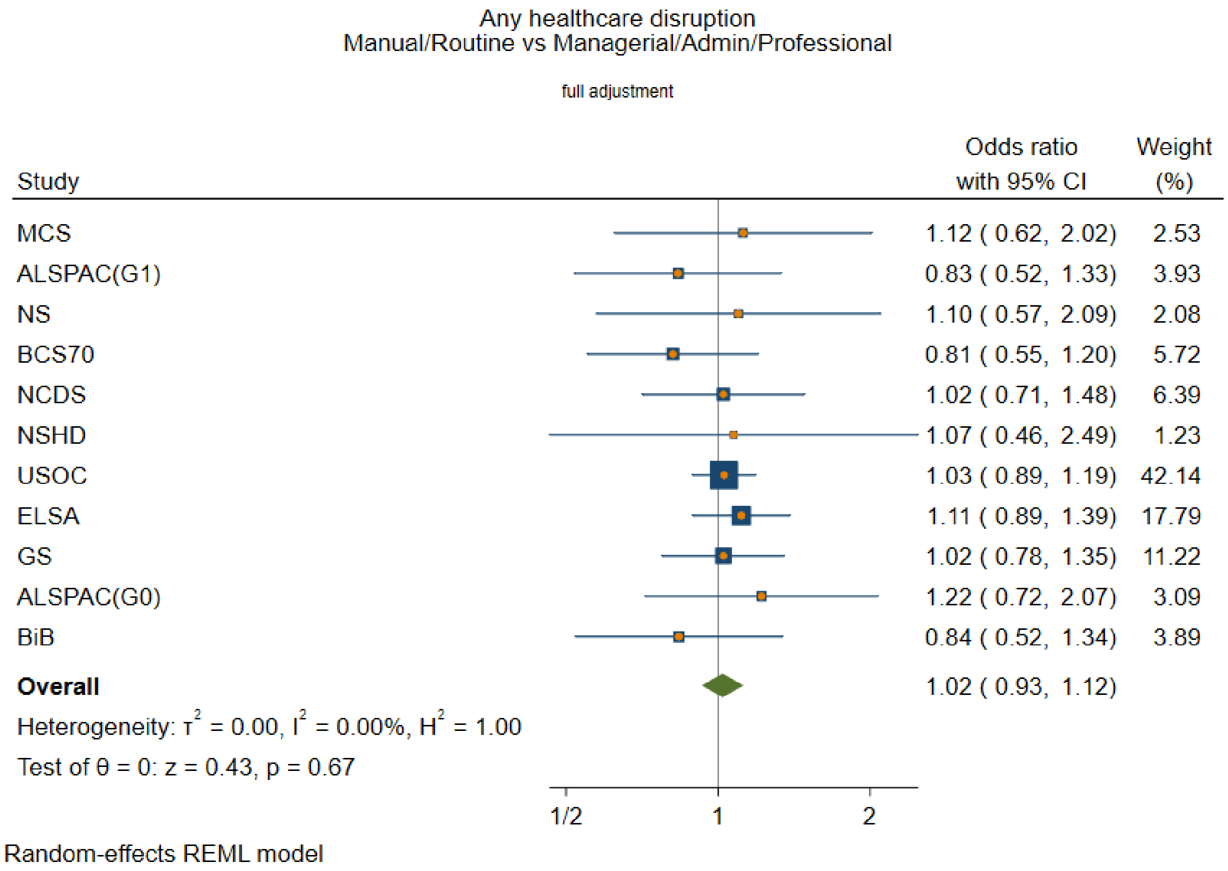

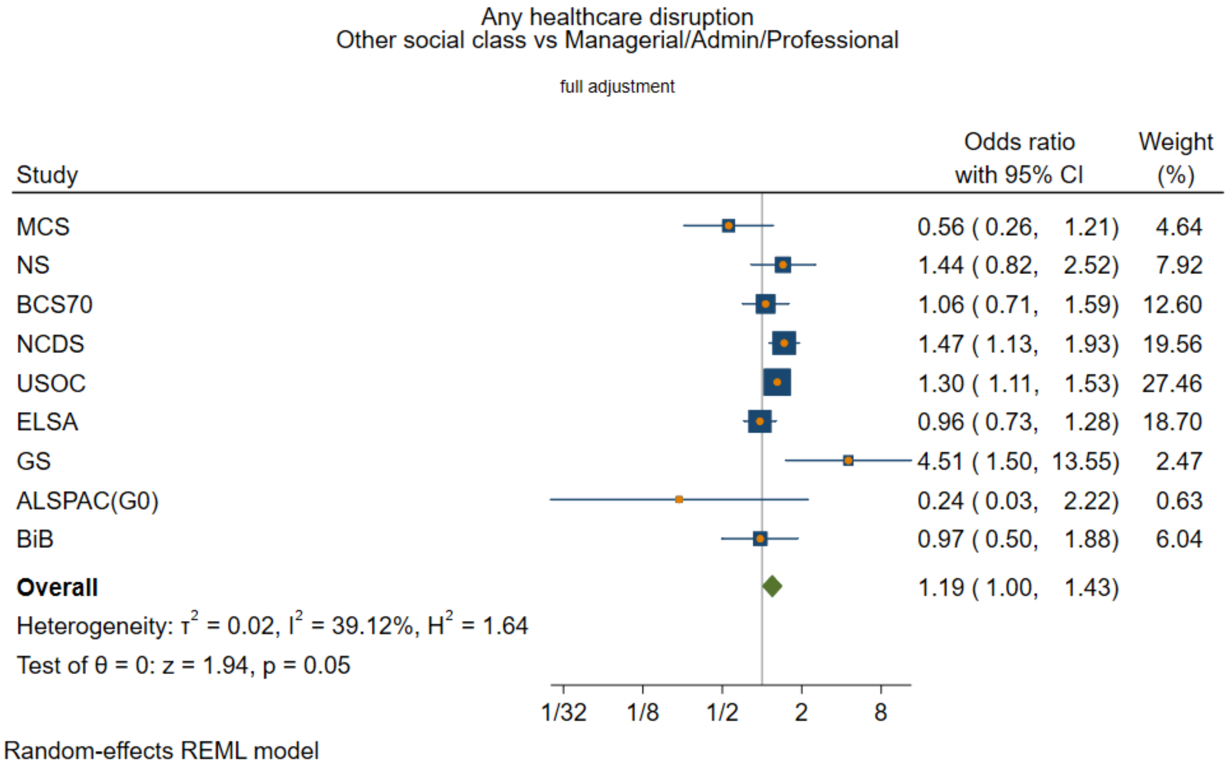

For peer review only - http://bmjopen.bmj.com/site/about/guidelines.xhtml

Education
Unadjusted

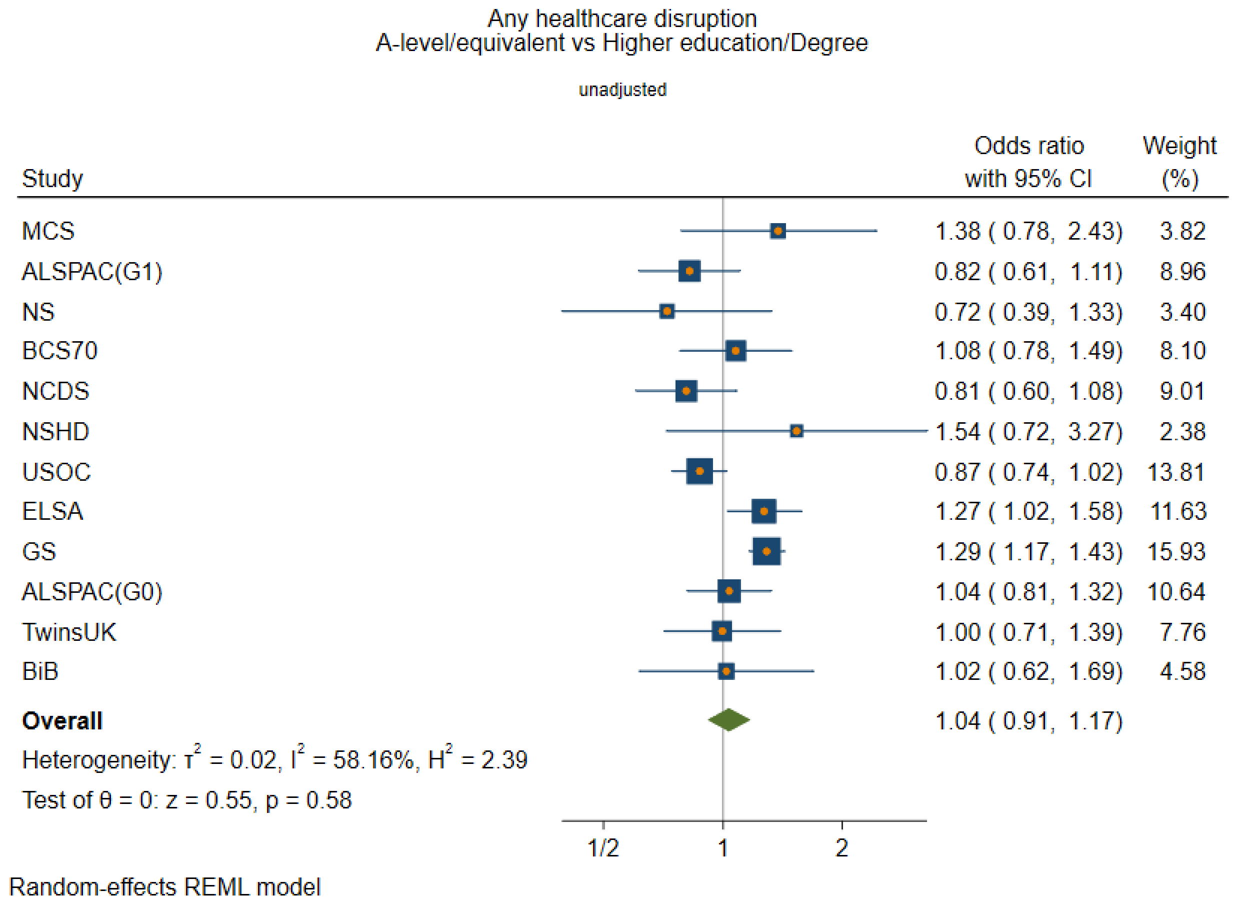

For peer review only - http://bmjopen.bmj.com/site/about/guidelines.xhtml

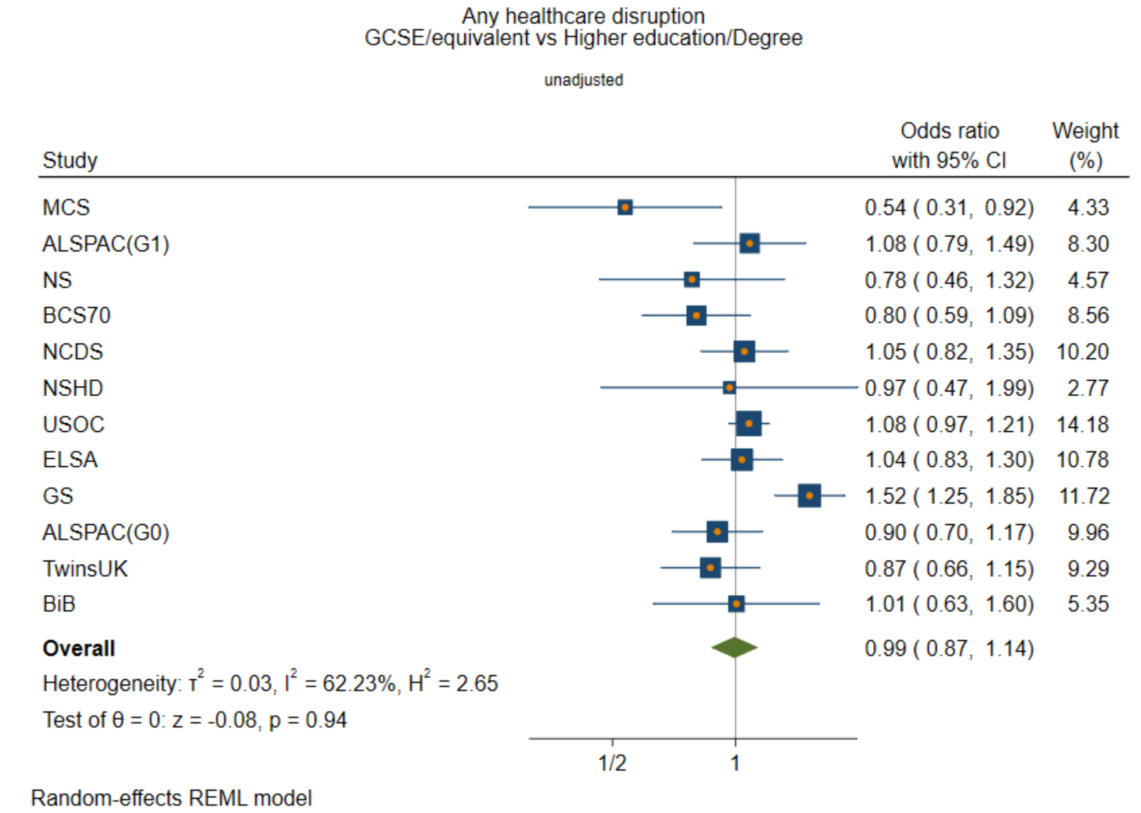
Any healthcare disruption
GCSE/equivalent vs Higher education/Degree
unadjusted
Study
Odds ratio with 95% CI
Weight (%)
MCS 0.54 (0.31, 0.92) 4.33
ALSPAC(G1) 1.08 (0.79, 1.49) 8.30
NS 0.78 (0.46, 1.32) 4.57
BCS70 0.80 (0.59, 1.09) 8.56
NCDS 1.05 (0.82, 1.35) 10.20
NSHD 0.97 (0.47, 1.99) 2.77
USOC 1.08 (0.97, 1.21) 14.18
ELSA 1.04 (0.83, 1.30) 10.78
GS 1.52 (1.25, 1.85) 11.72
ALSPAC(G0) 0.90 (0.70, 1.17) 9.96
TwinsUK 0.87 (0.66, 1.15) 9.29
BiB 1.01 (0.63, 1.60) 5.35
Overall 0.99 (0.87, 1.14)
Heterogeneity: τ^2 = 0.03, I^2 = 62.23%, H^2 = 2.65
Test of θ = 0: z = -0.08, p = 0.94
1/2
1
Random-effects REML model

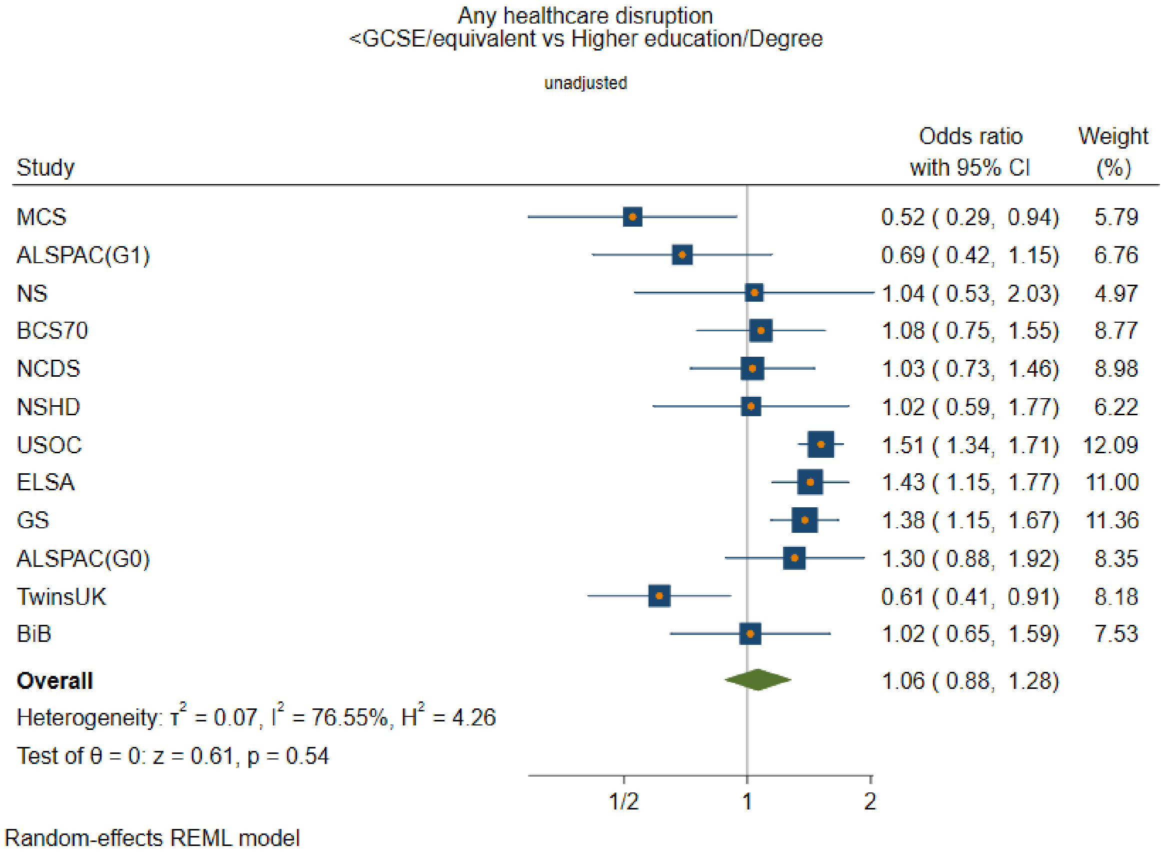
Any healthcare disruption
<GCSE/equivalent vs Higher education/Degree
unadjusted
Study
Odds ratio with 95% CI
Weight (%)
MCS 0.52 (0.29, 0.94) 5.79
ALSPAC(G1) 0.69 (0.42, 1.15) 6.76
NS 1.04 (0.53, 2.03) 4.97
BCS70 1.08 (0.75, 1.55) 8.77
NCDS 1.03 (0.73, 1.46) 8.98
NSHD 1.02 (0.59, 1.77) 6.22
USOC 1.51 (1.34, 1.71) 12.09
ELSA 1.43 (1.15, 1.77) 11.00
GS 1.38 (1.15, 1.67) 11.36
ALSPAC(G0) 1.30 (0.88, 1.92) 8.35
TwinsUK 0.61 (0.41, 0.91) 8.18
BiB 1.02 (0.65, 1.59) 7.53
Overall 1.06 (0.88, 1.28)
Heterogeneity: τ^2 = 0.07, I^2 = 76.55%, H^2 = 4.26
Test of θ = 0: z = 0.61, p = 0.54
1/2
1
2
Random-effects REML model

Basic adjustment

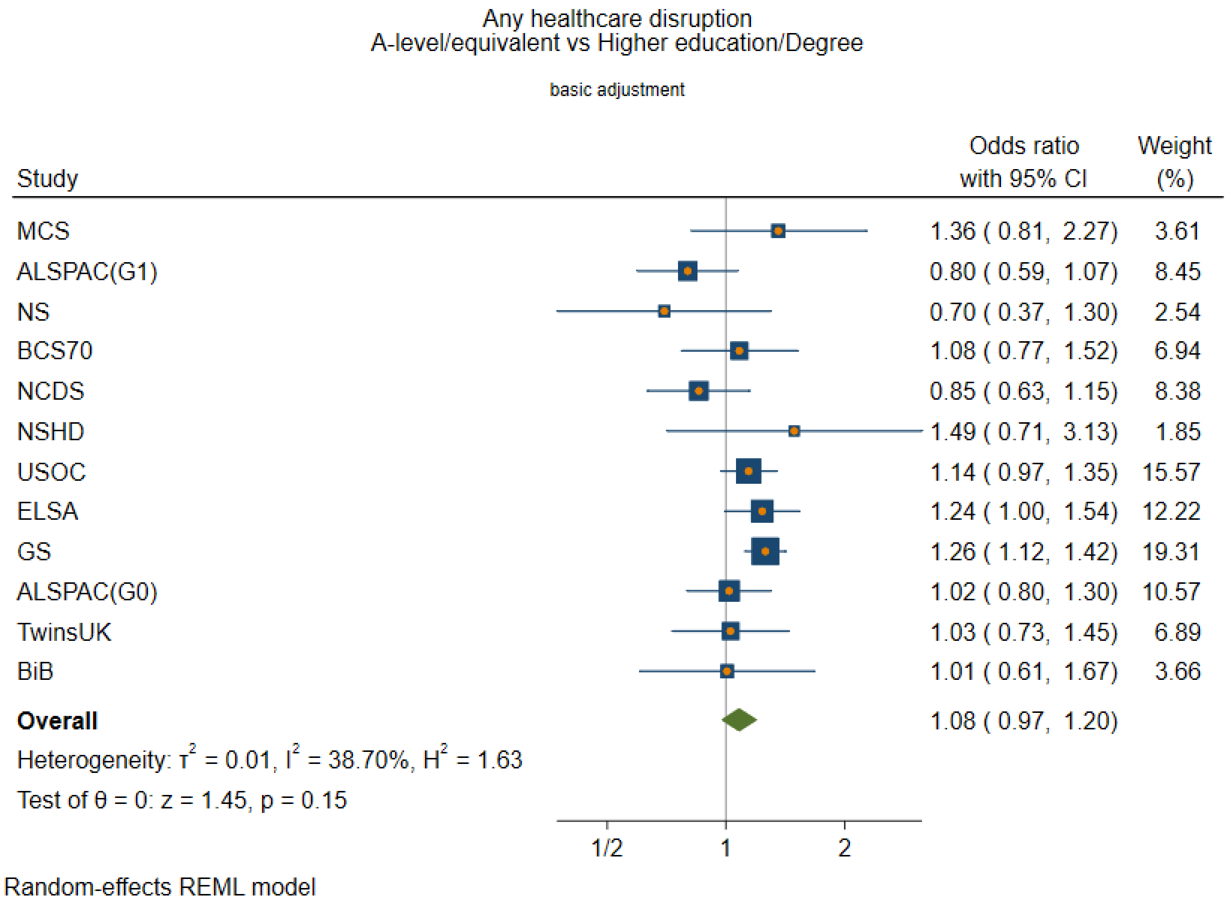

For peer review only - http://bmjopen.bmj.com/site/about/guidelines.xhtml

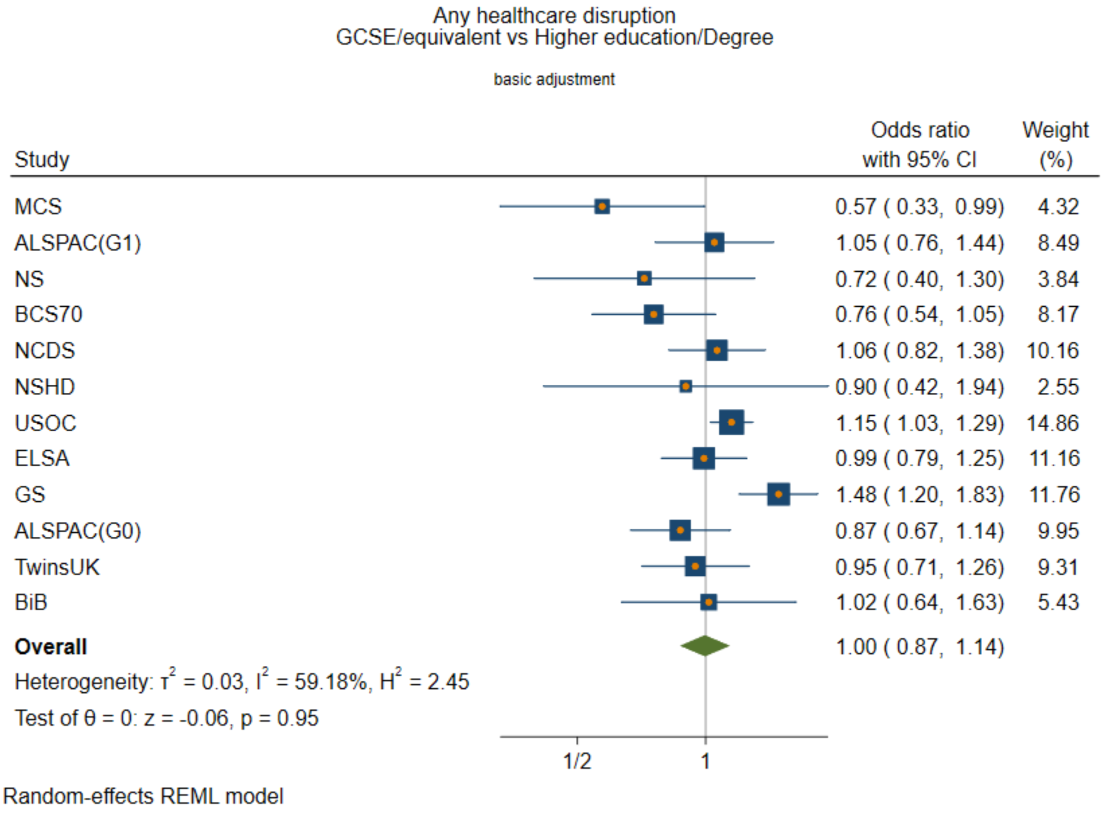
Any healthcare disruption
GCSE/equivalent vs Higher education/Degree
basic adjustment
Study
Odds ratio with 95% CI
Weight (%)
MCS 0.57 (0.33, 0.99) 4.32
ALSPAC(G1) 1.05 (0.76, 1.44) 8.49
NS 0.72 (0.40, 1.30) 3.84
BCS70 0.76 (0.54, 1.05) 8.17
NCDS 1.06 (0.82, 1.38) 10.16
NSHD 0.90 (0.42, 1.94) 2.55
USOC 1.15 (1.03, 1.29) 14.86
ELSA 0.99 (0.79, 1.25) 11.16
GS 1.48 (1.20, 1.83) 11.76
ALSPAC(G0) 0.87 (0.67, 1.14) 9.95
TwinsUK 0.95 (0.71, 1.26) 9.31
BiB 1.02 (0.64, 1.63) 5.43
Overall 1.00 (0.87, 1.14)
Heterogeneity: τ^2 = 0.03, I^2 = 59.18%, H^2 = 2.45
Test of θ = 0: z = -0.06, p = 0.95
1/2
1
Random-effects REML model

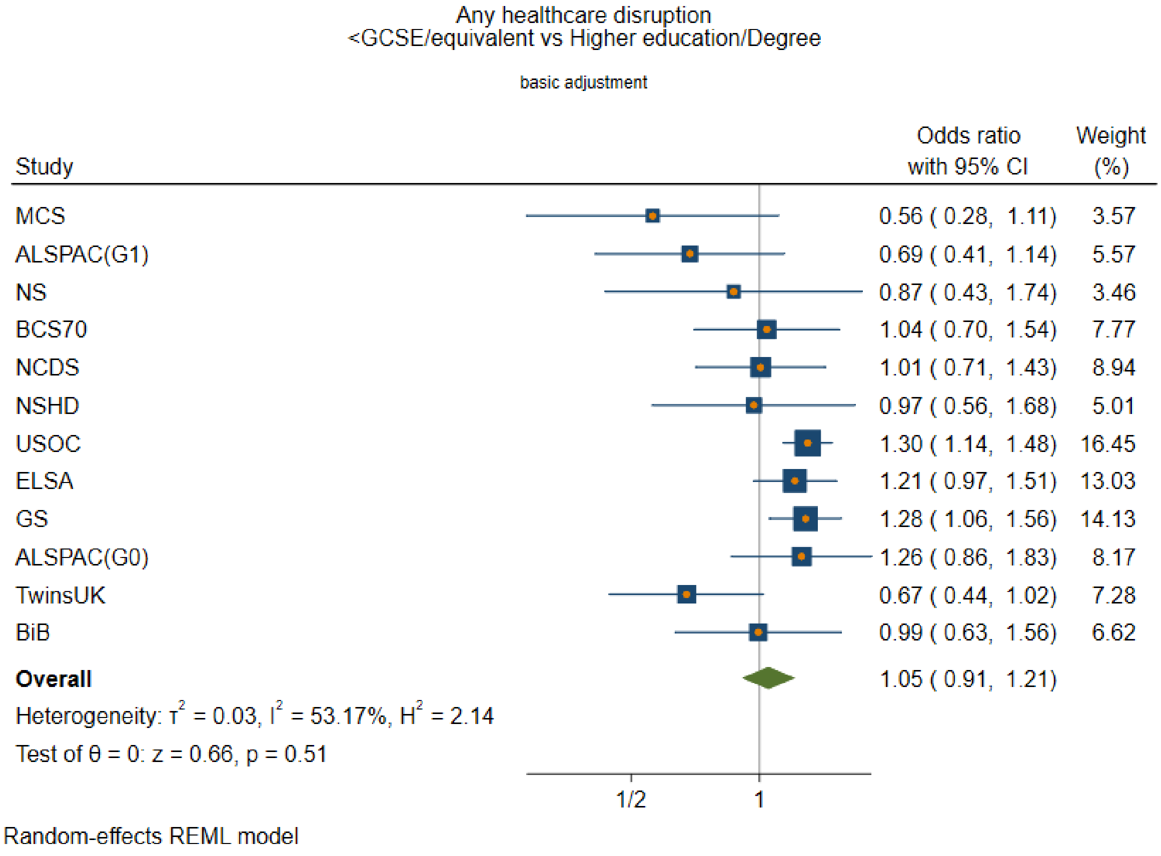
Any healthcare disruption
<GCSE/equivalent vs Higher education/Degree
basic adjustment
Study
Odds ratio with 95% CI
Weight (%)
MCS 0.56 (0.28, 1.11) 3.57
ALSPAC(G1) 0.69 (0.41, 1.14) 5.57
NS 0.87 (0.43, 1.74) 3.46
BCS70 1.04 (0.70, 1.54) 7.77
NCDS 1.01 (0.71, 1.43) 8.94
NSHD 0.97 (0.56, 1.68) 5.01
USOC 1.30 (1.14, 1.48) 16.45
ELSA 1.21 (0.97, 1.51) 13.03
GS 1.28 (1.06, 1.56) 14.13
ALSPAC(G0) 1.26 (0.86, 1.83) 8.17
TwinsUK 0.67 (0.44, 1.02) 7.28
BiB 0.99 (0.63, 1.56) 6.62
Overall 1.05 (0.91, 1.21)
Heterogeneity: τ^2 = 0.03, I^2 = 53.17%, H^2 = 2.14
Test of θ = 0: z = 0.66, p = 0.51
1/2
1
Random-effects REML model

Full adjustment

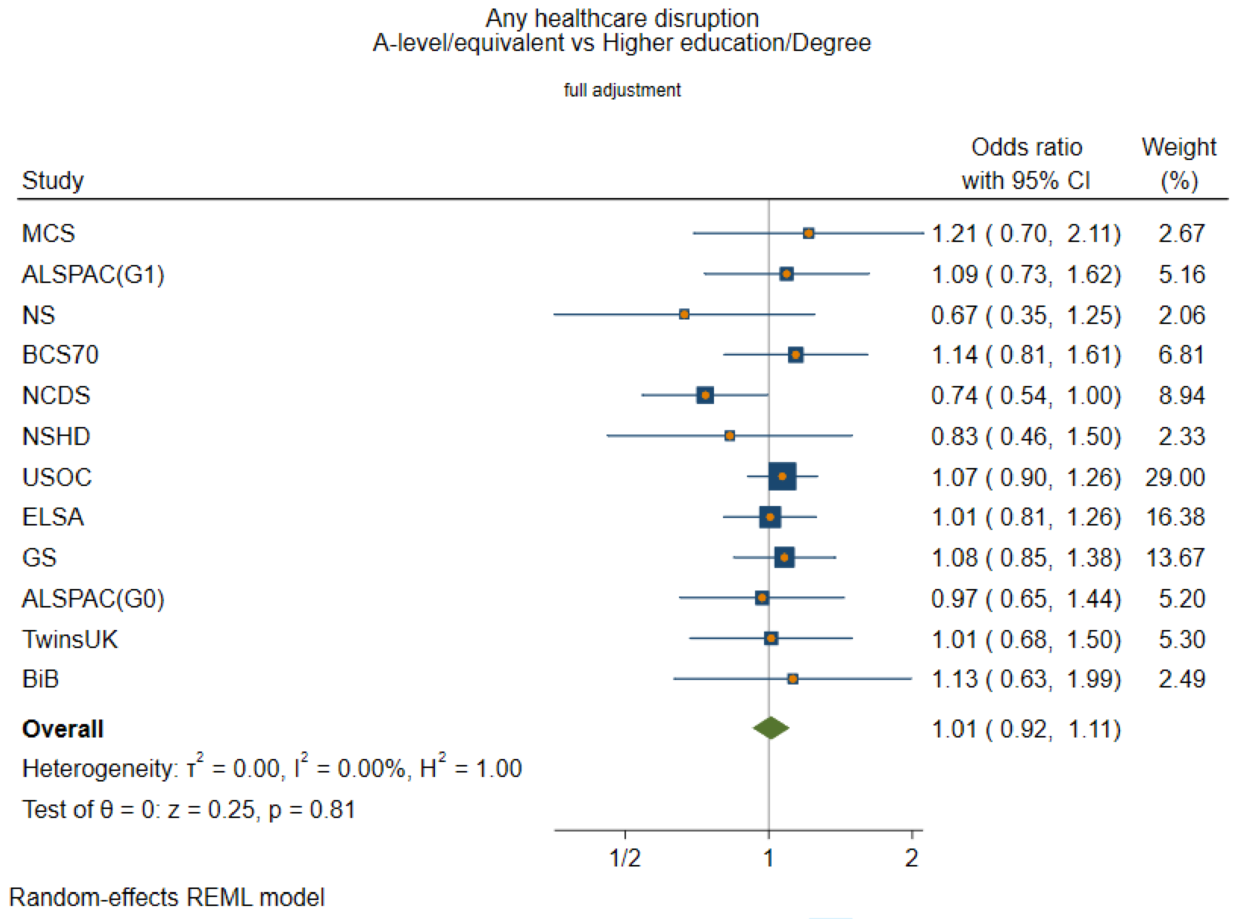

For peer review only - http://bmjopen.bmj.com/site/about/guidelines.xhtml

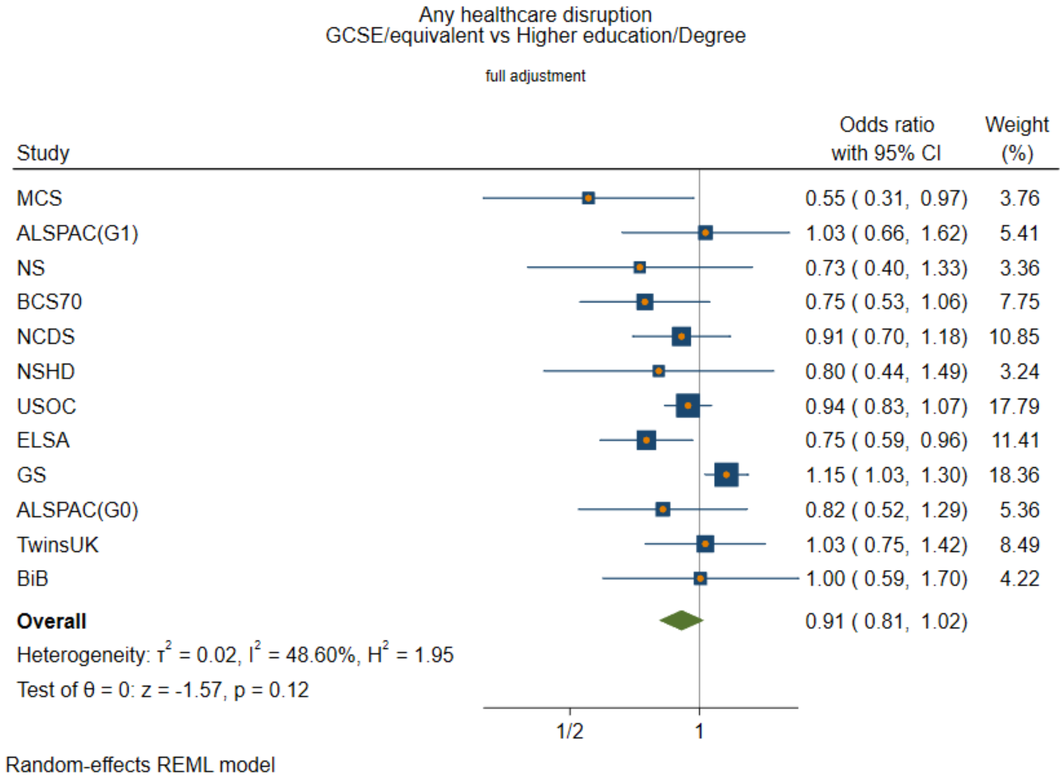

revie

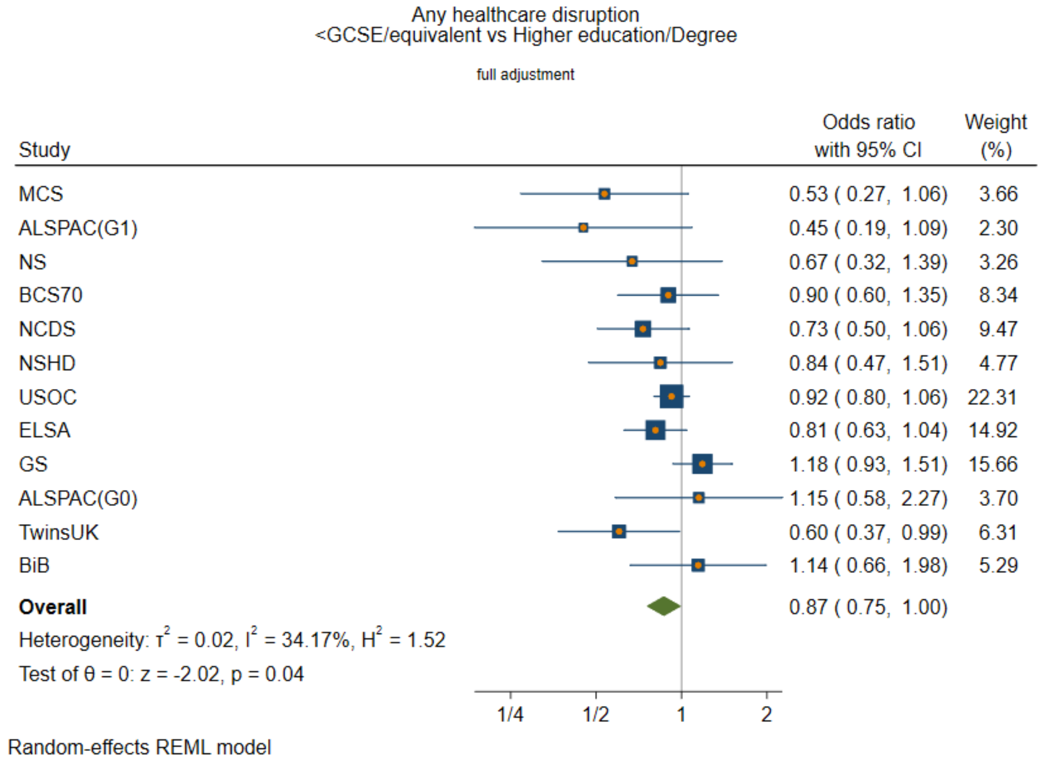

For peer review only - http://bmjopen.bmj.com/site/about/guidelines.xhtml

Age

<u>Unadjusted</u>

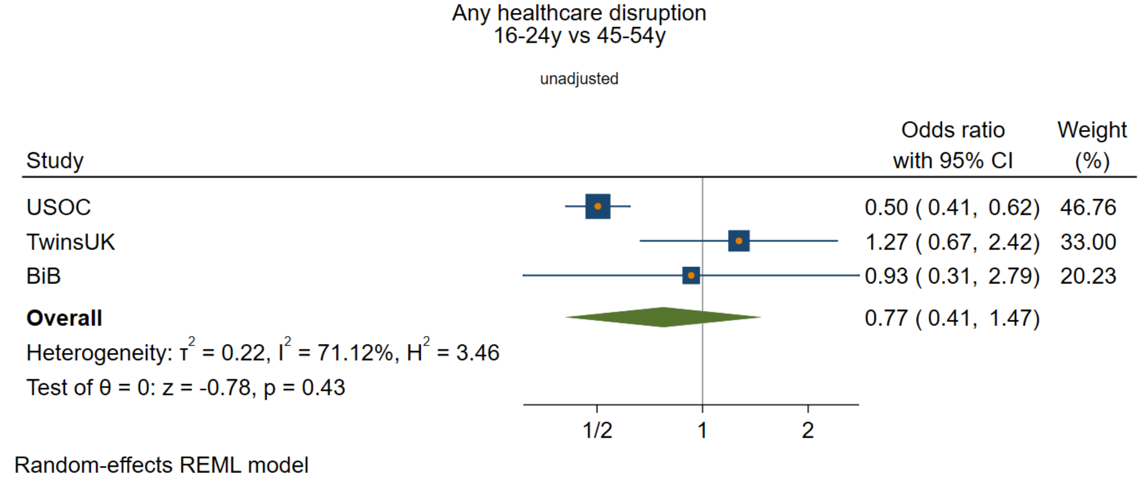

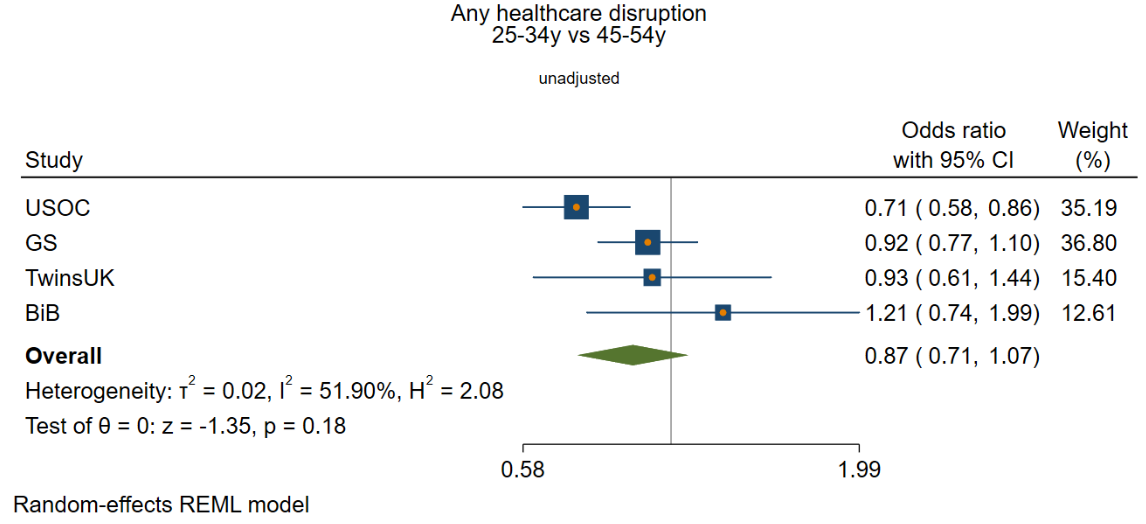

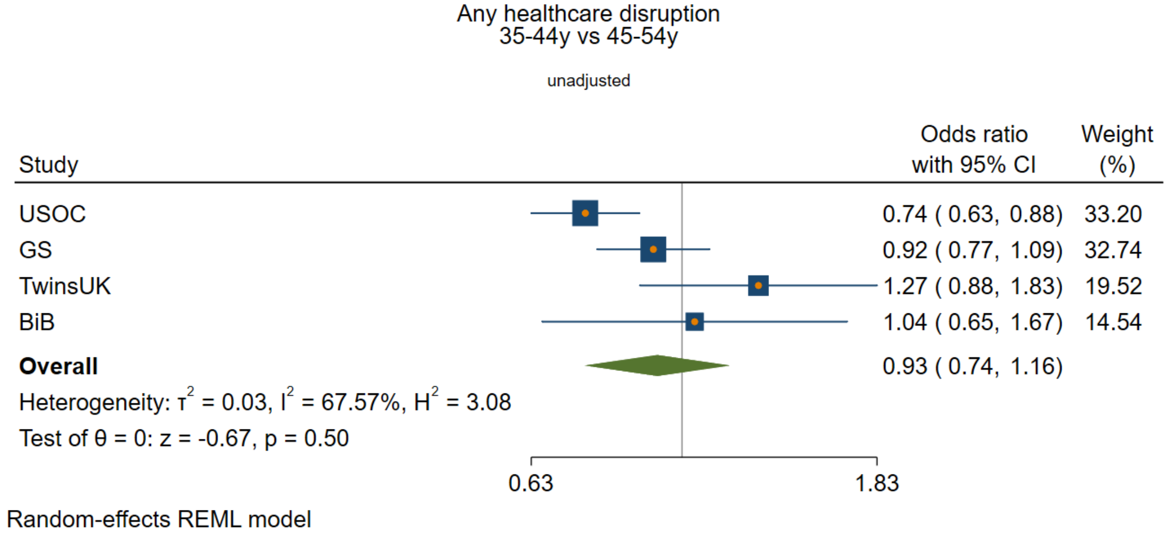

For peer review only - http://bmjopen.bmj.com/site/about/guidelines.xhtml

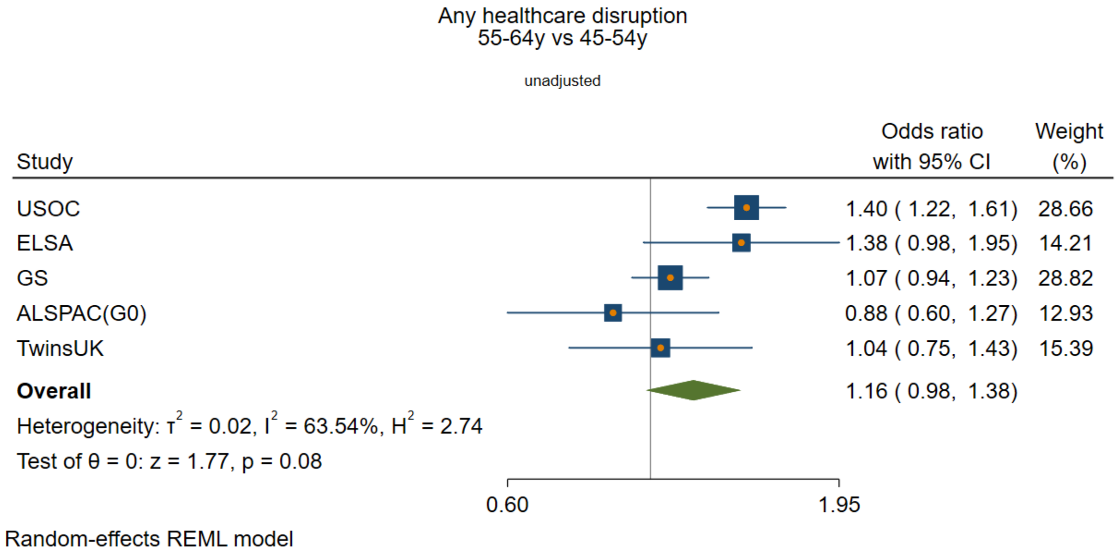

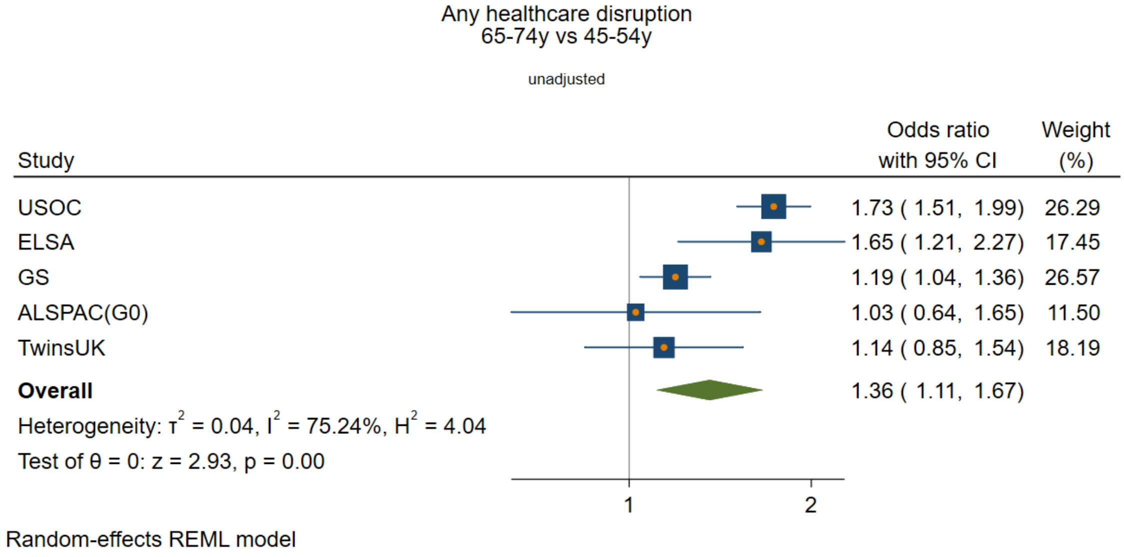

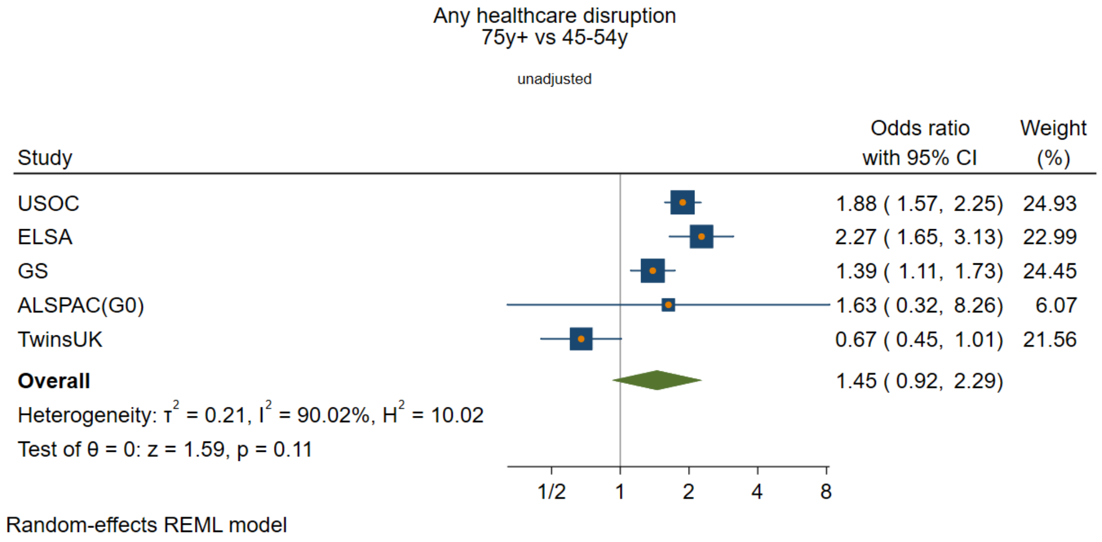

For peer review only - http://bmjopen.bmj.com/site/about/guidelines.xhtml

Basic adjustment

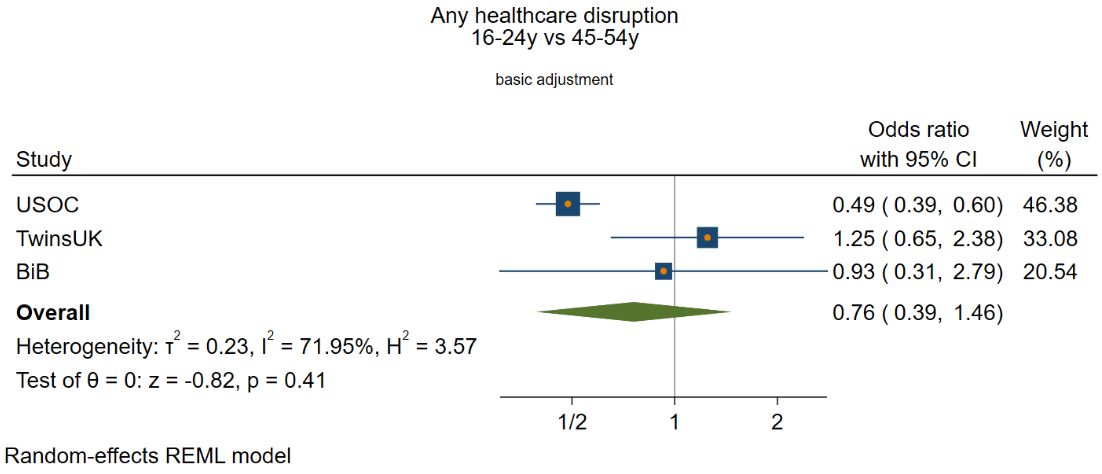

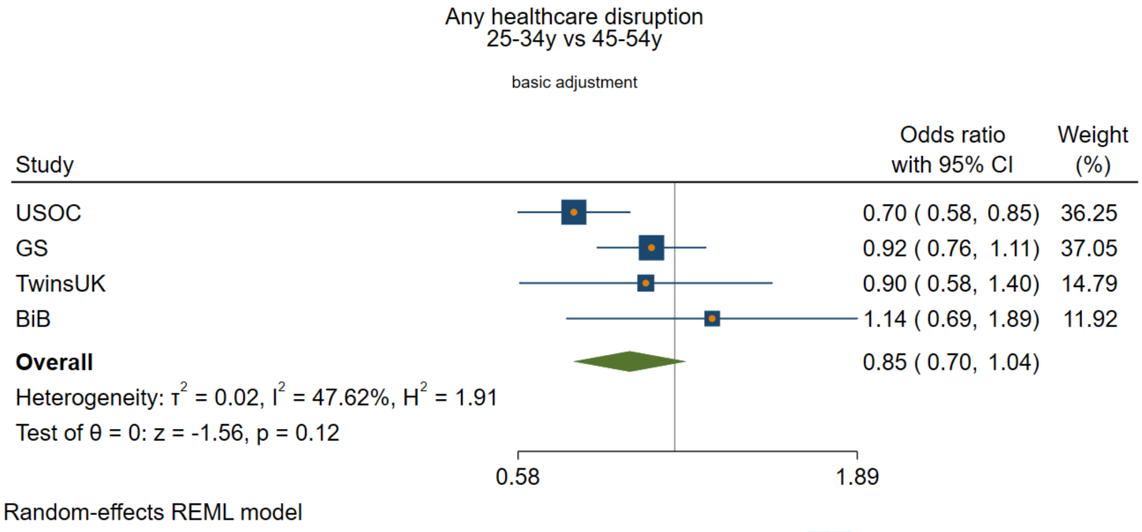

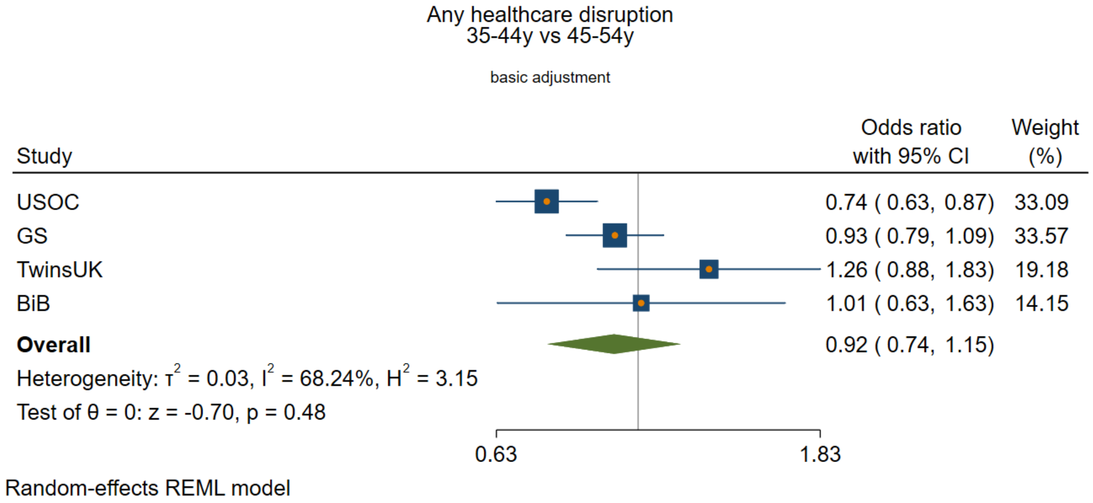

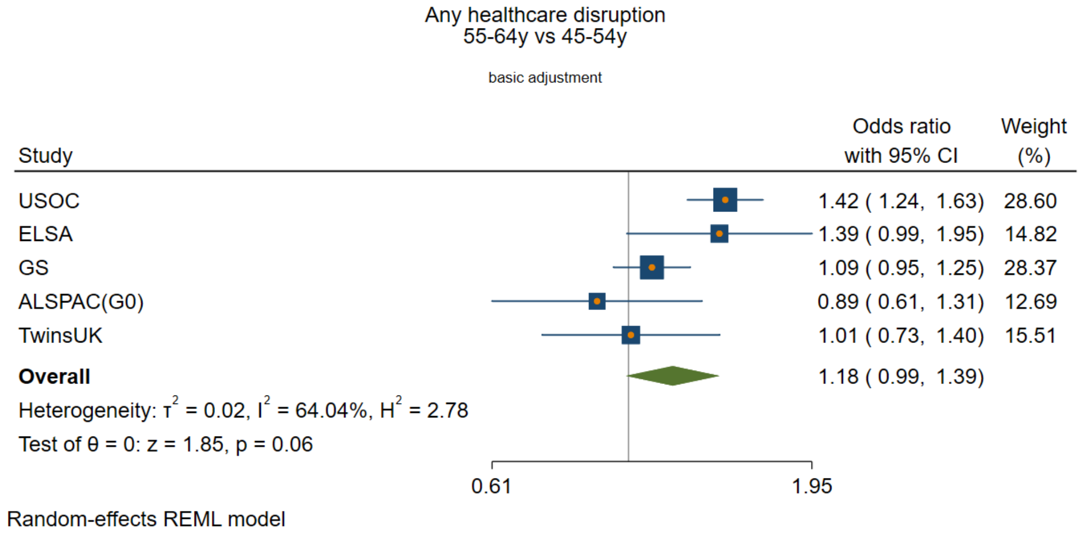
Any healthcare disruption
55-64y vs 45-54y
basic adjustment
Study
Odds ratio
with 95% CI
Weight
(%)
USOC 1.42 (1.24, 1.63) 28.60
ELSA 1.39 (0.99, 1.95) 14.82
GS 1.09 (0.95, 1.25) 28.37
ALSPAC(G0) 0.89 (0.61, 1.31) 12.69
TwinsUK 1.01 (0.73, 1.40) 15.51
Overall 1.18 (0.99, 1.39)
Heterogeneity: τ² = 0.02, I² = 64.04%, H² = 2.78
Test of θ = 0: z = 1.85, p = 0.06
0.61
1.95
Random-effects REML model

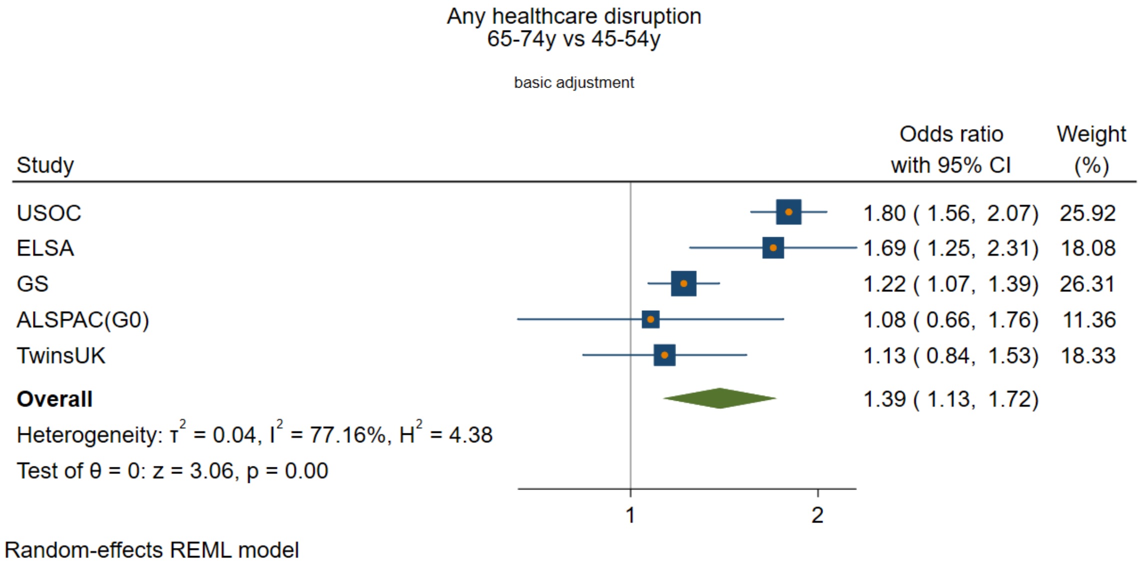
Any healthcare disruption
65-74y vs 45-54y
basic adjustment
Study
Odds ratio
with 95% CI
Weight
(%)
USOC 1.80 (1.56, 2.07) 25.92
ELSA 1.69 (1.25, 2.31) 18.08
GS 1.22 (1.07, 1.39) 26.31
ALSPAC(G0) 1.08 (0.66, 1.76) 11.36
TwinsUK 1.13 (0.84, 1.53) 18.33
Overall 1.39 (1.13, 1.72)
Heterogeneity: τ² = 0.04, I² = 77.16%, H² = 4.38
Test of θ = 0: z = 3.06, p = 0.00
1
2
Random-effects REML model

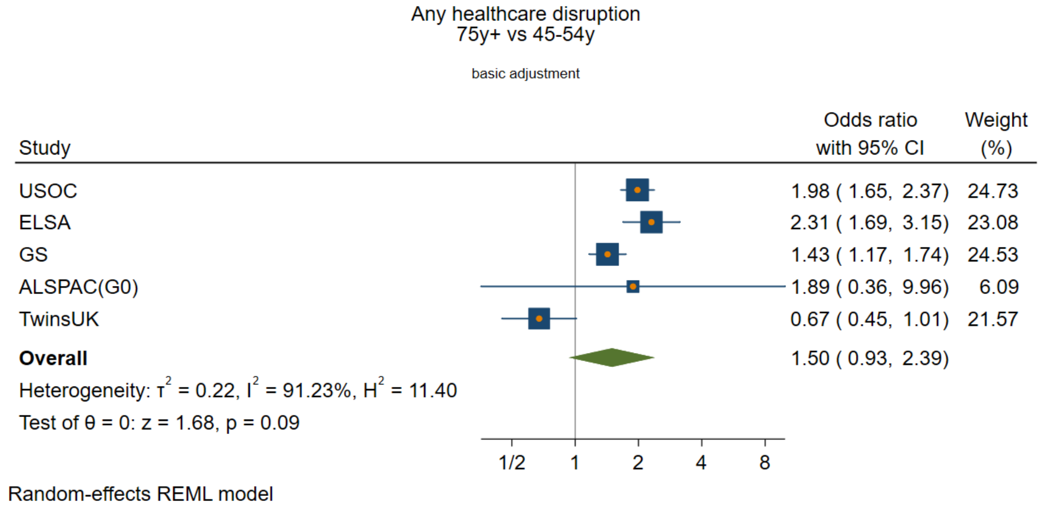
Any healthcare disruption
75y+ vs 45-54y
basic adjustment
Study
Odds ratio
with 95% CI
Weight
(%)
USOC 1.98 (1.65, 2.37) 24.73
ELSA 2.31 (1.69, 3.15) 23.08
GS 1.43 (1.17, 1.74) 24.53
ALSPAC(G0) 1.89 (0.36, 9.96) 6.09
TwinsUK 0.67 (0.45, 1.01) 21.57
Overall 1.50 (0.93, 2.39)
Heterogeneity: τ² = 0.22, I² = 91.23%, H² = 11.40
Test of θ = 0: z = 1.68, p = 0.09
1/2
1
2
4
8
Random-effects REML model

Full adjustment

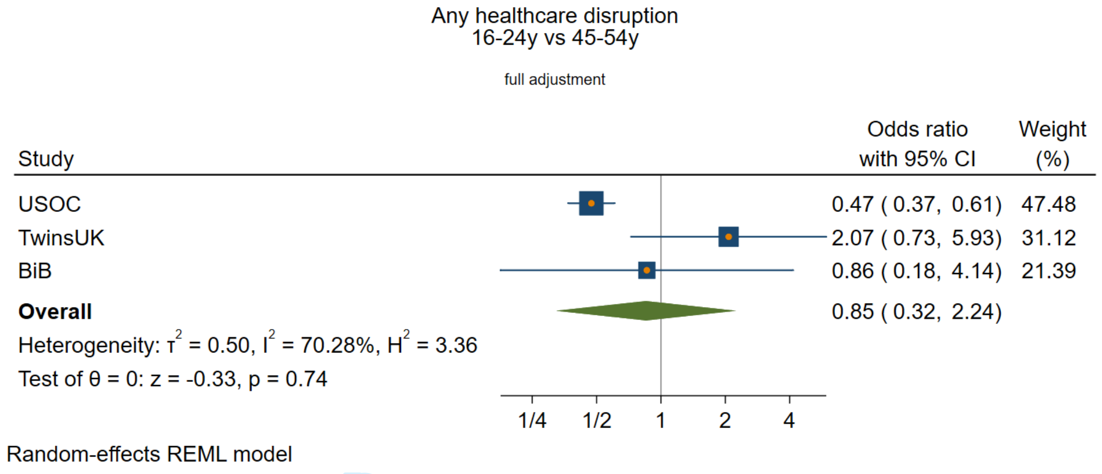

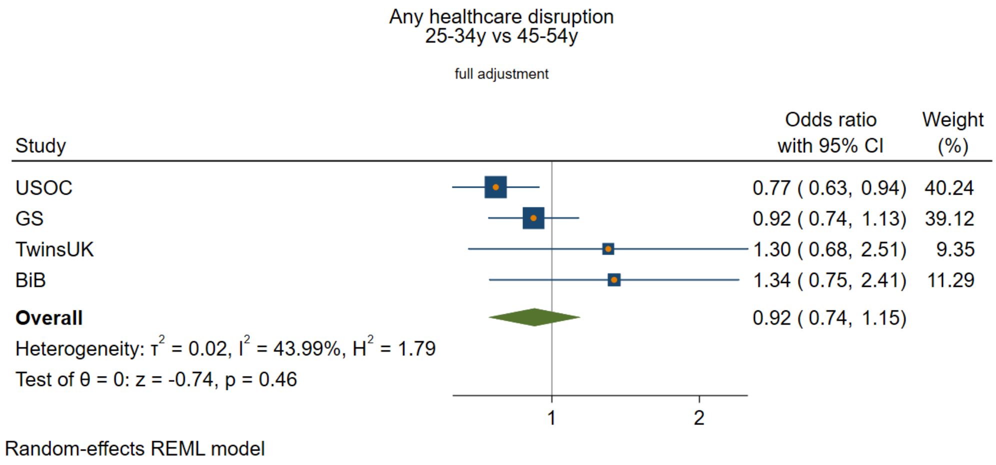

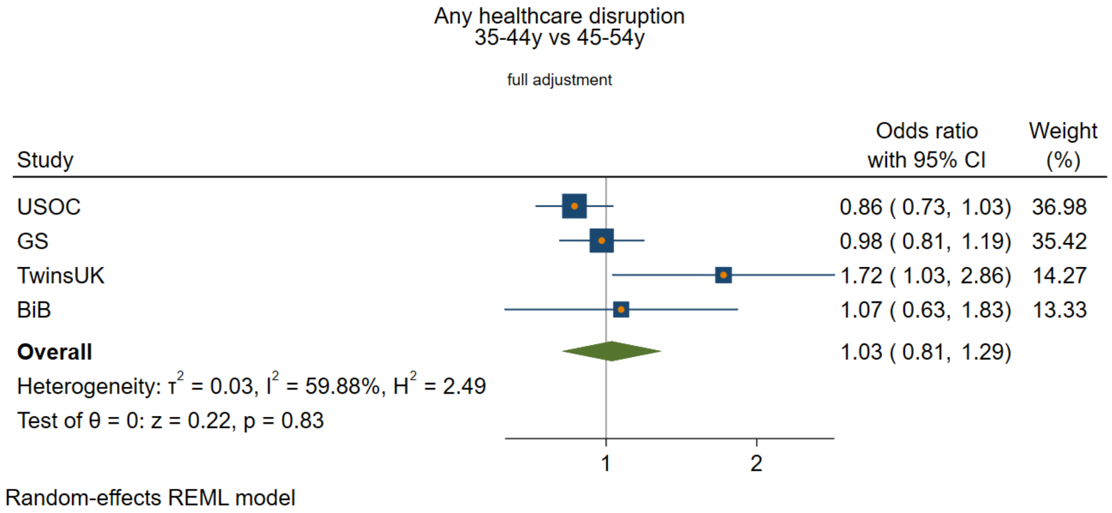

For peer review only - http://bmjopen.bmj.com/site/about/guidelines.xhtml

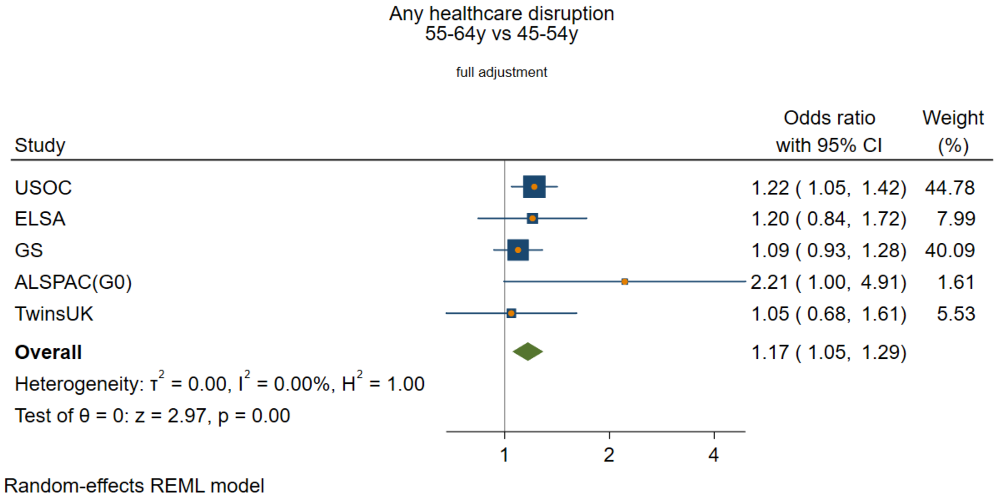

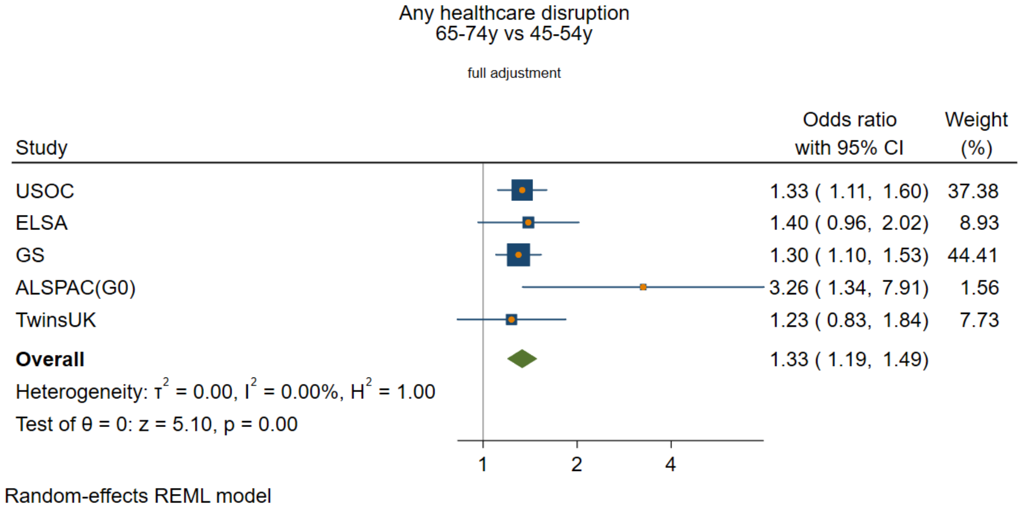

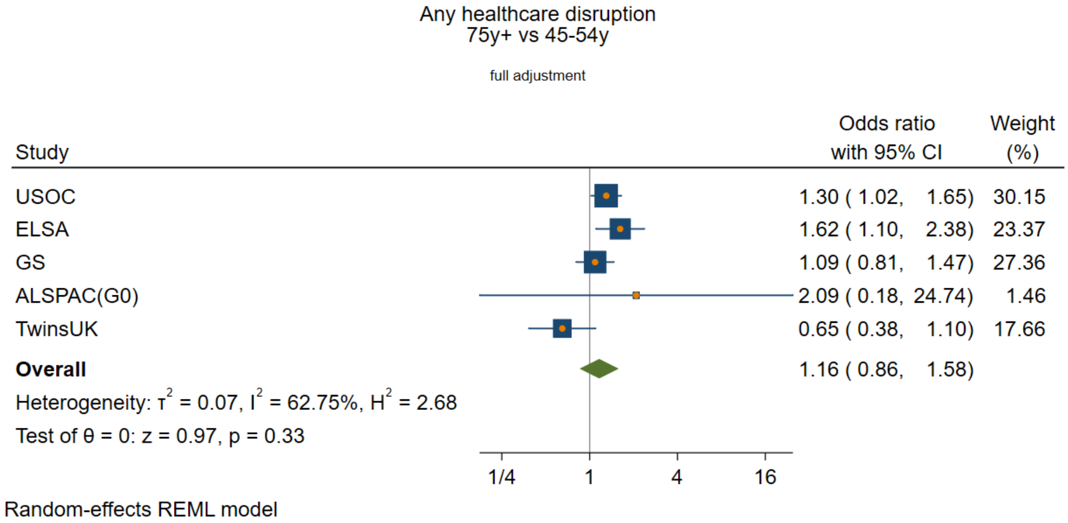

For peer review only - http://bmjopen.bmj.com/site/about/guidelines.xhtml

Ethnicity
Unadjusted

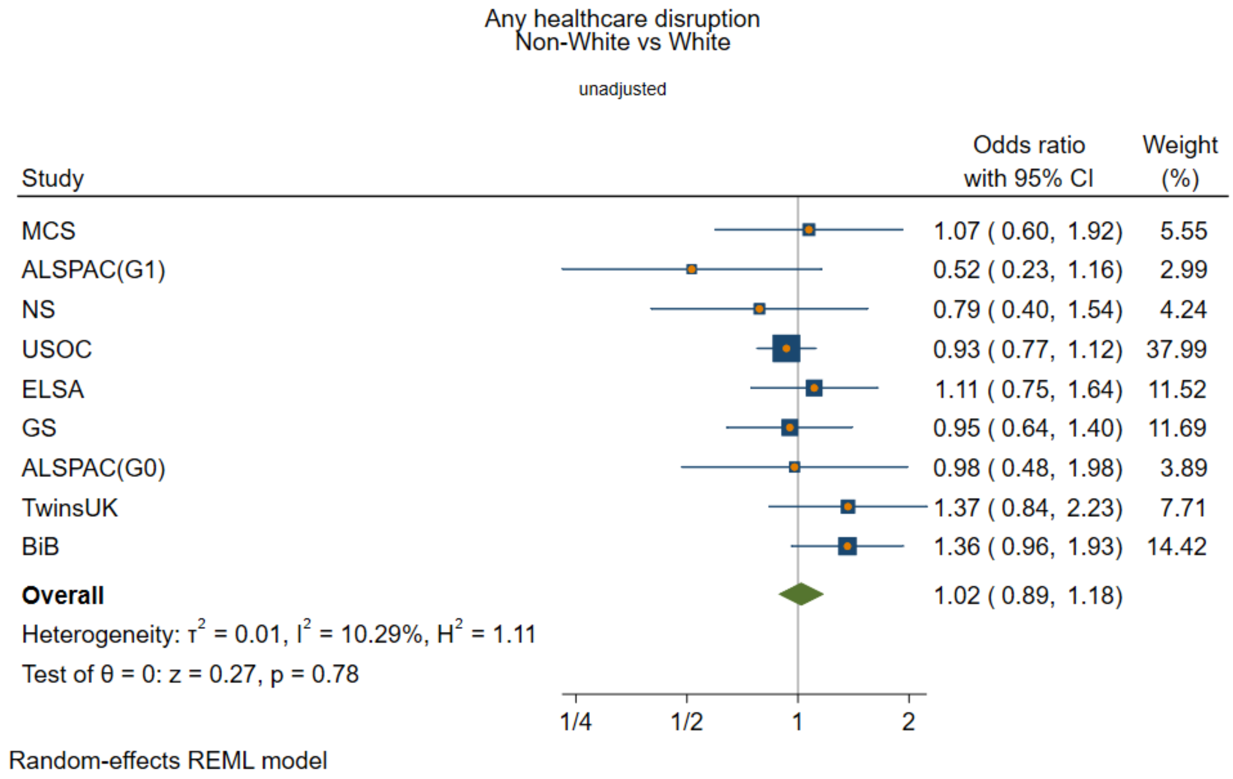

iew only

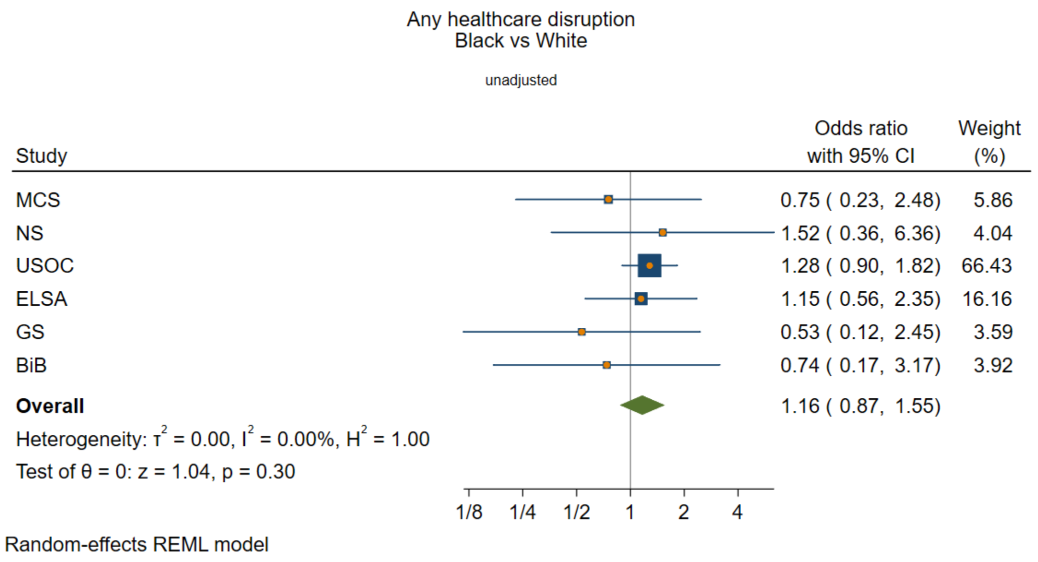

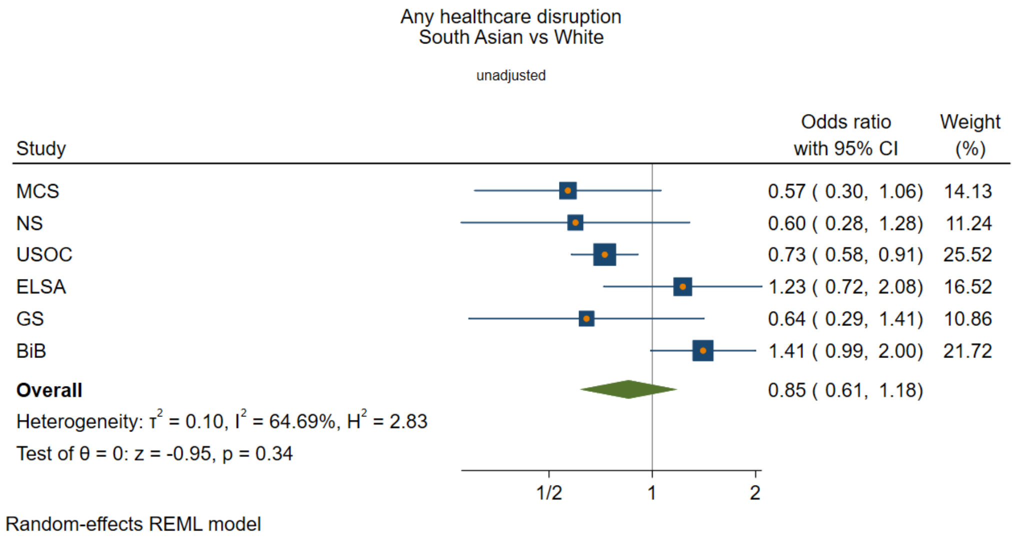

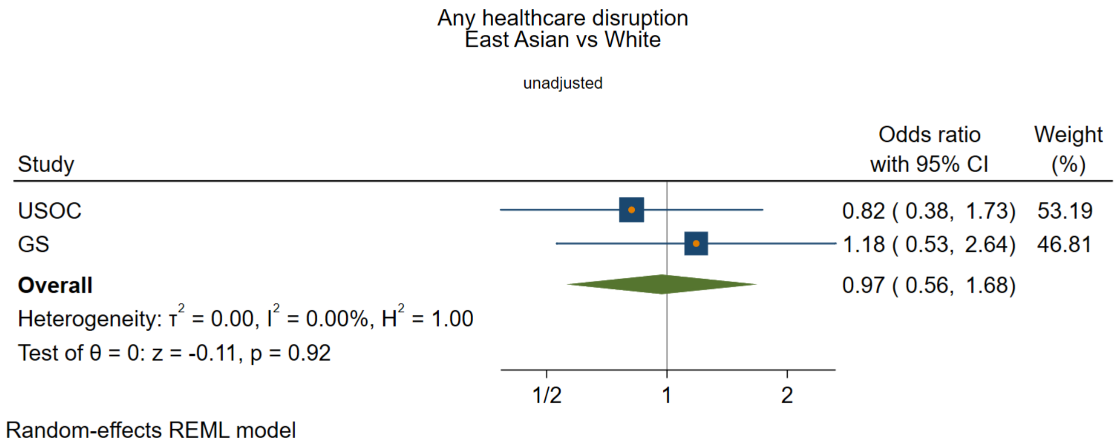

For peer review only - http://bmjopen.bmj.com/site/about/guidelines.xhtml

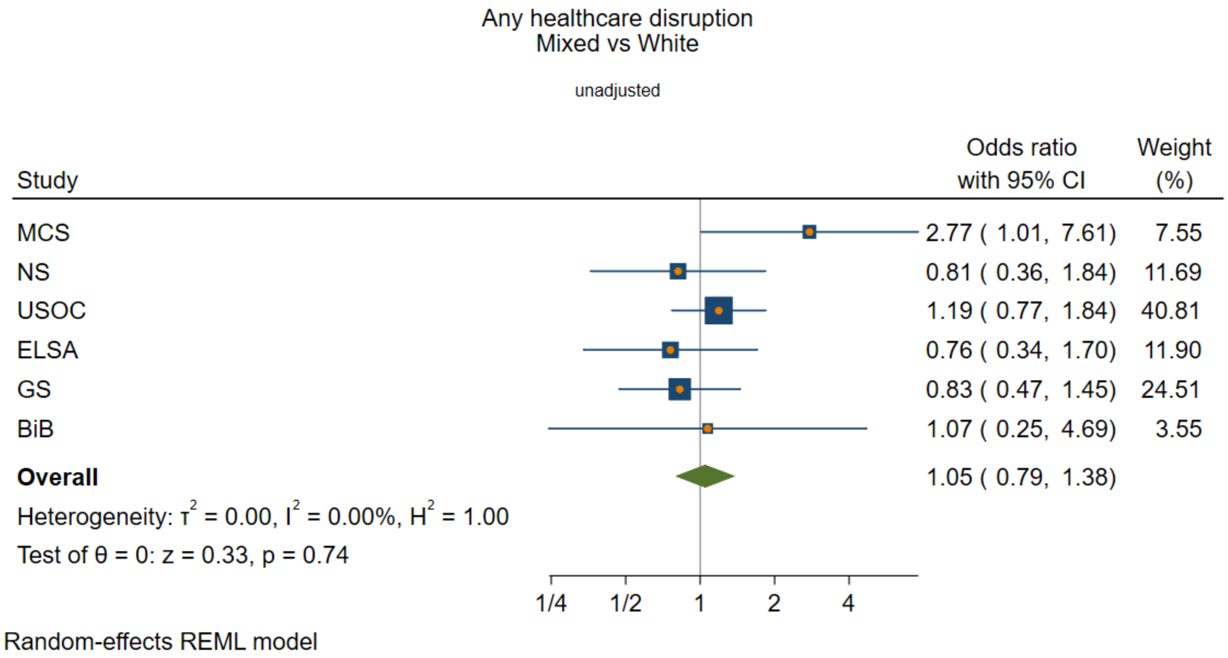

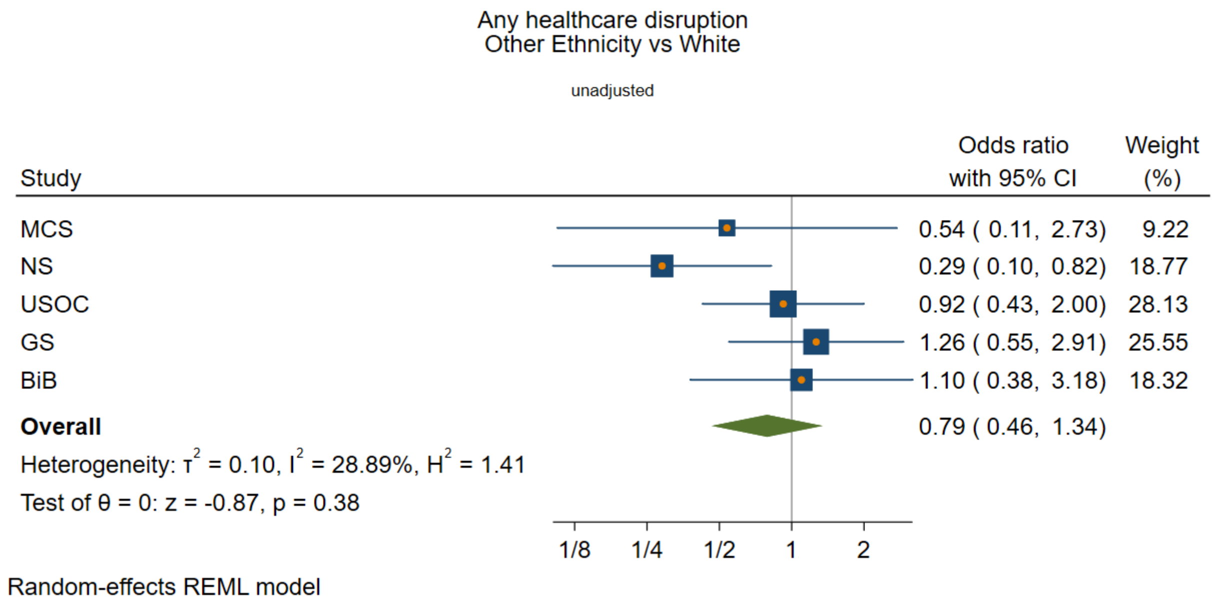

For peer review only - http://bmjopen.bmj.com/site/about/guidelines.xhtml

Basic adjustment

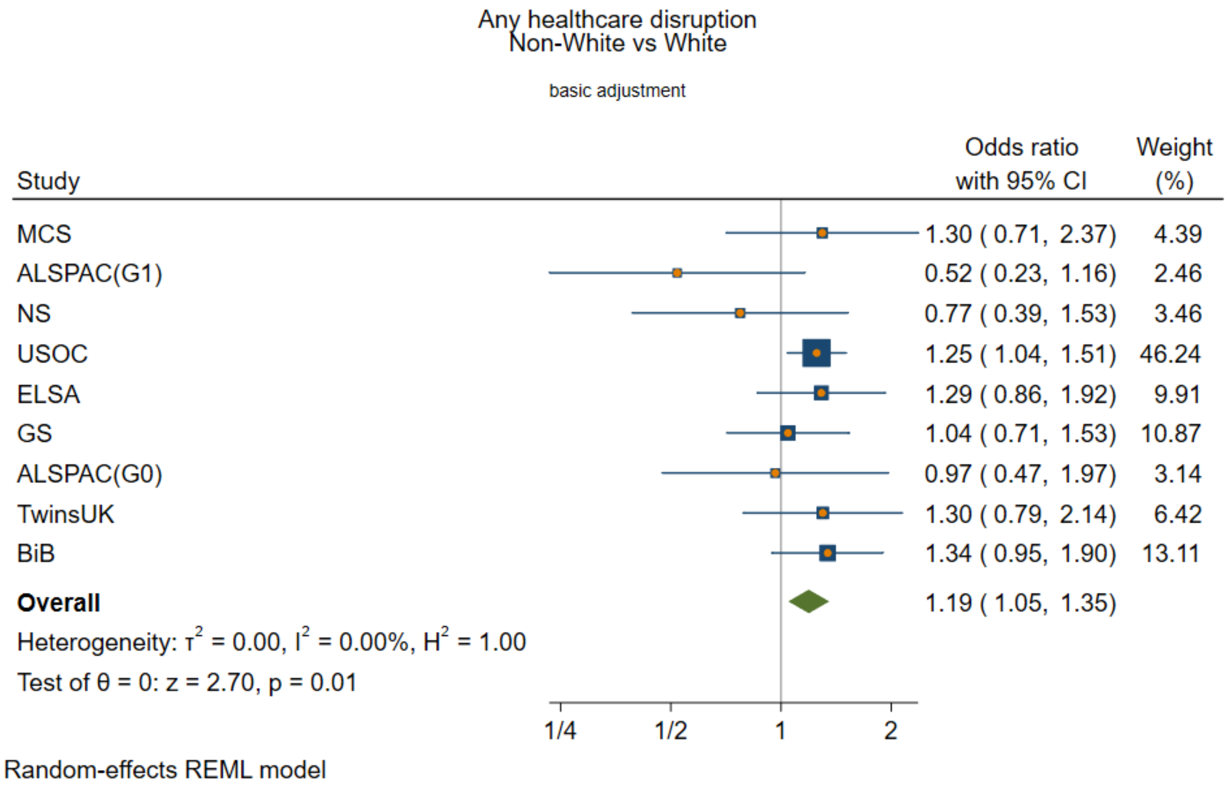

For peer review only - http://bmjopen.bmj.com/site/about/guidelines.xhtml

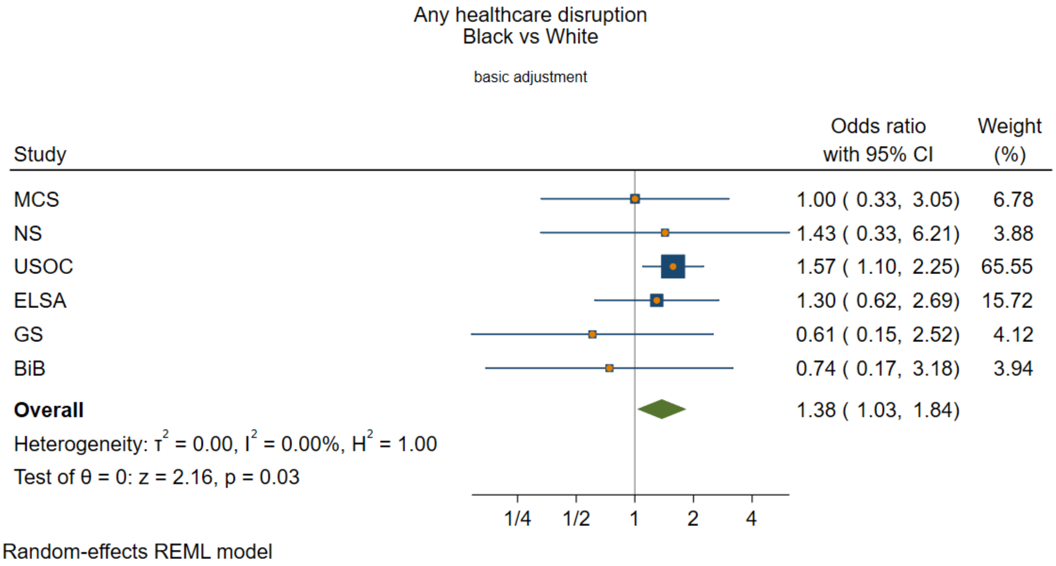

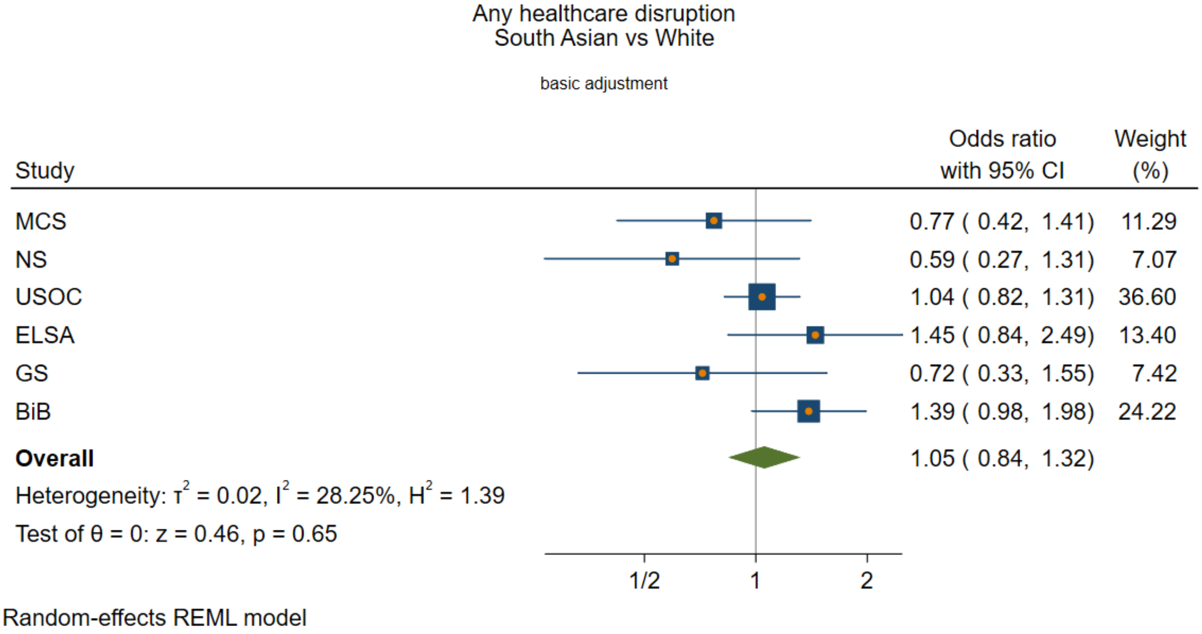

For peer review only - http://bmjopen.bmj.com/site/about/guidelines.xhtml

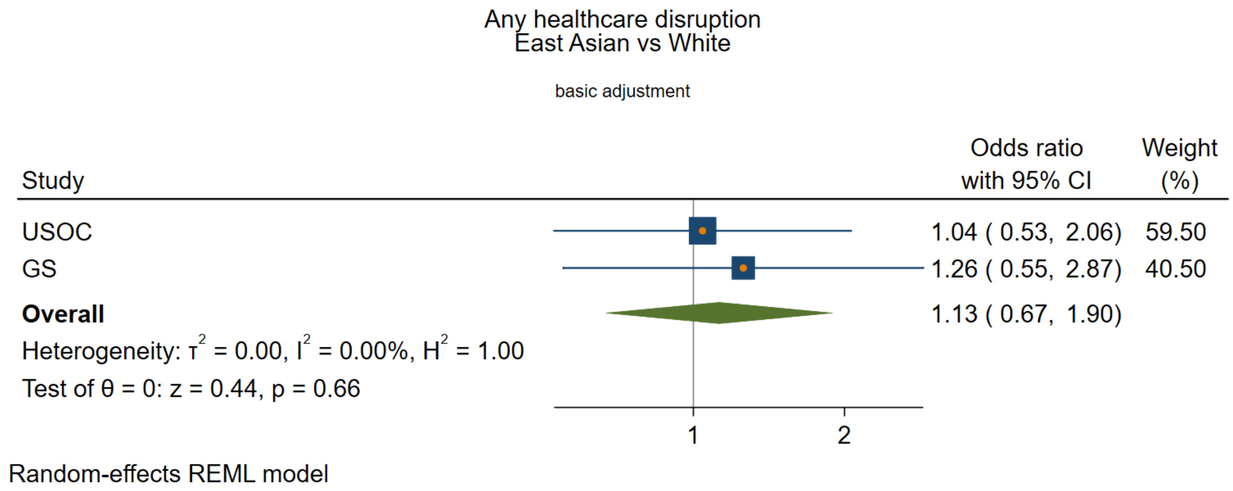

For peer review only - http://bmjopen.bmj.com/site/about/guidelines.xhtml

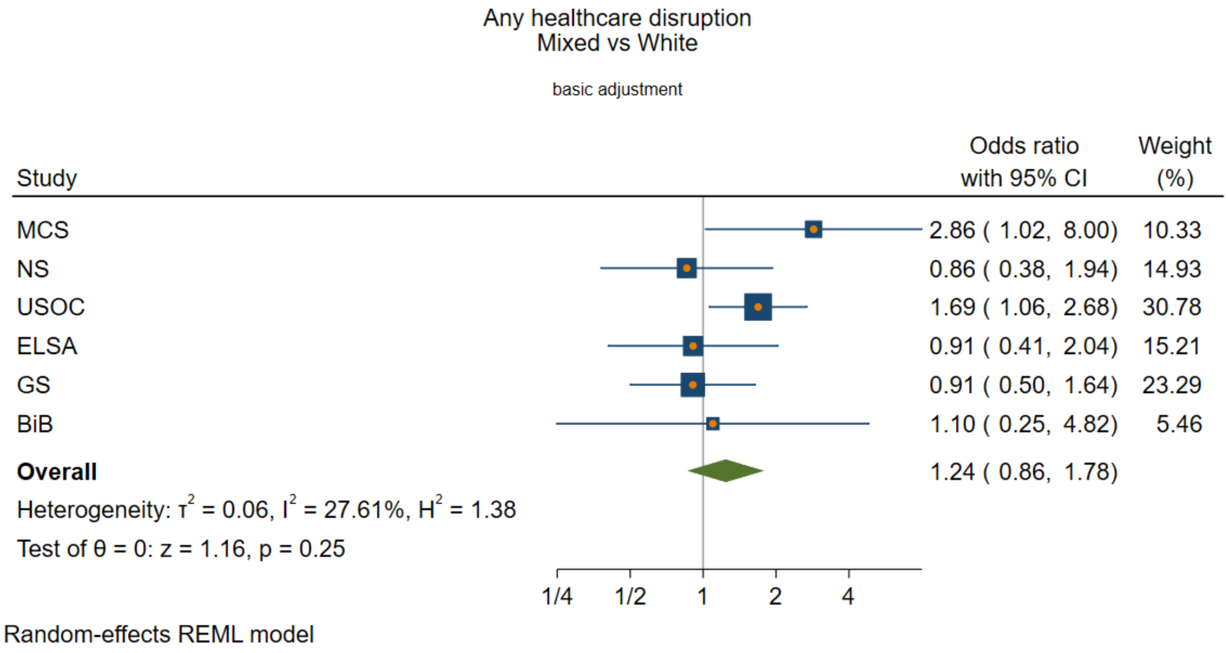

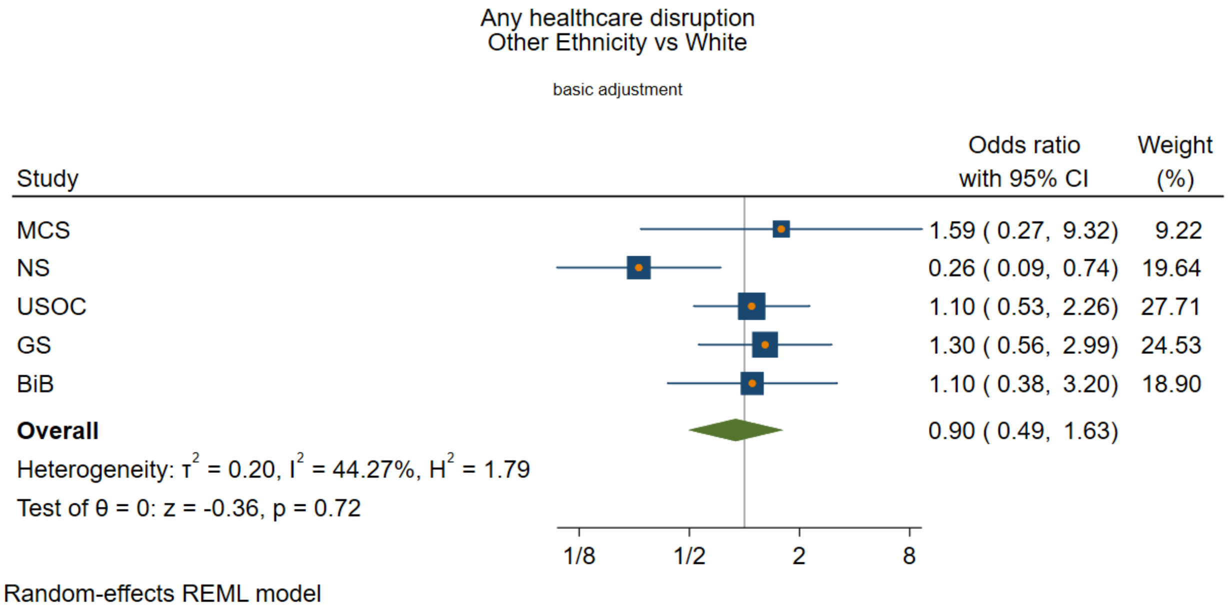

For peer review only - http://bmjopen.bmj.com/site/about/guidelines.xhtml

Full adjustment

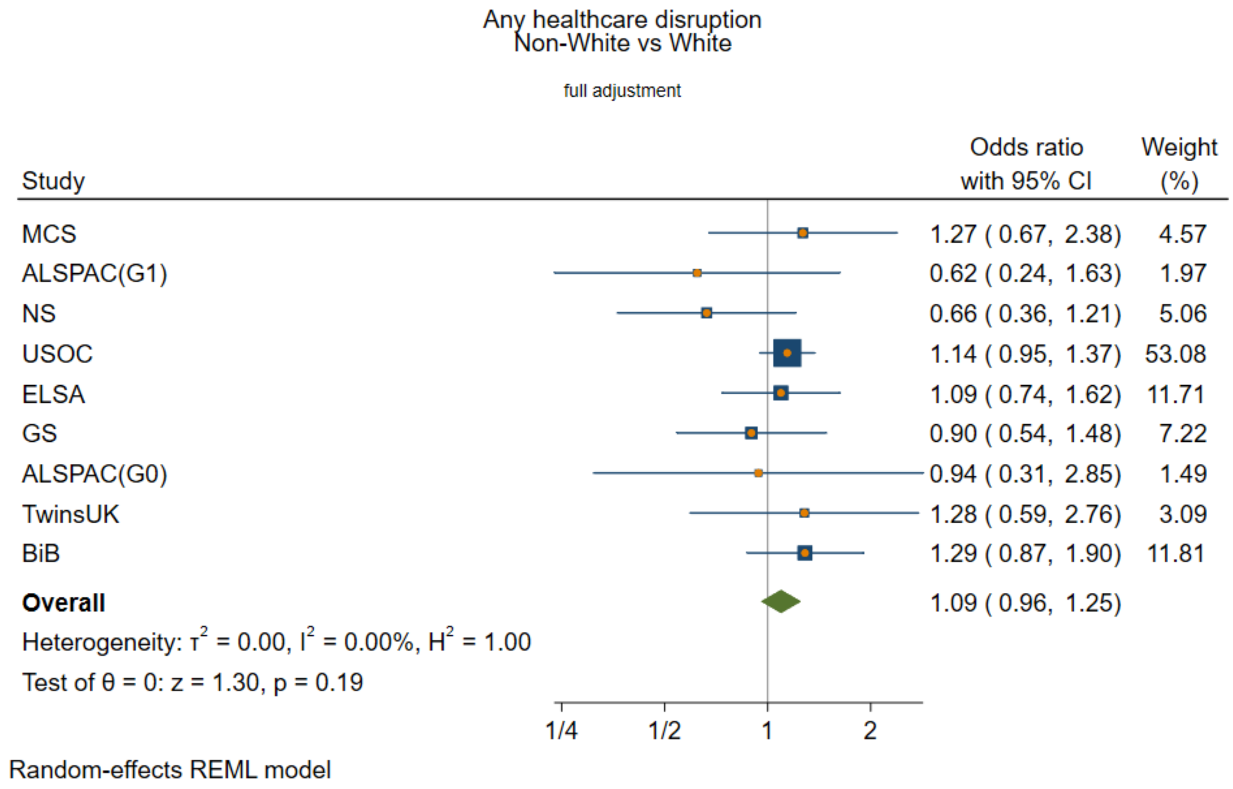

view only

For peer review only - http://bmjopen.bmj.com/site/about/guidelines.xhtml

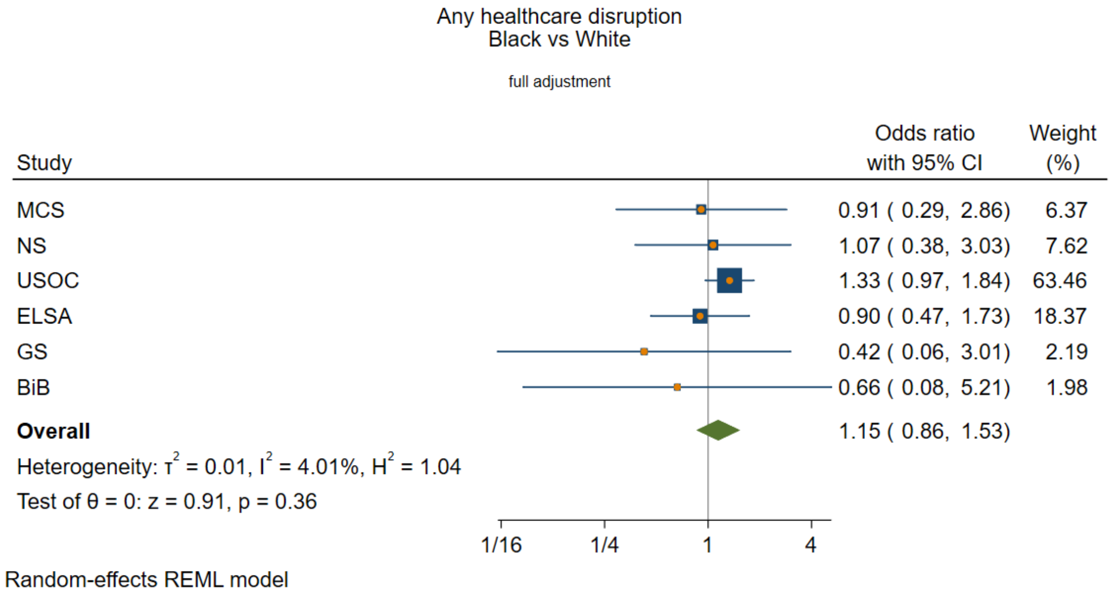

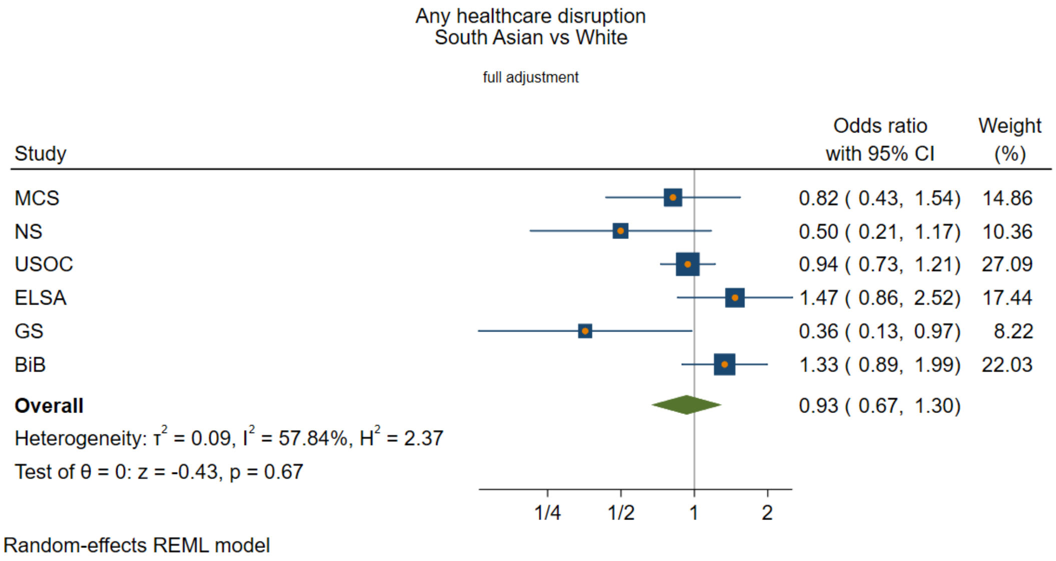

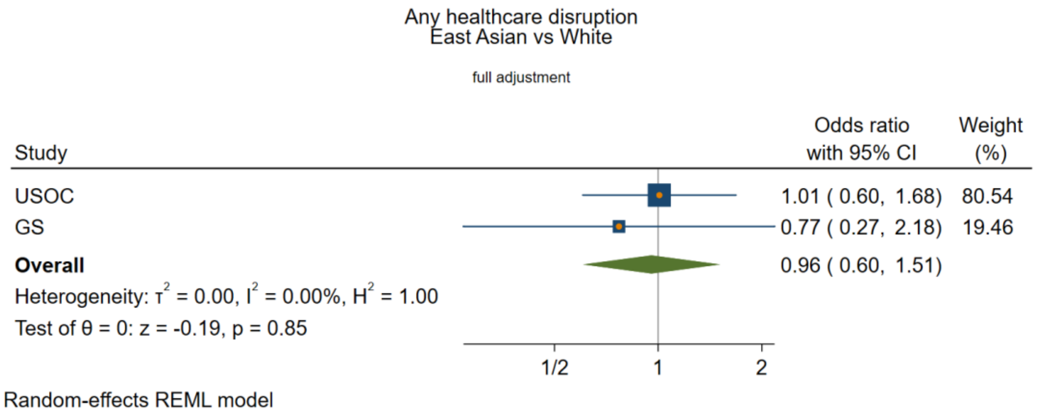

For peer review only - http://bmjopen.bmj.com/site/about/guidelines.xhtml

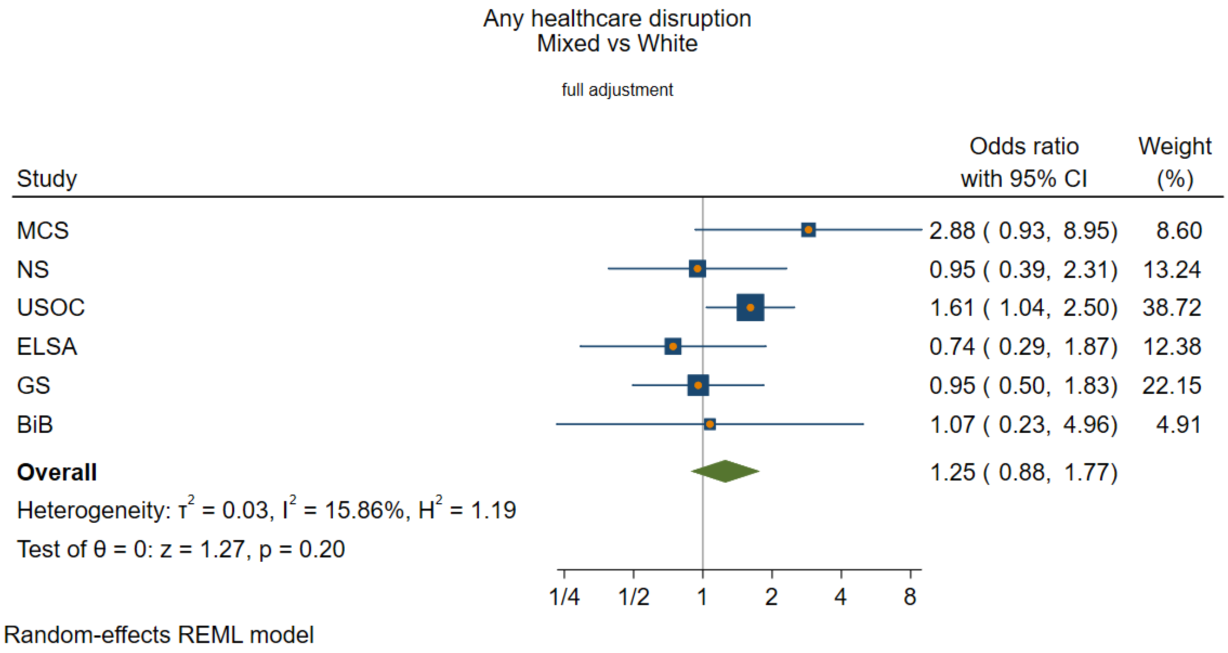

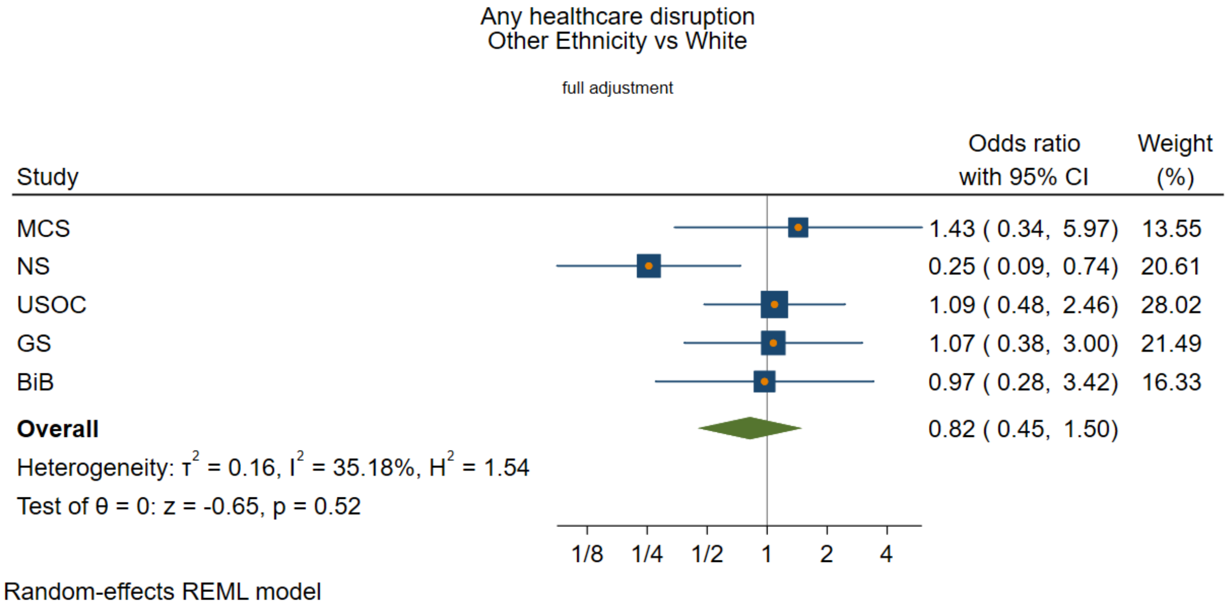

For peer review only - http://bmjopen.bmj.com/site/about/guidelines.xhtml

Appointments

Sex

<u>Unadjusted</u>

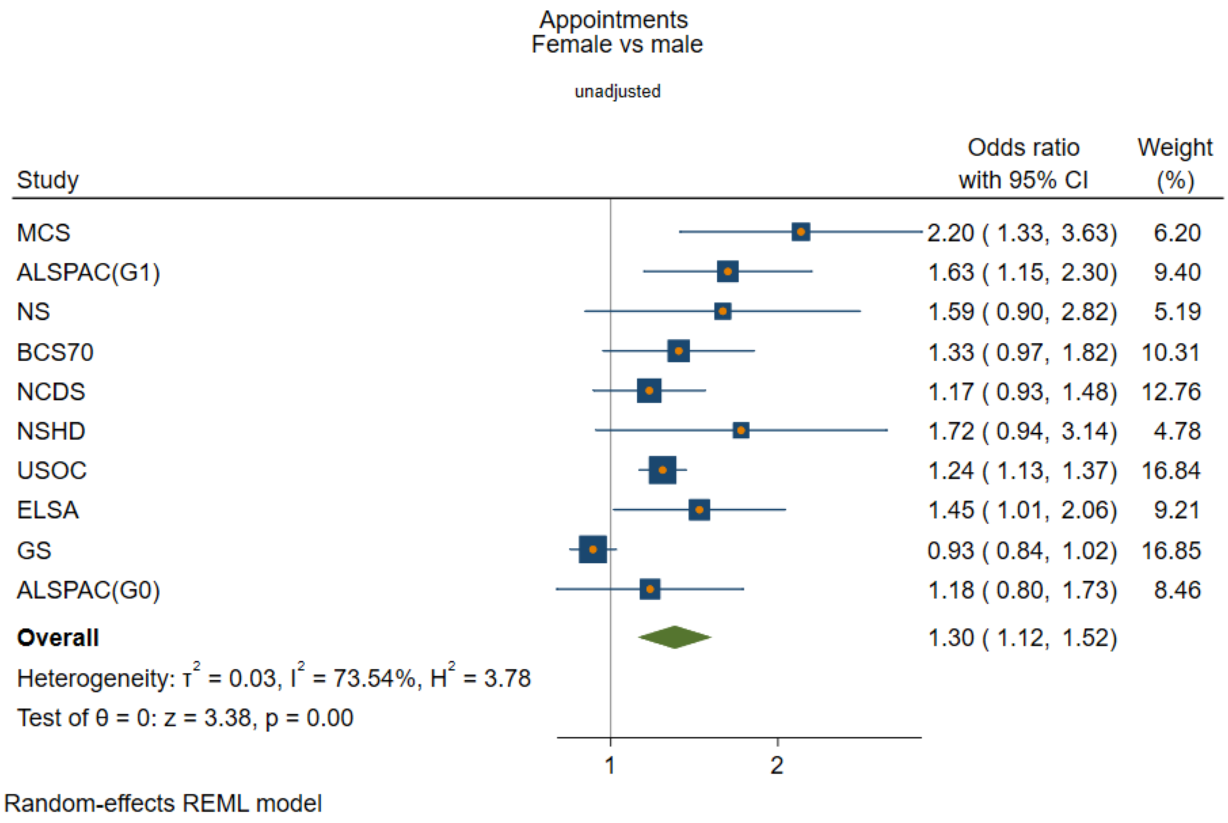

w only

For peer review only - http://bmjopen.bmj.com/site/about/guidelines.xhtml

Basic adjustment

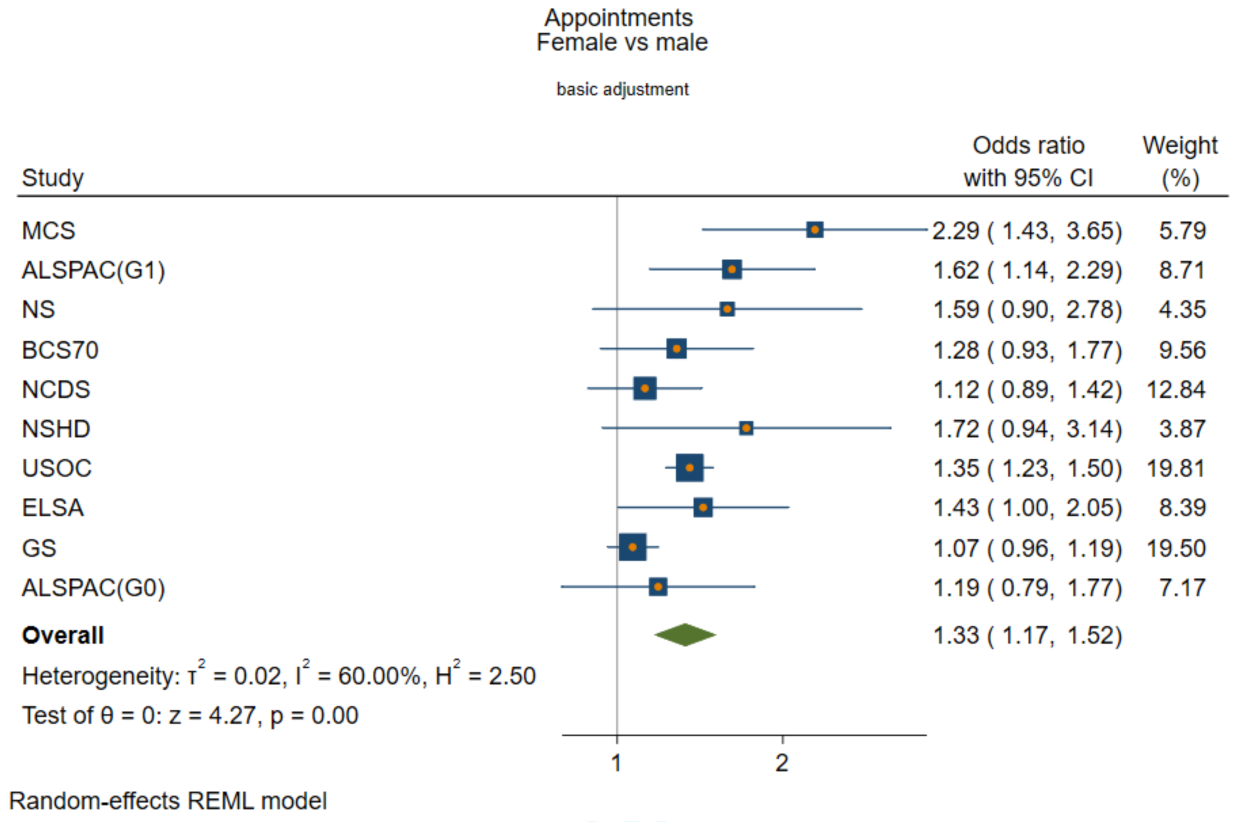

review only

Full adjustment

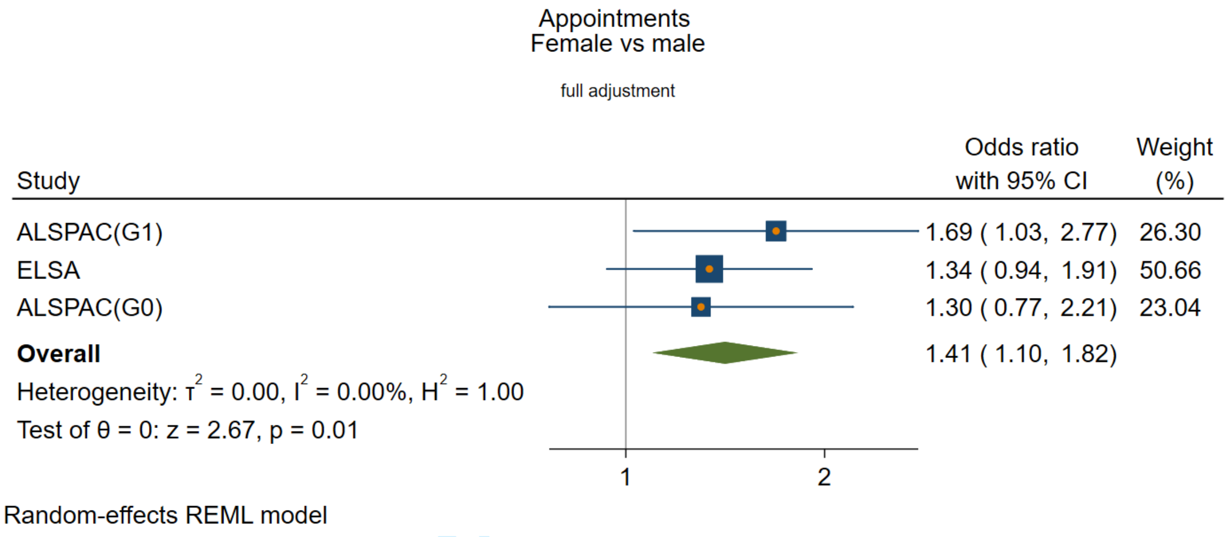

For peer review only - http://bmjopen.bmj.com/site/about/guidelines.xhtml

Occupational class
Unadjusted

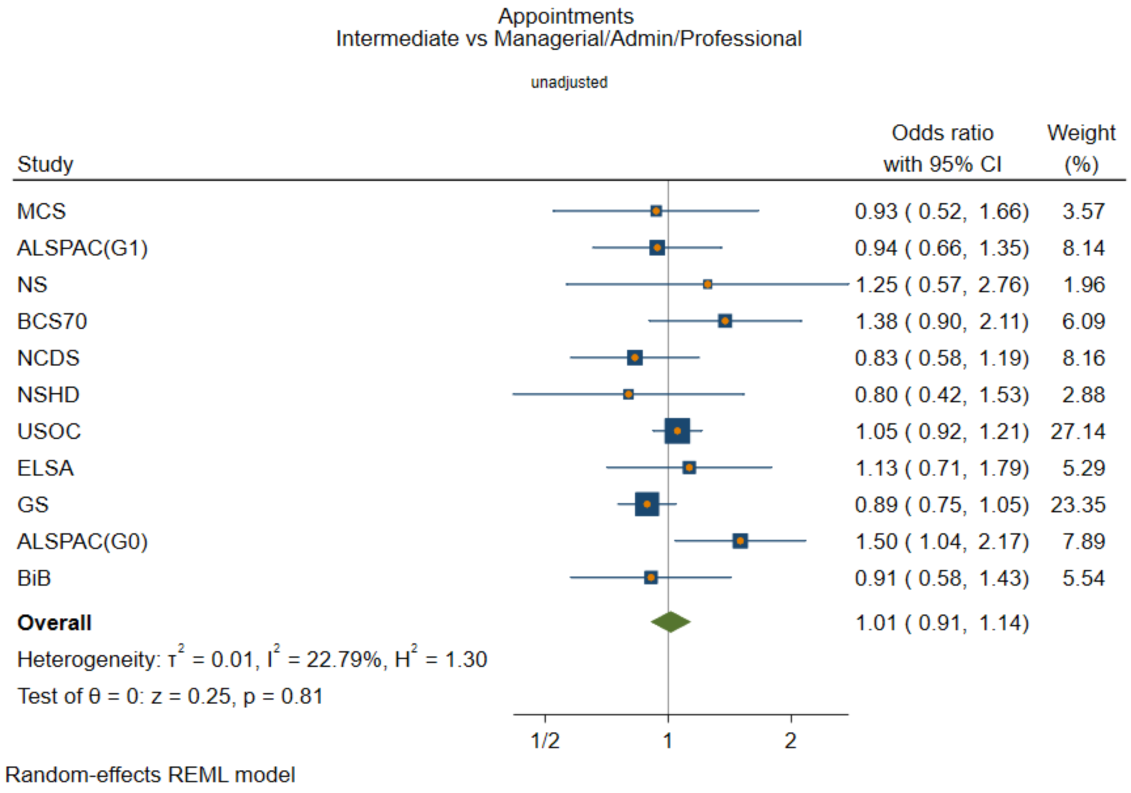

For peer review only - http://bmjopen.bmj.com/site/about/guidelines.xhtml

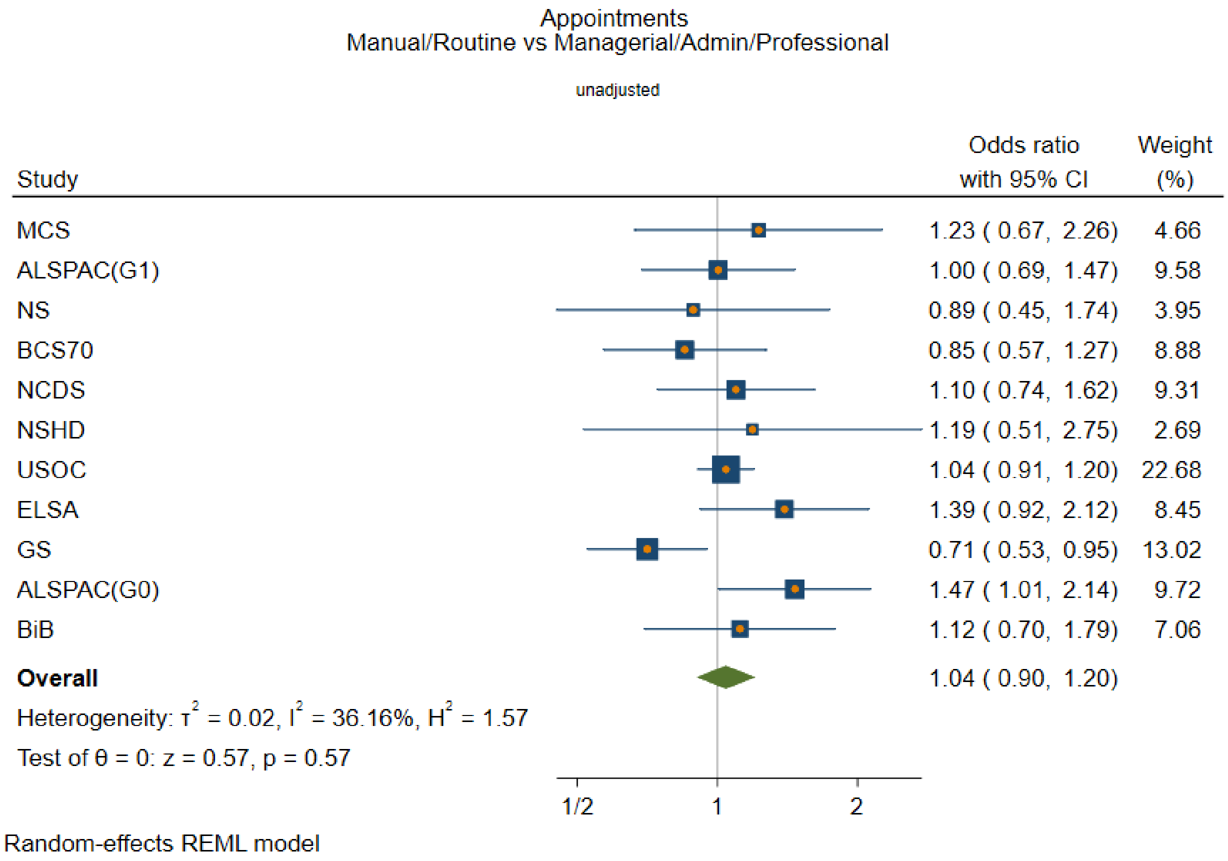
Appointments
Manual/Routine vs Managerial/Admin/Professional
unadjusted
Study
Odds ratio with 95% CI
Weight (%)
MCS 1.23 (0.67, 2.26) 4.66
ALSPAC(G1) 1.00 (0.69, 1.47) 9.58
NS 0.89 (0.45, 1.74) 3.95
BCS70 0.85 (0.57, 1.27) 8.88
NCDS 1.10 (0.74, 1.62) 9.31
NSHD 1.19 (0.51, 2.75) 2.69
USOC 1.04 (0.91, 1.20) 22.68
ELSA 1.39 (0.92, 2.12) 8.45
GS 0.71 (0.53, 0.95) 13.02
ALSPAC(G0) 1.47 (1.01, 2.14) 9.72
BiB 1.12 (0.70, 1.79) 7.06
Overall 1.04 (0.90, 1.20)
Heterogeneity: τ^2 = 0.02, I^2 = 36.16%, H^2 = 1.57
Test of θ = 0: z = 0.57, p = 0.57
1/2
1
2
Random-effects REML model

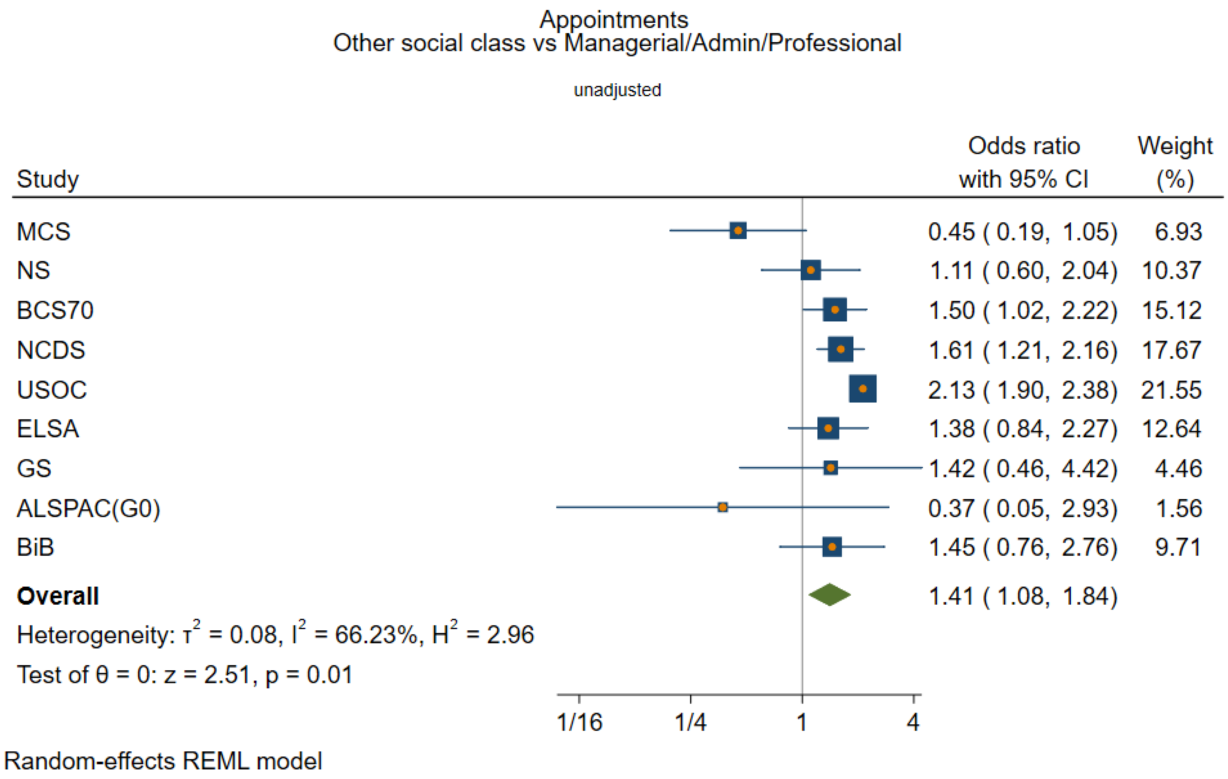
Appointments
Other social class vs Managerial/Admin/Professional
unadjusted
Study
Odds ratio with 95% CI
Weight (%)
MCS 0.45 (0.19, 1.05) 6.93
NS 1.11 (0.60, 2.04) 10.37
BCS70 1.50 (1.02, 2.22) 15.12
NCDS 1.61 (1.21, 2.16) 17.67
USOC 2.13 (1.90, 2.38) 21.55
ELSA 1.38 (0.84, 2.27) 12.64
GS 1.42 (0.46, 4.42) 4.46
ALSPAC(G0) 0.37 (0.05, 2.93) 1.56
BiB 1.45 (0.76, 2.76) 9.71
Overall 1.41 (1.08, 1.84)
Heterogeneity: τ^2 = 0.08, I^2 = 66.23%, H^2 = 2.96
Test of θ = 0: z = 2.51, p = 0.01
1/16
1/4
1
4
Random-effects REML model

Basic adjustment

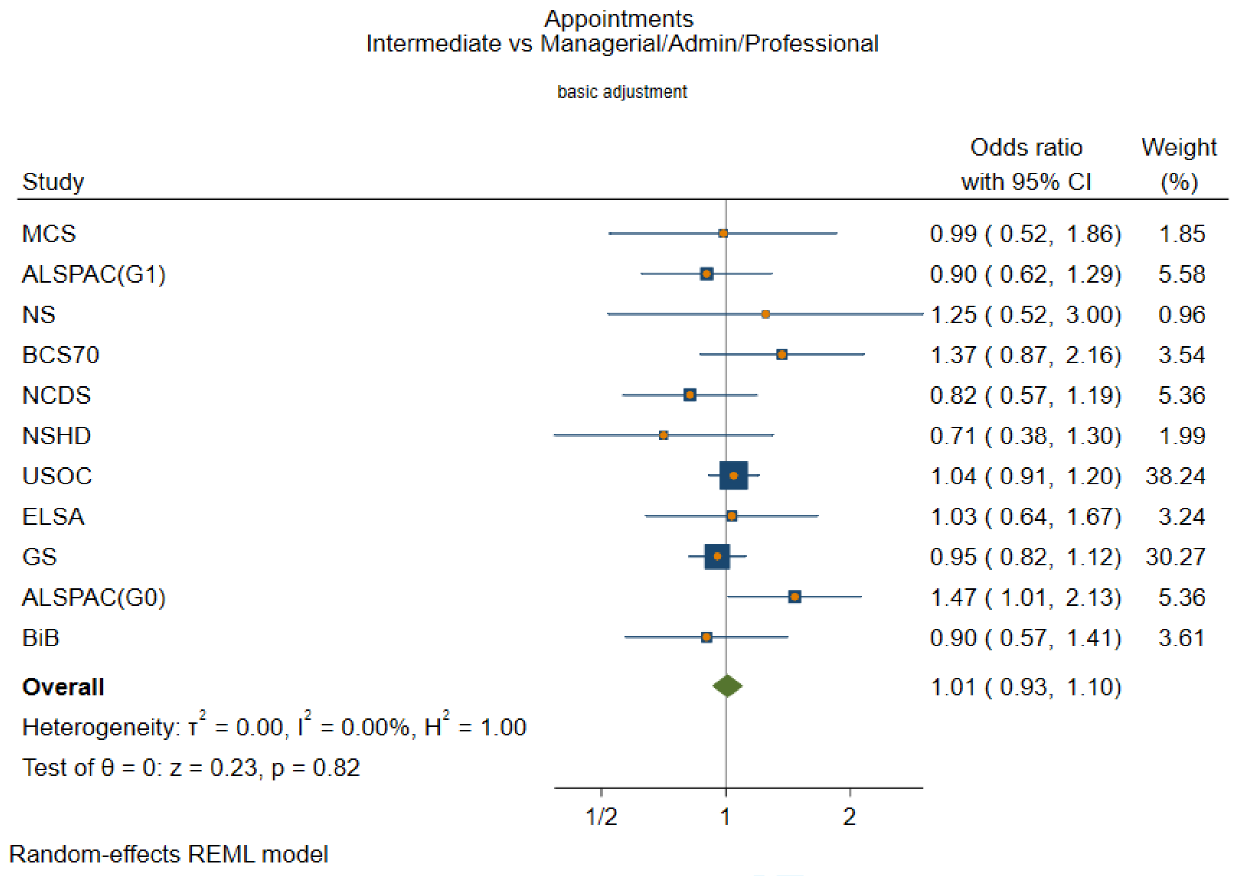

For peer review only - http://bmjopen.bmj.com/site/about/guidelines.xhtml

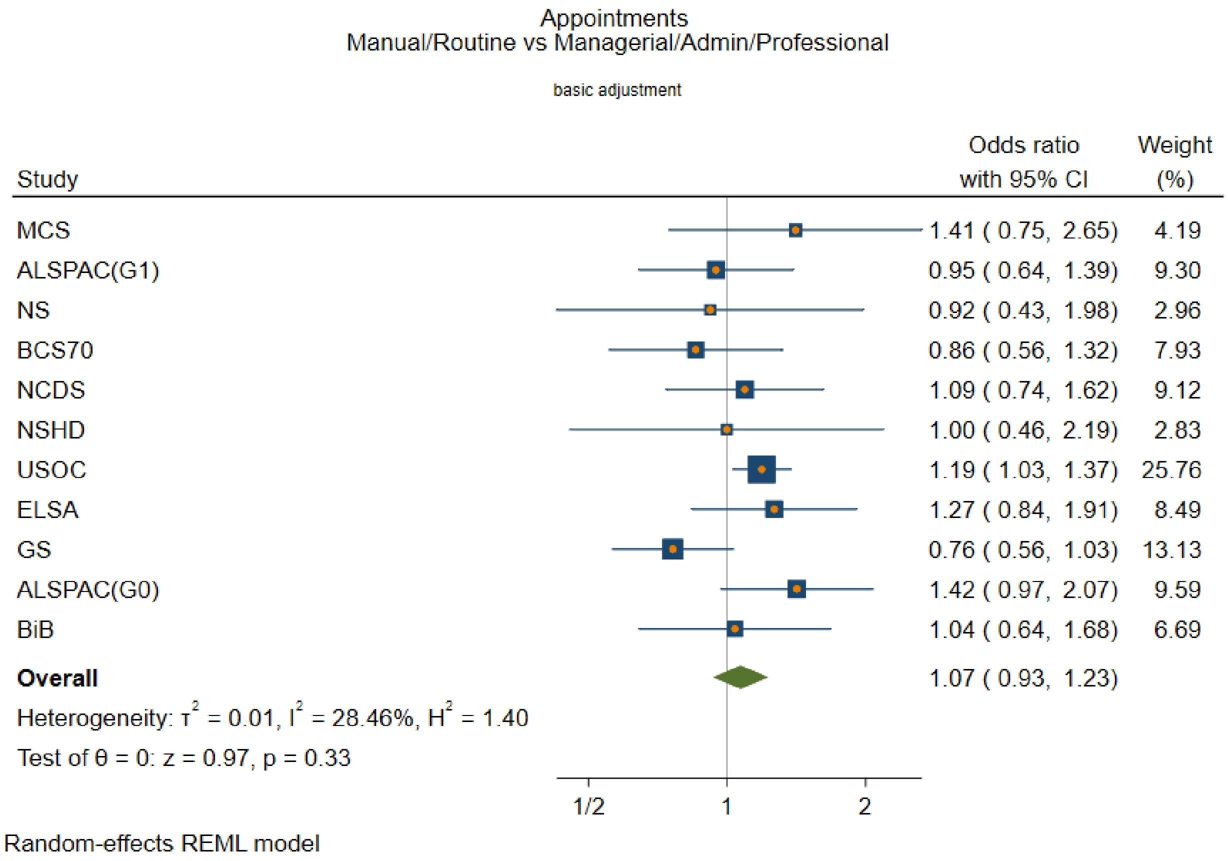

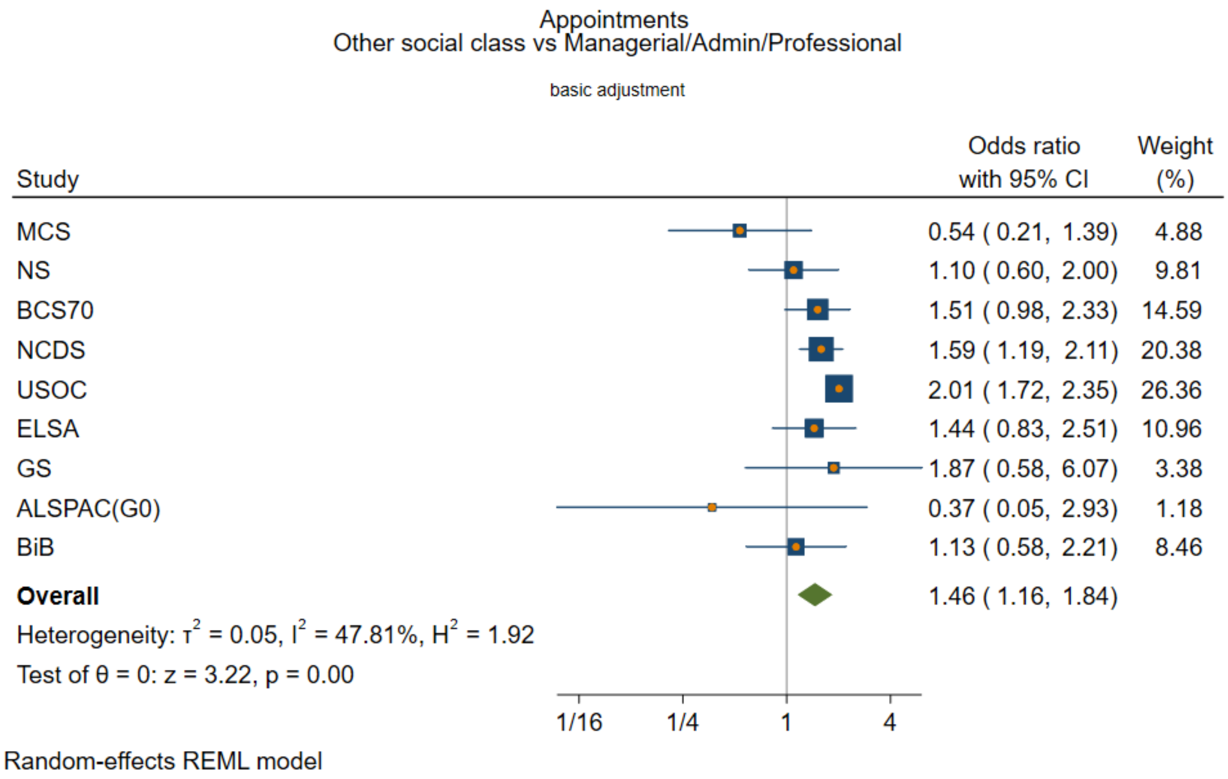

For peer review only - http://bmjopen.bmj.com/site/about/guidelines.xhtml

Full adjustment

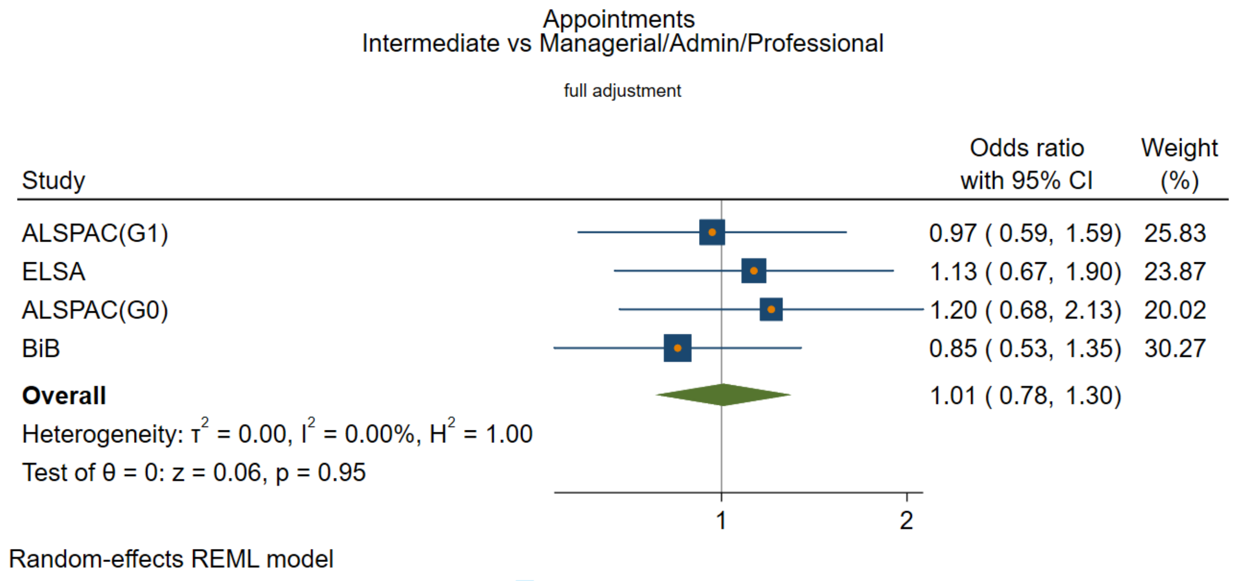

review only

For peer review only - http://bmjopen.bmj.com/site/about/guidelines.xhtml

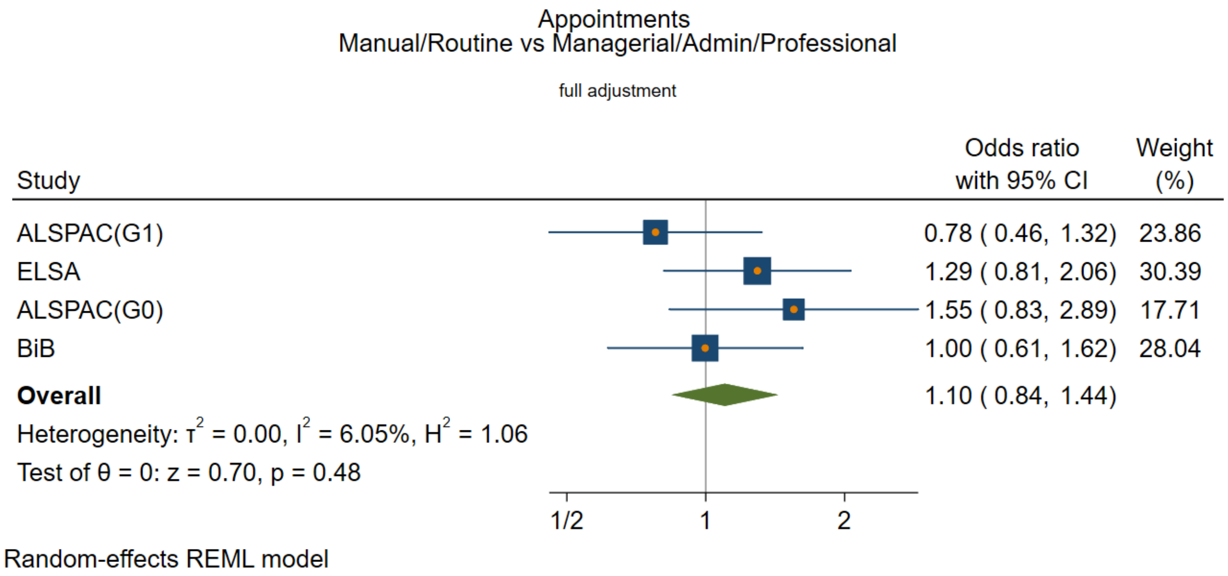

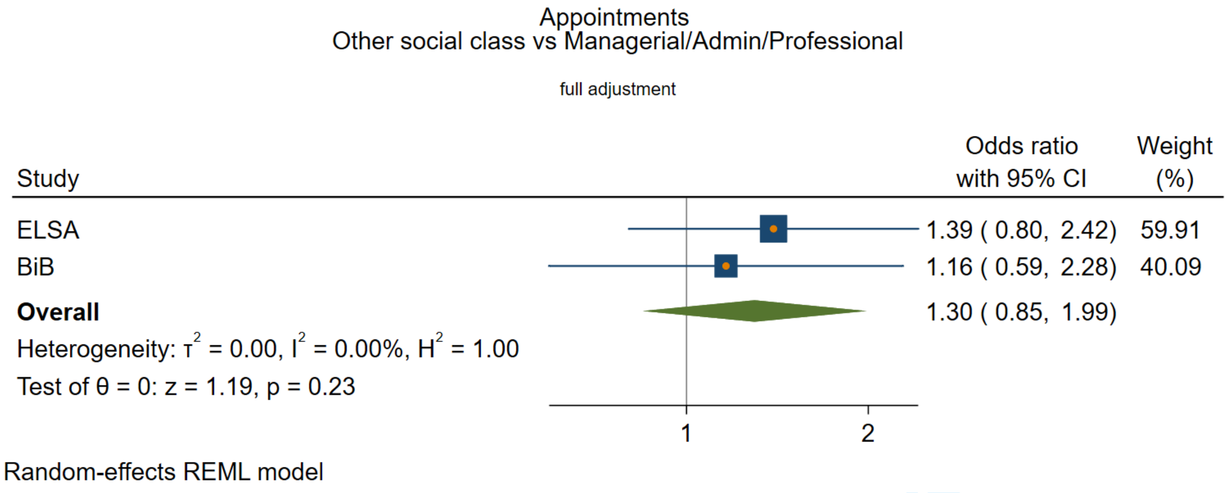

For peer review only - http://bmjopen.bmj.com/site/about/guidelines.xhtml

Education

Unadjusted

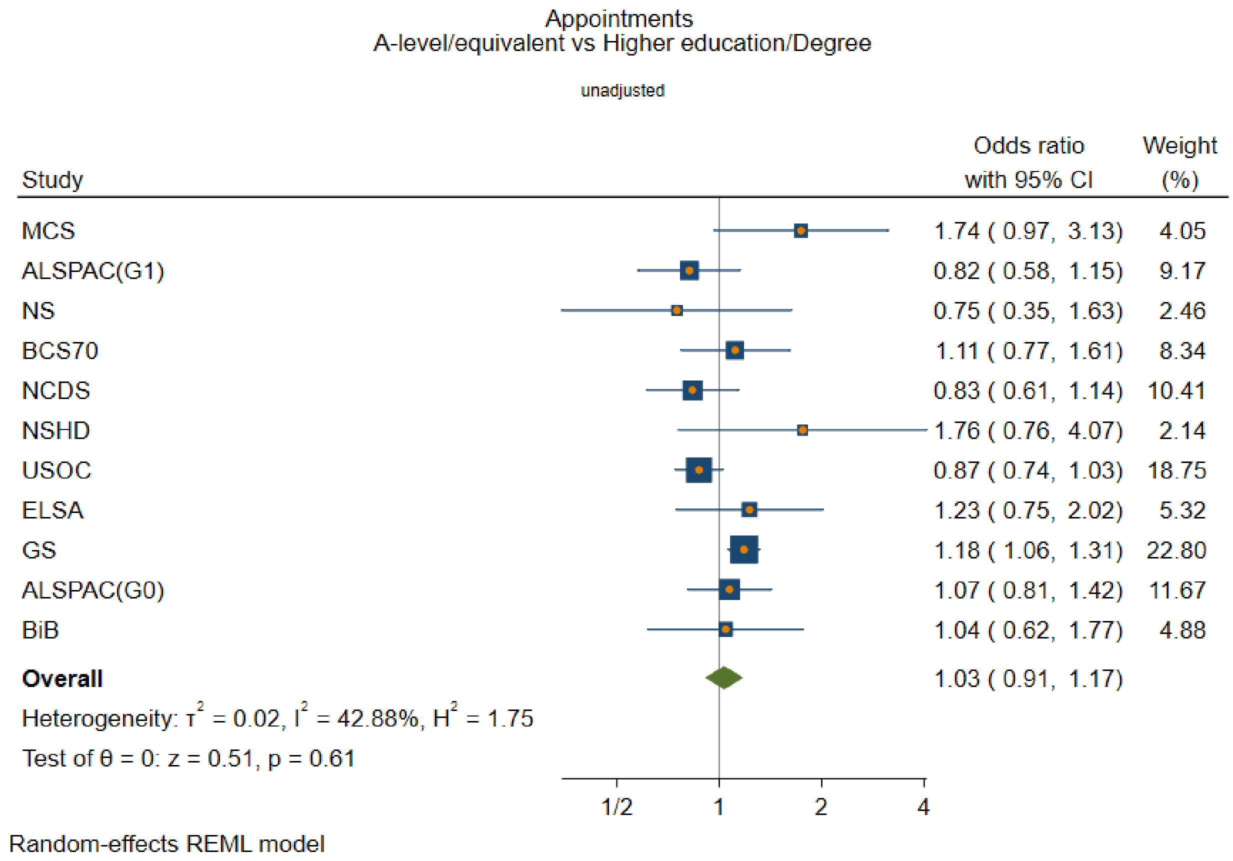

only

For peer review only - http://bmjopen.bmj.com/site/about/guidelines.xhtml

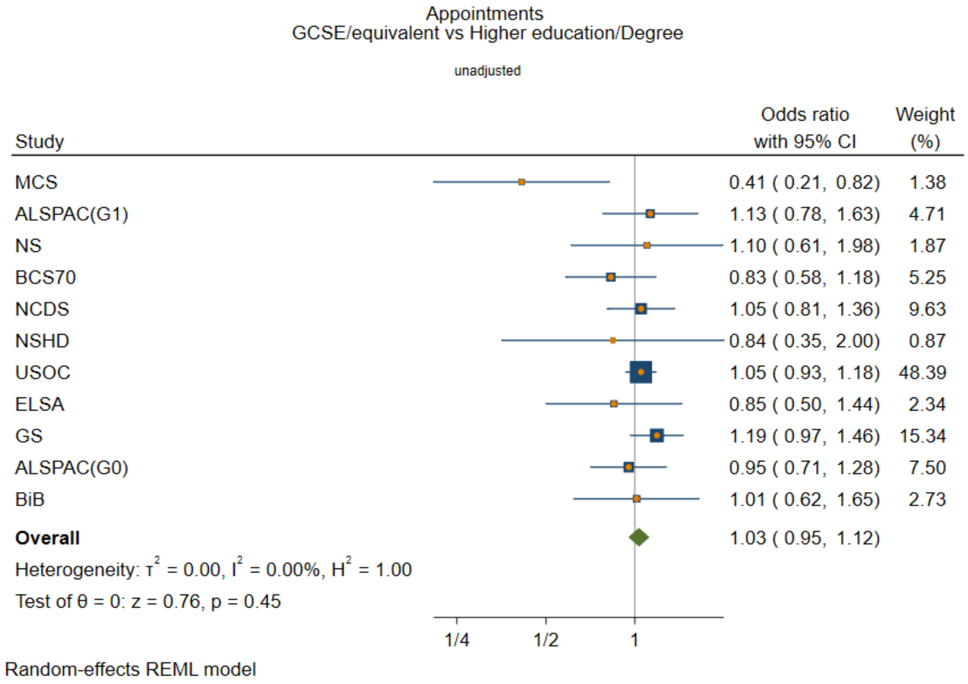
Appointments
GCSE/equivalent vs Higher education/Degree
unadjusted
Study
Odds ratio with 95% CI
Weight (%)
MCS 0.41 (0.21, 0.82) 1.38
ALSPAC(G1) 1.13 (0.78, 1.63) 4.71
NS 1.10 (0.61, 1.98) 1.87
BCS70 0.83 (0.58, 1.18) 5.25
NCDS 1.05 (0.81, 1.36) 9.63
NSHD 0.84 (0.35, 2.00) 0.87
USOC 1.05 (0.93, 1.18) 48.39
ELSA 0.85 (0.50, 1.44) 2.34
GS 1.19 (0.97, 1.46) 15.34
ALSPAC(G0) 0.95 (0.71, 1.28) 7.50
BiB 1.01 (0.62, 1.65) 2.73
Overall 1.03 (0.95, 1.12)
Heterogeneity: τ^2 = 0.00, I^2 = 0.00%, H^2 = 1.00
Test of θ = 0: z = 0.76, p = 0.45
1/4
1/2
1
Random-effects REML model

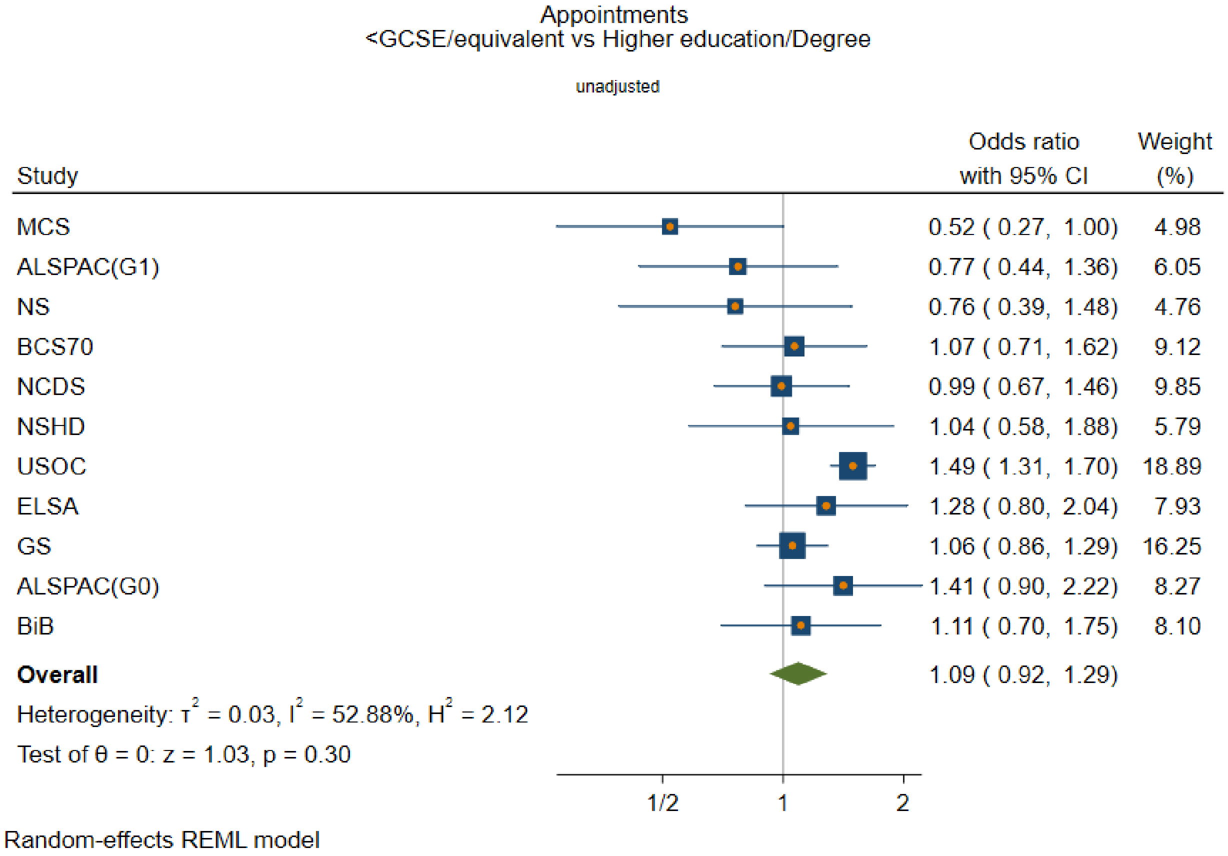
Appointments
<GCSE/equivalent vs Higher education/Degree
unadjusted
Study
Odds ratio with 95% CI
Weight (%)
MCS 0.52 (0.27, 1.00) 4.98
ALSPAC(G1) 0.77 (0.44, 1.36) 6.05
NS 0.76 (0.39, 1.48) 4.76
BCS70 1.07 (0.71, 1.62) 9.12
NCDS 0.99 (0.67, 1.46) 9.85
NSHD 1.04 (0.58, 1.88) 5.79
USOC 1.49 (1.31, 1.70) 18.89
ELSA 1.28 (0.80, 2.04) 7.93
GS 1.06 (0.86, 1.29) 16.25
ALSPAC(G0) 1.41 (0.90, 2.22) 8.27
BiB 1.11 (0.70, 1.75) 8.10
Overall 1.09 (0.92, 1.29)
Heterogeneity: τ^2 = 0.03, I^2 = 52.88%, H^2 = 2.12
Test of θ = 0: z = 1.03, p = 0.30
1/2
1
2
Random-effects REML model

Basic adjustment

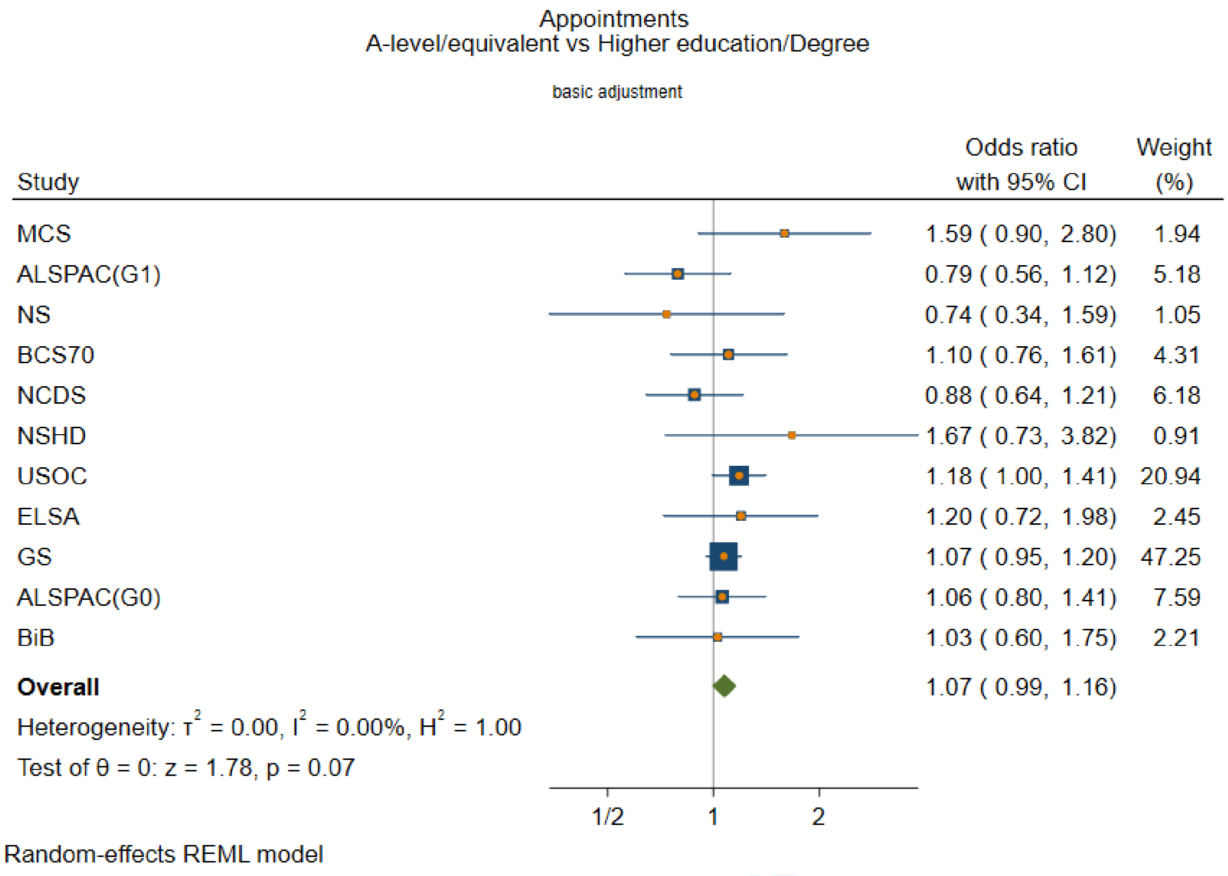

only

For peer review only - http://bmjopen.bmj.com/site/about/guidelines.xhtml

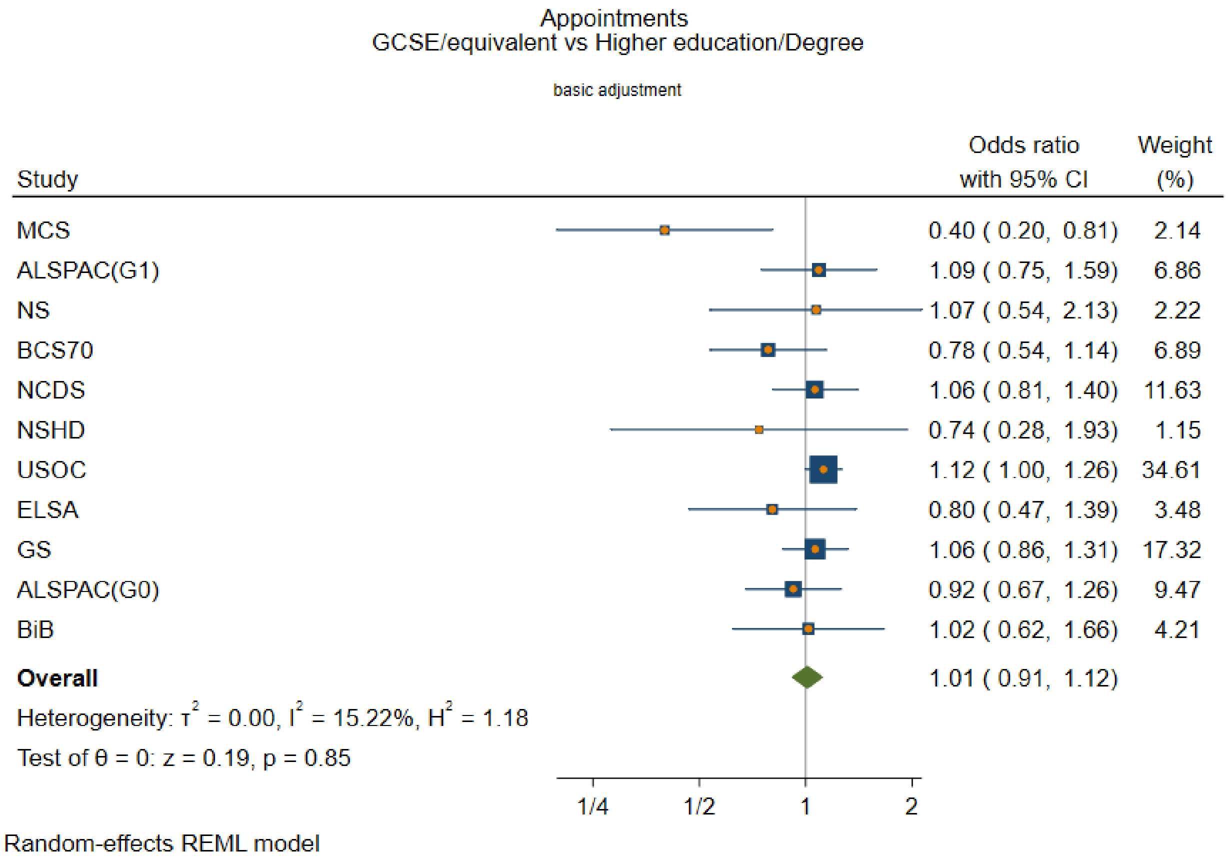
Appointments
GCSE/equivalent vs Higher education/Degree
basic adjustment
Study
Odds ratio with 95% CI
Weight (%)
MCS 0.40 (0.20, 0.81) 2.14
ALSPAC(G1) 1.09 (0.75, 1.59) 6.86
NS 1.07 (0.54, 2.13) 2.22
BCS70 0.78 (0.54, 1.14) 6.89
NCDS 1.06 (0.81, 1.40) 11.63
NSHD 0.74 (0.28, 1.93) 1.15
USOC 1.12 (1.00, 1.26) 34.61
ELSA 0.80 (0.47, 1.39) 3.48
GS 1.06 (0.86, 1.31) 17.32
ALSPAC(G0) 0.92 (0.67, 1.26) 9.47
BiB 1.02 (0.62, 1.66) 4.21
Overall 1.01 (0.91, 1.12)
Heterogeneity: τ^2 = 0.00, I^2 = 15.22%, H^2 = 1.18
Test of θ = 0: z = 0.19, p = 0.85
1/4
1/2
1
2
Random-effects REML model

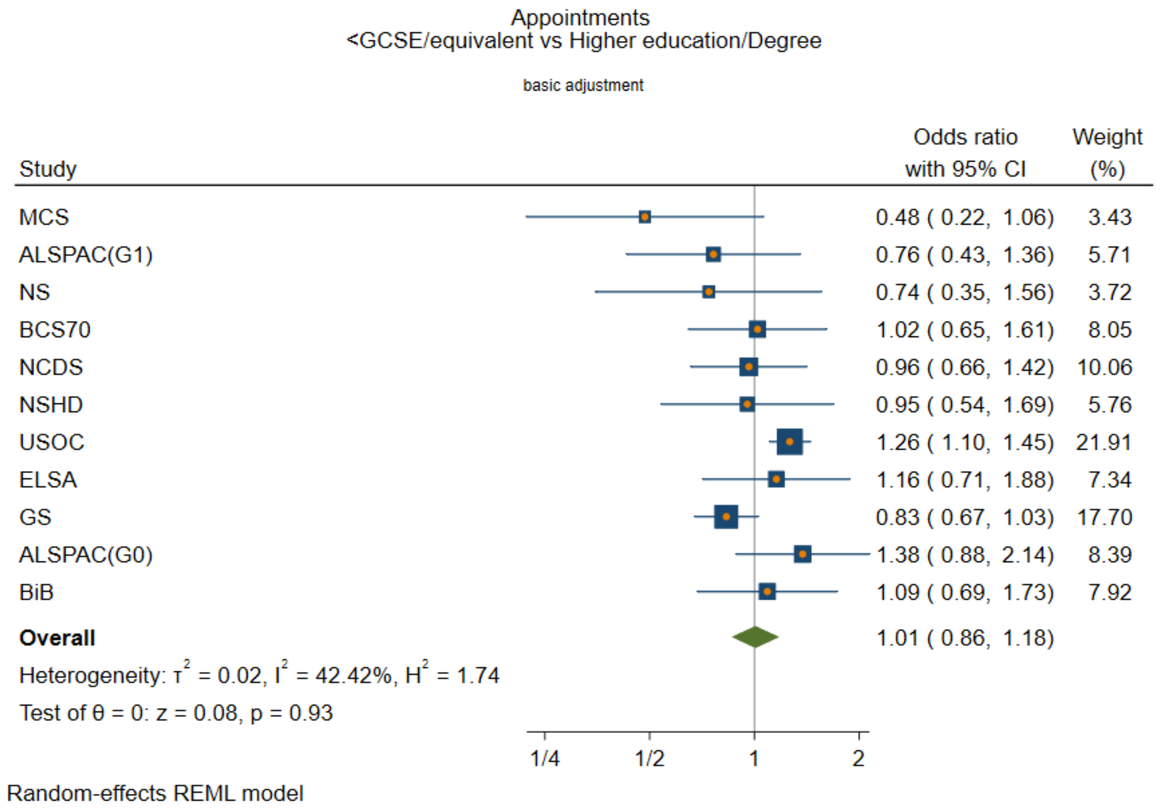
Appointments
<GCSE/equivalent vs Higher education/Degree
basic adjustment
Study
Odds ratio with 95% CI
Weight (%)
MCS 0.48 (0.22, 1.06) 3.43
ALSPAC(G1) 0.76 (0.43, 1.36) 5.71
NS 0.74 (0.35, 1.56) 3.72
BCS70 1.02 (0.65, 1.61) 8.05
NCDS 0.96 (0.66, 1.42) 10.06
NSHD 0.95 (0.54, 1.69) 5.76
USOC 1.26 (1.10, 1.45) 21.91
ELSA 1.16 (0.71, 1.88) 7.34
GS 0.83 (0.67, 1.03) 17.70
ALSPAC(G0) 1.38 (0.88, 2.14) 8.39
BiB 1.09 (0.69, 1.73) 7.92
Overall 1.01 (0.86, 1.18)
Heterogeneity: τ^2 = 0.02, I^2 = 42.42%, H^2 = 1.74
Test of θ = 0: z = 0.08, p = 0.93
1/4
1/2
1
2
Random-effects REML model

Full Adjustment

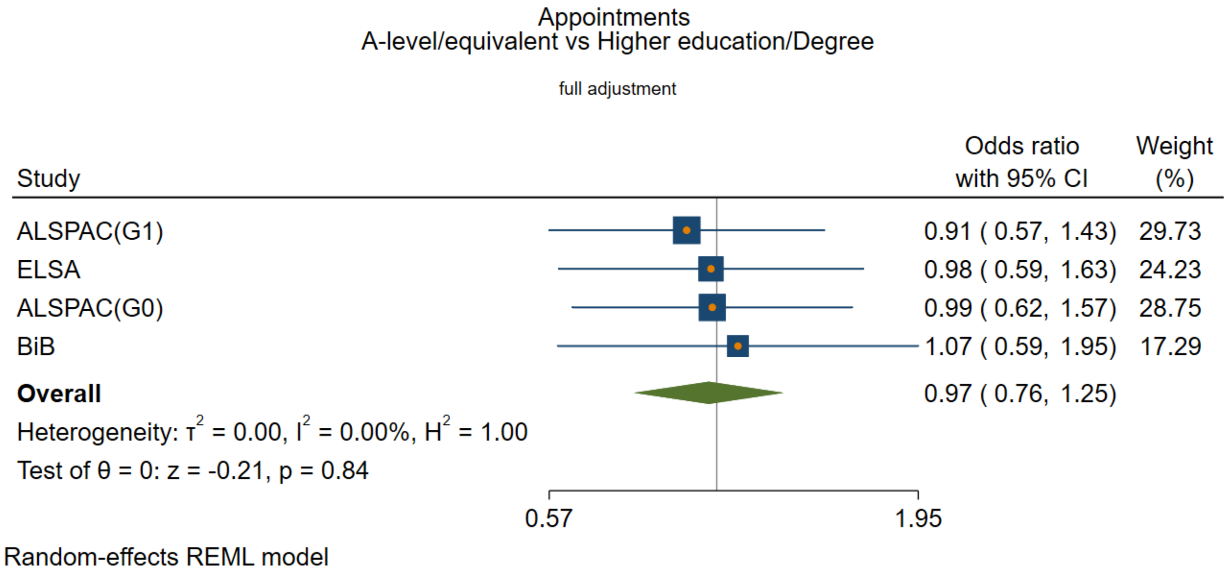

For peer review only

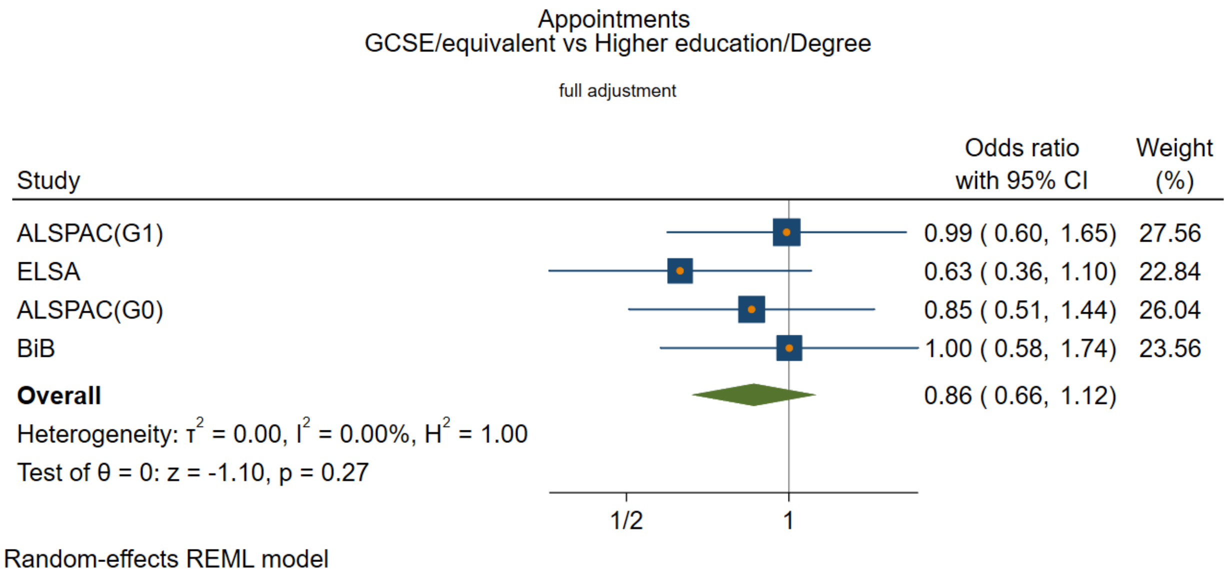

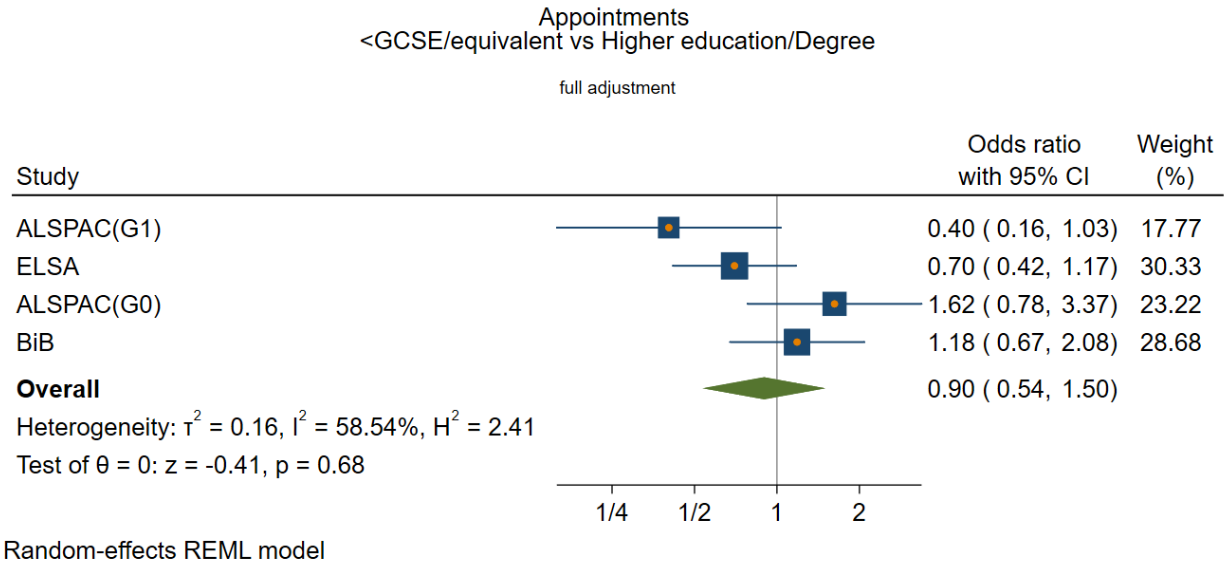

For peer review only - http://bmjopen.bmj.com/site/about/guidelines.xhtml

Age
<u>Unadjusted</u>

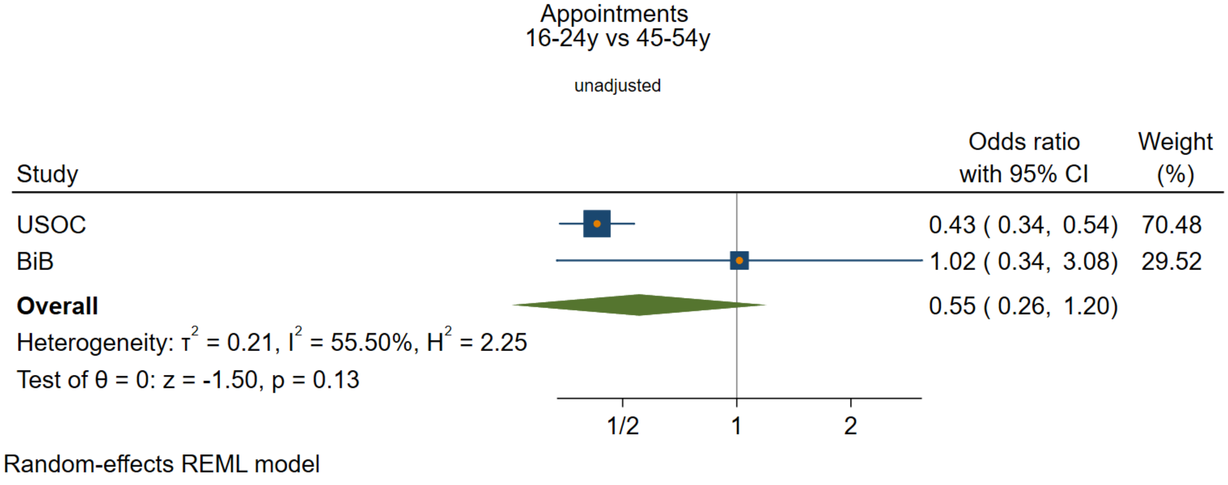

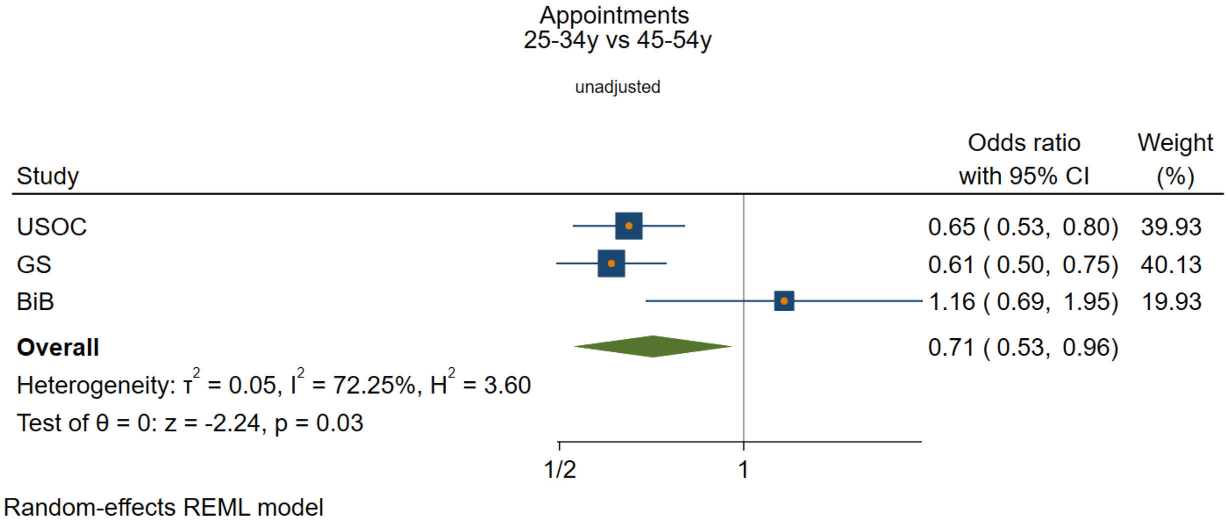

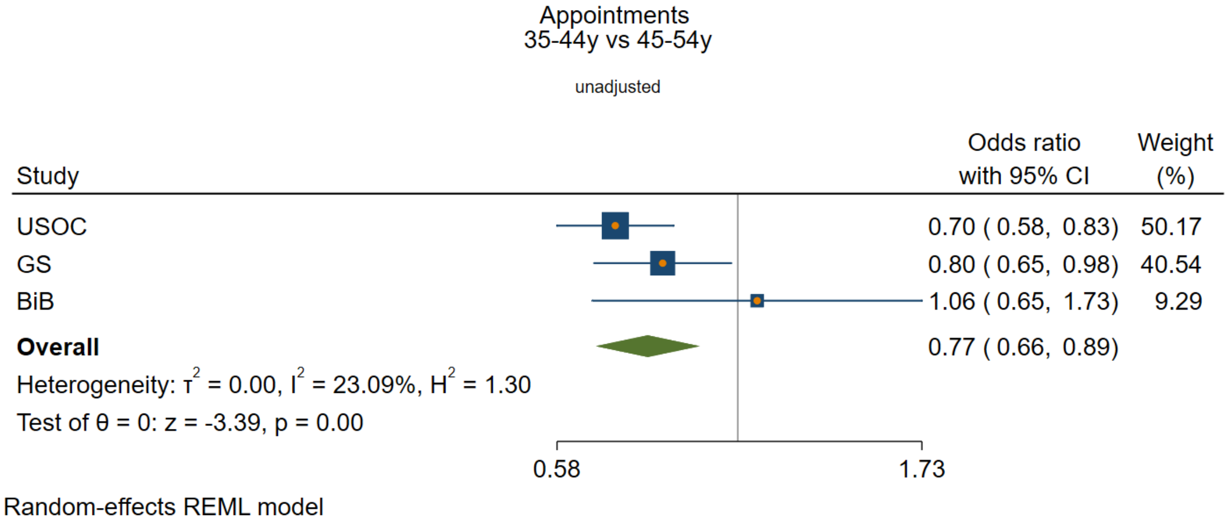

For peer review only - http://bmjopen.bmj.com/site/about/guidelines.xhtml

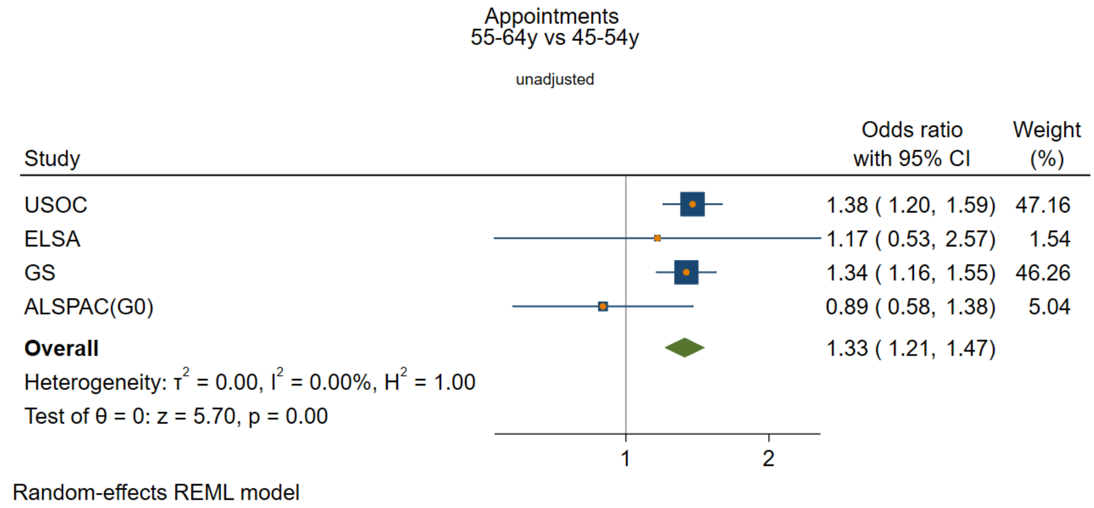

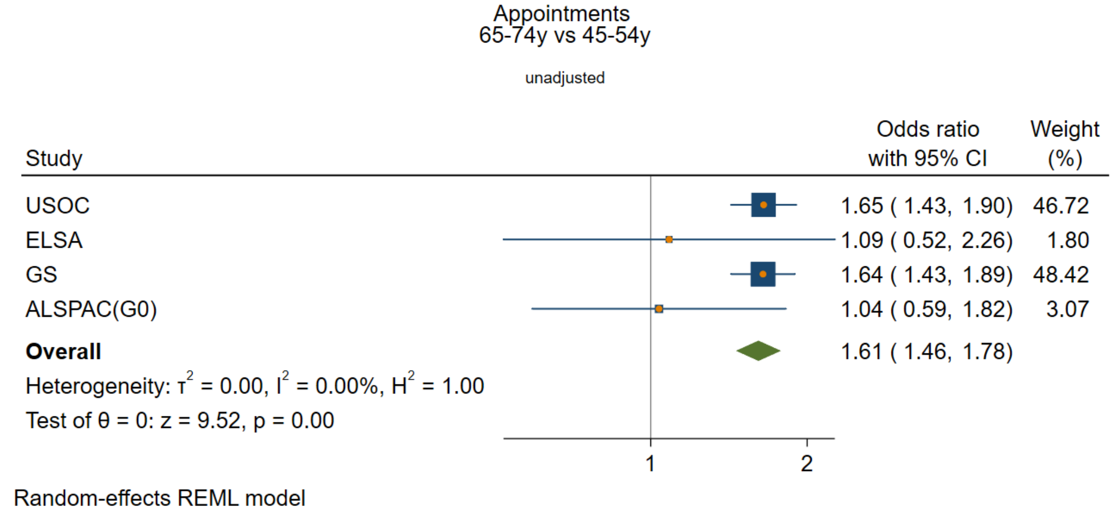

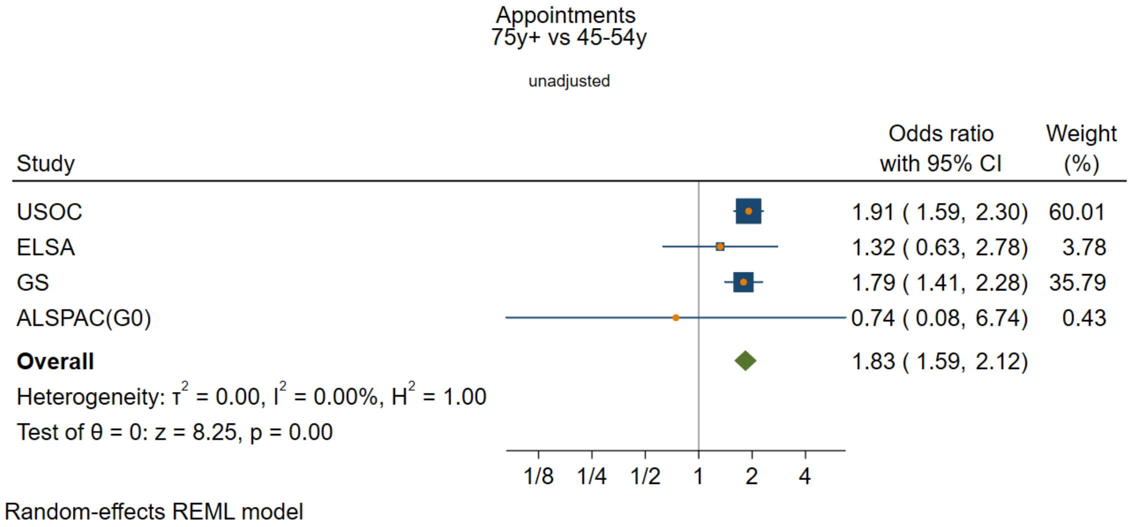

For peer review only - http://bmjopen.bmj.com/site/about/guidelines.xhtml

Basic adjustment

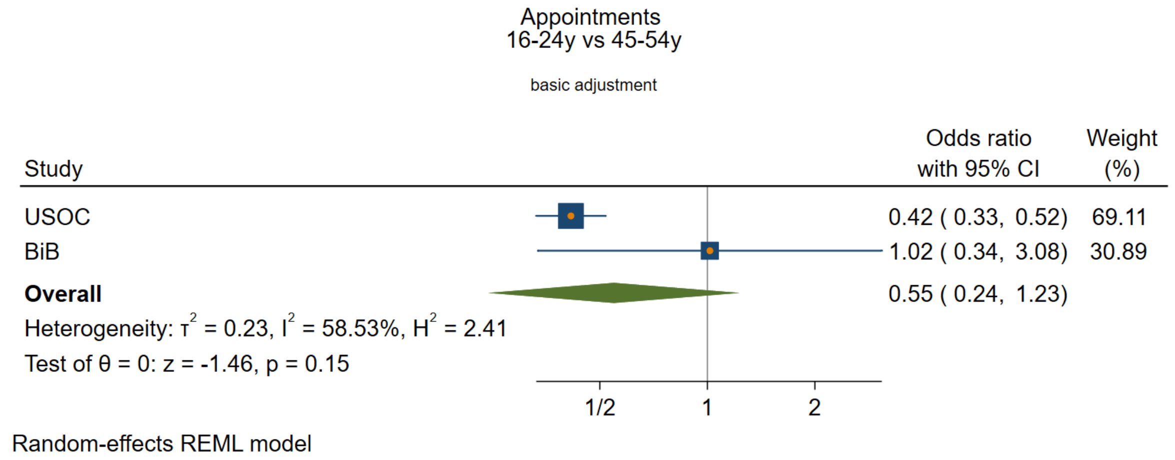

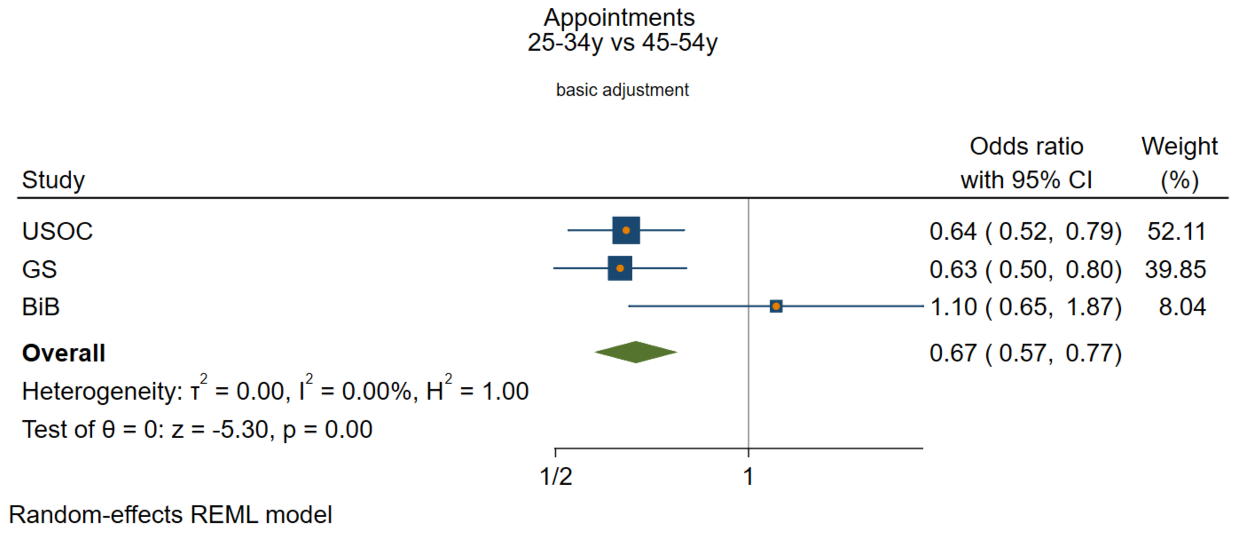

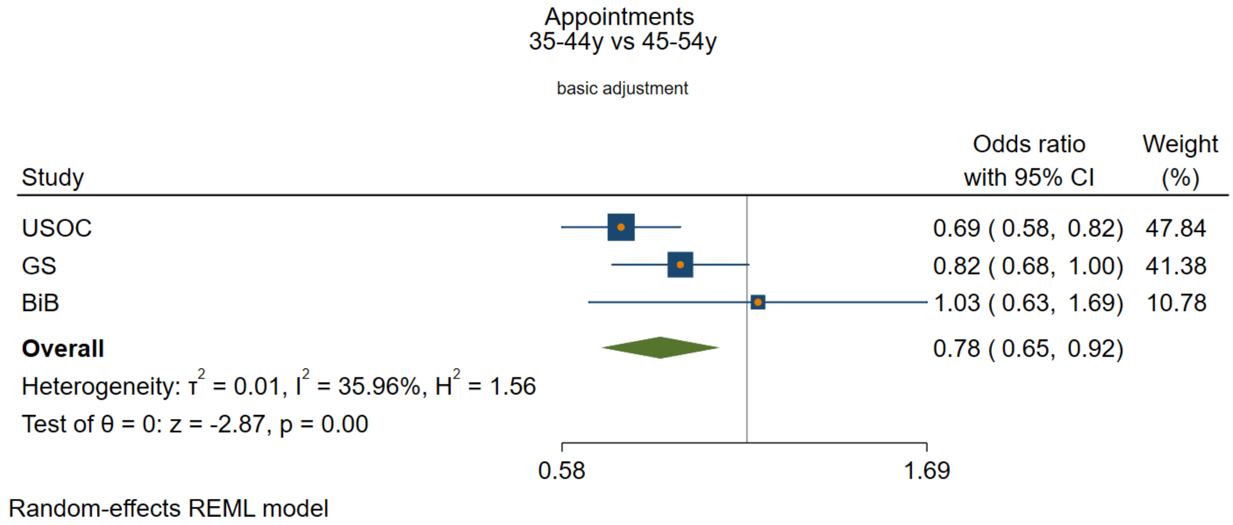

For peer review only - http://bmjopen.bmj.com/site/about/guidelines.xhtml

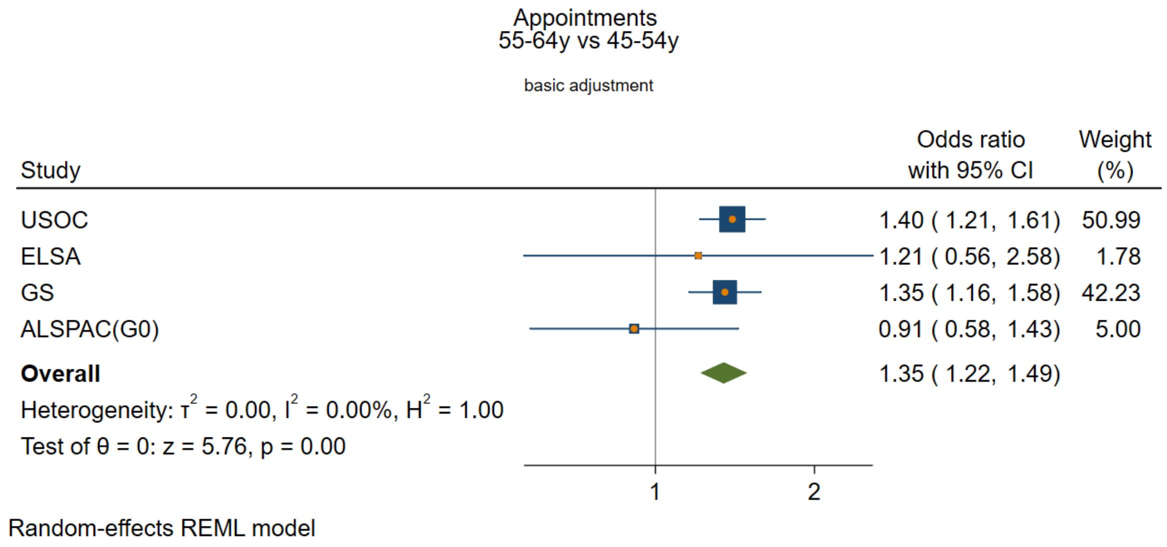

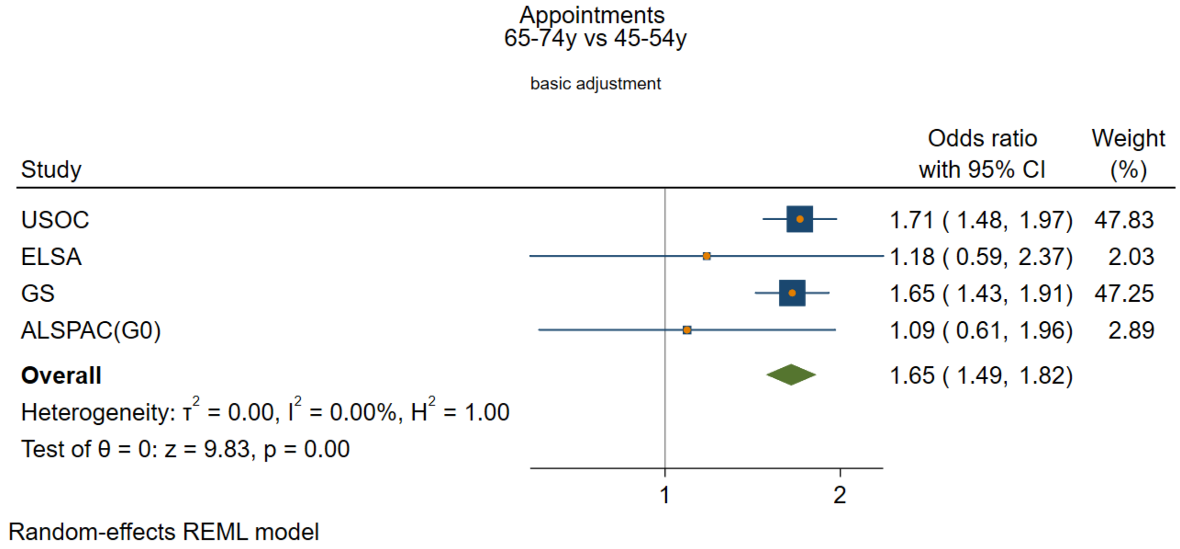

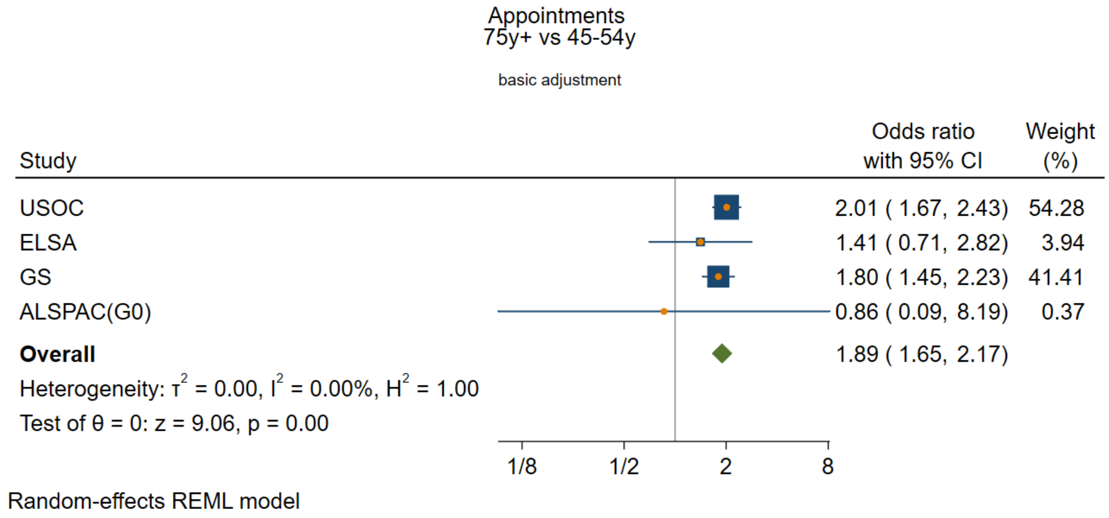

For peer review only - http://bmjopen.bmj.com/site/about/guidelines.xhtml

Full adjustment

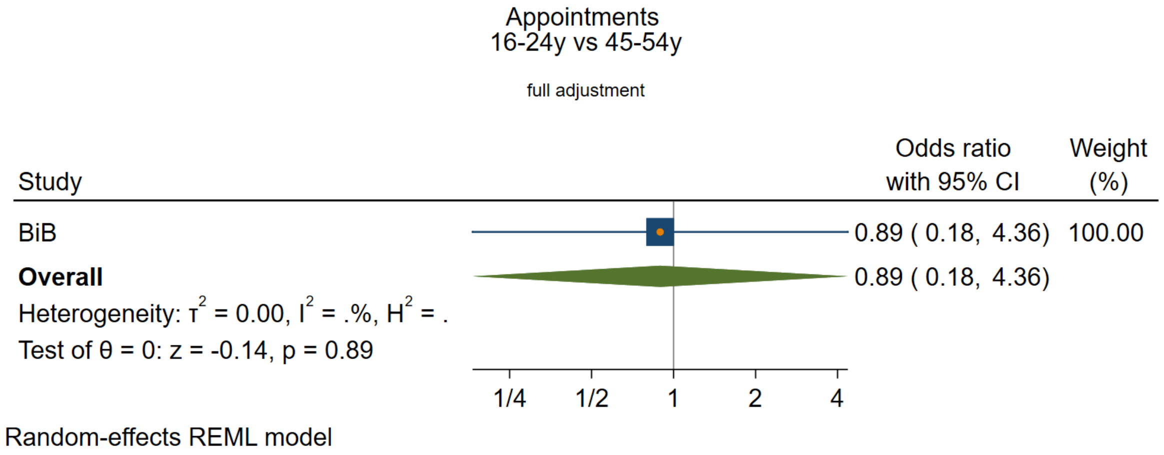

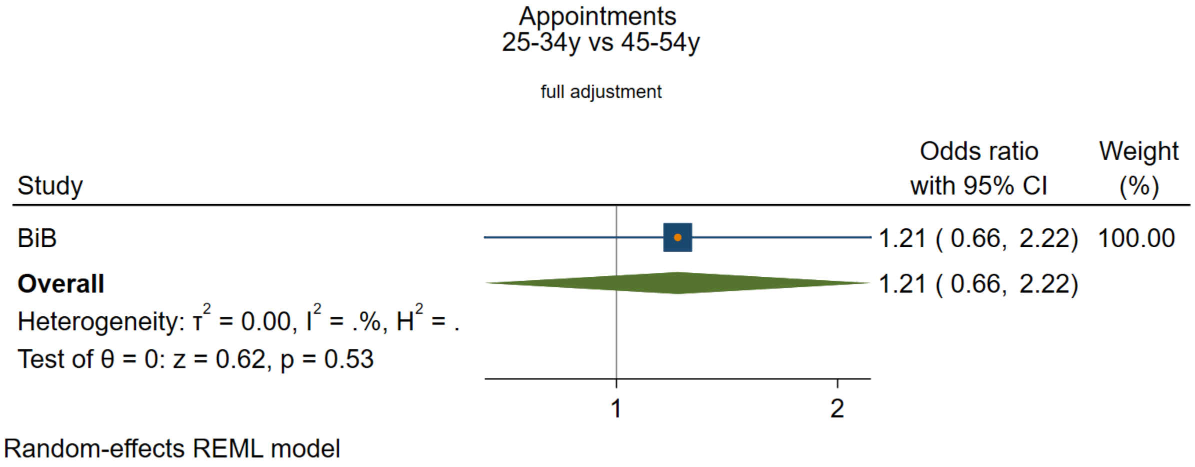

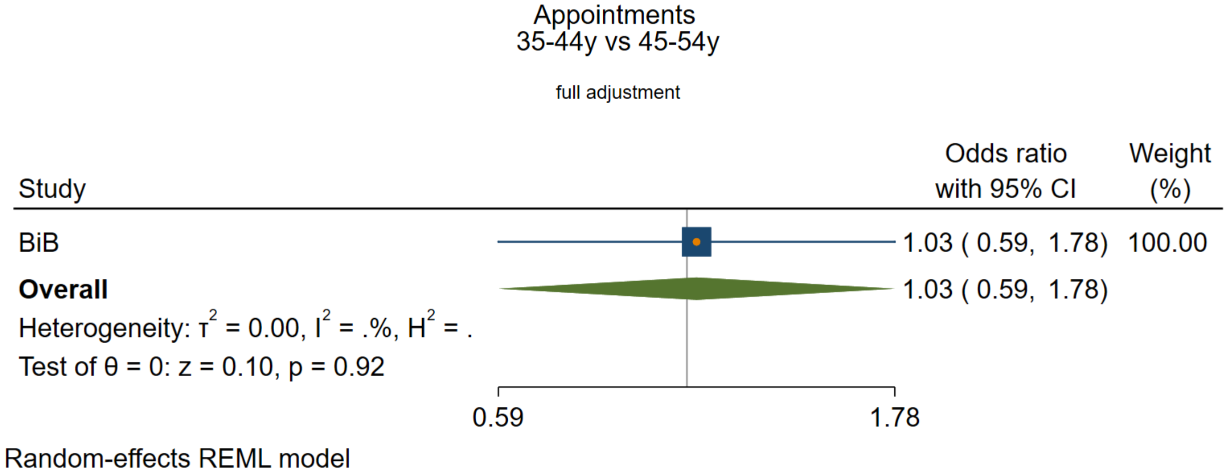

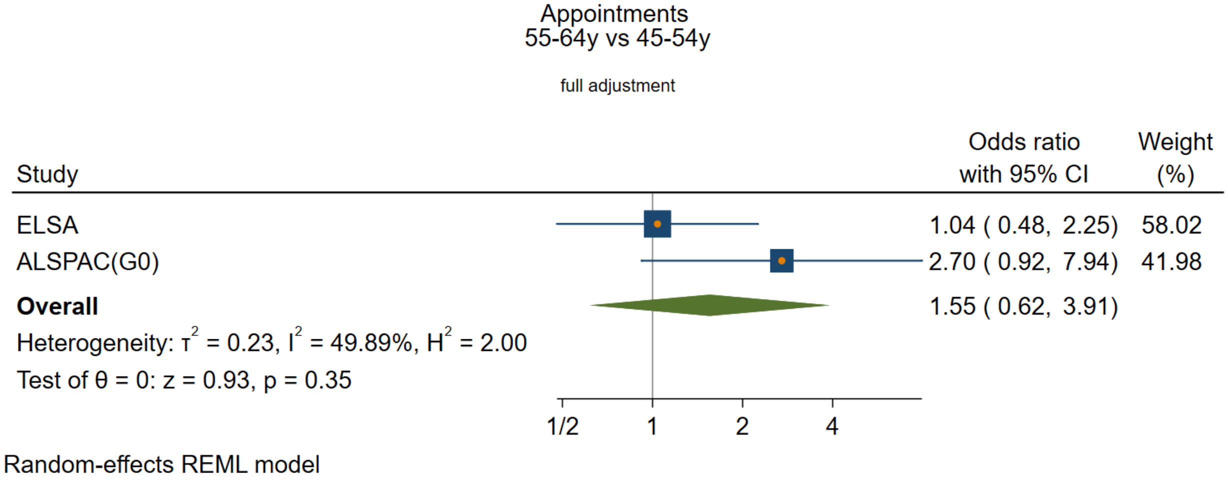
Appointments
55-64y vs 45-54y
full adjustment
Study
Odds ratio
with 95% CI
Weight
(%)
ELSA
1.04 (0.48, 2.25)
58.02
ALSPAC(G0)
2.70 (0.92, 7.94)
41.98
Overall
1.55 (0.62, 3.91)
Heterogeneity: τ² = 0.23, I² = 49.89%, H² = 2.00
Test of θ = 0: z = 0.93, p = 0.35
1/2
1
2
4
Random-effects REML model

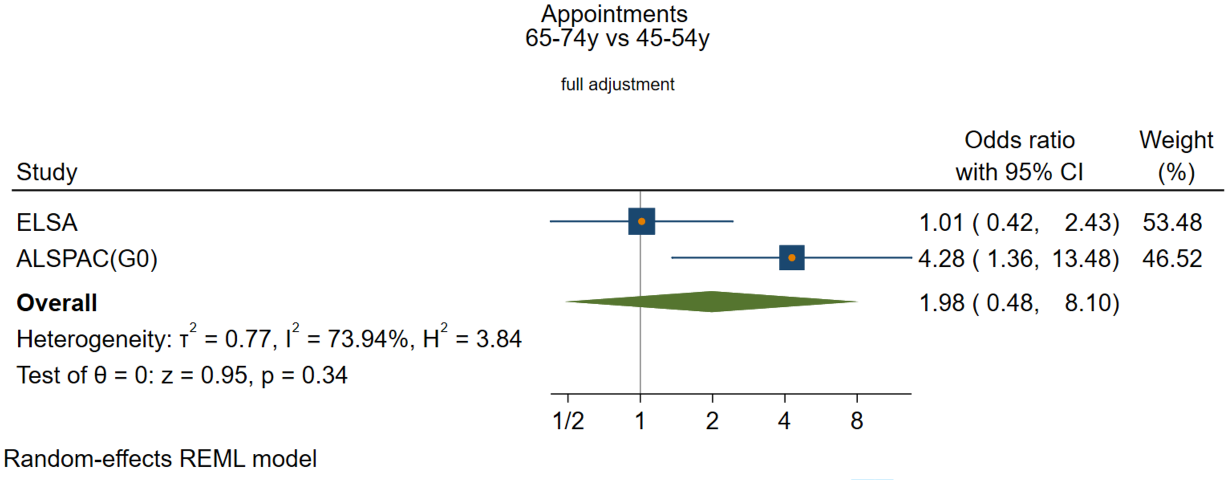
Appointments
65-74y vs 45-54y
full adjustment
Study
Odds ratio
with 95% CI
Weight
(%)
ELSA
1.01 (0.42, 2.43)
53.48
ALSPAC(G0)
4.28 (1.36, 13.48)
46.52
Overall
1.98 (0.48, 8.10)
Heterogeneity: τ² = 0.77, I² = 73.94%, H² = 3.84
Test of θ = 0: z = 0.95, p = 0.34
1/2
1
2
4
8
Random-effects REML model

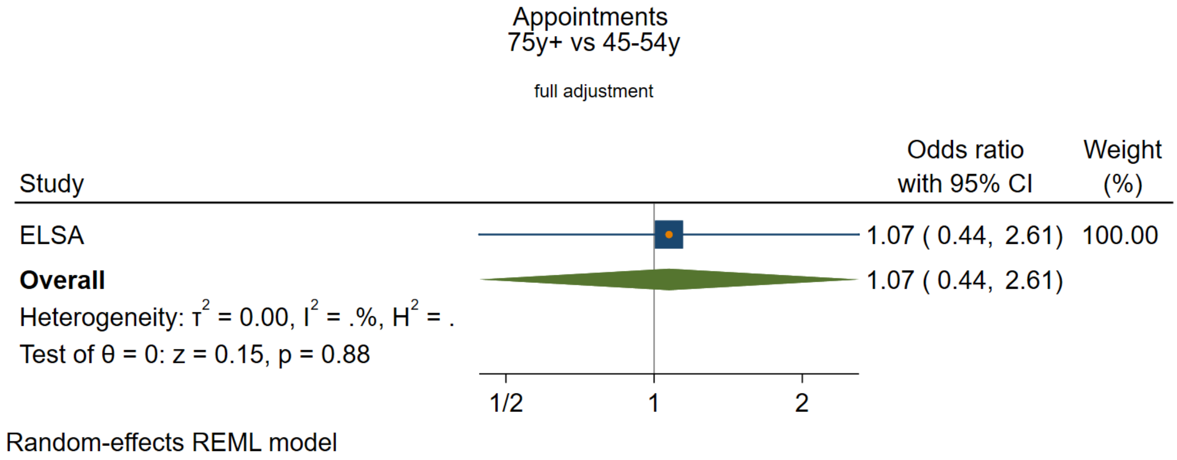
Appointments
75y+ vs 45-54y
full adjustment
Study
Odds ratio
with 95% CI
Weight
(%)
ELSA
1.07 (0.44, 2.61)
100.00
Overall
1.07 (0.44, 2.61)
Heterogeneity: τ² = 0.00, I² = .%, H² = .
Test of θ = 0: z = 0.15, p = 0.88
1/2
1
2
Random-effects REML model

Ethnicity
<u>Unadjusted</u>

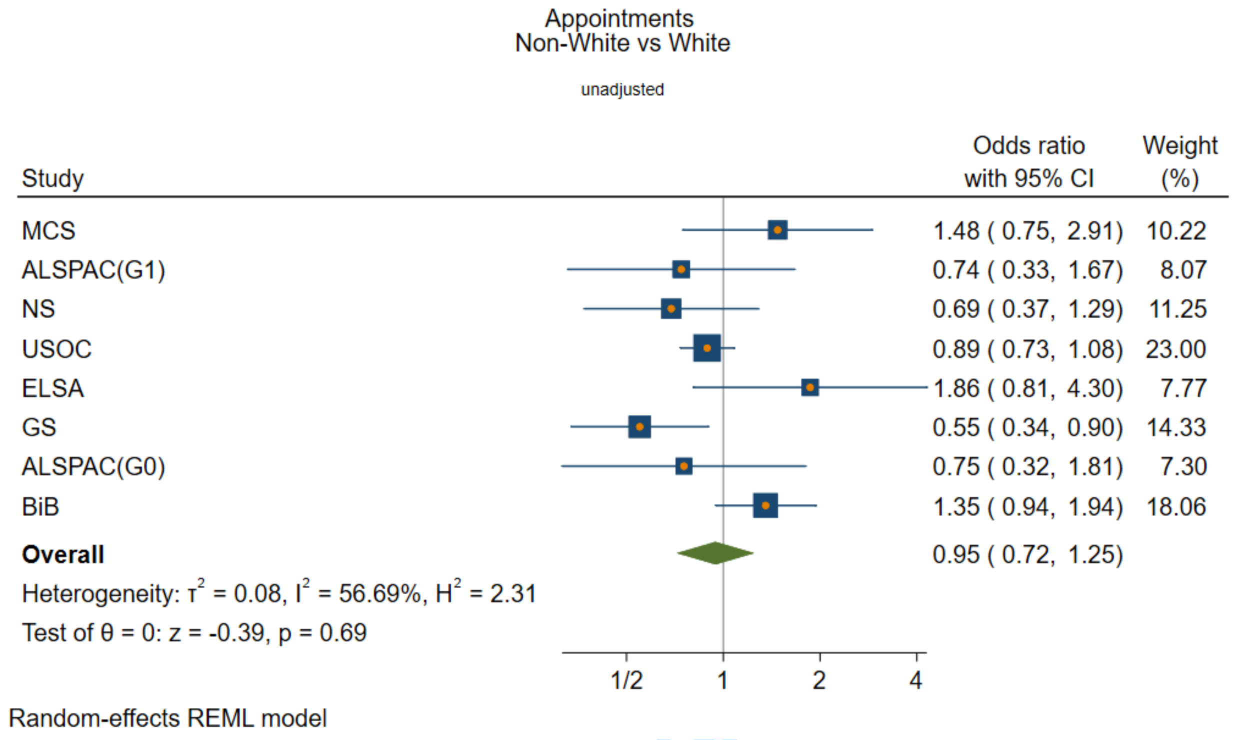

For peer review only - http://bmjopen.bmj.com/site/about/guidelines.xhtml

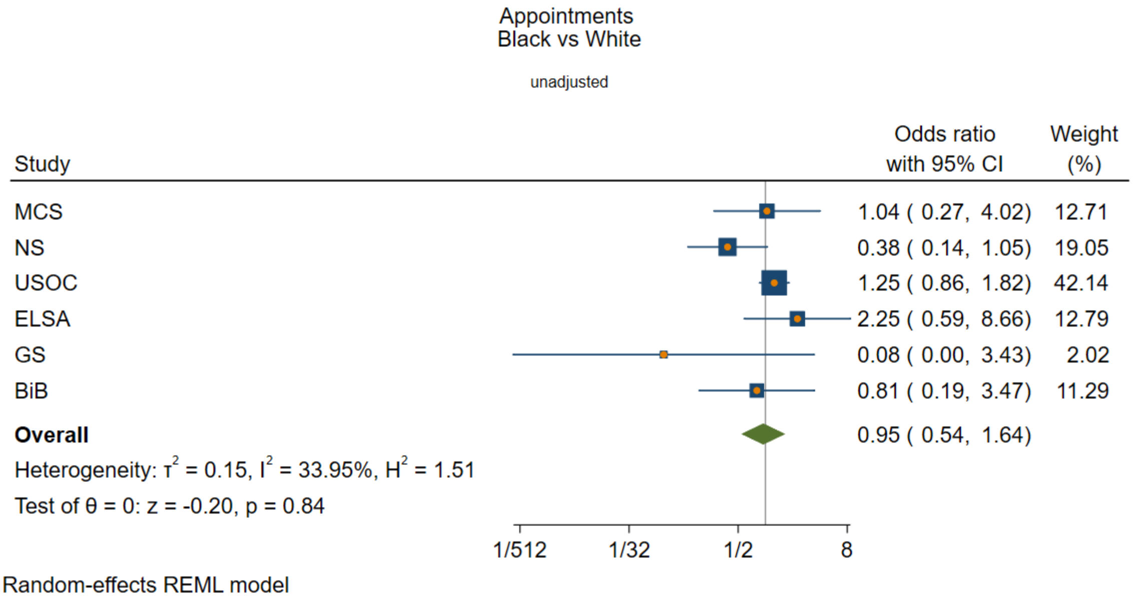

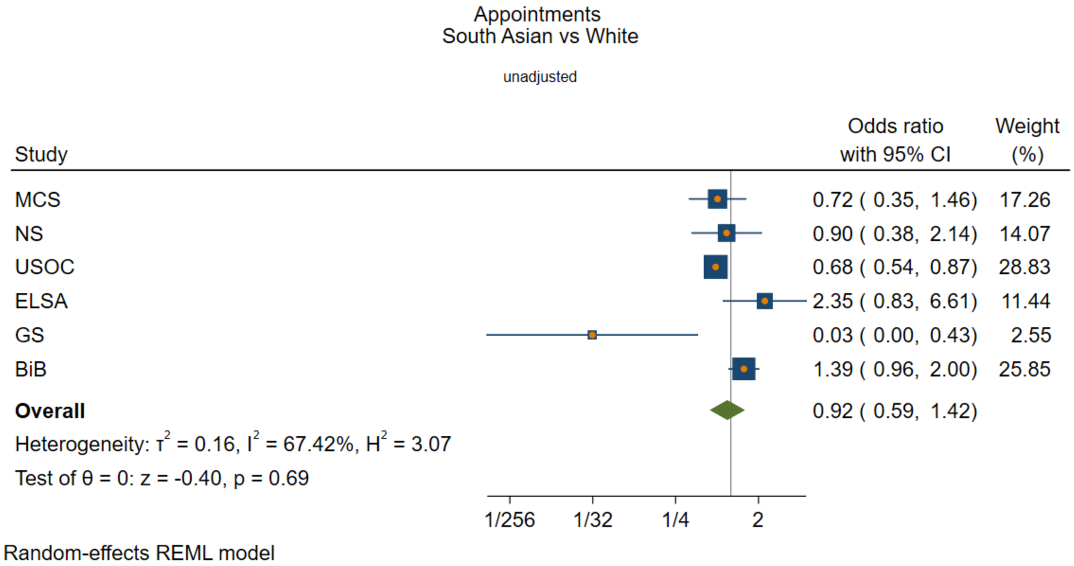

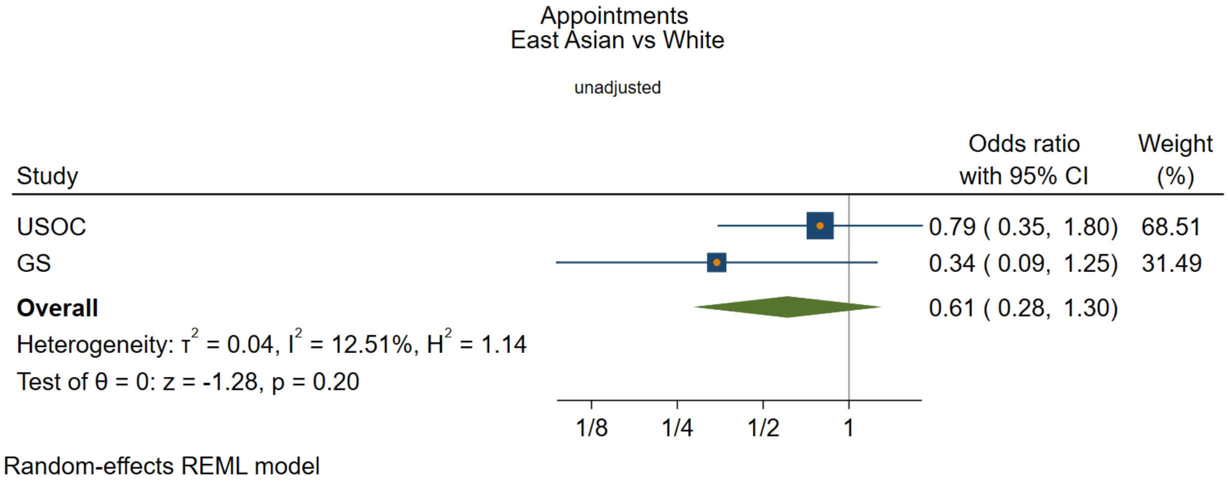

For peer review only - http://bmjopen.bmj.com/site/about/guidelines.xhtml

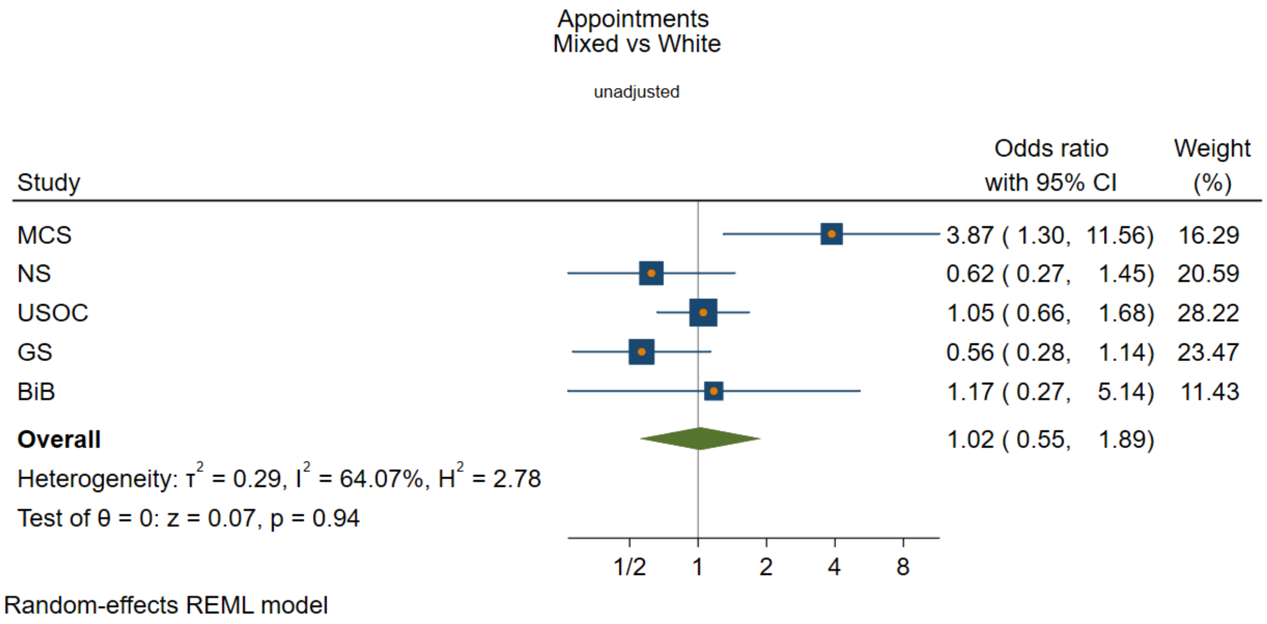

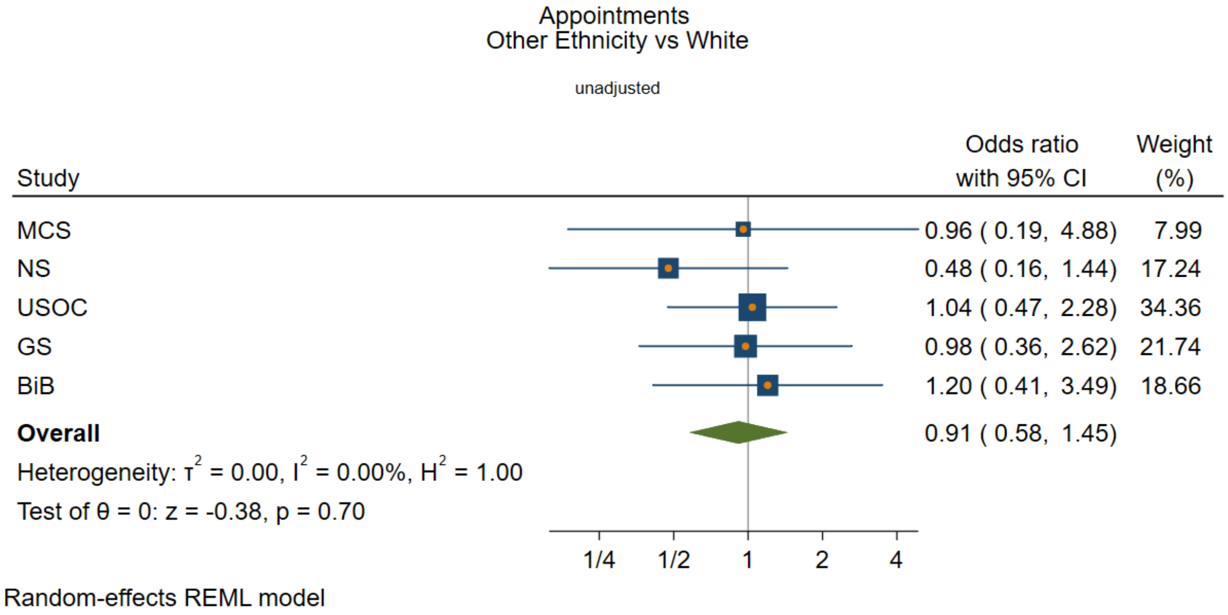

For peer review only - http://bmjopen.bmj.com/site/about/guidelines.xhtml

Basic adjustment

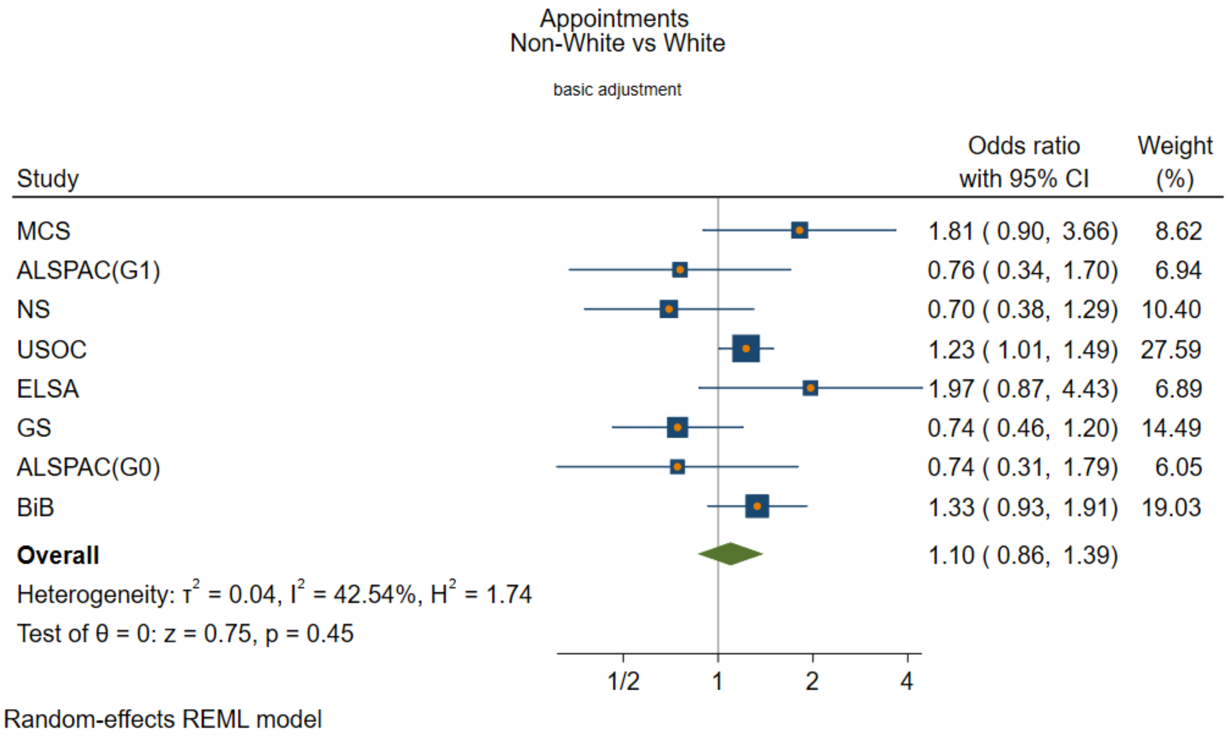

For peer review only - http://bmjopen.bmj.com/site/about/guidelines.xhtml

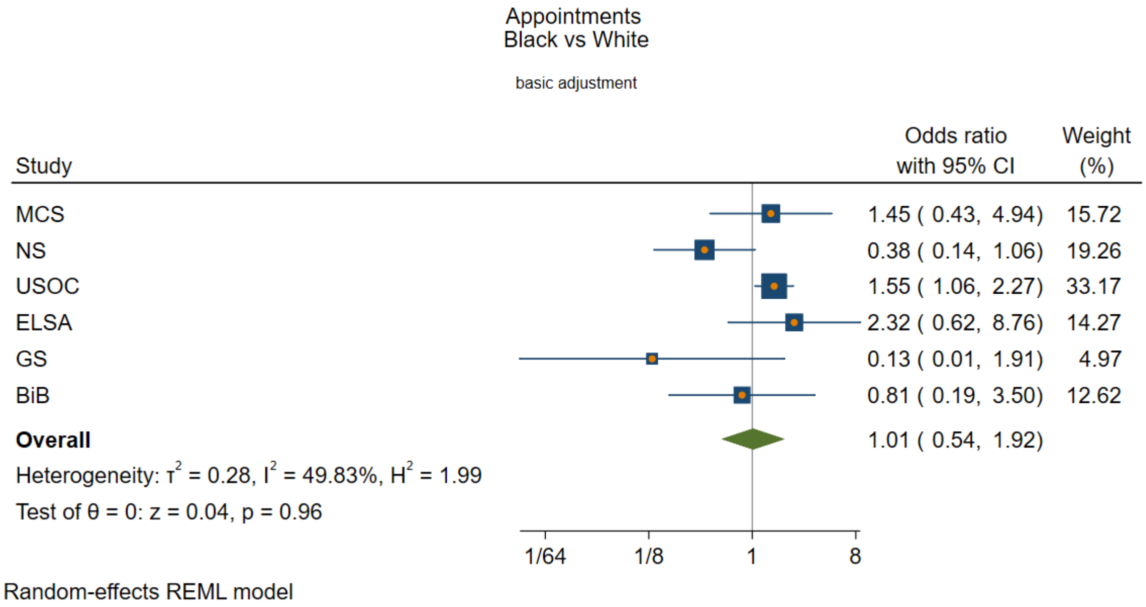

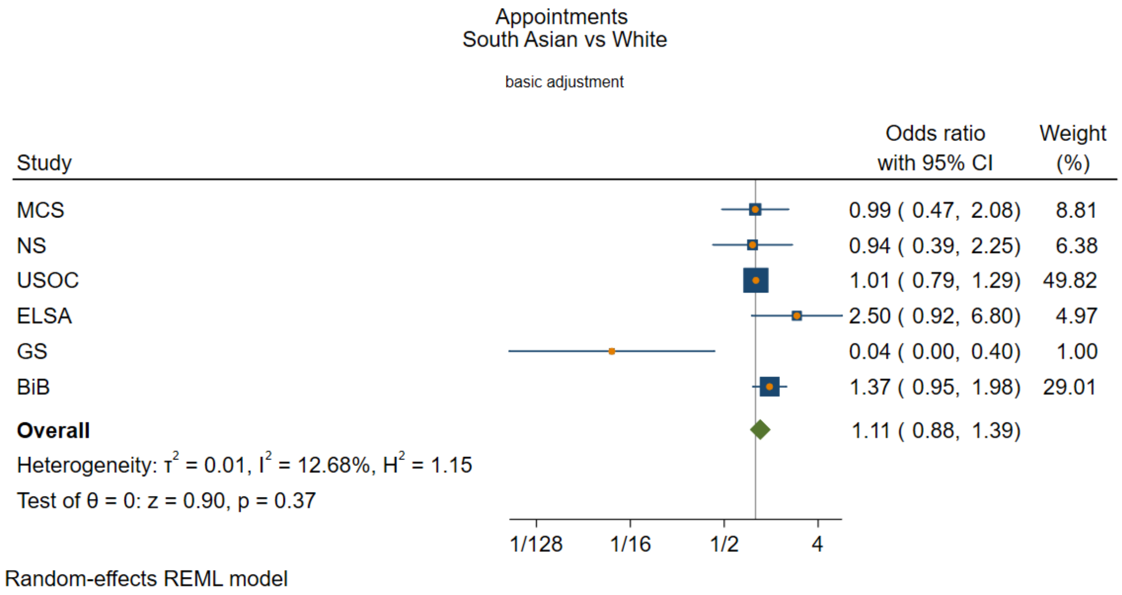

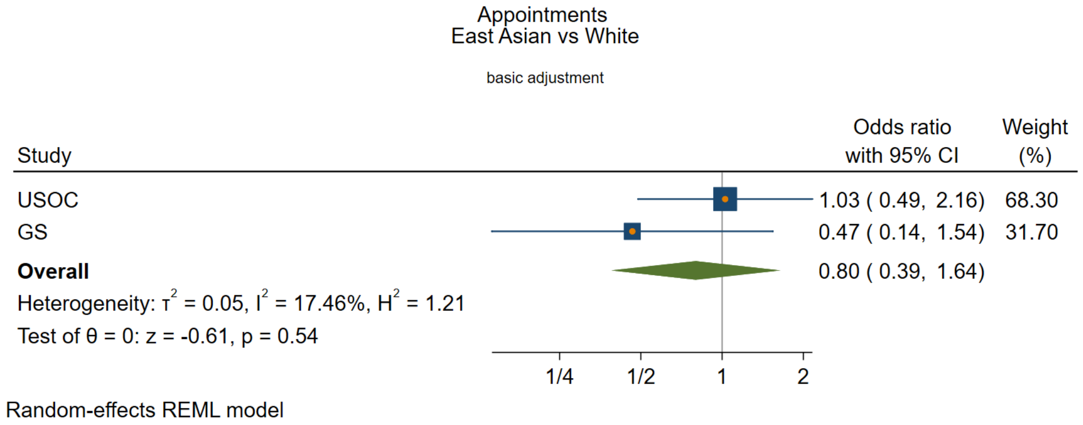

For peer review only - http://bmjopen.bmj.com/site/about/guidelines.xhtml

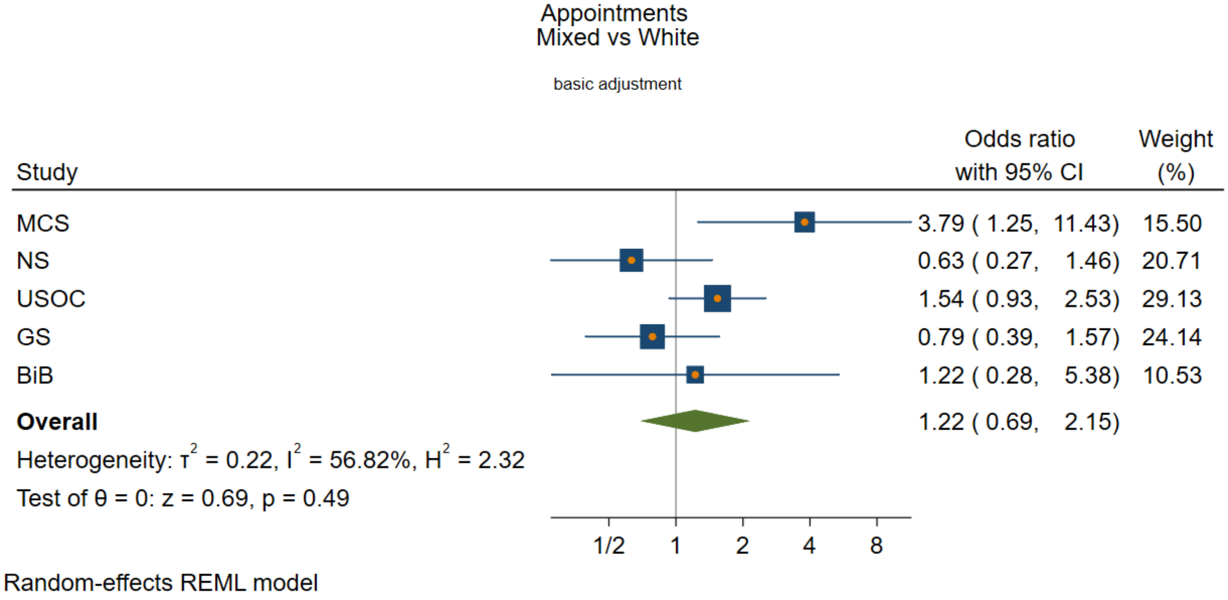

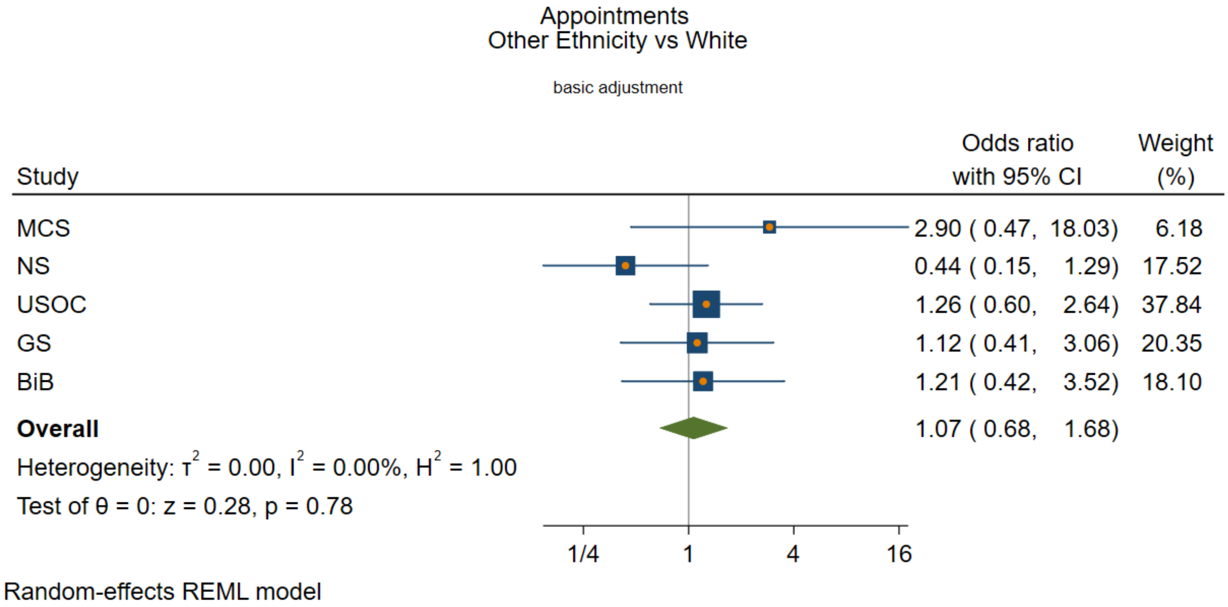

For peer review only - http://bmjopen.bmj.com/site/about/guidelines.xhtml

Full adjustment

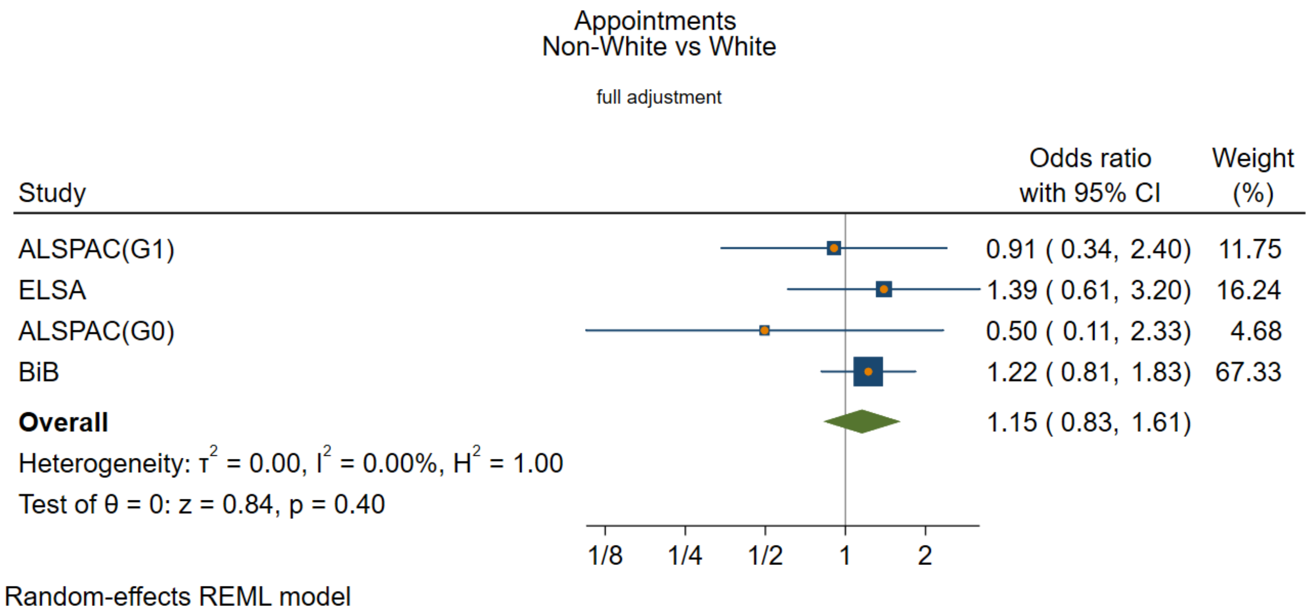

For peer review only - http://bmjopen.bmj.com/site/about/guidelines.xhtml

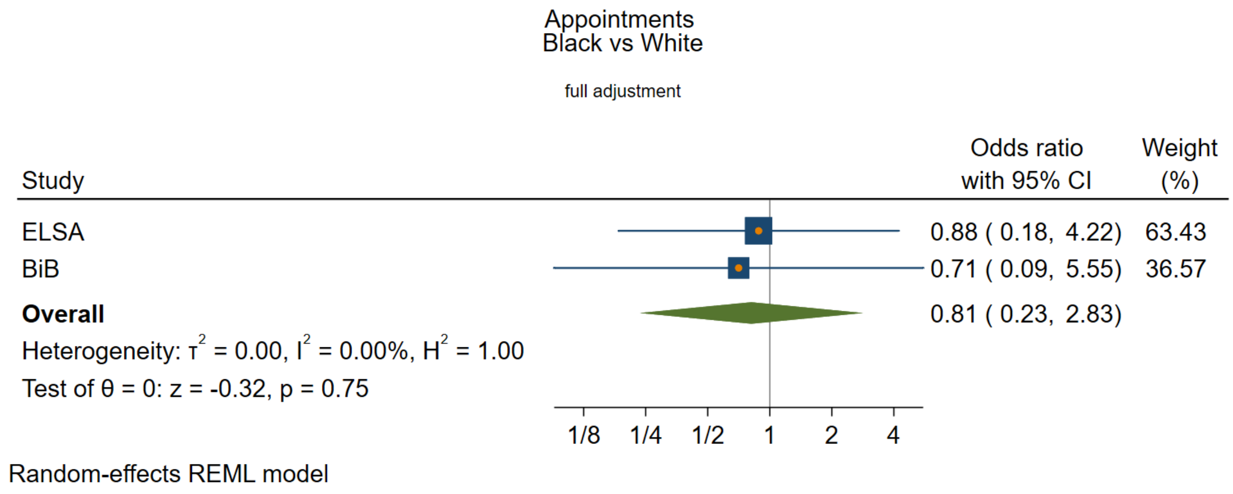

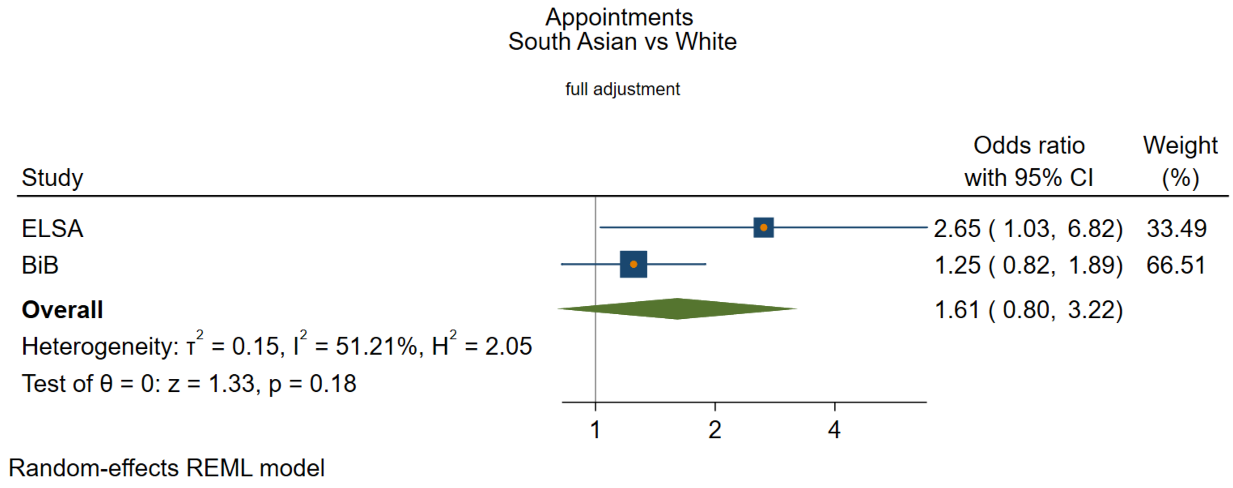

For peer review only - http://bmjopen.bmj.com/site/about/guidelines.xhtml

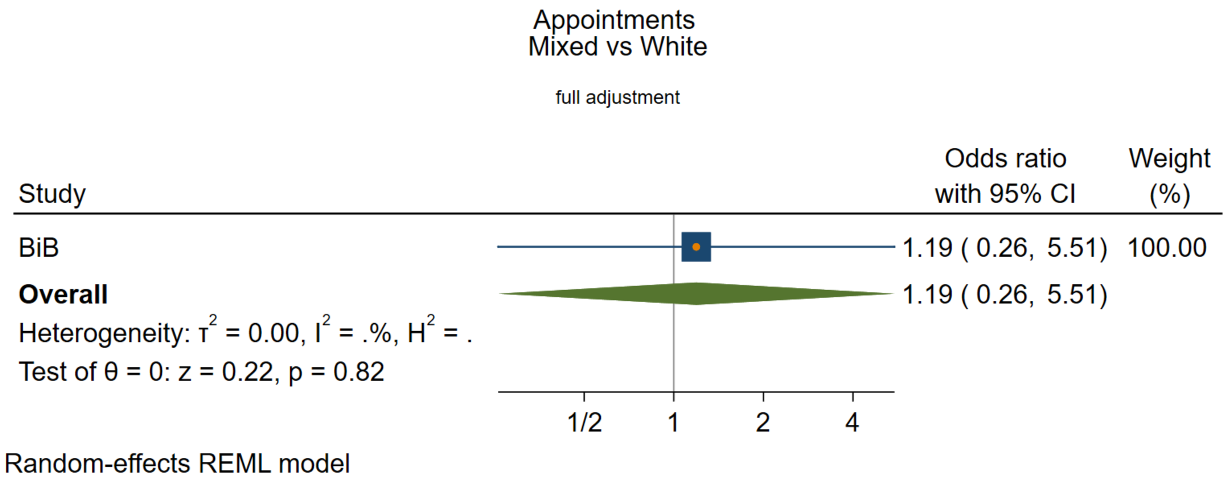

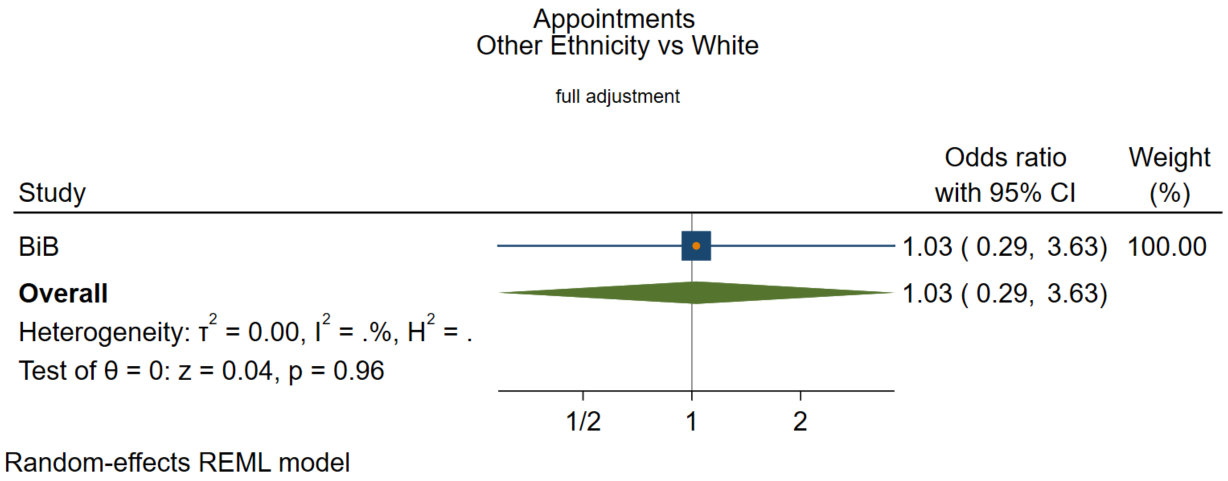

For peer review only - http://bmjopen.bmj.com/site/about/guidelines.xhtml

Prescription/Medication access

Sex

Unadjusted

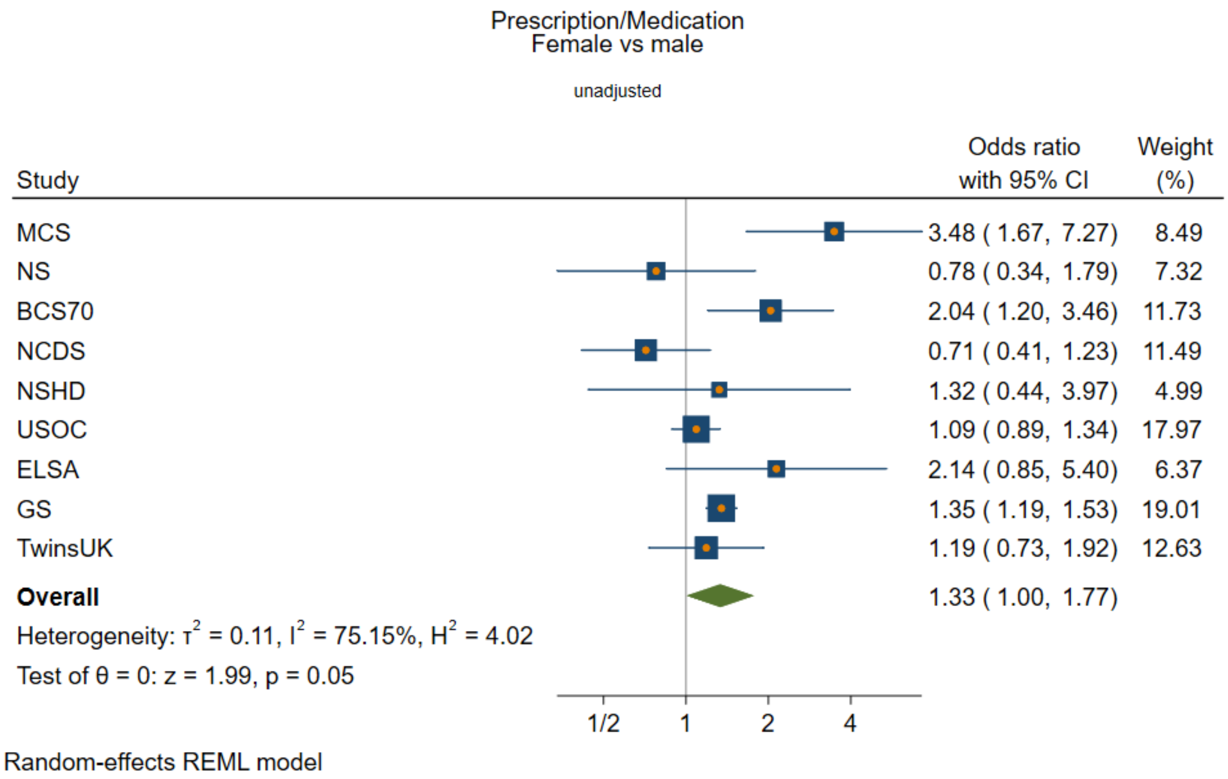

For peer review only - http://bmjopen.bmj.com/site/about/guidelines.xhtml

Basic adjustment

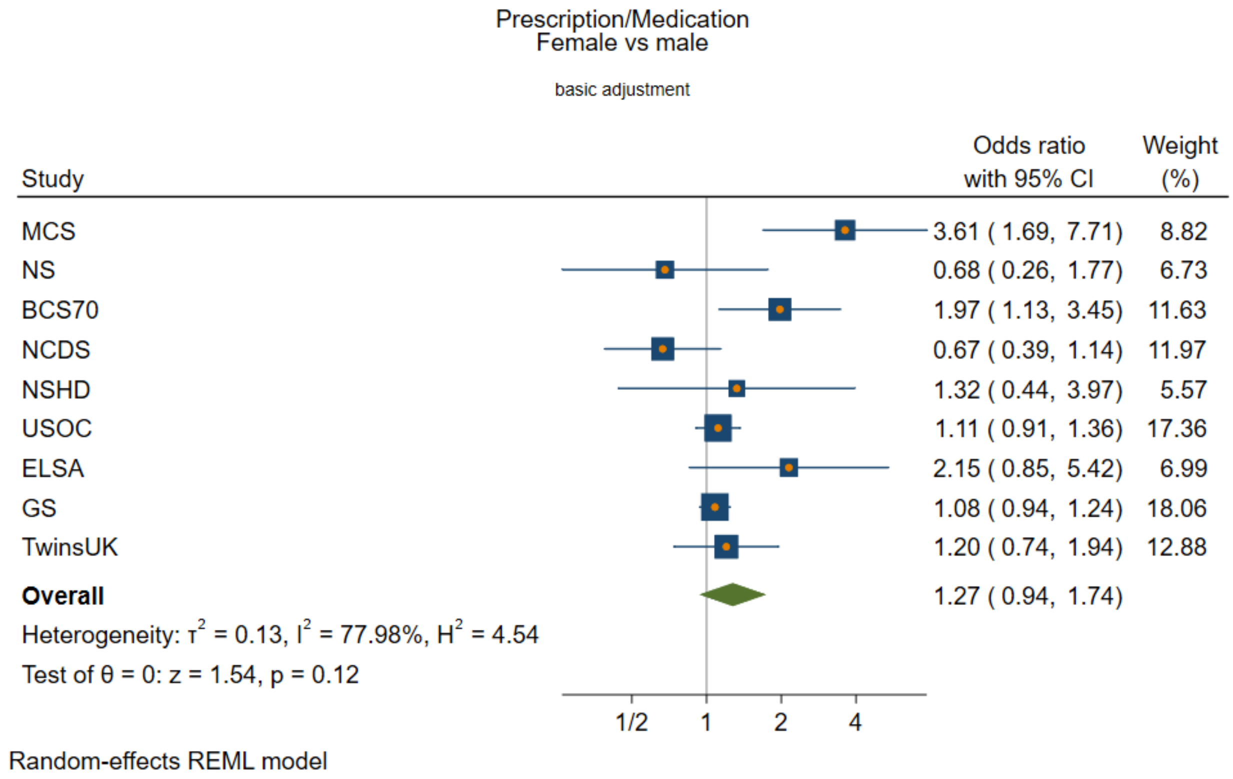

For peer review only - http://bmjopen.bmj.com/site/about/guidelines.xhtml

Full adjustment

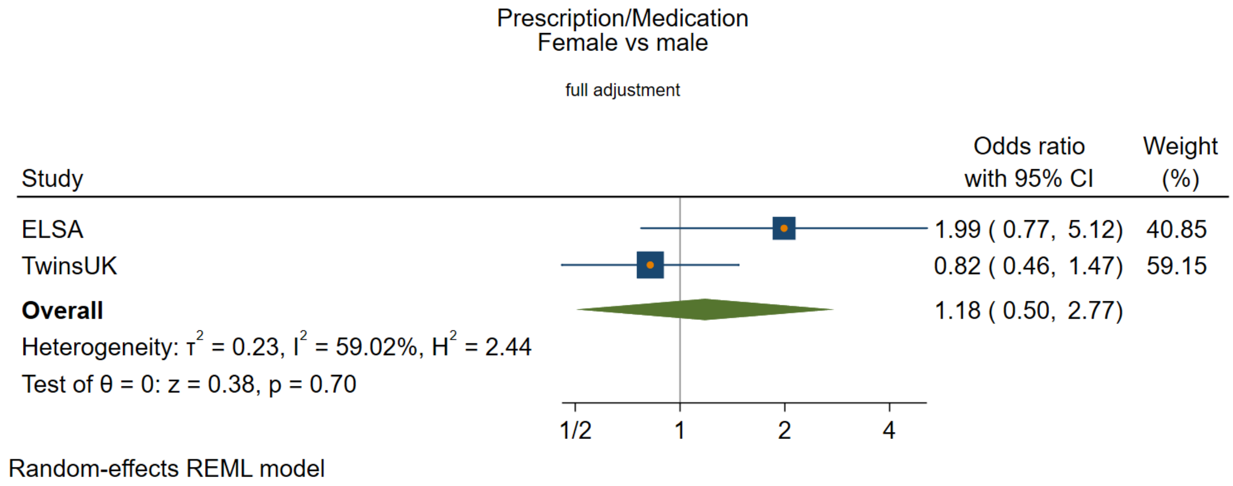

peer review only

For peer review only - http://bmjopen.bmj.com/site/about/guidelines.xhtml

Occupational class
Unadjusted

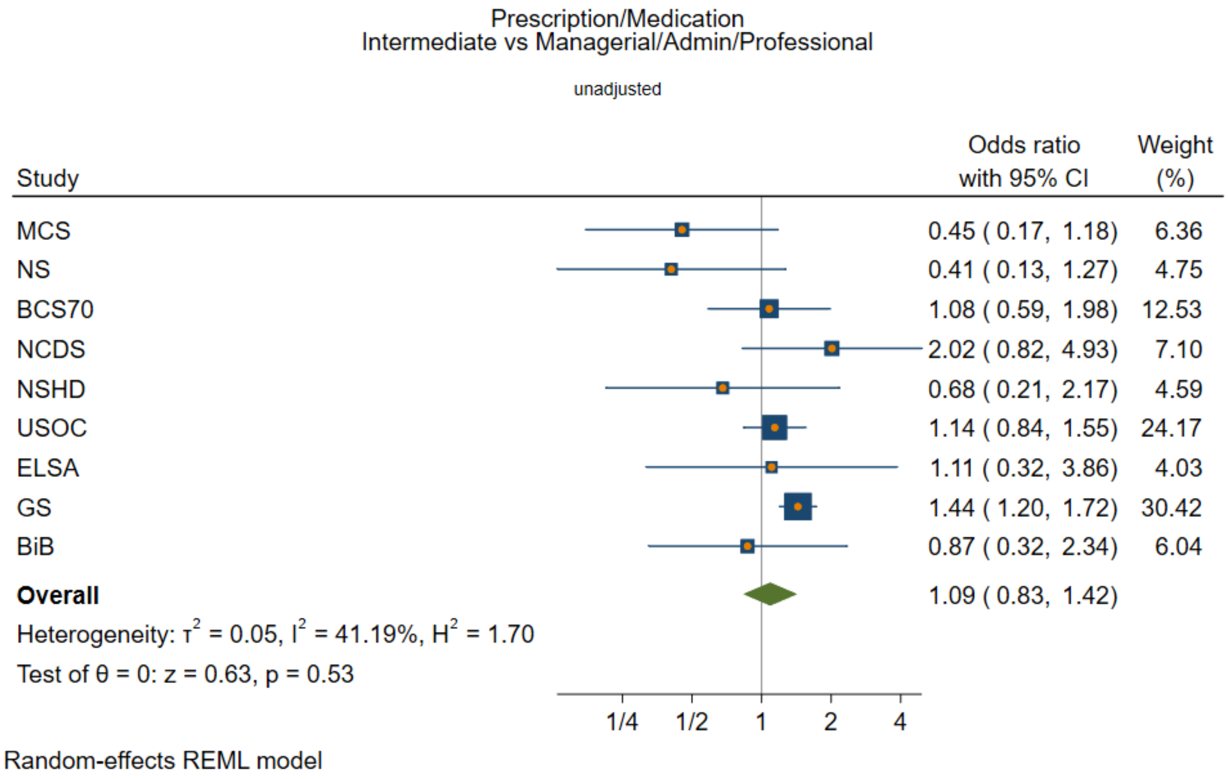

For peer review only - http://bmjopen.bmj.com/site/about/guidelines.xhtml

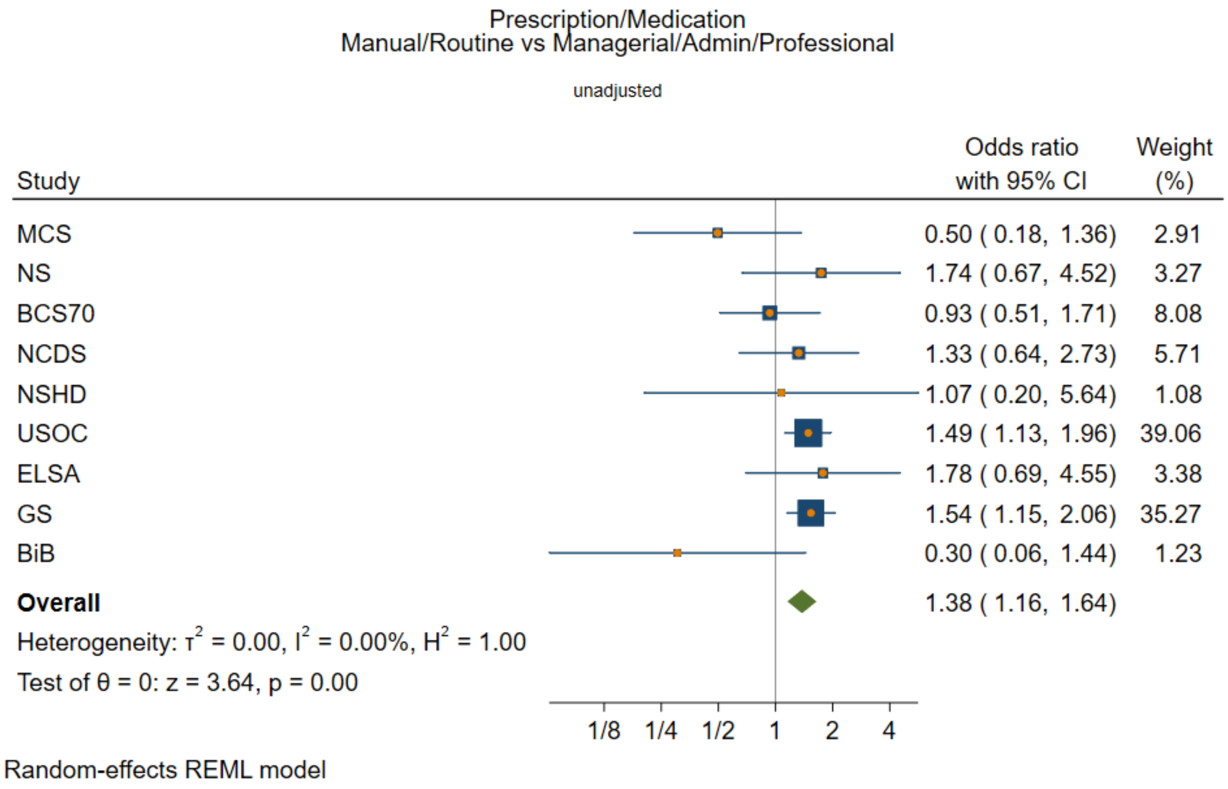

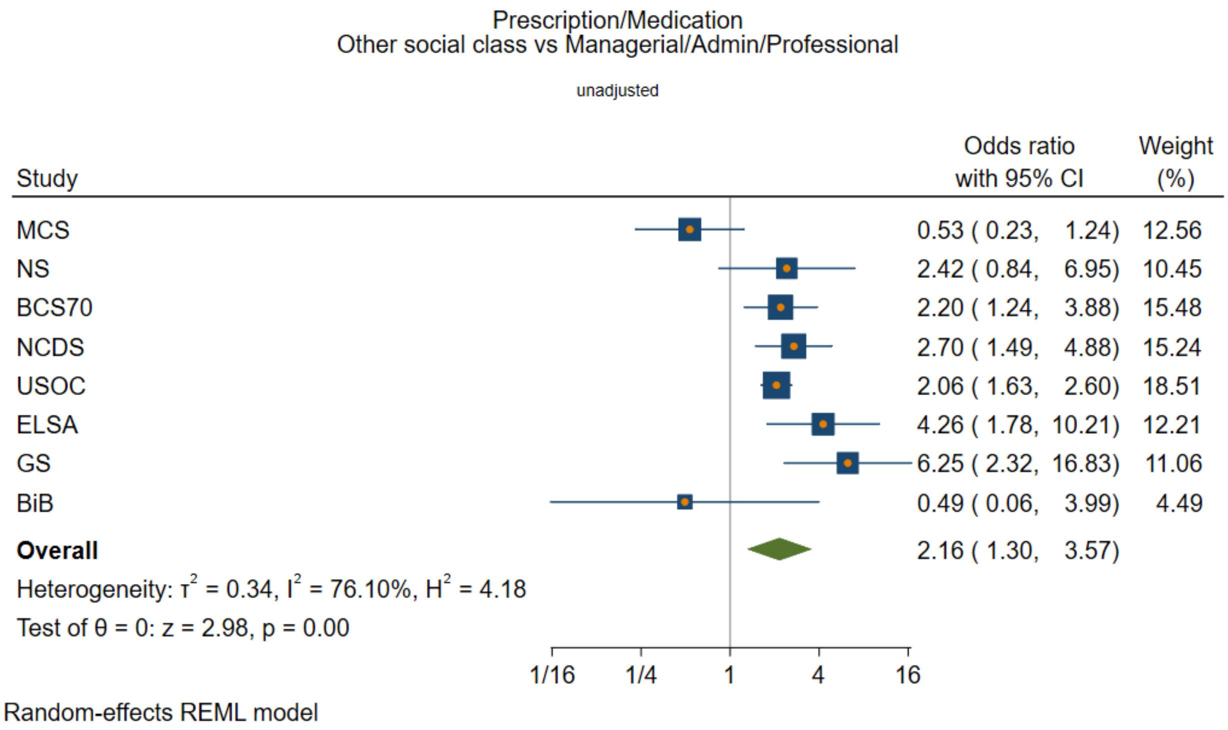

For peer review only - http://bmjopen.bmj.com/site/about/guidelines.xhtml

Basic adjustment

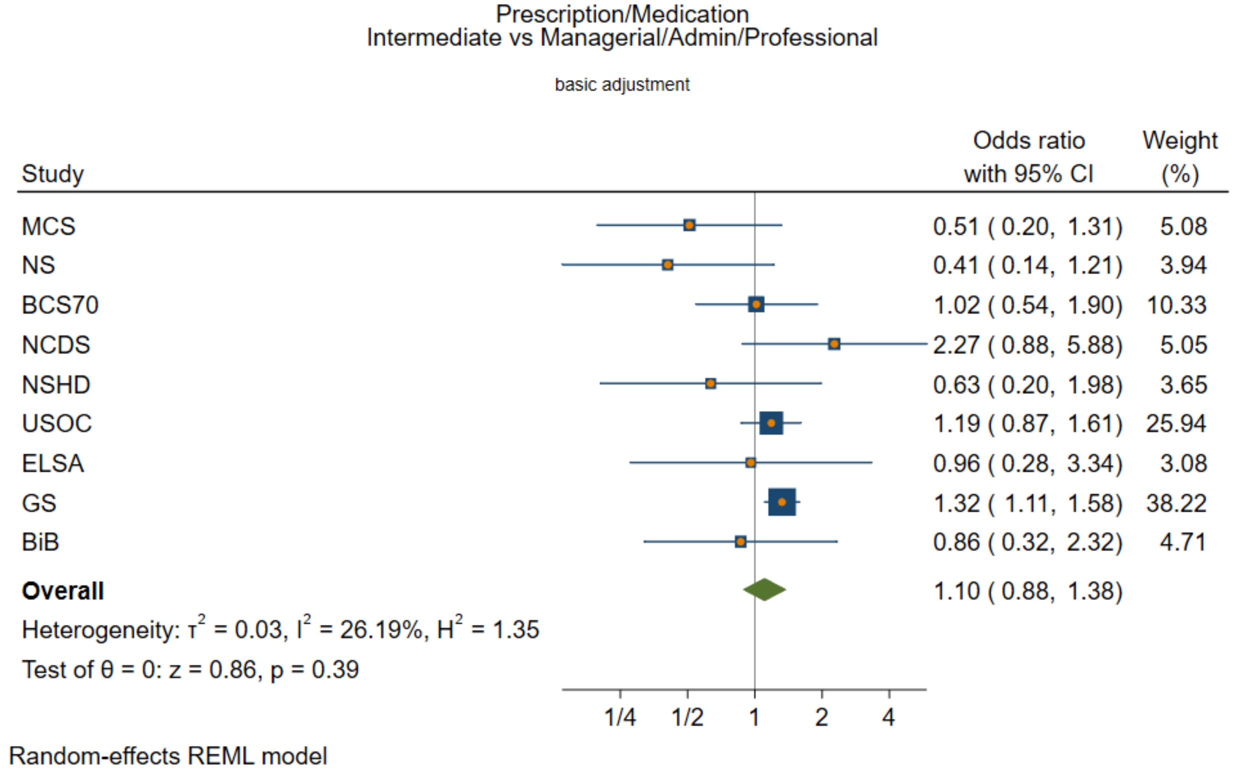

For peer review only - http://bmjopen.bmj.com/site/about/guidelines.xhtml

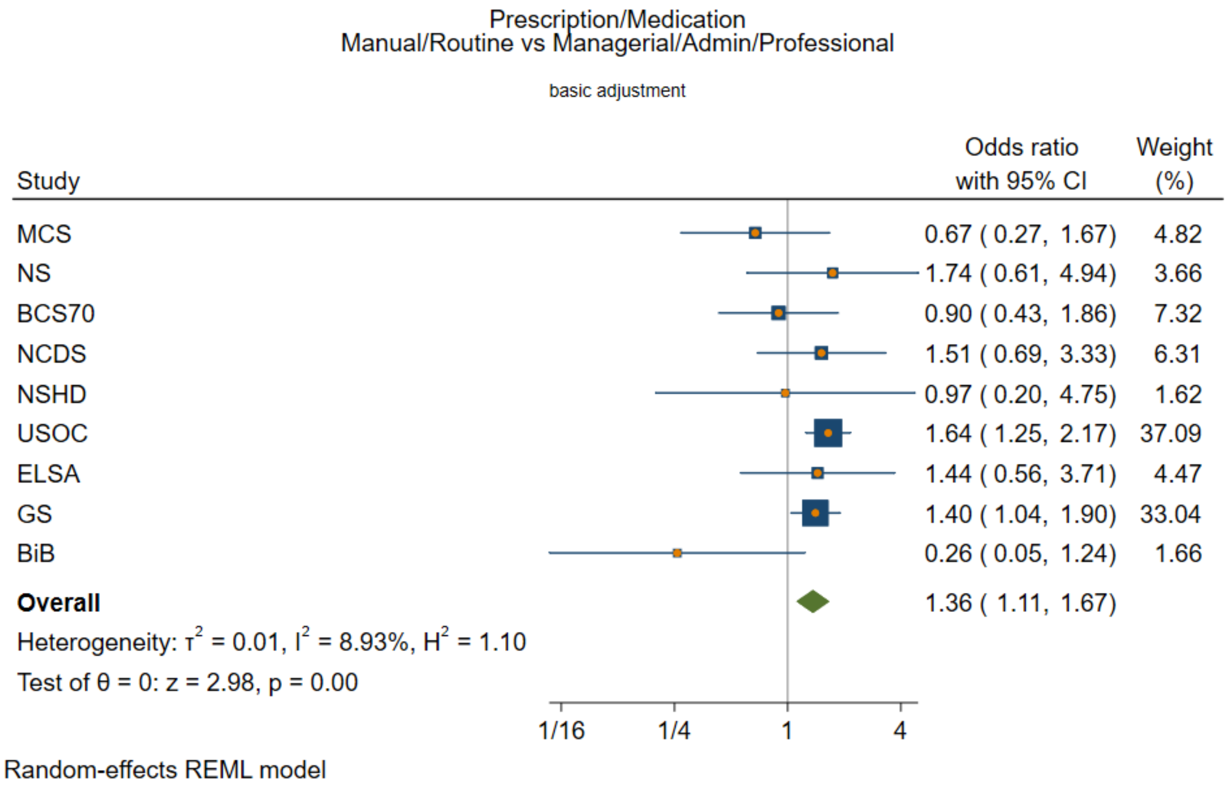
Prescription/Medication
Manual/Routine vs Managerial/Admin/Professional
basic adjustment
Study
Odds ratio with 95% CI
Weight (%)
MCS 0.67 (0.27, 1.67) 4.82
NS 1.74 (0.61, 4.94) 3.66
BCS70 0.90 (0.43, 1.86) 7.32
NCDS 1.51 (0.69, 3.33) 6.31
NSHD 0.97 (0.20, 4.75) 1.62
USOC 1.64 (1.25, 2.17) 37.09
ELSA 1.44 (0.56, 3.71) 4.47
GS 1.40 (1.04, 1.90) 33.04
BiB 0.26 (0.05, 1.24) 1.66
Overall 1.36 (1.11, 1.67)
Heterogeneity: τ^2 = 0.01, I^2 = 8.93%, H^2 = 1.10
Test of θ = 0: z = 2.98, p = 0.00
1/16
1/4
1
4
Random-effects REML model

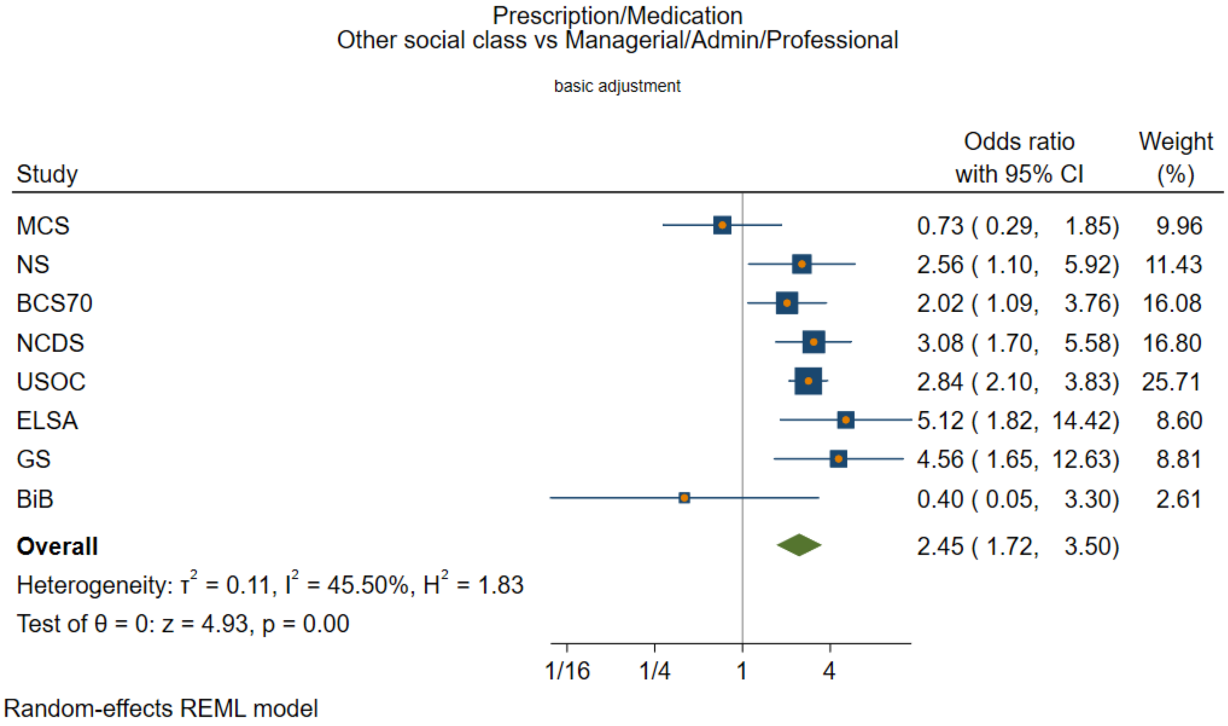
Prescription/Medication
Other social class vs Managerial/Admin/Professional
basic adjustment
Study
Odds ratio with 95% CI
Weight (%)
MCS 0.73 (0.29, 1.85) 9.96
NS 2.56 (1.10, 5.92) 11.43
BCS70 2.02 (1.09, 3.76) 16.08
NCDS 3.08 (1.70, 5.58) 16.80
USOC 2.84 (2.10, 3.83) 25.71
ELSA 5.12 (1.82, 14.42) 8.60
GS 4.56 (1.65, 12.63) 8.81
BiB 0.40 (0.05, 3.30) 2.61
Overall 2.45 (1.72, 3.50)
Heterogeneity: τ^2 = 0.11, I^2 = 45.50%, H^2 = 1.83
Test of θ = 0: z = 4.93, p = 0.00
1/16
1/4
1
4
Random-effects REML model

Full adjustment

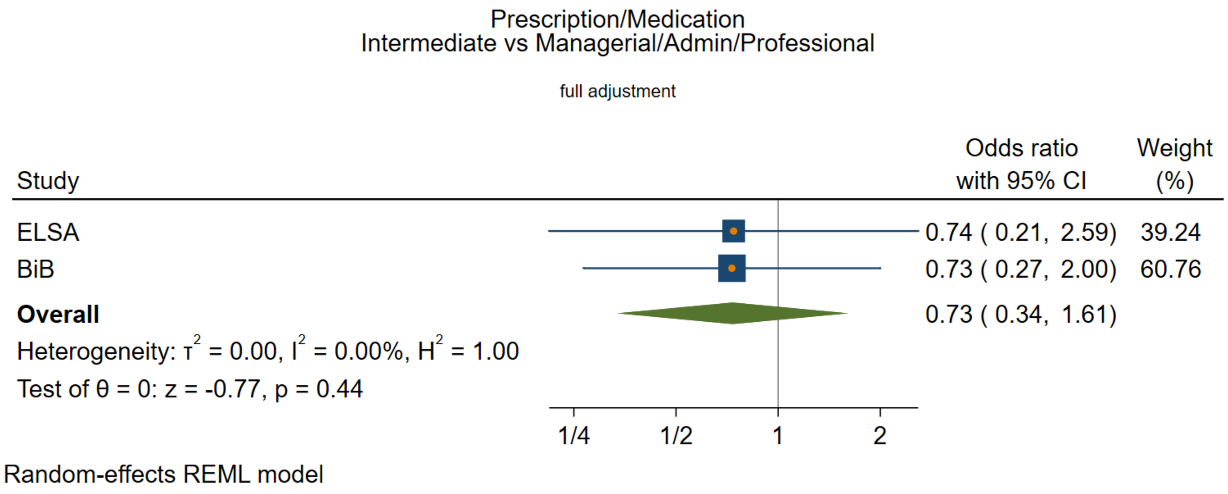

For peer review only

For peer review only - http://bmjopen.bmj.com/site/about/guidelines.xhtml

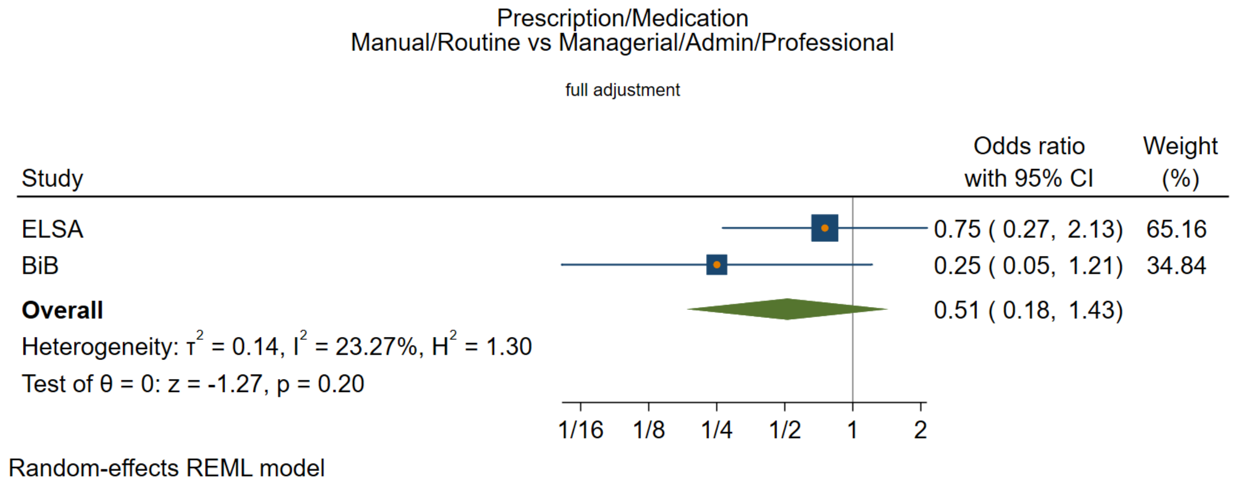

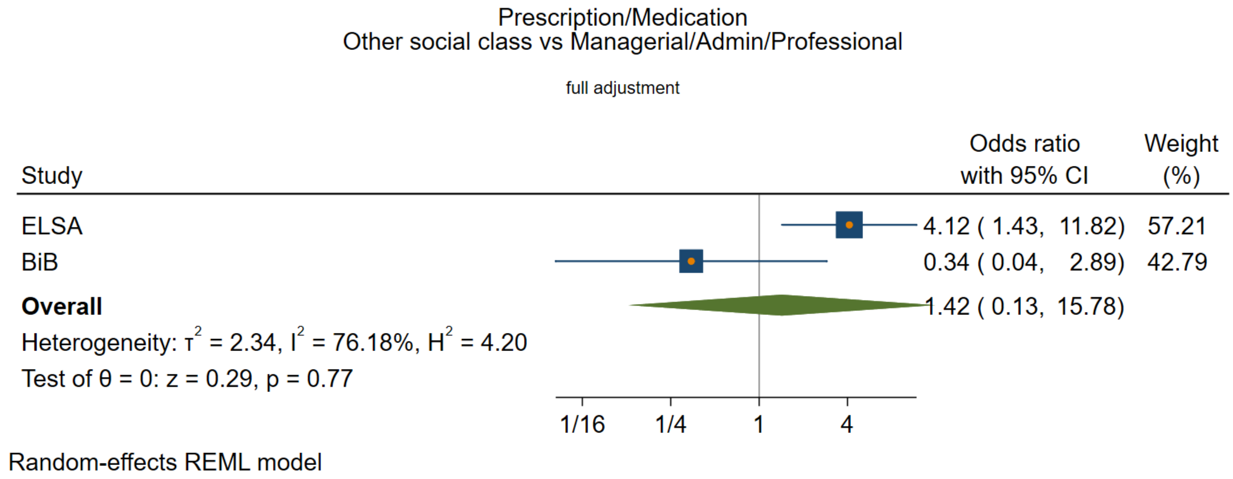

For peer review only - http://bmjopen.bmj.com/site/about/guidelines.xhtml

Education

Unadjusted

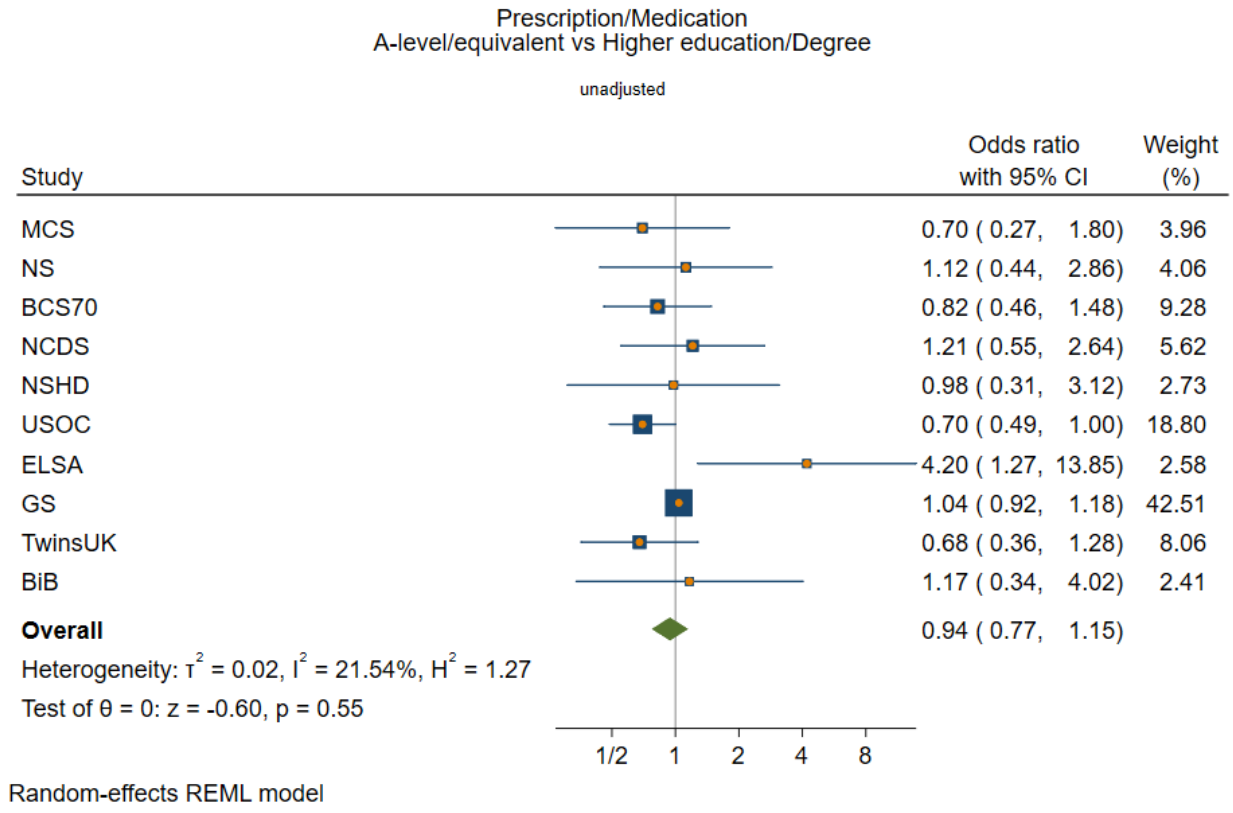

For peer review only - http://bmjopen.bmj.com/site/about/guidelines.xhtml

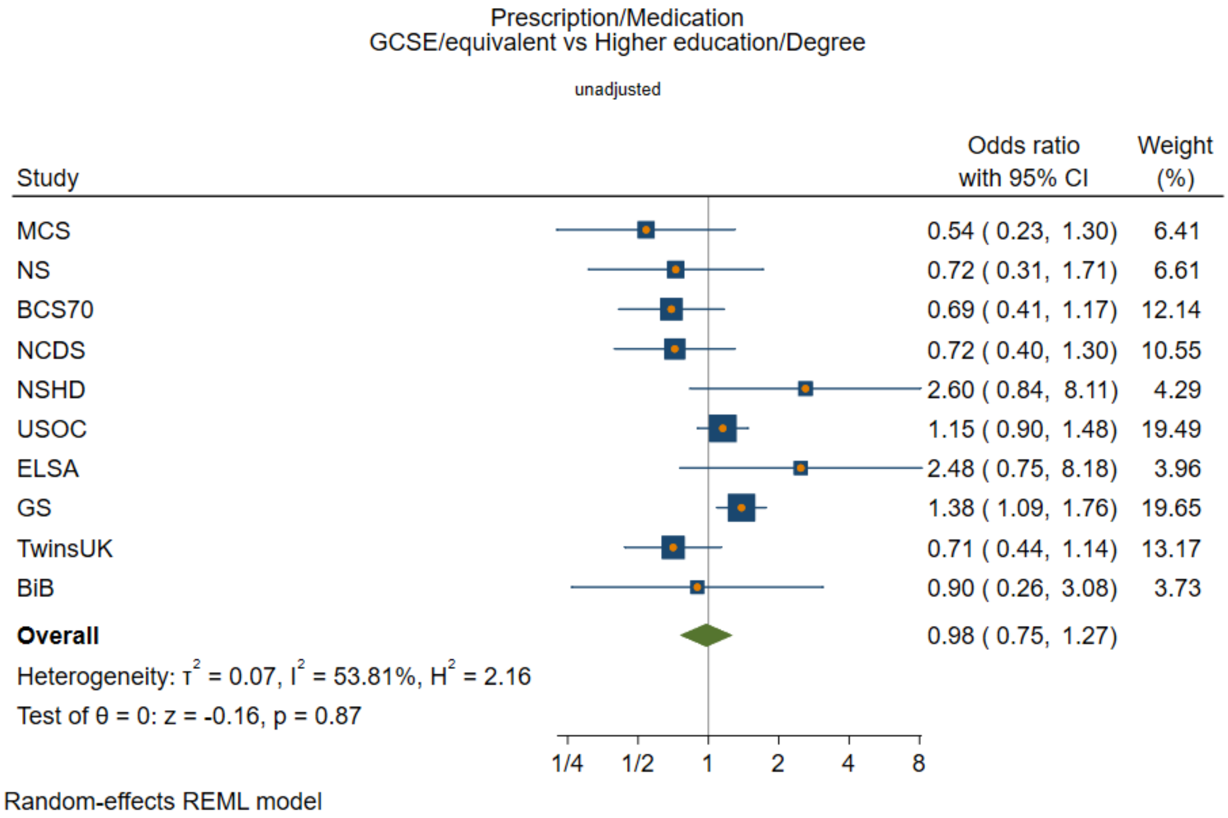
Prescription/Medication
GCSE/equivalent vs Higher education/Degree
unadjusted
Study
Odds ratio with 95% CI
Weight (%)
MCS 0.54 (0.23, 1.30) 6.41
NS 0.72 (0.31, 1.71) 6.61
BCS70 0.69 (0.41, 1.17) 12.14
NCDS 0.72 (0.40, 1.30) 10.55
NSHD 2.60 (0.84, 8.11) 4.29
USOC 1.15 (0.90, 1.48) 19.49
ELSA 2.48 (0.75, 8.18) 3.96
GS 1.38 (1.09, 1.76) 19.65
TwinsUK 0.71 (0.44, 1.14) 13.17
BiB 0.90 (0.26, 3.08) 3.73
Overall 0.98 (0.75, 1.27)
Heterogeneity: τ² = 0.07, I² = 53.81%, H² = 2.16
Test of θ = 0: z = -0.16, p = 0.87
1/4 1/2 1 2 4 8
Random-effects REML model

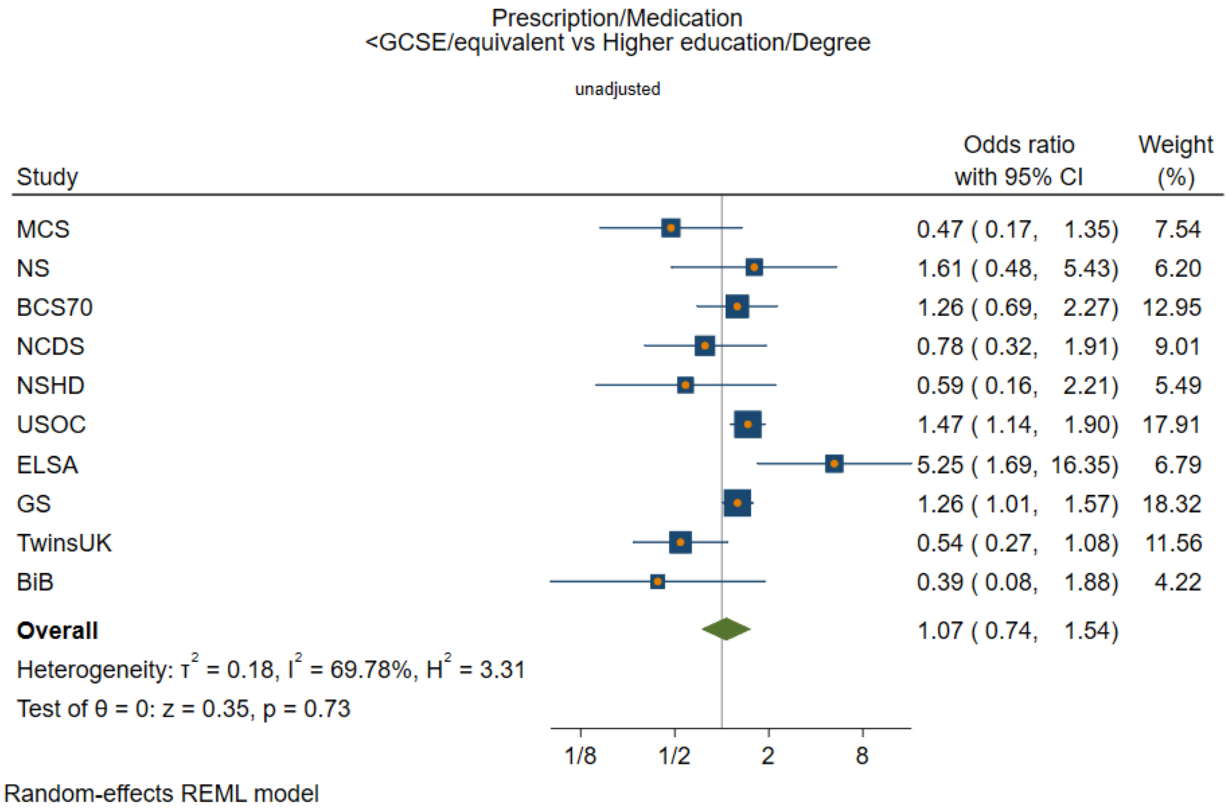
Prescription/Medication
<GCSE/equivalent vs Higher education/Degree
unadjusted
Study
Odds ratio with 95% CI
Weight (%)
MCS 0.47 (0.17, 1.35) 7.54
NS 1.61 (0.48, 5.43) 6.20
BCS70 1.26 (0.69, 2.27) 12.95
NCDS 0.78 (0.32, 1.91) 9.01
NSHD 0.59 (0.16, 2.21) 5.49
USOC 1.47 (1.14, 1.90) 17.91
ELSA 5.25 (1.69, 16.35) 6.79
GS 1.26 (1.01, 1.57) 18.32
TwinsUK 0.54 (0.27, 1.08) 11.56
BiB 0.39 (0.08, 1.88) 4.22
Overall 1.07 (0.74, 1.54)
Heterogeneity: τ² = 0.18, I² = 69.78%, H² = 3.31
Test of θ = 0: z = 0.35, p = 0.73
1/8 1/2 2 8
Random-effects REML model

Basic adjustment

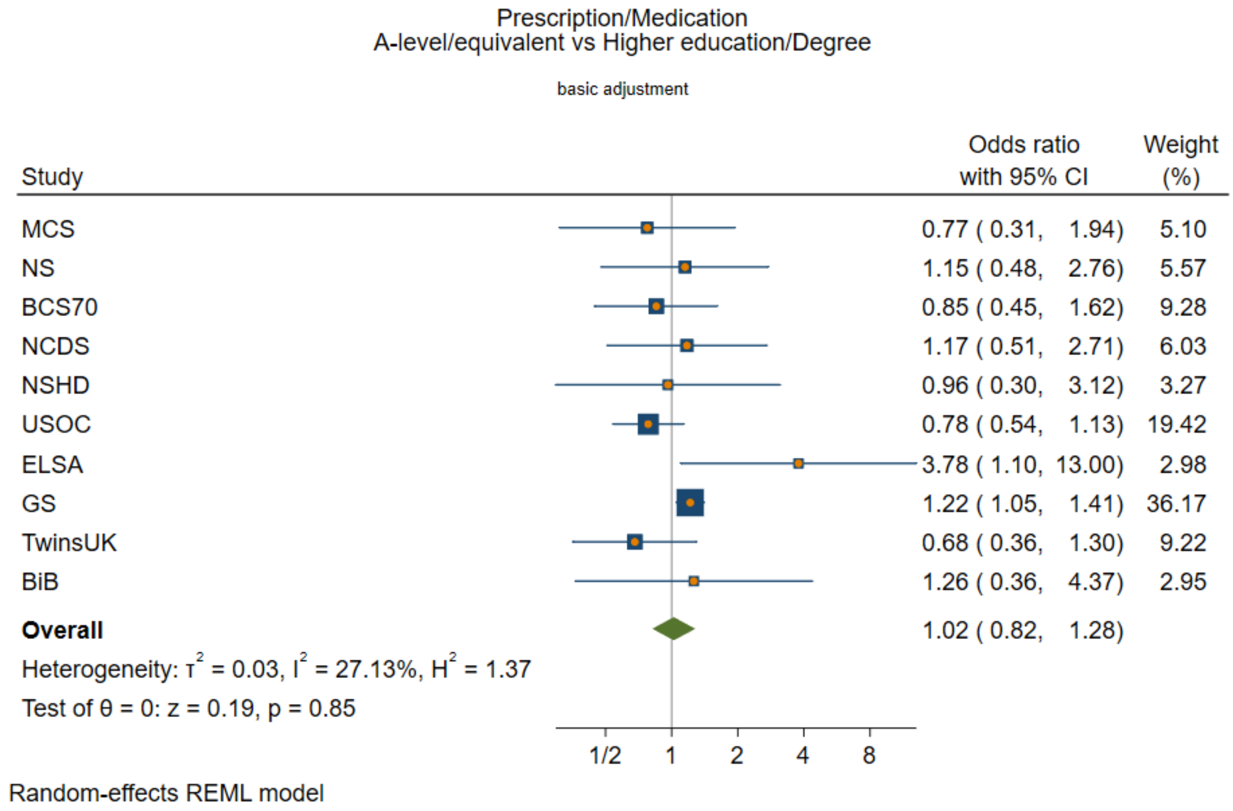

For peer review only - http://bmjopen.bmj.com/site/about/guidelines.xhtml

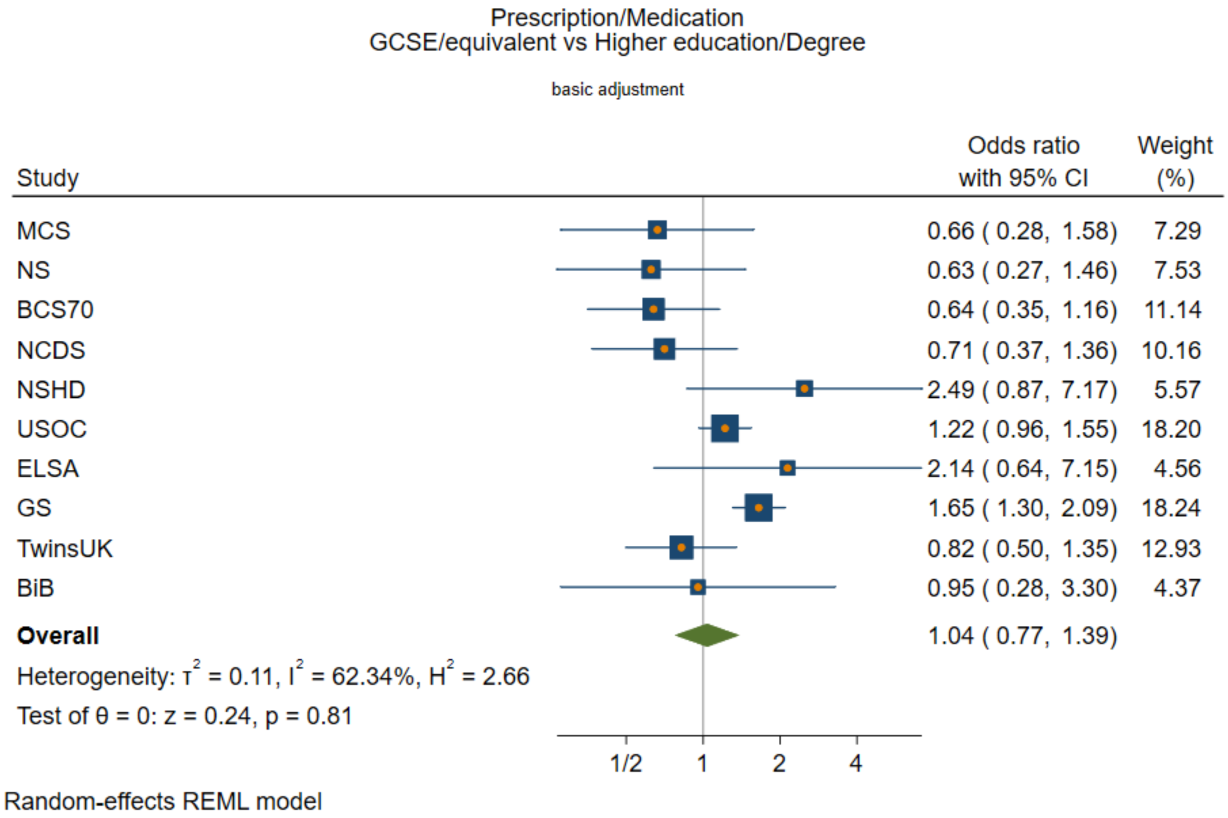
Prescription/Medication
GCSE/equivalent vs Higher education/Degree
basic adjustment
Study
Odds ratio with 95% CI
Weight (%)
MCS 0.66 (0.28, 1.58) 7.29
NS 0.63 (0.27, 1.46) 7.53
BCS70 0.64 (0.35, 1.16) 11.14
NCDS 0.71 (0.37, 1.36) 10.16
NSHD 2.49 (0.87, 7.17) 5.57
USOC 1.22 (0.96, 1.55) 18.20
ELSA 2.14 (0.64, 7.15) 4.56
GS 1.65 (1.30, 2.09) 18.24
TwinsUK 0.82 (0.50, 1.35) 12.93
BiB 0.95 (0.28, 3.30) 4.37
Overall 1.04 (0.77, 1.39)
Heterogeneity: τ² = 0.11, I² = 62.34%, H² = 2.66
Test of θ = 0: z = 0.24, p = 0.81
1/2
1
2
4
Random-effects REML model

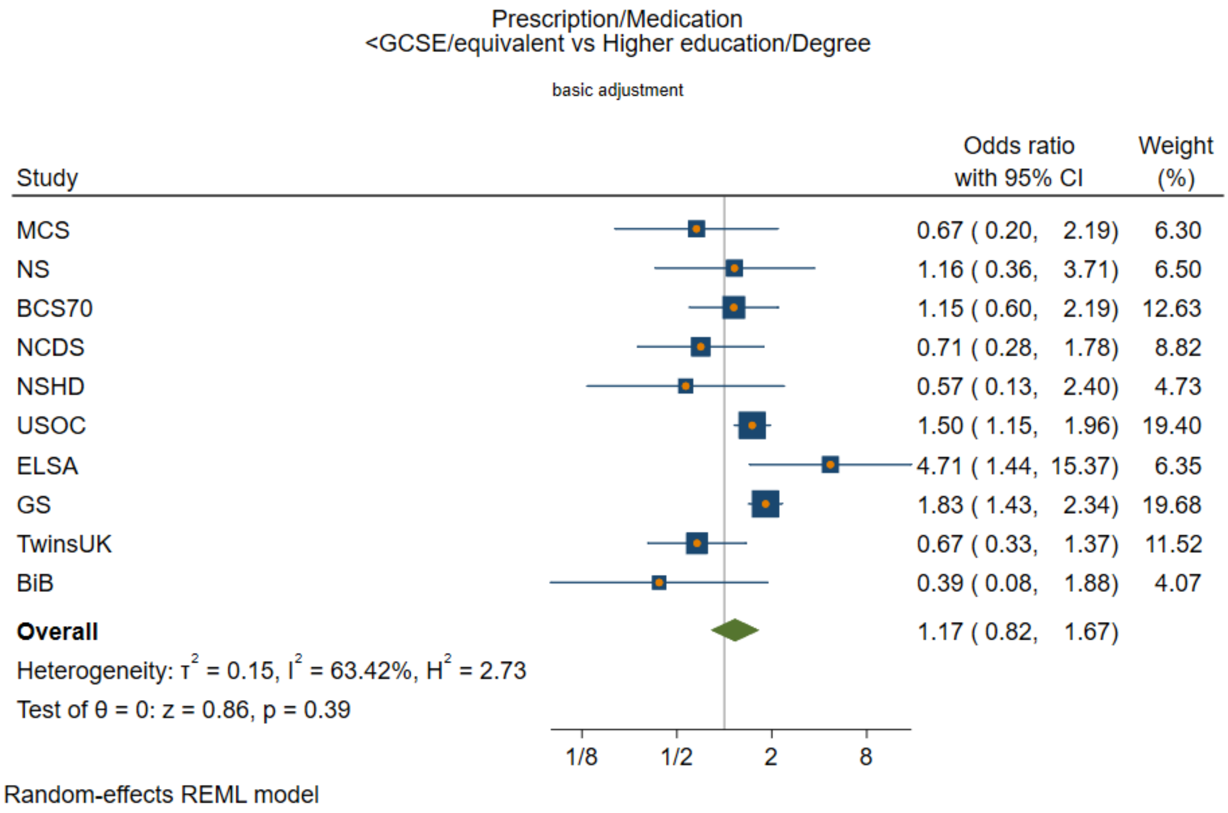
Prescription/Medication
<GCSE/equivalent vs Higher education/Degree
basic adjustment
Study
Odds ratio with 95% CI
Weight (%)
MCS 0.67 (0.20, 2.19) 6.30
NS 1.16 (0.36, 3.71) 6.50
BCS70 1.15 (0.60, 2.19) 12.63
NCDS 0.71 (0.28, 1.78) 8.82
NSHD 0.57 (0.13, 2.40) 4.73
USOC 1.50 (1.15, 1.96) 19.40
ELSA 4.71 (1.44, 15.37) 6.35
GS 1.83 (1.43, 2.34) 19.68
TwinsUK 0.67 (0.33, 1.37) 11.52
BiB 0.39 (0.08, 1.88) 4.07
Overall 1.17 (0.82, 1.67)
Heterogeneity: τ² = 0.15, I² = 63.42%, H² = 2.73
Test of θ = 0: z = 0.86, p = 0.39
1/8
1/2
2
8
Random-effects REML model

Full adjustment

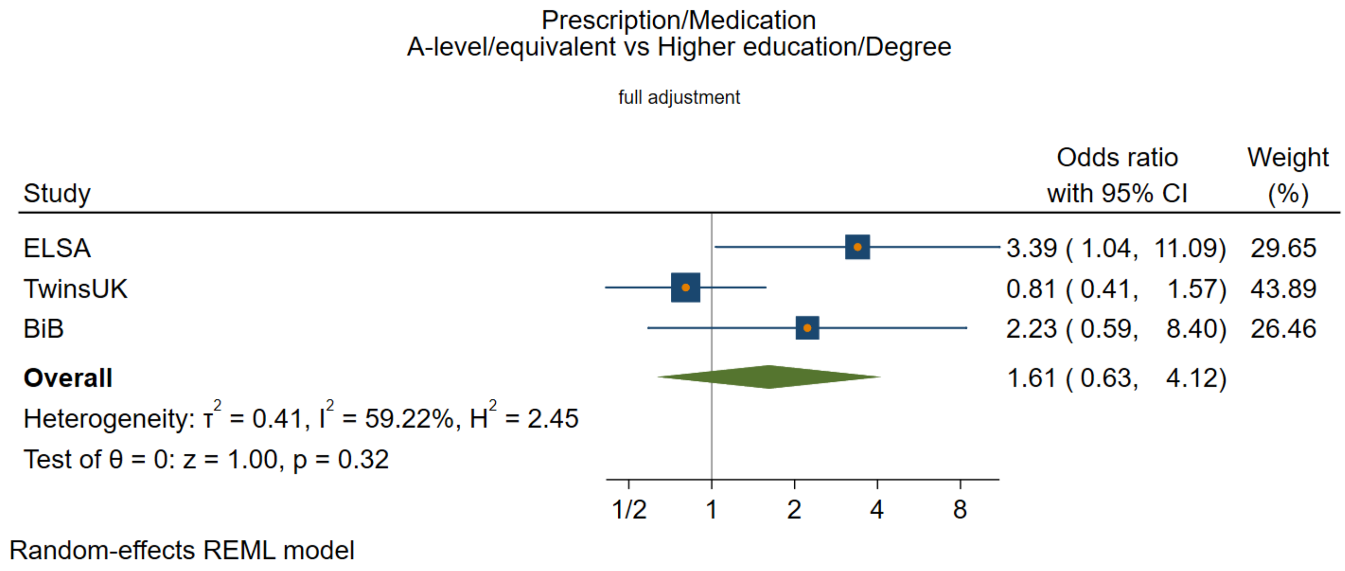

review only

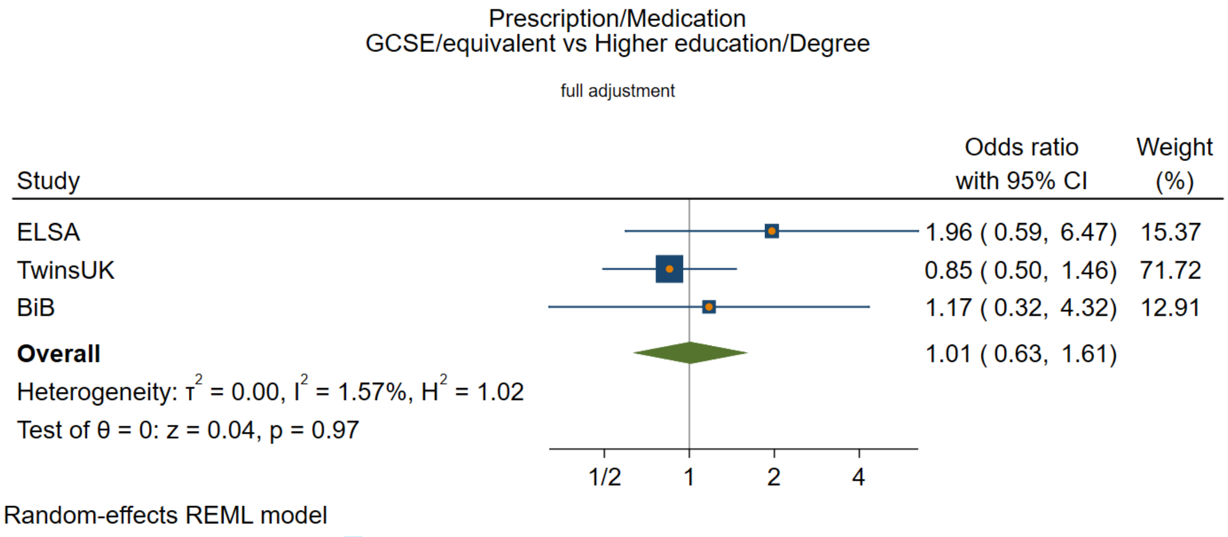
Prescription/Medication
GCSE/equivalent vs Higher education/Degree
full adjustment
Study
Odds ratio with 95% CI
Weight (%)
ELSA 1.96 (0.59, 6.47) 15.37
TwinsUK 0.85 (0.50, 1.46) 71.72
BiB 1.17 (0.32, 4.32) 12.91
Overall 1.01 (0.63, 1.61)
Heterogeneity: τ² = 0.00, I² = 1.57%, H² = 1.02
Test of θ = 0: z = 0.04, p = 0.97
1/2
1
2
4
Random-effects REML model

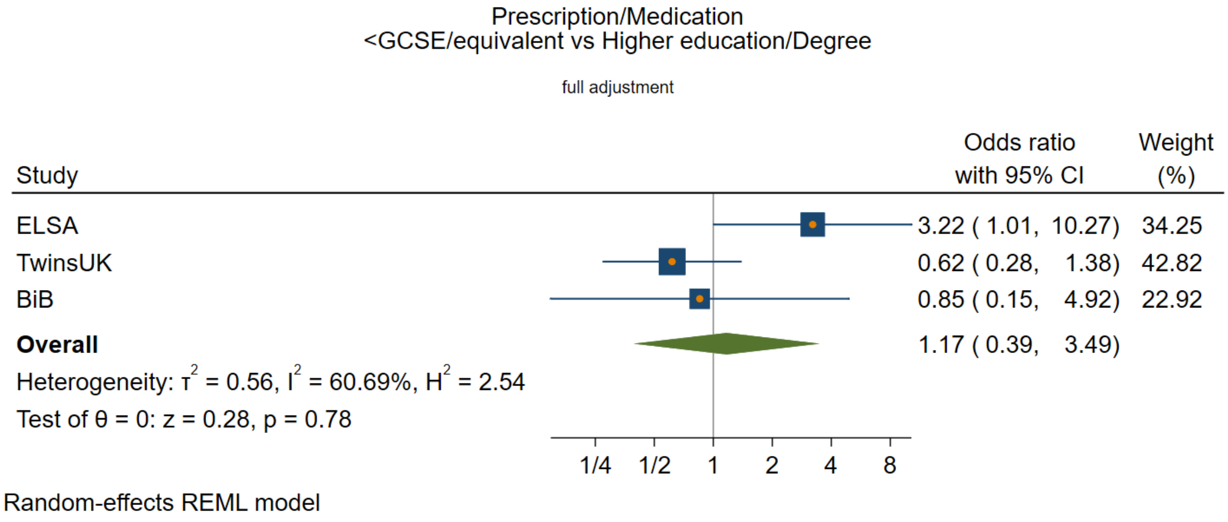
Prescription/Medication
<GCSE/equivalent vs Higher education/Degree
full adjustment
Study
Odds ratio with 95% CI
Weight (%)
ELSA 3.22 (1.01, 10.27) 34.25
TwinsUK 0.62 (0.28, 1.38) 42.82
BiB 0.85 (0.15, 4.92) 22.92
Overall 1.17 (0.39, 3.49)
Heterogeneity: τ² = 0.56, I² = 60.69%, H² = 2.54
Test of θ = 0: z = 0.28, p = 0.78
1/4
1/2
1
2
4
8
Random-effects REML model

Age

Unadjusted

For peer review only

For peer review only - http://bmjopen.bmj.com/site/about/guidelines.xhtml

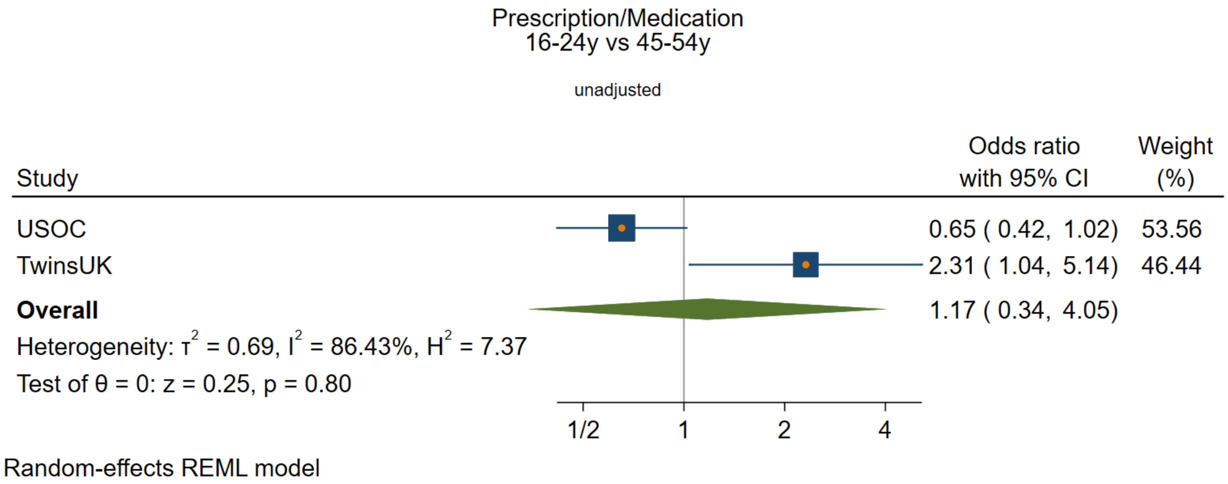

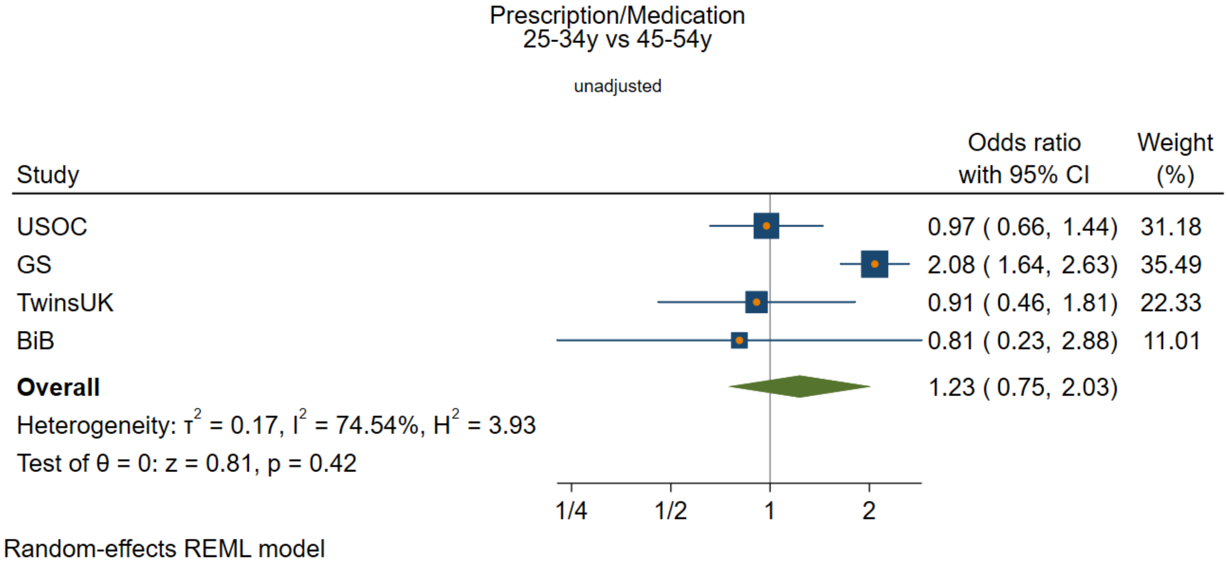

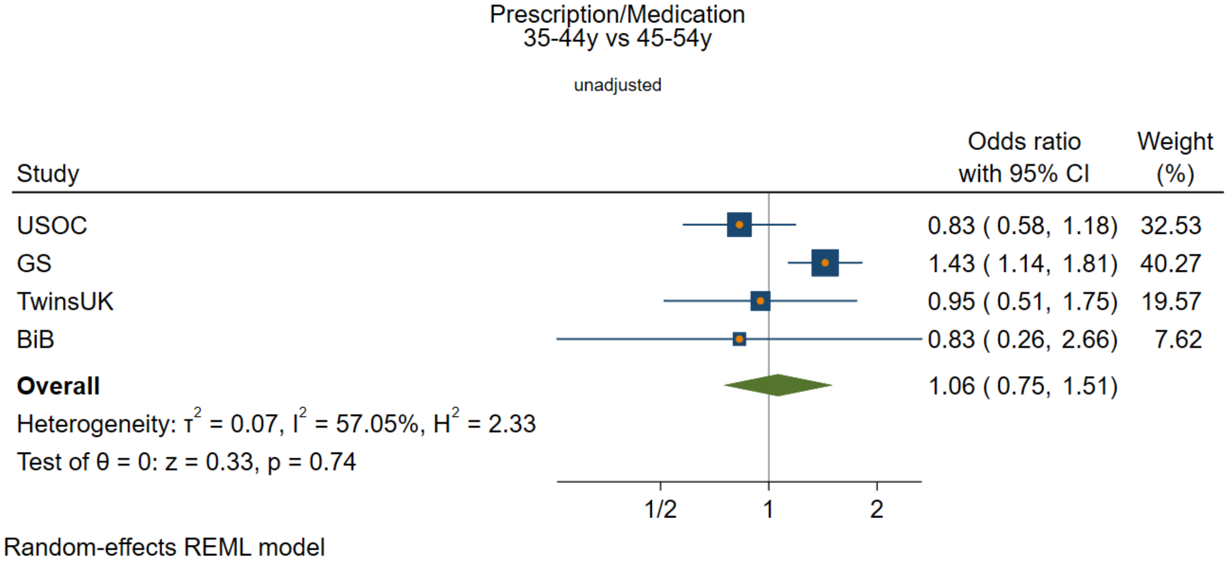

For peer review only - http://bmjopen.bmj.com/site/about/guidelines.xhtml

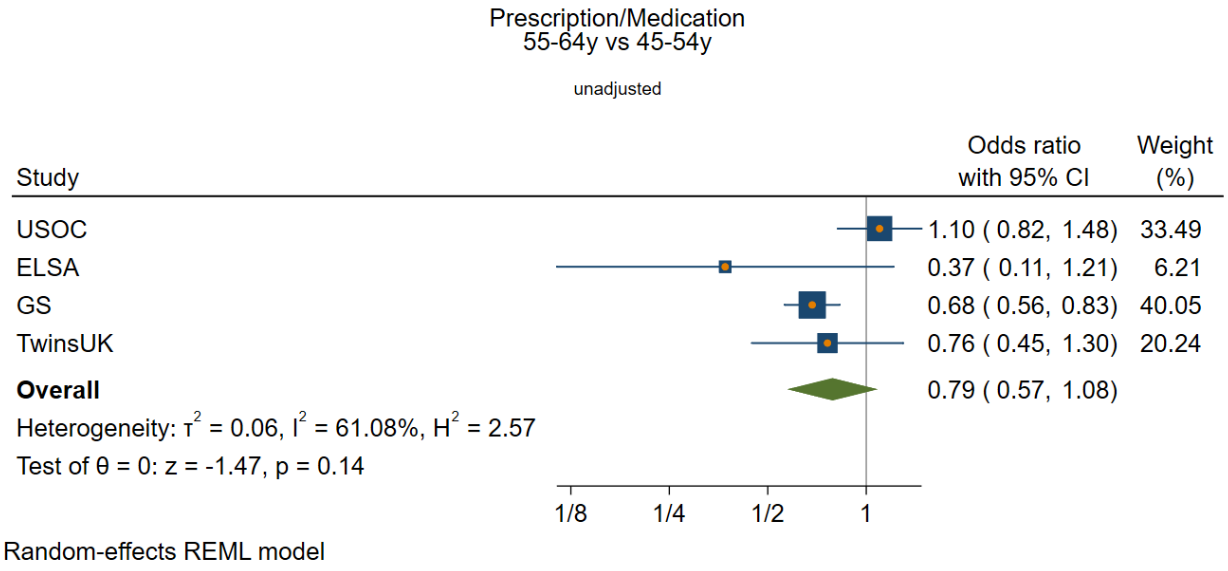

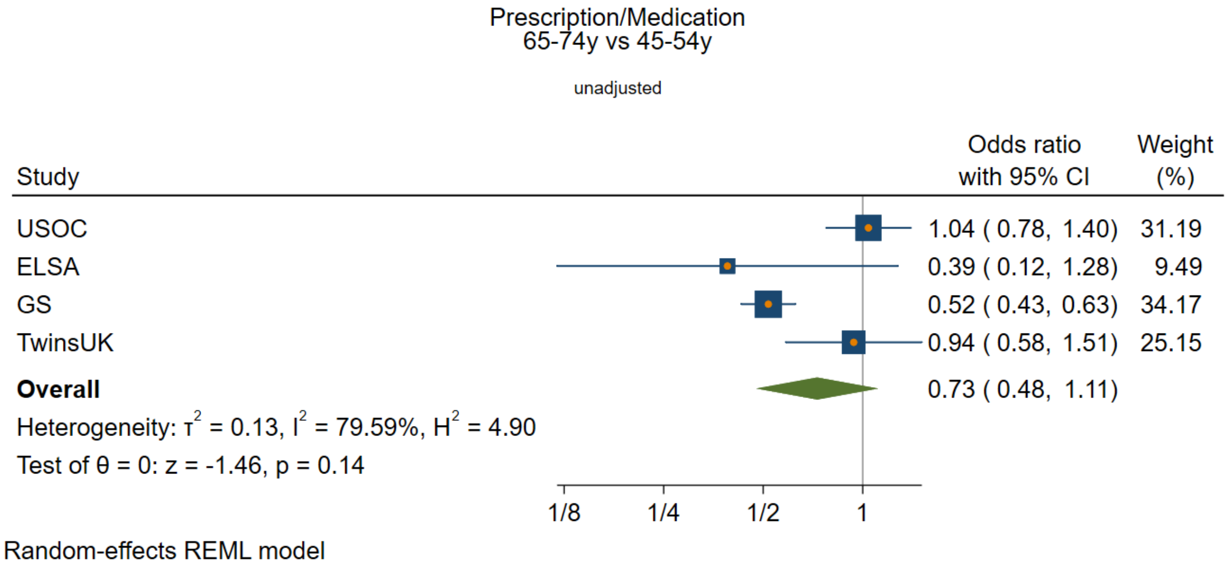

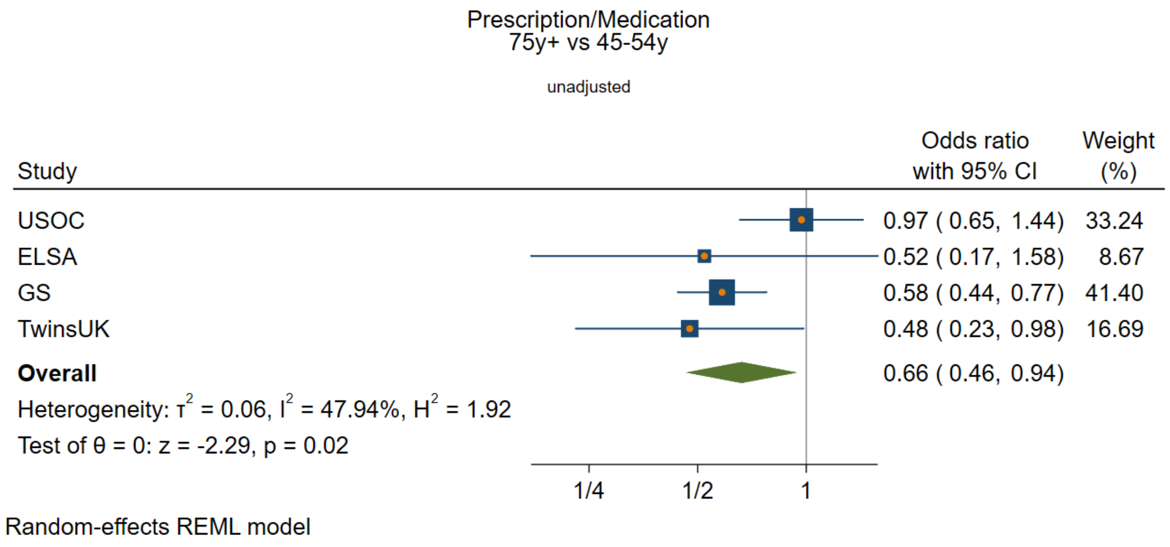

For peer review only - http://bmjopen.bmj.com/site/about/guidelines.xhtml

Basic adjustment

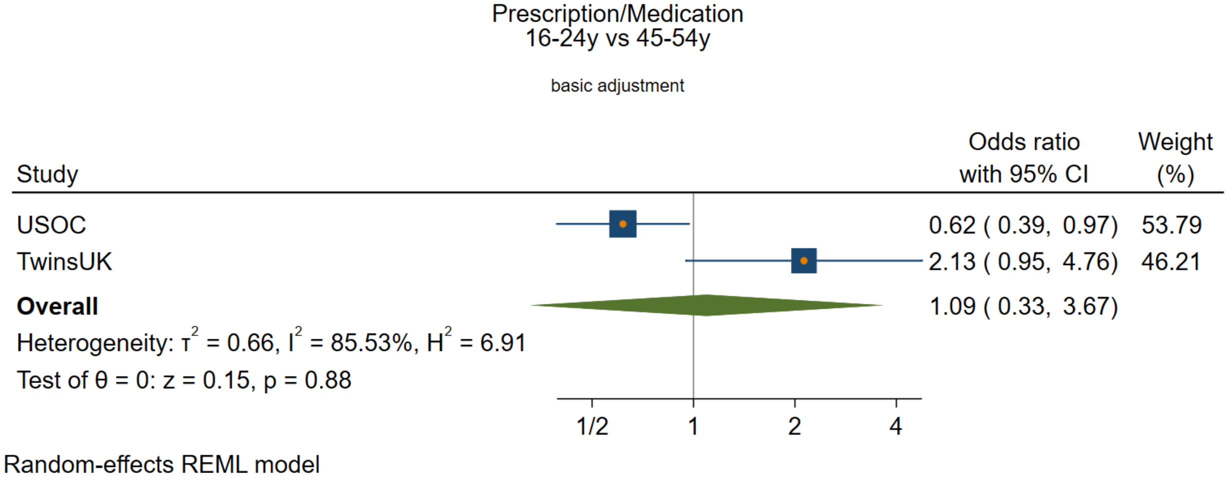

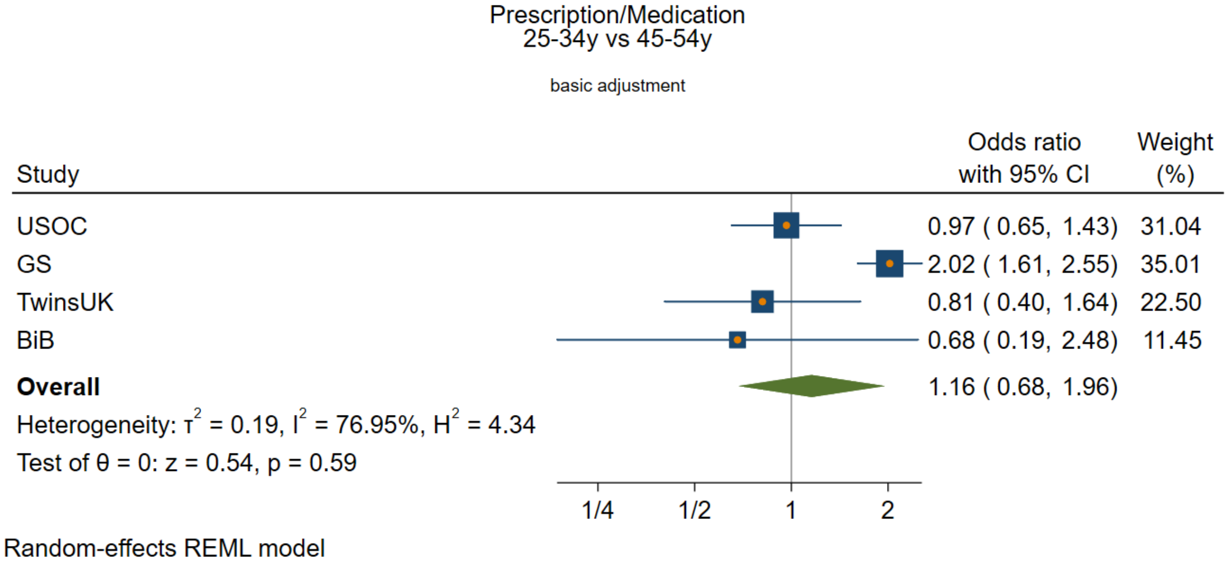

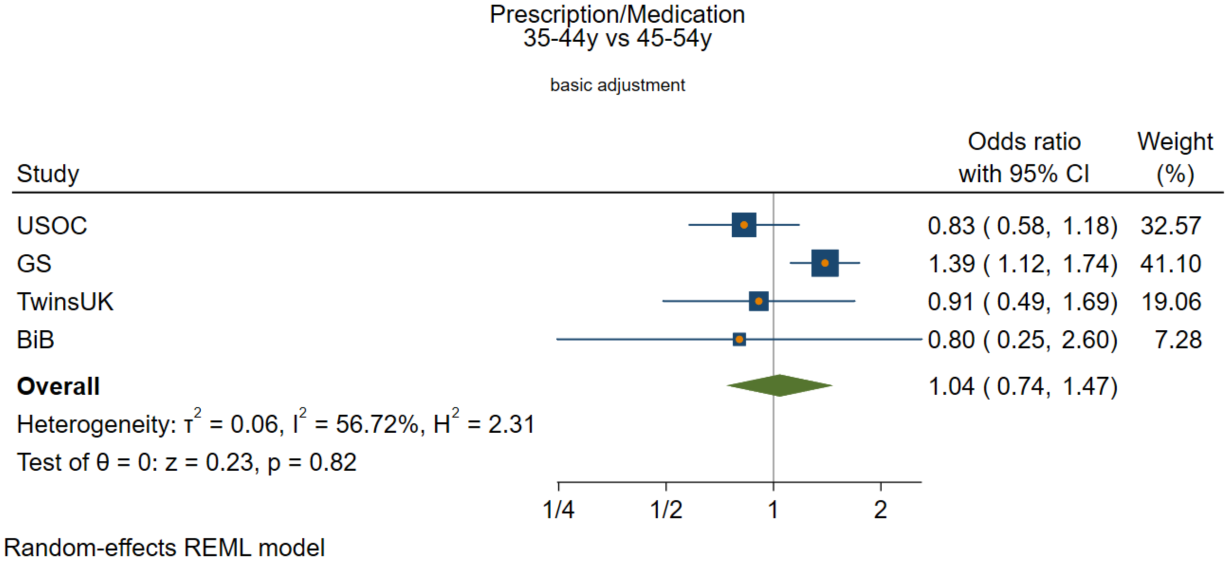

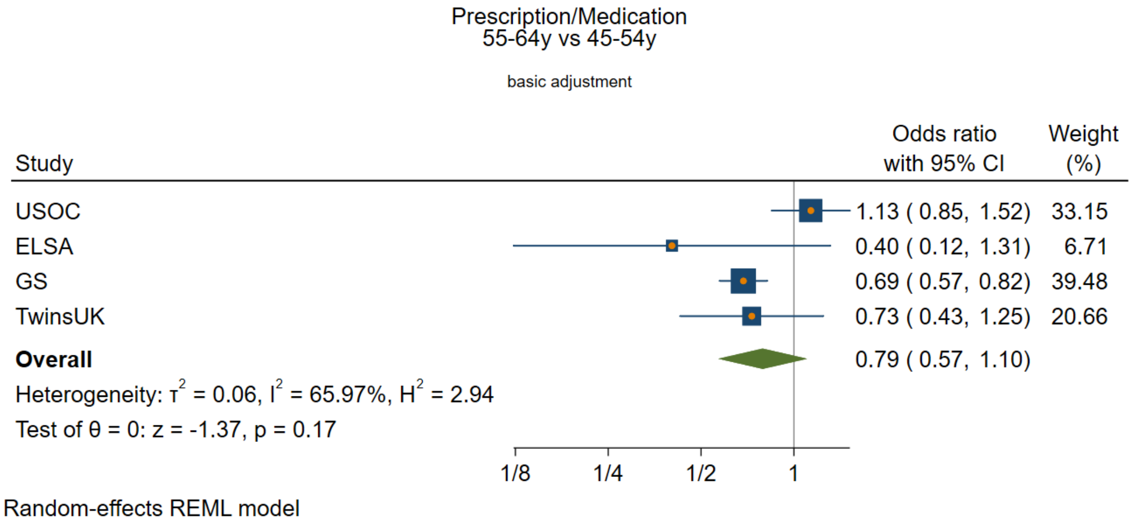

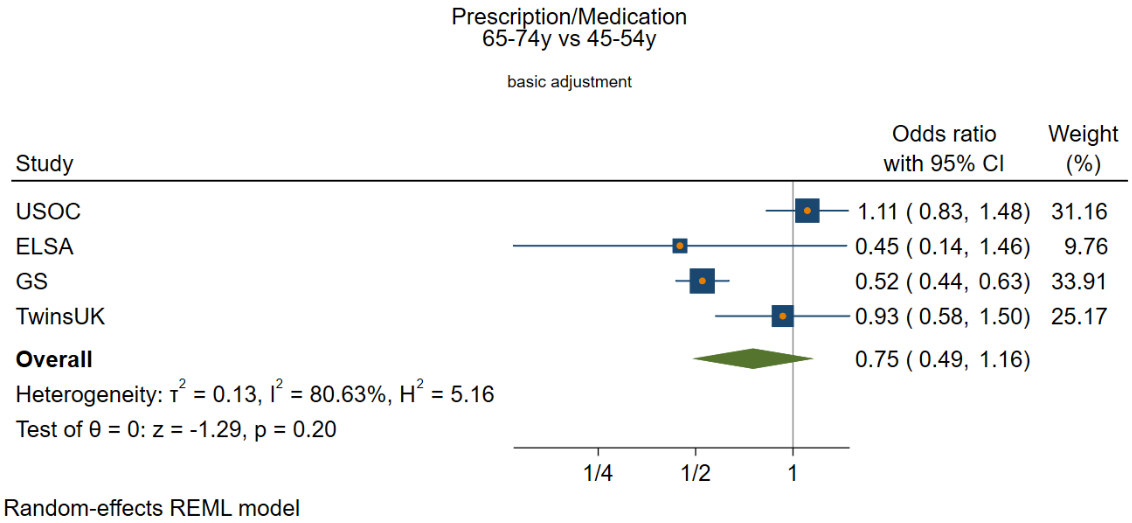

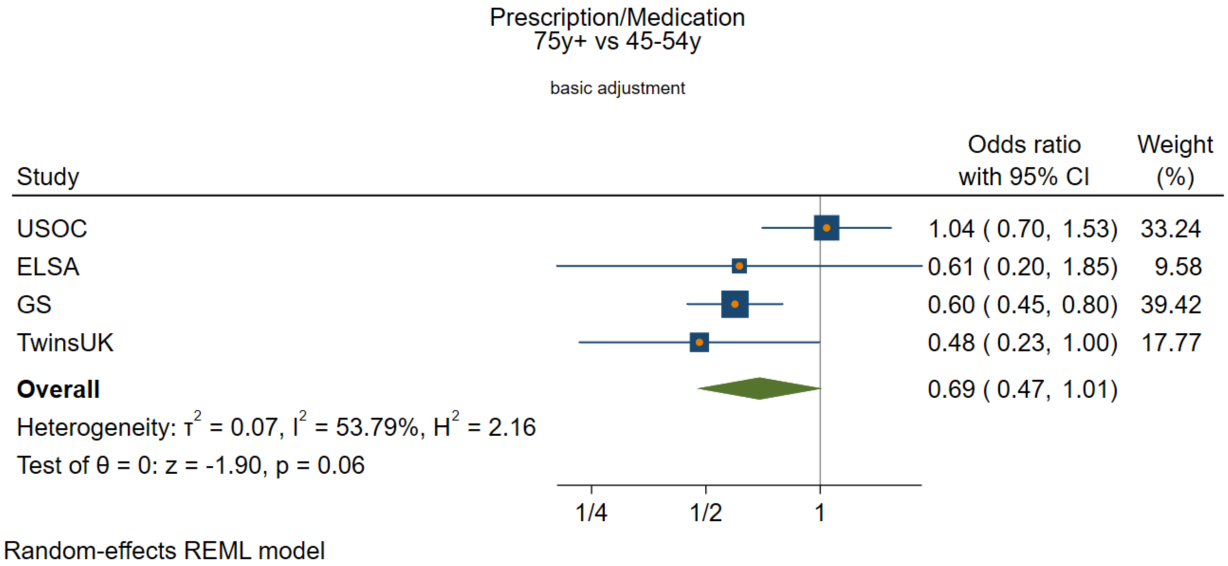

For peer review only - http://bmjopen.bmj.com/site/about/guidelines.xhtml

Full adjustment

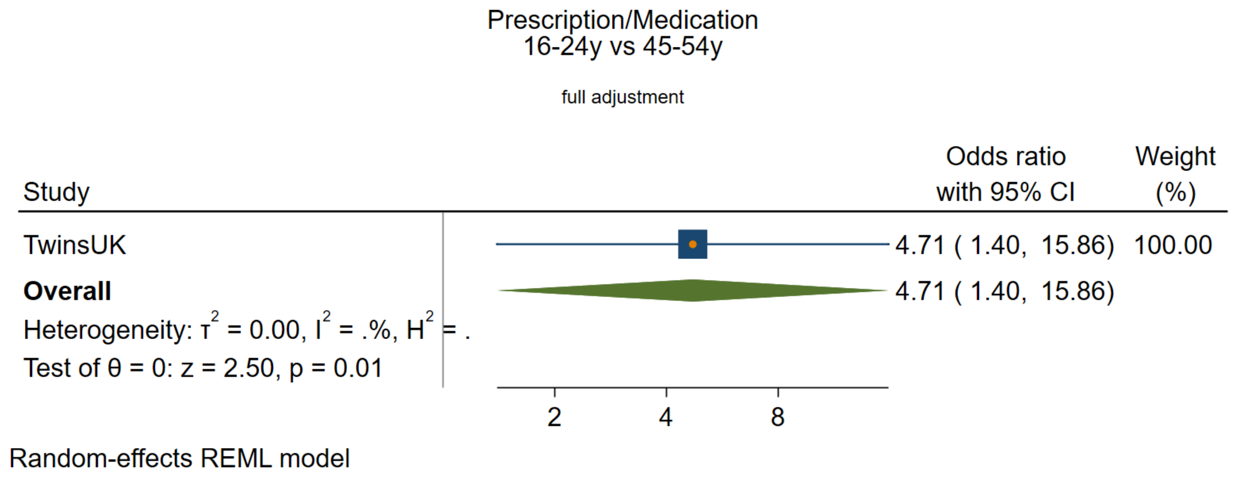

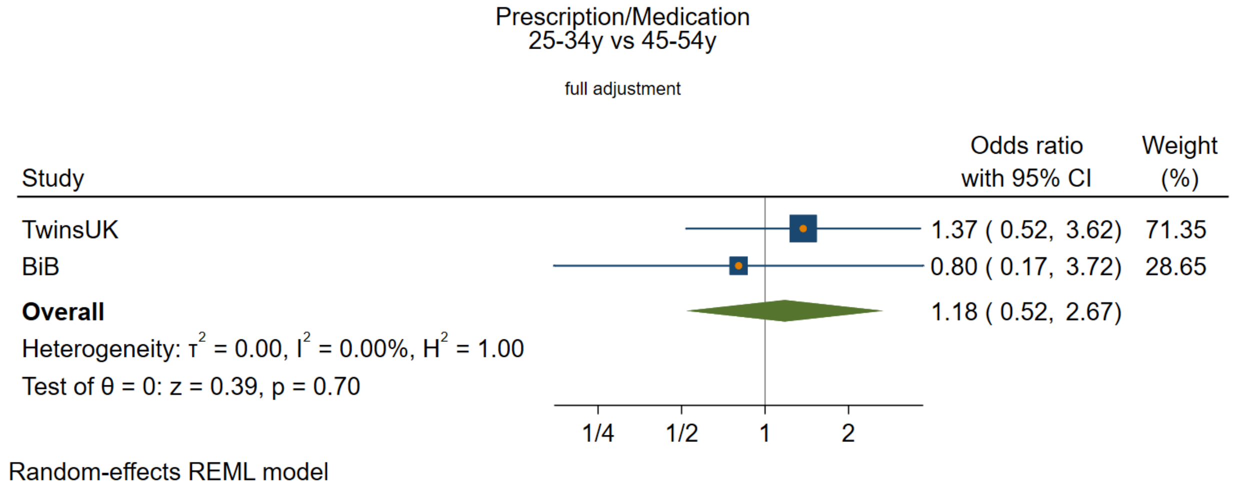

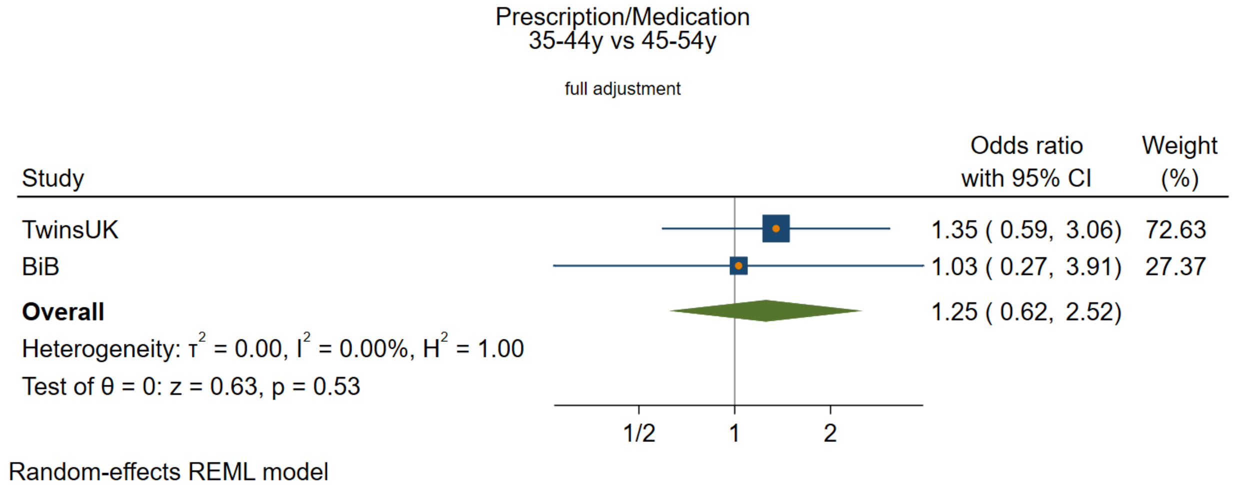

For peer review only - http://bmjopen.bmj.com/site/about/guidelines.xhtml

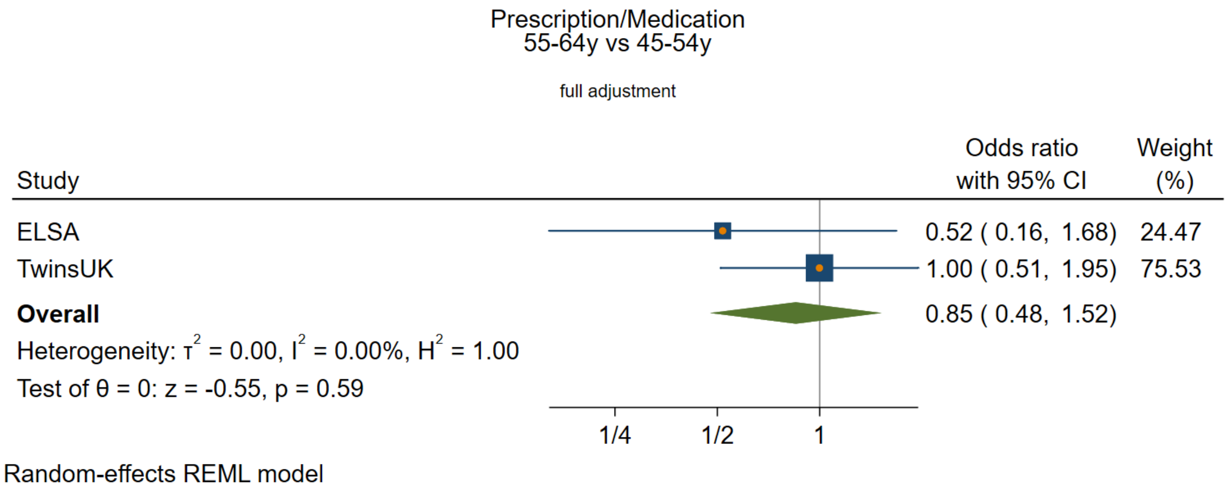

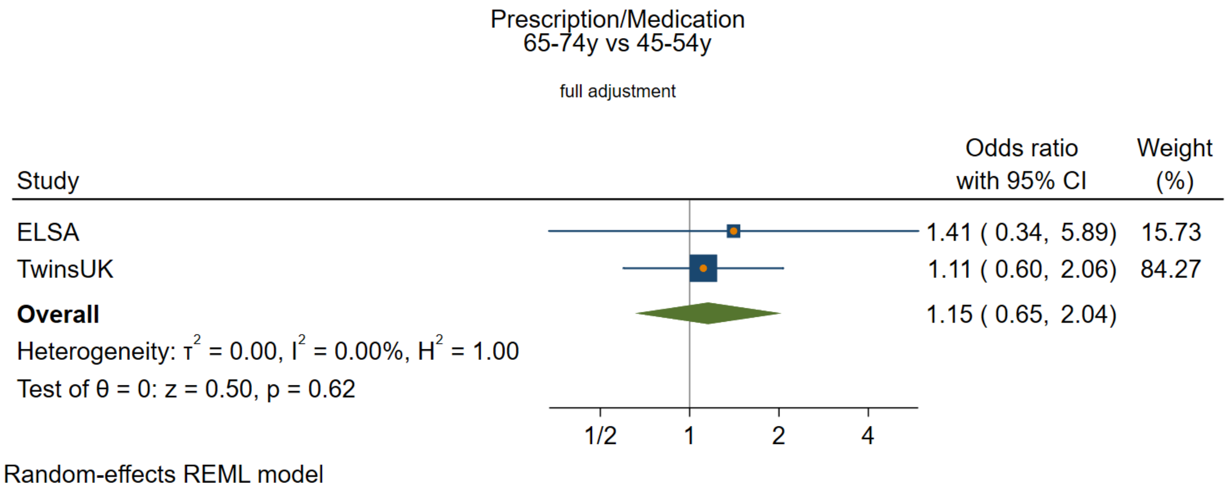

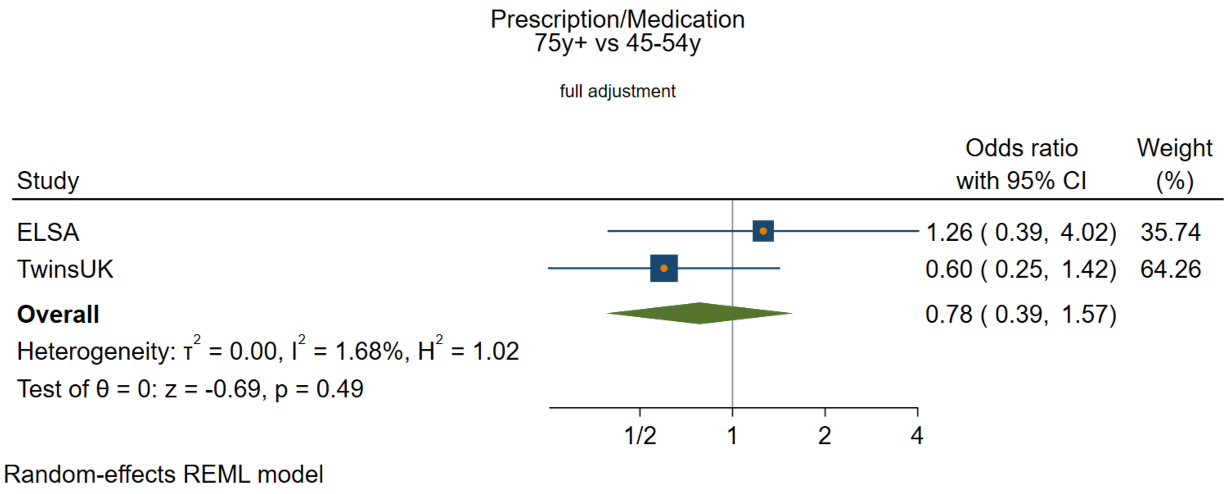

For peer review only - http://bmjopen.bmj.com/site/about/guidelines.xhtml

Ethnicity

Unadjusted

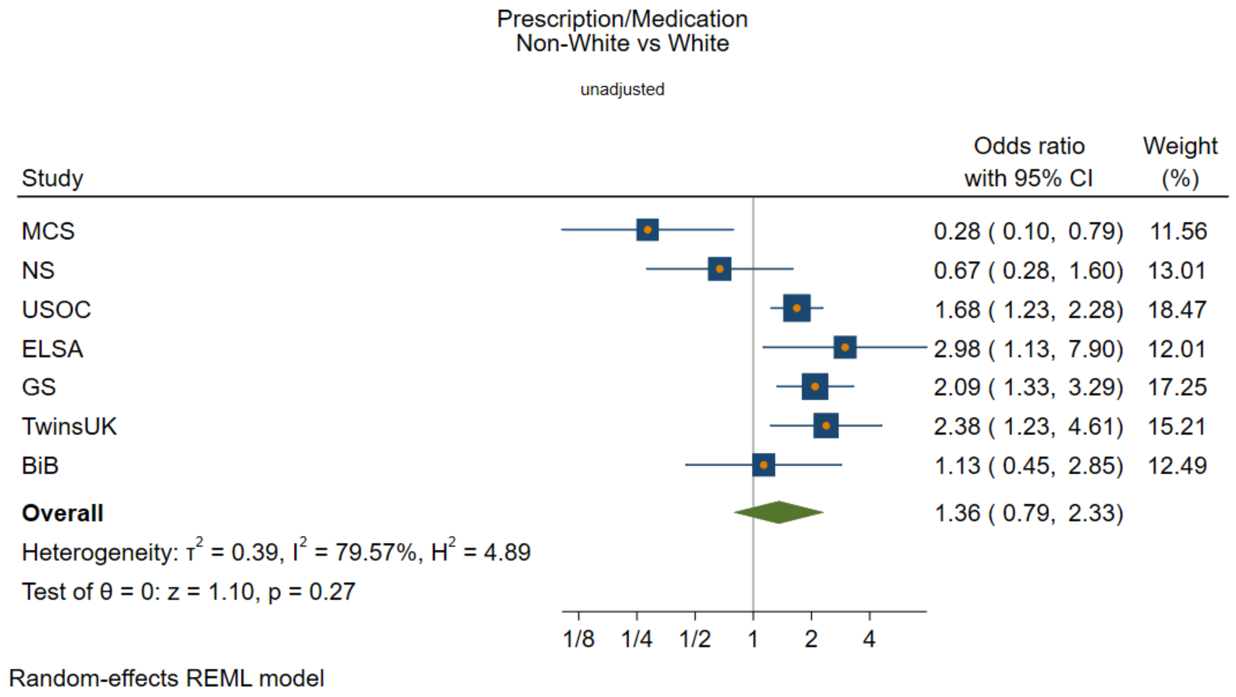

eview only

For peer review only - http://bmjopen.bmj.com/site/about/guidelines.xhtml

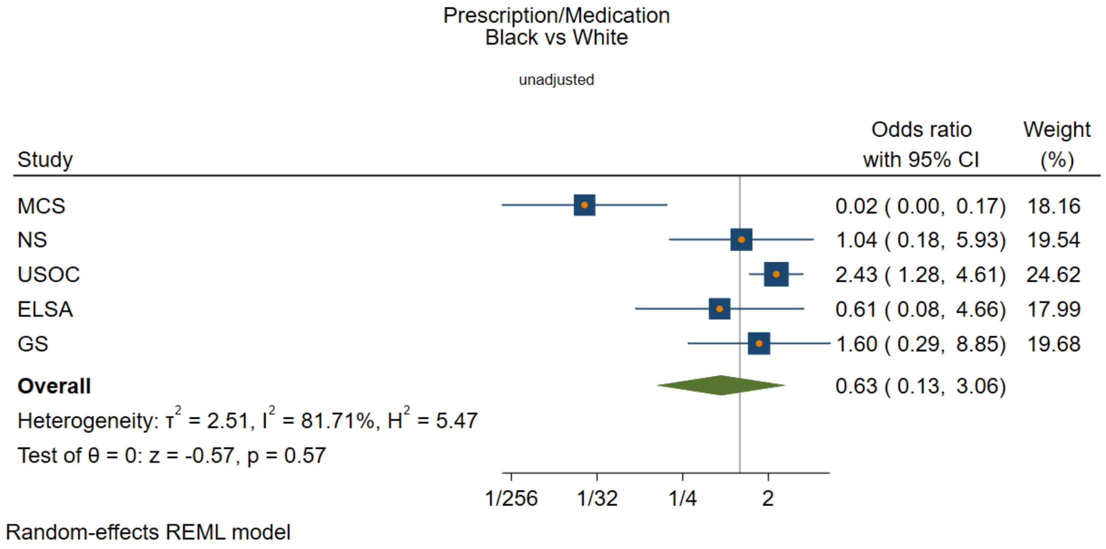

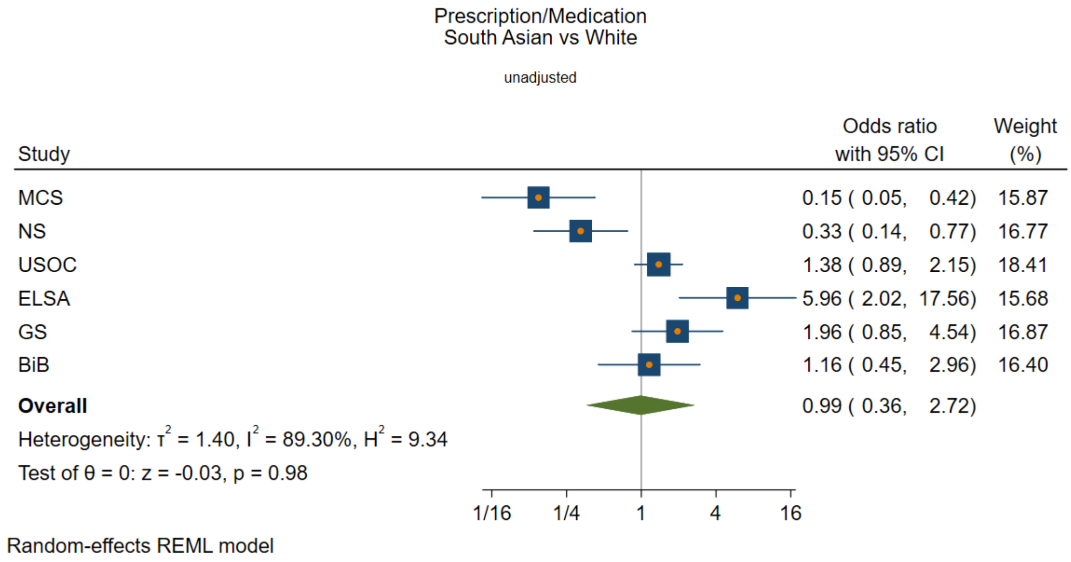

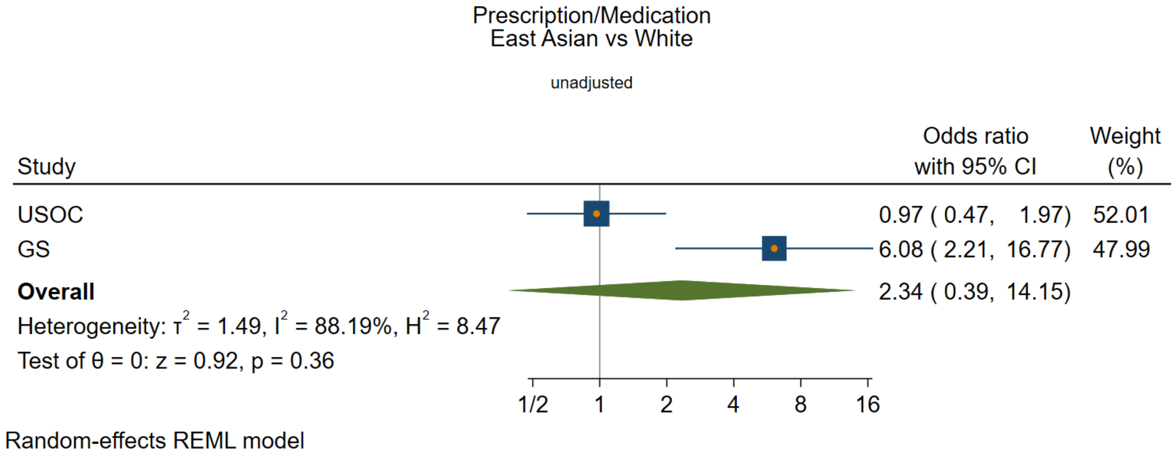

For peer review only - http://bmjopen.bmj.com/site/about/guidelines.xhtml

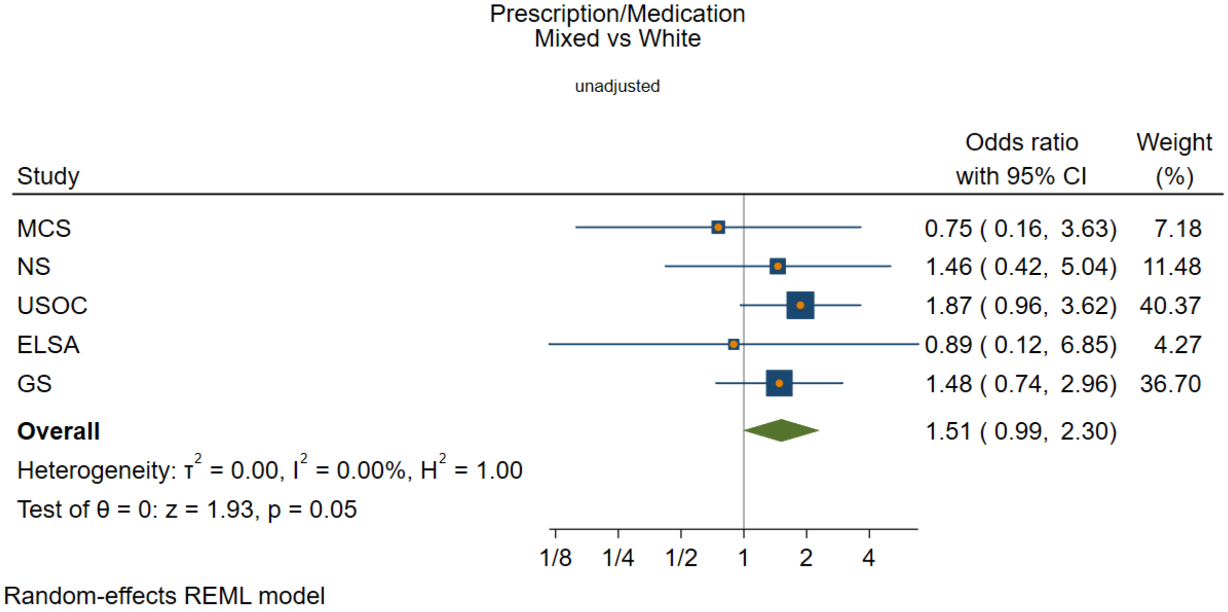

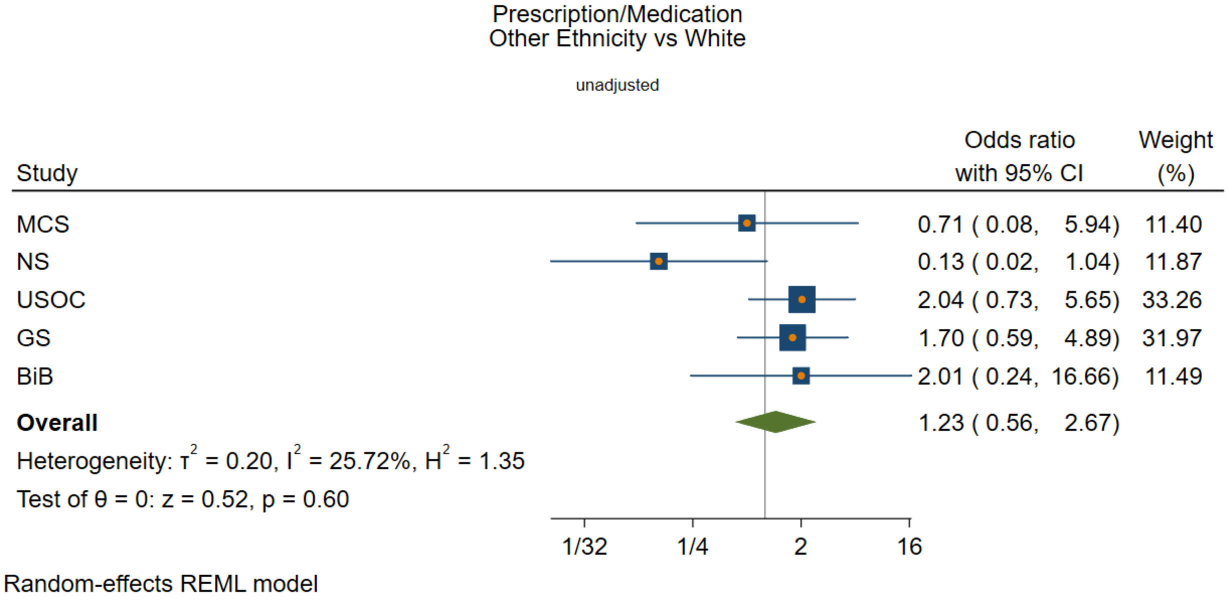

For peer review only - http://bmjopen.bmj.com/site/about/guidelines.xhtml

Basic adjustment

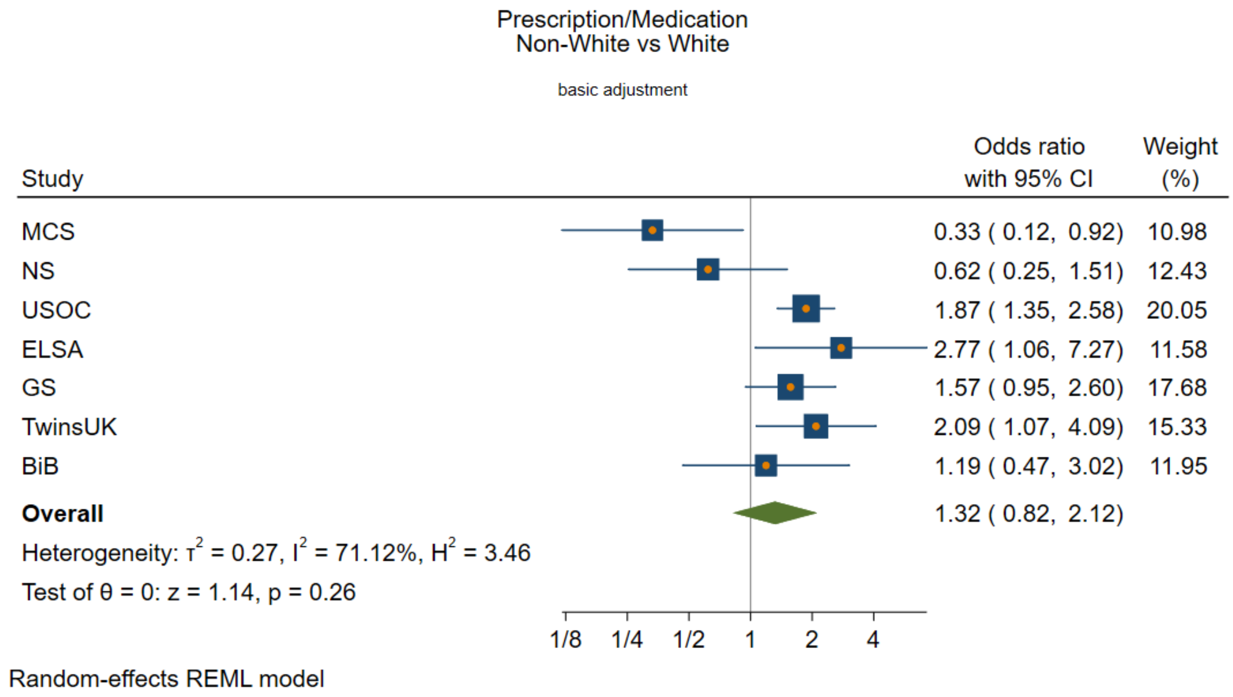

For peer review only - http://bmjopen.bmj.com/site/about/guidelines.xhtml

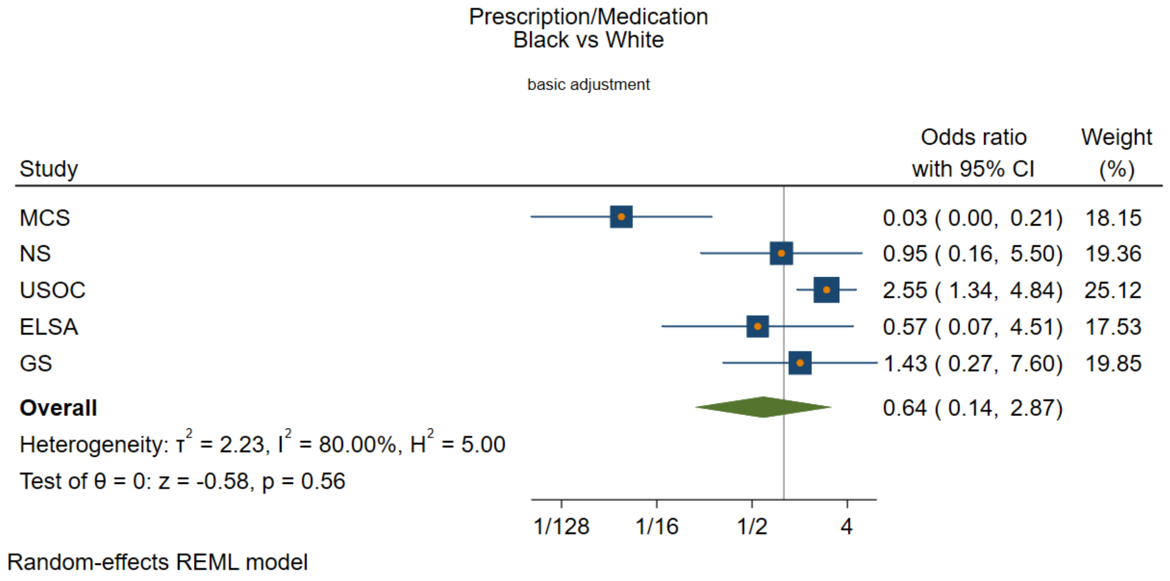

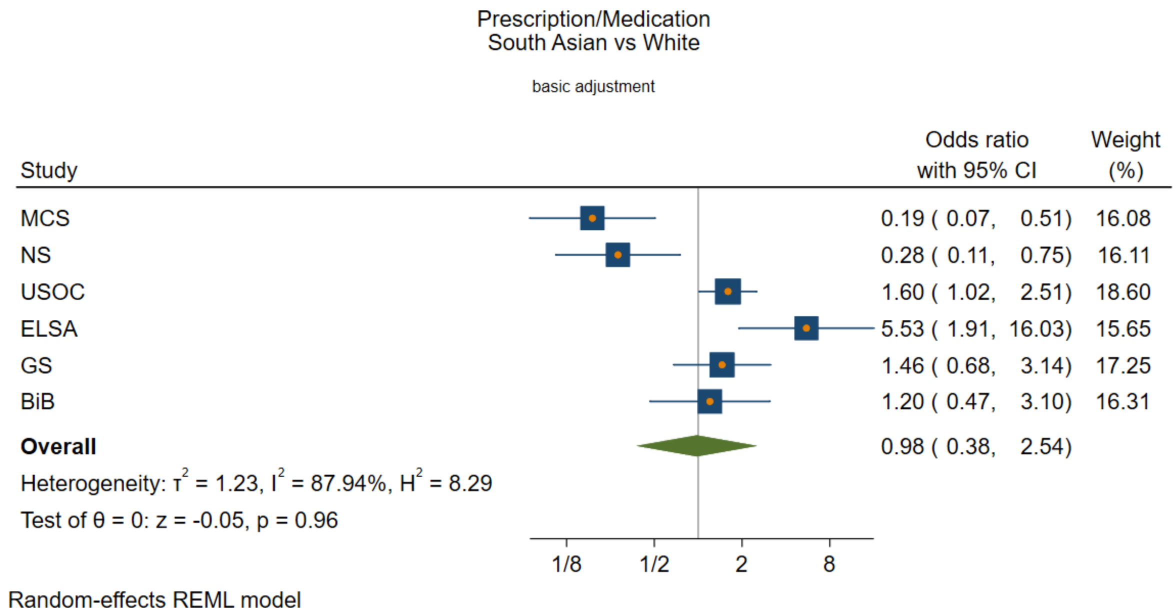

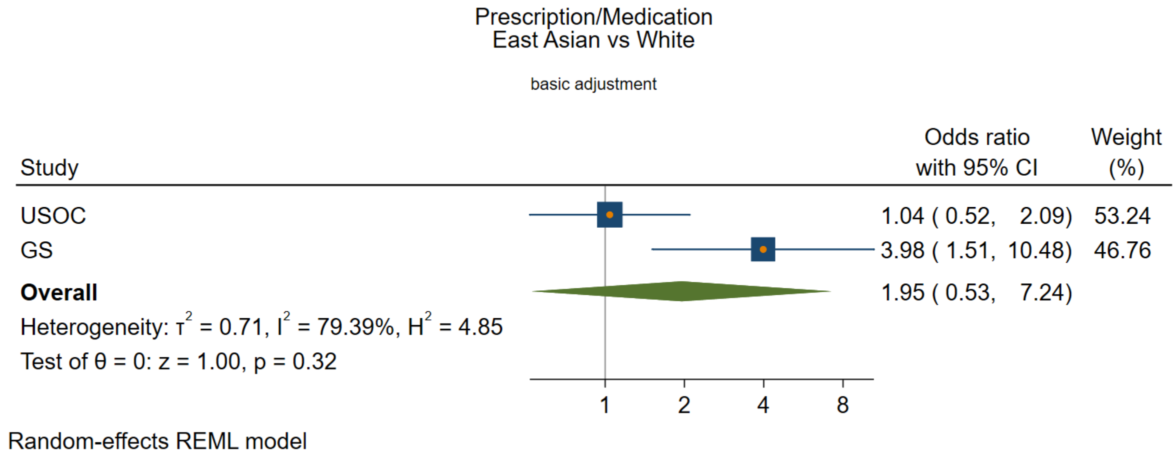

For peer review only - http://bmjopen.bmj.com/site/about/guidelines.xhtml

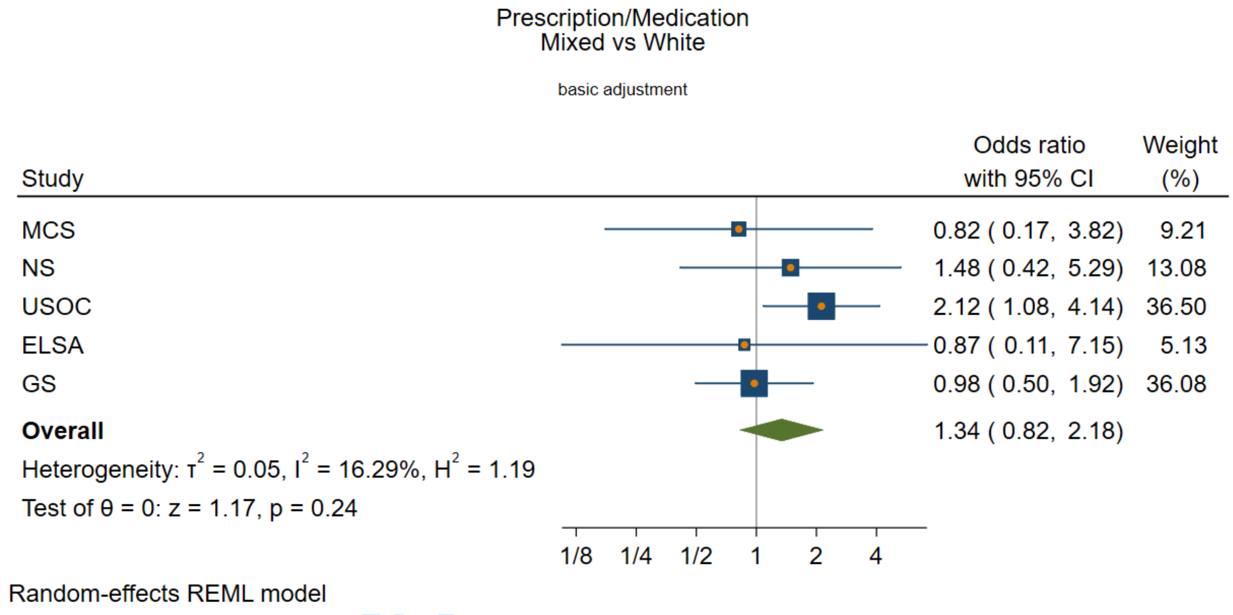

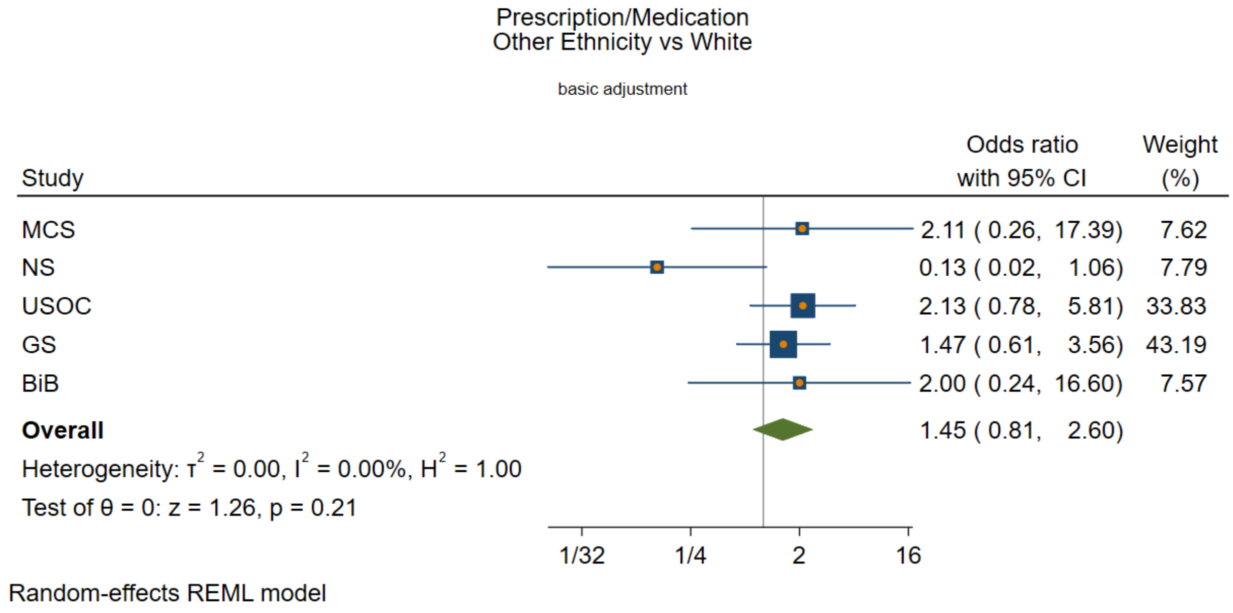

For peer review only - http://bmjopen.bmj.com/site/about/guidelines.xhtml

Full adjustment

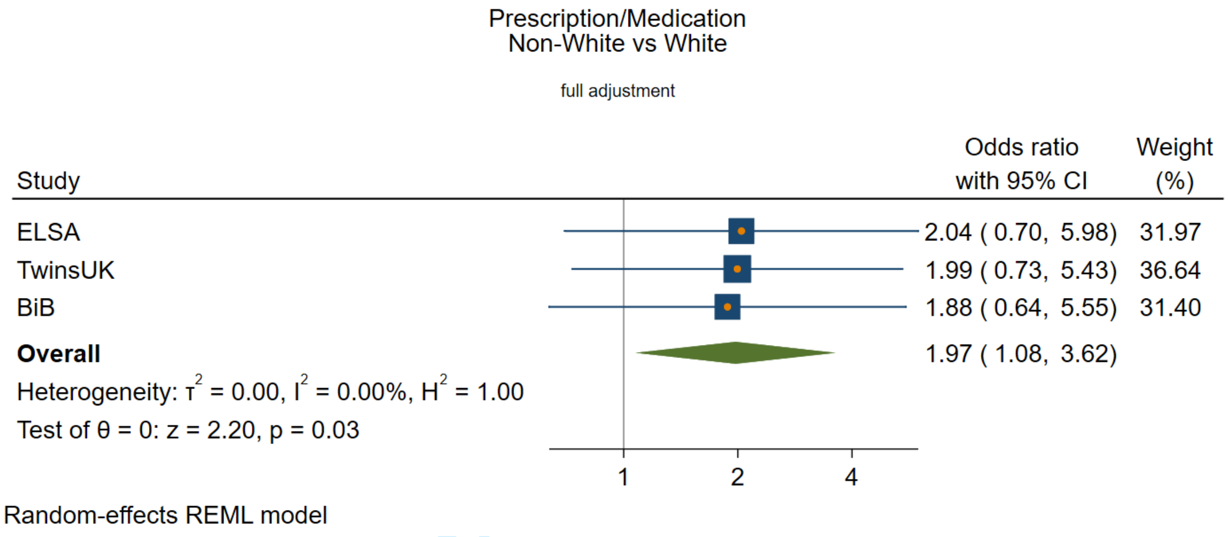

peer review only

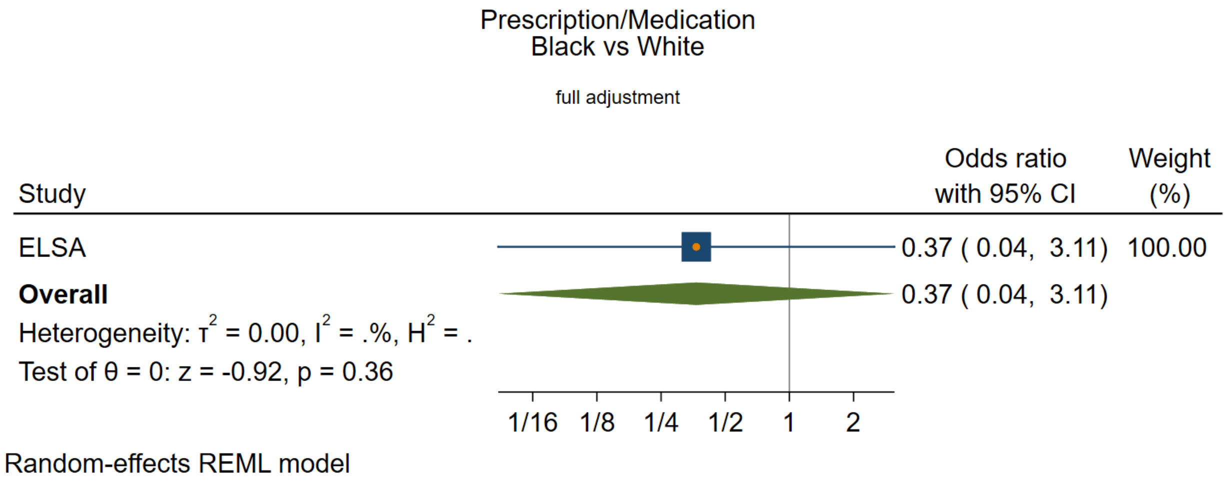

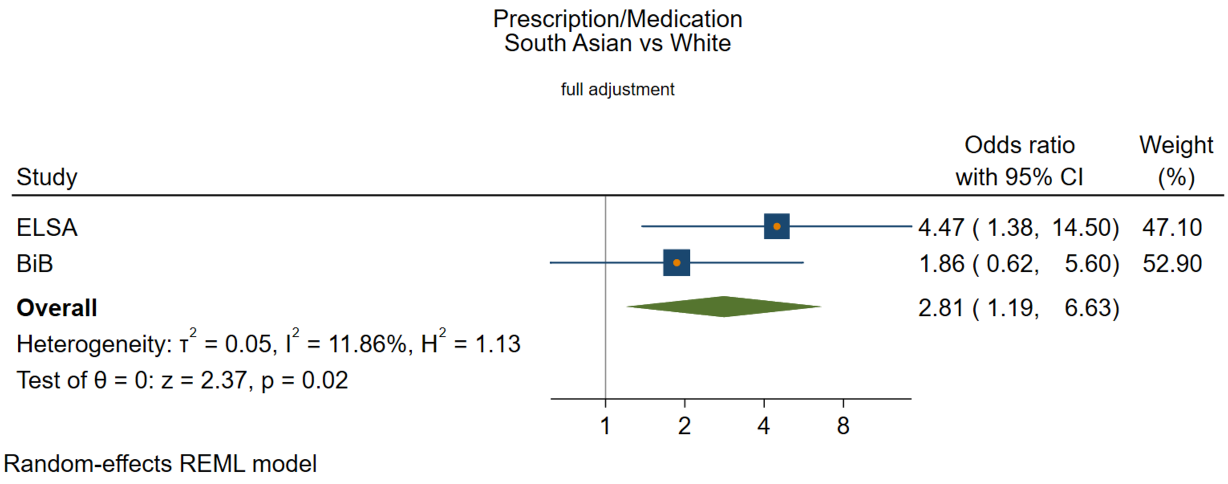

For peer review only - http://bmjopen.bmj.com/site/about/guidelines.xhtml

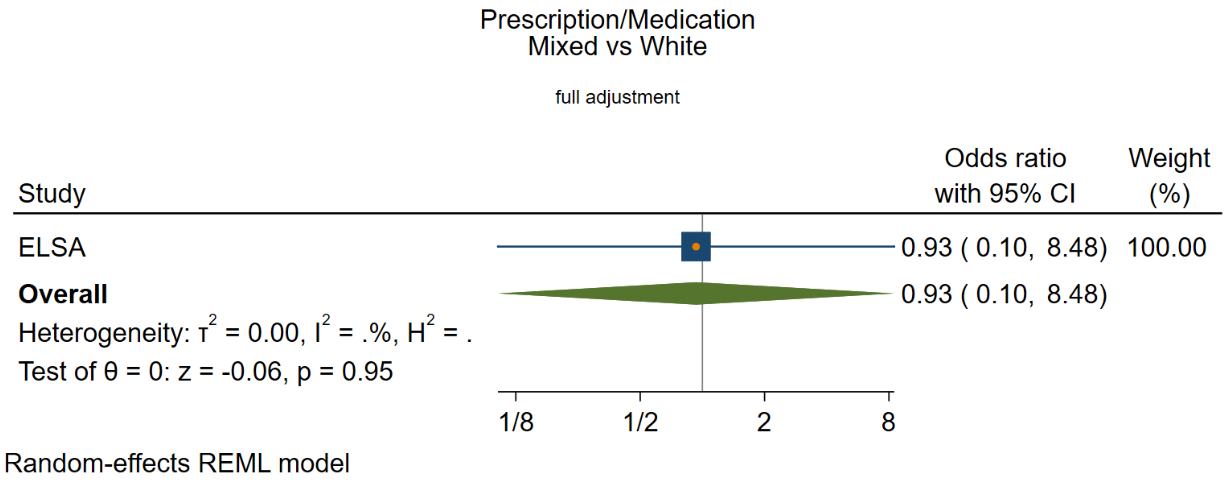

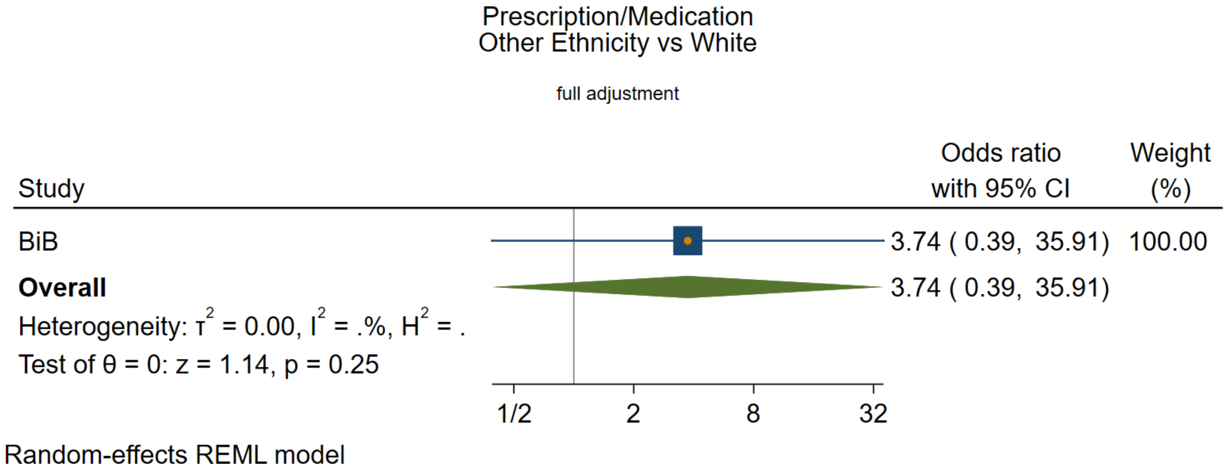

For peer review only - http://bmjopen.bmj.com/site/about/guidelines.xhtml

Procedures/surgery

Sex
Unadjusted

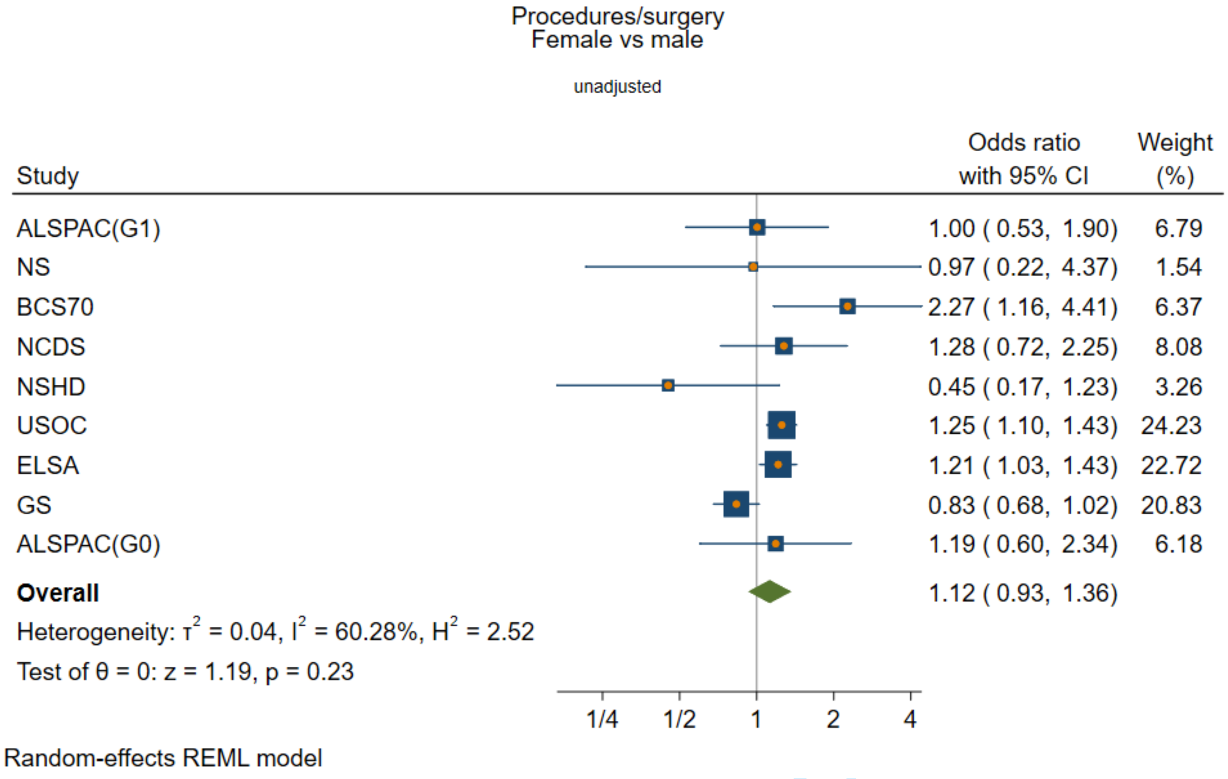

For peer review only - http://bmjopen.bmj.com/site/about/guidelines.xhtml

Basic adjustment

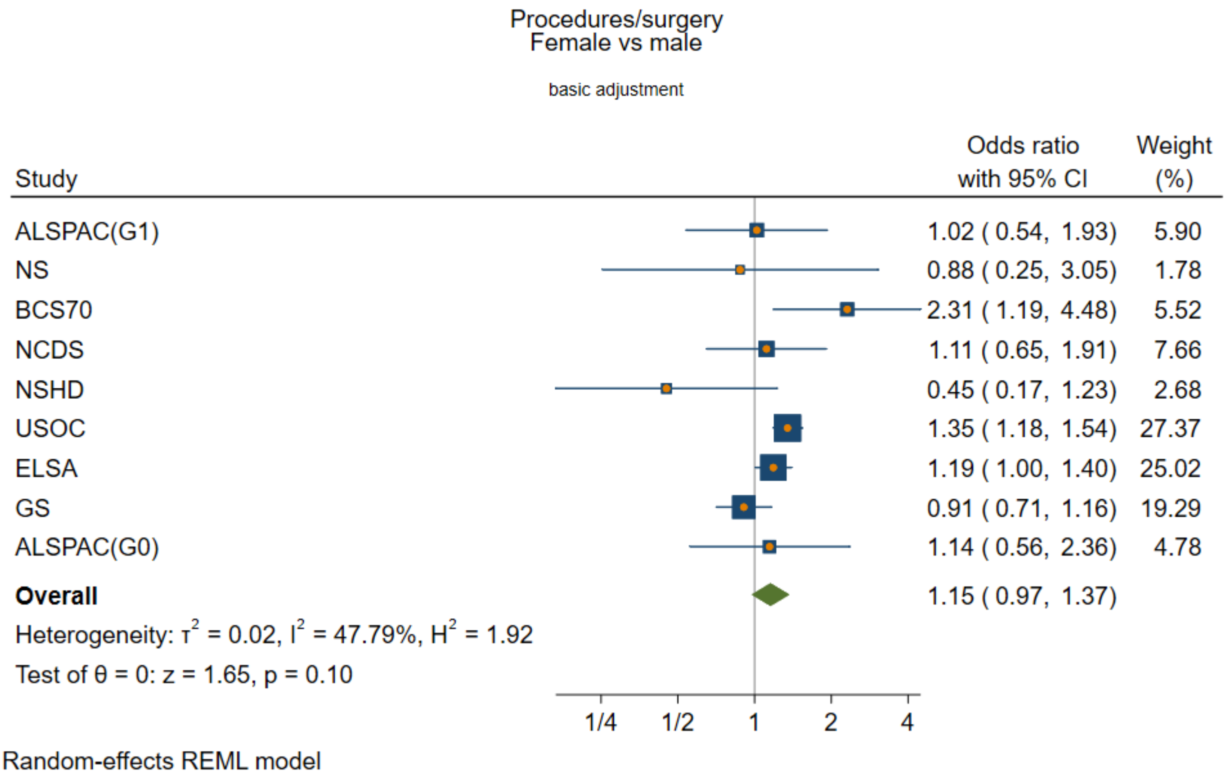

view only

For peer review only - http://bmjopen.bmj.com/site/about/guidelines.xhtml

Full adjustment

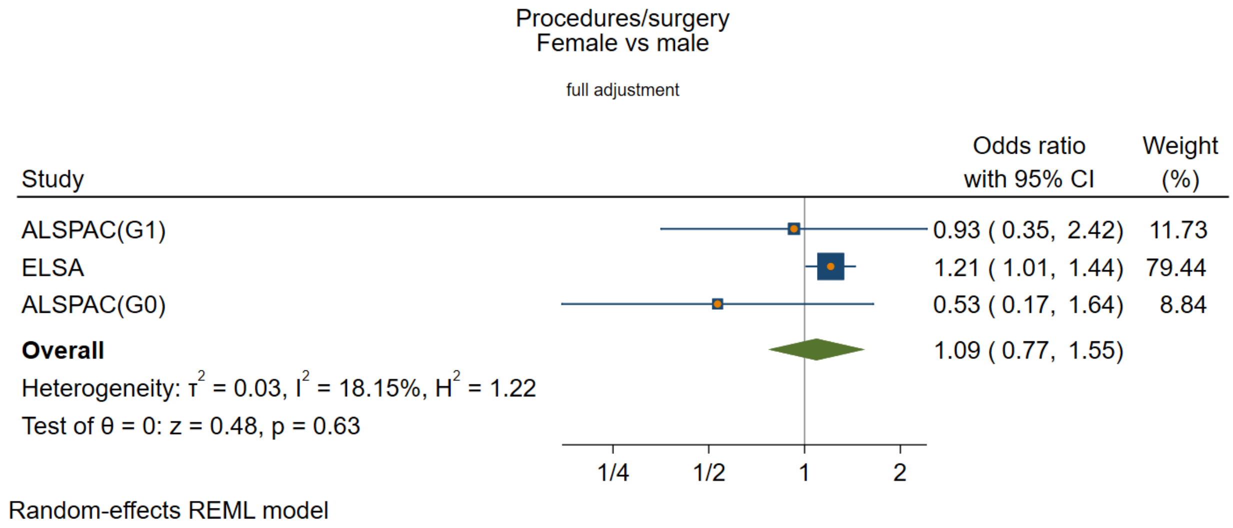

For peer review only

Occupational class

Unadjusted

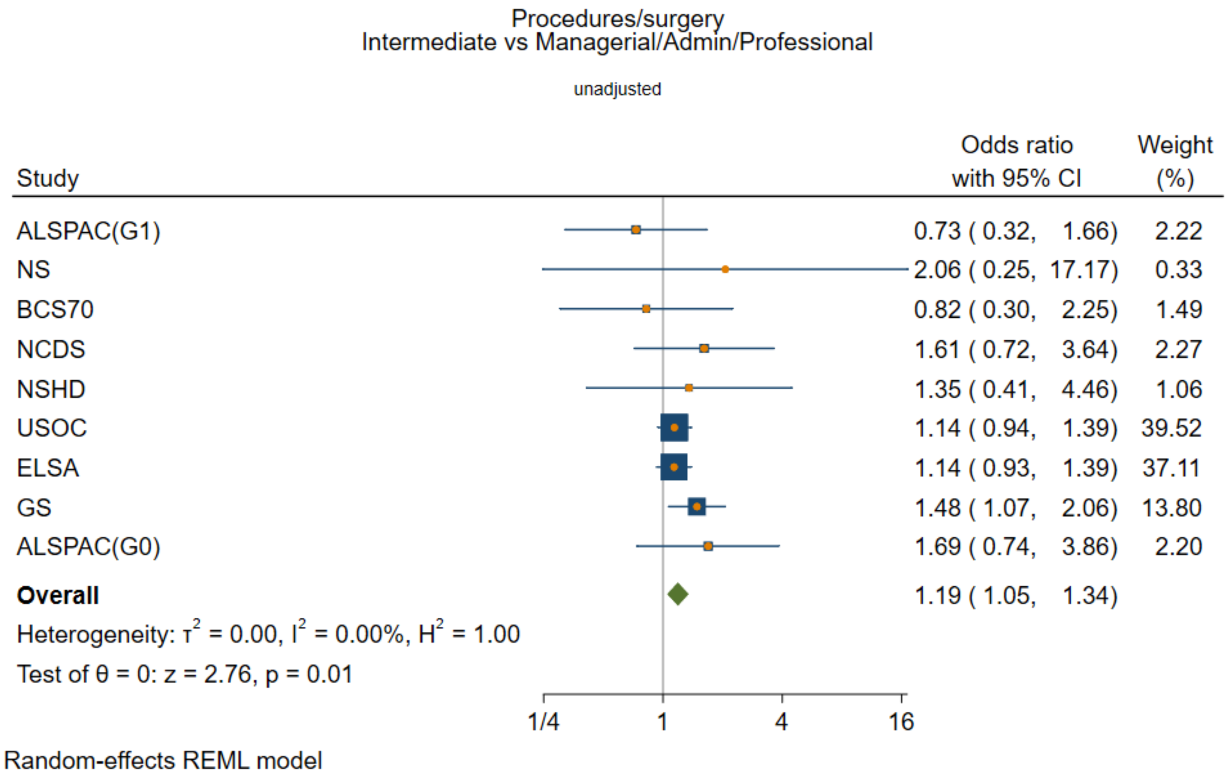

iew only

For peer review only - http://bmjopen.bmj.com/site/about/guidelines.xhtml

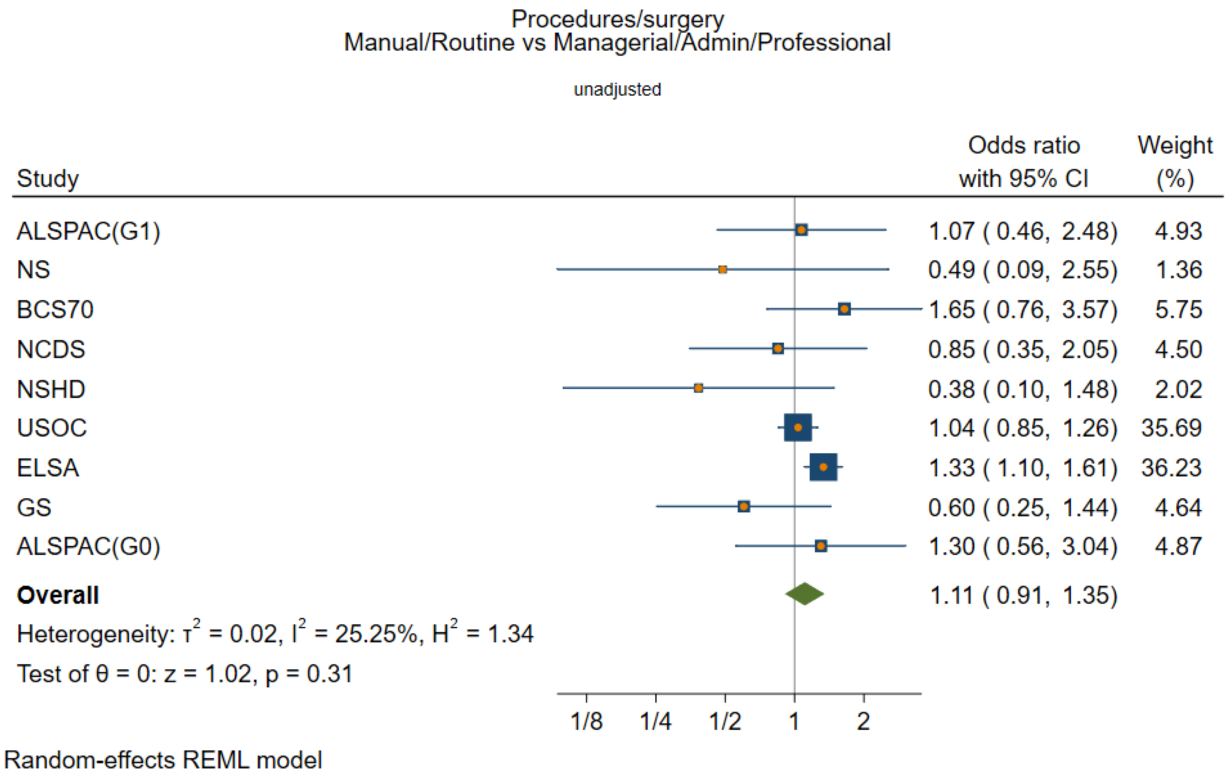
Procedures/surgery
Manual/Routine vs Managerial/Admin/Professional
unadjusted
Study
Odds ratio with 95% CI
Weight (%)
ALSPAC(G1) 1.07 (0.46, 2.48) 4.93
NS 0.49 (0.09, 2.55) 1.36
BCS70 1.65 (0.76, 3.57) 5.75
NCDS 0.85 (0.35, 2.05) 4.50
NSHD 0.38 (0.10, 1.48) 2.02
USOC 1.04 (0.85, 1.26) 35.69
ELSA 1.33 (1.10, 1.61) 36.23
GS 0.60 (0.25, 1.44) 4.64
ALSPAC(G0) 1.30 (0.56, 3.04) 4.87
Overall 1.11 (0.91, 1.35)
Heterogeneity: τ2 = 0.02, I2 = 25.25%, H2 = 1.34
Test of θ = 0: z = 1.02, p = 0.31
1/8
1/4
1/2
1
2
Random-effects REML model

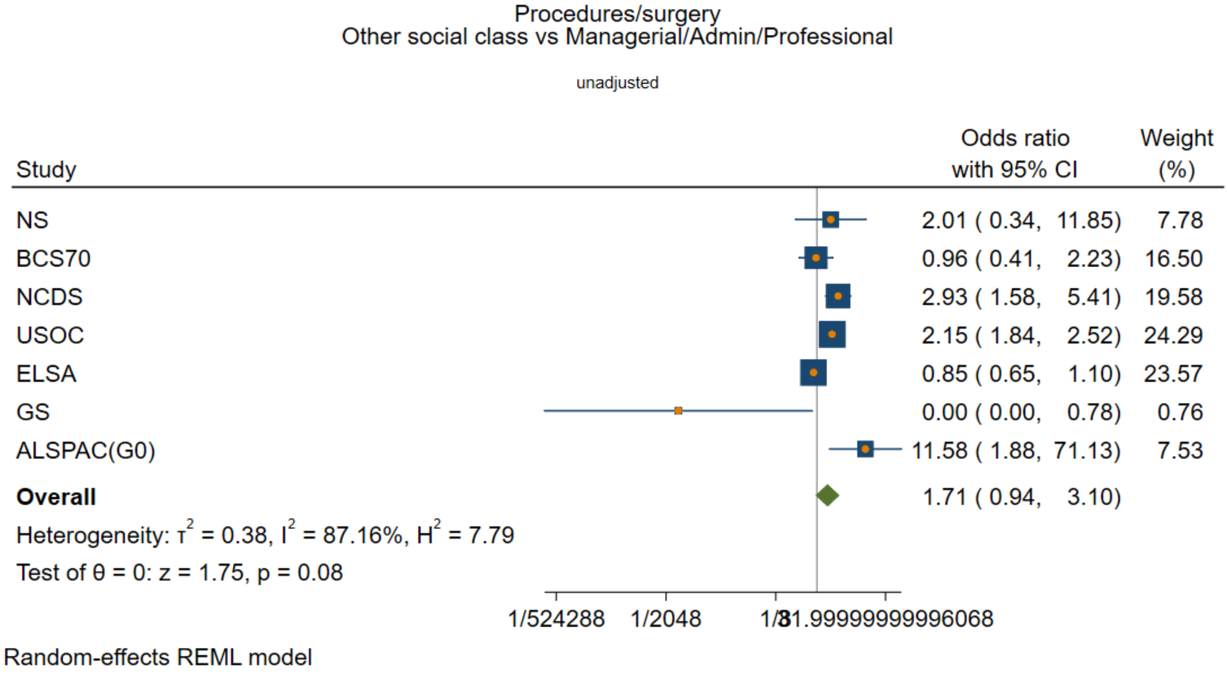
Procedures/surgery
Other social class vs Managerial/Admin/Professional
unadjusted
Study
Odds ratio with 95% CI
Weight (%)
NS 2.01 (0.34, 11.85) 7.78
BCS70 0.96 (0.41, 2.23) 16.50
NCDS 2.93 (1.58, 5.41) 19.58
USOC 2.15 (1.84, 2.52) 24.29
ELSA 0.85 (0.65, 1.10) 23.57
GS 0.00 (0.00, 0.78) 0.76
ALSPAC(G0) 11.58 (1.88, 71.13) 7.53
Overall 1.71 (0.94, 3.10)
Heterogeneity: τ2 = 0.38, I2 = 87.16%, H2 = 7.79
Test of θ = 0: z = 1.75, p = 0.08
1/524288
1/2048
1/8
31.99999999996068
Random-effects REML model

Basic adjustment

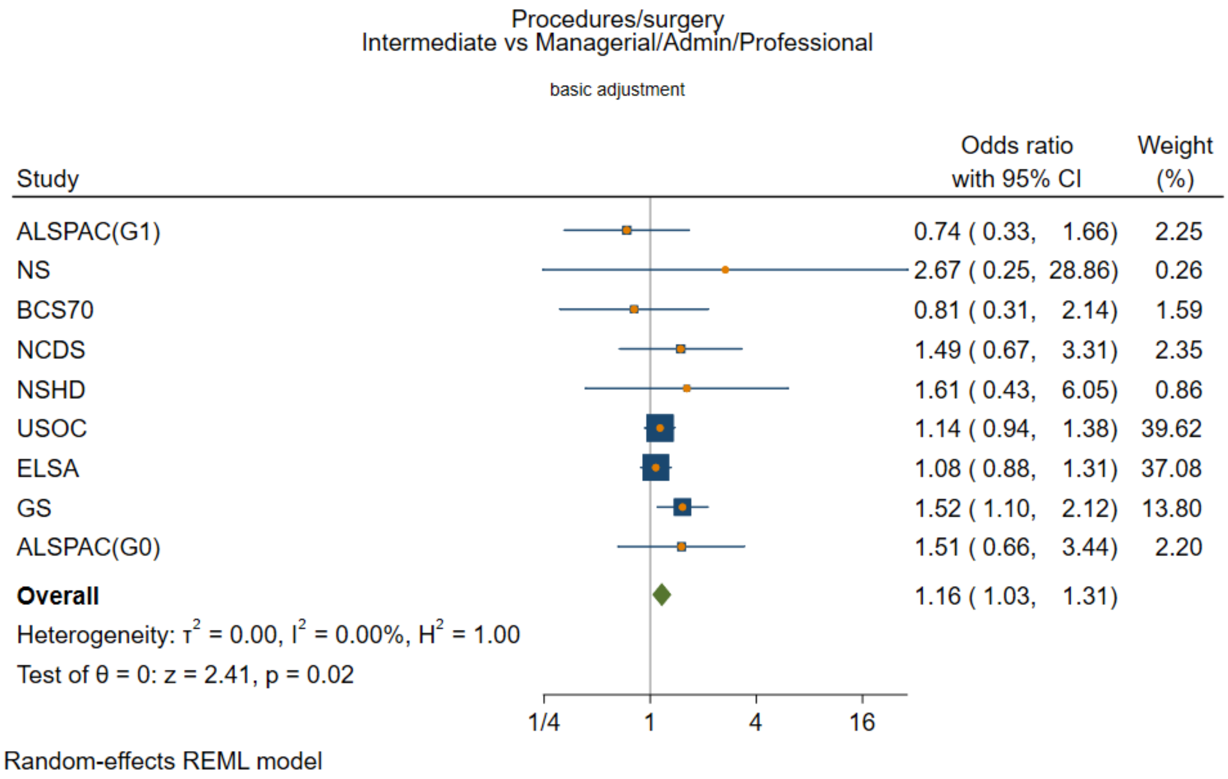

view only

For peer review only - http://bmjopen.bmj.com/site/about/guidelines.xhtml

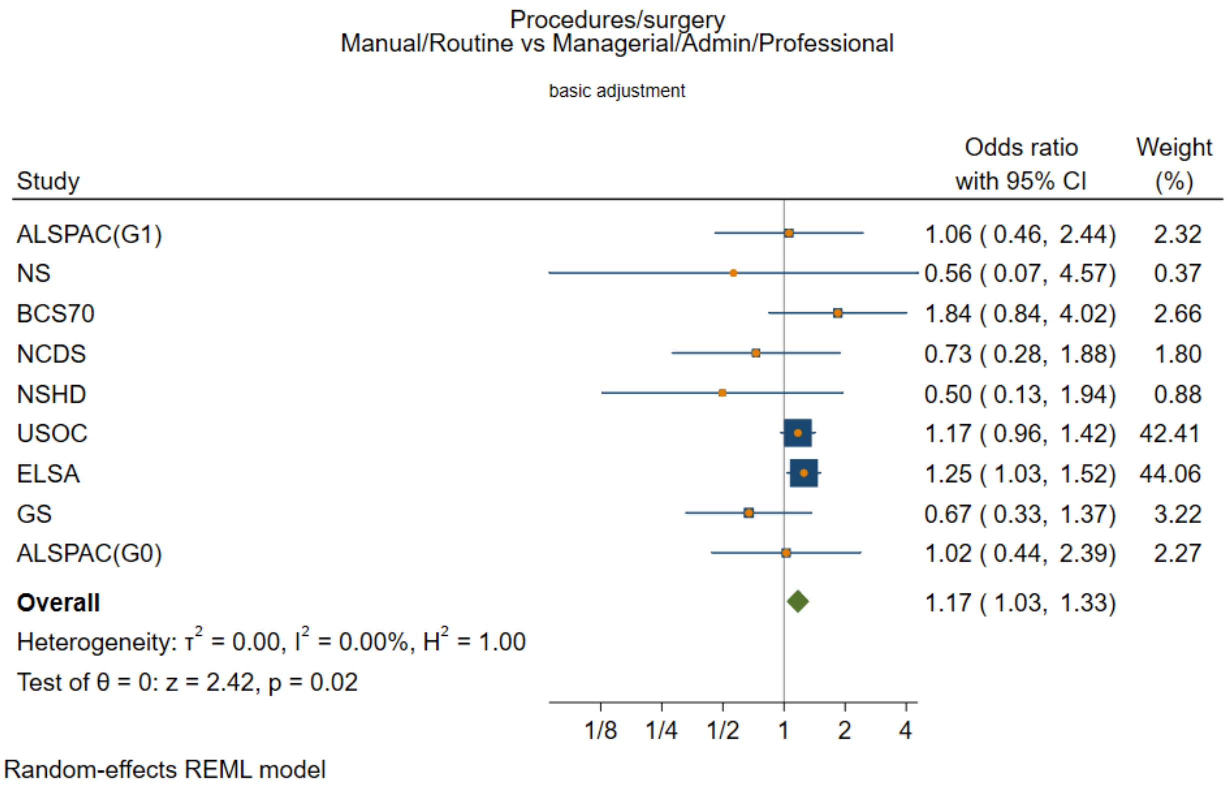
Procedures/surgery
Manual/Routine vs Managerial/Admin/Professional
basic adjustment
Study
Odds ratio with 95% CI
Weight (%)
ALSPAC(G1) 1.06 (0.46, 2.44) 2.32
NS 0.56 (0.07, 4.57) 0.37
BCS70 1.84 (0.84, 4.02) 2.66
NCDS 0.73 (0.28, 1.88) 1.80
NSHD 0.50 (0.13, 1.94) 0.88
USOC 1.17 (0.96, 1.42) 42.41
ELSA 1.25 (1.03, 1.52) 44.06
GS 0.67 (0.33, 1.37) 3.22
ALSPAC(G0) 1.02 (0.44, 2.39) 2.27
Overall 1.17 (1.03, 1.33)
Heterogeneity: τ² = 0.00, I² = 0.00%, H² = 1.00
Test of θ = 0: z = 2.42, p = 0.02
1/8 1/4 1/2 1 2 4
Random-effects REML model

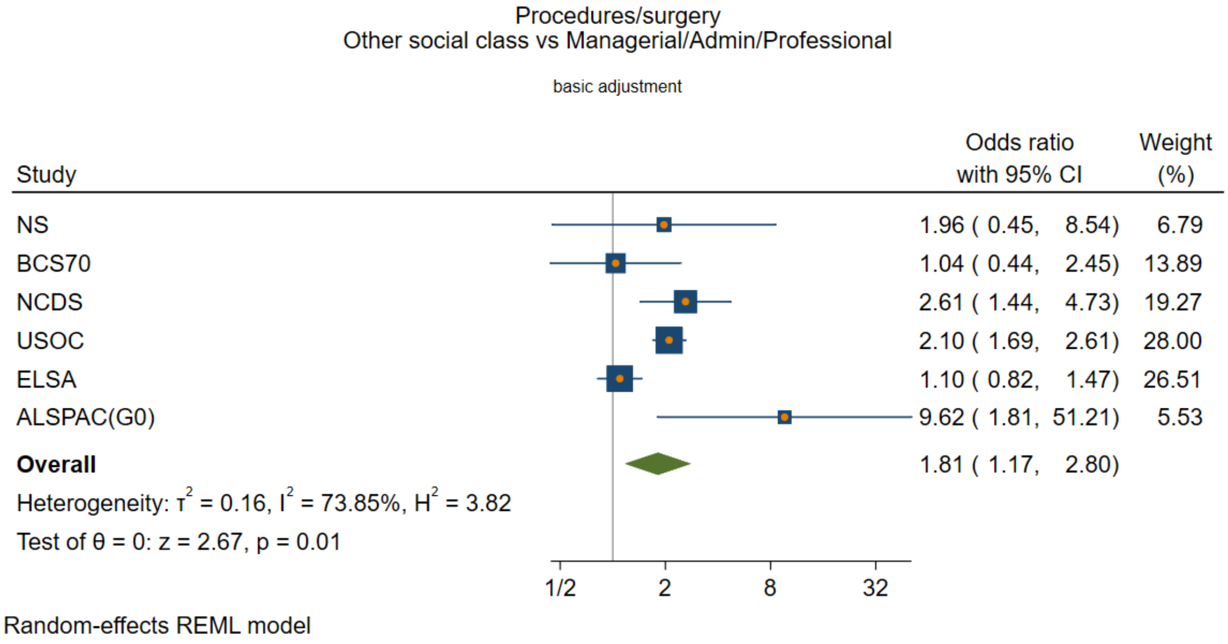
Procedures/surgery
Other social class vs Managerial/Admin/Professional
basic adjustment
Study
Odds ratio with 95% CI
Weight (%)
NS 1.96 (0.45, 8.54) 6.79
BCS70 1.04 (0.44, 2.45) 13.89
NCDS 2.61 (1.44, 4.73) 19.27
USOC 2.10 (1.69, 2.61) 28.00
ELSA 1.10 (0.82, 1.47) 26.51
ALSPAC(G0) 9.62 (1.81, 51.21) 5.53
Overall 1.81 (1.17, 2.80)
Heterogeneity: τ² = 0.16, I² = 73.85%, H² = 3.82
Test of θ = 0: z = 2.67, p = 0.01
1/2 2 8 32
Random-effects REML model

Full adjustment

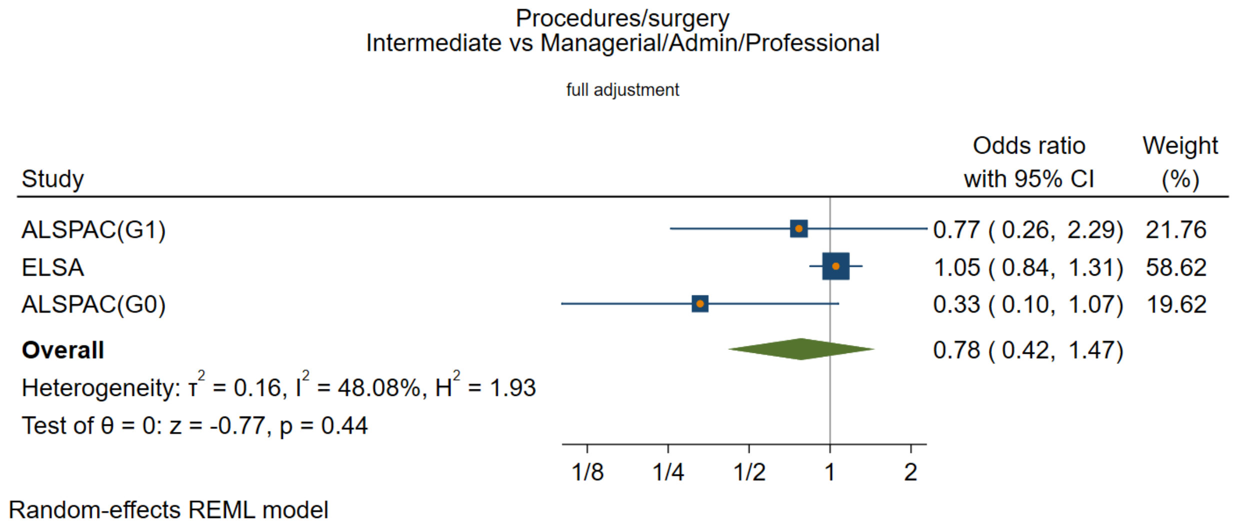

For peer review only

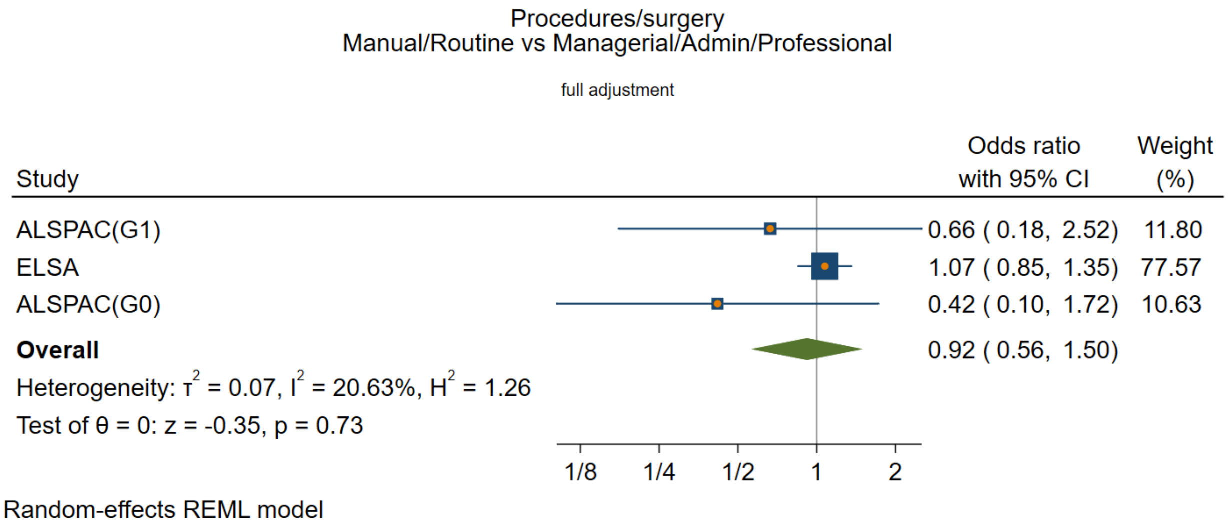

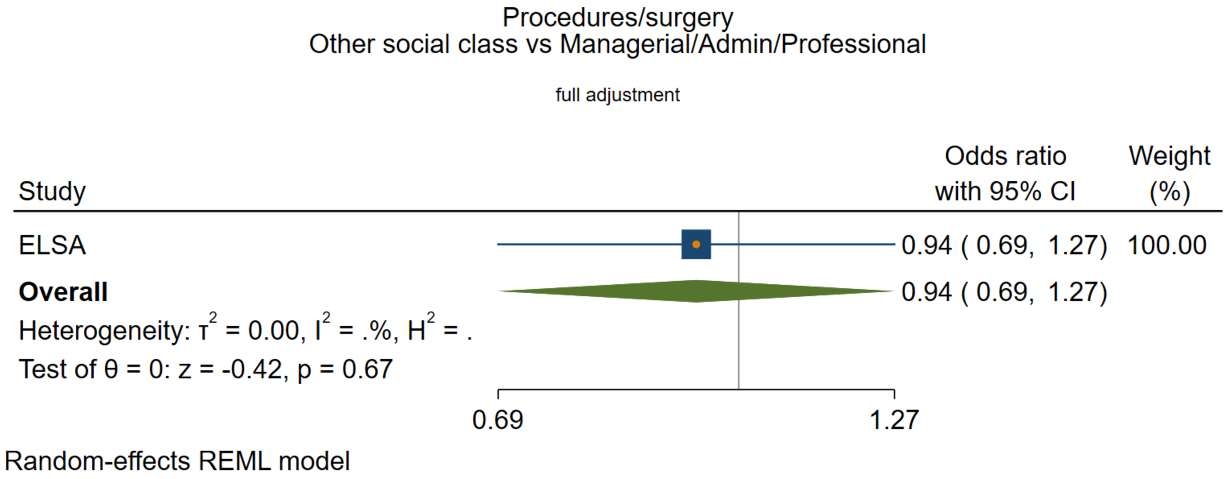

For peer review only - http://bmjopen.bmj.com/site/about/guidelines.xhtml

Education
unadjusted

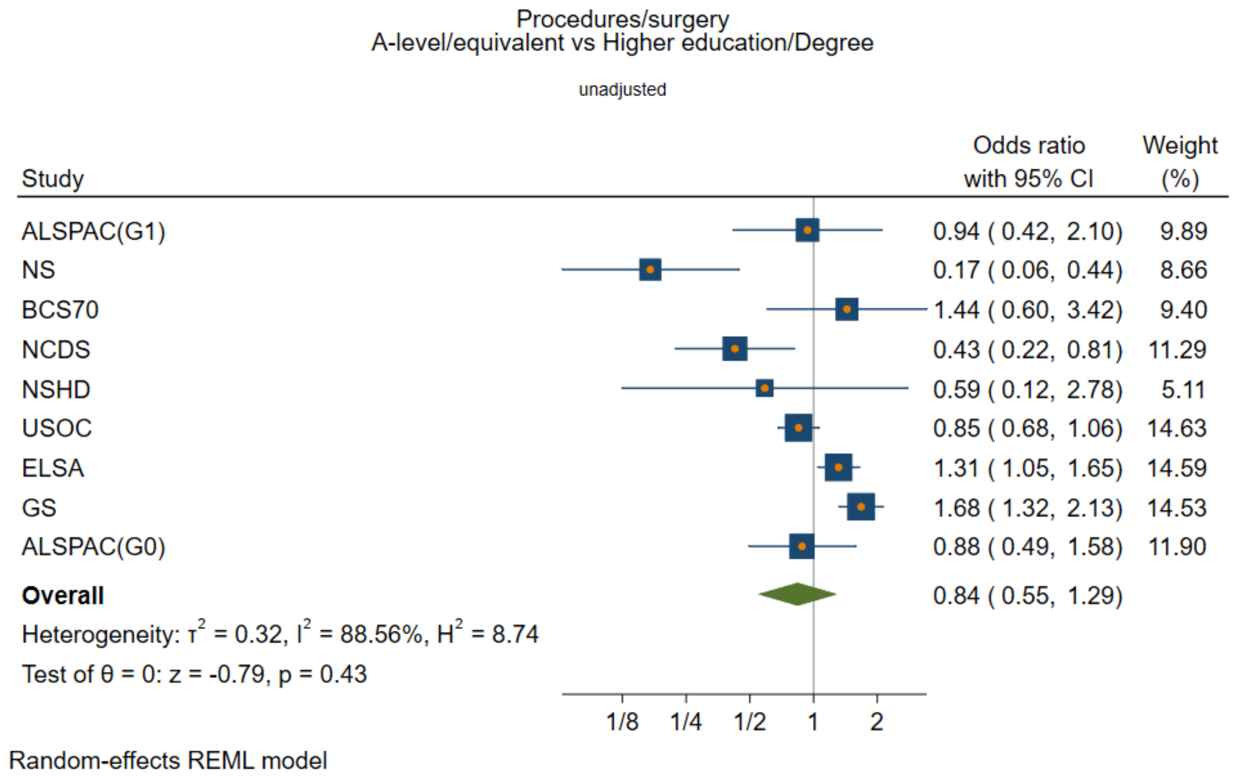

For peer review only

For peer review only - http://bmjopen.bmj.com/site/about/guidelines.xhtml

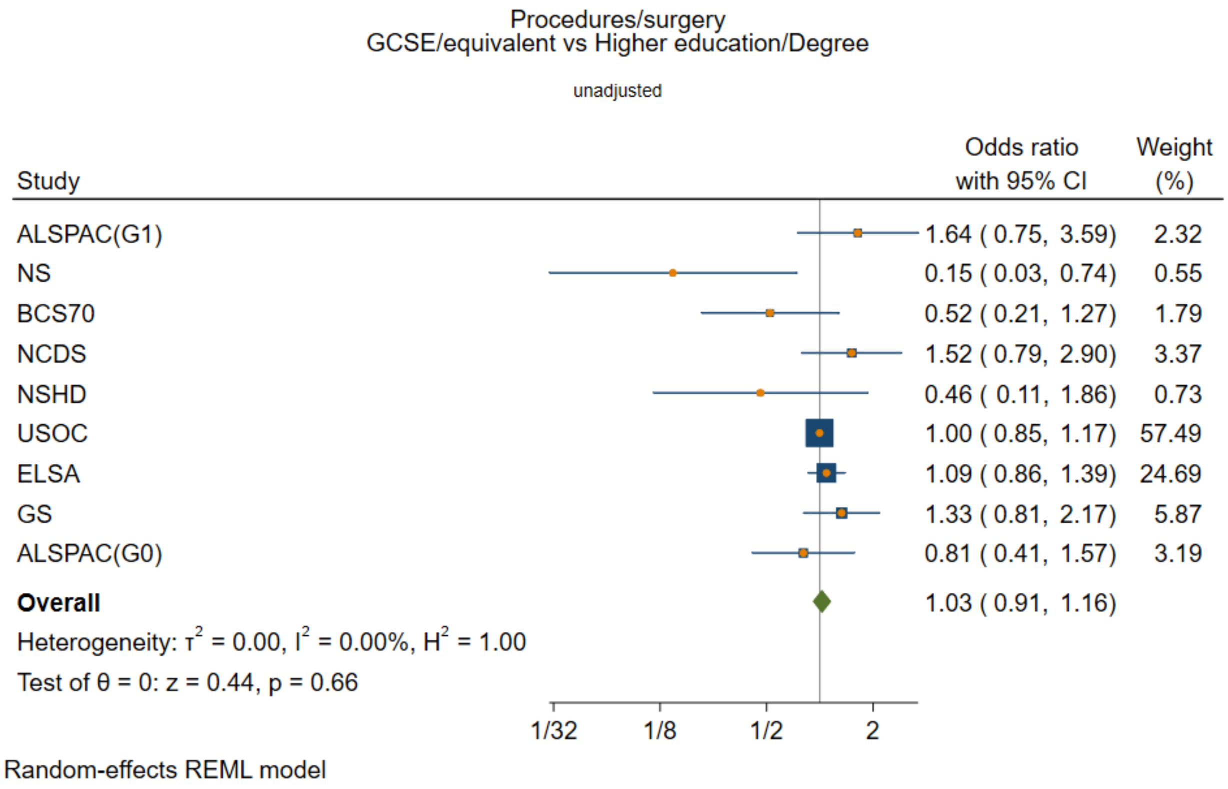

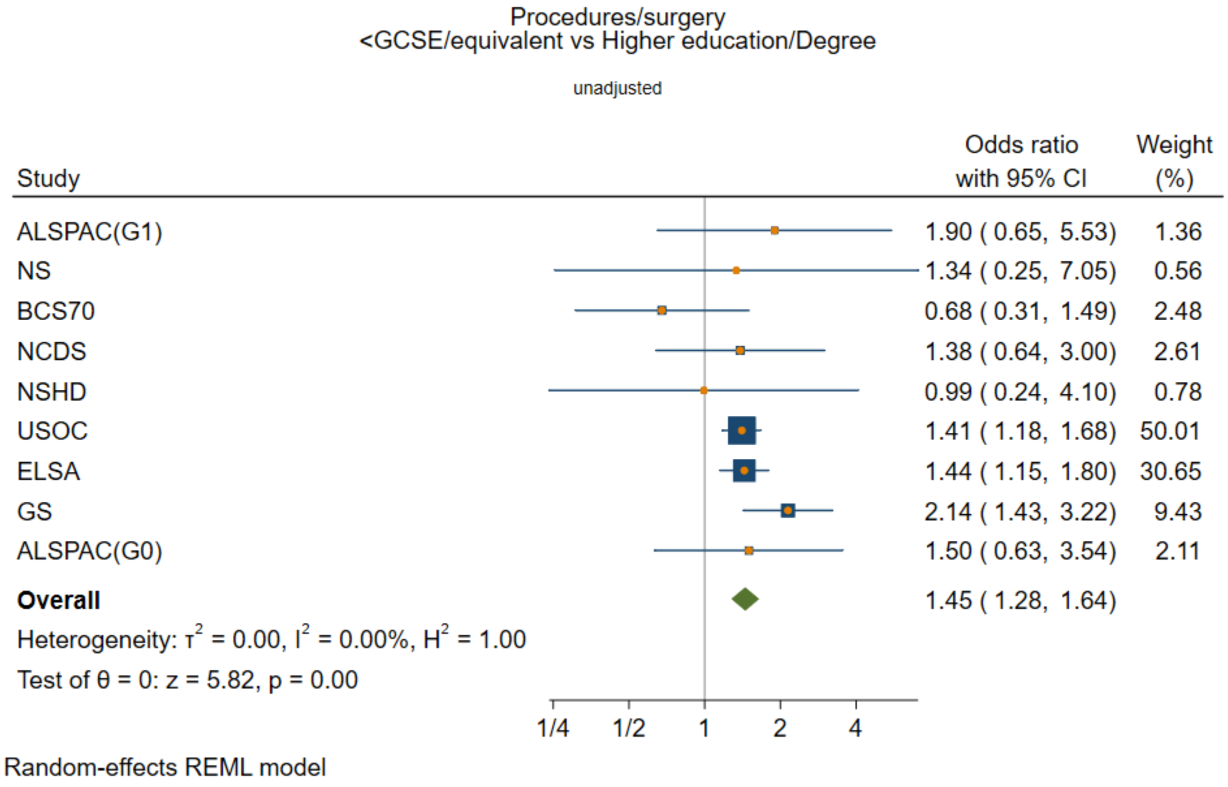

For peer review only - http://bmjopen.bmj.com/site/about/guidelines.xhtml

Basic adjustment

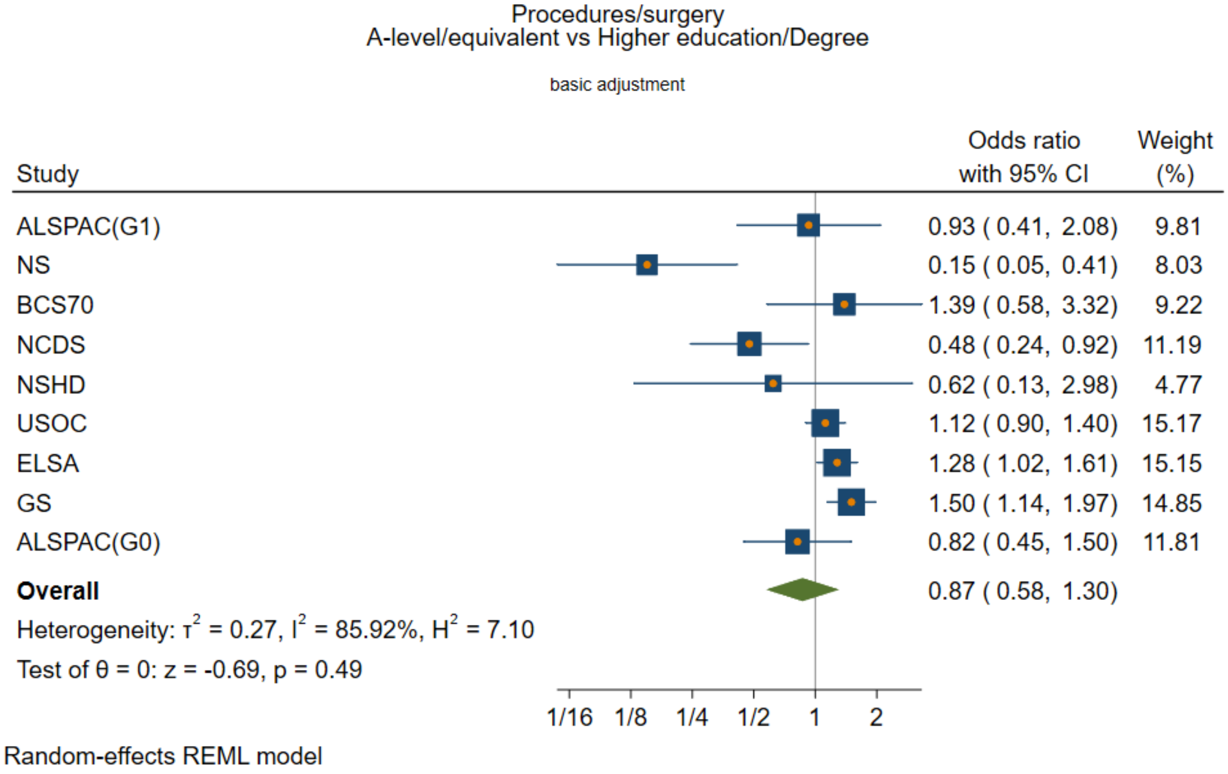

view only

For peer review only - http://bmjopen.bmj.com/site/about/guidelines.xhtml

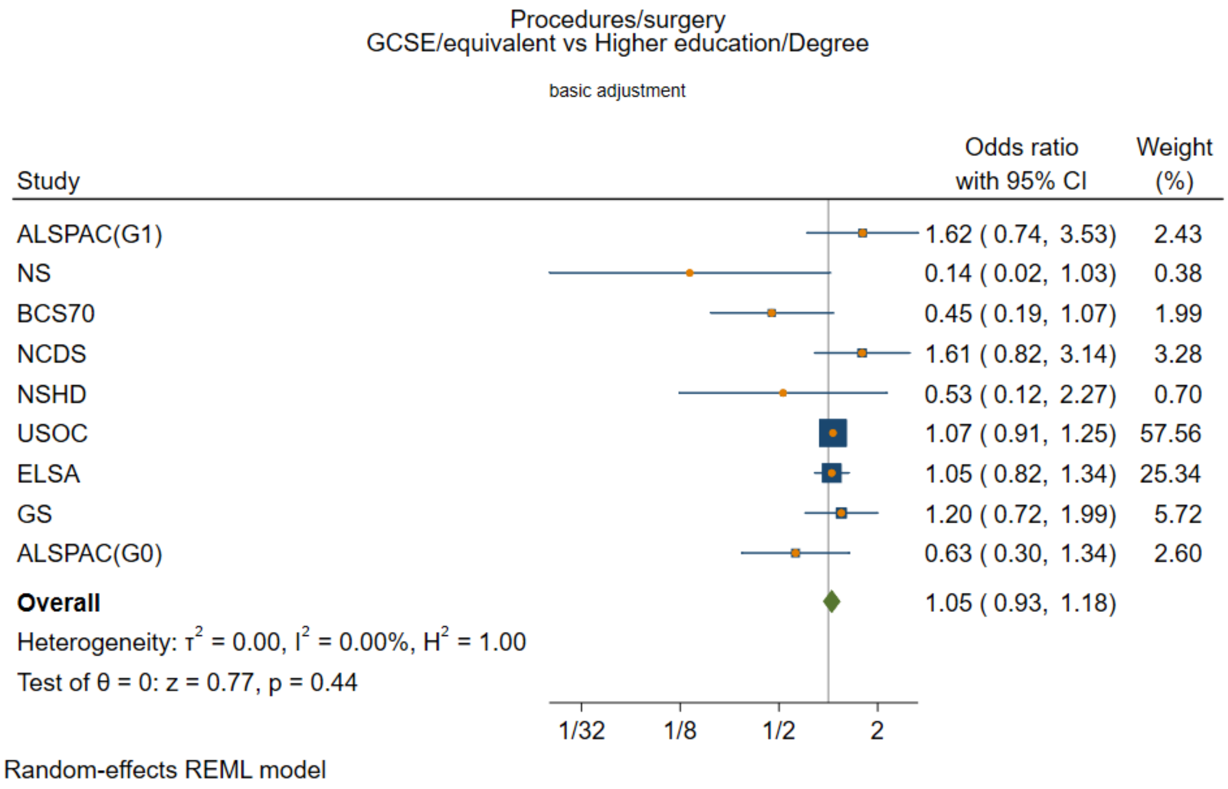

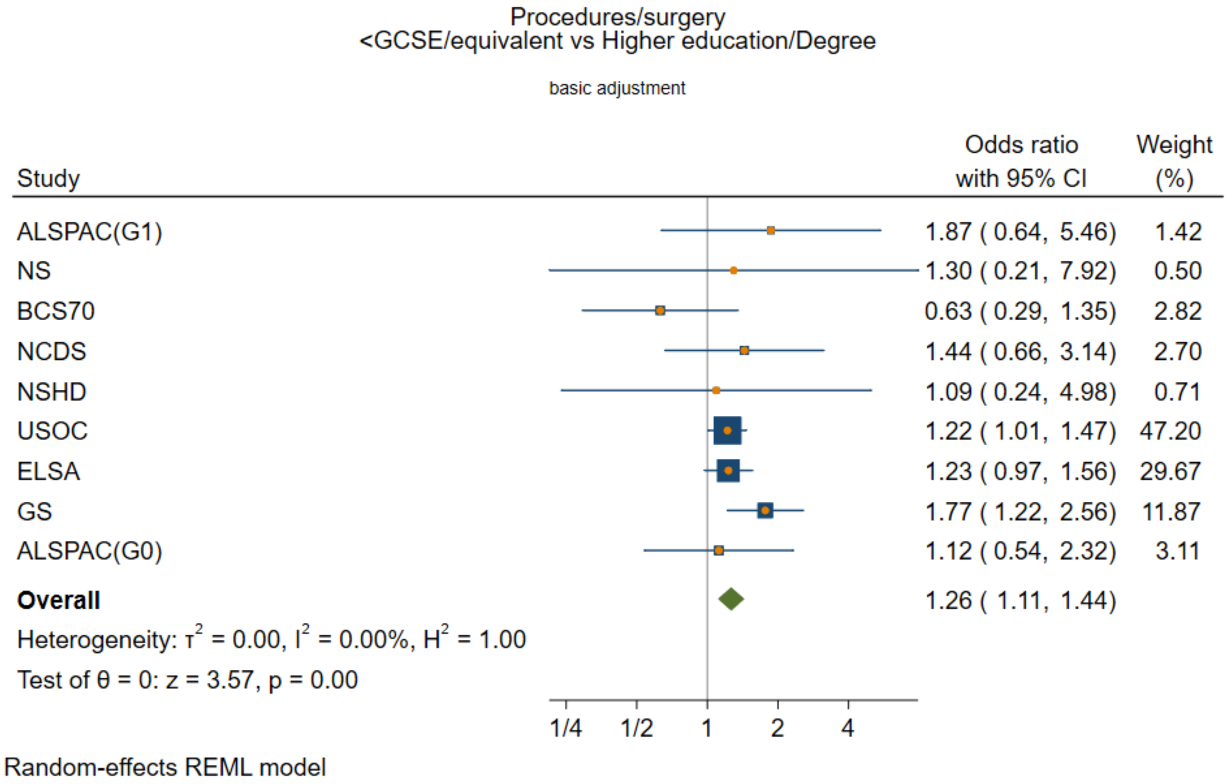

For peer review only - http://bmjopen.bmj.com/site/about/guidelines.xhtml

Full adjustment

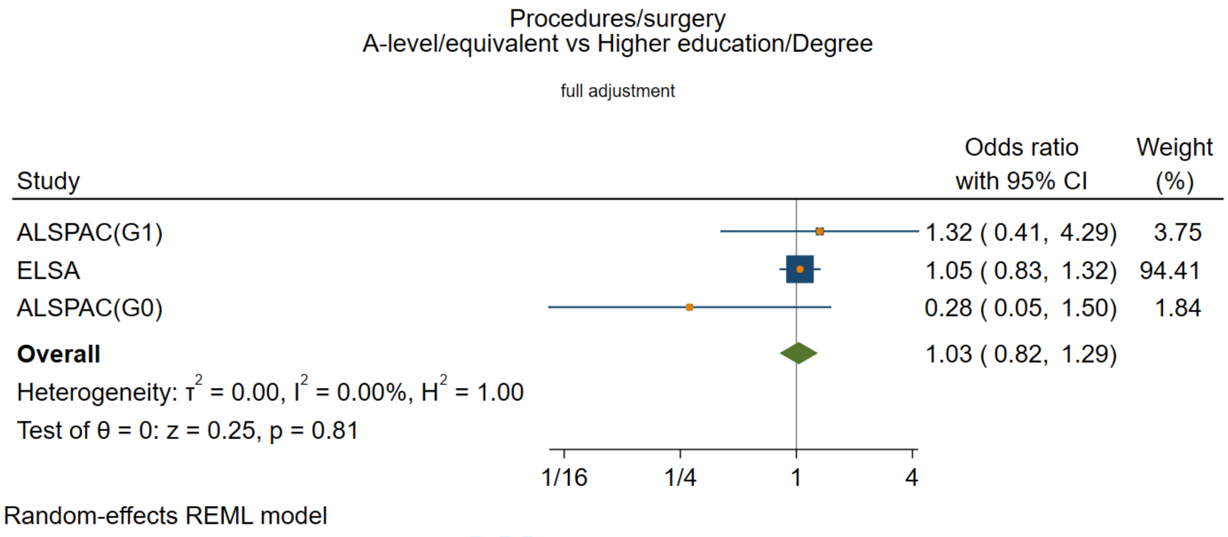

For peer review only

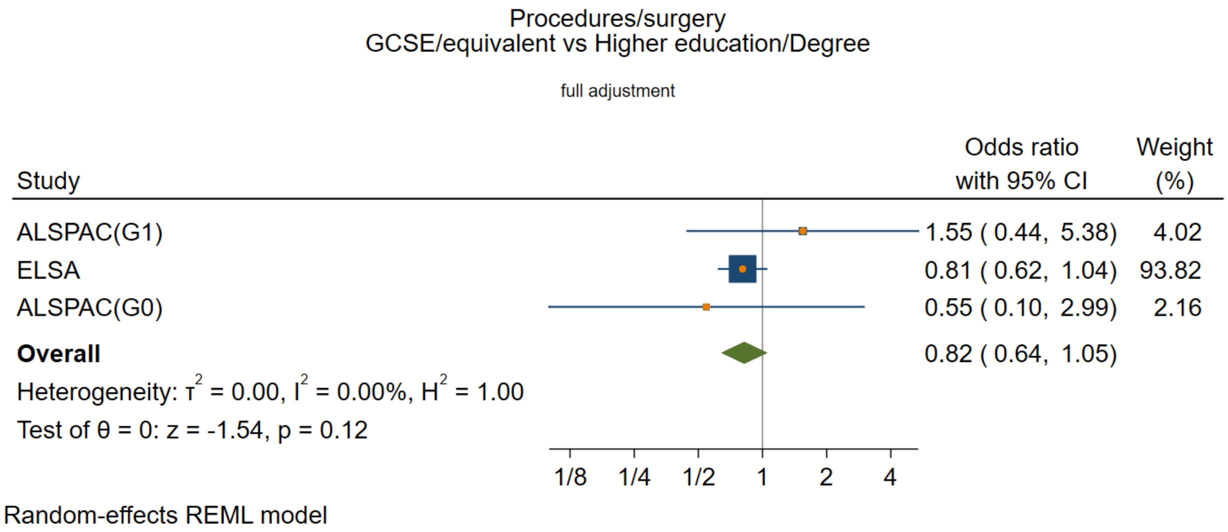

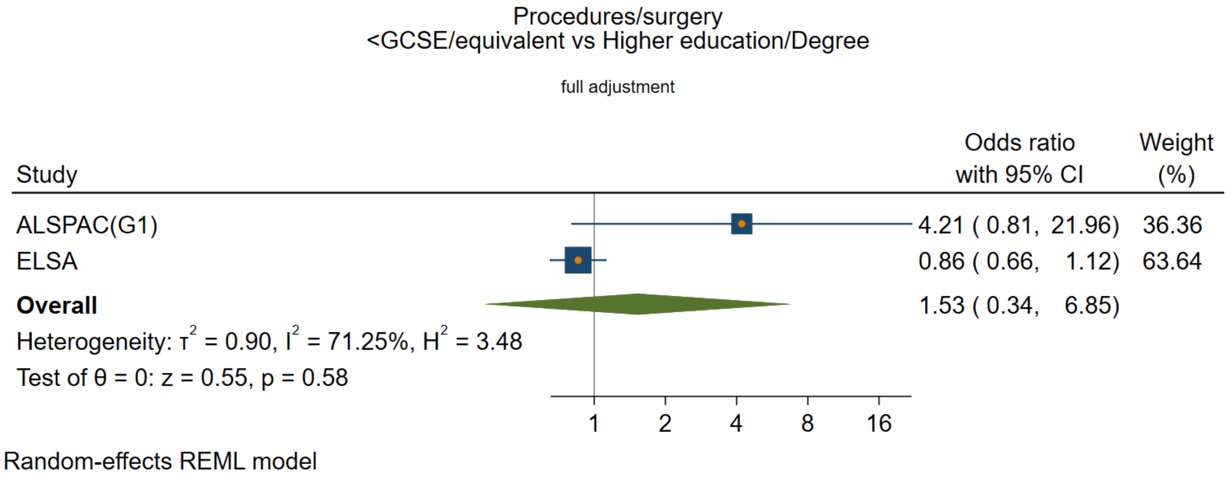

For peer review only - http://bmjopen.bmj.com/site/about/guidelines.xhtml

Age

Unadjusted

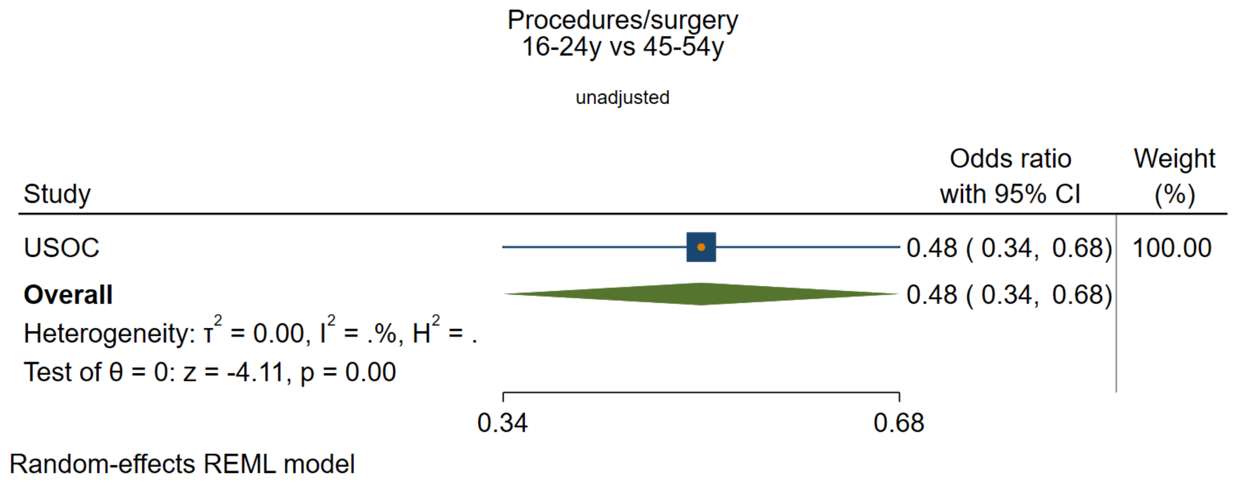

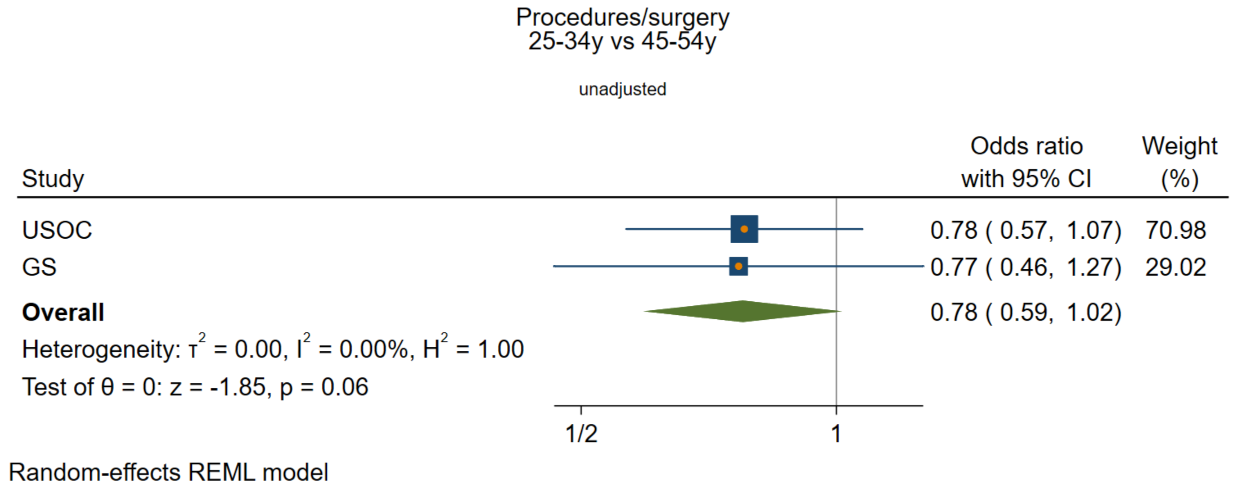

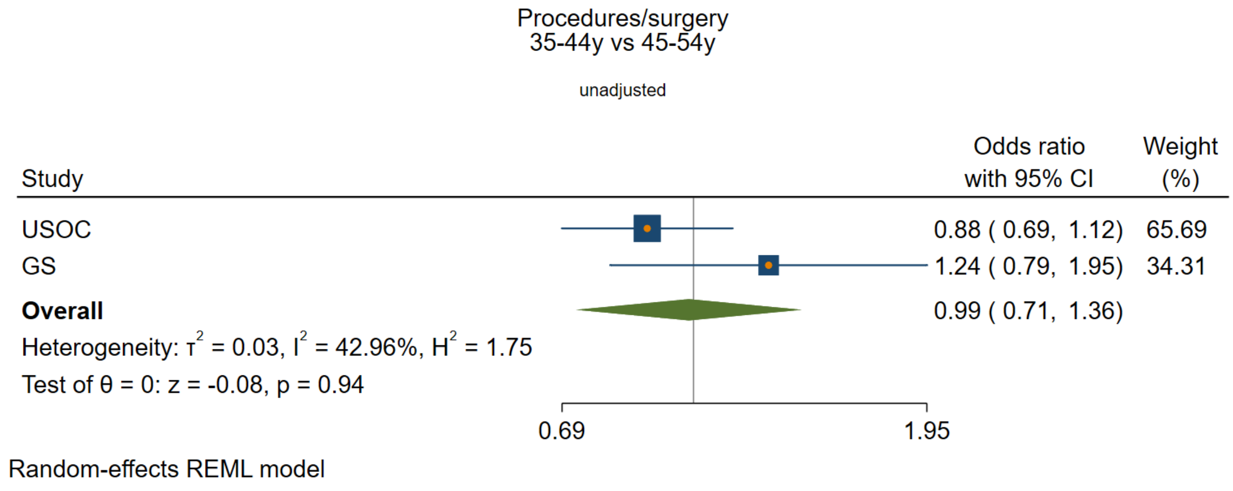

For peer review only - http://bmjopen.bmj.com/site/about/guidelines.xhtml

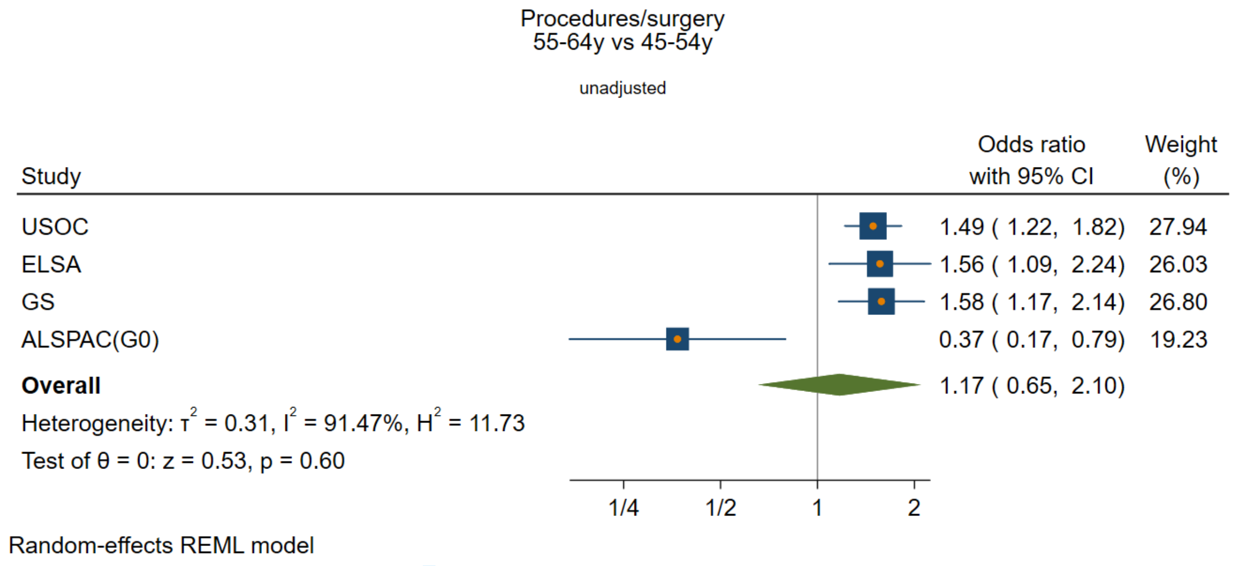

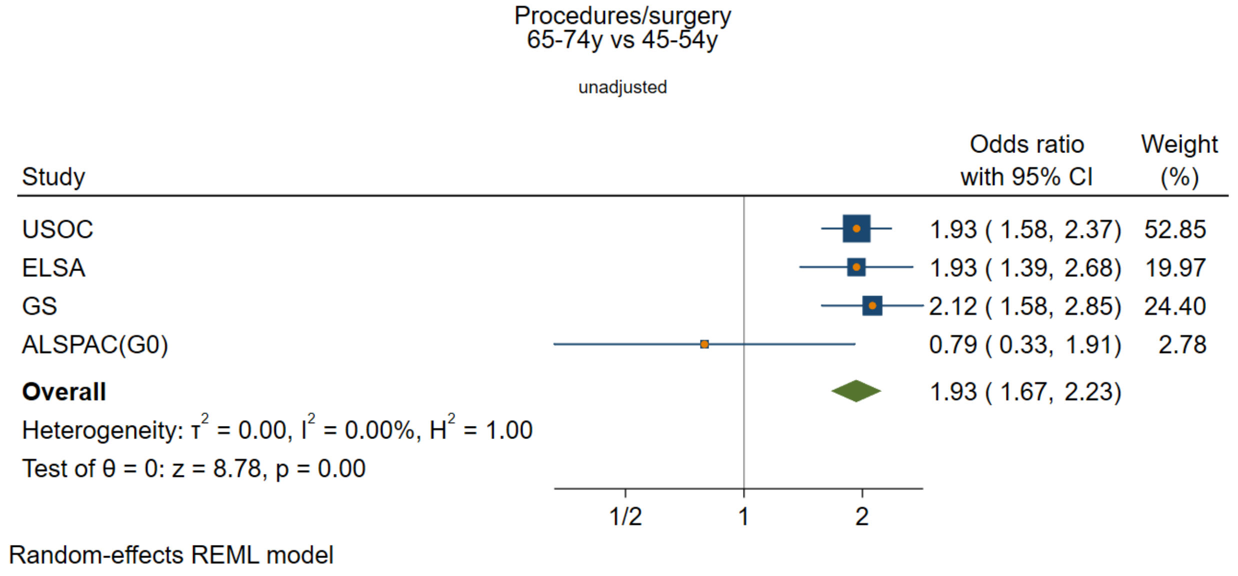

For peer review only - http://bmjopen.bmj.com/site/about/guidelines.xhtml

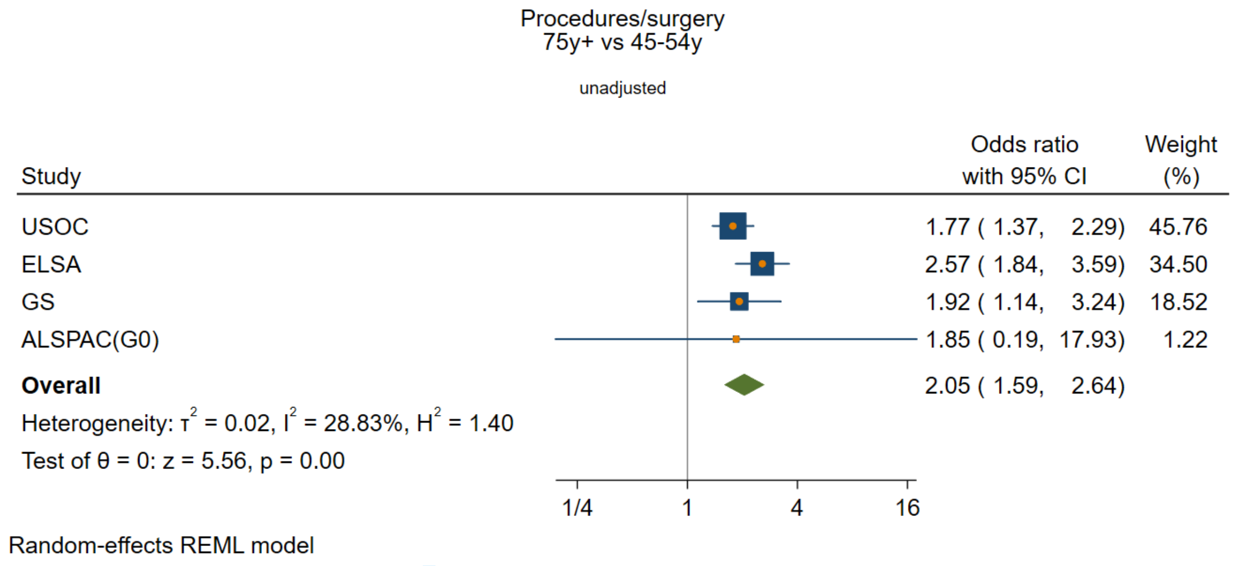

peer review only

Basic adjustment

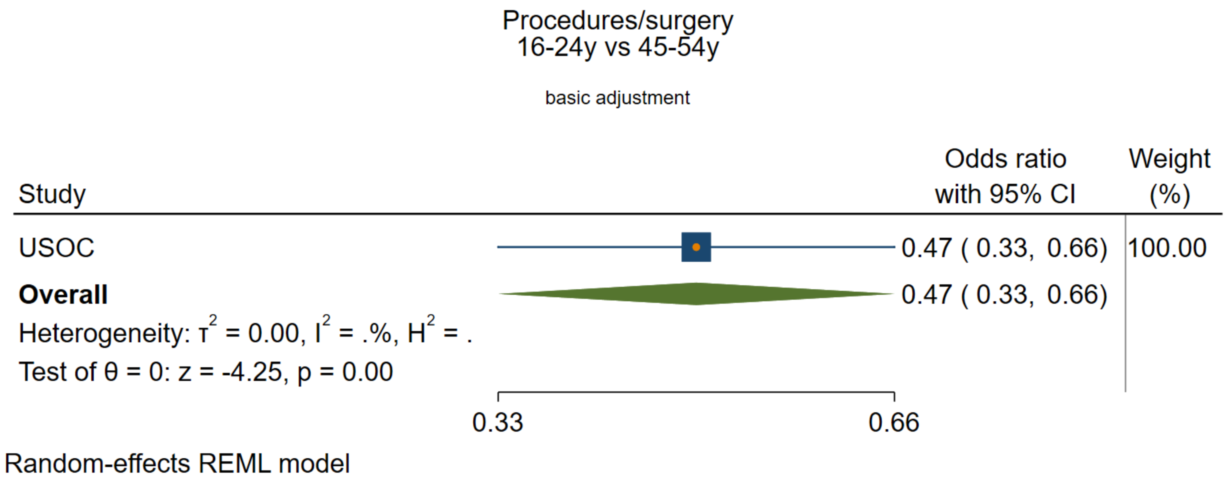

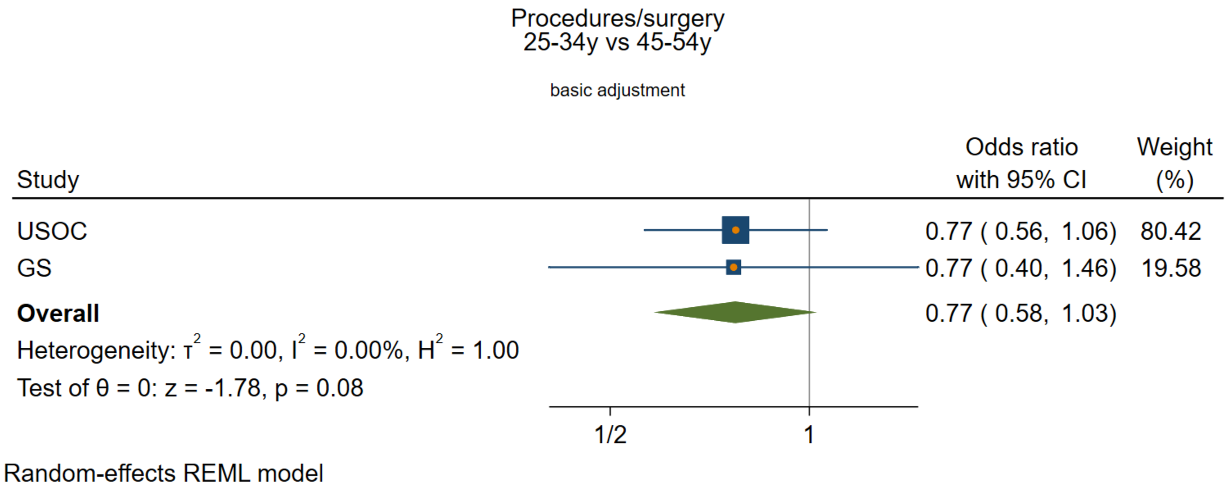

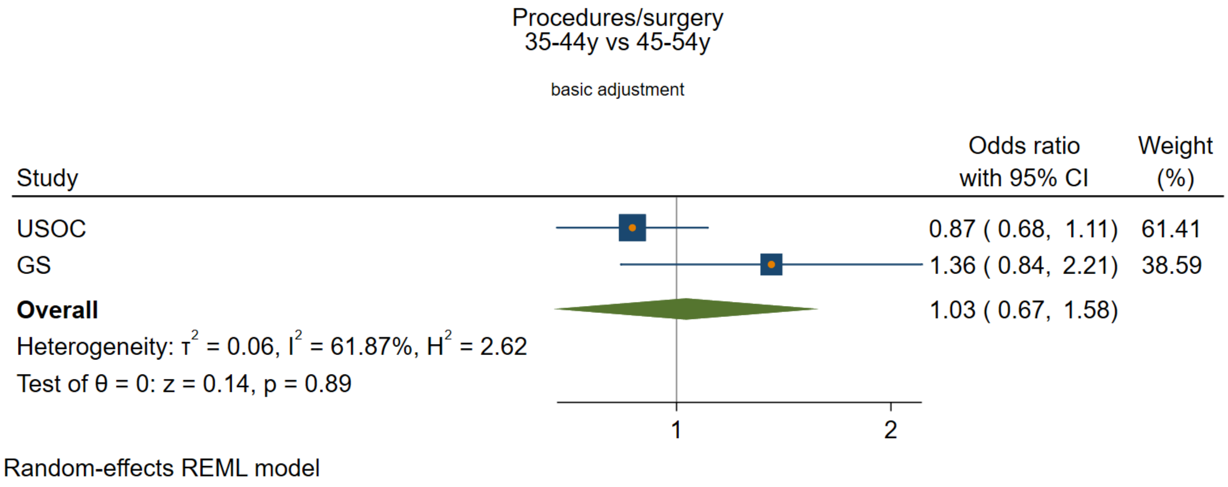

For peer review only - http://bmjopen.bmj.com/site/about/guidelines.xhtml

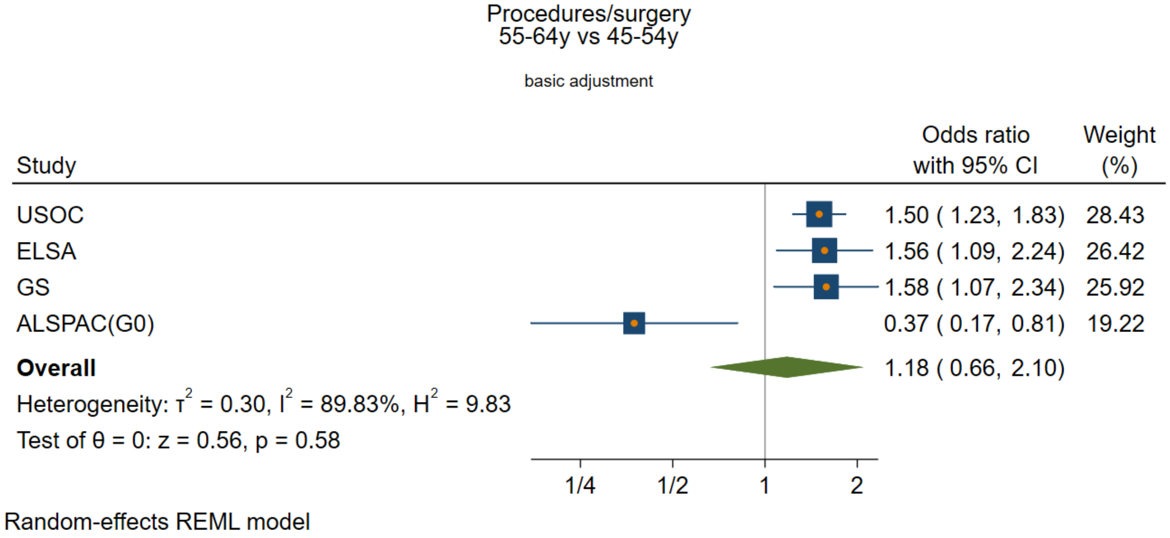

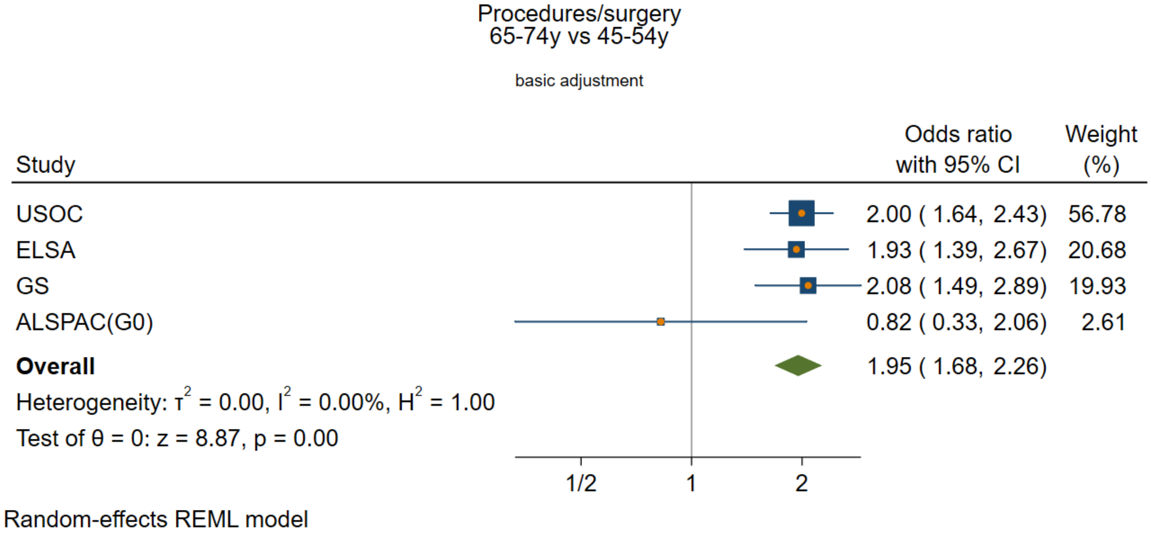

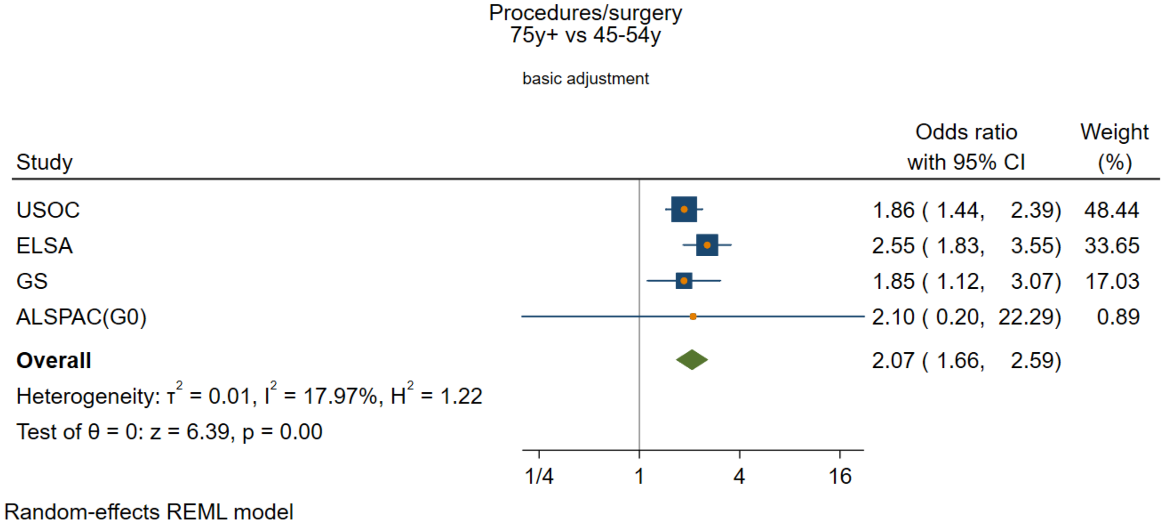

For peer review only - http://bmjopen.bmj.com/site/about/guidelines.xhtml

Full adjustment

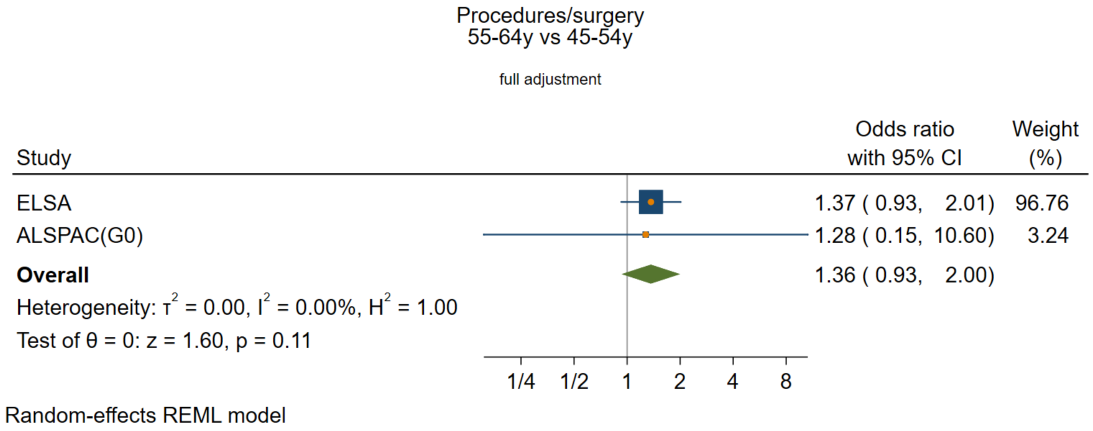

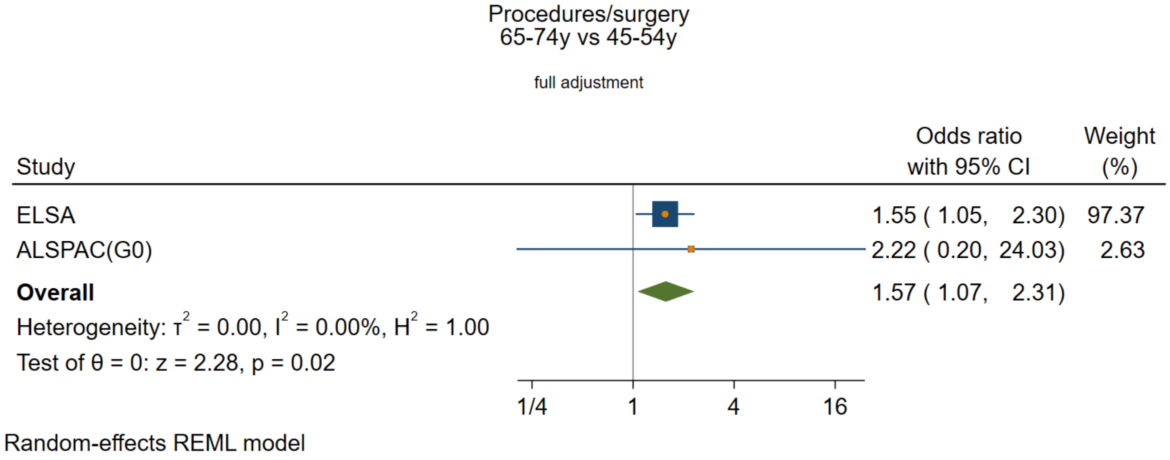

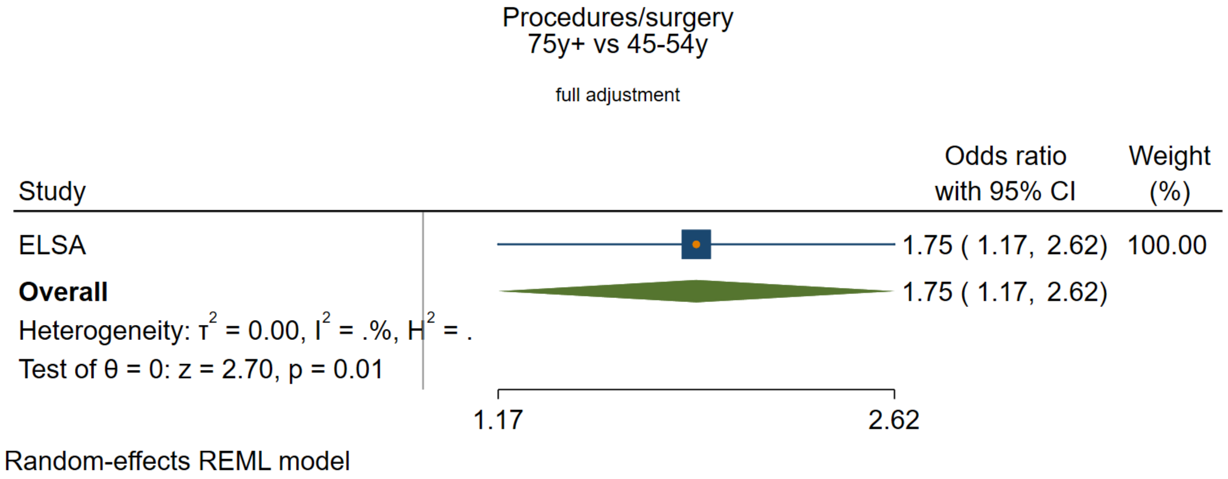

For peer review only - http://bmjopen.bmj.com/site/about/guidelines.xhtml

Ethnicity

Unadjusted

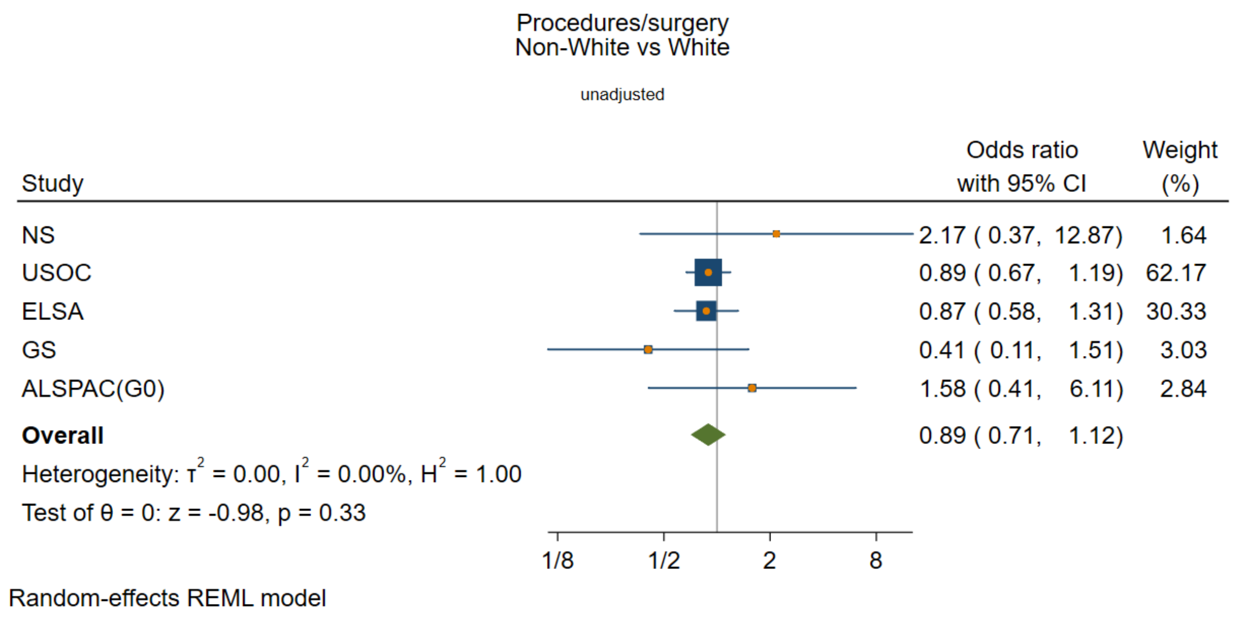

view only

For peer review only - http://bmjopen.bmj.com/site/about/guidelines.xhtml

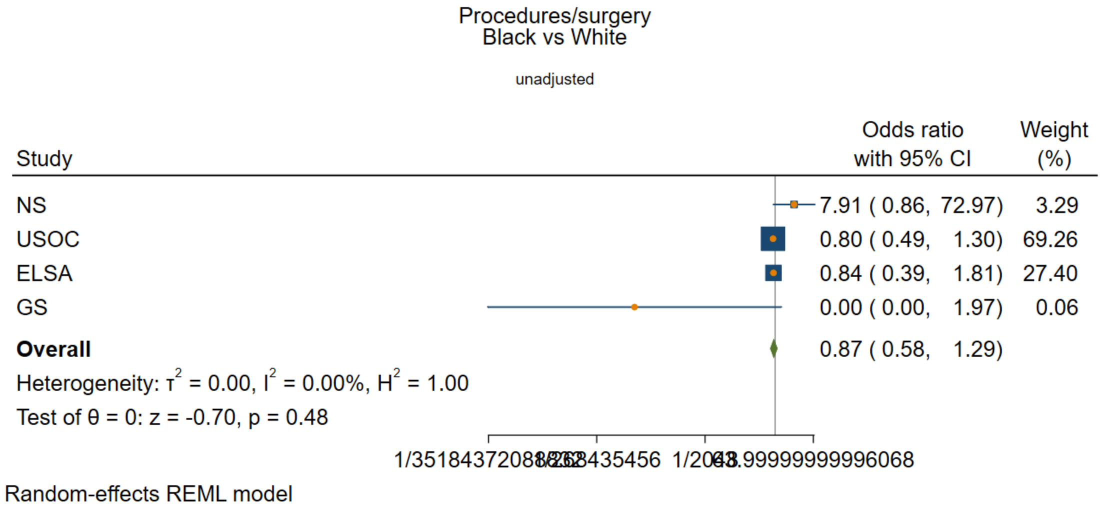

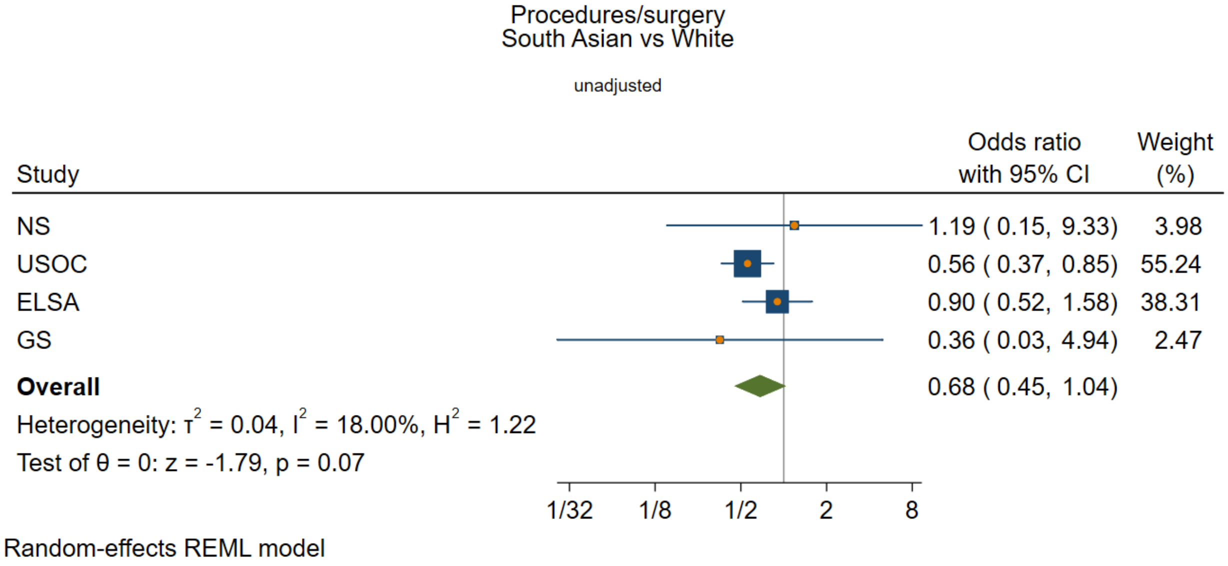

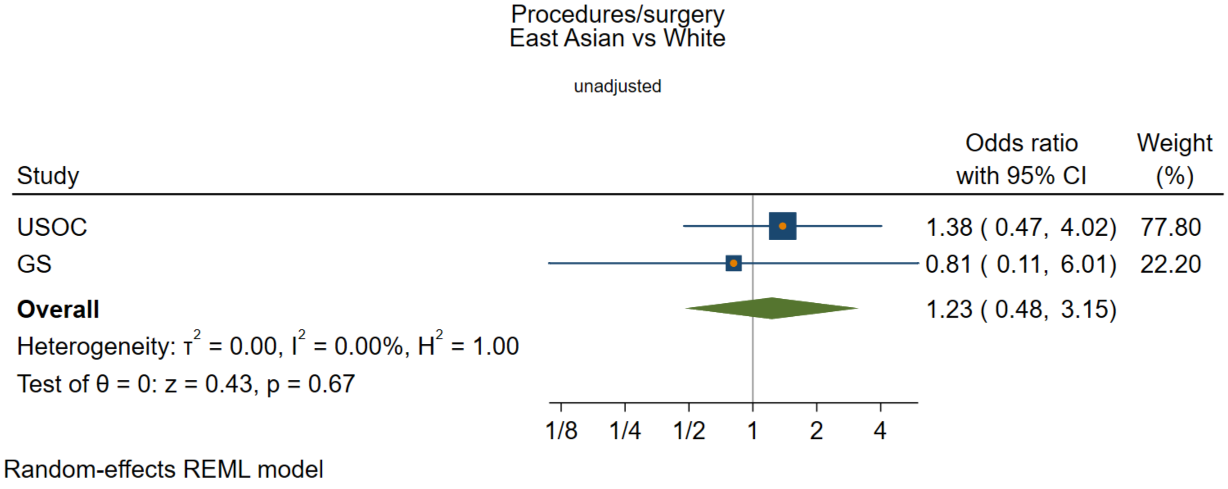

For peer review only - http://bmjopen.bmj.com/site/about/guidelines.xhtml

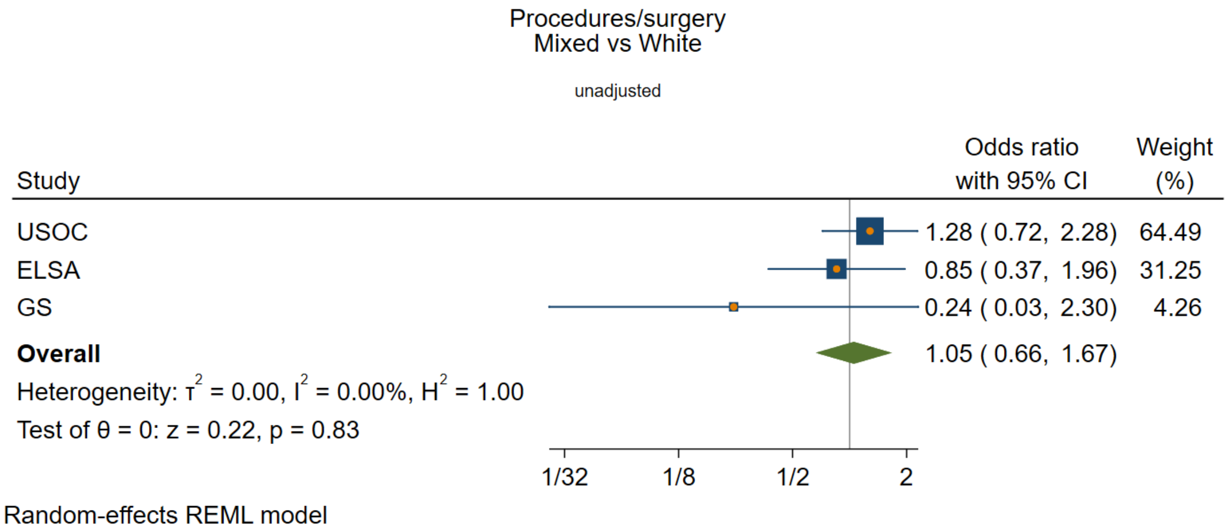

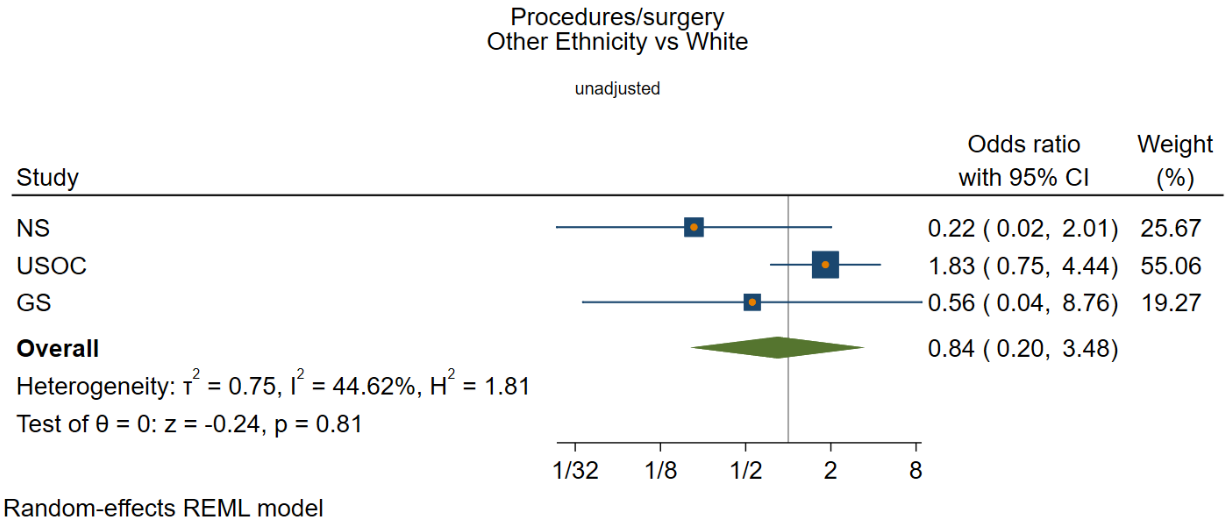

For peer review only - http://bmjopen.bmj.com/site/about/guidelines.xhtml

Basic adjustment

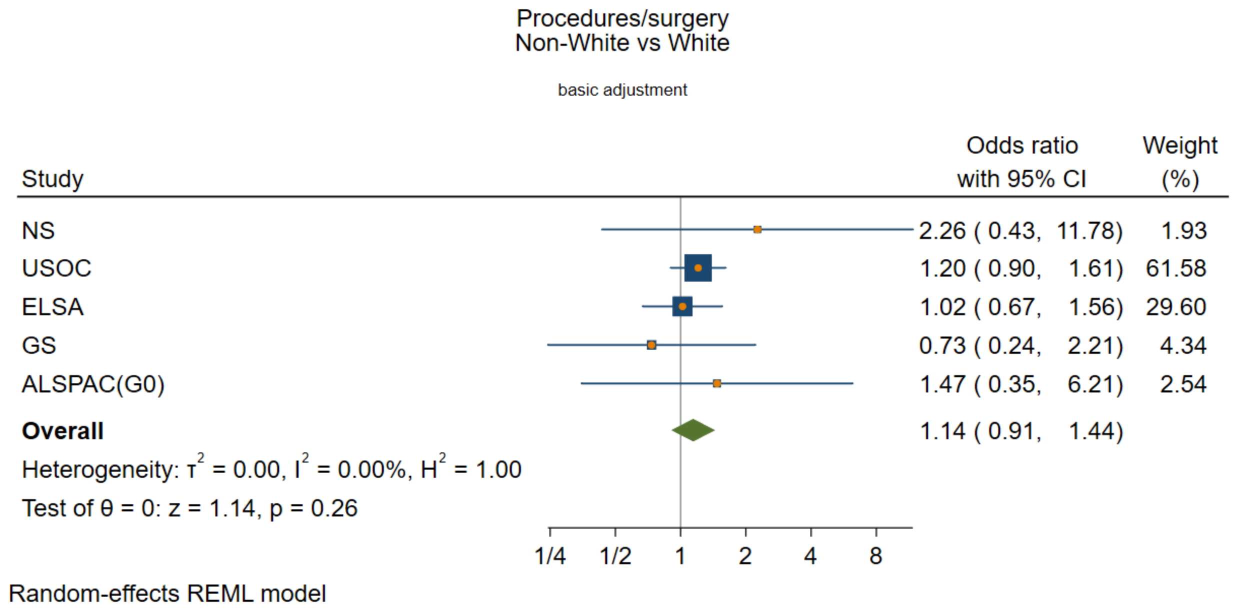

For peer review only - http://bmjopen.bmj.com/site/about/guidelines.xhtml

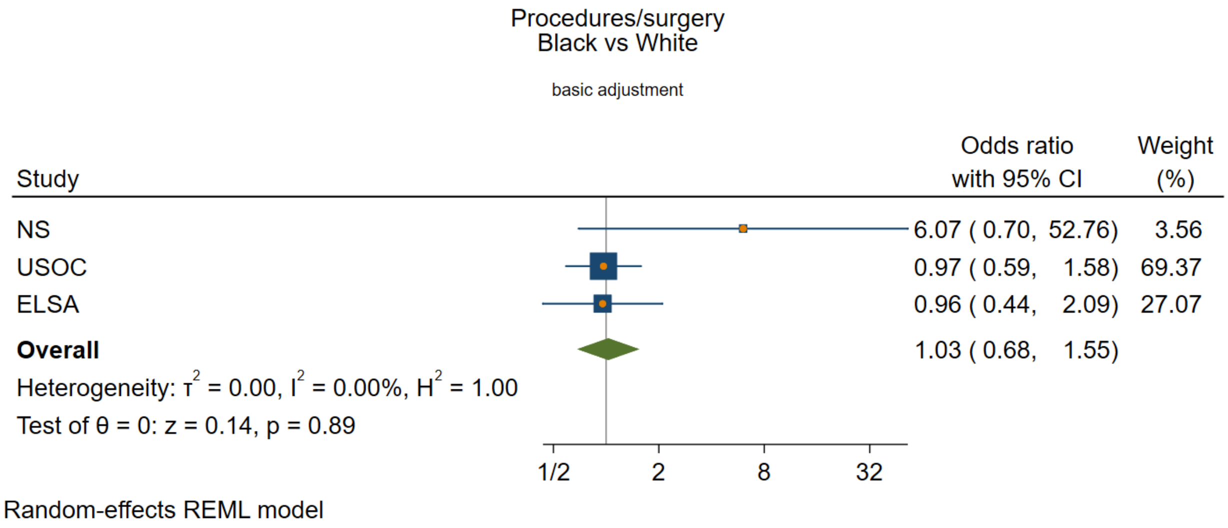

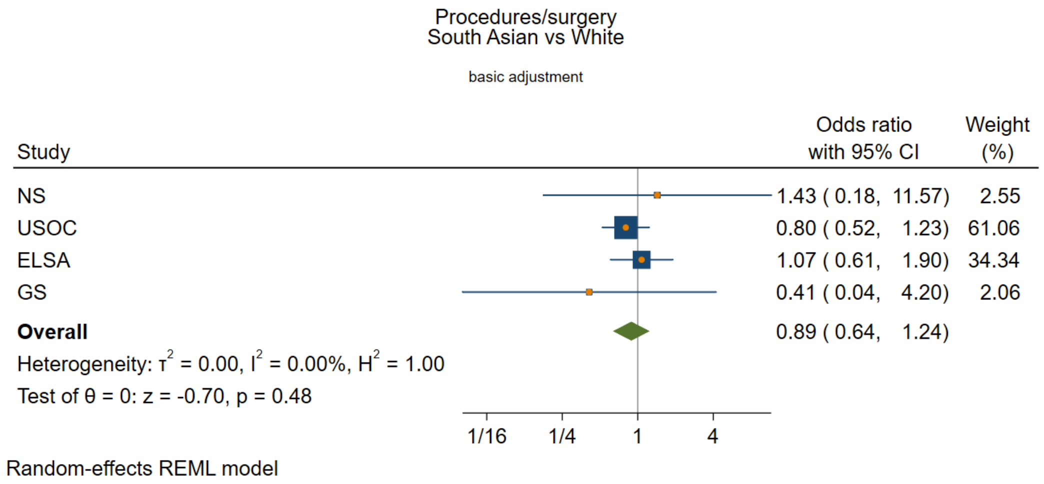

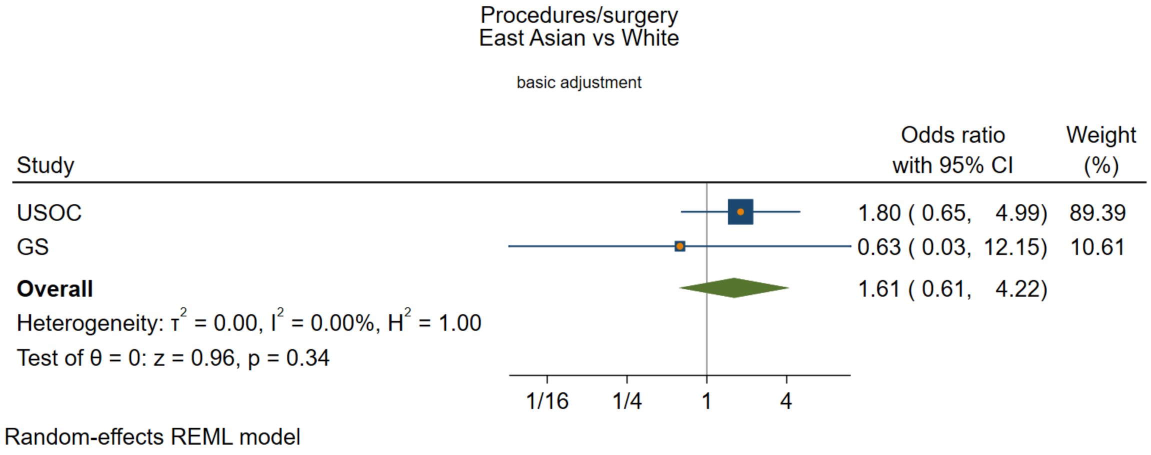

For peer review only - http://bmjopen.bmj.com/site/about/guidelines.xhtml

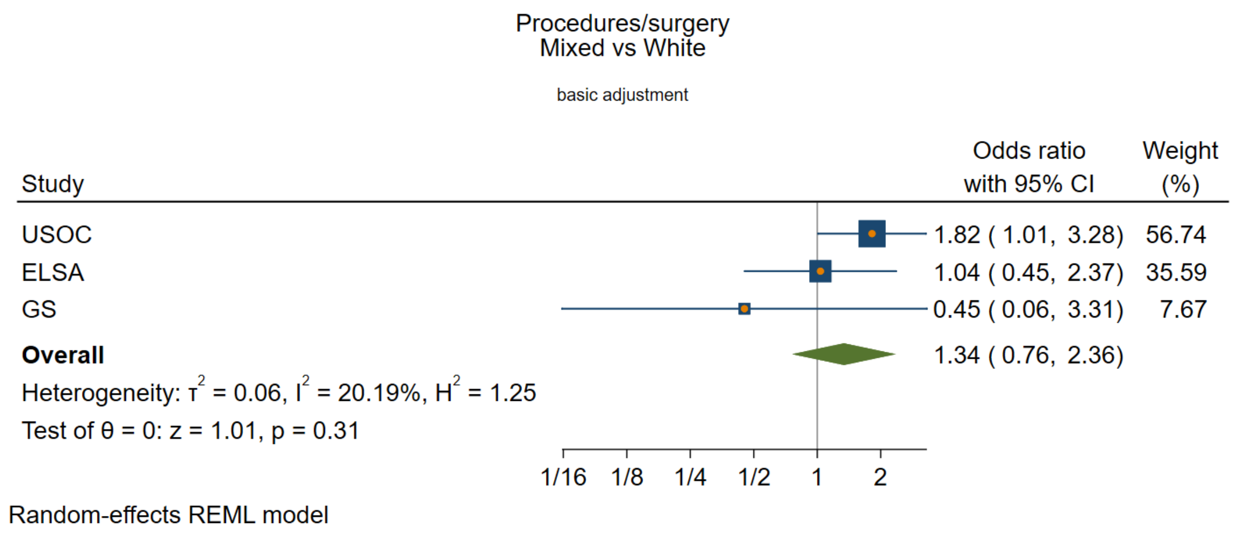

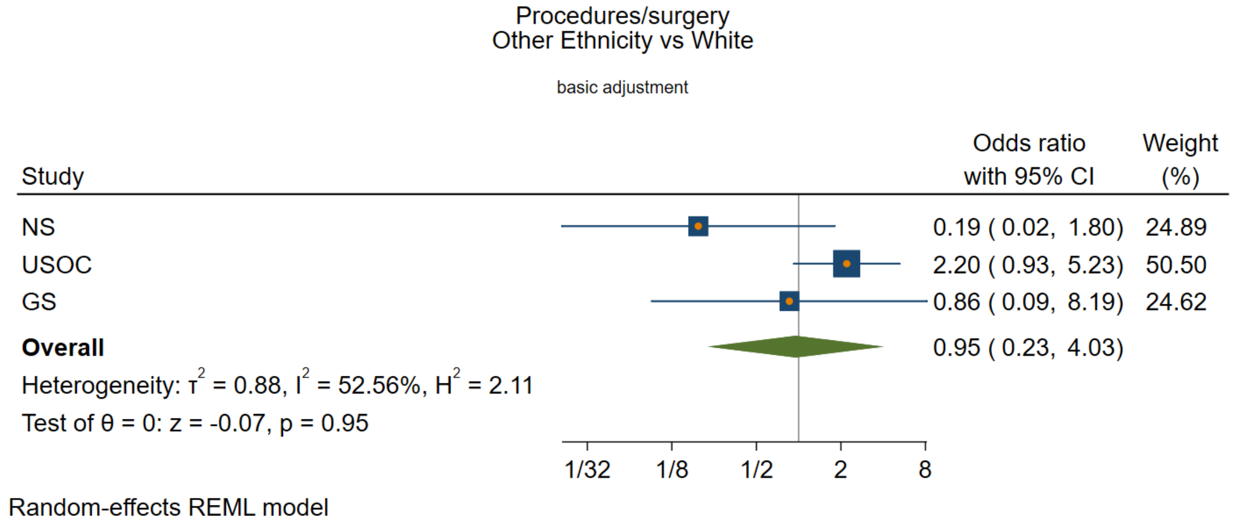

For peer review only - http://bmjopen.bmj.com/site/about/guidelines.xhtml

Full adjustment

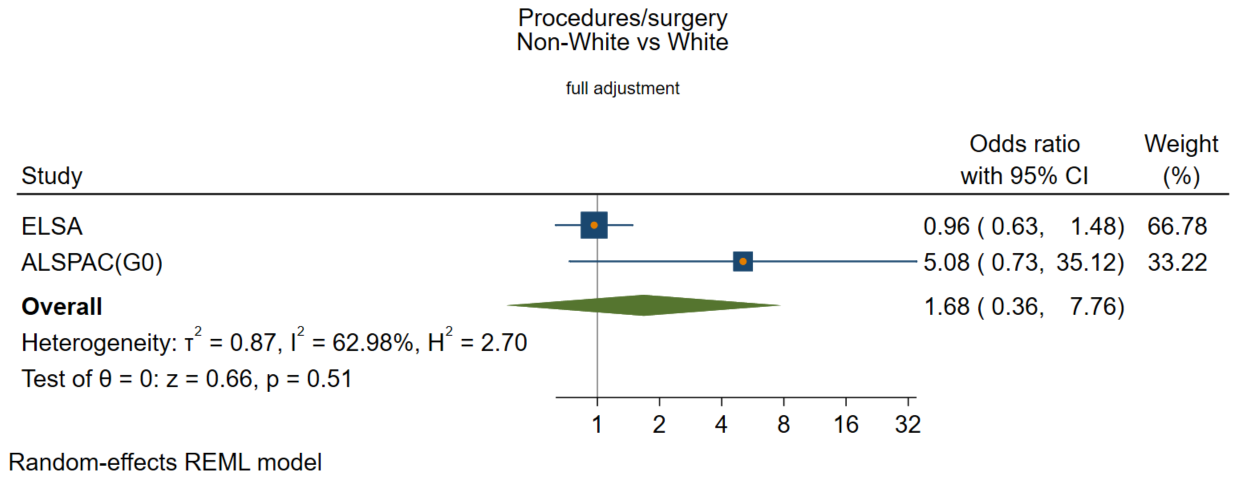

For peer review only - http://bmjopen.bmj.com/site/about/guidelines.xhtml

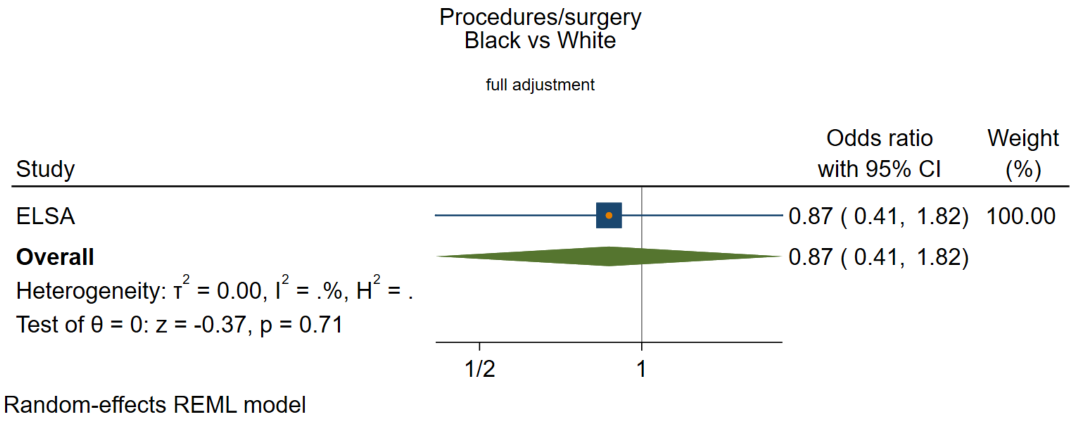

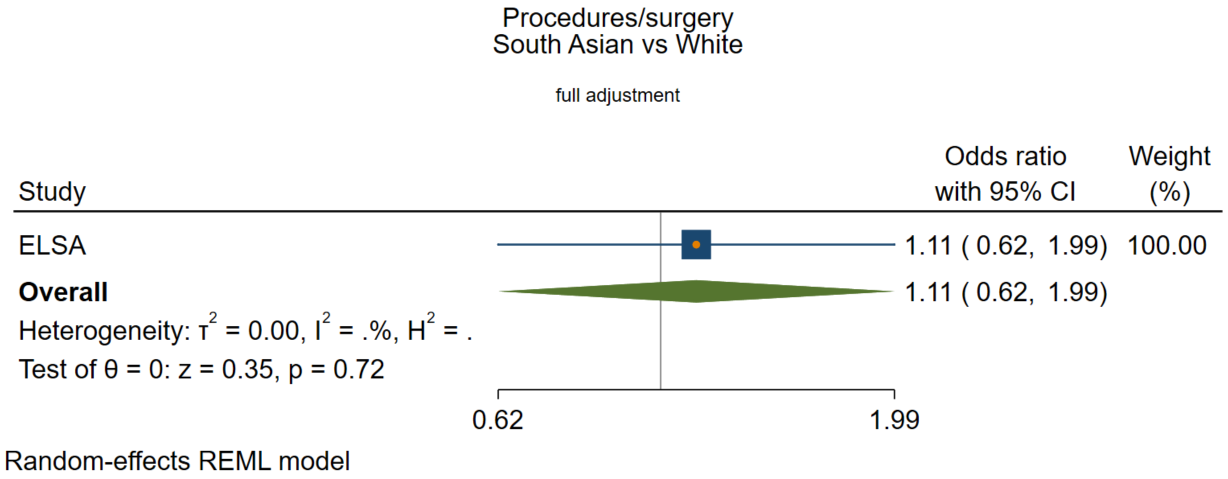

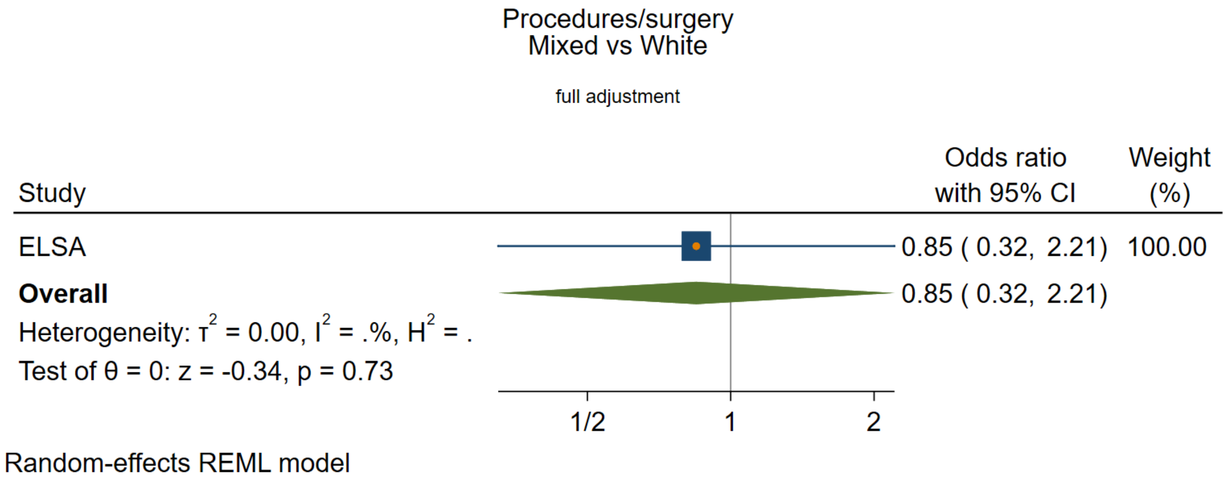

For peer review only - http://bmjopen.bmj.com/site/about/guidelines.xhtml

Any healthcare disruption stratified by shielding status

Sex

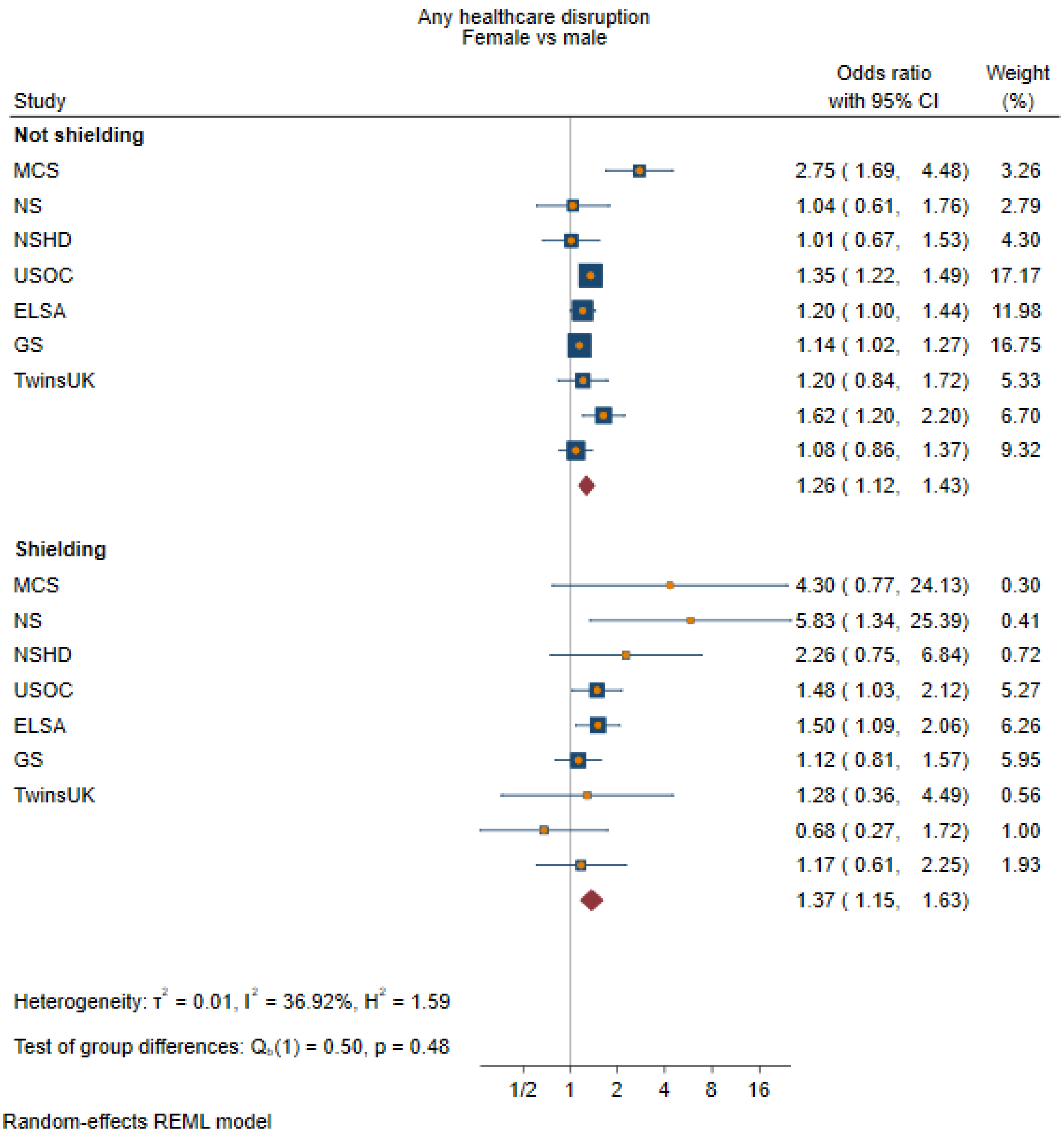

Occupational class

For peer review only

For peer review only - http://bmjopen.bmj.com/site/about/guidelines.xhtml

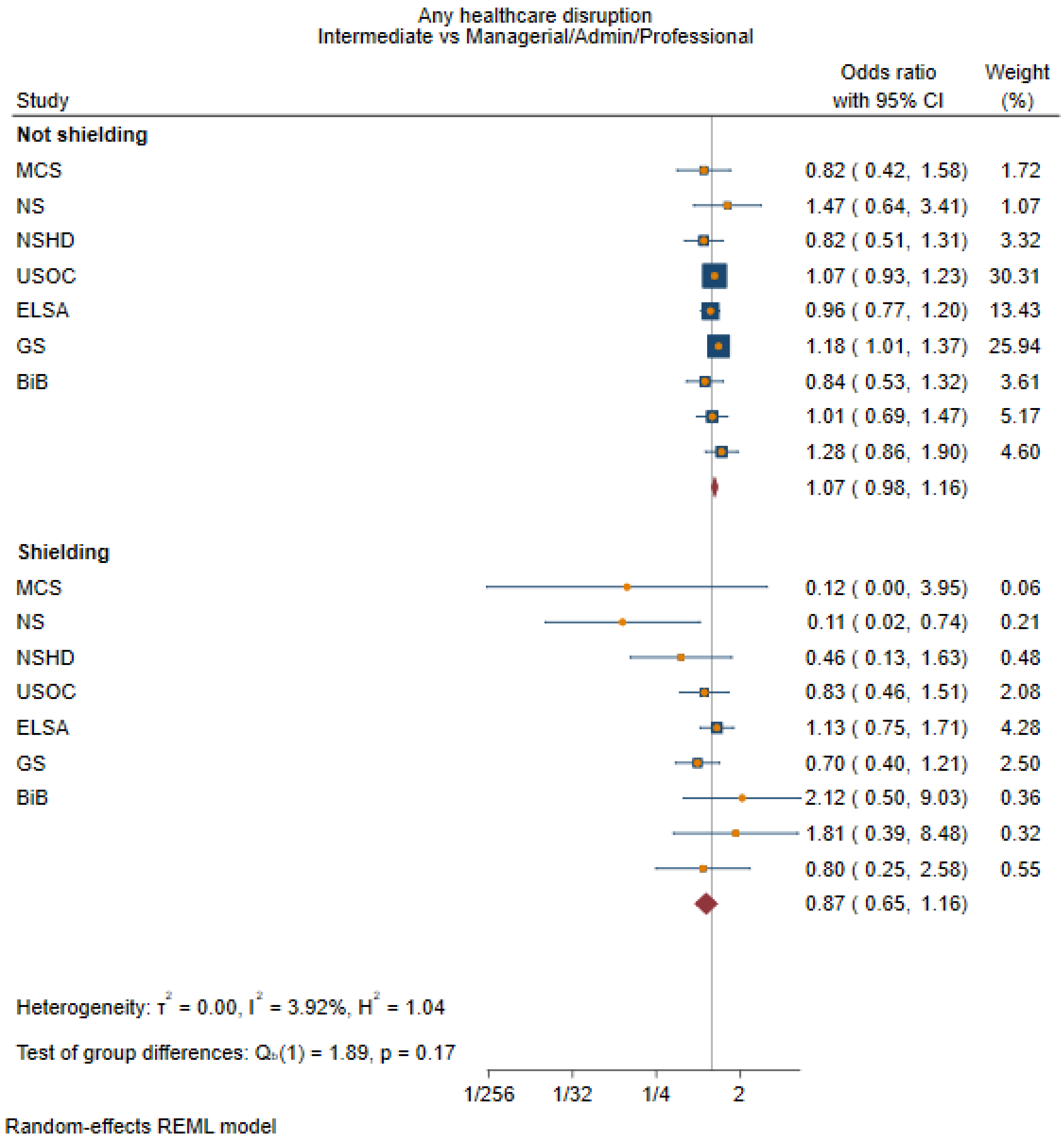
Any healthcare disruption
Intermediate vs Managerial/Admin/Professional
Study
Odds ratio with 95% CI
Weight (%)
Not shielding
MCS 0.82 (0.42, 1.58) 1.72
NS 1.47 (0.64, 3.41) 1.07
NSHD 0.82 (0.51, 1.31) 3.32
USOC 1.07 (0.93, 1.23) 30.31
ELSA 0.96 (0.77, 1.20) 13.43
GS 1.18 (1.01, 1.37) 25.94
BiB 0.84 (0.53, 1.32) 3.61
1.01 (0.69, 1.47) 5.17
1.28 (0.86, 1.90) 4.60
1.07 (0.98, 1.16)
Shielding
MCS 0.12 (0.00, 3.95) 0.06
NS 0.11 (0.02, 0.74) 0.21
NSHD 0.46 (0.13, 1.63) 0.48
USOC 0.83 (0.46, 1.51) 2.08
ELSA 1.13 (0.75, 1.71) 4.28
GS 0.70 (0.40, 1.21) 2.50
BiB 2.12 (0.50, 9.03) 0.36
1.81 (0.39, 8.48) 0.32
0.80 (0.25, 2.58) 0.55
0.87 (0.65, 1.16)
Heterogeneity: τ² = 0.00, I² = 3.92%, H² = 1.04
Test of group differences: Qb(1) = 1.89, p = 0.17
1/256
1/32
1/4
2
Random-effects REML model

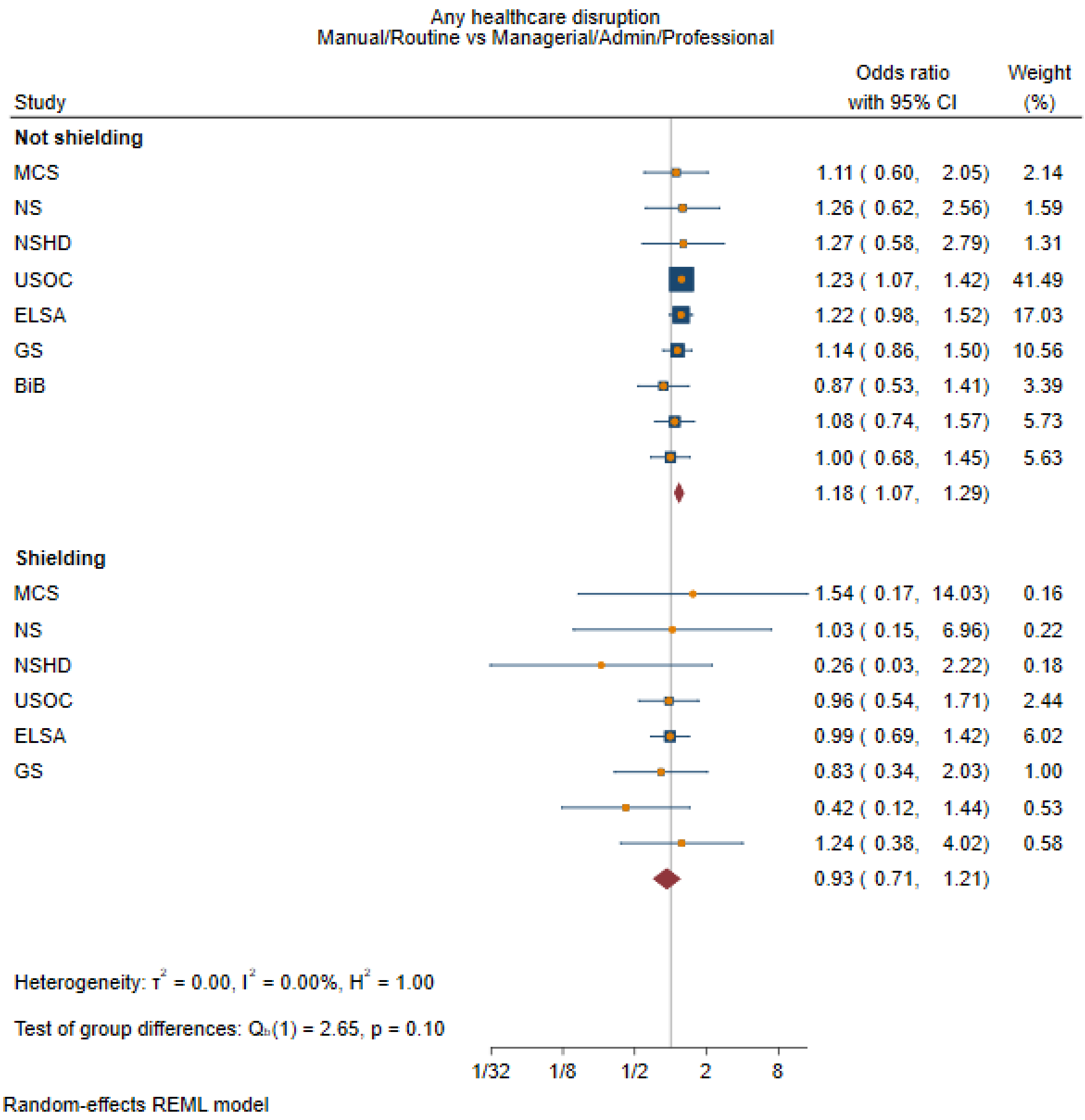

For peer review only - http://bmjopen.bmj.com/site/about/guidelines.xhtml

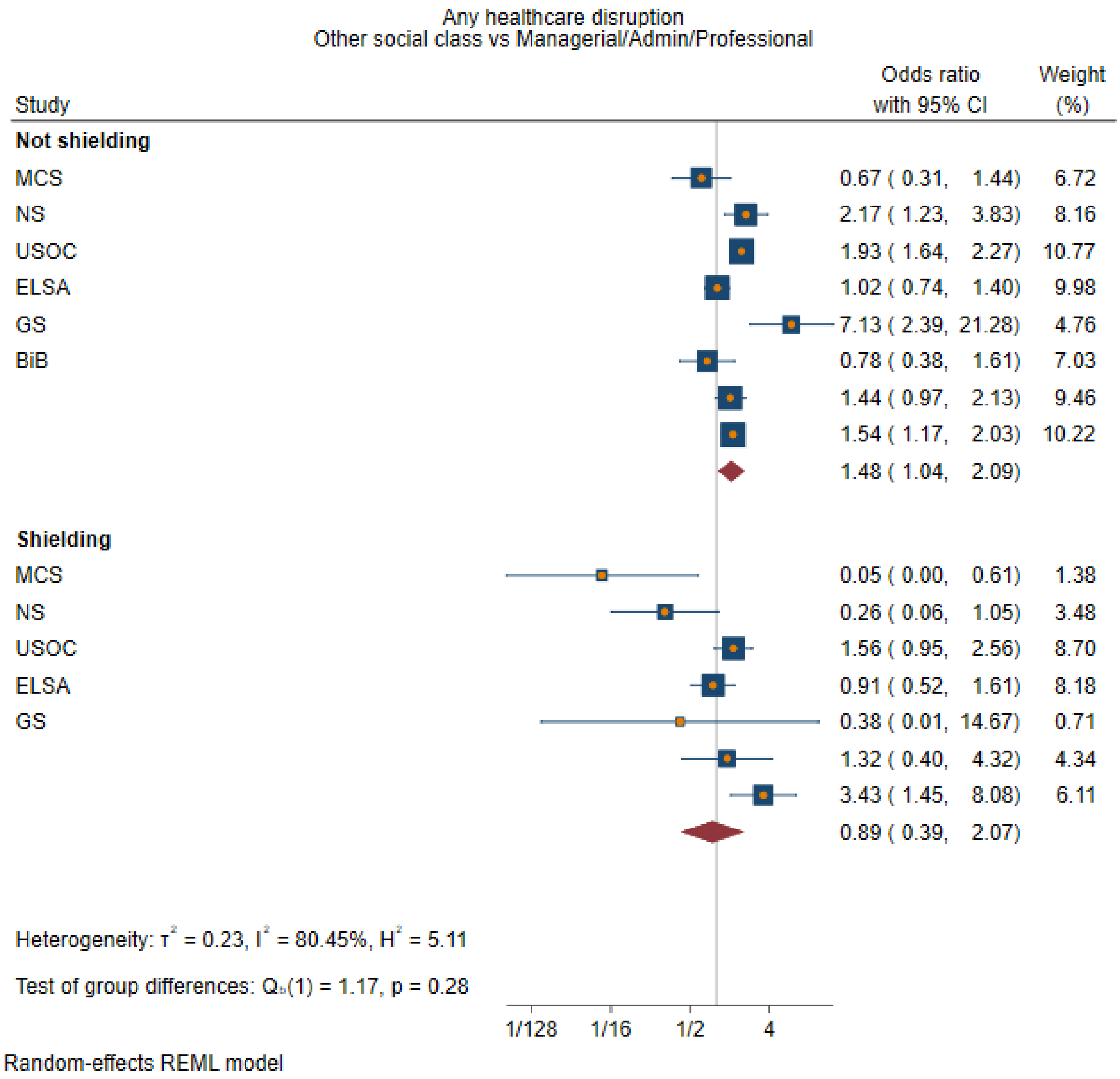

For peer review only - http://bmjopen.bmj.com/site/about/guidelines.xhtml

Education

For peer review only

For peer review only - http://bmjopen.bmj.com/site/about/guidelines.xhtml

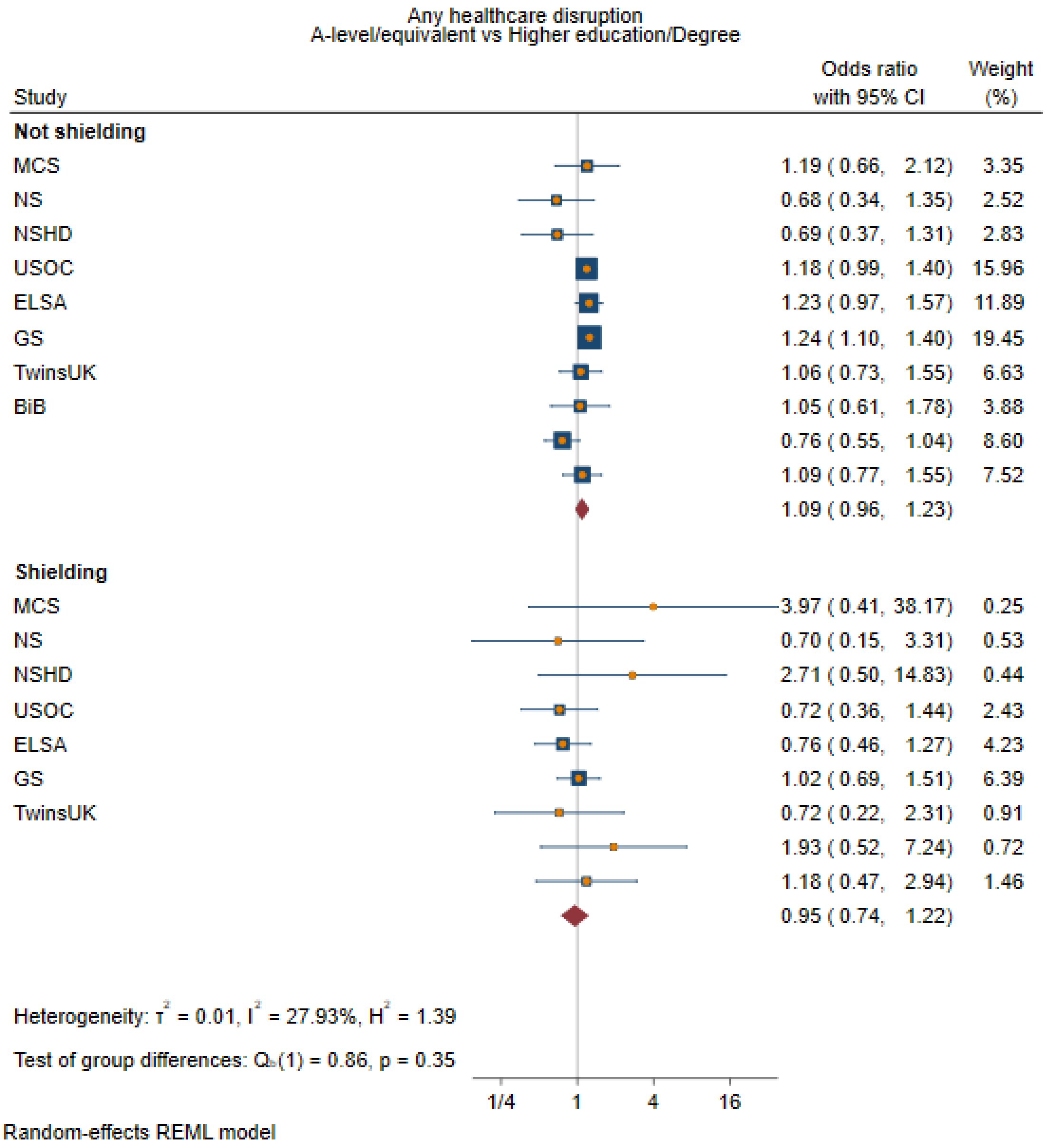
Any healthcare disruption
A-level/equivalent vs Higher education/Degree
Study
Odds ratio with 95% CI
Weight (%)
Not shielding
MCS 1.19 (0.66, 2.12) 3.35
NS 0.68 (0.34, 1.35) 2.52
NSHD 0.69 (0.37, 1.31) 2.83
USOC 1.18 (0.99, 1.40) 15.96
ELSA 1.23 (0.97, 1.57) 11.89
GS 1.24 (1.10, 1.40) 19.45
TwinsUK 1.06 (0.73, 1.55) 6.63
BiB 1.05 (0.61, 1.78) 3.88
0.76 (0.55, 1.04) 8.60
1.09 (0.77, 1.55) 7.52
1.09 (0.96, 1.23)
Shielding
MCS 3.97 (0.41, 38.17) 0.25
NS 0.70 (0.15, 3.31) 0.53
NSHD 2.71 (0.50, 14.83) 0.44
USOC 0.72 (0.36, 1.44) 2.43
ELSA 0.76 (0.46, 1.27) 4.23
GS 1.02 (0.69, 1.51) 6.39
TwinsUK 0.72 (0.22, 2.31) 0.91
1.93 (0.52, 7.24) 0.72
1.18 (0.47, 2.94) 1.46
0.95 (0.74, 1.22)
Heterogeneity: τ² = 0.01, I² = 27.93%, H² = 1.39
Test of group differences: Q_b(1) = 0.86, p = 0.35
1/4
1
4
16
Random-effects REML model

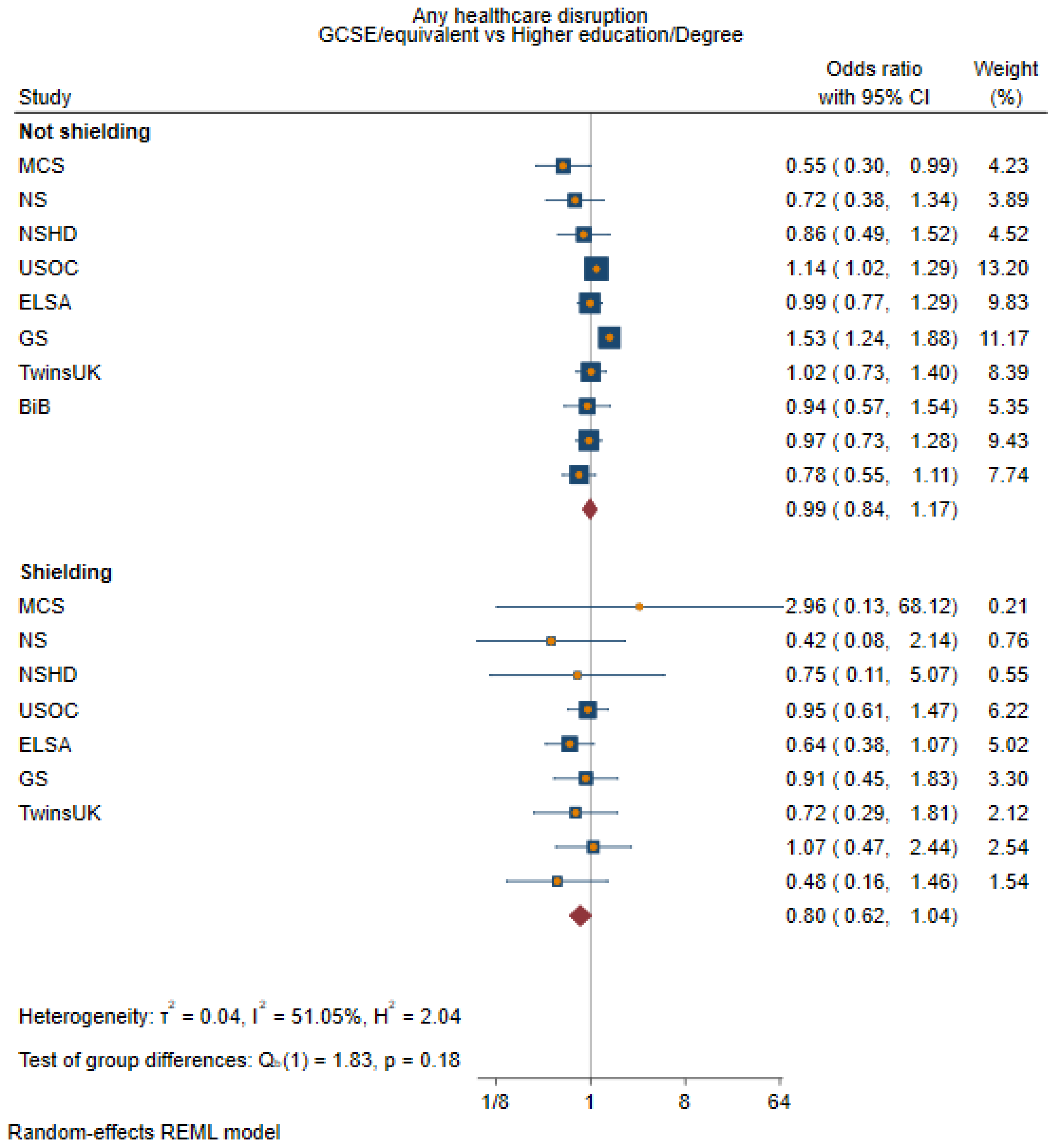

For peer review only - http://bmjopen.bmj.com/site/about/guidelines.xhtml

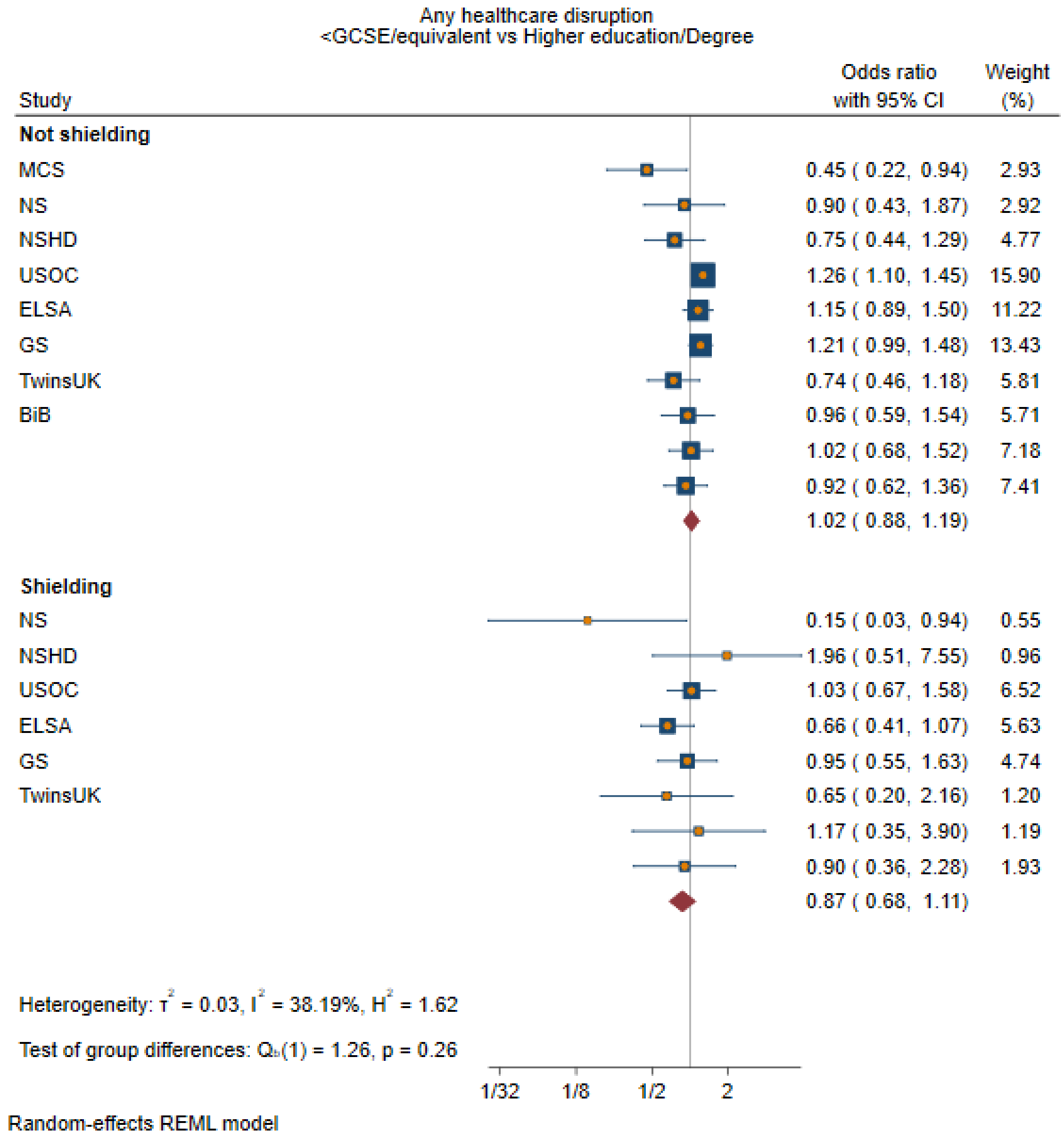

For peer review only - http://bmjopen.bmj.com/site/about/guidelines.xhtml

Age

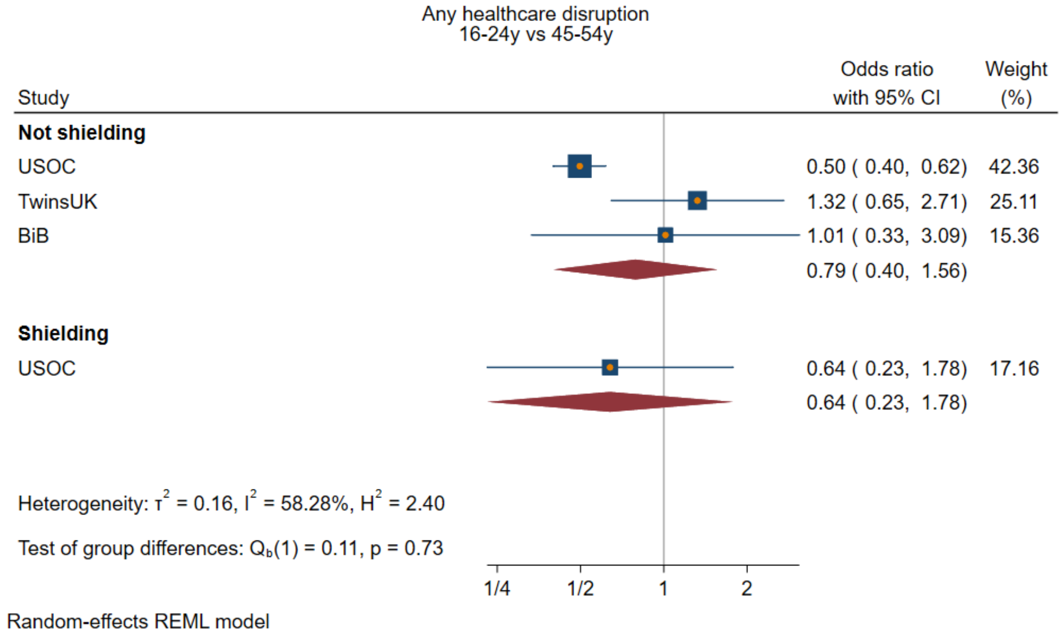

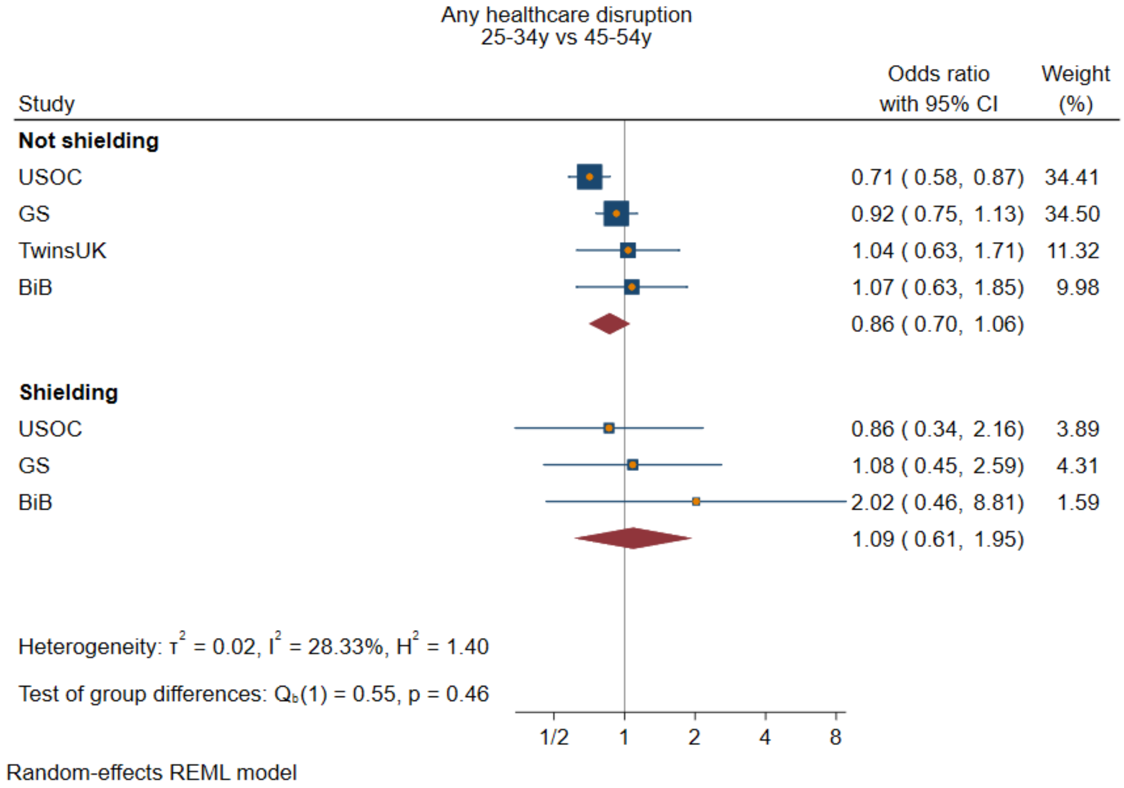

For peer review only - http://bmjopen.bmj.com/site/about/guidelines.xhtml

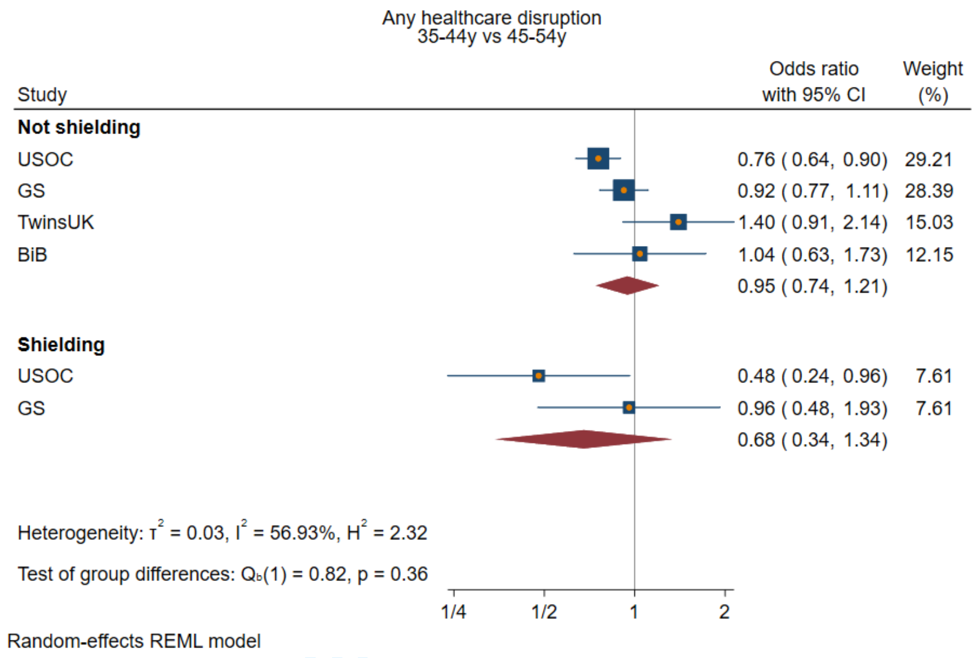

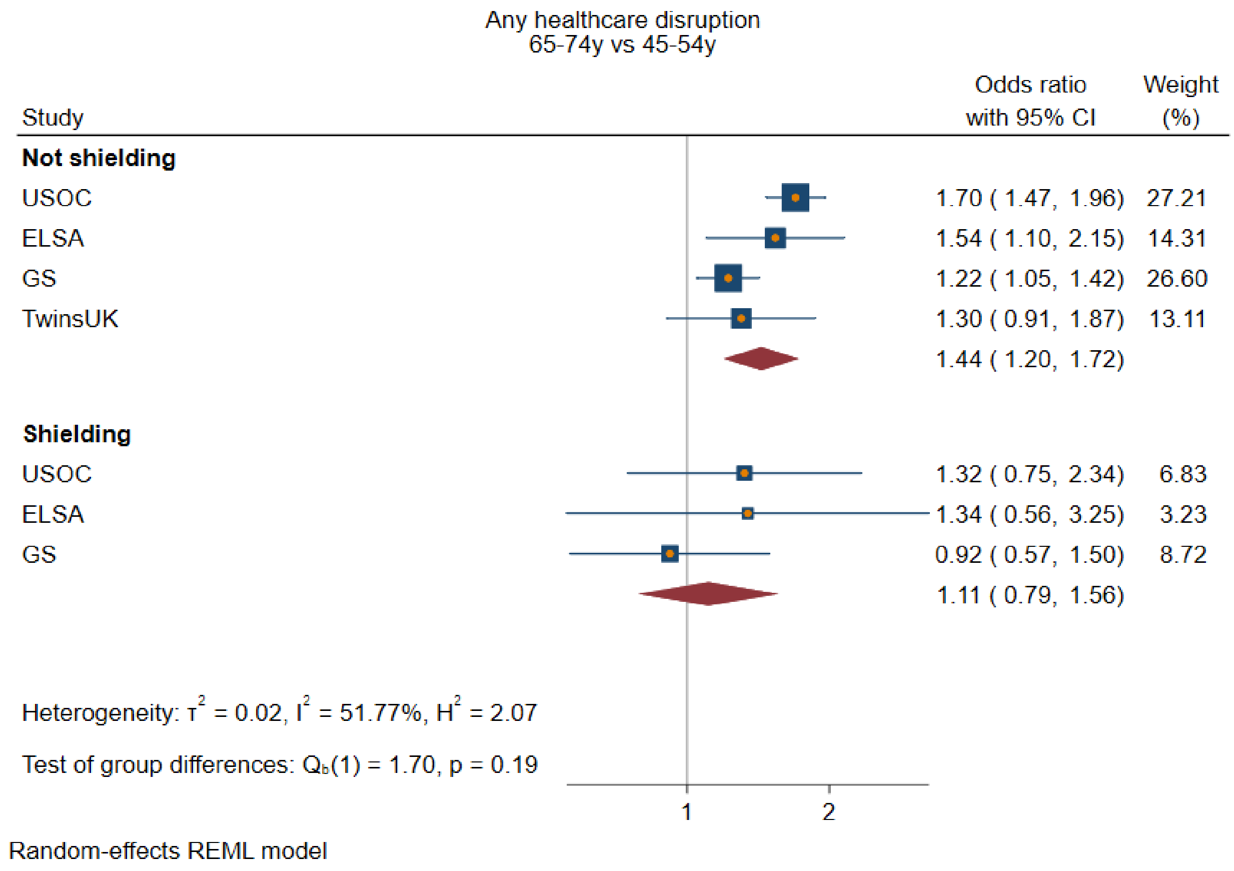

For peer review only - http://bmjopen.bmj.com/site/about/guidelines.xhtml

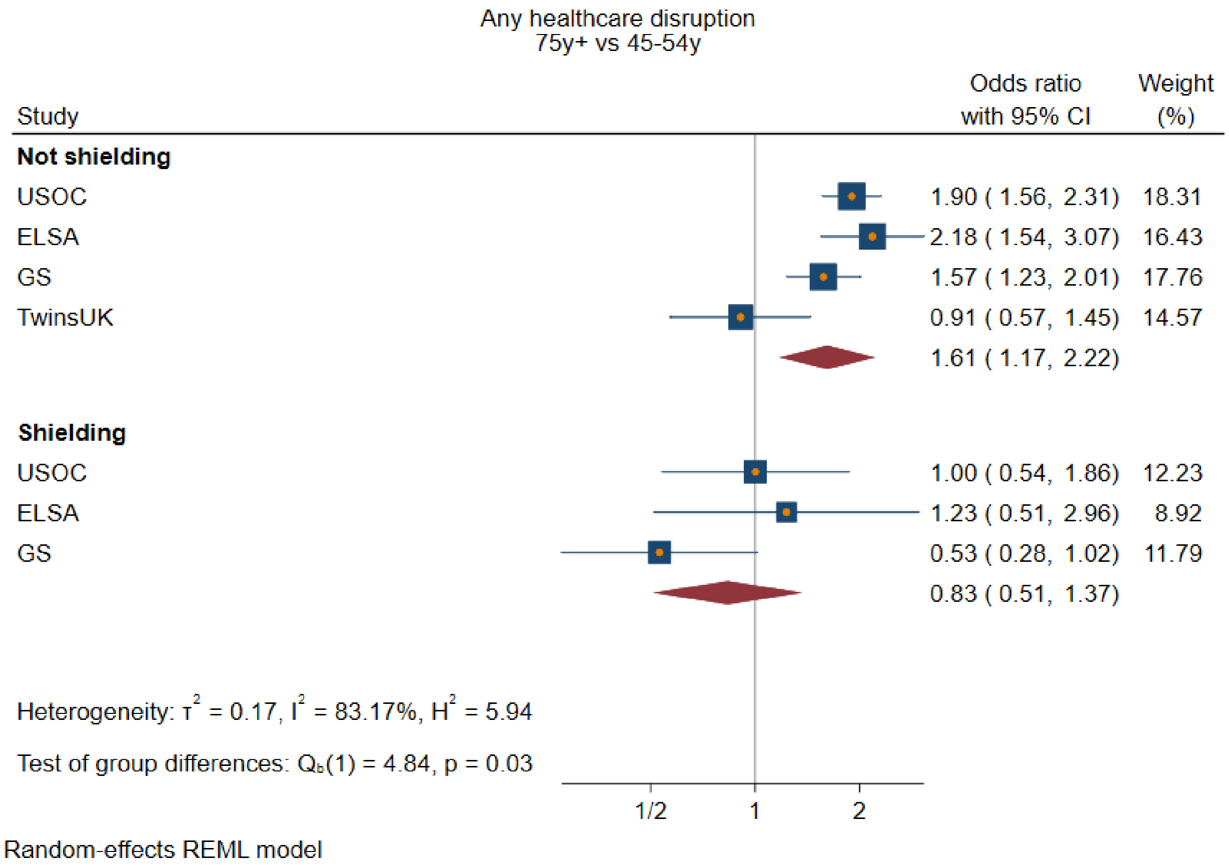
Any healthcare disruption
75y+ vs 45-54y
Study
Odds ratio
with 95% CI
Weight
(%)
Not shielding
USOC 1.90 (1.56, 2.31) 18.31
ELSA 2.18 (1.54, 3.07) 16.43
GS 1.57 (1.23, 2.01) 17.76
TwinsUK 0.91 (0.57, 1.45) 14.57
1.61 (1.17, 2.22)
Shielding
USOC 1.00 (0.54, 1.86) 12.23
ELSA 1.23 (0.51, 2.96) 8.92
GS 0.53 (0.28, 1.02) 11.79
0.83 (0.51, 1.37)
Heterogeneity: τ² = 0.17, I² = 83.17%, H² = 5.94
Test of group differences: Q_b(1) = 4.84, p = 0.03
1/2
1
2
Random-effects REML model

view only

Ethnicity

For peer review only

For peer review only - http://bmjopen.bmj.com/site/about/guidelines.xhtml

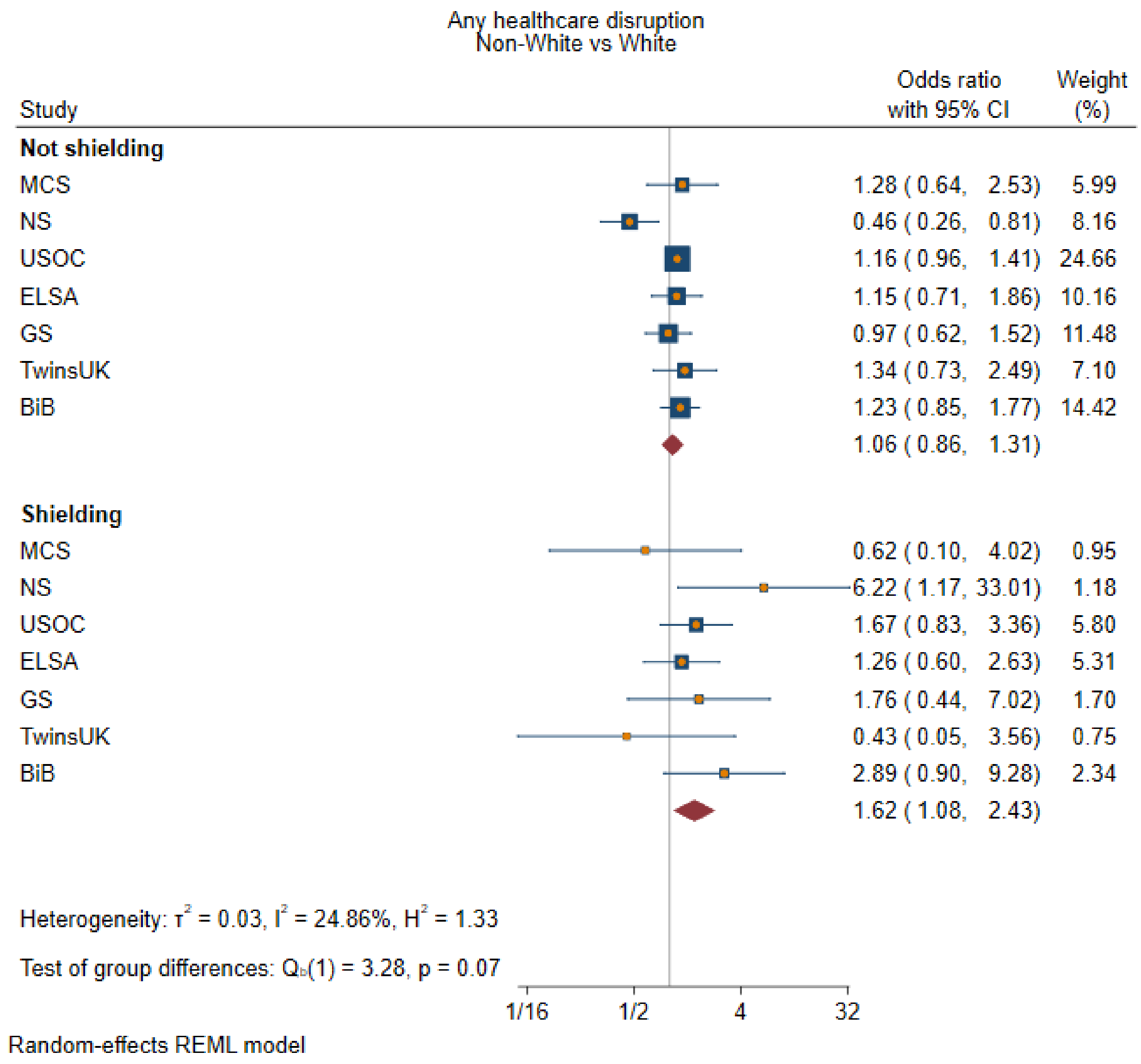

For peer review only - http://bmjopen.bmj.com/site/about/guidelines.xhtml

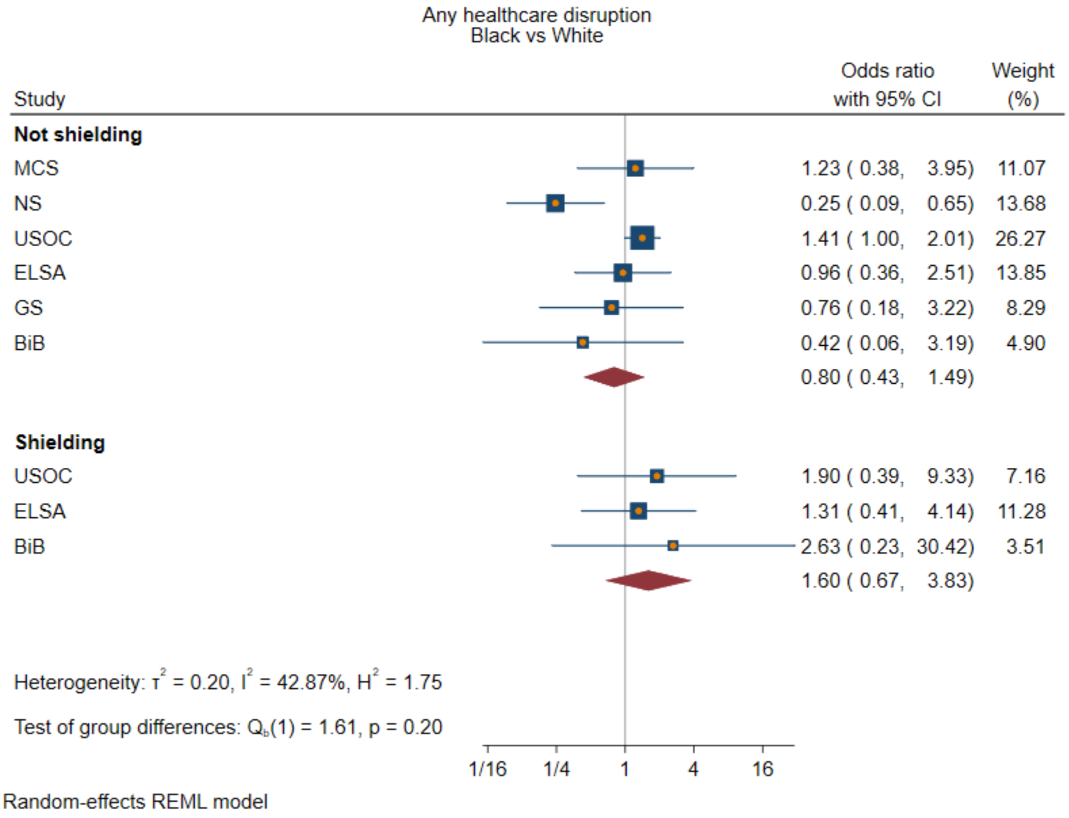

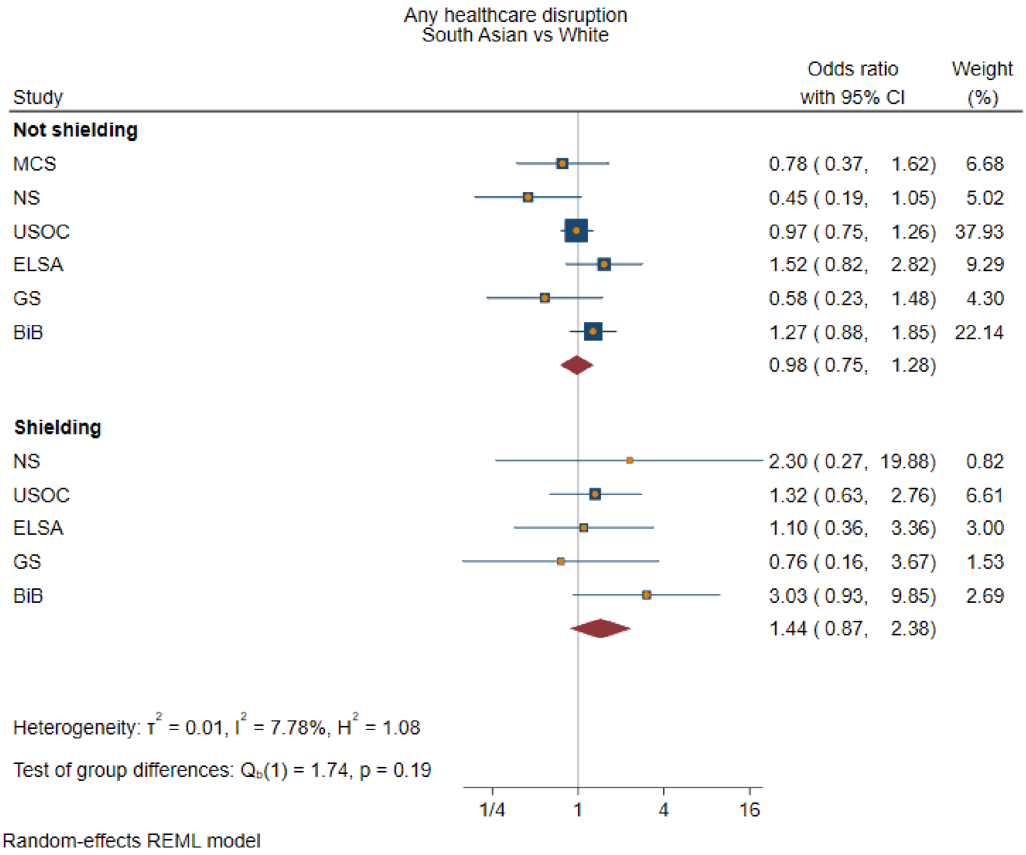

For peer review only - http://bmjopen.bmj.com/site/about/guidelines.xhtml

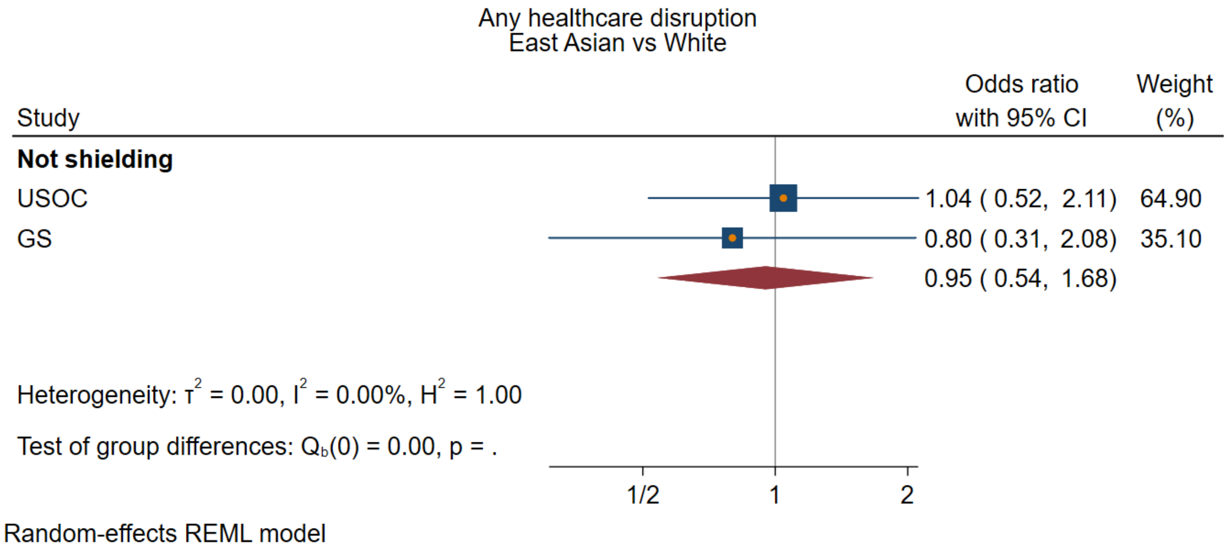
Any healthcare disruption
East Asian vs White
Study
Odds ratio with 95% CI
Weight (%)
Not shielding
USOC
1.04 (0.52, 2.11)
64.90
GS
0.80 (0.31, 2.08)
35.10
0.95 (0.54, 1.68)
Heterogeneity: τ² = 0.00, I² = 0.00%, H² = 1.00
Test of group differences: Qb(0) = 0.00, p = .
1/2
1
2
Random-effects REML model

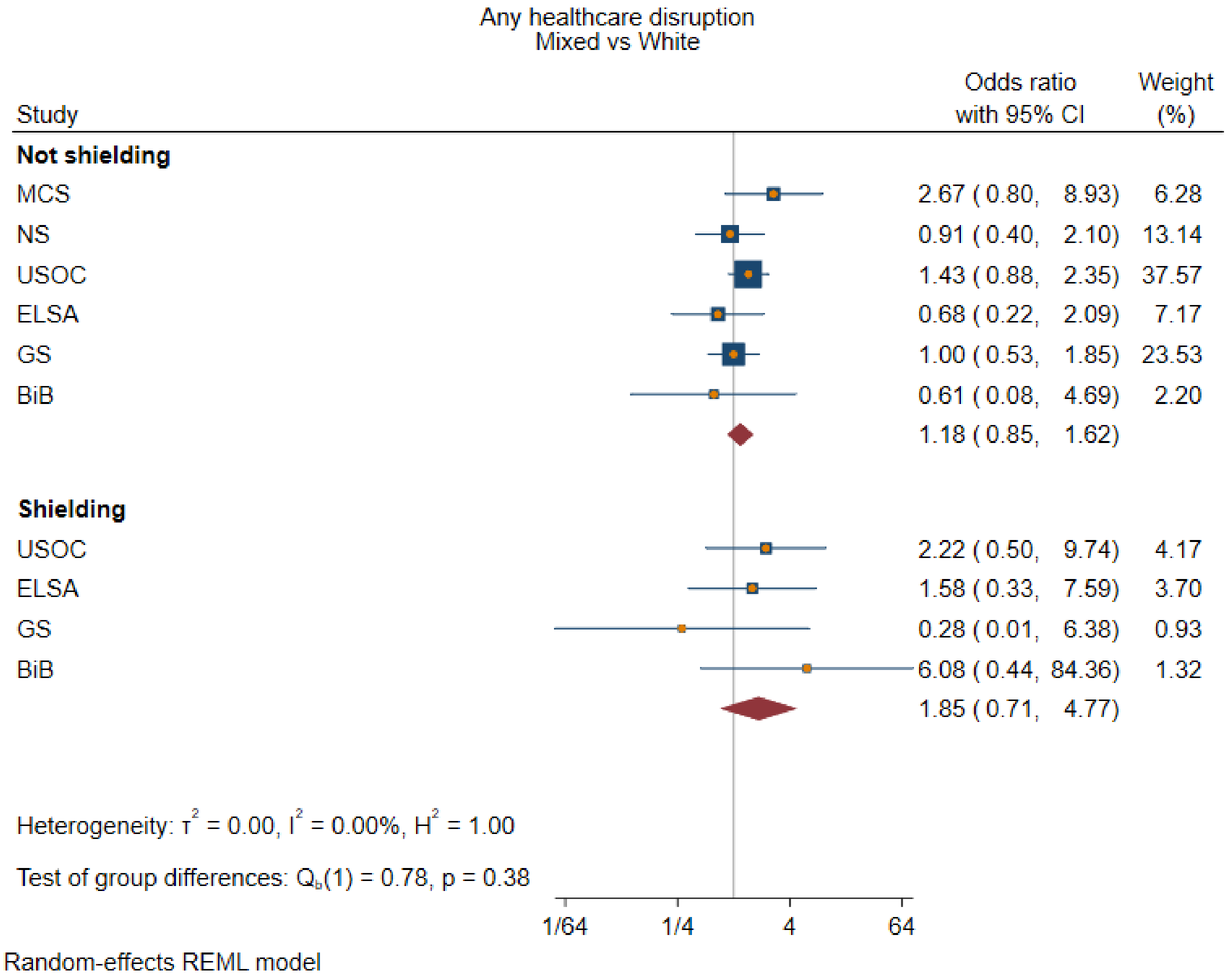
Any healthcare disruption
Mixed vs White
Study
Odds ratio with 95% CI
Weight (%)
Not shielding
MCS
2.67 (0.80, 8.93)
6.28
NS
0.91 (0.40, 2.10)
13.14
USOC
1.43 (0.88, 2.35)
37.57
ELSA
0.68 (0.22, 2.09)
7.17
GS
1.00 (0.53, 1.85)
23.53
BiB
0.61 (0.08, 4.69)
2.20
1.18 (0.85, 1.62)
Shielding
USOC
2.22 (0.50, 9.74)
4.17
ELSA
1.58 (0.33, 7.59)
3.70
GS
0.28 (0.01, 6.38)
0.93
BiB
6.08 (0.44, 84.36)
1.32
1.85 (0.71, 4.77)
Heterogeneity: τ² = 0.00, I² = 0.00%, H² = 1.00
Test of group differences: Qb(1) = 0.78, p = 0.38
1/64
1/4
4
64
Random-effects REML model

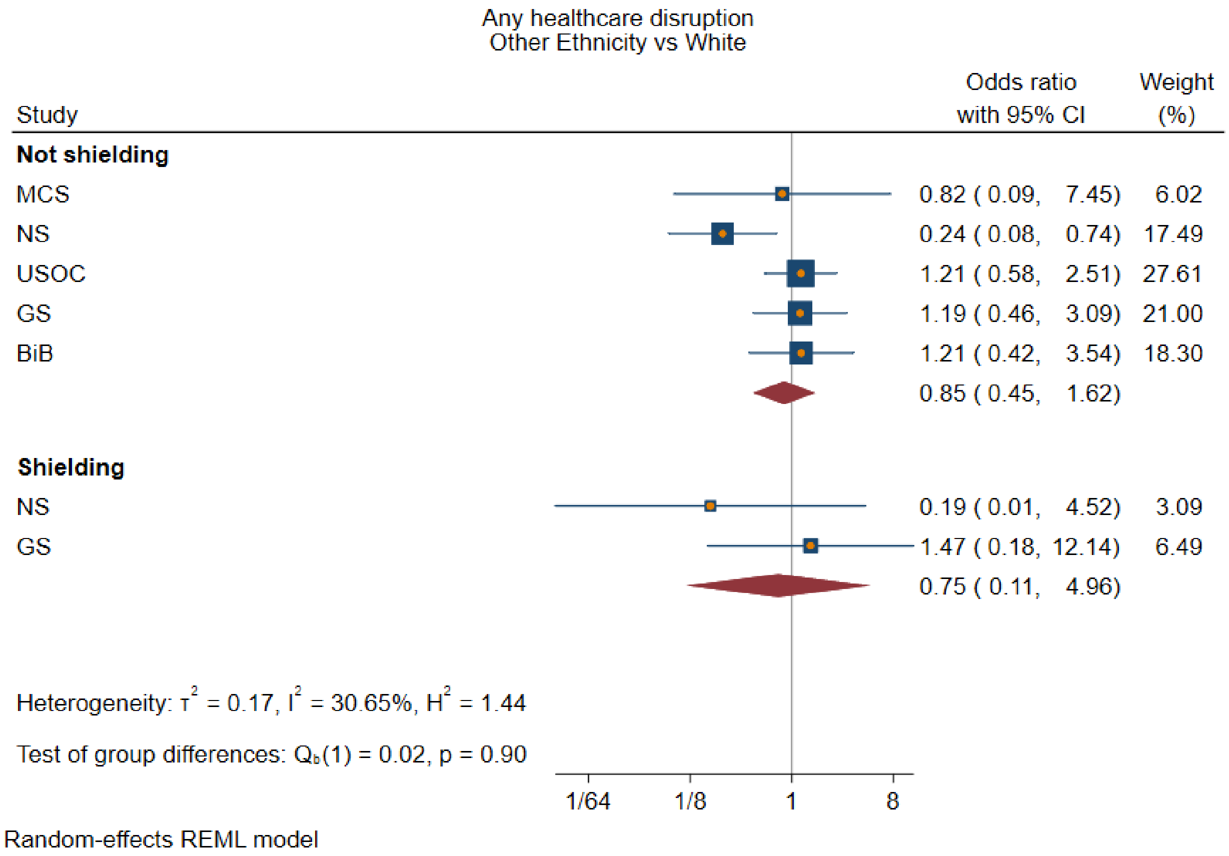
Any healthcare disruption
Other Ethnicity vs White
Study
Odds ratio with 95% CI
Weight (%)
Not shielding
MCS 0.82 (0.09, 7.45) 6.02
NS 0.24 (0.08, 0.74) 17.49
USOC 1.21 (0.58, 2.51) 27.61
GS 1.19 (0.46, 3.09) 21.00
BiB 1.21 (0.42, 3.54) 18.30
0.85 (0.45, 1.62)
Shielding
NS 0.19 (0.01, 4.52) 3.09
GS 1.47 (0.18, 12.14) 6.49
0.75 (0.11, 4.96)
Heterogeneity: τ² = 0.17, I² = 30.65%, H² = 1.44
Test of group differences: Qb(1) = 0.02, p = 0.90
1/64
1/8
1
8
Random-effects REML model

Any healthcare disruption stratified by age

Sex

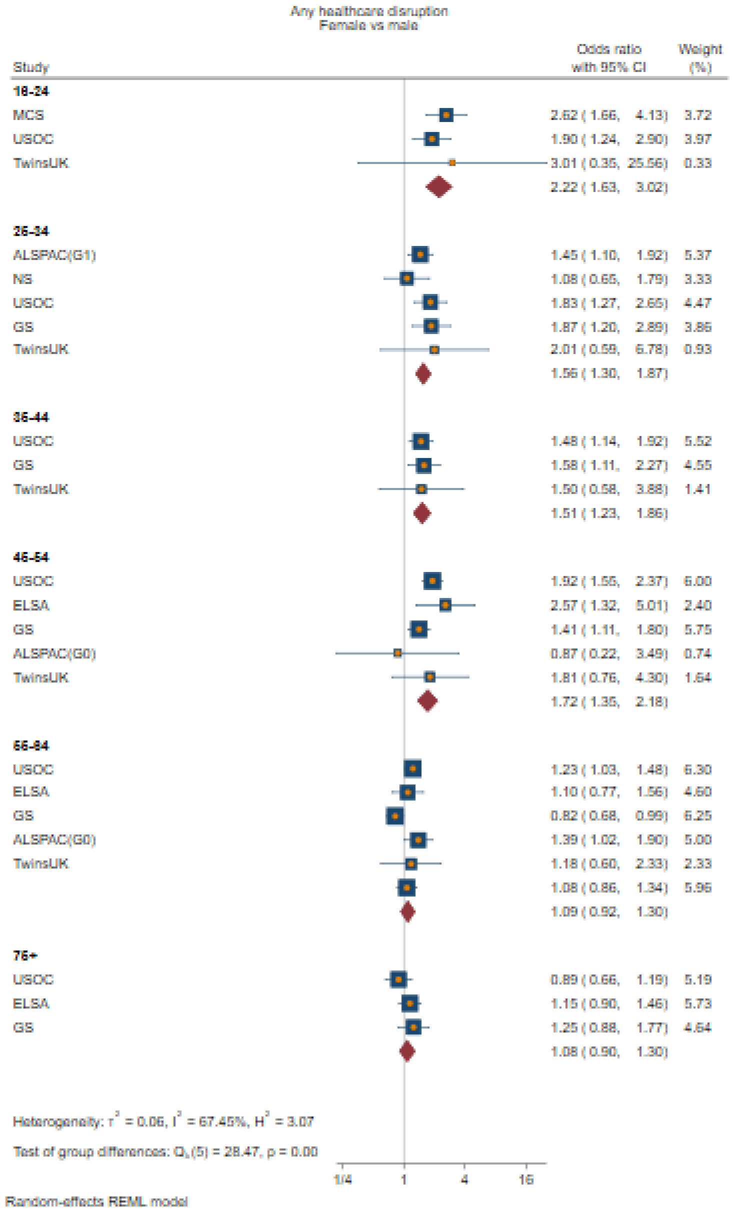

Occupational class

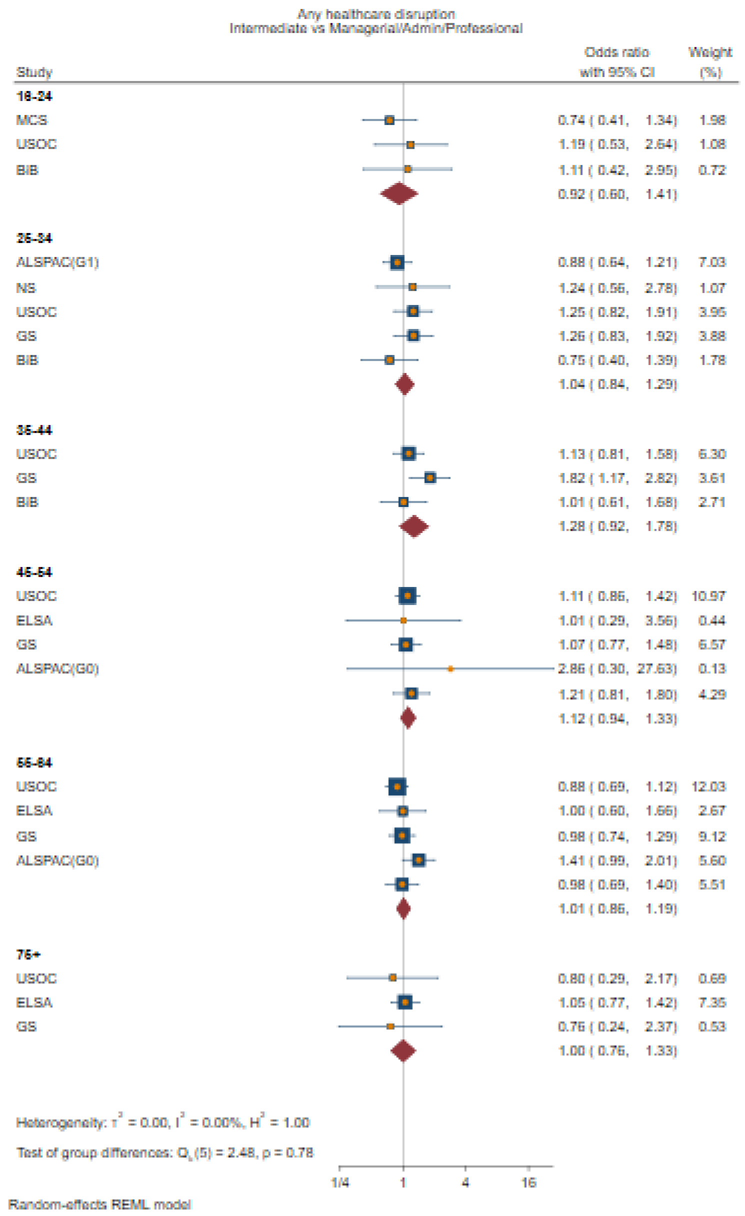

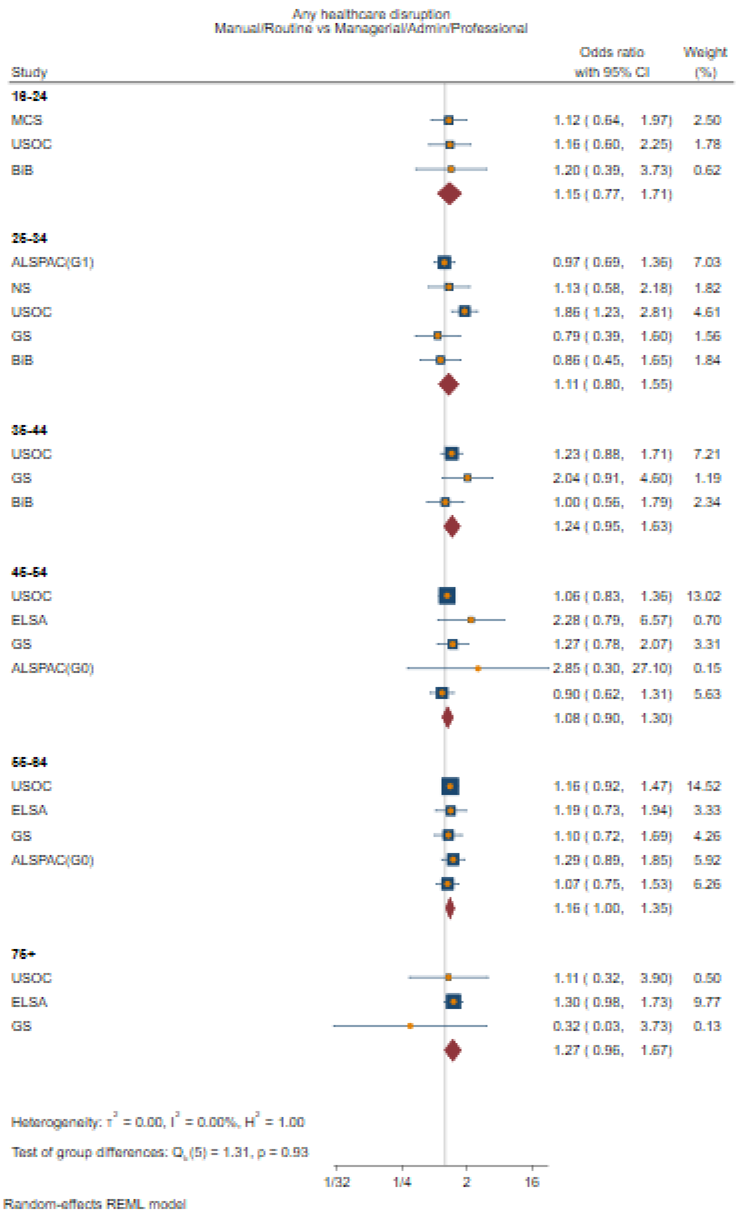
Any healthcare disruption
Manual/Routine vs Managerial/Admin/Professional
Study
Odds ratio with 95% CI
Weight (%)
18-24
MCS 1.12 (0.64, 1.97) 2.50
USOC 1.16 (0.60, 2.25) 1.78
BIB 1.20 (0.39, 3.73) 0.62
1.15 (0.77, 1.71)
25-34
ALSPAC(G1) 0.97 (0.69, 1.36) 7.03
NS 1.13 (0.58, 2.18) 1.82
USOC 1.86 (1.23, 2.81) 4.61
GS 0.79 (0.39, 1.60) 1.56
BIB 0.86 (0.45, 1.65) 1.84
1.11 (0.80, 1.55)
35-44
USOC 1.23 (0.88, 1.71) 7.21
GS 2.04 (0.91, 4.60) 1.19
BIB 1.00 (0.56, 1.79) 2.34
1.24 (0.95, 1.63)
45-54
USOC 1.06 (0.83, 1.36) 13.02
ELSA 2.28 (0.79, 6.57) 0.70
GS 1.27 (0.78, 2.07) 3.31
ALSPAC(G0) 2.85 (0.30, 27.10) 0.15
0.90 (0.62, 1.31) 5.63
1.08 (0.90, 1.30)
55-64
USOC 1.16 (0.92, 1.47) 14.52
ELSA 1.19 (0.73, 1.94) 3.33
GS 1.10 (0.72, 1.69) 4.26
ALSPAC(G0) 1.29 (0.89, 1.85) 5.92
1.07 (0.75, 1.53) 6.26
1.16 (1.00, 1.35)
75+
USOC 1.11 (0.32, 3.90) 0.50
ELSA 1.30 (0.98, 1.73) 9.77
GS 0.32 (0.03, 3.73) 0.13
1.27 (0.96, 1.67)
Heterogeneity: τ² = 0.00, I² = 0.00%, H² = 1.00
Test of group differences: Q_b(5) = 1.31, p = 0.93
1/32
1/4
2
16
Random-effects REML model

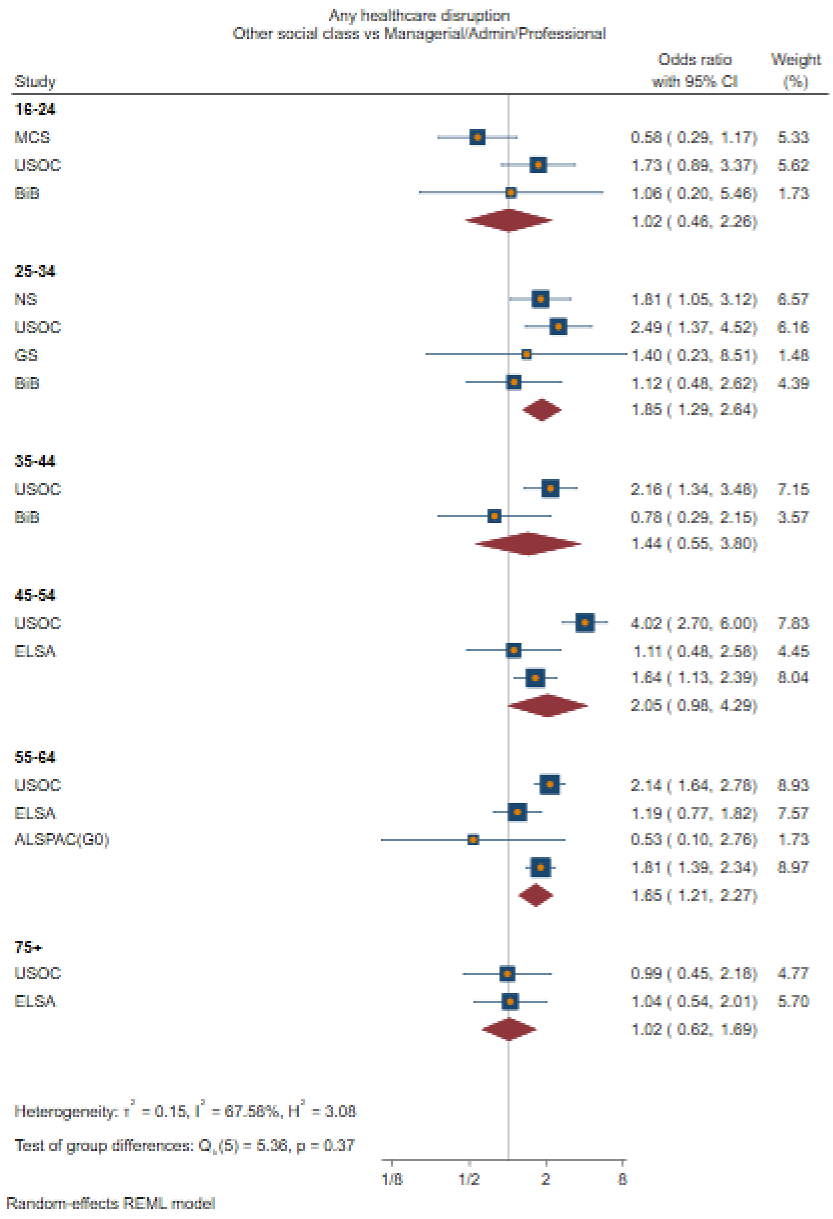
Any healthcare disruption
Other social class vs Managerial/Admin/Professional
Study
Odds ratio with 95% CI
Weight (%)
16-24
MCS
0.58 (0.29, 1.17) 5.33
USOC
1.73 (0.89, 3.37) 5.62
BiB
1.06 (0.20, 5.46) 1.73
1.02 (0.46, 2.26)
25-34
NS
1.81 (1.05, 3.12) 6.57
USOC
2.49 (1.37, 4.52) 6.16
GS
1.40 (0.23, 8.51) 1.48
BiB
1.12 (0.48, 2.62) 4.39
1.85 (1.29, 2.64)
35-44
USOC
2.16 (1.34, 3.48) 7.15
BiB
0.78 (0.29, 2.15) 3.57
1.44 (0.55, 3.80)
45-54
USOC
4.02 (2.70, 6.00) 7.83
ELSA
1.11 (0.48, 2.58) 4.45
1.64 (1.13, 2.39) 8.04
2.05 (0.98, 4.29)
55-64
USOC
2.14 (1.64, 2.78) 8.93
ELSA
1.19 (0.77, 1.82) 7.57
ALSPAC(G0)
0.53 (0.10, 2.76) 1.73
1.81 (1.39, 2.34) 8.97
1.65 (1.21, 2.27)
75+
USOC
0.99 (0.45, 2.18) 4.77
ELSA
1.04 (0.54, 2.01) 5.70
1.02 (0.62, 1.69)
Heterogeneity: τ² = 0.15, I² = 67.58%, H² = 3.08
Test of group differences: Q_b(5) = 5.36, p = 0.37
1/8
1/2
2
8
Random-effects REML model

Education

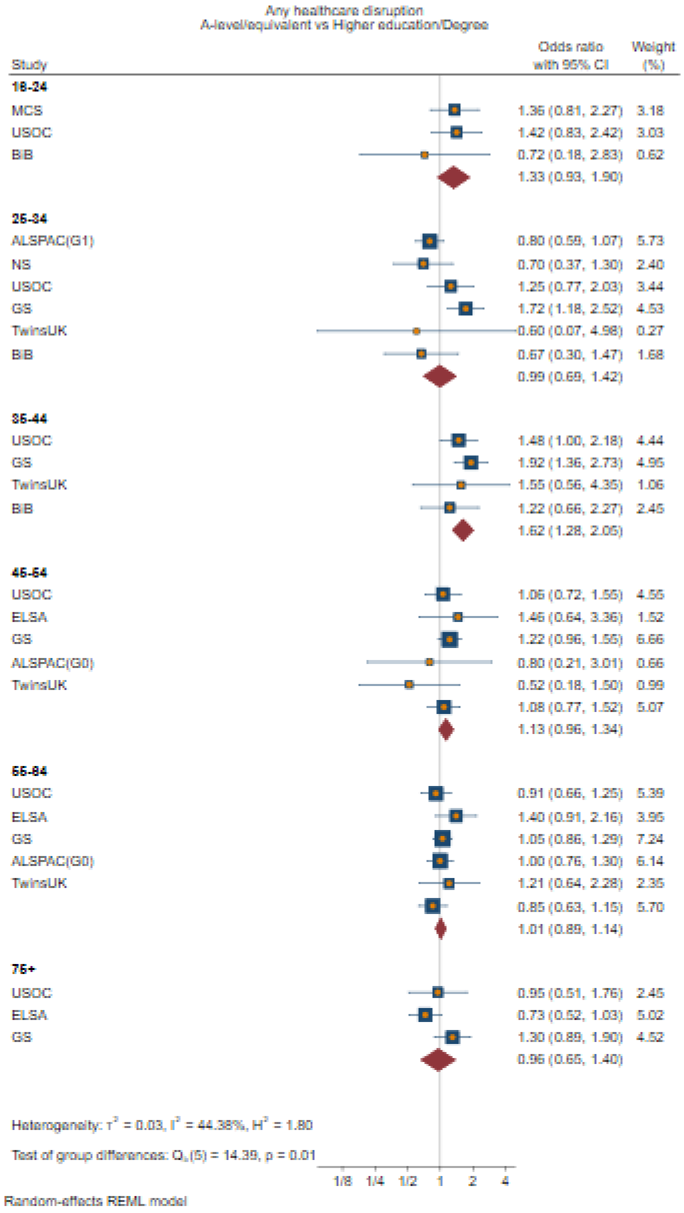
Any healthcare disruption
A-level/equivalent vs Higher education/Degree
Study
Odds ratio with 95% CI
Weight (%)
18-24
MCS 1.36 (0.81, 2.27) 3.18
USOC 1.42 (0.83, 2.42) 3.03
BIB 0.72 (0.18, 2.83) 0.62
1.33 (0.93, 1.90)
25-34
ALSPAC(G1) 0.80 (0.59, 1.07) 5.73
NS 0.70 (0.37, 1.30) 2.40
USOC 1.25 (0.77, 2.03) 3.44
GS 1.72 (1.18, 2.52) 4.53
TwinsUK 0.60 (0.07, 4.98) 0.27
BIB 0.67 (0.30, 1.47) 1.68
0.99 (0.69, 1.42)
35-44
USOC 1.48 (1.00, 2.18) 4.44
GS 1.92 (1.36, 2.73) 4.95
TwinsUK 1.55 (0.56, 4.35) 1.06
BIB 1.22 (0.66, 2.27) 2.45
1.62 (1.28, 2.05)
45-54
USOC 1.06 (0.72, 1.55) 4.55
ELSA 1.46 (0.64, 3.36) 1.52
GS 1.22 (0.96, 1.55) 6.66
ALSPAC(G0) 0.80 (0.21, 3.01) 0.66
TwinsUK 0.52 (0.18, 1.50) 0.99
1.08 (0.77, 1.52) 5.07
1.13 (0.96, 1.34)
55-64
USOC 0.91 (0.66, 1.25) 5.39
ELSA 1.40 (0.91, 2.16) 3.95
GS 1.05 (0.86, 1.29) 7.24
ALSPAC(G0) 1.00 (0.76, 1.30) 6.14
TwinsUK 1.21 (0.64, 2.28) 2.35
0.85 (0.63, 1.15) 5.70
1.01 (0.89, 1.14)
75+
USOC 0.95 (0.51, 1.76) 2.45
ELSA 0.73 (0.52, 1.03) 5.02
GS 1.30 (0.89, 1.90) 4.52
0.96 (0.65, 1.40)
Heterogeneity: τ² = 0.03, I² = 44.38%, H² = 1.80
Test of group differences: Qb(5) = 14.39, p = 0.01
1/8 1/4 1/2 1 2 4
Random-effects REML model

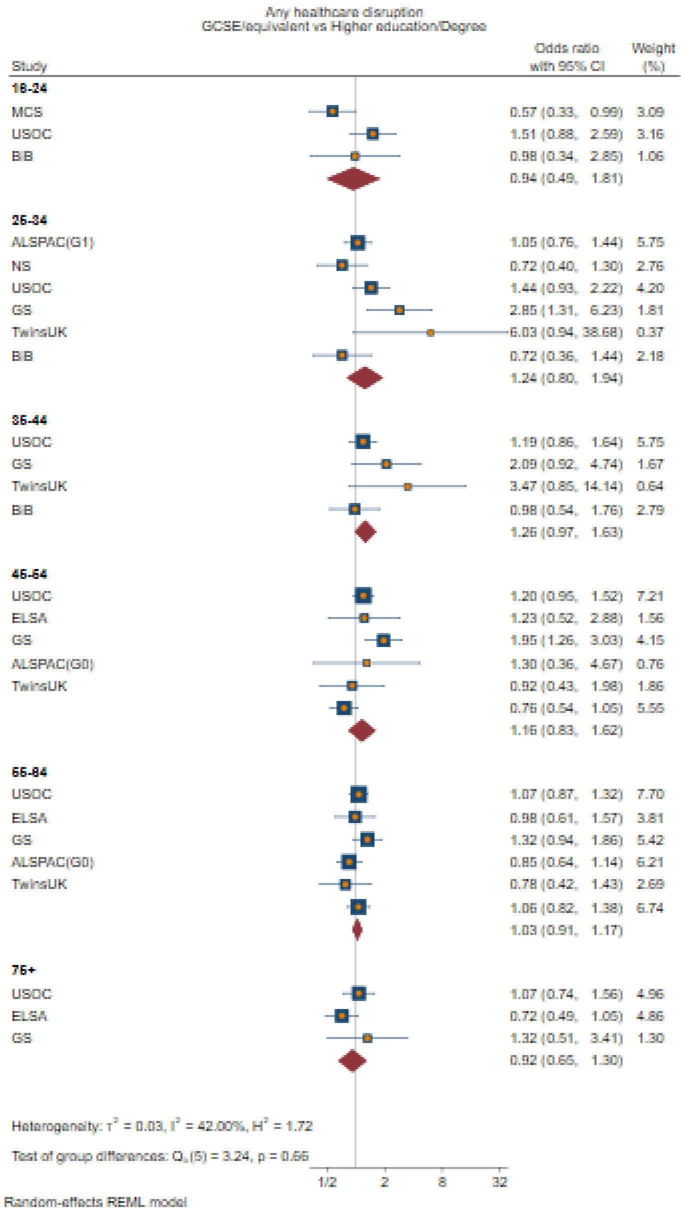
Any healthcare disruption
GCSE/equivalent vs Higher education/Degree
Study
Odds ratio with 95% CI
Weight (%)
16-24
MCS 0.57 (0.33, 0.99) 3.09
USOC 1.51 (0.88, 2.59) 3.16
BIB 0.98 (0.34, 2.85) 1.06
0.94 (0.49, 1.81)
25-34
ALSPAC(G1) 1.05 (0.76, 1.44) 5.75
NS 0.72 (0.40, 1.30) 2.76
USOC 1.44 (0.93, 2.22) 4.20
GS 2.85 (1.31, 6.23) 1.81
TwinsUK 6.03 (0.94, 38.68) 0.37
BIB 0.72 (0.36, 1.44) 2.18
1.24 (0.80, 1.94)
35-44
USOC 1.19 (0.86, 1.64) 5.75
GS 2.09 (0.92, 4.74) 1.67
TwinsUK 3.47 (0.85, 14.14) 0.64
BIB 0.98 (0.54, 1.76) 2.79
1.26 (0.97, 1.63)
45-54
USOC 1.20 (0.95, 1.52) 7.21
ELSA 1.23 (0.52, 2.88) 1.56
GS 1.95 (1.26, 3.03) 4.15
ALSPAC(G0) 1.30 (0.36, 4.67) 0.76
TwinsUK 0.92 (0.43, 1.98) 1.86
0.76 (0.54, 1.05) 5.55
1.16 (0.83, 1.62)
55-64
USOC 1.07 (0.87, 1.32) 7.70
ELSA 0.98 (0.61, 1.57) 3.81
GS 1.32 (0.94, 1.86) 5.42
ALSPAC(G0) 0.85 (0.64, 1.14) 6.21
TwinsUK 0.78 (0.42, 1.43) 2.69
1.06 (0.82, 1.38) 6.74
1.03 (0.91, 1.17)
75+
USOC 1.07 (0.74, 1.56) 4.96
ELSA 0.72 (0.49, 1.05) 4.86
GS 1.32 (0.51, 3.41) 1.30
0.92 (0.65, 1.30)
Heterogeneity: τ² = 0.03, I² = 42.00%, H² = 1.72
Test of group differences: Q_b(5) = 3.24, p = 0.66
1/2 2 8 32
Random-effects REML model

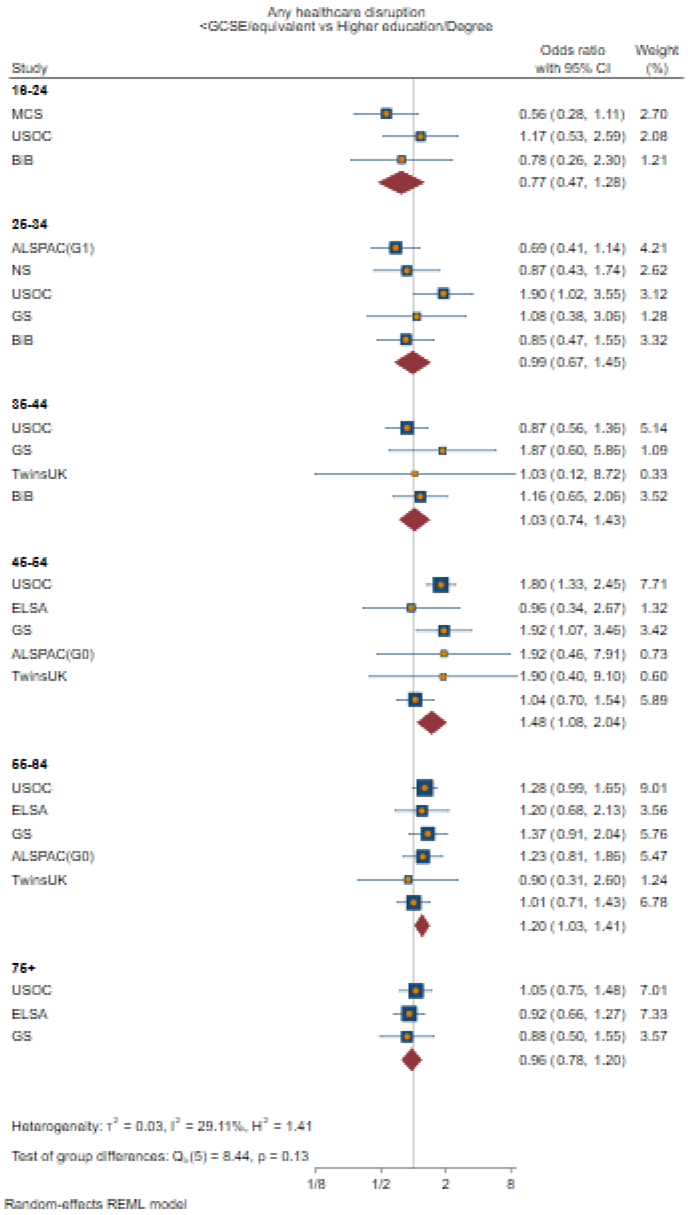
Any healthcare disruption
<GCSE/equivalent vs Higher education/Degree
Study
Odds ratio with 95% CI
Weight (%)
18-24
MCS 0.56 (0.28, 1.11) 2.70
USOC 1.17 (0.53, 2.59) 2.08
BIB 0.78 (0.26, 2.30) 1.21
0.77 (0.47, 1.28)
25-34
ALSPAC(G1) 0.69 (0.41, 1.14) 4.21
NS 0.87 (0.43, 1.74) 2.62
USOC 1.90 (1.02, 3.55) 3.12
GS 1.08 (0.38, 3.06) 1.28
BIB 0.85 (0.47, 1.55) 3.32
0.99 (0.67, 1.45)
35-44
USOC 0.87 (0.56, 1.36) 5.14
GS 1.87 (0.60, 5.86) 1.09
TwinsUK 1.03 (0.12, 8.72) 0.33
BIB 1.16 (0.65, 2.06) 3.52
1.03 (0.74, 1.43)
45-54
USOC 1.80 (1.33, 2.45) 7.71
ELSA 0.96 (0.34, 2.67) 1.32
GS 1.92 (1.07, 3.46) 3.42
ALSPAC(G0) 1.92 (0.46, 7.91) 0.73
TwinsUK 1.90 (0.40, 9.10) 0.60
1.04 (0.70, 1.54) 5.89
1.48 (1.08, 2.04)
55-64
USOC 1.28 (0.99, 1.65) 9.01
ELSA 1.20 (0.68, 2.13) 3.56
GS 1.37 (0.91, 2.04) 5.76
ALSPAC(G0) 1.23 (0.81, 1.86) 5.47
TwinsUK 0.90 (0.31, 2.60) 1.24
1.01 (0.71, 1.43) 6.78
1.20 (1.03, 1.41)
75+
USOC 1.05 (0.75, 1.48) 7.01
ELSA 0.92 (0.66, 1.27) 7.33
GS 0.88 (0.50, 1.55) 3.57
0.96 (0.78, 1.20)
Heterogeneity: τ² = 0.03, I² = 29.11%, H² = 1.41
Test of group differences: Q_b(5) = 8.44, p = 0.13
1/8
1/2
2
8
Random-effects REML model

Ethnicity

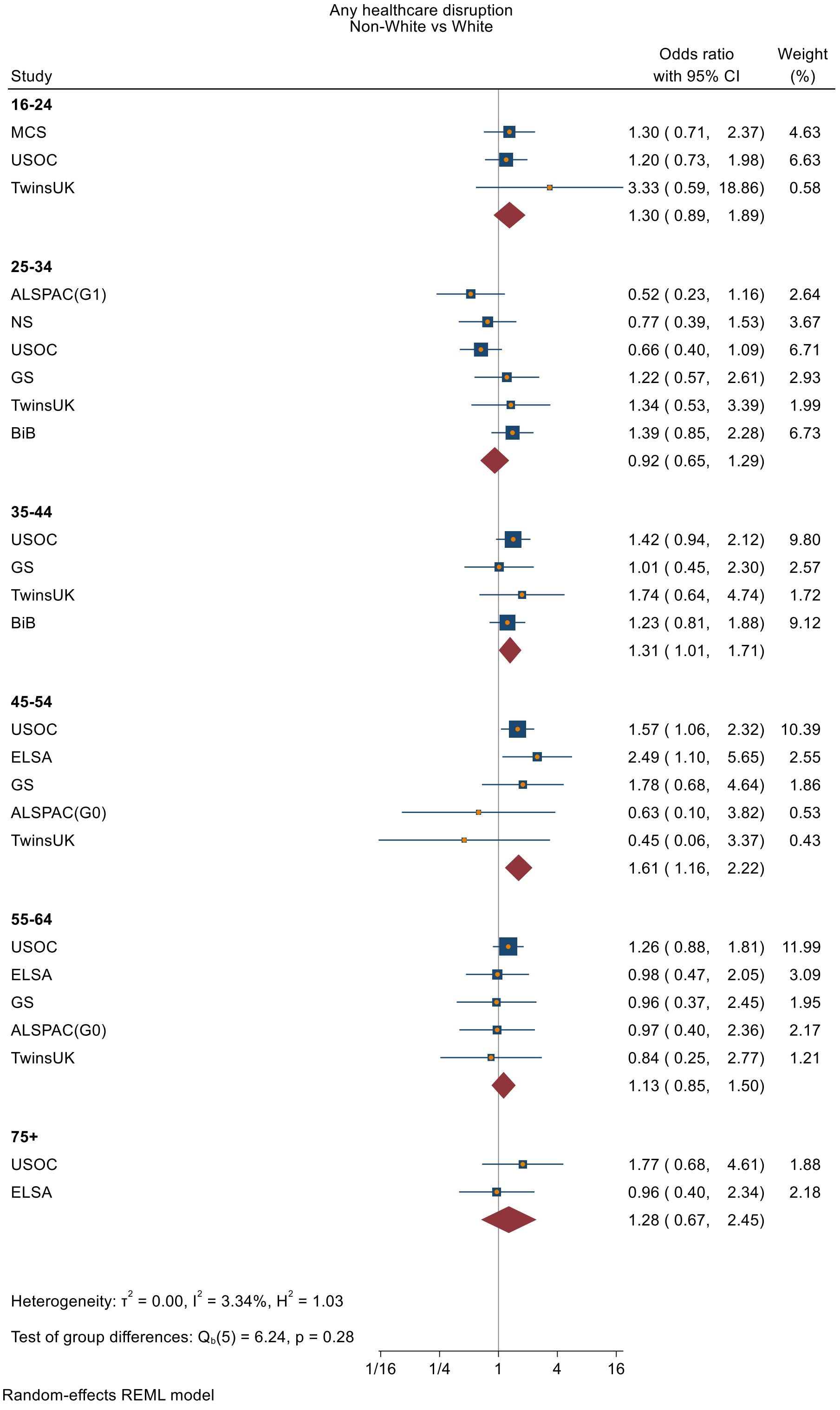

For peer review only - http://bmjopen.bmj.com/site/about/guidelines.xhtml

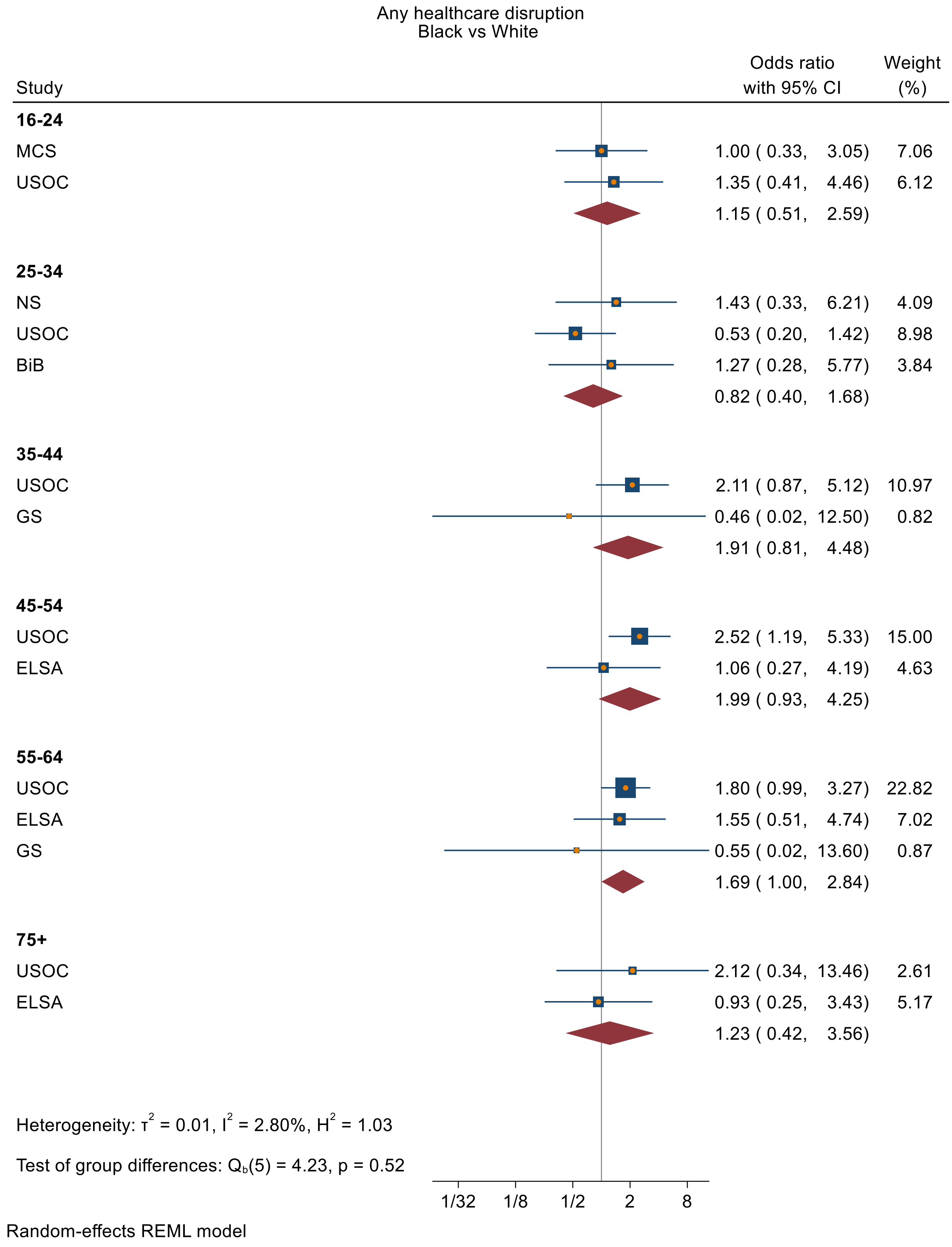

For peer review only - http://bmjopen.bmj.com/site/about/guidelines.xhtml

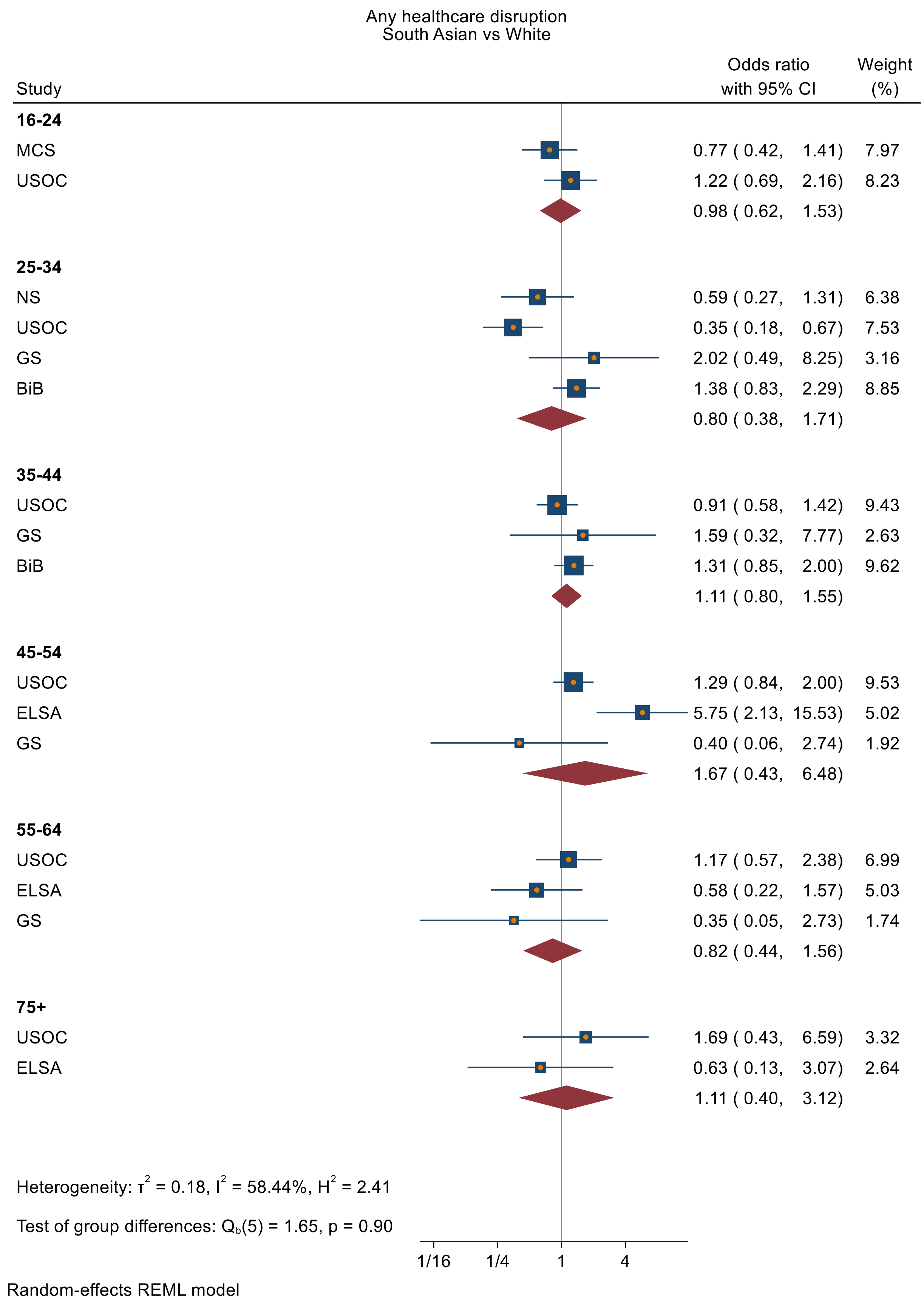

For peer review only - http://bmjopen.bmj.com/site/about/guidelines.xhtml

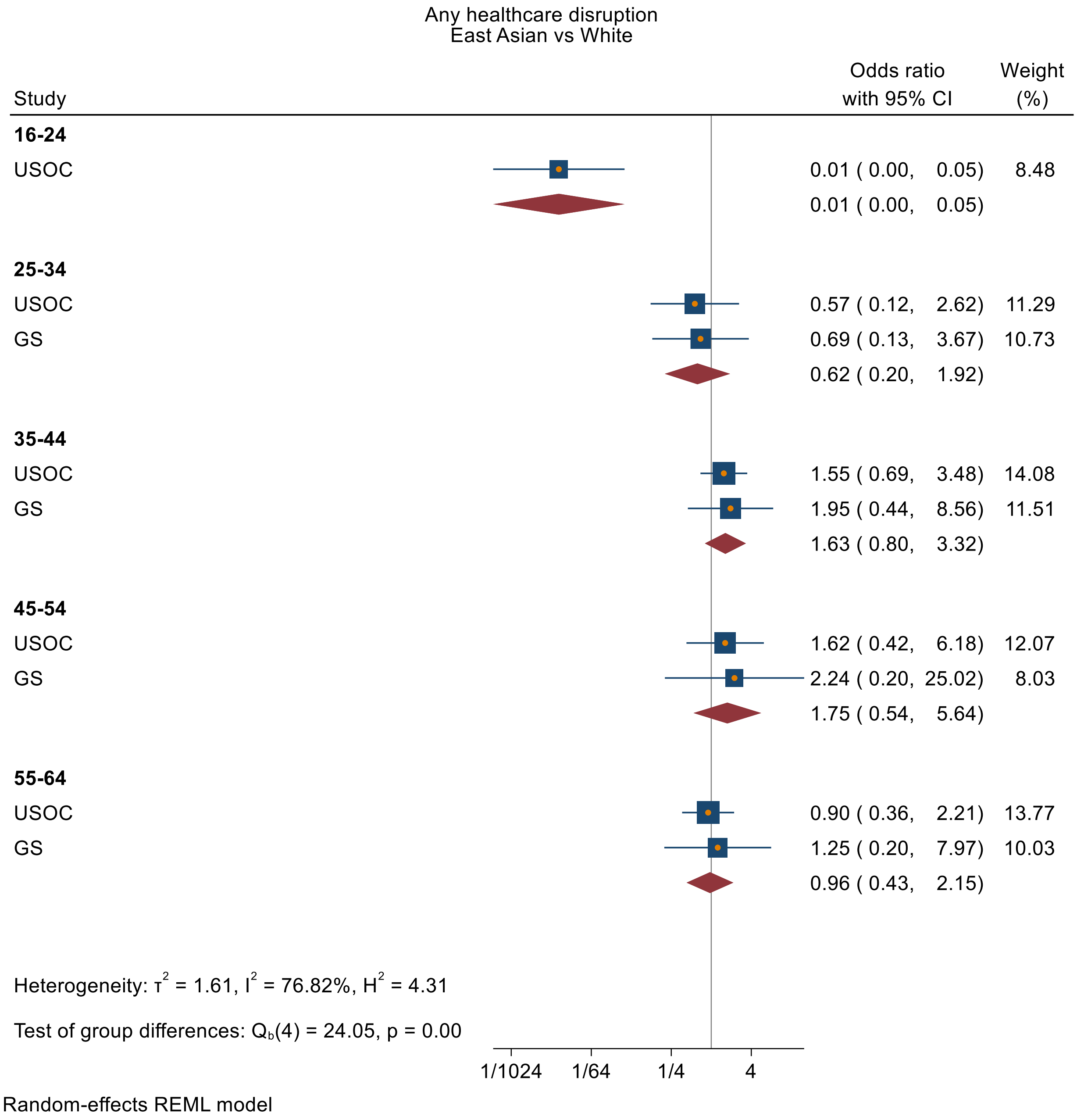
Any healthcare disruption
East Asian vs White
Study
Odds ratio with 95% CI
Weight (%)
16-24
USOC
0.01 (0.00, 0.05) 8.48
0.01 (0.00, 0.05)
25-34
USOC
0.57 (0.12, 2.62) 11.29
GS
0.69 (0.13, 3.67) 10.73
0.62 (0.20, 1.92)
35-44
USOC
1.55 (0.69, 3.48) 14.08
GS
1.95 (0.44, 8.56) 11.51
1.63 (0.80, 3.32)
45-54
USOC
1.62 (0.42, 6.18) 12.07
GS
2.24 (0.20, 25.02) 8.03
1.75 (0.54, 5.64)
55-64
USOC
0.90 (0.36, 2.21) 13.77
GS
1.25 (0.20, 7.97) 10.03
0.96 (0.43, 2.15)
Heterogeneity: τ^2 = 1.61, I^2 = 76.82%, H^2 = 4.31
Test of group differences: $Q_b(4)$ = 24.05, p = 0.00
1/1024
1/64
1/4
4
Random-effects REML model

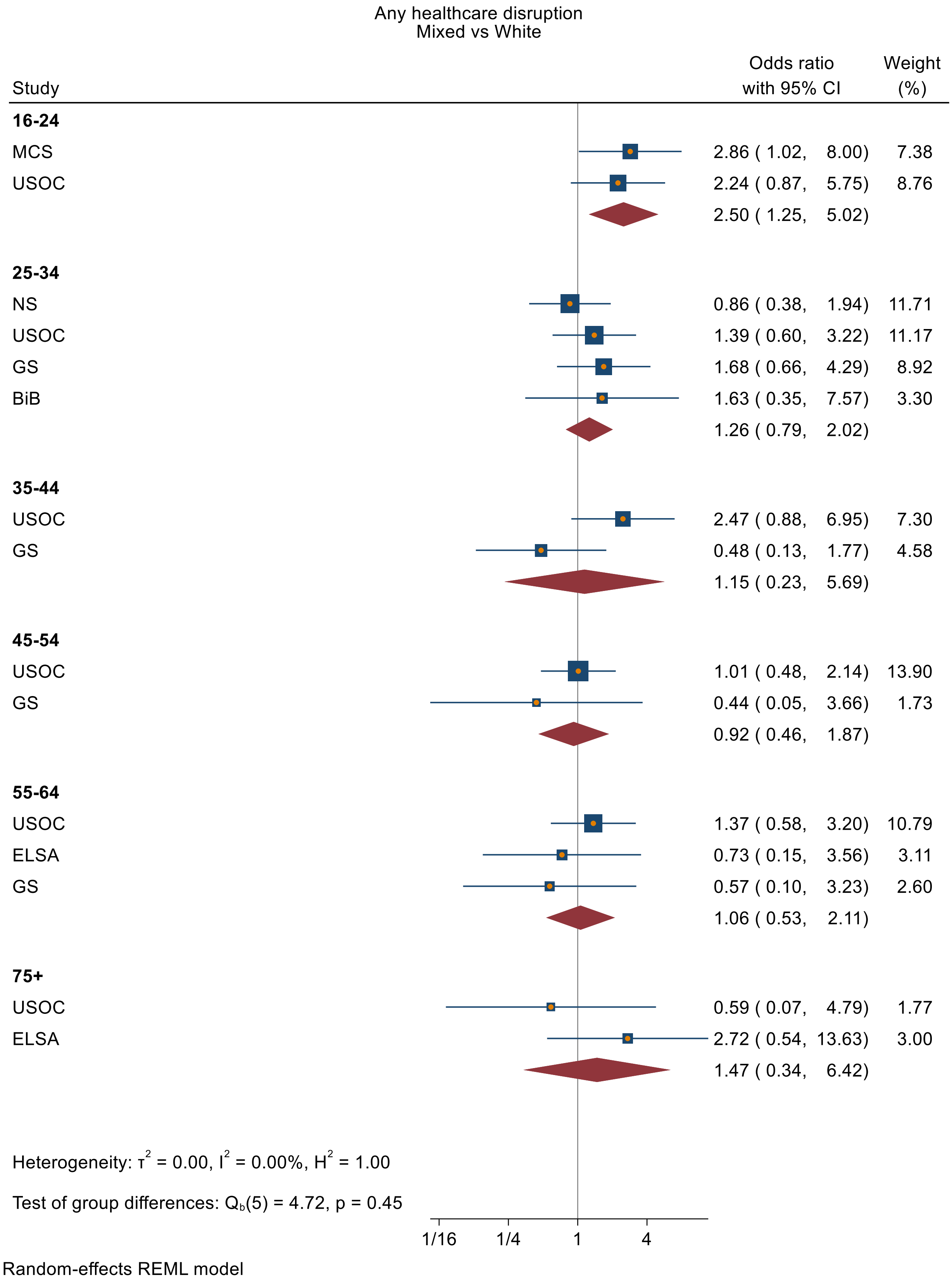

For peer review only - http://bmjopen.bmj.com/site/about/guidelines.xhtml

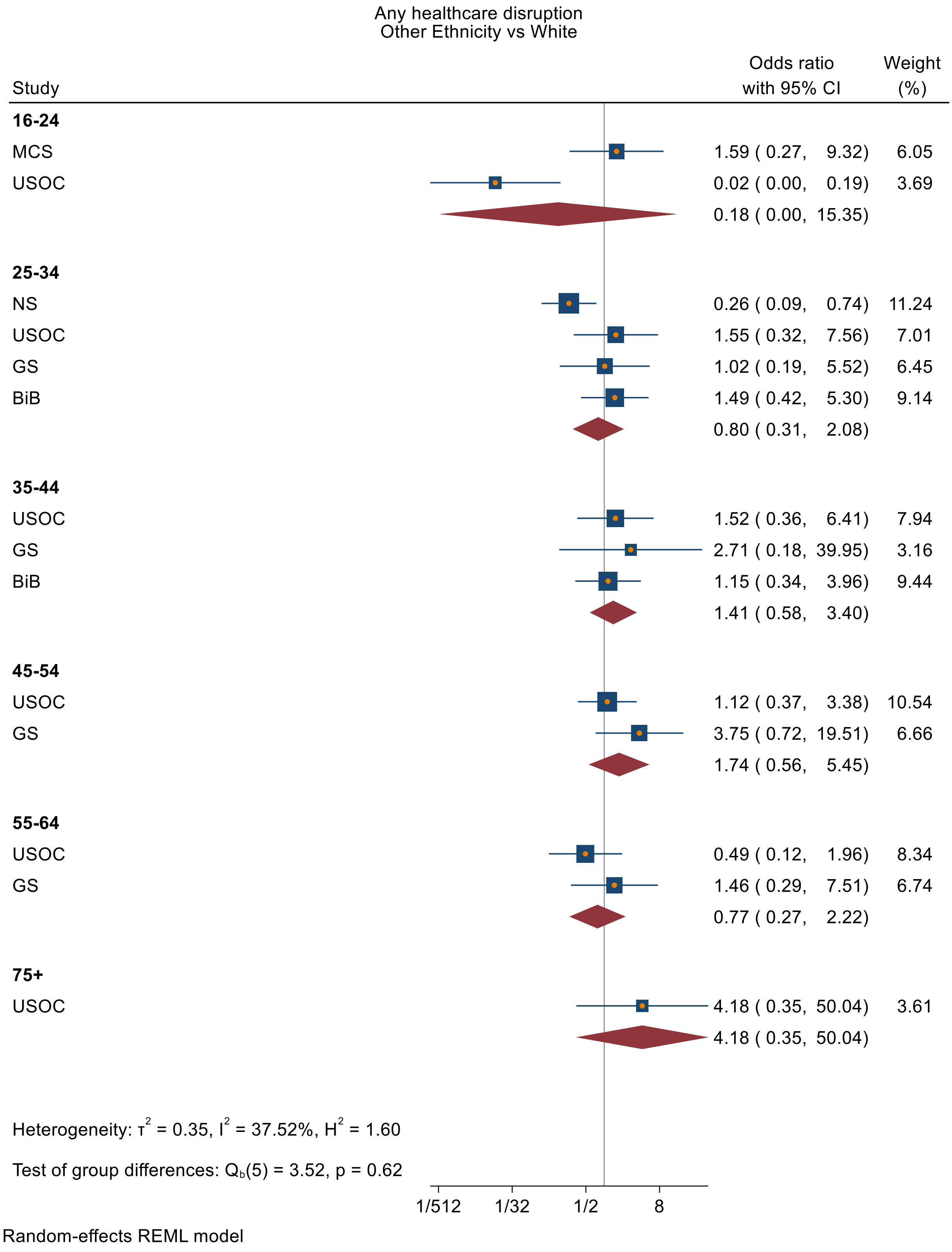

For peer review only - http://bmjopen.bmj.com/site/about/guidelines.xhtml

Supplementary file 3: Meta-analysis summary restricted to representative studies

Note: ASLPAC, GS, TwinsUK and BiB excluded.

Summary of results

		Any healthcare disruption				Appointments				Prescription/Medication				Procedures/surgery			
		OR	Lower CI	Upper CI	I^2%	OR	Lower CI	Upper CI	I^2%	OR	Lower CI	Upper CI	I^2%	OR	Lower CI	Upper CI	I^2%
**Sex**																	
Female vs. Male	Unadjusted[‡]	1.27	1.19	1.36	0	1.29	1.18	1.42	5.66	1.39	0.90	2.14	73.3	1.24	1.13	1.37	0
	**Basic adjustment**	**1.34**	**1.15**	**1.57**	**65.33**	**1.36**	**1.25**	**1.47**	**0**	**1.37**	**0.86**	**2.16**	**74.9**	**1.27**	**1.12**	**1.43**	**11.85**
	Full adjustment	1.34	1.15	1.56	61.89	1.34	0.94	1.91		1.99	0.77	5.12		1.21	1.01	1.44	
**Age**																	
16-24y vs 45-54y	Unadjusted	0.50	0.41	0.62		0.43	0.34	0.54		0.65	0.42	1.02		0.48	0.34	0.68	
	**Basic adjustment**	**0.49**	**0.39**	**0.60**		**0.42**	**0.33**	**0.52**		**0.62**	**0.39**	**0.97**		**0.47**	**0.33**	**0.66**	
	Full adjustment	0.47	0.37	0.61		no information				no information				no information			
25-34y vs 45-54y	Unadjusted	0.71	0.58	0.86		0.65	0.53	0.80		0.97	0.66	1.44		0.78	0.57	1.07	
	**Basic adjustment**	**0.70**	**0.58**	**0.85**		**0.64**	**0.52**	**0.79**		**0.97**	**0.65**	**1.43**		**0.77**	**0.56**	**1.06**	
	Full adjustment	0.77	0.63	0.94		no information				no information				no information			
35-44y vs 45-54y	Unadjusted	0.74	0.63	0.88		0.70	0.58	0.83		0.83	0.58	1.18		0.88	0.69	1.12	
	**Basic adjustment**	**0.74**	**0.63**	**0.87**		**0.69**	**0.58**	**0.82**		**0.83**	**0.58**	**1.18**		**0.87**	**0.68**	**1.11**	
	Full adjustment	0.86	0.73	1.03		no information				no information				no information			
55-64y vs 45-54y	Unadjusted	1.40	1.23	1.59	0	1.37	1.19	1.58	0	0.75	0.27	2.07	67.12	1.51	1.26	1.80	0
	**Basic adjustment**	**1.42**	**1.25**	**1.61**	**0**	**1.39**	**1.21**	**1.60**	**0**	**0.80**	**0.30**	**2.09**	**64.1**	**1.52**	**1.28**	**1.80**	**0**
	Full adjustment	1.21	1.06	1.40	0	1.04	0.48	2.25		0.52	0.16	1.68		1.37	0.93	2.01	

65-74y vs 45-54y	Unadjusted	1.72	1.51	1.96	0	1.57	1.21	2.04	16.91	0.76	0.31	1.86	59.63	1.93	1.63	2.30	0
	**Basic adjustment**	**1.78**	**1.56**	**2.02**	**0**	**1.67**	**1.42**	**1.97**	**2.81**	**0.85**	**0.38**	**1.91**	**52.96**	**1.98**	**1.67**	**2.34**	**0**
	Full adjustment	1.35	1.14	1.58	0	1.01	0.42	2.43		1.41	0.34	5.89		1.55	1.05	2.30	
75y+ vs 45-54y	Unadjusted	1.97	1.68	2.31	1.58	1.87	1.56	2.24	0	0.89	0.59	1.35	6.53	2.10	1.46	3.02	66.45
	**Basic adjustment**	**2.06**	**1.76**	**2.41**	**0**	**1.97**	**1.64**	**2.35**	**0**	**0.98**	**0.68**	**1.42**	**0**	**2.14**	**1.57**	**2.91**	**55.32**
	Full adjustment	1.38	1.13	1.70	0.00	1.07	0.44	2.61		1.26	0.39	4.02		1.75	1.17	2.62	
**Ethnicity**																	
Non-White vs White*	Unadjusted	0.96	0.82	1.12	0	1.02	0.72	1.46	44.43	1.02	0.39	2.67	84.87	0.90	0.71	1.14	0
	**Basic adjustment**	**1.23**	**1.05**	**1.44**	**0**	**1.25**	**0.87**	**1.81**	**48.35**	**1.06**	**0.42**	**2.67**	**83.18**	**1.16**	**0.91**	**1.47**	**0**
	Full adjustment	1.10	0.94	1.29	0	1.39	0.61	3.20		2.04	0.70	5.98		0.96	0.63	1.48	
Black vs White	Unadjusted	1.22	0.91	1.65	0	1.02	0.53	1.94	48.54	0.49	0.07	3.52	85.41	0.87	0.58	1.31	0
	**Basic adjustment**	**1.47**	**1.08**	**1.98**	**0**	**1.18**	**0.57**	**2.44**	**59.36**	**0.50**	**0.08**	**3.36**	**84**	**1.03**	**0.68**	**1.55**	**0**
	Full adjustment	1.20	0.92	1.58	0	0.88	0.18	4.22		0.37	0.04	3.11		0.87	0.41	1.82	
East Asian vs White	Unadjusted	0.82	0.38	1.73		0.79	0.35	1.80		0.97	0.47	1.97		1.38	0.47	4.02	
	**Basic adjustment**	**1.04**	**0.53**	**2.06**		**1.03**	**0.49**	**2.16**		**1.04**	**0.52**	**2.09**		**1.80**	**0.65**	**4.99**	
	Full adjustment	1.01	0.60	1.68		no information				no information				no information			
Mixed vs White	Unadjusted	1.13	0.82	1.57	0	1.27	0.49	3.29	77.57	1.53	0.90	2.60	0	1.12	0.70	1.80	0
	**Basic adjustment**	**1.38**	**0.88**	**2.17**	**34.69**	**1.47**	**0.59**	**3.67**	**75.14**	**1.67**	**0.98**	**2.86**	**0**	**no information**			
	Full adjustment	1.36	0.88	2.11	24.01	no information				0.93	0.10	8.48		0.85	0.32	2.21	
South Asian vs White	Unadjusted	0.76	0.58	1.01	29.78	0.84	0.56	1.25	38.69	0.80	0.17	3.77	93.25	0.70	0.45	1.09	28.01
	**Basic adjustment**	**1.02**	**0.84**	**1.24**	**0**	**1.05**	**0.84**	**1.31**	**0**	**0.83**	**0.18**	**3.76**	**92.41**	**0.90**	**0.64**	**1.26**	**0**
	Full adjustment	0.95	0.72	1.25	21.29	2.65	1.03	6.82		4.47	1.38	14.50		1.11	0.62	1.99	

For peer review only

Other Ethnicity vs White	Unadjusted	0.56	0.25	1.25	40.34	0.82	0.45	1.49	0	0.70	0.14	3.54	63.29	0.81	0.11	6.21	67.17
	**Basic adjustment**	**0.72**	**0.25**	**2.07**	**64.12**	**1.02**	**0.41**	**2.51**	**48.38**	**0.96**	**0.17**	**5.25**	**66.68**	**0.82**	**0.08**	**8.51**	**74.76**
	Full adjustment	0.72	0.25	2.02	64.08	no information				no information				no information			
**Education**																	
A-level/equivalent vs Higher education/Degree	Unadjusted	1.02	0.85	1.22	53.07	1.02	0.84	1.25	39.4	0.94	0.68	1.30	26.88	0.68	0.37	1.28	90.63
	**Basic adjustment**	**1.11**	**0.99**	**1.25**	**8.05**	**1.13**	**0.99**	**1.29**	**1.31**	**0.92**	**0.71**	**1.19**	**0**	**0.73**	**0.38**	**1.39**	**91.07**
	Full adjustment	0.98	0.85	1.12	21.18	0.98	0.59	1.63		3.39	1.04	11.09		1.05	0.83	1.32	
GCSE/equivalent vs Higher education/Degree	Unadjusted	0.96	0.84	1.10	36.2	0.96	0.84	1.11	19.4	0.95	0.69	1.30	39.93	1.01	0.89	1.14	0
	**Basic adjustment**	**0.94**	**0.79**	**1.12**	**55.76**	**0.91**	**0.73**	**1.13**	**53.46**	**0.96**	**0.68**	**1.35**	**45.45**	**1.04**	**0.92**	**1.19**	**0**
	Full adjustment	0.84	0.73	0.95	24.18	0.63	0.36	1.10		1.96	0.59	6.47		0.81	0.62	1.04	
<GCSE/equivalent vs Higher education/Degree	Unadjusted	1.13	0.89	1.43	72.27	1.06	0.83	1.36	61.17	1.22	0.77	1.94	58.91	1.38	1.21	1.58	0
	**Basic adjustment**	**1.12**	**0.96**	**1.30**	**33.28**	**1.04**	**0.85**	**1.27**	**34.32**	**1.25**	**0.88**	**1.78**	**27.88**	**1.20**	**1.04**	**1.38**	**0**
	Full adjustment	0.85	0.76	0.96	3.24	0.70	0.42	1.17		3.22	1.01	10.27		0.86	0.66	1.12	
**Occupational class**																	
Intermediate vs Managerial/Admin/Professional	Unadjusted	1.07	0.97	1.18	0	1.04	0.93	1.17	0	0.99	0.74	1.35	17.14	1.15	1.00	1.32	0
	**Basic adjustment**	**1.04**	**0.94**	**1.15**	**0**	**1.02**	**0.91**	**1.15**	**0**	**0.96**	**0.68**	**1.37**	**29.88**	**1.12**	**0.98**	**1.28**	**0**
	Full adjustment	0.97	0.88	1.08	0	1.13	0.67	1.90		0.74	0.21	2.59		1.05	0.84	1.31	
Manual/Routine vs Managerial/Admin/Professional	Unadjusted	1.13	0.99	1.29	29.12	1.06	0.94	1.18	0	1.30	1.00	1.68	9.9	1.13	0.91	1.41	33.75
	**Basic adjustment**	**1.20**	**1.09**	**1.32**	**0**	**1.15**	**1.03**	**1.30**	**0**	**1.35**	**1.01**	**1.81**	**16.85**	**1.20**	**1.05**	**1.37**	**0**
	Full adjustment	1.03	0.93	1.15	0	1.29	0.81	2.06		0.75	0.27	2.13		1.07	0.85	1.35	
Other social class vs	Unadjusted	1.36	0.90	2.06	92.46	1.40	1.01	1.94	79.32	2.03	1.25	3.29	73.7	1.55	0.90	2.68	87.81

Managerial/ Admin/Professional	**Basic adjustment**	**1.48**	**1.10**	**2.00**	**81.34**	**1.51**	**1.18**	**1.93**	**56.45**	**2.44**	**1.71**	**3.49**	**45.49**	**1.64**	**1.10**	**2.46**	**72.22**
	Full adjustment	1.19	0.99	1.42	44.84	1.39	0.80	2.42		4.12	1.43	11.82		0.94	0.69	1.27	

Basic adjustment: sex, age, and ethnicity (where available)
Full adjustment: sex, age, and ethnicity (where available) , education, occupational class, UK Nation (where appropriate), household composition, and pre-pandemic self-reported health.

Empty I^2% column indicates only one study included

*Binary variable including Black, East Asian, Mixed, South Asian, and other ethnicity in 'non-White'

For peer review only

Summary of stratified results

		Any healthcare disruption			
Sex		OR	Lower CI	Upper CI	I2%
Female vs. Male	Overall	1.34	1.15	1.57	65.33
	Not shielding	1.32	1.09	1.61	75.25
	Shielding	1.48	1.20	1.83	0
	16-24y	2.21	1.61	3.03	3.99
	25-34y	1.45	0.86	2.43	63.72
	35-44y	1.48	1.14	1.92	
	45-54	1.97	1.61	2.42	0
	55-64	1.16	1.02	1.32	0
	75+	1.03	0.80	1.32	42.24
Age		OR	Lower CI	Upper CI	I2%
16-24y vs 45-54y	Overall	0.49	0.39	0.60	
	Not shielding	0.50	0.40	0.62	
	Shielding	0.64	0.23	1.78	
25-34y vs 45-54y	Overall	0.70	0.58	0.85	
	Not shielding	0.71	0.58	0.87	
	Shielding	0.86	0.34	2.16	
35-44y vs 45-54y	Overall	0.74	0.63	0.87	
	Not shielding	0.76	0.64	0.90	
	Shielding	0.48	0.24	0.96	
55-64y vs 45-54y	Overall	1.42	1.25	1.61	0
	Not shielding	1.37	1.20	1.57	0
	Shielding	1.32	0.80	2.17	0
65-74y vs 45-54y	Overall	1.78	1.56	2.02	0
	Not shielding	1.67	1.46	1.91	0
	Shielding	1.33	0.82	2.15	0
75y+ vs 45-54y	Overall	2.06	1.76	2.41	0
	Not shielding	1.96	1.66	2.33	0
	Shielding	1.07	0.65	1.78	0
Ethnicity		OR	Lower CI	Upper CI	I2%
Non-White vs White*	Overall	1.23	1.05	1.44	0

For peer review only

	Not shielding	0.96	0.62	1.48	73.47
	Shielding	1.56	0.97	2.49	0
	16-24y	1.24	0.84	1.82	0
	25-34y	0.70	0.47	1.04	0
	35-44y	1.42	0.94	2.12	
	45-54	1.71	1.20	2.44	0
	55-64	1.20	0.87	1.66	0
	75+	1.28	0.67	2.45	0
Black vs White	Overall	1.47	1.08	1.98	0
	Not shielding	0.84	0.38	1.83	72.85
	Shielding	1.49	0.59	3.78	0
	16-24y	1.15	0.51	2.59	0
	25-34y	0.74	0.30	1.86	16.69
	35-44y	2.11	0.87	5.12	
	45-54	1.99	0.93	4.25	15.25
	55-64	1.74	1.03	2.95	0
	75+	1.23	0.42	3.56	0
East Asian vs White	Overall	1.04	0.53	2.06	
	Not shielding	1.04	0.52	2.11	
	Shielding				
	16-24y	0.01	0.00	0.05	
	25-34y	0.57	0.12	2.62	
	35-44y	1.55	0.69	3.48	
	45-54	1.62	0.42	6.18	
	55-64	0.90	0.36	2.21	
	75+				
Mixed vs White	Overall	1.38	0.88	2.17	34.69
	Not shielding	1.28	0.88	1.86	0
	Shielding	1.89	0.64	5.55	0
	16-24y	2.50	1.25	5.02	0
	25-34y	1.09	0.61	1.95	0
	35-44y	2.47	0.88	6.95	
	45-54	1.01	0.48	2.14	
	55-64	1.19	0.56	2.51	0
	75+	1.47	0.34	6.42	22.46
South Asian vs White	Overall	1.02	0.84	1.24	0
	Not shielding	0.92	0.64	1.34	42.86
	Shielding	1.30	0.72	2.36	0
	16-24y	0.98	0.62	1.53	13.95

For peer review only

	25-34y	0.43	0.26	0.72	2.58
	35-44y	0.91	0.58	1.42	
	45-54	2.55	0.59	10.92	86.27
	55-64	0.90	0.47	1.74	19
	75+	1.11	0.40	3.12	0
Other Ethnicity vs White	Overall	0.72	0.25	2.07	64.12
	Not shielding	0.63	0.20	1.95	62.21
	Shielding	0.19	0.01	4.52	
	16-24y	0.18	0.00	15.35	88.56
	25-34y	0.57	0.10	3.20	70.09
	35-44y	1.52	0.36	6.41	
	45-54	1.12	0.37	3.38	
	55-64	0.49	0.12	1.96	
	75+	4.18	0.35	50.04	
Education		OR	Lower CI	Upper CI	I2%
A-level/equivalent vs Higher education/Degree	Overall	1.11	0.99	1.25	8.05
	Not shielding	1.02	0.85	1.23	47.74
	Shielding	0.92	0.66	1.30	0
	16-24y	1.39	0.96	2.01	0
	25-34y	0.97	0.55	1.71	52.33
	35-44y	1.48	1.00	2.18	
	45-54	1.10	0.86	1.40	0
	55-64	0.99	0.76	1.29	44.12
	75+	0.77	0.57	1.05	0
GCSE/equivalent vs Higher education/Degree	Overall	0.94	0.79	1.12	55.76
	Not shielding	0.93	0.79	1.10	47.54
	Shielding	0.80	0.60	1.06	0
	16-24y	0.93	0.36	2.40	83.45
	25-34y	1.05	0.53	2.07	70.84
	35-44y	1.19	0.86	1.64	
	45-54	1.00	0.70	1.44	60.4
	55-64	1.06	0.91	1.24	0
	75+	0.88	0.59	1.31	54.52
<GCSE/equivalent vs Higher education/Degree	Overall	1.12	0.96	1.30	33.28
	Not shielding	1.01	0.83	1.23	50.08
	Shielding	0.86	0.63	1.18	8.77
	16-24y	0.79	0.38	1.61	46.71
	25-34y	1.31	0.61	2.81	62.99

For peer review only

	35-44y	0.87	0.56	1.36	
	45-54	1.32	0.85	2.06	61.45
	55-64	1.18	0.97	1.43	0
	75+	0.98	0.78	1.24	0
**Occupational class**		**OR**	**Lower CI**	**Upper CI**	**I2%**
Intermediate vs Managerial/Admin/Professional	Overall	1.04	0.94	1.15	0
	Not shielding	1.04	0.94	1.15	0
	Shielding	0.86	0.59	1.25	13.43
	16-24y	0.88	0.55	1.41	0
	25-34y	1.25	0.86	1.81	0
	35-44y	1.13	0.81	1.58	
	45-54	1.13	0.92	1.39	0
	55-64	0.92	0.77	1.11	0
	75+	1.02	0.76	1.37	0
Manual/Routine vs Managerial/Admin/Professional	Overall	1.20	1.09	1.32	0
	Not shielding	1.20	1.08	1.33	0
	Shielding	0.94	0.71	1.24	0
	16-24y	1.14	0.74	1.75	0
	25-34y	1.55	0.97	2.48	36.45
	35-44y	1.23	0.88	1.71	
	45-54	1.04	0.85	1.27	0
	55-64	1.14	0.95	1.37	0
	75+	1.29	0.98	1.70	0
Other social class vs Managerial/Admin/Professional	Overall	1.48	1.10	2.00	81.34
	Not shielding	1.44	1.10	1.89	73.49
	Shielding	0.92	0.38	2.22	82.67
	16-24y	1.01	0.34	2.95	79.64
	25-34y	2.09	1.40	3.13	0
	35-44y	2.16	1.34	3.48	
	45-54	2.05	0.98	4.29	85.15
	55-64	1.73	1.28	2.33	64.79
	75+	1.02	0.62	1.69	0

Adjusted for sex, age, and ethnicity (where available)

Empty I^2% column indicates only one study included

*Binary variable including Black, East Asian, Mixed, South Asian, and other ethnicity in 'non-White'

For peer review only

STROBE Statement—Checklist of items that should be included in reports of ***cohort studies***

	Item No	Recommendation	Page No
**Title and abstract**	1	(*a*) Indicate the study's design with a commonly used term in the title or the abstract<br>(*b*) Provide in the abstract an informative and balanced summary of what was done and what was found	1-2
**Introduction**			
Background/rationale	2	Explain the scientific background and rationale for the investigation being reported	3-4
Objectives	3	State specific objectives, including any prespecified hypotheses	4
**Methods**			
Study design	4	Present key elements of study design early in the paper	4-5
Setting	5	Describe the setting, locations, and relevant dates, including periods of recruitment, exposure, follow-up, and data collection	4-5 & supplementary
Participants	6	(*a*) Give the eligibility criteria, and the sources and methods of selection of participants. Describe methods of follow-up<br>(*b*) For matched studies, give matching criteria and number of exposed and unexposed	4 & supplementary
Variables	7	Clearly define all outcomes, exposures, predictors, potential confounders, and effect modifiers. Give diagnostic criteria, if applicable	4-7 & supplementary
Data sources/ measurement	8*	For each variable of interest, give sources of data and details of methods of assessment (measurement). Describe comparability of assessment methods if there is more than one group	4-7 & supplementary
Bias	9	Describe any efforts to address potential sources of bias	4 & 8
Study size	10	Explain how the study size was arrived at	4 & supplementary
Quantitative variables	11	Explain how quantitative variables were handled in the analyses. If applicable, describe which groupings were chosen and why	7-8 & supplementary
Statistical methods	12	(*a*) Describe all statistical methods, including those used to control for confounding<br>(*b*) Describe any methods used to examine subgroups and interactions<br>(*c*) Explain how missing data were addressed<br>(*d*) If applicable, explain how loss to follow-up was addressed<br>(*e*) Describe any sensitivity analyses	7-8 & supplementary
**Results**			
Participants	13*	(a) Report numbers of individuals at each stage of study—eg numbers potentially eligible, examined for eligibility, confirmed eligible, included in the study, completing follow-up, and analysed<br>(b) Give reasons for non-participation at each stage<br>(c) Consider use of a flow diagram	9 & supplementary
Descriptive data	14*	(a) Give characteristics of study participants (eg demographic, clinical, social) and information on exposures and potential confounders<br>(b) Indicate number of participants with missing data for each variable of interest<br>(c) Summarise follow-up time (eg, average and total amount)	9 & supplementary
Outcome data	15*	Report numbers of outcome events or summary measures over time	9-12 & supplementary

For peer review only - http://bmjopen.bmj.com/site/about/guidelines.xhtml

Main results	16	(*a*) Give unadjusted estimates and, if applicable, confounder-adjusted estimates and their precision (eg, 95% confidence interval). Make clear which confounders were adjusted for and why they were included<br>(*b*) Report category boundaries when continuous variables were categorized<br>(*c*) If relevant, consider translating estimates of relative risk into absolute risk for a meaningful time period	9-12 & supplementary
Other analyses	17	Report other analyses done—eg analyses of subgroups and interactions, and sensitivity analyses	12& supplementary
**Discussion**			
Key results	18	Summarise key results with reference to study objectives	13
Limitations	19	Discuss limitations of the study, taking into account sources of potential bias or imprecision. Discuss both direction and magnitude of any potential bias	14-15
Interpretation	20	Give a cautious overall interpretation of results considering objectives, limitations, multiplicity of analyses, results from similar studies, and other relevant evidence	15-16
Generalisability	21	Discuss the generalisability (external validity) of the study results	14-15
**Other information**			
Funding	22	Give the source of funding and the role of the funders for the present study and, if applicable, for the original study on which the present article is based	17

*Give information separately for exposed and unexposed groups.

Note: An Explanation and Elaboration article discusses each checklist item and gives methodological background and published examples of transparent reporting. The STROBE checklist is best used in conjunction with this article (freely available on the Web sites of PLoS Medicine at http://www.plosmedicine.org/, Annals of Internal Medicine at http://www.annals.org/, and Epidemiology at http://www.epidem.com/). Information on the STROBE Initiative is available at http://www.strobe-statement.org.

For peer review only - http://bmjopen.bmj.com/site/about/guidelines.xhtml

BMJ Open

Inequalities in healthcare disruptions during the COVID-19 pandemic: evidence from 12 UK population-based longitudinal studies

Journal:	*BMJ Open*
Manuscript ID	bmjopen-2022-064981.R1
Article Type:	Original research
Date Submitted by the Author:	25-Aug-2022
Complete List of Authors:	Maddock, Jane ; University College London Faculty of Population Health Sciences, Cardiovascular Science<br>Parsons, Sam; UCL IOE, Centre for Longitudinal Studies<br>Di Gessa, Giorgio; UCL, Institute of Epidemiology and Health Care<br>Green, Michael; University of Glasgow MRC/CSO Social and Public Health Sciences Unit<br>Thompson, Ellen J.; King's College London Department of Twin Research and Genetic Epidemiology<br>Stevenson, Anna J.; The University of Edinburgh Centre for Genomic and Experimental Medicine<br>Kwong, Alex; MRC Integrative Epidemiology Unit; The University of Edinburgh Division of Psychiatry<br>McElroy, Eoin; University of Leicester, Department of Neuroscience, Psychology and Behaviour<br>Santorelli, Gillian; Bradford Teaching Hospitals NHS Foundation Trust<br>Silverwood, Richard; UCL Institute of Education Centre for Longitudinal Studies<br>Captur, Gaby; MRC Unit for Lifelong Health and Ageing, UCL<br>Chaturvedi, Nishi; University College London<br>Steves, Claire J.; King's College London Department of Twin Research and Genetic Epidemiology<br>Steptoe, Andrew; University College London, Department of Behavioural Science and Health<br>Patalay, Praveetha; University College London, Population Health and Experimental Medicine<br>Ploubidis, George; UCL Institute of Education Centre for Longitudinal Studies<br>Katikireddi, Srinivasa; University of Glasgow
**Primary Subject Heading**:	Epidemiology
Secondary Subject Heading:	Public health
Keywords:	COVID-19, EPIDEMIOLOGY, PUBLIC HEALTH, SOCIAL MEDICINE

SCHOLARONE™
Manuscripts

I, the Submitting Author has the right to grant and does grant on behalf of all authors of the Work (as defined in the below author licence), an exclusive licence and/or a non-exclusive licence for contributions from authors who are: i) UK Crown employees; ii) where BMJ has agreed a CC-BY licence shall apply, and/or iii) in accordance with the terms applicable for US Federal Government officers or employees acting as part of their official duties; on a worldwide, perpetual, irrevocable, royalty-free basis to BMJ Publishing Group Ltd ("BMJ") its licensees and where the relevant Journal is co-owned by BMJ to the co-owners of the Journal, to publish the Work in this journal and any other BMJ products and to exploit all rights, as set out in our licence.

The Submitting Author accepts and understands that any supply made under these terms is made by BMJ to the Submitting Author unless you are acting as an employee on behalf of your employer or a postgraduate student of an affiliated institution which is paying any applicable article publishing charge ("APC") for Open Access articles. Where the Submitting Author wishes to make the Work available on an Open Access basis (and intends to pay the relevant APC), the terms of reuse of such Open Access shall be governed by a Creative Commons licence – details of these licences and which Creative Commons licence will apply to this Work are set out in our licence referred to above.

Other than as permitted in any relevant BMJ Author's Self Archiving Policies, I confirm this Work has not been accepted for publication elsewhere, is not being considered for publication elsewhere and does not duplicate material already published. I confirm all authors consent to publication of this Work and authorise the granting of this licence.

For peer review only

Inequalities in healthcare disruptions during the COVID-19 pandemic: evidence from 12 UK population-based longitudinal studies

Jane Maddock*[1^], Sam Parsons[2^] Giorgio Di Gessa[3^], Michael J. Green[4^], Ellen J. Thompson[5^], Anna J. Stevenson[6], Alex S.F. Kwong[6,7], Eoin McElroy[8], Gillian Santorelli[9], Richard J. Silverwood[2], Gabriella Captur[1], Nishi Chaturvedi[1], Claire J. Steves[5], Andrew Steptoe[3], Praveetha Patalay[1,2], George B. Ploubidis[2], Srinivasa Vittal Katikireddi[4]

^Joint first authors

1 MRC Unit for Lifelong Health and Ageing, University College London

2 Centre for Longitudinal Studies, UCL Social Research Institute, University College London

3 Institute of Epidemiology and Health Care, University College London

4 MRC/CSO Social & Public Health Sciences Unit, University of Glasgow

5 Department of Twin Research and Genetic Epidemiology, School of Life Course Sciences, King's College London

6 Division of Psychiatry, University of Edinburgh

7 MRC Integrative Epidemiology Unit, University of Bristol

8 Department of Neuroscience, Psychology and Behaviour, University of Leicester

9 Born in Bradford, Bradford Institute for Health Research, Temple Bank House, Bradford Royal Infirmary

*Correspondence to:

Dr Jane Maddock

jane.maddock@ucl.ac.uk

For peer review only

For peer review only - http://bmjopen.bmj.com/site/about/guidelines.xhtml

Abstract

Objectives: We investigated associations between multiple socio-demographic characteristics (sex, age, occupational social class, education, and ethnicity) and self-reported healthcare disruptions during the early stages of the COVID-19 pandemic.

Design: Co-ordinated analysis of prospective population surveys.

Setting: Community-dwelling participants in the United Kingdom between April 2020 up to January 2021.

Participants: Over 68,000 participants from 12 longitudinal studies.

Outcomes: Self-reported healthcare disruption to medication access, procedures, appointments.

Results: Prevalence of healthcare disruption varied substantially across studies: between 6% and 32% reported any disruption, with 1% to 10% experiencing disruptions in medication, 1% to 17% experiencing disruption in procedures and 4% to 28% experiencing disruption in clinical appointments. Females (Odd Ratio (OR): 1.27 [95%CI: 1.15,1.40]; I^2=54%), older persons (e.g., OR: 1.39 [1.13,1.72]; I^2=77% for 65-75y vs 45-54y), and Ethnic minorities (excluding White minorities) (OR: 1.19 [1.05,1.35]; I^2=0% vs White) were more likely to report healthcare disruptions. Those in a more disadvantaged social class were also more likely to report healthcare disruptions (e.g., OR: 1.17 [1.08, 1.27]; I^2=0% for manual/routine vs managerial/professional), but no clear differences were observed by education. We did not find evidence that these associations differed by shielding status or age.

Conclusions: Healthcare disruptions during the COVID-19 pandemic could contribute to the maintenance or widening of existing health inequalities.

Keywords: Healthcare Disparities; Health Services Accessibility; Longitudinal Studies; United Kingdom; Socioeconomic Factors; Ethnicity; Social Class

For peer review only

Strengths and limitations of this study

- We conducted co-ordinated primary analyses in 12 UK longitudinal population studies, and pooled results using a random effects meta-analysis.
- Use of multiple studies increased statistical power to look at subpopulations such as ethnic minority groups across cohorts and allowed for greater examination of how inequalities were patterned by age.
- Most studies were weighted to be representative of their target ages in the UK population, and findings were robust to excluding those that were not.
- We did not adjust for whether respondents needed healthcare, so the inequalities observed may be at least partly attributable to inequalities in needing healthcare.
- Data on pre-pandemic healthcare disruption was not available, so we could not tell if inequalities in healthcare disruption had widened or narrowed during the pandemic.

Introduction

The coronavirus disease 2019 (COVID-19) pandemic has affected all aspects of society. Health systems world-wide have faced major disruption as they respond to large increases in demand arising from the COVID-19 disease.[1–5] Furthermore, healthcare access has been reduced by governmental control measures and the public's fear of contracting infection.[6] Disruptions may have both short and long-term health consequences as preventive treatments are foregone, disease surveillance is interrupted and disease diagnoses are delayed. While the disruption of health systems can impact the entire population, it has become apparent that not all groups have been affected equally. For example, recent evidence has demonstrated that both elective and emergency hospital admissions vary by socioeconomic deprivation and ethnic minority quintiles, with the more deprived areas showing a large fall in elective admissions, and areas with high ethnic minority populations showing larger falls in emergency admissions.[5] Understanding the impacts of the pandemic on health systems and on equity of healthcare access is therefore a major policy priority.

In the UK, the National Health Service (NHS) provides free healthcare and prioritises equity of delivery. However, the UK's relatively high COVID-19 burden and associated repeated lockdown

For peer review only - http://bmjopen.bmj.com/site/about/guidelines.xhtml

measures have raised concerns that the health system may not be providing accessible care to those who need it most. Recent reports from NHS Digital indicate a large increase in those waiting 12 months or more for elective treatments in February 2021 compared to March 2020.[7] Furthermore, despite decreases in attendance at accident and emergency services,[4] the number of patients waiting over 12 hours for admission was 34% higher in January 2021 than January 2020. Disruption to pharmacological treatments has also been reported with delays in accessing medication.[8,9] However, a comprehensive assessment of inequalities in healthcare disruption in the community is lacking.

It is well known that health systems do not meet the needs of all social groups equitably, with marked health inequalities by sex, ethnicity, and socioeconomic position.[10,11] For example, the inverse care law demonstrates that health service provision is often not allocated according to need, with more socioeconomically deprived areas relatively under-served.[12] Given the barriers that some social groups face in accessing high quality healthcare, there is considerable concern that disadvantaged groups (e.g., ethnic minorities) will be disproportionately impacted by healthcare disruption during the COVID-19 pandemic, as some emerging evidence suggests.[13,14]

Harnessing multiple longitudinal studies allows inequalities to be studied in detail by improving statistical power and allows consistency of findings to be investigated. We therefore aimed to investigate inequalities in healthcare disruption during the COVID-19 pandemic in 12 population-based longitudinal studies, to help inform targeting of policy responses as we move out of the acute phase of the pandemic. We investigate healthcare disruptions (including prescription or medication access, procedures or surgery, clinical appointments) by sex, age, ethnicity, education, and occupational social class and we explore whether associations differ by age, or for those who have been recommended to 'shield' due to clinical vulnerability.

Methods

Design

The UK National Core Studies – Longitudinal Health and Wellbeing programme aims to draw together data from multiple UK population-based longitudinal studies to answer questions relevant to the

For peer review only - http://bmjopen.bmj.com/site/about/guidelines.xhtml

pandemic response. By coordinating analyses within each study and statistically pooling results in a meta-analysis, we can provide robust evidence to understand healthcare disruptions during the pandemic.

Participants

Data were from 12 UK population studies which had conducted surveys both before and during the COVID-19 pandemic. Details of the design, sample frames, current age range, timing of the COVID-19 surveys, response rates, and analytical sample size are available in Supplementary Table S1.

Our population of interest is the current UK population aged 16 years or older. The following studies are considered to be nationally representative samples of their target age-groups: the Millennium Cohort Study (MCS);[15] Next Steps (NS);[16] the 1970 British Cohort Study (BCS);[17] the National Child Development Study (NCDS);[18] the National Survey of Health and Development (NSHD);[19,20] Understanding Society (USOC);[21] and the English Longitudinal Study of Ageing (ELSA).[22] We also included the Avon Longitudinal Study of Parents and Children (ALSPAC-G1);[23] the parents of the ALSPAC-G1 cohort which we refer to as ALSPAC-G0;[24] the Born in Bradford study (BIB);[25,26] Generation Scotland: the Scottish Family Health Study (GS);[27] and the UK Adult Twin Registry (TwinsUK).[28,29] We present results from all 12 studies in the main manuscript and results restricted to representative samples in Supplementary File 1.

We can further categorise these studies into age homogenous birth cohorts (where all individuals were of similar age within each cohort) and age heterogeneous studies (each covering a range of age groups). The age homogenous studies include: MCS; ALSPAC-G1; NS; BCS; NCDS; and NSHD. The age heterogenous studies include: BIB; USOC; GS; ALSPAC-G0; TwinsUK; and ELSA. Analytical samples were defined within each study based on respondents who had no missing data on at least one healthcare disruption outcome in a COVID-19 survey and on a minimum set of covariates (sex, ethnicity, and age where relevant). Most studies were weighted to be representative of their target populations accounting for differential non-response.[20,30,31] Weights were not available for BIB or TwinsUK. Studies were ordered for presentation by age of sample (youngest to oldest), with the age homogenous cohorts first, followed by the age heterogenous studies. Missing data within surveys was

For peer review only - http://bmjopen.bmj.com/site/about/guidelines.xhtml

generally low, especially for healthcare disruption variables, but approximately 5-10% of respondents across studies were excluded due to missing baseline covariates.

Ethical approval

Ethics statements and data access details for each study can be found in Supplementary Table S2.

Measures

Below we describe the overall approach to measuring each variable in the analysis.

Outcomes

We assessed self-reported disruptions to prescriptions or medication access; procedures or surgery; and appointments (e.g., with a GP or outpatient services); and a combined variable indicating disruptions to any of the afore mentioned. Any deviation from planned or existing treatment was coded as a disruption, regardless of the reason for the disruption. The wording of the questions was the same for MCS, NS, BCS, NCDS and NSHD. There was variation in how the questions were asked in the other studies. Full details of the questions and coding used within each study are available in Supplementary File 2. ALSPAC did not have information about prescriptions or medication access. BIB did not have information about procedures or surgery. TwinsUK did not have information about procedures or surgery or appointments. Where multiple pandemic survey waves had been included, we coded for any disruptions reported up to and including the most recent. This meant at least 7 months of follow-up for most studies (GS had five and ELSA four, while ALSPAC had the longest follow-up period at nine months). Supplementary Table S3 shows how the prevalence for any experience of each disruption accumulated across the six USOC surveys. The majority of those who experienced each type of healthcare disruption had already experienced it by the end of May 2020.

Indicators of inequality

We assessed inequalities associated with key sociodemographic characteristics i.e., sex, age, ethnicity, education, and occupational social class. For age, we considered age-groups categorised as: 16-24; 25-34; 35-44; 45-54; 55-64; 65-74; and 75+ years. Depending on the level of detail of ethnicity available, we examined both a binary (White [including White minorities] vs Ethnic minorities [excluding White minorities]) and a finer categorisation of ethnicity (White, South Asian, Black, Mixed, Other Asian,

For peer review only - http://bmjopen.bmj.com/site/about/guidelines.xhtml

Other Ethnic Minority). For education we distinguished between degree or equivalent; A-level or equivalent (i.e., post-compulsory schooling qualifications); GCSE or equivalent (i.e., qualifications for completing compulsory schooling); and fewer or no qualifications. We also examined occupational class with the following categories (based on different coding schemes in different studies): Professional/Managerial; Intermediate; Routine/Manual; and Other (which included never/long-term non-employed and, in some studies, respondents who could not be classified elsewhere). Respondents' education and occupational class were not available in the MCS or ALSPAC-G1, so we considered parental education or household social class. For full details, see Supplementary File 2.

Moderators

We decided *a priori* to examine modification by age and clinical vulnerability to COVID-19 to see whether inequalities varied by life-stage or were particularly acute for those with higher healthcare needs and at higher risk from COVID-19 harms. For moderation by age, the age-heterogeneous studies split their samples into the age-bands covered, while age homogeneous cohorts were included within the appropriate age bands (see above for banding). In the UK, clinically extremely vulnerable people were advised to stay at home ('shield') during the pandemic. Respondents were directly asked whether they had received a letter from the NHS advising them to stay at home and protect themselves. Specific survey questions can be found in Supplementary File 2.

Other variables

The following covariates were also included where relevant and available within each study: UK Nation (i.e., England, Scotland, Wales, or Northern Ireland); household composition (based on partnership status and whether there were children in the household); and pre-pandemic self-reported health (good vs poor).

Analysis

Within each study, distributions of sociodemographic characteristics and healthcare disruption were examined. Then, each healthcare disruption outcome was regressed on each indicator of inequality (i.e., sex, age, ethnicity, education, and occupational class). Unadjusted associations are included in Supplementary File 3. Since our aim was primarily to describe inequalities, we focus on presenting

For peer review only

associations with minimal adjustment only for sex, age, and ethnicity when applicable. To assess whether associations were independent of other related factors, we also provide results in Supplementary File 3 for any healthcare disruption which additionally adjust for: education, occupational class, UK Nation (where appropriate), household composition, and pre-pandemic self-reported health. Moderation by age and shielding status was assessed using stratified models.

Results were then meta-analysed for each outcome for the full sample, and within age and shielding strata. We used a random effects meta-analysis with restricted maximum likelihood. For stratified results, a test of group differences was performed using the subgroup meta-analysis command. We report heterogeneity using the I^2 statistic (0% indicates low variation between estimates across studies, while values closer to 100% indicate greater heterogeneity).

Finally, in sensitivity analyses we restricted meta-analyses to representative studies (MCS, NS, BCS, NCDS and NSHD, USOC and ELSA). Meta-analyses were conducted in Stata 16 (V.16).[32]

Patient and public involvement

None.

Results

Descriptive statistics

The distribution of demographic and socio-economic characteristics within each study is presented in Table 1. A total of 68,912 participants were included in the coordinated analysis. Due to study design, participants from BIB were all female, as were the vast majority (89.4%) from TwinsUK. The age ranged from 16 years in BIB and USOC to 90+ years in TwinsUK and ELSA.

Overall, the prevalence of any healthcare disruption ranged from 6.4% in TwinsUK to 31.8% in USOC (Figure 1). Table 2 shows that disruptions to medical appointments were most common, ranging from 3.5% (ELSA) to 28.4% (USOC). Disruptions in prescriptions or medication access varied from 1.2% (BIB) to 10.4% (GS). Disruptions to procedures or surgery were least common ranging from 0.7% (MCS) to 16.8% (ELSA).

For peer review only - http://bmjopen.bmj.com/site/about/guidelines.xhtml

Table 1. Percent (and N) distribution of demographic and socio-economic characteristics by study

		MCS	*ALSPAC G1*	*NS*	*BCS70*	*NCDS*	*NSHD*	*BIB*	*USOC*	*GS*	*ALSPAC G0*	*TWINS UK*	*ELSA*
**Total analytic N**		3,147	3,430	3,311	5,175	5,747	1,569	1,726	13,253	17,139	3,625	4,282	6,508
**Female**		65.0 (2,045)	65.3 (2,240)	64.8 (2,145)	57.9 (2,994)	53.7 (3,086)	52.6 (825)	100.0 (1,726)	57.9 (7,668)	67.0 (11,476)	73.1 (2,651)	89.4 (3,830)	56.3 (3,663)
**Mean Age in 2020 (range)**		19.5 (18.7-20.1)	28.4 (27-29)	30.6 (29.9-31.4)	50.5 (50.4-50.6)	62.6 (62.5-62.7)	74	37.5 (16-54)	51.1 (16-96.2)	57.0 (18-100)	59.4 (45-89)	61.2 (22-96)	69.3 (52-90+)
**Ethnicity**	*White*	86.1 (2,708)	98.4 (3,330)	74.6 (2,470)	NA	NA	NA	37.8 (653)	98.3 (16,843)	87.2 (11,561)	98.4 (3,567)	97.1 (4,156)	95.9 (6,239)
	South Asian	7.6 (240)	NA	15.0 (496)	NA	NA	NA	56.1 (968)	0.4 (70)	6.7 (885)	NA	0.7 (28)	2.1 (135)
	East Asian	1.0 (30)	NA	NA	NA	NA	NA	NA	0 .3 (51)	1.2 (155)	NA	0.1 (3)	NA
	Black	2.6 (83)	NA	3.8 (127)	NA	NA	NA	2.0 (34)	0.1 (21)	2.5 (334)	NA	1.1 (45)	1.2 (75)
	Mixed	2.4 (76)	NA	4.6 (152)	NA	NA	NA	1.4 (24)	0.6 (105)	1.8 (241)	NA	0.9 (38)	0.9 (59)
	Other	0.3 (10)	NA	2.0 (66)	NA	NA	NA	2.7 (47)	0.3 (49)	0.6 (77)	NA	0.3 (12)	NA
	All ethnic minorities	13.9 (439)	2.9 (100)	25.4 (841)	NA	NA	NA	62.2 (1,073)	1.3 (226)	12.8 (1692)	1.6 (58)	2.9 (126)	4.1 (269)
**Education**	*Higher Education or Degree*	55.9 (1,758)	29.0 (994)	48.9 (1,620)	46.6 (2,411)	46.0 (2,646)	29.0 (994)	35.1 (556)	50.7 (8,602)	47.1 (6,238)	29.7 (1,075)	55.7 (2,386)	25.6 (1,666)
	A-level or equivalent	15.0 (473)	35.1 (1,203)	23.4 (773)	14.2 (733)	18.0 (1,034)	35.1 (1203)	17.2 (273)	35.9 (6,096)	11.6 (1,543)	29.7 (1,078)	11.6 (498)	27.6 (1,798)
	GCSE or equivalent	19.5 (615)	26.1 (896)	19.0 (628)	23.4 (1,209)	22.8 (1,311)	26.1 (896)	22.3 (354)	6.2 (1046)	25.2 (3,341)	30.3 (1,098)	20.5 (877)	22.3 (1,452)
	<GCSE or none	9.6 (301)	9.83 (337)	8.8 (290)	15.9 (822)	13.2 (756)	9.8 (337)	25.5 (405)	7.2 (1,214)	16.1 (2,131)	10.3 (374)	12.2 (521)	24.5 (1,592)
**Social Class**	*Managerial, Admin, Professional*	51.3 (1,614)	18.0 (616)	47.6 (1,575)	42.7 (2,209)	23.0 (1,319)	18 (616)	31.2 (475)	81.0 (10,716)	35.0 (4,639)	13.4 (486)	NA	32.4 (2,111)
	Intermediate	15.4 (484)	46.2 (1,583)	18.9 (625)	21.1 (1,091)	14.9 (856)	46.1 (1,583)	35.7 (545)	14.4 (1,906)	17.1 (2,264)	41.2 (1,492)	NA	23.0 (1,497)
	Manual/Routine	18.9 (595)	35.3 (1,212)	15.0 (495)	19.5 (1,009)	16.5 (948)	35.3 (1,212)	25.3 (386)	4.4 (581)	20.1 (2,663)	44.6 (1,617)	NA	28.2 (1,834)
	Other	14.4 (454)	0.6 (19)	18.6 (616)	16.7 (866)	45.7 (2,624)	0.6 (19)	7.8 (119)	0.2 (27)	27.8 (3687)	0.8 (30)	NA	16.4 (1,066)
**Instructed to Shield**		2.5 (79)	NA	3.3 (110)	5.2 (267)	6.9 (393)	8.8 (101)	7.6 (131)	6.2 (825)	7.8 (1,332)	NA	5.9 (252)	16.3 (1,062)

For peer review only

Sources: MCS (Millennium Cohort Study); ALSPAC G1 (Children of the Avon Longitudinal Study of Parents and Children); NS (Next Steps); BCS 70 (1970 British Cohort Study), NCDS (National Child Development Study); NSHD (National Survey of Health and Development); BIB (Born in Bradford); USOC (Understanding Society); GS (Generation Scotland: the Scottish Family Health Study); ALSPAC G0 (parents of ALSPAC); TWINS UK (UK Adult Twin Registry); ELSA (English Longitudinal Study of Ageing). Notes: Studies are ordered by age homogeneity/heterogeneity and mean age of respondents at the time of the interview. Samples for each study restricted to respondents with non-missing information on healthcare disruptions and valid information on sex, social class, education and (where applicable) age and ethnicity. All information about how data were collected and variables were coded is available in Supplementary File 2. NA = Not available/Info not collected. Unweighted data.

For peer review only

For peer review only - http://bmjopen.bmj.com/site/about/guidelines.xhtml

Table 2. Percent prevalence (and 95% confidence intervals) of healthcare disruptions during the pandemic, by study

	MCS	*ALSPAC (G1)*	*NS*	*BCS70*	*NCDS*	*NSHD*	*BIB*	*USOC*	*GS*	*ALSPAC (G0)*	*TWINS UK*	*ELSA*
**Prescription/ medication access**	4.0 (2.3-5.5)	NA	3.8 (2.3-5.3)	3.4 (2.7-4.2)	2.4 (1.8-3.0)	2.2 (1.3-3.8)	1.2 (0.7-1.7)	5.5 (5.0-6.1)	10.4 (9.9-10.9)	NA	2.9 (2.5-3.3)	0.8 (0.6-1.2)
**Procedures or surgery**	0.7 (0.0-1.2)	1.6 (1.2-2.1)	2.1 (0.0-3.8)	1.0 (0.7-1.2)	2.8 (2.0-3.5)	2.5 (1.4-4.4)	NA	12.3 (11.6-13.0)	2.1 (1.9-2.4)	2.9 (2.1-3.9)	NA	16.8 (15.7-17.9)
**Appointments**	6.2 (4.9-7.6)	11.7 (10.3-13.2)	7.3 (5.6-9.0)	10.6 (9.2-12.1)	12.1 (10.9-13.3)	12.0 (9.3-15.6)	8.6 (7.4-10.1)	28.4 (27.4-29.4)	16.6 (16.0-17.1)	14.4 (12.8-16.2)	NA	3.5 (2.9-4.1)
**Any healthcare disruption**	10.1 (8.1-12.1)	15.9 (14.3-17.6)	12.8 (10.3-15.4)	14.3 (12.7-15.9)	16.7 (15.2-18.2)	16.4 (13.2-20.2)	9.4 (8.1-10.9)	31.8 (30.8-32.8)	25.3 (24.6-25.9)	19.9 (18.1-21.9)	6.35 (5.9-7.2)	19.5 (18.3-20.8)

Sources: MCS (Millennium Cohort Study); ALSPAC G1 (Children of the Avon Longitudinal Study of Parents and Children); NS (Next Steps); BCS 70 (1970 British Cohort Study), NCDS (National Child Development Study); NSHD (National Survey of Health and Development); BIB (Born in Bradford); USOC (Understanding Society); GS (Generation Scotland: the Scottish Family Health Study); ALSPAC G0 (parents of ALSPAC); TWINS UK (UK Adult Twin Registry); ELSA (English Longitudinal Study of Ageing). Notes: Studies are ordered by age homogeneity/heterogeneity and mean age of respondents at the time of the interview. Samples for each study restricted to respondents with non-missing information on healthcare disruptions and valid information on sex, social class, education and (where applicable) age and ethnicity. All information about how data were collected and variables were coded is available in Supplementary File 2. TWINSUK had an additional question: “Have you experienced healthcare disruption as a result of the COVID-19 pandemic?” This data was also used to derive the ‘any healthcare disruption’ variable for TWINSUK. NA = Not available/Info not collected. Weighted data where applicable

For peer review only

The following sections describe results adjusted for sex, age, and ethnicity when applicable. Unadjusted results and results adjusted for education, occupational class, UK Nation (where appropriate), household composition, and pre-pandemic self-reported health can be found in Supplementary File 3. The associations were largely robust to further adjustment.

Sex and healthcare disruptions

Across all studies females were generally more likely to report any healthcare disruptions than males (Supplementary Table S4 for details).

Pooled results from the meta-analysis demonstrate that females had increased odds of any healthcare disruption compared with males (OR: 1.27 [95%CI: 1.15,1.40]; I^2=54%, figure 2, supplementary file 3). Similar associations were observed for disruptions to appointments (OR: 1.33 [95%CI: 1.17,1.52]; I^2=60%). The association between sex and the less prevalent disruptions to procedures and medications crossed the null (Supplementary File 3 and Figure 2).

There were differences in the association between sex and healthcare disruption when stratified by age (p<0.001. Supplementary File 3). The odds of having any healthcare disruption for females was highest among 16–24-year-olds (OR: 2.22 [95% CI 1.63, 3.02]; I^2=0%, and Supplementary File 3). An association between sex and healthcare disruption was observed up to age 54 years but there were no clear associations among those aged 55 years and above. There was no evidence that the association between sex and healthcare disruption differed by shielding and non-shielding groups (Supplementary File 3).

<Figure 1 and 2 about here>

Age and healthcare disruptions

A higher prevalence of having any healthcare disruption was observed among older participants of the national birth cohorts where the same questionnaire was used (Figure 1). This age difference was also observed among the ALSPAC studies and for other age-heterogenous studies as seen in Supplementary Table S4.

For peer review only

For peer review only - http://bmjopen.bmj.com/site/about/guidelines.xhtml

267 The meta-analysis including age-heterogenous studies were supportive of age differences for any healthcare disruptions e.g., OR: 1.39 [1.13,1.72]; I^2=77% for 65-75y vs 45-54y (Figure 3, Supplementary File 3). Disruptions seemed less likely in younger age groups and more likely among older age groups, though some estimates cross the null and had high heterogeneity, which may be because of few studies in specific age categories (Figure 3, Supplementary File 3). Associations for disruptions to medical appointments and procedures or surgery showed these age differences more clearly (Figure 3, Supplementary File 3).

There were no clear differences in the association with age and any healthcare disruption by shielding status. However, for those who were shielding, confidence intervals were wide (Supplementary File 3). The magnitude for the association of healthcare disruption among 75-year-olds and above vs 45–54-year-olds was higher among the non-shielding group (OR: 1.61 [95%CI: 1.17,2.22]; I^2=79%) compared with the shielding group (OR: 0.83 [95%CI:0.51,1.37]; I^2=83%, Supplementary File3).

<Figure 3 about here>

Ethnicity and healthcare disruptions

Among the studies that had data on ethnicity, between 7.8% (BIB) and 31.9% (USOC) of the White groups reported healthcare disruption. Between 8.3% (TWINSUK) and 23.6% (GS) of Ethnic minority groups reported having any healthcare disruption (Supplementary Table S4).

In meta-analysis, Ethnic minorities compared to White groups had increased odds of any healthcare disruption (OR: 1.19 [1.05,1.35]; I^2=0%, Figure 4 and Supplementary File 3). This association was less clear for specific domains of healthcare disruption (Figure 4, Supplementary File 3). Among the studies that had a finer categorisation of ethnicity, only the Black ethnic groups had clearly raised odds for any healthcare disruption compared with White groups (OR: 1.38 [1.03,1.84]; I^2=0%). Associations with healthcare disruption were less evident for other ethnic groups but were imprecisely estimated (Figure 4, Supplementary File 3).

For peer review only - http://bmjopen.bmj.com/site/about/guidelines.xhtml

There were no major differences in associations between ethnicity and any healthcare disruption by age, though this may simply be due to low power as confidence intervals were wide (Supplementary File 3). The clearest associations with Ethnic minority groups were within the 35-44- and 45-74-year age ranges (OR:1.31 [1.01,1.71]; I^2=0% and OR:1.61 [1.16,2.22]; I^2=0%). The mixed ethnicity group were also at particular risk for disruption in the 16–24-year age range too (OR:2.50 [1.25,5.02]; I^2=0%). The magnitude for the association between any healthcare disruption among Ethnic minority groups vs. White groups was higher among those who were shielding (OR: 1.56[1.01 to 2.39]; compared to OR: 1.06[0.86 to 1.31] for non-shielding). This observation was consistent across more granular ethnicity categories, but confidence intervals were wide (Supplementary File 3).

<Figure 4 about here>

Education and healthcare disruptions

There was no clear pattern in the prevalence of healthcare disruption across education levels. For example, in USOC 29.7% of those with any healthcare disruption had a degree or equivalent and 39% had no school-leaving qualifications. In TWINSUK 9.9% of those with any healthcare disruption had a degree or equivalent and 6.1% had no school-leaving (Supplementary Table S4).

In meta-analysis, we did not observe clear associations between education level and healthcare disruption, other than that those without school-leaving qualifications had raised odds of disruptions to procedures or surgery (OR: 1.26 [1.11,1.44]; I^2=0%; Supplementary File 3 and Figure 5). We did not observe differences by age or shielding status (Supplementary File 3).

<Figure 5 about here>

Occupational class and healthcare disruptions

The prevalence of any healthcare disruption ranged between 9.7% (BIB) and 25.7% (USOC) among the Professional/Managerial social class and between 9.3% (BIB) and 27.6% (USOC) for the Manual/Routine social class (Supplementary Table S4).

For peer review only - http://bmjopen.bmj.com/site/about/guidelines.xhtml

Results from meta-analysis show that those in a more disadvantaged occupational class were more likely to report any healthcare disruptions (e.g., OR: 1.17 [95%CI: 1.08, 1.27]; I^2=0% for Manual/Routine compared with Professional/Managerial, Figure 6, Supplementary File 3). The OR was greatest for the non-employed occupational class category (OR: 1.51 [95%CI: 1.12,2.04]), however the I^2 was also large (80%). This implies considerable between study heterogeneity, though two of the four individual studies (MCS and ELSA) that did not show clear associations for this category were at the extremes of the age range considered. Similar associations were seen for domains of healthcare disruption, with the largest inequalities seen for access to medications. We did not observe differences by age or shielding status (Supplementary File 3).

<Figure 6 about here>

Sensitivity analysis

There were no major differences in the results after restricting to representative samples (Supplementary File 1).

For peer review only - http://bmjopen.bmj.com/site/about/guidelines.xhtml

Discussion

Our study demonstrates marked inequalities in healthcare disruption during the COVID-19 pandemic by harnessing data from 12 UK longitudinal studies. Females were more likely to report healthcare disruptions than males, especially at younger ages (<55 years). This inequality was observed for each healthcare disruption type including prescription medication, procedures or surgery, and appointments as well as a combined measure for any of these disruptions. Older adults were especially likely to report disruptions to medical appointments and procedures and surgeries compared to their younger counterparts. Ethnic minority (excluding White minorities) groups were more likely to report healthcare disruption compared to White (including White minorities) groups. Furthermore, when stratifying results by shielding status, the magnitude for the association between any healthcare disruption among Ethnic minority groups (compared to White groups) was higher among those who were shielding. In studies where a finer breakdown of ethnicity was possible, Black ethnic minority groups had the most clearly increased odds of disruption compared to White ethnic groups. Occupational class was also found to be associated with healthcare disruption with those in a routine/manual occupation or other (which included never/long-term non-employed) being more likely to experience healthcare disruption than those in a managerial/professional occupation. No clear association between education and healthcare disruption was found in the main, age or shielding status stratified analyses.

The direct burden of COVID-19 on health services across the globe has been colossal and remains so in some countries, with prioritisation of COVID-19 patients, leaving less capacity and resources for non-COVID-19 healthcare. Furthermore, associated repeated lockdown measures are also likely to decrease healthcare access and availability with a decrease in the number of people attending A&E services,[4] and reports of difficulties accessing medication.[9]

Our findings are consistent with current evidence from a smaller sub-set of the studies examined here suggesting that females are more likely to experience disruption to planned surgery, medical procedures, or other medical appointments during lockdown.[13] Furthermore, our results show that older adults were more likely to report healthcare disruption as compared to their younger counterparts,

For peer review only - http://bmjopen.bmj.com/site/about/guidelines.xhtml

especially disruptions to medical appointments and planned procedures or surgeries. This finding is consistent with current UK evidence indicating that older adults experience more delays and disruption to health services.[33–36] Black ethnic minority groups were also found to be at increased risk of healthcare disruption compared to white ethnic groups – an issue of particular concern given pre-pandemic ethnic inequalities in healthcare.[37] The inequalities by occupational class we found are consistent with prior evidence of socioeconomic healthcare inequalities reported in the UK in the past decade,[38] and highlight that these have still been present in the COVID-19 pandemic. Associations with occupational class were clearer than those for education, which is also an indicator of socioeconomic position but may have been a more distal influence.

The sex inequalities observed in this study could partially be explained by a disproportional increase in childcare responsibilities for women,[39] which may have made it more difficult to access healthcare. However, in this study we adjusted for household composition and associations for sex were robust to further adjustment on this variable.

Our result also show that older adults were more likely to report healthcare disruption than younger adults. There are many reasons why older people may have experienced an increase in healthcare disruption during the COVID-19 pandemic compared to younger people, including fear of becoming infected while visiting a care facility, difficulties engaging in telemedicine (using technology to deliver care),[33–35] and greater frailty, resulting in more healthcare utilisation and subsequent disruption.[36]

One explanation for the inequality in healthcare disruption among black ethnic minority group may be due to adverse effects of loss of income, unstable housing, increased psychological distress and reduced community support brought about by lockdown restrictions. Another explanation could stem from a disproportionate representation of ethnic minority populations among keyworkers, who are subjected to increased and antisocial working hours.

Further, those in routine/manual occupations were more likely to experience healthcare disruption than those in managerial/professional occupations. This finding is consistent with prior evidence of socioeconomic healthcare inequalities reported in the UK in the past decade.[38]

For peer review only - http://bmjopen.bmj.com/site/about/guidelines.xhtml

Strengths and limitations

The analysis brings together data from 12 longitudinal studies with rich and sensitive information on healthcare disruption. This study is strengthened by the coordinated investigation in multiple longitudinal studies with differing study designs, different target populations, and varying selection and attrition processes. Our combined approach provides the largest sample size available to prospectively investigate differences between ethnic groups, within representative population-based samples. What's more, though utilising non-response weights available, the proportion of ethnic minority groups within most studies is representative of the UK population. Moreover, the use of multiple studies increased statistical power to look at subpopulations such as ethnic minority groups across cohorts and allowed for greater examination of how inequalities were patterned by age. While not all 12 studies were representative of the population of interest, removing them in sensitivity analyses did not change our conclusions. Our novel approach to coordinated analyses harnessing multiple datasets therefore allowed research questions to be addressed which would not otherwise be possible.

Differences between studies in a range of factors including measurement of healthcare disruption, timing of surveys, design, response rates, and differential selection into the COVID-19 sweeps are potentially responsible for heterogeneity in estimates. However, despite this heterogeneity, the key findings were consistent across most datasets. Furthermore, this heterogeneity can be informative, for example, by virtue of mixing age-specific and age range studies, we identified that sex inequalities were stronger at younger ages, and inequalities by occupational class were concentrated within working ages. The definition of healthcare disruption used may also have contained a range of disruptions of greater or lesser severity, and there may have been further inequalities in the severity of disruptions experienced, however we were not able to assess this using the available data. We also could not assess pre-pandemic inequalities in healthcare disruption, though other studies have indicated massive increases in the prevalence of healthcare disruption (at least in part from the supply side with non-urgent procedures cancelled to reduce risk of infection transmission), and that inequalities related to geographic measures of deprivation (rather than individual-level measures as used here) have widened during the pandemic.[5,40,41]

For peer review only - http://bmjopen.bmj.com/site/about/guidelines.xhtml

We have focused on our aim of identifying who experienced greater disruptions in healthcare, rather than on adjustment for confounders to estimate causal effects of the exposures in question.[42] Nevertheless, many of the associations we observed were robust to adjustment for a wider range of related variables, but bias due to residual confounding cannot be ruled out. Importantly, we did not condition our analyses on healthcare need. Many of the inequalities we observed for healthcare disruptions may be due to inequalities in health, with those who have greater health needs being more likely to require healthcare that could be disrupted. Accounting for differences in need could have masked inequalities in healthcare disruptions that are caused by inequalities in health and could have made it less clear which groups have been more likely to experience disruption during the pandemic. Restricting analyses to those who needed care could also induce bias if there were unmeasured determinants of both need and disruption.[43] Nevertheless, another study of the USOC data analysed here that did restrict analyses to those needing care still found income-related inequalities in healthcare disruption, and most of the associations we observed were robust to adjustment for pre-pandemic self-assessed health.[44]

Impact of healthcare disruption

Disadvantaged groups such as females, older adults, Black ethnic minority groups, and those in routine/manual occupations have had elevated odds of healthcare disruption in the first 8-10 months of the COVID-19 pandemic.

Delays and disruptions to treatment could have ongoing implications for patients' physical and mental health.[45] Action is needed to remedy these inequalities, and efforts to ensure continuity of care during pandemic-related disruptions may need to be more clearly targeted to those who most need that care. Actions to alleviate healthcare disruption inequalities critically rely on better understanding the causes. For example, barriers to accessing care such as working hours or fear of infection, may require measures to make care more accessible outside of working hours, or to increase public confidence that patients can attend safely.

As healthcare access resumes, given the forgone delays in treatments and the subsequent backlog of postponed surgeries,[46] these groups may require prioritised support to address unmet needs experienced during the pandemic.

For peer review only - http://bmjopen.bmj.com/site/about/guidelines.xhtml

Conclusion

There have been clear inequalities in disruptions to healthcare during the COVID-19 pandemic in the UK. Females (especially those aged 54 or younger), older adults, ethnic minorities, and those in disadvantaged occupational classes have been more likely to experience healthcare disruptions. These are groups who usually experience worse health, so considering the massive increases in the prevalence of healthcare disruptions related to COVID-19, these inequalities in disruption have clear potential to maintain or even exacerbate existing health inequalities.

For peer review only

Contributors

Katikireddi, Ploubidis, Maddock, Parsons, Di Gessa, Green and Thompson conceptualised the study and design. Katikireddi, Ploubidis, Maddock Parsons, Di Gessa, Green, Thompson, Silverwood designed the methodology, Maddock, Parsons, Di Gessa, Green, Thompson, Stevenson, Kwong, McElroy and Santorelli conducted the formal analysis. Maddock, Parsons, Di Gessa, Green, Thompson drafted the manuscript. Captur, Steves and Steptoe and all authors contributed to critical revision and provided final approval of the manuscript. The project was supervised by Ploubidis and Katikireddi. Funding was acquired by Patalay, Katikireddi, Ploubidis, Silverwood, and Chaturvedi.

Competing interests

No conflicts of interest were declared by JM, SP, GDG, MJG, EJT, AJS, GS, RJS, GC, NC, AS, ASFK, CJS, PP, GBP. SVK is a member of the Scientific Advisory Group on Emergencies subgroup on ethnicity and COVID-19 and is co-chair of the Scottish Government's Ethnicity Reference Group on COVID-19. NC serves on a data safety monitoring board for trials sponsored by Astra-Zeneca.

Funding

This work was supported by the National Core Studies, an initiative funded by UKRI, NIHR and the Health and Safety Executive. The COVID-19 Longitudinal Health and Wellbeing National Core Study was funded by the Medical Research Council (MC_PC_20030).

Understanding Society is an initiative funded by the Economic and Social Research Council and various Government Departments, with scientific leadership by the Institute for Social and Economic Research, University of Essex, and survey delivery by NatCen Social Research and Kantar Public. The Understanding Society COVID-19 study is funded by the Economic and Social Research Council (ES/K005146/1) and the Health Foundation (2076161). The research data are distributed by the UK Data Service.

The Millennium Cohort Study, Next Steps, British Cohort Study 1970 and National Child Development Study 1958 are supported by the Centre for Longitudinal Studies, Resource Centre 2015-20 grant (ES/M001660/1) and a host of other co-funders. The 1946 NSHD cohort is hosted by the MRC Unit for Lifelong Health and Ageing funded by the Medical Research Council (MC_UU_00019/1 Theme 1: Cohorts and Data Collection). The COVID-19 data collections in these five cohorts were funded by the UKRI grant Understanding the economic, social and health impacts of COVID-19 using lifetime data: evidence from 5 nationally representative UK cohorts (ES/V012789/1).

The English Longitudinal Study of Ageing was developed by a team of researchers based at University College London, NatCen Social Research, the Institute for Fiscal Studies, the University of Manchester

For peer review only - http://bmjopen.bmj.com/site/about/guidelines.xhtml

and the University of East Anglia. The data were collected by NatCen Social Research. The funding is currently provided by the National Institute on Aging in the US, and a consortium of UK government departments coordinated by the National Institute for Health Research. Funding has also been received by the Economic and Social Research Council. The English Longitudinal Study of Ageing Covid-19 Sub-study was supported by the UK Economic and Social Research Grant (ESRC) ES/V003941/1.

The UK Medical Research Council and Wellcome (Grant Ref: 217065/Z/19/Z) and the University of Bristol provide core support for ALSPAC. A comprehensive list of grants funding is available on the ALSPAC website (http://www.bristol.ac.uk/alspac/external/documents/grant-acknowledgements.pdf). We are extremely grateful to all the families who took part in this study, the midwives for their help in recruiting them, and the whole ALSPAC team, which includes interviewers, computer and laboratory technicians, clerical workers, research scientists, volunteers, managers, receptionists and nurses. Please note that the study website contains details of all the data that is available through a fully searchable data dictionary and variable search tool" and reference the following webpage: http://www.bristol.ac.uk/alspac/researchers/our-data/. Ethical approval for the study was obtained from the ALSPAC Ethics and Law Committee and the Local Research Ethics Committees

TwinsUK receives funding from the Wellcome Trust (WT212904/Z/18/Z), the National Institute for Health Research (NIHR) Biomedical Research Centre based at Guy's and St Thomas' NHS Foundation Trust and King's College London. TwinsUK is also supported by the Chronic Disease Research Foundation and Zoe Global Ltd. The funders had no role in study design, data collection and analysis, decision to publish, or preparation of the manuscript.

Generation Scotland received core support from the Chief Scientist Office of the Scottish Government Health Directorates [CZD/16/6] and the Scottish Funding Council [HR03006]. Genotyping of the GS:SFHS samples was carried out by the Genetics Core Laboratory at the Wellcome Trust Clinical Research Facility, Edinburgh, Scotland and was funded by the Medical Research Council UK and the Wellcome Trust (Wellcome Trust Strategic Award "STratifying Resilience and Depression Longitudinally" (STRADL) Reference 104036/Z/14/Z). Generation Scotland is funded by the Wellcome Trust (216767/Z/19/Z).

Born in Bradford (BiB) receives core infrastructure funding from the Wellcome Trust (WT101597MA), and a joint grant from the UK Medical Research Council (MRC) and UK Economic and Social Science Research Council (ESRC) (MR/N024397/1) and one from the British Heart Foundation (BHF) (CS/16/4/32482). The National Institute for Health Research Yorkshire and Humber ARC, and Clinical Research Network both provide support for BiB research.

SVK acknowledges funding from a NRS Senior Clinical Fellowship (SCAF/15/02), the Medical Research Council (MC_UU_00022/2) and the Scottish Government Chief Scientist Office (SPHSU17). ASFK acknowledges funding from the ESRC (ES/V011650/1). DJP acknowledges

For peer review only - http://bmjopen.bmj.com/site/about/guidelines.xhtml

funding from the Wellcome Trust (216767/Z/19/Z and 221574/Z/20/Z). EJT acknowledges funding from the Wellcome Trust (WT212904/Z/18/Z). GBP acknowledges funding from the Economic and Social Research Council (ES/V012789/1).

Acknowledgements

The contributing studies have been made possible because of the tireless dedication, commitment and enthusiasm of the many people who have taken part. We would like to thank the participants and the numerous team members involved in the studies including interviewers, technicians, researchers, administrators, managers, health professionals and volunteers including:

Generation Scotland: Drew Altschul, Chloe Fawns-Ritchie, Archie Campbell, Robin Flaig.
ALSPAC: Daniel J Smith, Nicholas J Timpson, Kate Northstone.
Understanding Society: Michaela Benzeval
TwinsUK: Deborah Hart, María Paz García, Rachel Horsfall, Ruth C.E. Bowyer.
Centre for Longitudinal Studies: Matt Brown, Lisa Calderwood, Emla Fitzsimons, Alissa Goodman, Aida Sanchez
NSHD: Andrew Wong, Maria Popham, Karen MacKinnon, Imran Shah, Philip Curran

We are additionally grateful to our funders for their financial input and support in making this research happen.

Ethics approval

The most recent sweeps of the **NSHD, NCDS, BCS70**, **Next Steps** and **MCS** have all been granted ethical approval by the National Health Service (NHS) Research Ethics Committee and all participants have given informed consent. Data for NCDS (SN 6137), BCS70 (SN 8547), Next Steps (SN 5545), MCS (SN 8682) and all four COVID-19 surveys (SN 8658) are available through the UK Data Service. Ethical approval was obtained from the **ALSPAC** Ethics and Law Committee and the Local Research Ethics Committees. Ethical approval for **Born in Bradford** was granted by the National Health Service Health Research Authority Yorkshire and the Humber (Bradford Leeds) Research Ethics Committee (reference: 16/YH/0320). The University of Essex Ethics Committee has approved all data collection for the **Understanding Society** main study and COVID-19 waves. No additional ethical approval was necessary for this secondary data analysis. Waves 1-9 of **ELSA** were approved through the National Research Ethics Service, while the COVID-19 Sub-study was approved by the UCL Research Ethics Committee. All participants provided informed consent. **Generation Scotland** obtained ethical approval from the East of Scotland Committee on Medical Research Ethics (on behalf of the National Health Service). Reference number 20/ES/0021. All waves of **TwinsUK** have received ethical approval associated with TwinsUK Biobank (19/NW/0187),

For peer review only - http://bmjopen.bmj.com/site/about/guidelines.xhtml

TwinsUK (EC04/015) or Healthy Ageing Twin Study (H.A.T.S) (07/H0802/84) studies from NHS Research Ethics Committees at the Department of Twin Research and Genetic Epidemiology, King's College London.

Data availability statement

Data for NCDS (SN 6137), BCS70 (SN 8547), Next Steps (SN 5545), MCS (SN 8682) and all four COVID-19 surveys (SN 8658) are available through the UK Data Service. NSHD data are available on request to the NSHD Data Sharing Committee. Interested researchers can apply to access the NSHD data via a standard application procedure. Data requests should be submitted to mrclha.swiftinfo@ucl.ac.uk; further details can be found at http://www.nshd.mrc.ac.uk/data.aspx. doi:10.5522/NSHD/Q101; doi:10.5522/NSHD/Q10. The ALSPAC study website contains details of all the data that is available through a fully searchable data dictionary and variable search tool: http://www.bristol.ac.uk/alspac/researchers/our-data. ALSPAC data is available to researchers through an online proposal system. Information regarding access can be found on the ALSPAC website (http://www.bristol.ac.uk/media-library/sites/alspac/documents/researchers/data-access/ALSPAC_Access_Policy.pdf). Data from the various BiB family studies are available to researchers; see the study website for information on how to access data (https://borninbradford.nhs.uk/research/how-to-access-data/). All **USOC** data are available through the UK Data Service (SN 6614 and SN 8644). All **ELSA** data are available through the UK Data Service (SN 8688 and 5050). Access to data from **GS** is approved by the Generation Scotland Access Committee. See https://www.ed.ac.uk/generation-scotland/for-researchers/access or email access@generationscotland.org for further details. The TwinsUK Resource Executive Committee (TREC) oversees management, data sharing and collaborations involving the TwinsUK registry (for further details see https://twinsuk.ac.uk/resources-for-researchers/access-our-data/).

For peer review only - http://bmjopen.bmj.com/site/about/guidelines.xhtml

For peer review only

For peer review only - http://bmjopen.bmj.com/site/about/guidelines.xhtml

References

1 Boserup B, Mckenney M, Elkbuli A. The impact of the COVID-19 pandemic on emergency department visits and patient safety in the United States. *Am J Emerg Med* 2020;**38**:1732–6.

2 Vuma CD, Manganyi J, Wilson K, *et al.* The Effect on Fit of Multiple Consecutive Donning and Doffing of N95 Filtering Facepiece Respirators. *Ann Work Expo Heal* 2019;**63**:930–6. doi:10.1093/annweh/wxz060

3 Salerno R, Conti CB, De Silvestri A, *et al.* The impact of covid-19 pandemic on urgent endoscopy in Italy: a nation-wide multicenter study. *Scand J Gastroenterol* 2020;**55**:870–6. doi:10.1080/00365521.2020.1782466

4 Kelly E, Firth Z. How is COVID-19 changing the use of emergency care by region? Heal. Found. 2020.https://www.health.org.uk/news-and-comment/charts-and-infographics/how-is-covid-19-changing-the-use-of-emergency-care-by-region

5 Warner M, Burn S, Stoye G, *et al.* Socioeconomic deprivation and ethnicity inequalities in disruption to NHS hospital admissions during the COVID-19 pandemic: a national observational study. *BMJ Qual Saf* 2021;:bmjqs-2021-013942. doi:10.1136/bmjqs-2021-013942

6 Lazzerini M, Barbi E, Apicella A, *et al.* Delayed access or provision of care in Italy resulting from fear of COVID-19. *Lancet Child Adolesc Heal* 2020;**4**:e10–1. doi:10.1016/S2352-4642(20)30108-5

7 Association BM. Pressure points in the NHS. Br. Med. Assoc. 2021.https://www.bma.org.uk/advice-and-support/nhs-delivery-and-workforce/pressures/pressure-points-in-the-nhs

8 Cheong JLY, Goh ZHK, Marras C, *et al.* The Impact of COVID-19 on Access to Parkinson's Disease Medication. *Mov Disord* 2020;**35**:2129–33. doi:10.1002/mds.28293

9 Maldonado D, Tu E, Mahmood S, *et al.* Medication access difficulty and COVID-related distress are associated with disease flares in rheumatology patients during the COVID-19 pandemic. *Arthritis Care Res (Hoboken)* 2020;:0–3. doi:10.1002/acr.24531

10 Bleich SN, Jarlenski MP, Bell CN, *et al.* Health inequalities: Trends, progress, and policy. *Annu Rev Public Health* 2012;**33**:7–40. doi:10.1146/annurev-publhealth-031811-124658

11 Immergut EM, Schneider SM. Is it unfair for the affluent to be able to purchase "better" healthcare? Existential standards and institutional norms in healthcare attitudes across 28 countries. *Soc Sci Med* 2020;**267**:113146. doi:10.1016/j.socscimed.2020.113146

12 Tudor Hart J. the Inverse Care Law. *Lancet* 1971;**297**:405–12. doi:10.1016/S0140-6736(71)92410-X

13 Topriceanu CC, Wong A, Moon JC, *et al.* Evaluating access to health and care services during lockdown by the COVID-19 survey in five UK national longitudinal studies. *BMJ Open* 2021;**11**. doi:10.1136/bmjopen-2020-045813

14 Institute for Fiscal Studies. COVID-19 and disruptions to the health and social care of older people in England. 2020;:1–21.

15 Joshi HE, Fitzsimons E. The UK Millennium Cohort Study: the making of a multi- purpose resource for social science and policy in the UK. *Longit Life Course Stud* 2016;**7**:409–30. doi:10.14301/llcs.v7i4.416

16 Calderwood L, Sanchez C. Next Steps (formerly known as the Longitudinal Study of Young

For peer review only - http://bmjopen.bmj.com/site/about/guidelines.xhtml

People in England). 2016;:2–4.

17 Sullivan A, Brown M, Hamer M, *et al.* Cohort Profile Update: The 1970 British Cohort Study (BCS70). 2022;:1–8.

18 Power C, Elliott J. Cohort profile: 1958 British birth cohort (National Child Development Study). *Int J Epidemiol* 2006;**35**:34–41. doi:10.1093/ije/dyi183

19 Wadsworth M, Kuh D, Richards M, *et al.* Cohort profile: The 1946 National Birth Cohort (MRC National Survey of Health and Development). *Int J Epidemiol* 2006;**35**:49–54. doi:10.1093/ije/dyi201

20 Brown M, Goodman A, Peters A, *et al.* COVID-19 Survey in Five National Longitudinal Studies: Wave 1 User Guide (Version 1). *UCL Cent Longitud Stud MRC Unit Lifelong Heal Ageing London, UK* 2020;:1–62.https://cls.ucl.ac.uk/wp-content/uploads/2021/01/UCL-Cohorts-COVID-19-Survey-user-guide.pdf

21 University of Essex, Institute for Social and Economic Research, NatCen Social Research KP. Understanding Society: Waves 1-9, 2009-2019 and Harmonised BHPS: Waves 1-18, 1991-2009. [data Collect. 2020.

22 Steptoe A, Breeze E, Banks J, *et al.* Cohort profile: The English Longitudinal Study of Ageing. *Int J Epidemiol* 2013;**42**:1640–8. doi:10.1093/ije/dys168

23 Boyd A, Golding J, Macleod J, *et al.* Cohort profile: The 'Children of the 90s'-The index offspring of the avon longitudinal study of parents and children. *Int J Epidemiol* 2013;**42**:111–27. doi:10.1093/ije/dys064

24 Fraser A, Macdonald-wallis C, Tilling K, *et al.* Cohort Profile : The Avon Longitudinal Study of Parents and Children : ALSPAC mothers cohort. 2013;:97–110. doi:10.1093/ije/dys066

25 Wright J, Small N, Raynor P, *et al.* Cohort profile: The born in bradford multi-ethnic family cohort study. *Int J Epidemiol* 2013;**42**:978–91. doi:10.1093/ije/dys112

26 Dickerson J, Bird PK, McEachan RRC, *et al.* Born in Bradford's Better Start: An experimental birth cohort study to evaluate the impact of early life interventions. *BMC Public Health* 2016;**16**:1–14. doi:10.1186/s12889-016-3318-0

27 Smith BH, Campbell A, Linksted P, *et al.* Cohort profile: Generation scotland: Scottish family health study (GS: SFHS). The study, its participants and their potential for genetic research on health and illness. *Int J Epidemiol* 2013;**42**:689–700. doi:10.1093/ije/dys084

28 Suthahar A, Sharma P, Hart D, *et al.* TwinsUK COVID-19 personal experience questionnaire (CoPE): wave 1 data capture April-May 2020 [version 1 ; peer review : awaiting peer review]. 2021;:1–10.

29 Verdi S, Abbasian G, Bowyer RCE, *et al.* TwinsUK: The UK Adult Twin Registry Update. *Twin Res Hum Genet* 2019;:1–7. doi:10.1017/thg.2019.65

30 Addario G, Dangerfield P, Hussey D, *et al.* Adapting fieldwork during the COVID-19 outbreak A methodological overview of the ELSA COVID-19 Substudy (wave 1). London: 2020.

31 Institute for Social and Economic Research. Understanding Society COVID-19 User Guide. Colchester: 2021. doi:10.1007/978-1-349-15392-3

32 Press S. Stata Statistical Software: Release 16. StataCorp LLC. 2019.

33 Mann DM, Chen J, Chunara R, *et al.* COVID-19 transforms health care through telemedicine: Evidence from the field. *J Am Med Informatics Assoc* 2020;**27**:1132–5. doi:10.1093/jamia/ocaa072

For peer review only - http://bmjopen.bmj.com/site/about/guidelines.xhtml

34 Beaunoyer E, Dupéré S, Guitton MJ. COVID-19 and digital inequalities: Reciprocal impacts and mitigation strategies. *Comput Human Behav* 2020;**111**. doi:10.1016/j.chb.2020.106424

35 Crawford A, Serhal E. Digital health equity and COVID-19: The innovation curve cannot reinforce the social gradient of health. *J Med Internet Res* 2020;**22**:1–5. doi:10.2196/19361

36 Kojima G, Liljas AEM, Iliffe S. Frailty syndrome: Implications and challenges for health care policy. *Risk Manag Healthc Policy* 2019;**12**:23–30. doi:10.2147/RMHP.S168750

37 Katikireddi SV, Cezard G, Bhopal RS, *et al.* Assessment of health care, hospital admissions, and mortality by ethnicity: population-based cohort study of health-system performance in Scotland. *Lancet Public Heal* 2018;**3**:e226–36. doi:10.1016/S2468-2667(18)30068-9

38 Cookson R, Propper C, Asaria M, *et al.* Socio-Economic Inequalities in Health Care in England. *Fisc Stud* 2016;**37**:371–403. doi:10.1111/j.1475-5890.2016.12109

39 Xue B, McMunn A. Gender differences in unpaid care work and psychological distress in the UK Covid-19 lockdown. *PLoS One* 2021;**16**:e0247959.https://doi.org/10.1371/journal.pone.0247959

40 Stokes J. Inequalities exacerbated: an all-too-familiar story. *BMJ Qual & Saf* 2022;**31**:561 LP – 564. doi:10.1136/bmjqs-2021-014422

41 Nguyen LH, Drew DA, Graham MS, *et al.* Risk of COVID-19 among front-line health-care workers and the general community: a prospective cohort study. *Lancet Public Heal* 2020;**5**:e475–83. doi:10.1016/S2468-2667(20)30164-X

42 Conroy S, Murray EJ. Let the question determine the methods: descriptive epidemiology done right. *Br J Cancer* 2020;**123**:1351–2. doi:10.1038/s41416-020-1019-z

43 Cole SR, Platt RW, Schisterman EF, *et al.* Illustrating bias due to conditioning on a collider. *Int J Epidemiol* 2010;**39**:417–20. doi:10.1093/ije/dyp334

44 Davillas A, Jones AM. Unmet health care need and income-Related horizontal equity in use ofhealth care during the COVID-19 pandemic. *Health Econ* 2021;:1–6. doi:10.1002/hec.4282

45 Barach P, Fisher SD. Disruption of healthcare: Will the COVID pandemic worsen non-COVID outcomes and disease outbreaks? *Prog Pediatr Cardiol* 2020.

46 Nepogodiev D, Omar OM, Glasbey JC, *et al.* Elective surgery cancellations due to the COVID-19 pandemic: global predictive modelling to inform surgical recovery plans. *Br J Surg* 2020;**107**:1440–9. doi:10.1002/bjs.11746

For peer review only

Figure titles and legends:

Figure 1. Prevalence (and 95% CIs) of any healthcare disruption by study
Sources: MCS (Millennium Cohort Study); ALSPAC G1 (Children of the Avon Longitudinal Study of Parents and Children); NS (Next Steps); BCS 70 (1970 British Cohort Study), NCDS (National Child Development Study); NSHD (National Survey of Health and Development); BIB (Born in Bradford); USOC (Understanding Society); GS (Generation Scotland: the Scottish Family Health Study); ALSPAC G0 (parents of ALSPAC); TWINS UK (UK Adult Twin Registry); ELSA (English Longitudinal Study of Ageing). Notes: Studies are ordered by age homogeneity/heterogeneity and mean age of respondents at the time of the interview. Samples for each study restricted to respondents with non-missing information on healthcare disruptions and valid information on sex, social class, education and (where applicable) age and ethnicity. All information about how data were collected and variables were coded is available in Supplementary File 2.

Figure 2. Associations between female (compared to male) sex and healthcare disruption

Notes: Adjusted for age and ethnicity where applicable.

Figure 3. Associations between age (compared to 45-54-year-olds) and healthcare disruption

Notes: Adjusted for sex and ethnicity where applicable.

Figure 4. Associations between Ethnicity (compared to White groups) and healthcare disruption
Notes: Panels illustrate findings for some larger ethnic groups separately and the final panel presents results for all non-White ethnic minorities combined. Adjusted for age and sex where applicable.

Figure 5. Associations between education (compared to degree level) and healthcare disruption

Notes: Adjusted for age, sex and ethnicity where applicable.

Figure 6. Associations between occupational social class (compared to Professional/Managerial) and healthcare disruption

Notes: Adjusted for age, sex and ethnicity where applicable.

For peer review only

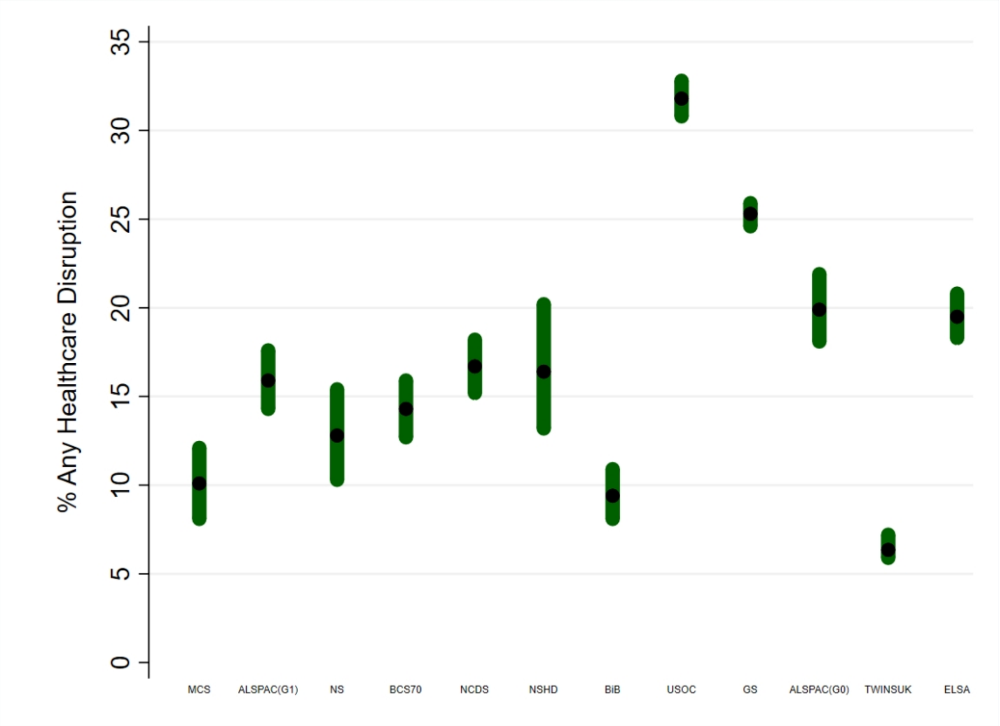

Figure 1. Prevalence (and 95% CIs) of any healthcare disruption by study
Sources: MCS (Millennium Cohort Study); ALSPAC G1 (Children of the Avon Longitudinal Study of Parents and Children); NS (Next Steps); BCS 70 (1970 British Cohort Study), NCDS (National Child Development Study); NSHD (National Survey of Health and Development); BIB (Born in Bradford); USOC (Understanding Society); GS (Generation Scotland: the Scottish Family Health Study); ALSPAC G0 (parents of ALSPAC); TWINS UK (UK Adult Twin Registry); ELSA (English Longitudinal Study of Ageing). Notes: Studies are ordered by age homogeneity/heterogeneity and mean age of respondents at the time of the interview. Samples for each study restricted to respondents with non-missing information on healthcare disruptions and valid information on sex, social class, education and (where applicable) age and ethnicity. All information about how data were collected and variables were coded is available in Supplementary File 1.

416x303mm (72 x 72 DPI)

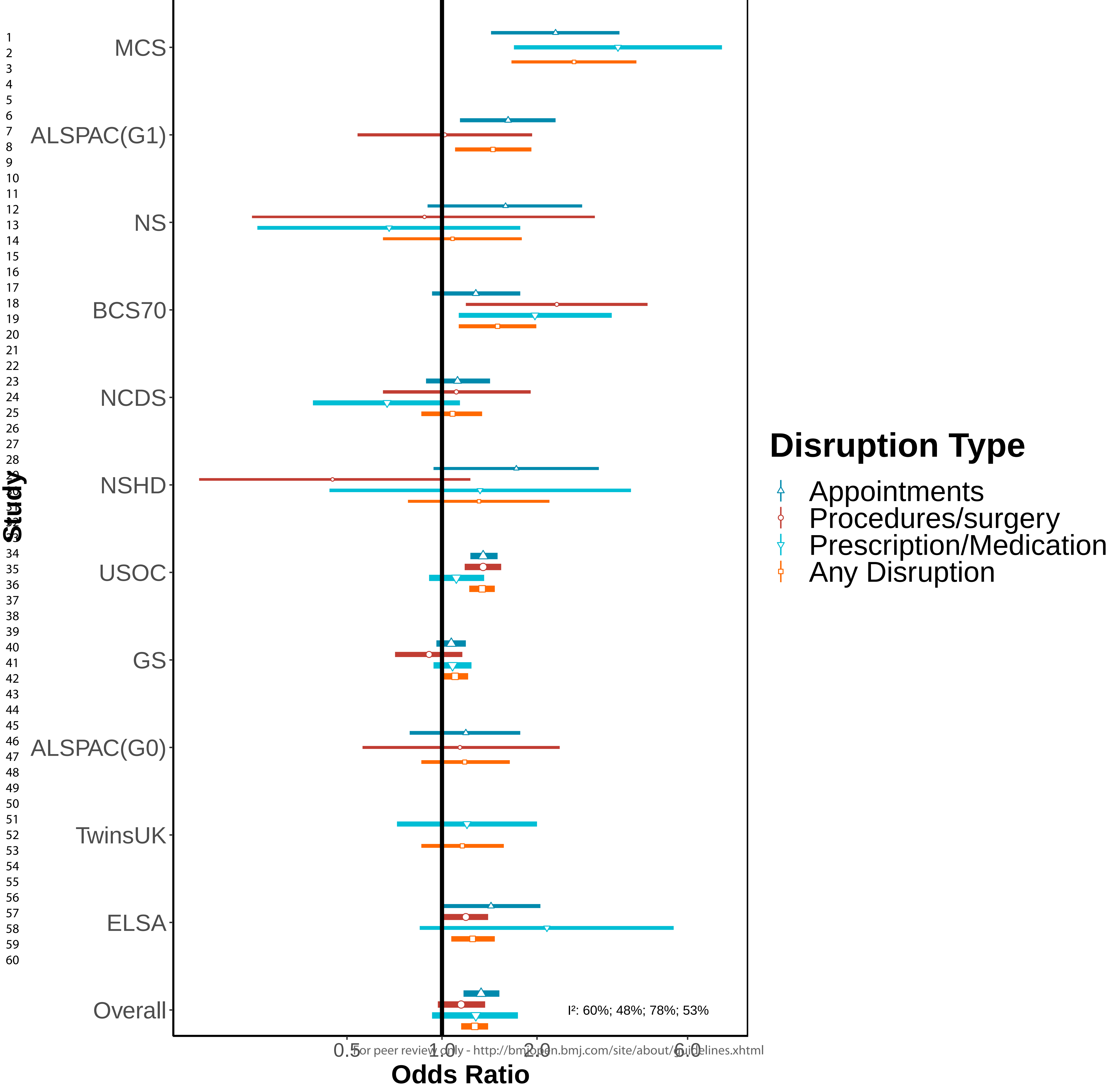
MCS
ALSPAC(G1)
NS
BCS70
NCDS
NSHD
USOC
GS
ALSPAC(G0)
TwinsUK
ELSA
Overall
Study
I²: 60%; 48%; 78%; 53%
0.5
1.0
2.0
6.0
Odds Ratio
Disruption Type
Appointments
Procedures/surgery
Prescription/Medication
Any Disruption

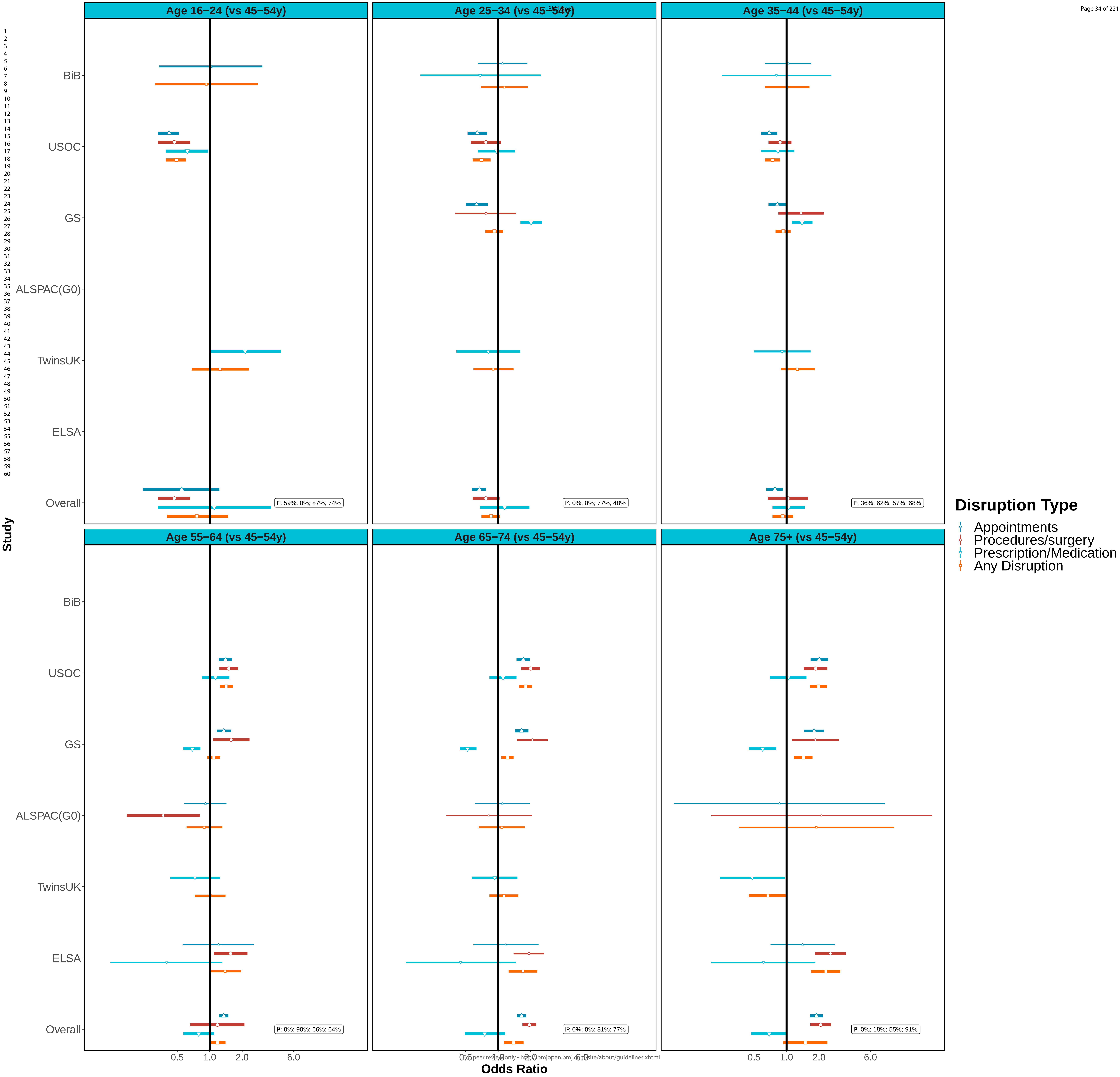
Age 16–24 (vs 45–54y)
Age 25–34 (vs 45–54y)
Age 35–44 (vs 45–54y)
Age 55–64 (vs 45–54y)
Age 65–74 (vs 45–54y)
Age 75+ (vs 45–54y)
BiB
USOC
GS
ALSPAC(G0)
TwinsUK
ELSA
Overall
I²: 59%; 0%; 87%; 74%
I²: 0%; 0%; 77%; 48%
I²: 36%; 62%; 57%; 68%
I²: 0%; 90%; 66%; 64%
I²: 0%; 0%; 81%; 77%
I²: 0%; 18%; 55%; 91%
0.5
1.0
2.0
6.0
Study
Odds Ratio
Disruption Type
Appointments
Procedures/surgery
Prescription/Medication
Any Disruption

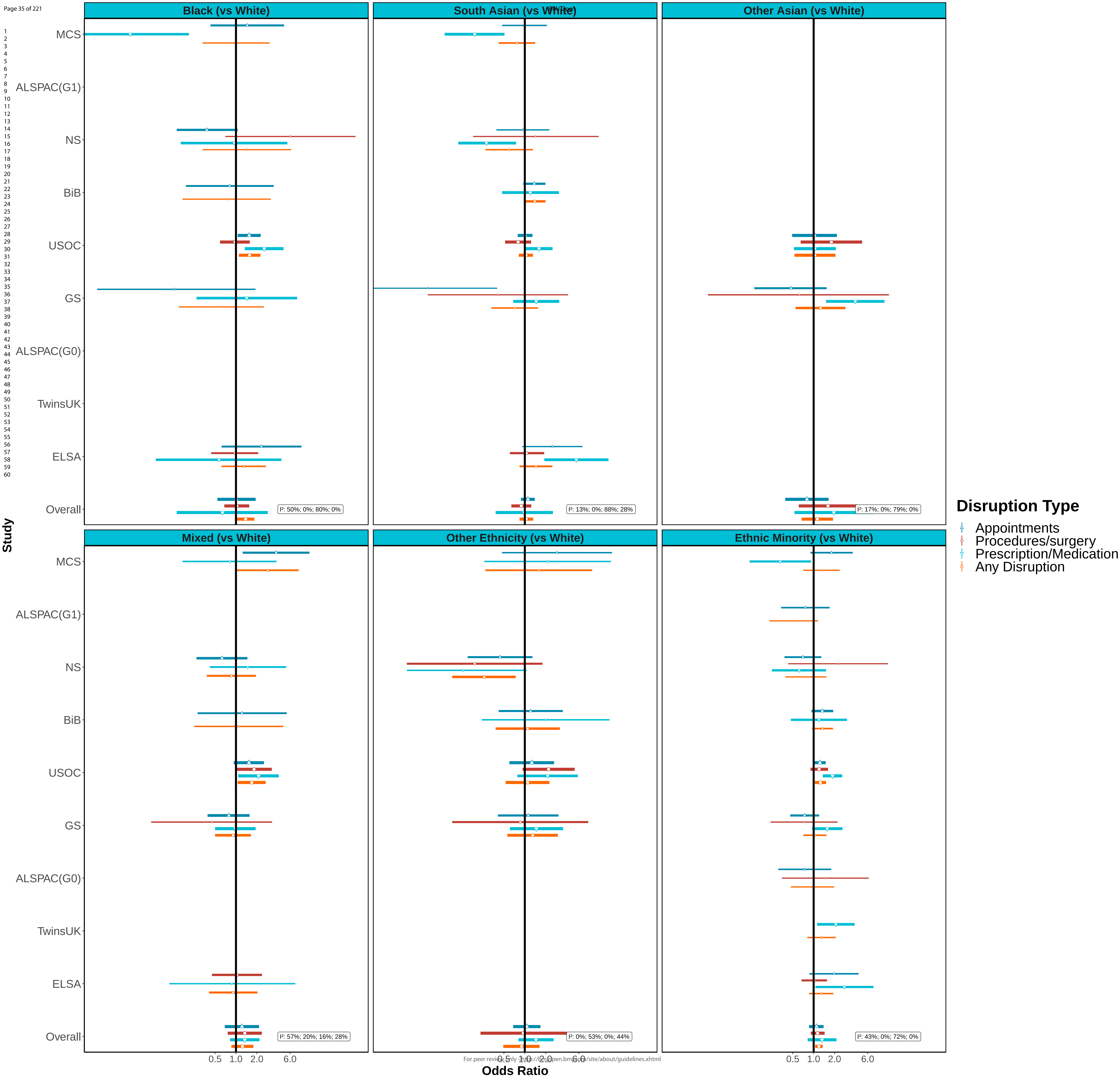
Black (vs White)
South Asian (vs White)
Other Asian (vs White)
Mixed (vs White)
Other Ethnicity (vs White)
Ethnic Minority (vs White)
MCS
ALSPAC(G1)
NS
BiB
USOC
GS
ALSPAC(G0)
TwinsUK
ELSA
Overall
I²: 50%; 0%; 80%; 0%
I²: 13%; 0%; 88%; 28%
I²: 17%; 0%; 79%; 0%
I²: 57%; 20%; 16%; 28%
I²: 0%; 53%; 0%; 44%
I²: 43%; 0%; 72%; 0%
0.5
1.0
2.0
6.0
Odds Ratio
Study
Disruption Type
Appointments
Procedures/surgery
Prescription/Medication
Any Disruption

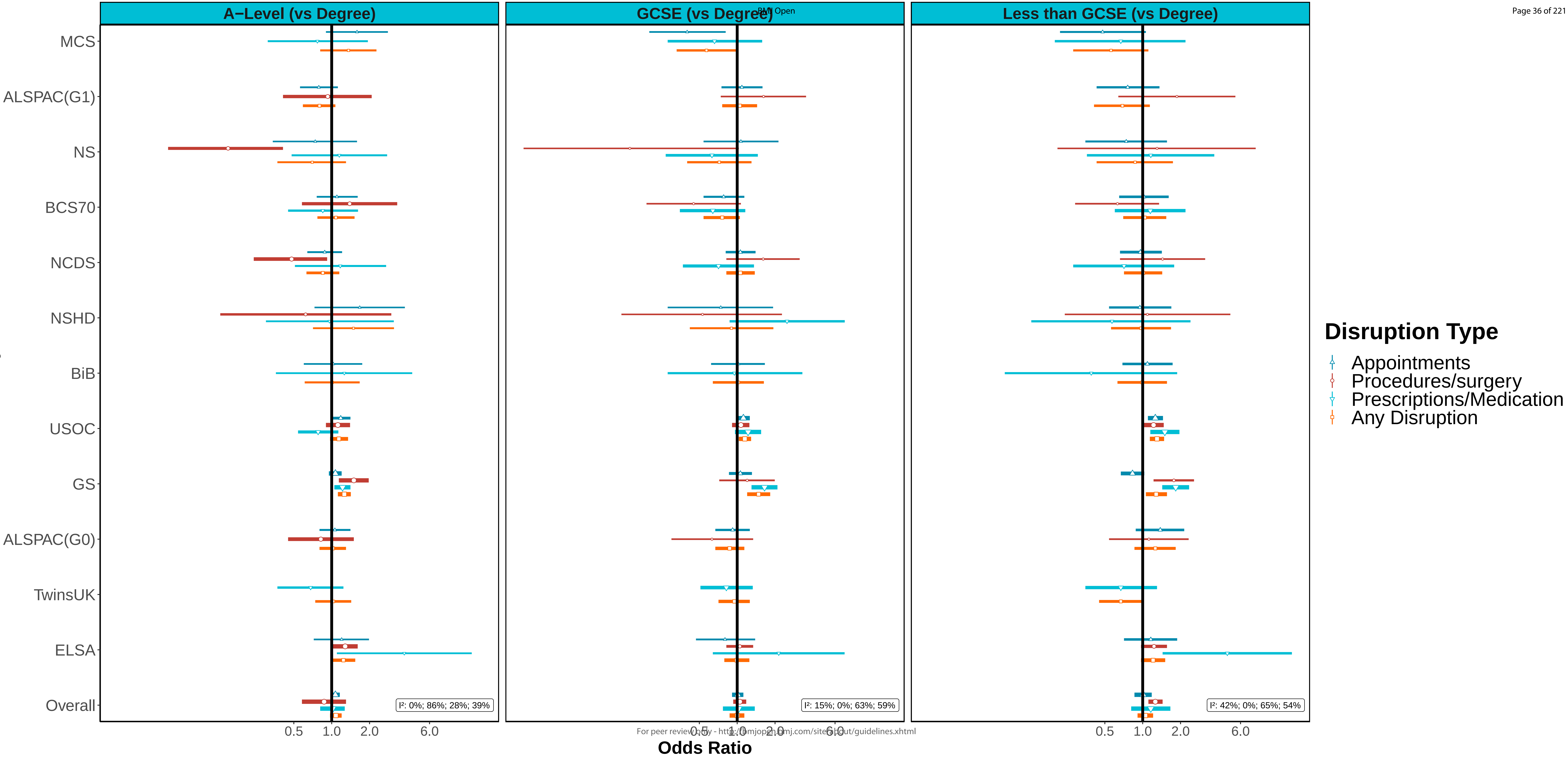
A−Level (vs Degree)
GCSE (vs Degree)
Less than GCSE (vs Degree)
Study
MCS
ALSPAC(G1)
NS
BCS70
NCDS
NSHD
BiB
USOC
GS
ALSPAC(G0)
TwinsUK
ELSA
Overall
I²: 0%; 86%; 28%; 39%
I²: 15%; 0%; 63%; 59%
I²: 42%; 0%; 65%; 54%
0.5
1.0
2.0
6.0
Odds Ratio
Disruption Type
Appointments
Procedures/surgery
Prescriptions/Medication
Any Disruption

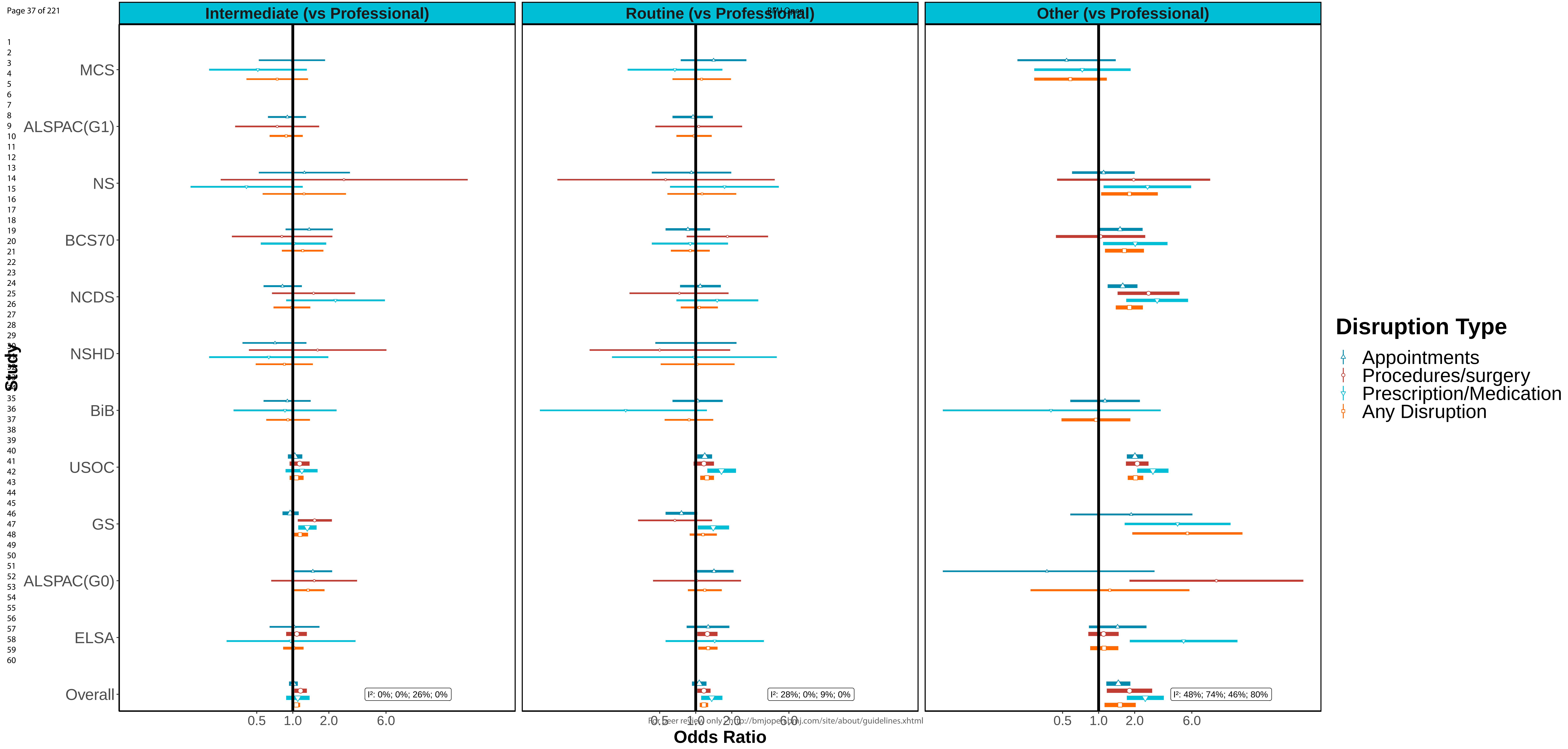

Intermediate (vs Professional)
Routine (vs Professional)
Other (vs Professional)
Study
MCS
ALSPAC(G1)
NS
BCS70
NCDS
NSHD
BiB
USOC
GS
ALSPAC(G0)
ELSA
Overall
I²: 0%; 0%; 26%; 0%
I²: 28%; 0%; 9%; 0%
I²: 48%; 74%; 46%; 80%
0.5
1.0
2.0
6.0
Odds Ratio
Disruption Type
Appointments
Procedures/surgery
Prescription/Medication
Any Disruption

Inequalities in healthcare disruptions during Covid-19 in the UK: Evidence from 12 population-based longitudinal studies

List of Supplementary Tables

Supplementary Table S1. Details of each study

Supplementary Table S2. Ethics and data access statements for each study

Supplementary Table S3. Percentage of USOC respondents who had reported specific disruptions at any point April – November 2020

Supplementary Table S4. Percent prevalence of any healthcare disruptions by selected characteristics and study

For peer review only

Supplementary Table S1. Details of each study

Study Population	Design and Sample Frame	2020 Age Range	Pre-pandemic Survey	Details of Covid surveys (response rate)	Analytic N
Age Homogenous Cohorts					
MCS: Millennium Cohort Study	Cohort of UK children born between Sept 2000 and Jan 2002 with regular follow-up surveys from birth.	18-20	2018	Two surveys: May (26.6%) & Sep-Oct (24.2%)	3147
ALSPAC (G1): Avon Longitudinal Study of Parents and Children- Generation 1	Cohort of children born in the South-West of England between April 1991 and Dec 1992, with regular follow-up surveys from birth. (original young people)	27-29	2017-2018	Three questionnaires: April (19%), June (17.4%), December (26.4%)	3430
NS: Next Steps, formerly known as Longitudinal Study of Young People in England	Sample recruited via secondary schools in England at around age 13 with regular follow-up surveys thereafter.	29-31	2015	Two surveys: May (20.3%) & Sep-Oct (31.8%)	3311
BCS70: British Cohort Study 1970	Cohort of all children born in Great Britain (i.e. England, Wales & Scotland) in one week in 1970, with regular follow-up surveys from birth.	50	2016	Two surveys: May (40.4%) & Sep-Oct (43.9%)	5175
NCDS: National Child Development Study	Cohort of all children born in Great Britain (i.e. England, Wales & Scotland) in one week in 1958, with regular follow-up surveys from birth.	62	2013	Two surveys: May (57.9%) & Sep-Oct (53.9%)	5747
NSHD: National Survey of Health and Development	Cohort of all children born in Great Britain (i.e. England, Wales & Scotland) in one week in 1946, with regular follow-up surveys from birth.	74	2015	Two surveys: May (68.2%) & Sep-Oct (61.5%)	1569
Age Heterogeneous Studies					
BIB: Born in Bradford	Birth cohort recruiting pregnant women and their children between 2007 and 2010; and pregnant women and their children in three deprived areas of Bradford between 2016 and 2020	17-54	2016-2020	Two surveys: April-Jun (28%) & Oct-Nov (24%)	1726
USOC: Understanding Society: the UK Household Longitudinal Survey	A nationally representative longitudinal household panel study, based on a clustered-stratified probability sample of UK households, with all adults aged 16+ in chosen households surveyed annually.	16-96	2018-2019	Six: surveys: April (40.3%); May (33.6%); Jun (32.0%); July (31.2%); Sep (29.2%) & Nov (27.3%)	13253
ELSA: English Longitudinal Study of Aging	A nationally-representative population study of individuals aged 50+ living in England, with biennial surveys since 2002/03.	52-90+	2018-2019	First Covid-19 sub-study: Jun-July (75%)	6508
GS: Generation Scotland: the Scottish Family Health Study	A family-structured, population-based Scottish cohort, with participants aged 18-99 recruited between 2006-2011	27-100	2006-2011	Two surveys: April-Jun (21.6%) & Jul-Aug (15.6%)	17139
ALSPAC(G0): Avon Longitudinal Study of Parents and Children- Generation 0	Parents of the ALSPAC(G1) cohort described above, treated as a separate age-heterogenous study population. (original parents)	45-81	2011-2013	Three questionnaires: April (12.4%), June (12.2%), December (14.3%)	3625
TWINSUK: the UK Adult Twin Registry	A cohort of UK volunteer adult twins (55% monozygotic and 43% dizygotic) who were sampled between 18-101 years of age.	22-96	2017-2018	Three surveys: April (64.3%), July (77.6%) & November (76.1%)	4282

For peer review only

Supplementary Table S2. Ethics and data access statements for each study

**NSHD, NCDS, BCS70**, **NS** and **MCS**	The most recent sweeps of the **NSHD, NCDS, BCS70**, **Next Steps** and **MCS** have all been granted ethical approval by the National Health Service (NHS) Research Ethics Committee and all participants have given informed consent. Data for NCDS (SN 6137), BCS70 (SN 8547), Next Steps (SN 5545), MCS (SN 8682) and all four COVID-19 surveys (SN 8658) are available through the UK Data Service. NSHD data are available on request to the NSHD Data Sharing Committee. Interested researchers can apply to access the NSHD data via a standard application procedure. Data requests should be submitted to mrclha.swiftinfo@ucl.ac.uk; further details can be found at http://www.nshd.mrc.ac.uk/data.aspx. doi:10.5522/NSHD/Q101; doi:10.5522/NSHD/Q10.
**ALSPAC**	Ethical approval was obtained from the **ALSPAC** Ethics and Law Committee and the Local Research Ethics Committees. The study website contains details of all the data that is available through a fully searchable data dictionary and variable search tool: http://www.bristol.ac.uk/alspac/researchers/our-data. ALSPAC data is available to researchers through an online proposal system. Information regarding access can be found on the ALSPAC website (http://www.bristol.ac.uk/media-library/sites/alspac/documents/researchers/data-access/ALSPAC_Access_Policy.pdf).
**BIB**	Ethical approval for **Born in Bradford** was granted by the National Health Service Health Research Authority Yorkshire and the Humber (Bradford Leeds) Research Ethics Committee (reference: 16/YH/0320). Data from the various BiB family studies are available to researchers; see the study website for information on how to access data (https://borninbradford.nhs.uk/research/how-to-access-data/).
**USOC**	The University of Essex Ethics Committee has approved all data collection for the **Understanding Society** main study and COVID-19 waves. No additional ethical approval was necessary for this secondary data analysis. All data are available through the UK Data Service (SN 6614 and SN 8644).
**ELSA**	Waves 1-9 of **ELSA** were approved through the National Research Ethics Service, while the COVID-19 Sub-study was approved by the UCL Research Ethics Committee. All participants provided informed consent. All data are available through the UK Data Service (SN 8688 and 5050).
**GS**	**Generation Scotland** obtained ethical approval from the East of Scotland Committee on Medical Research Ethics (on behalf of the National Health Service). Reference number 20/ES/0021. Access to data is approved by the Generation Scotland Access Committee. See https://www.ed.ac.uk/generation-scotland/for-researchers/access or email access@generationscotland.org for further details.
**TWINSUK**	All wave of **TwinsUK** have received ethical approval associated with TwinsUK Biobank (19/NW/0187), TwinsUK (EC04/015) or Healthy Ageing Twin Study (H.A.T.S) (07/H0802/84) studies from NHS Research Ethics Committees at the Department of Twin Research and Genetic Epidemiology, King's College London. The TwinsUK Resource Executive Committee (TREC) oversees management, data sharing and collaborations involving the TwinsUK registry (for further details see https://twinsuk.ac.uk/resources-for-researchers/access-our-data/).

For peer review only

Supplementary Table S3. Percentage of USOC respondents who had reported specific disruptions at any point April – November 2020

Percentage of USOC respondents who had reported specific disruptions at any point up to and including the survey in…

	April	May	June	July	September	November
**Prescription/ medication access**	2.4	3.3	3.9	4.4	4.7	5.5
**Procedures or surgery**	7.1	9.1	10.1	11.0	11.6	12.3
**Appointments**	18.5	22.2	24.0	25.1	26.3	28.4

For peer review only

Supplementary Table S4. Percent prevalence of any healthcare disruptions by selected characteristics and study

		MCS	ALSPAC (G1)	NS	BCS70	NCDS	NSHD	BIB	USOC	GS	ALSPAC (G0)	TWINS UK	ELSA
**Sex**	*Male*	6.1	12.8	12.1	11.7	15.6	14.5	NA	29.4	24.9	18.1	7.4	17.5
	Female	14.1	17.5	13.8	16.9	17.4	18.2	9.4	34.0	25.5	20.5	8.5	21.3
**Age**	*16-24*	10.1						8.2	18.3	NA	NA	10	NA
	25-34		15.9	12.8				10.4	24.0	22.9	NA	7.7	NA
	35-44							9.1	24.9	23.0	NA	13.2	NA
	45-54				14.3			8.7	30.9	24.2	21.3	13.9	13.0
	55-64					16.7		--	38.6	25.2	19.2	21.6	17.2
	65-74						16.4	--	43.6	26.8	21.8	31.4	20.0
	75+							--	45.6	29.2	30.6	9.2	25.5
**Ethnicity**	*White*	10.0	16.1	13.3	--	--	--	7.8	31.9	25.4	19.9	8.3	19.5
	South Asian	6.6	NA	8.4	--	--	--	10.6	25.4	20.0	NA	5.1	22.9
	OtherAsian	NA	NA	NA	--	--	--	NA	37.5	27.4	NA	11.1	NA
	Black	7.7	NA	18.8	--	--	--	5.9	35.8	19.0	NA	11.5	21.7
	Mixed	23.5	NA	11.1	--	--	--	8.3	27.7	22.9	NA	10	15.5
	Other	11.1	NA	4.2	--	--	--	8.5	30.2	28.6	NA	9.1	NA
	All ethnic Minorities	10.6	9.0	10.7	--	--	--	10.3	30.4	23.6	19.6	8.3	21.1
**Education**	*Higher Ed*	11.2	16.9	14.0	14.5	16.8	16.03	9.0	29.7	23.3	19.4	9.9	16.9
	A-level	14.8	14.4	10.5	15.5	14.0	22.67	9.2	27.0	26.7	20.0	10.3	20.5
	GCSE	6.3	18.1	11.3	12.0	17.6	15.6	9.0	31.3	29.3	17.8	9.2	17.4
	<GCSE/ None	6.2	12.4	14.5	15.5	17.2	16.3	9.1	39.0	27.8	23.9	6.1	22.4
**Social Class**	*Managerial/ Admin/ Professional*	11.6	16.4	11.1	12.6	12.7	17.0	9.7	25.7	24.3	16.4	-	18.3
	Intermediate	8.5	15.2	12.7	15.3	12.6	15.5	9.0	27.2	25.7	21.3	-	19.5
	Manual/Routine	11.2	16.7	11.6	11.6	13.6	18.6	9.3	27.6	25.6	19.6	-	23.4
	Other	6.0	0	18.0	19.3	21.1	0	11.8	42.6	51.9	20.0	-	16.6
**Not Instructed to Shield**		9.0	--	12.0	12.4	14.6	16.7	--	29.6	23.9	--	8.9	16.2
**Instructed to Shield**		47.5	--	44.3	49.4	41.9	28.4	--	61.0	42.0	--	15.3	35.5

Sources: MCS (Millennium Cohort Study); ALSPAC G1 (Children of the Avon Longitudinal Study of Parents and Children); NS (Next Steps); BCS 70 (1970 British Cohort Study), NCDS (National Child Development Study); NSHD (National Survey of Health and Development); BIB (Born in Bradford); ; USOC (Understanding Society); GS (Generation Scotland: the Scottish Family Health Study); ALSPAC G0 (parents of ALSPAC); TWINS UK (UK Adult Twin Registry); ELSA (English Longitudinal Study of Ageing). Notes: Samples for each study restricted to respondents with non-missing information on healthcare disruptions and valid information on sex, social class, education and (where applicable) age and ethnicity. All information about how information was collected and variables were coded is available in Supplementary File 1. NA= Not available; (--)= Info not collected. Weighted data where applicable

For peer review only

Supplementary File 1: Meta-analysis summary restricted to representative studies

Note: ALSPAC, GS, TwinsUK and BiB

excluded. **Summary of results**

		Any healthcare disruption				Appointments				Prescription/Medication				Procedures/surgery			
		OR	Lower CI	Upper CI	I^2%	OR	Lower CI	Upper CI	I^2%	OR	Lower CI	Upper CI	I^2%	OR	Lower CI	Upper CI	I^2%
**Sex**																	
Female vs. Male	Unadjusted[ǂ]	1.27	1.19	1.36	0	1.29	1.18	1.42	5.66	1.39	0.90	2.14	73.3	1.24	1.13	1.37	0
	**Basic adjustment**	**1.34**	**1.15**	**1.57**	**65.33**	**1.36**	**1.25**	**1.47**	**0**	**1.37**	**0.86**	**2.16**	**74.9**	**1.27**	**1.12**	**1.43**	**11.85**
	Full adjustment	1.34	1.15	1.56	61.89	1.34	0.94	1.91		1.99	0.77	5.12		1.21	1.01	1.44	
**Age**																	
16-24y vs 45-54y	Unadjusted	0.50	0.41	0.62		0.43	0.34	0.54		0.65	0.42	1.02		0.48	0.34	0.68	
	**Basic adjustment**	**0.49**	**0.39**	**0.60**		**0.42**	**0.33**	**0.52**		**0.62**	**0.39**	**0.97**		**0.47**	**0.33**	**0.66**	
	Full adjustment	0.47	0.37	0.61			no information				no information				no information		
25-34y vs 45-54y	Unadjusted	0.71	0.58	0.86		0.65	0.53	0.80		0.97	0.66	1.44		0.78	0.57	1.07	
	**Basic adjustment**	**0.70**	**0.58**	**0.85**		**0.64**	**0.52**	**0.79**		**0.97**	**0.65**	**1.43**		**0.77**	**0.56**	**1.06**	
	Full adjustment	0.77	0.63	0.94			no information				no information				no information		
35-44y vs 45-54y	Unadjusted	0.74	0.63	0.88		0.70	0.58	0.83		0.83	0.58	1.18		0.88	0.69	1.12	
	**Basic adjustment**	**0.74**	**0.63**	**0.87**		**0.69**	**0.58**	**0.82**		**0.83**	**0.58**	**1.18**		**0.87**	**0.68**	**1.11**	
	Full adjustment	0.86	0.73	1.03			no information				no information				no information		
55-64y vs 45-54y	Unadjusted	1.40	1.23	1.59	0	1.37	1.19	1.58	0	0.75	0.27	2.07	67.12	1.51	1.26	1.80	0
	**Basic adjustment**	**1.42**	**1.25**	**1.61**	**0**	**1.39**	**1.21**	**1.60**	**0**	**0.80**	**0.30**	**2.09**	**64.1**	**1.52**	**1.28**	**1.80**	**0**
	Full adjustment	1.21	1.06	1.40	0	1.04	0.48	2.25		0.52	0.16	1.68		1.37	0.93	2.01	

For peer review only

65-74y vs 45-54y	Unadjusted	1.72	1.51	1.96	0	1.57	1.21	2.04	16.91	0.76	0.31	1.86	59.63	1.93	1.63	2.30	0
	**Basic adjustment**	**1.78**	**1.56**	**2.02**	**0**	**1.67**	**1.42**	**1.97**	**2.81**	**0.85**	**0.38**	**1.91**	**52.96**	**1.98**	**1.67**	**2.34**	**0**
	Full adjustment	1.35	1.14	1.58	0	1.01	0.42	2.43		1.41	0.34	5.89		1.55	1.05	2.30	
75y+ vs 45-54y	Unadjusted	1.97	1.68	2.31	1.58	1.87	1.56	2.24	0	0.89	0.59	1.35	6.53	2.10	1.46	3.02	66.45
	**Basic adjustment**	**2.06**	**1.76**	**2.41**	**0**	**1.97**	**1.64**	**2.35**	**0**	**0.98**	**0.68**	**1.42**	**0**	**2.14**	**1.57**	**2.91**	**55.32**
	Full adjustment	1.38	1.13	1.70	0.00	1.07	0.44	2.61		1.26	0.39	4.02		1.75	1.17	2.62	
**Ethnicity**																	
Non-White vs White*	Unadjusted	0.96	0.82	1.12	0	1.02	0.72	1.46	44.43	1.02	0.39	2.67	84.87	0.90	0.71	1.14	0
	**Basic adjustment**	**1.23**	**1.05**	**1.44**	**0**	**1.25**	**0.87**	**1.81**	**48.35**	**1.06**	**0.42**	**2.67**	**83.18**	**1.16**	**0.91**	**1.47**	**0**
	Full adjustment	1.10	0.94	1.29	0	1.39	0.61	3.20		2.04	0.70	5.98		0.96	0.63	1.48	
Black vs White	Unadjusted	1.22	0.91	1.65	0	1.02	0.53	1.94	48.54	0.49	0.07	3.52	85.41	0.87	0.58	1.31	0
	**Basic adjustment**	**1.47**	**1.08**	**1.98**	**0**	**1.18**	**0.57**	**2.44**	**59.36**	**0.50**	**0.08**	**3.36**	**84**	**1.03**	**0.68**	**1.55**	**0**
	Full adjustment	1.20	0.92	1.58	0	0.88	0.18	4.22		0.37	0.04	3.11		0.87	0.41	1.82	
East Asian vs White	Unadjusted	0.82	0.38	1.73		0.79	0.35	1.80		0.97	0.47	1.97		1.38	0.47	4.02	
	**Basic adjustment**	**1.04**	**0.53**	**2.06**		**1.03**	**0.49**	**2.16**		**1.04**	**0.52**	**2.09**		**1.80**	**0.65**	**4.99**	
	Full adjustment	1.01	0.60	1.68		no information				no information				no information			
Mixed vs White	Unadjusted	1.13	0.82	1.57	0	1.27	0.49	3.29	77.57	1.53	0.90	2.60	0	1.12	0.70	1.80	0
	**Basic adjustment**	**1.38**	**0.88**	**2.17**	**34.69**	**1.47**	**0.59**	**3.67**	**75.14**	**1.67**	**0.98**	**2.86**	**0**	**no information**			
	Full adjustment	1.36	0.88	2.11	24.01	no information				0.93	0.10	8.48		0.85	0.32	2.21	
South Asian vs White	Unadjusted	0.76	0.58	1.01	29.78	0.84	0.56	1.25	38.69	0.80	0.17	3.77	93.25	0.70	0.45	1.09	28.01
	**Basic adjustment**	**1.02**	**0.84**	**1.24**	**0**	**1.05**	**0.84**	**1.31**	**0**	**0.83**	**0.18**	**3.76**	**92.41**	**0.90**	**0.64**	**1.26**	**0**
	Full adjustment	0.95	0.72	1.25	21.29	2.65	1.03	6.82		4.47	1.38	14.50		1.11	0.62	1.99	

For peer review only

Other Ethnicity vs White	Unadjusted	0.56	0.25	1.25	40.34	0.82	0.45	1.49	0	0.70	0.14	3.54	63.29	0.81	0.11	6.21	67.17
	**Basic adjustment**	**0.72**	**0.25**	**2.07**	**64.12**	**1.02**	**0.41**	**2.51**	**48.38**	**0.96**	**0.17**	**5.25**	**66.68**	**0.82**	**0.08**	**8.51**	**74.76**
	Full adjustment	0.72	0.25	2.02	64.08	no information				no information				no information			
**Education**																	
A-level/equivalent vs Higher education/Degree	Unadjusted	1.02	0.85	1.22	53.07	1.02	0.84	1.25	39.4	0.94	0.68	1.30	26.88	0.68	0.37	1.28	90.63
	**Basic adjustment**	**1.11**	**0.99**	**1.25**	**8.05**	**1.13**	**0.99**	**1.29**	**1.31**	**0.92**	**0.71**	**1.19**	**0**	**0.73**	**0.38**	**1.39**	**91.07**
	Full adjustment	0.98	0.85	1.12	21.18	0.98	0.59	1.63		3.39	1.04	11.09		1.05	0.83	1.32	
GCSE/equivalent vs Higher education/Degree	Unadjusted	0.96	0.84	1.10	36.2	0.96	0.84	1.11	19.4	0.95	0.69	1.30	39.93	1.01	0.89	1.14	0
	**Basic adjustment**	**0.94**	**0.79**	**1.12**	**55.76**	**0.91**	**0.73**	**1.13**	**53.46**	**0.96**	**0.68**	**1.35**	**45.45**	**1.04**	**0.92**	**1.19**	**0**
	Full adjustment	0.84	0.73	0.95	24.18	0.63	0.36	1.10		1.96	0.59	6.47		0.81	0.62	1.04	
<GCSE/equivalent vs Higher education/Degree	Unadjusted	1.13	0.89	1.43	72.27	1.06	0.83	1.36	61.17	1.22	0.77	1.94	58.91	1.38	1.21	1.58	0
	**Basic adjustment**	**1.12**	**0.96**	**1.30**	**33.28**	**1.04**	**0.85**	**1.27**	**34.32**	**1.25**	**0.88**	**1.78**	**27.88**	**1.20**	**1.04**	**1.38**	**0**
	Full adjustment	0.85	0.76	0.96	3.24	0.70	0.42	1.17		3.22	1.01	10.27		0.86	0.66	1.12	
**Occupational class**																	
Intermediate vs Managerial/Admin/Professional	Unadjusted	1.07	0.97	1.18	0	1.04	0.93	1.17	0	0.99	0.74	1.35	17.14	1.15	1.00	1.32	0
	**Basic adjustment**	**1.04**	**0.94**	**1.15**	**0**	**1.02**	**0.91**	**1.15**	**0**	**0.96**	**0.68**	**1.37**	**29.88**	**1.12**	**0.98**	**1.28**	**0**
	Full adjustment	0.97	0.88	1.08	0	1.13	0.67	1.90		0.74	0.21	2.59		1.05	0.84	1.31	
Manual/Routine vs Managerial/Admin/Professional	Unadjusted	1.13	0.99	1.29	29.12	1.06	0.94	1.18	0	1.30	1.00	1.68	9.9	1.13	0.91	1.41	33.75
	**Basic adjustment**	**1.20**	**1.09**	**1.32**	**0**	**1.15**	**1.03**	**1.30**	**0**	**1.35**	**1.01**	**1.81**	**16.85**	**1.20**	**1.05**	**1.37**	**0**
	Full adjustment	1.03	0.93	1.15	0	1.29	0.81	2.06		0.75	0.27	2.13		1.07	0.85	1.35	
Other social class vs	Unadjusted	1.36	0.90	2.06	92.46	1.40	1.01	1.94	79.32	2.03	1.25	3.29	73.7	1.55	0.90	2.68	87.81

Managerial/Admin/Professional	**Basic adjustment**	**1.48**	**1.10**	**2.00**	**81.34**	**1.51**	**1.18**	**1.93**	**56.45**	**2.44**	**1.71**	**3.49**	**45.49**	**1.64**	**1.10**	**2.46**	**72.22**
	Full adjustment	1.19	0.99	1.42	44.84	1.39	0.80	2.42		4.12	1.43	11.82		0.94	0.69	1.27	

Basic adjustment: sex, age, and ethnicity (where available)
Full adjustment: sex, age, and ethnicity (where available) , education, occupational class, UK Nation (where appropriate), household composition, and pre-pandemic self-reported health.

Empty I^2% column indicates only one study included

*Binary variable including Black, East Asian, Mixed, South Asian, and other ethnicity in 'non-White'

For peer review only

Summary of stratified results

		Any healthcare disruption			
**Sex**		OR	Lower CI	Upper CI	I2%
Female vs. Male	Overall	1.34	1.15	1.57	65.33
	Not shielding	1.32	1.09	1.61	75.25
	Shielding	1.48	1.20	1.83	0
	16-24y	2.21	1.61	3.03	3.99
	25-34y	1.45	0.86	2.43	63.72
	35-44y	1.48	1.14	1.92	
	45-54	1.97	1.61	2.42	0
	55-64	1.16	1.02	1.32	0
	75+	1.03	0.80	1.32	42.24
**Age**		OR	Lower CI	Upper CI	I2%
16-24y vs 45-54y	Overall	0.49	0.39	0.60	
	Not shielding	0.50	0.40	0.62	
	Shielding	0.64	0.23	1.78	
25-34y vs 45-54y	Overall	0.70	0.58	0.85	
	Not shielding	0.71	0.58	0.87	
	Shielding	0.86	0.34	2.16	
35-44y vs 45-54y	Overall	0.74	0.63	0.87	
	Not shielding	0.76	0.64	0.90	
	Shielding	0.48	0.24	0.96	
55-64y vs 45-54y	Overall	1.42	1.25	1.61	0
	Not shielding	1.37	1.20	1.57	0
	Shielding	1.32	0.80	2.17	0
65-74y vs 45-54y	Overall	1.78	1.56	2.02	0
	Not shielding	1.67	1.46	1.91	0
	Shielding	1.33	0.82	2.15	0
75y+ vs 45-54y	Overall	2.06	1.76	2.41	0
	Not shielding	1.96	1.66	2.33	0
	Shielding	1.07	0.65	1.78	0
**Ethnicity**		OR	Lower CI	Upper CI	I2%
Non-White vs White*	Overall	1.23	1.05	1.44	0

For peer review only

	Not shielding	0.96	0.62	1.48	73.47
	Shielding	1.56	0.97	2.49	0
	16-24y	1.24	0.84	1.82	0
	25-34y	0.70	0.47	1.04	0
	35-44y	1.42	0.94	2.12	
	45-54	1.71	1.20	2.44	0
	55-64	1.20	0.87	1.66	0
	75+	1.28	0.67	2.45	0
Black vs White	Overall	1.47	1.08	1.98	0
	Not shielding	0.84	0.38	1.83	72.85
	Shielding	1.49	0.59	3.78	0
	16-24y	1.15	0.51	2.59	0
	25-34y	0.74	0.30	1.86	16.69
	35-44y	2.11	0.87	5.12	
	45-54	1.99	0.93	4.25	15.25
	55-64	1.74	1.03	2.95	0
	75+	1.23	0.42	3.56	0
East Asian vs White	Overall	1.04	0.53	2.06	
	Not shielding	1.04	0.52	2.11	
	Shielding				
	16-24y	0.01	0.00	0.05	
	25-34y	0.57	0.12	2.62	
	35-44y	1.55	0.69	3.48	
	45-54	1.62	0.42	6.18	
	55-64	0.90	0.36	2.21	
	75+				
Mixed vs White	Overall	1.38	0.88	2.17	34.69
	Not shielding	1.28	0.88	1.86	0
	Shielding	1.89	0.64	5.55	0
	16-24y	2.50	1.25	5.02	0
	25-34y	1.09	0.61	1.95	0
	35-44y	2.47	0.88	6.95	
	45-54	1.01	0.48	2.14	
	55-64	1.19	0.56	2.51	0
	75+	1.47	0.34	6.42	22.46
South Asian vs White	Overall	1.02	0.84	1.24	0
	Not shielding	0.92	0.64	1.34	42.86
	Shielding	1.30	0.72	2.36	0
	16-24y	0.98	0.62	1.53	13.95

For peer review only

	25-34y	0.43	0.26	0.72	2.58
	35-44y	0.91	0.58	1.42	
	45-54	2.55	0.59	10.92	86.27
	55-64	0.90	0.47	1.74	19
	75+	1.11	0.40	3.12	0
Other Ethnicity vs White	Overall	0.72	0.25	2.07	64.12
	Not shielding	0.63	0.20	1.95	62.21
	Shielding	0.19	0.01	4.52	
	16-24y	0.18	0.00	15.35	88.56
	25-34y	0.57	0.10	3.20	70.09
	35-44y	1.52	0.36	6.41	
	45-54	1.12	0.37	3.38	
	55-64	0.49	0.12	1.96	
	75+	4.18	0.35	50.04	
**Education**		OR	Lower CI	Upper CI	I2%
A-level/equivalent vs Higher education/Degree	Overall	1.11	0.99	1.25	8.05
	Not shielding	1.02	0.85	1.23	47.74
	Shielding	0.92	0.66	1.30	0
	16-24y	1.39	0.96	2.01	0
	25-34y	0.97	0.55	1.71	52.33
	35-44y	1.48	1.00	2.18	
	45-54	1.10	0.86	1.40	0
	55-64	0.99	0.76	1.29	44.12
	75+	0.77	0.57	1.05	0
GCSE/equivalent vs Higher education/Degree	Overall	0.94	0.79	1.12	55.76
	Not shielding	0.93	0.79	1.10	47.54
	Shielding	0.80	0.60	1.06	0
	16-24y	0.93	0.36	2.40	83.45
	25-34y	1.05	0.53	2.07	70.84
	35-44y	1.19	0.86	1.64	
	45-54	1.00	0.70	1.44	60.4
	55-64	1.06	0.91	1.24	0
	75+	0.88	0.59	1.31	54.52
<GCSE/equivalent vs Higher education/Degree	Overall	1.12	0.96	1.30	33.28
	Not shielding	1.01	0.83	1.23	50.08
	Shielding	0.86	0.63	1.18	8.77
	16-24y	0.79	0.38	1.61	46.71
	25-34y	1.31	0.61	2.81	62.99

For peer review only

		OR	Lower CI	Upper CI	I2%
	35-44y	0.87	0.56	1.36	
	45-54	1.32	0.85	2.06	61.45
	55-64	1.18	0.97	1.43	0
	75+	0.98	0.78	1.24	0
**Occupational class**		OR	Lower CI	Upper CI	I2%
Intermediate vs Managerial/Admin/Professional	Overall	1.04	0.94	1.15	0
	Not shielding	1.04	0.94	1.15	0
	Shielding	0.86	0.59	1.25	13.43
	16-24y	0.88	0.55	1.41	0
	25-34y	1.25	0.86	1.81	0
	35-44y	1.13	0.81	1.58	
	45-54	1.13	0.92	1.39	0
	55-64	0.92	0.77	1.11	0
	75+	1.02	0.76	1.37	0
Manual/Routine vs Managerial/Admin/Professional	Overall	1.20	1.09	1.32	0
	Not shielding	1.20	1.08	1.33	0
	Shielding	0.94	0.71	1.24	0
	16-24y	1.14	0.74	1.75	0
	25-34y	1.55	0.97	2.48	36.45
	35-44y	1.23	0.88	1.71	
	45-54	1.04	0.85	1.27	0
	55-64	1.14	0.95	1.37	0
	75+	1.29	0.98	1.70	0
Other social class vs Managerial/Admin/Professional	Overall	1.48	1.10	2.00	81.34
	Not shielding	1.44	1.10	1.89	73.49
	Shielding	0.92	0.38	2.22	82.67
	16-24y	1.01	0.34	2.95	79.64
	25-34y	2.09	1.40	3.13	0
	35-44y	2.16	1.34	3.48	
	45-54	2.05	0.98	4.29	85.15
	55-64	1.73	1.28	2.33	64.79
	75+	1.02	0.62	1.69	0

Adjusted for sex, age, and ethnicity (where available)

Empty I^2% column indicates only one study included

*Binary variable including Black, East Asian, Mixed, South Asian, and other ethnicity in 'non-White'

For peer review only

Supplementary File 2: Variable coding

Contents

Healthcare disruptions 2

Covariates 6

A note about shielding 11

For peer review only

For peer review only - http://bmjopen.bmj.com/site/about/guidelines.xhtml

Healthcare disruptions

Study	*Question (exact wording)*	*Possible Answers*	*Recoding if needed*
*** PRESCRIPTION or MEDICATION ACCESS ***			
**MCS NS BCS 70 NCDS NSHD**	Since the Coronavirus outbreak in March, have you had any difficulty obtaining any of your prescribed medication?	1=Yes; 2=No/Not applicable	= 1
**ALSPAC**	Not Available		
**USOC**	Q1: Still thinking about your situation now, have you been able to access the NHS services you need: Prescription medicine?<br>Q2: Still thinking about your situation now, have you been able to access the community health and social care services and support you need... Over the counter medications?	For both Q1 and Q2:<br>1=Yes; 2=No; 3=Not required	Q1=2 OR Q2=2
**ELSA**	Since the coronavirus outbreak, have you been able to get access to your regular medications?	1=Yes; 2=No; 3=No need	= 2
**GS**	How strongly do you agree with the following statements: Accessing and remembering to take my medication has become more difficult during the COVID-19 pandemic	From 1 (do not agree at all) to 10 (agree very strongly)	=6/10
**TWINS UK**	Have you experienced any of the following as a result of COVID-19? Unable to access required medication	0= No; 1 = Yes	= 1
**BIB**	Q1: Have you or a member of your household needed to access pharmacy services since lockdown began?<br>Q2: If yes, did you receive the support you needed?	Q1. 0=No; 1=Yes<br>Q2. 0= No; 1=Yes; 2=Haven't tried	Q1=1 & Q2=0
*** PROCEDURES or SURGERIES ***			
**MCS NS BCS 70 NCDS NSHD**	Q1: At the time of the Coronavirus outbreak in March, did you have an in-patient or out-patient appointment booked at a hospital for a consultation, investigation, treatment or surgery?<br>Q2: Have you now had your surgery?<br>Q3: Did your (last) surgery take place on the planned date or was it delayed?<br>Q4: Why has your surgery not taken place?	Q1. 1=Yes - for a consultation investigation or treatment; 2=Yes - for surgery; 3=No.<br>Q2. 1=Yes; 2=No.<br>Q3. 1=Surgery took place on the planned date; 2=Surgery was delayed.<br>Q4. 1=My surgery was postponed and	Q1=2 & Q2=1 & Q3=2<br>*OR*<br>Q1=2 & Q2=2 & Q4=(1 OR 3)

		has not yet happened; 2=My surgery was not postponed, but it hasn't happened yet; 3=My surgery was cancelled	
**ALSPAC GS**	Q1: Have you had any medical treatments or appointments that have had to be cancelled or postponed during the COVID-19 pandemic? For example, hospital referral, non-emergency surgery, cancer, treatment, etc.<br>Q2: What types of medical treatments or appointments were cancelled or postponed?	Q1. 1=Yes; 2=No.<br>Q2. a -- surgery: 1=Yes; -9=Not applicable<br>b -- cancer treatment: 1=Yes; -9=Not applicable<br>c -- dialysis: 1=Yes; -9=Not applicable	Q1=1 & Q2 (a OR b OR c)=1
**USOC**	Q1: [since previous survey] have you had or been waiting for NHS treatment? Please select all that apply.<br>Q2: Has your treatment plan(s) been changed in any way?	Q1. 1=Yes, tests/consultations planned or in progress; 2=Yes, operation or procedure planned; 3=Yes, targeted therapy, chemotherapy or radiotherapy planned or in progress; 4=Yes, other treatment planned; 5=No<br>Q2. 1=Yes, consultations/treatments cancelled or postponed by NHS; 2=Yes, alternative treatment provided; 3=Yes, I cancelled or postponed treatment; 4=No, treatment continuing as planned	Q1=2/4 & Q2=1/3
**ELSA**	Since the coronavirus outbreak, have you had a hospital operation or treatment cancelled?	1.Yes; 2.No	=1
**TWINS UK**	Not Available		
**BIB**	Not Available		
*** APPOINTMENTS ***			
**MCS<br>NS<br>BCS 70<br>NCDS<br>NSHD**	Q1: At the time of the Coronavirus outbreak in March, did you have an in-patient or out-patient appointment booked at a hospital for a consultation, investigation, treatment or surgery?<br>Q2: Have you now had your in/ out-patient hospital appointment for a consultation, investigation or treatment?<br>Q3: Did your (last) appointment take place on the planned date or was it delayed?<br>Q4: Why has your in-/out-patient hospital appointment for a consultation, investigation or treatment not taken place?	Q1. 1=Yes - for a consultation investigation or treatment; 2=Yes - for surgery; 3=No.<br>Q2. 1=Yes; 2=No.<br>Q3. 1=Appointment took place on the planned date; 2=Appointment was delayed.<br>Q4. 1=My appointment was postponed	Q1=1 & Q2=1 & Q3=2 OR<br>Q1=1 & Q2=2 & Q4=(1 OR 3)

For peer review only - http://bmjopen.bmj.com/site/about/guidelines.xhtml

		and has not yet happened; 2=My appointment was not postponed, but it hasn't happened yet; 3=My appointment was cancelled	
**ALSPAC GS**	Q1. Have you had any medical treatments or appointments that have had to be cancelled or postponed during the COVID-19 pandemic? For example, hospital referral, non-emergency surgery, cancer, treatment, etc.<br>Q2. What types of medical treatments or appointments were cancelled or postponed?	Q1. 1=Yes; 2=No.<br>Q2= d -- GP referral: 1=Yes; -9=Not applicable<br>e -- Hospital referral: 1=Yes; -9=Not applicable<br>f -- Routine clinical appointment: 1=Yes; -9=Not applicable<br>g -- Cancer testing: 1=Yes; -9=Not applicable<br>h -- Cancer screening: 1=Yes; -9=Not applicable	Q1=1 & Q2(d OR e OR f OR g OR h)=1
**USOC**	Thinking about your situation now, have you been able to access the NHS services you need to help manage your condition(s) over the last 4 weeks?<br>Q1: GP or primary care practice staff?<br>Q2: Hospital or clinic outpatient?<br>Q3: Hospital or clinic inpatient?<br>Q4: [since previous survey] have you had or been waiting for NHS treatment? Please select all that apply.<br>Q5: Has your treatment plan(s) been changed in any way?	Q1-3. 1=Yes, in person; 2=(Q1 & Q2 only) Yes, online or by phone only; 3=No, not able to access; 4=No, decided not to seek help at this time/cancelled; 5=Alternative treatment provided; 6=Not required<br>Q4. 1=Yes, tests/consultations planned or in progress; 2=Yes, operation or procedure planned; 3=Yes, targeted therapy, chemotherapy or radiotherapy planned or in progress; 4=Yes, other treatment planned; 5=No<br>Q5. 1=Yes, consultations/treatments cancelled or postponed by NHS; 2=Yes, alternative treatment provided; 3=Yes, I cancelled or postponed treatment; 4=No, treatment continuing as planned	Q1|Q2|Q3=(3 OR 4 OR 5)<br>OR<br>Q4=1 AND Q5=(1 OR 2 OR3)
**ELSA**	Q1: Since the coronavirus outbreak, have you wanted to see or talk to a GP?<br>Q2: Have you been able to see or talk to a GP?	Q1: 1=Yes; 2=No<br>Q2: 1=Yes; 2=No; 3=I did not attempt to contact them 4.I did not need to contact them	Q1= & Q2=2
**TWINS UK**	Not Available		

**BIB**	Q1: Have you or a member of your household needed to access<br>-- (1) your doctor (GP) or nurse<br>-- (2) NHS111<br>-- (3) Health emergency services (A&E)<br>-- (4) A specialist (consultant) doctor or specialist clinic (hospital outpatient) appointment since lockdown began?<br>Q2: If yes, were you able to access (1, 2, 3, or 4)?	Q1. 0=No; 1=Yes<br>Q2. 0= No; 1=Yes; 2=Haven't tried	Q1= & Q2=0

For peer review only

For peer review only - http://bmjopen.bmj.com/site/about/guidelines.xhtml

Covariates

Variables	*Study*	*Options*	*Recoding if needed*
*** Sex * 0=Male; 1=Female**			
	**All**	0=Male; 1=Female	
*** Ethnicity * 0=White; 1=South East Asian; 2=Other Asian; 3=Black; 4=Mixed; 5=Other Non-White**			
	**MCS**	1=White; 2=Mixed; 3=Indian; 4=Pakistani; 5=Bangladeshi; 6=Other Asian; 7=Black Caribbean; 8=Black African; 9=Other Black; 10=Chinese; 11=Other ethnic group	1=0, 2=4, 3-5=1, 6 & 10=2, 7-9=3, 11=5
	**NS**	1=White; 2=Mixed; 3=Indian; 4=Pakistani; 5=Bangladeshi; 6=Black Caribbean; 7=Black African; 8=Other	1=0, 2=4, 3-5=1, 6-7=3, 8=5
	**BCS70**	Not Available	
	**NCDS**	Not Available	
	**NSHD**	Not Available	
	**ALSPAC**	G0 (Parents) 1=White; 2=Black Caribbean; 3=Black African; 4=Other black; 5=Indian; 6=Pakistani; 7=Bangladeshi; 8=Chinese; 9=Other<br>G1 (Children) 1=White; 2=Mixed/Multiple Ethnic group; 3=Asian; 4=Black/African/Caribbean/Black British; 5=Arab or Other	G0: 1=0; 5/7=1, 8=2, 2/4=3, 9=5<br>G1: 1=0; 3=2, 4=3, 2=4
	**USOC**	1=White British; 2=Irish (White); 3=Gypsy or Irish Traveller (white); 4=Any other white background; 5=White and black Caribbean (mixed); 6=White and black African (mixed); 7=White and Asian (mixed); 8=Any other mixed background; 9=Indian (Asian or Asian British); 10=Pakistani (Asian or Asian British); 11=Bangladeshi (Asian or Asian British); 12=Chinese (Asian or Asian British); 13=Any other Asian background (Asian or Asian British); 14=Caribbean (Black or Black British); 15=African (Black or Black British); 16=Any other Black background (Black or Black British); 17=Arab (other Ethnic group); 97=Any other ethnic group	1-4=0, 5-8=4, 9-11=1, 12-13=2, 14-16=3, 17-97=5
	**ELSA**	1.White; 2=Mixed ethnic group; 3=Black; 4=Black British; 5=Asian; 6=Asian British	1=0; 2=4; 3/4=3; 5/6=1
	**GS**	1=White Scottish; 2=White English; 3=White Welsh; 4=White N. Irish; 5=White Irish; 6=White Gypsy/Irish traveller; 7=White Polish; 8=Any other white; 9=Asian/British Asian - Indian; 10=Asian/British Asian - Pakistani; 11=Asian/British Asian - Bangladeshi; 12=Asian/British Asian - Chinese; 13=Any other Asian background; 14=Black or Black British - African; 15=Black or Black British - Caribbean; 16=Any other Black/African/Caribbean background; 17=Arab or Arab British; 18=Mixed - White and Black Caribbean; 19=Mixed - White and Black African; 20=Mixed - White and Asian; 21=Any other Mixed/Multiple ethnic background; 22=Any other ethnic group	1/8=0, 9/11=1, 12/13=2, 14/16=3, 18/21=4, 17&22=5

For peer review only - http://bmjopen.bmj.com/site/about/guidelines.xhtml

	**TWINS UK**	1=White- English, Welsh, Scottish, Northern Irish, Irish; 2=White- Other white background; 3=Mixed/multiple ethnic groups - White and Black Caribbean; 4=Mixed/multiple ethnic groups - White and Black African; 5=Mixed/multiple ethnic groups - White and Asian; 6=Mixed/multiple ethnic groups - Other mixed/ multiple ethnic background; 7=Asian/Asian British- Indian; 8=Asian/Asian British - Pakistani; 9=Asian/Asian British - Bangladeshi; 10=Asian/Asian British - Chinese; 11=Asian/Asian British - Other Asian background; 12=Black/Black British - African; 13=Black/Black British - Caribbean; 14=Black/Black British - Other Black Background; 15=Middle-Eastern; 16=Other ethnic group	1/2=0; 10=1; 7/9|11=2; 12/14=3; 3/6=4; 15/16=5
	**BIB**	BiB: 1=White British; 2=White other; 3=Mixed-White and Black; 4=Mixed-White and South Asian; 5=Black; 6=Indian; 7=Pakistani; 8=Bangladeshi; 9=Other<br>BIBBS: 1=White British; 2=White Irish; 3=Pakistani; 4=Indian; 5=Bangladeshi; 6=White Polish; 7=White Slovakian; 8=White Romanian; 9=White Czech; 10=Other White; 11=White Gypsy/Roma/Irish traveller; 12=Chinese; 13=African; 14=Caribbean; 15=Mixed White/Black Caribbean; 16=Mixed White/Black African; 17=Mixed White/Asian; 18=Do not wish to answer; 19=Other	BiB: 1/2=0; 6/8=1; 5=3; 3/4=4; 9=5<br>BiBBs: 1/2=0; 6/11=0; 3/5=1; 13/14=3; 15/18=4; all other options=5
*** Education * 0= Degree; 1=A-Level; 2=GCSE; 3=Low or None**			
	**MCS**<br>**NS**<br>**BCS 70**<br>**NCDS**	0=None; 1=Nvq1; 2=Nvq2; 3=Nvq3; 4=Nvq4; 5=Nvq5<br>*parent's education for MCS	0/1 = 0 2=1 3=2 4/5=3
	**NSHD**	0=None attempted; 1.=Vocational course, proficiency only; 2=Sub GCE or sub Burnham C; 3=GCE 'O' level or Burnham C; 4=GCE 'A' Level or Burnham B; 5=Burnham A2; 6= 1st Degree or graduate equivalent; 7= Higher degree, Masters; 8= Higher degree, doctorate; 9=Unknown	6|7|8=0; 4|5=1; 3=2; 0|1|2|9=3
	**ALSPAC**	1=Degree; 2=A levels/AS levels or equivalent; 3=O levels; 4=Vocational; 5=CSE<br>*parent's education for G1 (Children)	1=0; 2=1; 3=2; 4/5=3
	**USOC**	1.Higher degree 2. 1st degree or equivalent 3. Diploma in Higher Education 4. Teaching qualification (not PGCE) 5. Nursing or other medical qualification 6. Other higher degree 7. A-Level 8. Welsh baccalaureate 9. International baccalaureate 10. AS Level 11. Scottish Highers 12. Certificate of 6th year studies 13. GCSE/O-Level 14. Certificate of secondary education 15. Standard or lower 16. Other school certificate 96. No qualifications	1-6=0, 7-12=1, 13-16=2, 96=3
	**ELSA**	1=Nvq4/nvq5/degree or equivalent; 2=Higher Education below degree; 3=Nvq3/GCE A level equivalent; 4=Nvq2/GCE O level equivalent; 5=Nvq1/CSE other grade equivalent; 6=Foreign/other; 7=No qualification	1=0; 2/3=1; 4=2; 5/7=3
	**GS**	1=No qualifications; 2=Other (please specify); 3=School leavers certificate; 4=CSEs or equivalent; 5=Standard grade, National 4 or 5, O levels, GCSEs or equivalent; 6=Higher grade, A levels, AS levels or equivalent; 7=NVQ or HND or HNC or equivalent; 8=Other professional or technical qualification; 9=Undergraduate degree; 10=Postgraduate degree	9|10=0; 6|7|8 =1; 5=2; <5=3

For peer review only - http://bmjopen.bmj.com/site/about/guidelines.xhtml

**TWINS UK**	1=No qualification; 2=NVQ1/SVQ1; 3=O-level/GCSE/NVQ2/SVQ2/Scottish intermediate; 4=Scottish Higher, NVQ3, City and Guilds, Pitman; 5=A-level, Scottish Advanced Higher; 6=Higher vocational training (e.g. Diploma, NVQ4, SVQ4); 7=Undergraduate degree; 8=Postgraduate degree (e.g. Masters or PhD), NVQ5, SVQ5	6/8=0; 4/5=1; 3=2; 1/2=3
**BIB**	1=<5 GCSE equivalent; 2=5 GCSE equivalent; 3=A-level equivalent; 4=Higher than A-level; 5=Other; 6=Don't know; 7=Foreign unknown	4=0; 3=1; 5/7=2; 1=3; missing=1

*** Occupational Social Class * 1=Managerial/Admin/Professional; 2=Intermediate; 3=Manual/routine; 4=Other**		
NS-SEC: National Statistics Socioeconomic Classification. RGSC: Registrat General's Social Class. ONS SOC: Office of National Statistics Standard Occupational Classification		
**MCS**<br>**NS**<br>**BCS 70**<br>**NCDS**	[NS-SEC] 1=Higher managerial and professional; 2=Lower managerial and professional; 3=Intermediate occupations; 4=Small employers and own account workers; 5=Lower supervisory and technical; 6=Semi-routine occupations; 7=Routine occupations; 8=Never worked and long-term unemployed<br>*parent's occupational social class for MCS	2=1; 3-4=2; 5-7=3; 8=4
**NSHD**	[RGSC] 1=I Professional; 2=II Managerial and Technical; 3=IIINM Skilled non-manual; 4=IIIM Skilled manual; 5=IV Partly skilled; 6=V Unskilled;	2=1; 3/5=2; 6=3;
**ALSPAC**	[RGSC] 1=I Professional; 2=II Managerial and Technical; 3=IIINM Skilled non-manual; 4=IIIM Skilled manual; 5=IV Partly skilled; 6=V Unskilled; 7=Armed Forces<br>*parent's occupational social class for G1 (Children)	2=1; 3/5=2; 6=3; 7=4
**USOC**	[NS-SEC] 1=Higher managerial and professional; 2=Lower managerial and professional; 3=Intermediate occupations; 4=Small employers and own account workers; 5=Lower supervisory and technical; 6=Semi-routine occupations; 7=Routine occupations; 8=Never worked and long-term unemployed	2=1; 3-4=2; 5-7=3; 8=4
**ELSA**	[NS-SEC] -3=Incomplete/No job info; 1=Higher and Lower managerial/ professional; 2=Intermediate occupations; 3=Routine and manual occupations; 99=Other	99=4; -3=4
**GS**	[ONS SOC] 1=Managers, directors, senior officials; 2=Associate professional and technical occupations; 3=Administrative and secretarial occupations; 4=Skilled trades occupations; 5=Sales and customer service occupations; 6=Process, plant and machine operatives; 7=Elementary (unskilled) occupations; 8=Never worked	1/3=1; 4/5=2; 6/7=3; 8=4
**TWINS UK**	Not Available	
**BIB**	1=Modern professional occupations; 2=Clerical and intermediate occupations; 3=Senior managers or administrators; 4=Technical and craft occupations; 5=Semi-routine manual and service occupations; 6=Routine manual and service occupations; 7=Middle or junior managers; 8=Traditional professional occupations;<br>9=Self-employed; 10=Student/in training; 11=Does not work-long term unemployed/sick; 12=Don't know<br>*Based on either own class (80.7%) or partner's (19.3%)	3=1; 8=1; 4=2; 7=2; 5/6=3; all other options=4

*** Living Arrangement * 1=Alone; 2=With partner/spouse only; 3=With partner/spouse and child(ren); 4=With child(ren), without partner/spouse; 5=Any other living arrangement<br><br>OR * Partnership Status * 1=Married/Partnered; 0=Not married/partnered**		
**MCS<br>NS<br>BCS 70<br>NCDS**	Who do you currently live with? 1. Husband/Wife/Cohabiting Partner2. Children (including adult children, step-children, adopted children, foster children or any other children you consider yourself parent to) 3. Parent or Parent-in-law (including step-parent or adoptive parent) 4. Grandparent 5. Grandchild 6. Sibling 7. Other relative 8. Friend / unrelated sharer 9. Other	1 = Husband/Wife/Cohabiting Partner; 0 = Other
**NSHD**	Who do you currently live with? (Options include Husband/Wife/Cohabiting Partner)	1= Partner in HH 0= No partner in household
**ALSPAC**	NA	NA
**USOC**	Derived from Household Grid	0=partner present; 1=Single
**ELSA**	IF respondents live with other people, they are asked for each person "what is this person's relationship to you". Options include "1. Husband/wife/partner"	1=Partner in HH 0=No partner in HH
**GS**	1. Married/ Civil partnership 2. In a relationship, living together 3. In a relationship, not living together 4. Single 5. Separated 6. Divorced 7. Widowed 8. Other	1-3=1 4-8 = 0
**TWINS UK**	Single, never married (1); Single, divorced or widowed (2); In a relationship/married but living apart (3); In a relationship/married and cohabiting (4)	1, 2 = 0; 3, 4 = 1
**BIB**	What is your current relationship status? 0=do not wish to answer; 1=single; 2=married; 3=not married but in a relationship	1=0; 2/3=1
*** Shielding Status * 1=Advised to Shield; 0=Not advised to shield**		
**MCS<br>NS<br>BCS 70<br>NCDS<br>NSHD**	Did you at any time receive a letter or text message from the NHS or Chief Medical Officer saying that you have been identified as someone at risk of severe illness if you catch Coronavirus, because you have an underlying disease or health condition? 1=Yes; 2=No	2=0
**ALSPAC**	Not Available	

**USOC**	Have you received a letter, text or email from the NHS or Chief Medical Officer saying that you have been identified as someone at risk of severe illness if you catch coronavirus, because you have an underlying disease or health condition? 1=Yes; 2=No	2=0
**ELSA**	Have you been contacted by the NHS or your GP and advised that you are vulnerable and at risk of severe illness if you catch coronavirus (Covid-19), and should stay at home at all times and avoid any face-to-face contact? 1=Yes; 2=No	2=0
**GS**	Have you been contacted by letter or text message to say you are at sever risk from COVID-19 due to and underlying health condition and should be shielding? 1=Yes; 2=No	2=0
**TWINS UK**	Have you received a letter or text message over the past few months to say you are at high risk from COVID-19 due to an underlying health condition, and should be 'shielding'? 1=Yes; 2=No	2=0
**BIB**	Have you been advised by a health professional that you are high risk or vulnerable and should self-isolate for 12 weeks to protect yourself from coronavirus? 0=No; 1=Yes	

*** Pre-Pandemic Self-Assessed Health * 1=Good/Very Good/Excellent; 0=Fair/Poor**		
**MCS**<br>**NS**<br>**BCS 70**<br>**NCDS**<br>**NSHD**	In general, in the 3 months before the Coronavirus outbreak would you say your health was … 1=Excellent; 2=Very Good; 3=Good; 4=Fair; 5=Poor	1/3=1; 4/5=0
**ALSPAC (G0 & G1)**	(2020) Do you have a history of diabetes (A), obesity (B) or asthma (C)?	1 if A & B & C==0<br>0 if A | B | C==1
**USOC**	(2018/19) In general, would you say your health is… 1=Excellent; 2=Very Good; 3=Good; 4=Fair; 5=Poor	1/3=1; 4/5=0
**ELSA**	(2018/19) Would you say your health is… 1=Excellent; 2=Very Good; 3=Good; 4=Fair; 5=Poor	1/3=1; 4/5=0
**GS**	NA	
**TWINS UK**	(2020) In general, would you say your health is… 1=Excellent; 2=Very Good; 3=Good; 4=Fair; 5=Poor	1/3=1; 4/5=0
**BIB**	(2016 - 2020) In general, would you say your health is… 1=Excellent; 2=Very Good; 3=Good; 4=Fair; 5=Poor	1/3=1; 4/5=0

For peer review only - http://bmjopen.bmj.com/site/about/guidelines.xhtml

A note about shielding

Who had to shield?

Initially 1.5 million, increasing to 2.2 million, people in the UK were identified as clinically extremely vulnerable (CEV) by their GP. They were sent a letter asking them to shield – not go out – for at least 12 weeks until the end of June. This timeframe was extended, and on 1st August, CEV individuals in England, Scotland and Northern Ireland were told that shielding had been paused. In Wales shielding continued until 16th August.

Who was classed as clinically extremely vulnerable?

People falling into the clinically extremely vulnerable group include:

- Solid organ transplant recipients
- People with cancer who are undergoing active chemotherapy or radical radiotherapy for lung cancer
- People with cancers of the blood or bone marrow such as leukaemia, lymphoma or myeloma who are at any stage of treatment
- People having immunotherapy or other continuing antibody treatments for cancer
- People having other targeted cancer treatments which can affect the immune system, such as protein kinase inhibitors or PARP inhibitors (which prevent cancer cells from repairing)
- People who have had bone marrow or stem cell transplants in the last 6 months, or who are still taking immunosuppression drugs
- People with severe respiratory conditions including all cystic fibrosis, severe asthma and severe chronic obstructive pulmonary disease (COPD)
- People with rare diseases and inborn errors of metabolism that significantly increase the risk of infections such as Severe combined immunodeficiency (SCID) or homozygous sickle cell)
- People on immunosuppression therapies sufficient to significantly increase risk of infection
- Women who are pregnant with significant heart disease, congenital or acquired.

Source:
https://web.archive.org/web/20200330181117/https://www.gov.uk/government/publications/covid-19-guidance-on-social-distancing-and-for-vulnerable-people/guidance-on-social-distancing-for-everyone-in-the-uk-and-protecting-older-people-and-vulnerable-adults

For peer review only

For peer review only - http://bmjopen.bmj.com/site/about/guidelines.xhtml

Supplementary File 3: Meta-analysis results

Contents

Summary of results....3
Summary of stratified results....6
Any healthcare disruption....10
Sex....10
Unadjusted....10
Basic adjustment....11
Full adjustment....12
Occupational class....13
Unadjusted....13
Basic adjustment....15
Full adjustment....17
Education....19
Unadjusted....19
Basic adjustment....21
Full adjustment....23
Age....25
Unadjusted....25
Basic adjustment....27
Full adjustment....29
Ethnicity....31
Unadjusted....31
Basic adjustment....34
Full adjustment....38
Appointments....41
Sex....41
Unadjusted....41
Basic adjustment....42
Full adjustment....43
Occupational class....44
Unadjusted....44
Basic adjustment....46
Full adjustment....48
Education....50
Unadjusted....50
Basic adjustment....52
Full Adjustment....54
Age....56
Unadjusted....56
Basic adjustment....58
Full adjustment....60
Ethnicity....62
Unadjusted....62
Basic adjustment....65
Full adjustment....68
Prescription/Medication access....71
Sex....71
Unadjusted....71
Basic adjustment....72
Full adjustment....73
Occupational class....74

For peer review only - http://bmjopen.bmj.com/site/about/guidelines.xhtml

Unadjusted........ 74
Basic adjustment........ 76
Full adjustment........ 78
Education........ 80
Unadjusted........ 80
Basic adjustment........ 82
Full adjustment........ 84
Age........ 86
Unadjusted........ 86
Basic adjustment........ 89
Full adjustment........ 91
Ethnicity........ 93
Unadjusted........ 93
Basic adjustment........ 96
Full adjustment........ 99
Procedures/surgery........ 102
Sex........ 102
Unadjusted........ 102
Basic adjustment........ 103
Full adjustment........ 104
Occupational class........ 105
Unadjusted........ 105
Basic adjustment........ 107
Full adjustment........ 109
Education........ 111
unadjusted........ 111
Basic adjustment........ 113
Full adjustment........ 115
Age........ 117
Unadjusted........ 117
Basic adjustment........ 120
Full adjustment........ 122
Ethnicity........ 123
Unadjusted........ 123
Basic adjustment........ 126
Full adjustment........ 129
Any healthcare disruption stratified by shielding status........ 131
Sex........ 131
Occupational class........ 132
Education........ 136
Age........ 140
Ethnicity........ 143
Any healthcare disruption stratified by age........ 148
Sex........ 148
Occupational class........ 149
Education........ 152
Ethnicity........ 155

For peer review only - http://bmjopen.bmj.com/site/about/guidelines.xhtml

Summary of results

		Any healthcare disruption				Appointments				Prescription/Medication				Procedures/surgery			
		OR	Lower CI	Upper CI	I^2%	OR	Lower CI	Upper CI	I^2%	OR	Lower CI	Upper CI	I^2%	OR	Lower CI	Upper CI	I^2%
**Sex**																	
Female vs. Male	Unadjusted	1.26	1.14	1.39	58.51	1.30	1.12	1.52	73.54	1.33	1.00	1.77	75.15	1.12	0.93	1.36	60.28
	**Basic adjustment**	**1.27**	**1.15**	**1.40**	**53.11**	**1.33**	**1.17**	**1.52**	**60**	**1.27**	**0.94**	**1.74**	**77.98**	**1.15**	**0.97**	**1.37**	**47.79**
	Full adjustment	1.30	1.15	1.46	44.89	1.41	1.10	1.82	0.00	1.18	0.50	2.77	59.02	1.09	0.77	1.55	18.15
**Age**																	
16-24y vs 45-54y	Unadjusted	0.77	0.41	1.47	71.12	0.55	0.26	1.20	55.5	1.17	0.34	4.05	86.43	0.48	0.34	0.68	
	**Basic adjustment**	**0.76**	**0.39**	**1.46**	**71.95**	**0.55**	**0.24**	**1.23**	**58.53**	**1.09**	**0.33**	**3.67**	**85.53**	**0.47**	**0.33**	**0.66**	
	Full adjustment	0.85	0.32	2.24	70.28	0.89	0.18	4.36		4.71	1.40	15.86		no information			
25-34y vs 45-54y	Unadjusted	0.87	0.71	1.07	51.9	0.71	0.53	0.96	72.25	1.23	0.75	2.03	74.54	0.78	0.59	1.02	0
	**Basic adjustment**	**0.85**	**0.70**	**1.04**	**47.62**	**0.67**	**0.57**	**0.77**	**0**	**1.16**	**0.68**	**1.96**	**76.95**	**0.77**	**0.58**	**1.03**	**0**
	Full adjustment	0.92	0.74	1.15	43.99	1.21	0.66	2.22		1.18	0.52	2.67	0	no information			
35-44y vs 45-54y	Unadjusted	0.93	0.74	1.16	67.57	0.77	0.66	0.89	23.09	1.06	0.75	1.51	57.05	0.99	0.71	1.36	42.96
	**Basic adjustment**	**0.92**	**0.74**	**1.15**	**68.24**	**0.78**	**0.65**	**0.92**	**35.96**	**1.04**	**0.74**	**1.47**	**56.72**	**1.03**	**0.67**	**1.58**	**61.87**
	Full adjustment	1.03	0.81	1.29	59.88	1.03	0.59	1.78		1.25	0.62	2.52	0	1.36	0.93	2.00	0
55-64y vs 45-54y	Unadjusted	1.16	0.98	1.38	63.54	1.33	1.21	1.47	0	0.79	0.57	1.08	61.08	1.17	0.65	2.10	91.47
	**Basic adjustment**	**1.18**	**0.99**	**1.39**	**64.04**	**1.35**	**1.22**	**1.49**	**0**	**0.79**	**0.57**	**1.10**	**65.97**	**1.18**	**0.66**	**2.10**	**89.83**
	Full adjustment	1.17	1.05	1.29	0	1.55	0.62	3.91	49.89	0.85	0.48	1.52	0	no information			
65-74y vs 45-54y	Unadjusted	1.36	1.11	1.67	75.24	1.61	1.46	1.78	0	0.73	0.48	1.11	79.59	1.93	1.67	2.23	0
	**Basic adjustment**	**1.39**	**1.13**	**1.72**	**77.16**	**1.65**	**1.49**	**1.82**	**0**	**0.75**	**0.49**	**1.16**	**80.63**	**1.95**	**1.68**	**2.26**	**0**
	Full adjustment	1.33	1.19	1.49	0	1.98	0.48	8.10	73.94	1.15	0.65	2.04	0	1.57	1.07	2.31	0
75y+ vs 45-54y	Unadjusted	1.45	0.92	2.29	90.02	1.83	1.59	2.12	0	0.66	0.46	0.94	47.94	2.05	1.59	2.64	28.83
	**Basic adjustment**	**1.50**	**0.93**	**2.39**	**91.23**	**1.89**	**1.65**	**2.17**	**0**	**0.69**	**0.47**	**1.01**	**53.79**	**2.07**	**1.66**	**2.59**	**17.97**
	Full adjustment	1.16	0.86	1.58	62.75	1.07	0.44	2.61		0.78	0.39	1.57	1.68	1.75	1.17	2.62	
**Ethnicity**																	

For peer review only

For peer review only - http://bmjopen.bmj.com/site/about/guidelines.xhtml

Non-White vs White*	Unadjusted	1.02	0.89	1.18	10.29	0.95	0.72	1.25	56.69	1.36	0.79	2.33	79.57	0.89	0.71	1.12	0
	**Basic adjustment**	**1.19**	**1.05**	**1.35**	**0**	**1.10**	**0.86**	**1.39**	**42.54**	**1.32**	**0.82**	**2.12**	**71.12**	**1.14**	**0.91**	**1.44**	**0**
	Full adjustment	1.09	0.96	1.25	0	1.15	0.83	1.61	0	1.97	1.08	3.62	0	1.68	0.36	7.76	62.98
Black vs White	Unadjusted	1.16	0.87	1.55	0	0.95	0.54	1.64	33.95	0.63	0.13	3.06	81.71	0.87	0.58	1.29	0
	**Basic adjustment**	**1.38**	**1.03**	**1.84**	**0**	**1.01**	**0.54**	**1.92**	**49.83**	**0.64**	**0.14**	**2.87**	**80**	**1.03**	**0.68**	**1.55**	**0**
	Full adjustment	1.15	0.86	1.53	4.01	0.81	0.23	2.83	0	0.37	0.04	3.11		0.87	0.41	1.82	
East Asian vs White	Unadjusted	0.97	0.56	1.68	0	0.61	0.28	1.30	12.51	2.34	0.39	14.15	88.19	1.23	0.48	3.15	0
	**Basic adjustment**	**1.13**	**0.67**	**1.90**	**0**	**0.80**	**0.39**	**1.64**	**17.46**	**1.95**	**0.53**	**7.24**	**79.39**	**1.61**	**0.61**	**4.22**	**0**
	Full adjustment	0.96	0.60	1.51	0	1.19	0.26	5.51		no information				no information			
Mixed vs White	Unadjusted	1.05	0.79	1.38	0	1.02	0.55	1.89	64.07	1.51	0.99	2.30	0	1.05	0.66	1.67	0
	**Basic adjustment**	**1.24**	**0.86**	**1.78**	**27.61**	**1.22**	**0.69**	**2.15**	**56.82**	**1.34**	**0.82**	**2.18**	**16.29**	**1.34**	**0.76**	**2.36**	**20.19**
	Full adjustment	1.25	0.88	1.77	15.86	1.61	0.80	3.22	51.21	0.93	0.10	8.48		0.85	0.32	2.21	
South Asian vs White	Unadjusted	0.85	0.61	1.18	64.69	0.92	0.59	1.42	67.42	0.99	0.36	2.72	89.3	0.68	0.45	1.04	18
	**Basic adjustment**	**1.05**	**0.84**	**1.32**	**28.25**	**1.11**	**0.88**	**1.39**	**12.68**	**0.98**	**0.38**	**2.54**	**87.94**	**0.89**	**0.64**	**1.24**	**0**
	Full adjustment	0.93	0.67	1.30	57.84	1.03	0.29	3.63		2.81	1.19	6.63	11.86	1.11	0.62	1.99	
Other Ethnicity vs White	Unadjusted	0.79	0.46	1.34	28.89	0.91	0.58	1.45	0	1.23	0.56	2.67	25.72	0.84	0.20	3.48	44.62
	**Basic adjustment**	**0.90**	**0.49**	**1.63**	**44.27**	**1.07**	**0.68**	**1.68**	**0**	**1.45**	**0.81**	**2.60**	**0**	**0.95**	**0.23**	**4.03**	**52.56**
	Full adjustment	0.82	0.45	1.50	35.18	1.97	1.08	3.62	0.00	3.74	0.39	35.91		no information			
**Education**																	
A-level/equivalent vs Higher education/Degree	Unadjusted	1.04	0.91	1.17	58.16	1.03	0.91	1.17	42.88	0.94	0.77	1.15	21.54	0.84	0.55	1.29	88.56
	**Basic adjustment**	**1.08**	**0.97**	**1.20**	**38.7**	**1.07**	**0.99**	**1.16**	**0**	**1.02**	**0.82**	**1.28**	**27.13**	**0.87**	**0.58**	**1.30**	**85.92**
	Full adjustment	1.01	0.92	1.11	0	0.97	0.76	1.25	0	1.61	0.63	4.12	59.22	1.03	0.82	1.29	0
GCSE/equivalent vs Higher education/Degree	Unadjusted	0.99	0.87	1.14	62.23	1.03	0.95	1.12	0	0.98	0.75	1.27	53.81	1.03	0.91	1.16	0
	**Basic adjustment**	**1.00**	**0.87**	**1.14**	**59.18**	**1.01**	**0.91**	**1.12**	**15.22**	**1.04**	**0.77**	**1.39**	**62.34**	**1.05**	**0.93**	**1.18**	**0**
	Full adjustment	0.91	0.81	1.02	48.6	0.86	0.66	1.12	0	1.01	0.63	1.61	1.57	0.82	0.64	1.05	0
<GCSE/equivalent vs Higher education/Degree	Unadjusted	1.06	0.88	1.28	76.55	1.09	0.92	1.29	52.88	1.07	0.74	1.54	69.78	1.45	1.28	1.64	0
	**Basic adjustment**	**1.05**	**0.91**	**1.21**	**53.17**	**1.01**	**0.86**	**1.18**	**42.42**	**1.17**	**0.82**	**1.67**	**63.42**	**1.26**	**1.11**	**1.44**	**0**
	Full adjustment	0.87	0.75	1.00	34.17	0.90	0.54	1.50	58.54	1.17	0.39	3.49	60.69	1.53	0.34	6.85	71.25
**Occupational class**																	

For peer review only - http://bmjopen.bmj.com/site/about/guidelines.xhtml

Intermediate vs Managerial/Admin/Professional	Unadjusted	1.08	1.00	1.16	0	1.01	0.91	1.14	22.79	1.09	0.83	1.42	41.19	1.19	1.05	1.34	0
	**Basic adjustment**	**1.07**	**0.99**	**1.15**	**0**	**1.01**	**0.93**	**1.10**	**0**	**1.10**	**0.88**	**1.38**	**26.19**	**1.16**	**1.03**	**1.31**	**0**
	Full adjustment	1.00	0.92	1.08	0	1.01	0.78	1.30	0	0.73	0.34	1.61	0	0.78	0.42	1.47	48.08
Manual/Routine vs Managerial/Admin/Professional	Unadjusted	1.13	1.03	1.23	12.12	1.04	0.90	1.20	36.16	1.38	1.16	1.64	0	1.11	0.91	1.35	25.25
	**Basic adjustment**	**1.17**	**1.08**	**1.27**	**0**	**1.07**	**0.93**	**1.23**	**28.46**	**1.36**	**1.11**	**1.67**	**8.93**	**1.17**	**1.03**	**1.33**	**0**
	Full adjustment	1.02	0.93	1.12	0	1.10	0.84	1.44	6.05	0.51	0.18	1.43	23.27	0.92	0.56	1.50	20.63
Other social class vs Managerial/Admin/Professional	Unadjusted	1.47	1.02	2.13	89.12	1.41	1.08	1.84	66.23	2.16	1.30	3.57	76.1	1.71	0.94	3.10	87.16
	**Basic adjustment**	**1.51**	**1.12**	**2.04**	**79.69**	**1.46**	**1.16**	**1.84**	**47.81**	**2.45**	**1.72**	**3.50**	**45.5**	**1.81**	**1.17**	**2.80**	**73.85**
	Full adjustment	1.19	1.00	1.43	39.12	1.30	0.85	1.99	0.00	1.42	0.13	15.78	76.18	0.94	0.69	1.27	

Basic adjustment: sex, age, and ethnicity (where available)

Full adjustment: sex, age, and ethnicity (where available) education, occupational class, UK Nation (where appropriate), household composition, and pre-pandemic self-reported health.

Empty I^2% column indicates only one study included

*Binary variable including Black, East Asian, Mixed, South Asian, and other ethnicity in 'non-White'

For peer review only

Summary of stratified results

		Any healthcare disruption			
**Sex**		OR	Lower CI	Upper CI	I2%
Female vs. Male	Overall	1.27	1.15	1.40	53.11
	Not shielding	1.26	1.12	1.43	61.12
	Shielding	1.37	1.15	1.63	0
	16-24y	2.22	1.63	3.02	0
	25-34y	1.56	1.30	1.87	0
	35-44y	1.51	1.23	1.86	0
	45-54	1.72	1.35	2.18	36.61
	55-64	1.09	0.92	1.30	59.58
	75+	1.08	0.90	1.30	20
**Age**		OR	Lower CI	Upper CI	I2%
16-24y vs 45-54y	Overall	0.76	0.39	1.46	71.95
	Not shielding	0.79	0.40	1.56	70.32
	Shielding	0.64	0.23	1.78	
25-34y vs 45-54y	Overall	0.85	0.70	1.04	47.62
	Not shielding	0.86	0.70	1.06	43.4
	Shielding	1.09	0.61	1.95	0
35-44y vs 45-54y	Overall	0.92	0.74	1.15	68.24
	Not shielding	0.95	0.74	1.21	68.26
	Shielding	0.68	0.34	1.34	47.41
55-64y vs 45-54y	Overall	1.18	0.99	1.39	64.04
	Not shielding	1.21	1.02	1.43	53.82
	Shielding	1.24	0.87	1.77	0
65-74y vs 45-54y	Overall	1.39	1.13	1.72	77.16
	Not shielding	1.44	1.20	1.72	64.1
	Shielding	1.11	0.79	1.56	0
75y+ vs 45-54y	Overall	1.50	0.93	2.39	91.23
	Not shielding	1.61	1.17	2.22	79.38
	Shielding	0.83	0.51	1.37	32.84
**Ethnicity**		OR	Lower CI	Upper CI	I2%
Non-White vs White*	Overall	1.19	1.05	1.35	0
	Not shielding	1.06	0.86	1.31	41.46
	Shielding	1.62	1.08	2.43	0
	16-24y	1.30	0.89	1.89	0
	25-34y	0.92	0.65	1.29	36.48
	35-44y	1.31	1.01	1.71	0
	45-54	1.61	1.16	2.22	0
	55-64	1.13	0.85	1.50	0

For peer review only

	75+	1.28	0.67	2.45	0
Black vs White	Overall	1.38	1.03	1.84	0
	Not shielding	0.80	0.43	1.49	58.06
	Shielding	1.60	0.67	3.83	0
	16-24y	1.15	0.51	2.59	0
	25-34y	0.82	0.40	1.68	0
	35-44y	1.91	0.81	4.48	0
	45-54	1.99	0.93	4.25	15.25
	55-64	1.69	1.00	2.84	0
	75+	1.23	0.42	3.56	0
East Asian vs White	Overall	1.13	0.67	1.90	0
	Not shielding	0.95	0.54	1.68	0
	Shielding		no information		
	16-24y	0.01	0.00	0.05	
	25-34y	0.62	0.20	1.92	0
	35-44y	1.63	0.80	3.32	0
	45-54	1.75	0.54	5.64	0
	55-64	0.96	0.43	2.15	0
	75+		no information		
Mixed vs White	Overall	1.24	0.86	1.78	27.61
	Not shielding	1.18	0.85	1.62	0
	Shielding	1.85	0.71	4.77	0
	16-24y	2.50	1.25	5.02	0
	25-34y	1.26	0.79	2.02	0
	35-44y	1.15	0.23	5.69	73.12
	45-54	0.92	0.46	1.87	0
	55-64	1.06	0.53	2.11	0
	75+	1.47	0.34	6.42	22.46
South Asian vs White	Overall	1.05	0.84	1.32	28.25
	Not shielding	0.98	0.75	1.28	35.03
	Shielding	1.44	0.87	2.38	0
	16-24y	0.98	0.62	1.53	13.95
	25-34y	0.80	0.38	1.71	74.73
	35-44y	1.11	0.80	1.55	10.1
	45-54	1.67	0.43	6.48	82
	55-64	0.82	0.44	1.56	14.81
	75+	1.11	0.40	3.12	0
Other Ethnicity vs White	Overall	0.90	0.49	1.63	44.27
	Not shielding	0.85	0.45	1.62	43.11
	Shielding	0.75	0.11	4.96	10.15
	16-24y	0.18	0.00	15.35	88.56
	25-34y	0.80	0.31	2.08	49.28

For peer review only - http://bmjopen.bmj.com/site/about/guidelines.xhtml

		OR	Lower CI	Upper CI	I2%
	35-44y	1.41	0.58	3.40	0
	45-54	1.74	0.56	5.45	29.75
	55-64	0.77	0.27	2.22	0
	75+	4.18	0.35	50.04	
**Education**		OR	Lower CI	Upper CI	I2%
A-level/equivalent vs Higher education/Degree	Overall	1.08	0.97	1.20	38.7
	Not shielding	1.09	0.96	1.23	39.28
	Shielding	0.95	0.74	1.22	0
	16-24y	1.33	0.93	1.90	0
	25-34y	0.99	0.69	1.42	62.16
	35-44y	1.62	1.28	2.05	0
	45-54	1.13	0.96	1.34	0
	55-64	1.01	0.89	1.14	0
	75+	0.96	0.65	1.40	57.49
GCSE/equivalent vs Higher education/Degree	Overall	1.00	0.87	1.14	59.18
	Not shielding	0.99	0.84	1.17	64.95
	Shielding	0.80	0.62	1.04	0
	16-24y	0.94	0.49	1.81	64.06
	25-34y	1.24	0.80	1.94	69.1
	35-44y	1.26	0.97	1.63	0
	45-54	1.16	0.83	1.62	62.52
	55-64	1.03	0.91	1.17	0
	75+	0.92	0.65	1.30	35.17
<GCSE/equivalent vs Higher education/Degree	Overall	1.05	0.91	1.21	53.17
	Not shielding	1.02	0.88	1.19	46.14
	Shielding	0.87	0.68	1.11	0
	16-24y	0.77	0.47	1.28	11.51
	25-34y	0.99	0.67	1.45	42.2
	35-44y	1.03	0.74	1.43	0
	45-54	1.48	1.08	2.04	34.96
	55-64	1.20	1.03	1.41	0
	75+	0.96	0.78	1.20	0
**Occupational class**		OR	Lower CI	Upper CI	I2%
Intermediate vs Managerial/Admin/Professional	Overall	1.07	0.99	1.15	0
	Not shielding	1.07	0.98	1.16	0
	Shielding	0.87	0.65	1.16	7.88
	16-24y	0.92	0.60	1.41	0
	25-34y	1.04	0.84	1.29	10.79
	35-44y	1.28	0.92	1.78	46.81
	45-54	1.12	0.94	1.33	0
	55-64	1.01	0.86	1.19	22.02

For peer review only - http://bmjopen.bmj.com/site/about/guidelines.xhtml

	75+	1.00	0.76	1.33	0
Manual/Routine vs Managerial/Admin/Professional	Overall	1.17	1.08	1.27	0
	Not shielding	1.18	1.07	1.29	0
	Shielding	0.93	0.71	1.21	0
	16-24y	1.15	0.77	1.71	0
	25-34y	1.11	0.80	1.55	50.55
	35-44y	1.24	0.95	1.63	0
	45-54	1.08	0.90	1.30	0
	55-64	1.16	1.00	1.35	0
	75+	1.27	0.96	1.67	0
Other social class vs Managerial/Admin/Professional	Overall	1.51	1.12	2.04	79.69
	Not shielding	1.48	1.04	2.09	83.37
	Shielding	0.89	0.39	2.07	78.87
	16-24y	1.02	0.46	2.26	58.35
	25-34y	1.85	1.29	2.64	0
	35-44y	1.44	0.55	3.80	68.27
	45-54	2.05	0.98	4.29	85.15
	55-64	1.65	1.21	2.27	60.63
	75+	1.02	0.62	1.69	0

Adjusted for sex, age, and ethnicity (where available)

Empty I^2% column indicates only one study included

*Binary variable including Black, East Asian, Mixed, South Asian, and other ethnicity in 'non-White'

For peer review only

Any healthcare disruption

Sex

Unadjusted

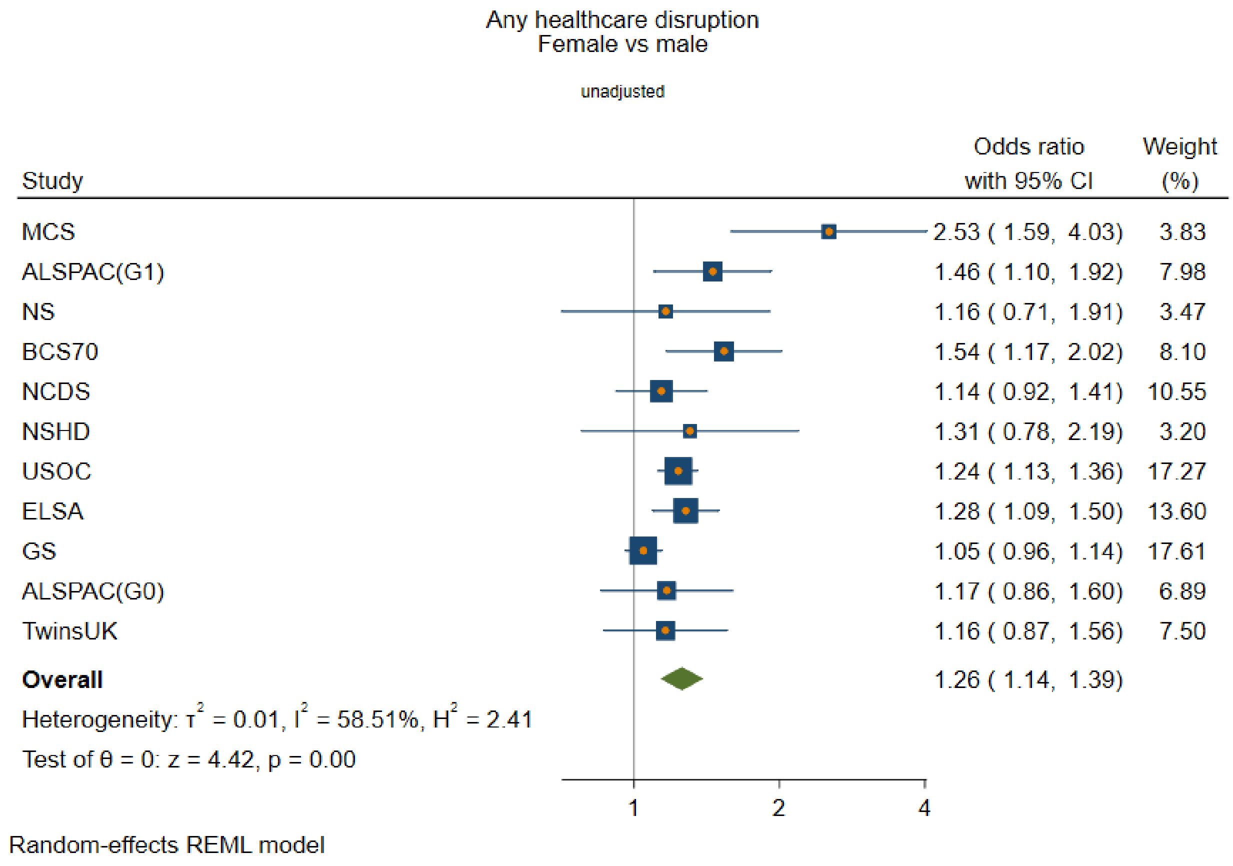

For peer review only - http://bmjopen.bmj.com/site/about/guidelines.xhtml

Basic adjustment

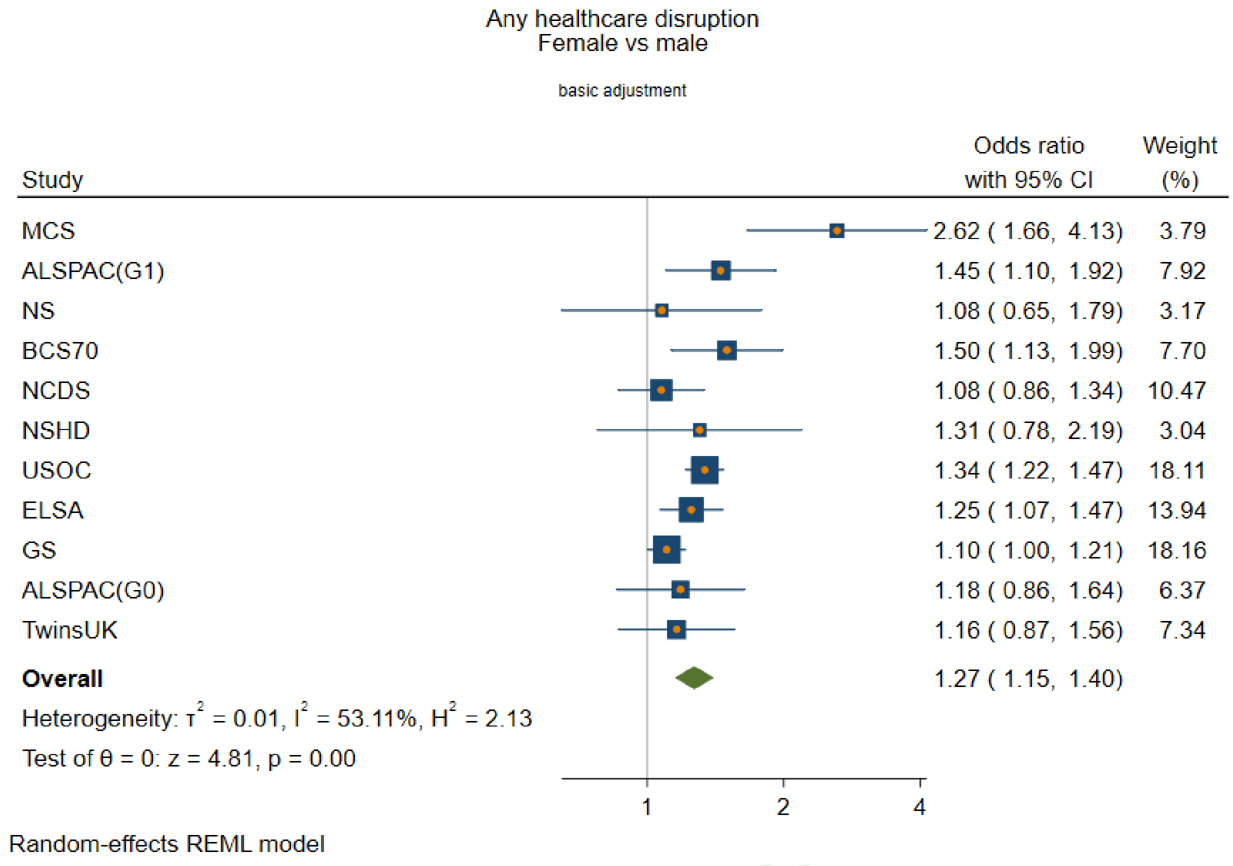

For peer review only - http://bmjopen.bmj.com/site/about/guidelines.xhtml

Full adjustment

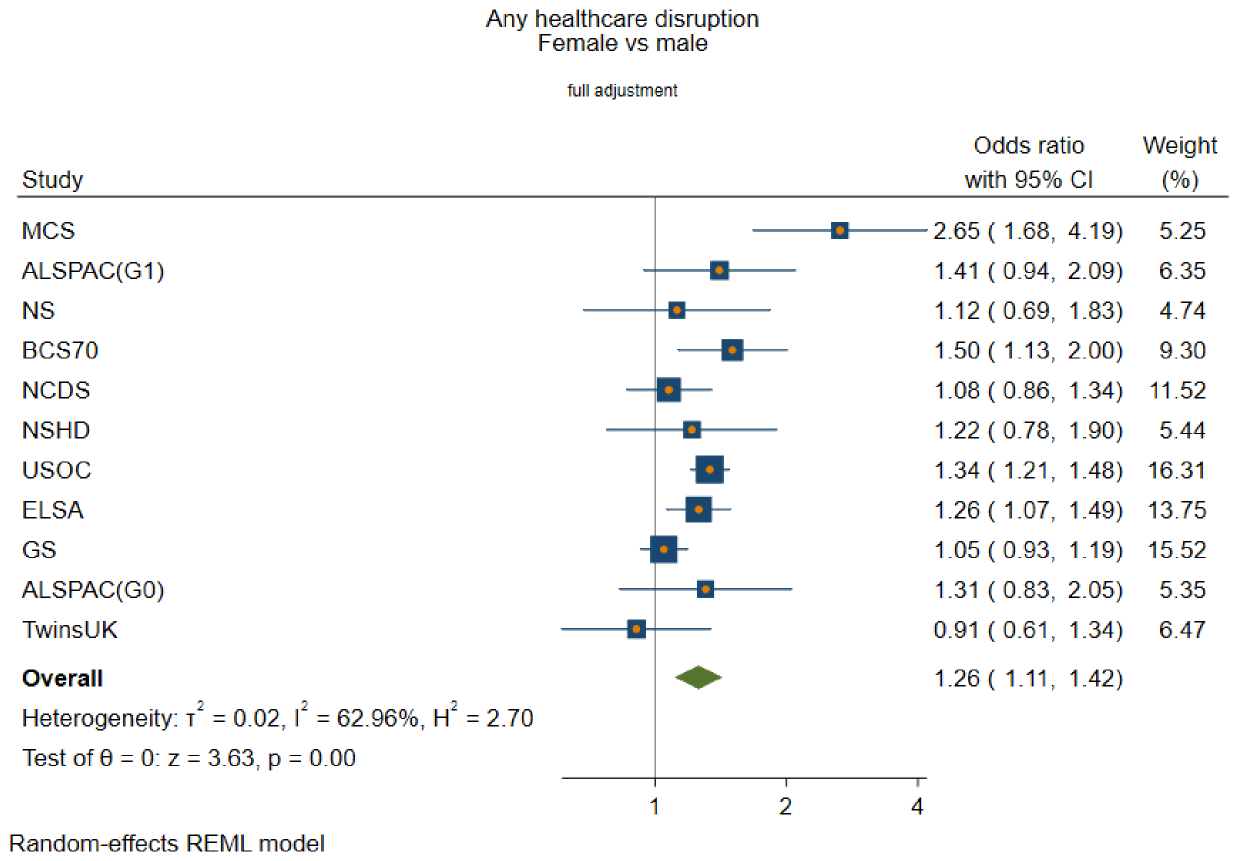

For peer review only - http://bmjopen.bmj.com/site/about/guidelines.xhtml

Occupational class

Unadjusted

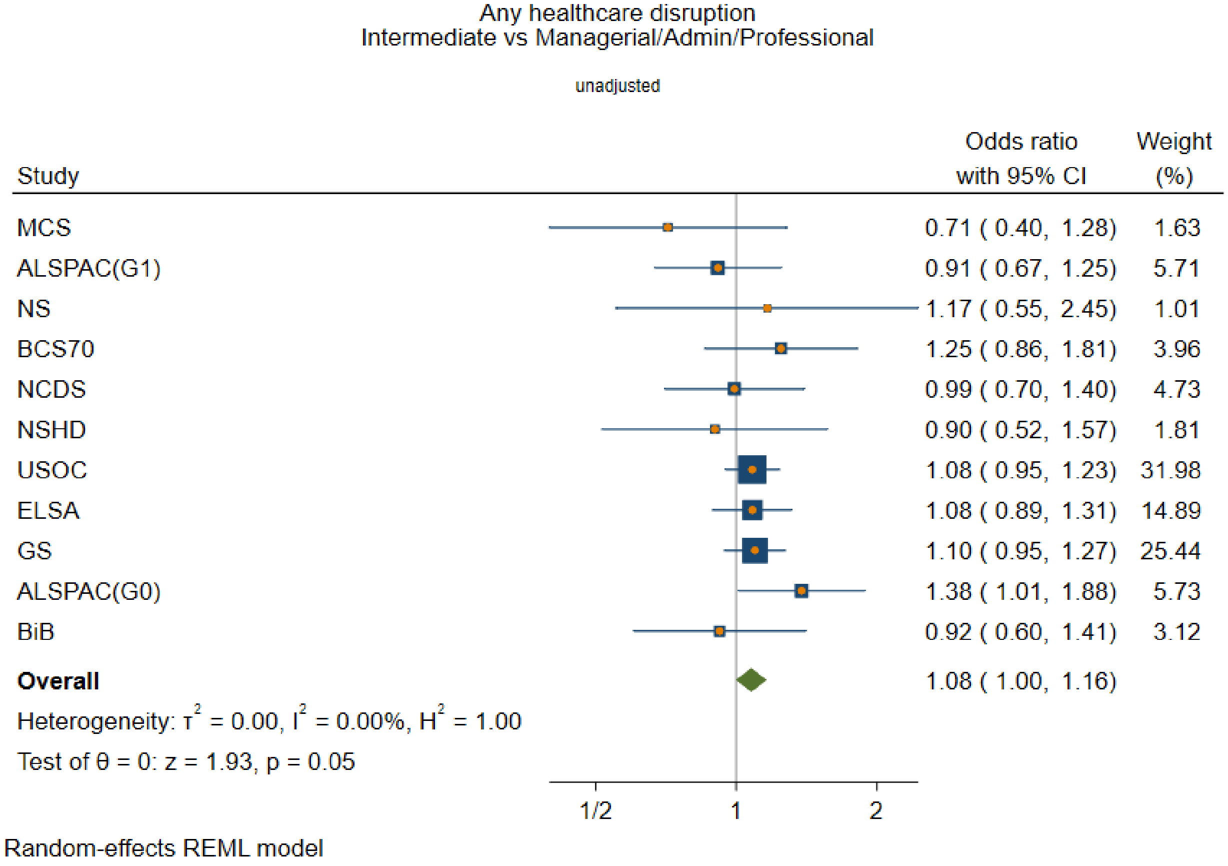

only

For peer review only - http://bmjopen.bmj.com/site/about/guidelines.xhtml

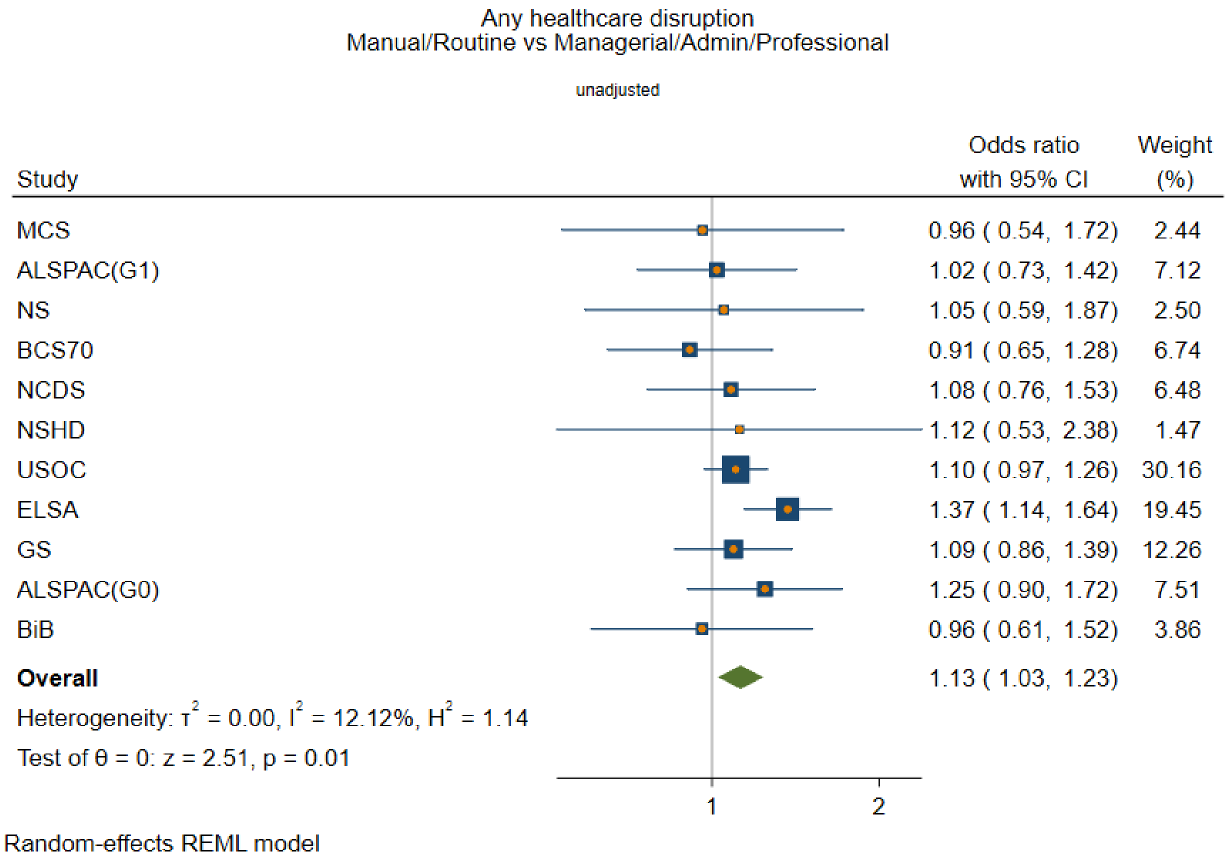
Any healthcare disruption
Manual/Routine vs Managerial/Admin/Professional
unadjusted
Study
Odds ratio with 95% CI
Weight (%)
MCS 0.96 (0.54, 1.72) 2.44
ALSPAC(G1) 1.02 (0.73, 1.42) 7.12
NS 1.05 (0.59, 1.87) 2.50
BCS70 0.91 (0.65, 1.28) 6.74
NCDS 1.08 (0.76, 1.53) 6.48
NSHD 1.12 (0.53, 2.38) 1.47
USOC 1.10 (0.97, 1.26) 30.16
ELSA 1.37 (1.14, 1.64) 19.45
GS 1.09 (0.86, 1.39) 12.26
ALSPAC(G0) 1.25 (0.90, 1.72) 7.51
BiB 0.96 (0.61, 1.52) 3.86
Overall 1.13 (1.03, 1.23)
Heterogeneity: τ^2 = 0.00, I^2 = 12.12%, H^2 = 1.14
Test of θ = 0: z = 2.51, p = 0.01
1
2
Random-effects REML model

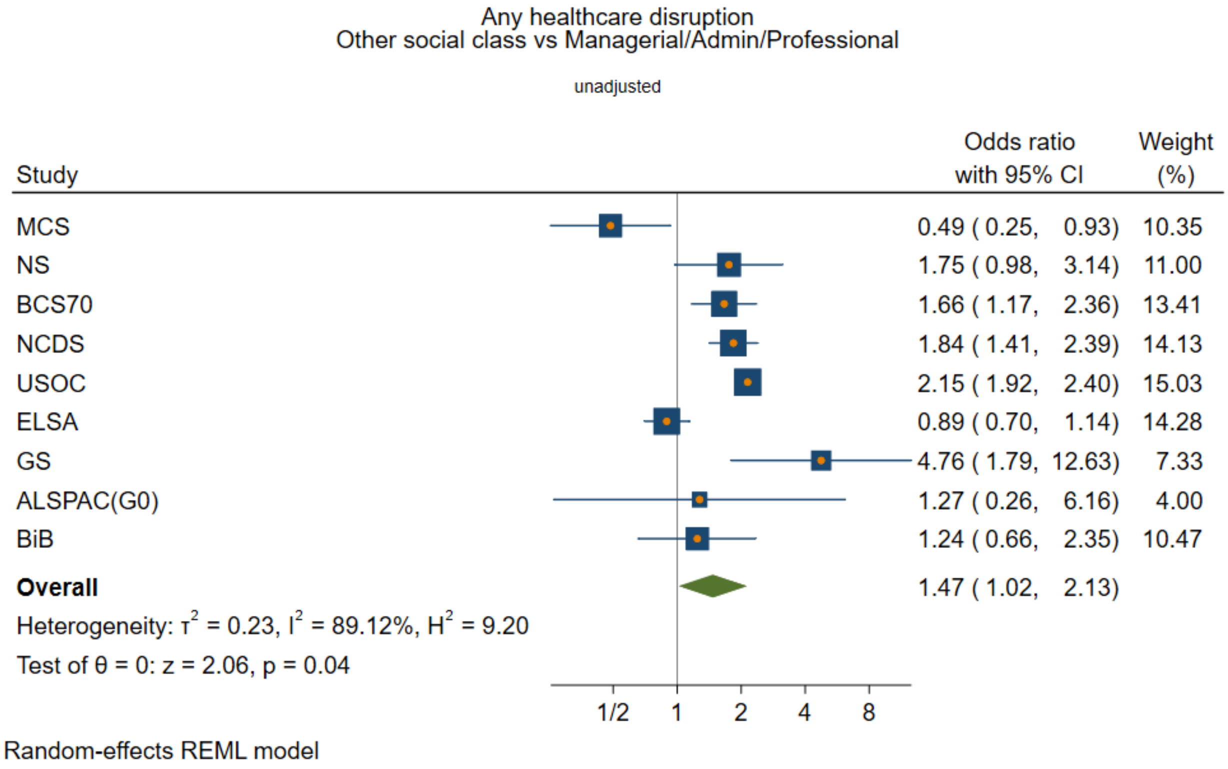
Any healthcare disruption
Other social class vs Managerial/Admin/Professional
unadjusted
Study
Odds ratio with 95% CI
Weight (%)
MCS 0.49 (0.25, 0.93) 10.35
NS 1.75 (0.98, 3.14) 11.00
BCS70 1.66 (1.17, 2.36) 13.41
NCDS 1.84 (1.41, 2.39) 14.13
USOC 2.15 (1.92, 2.40) 15.03
ELSA 0.89 (0.70, 1.14) 14.28
GS 4.76 (1.79, 12.63) 7.33
ALSPAC(G0) 1.27 (0.26, 6.16) 4.00
BiB 1.24 (0.66, 2.35) 10.47
Overall 1.47 (1.02, 2.13)
Heterogeneity: τ^2 = 0.23, I^2 = 89.12%, H^2 = 9.20
Test of θ = 0: z = 2.06, p = 0.04
1/2
1
2
4
8
Random-effects REML model

Basic adjustment

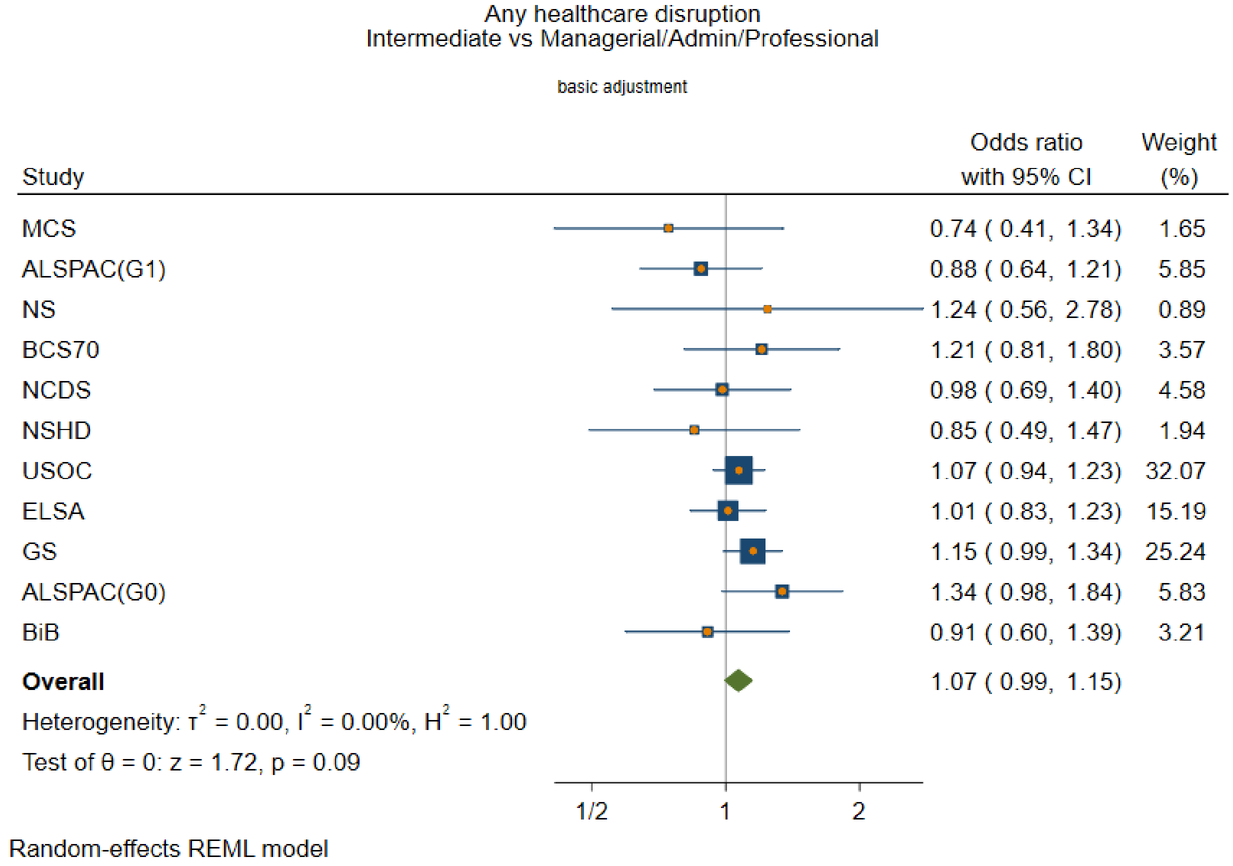

For peer review only - http://bmjopen.bmj.com/site/about/guidelines.xhtml

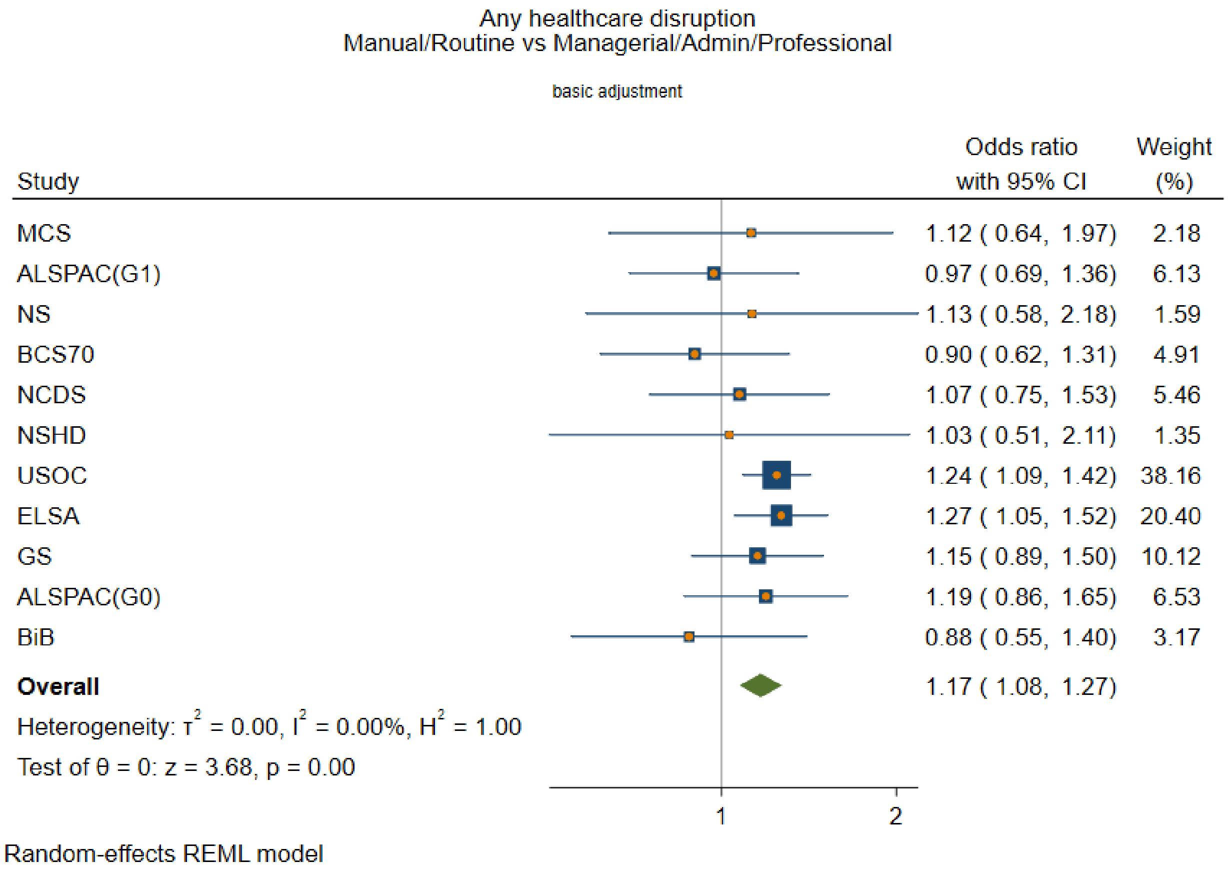

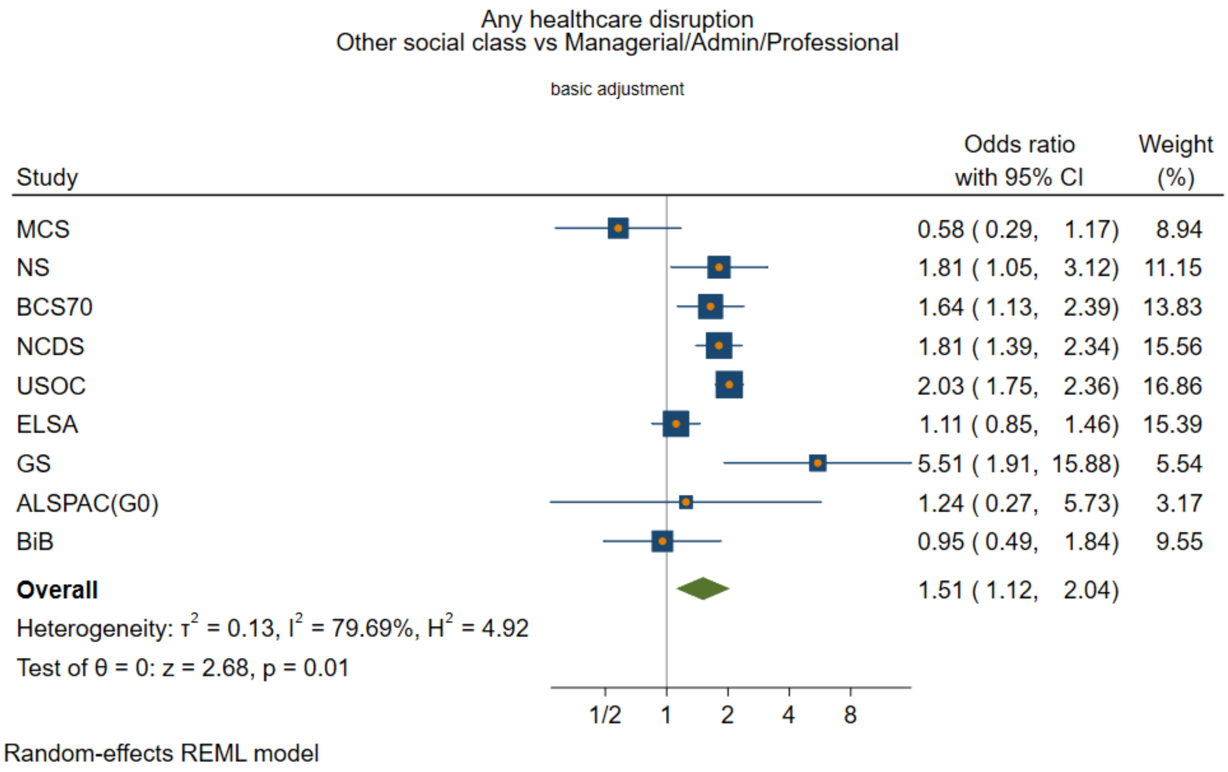

For peer review only - http://bmjopen.bmj.com/site/about/guidelines.xhtml

Full adjustment

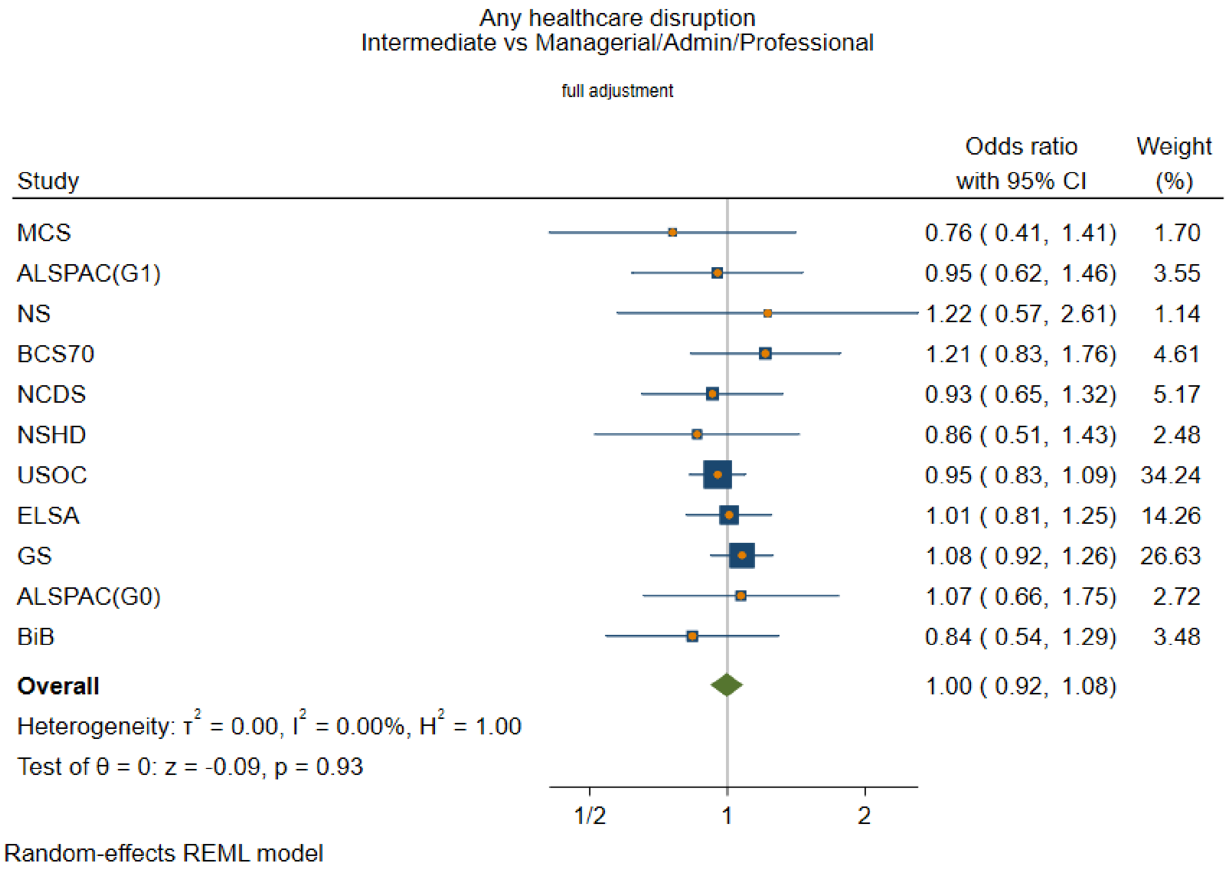

w only

For peer review only - http://bmjopen.bmj.com/site/about/guidelines.xhtml

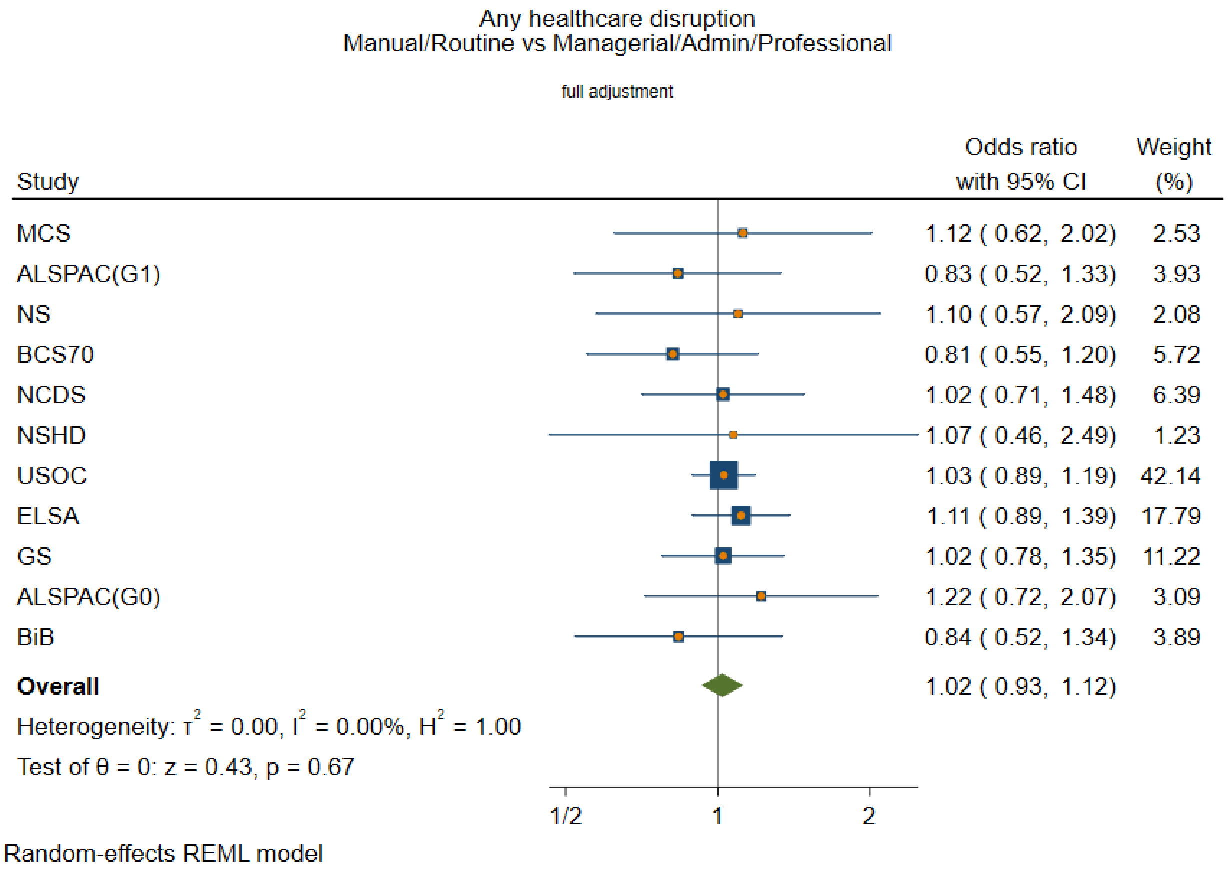
Any healthcare disruption
Manual/Routine vs Managerial/Admin/Professional
full adjustment
Study
Odds ratio with 95% CI
Weight (%)
MCS 1.12 (0.62, 2.02) 2.53
ALSPAC(G1) 0.83 (0.52, 1.33) 3.93
NS 1.10 (0.57, 2.09) 2.08
BCS70 0.81 (0.55, 1.20) 5.72
NCDS 1.02 (0.71, 1.48) 6.39
NSHD 1.07 (0.46, 2.49) 1.23
USOC 1.03 (0.89, 1.19) 42.14
ELSA 1.11 (0.89, 1.39) 17.79
GS 1.02 (0.78, 1.35) 11.22
ALSPAC(G0) 1.22 (0.72, 2.07) 3.09
BiB 0.84 (0.52, 1.34) 3.89
Overall 1.02 (0.93, 1.12)
Heterogeneity: τ² = 0.00, I² = 0.00%, H² = 1.00
Test of θ = 0: z = 0.43, p = 0.67
1/2
1
2
Random-effects REML model

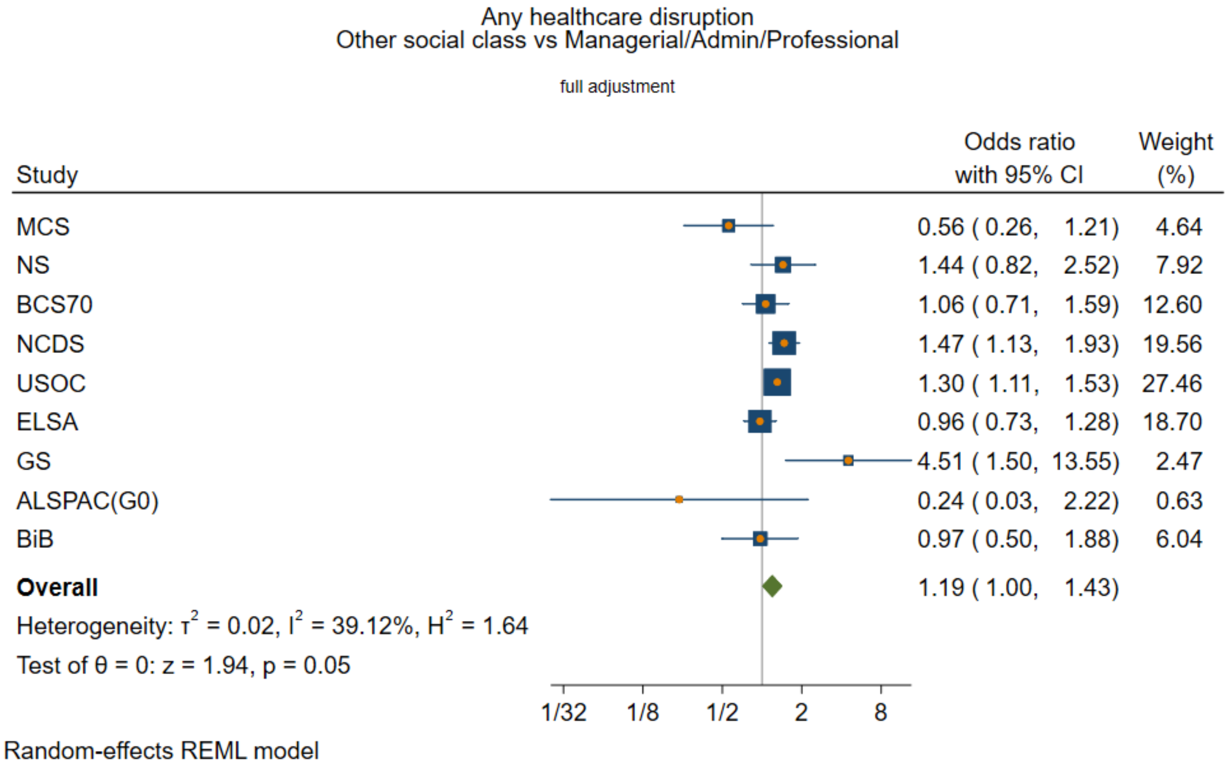
Any healthcare disruption
Other social class vs Managerial/Admin/Professional
full adjustment
Study
Odds ratio with 95% CI
Weight (%)
MCS 0.56 (0.26, 1.21) 4.64
NS 1.44 (0.82, 2.52) 7.92
BCS70 1.06 (0.71, 1.59) 12.60
NCDS 1.47 (1.13, 1.93) 19.56
USOC 1.30 (1.11, 1.53) 27.46
ELSA 0.96 (0.73, 1.28) 18.70
GS 4.51 (1.50, 13.55) 2.47
ALSPAC(G0) 0.24 (0.03, 2.22) 0.63
BiB 0.97 (0.50, 1.88) 6.04
Overall 1.19 (1.00, 1.43)
Heterogeneity: τ² = 0.02, I² = 39.12%, H² = 1.64
Test of θ = 0: z = 1.94, p = 0.05
1/32
1/8
1/2
2
8
Random-effects REML model

Education
Unadjusted

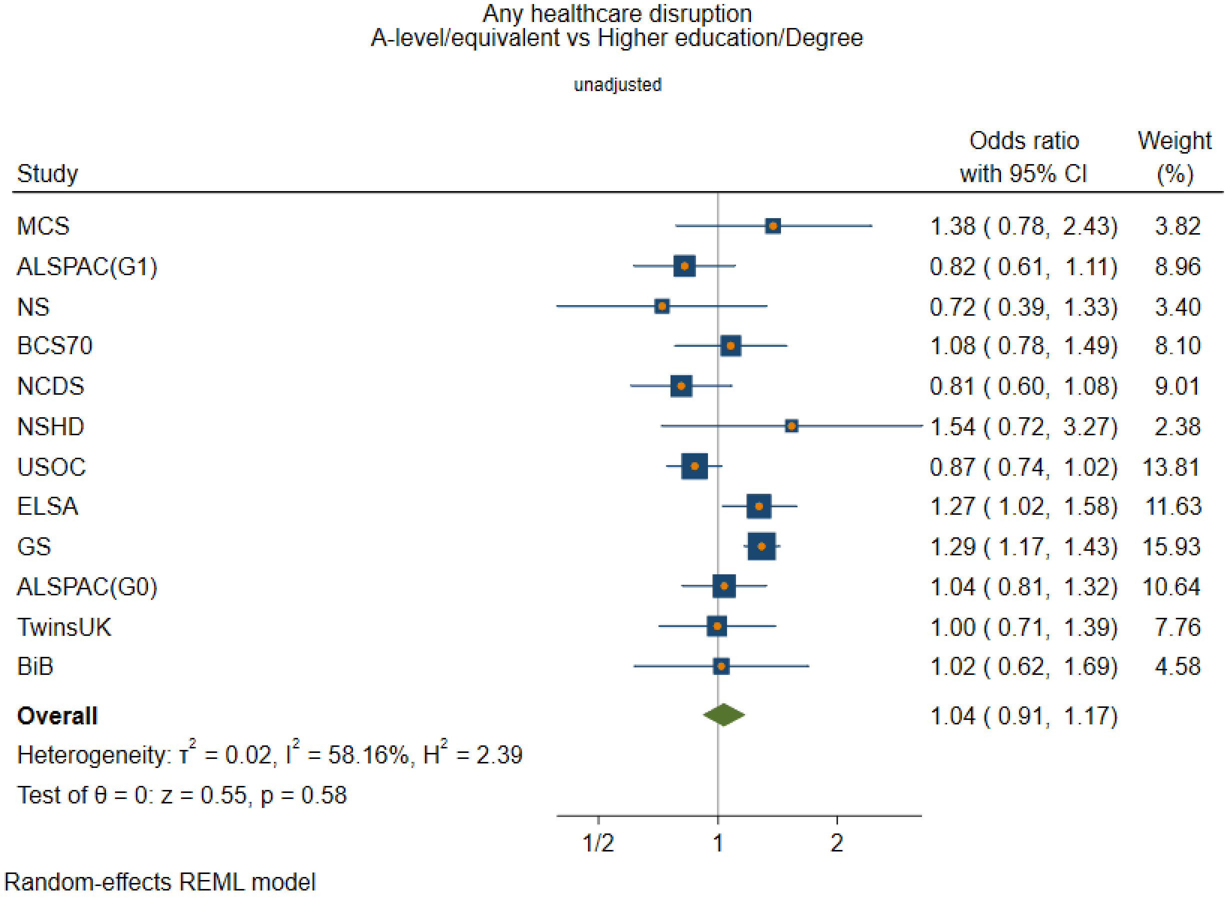

only

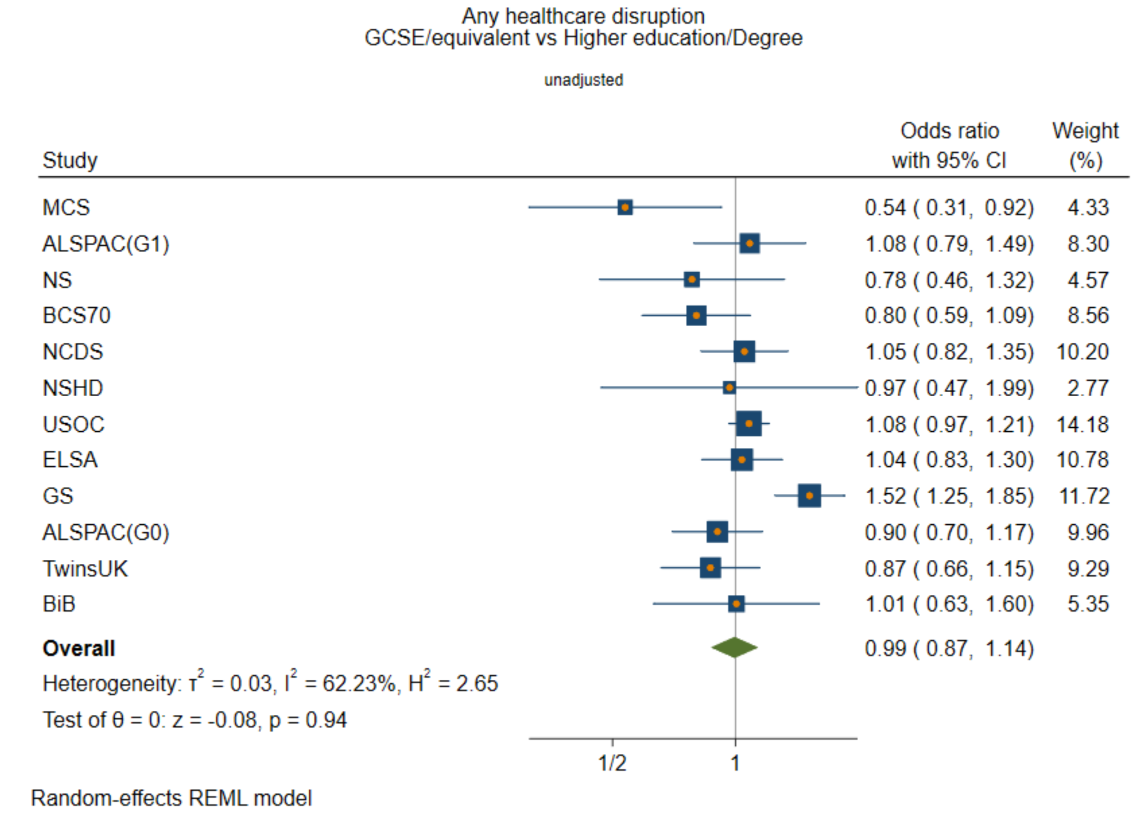
Any healthcare disruption
GCSE/equivalent vs Higher education/Degree
unadjusted
Study
Odds ratio with 95% CI
Weight (%)
MCS 0.54 (0.31, 0.92) 4.33
ALSPAC(G1) 1.08 (0.79, 1.49) 8.30
NS 0.78 (0.46, 1.32) 4.57
BCS70 0.80 (0.59, 1.09) 8.56
NCDS 1.05 (0.82, 1.35) 10.20
NSHD 0.97 (0.47, 1.99) 2.77
USOC 1.08 (0.97, 1.21) 14.18
ELSA 1.04 (0.83, 1.30) 10.78
GS 1.52 (1.25, 1.85) 11.72
ALSPAC(G0) 0.90 (0.70, 1.17) 9.96
TwinsUK 0.87 (0.66, 1.15) 9.29
BiB 1.01 (0.63, 1.60) 5.35
Overall 0.99 (0.87, 1.14)
Heterogeneity: $\tau^2 = 0.03$, $I^2 = 62.23\%$, $H^2 = 2.65$
Test of θ = 0: z = -0.08, p = 0.94
1/2
1
Random-effects REML model

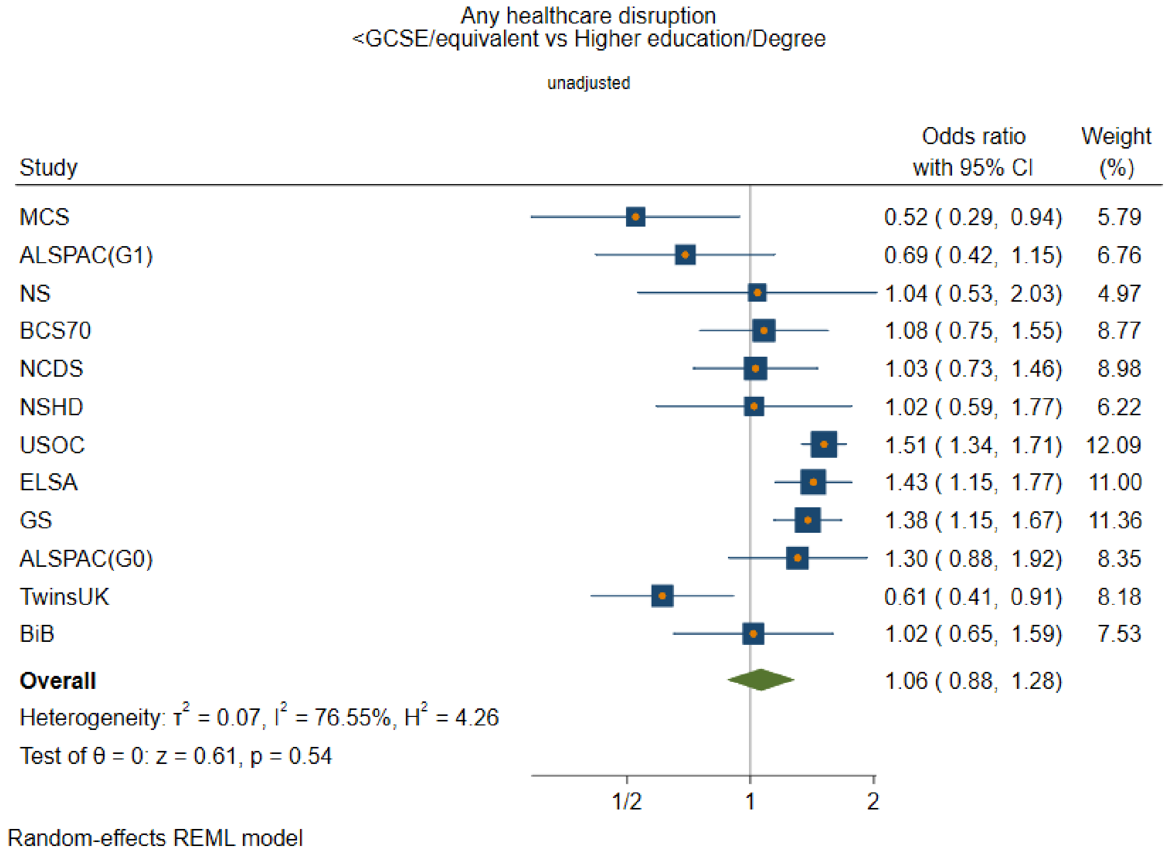
Any healthcare disruption
<GCSE/equivalent vs Higher education/Degree
unadjusted
Study
Odds ratio with 95% CI
Weight (%)
MCS 0.52 (0.29, 0.94) 5.79
ALSPAC(G1) 0.69 (0.42, 1.15) 6.76
NS 1.04 (0.53, 2.03) 4.97
BCS70 1.08 (0.75, 1.55) 8.77
NCDS 1.03 (0.73, 1.46) 8.98
NSHD 1.02 (0.59, 1.77) 6.22
USOC 1.51 (1.34, 1.71) 12.09
ELSA 1.43 (1.15, 1.77) 11.00
GS 1.38 (1.15, 1.67) 11.36
ALSPAC(G0) 1.30 (0.88, 1.92) 8.35
TwinsUK 0.61 (0.41, 0.91) 8.18
BiB 1.02 (0.65, 1.59) 7.53
Overall 1.06 (0.88, 1.28)
Heterogeneity: $\tau^2 = 0.07$, $I^2 = 76.55\%$, $H^2 = 4.26$
Test of θ = 0: z = 0.61, p = 0.54
1/2
1
2
Random-effects REML model

Basic adjustment

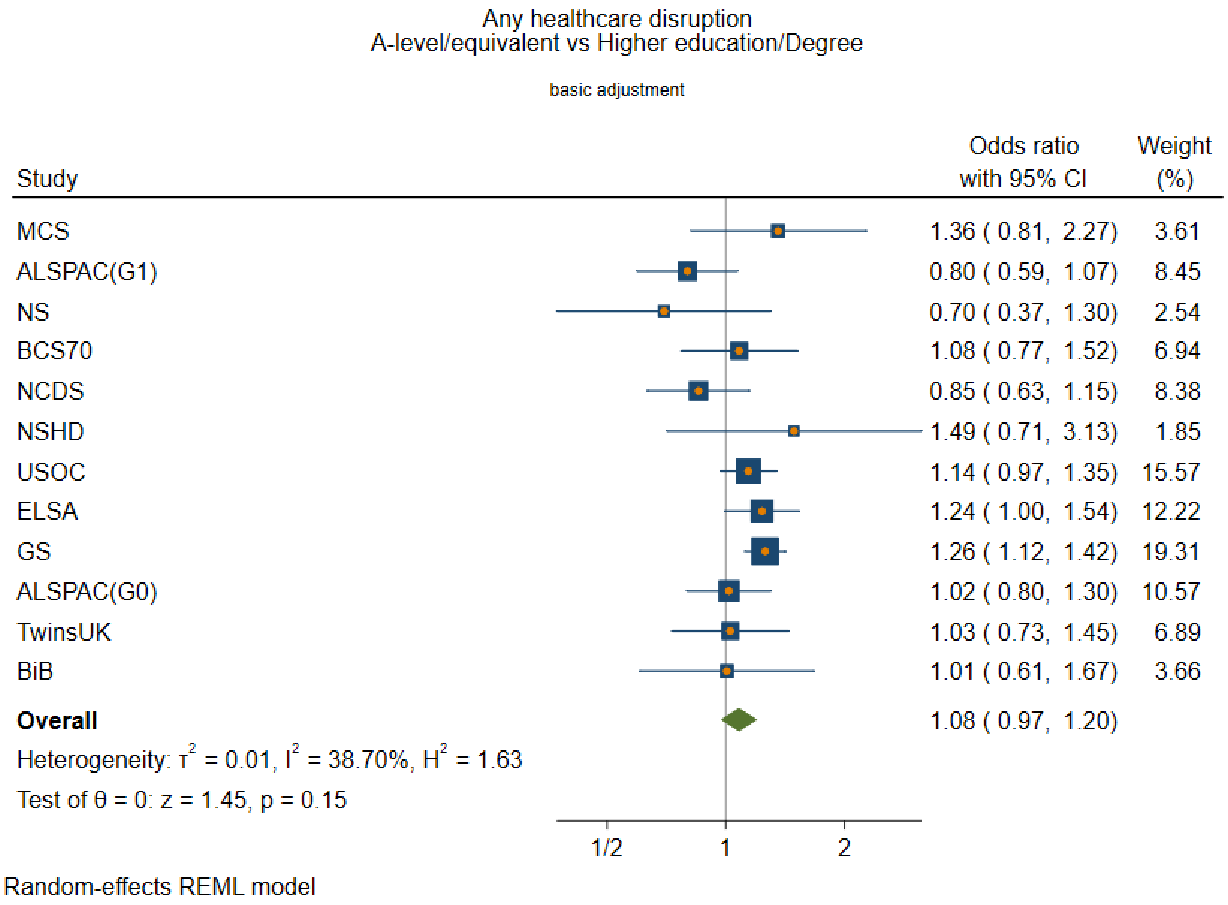

For peer review only - http://bmjopen.bmj.com/site/about/guidelines.xhtml

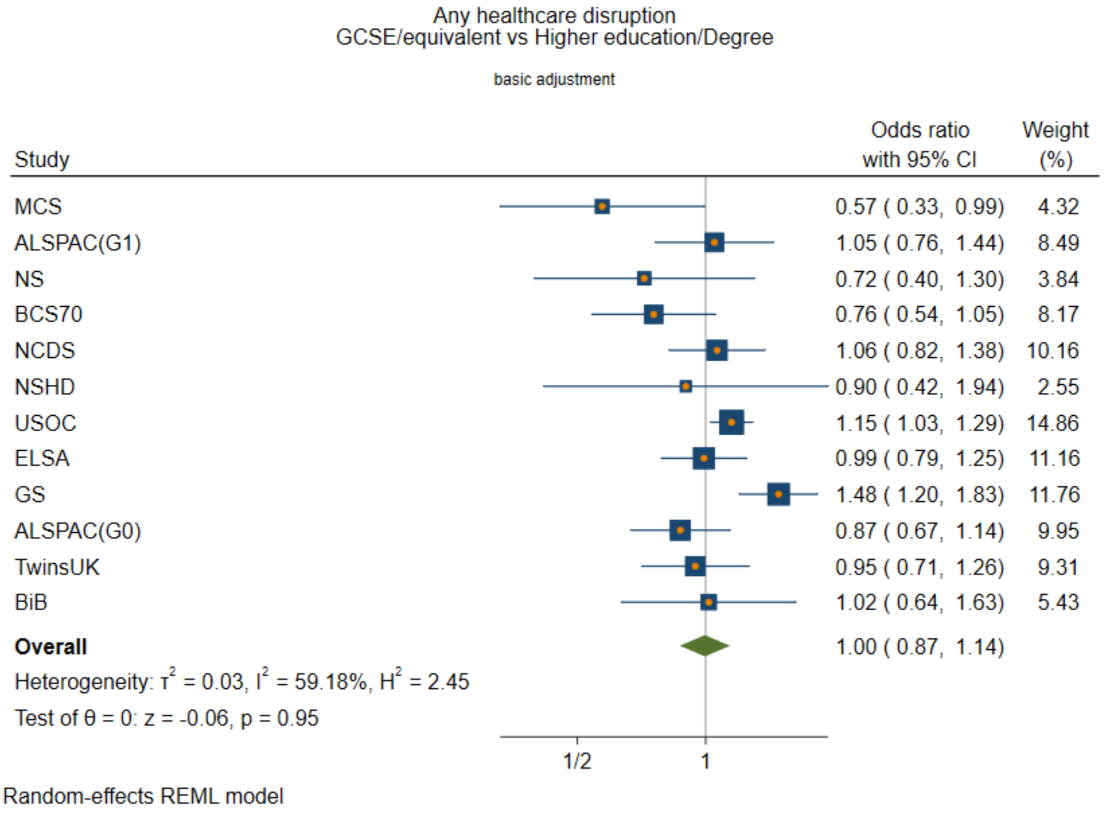

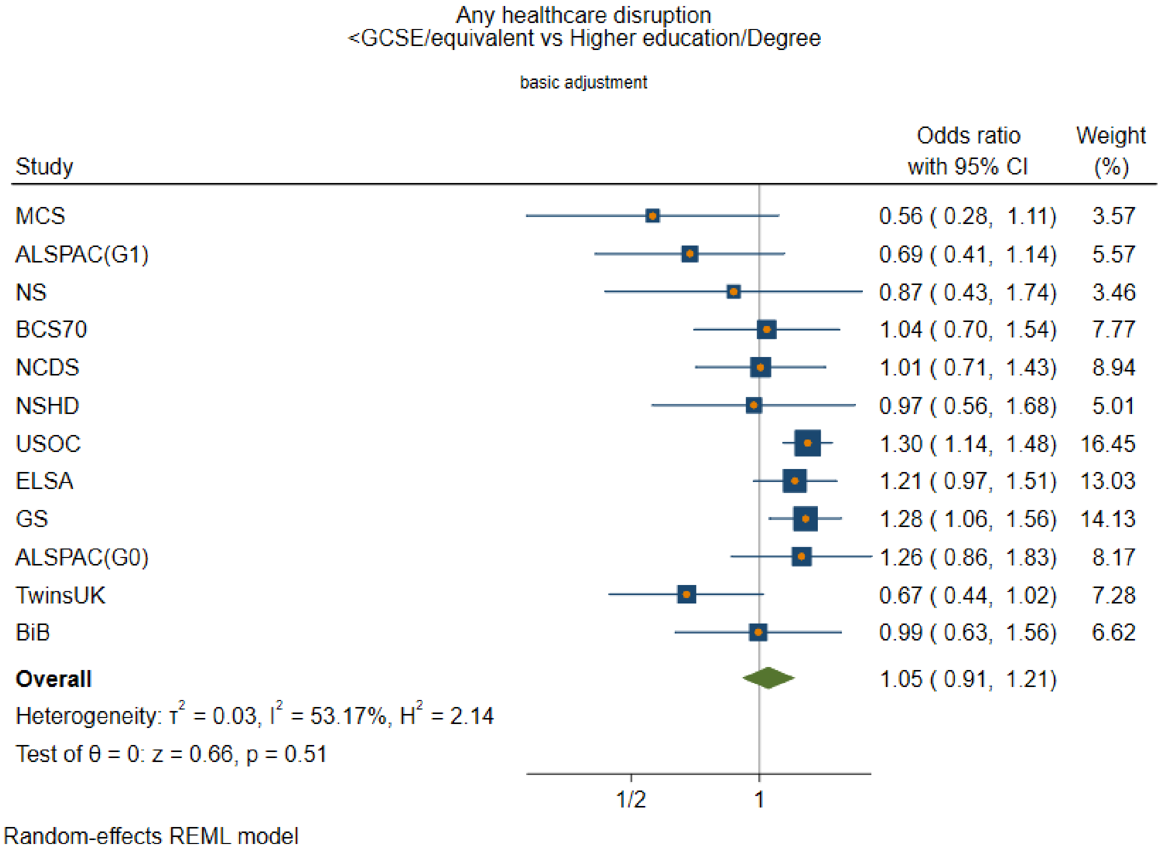

For peer review only - http://bmjopen.bmj.com/site/about/guidelines.xhtml

Full adjustment

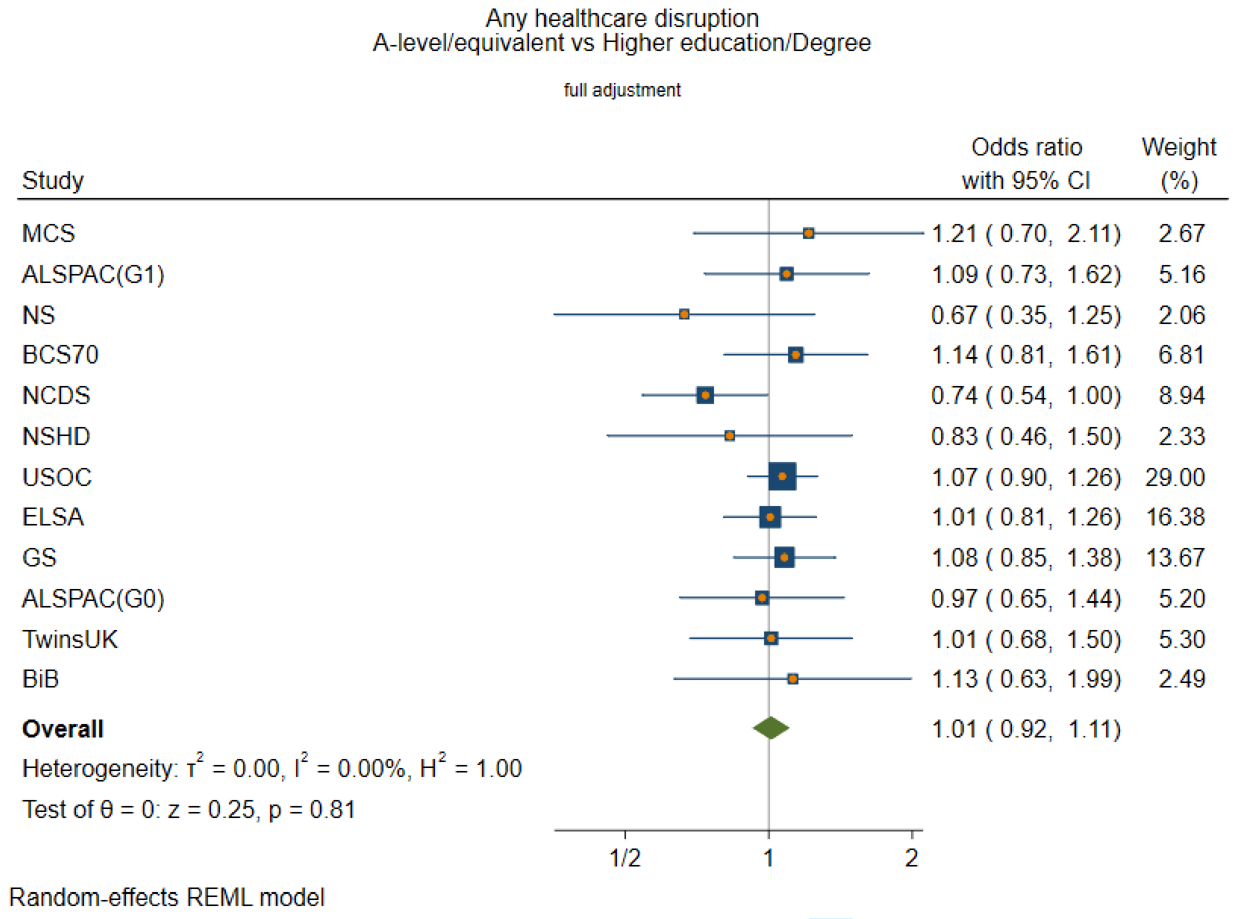

only

For peer review only - http://bmjopen.bmj.com/site/about/guidelines.xhtml

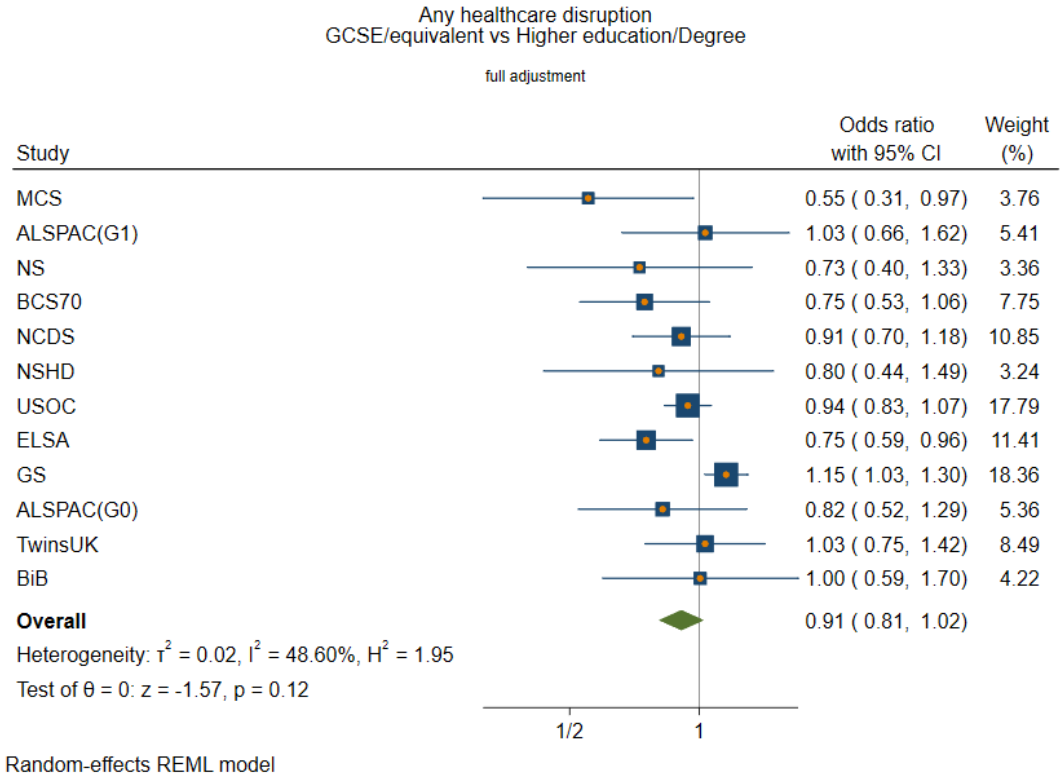
Any healthcare disruption
GCSE/equivalent vs Higher education/Degree
full adjustment
Study
Odds ratio with 95% CI
Weight (%)
MCS 0.55 (0.31, 0.97) 3.76
ALSPAC(G1) 1.03 (0.66, 1.62) 5.41
NS 0.73 (0.40, 1.33) 3.36
BCS70 0.75 (0.53, 1.06) 7.75
NCDS 0.91 (0.70, 1.18) 10.85
NSHD 0.80 (0.44, 1.49) 3.24
USOC 0.94 (0.83, 1.07) 17.79
ELSA 0.75 (0.59, 0.96) 11.41
GS 1.15 (1.03, 1.30) 18.36
ALSPAC(G0) 0.82 (0.52, 1.29) 5.36
TwinsUK 1.03 (0.75, 1.42) 8.49
BiB 1.00 (0.59, 1.70) 4.22
Overall 0.91 (0.81, 1.02)
Heterogeneity: τ² = 0.02, I² = 48.60%, H² = 1.95
Test of θ = 0: z = -1.57, p = 0.12
1/2
1
Random-effects REML model

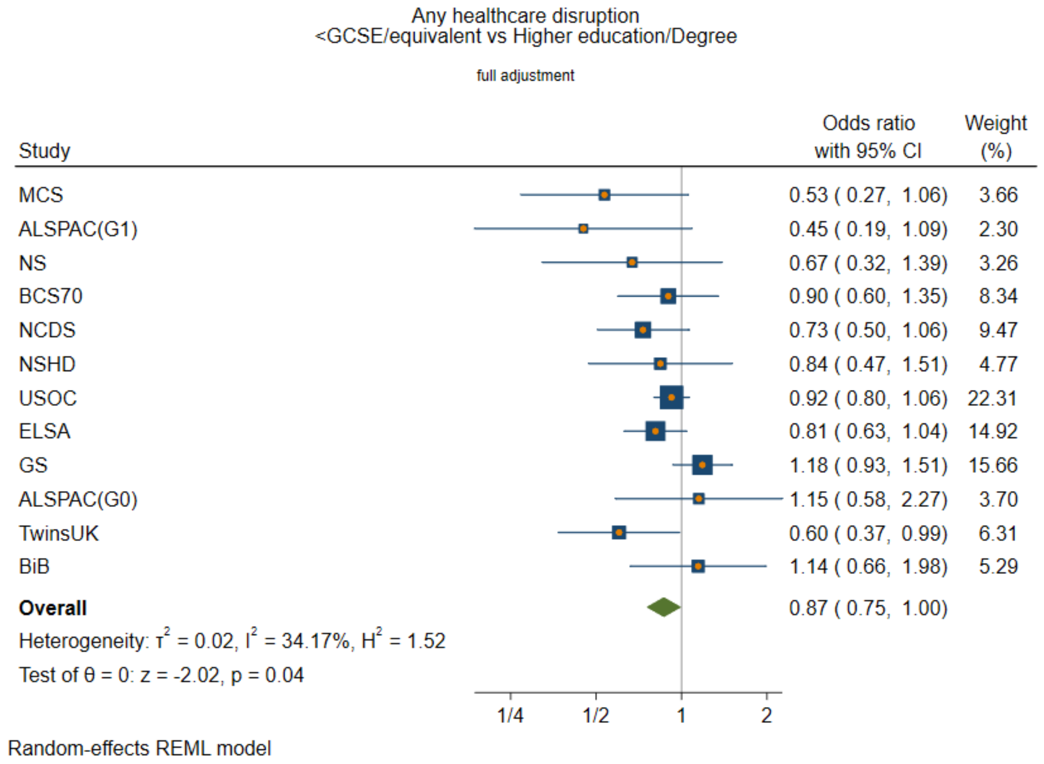
Any healthcare disruption
<GCSE/equivalent vs Higher education/Degree
full adjustment
Study
Odds ratio with 95% CI
Weight (%)
MCS 0.53 (0.27, 1.06) 3.66
ALSPAC(G1) 0.45 (0.19, 1.09) 2.30
NS 0.67 (0.32, 1.39) 3.26
BCS70 0.90 (0.60, 1.35) 8.34
NCDS 0.73 (0.50, 1.06) 9.47
NSHD 0.84 (0.47, 1.51) 4.77
USOC 0.92 (0.80, 1.06) 22.31
ELSA 0.81 (0.63, 1.04) 14.92
GS 1.18 (0.93, 1.51) 15.66
ALSPAC(G0) 1.15 (0.58, 2.27) 3.70
TwinsUK 0.60 (0.37, 0.99) 6.31
BiB 1.14 (0.66, 1.98) 5.29
Overall 0.87 (0.75, 1.00)
Heterogeneity: τ² = 0.02, I² = 34.17%, H² = 1.52
Test of θ = 0: z = -2.02, p = 0.04
1/4
1/2
1
2
Random-effects REML model

Age

Unadjusted

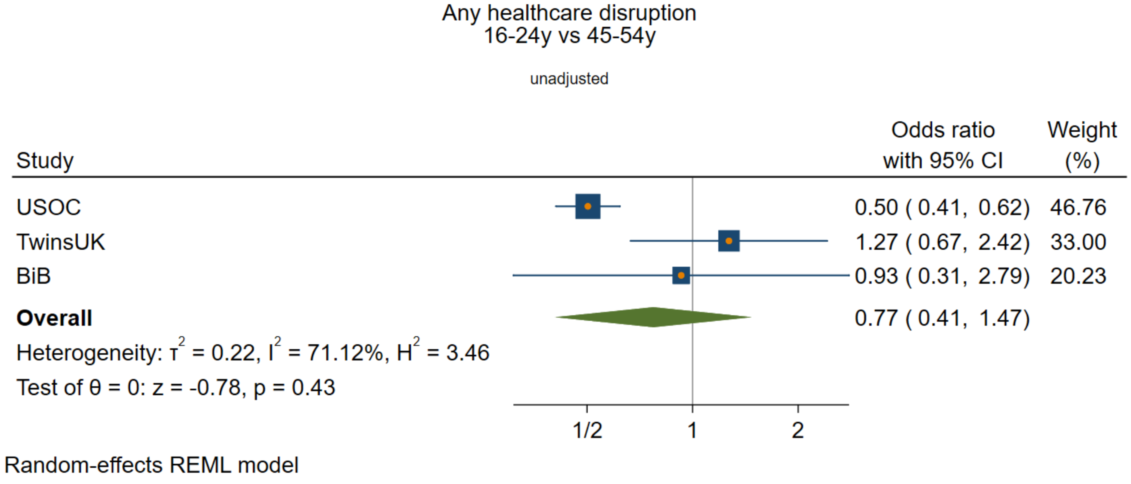

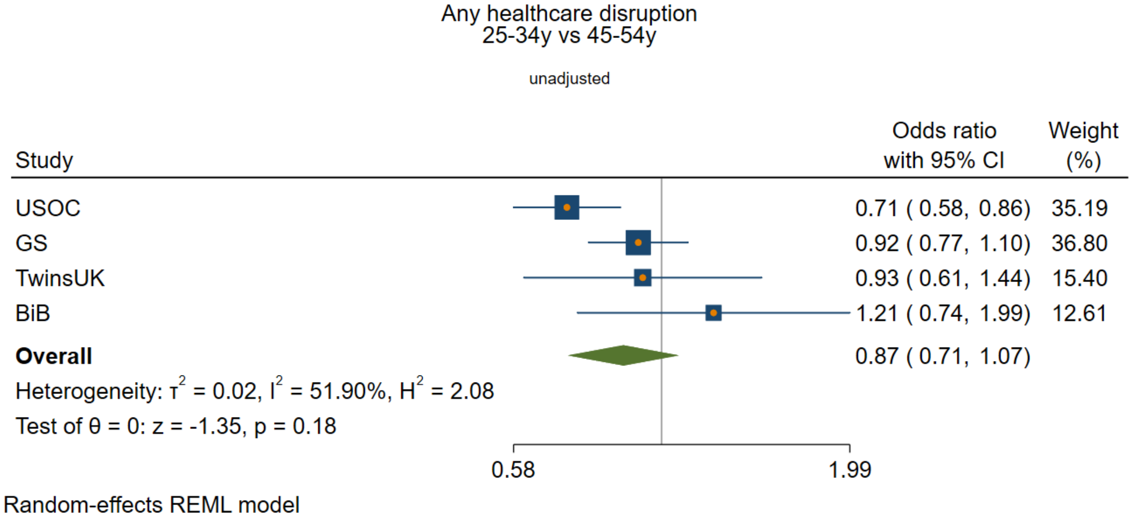

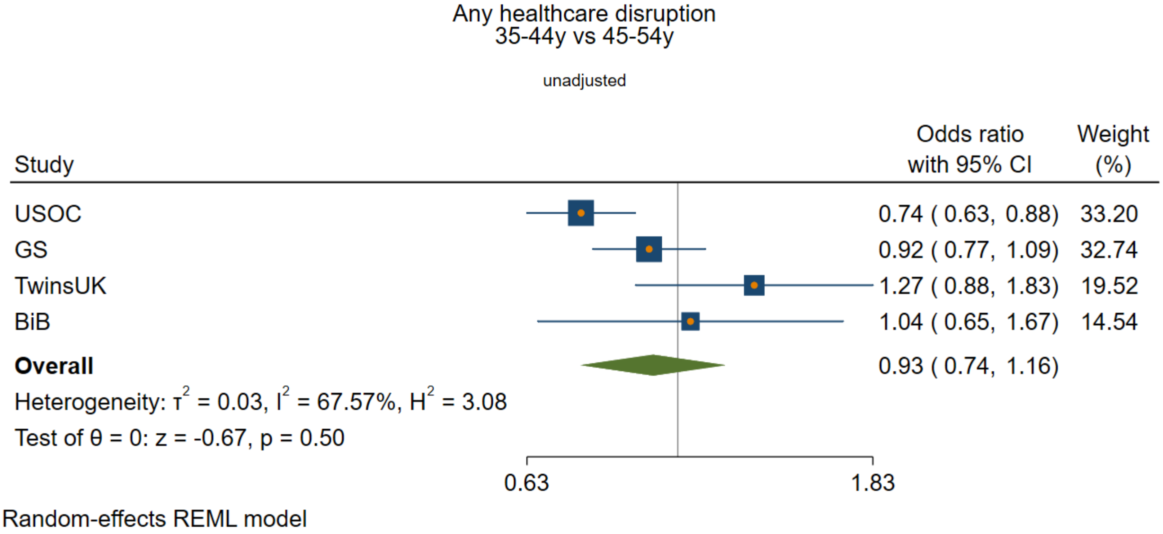

For peer review only - http://bmjopen.bmj.com/site/about/guidelines.xhtml

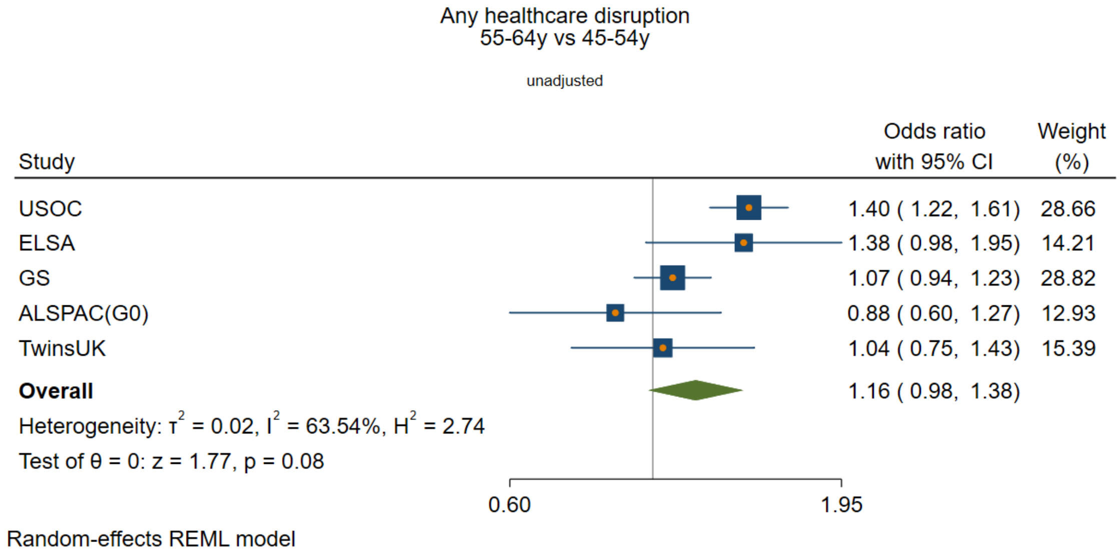

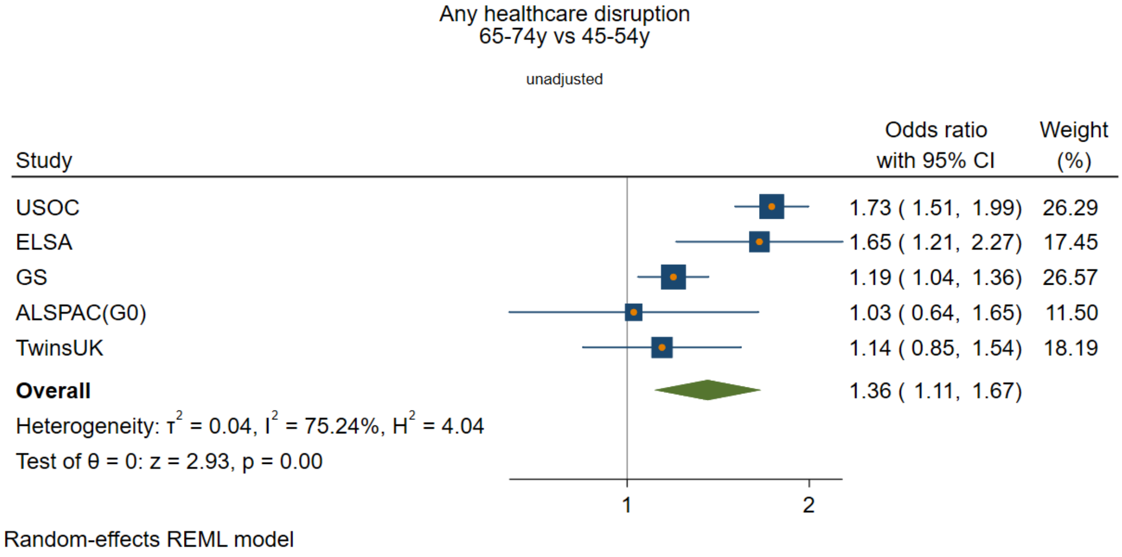

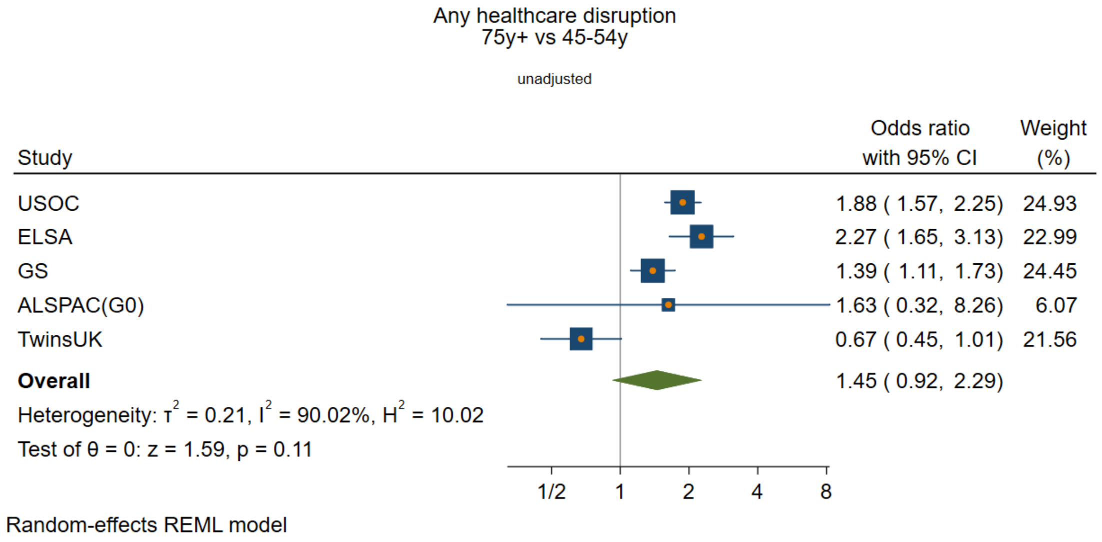

For peer review only - http://bmjopen.bmj.com/site/about/guidelines.xhtml

Basic adjustment

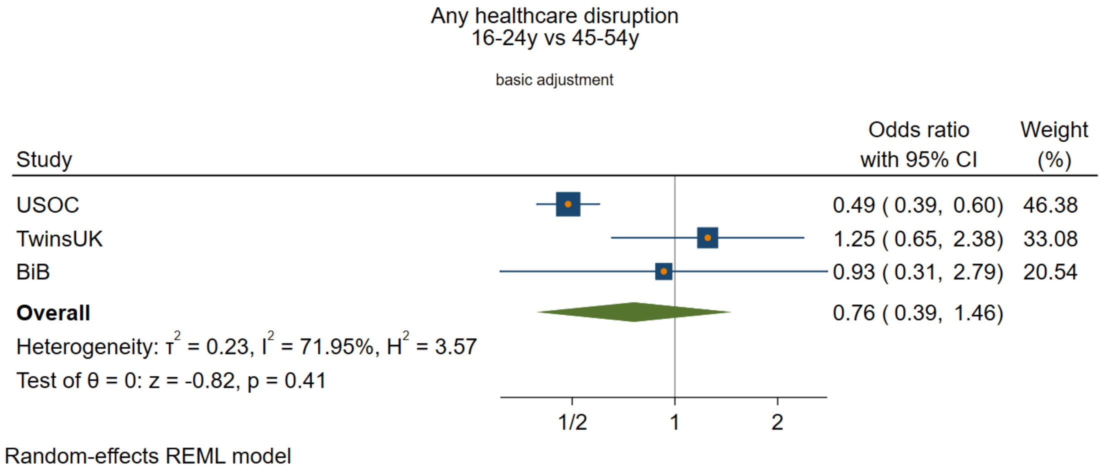

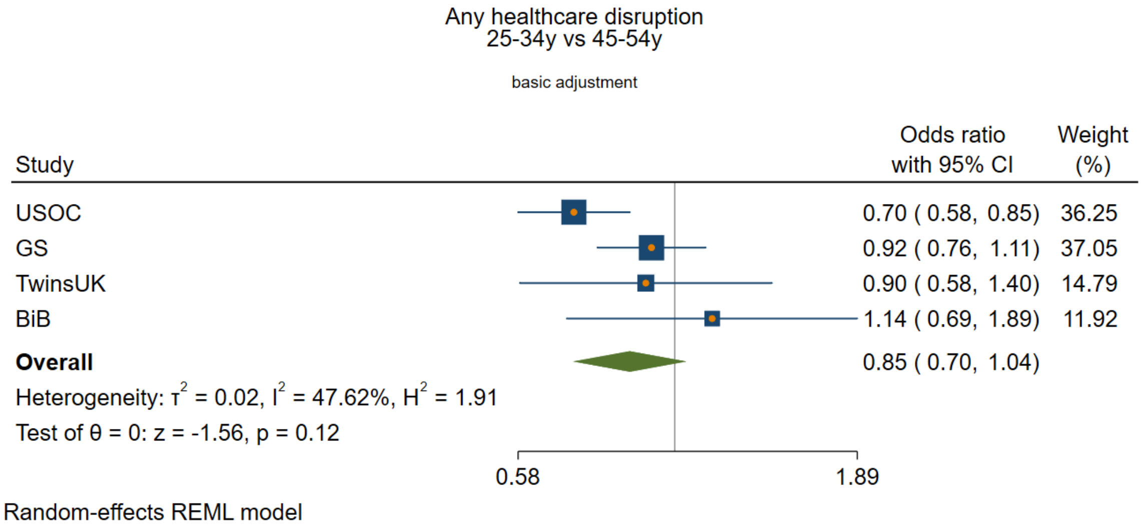

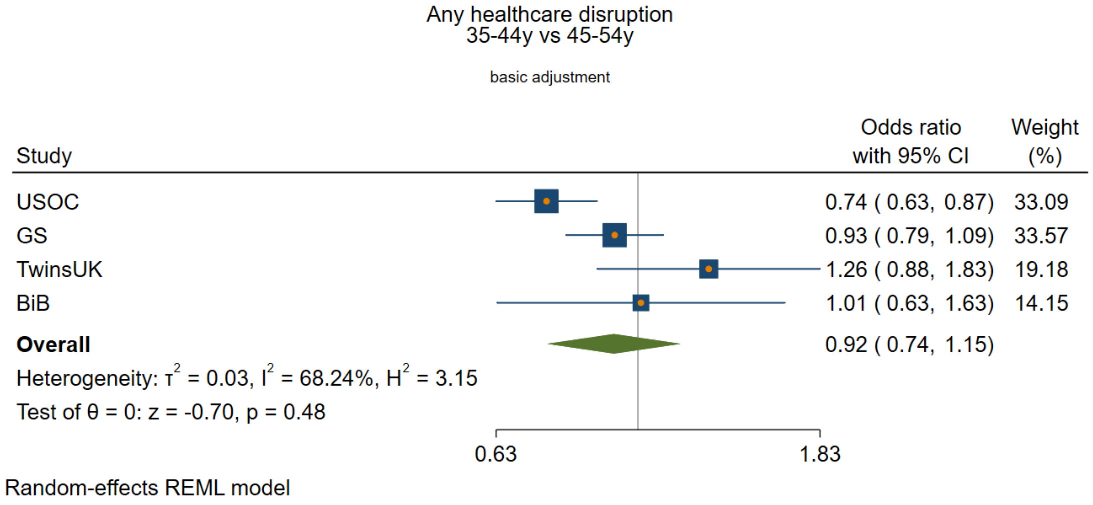

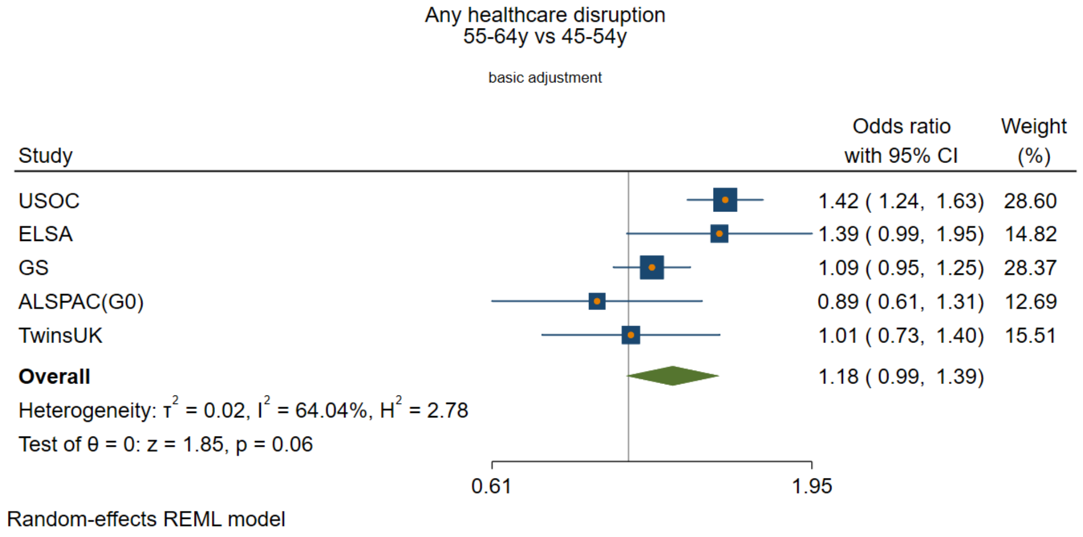

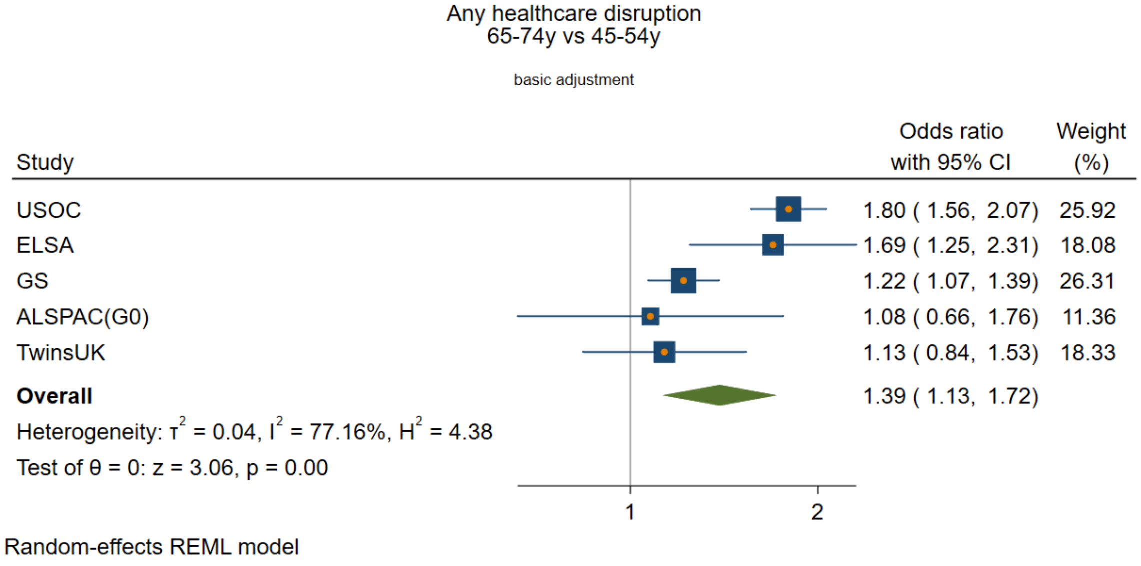

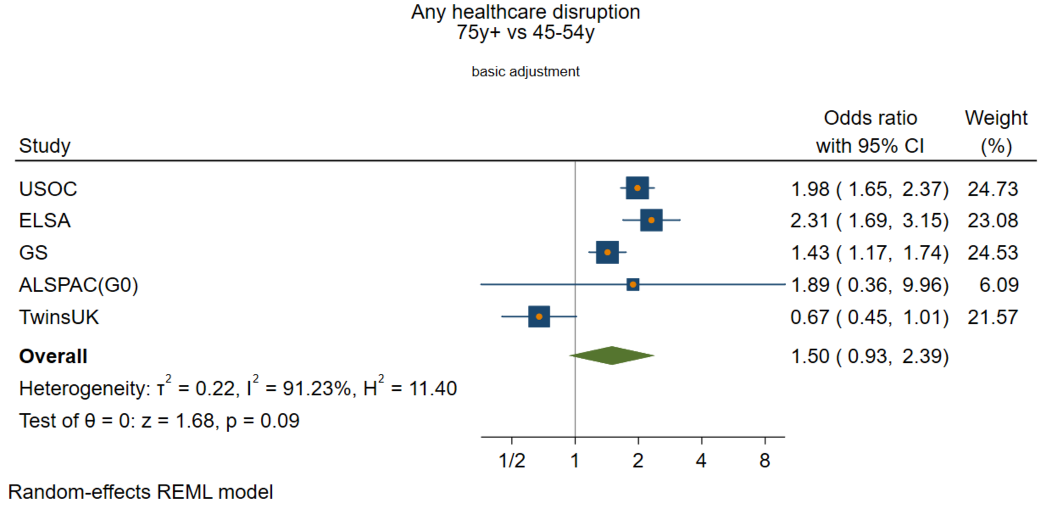

For peer review only - http://bmjopen.bmj.com/site/about/guidelines.xhtml

Full adjustment

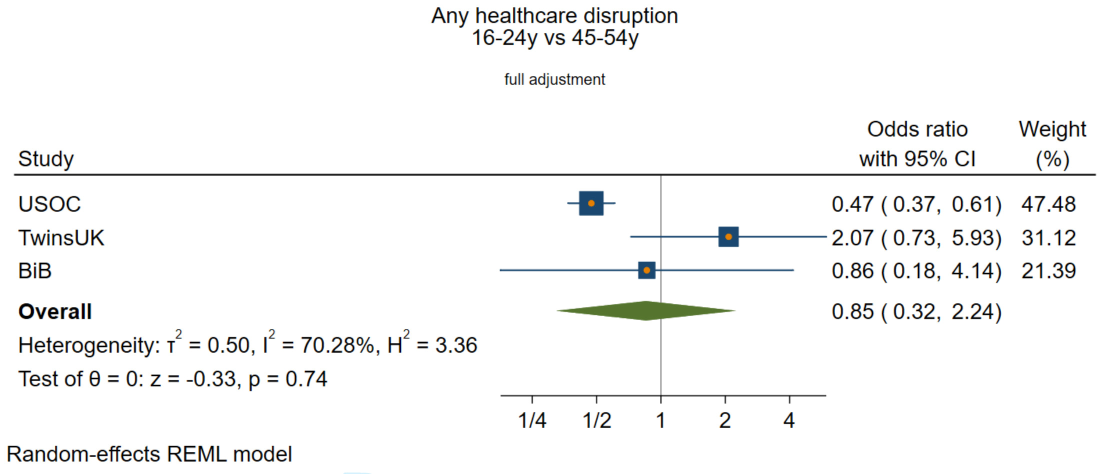

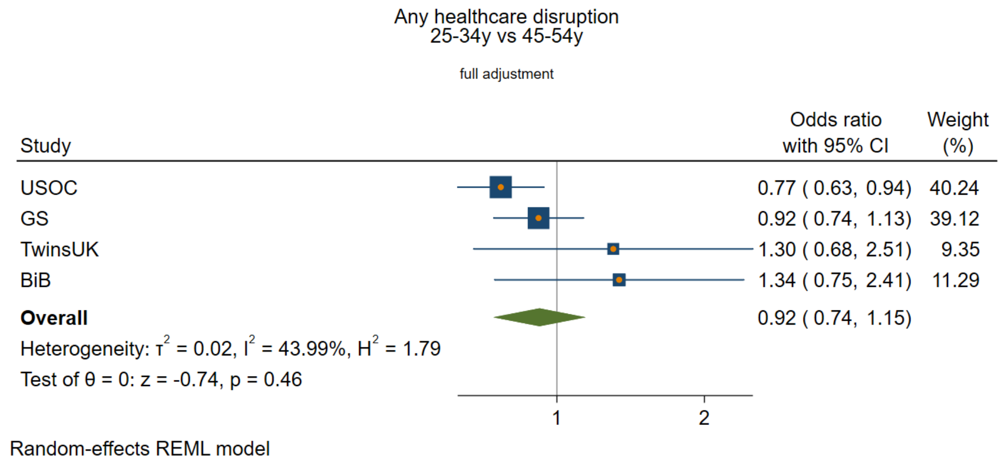

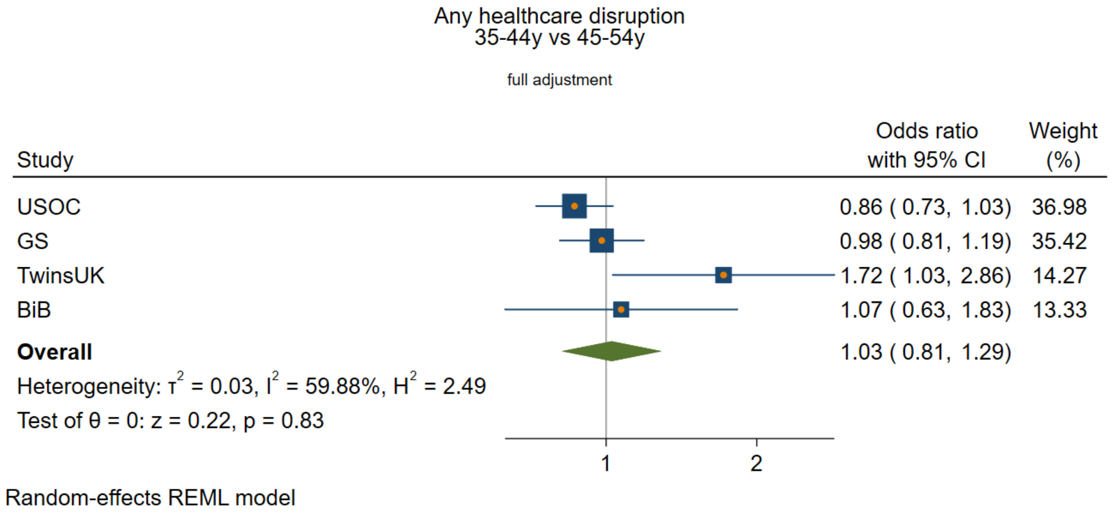

For peer review only - http://bmjopen.bmj.com/site/about/guidelines.xhtml

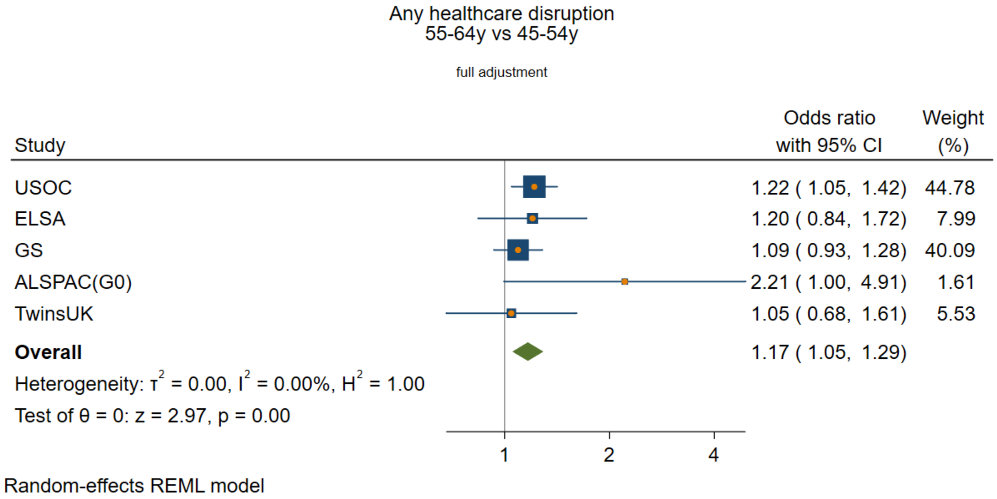

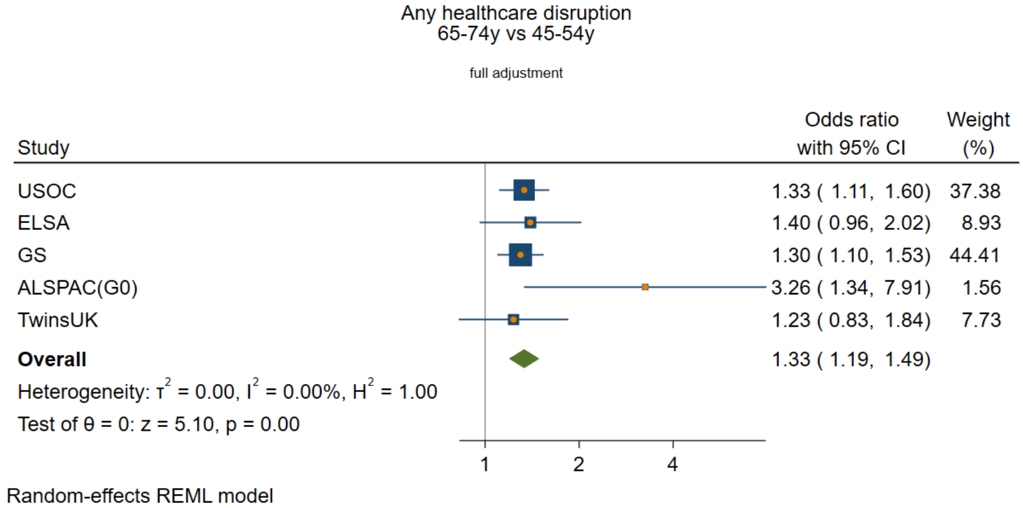

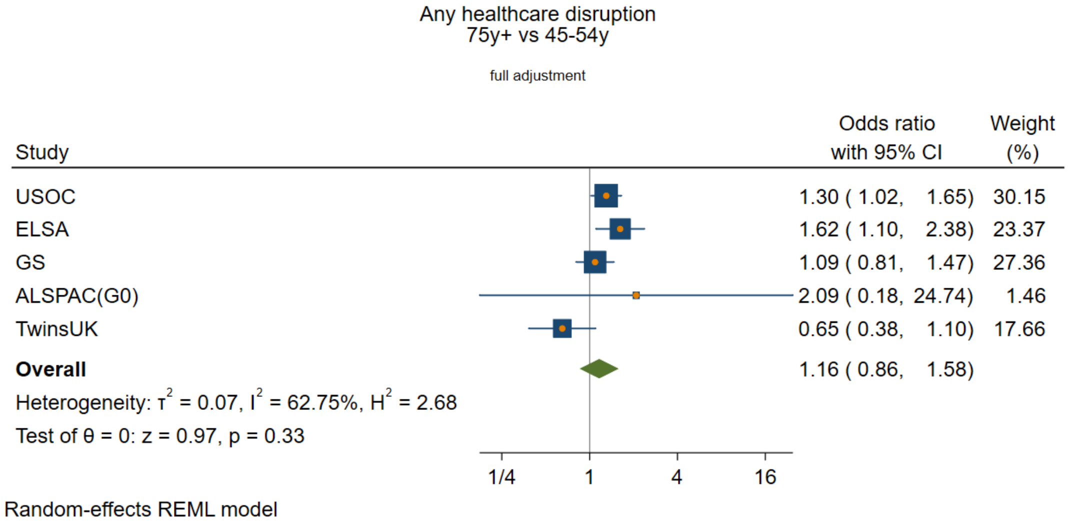

For peer review only - http://bmjopen.bmj.com/site/about/guidelines.xhtml

Ethnicity

Unadjusted

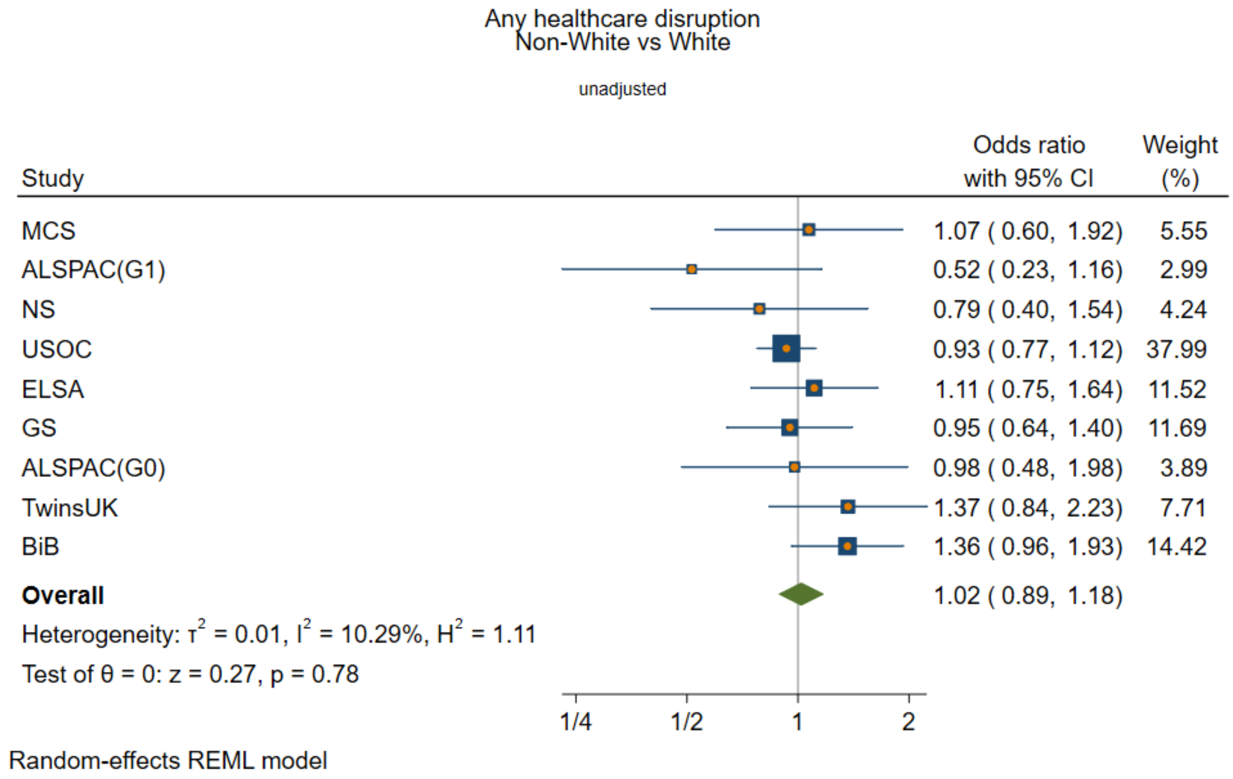

For peer review only - http://bmjopen.bmj.com/site/about/guidelines.xhtml

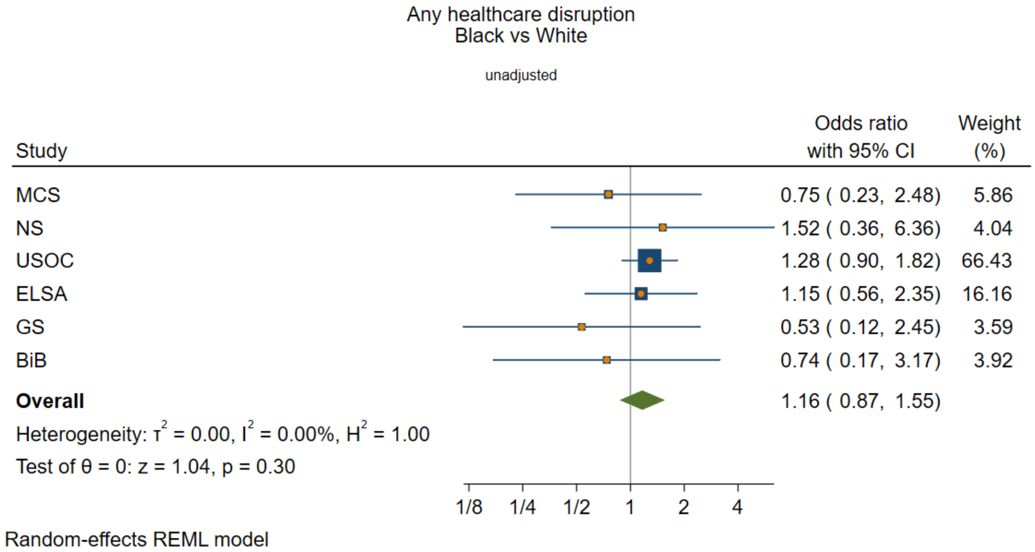

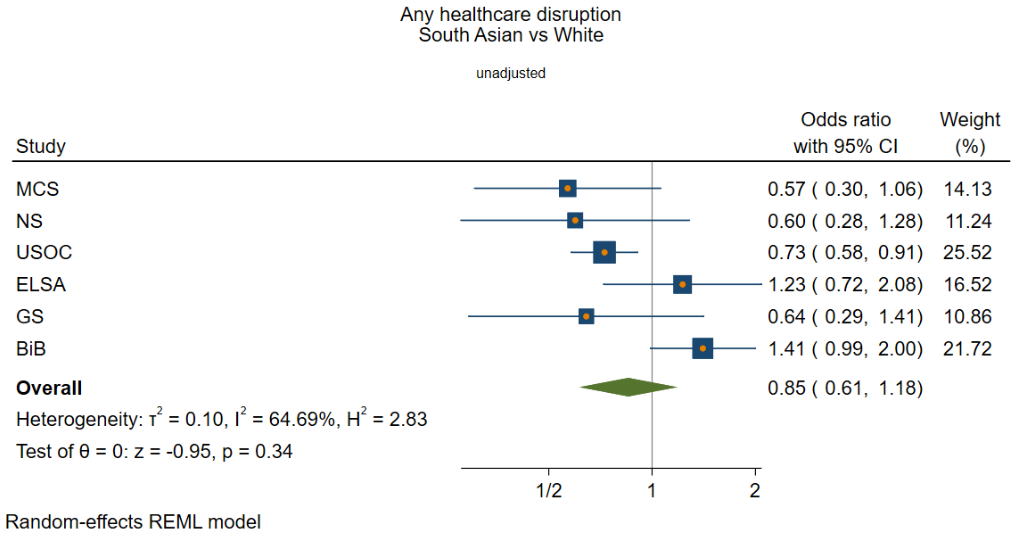

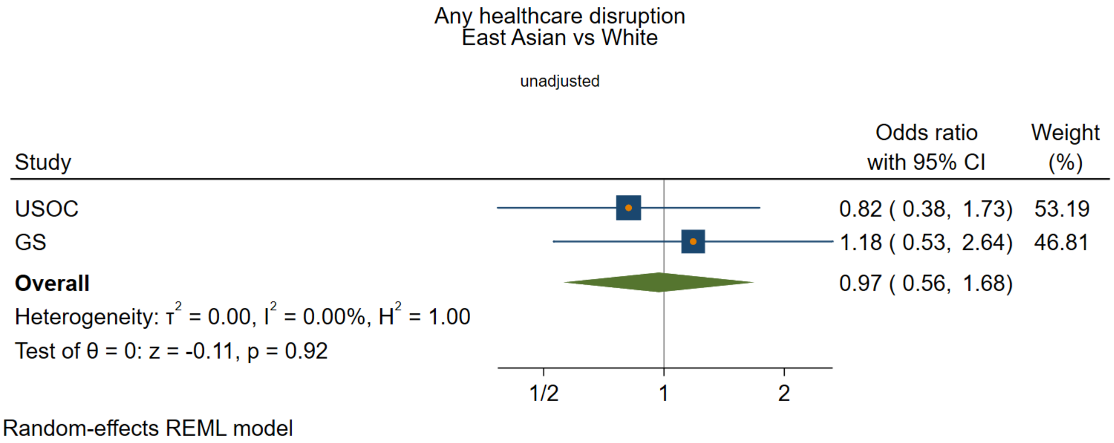

For peer review only - http://bmjopen.bmj.com/site/about/guidelines.xhtml

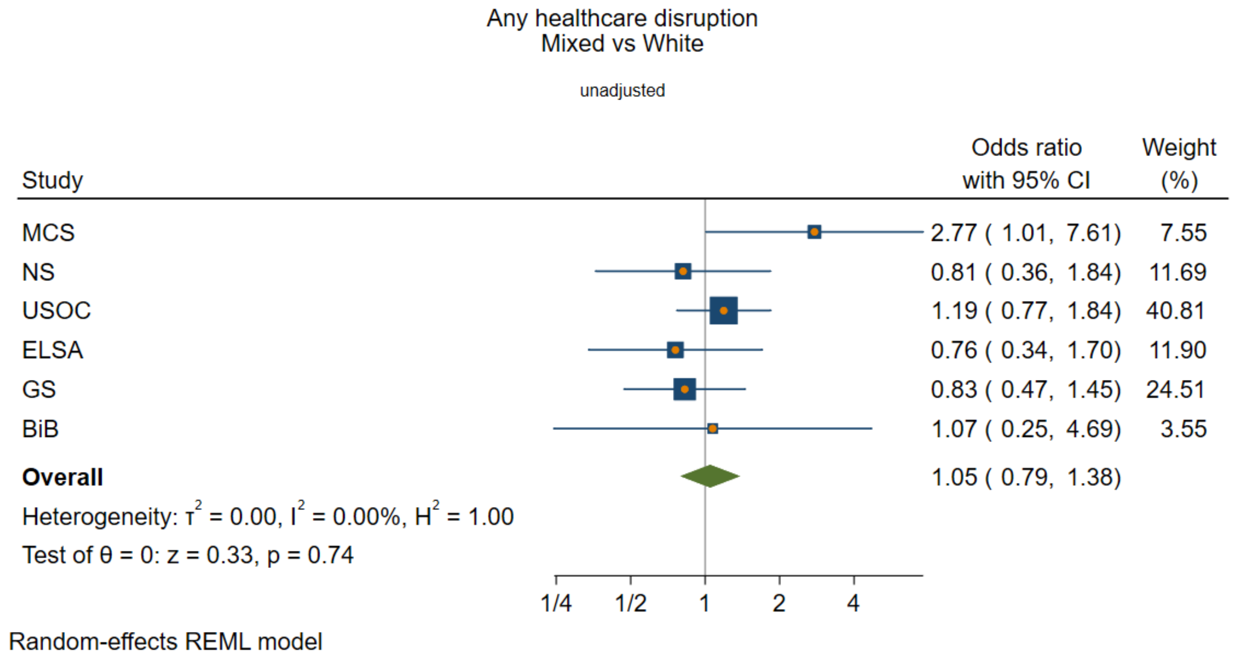

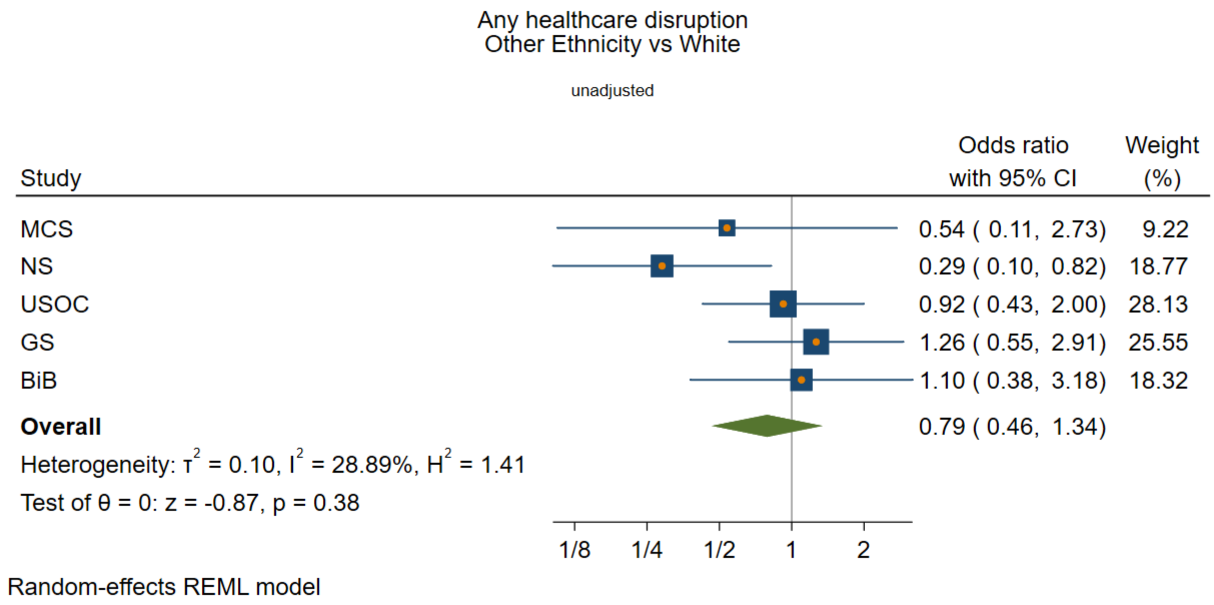

For peer review only - http://bmjopen.bmj.com/site/about/guidelines.xhtml

Basic adjustment

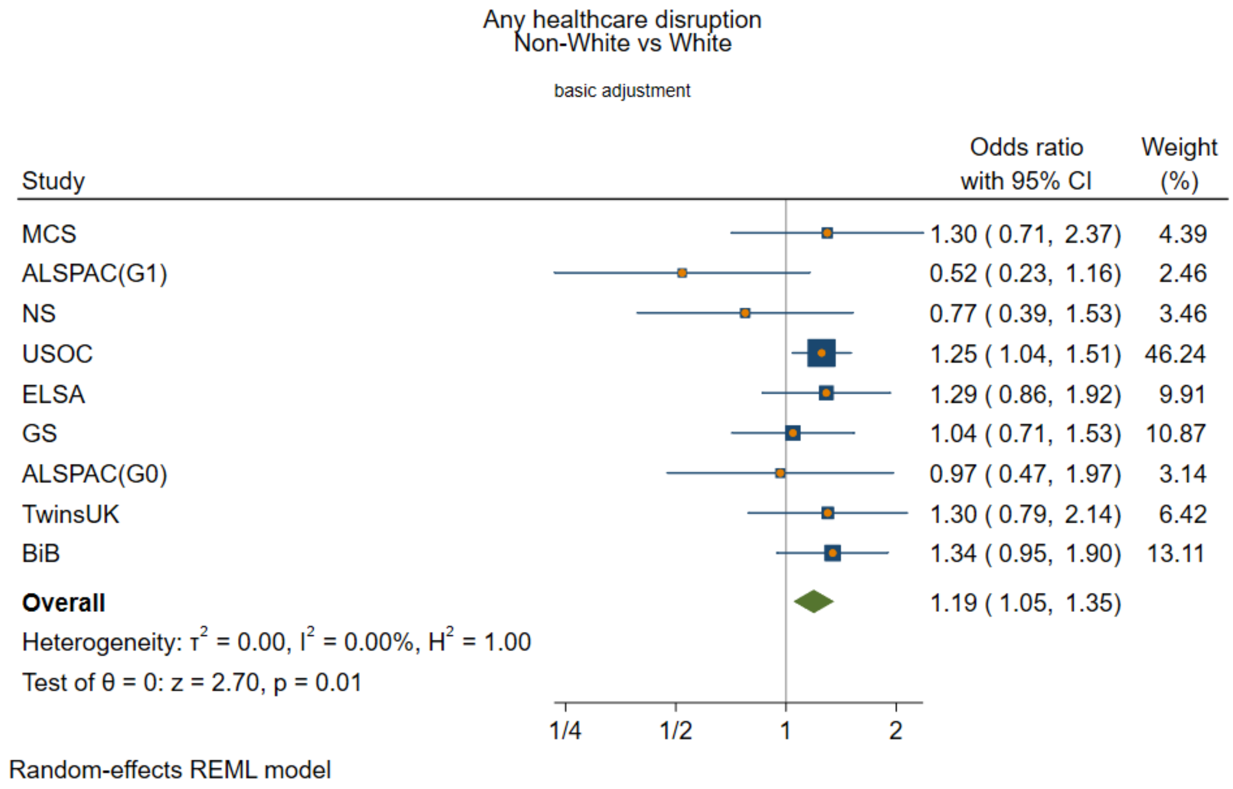

For peer review only - http://bmjopen.bmj.com/site/about/guidelines.xhtml

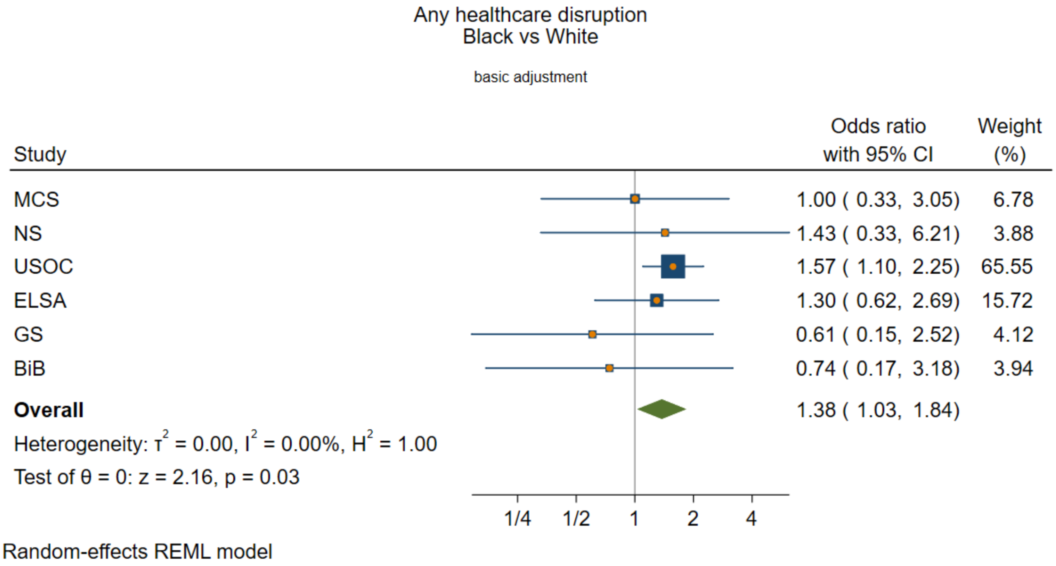

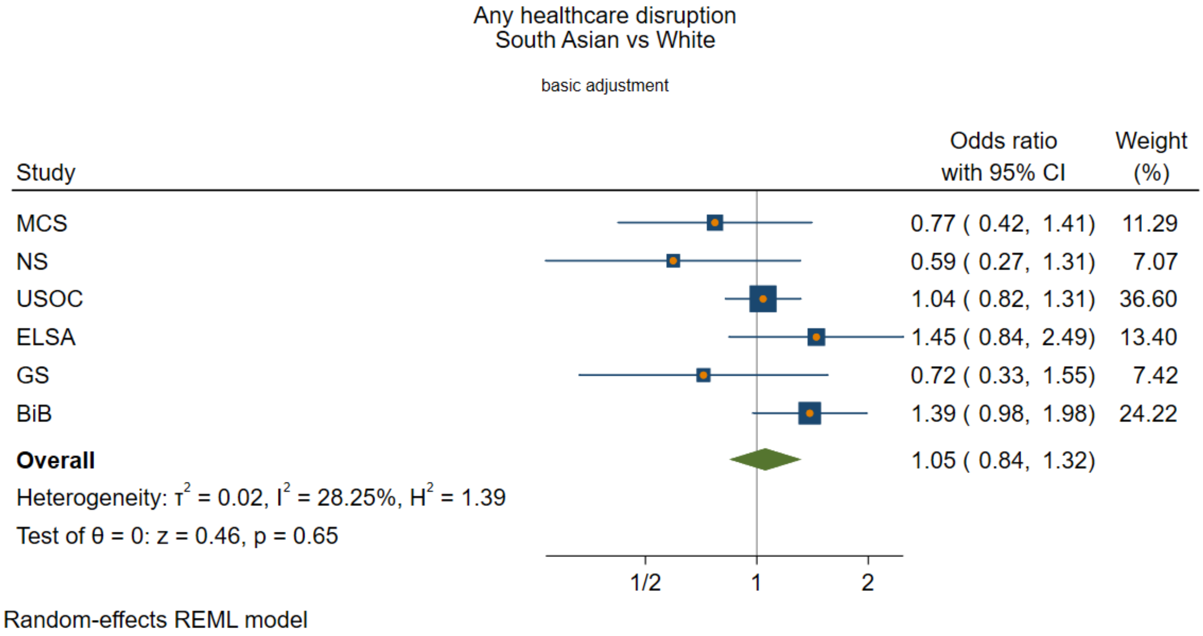

For peer review only - http://bmjopen.bmj.com/site/about/guidelines.xhtml

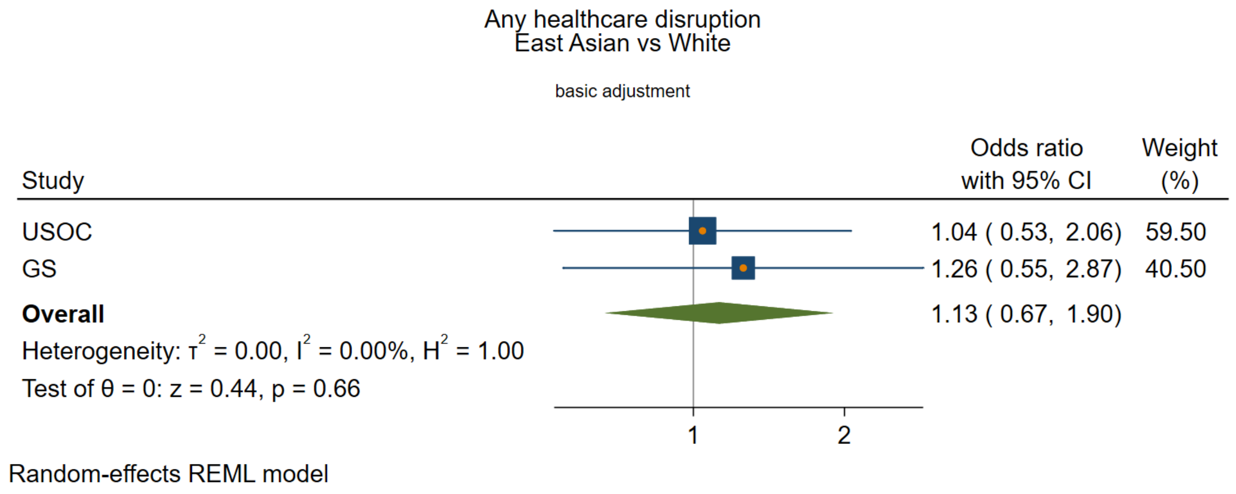

For peer review only

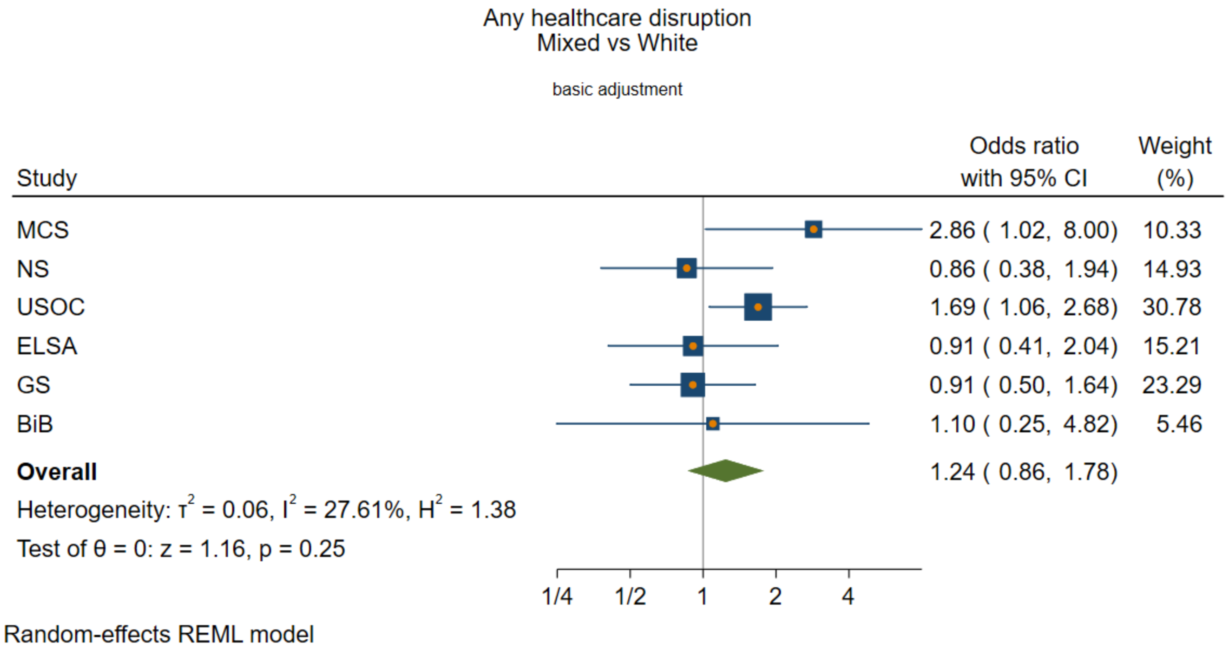

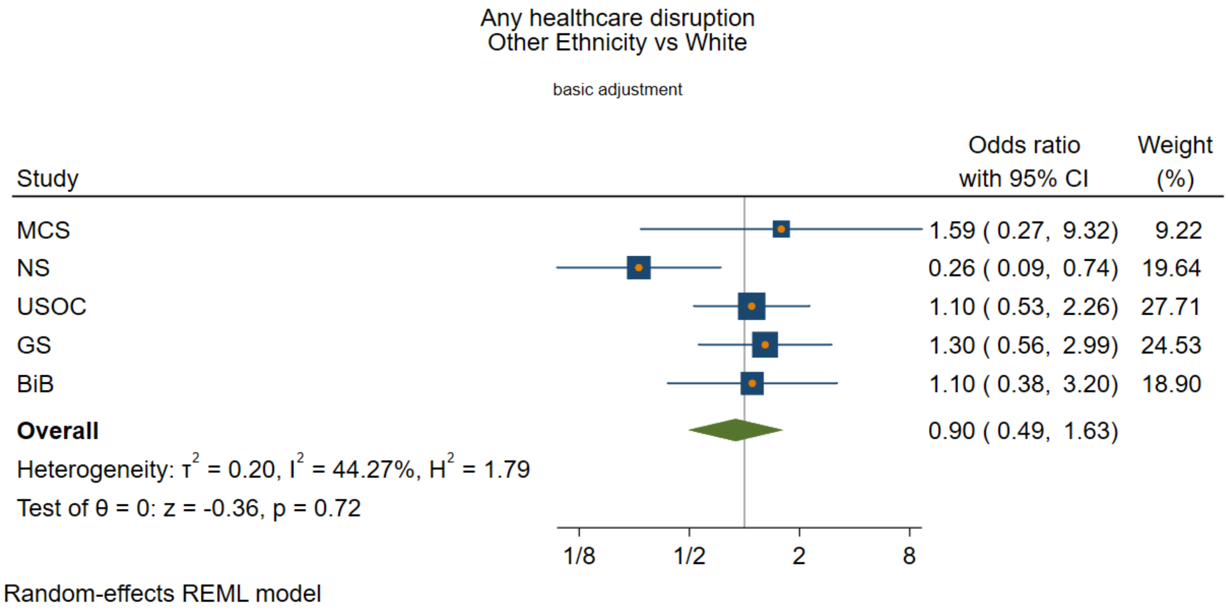

For peer review only - http://bmjopen.bmj.com/site/about/guidelines.xhtml

Full adjustment

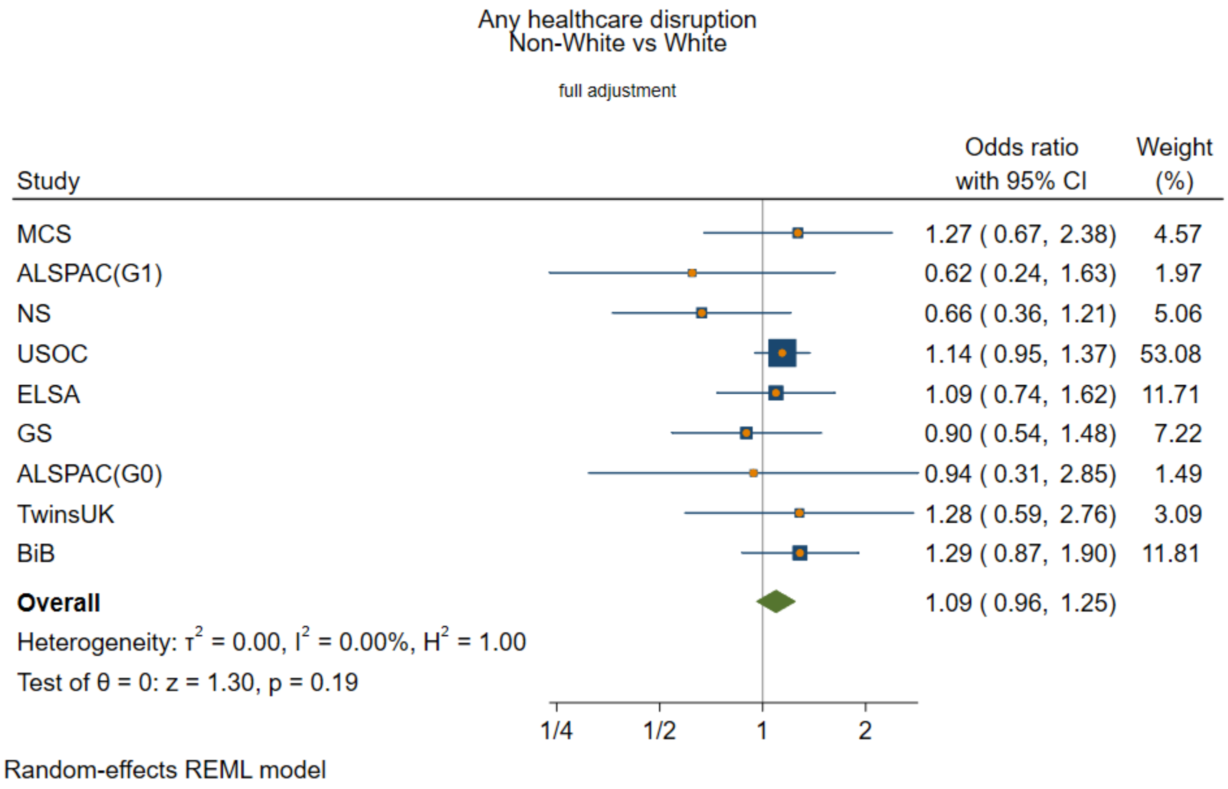

view only

For peer review only - http://bmjopen.bmj.com/site/about/guidelines.xhtml

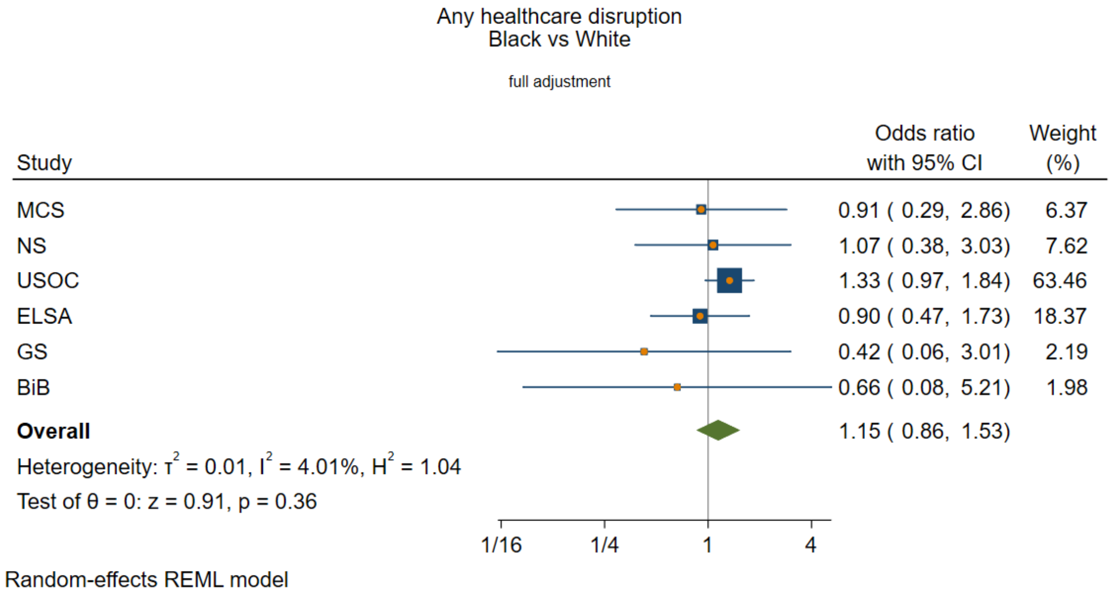

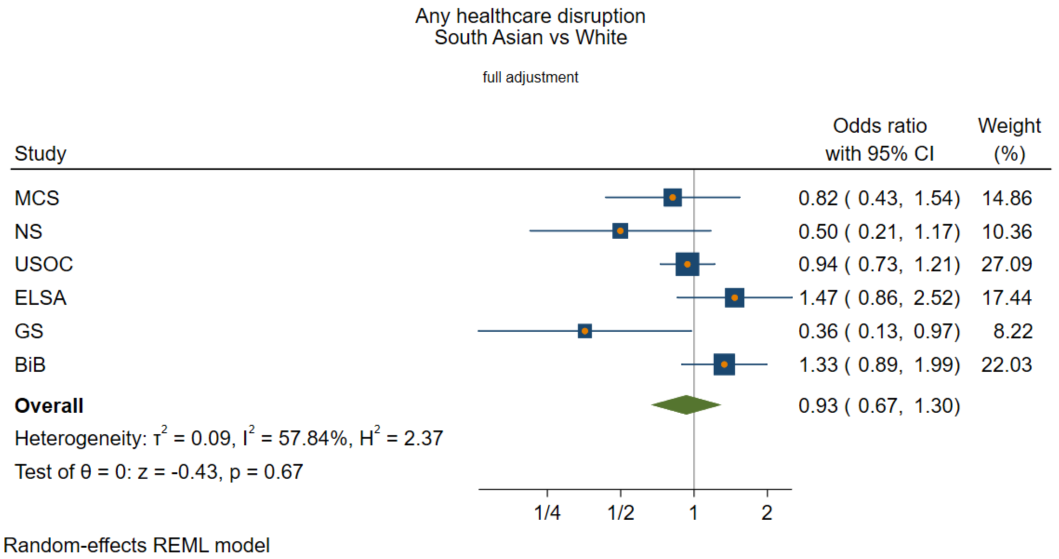

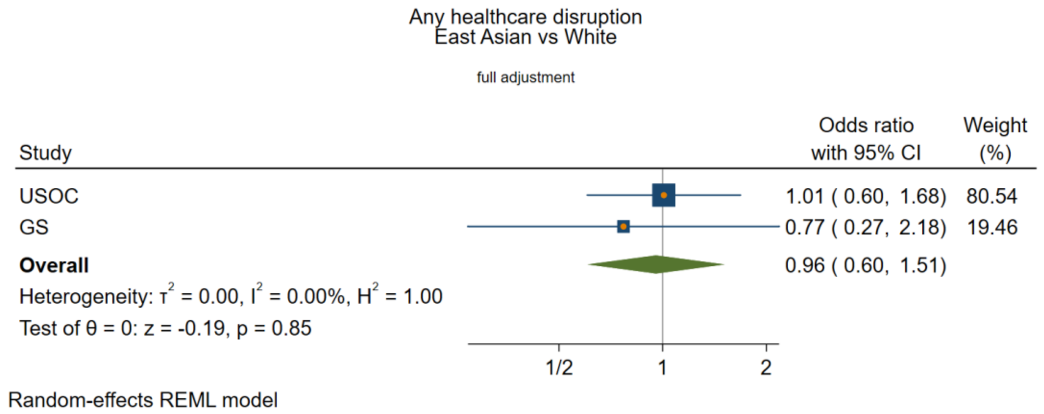

For peer review only - http://bmjopen.bmj.com/site/about/guidelines.xhtml

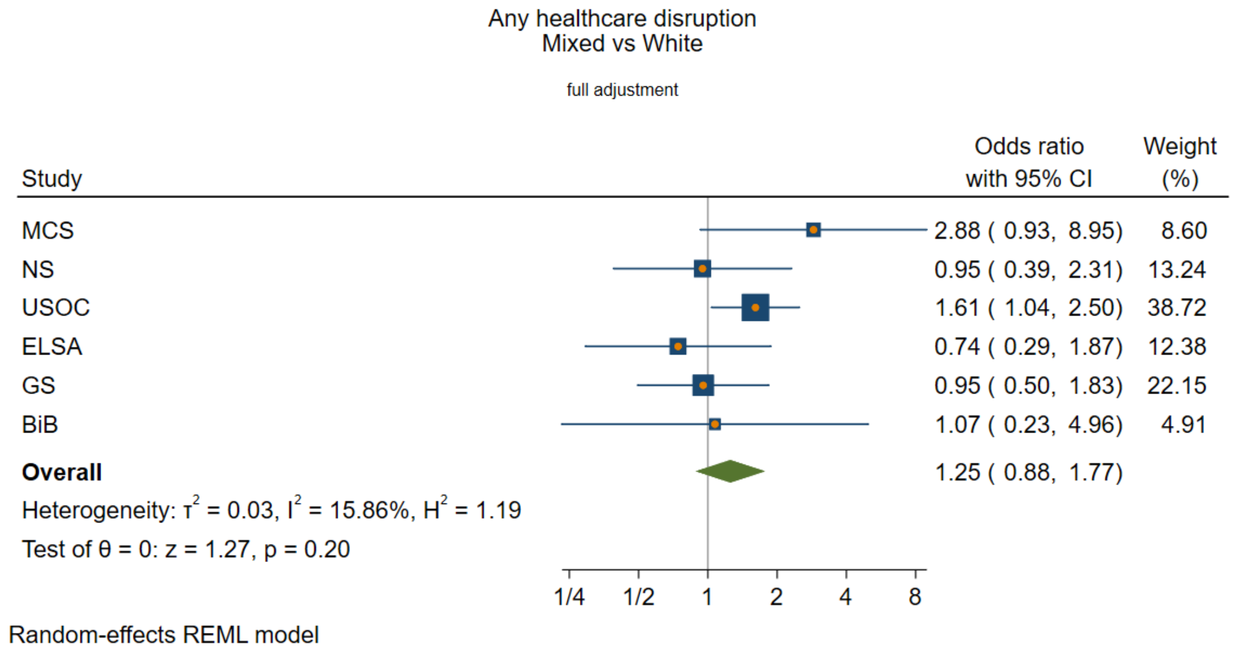

review

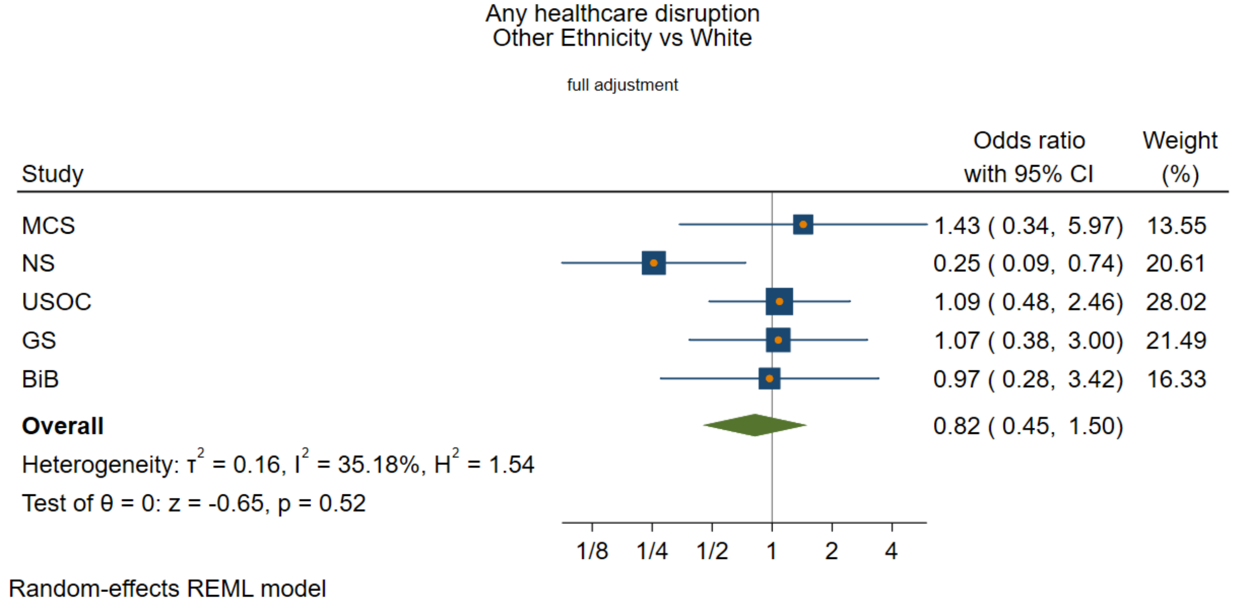

For peer review only - http://bmjopen.bmj.com/site/about/guidelines.xhtml

Appointments

Sex

Unadjusted

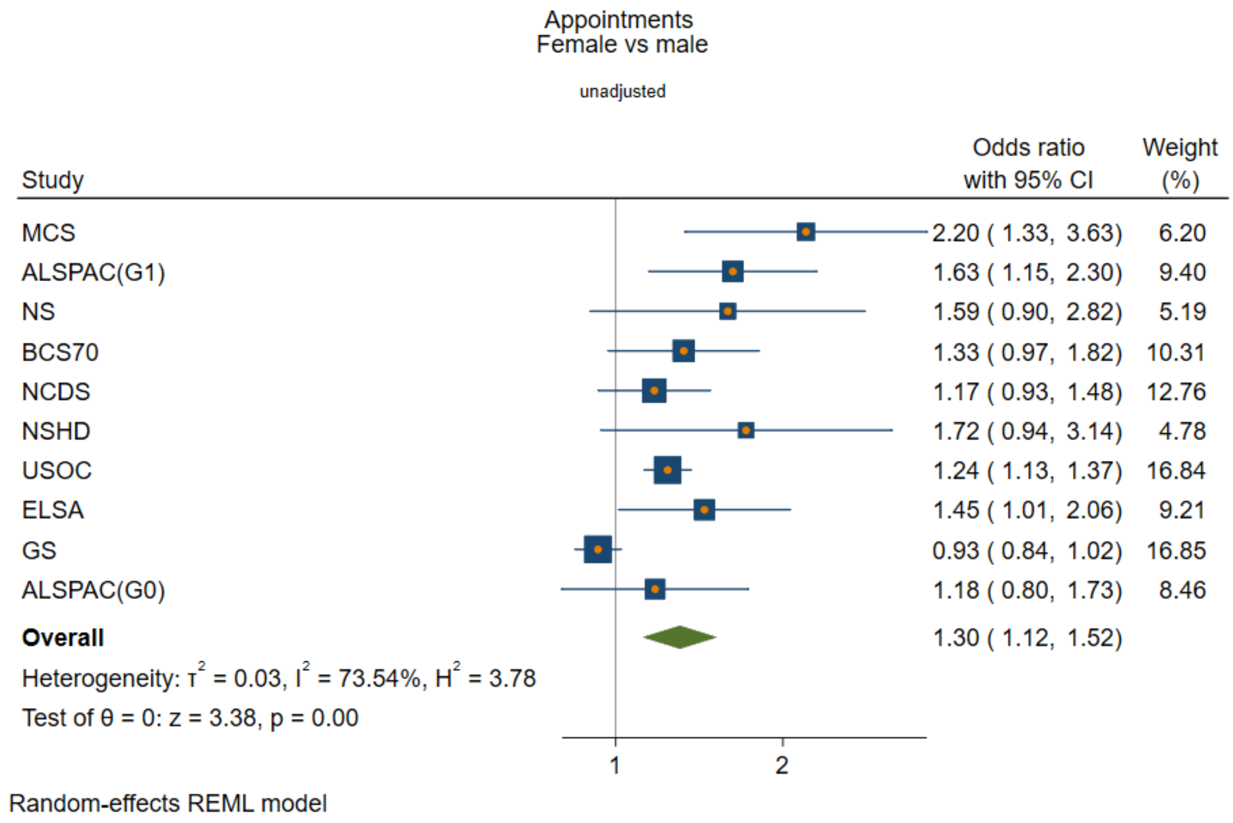

w only

For peer review only - http://bmjopen.bmj.com/site/about/guidelines.xhtml

Basic adjustment

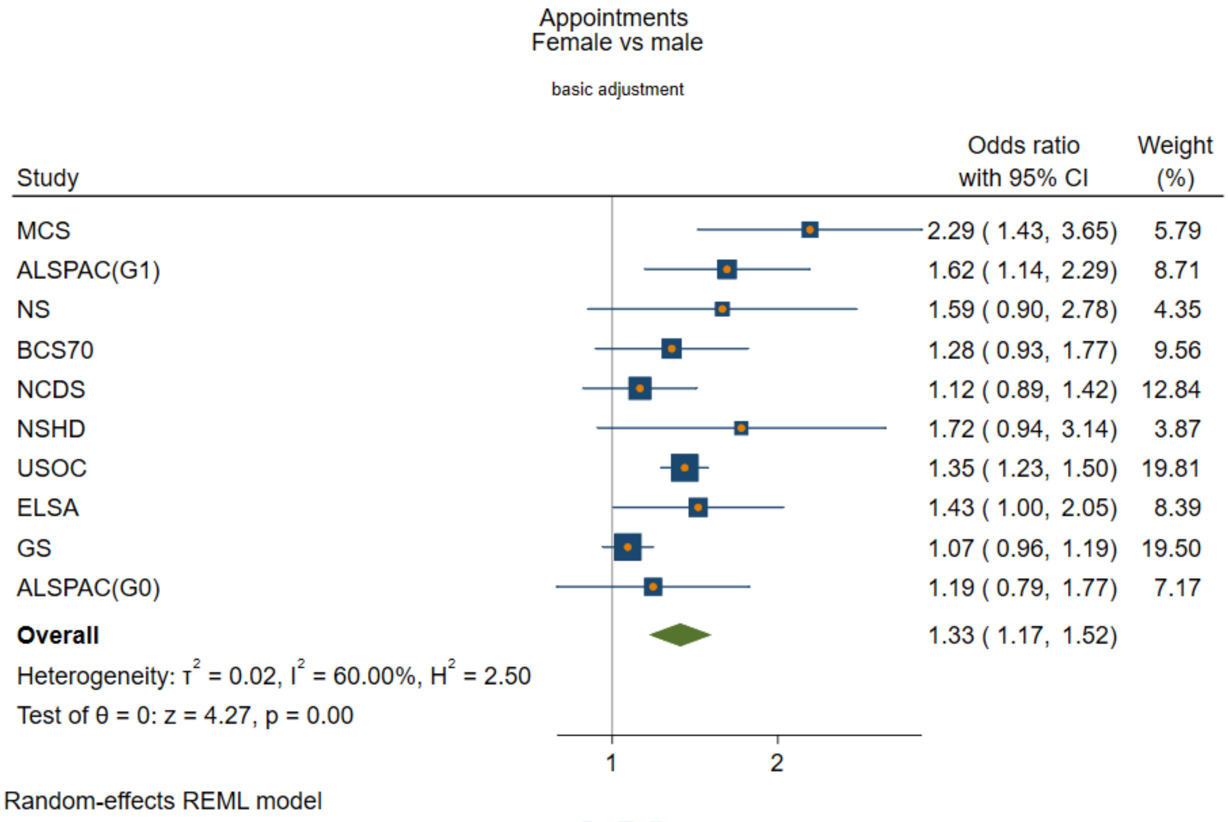

For peer review only - http://bmjopen.bmj.com/site/about/guidelines.xhtml

Full adjustment

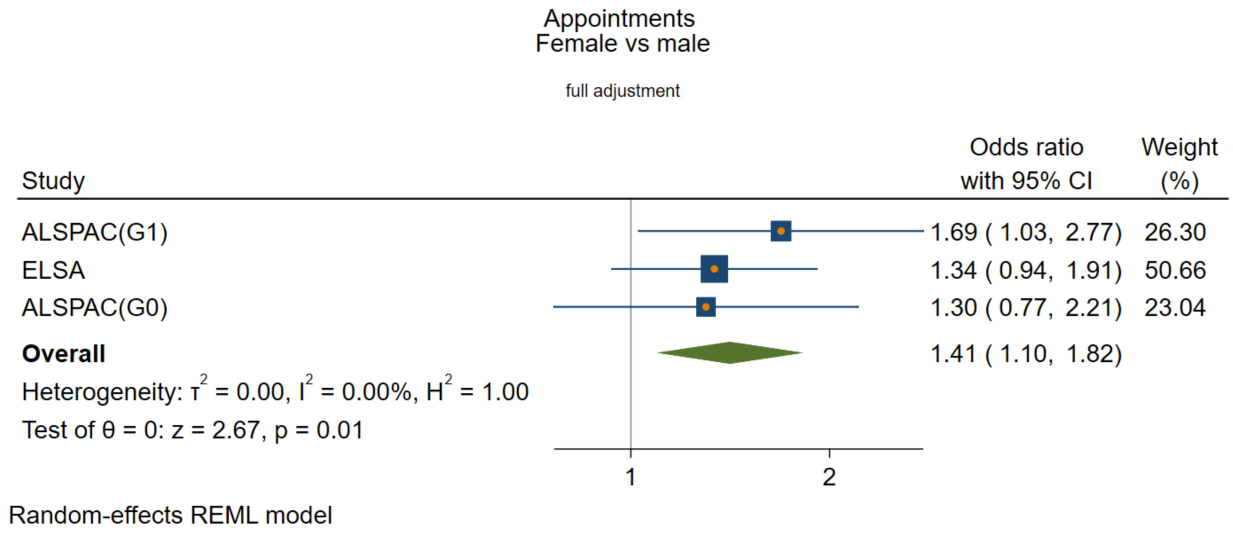

For peer review only - http://bmjopen.bmj.com/site/about/guidelines.xhtml

Occupational class
Unadjusted

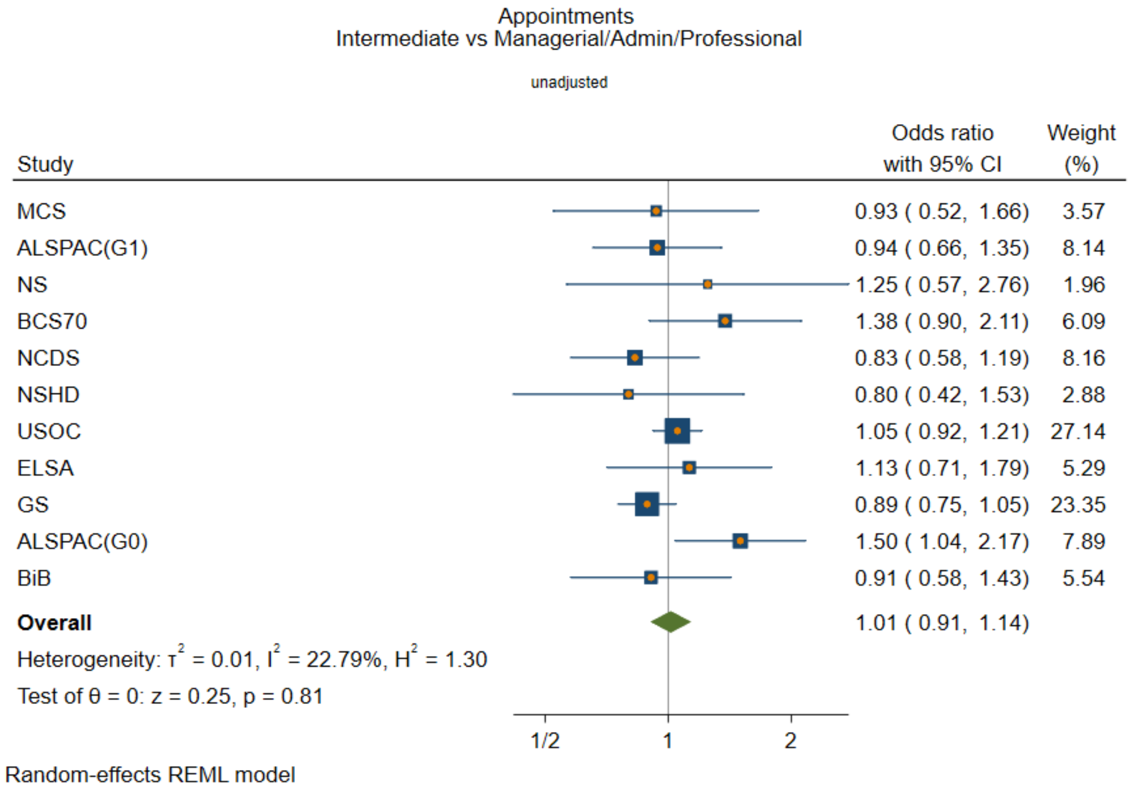

For peer review only - http://bmjopen.bmj.com/site/about/guidelines.xhtml

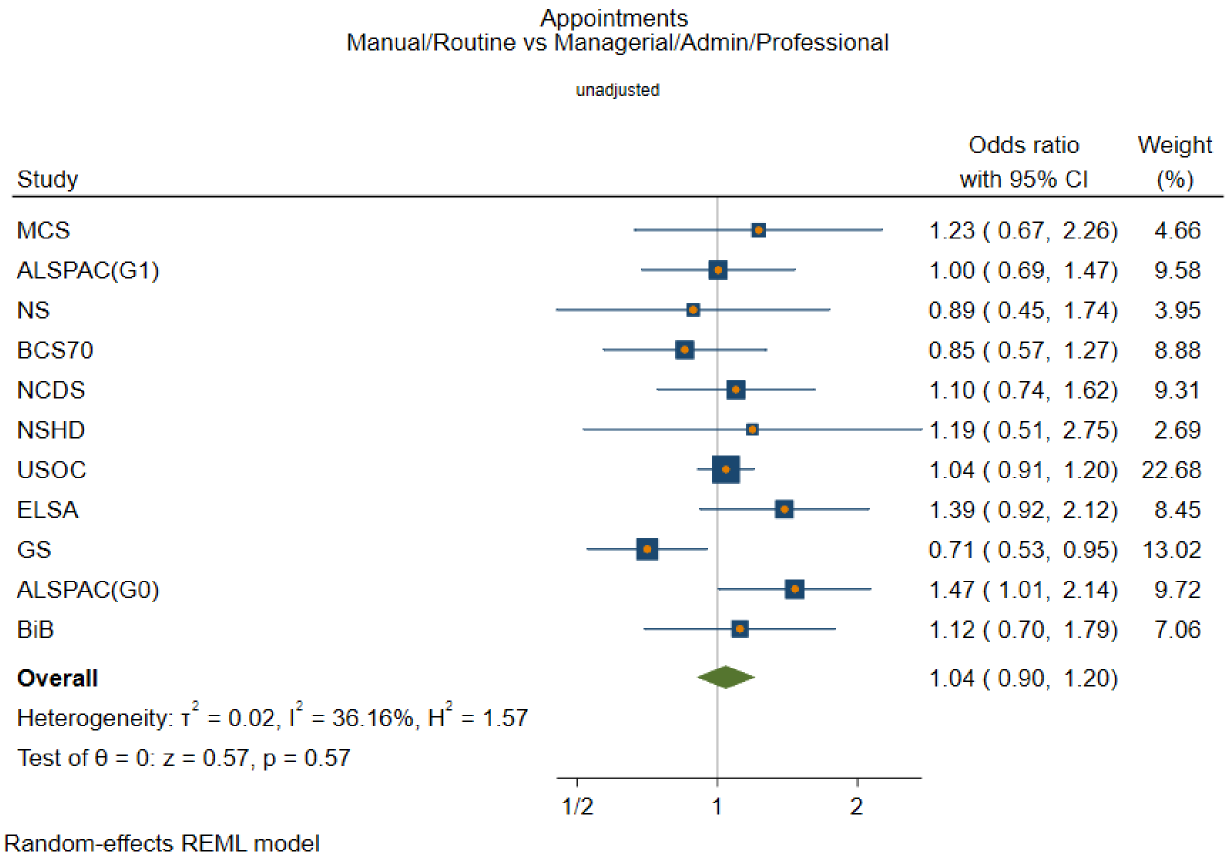
Appointments
Manual/Routine vs Managerial/Admin/Professional
unadjusted
Study
Odds ratio with 95% CI
Weight (%)
MCS 1.23 (0.67, 2.26) 4.66
ALSPAC(G1) 1.00 (0.69, 1.47) 9.58
NS 0.89 (0.45, 1.74) 3.95
BCS70 0.85 (0.57, 1.27) 8.88
NCDS 1.10 (0.74, 1.62) 9.31
NSHD 1.19 (0.51, 2.75) 2.69
USOC 1.04 (0.91, 1.20) 22.68
ELSA 1.39 (0.92, 2.12) 8.45
GS 0.71 (0.53, 0.95) 13.02
ALSPAC(G0) 1.47 (1.01, 2.14) 9.72
BiB 1.12 (0.70, 1.79) 7.06
Overall 1.04 (0.90, 1.20)
Heterogeneity: τ² = 0.02, I² = 36.16%, H² = 1.57
Test of θ = 0: z = 0.57, p = 0.57
1/2
1
2
Random-effects REML model

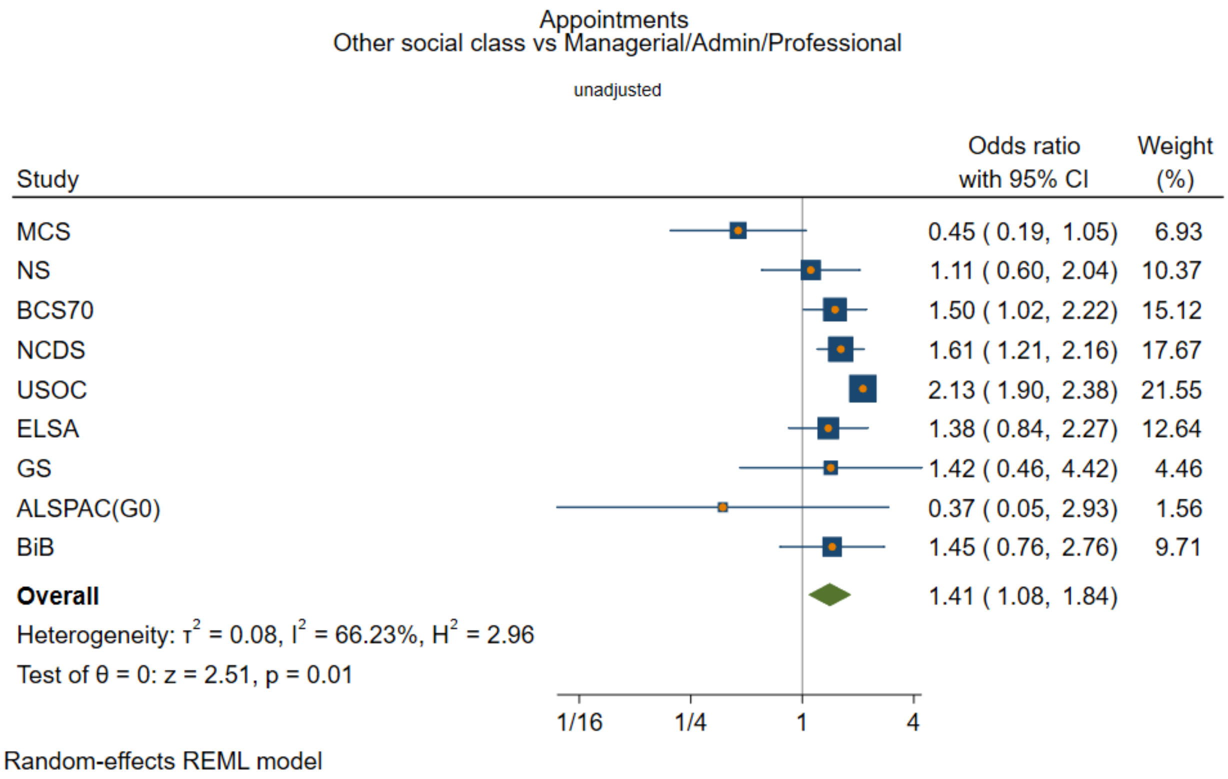
Appointments
Other social class vs Managerial/Admin/Professional
unadjusted
Study
Odds ratio with 95% CI
Weight (%)
MCS 0.45 (0.19, 1.05) 6.93
NS 1.11 (0.60, 2.04) 10.37
BCS70 1.50 (1.02, 2.22) 15.12
NCDS 1.61 (1.21, 2.16) 17.67
USOC 2.13 (1.90, 2.38) 21.55
ELSA 1.38 (0.84, 2.27) 12.64
GS 1.42 (0.46, 4.42) 4.46
ALSPAC(G0) 0.37 (0.05, 2.93) 1.56
BiB 1.45 (0.76, 2.76) 9.71
Overall 1.41 (1.08, 1.84)
Heterogeneity: τ² = 0.08, I² = 66.23%, H² = 2.96
Test of θ = 0: z = 2.51, p = 0.01
1/16
1/4
1
4
Random-effects REML model

Basic adjustment

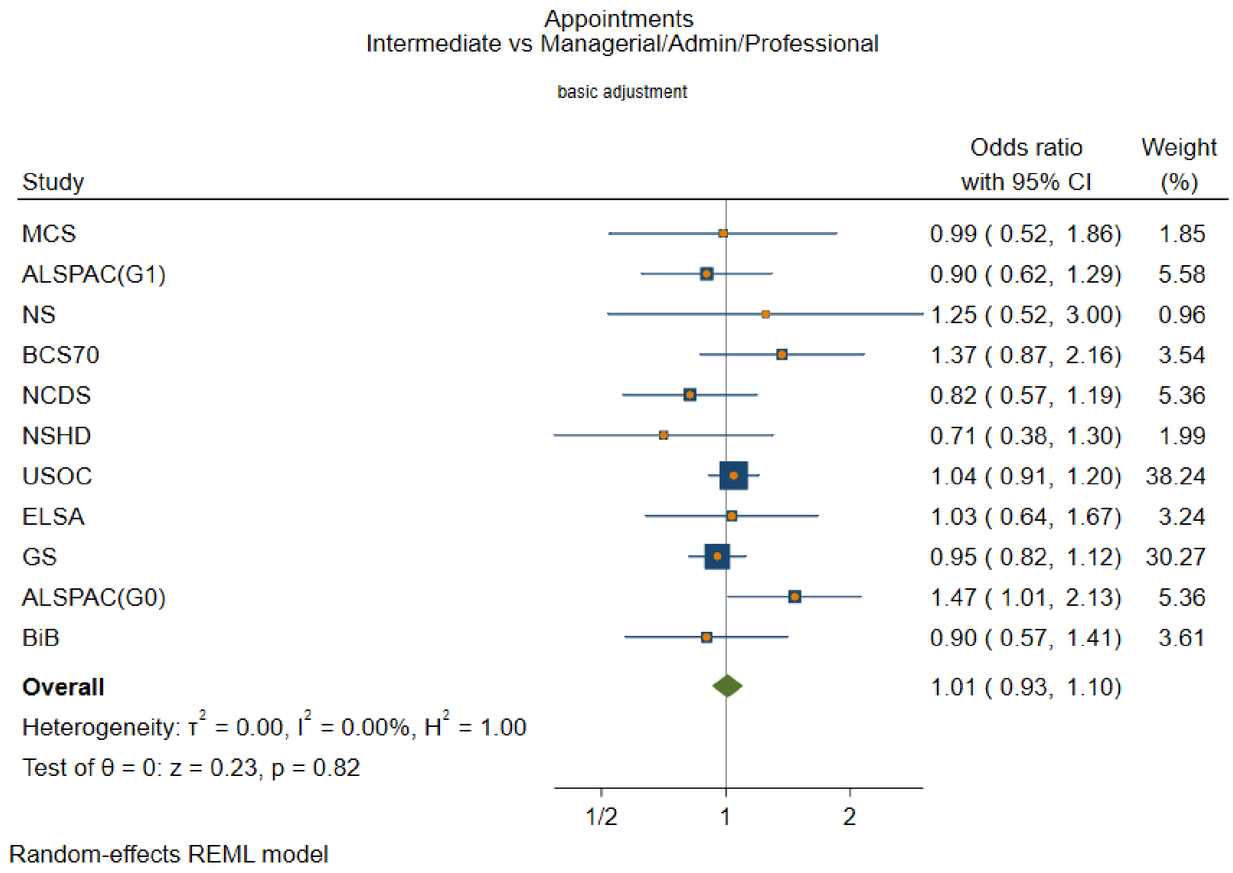

only

For peer review only - http://bmjopen.bmj.com/site/about/guidelines.xhtml

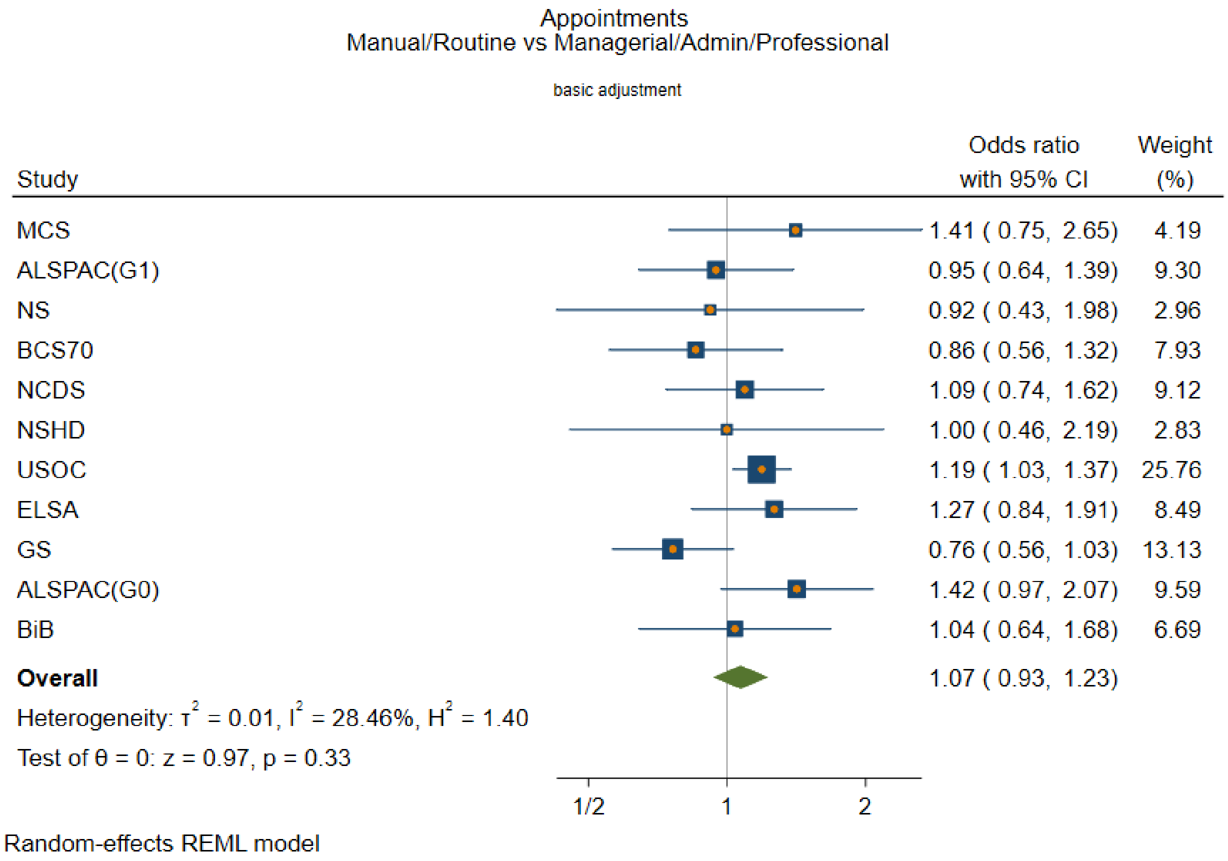
Appointments
Manual/Routine vs Managerial/Admin/Professional
basic adjustment
Study
Odds ratio with 95% CI
Weight (%)
MCS 1.41 (0.75, 2.65) 4.19
ALSPAC(G1) 0.95 (0.64, 1.39) 9.30
NS 0.92 (0.43, 1.98) 2.96
BCS70 0.86 (0.56, 1.32) 7.93
NCDS 1.09 (0.74, 1.62) 9.12
NSHD 1.00 (0.46, 2.19) 2.83
USOC 1.19 (1.03, 1.37) 25.76
ELSA 1.27 (0.84, 1.91) 8.49
GS 0.76 (0.56, 1.03) 13.13
ALSPAC(G0) 1.42 (0.97, 2.07) 9.59
BiB 1.04 (0.64, 1.68) 6.69
Overall 1.07 (0.93, 1.23)
Heterogeneity: τ² = 0.01, I² = 28.46%, H² = 1.40
Test of θ = 0: z = 0.97, p = 0.33
1/2
1
2
Random-effects REML model

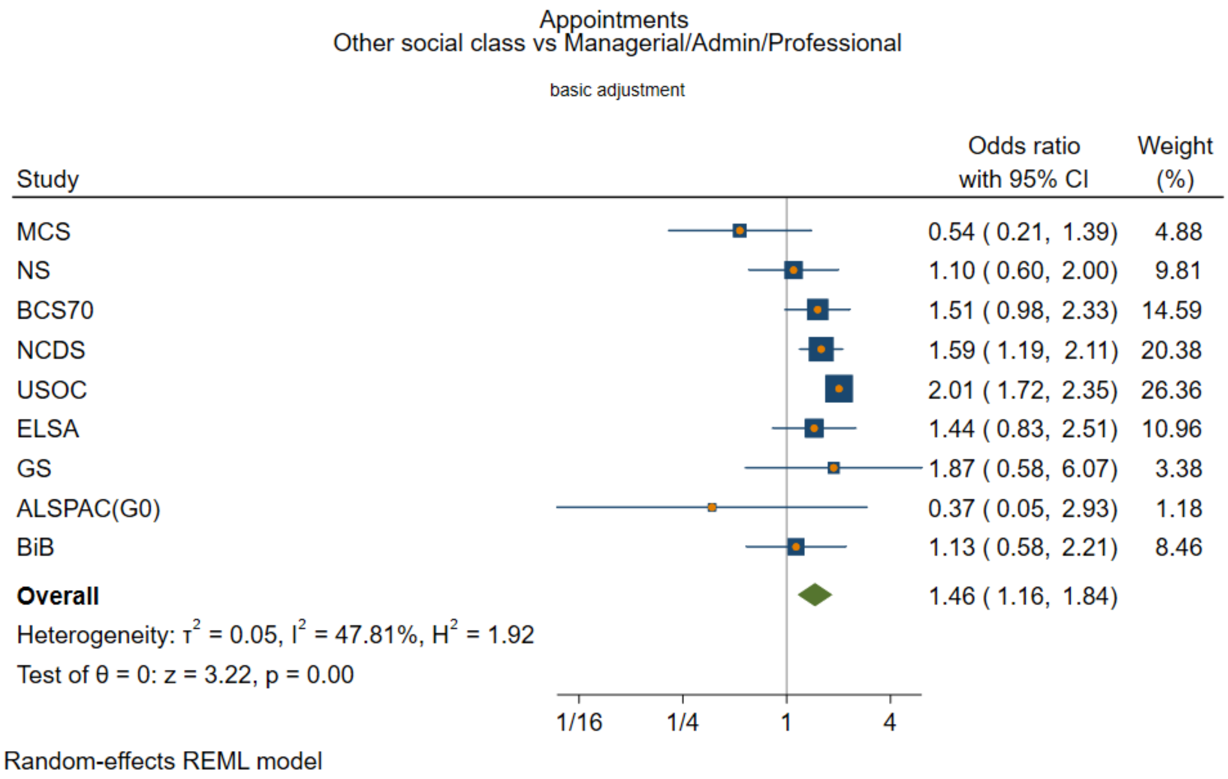
Appointments
Other social class vs Managerial/Admin/Professional
basic adjustment
Study
Odds ratio with 95% CI
Weight (%)
MCS 0.54 (0.21, 1.39) 4.88
NS 1.10 (0.60, 2.00) 9.81
BCS70 1.51 (0.98, 2.33) 14.59
NCDS 1.59 (1.19, 2.11) 20.38
USOC 2.01 (1.72, 2.35) 26.36
ELSA 1.44 (0.83, 2.51) 10.96
GS 1.87 (0.58, 6.07) 3.38
ALSPAC(G0) 0.37 (0.05, 2.93) 1.18
BiB 1.13 (0.58, 2.21) 8.46
Overall 1.46 (1.16, 1.84)
Heterogeneity: τ² = 0.05, I² = 47.81%, H² = 1.92
Test of θ = 0: z = 3.22, p = 0.00
1/16
1/4
1
4
Random-effects REML model

Full adjustment

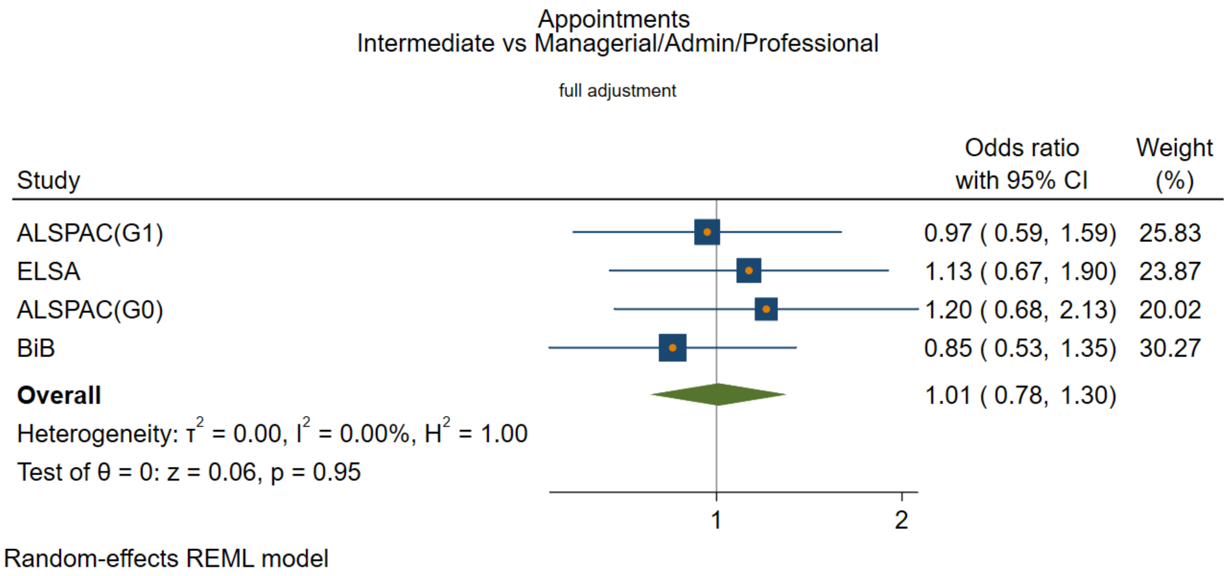

For peer review only - http://bmjopen.bmj.com/site/about/guidelines.xhtml

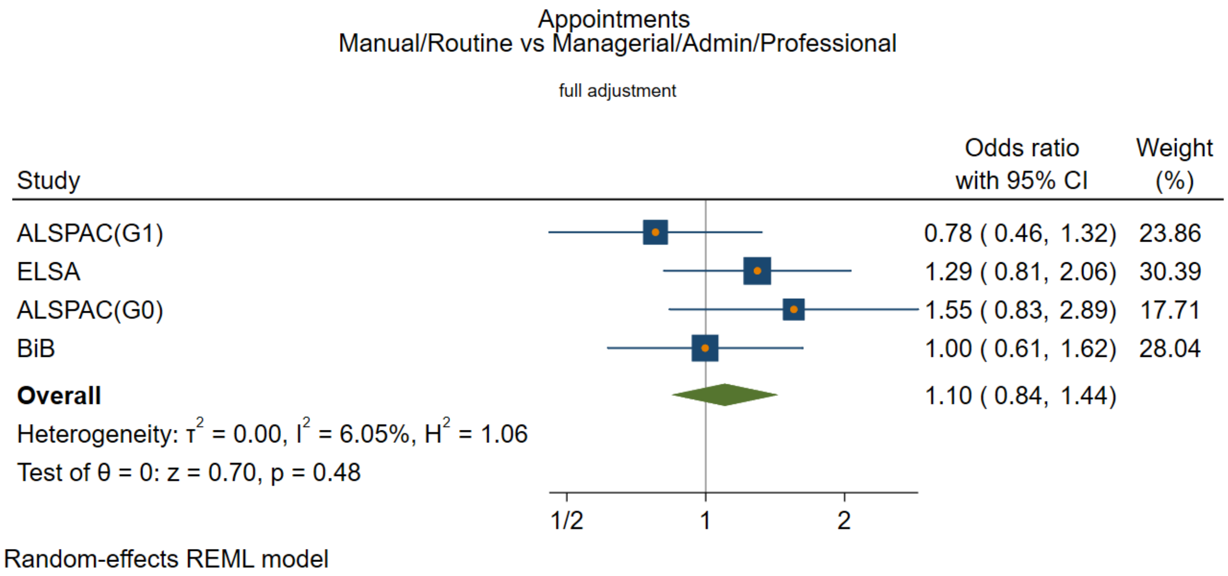

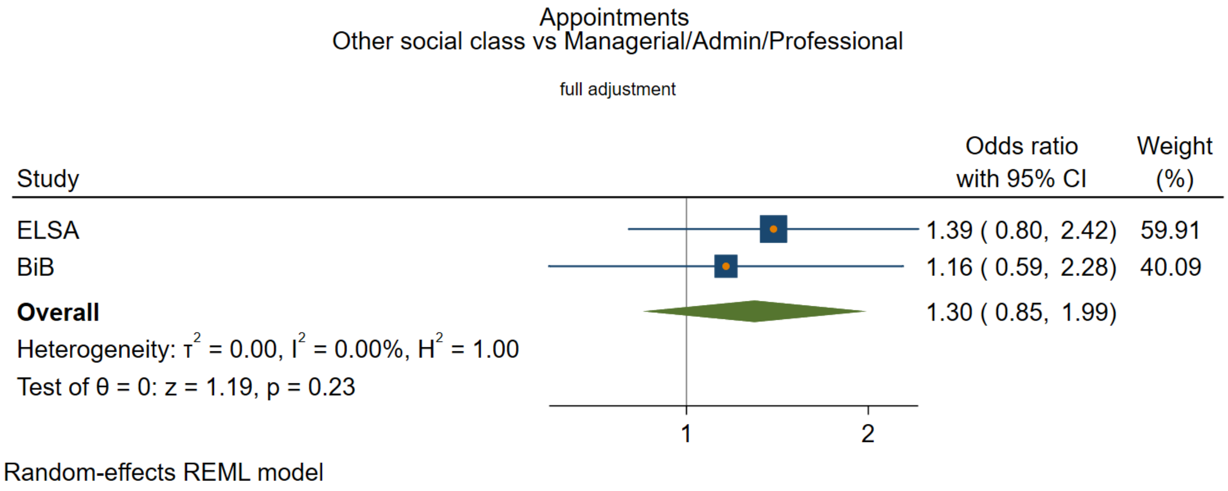

For peer review only - http://bmjopen.bmj.com/site/about/guidelines.xhtml

Education
Unadjusted

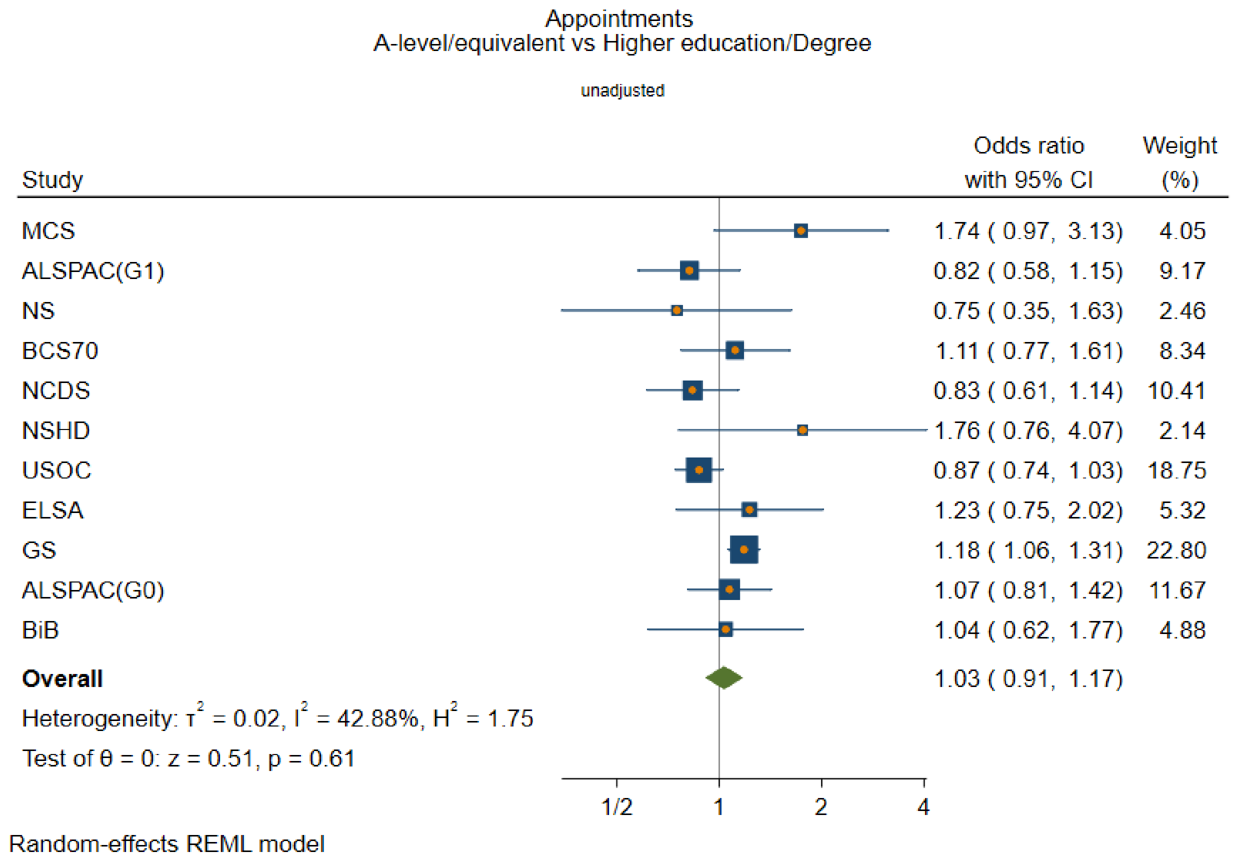

only

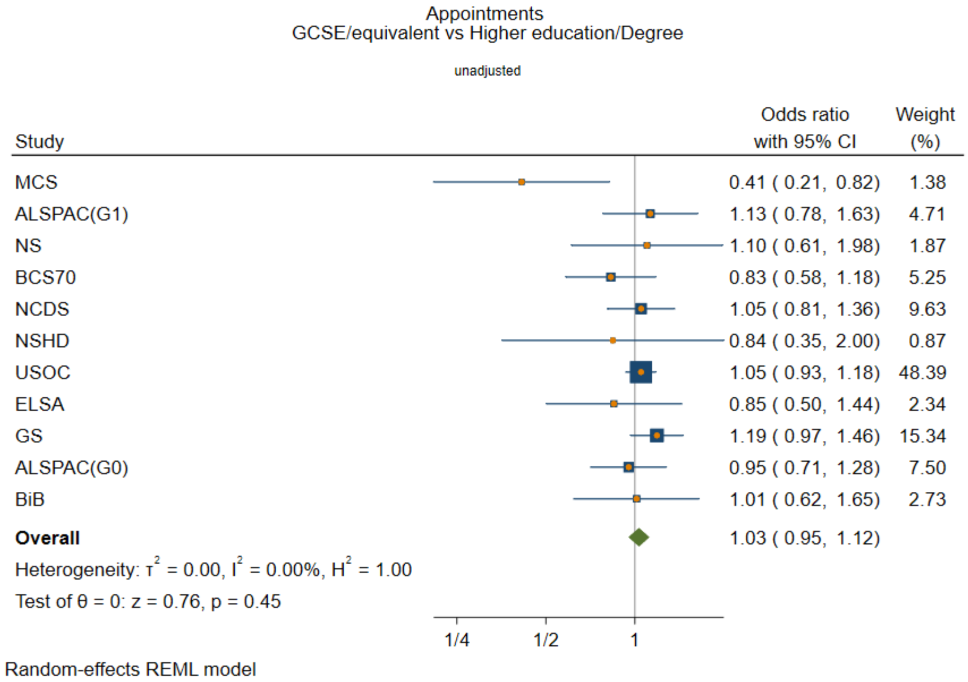

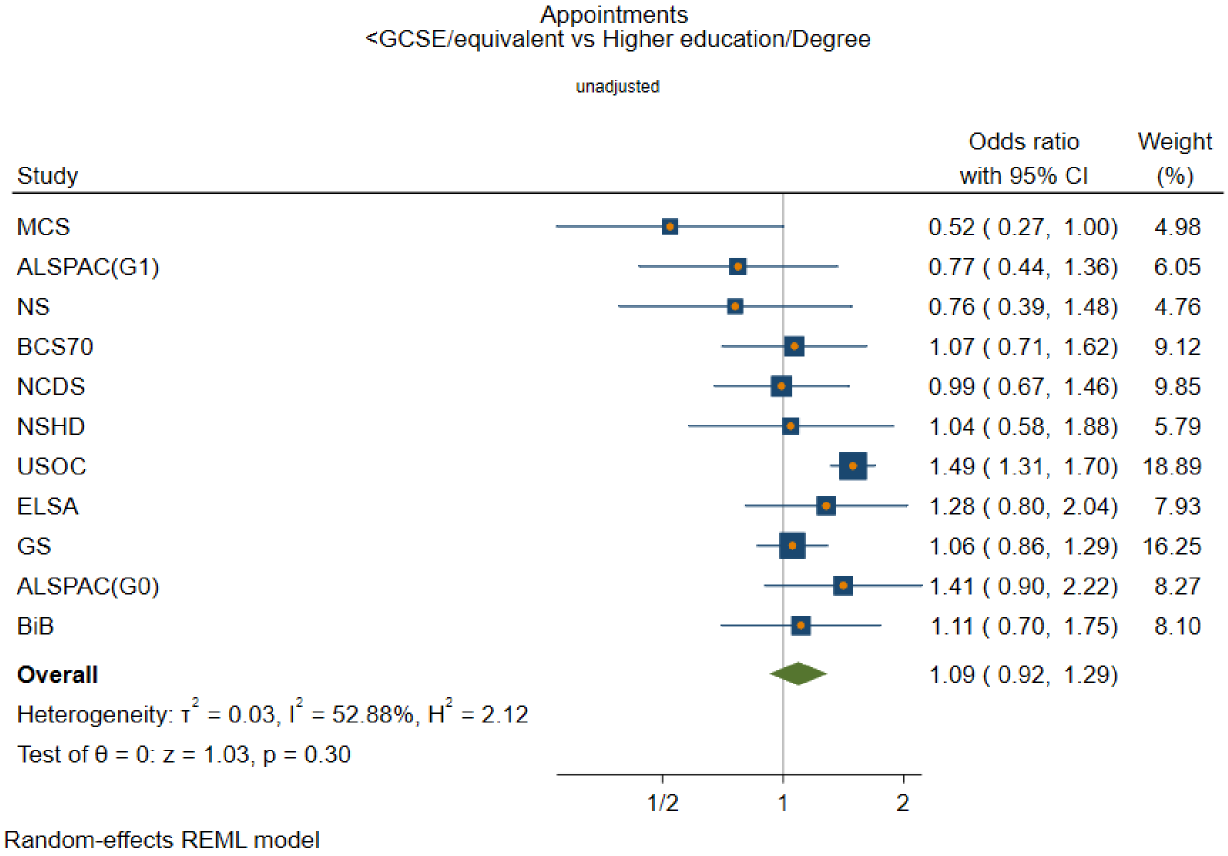

For peer review only - http://bmjopen.bmj.com/site/about/guidelines.xhtml

Basic adjustment

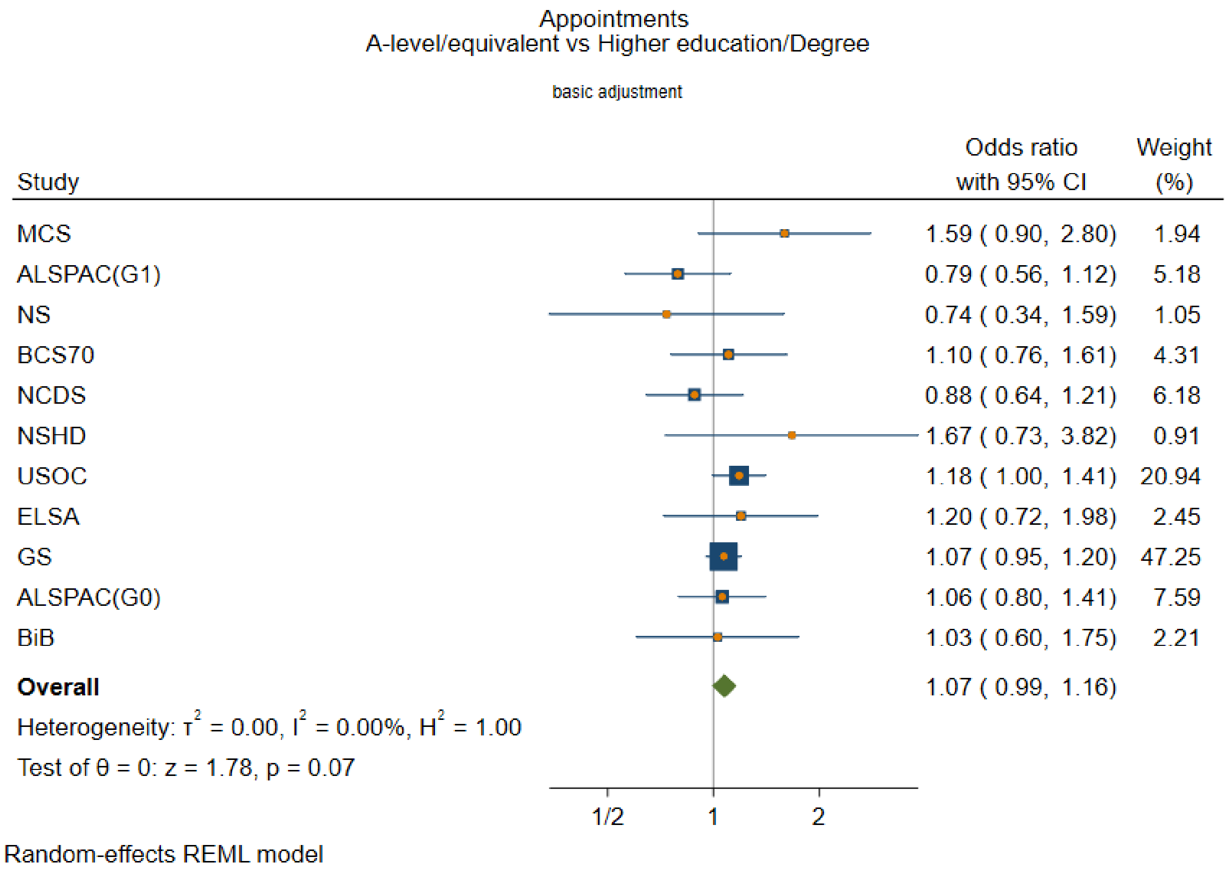

only

For peer review only - http://bmjopen.bmj.com/site/about/guidelines.xhtml

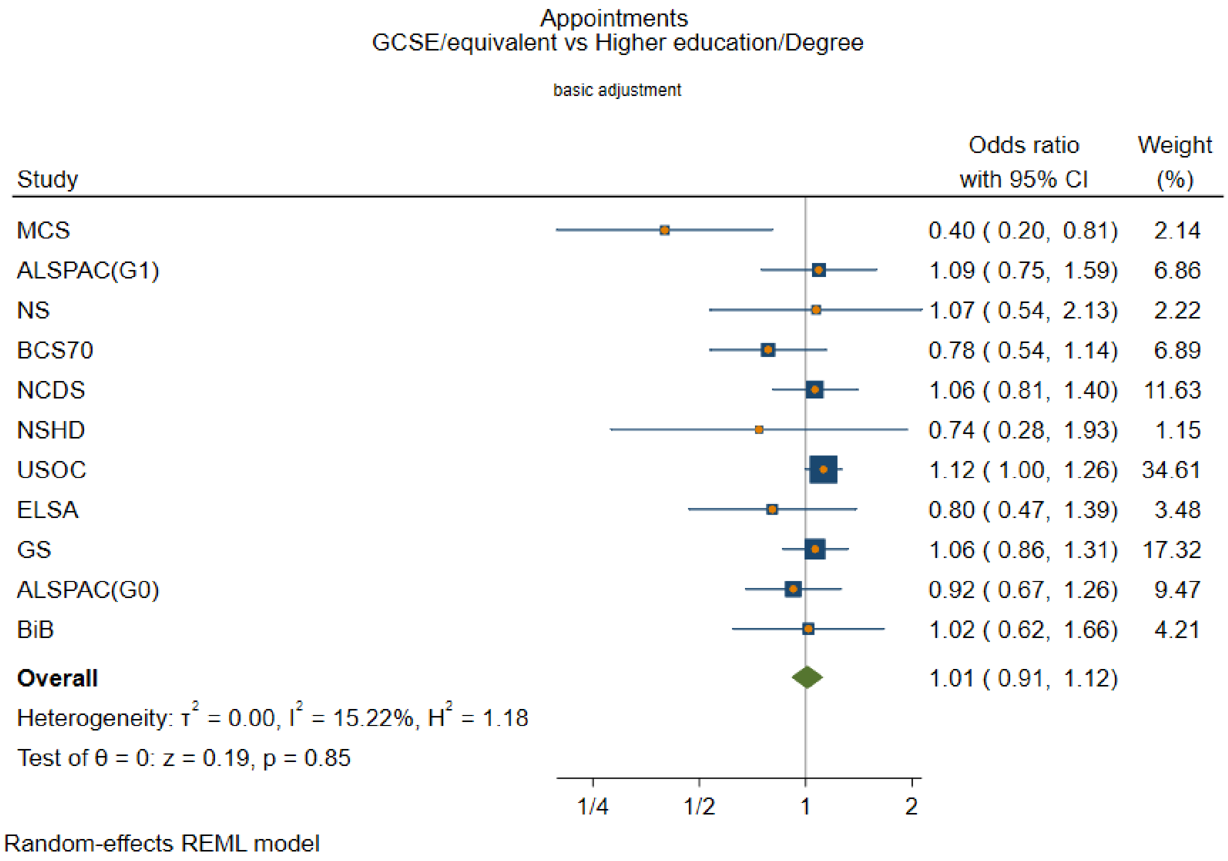

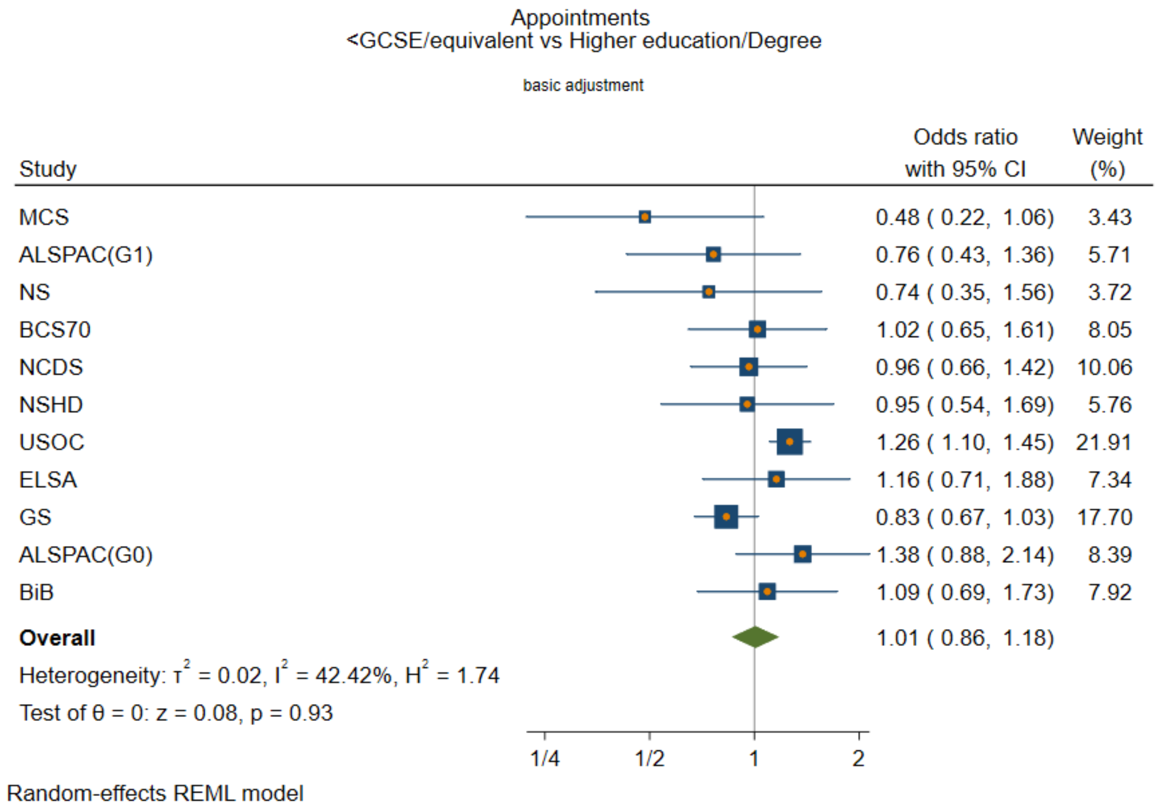

For peer review only - http://bmjopen.bmj.com/site/about/guidelines.xhtml

Full Adjustment

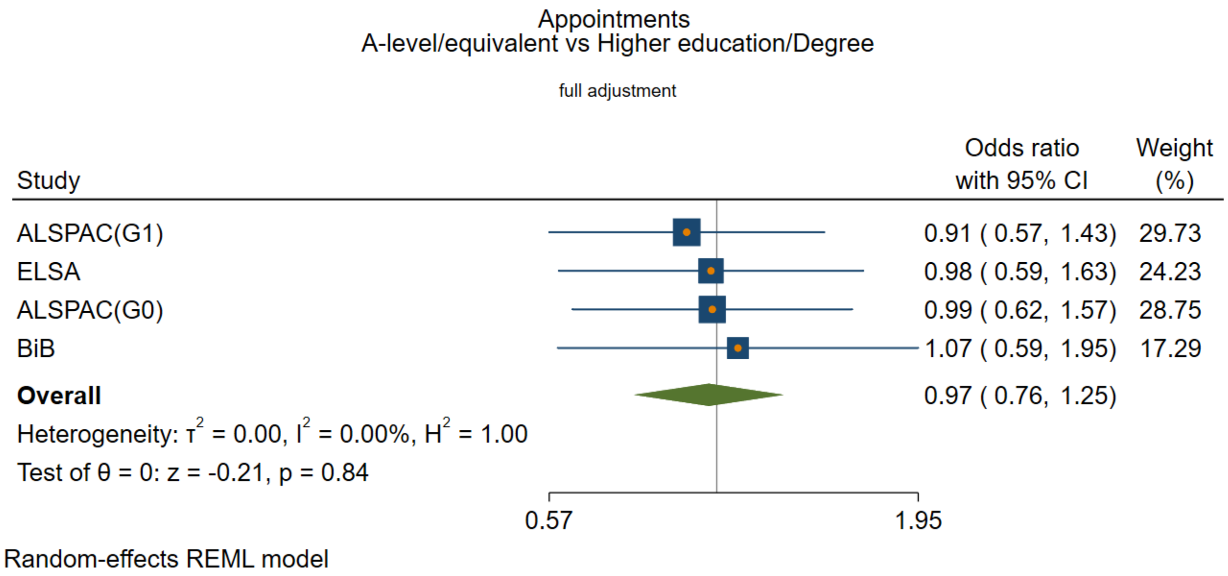

For peer review only - http://bmjopen.bmj.com/site/about/guidelines.xhtml

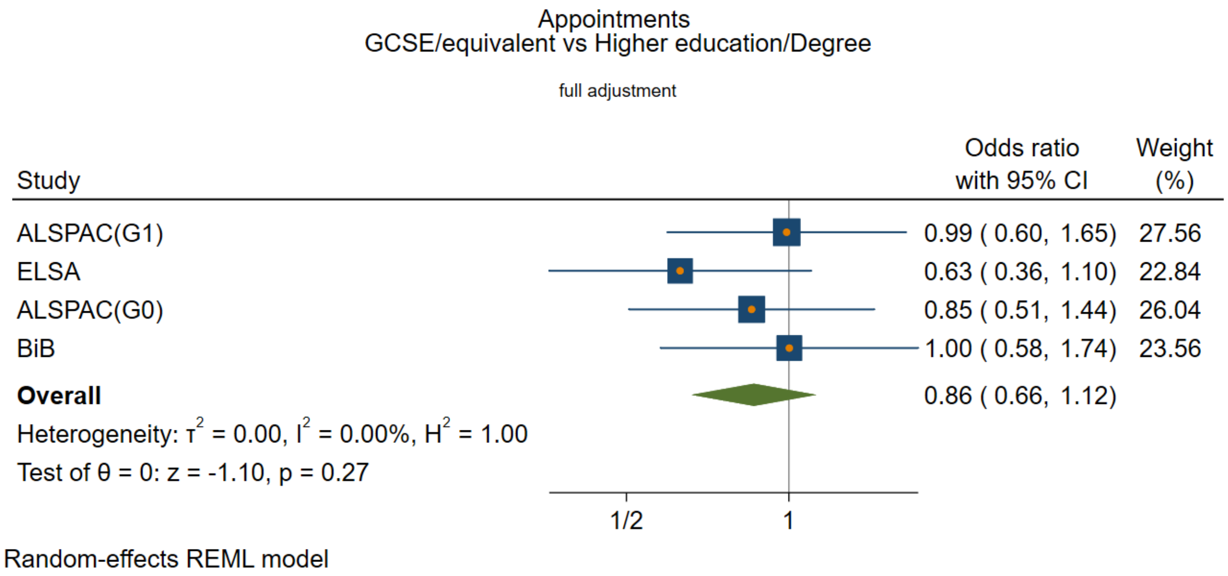
Appointments
GCSE/equivalent vs Higher education/Degree
full adjustment
Study
Odds ratio with 95% CI
Weight (%)
ALSPAC(G1) 0.99 (0.60, 1.65) 27.56
ELSA 0.63 (0.36, 1.10) 22.84
ALSPAC(G0) 0.85 (0.51, 1.44) 26.04
BiB 1.00 (0.58, 1.74) 23.56
Overall 0.86 (0.66, 1.12)
Heterogeneity: τ^2 = 0.00, I^2 = 0.00%, H^2 = 1.00
Test of θ = 0: z = -1.10, p = 0.27
1/2
1
Random-effects REML model

peer

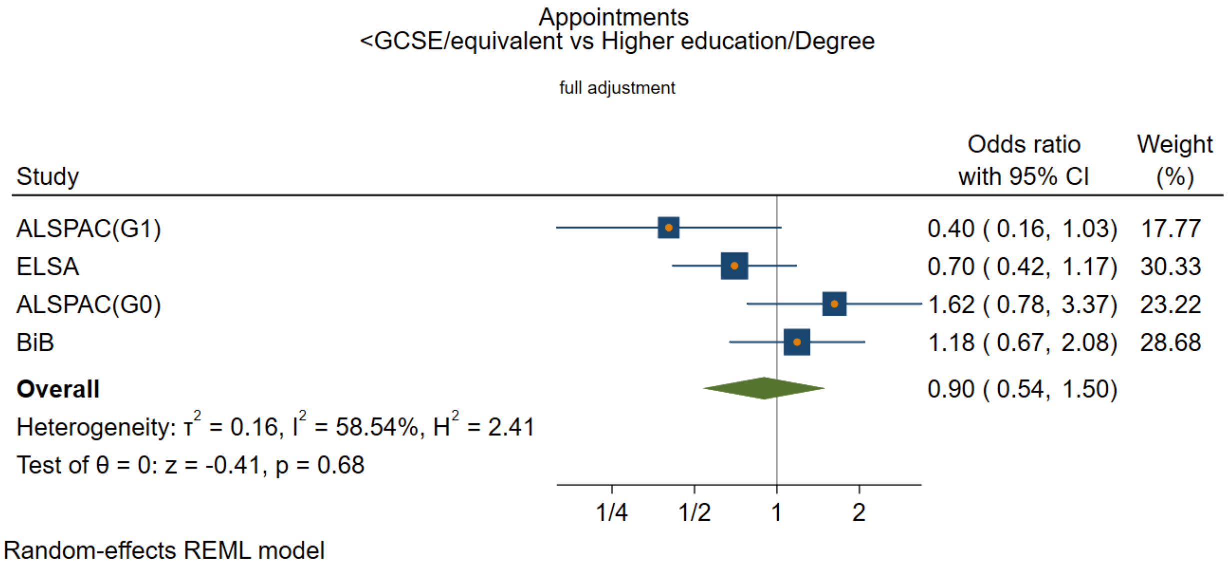
Appointments
<GCSE/equivalent vs Higher education/Degree
full adjustment
Study
Odds ratio with 95% CI
Weight (%)
ALSPAC(G1) 0.40 (0.16, 1.03) 17.77
ELSA 0.70 (0.42, 1.17) 30.33
ALSPAC(G0) 1.62 (0.78, 3.37) 23.22
BiB 1.18 (0.67, 2.08) 28.68
Overall 0.90 (0.54, 1.50)
Heterogeneity: τ^2 = 0.16, I^2 = 58.54%, H^2 = 2.41
Test of θ = 0: z = -0.41, p = 0.68
1/4
1/2
1
2
Random-effects REML model

Age

<u>Unadjusted</u>

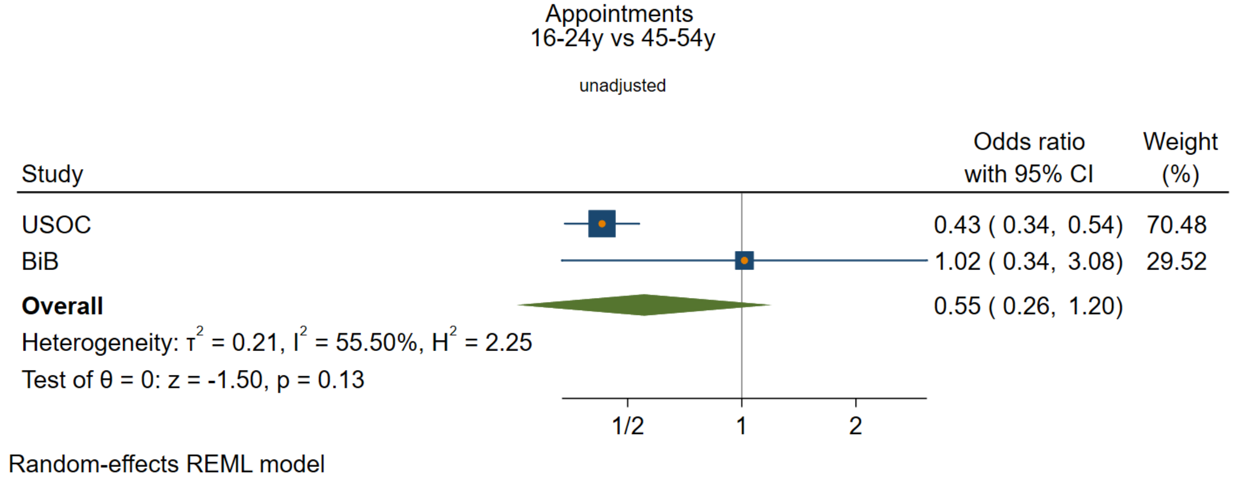

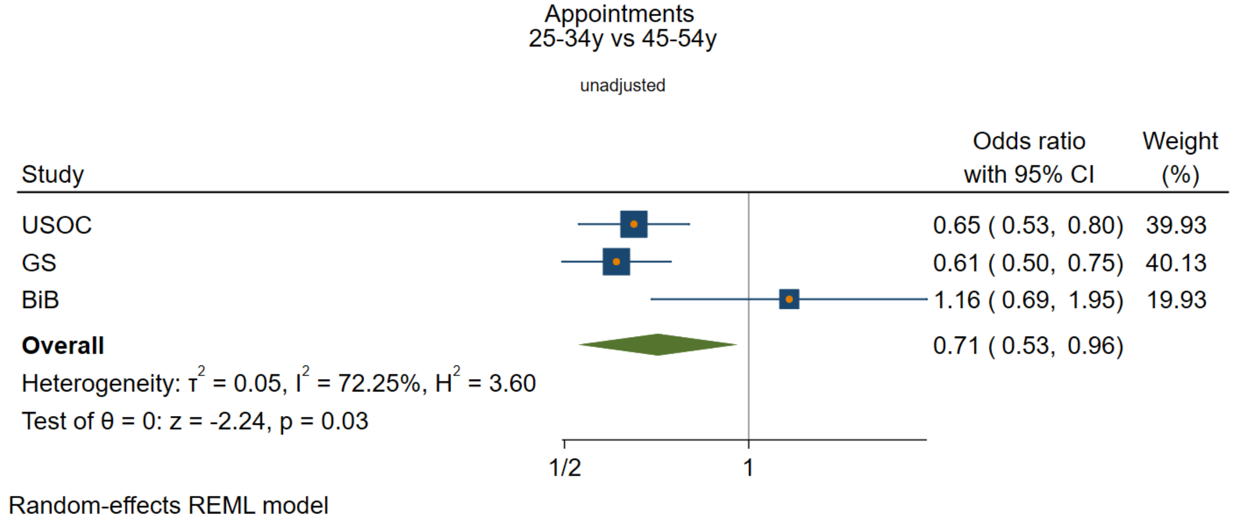

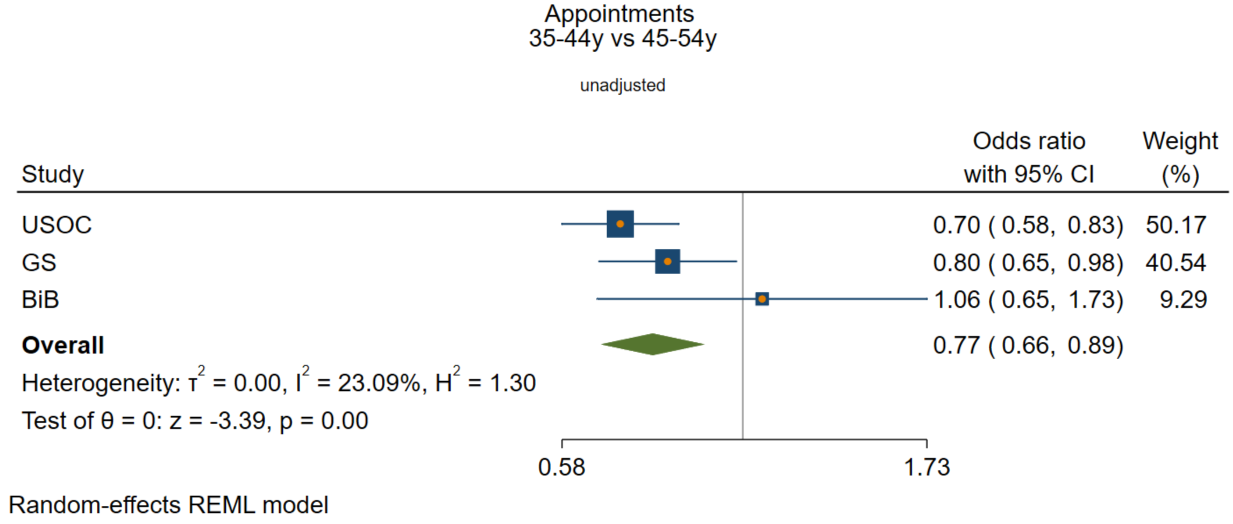

For peer review only - http://bmjopen.bmj.com/site/about/guidelines.xhtml

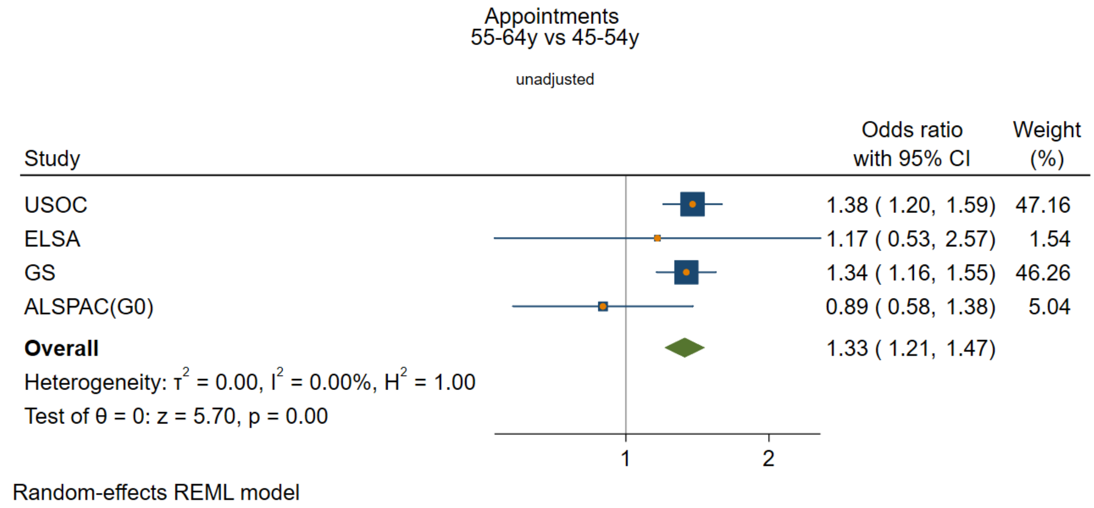
Appointments
55-64y vs 45-54y
unadjusted
Study
Odds ratio with 95% CI
Weight (%)
USOC 1.38 (1.20, 1.59) 47.16
ELSA 1.17 (0.53, 2.57) 1.54
GS 1.34 (1.16, 1.55) 46.26
ALSPAC(G0) 0.89 (0.58, 1.38) 5.04
Overall 1.33 (1.21, 1.47)
Heterogeneity: τ² = 0.00, I² = 0.00%, H² = 1.00
Test of θ = 0: z = 5.70, p = 0.00
1
2
Random-effects REML model

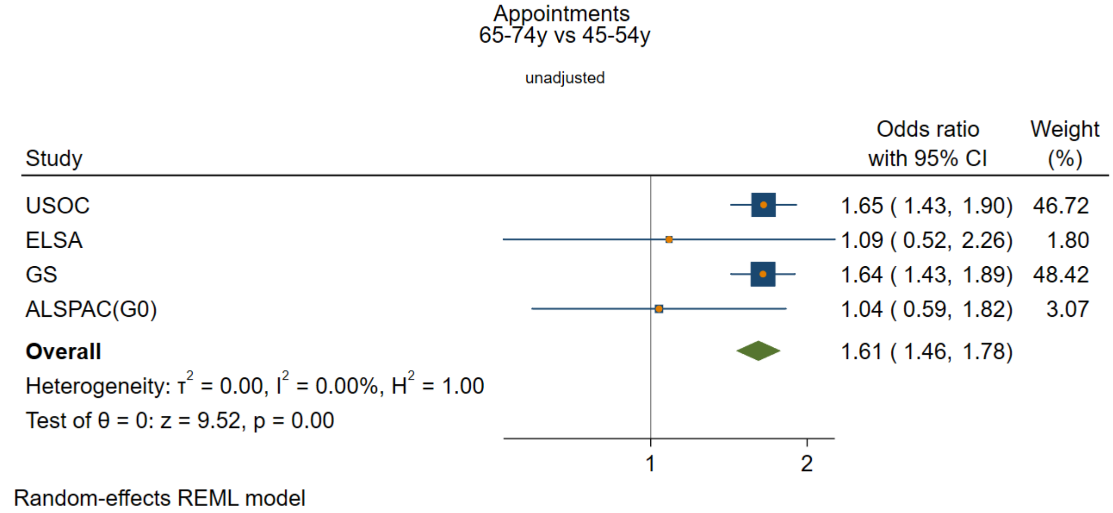
Appointments
65-74y vs 45-54y
unadjusted
Study
Odds ratio with 95% CI
Weight (%)
USOC 1.65 (1.43, 1.90) 46.72
ELSA 1.09 (0.52, 2.26) 1.80
GS 1.64 (1.43, 1.89) 48.42
ALSPAC(G0) 1.04 (0.59, 1.82) 3.07
Overall 1.61 (1.46, 1.78)
Heterogeneity: τ² = 0.00, I² = 0.00%, H² = 1.00
Test of θ = 0: z = 9.52, p = 0.00
1
2
Random-effects REML model

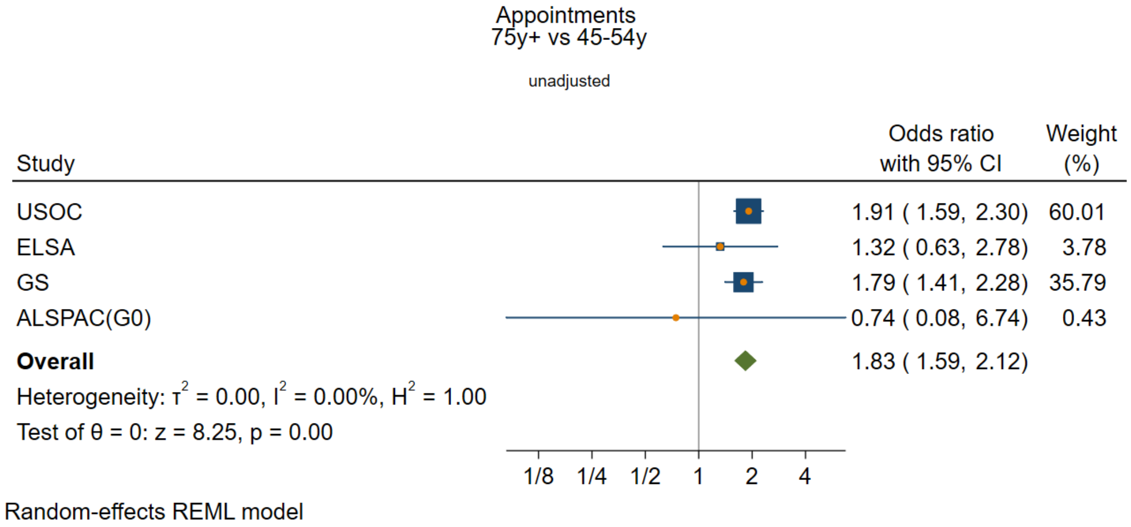
Appointments
75y+ vs 45-54y
unadjusted
Study
Odds ratio with 95% CI
Weight (%)
USOC 1.91 (1.59, 2.30) 60.01
ELSA 1.32 (0.63, 2.78) 3.78
GS 1.79 (1.41, 2.28) 35.79
ALSPAC(G0) 0.74 (0.08, 6.74) 0.43
Overall 1.83 (1.59, 2.12)
Heterogeneity: τ² = 0.00, I² = 0.00%, H² = 1.00
Test of θ = 0: z = 8.25, p = 0.00
1/8
1/4
1/2
1
2
4
Random-effects REML model

Basic adjustment

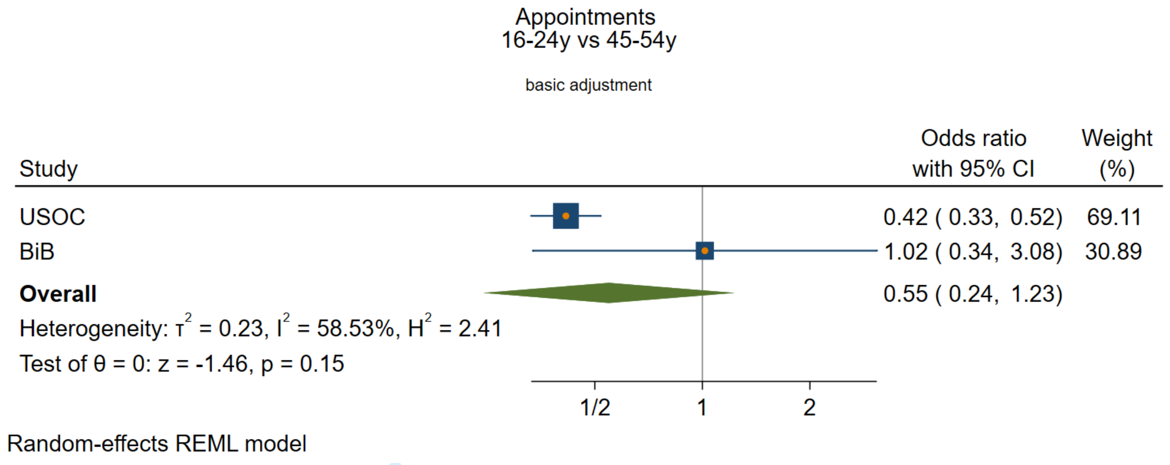

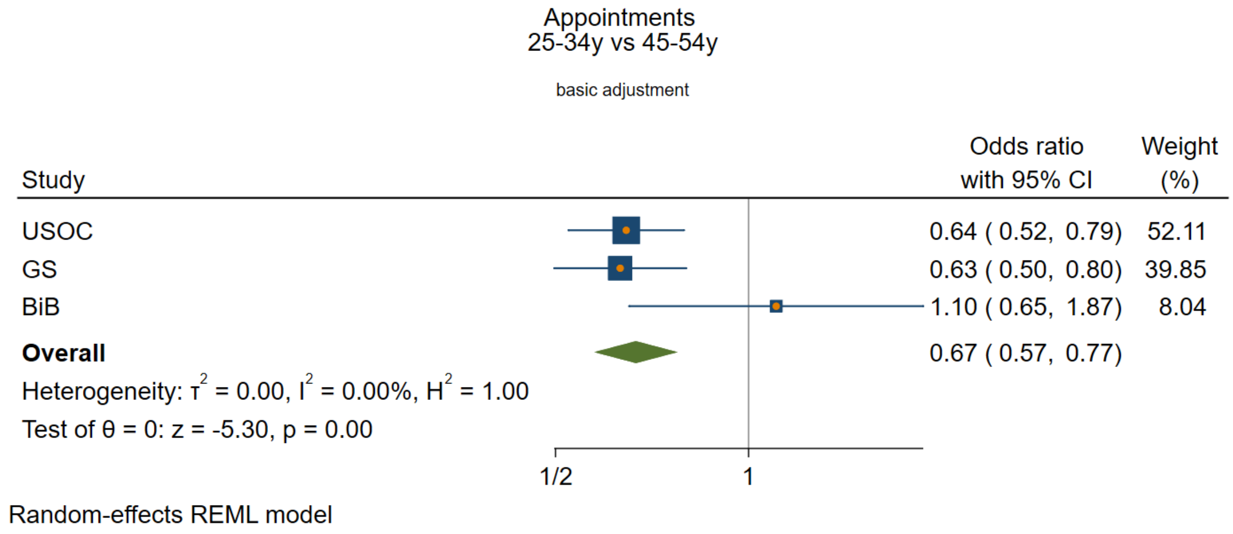

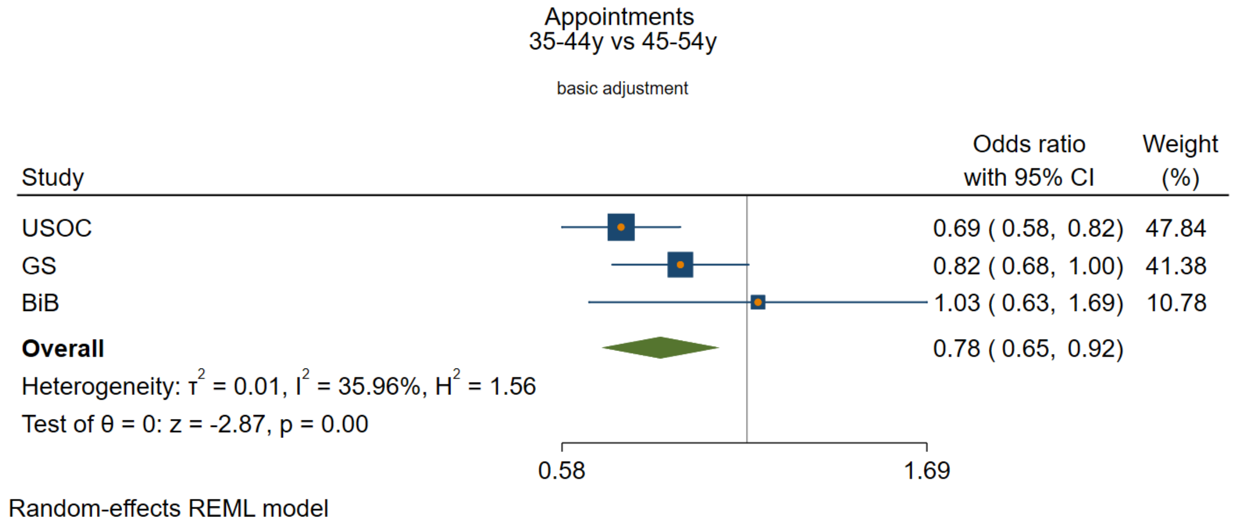

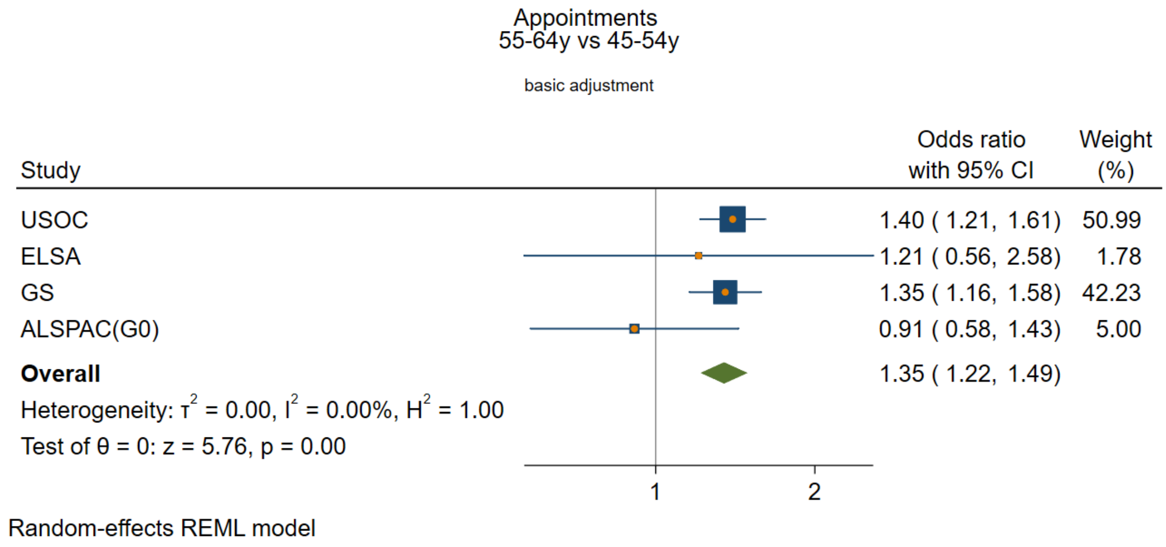
Appointments
55-64y vs 45-54y
basic adjustment
Study
Odds ratio with 95% CI
Weight (%)
USOC
1.40 (1.21, 1.61)
50.99
ELSA
1.21 (0.56, 2.58)
1.78
GS
1.35 (1.16, 1.58)
42.23
ALSPAC(G0)
0.91 (0.58, 1.43)
5.00
Overall
1.35 (1.22, 1.49)
Heterogeneity: τ² = 0.00, I² = 0.00%, H² = 1.00
Test of θ = 0: z = 5.76, p = 0.00
1
2
Random-effects REML model

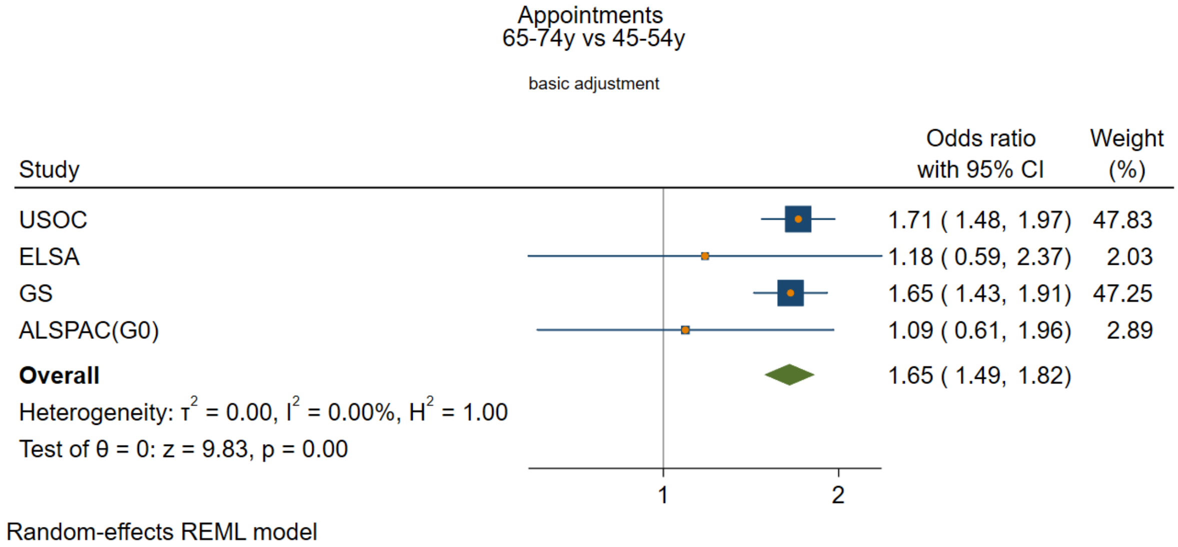
Appointments
65-74y vs 45-54y
basic adjustment
Study
Odds ratio with 95% CI
Weight (%)
USOC
1.71 (1.48, 1.97)
47.83
ELSA
1.18 (0.59, 2.37)
2.03
GS
1.65 (1.43, 1.91)
47.25
ALSPAC(G0)
1.09 (0.61, 1.96)
2.89
Overall
1.65 (1.49, 1.82)
Heterogeneity: τ² = 0.00, I² = 0.00%, H² = 1.00
Test of θ = 0: z = 9.83, p = 0.00
1
2
Random-effects REML model

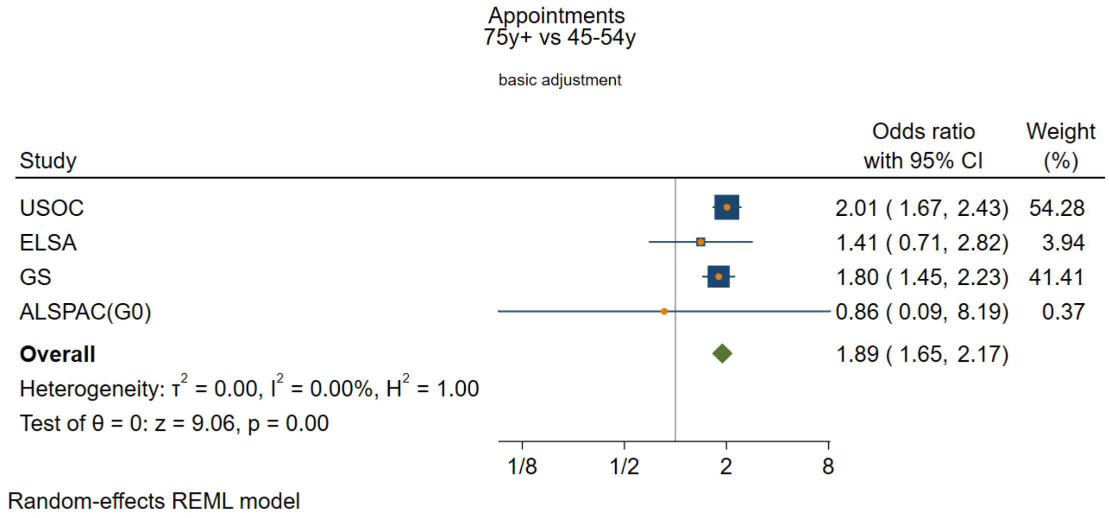
Appointments
75y+ vs 45-54y
basic adjustment
Study
Odds ratio with 95% CI
Weight (%)
USOC
2.01 (1.67, 2.43)
54.28
ELSA
1.41 (0.71, 2.82)
3.94
GS
1.80 (1.45, 2.23)
41.41
ALSPAC(G0)
0.86 (0.09, 8.19)
0.37
Overall
1.89 (1.65, 2.17)
Heterogeneity: τ² = 0.00, I² = 0.00%, H² = 1.00
Test of θ = 0: z = 9.06, p = 0.00
1/8
1/2
2
8
Random-effects REML model

Full adjustment

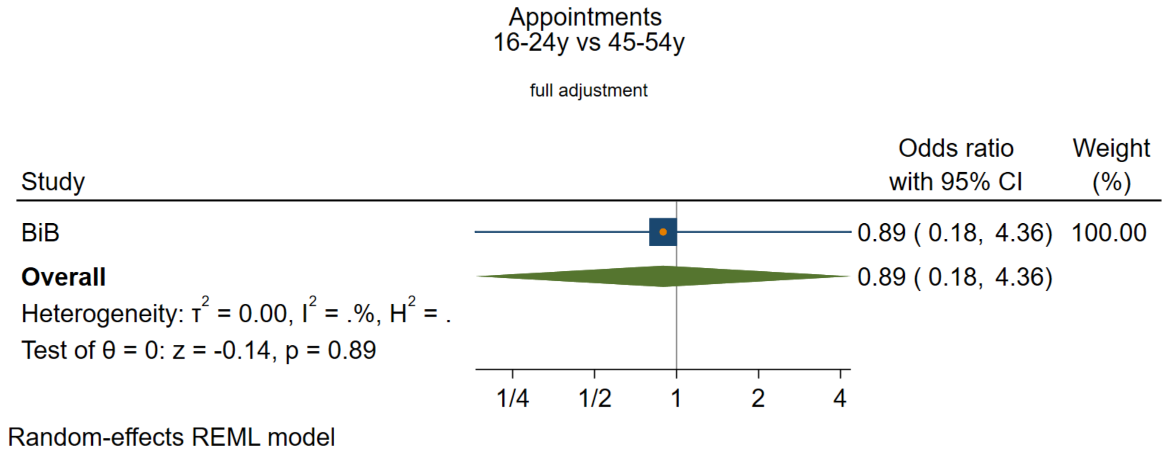

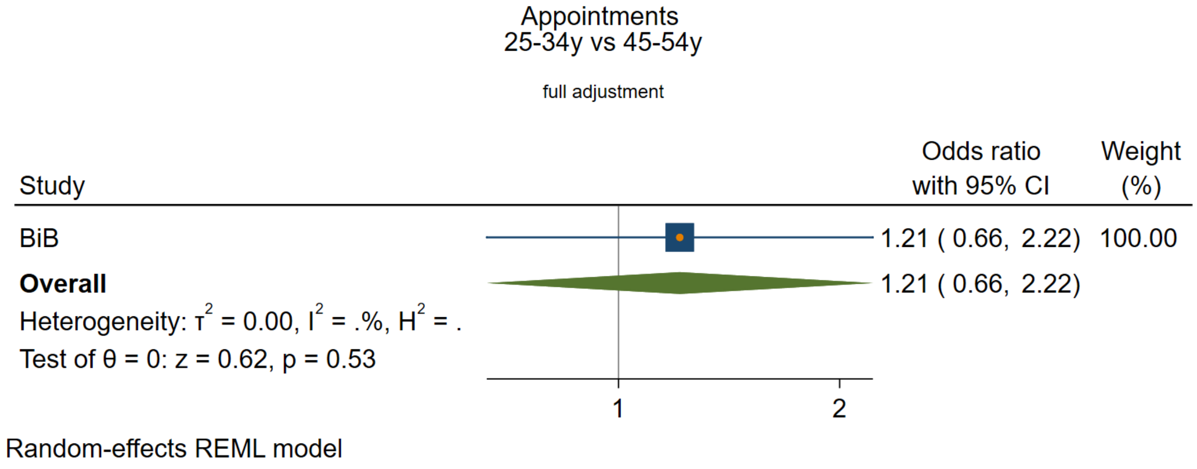

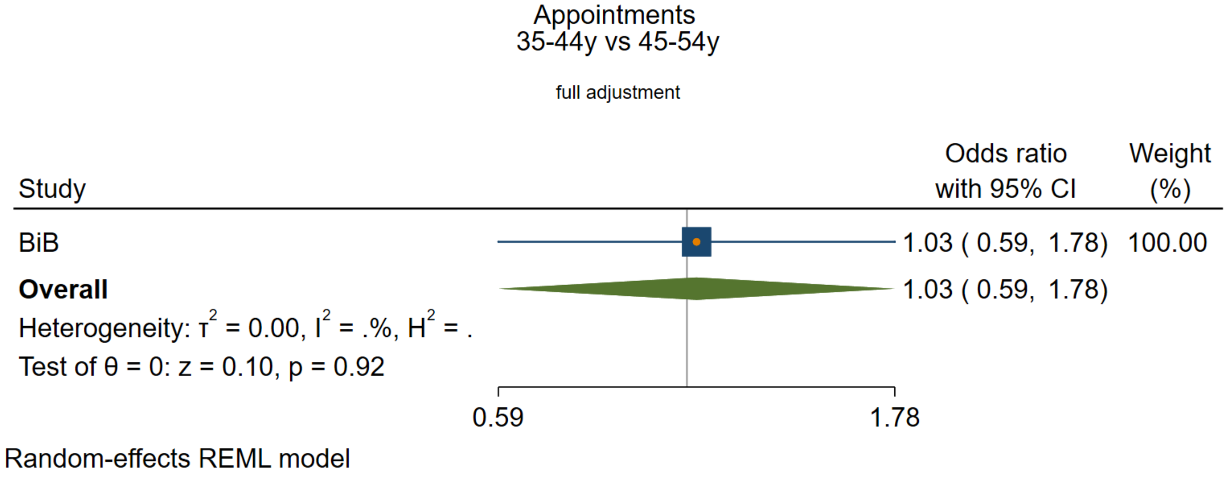

For peer review only - http://bmjopen.bmj.com/site/about/guidelines.xhtml

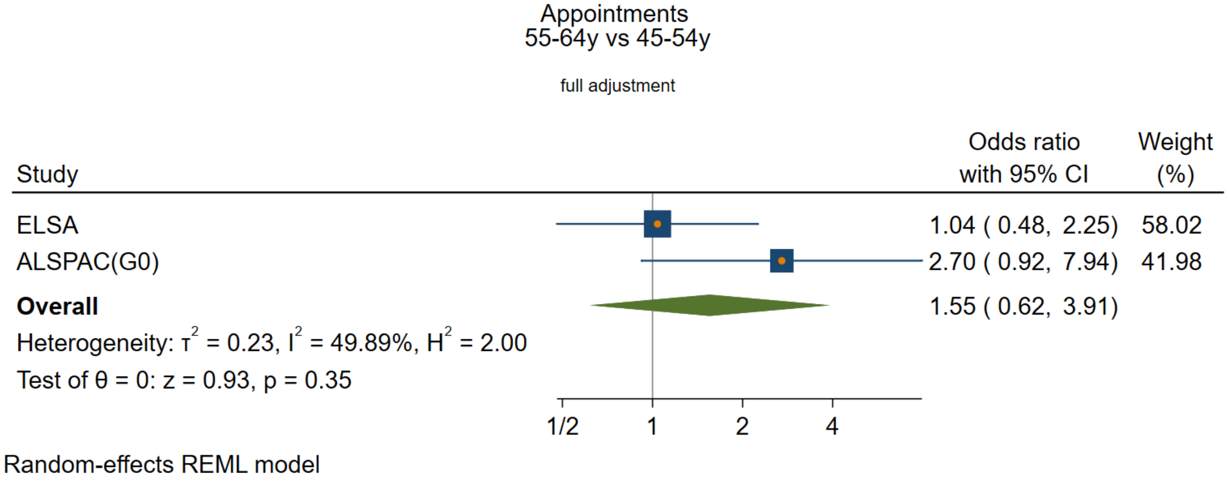

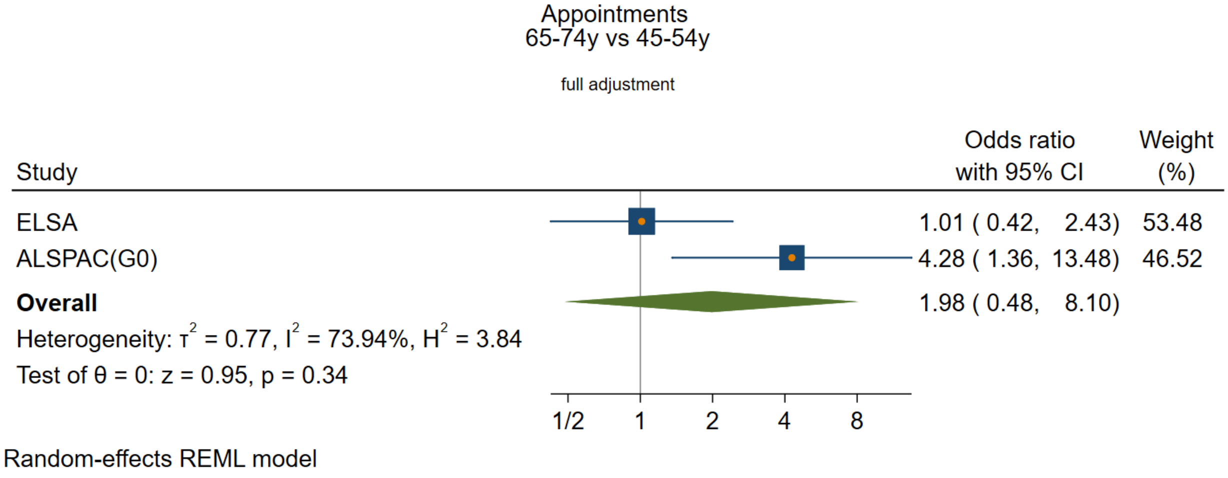

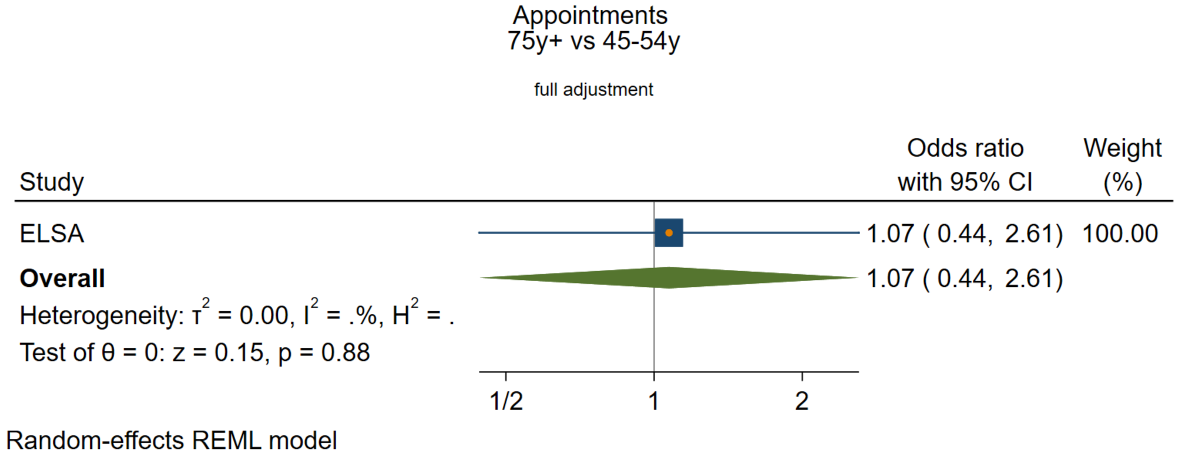

For peer review only - http://bmjopen.bmj.com/site/about/guidelines.xhtml

Ethnicity
Unadjusted

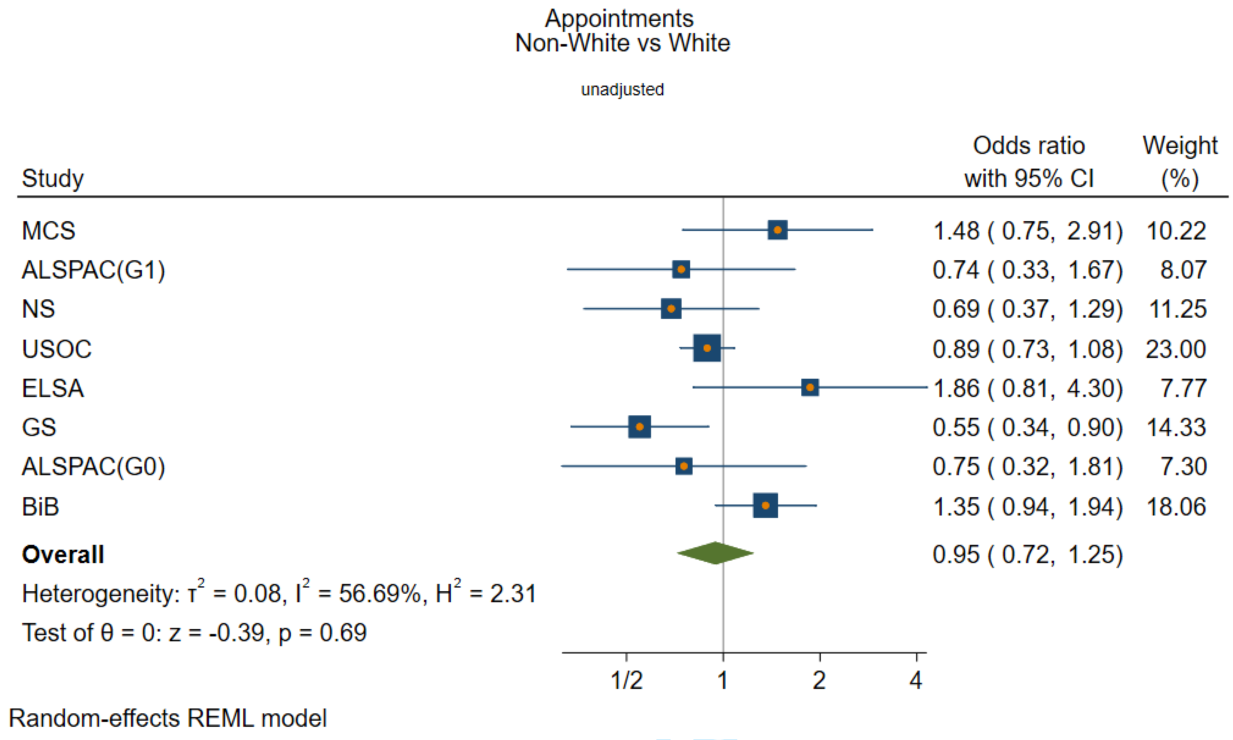

For peer review only - http://bmjopen.bmj.com/site/about/guidelines.xhtml

Appointments
Black vs White

unadjusted

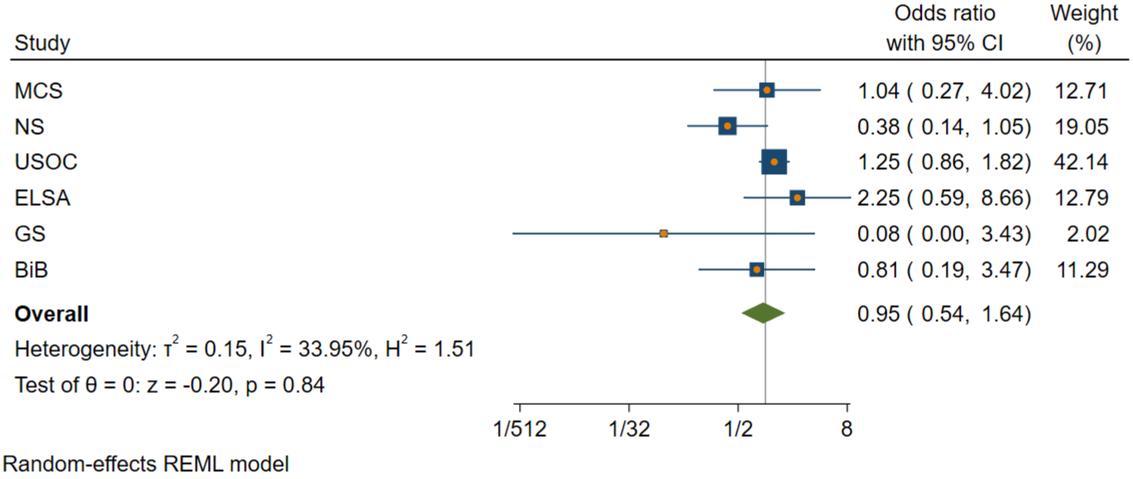

Appointments
South Asian vs White

unadjusted

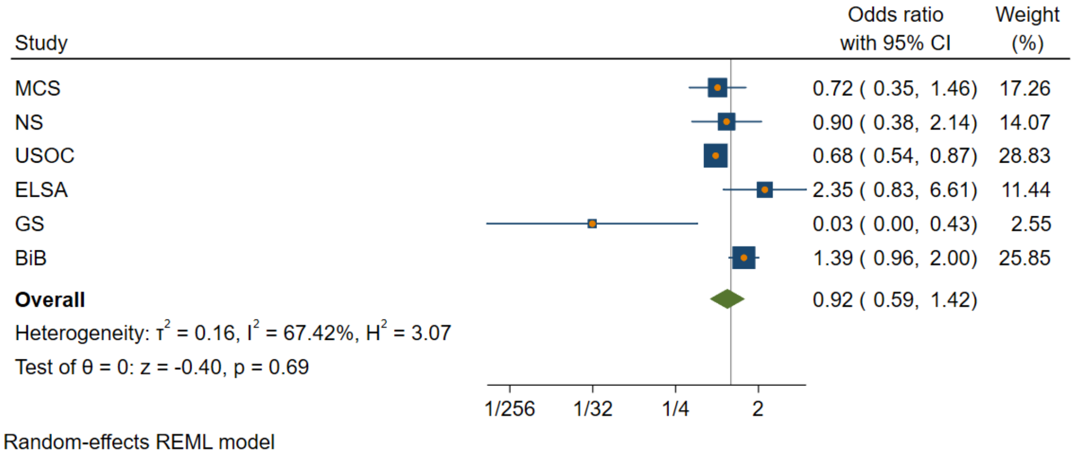

Appointments
East Asian vs White

unadjusted

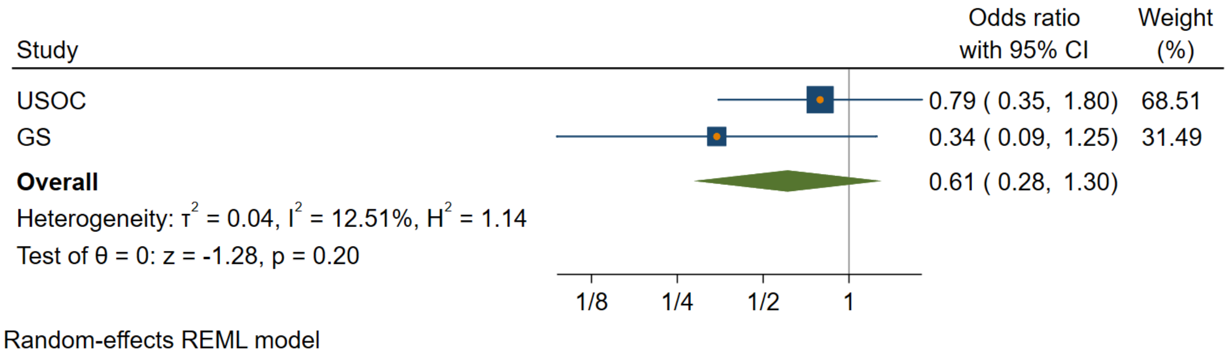

For peer review only - http://bmjopen.bmj.com/site/about/guidelines.xhtml

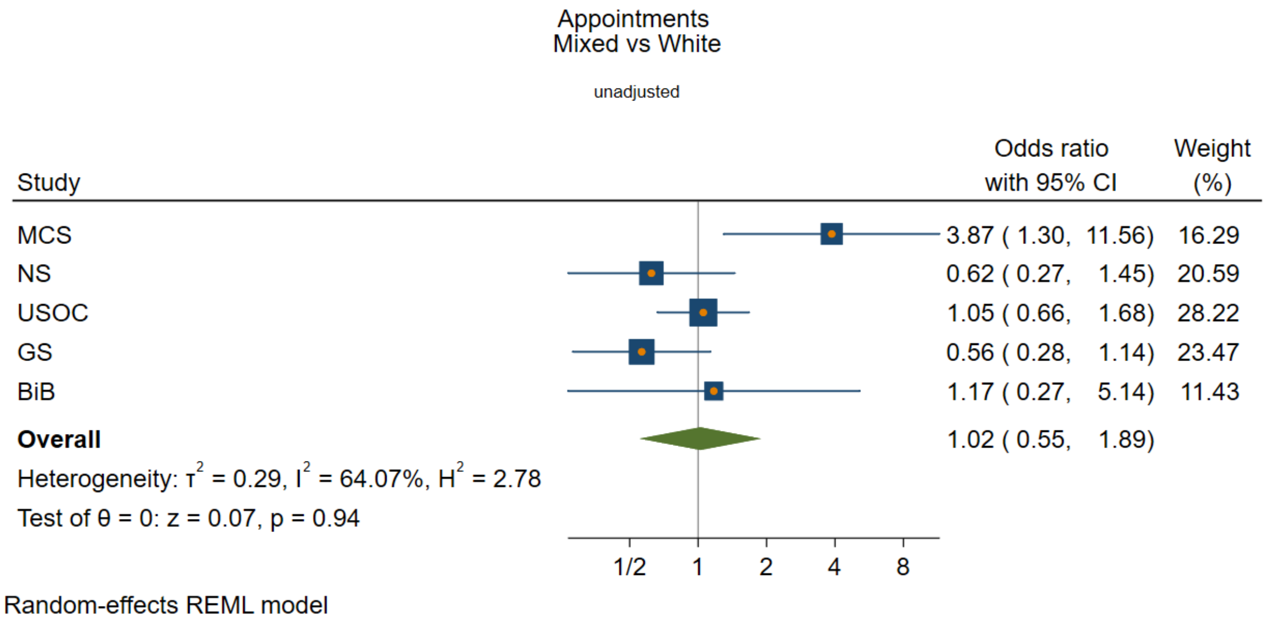

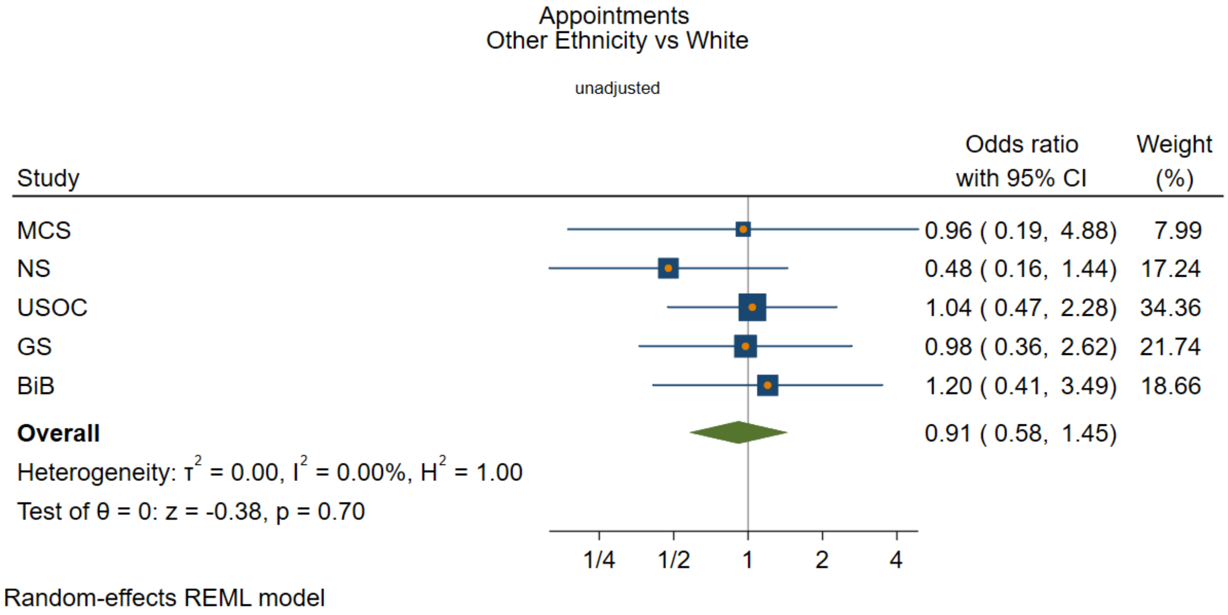

For peer review only - http://bmjopen.bmj.com/site/about/guidelines.xhtml

Basic adjustment

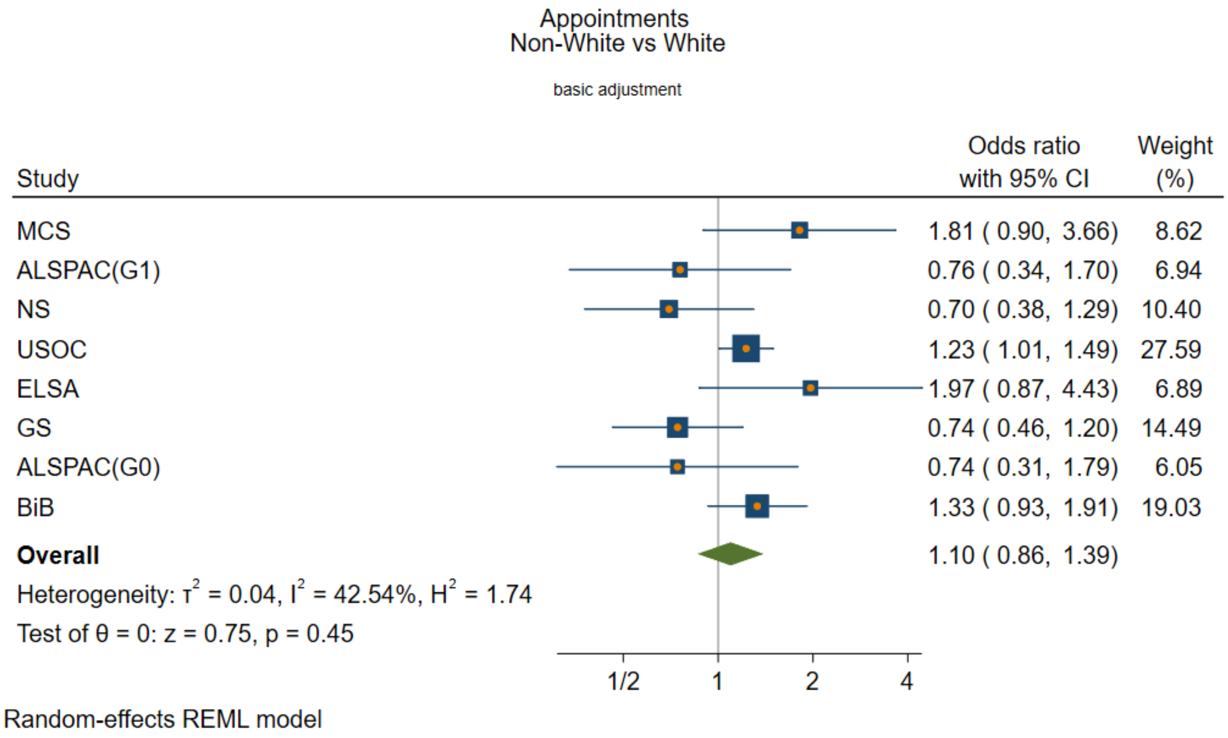

iew only

For peer review only - http://bmjopen.bmj.com/site/about/guidelines.xhtml

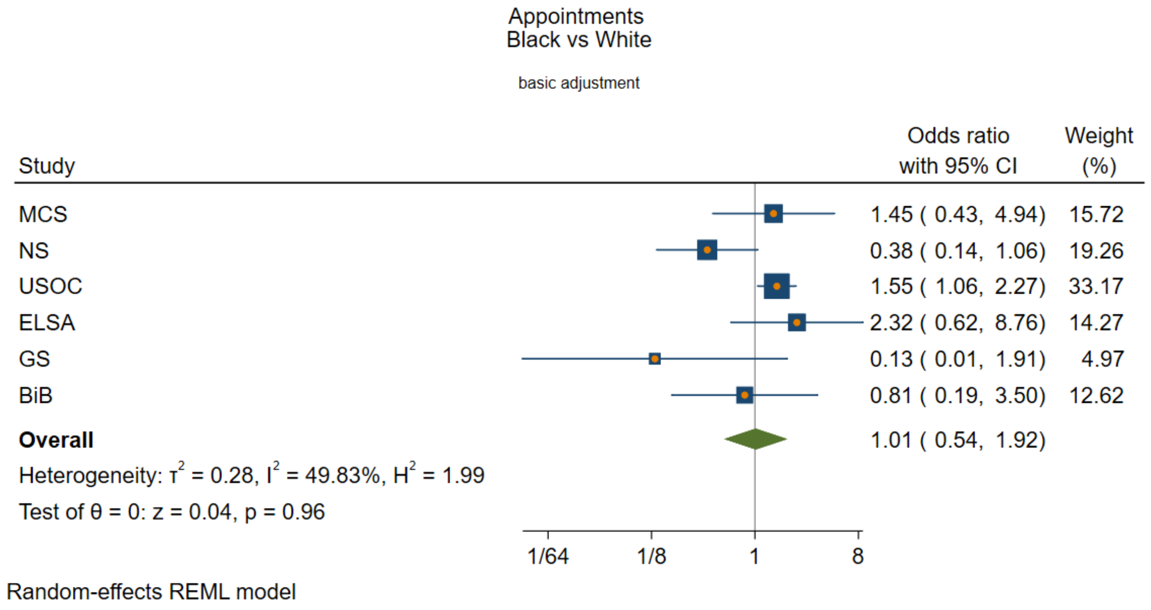

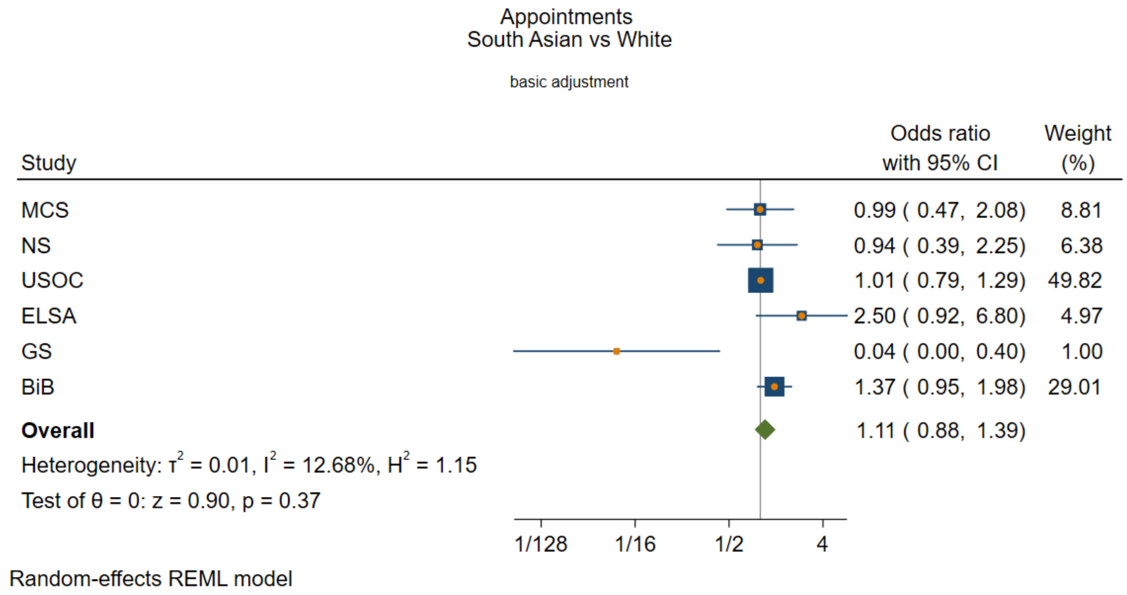

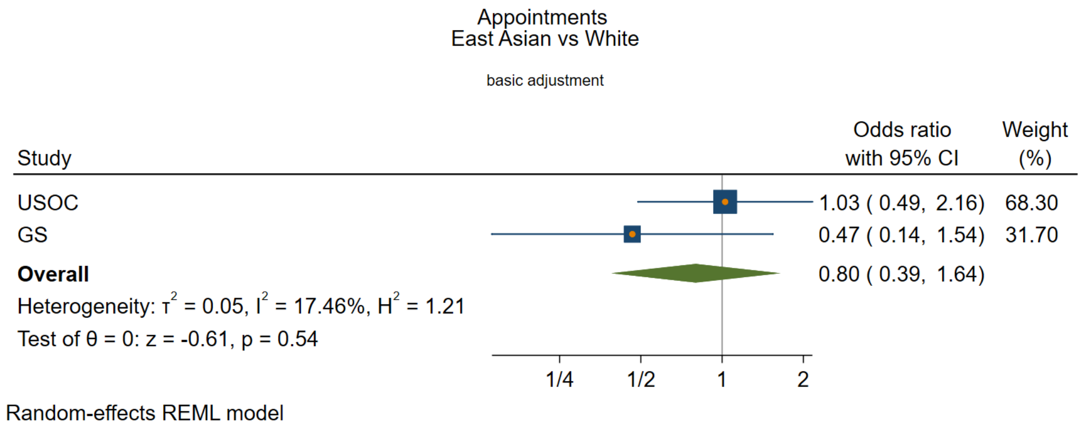

For peer review only - http://bmjopen.bmj.com/site/about/guidelines.xhtml

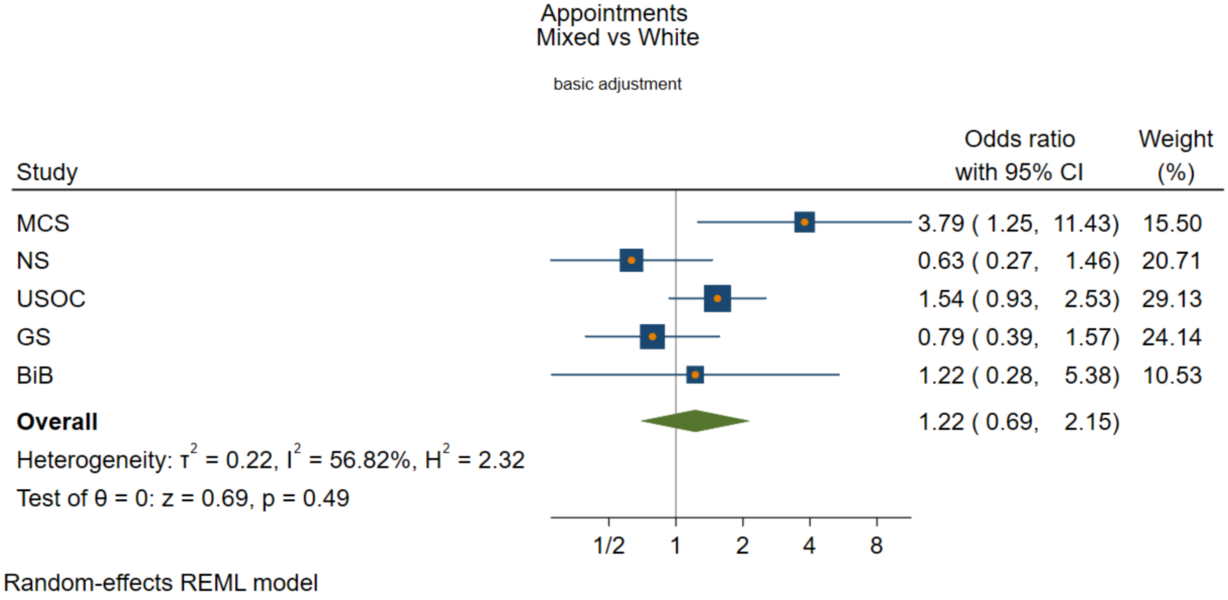

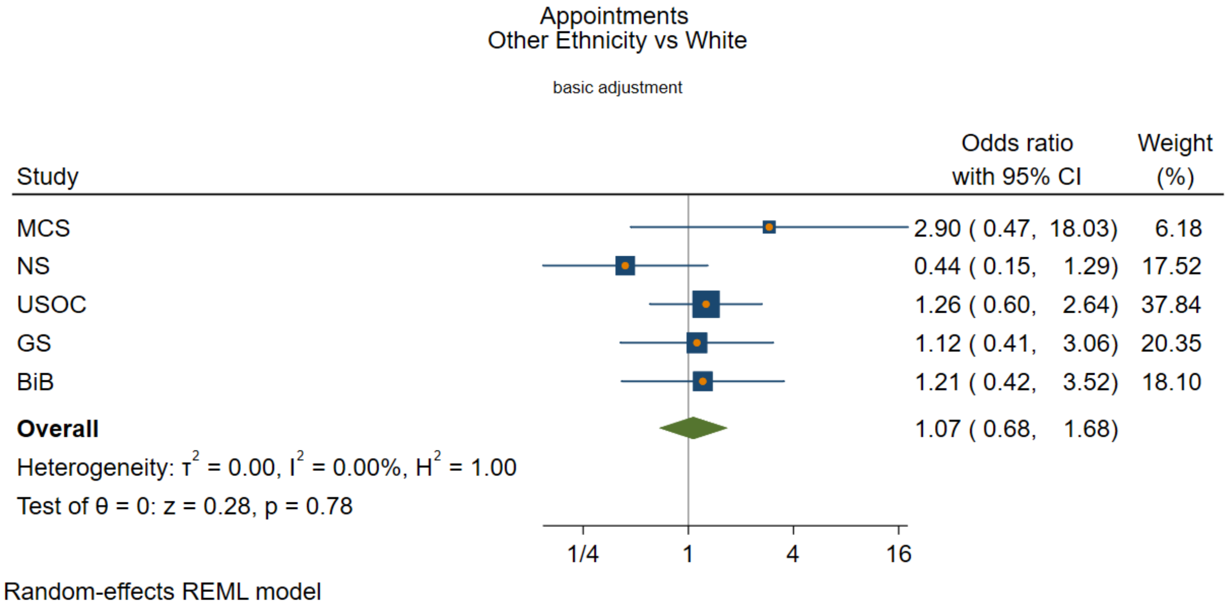

For peer review only - http://bmjopen.bmj.com/site/about/guidelines.xhtml

Full adjustment

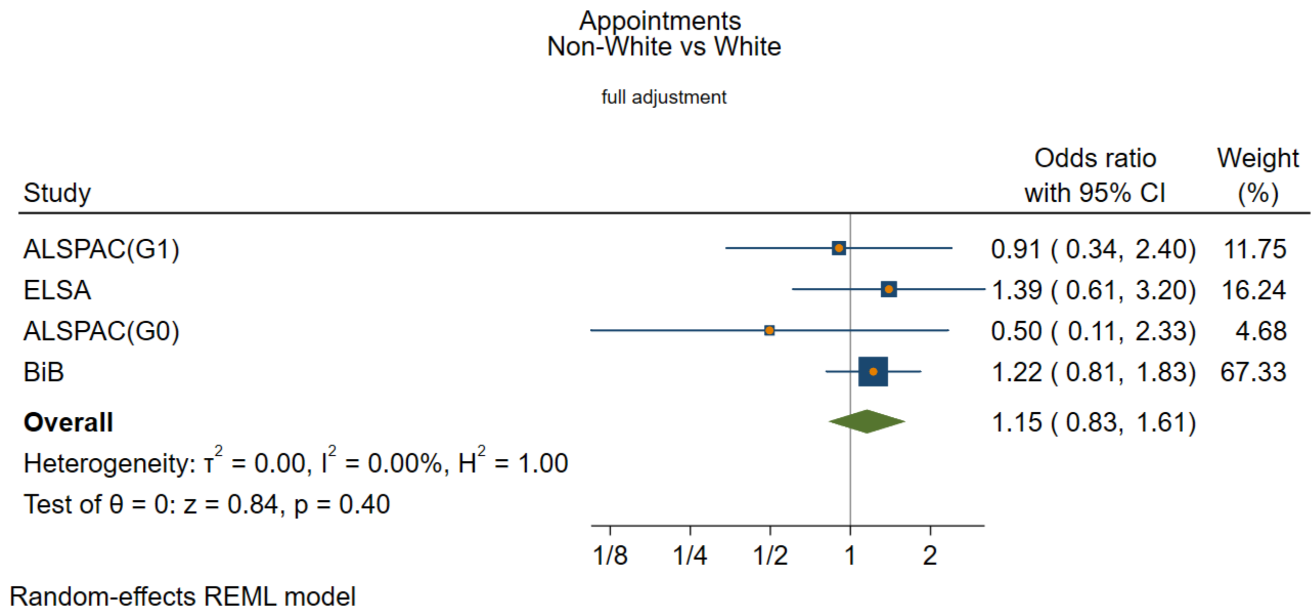

For peer review only

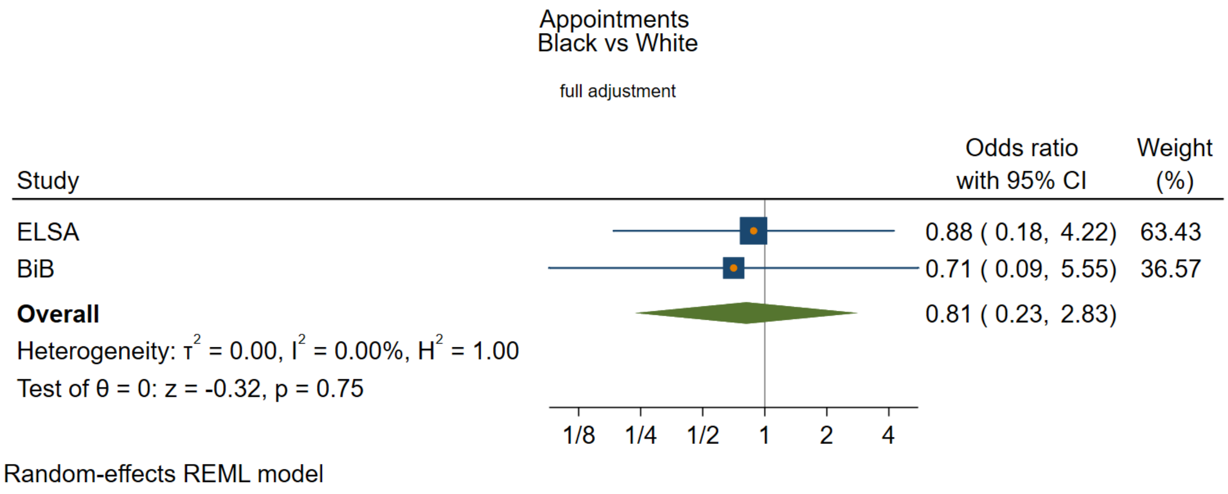

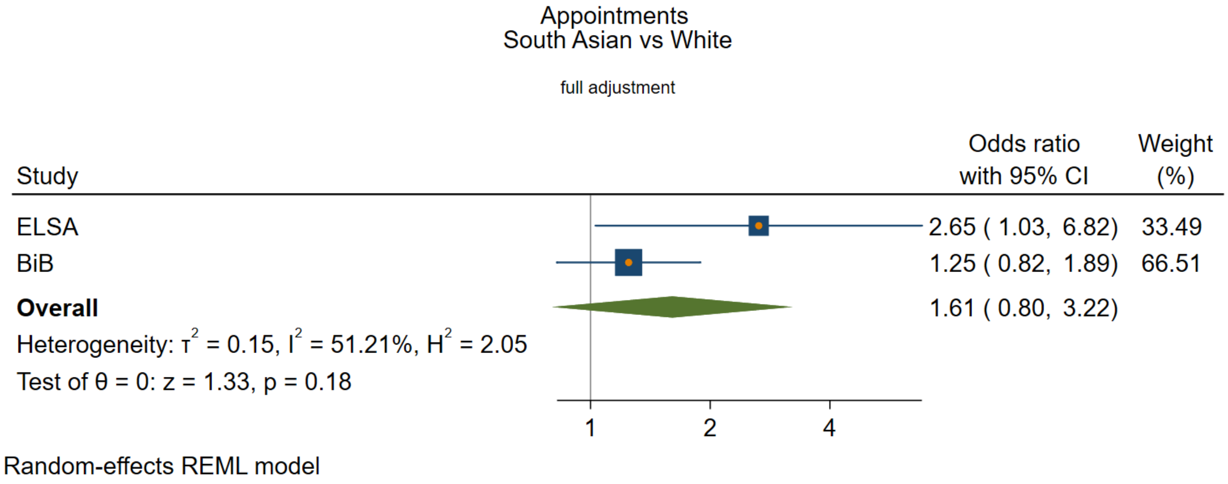

For peer review only - http://bmjopen.bmj.com/site/about/guidelines.xhtml

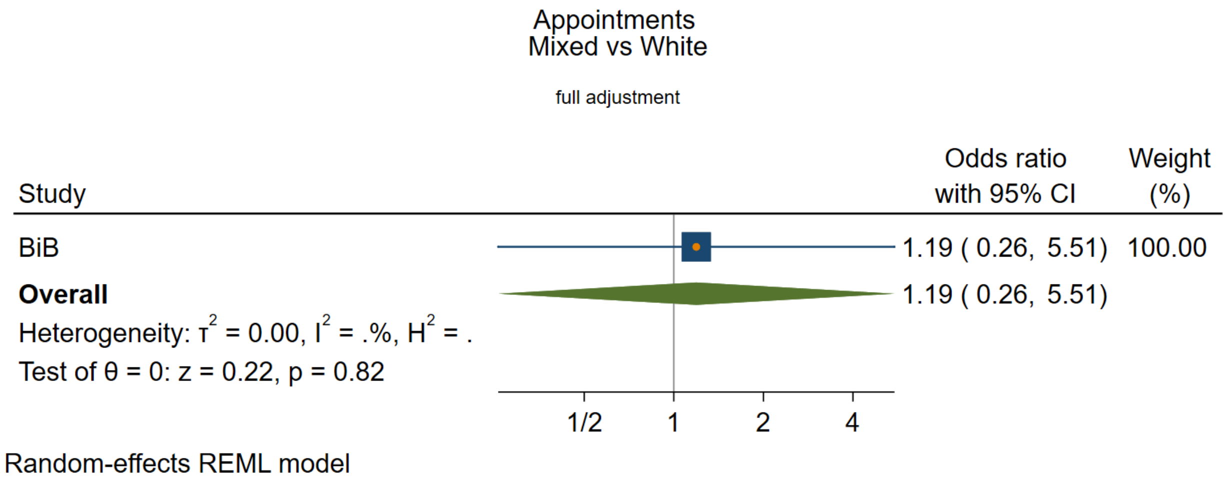

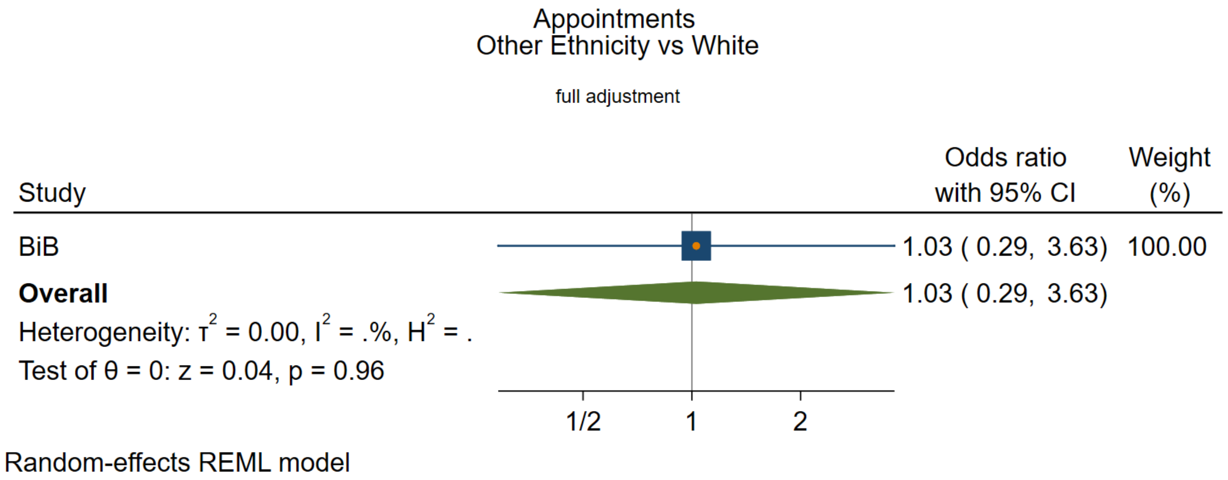

For peer review only - http://bmjopen.bmj.com/site/about/guidelines.xhtml

Prescription/Medication access

Sex

<u>Unadjusted</u>

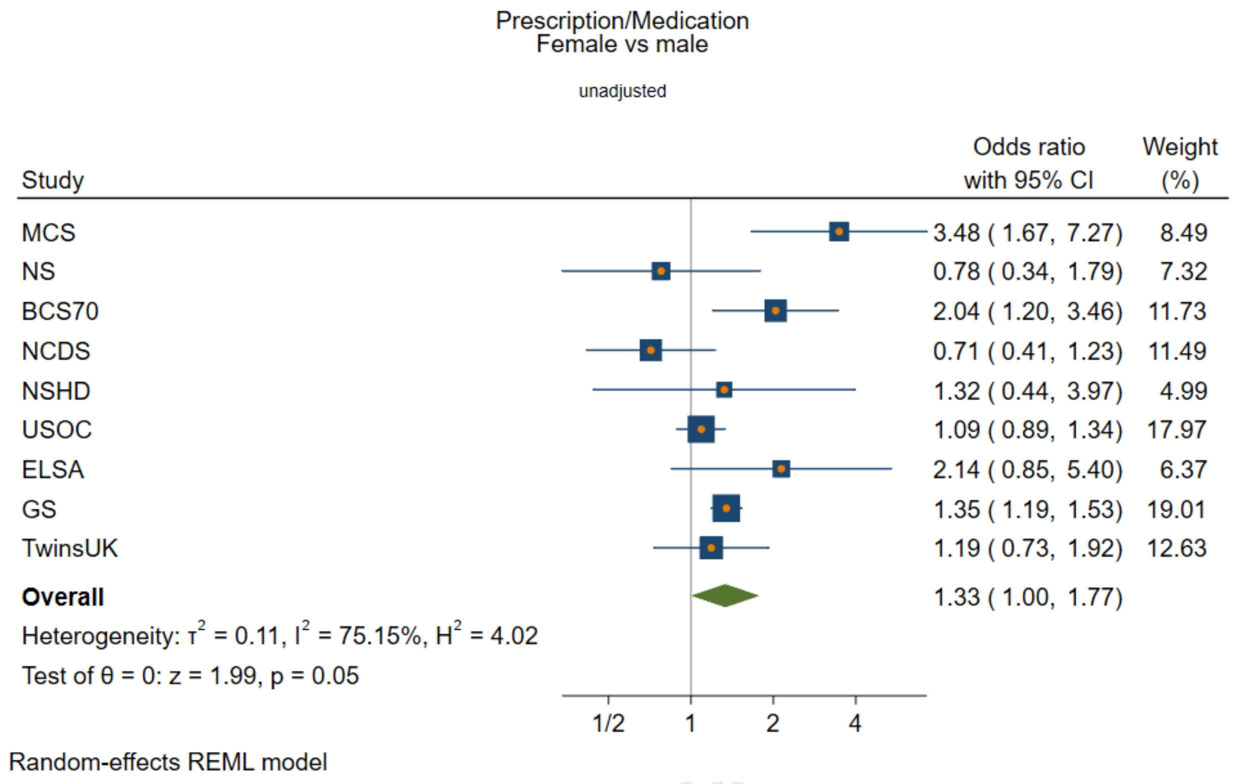

For peer review only - http://bmjopen.bmj.com/site/about/guidelines.xhtml

Basic adjustment

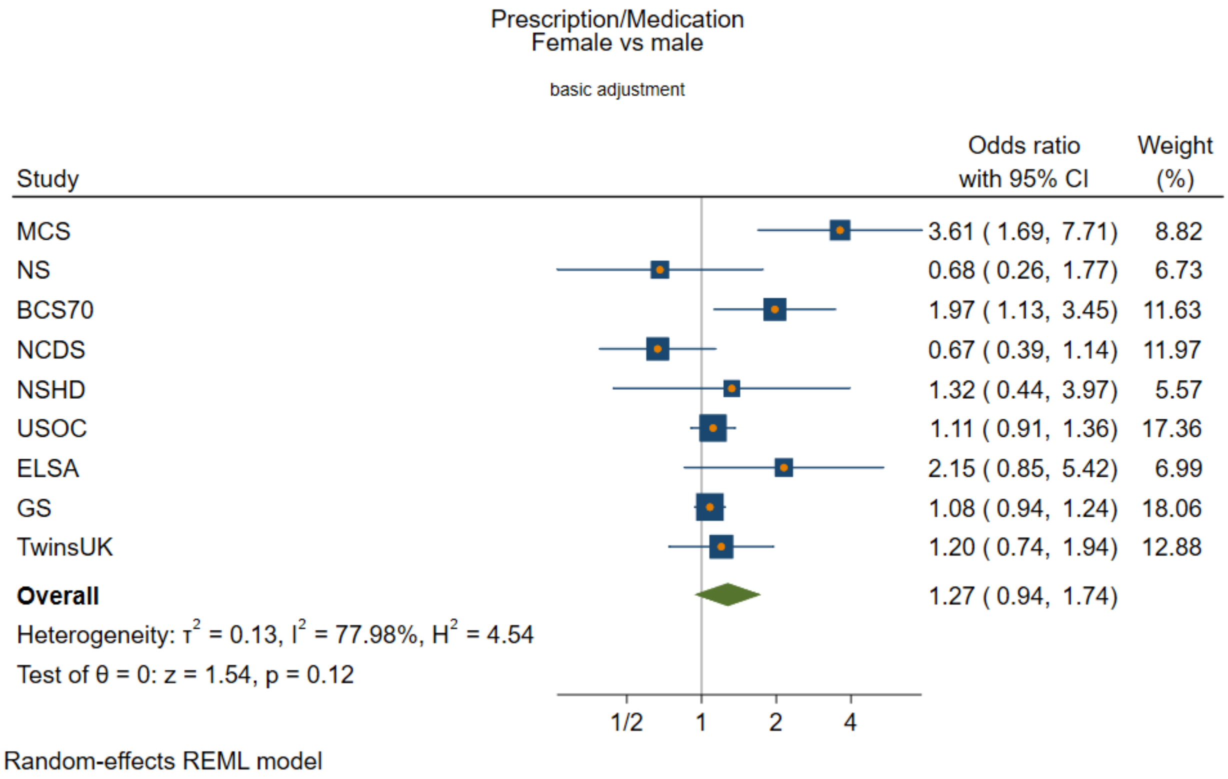

view only

For peer review only - http://bmjopen.bmj.com/site/about/guidelines.xhtml

Full adjustment

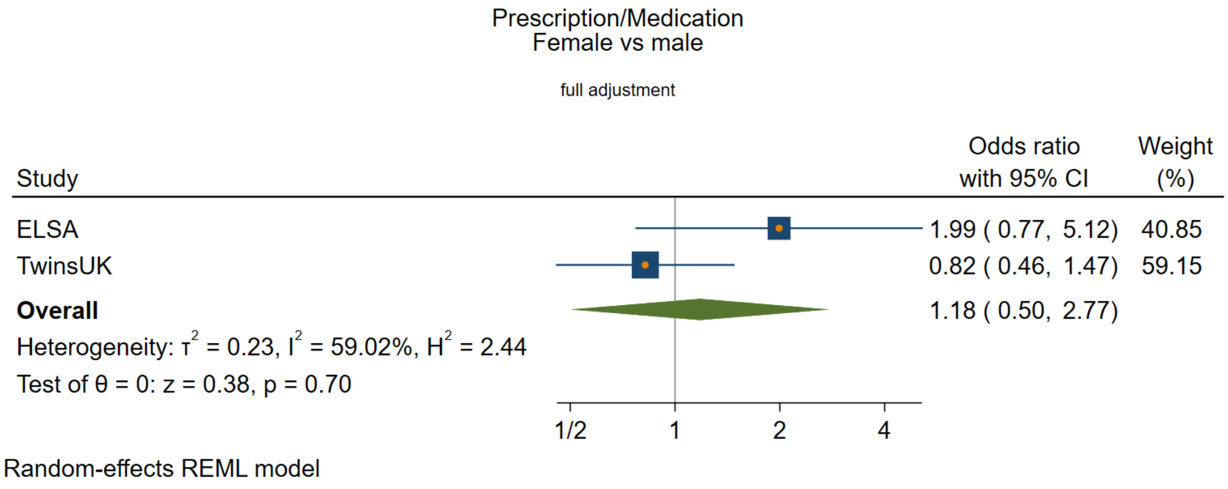

peer review only

For peer review only - http://bmjopen.bmj.com/site/about/guidelines.xhtml

Occupational class
Unadjusted

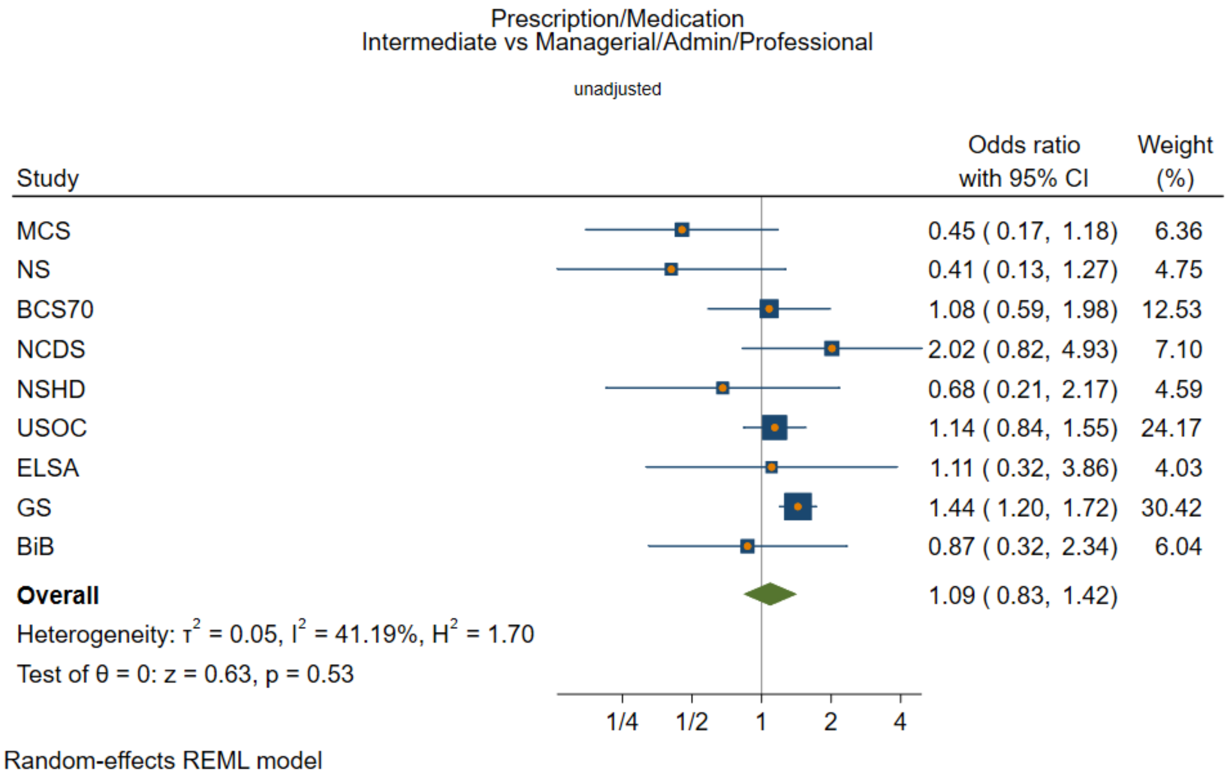

For peer review only - http://bmjopen.bmj.com/site/about/guidelines.xhtml

Prescription/Medication
Manual/Routine vs Managerial/Admin/Professional

unadjusted

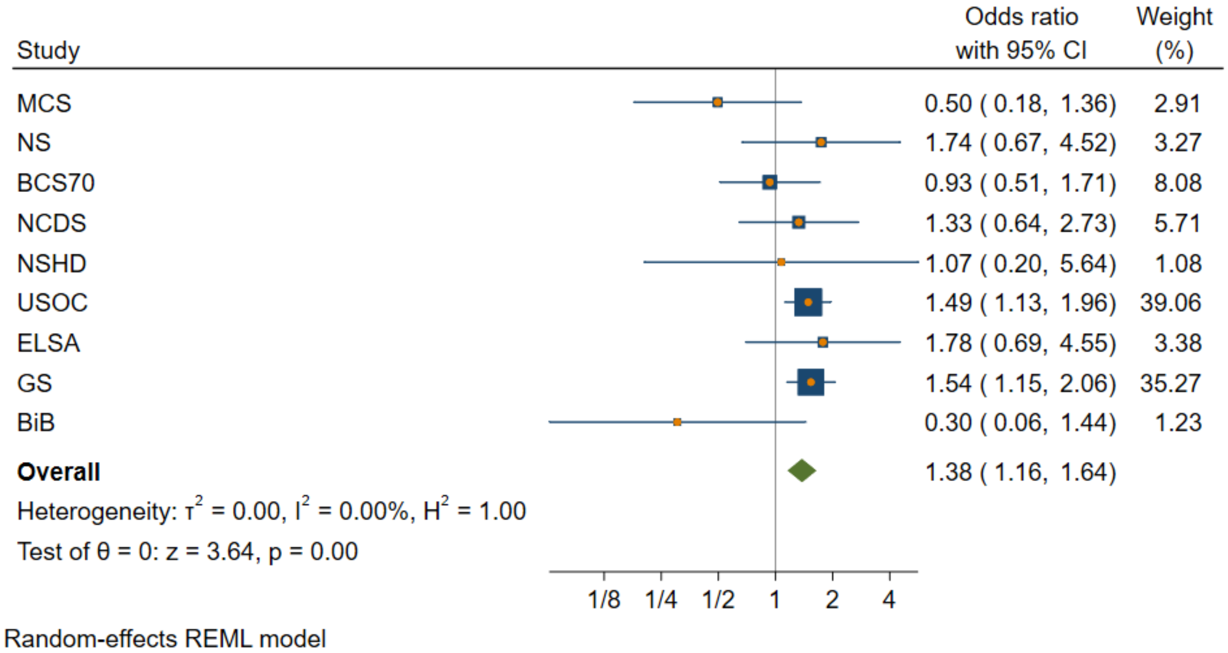

Prescription/Medication
Other social class vs Managerial/Admin/Professional

unadjusted

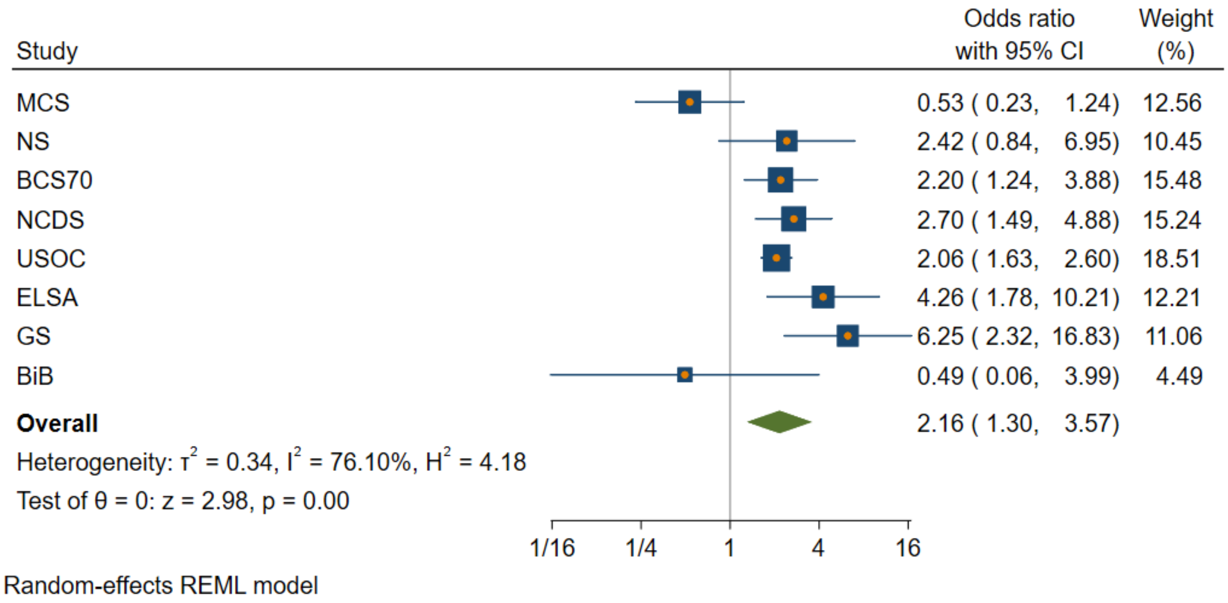

For peer review only - http://bmjopen.bmj.com/site/about/guidelines.xhtml

Basic adjustment

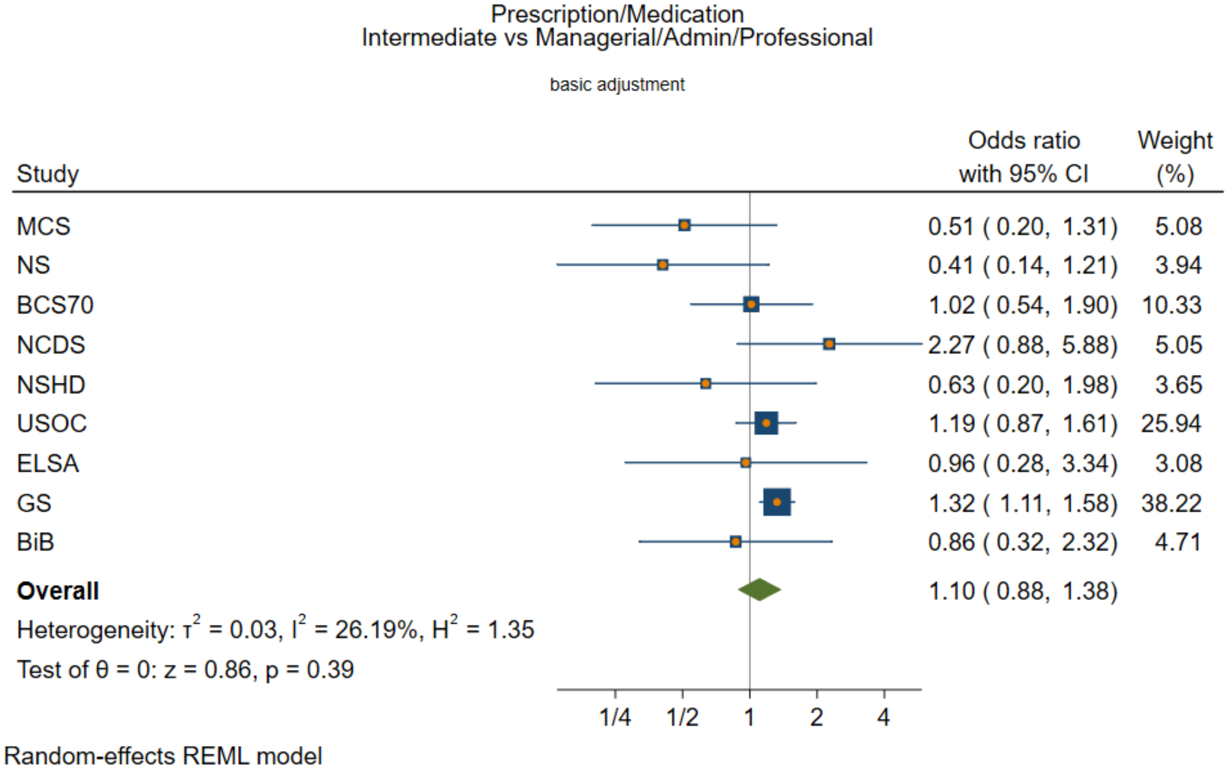

For peer review only

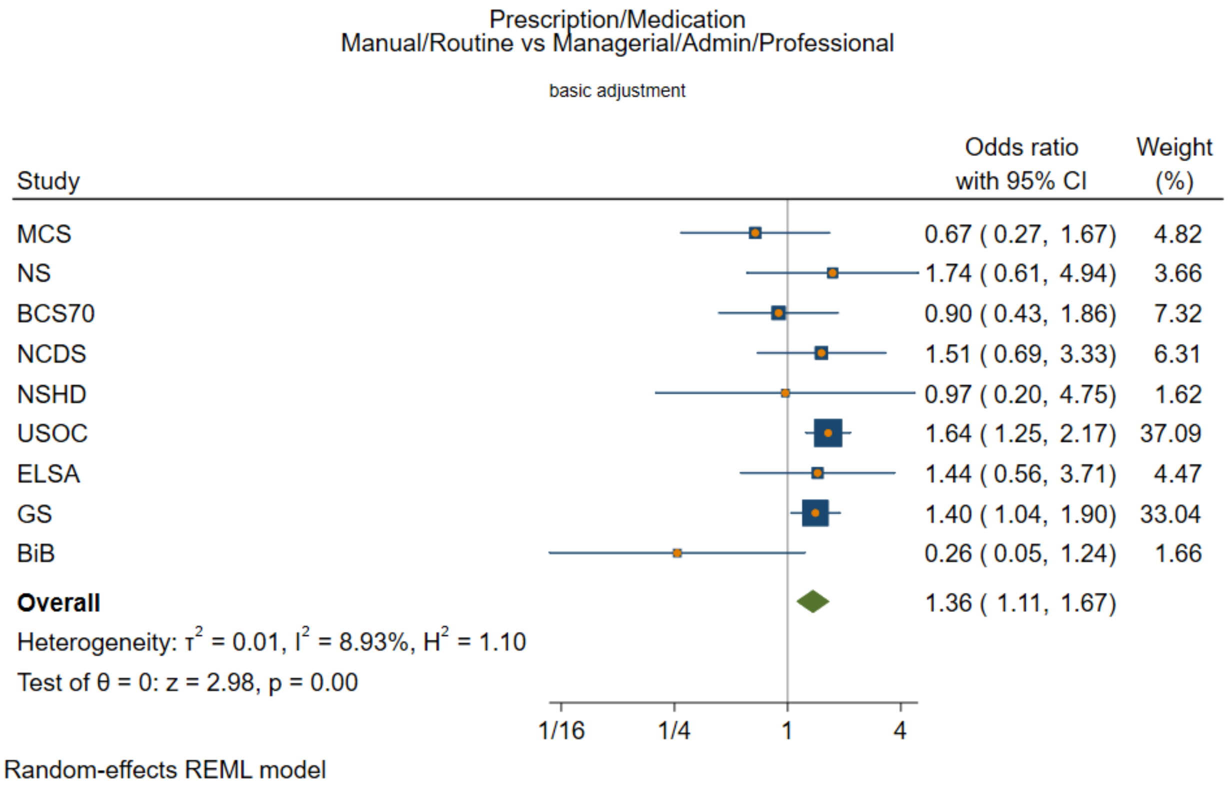

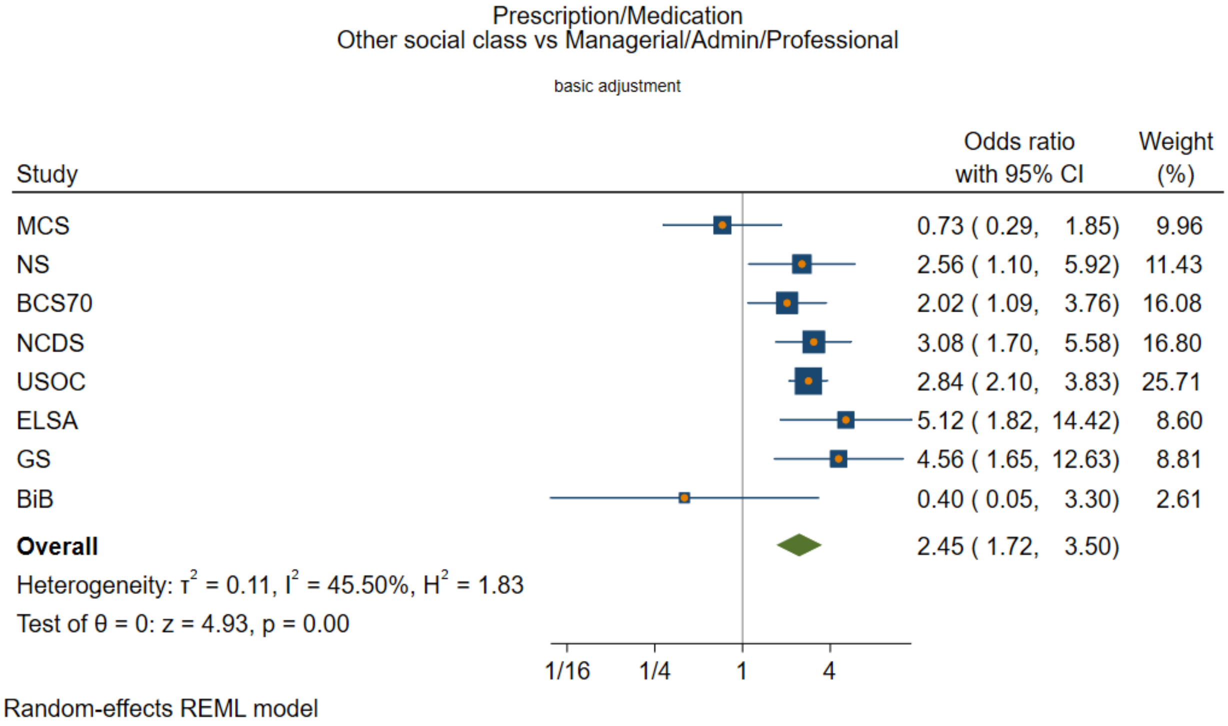

For peer review only - http://bmjopen.bmj.com/site/about/guidelines.xhtml

Full adjustment

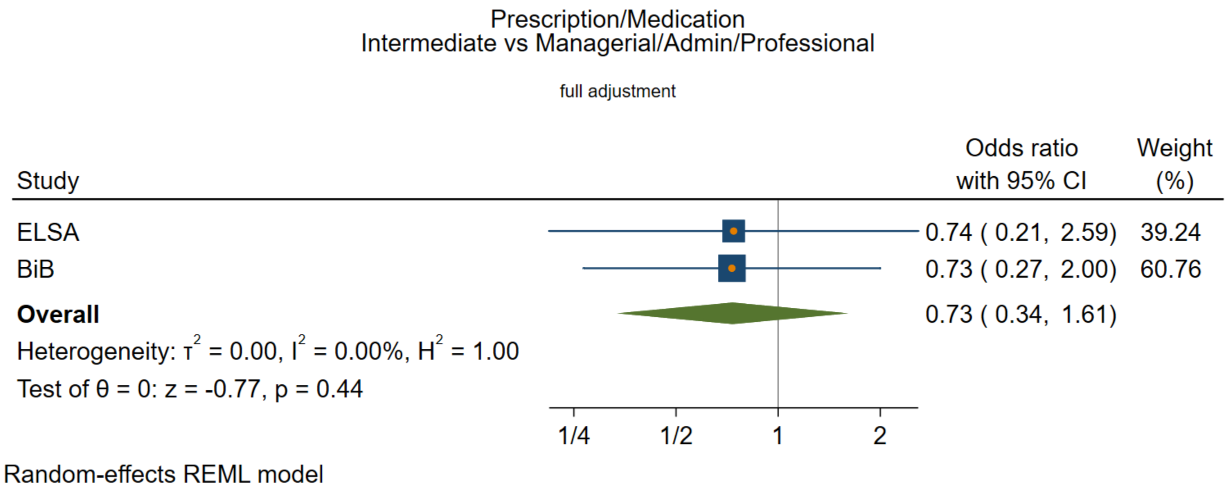

peer review only

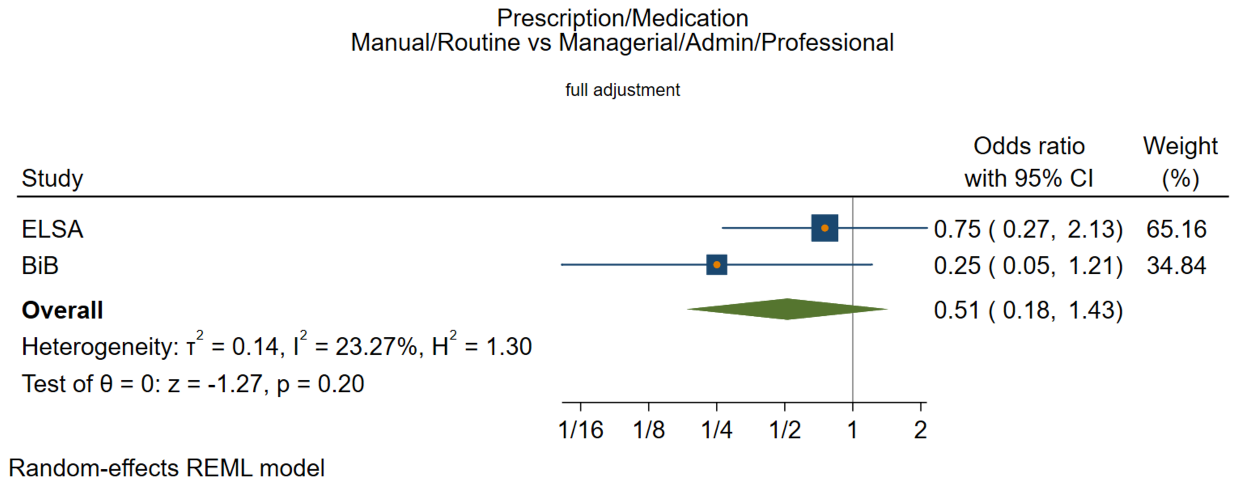
Prescription/Medication
Manual/Routine vs Managerial/Admin/Professional
full adjustment
Study
Odds ratio with 95% CI
Weight (%)
ELSA 0.75 (0.27, 2.13) 65.16
BiB 0.25 (0.05, 1.21) 34.84
Overall 0.51 (0.18, 1.43)
Heterogeneity: τ² = 0.14, I² = 23.27%, H² = 1.30
Test of θ = 0: z = -1.27, p = 0.20
1/16 1/8 1/4 1/2 1 2
Random-effects REML model

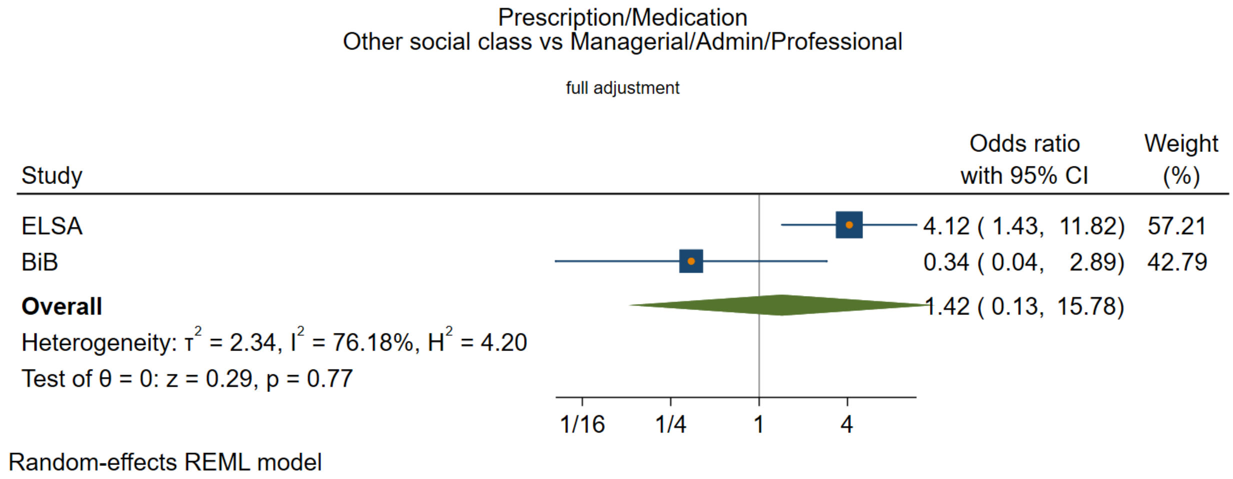
Prescription/Medication
Other social class vs Managerial/Admin/Professional
full adjustment
Study
Odds ratio with 95% CI
Weight (%)
ELSA 4.12 (1.43, 11.82) 57.21
BiB 0.34 (0.04, 2.89) 42.79
Overall 1.42 (0.13, 15.78)
Heterogeneity: τ² = 2.34, I² = 76.18%, H² = 4.20
Test of θ = 0: z = 0.29, p = 0.77
1/16 1/4 1 4
Random-effects REML model

Education

<u>Unadjusted</u>

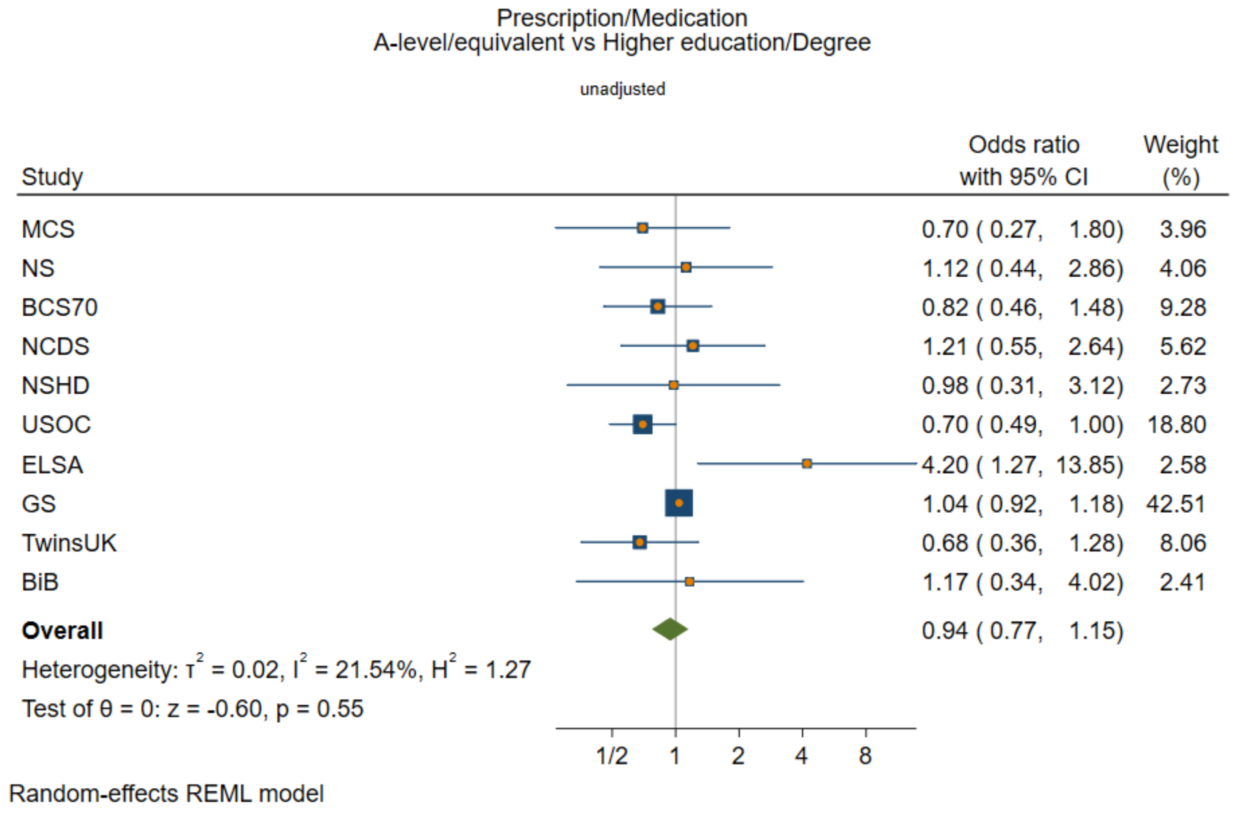

For peer review only - http://bmjopen.bmj.com/site/about/guidelines.xhtml

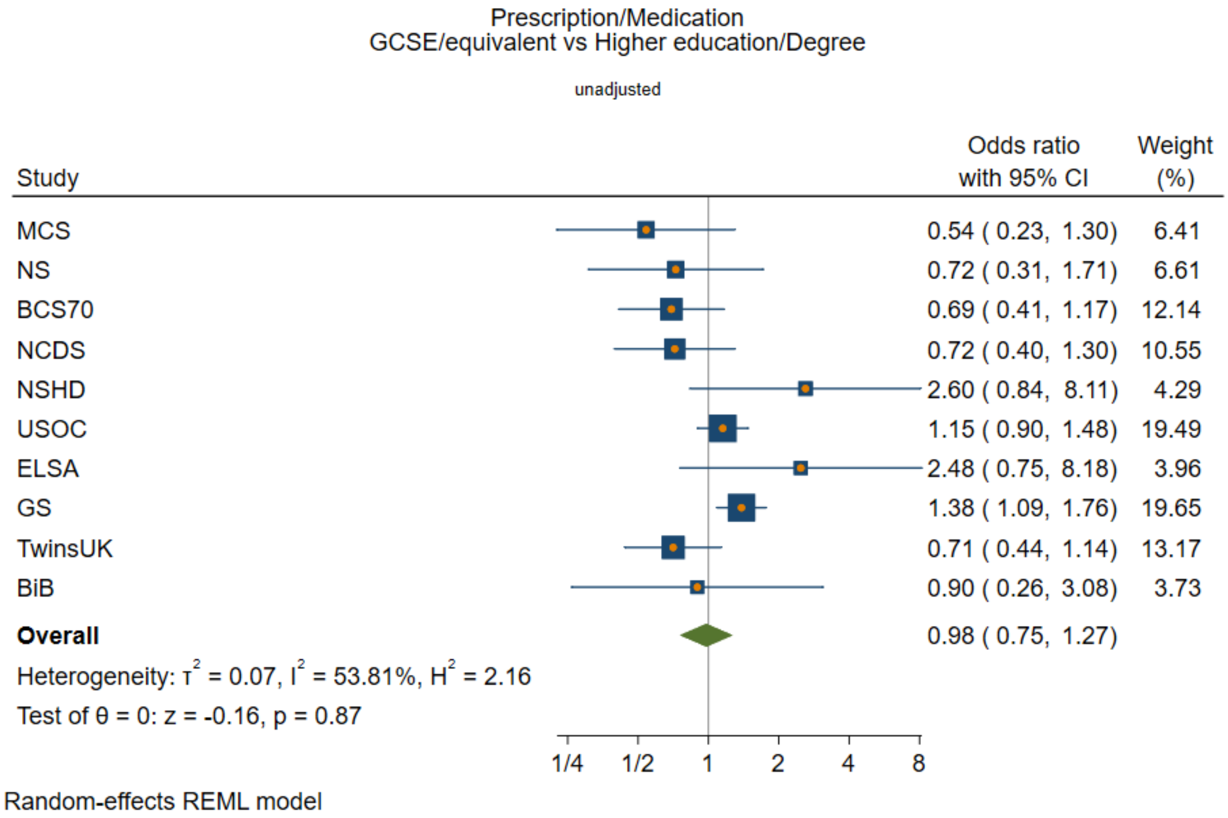

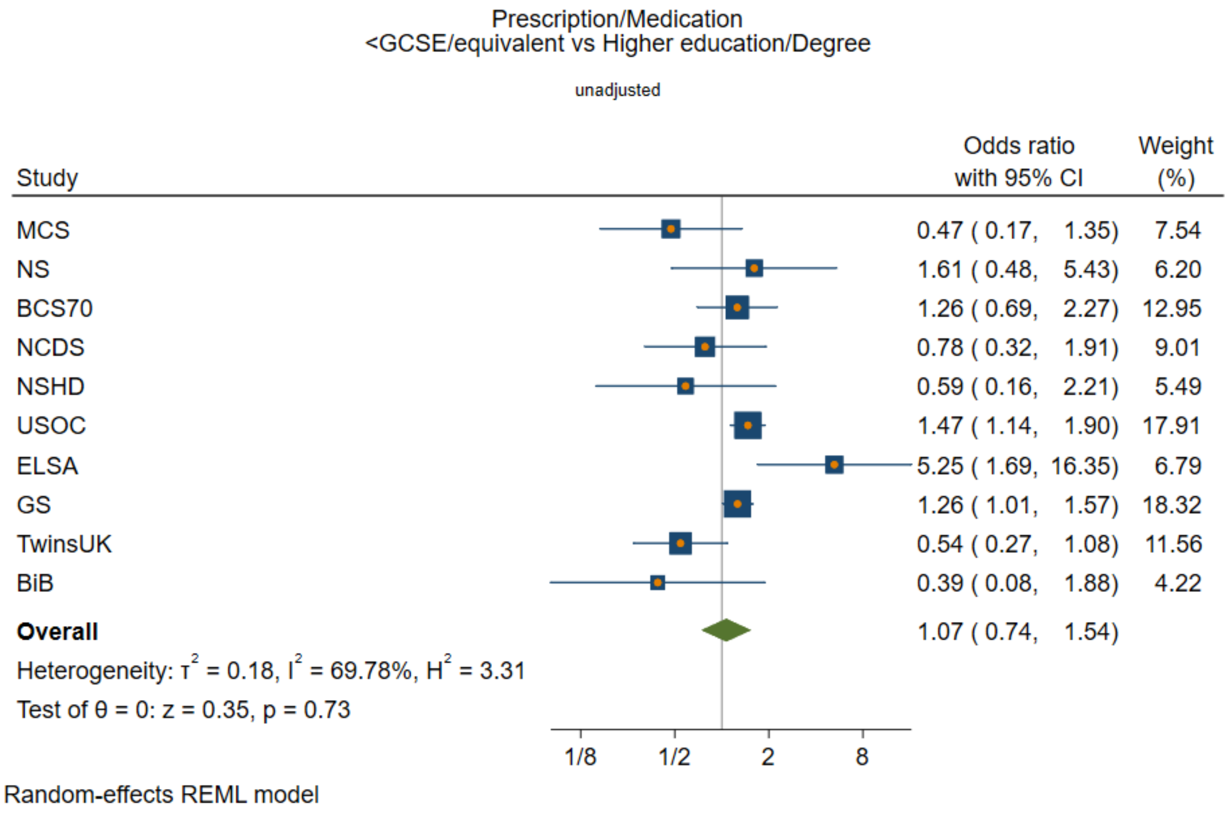

For peer review only - http://bmjopen.bmj.com/site/about/guidelines.xhtml

Basic adjustment

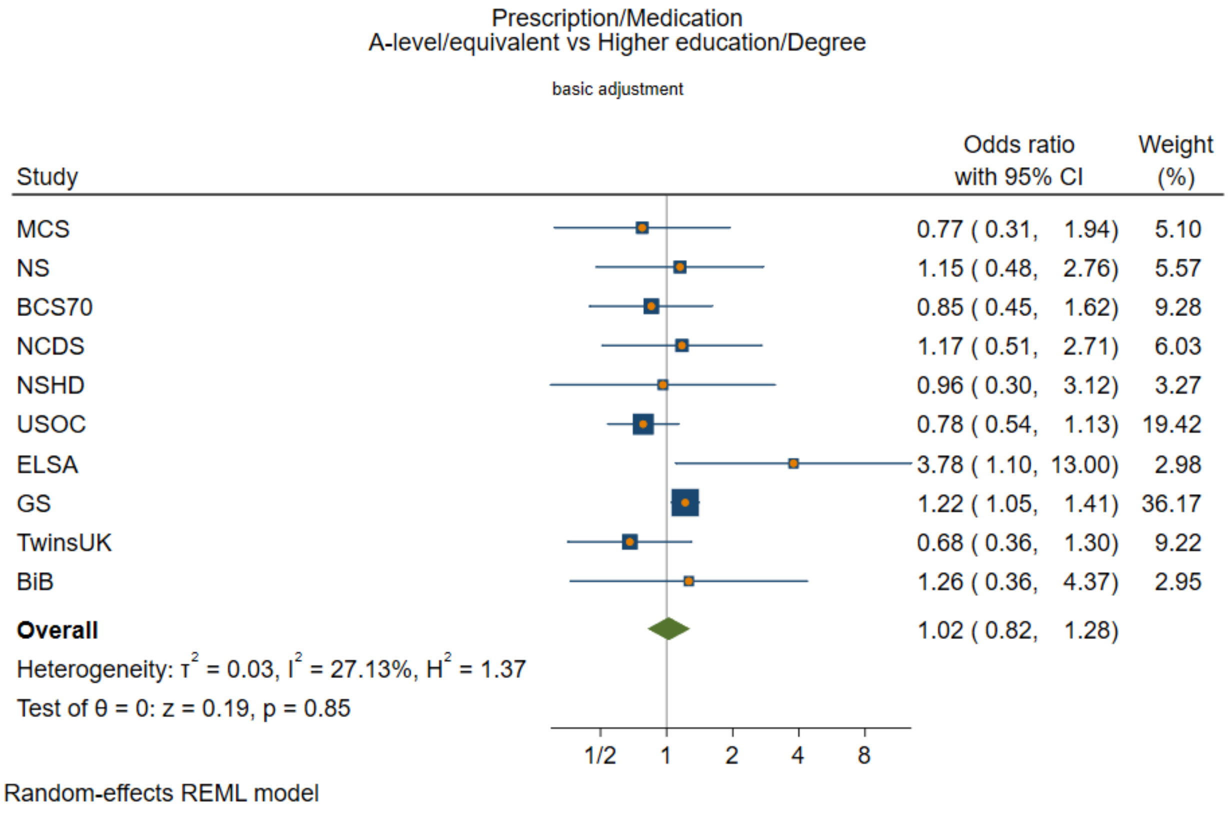

For peer review only - http://bmjopen.bmj.com/site/about/guidelines.xhtml

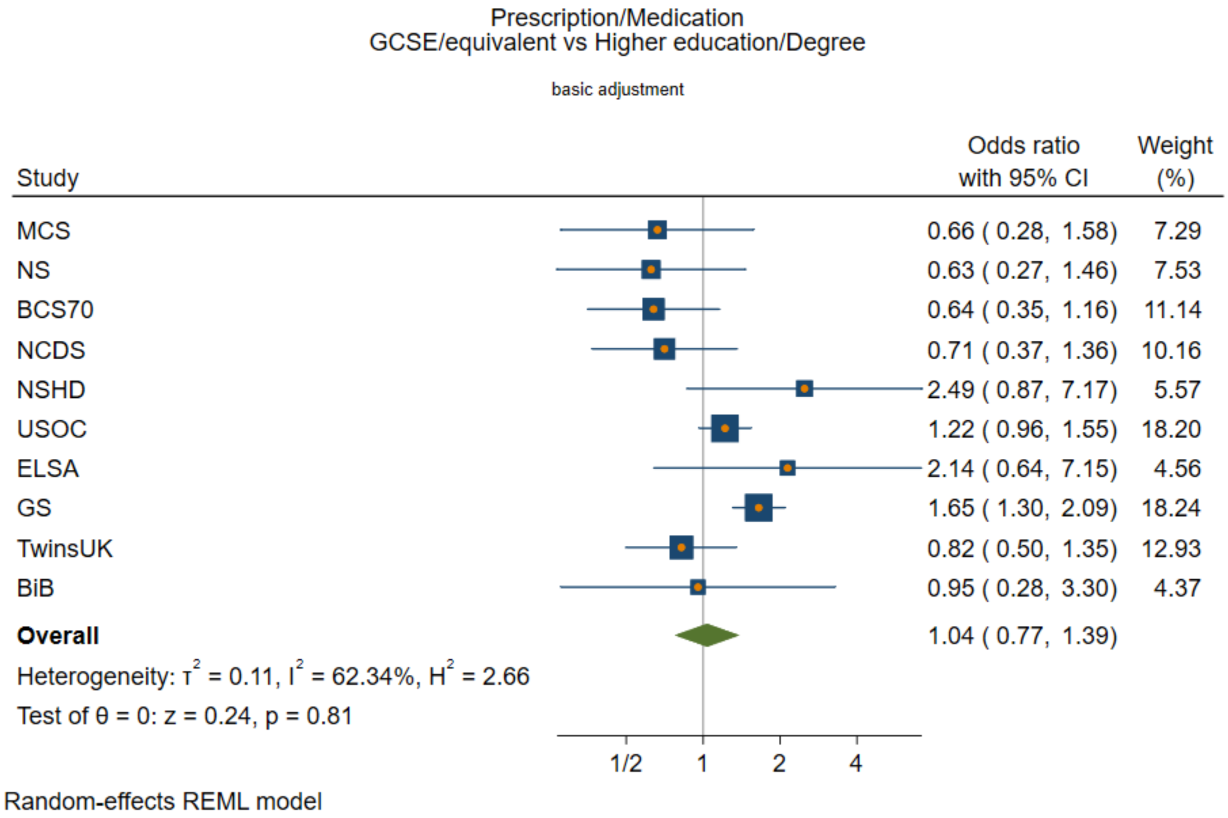

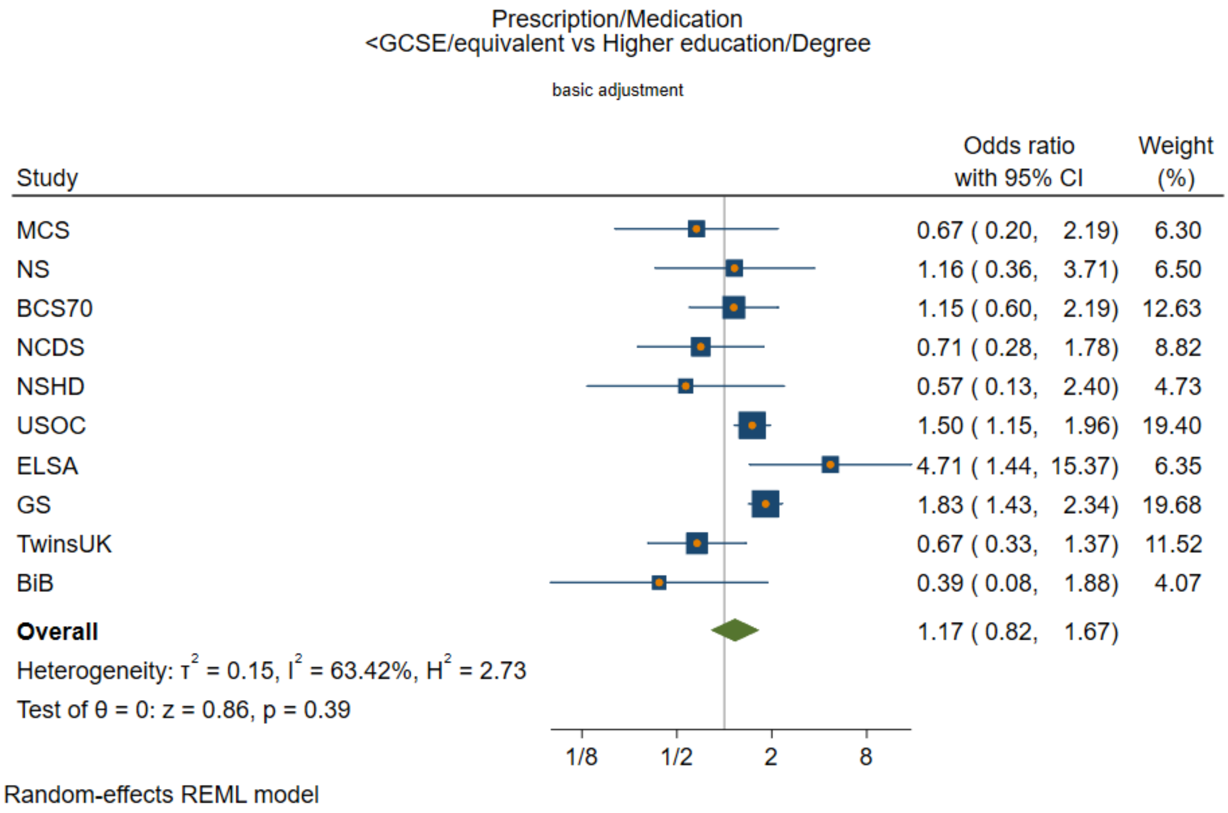

For peer review only - http://bmjopen.bmj.com/site/about/guidelines.xhtml

Full adjustment

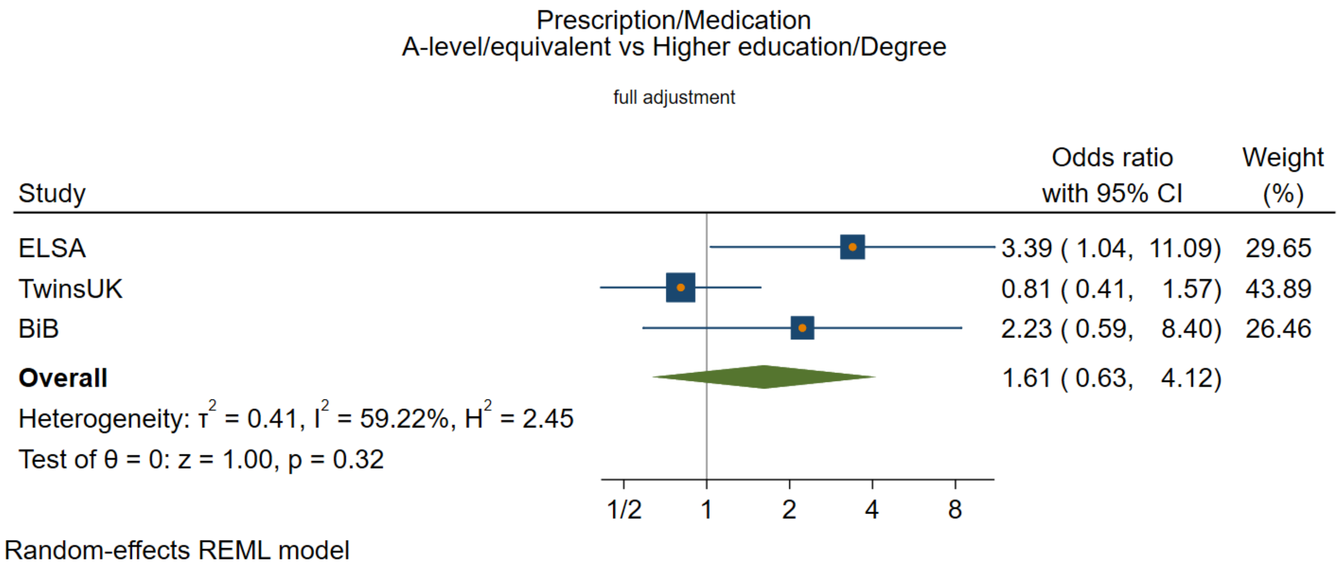

For peer review only

For peer review only - http://bmjopen.bmj.com/site/about/guidelines.xhtml

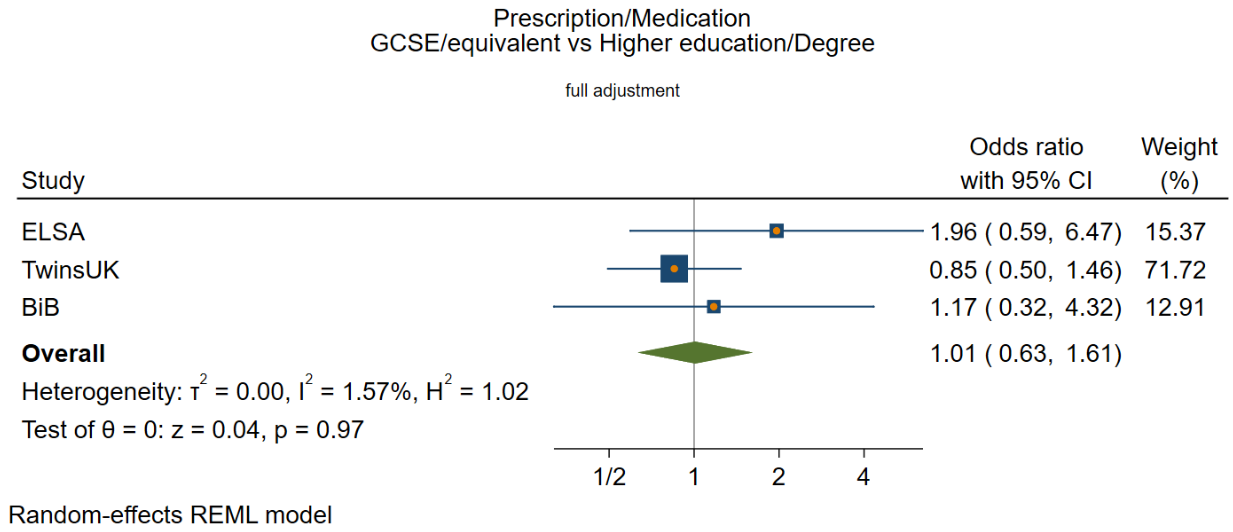

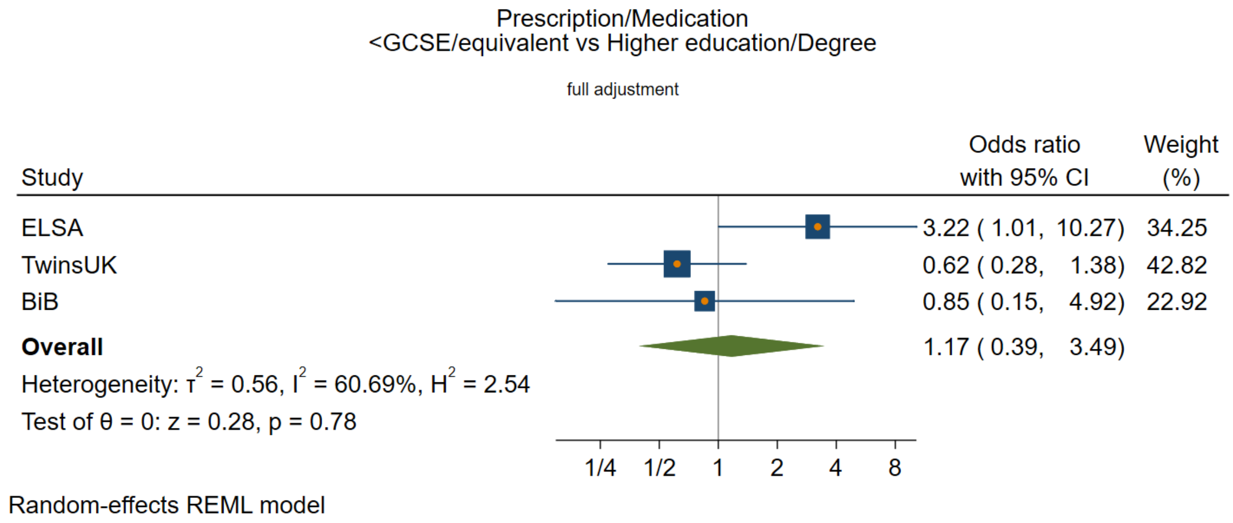

For peer review only - http://bmjopen.bmj.com/site/about/guidelines.xhtml

Age

Unadjusted

For peer review only - http://bmjopen.bmj.com/site/about/guidelines.xhtml

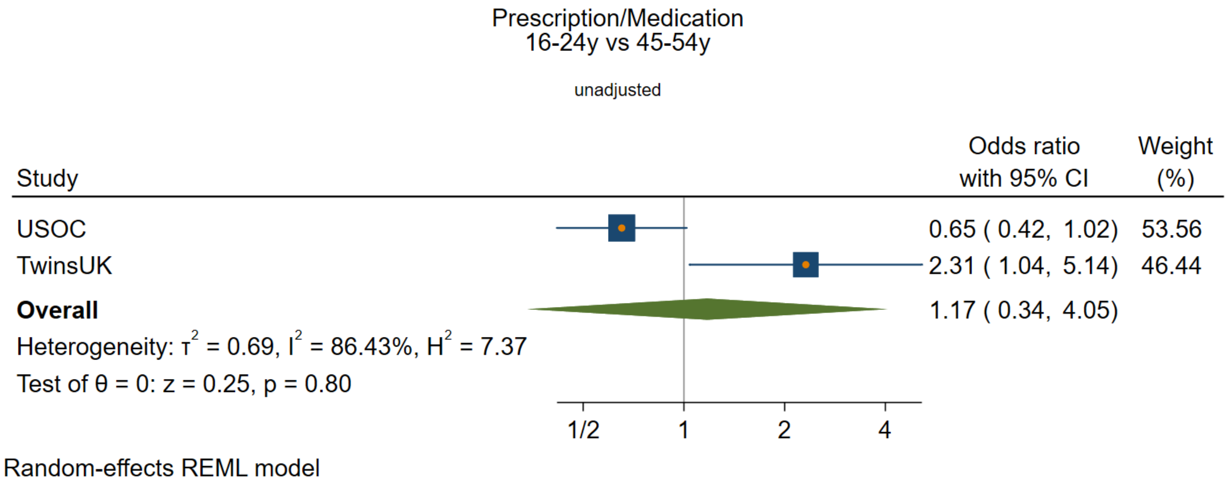

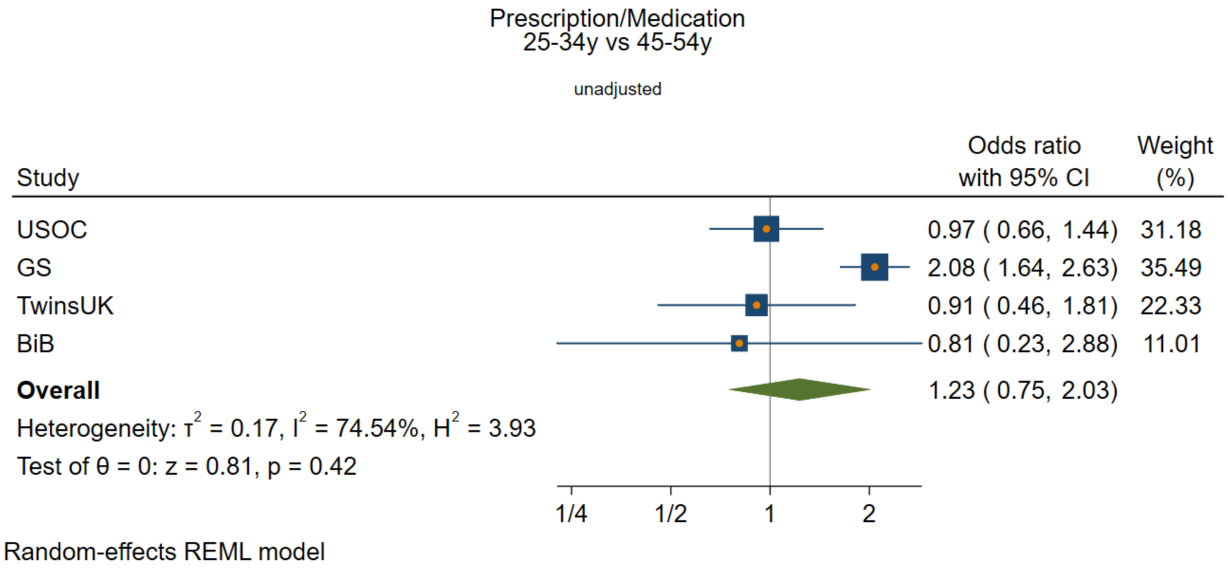

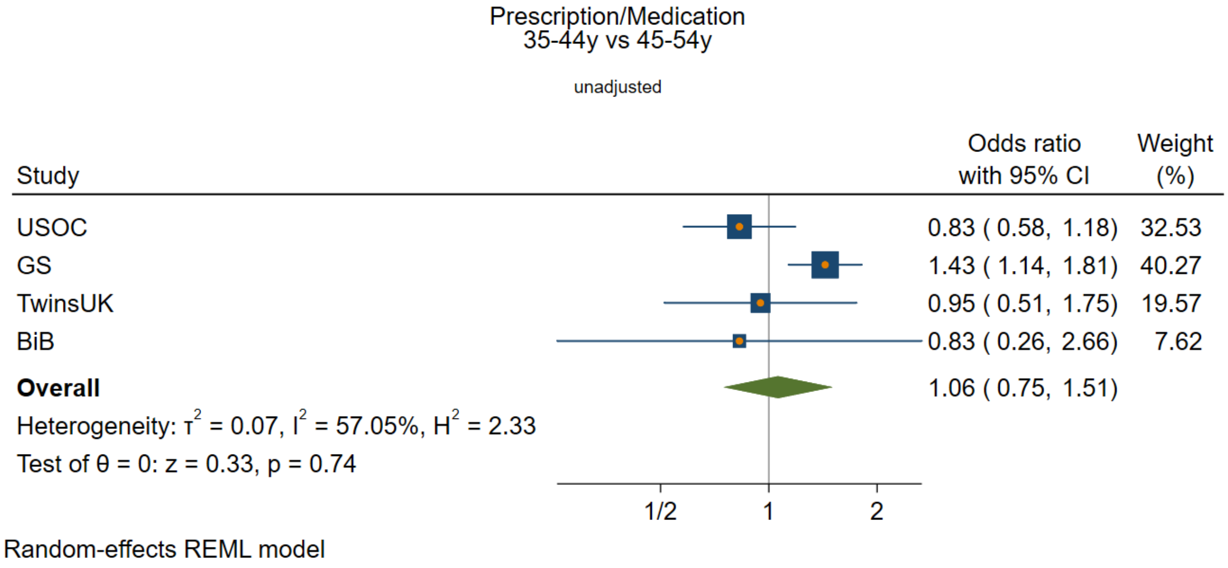

For peer review only - http://bmjopen.bmj.com/site/about/guidelines.xhtml

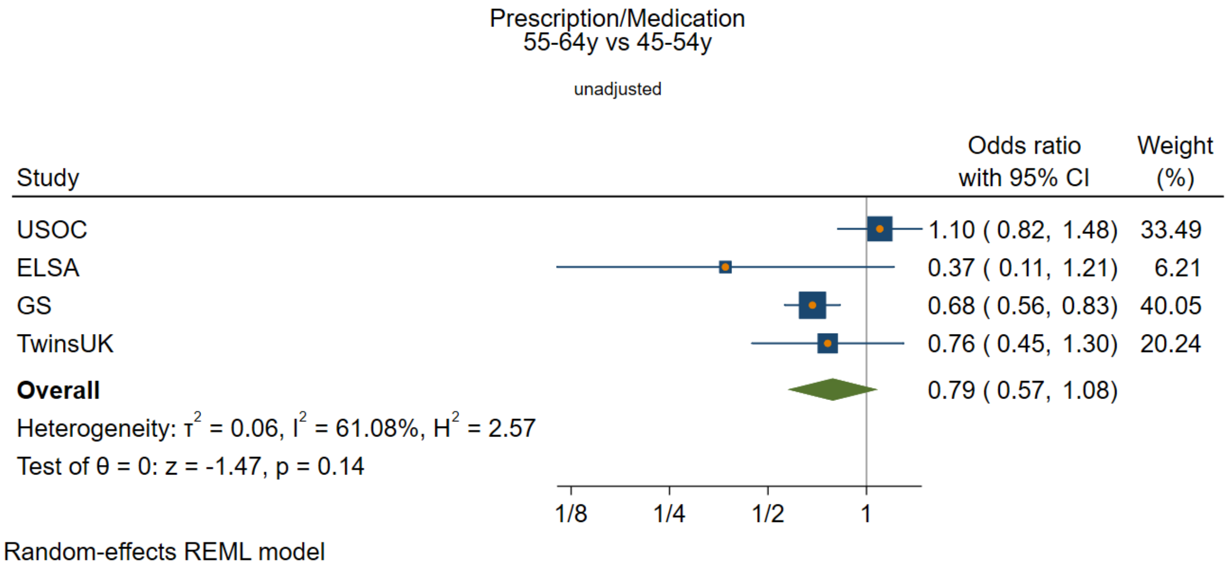

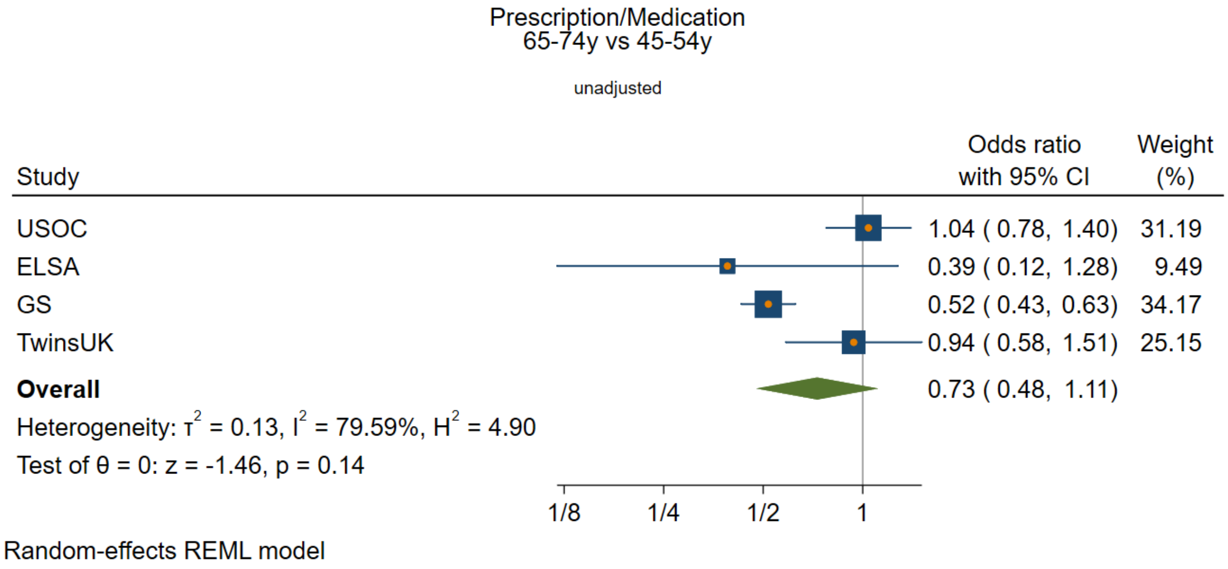

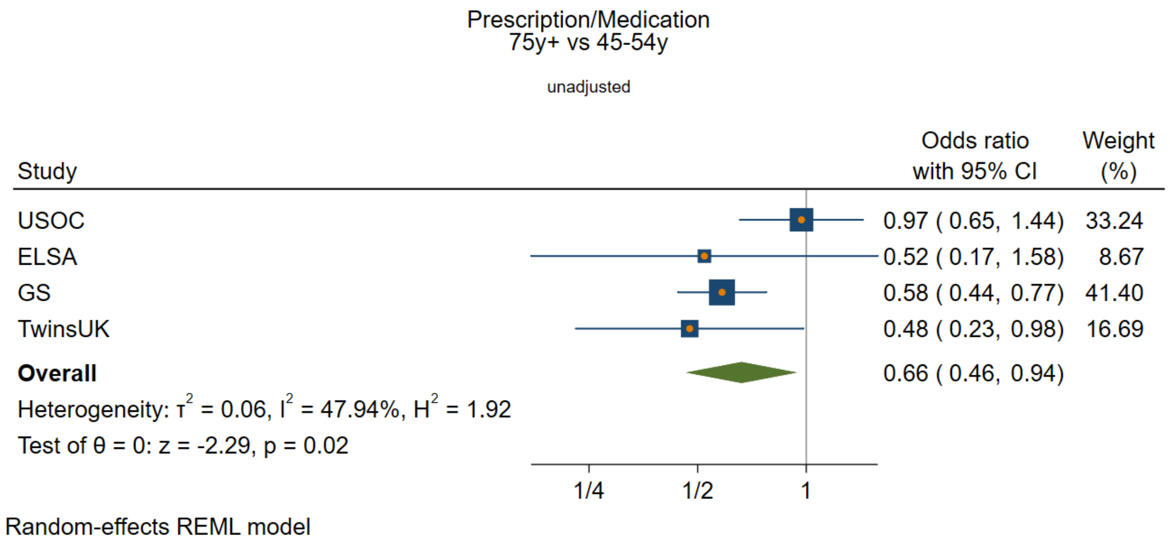

For peer review only - http://bmjopen.bmj.com/site/about/guidelines.xhtml

Basic adjustment

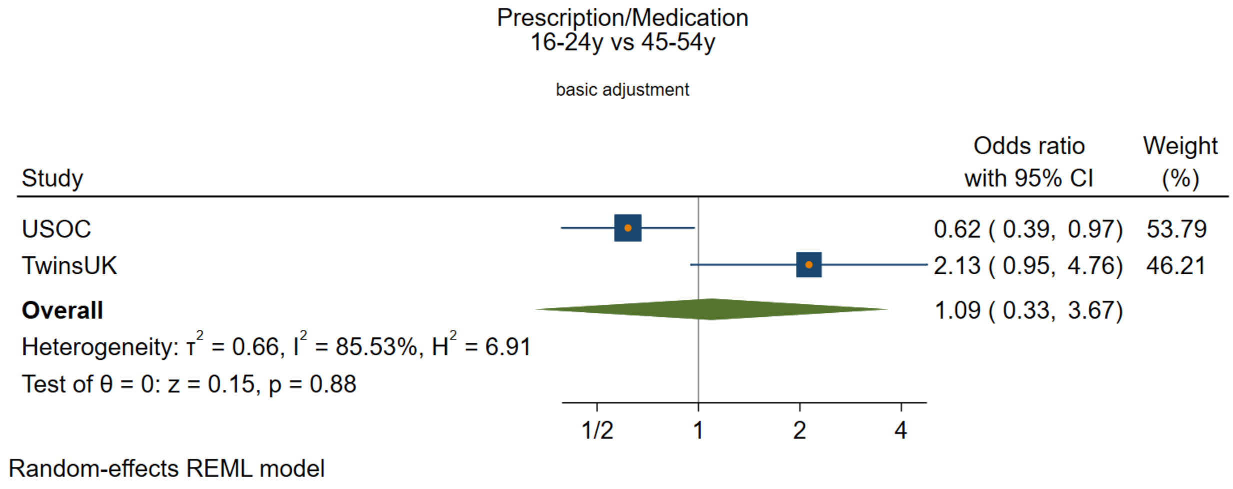

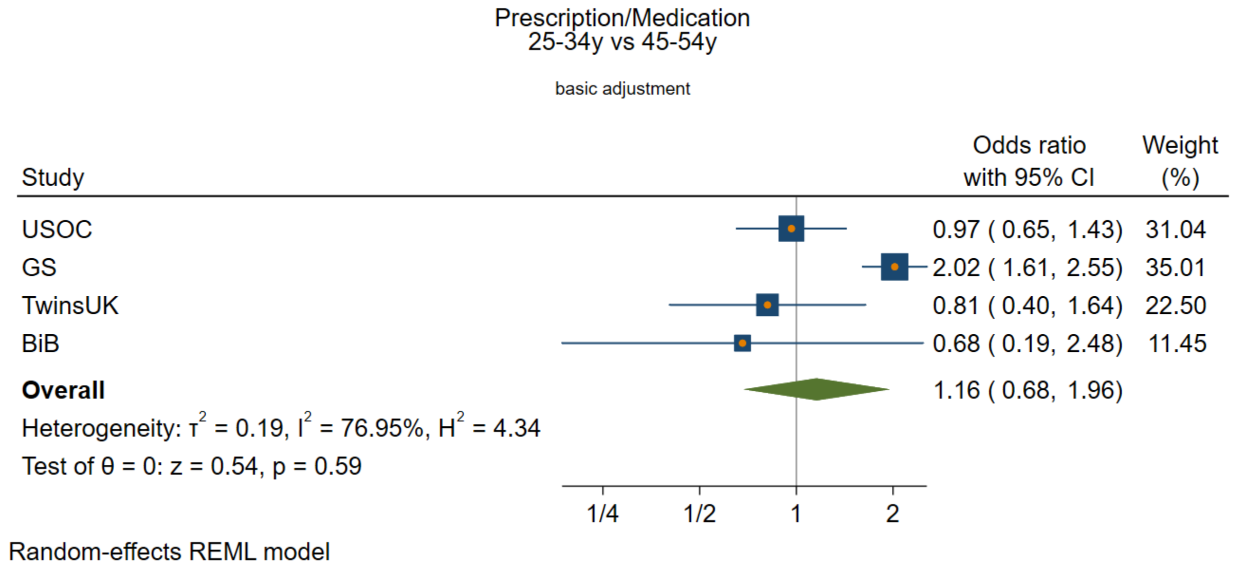

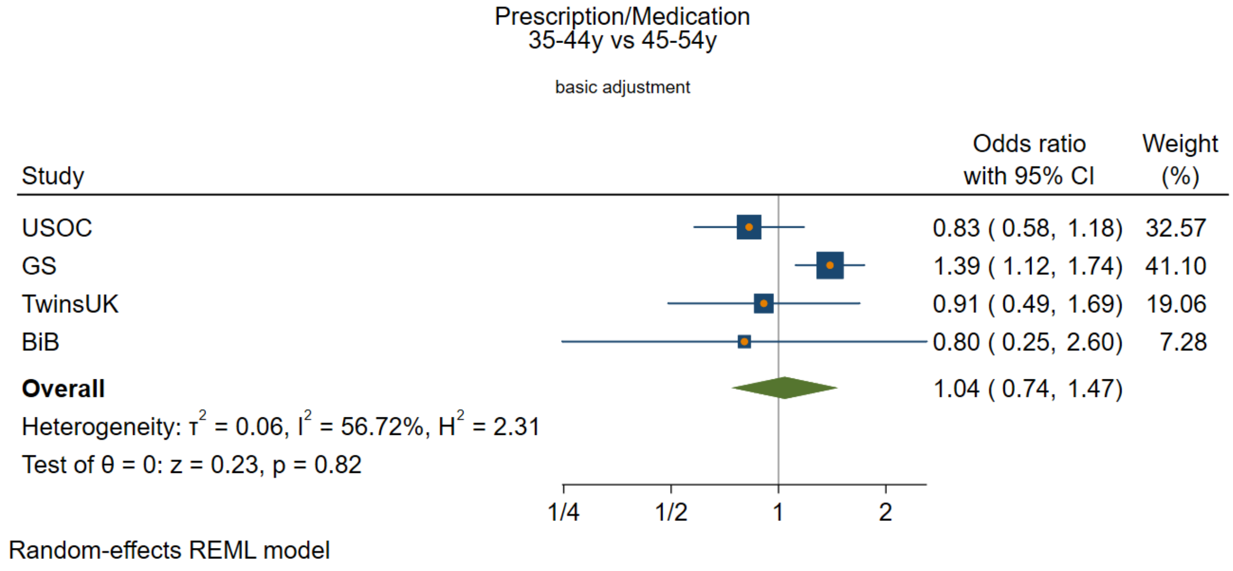

For peer review only - http://bmjopen.bmj.com/site/about/guidelines.xhtml

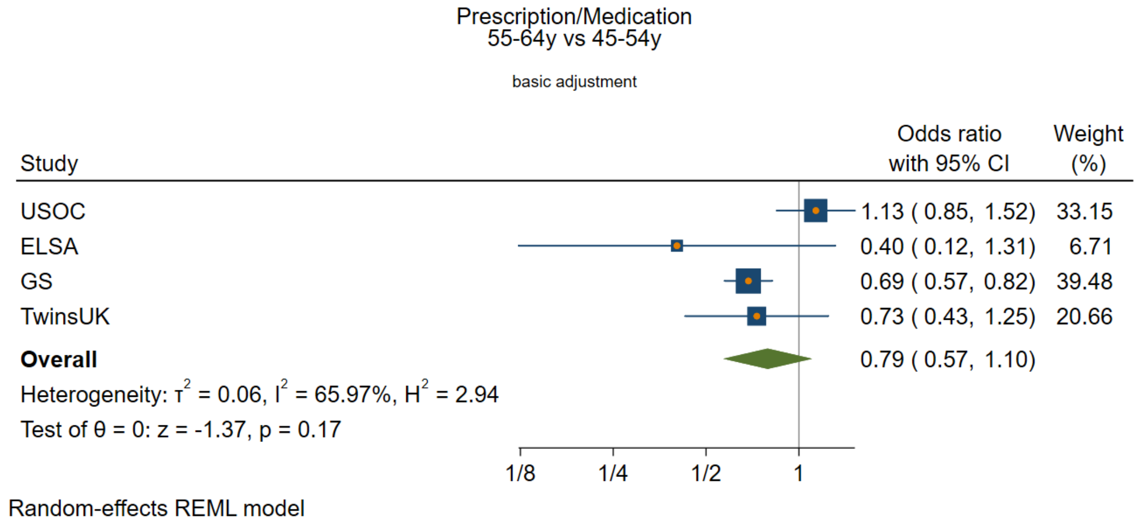
Prescription/Medication
55-64y vs 45-54y
basic adjustment
Study
Odds ratio with 95% CI
Weight (%)
USOC 1.13 (0.85, 1.52) 33.15
ELSA 0.40 (0.12, 1.31) 6.71
GS 0.69 (0.57, 0.82) 39.48
TwinsUK 0.73 (0.43, 1.25) 20.66
Overall 0.79 (0.57, 1.10)
Heterogeneity: τ² = 0.06, I² = 65.97%, H² = 2.94
Test of θ = 0: z = -1.37, p = 0.17
1/8
1/4
1/2
1
Random-effects REML model

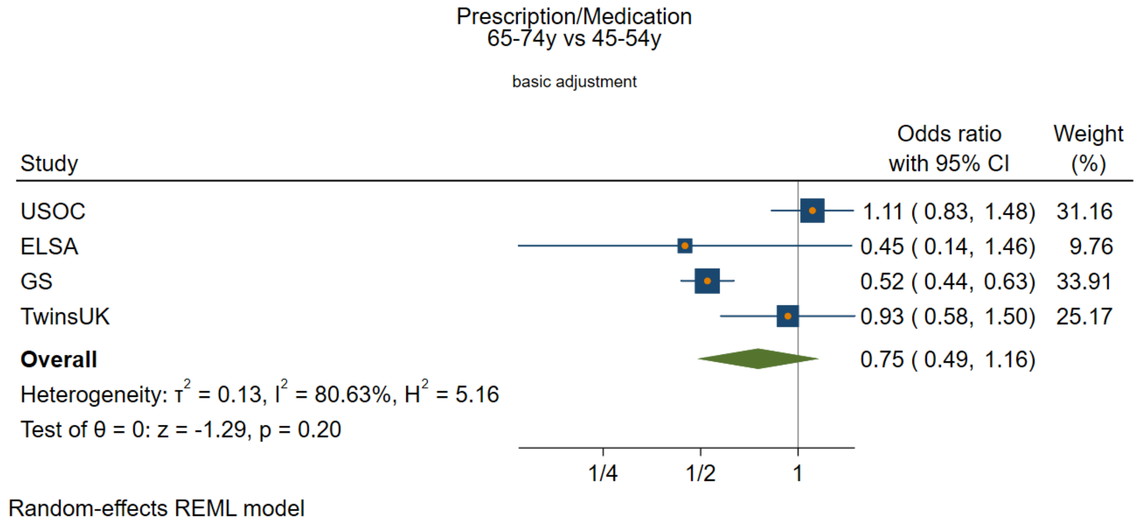
Prescription/Medication
65-74y vs 45-54y
basic adjustment
Study
Odds ratio with 95% CI
Weight (%)
USOC 1.11 (0.83, 1.48) 31.16
ELSA 0.45 (0.14, 1.46) 9.76
GS 0.52 (0.44, 0.63) 33.91
TwinsUK 0.93 (0.58, 1.50) 25.17
Overall 0.75 (0.49, 1.16)
Heterogeneity: τ² = 0.13, I² = 80.63%, H² = 5.16
Test of θ = 0: z = -1.29, p = 0.20
1/4
1/2
1
Random-effects REML model

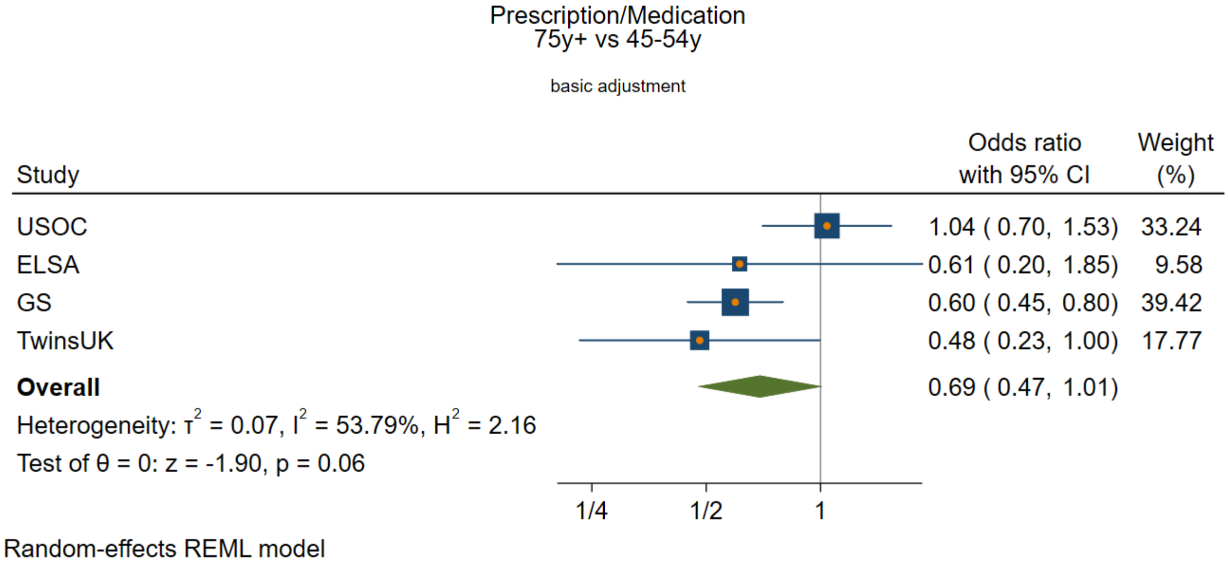
Prescription/Medication
75y+ vs 45-54y
basic adjustment
Study
Odds ratio with 95% CI
Weight (%)
USOC 1.04 (0.70, 1.53) 33.24
ELSA 0.61 (0.20, 1.85) 9.58
GS 0.60 (0.45, 0.80) 39.42
TwinsUK 0.48 (0.23, 1.00) 17.77
Overall 0.69 (0.47, 1.01)
Heterogeneity: τ² = 0.07, I² = 53.79%, H² = 2.16
Test of θ = 0: z = -1.90, p = 0.06
1/4
1/2
1
Random-effects REML model

Full adjustment

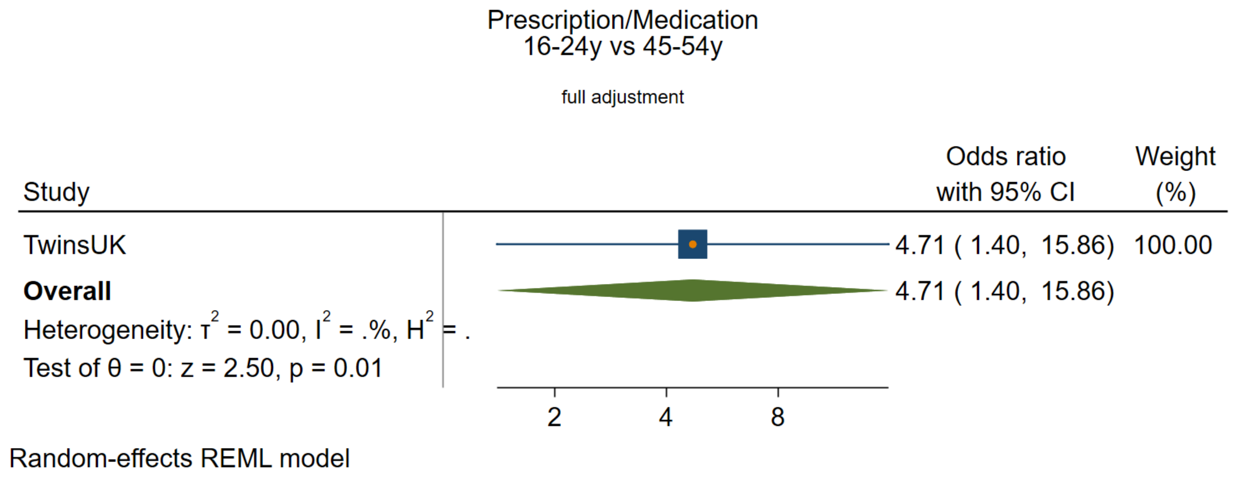

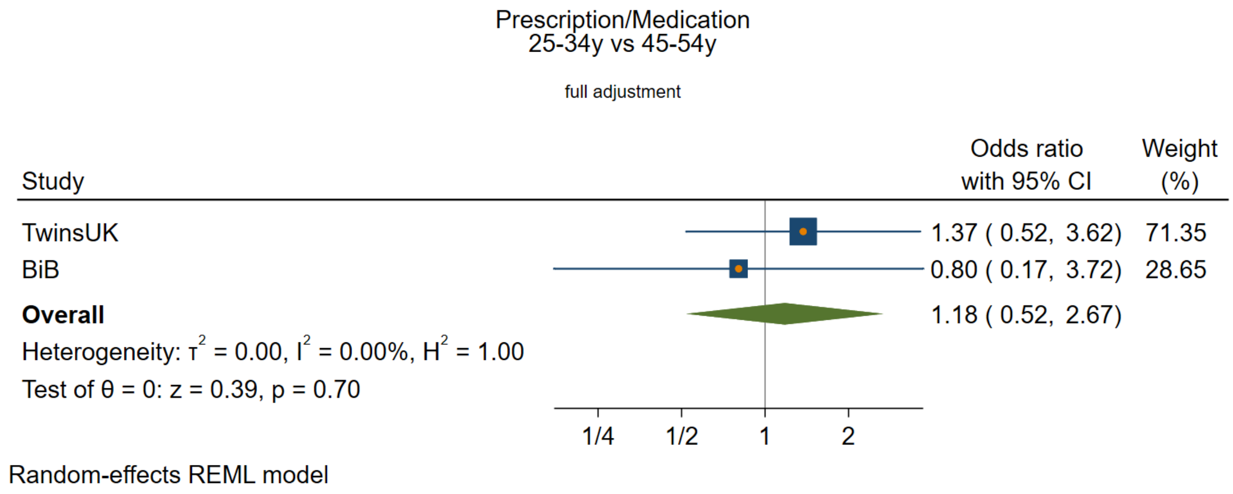

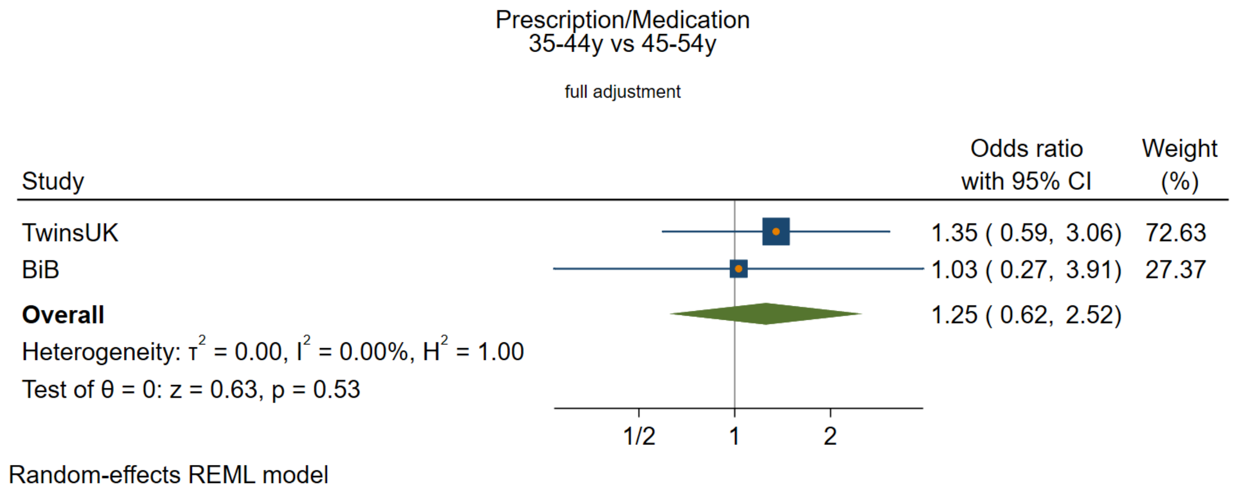

For peer review only - http://bmjopen.bmj.com/site/about/guidelines.xhtml

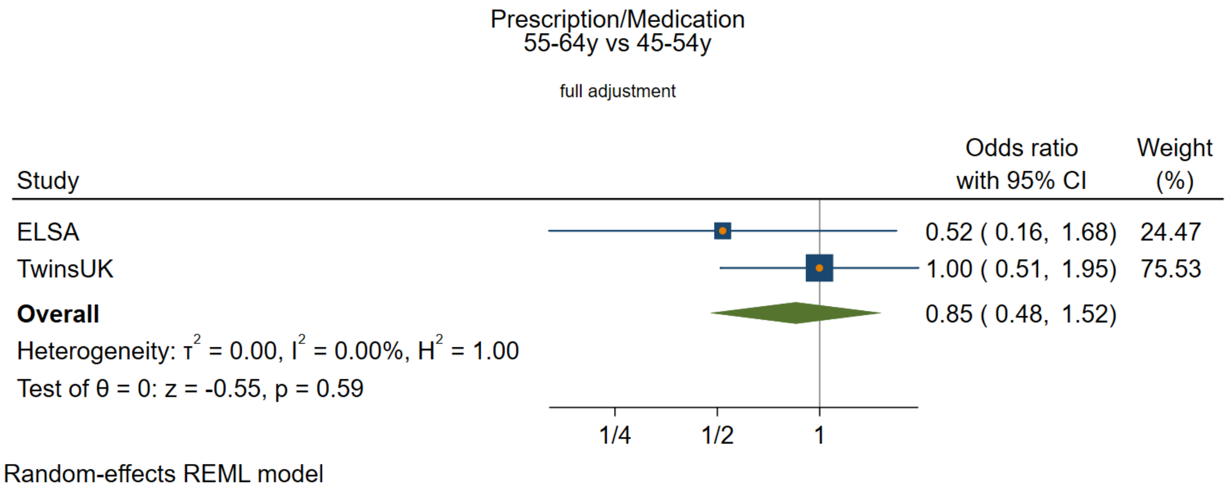

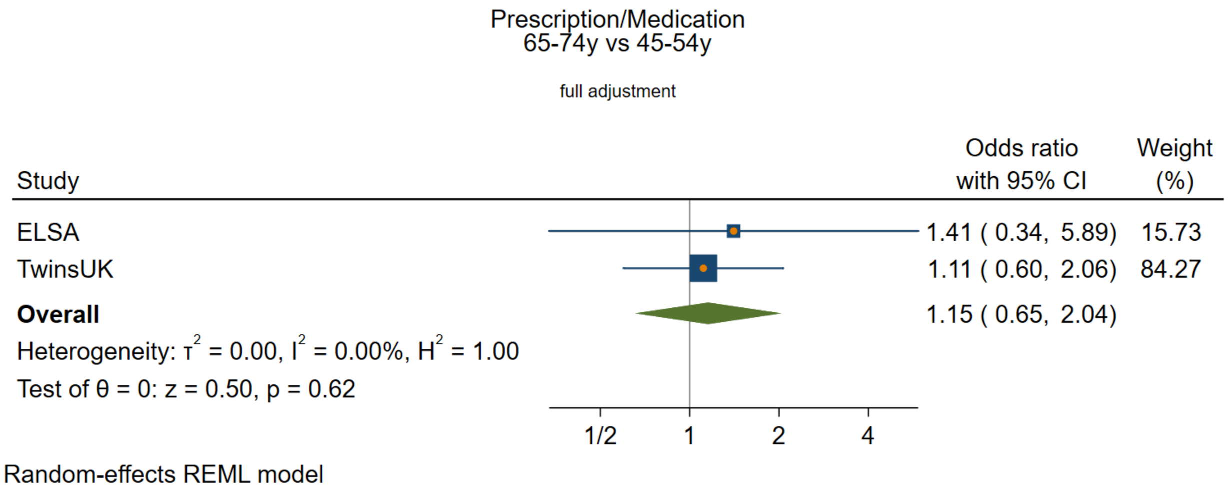

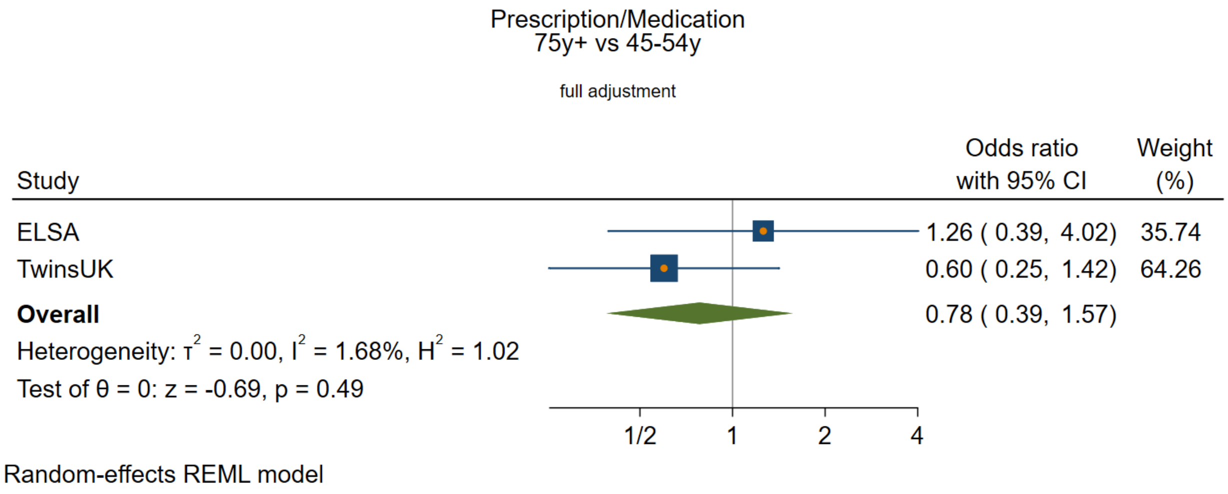

For peer review only - http://bmjopen.bmj.com/site/about/guidelines.xhtml

Ethnicity

Unadjusted

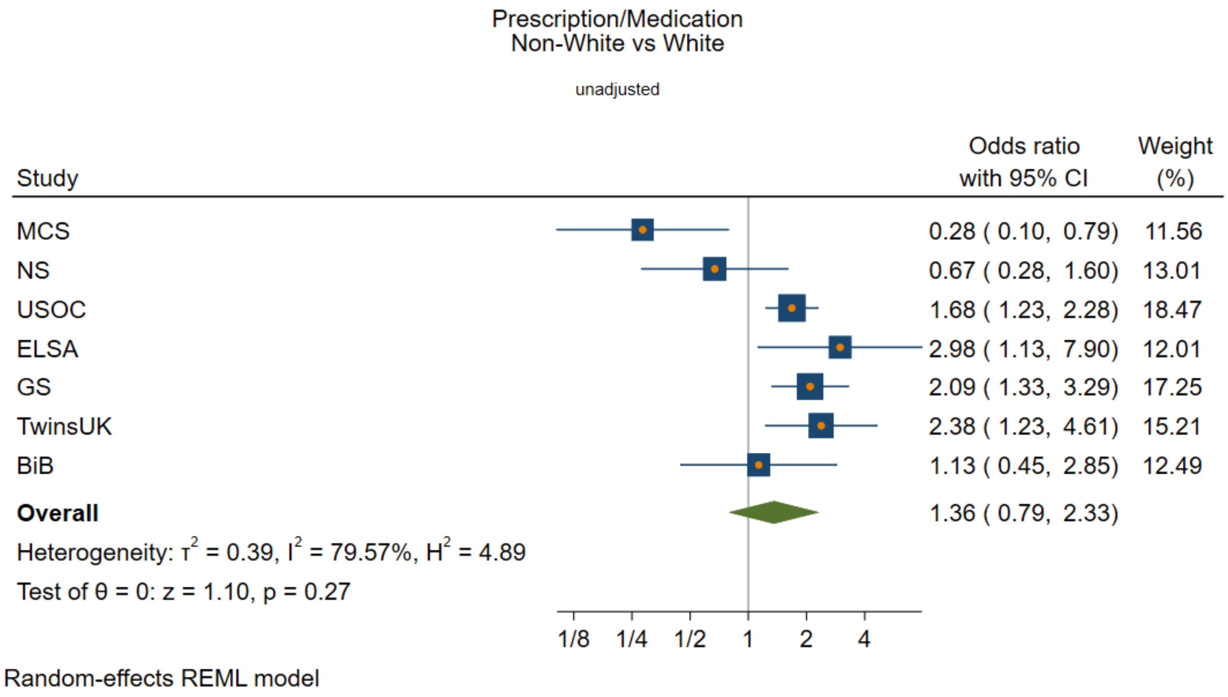

For peer review only - http://bmjopen.bmj.com/site/about/guidelines.xhtml

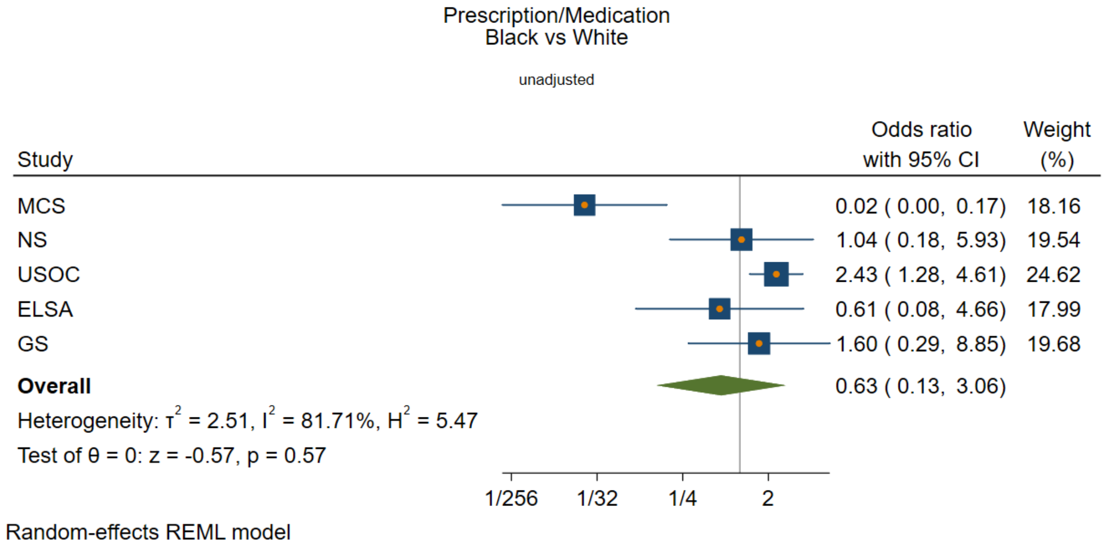

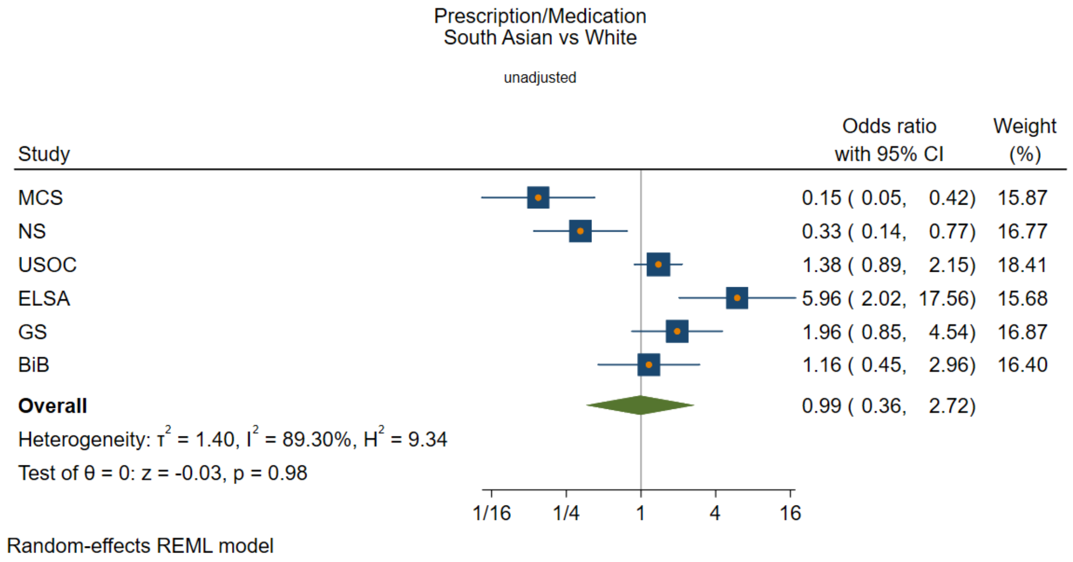

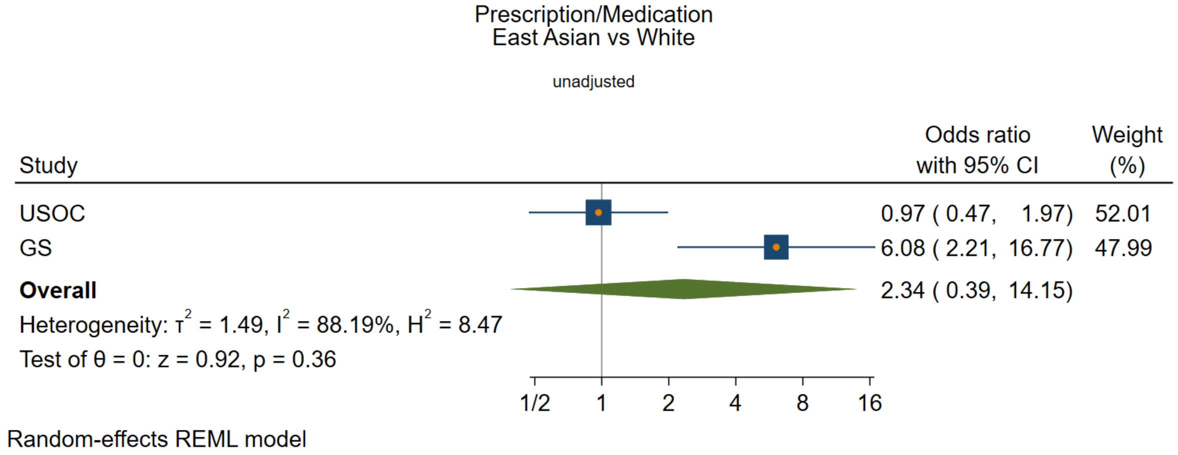

For peer review only - http://bmjopen.bmj.com/site/about/guidelines.xhtml

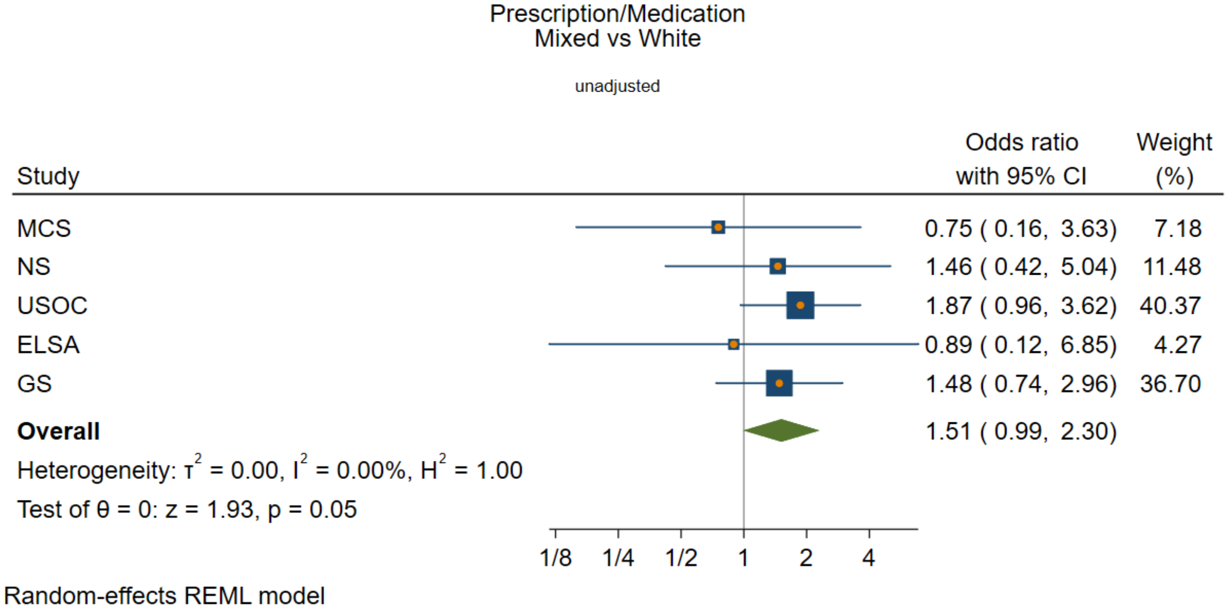

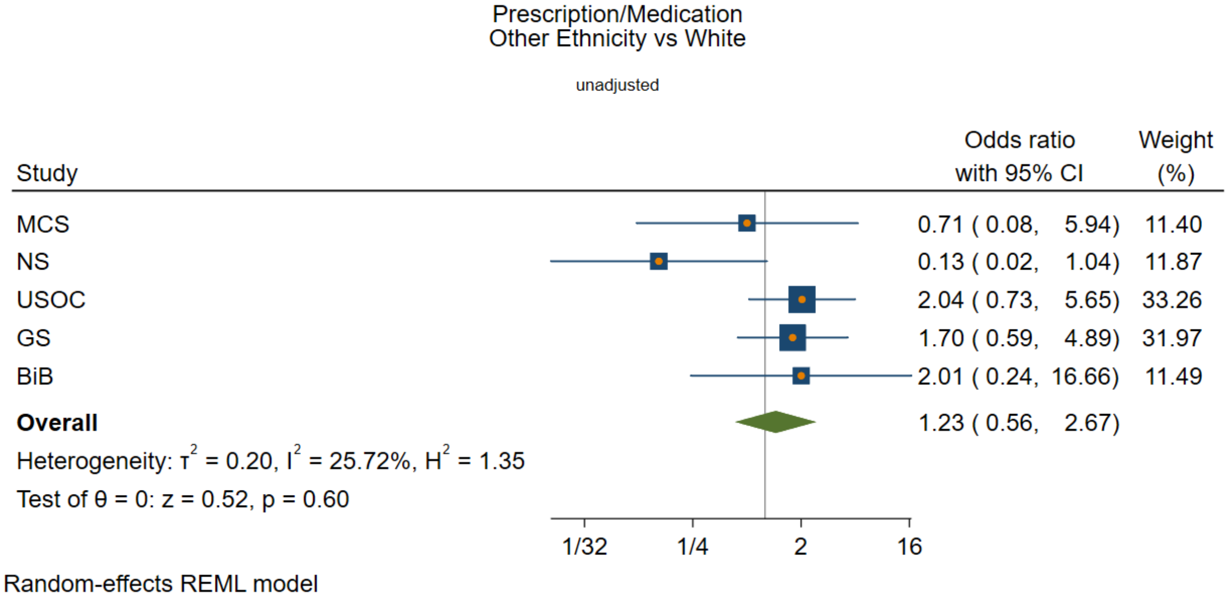

For peer review only - http://bmjopen.bmj.com/site/about/guidelines.xhtml

Basic adjustment

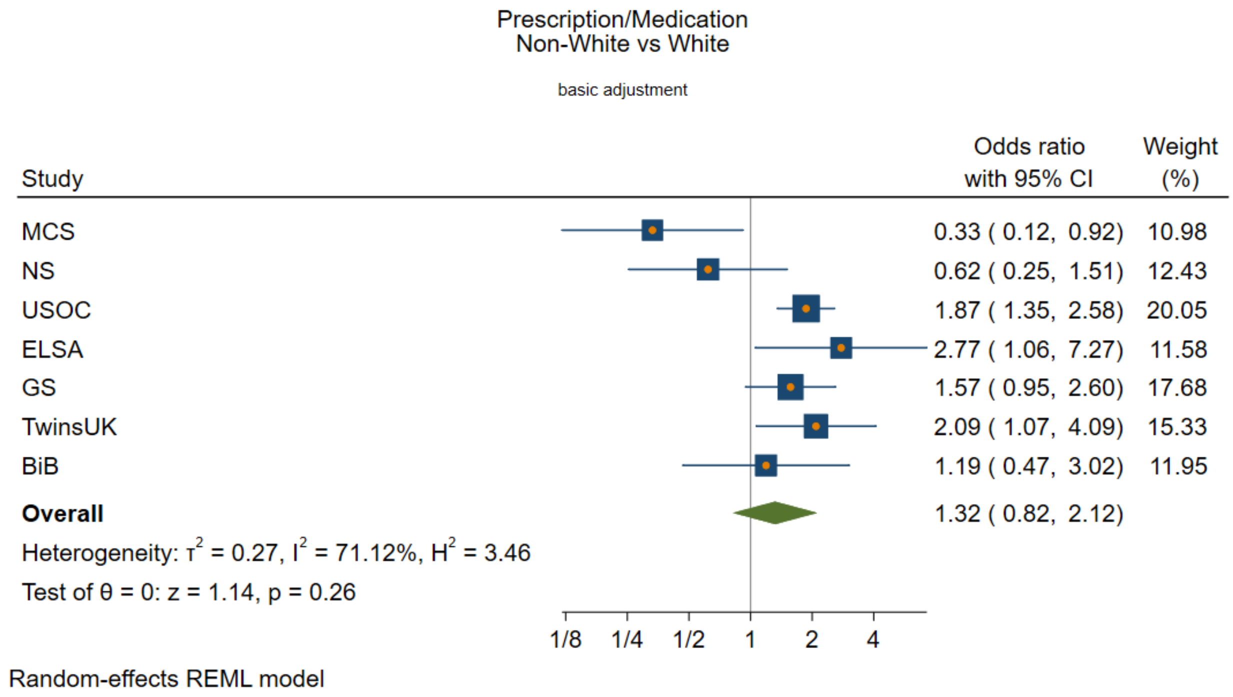

For peer review only - http://bmjopen.bmj.com/site/about/guidelines.xhtml

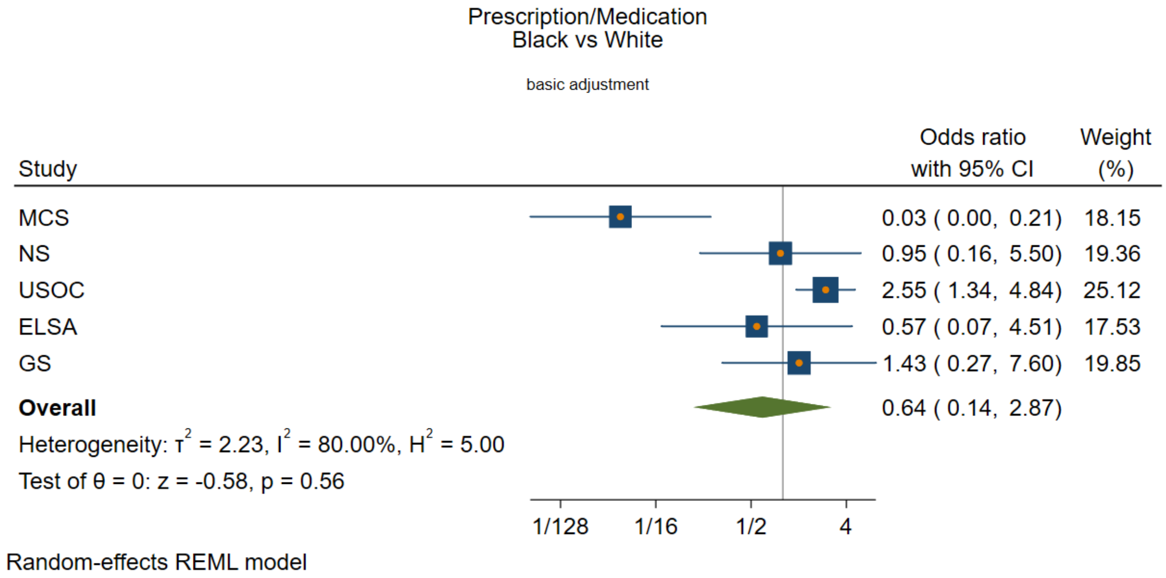

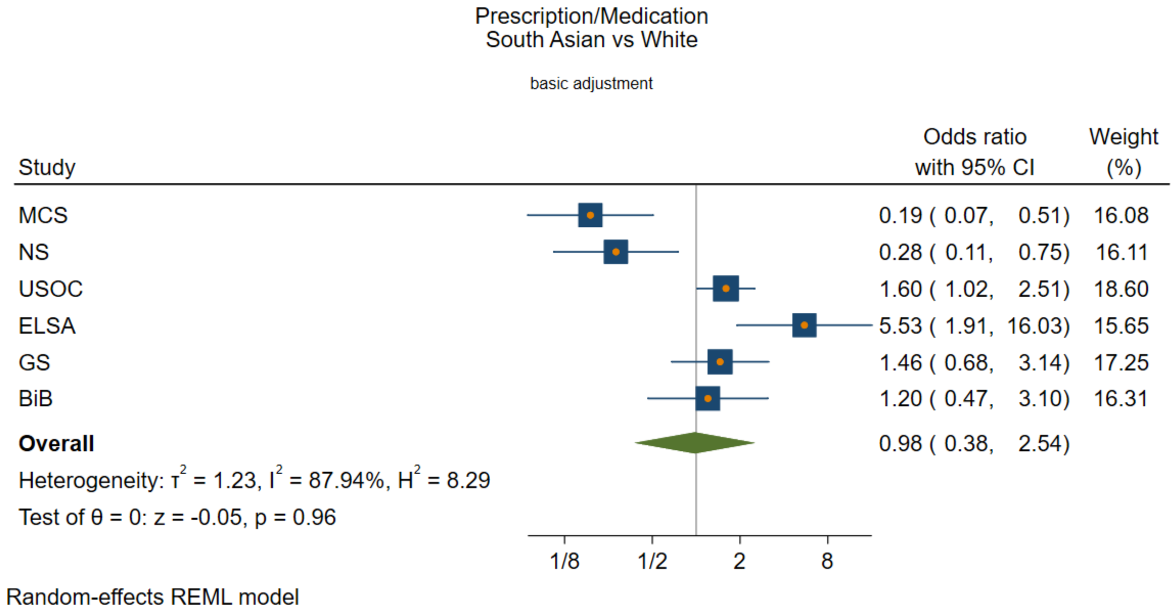

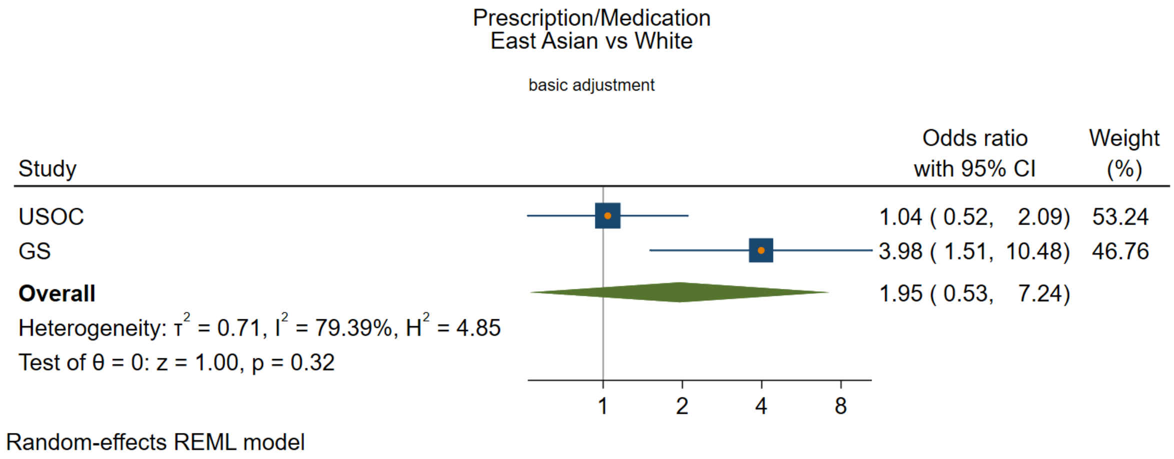

For peer review only - http://bmjopen.bmj.com/site/about/guidelines.xhtml

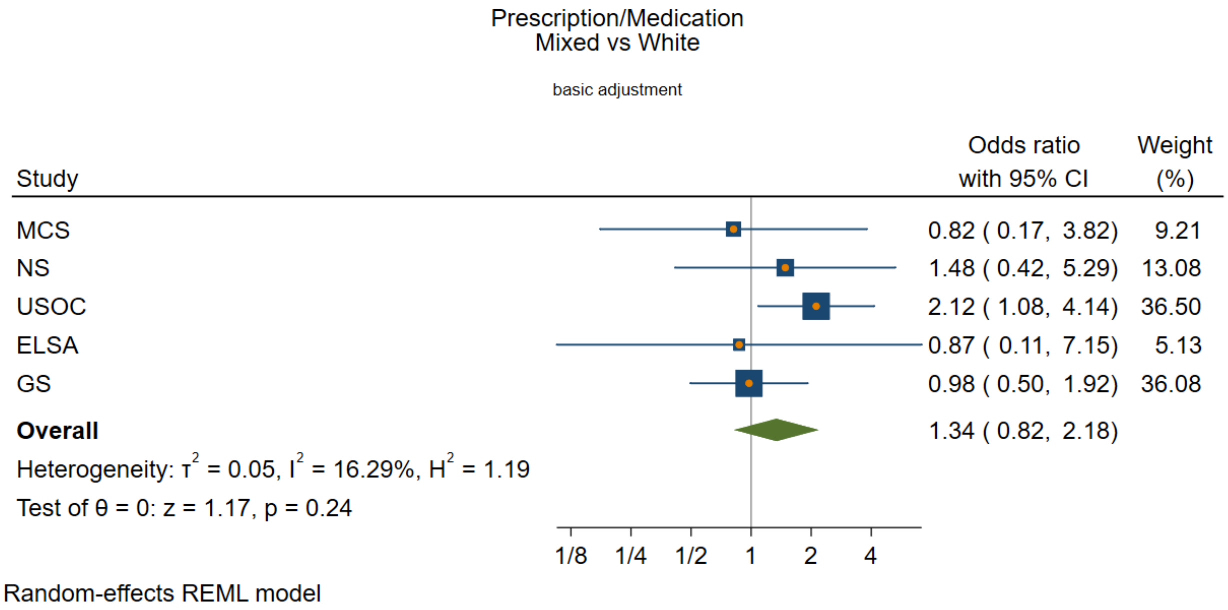
Prescription/Medication
Mixed vs White
basic adjustment
Study
Odds ratio with 95% CI
Weight (%)
MCS 0.82 (0.17, 3.82) 9.21
NS 1.48 (0.42, 5.29) 13.08
USOC 2.12 (1.08, 4.14) 36.50
ELSA 0.87 (0.11, 7.15) 5.13
GS 0.98 (0.50, 1.92) 36.08
Overall 1.34 (0.82, 2.18)
Heterogeneity: τ² = 0.05, I² = 16.29%, H² = 1.19
Test of θ = 0: z = 1.17, p = 0.24
1/8 1/4 1/2 1 2 4
Random-effects REML model

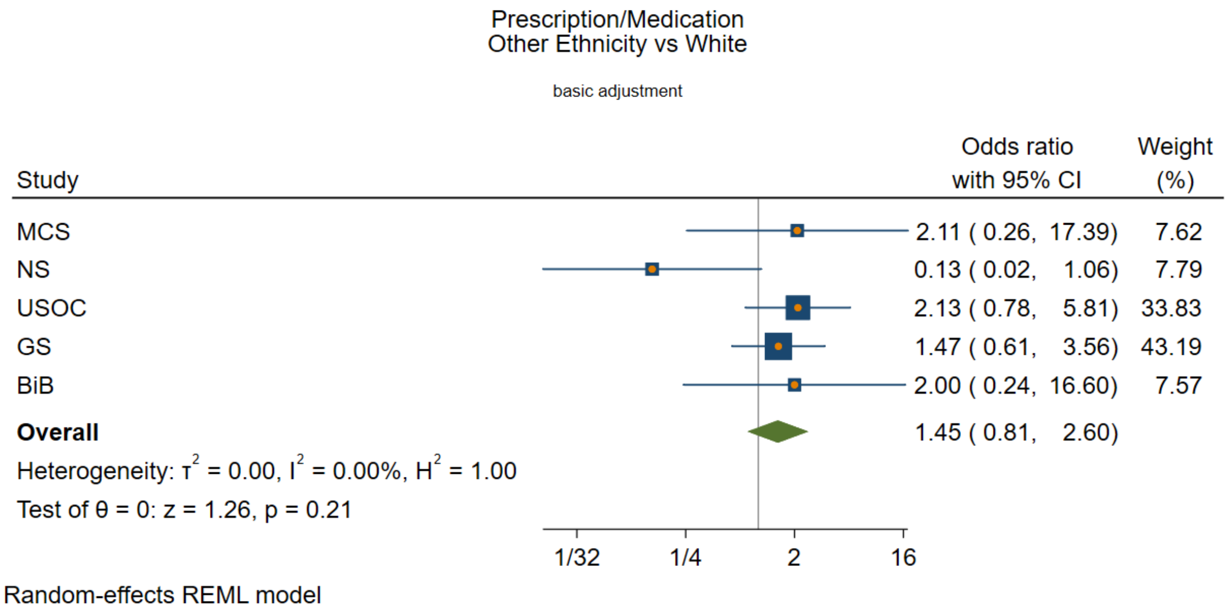
Prescription/Medication
Other Ethnicity vs White
basic adjustment
Study
Odds ratio with 95% CI
Weight (%)
MCS 2.11 (0.26, 17.39) 7.62
NS 0.13 (0.02, 1.06) 7.79
USOC 2.13 (0.78, 5.81) 33.83
GS 1.47 (0.61, 3.56) 43.19
BiB 2.00 (0.24, 16.60) 7.57
Overall 1.45 (0.81, 2.60)
Heterogeneity: τ² = 0.00, I² = 0.00%, H² = 1.00
Test of θ = 0: z = 1.26, p = 0.21
1/32 1/4 2 16
Random-effects REML model

Full adjustment

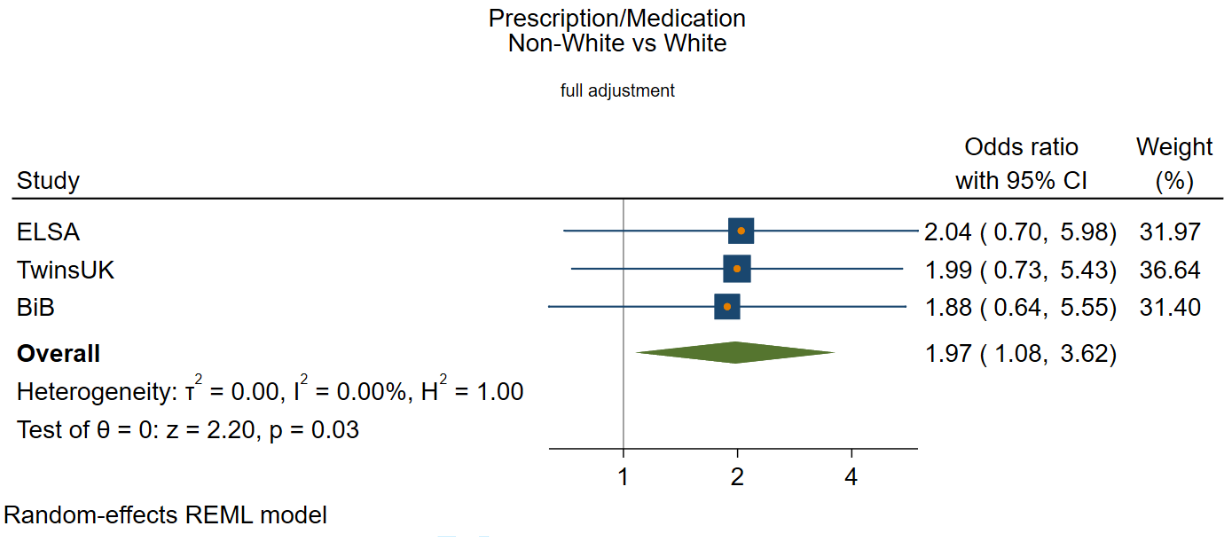

For peer review only - http://bmjopen.bmj.com/site/about/guidelines.xhtml

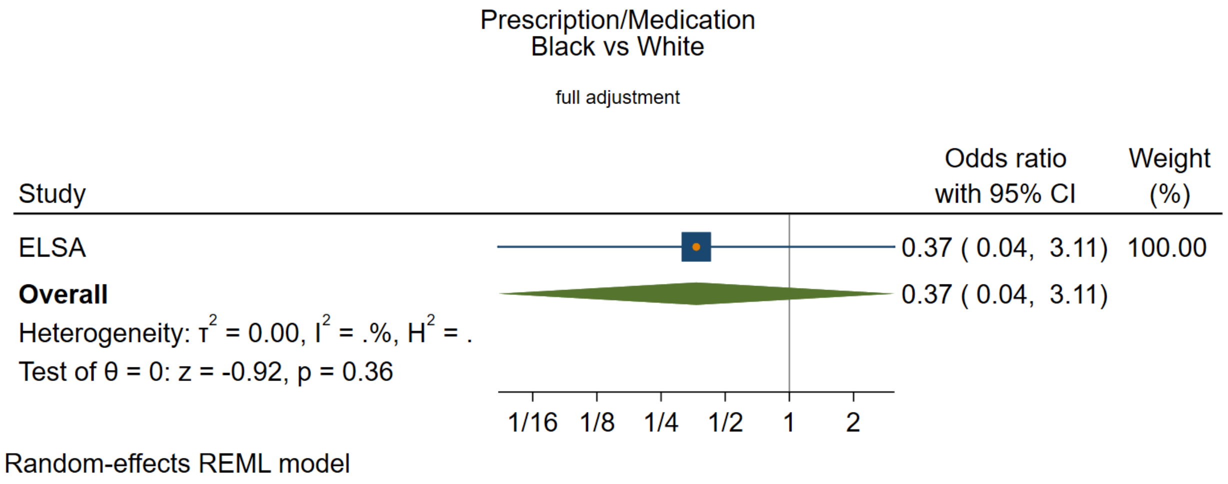

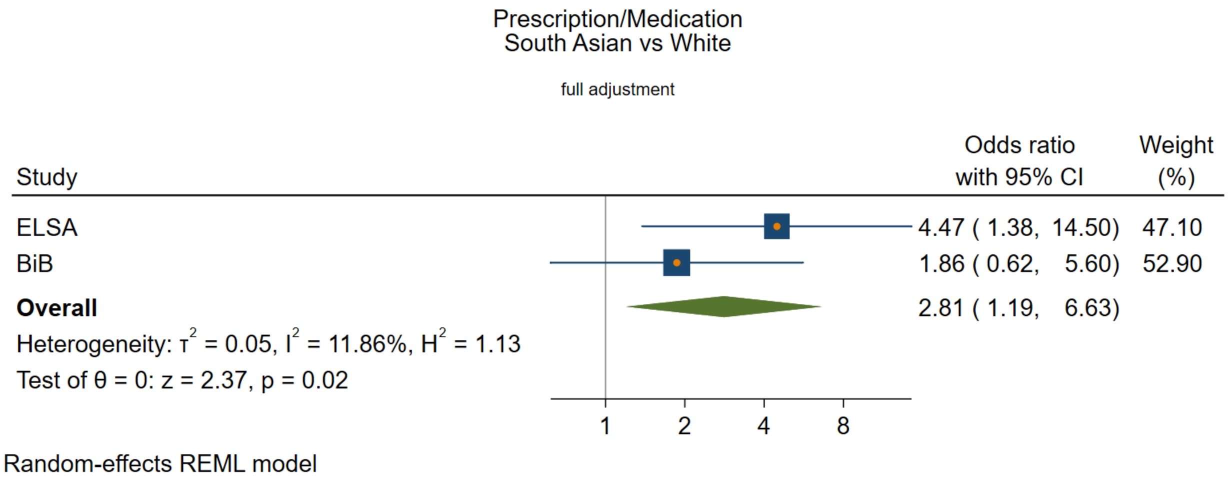

For peer review only - http://bmjopen.bmj.com/site/about/guidelines.xhtml

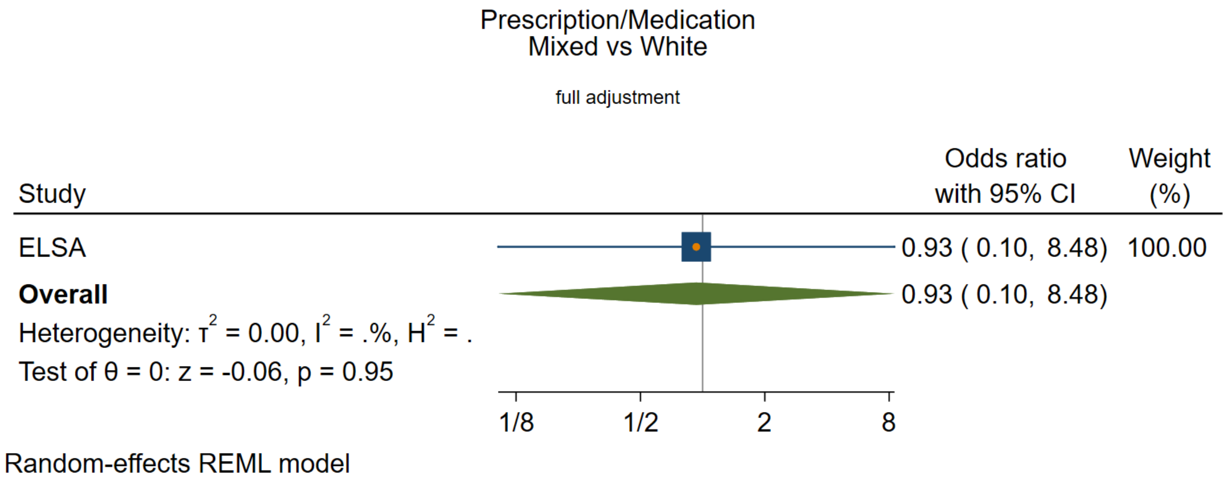

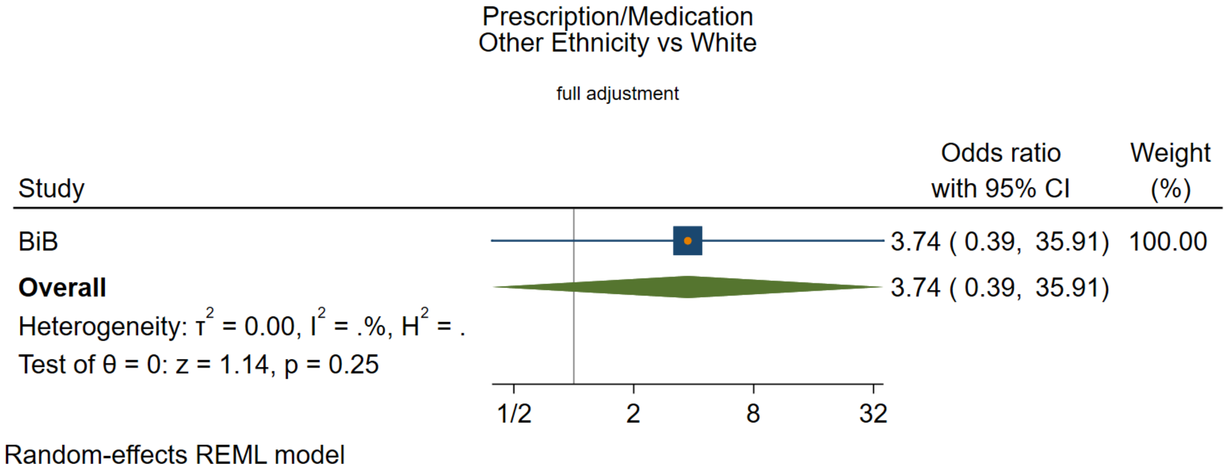

For peer review only - http://bmjopen.bmj.com/site/about/guidelines.xhtml

Procedures/surgery

Sex
Unadjusted

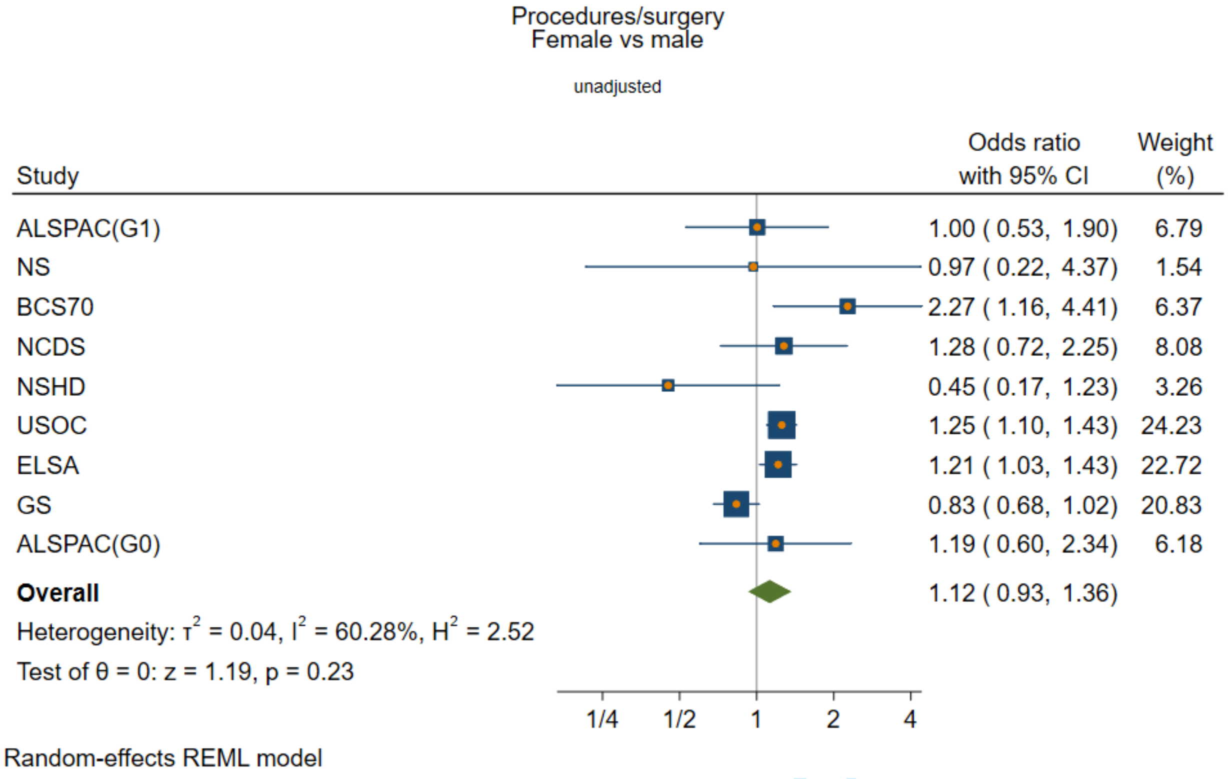

For peer review only - http://bmjopen.bmj.com/site/about/guidelines.xhtml

Basic adjustment

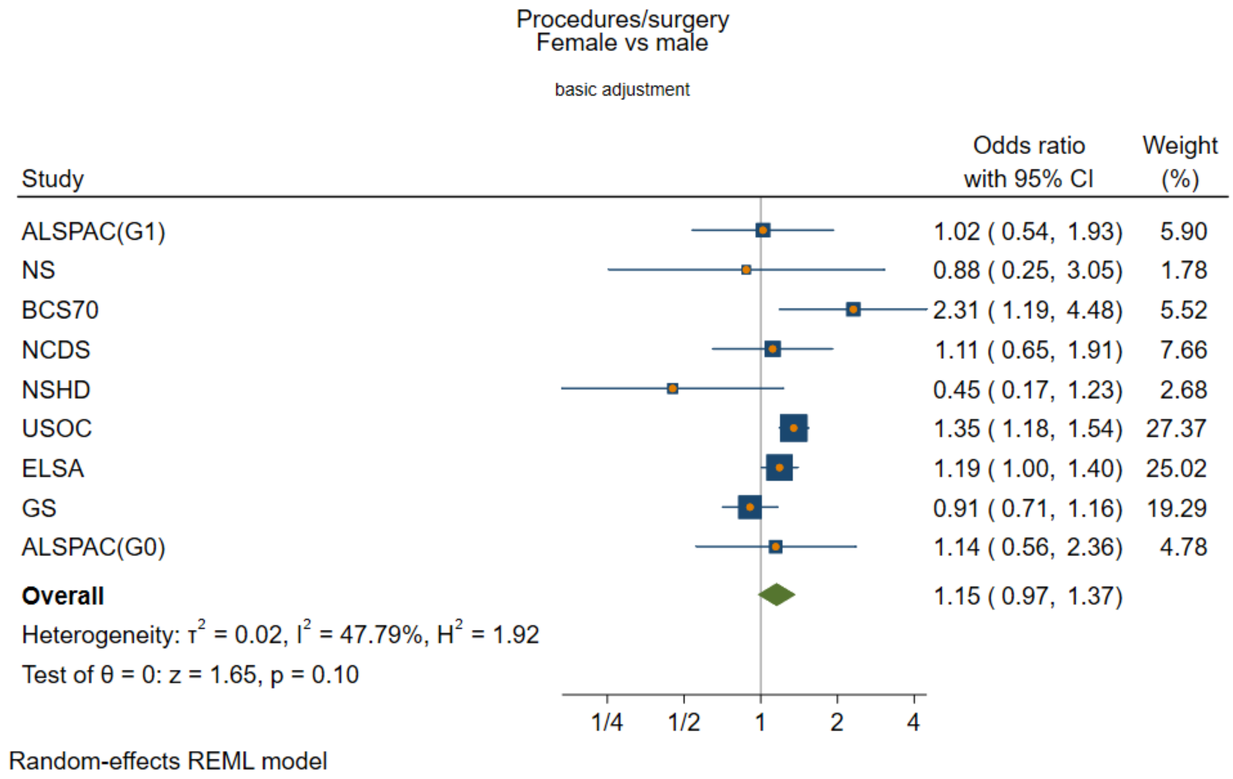

For peer review only - http://bmjopen.bmj.com/site/about/guidelines.xhtml

Full adjustment

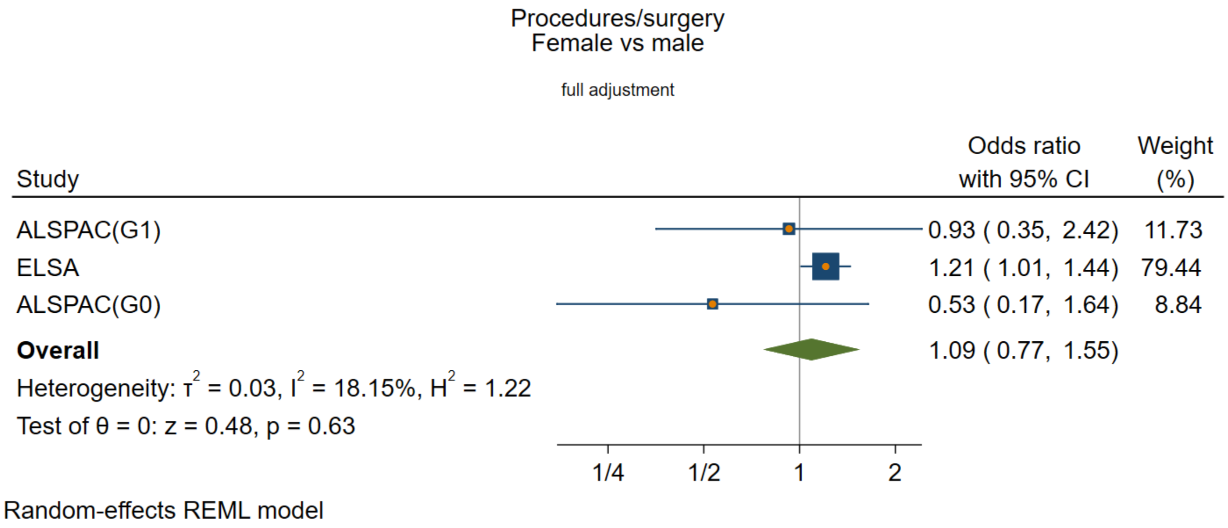

For peer review only

Occupational class
Unadjusted

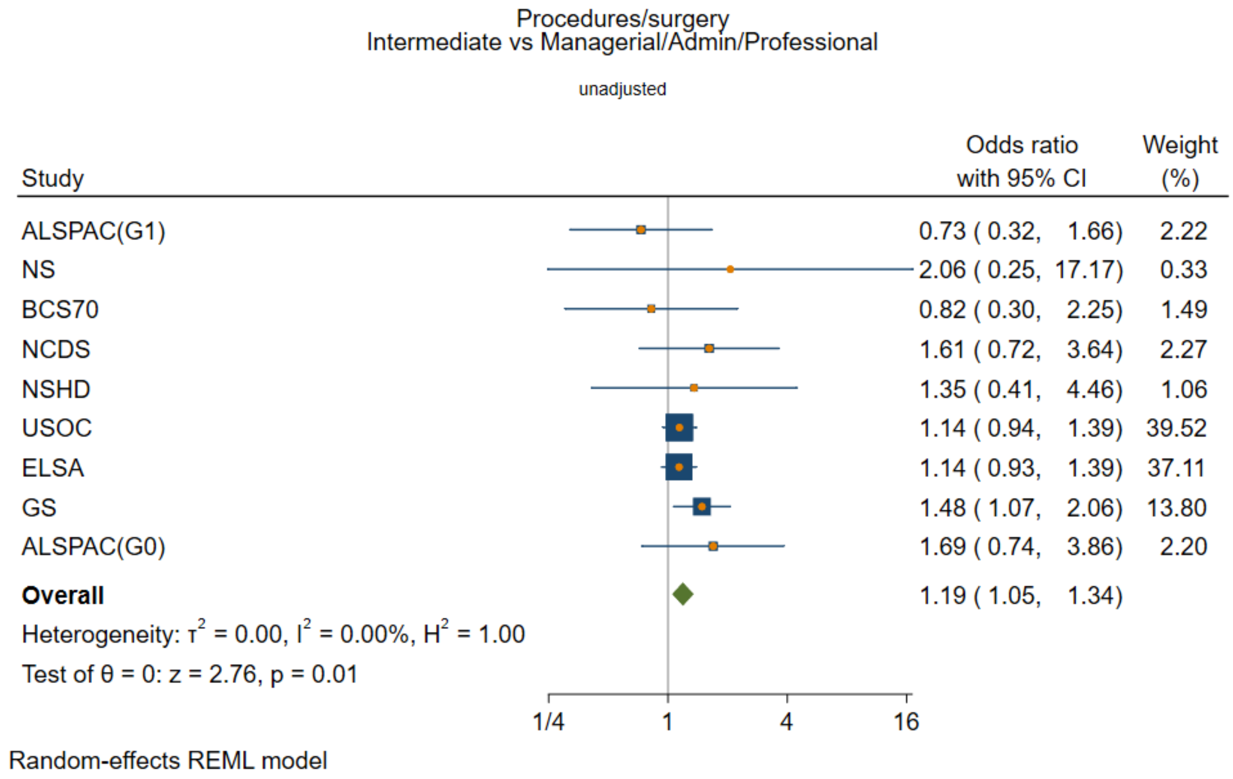

review only

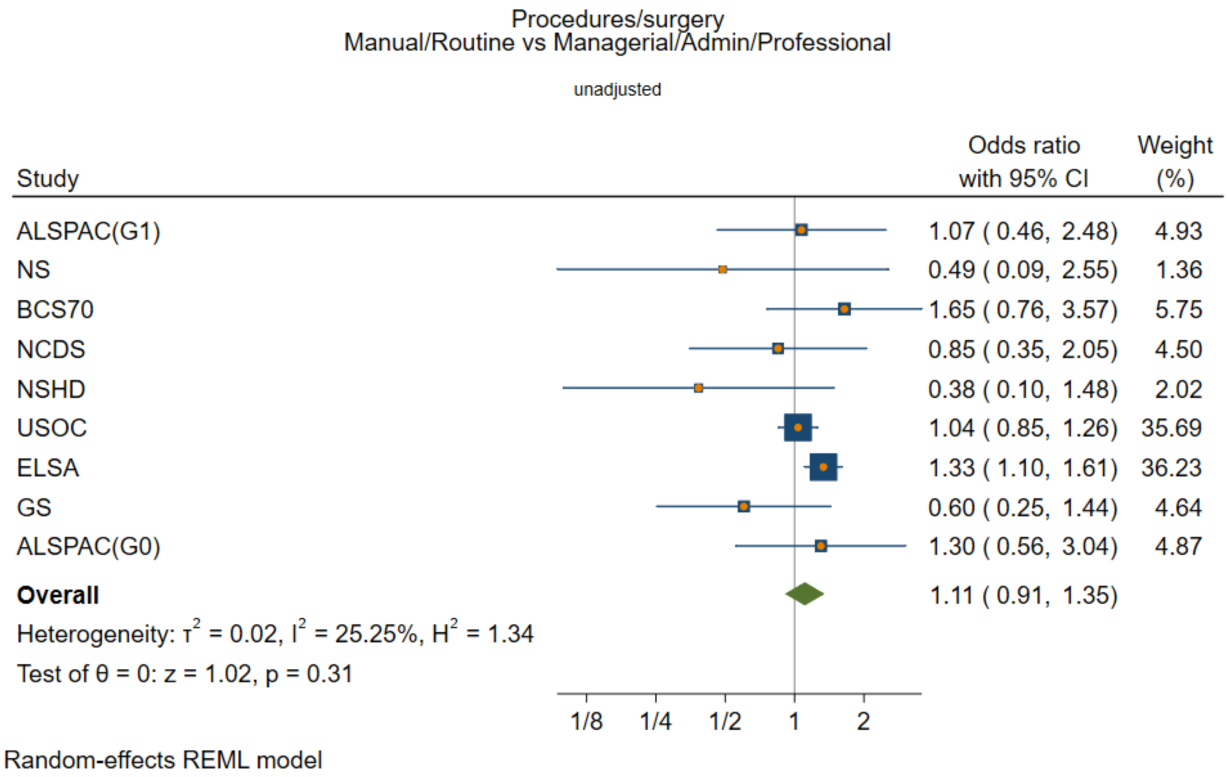

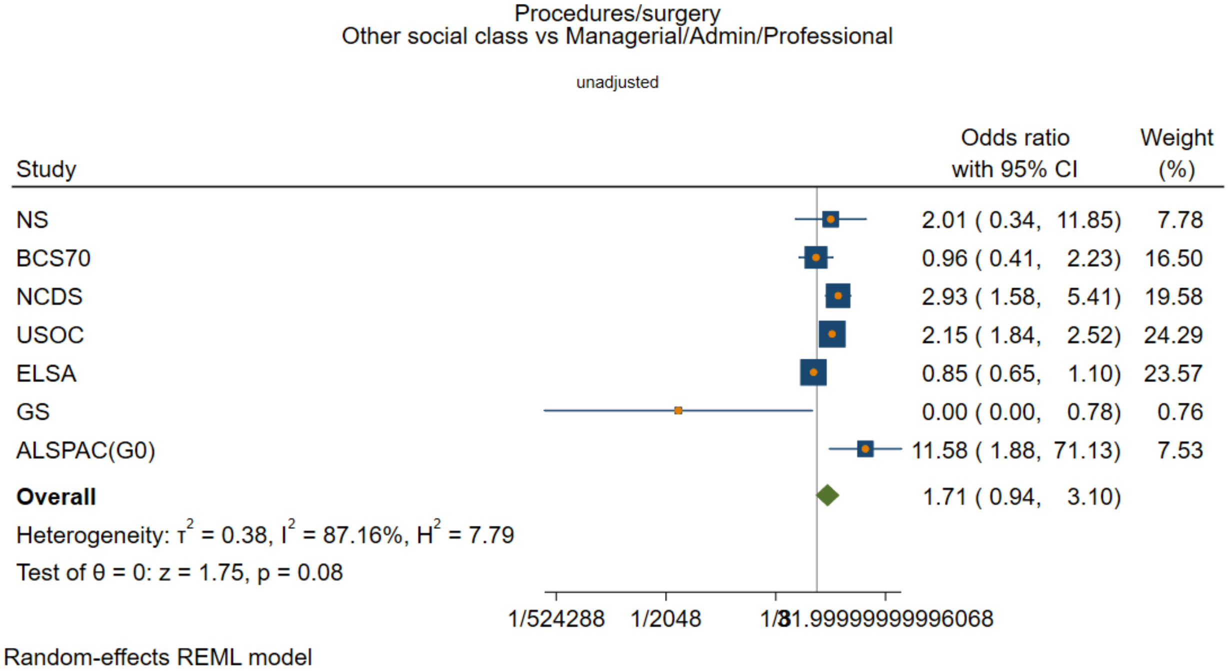

For peer review only - http://bmjopen.bmj.com/site/about/guidelines.xhtml

Basic adjustment

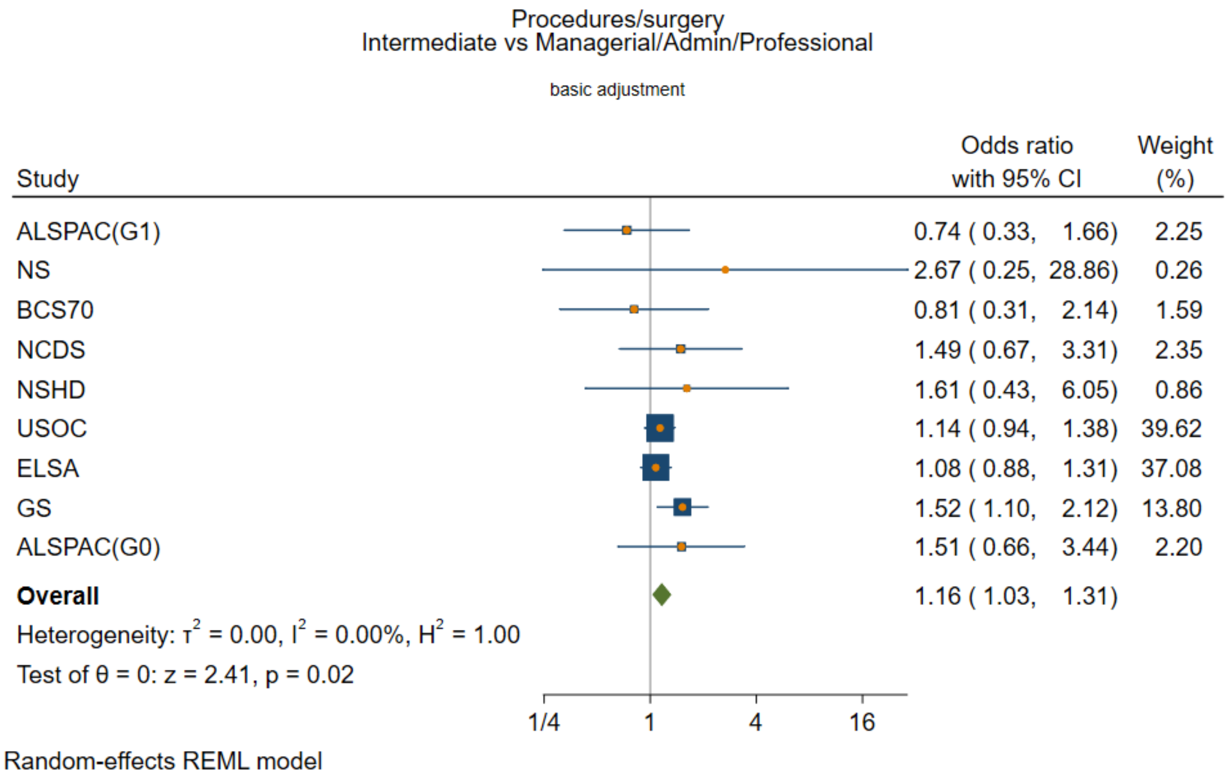

view only

For peer review only - http://bmjopen.bmj.com/site/about/guidelines.xhtml

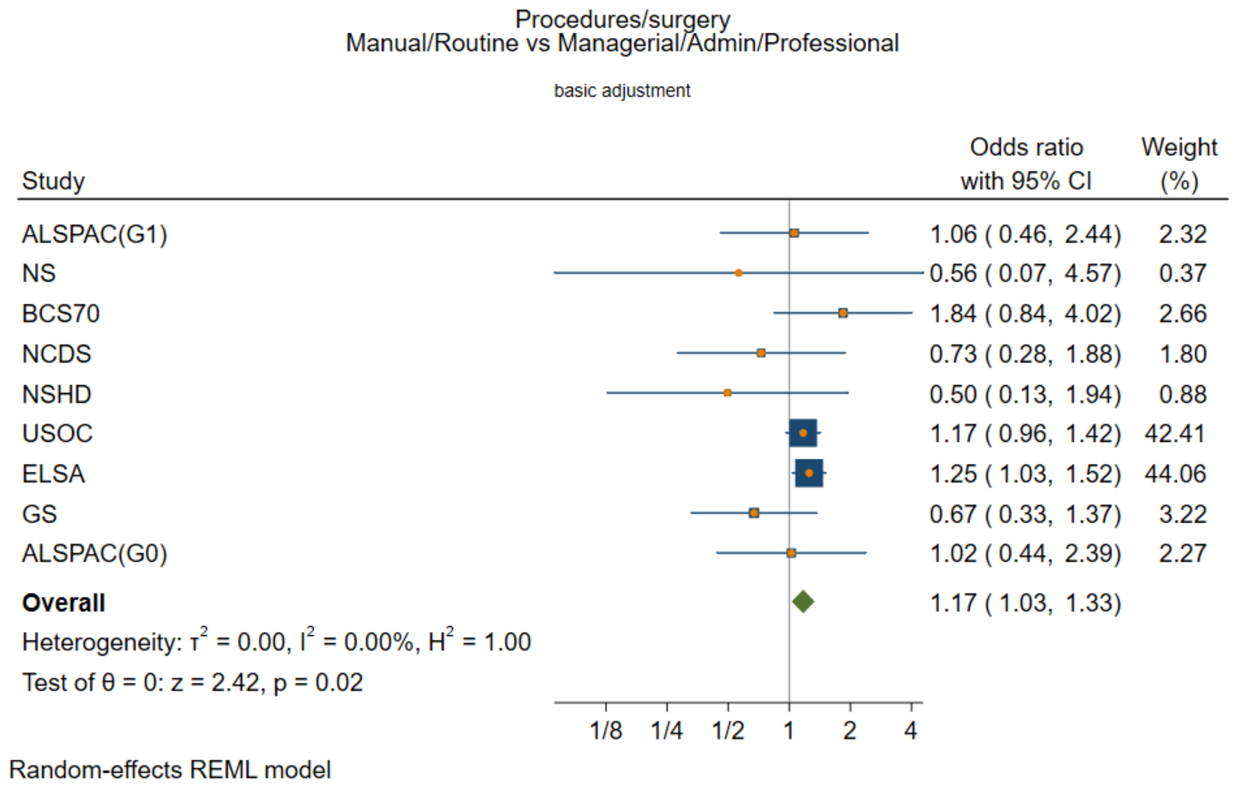

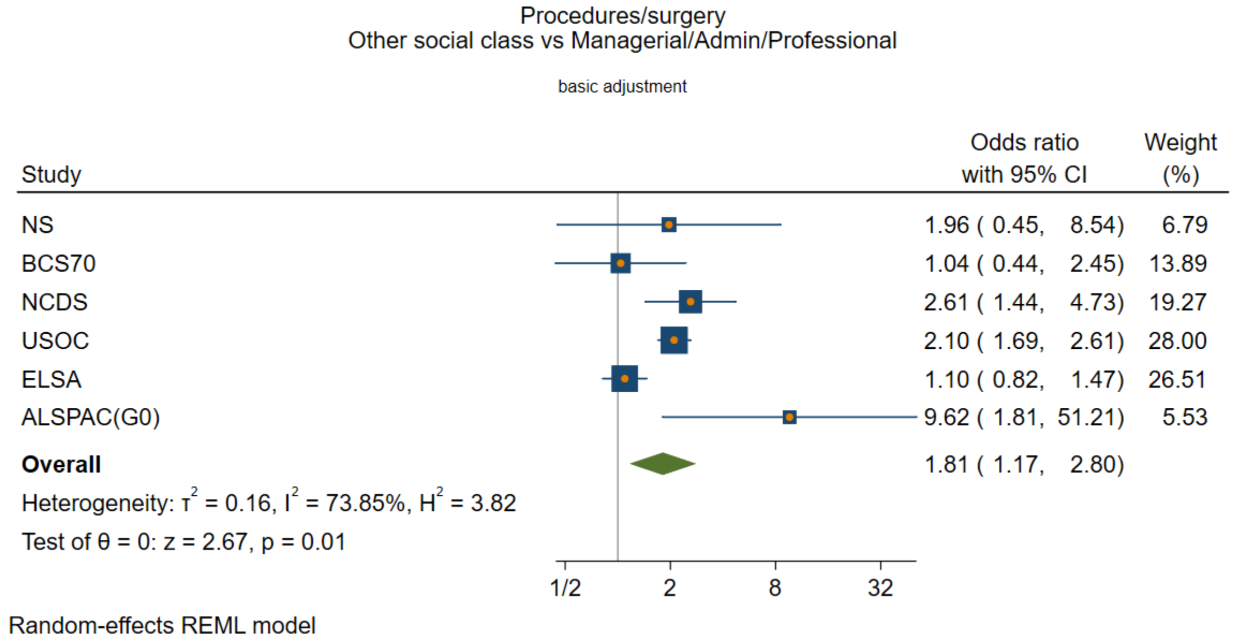

For peer review only - http://bmjopen.bmj.com/site/about/guidelines.xhtml

Full adjustment

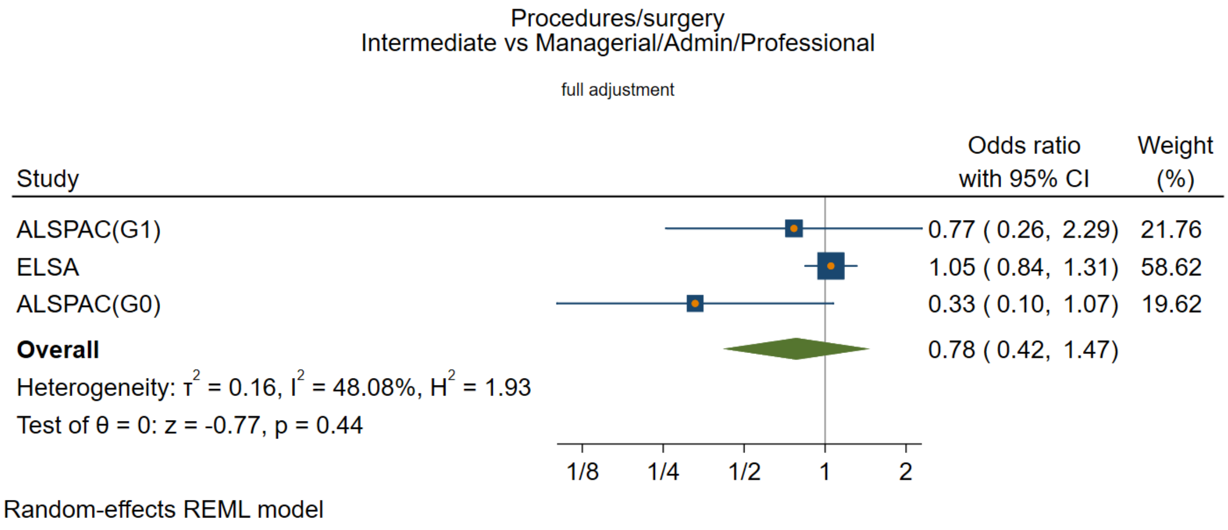

For peer review only

For peer review only - http://bmjopen.bmj.com/site/about/guidelines.xhtml

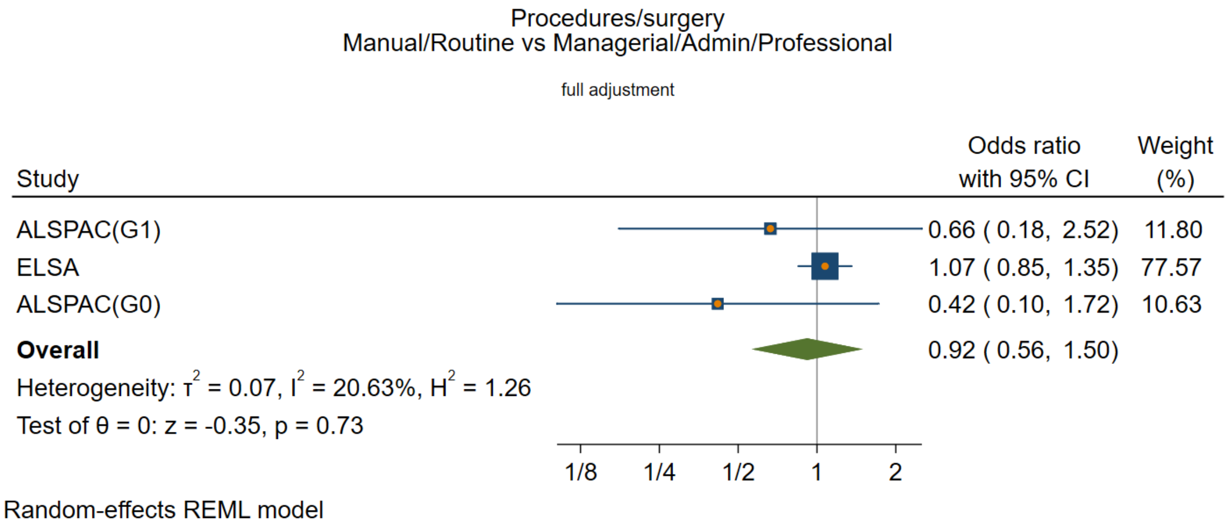

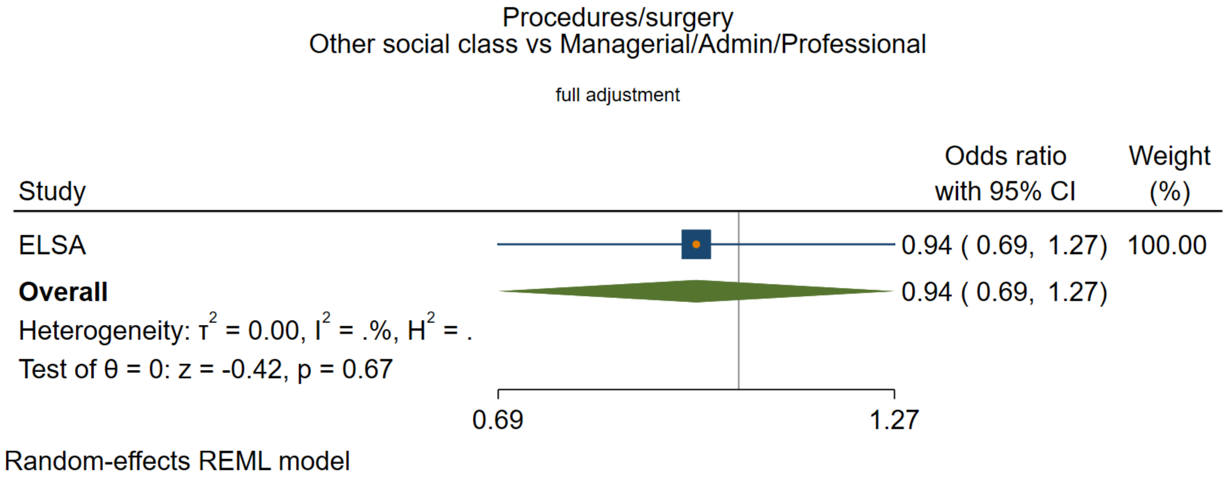

For peer review only - http://bmjopen.bmj.com/site/about/guidelines.xhtml

Education
<u>unadjusted</u>

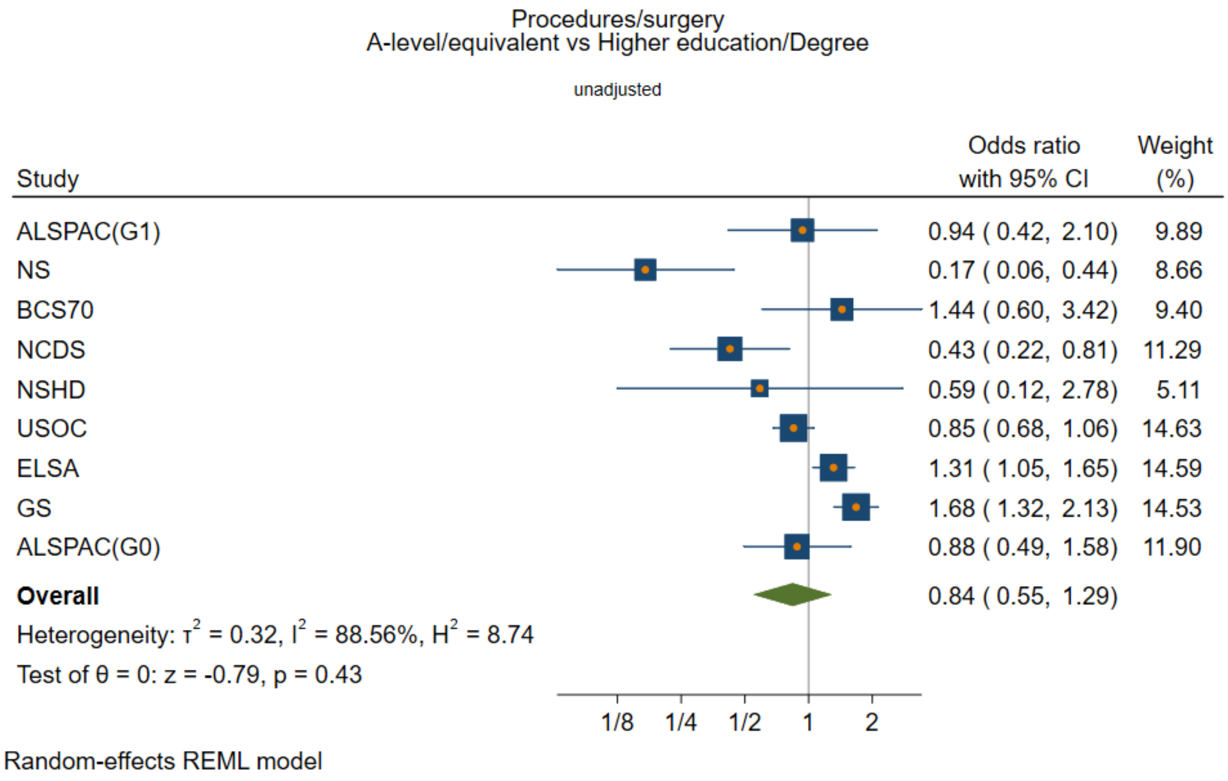

For peer review only - http://bmjopen.bmj.com/site/about/guidelines.xhtml

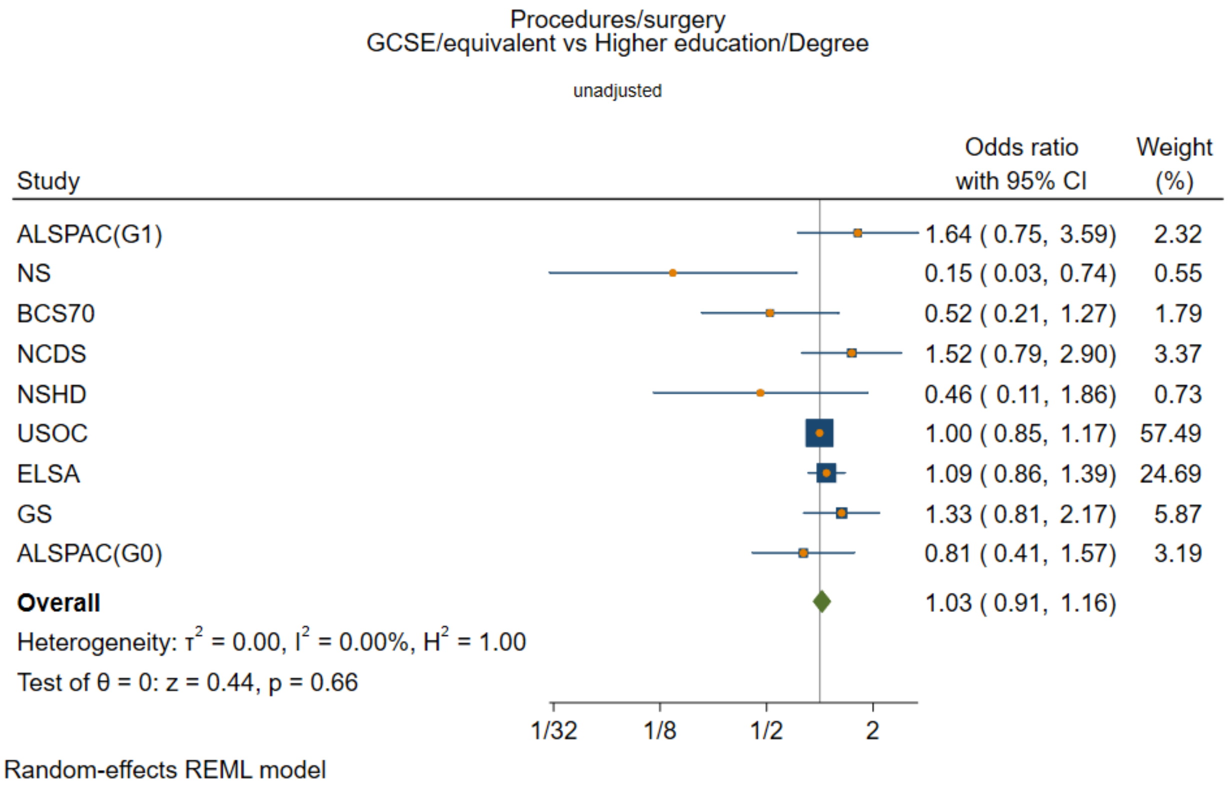

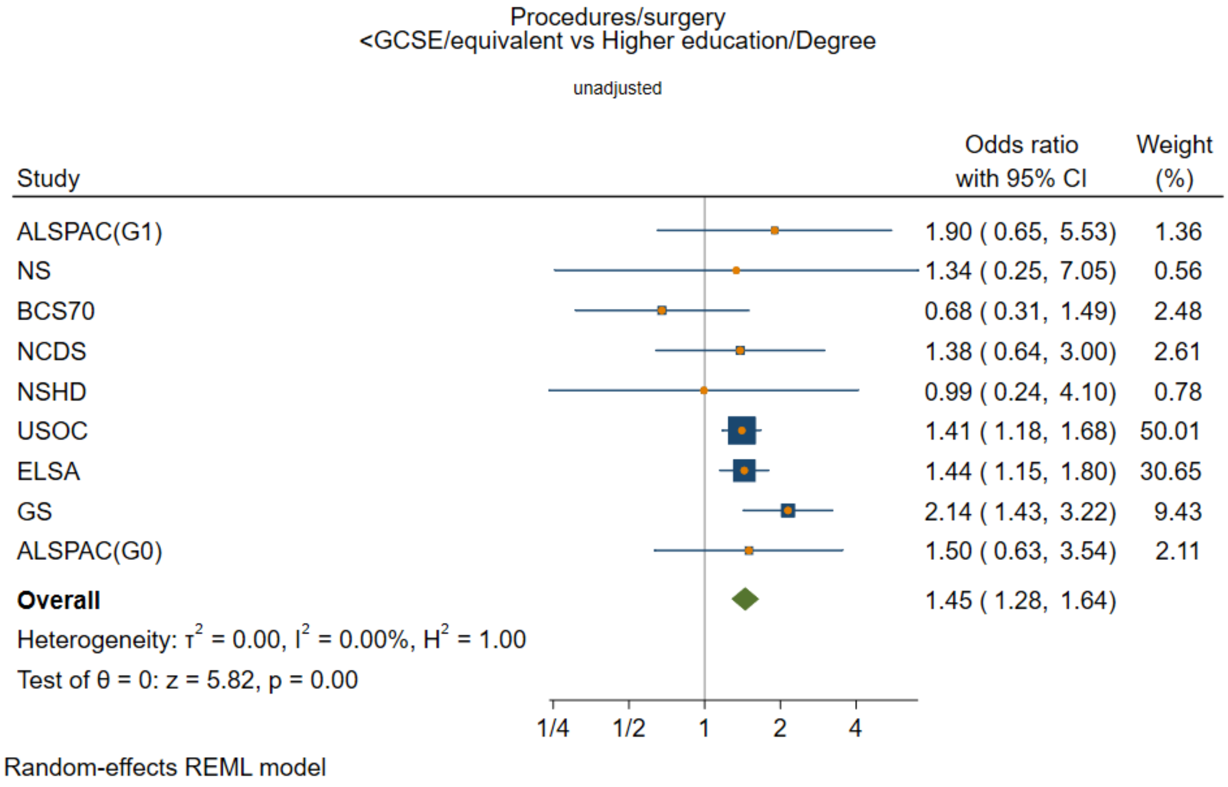

For peer review only - http://bmjopen.bmj.com/site/about/guidelines.xhtml

Basic adjustment

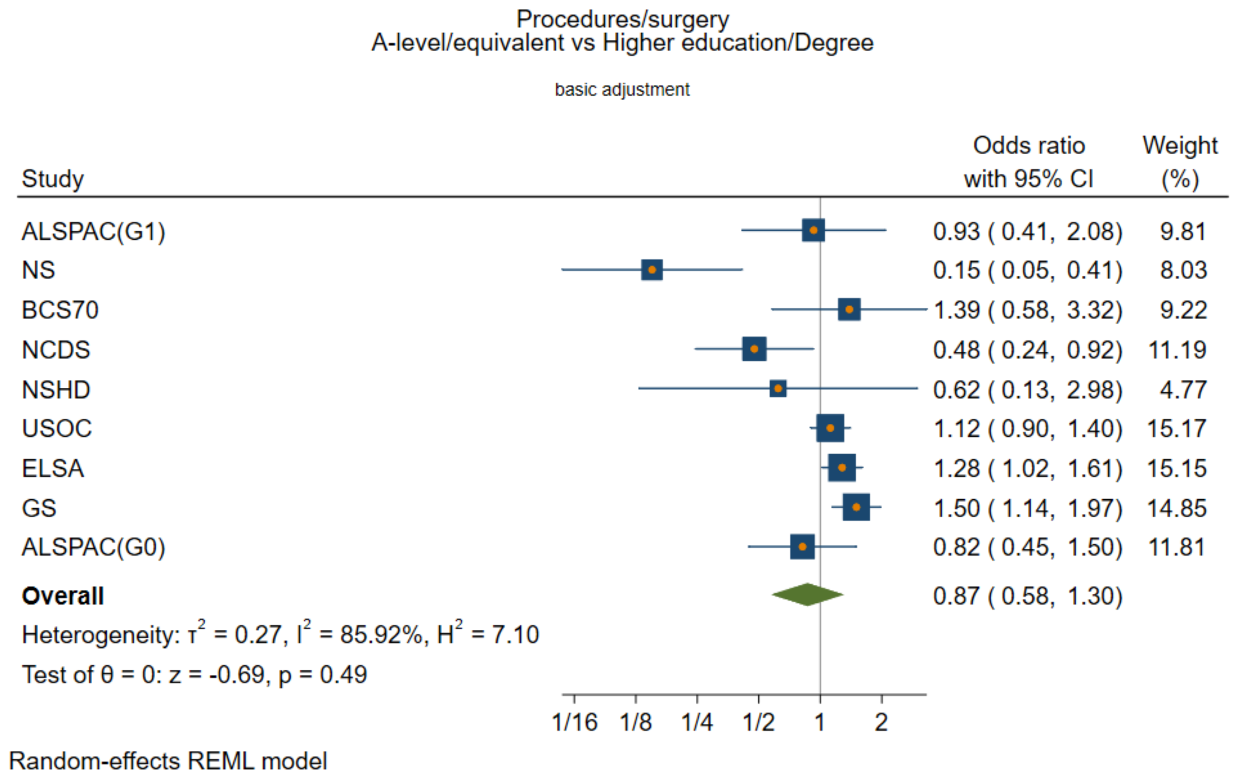

view only

For peer review only - http://bmjopen.bmj.com/site/about/guidelines.xhtml

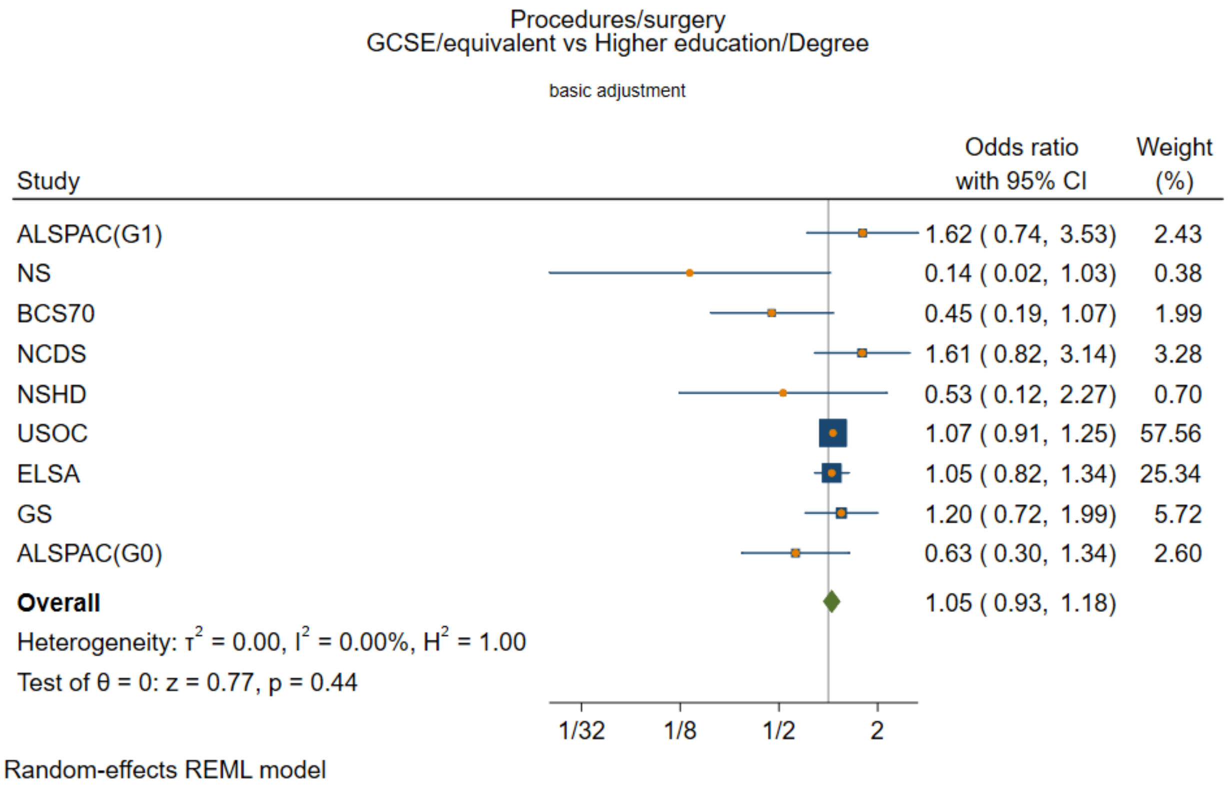
Procedures/surgery
GCSE/equivalent vs Higher education/Degree
basic adjustment
Study
Odds ratio with 95% CI
Weight (%)
ALSPAC(G1) 1.62 (0.74, 3.53) 2.43
NS 0.14 (0.02, 1.03) 0.38
BCS70 0.45 (0.19, 1.07) 1.99
NCDS 1.61 (0.82, 3.14) 3.28
NSHD 0.53 (0.12, 2.27) 0.70
USOC 1.07 (0.91, 1.25) 57.56
ELSA 1.05 (0.82, 1.34) 25.34
GS 1.20 (0.72, 1.99) 5.72
ALSPAC(G0) 0.63 (0.30, 1.34) 2.60
Overall 1.05 (0.93, 1.18)
Heterogeneity: τ² = 0.00, I² = 0.00%, H² = 1.00
Test of θ = 0: z = 0.77, p = 0.44
1/32
1/8
1/2
2
Random-effects REML model

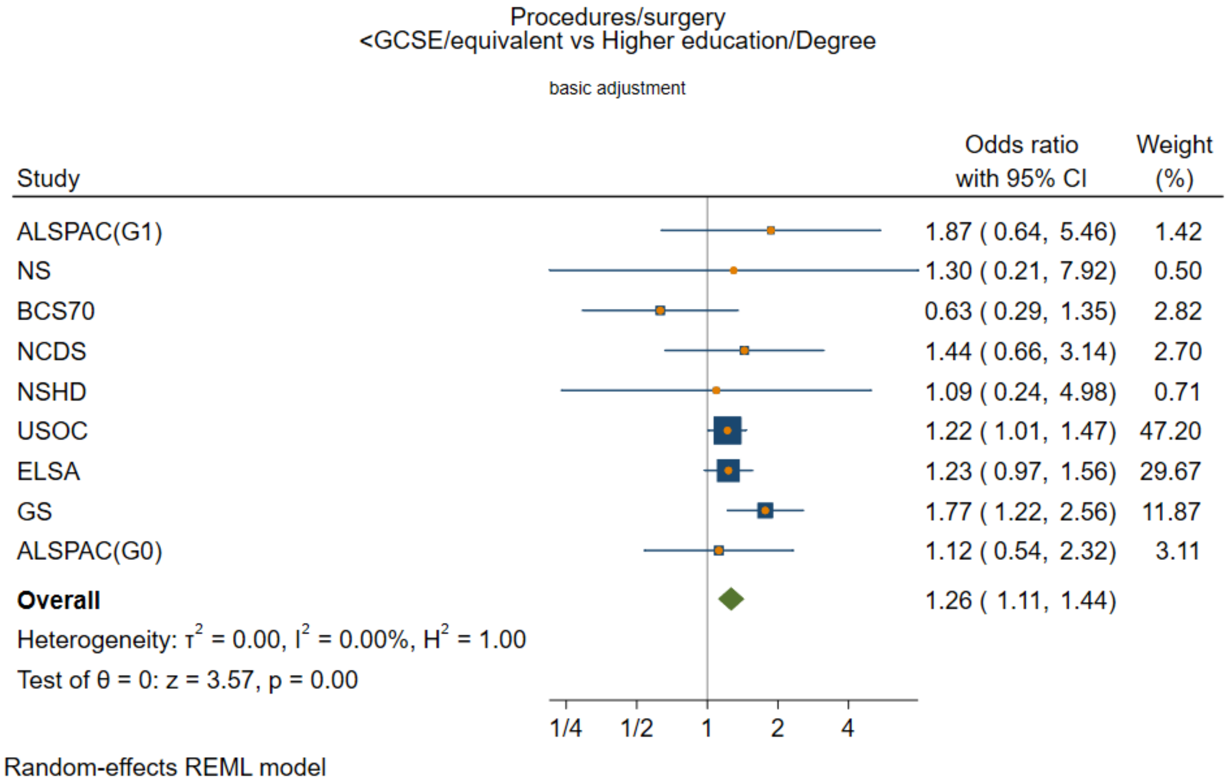
Procedures/surgery
<GCSE/equivalent vs Higher education/Degree
basic adjustment
Study
Odds ratio with 95% CI
Weight (%)
ALSPAC(G1) 1.87 (0.64, 5.46) 1.42
NS 1.30 (0.21, 7.92) 0.50
BCS70 0.63 (0.29, 1.35) 2.82
NCDS 1.44 (0.66, 3.14) 2.70
NSHD 1.09 (0.24, 4.98) 0.71
USOC 1.22 (1.01, 1.47) 47.20
ELSA 1.23 (0.97, 1.56) 29.67
GS 1.77 (1.22, 2.56) 11.87
ALSPAC(G0) 1.12 (0.54, 2.32) 3.11
Overall 1.26 (1.11, 1.44)
Heterogeneity: τ² = 0.00, I² = 0.00%, H² = 1.00
Test of θ = 0: z = 3.57, p = 0.00
1/4
1/2
1
2
4
Random-effects REML model

Full adjustment

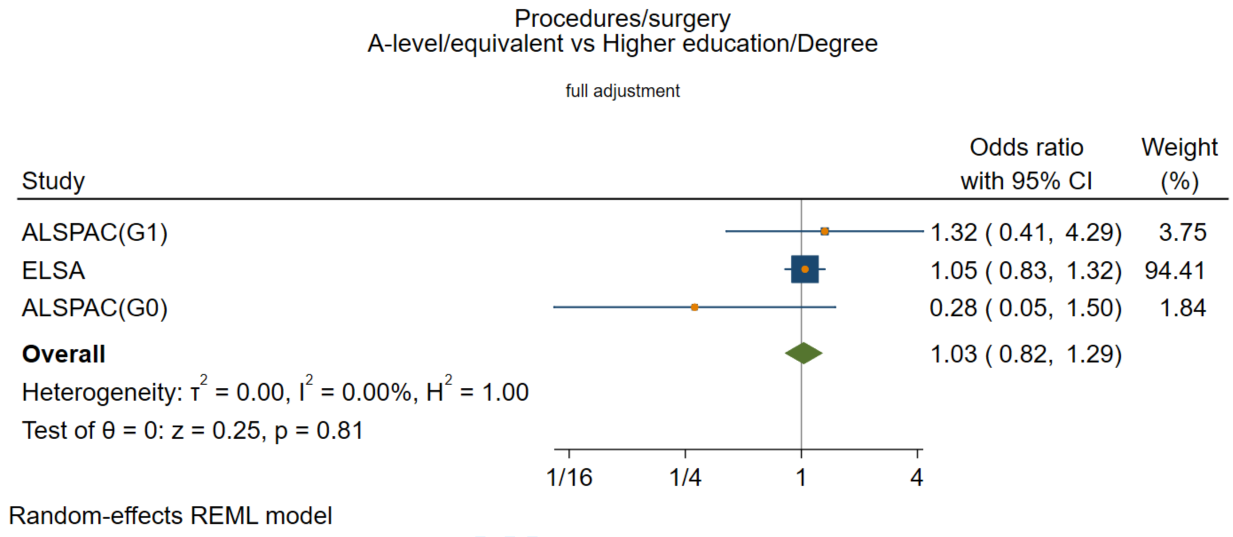

For peer review only - http://bmjopen.bmj.com/site/about/guidelines.xhtml

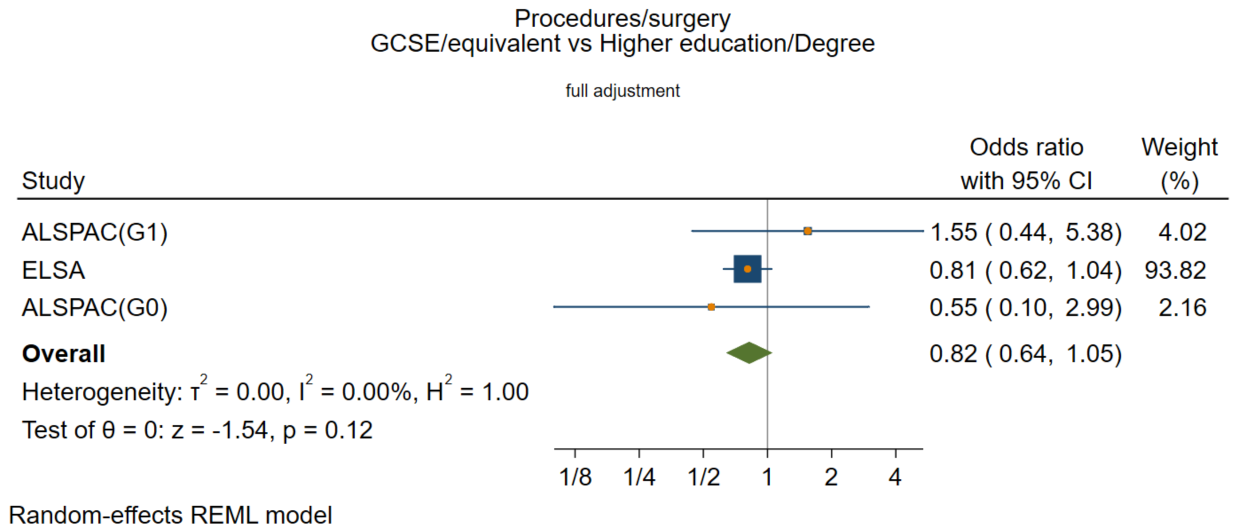

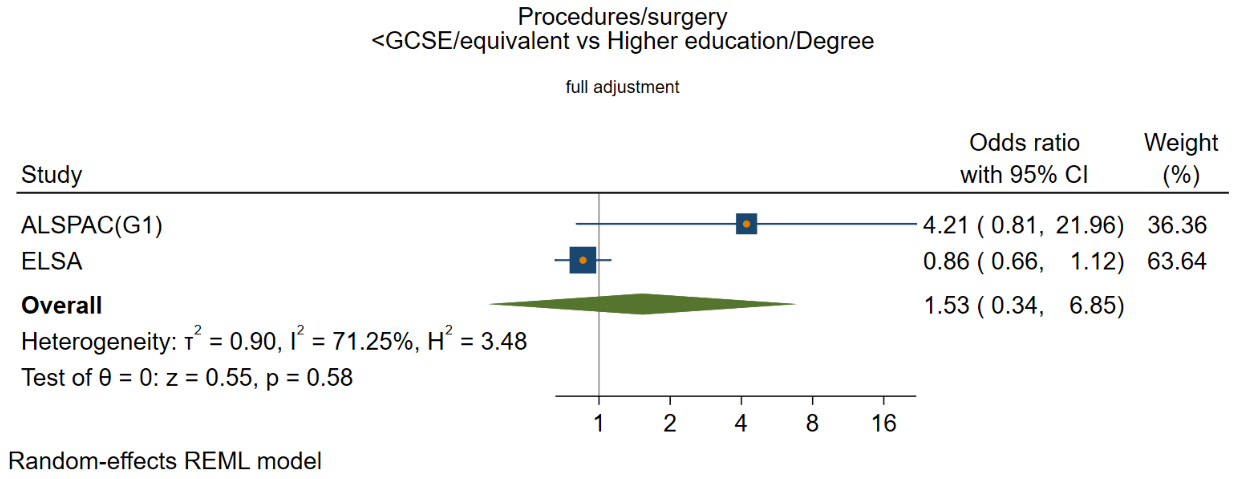

For peer review only - http://bmjopen.bmj.com/site/about/guidelines.xhtml

Age

Unadjusted

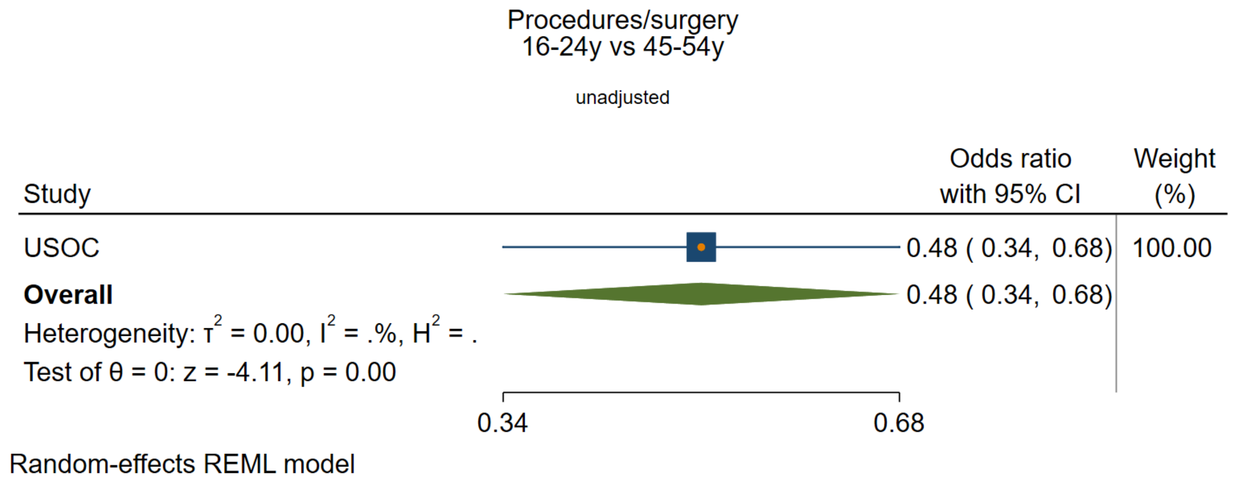

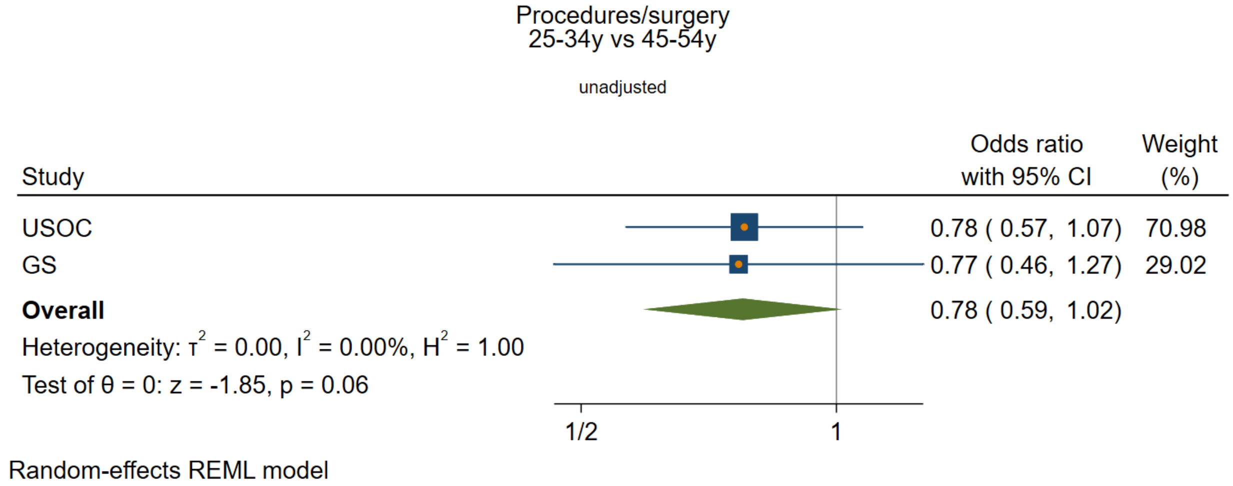

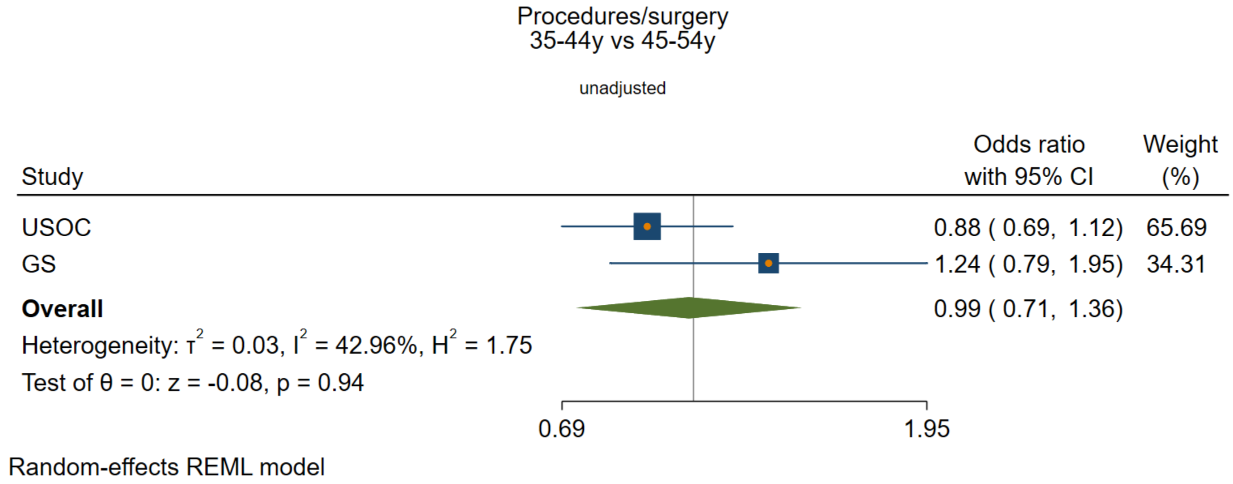

For peer review only - http://bmjopen.bmj.com/site/about/guidelines.xhtml

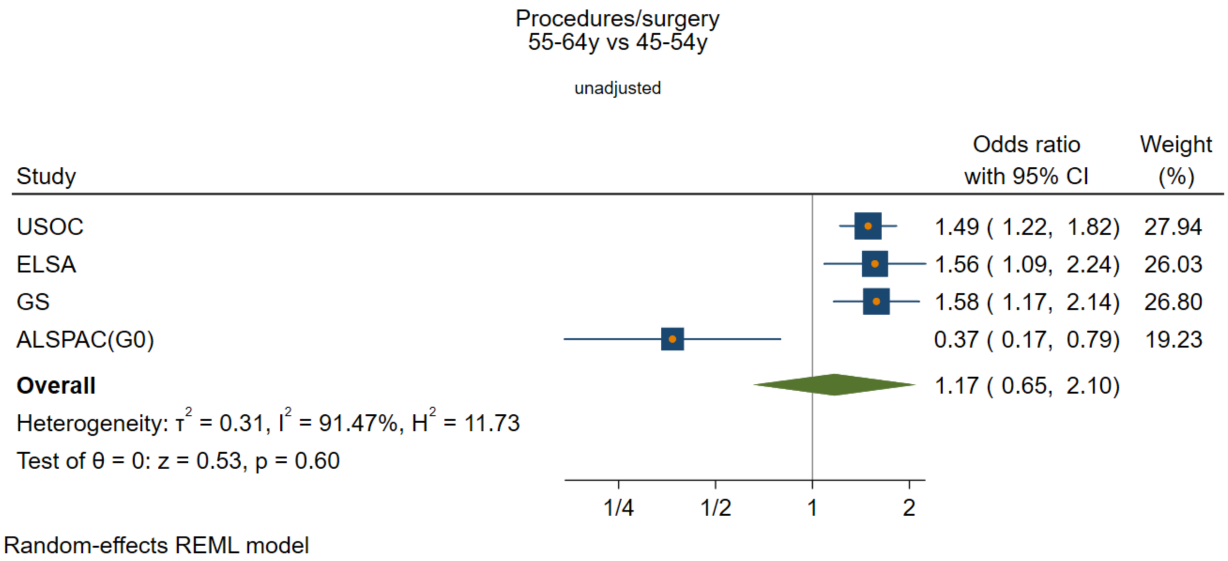

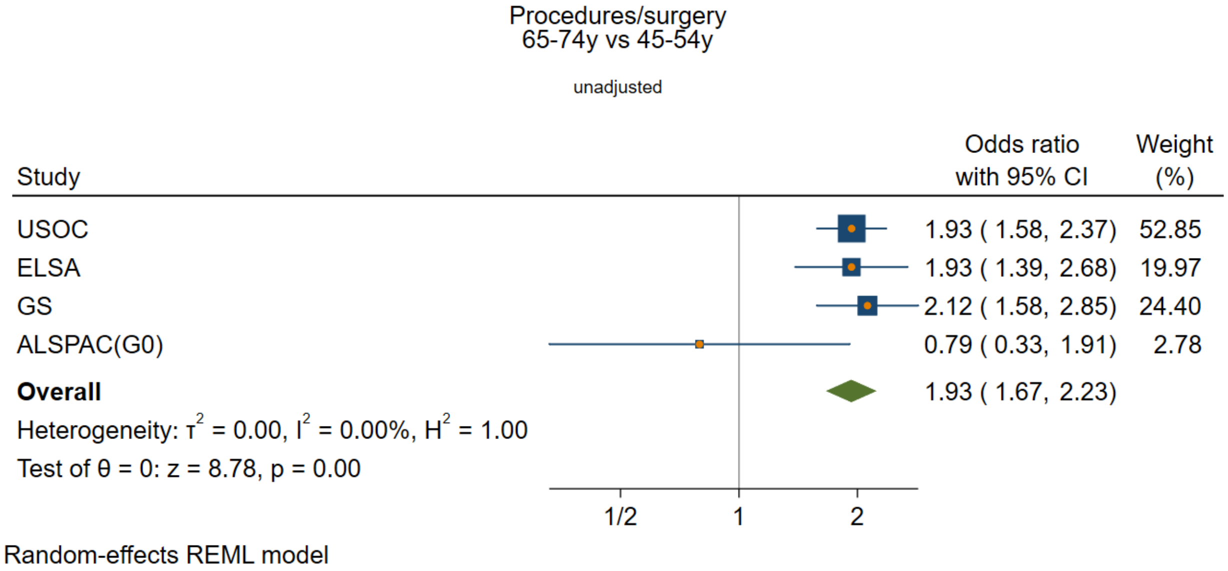

For peer review only - http://bmjopen.bmj.com/site/about/guidelines.xhtml

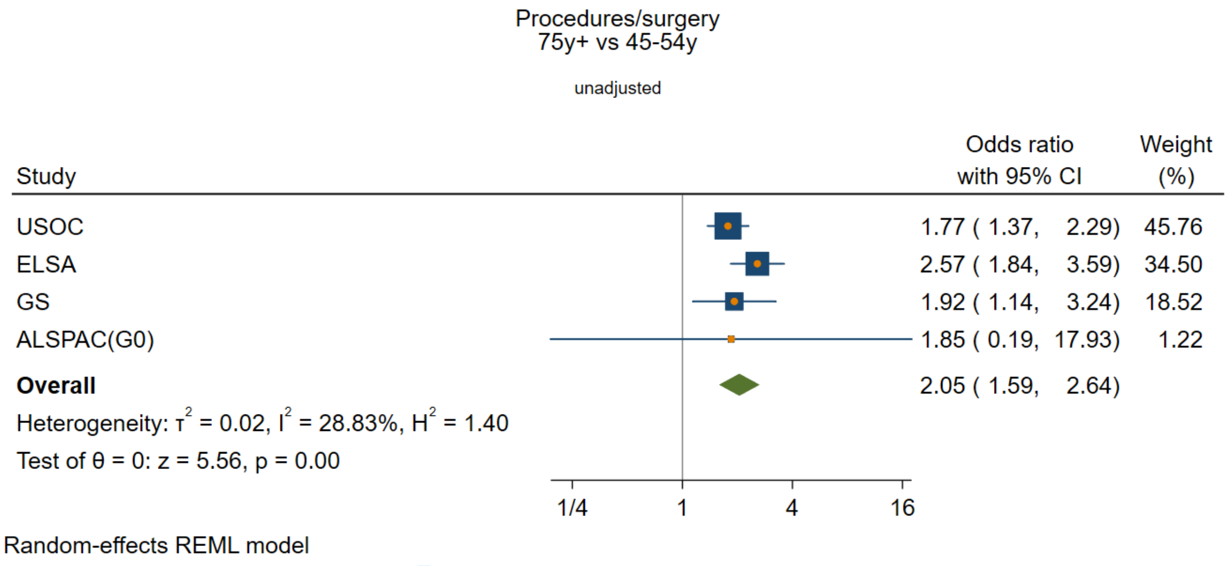

peer review only

Basic adjustment

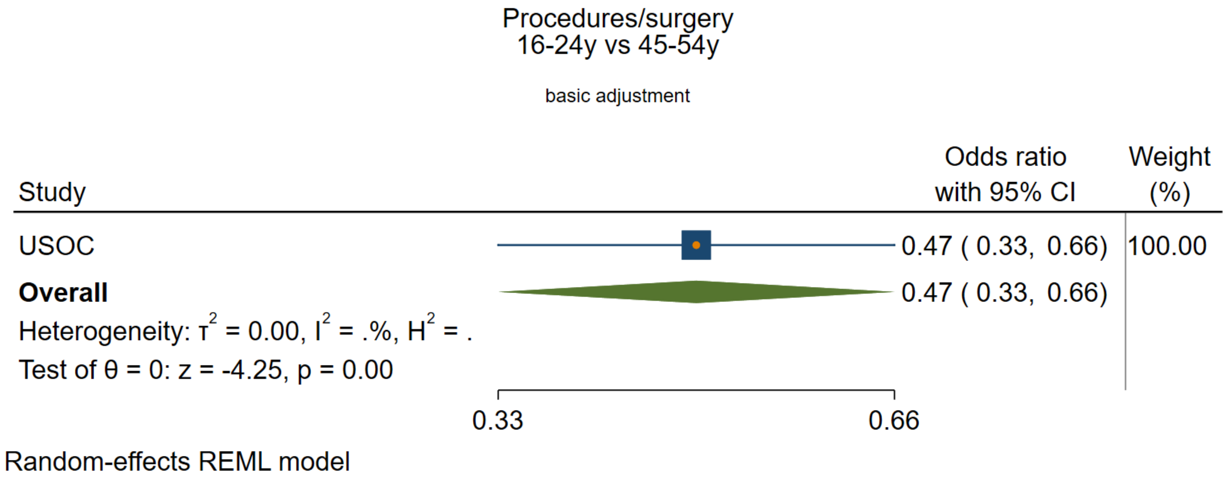

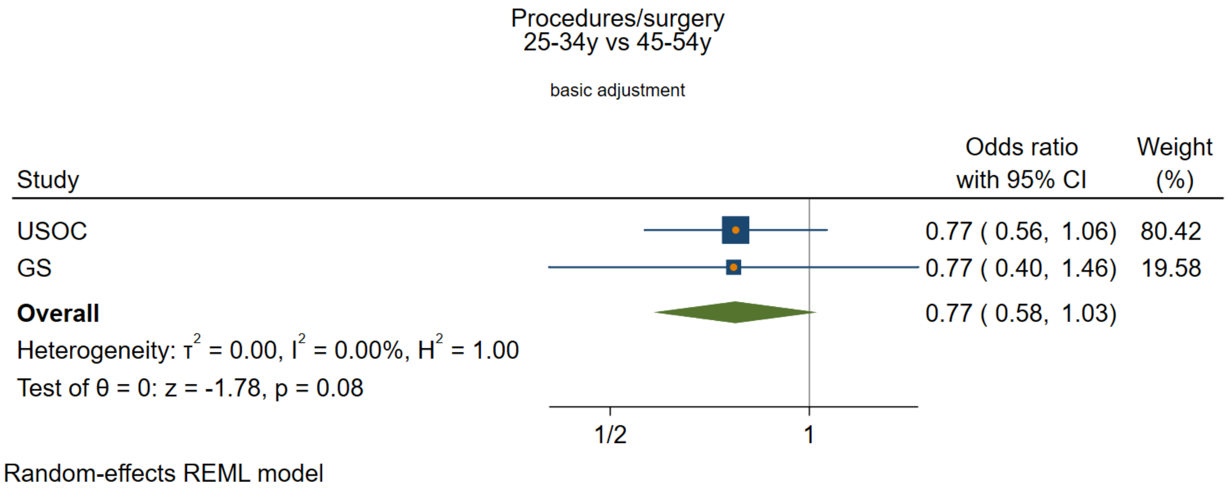

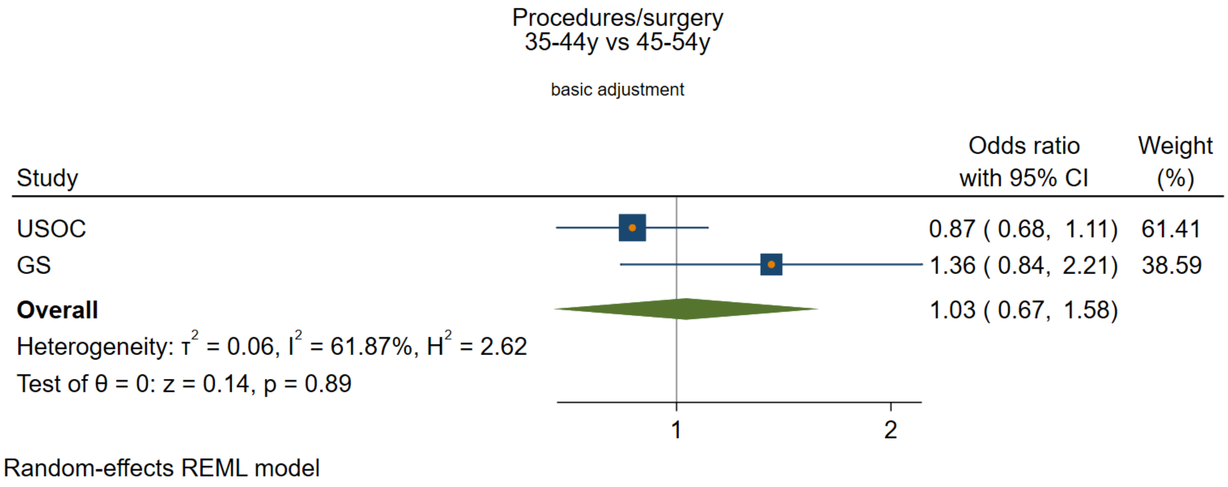

For peer review only - http://bmjopen.bmj.com/site/about/guidelines.xhtml

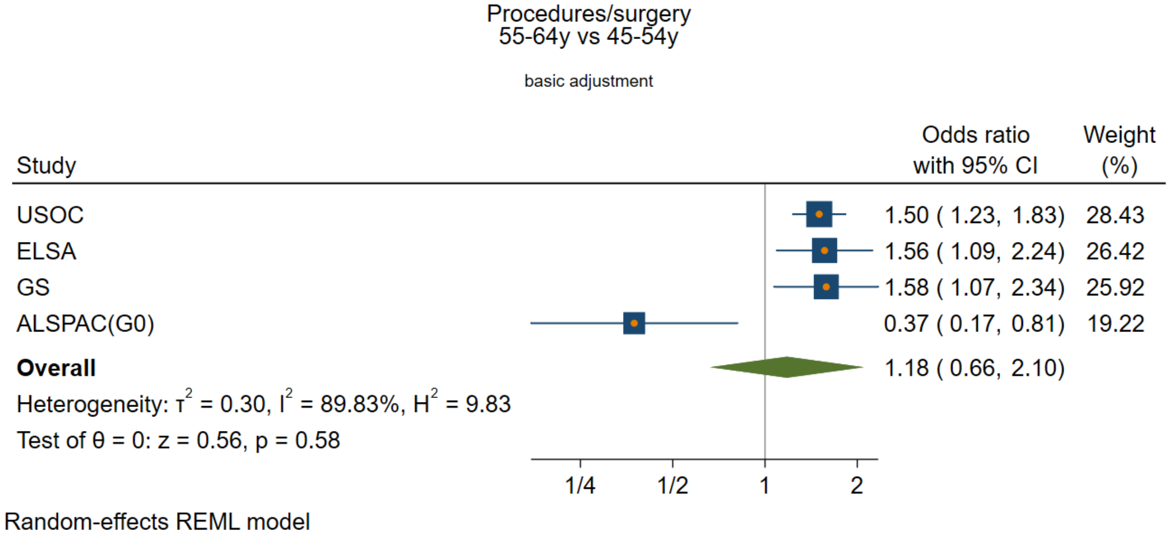

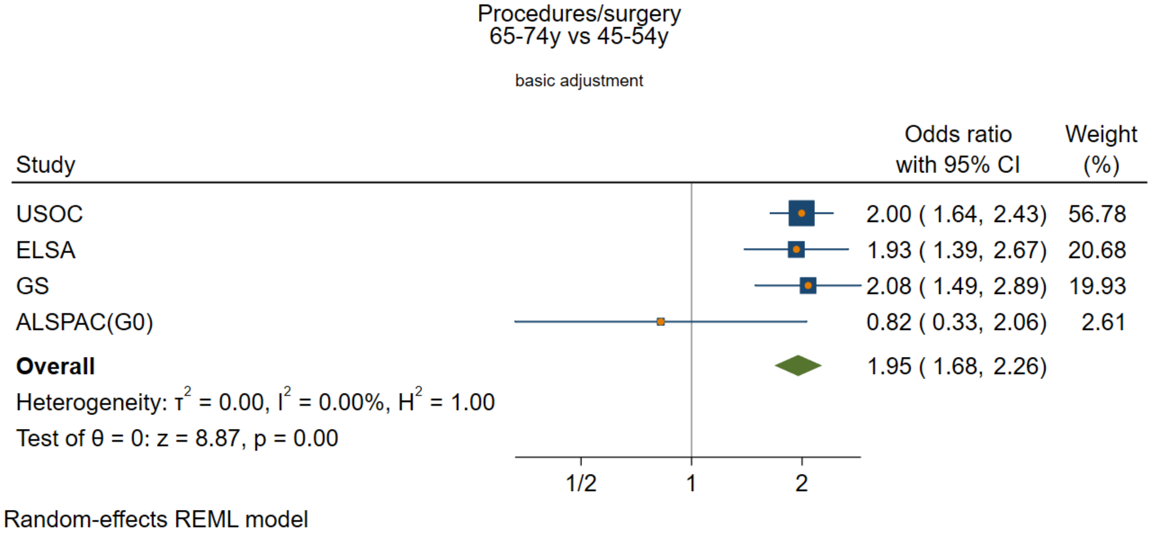

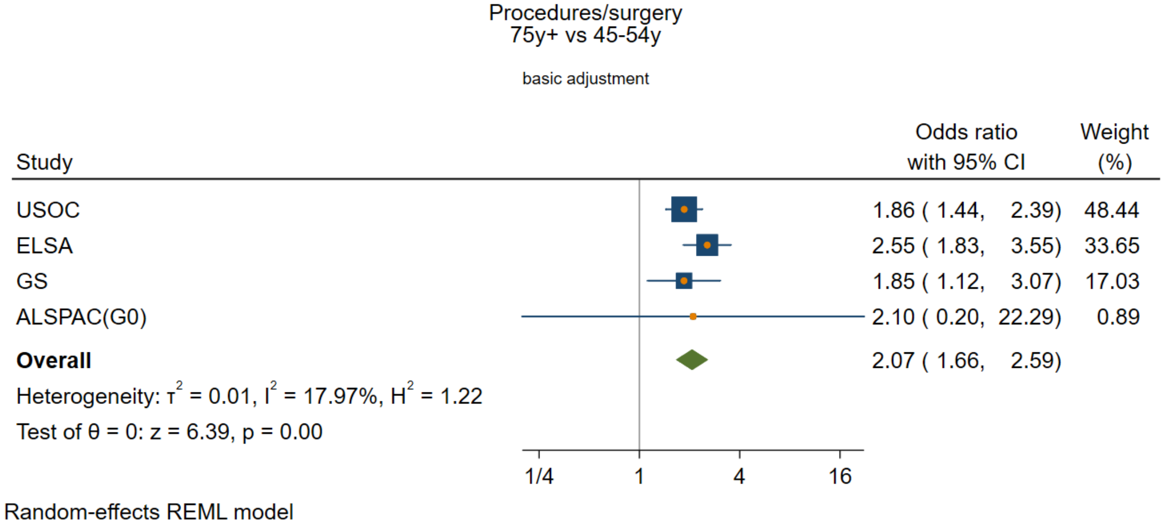

For peer review only - http://bmjopen.bmj.com/site/about/guidelines.xhtml

Full adjustment

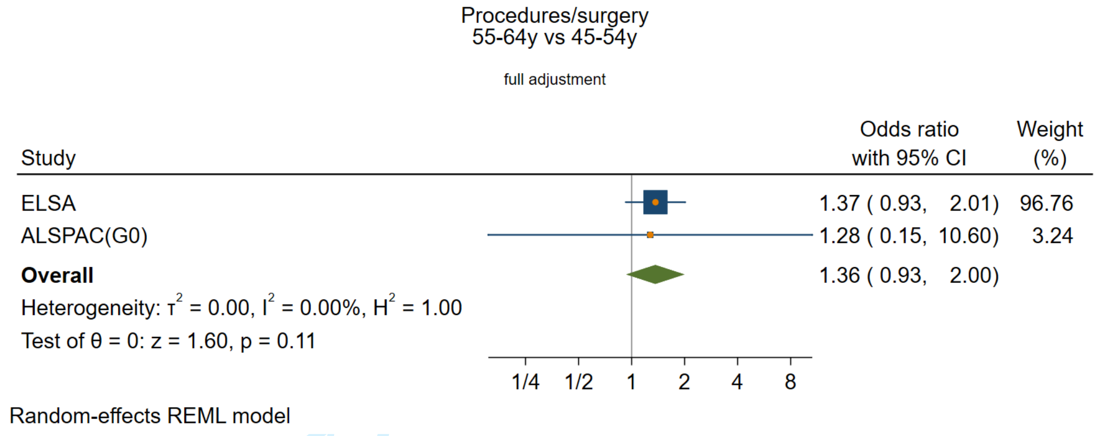

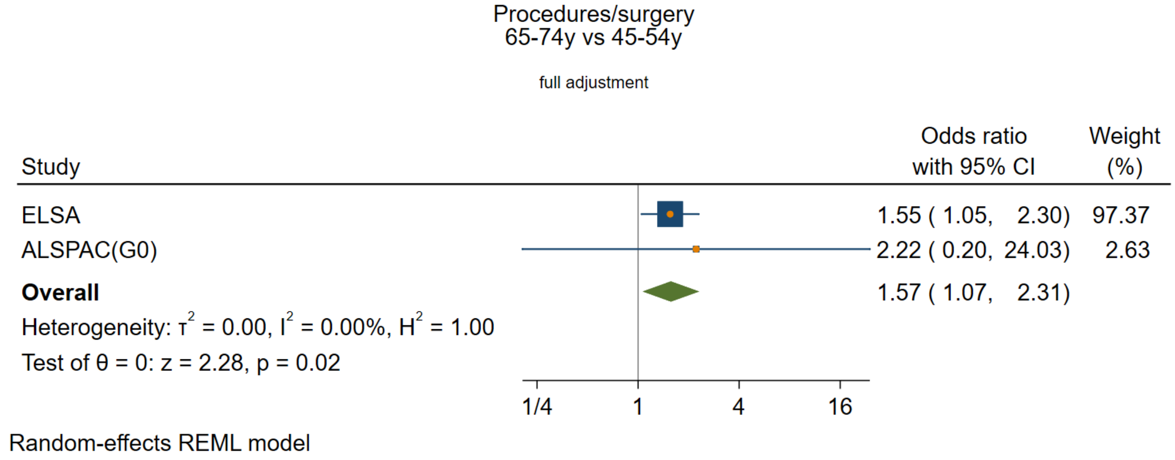

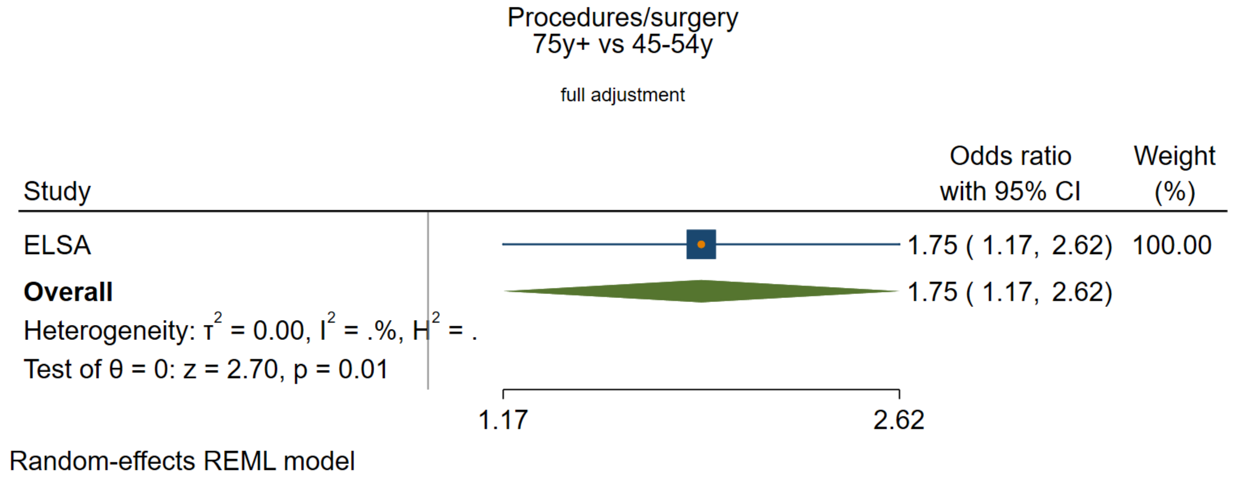

For peer review only - http://bmjopen.bmj.com/site/about/guidelines.xhtml

Ethnicity
Unadjusted

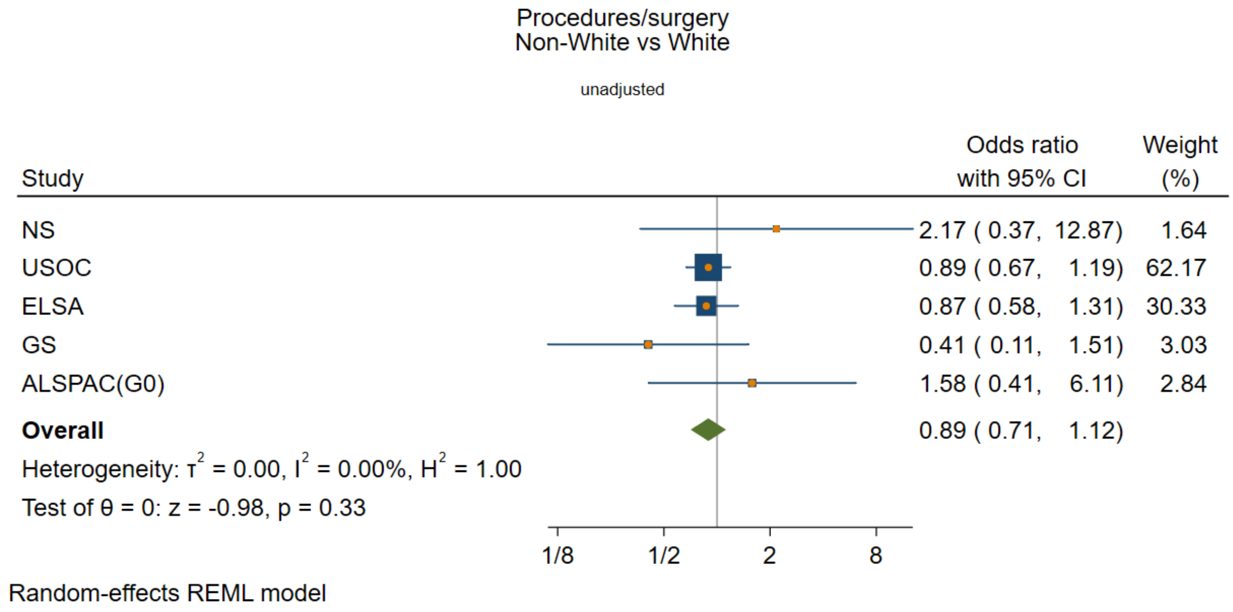

view only

For peer review only - http://bmjopen.bmj.com/site/about/guidelines.xhtml

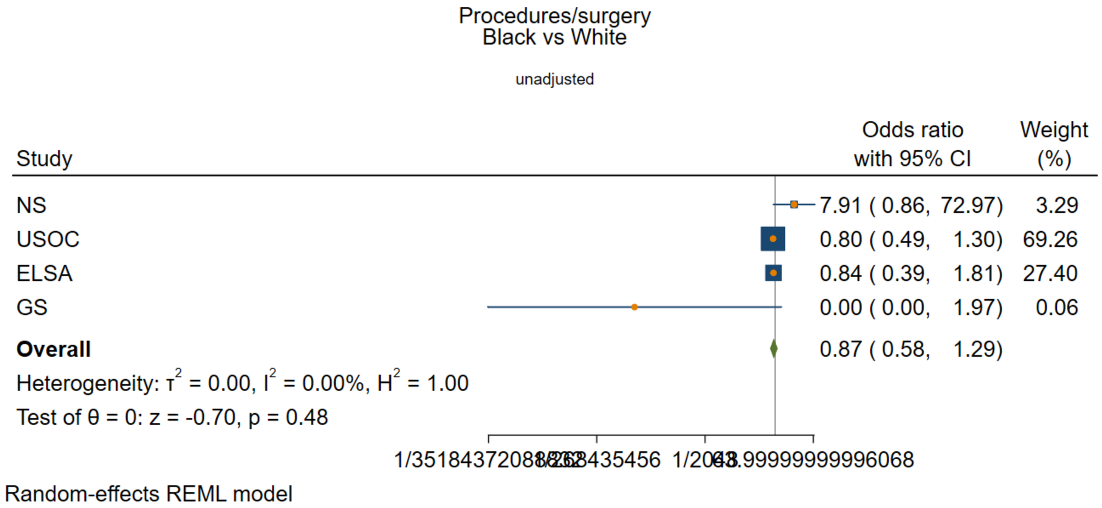

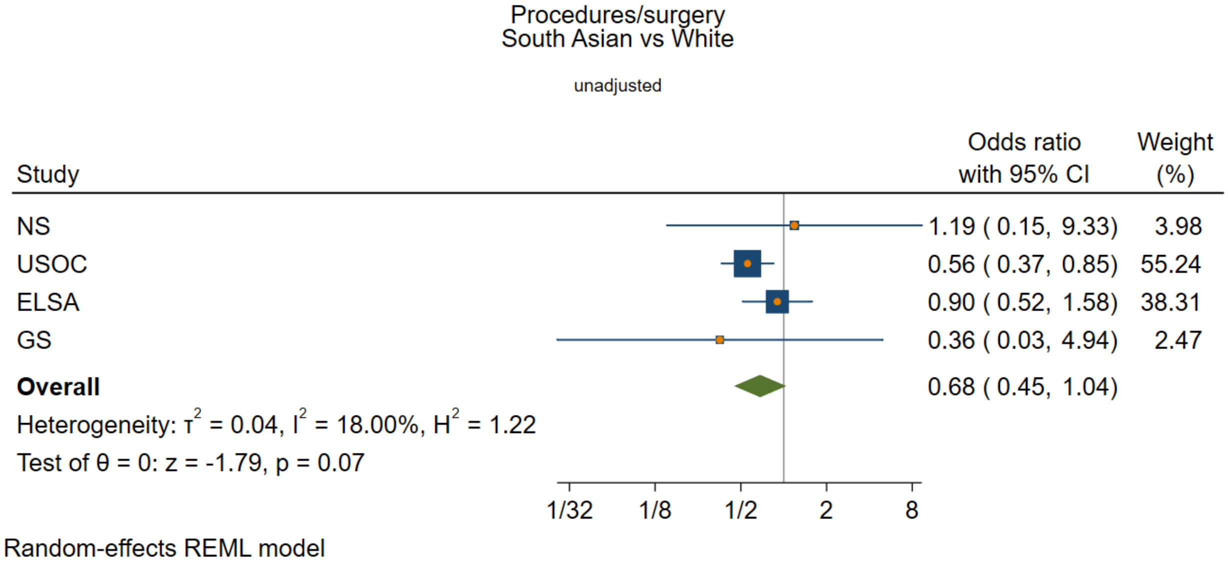

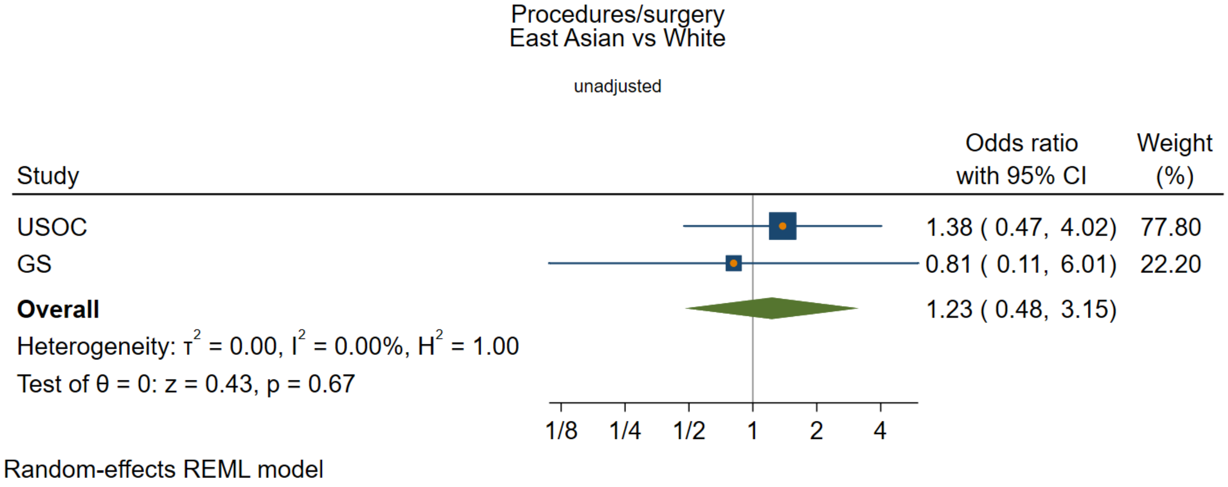

For peer review only - http://bmjopen.bmj.com/site/about/guidelines.xhtml

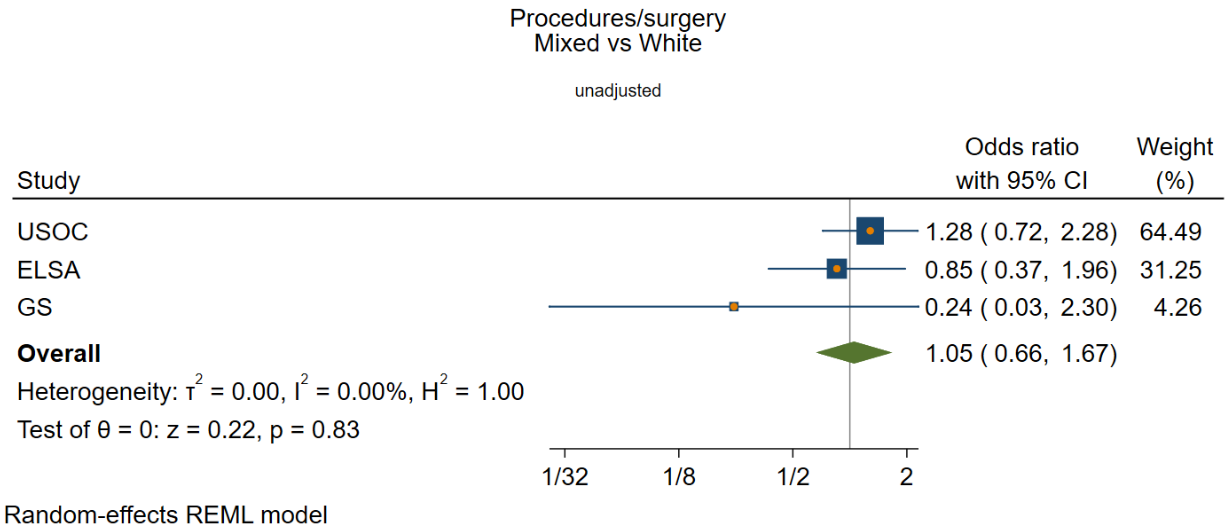

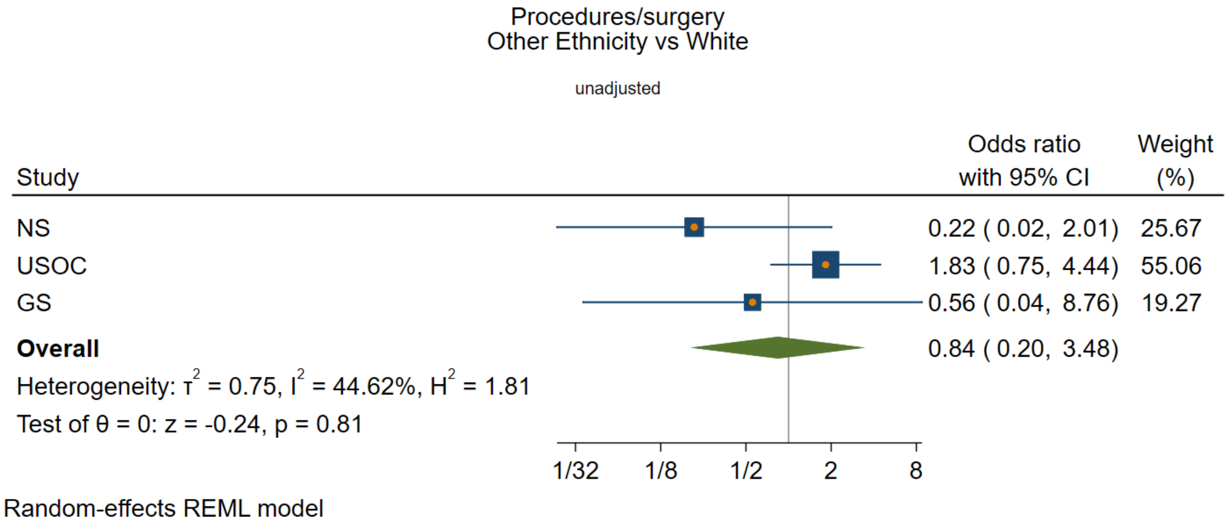

For peer review only - http://bmjopen.bmj.com/site/about/guidelines.xhtml

Basic adjustment

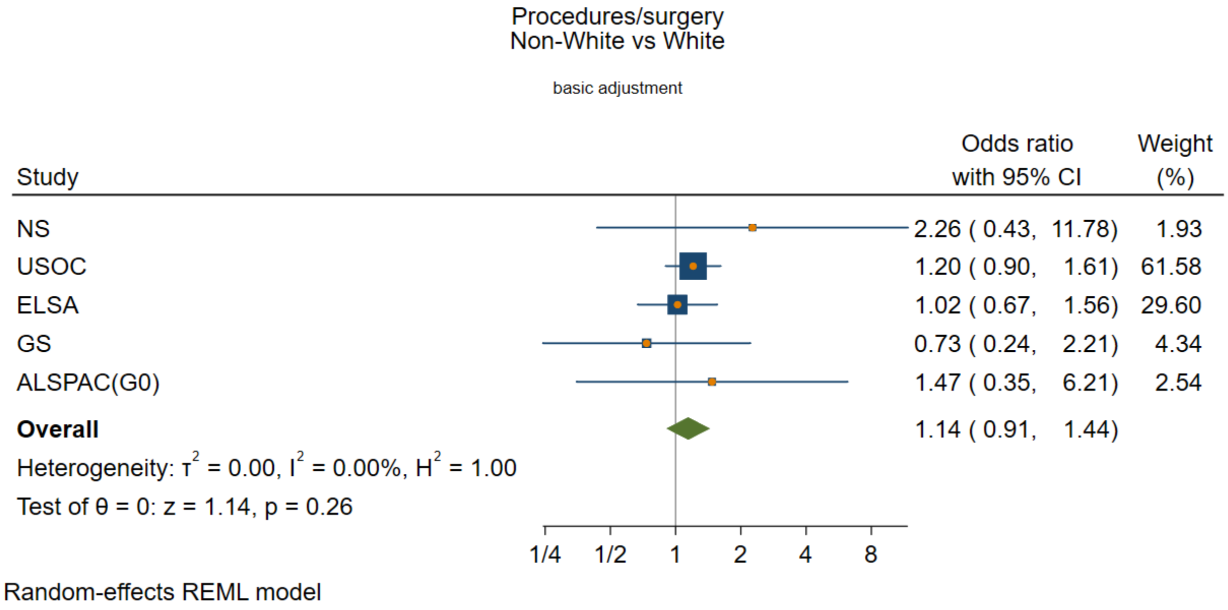

For peer review only

For peer review only - http://bmjopen.bmj.com/site/about/guidelines.xhtml

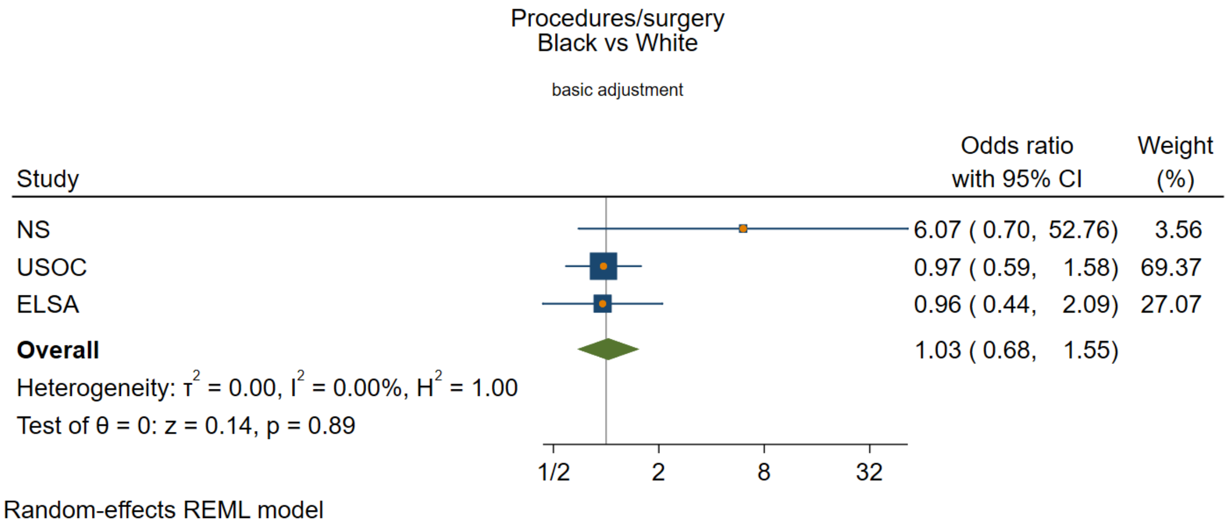

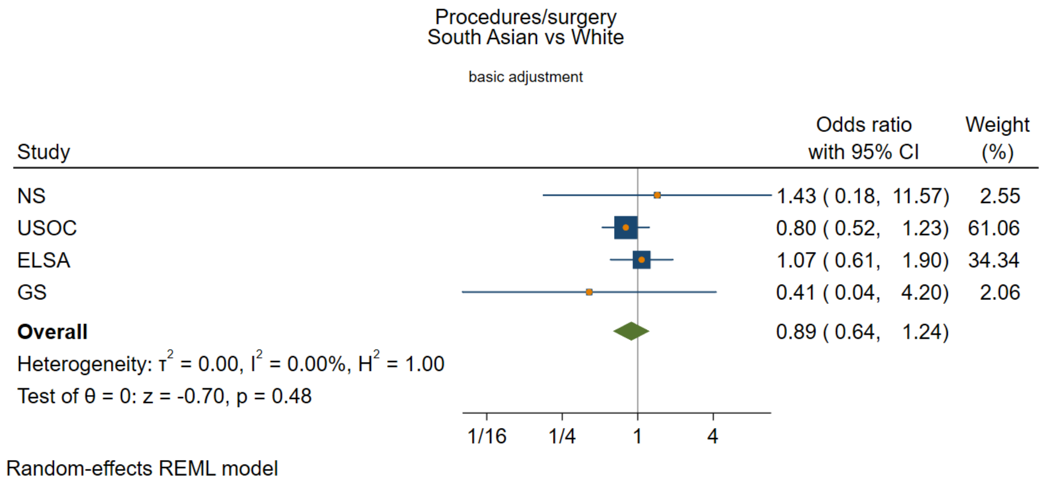

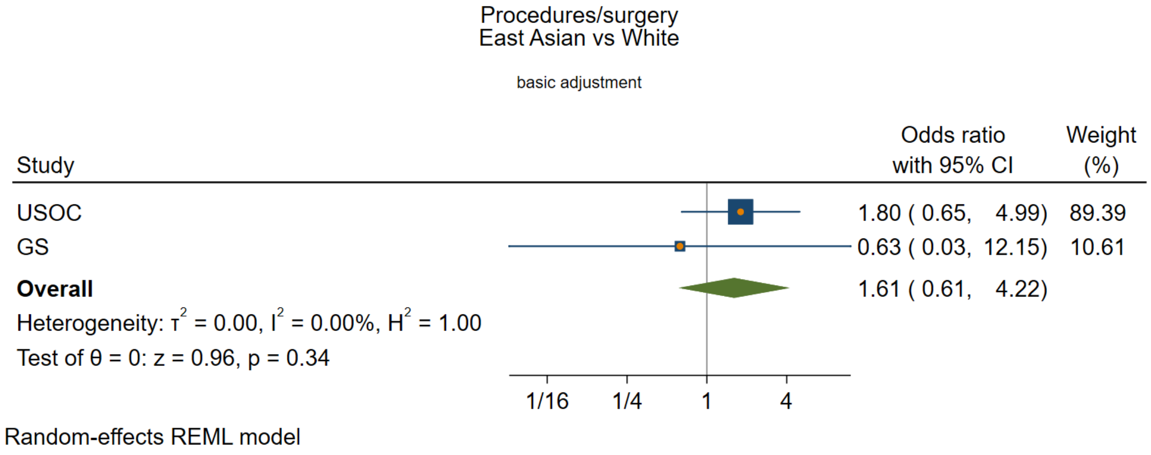

For peer review only - http://bmjopen.bmj.com/site/about/guidelines.xhtml

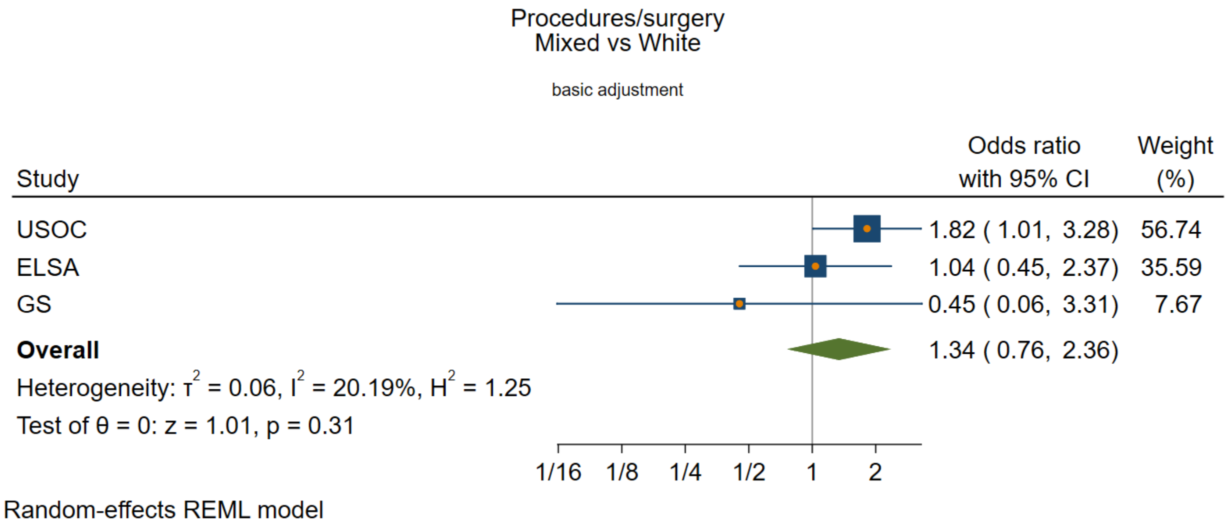

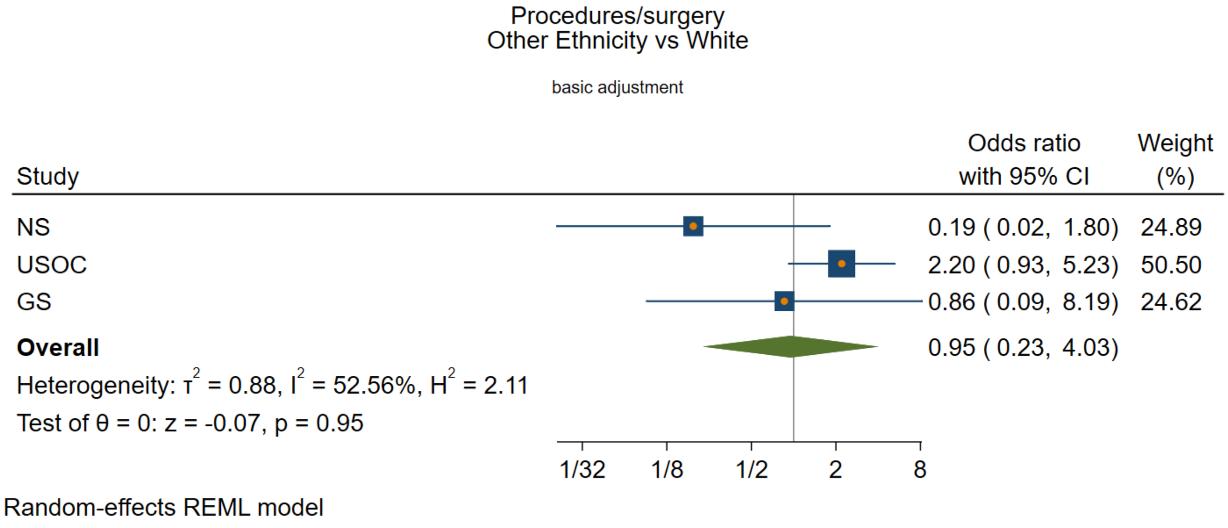

For peer review only - http://bmjopen.bmj.com/site/about/guidelines.xhtml

Full adjustment

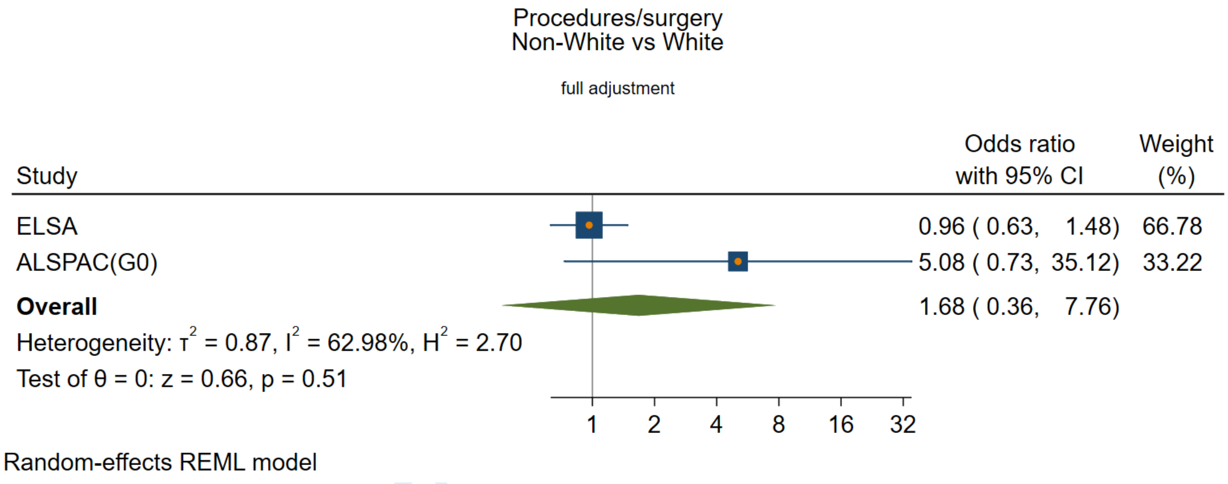

For peer review only - http://bmjopen.bmj.com/site/about/guidelines.xhtml

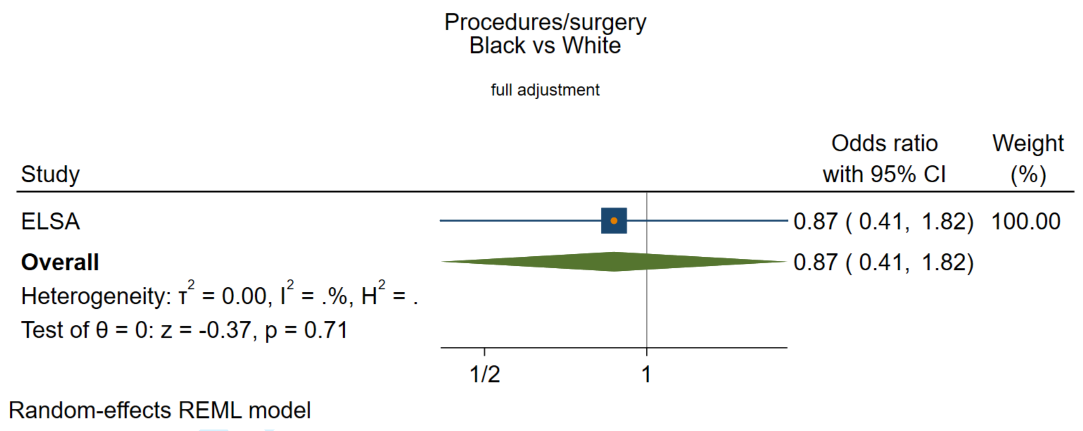

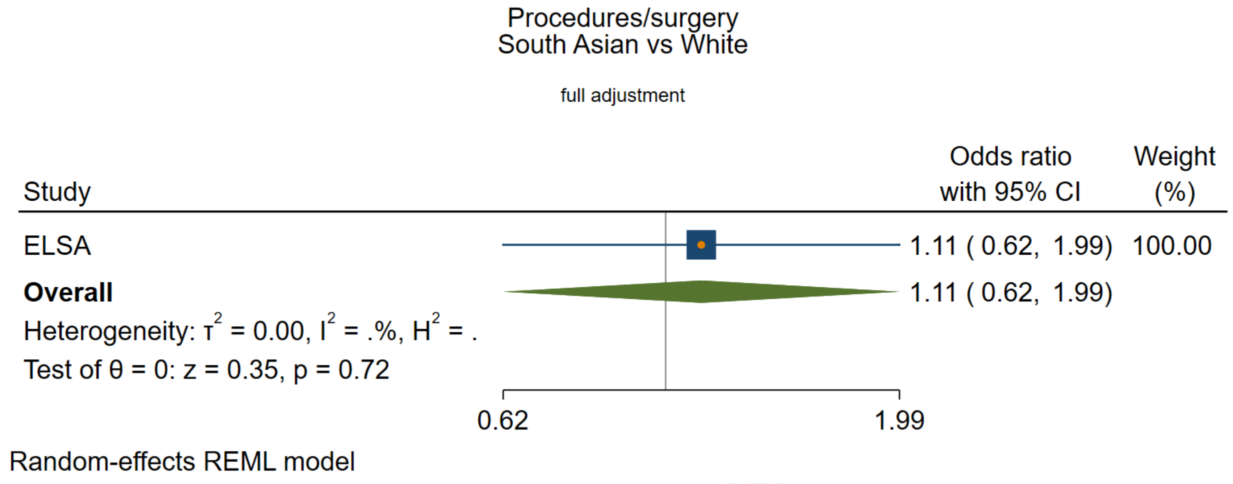

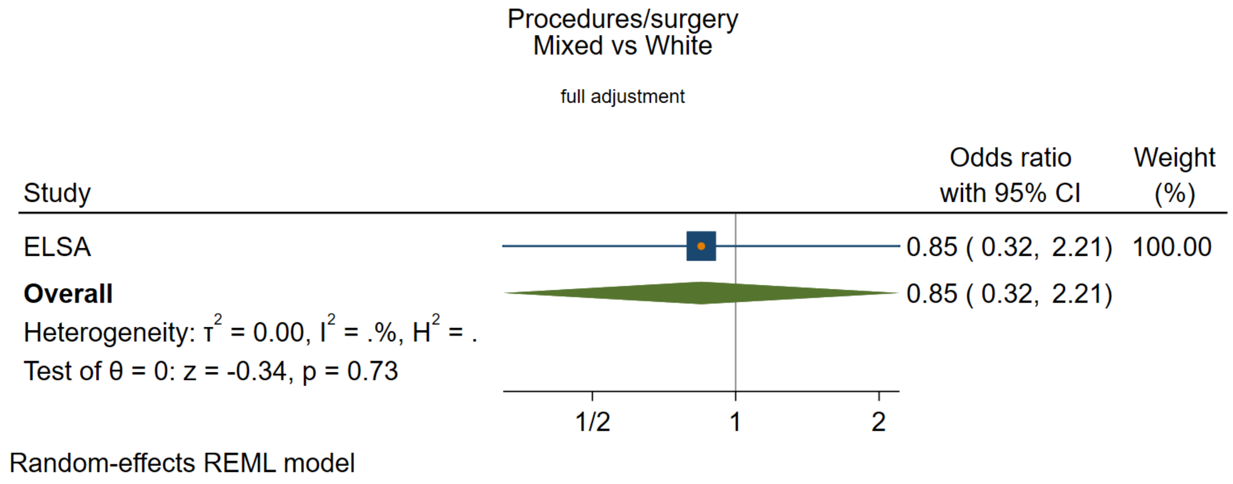

For peer review only - http://bmjopen.bmj.com/site/about/guidelines.xhtml

Any healthcare disruption stratified by shielding status

Sex

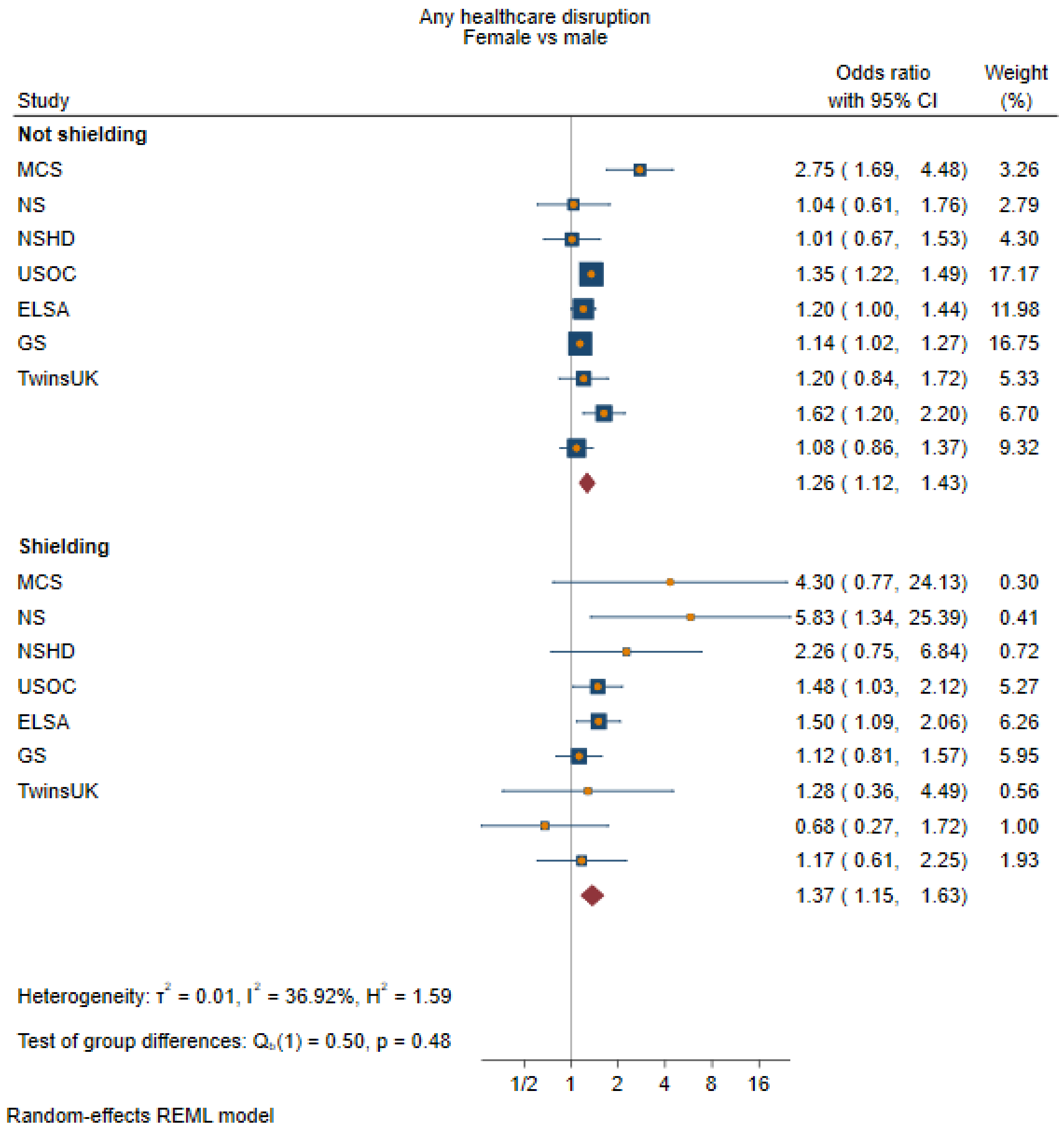

Occupational class

For peer review only

For peer review only - http://bmjopen.bmj.com/site/about/guidelines.xhtml

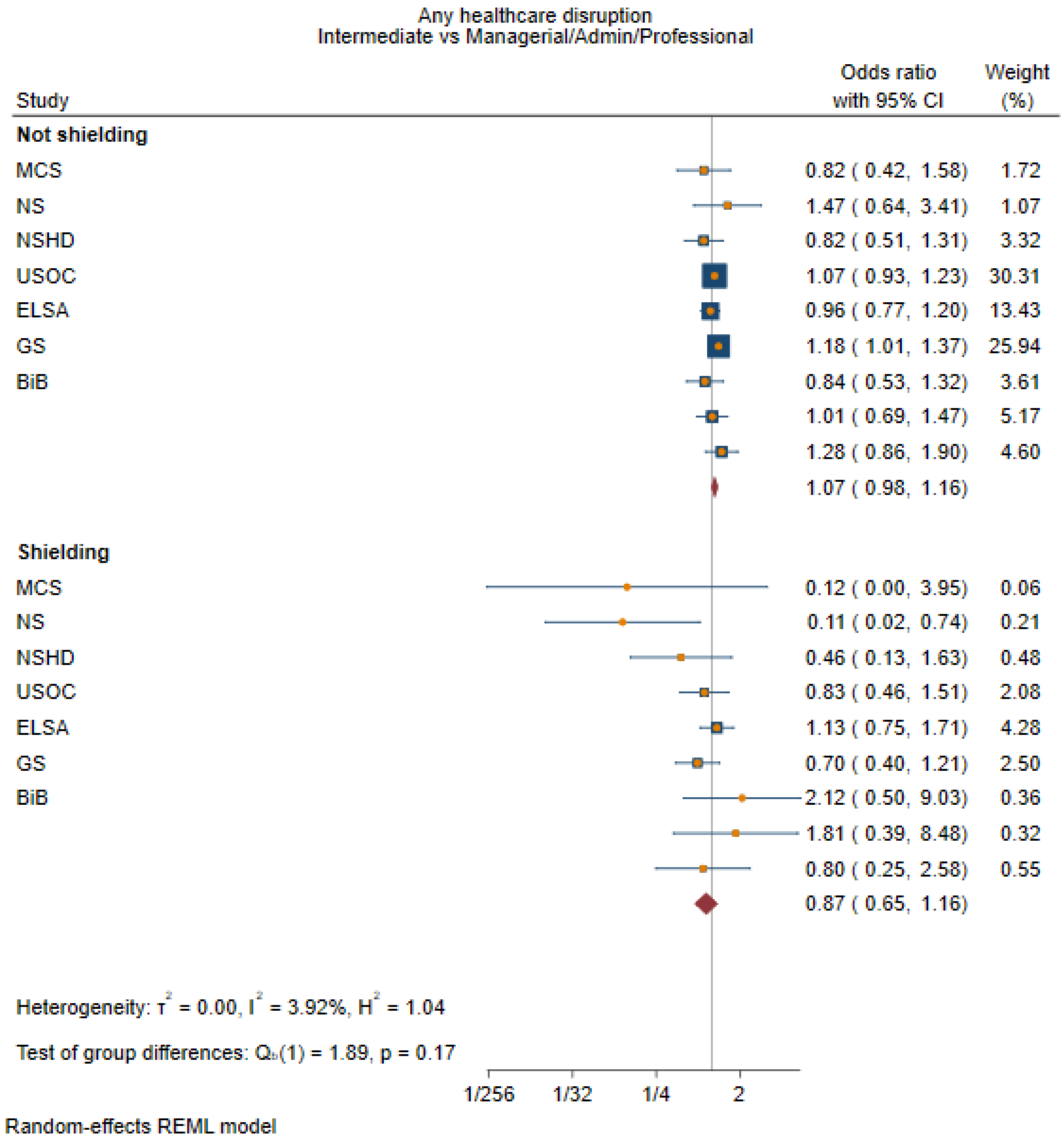

For peer review only - http://bmjopen.bmj.com/site/about/guidelines.xhtml

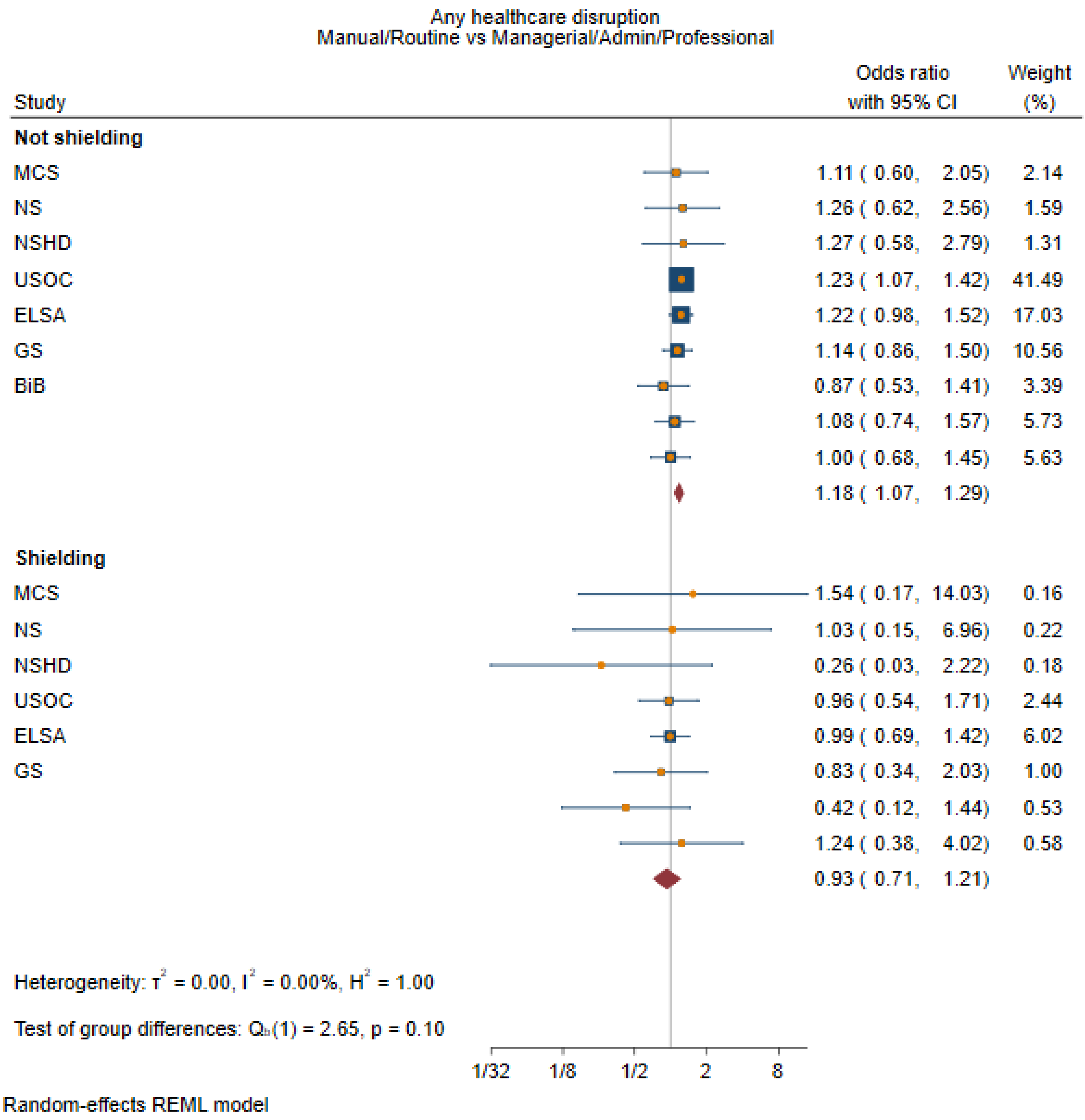
Any healthcare disruption
Manual/Routine vs Managerial/Admin/Professional
Study
Odds ratio with 95% CI
Weight (%)
Not shielding
MCS 1.11 (0.60, 2.05) 2.14
NS 1.26 (0.62, 2.56) 1.59
NSHD 1.27 (0.58, 2.79) 1.31
USOC 1.23 (1.07, 1.42) 41.49
ELSA 1.22 (0.98, 1.52) 17.03
GS 1.14 (0.86, 1.50) 10.56
BiB 0.87 (0.53, 1.41) 3.39
1.08 (0.74, 1.57) 5.73
1.00 (0.68, 1.45) 5.63
1.18 (1.07, 1.29)
Shielding
MCS 1.54 (0.17, 14.03) 0.16
NS 1.03 (0.15, 6.96) 0.22
NSHD 0.26 (0.03, 2.22) 0.18
USOC 0.96 (0.54, 1.71) 2.44
ELSA 0.99 (0.69, 1.42) 6.02
GS 0.83 (0.34, 2.03) 1.00
0.42 (0.12, 1.44) 0.53
1.24 (0.38, 4.02) 0.58
0.93 (0.71, 1.21)
Heterogeneity: τ² = 0.00, I² = 0.00%, H² = 1.00
Test of group differences: Qb(1) = 2.65, p = 0.10
1/32
1/8
1/2
2
8
Random-effects REML model

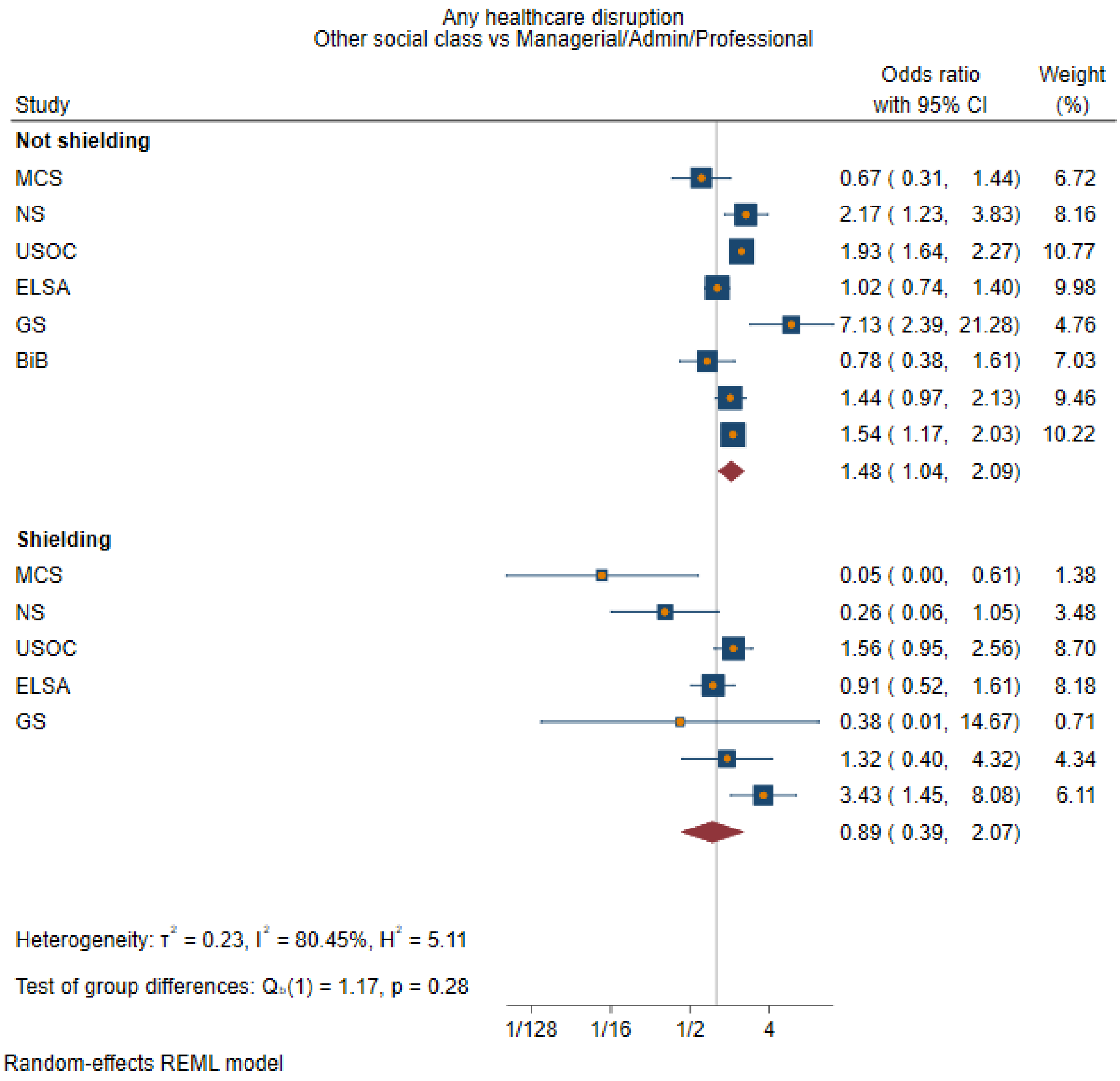

For peer review only - http://bmjopen.bmj.com/site/about/guidelines.xhtml

Education

For peer review only - http://bmjopen.bmj.com/site/about/guidelines.xhtml

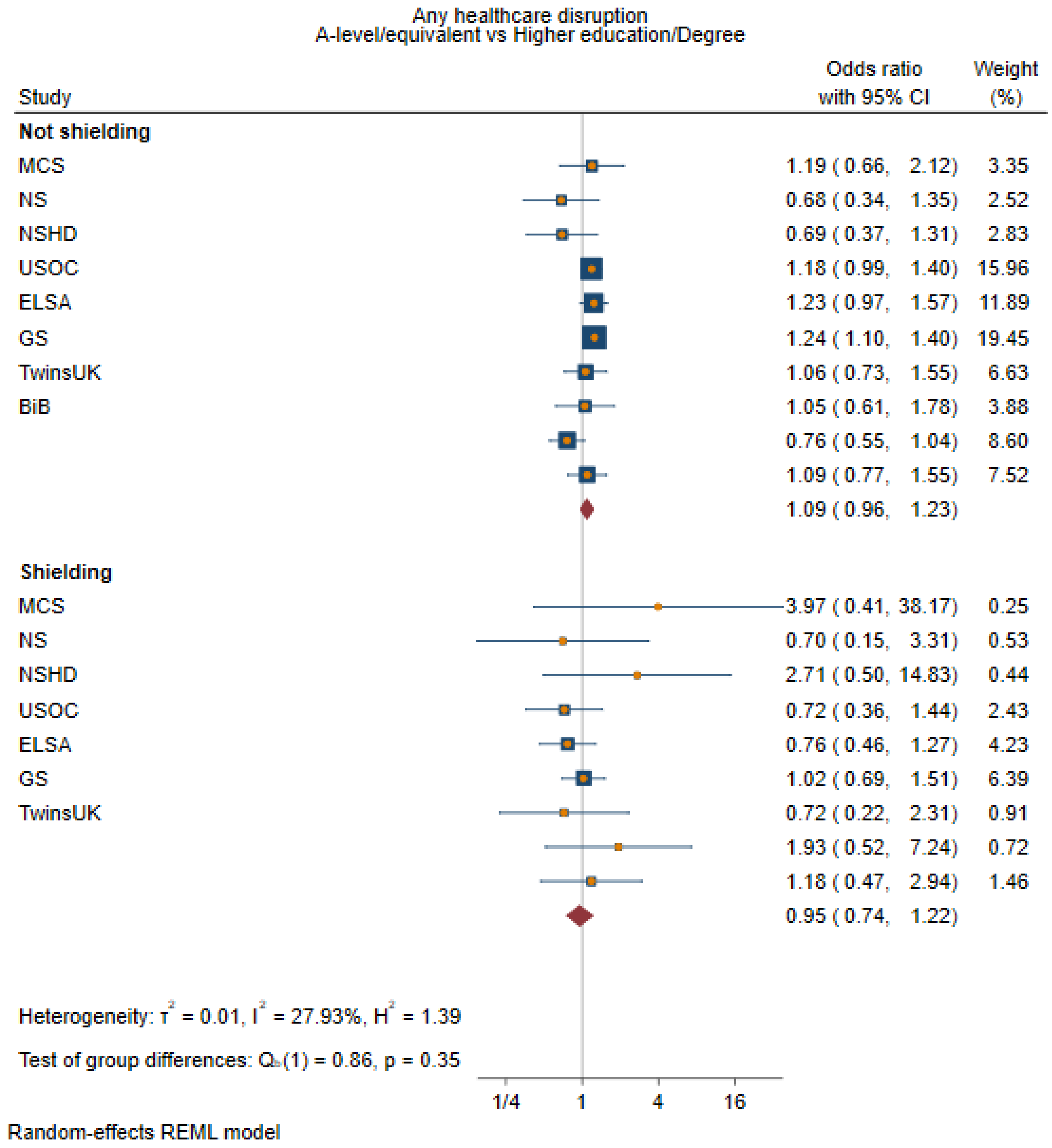
Any healthcare disruption
A-level/equivalent vs Higher education/Degree
Study
Odds ratio with 95% CI
Weight (%)
Not shielding
MCS
1.19 (0.66, 2.12)
3.35
NS
0.68 (0.34, 1.35)
2.52
NSHD
0.69 (0.37, 1.31)
2.83
USOC
1.18 (0.99, 1.40)
15.96
ELSA
1.23 (0.97, 1.57)
11.89
GS
1.24 (1.10, 1.40)
19.45
TwinsUK
1.06 (0.73, 1.55)
6.63
BiB
1.05 (0.61, 1.78)
3.88
0.76 (0.55, 1.04)
8.60
1.09 (0.77, 1.55)
7.52
1.09 (0.96, 1.23)
Shielding
MCS
3.97 (0.41, 38.17)
0.25
NS
0.70 (0.15, 3.31)
0.53
NSHD
2.71 (0.50, 14.83)
0.44
USOC
0.72 (0.36, 1.44)
2.43
ELSA
0.76 (0.46, 1.27)
4.23
GS
1.02 (0.69, 1.51)
6.39
TwinsUK
0.72 (0.22, 2.31)
0.91
1.93 (0.52, 7.24)
0.72
1.18 (0.47, 2.94)
1.46
0.95 (0.74, 1.22)
Heterogeneity: τ² = 0.01, I² = 27.93%, H² = 1.39
Test of group differences: Qb(1) = 0.86, p = 0.35
1/4
1
4
16
Random-effects REML model

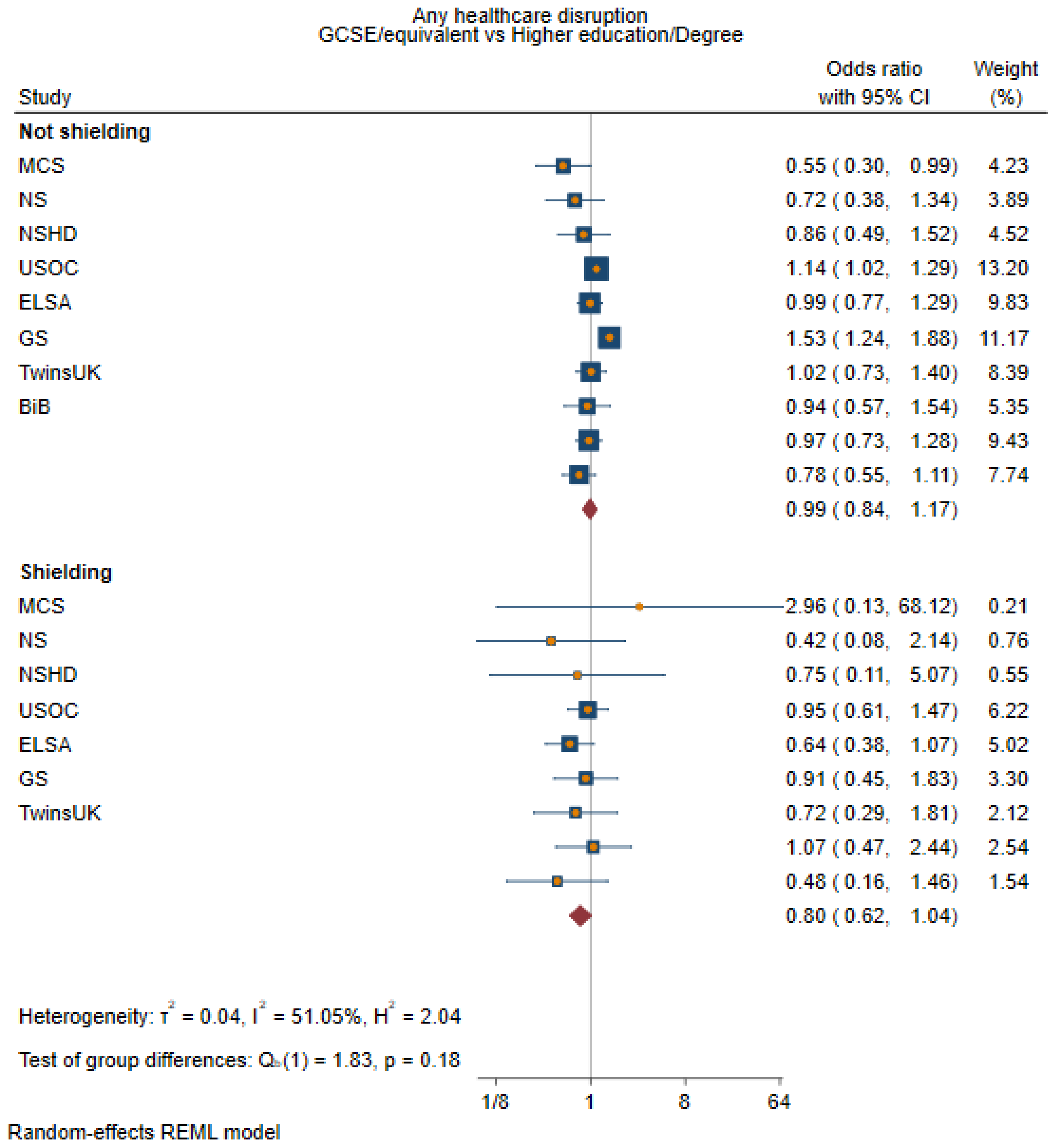
Any healthcare disruption
GCSE/equivalent vs Higher education/Degree
Study
Odds ratio with 95% CI
Weight (%)
Not shielding
MCS 0.55 (0.30, 0.99) 4.23
NS 0.72 (0.38, 1.34) 3.89
NSHD 0.86 (0.49, 1.52) 4.52
USOC 1.14 (1.02, 1.29) 13.20
ELSA 0.99 (0.77, 1.29) 9.83
GS 1.53 (1.24, 1.88) 11.17
TwinsUK 1.02 (0.73, 1.40) 8.39
BiB 0.94 (0.57, 1.54) 5.35
0.97 (0.73, 1.28) 9.43
0.78 (0.55, 1.11) 7.74
0.99 (0.84, 1.17)
Shielding
MCS 2.96 (0.13, 68.12) 0.21
NS 0.42 (0.08, 2.14) 0.76
NSHD 0.75 (0.11, 5.07) 0.55
USOC 0.95 (0.61, 1.47) 6.22
ELSA 0.64 (0.38, 1.07) 5.02
GS 0.91 (0.45, 1.83) 3.30
TwinsUK 0.72 (0.29, 1.81) 2.12
1.07 (0.47, 2.44) 2.54
0.48 (0.16, 1.46) 1.54
0.80 (0.62, 1.04)
Heterogeneity: τ² = 0.04, I² = 51.05%, H² = 2.04
Test of group differences: Q_b(1) = 1.83, p = 0.18
1/8
1
8
64
Random-effects REML model

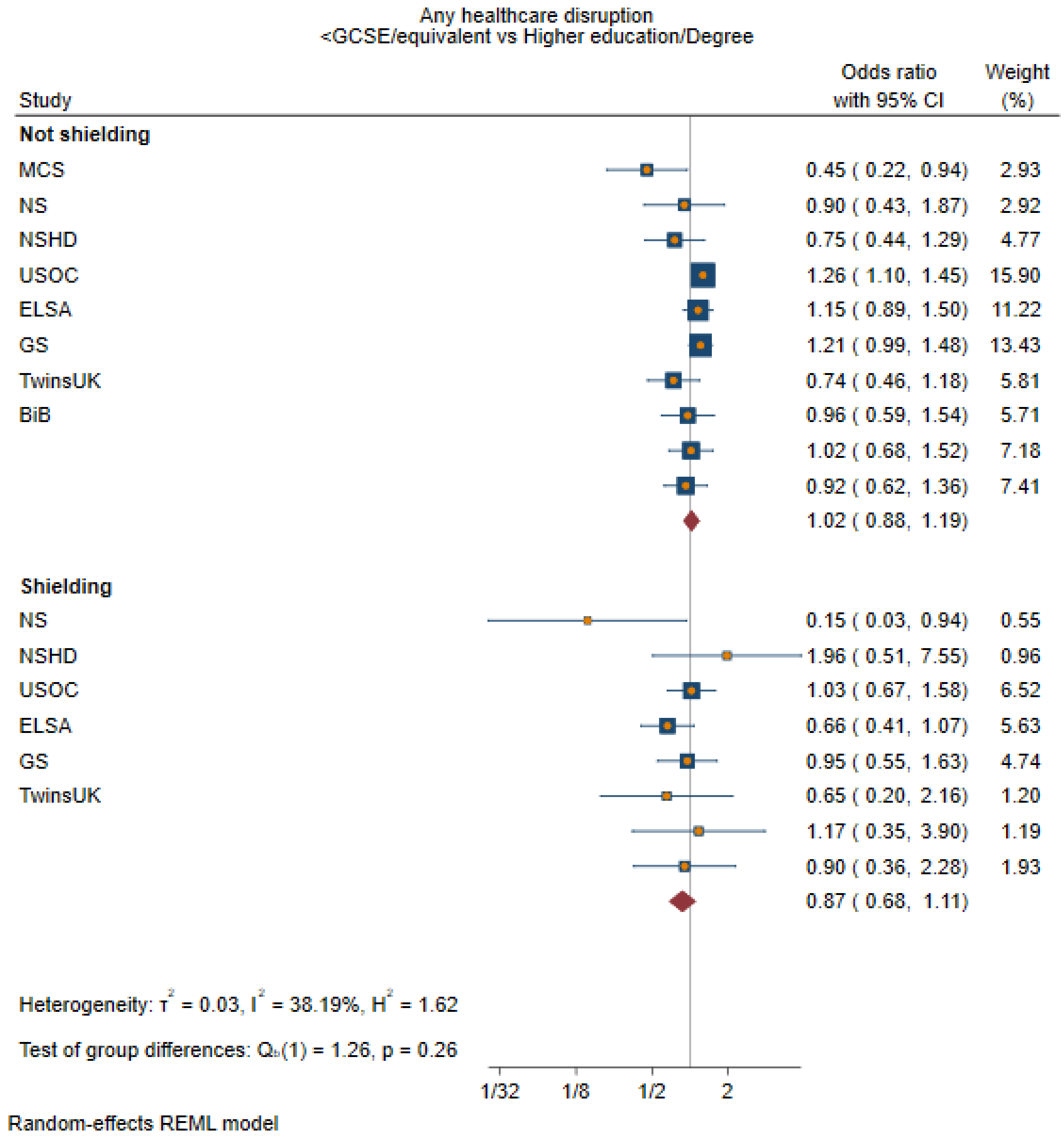

For peer review only - http://bmjopen.bmj.com/site/about/guidelines.xhtml

Age

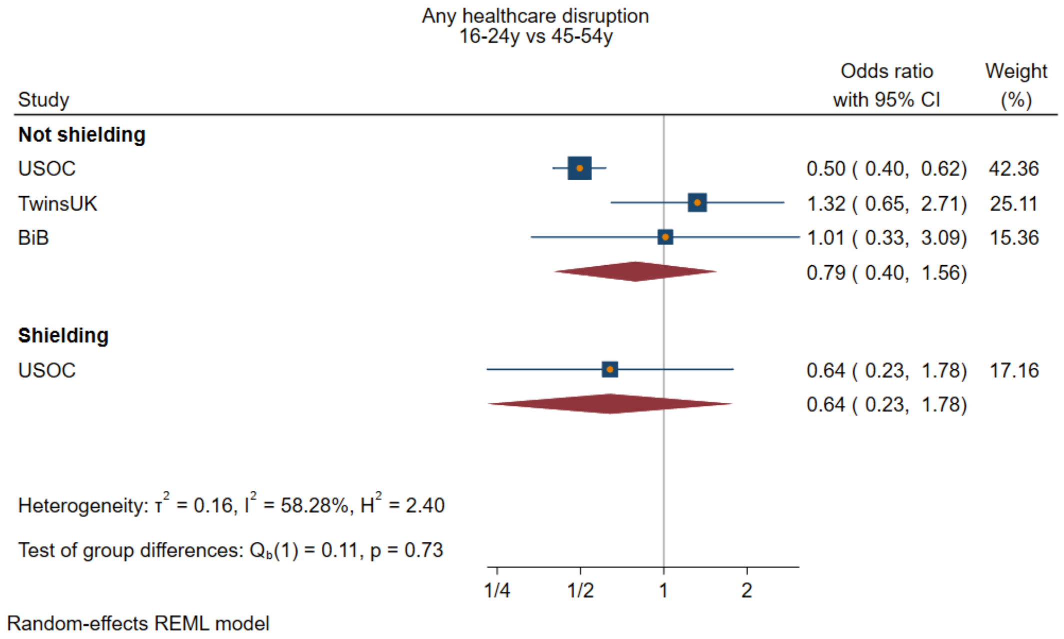
Any healthcare disruption
16-24y vs 45-54y
Study
Odds ratio with 95% CI
Weight (%)
Not shielding
USOC 0.50 (0.40, 0.62) 42.36
TwinsUK 1.32 (0.65, 2.71) 25.11
BiB 1.01 (0.33, 3.09) 15.36
0.79 (0.40, 1.56)
Shielding
USOC 0.64 (0.23, 1.78) 17.16
0.64 (0.23, 1.78)
Heterogeneity: τ^2 = 0.16, I^2 = 58.28%, H^2 = 2.40
Test of group differences: $Q_b(1)$ = 0.11, p = 0.73
1/4
1/2
1
2
Random-effects REML model

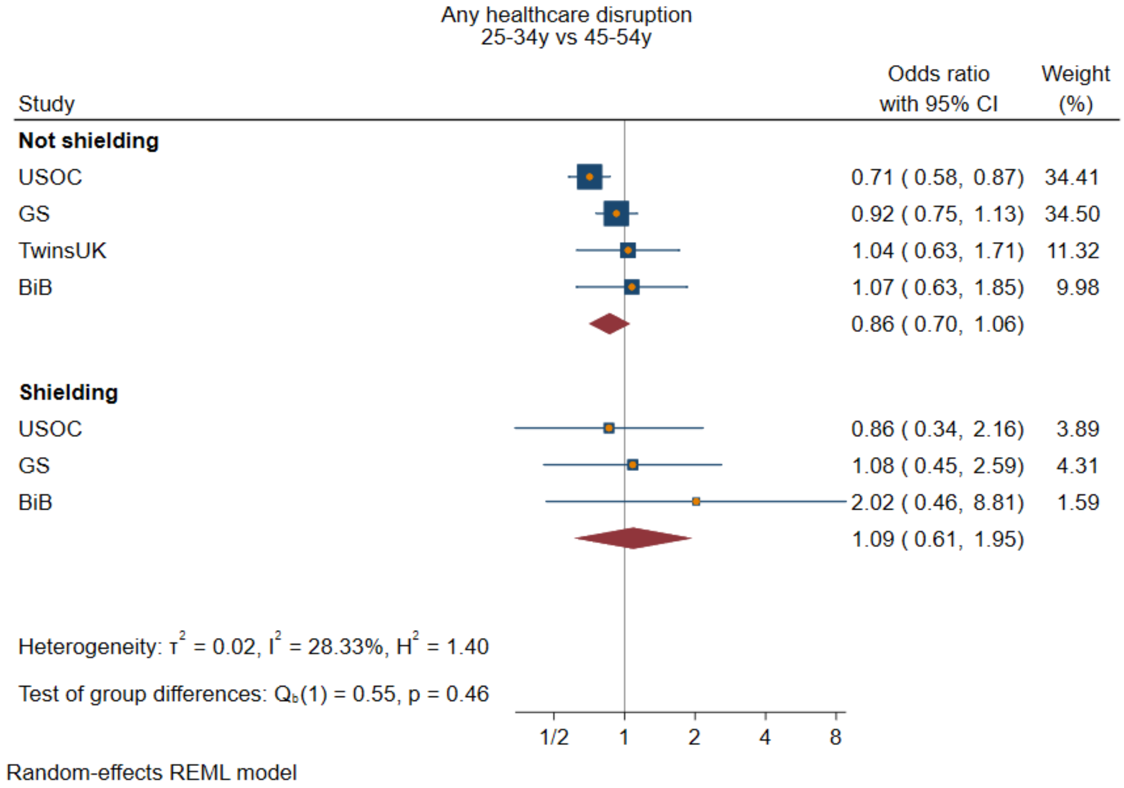
Any healthcare disruption
25-34y vs 45-54y
Study
Odds ratio with 95% CI
Weight (%)
Not shielding
USOC 0.71 (0.58, 0.87) 34.41
GS 0.92 (0.75, 1.13) 34.50
TwinsUK 1.04 (0.63, 1.71) 11.32
BiB 1.07 (0.63, 1.85) 9.98
0.86 (0.70, 1.06)
Shielding
USOC 0.86 (0.34, 2.16) 3.89
GS 1.08 (0.45, 2.59) 4.31
BiB 2.02 (0.46, 8.81) 1.59
1.09 (0.61, 1.95)
Heterogeneity: τ^2 = 0.02, I^2 = 28.33%, H^2 = 1.40
Test of group differences: $Q_b(1)$ = 0.55, p = 0.46
1/2
1
2
4
8
Random-effects REML model

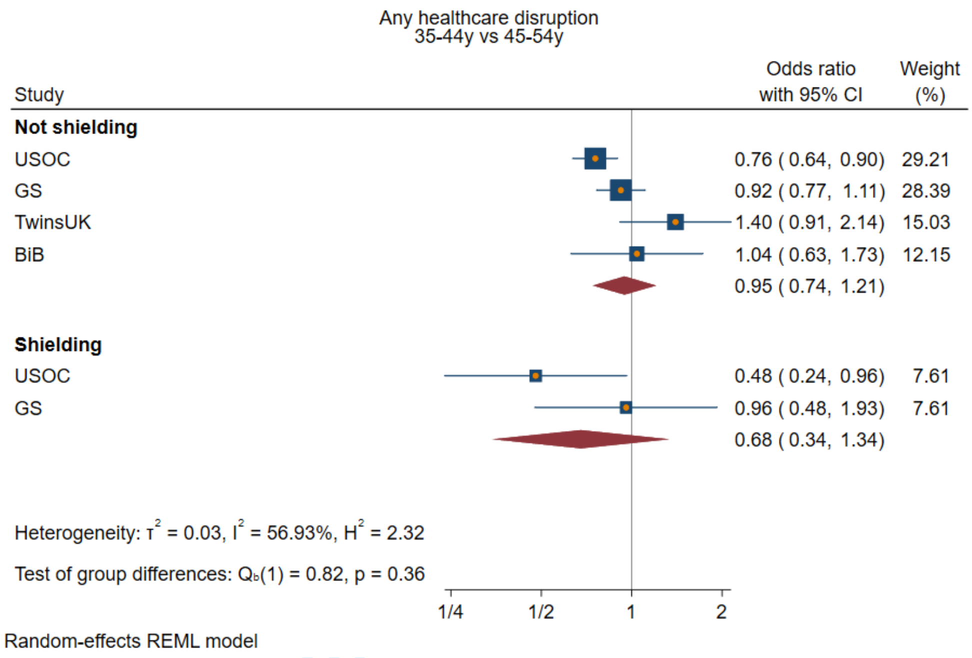
Any healthcare disruption
35-44y vs 45-54y
Study
Odds ratio with 95% CI
Weight (%)
Not shielding
USOC 0.76 (0.64, 0.90) 29.21
GS 0.92 (0.77, 1.11) 28.39
TwinsUK 1.40 (0.91, 2.14) 15.03
BiB 1.04 (0.63, 1.73) 12.15
0.95 (0.74, 1.21)
Shielding
USOC 0.48 (0.24, 0.96) 7.61
GS 0.96 (0.48, 1.93) 7.61
0.68 (0.34, 1.34)
Heterogeneity: τ2 = 0.03, I2 = 56.93%, H2 = 2.32
Test of group differences: Qb(1) = 0.82, p = 0.36
1/4
1/2
1
2
Random-effects REML model

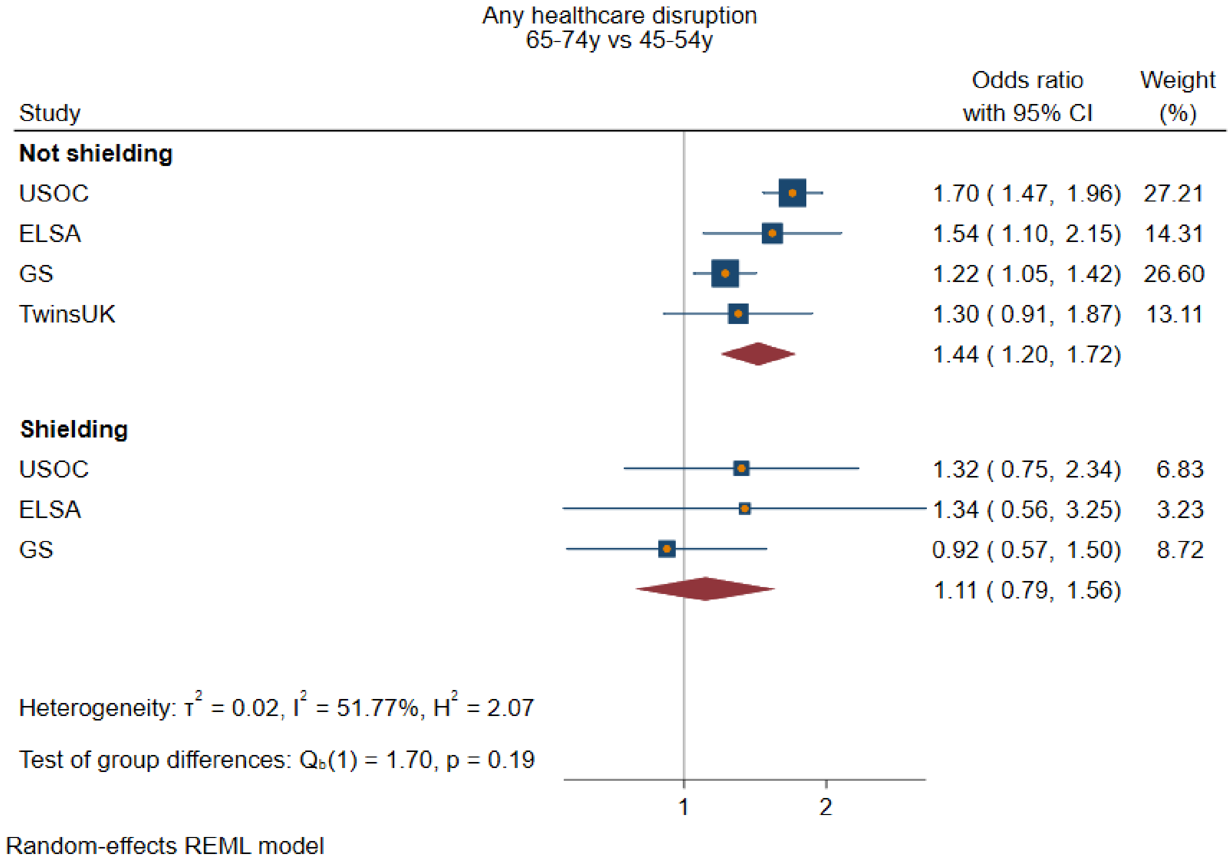
Any healthcare disruption
65-74y vs 45-54y
Study
Odds ratio with 95% CI
Weight (%)
Not shielding
USOC 1.70 (1.47, 1.96) 27.21
ELSA 1.54 (1.10, 2.15) 14.31
GS 1.22 (1.05, 1.42) 26.60
TwinsUK 1.30 (0.91, 1.87) 13.11
1.44 (1.20, 1.72)
Shielding
USOC 1.32 (0.75, 2.34) 6.83
ELSA 1.34 (0.56, 3.25) 3.23
GS 0.92 (0.57, 1.50) 8.72
1.11 (0.79, 1.56)
Heterogeneity: τ2 = 0.02, I2 = 51.77%, H2 = 2.07
Test of group differences: Qb(1) = 1.70, p = 0.19
1
2
Random-effects REML model

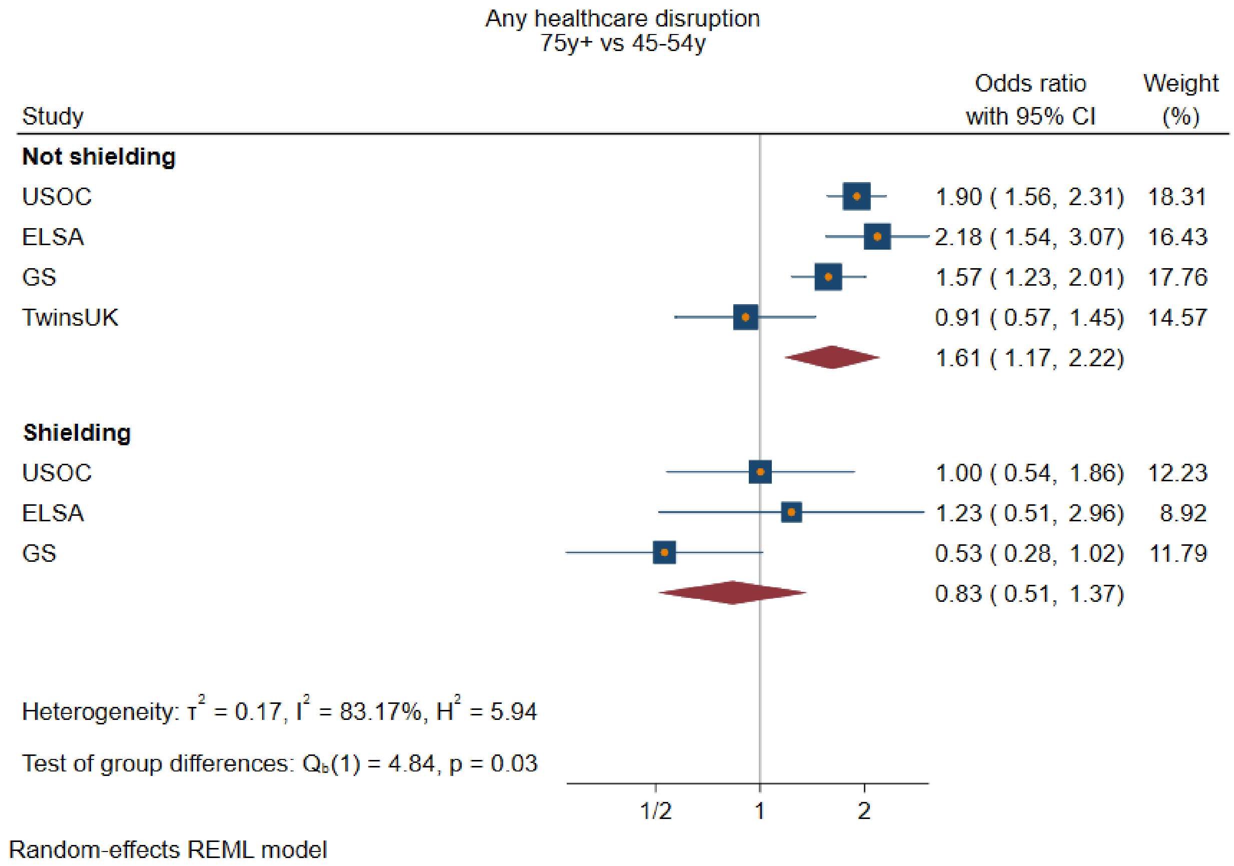

For peer review only - http://bmjopen.bmj.com/site/about/guidelines.xhtml

Ethnicity

For peer review only

For peer review only - http://bmjopen.bmj.com/site/about/guidelines.xhtml

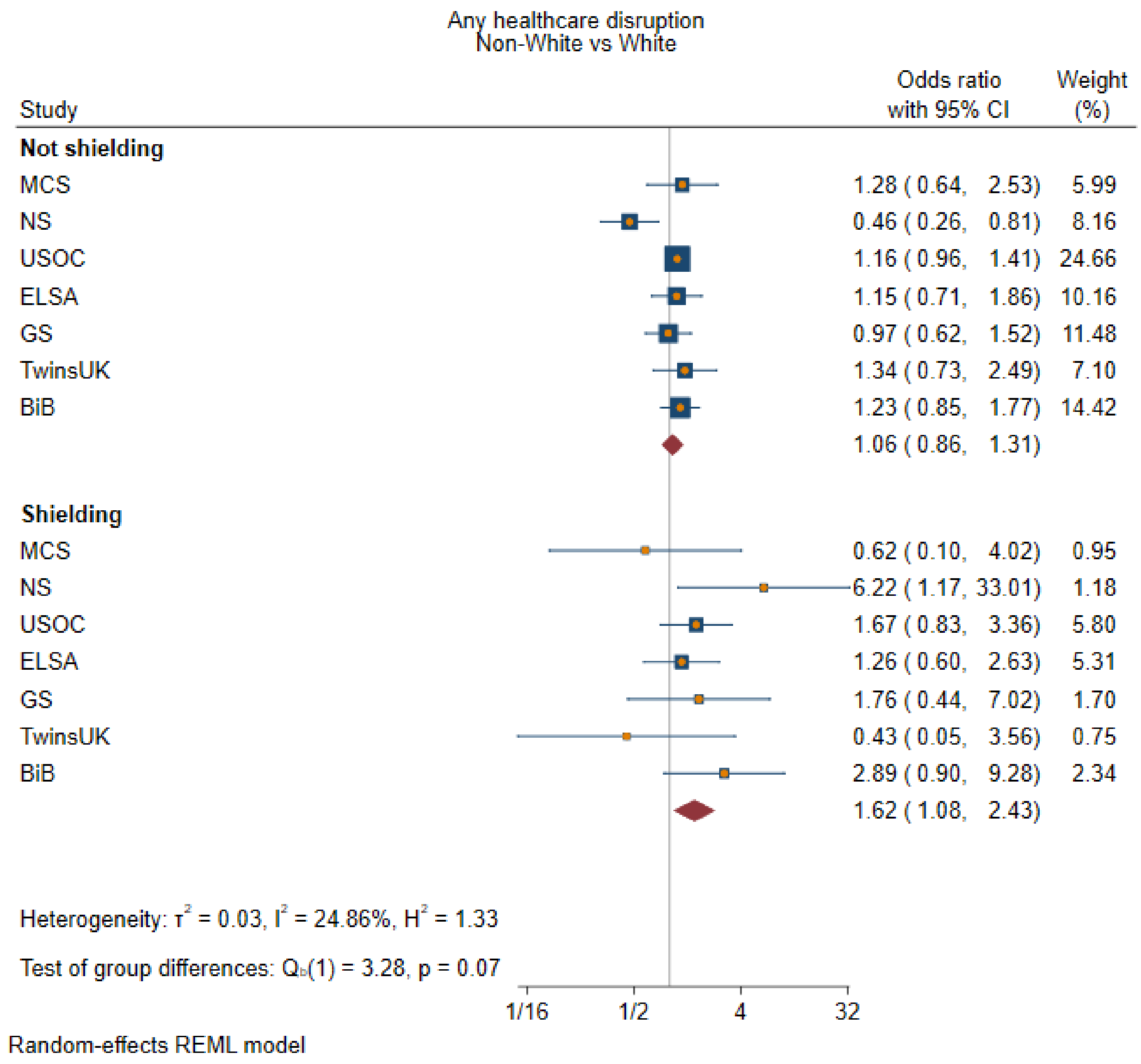

For peer review only - http://bmjopen.bmj.com/site/about/guidelines.xhtml

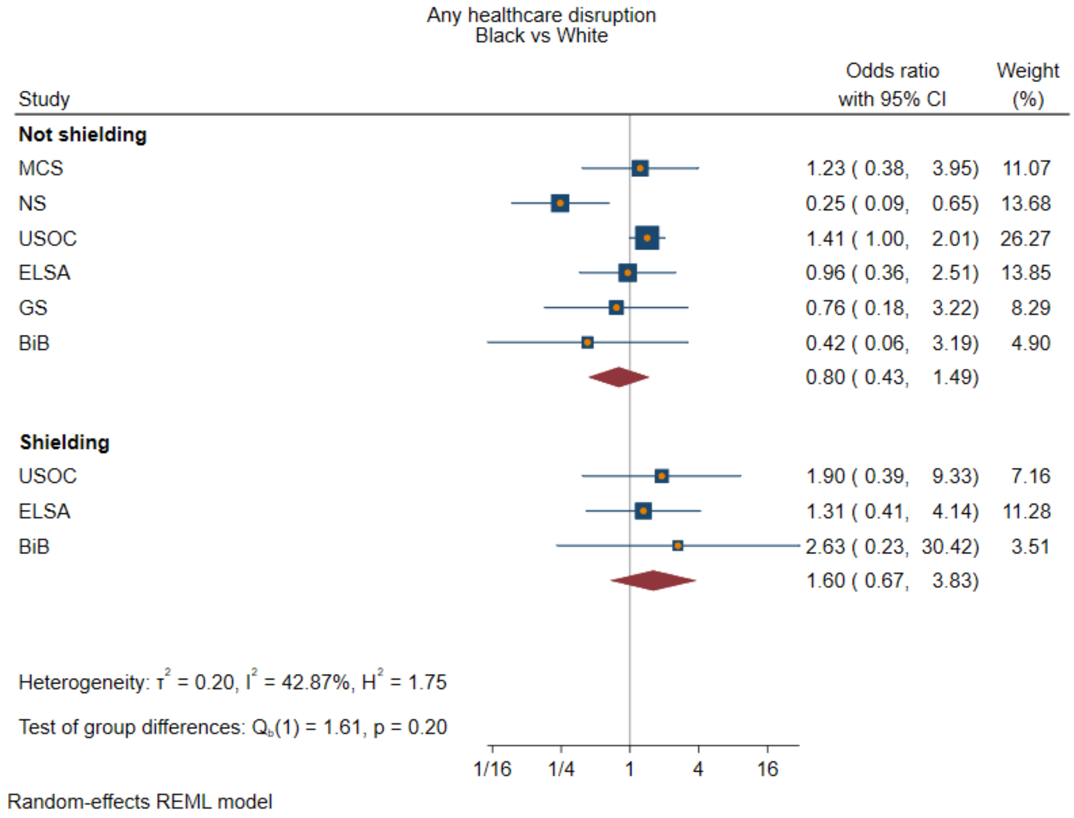

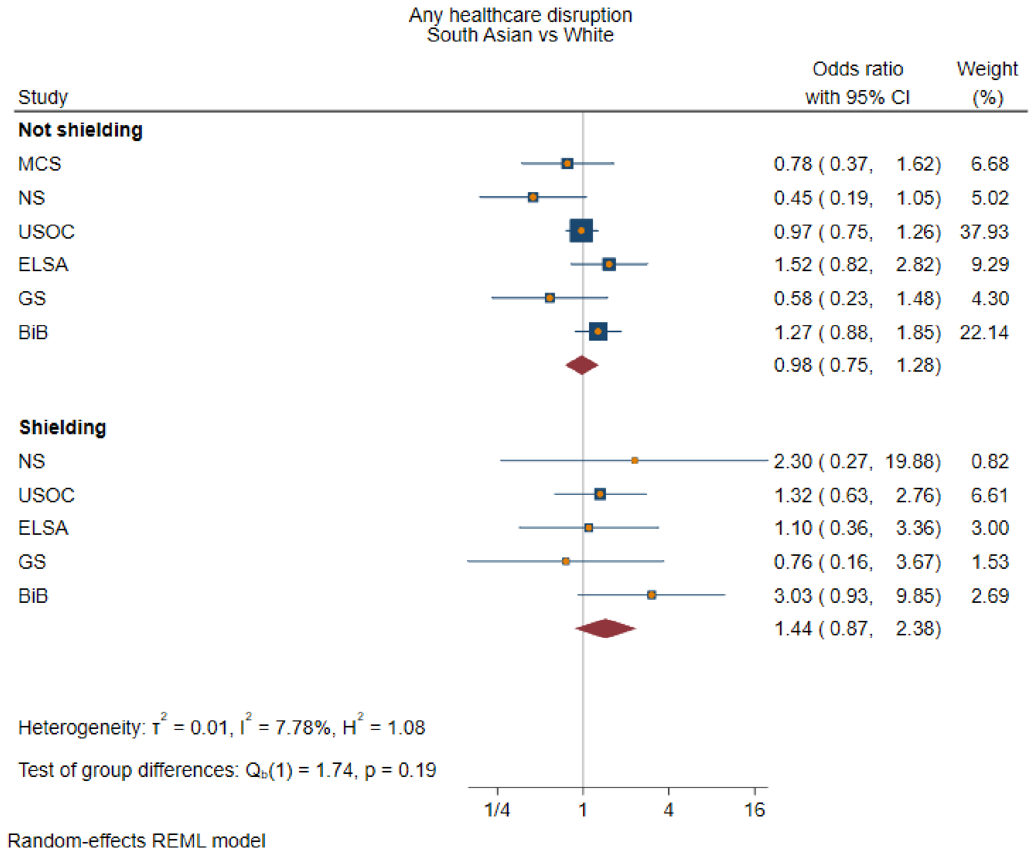

For peer review only - http://bmjopen.bmj.com/site/about/guidelines.xhtml

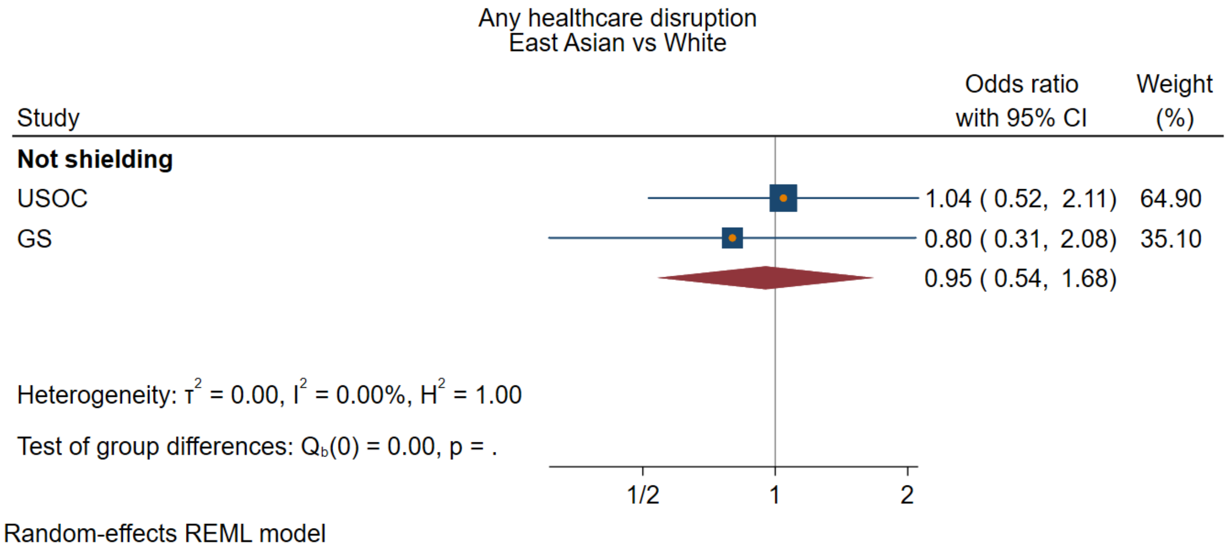

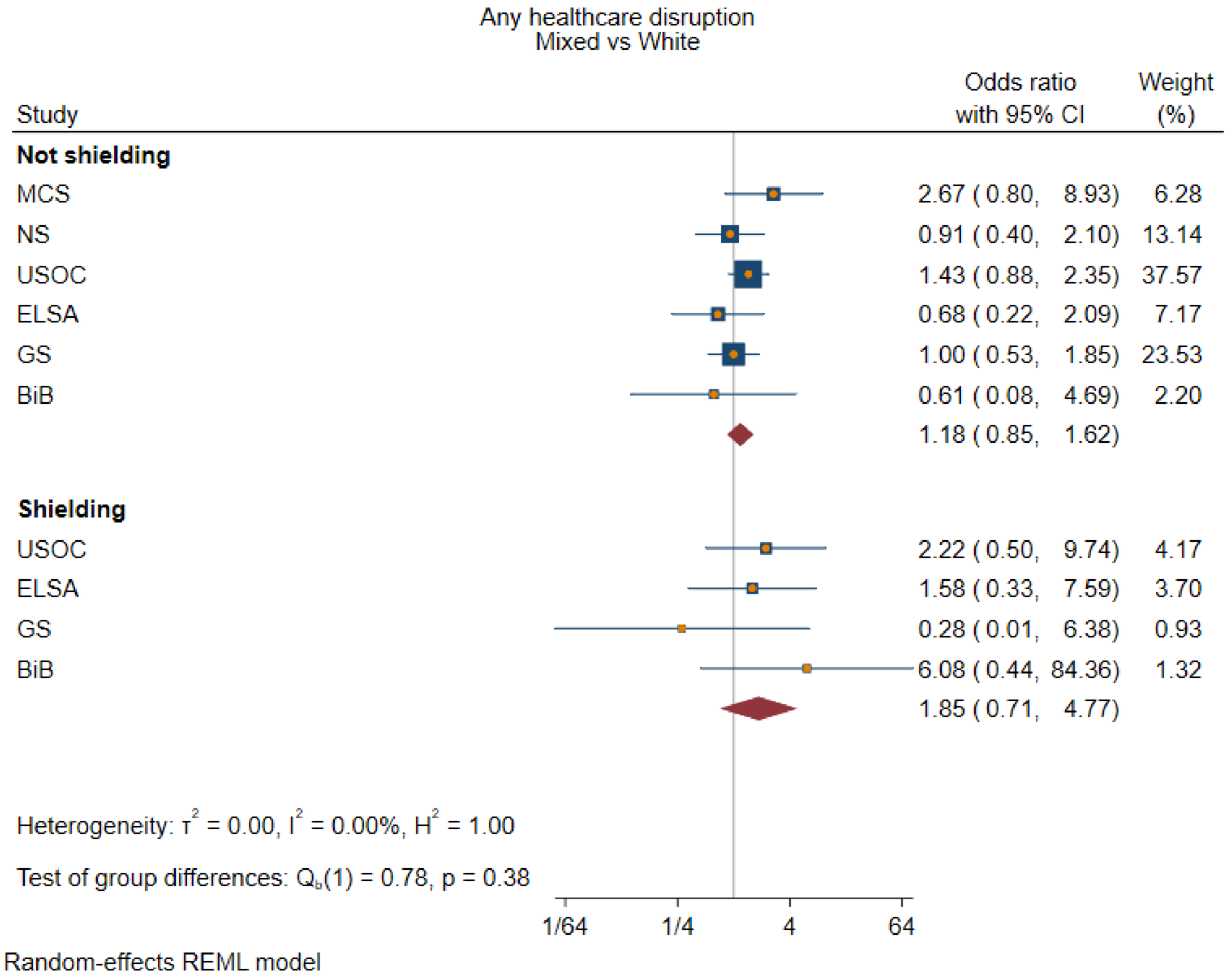

For peer review only - http://bmjopen.bmj.com/site/about/guidelines.xhtml

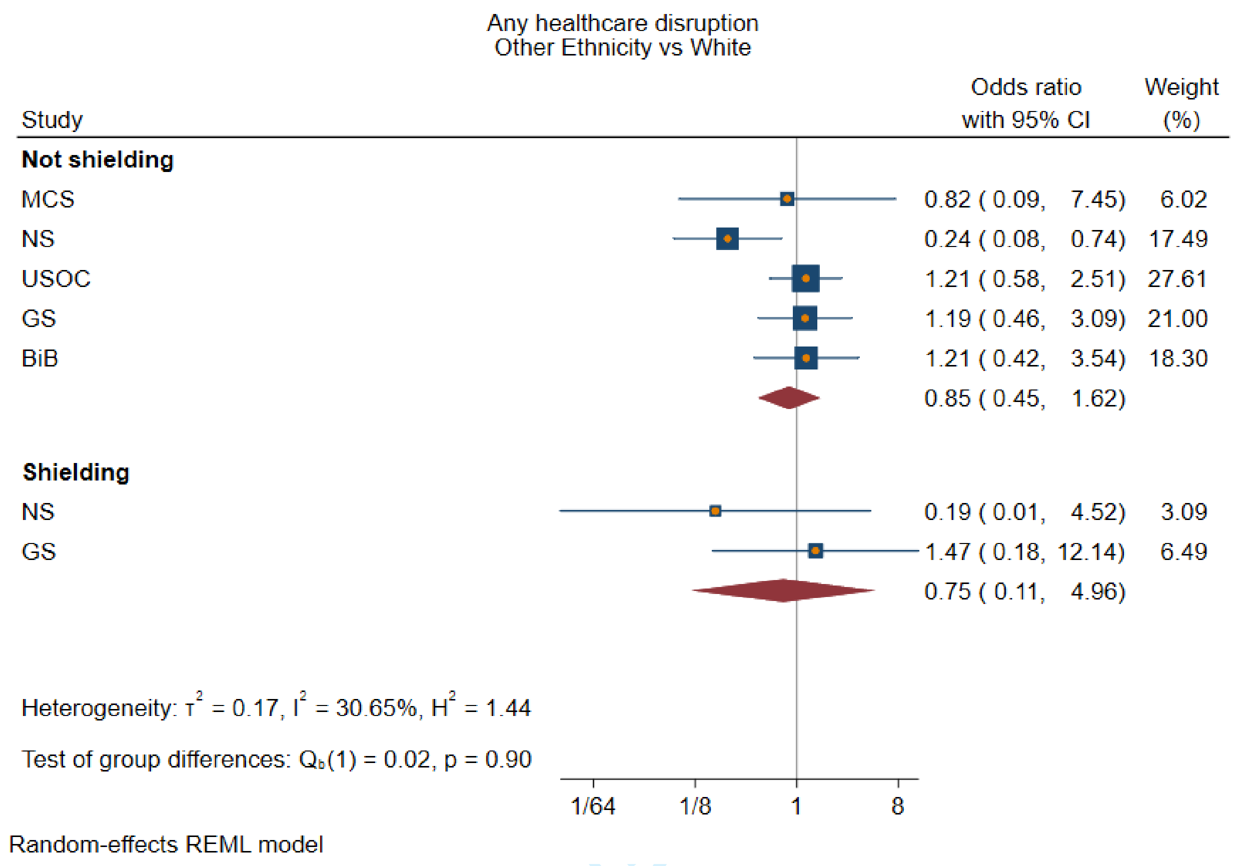
Any healthcare disruption
Other Ethnicity vs White
Study
Odds ratio with 95% CI
Weight (%)
Not shielding
MCS 0.82 (0.09, 7.45) 6.02
NS 0.24 (0.08, 0.74) 17.49
USOC 1.21 (0.58, 2.51) 27.61
GS 1.19 (0.46, 3.09) 21.00
BiB 1.21 (0.42, 3.54) 18.30
0.85 (0.45, 1.62)
Shielding
NS 0.19 (0.01, 4.52) 3.09
GS 1.47 (0.18, 12.14) 6.49
0.75 (0.11, 4.96)
Heterogeneity: τ2 = 0.17, I2 = 30.65%, H2 = 1.44
Test of group differences: Qb(1) = 0.02, p = 0.90
1/64
1/8
1
8
Random-effects REML model

Any healthcare disruption stratified by age

Sex

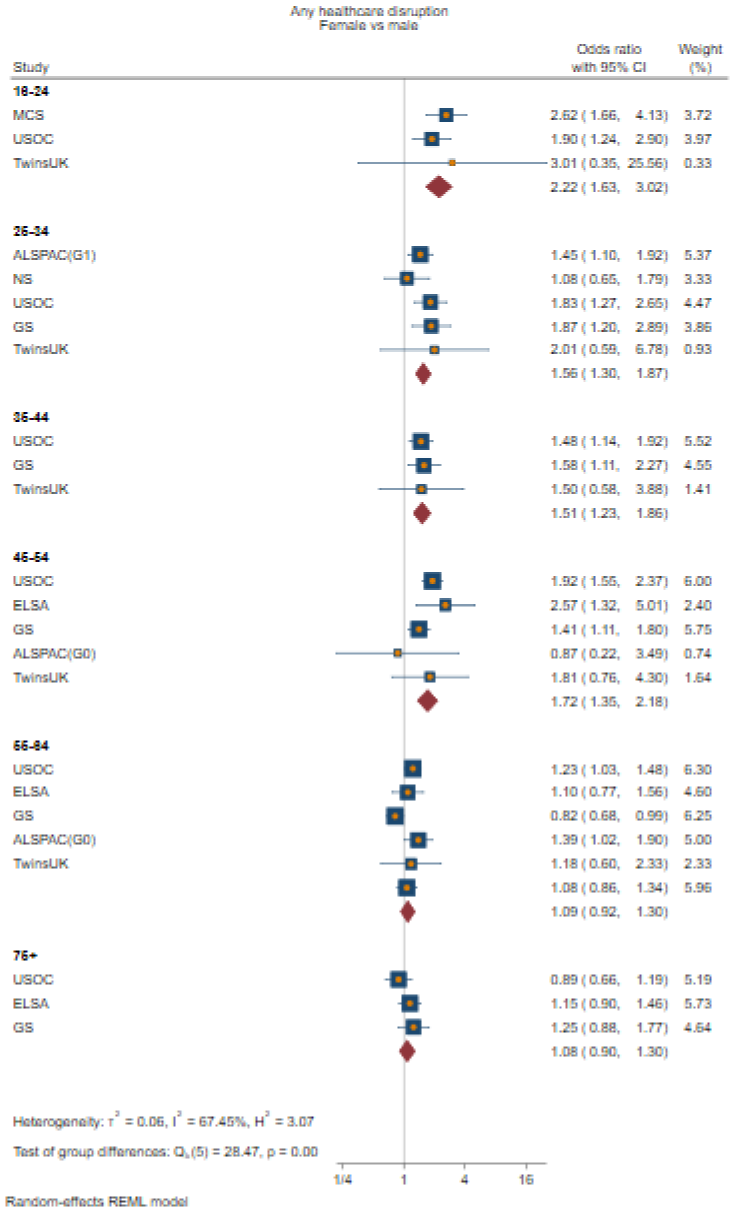

Occupational class

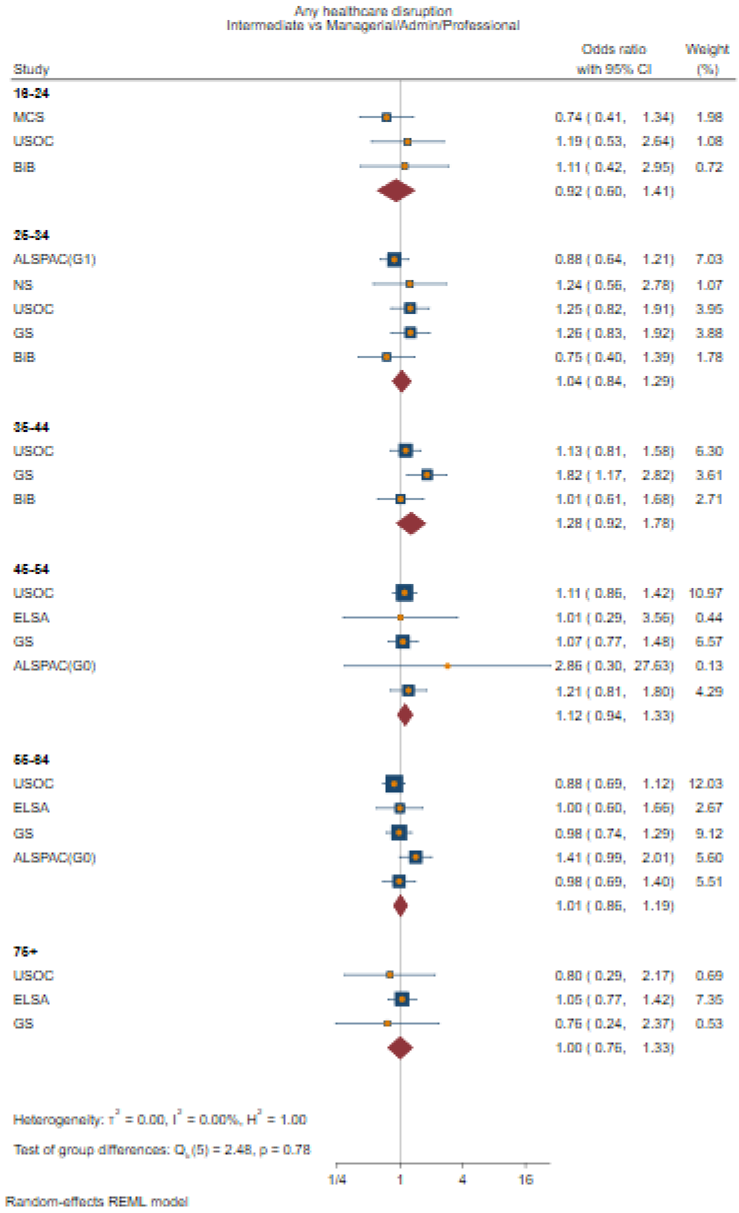

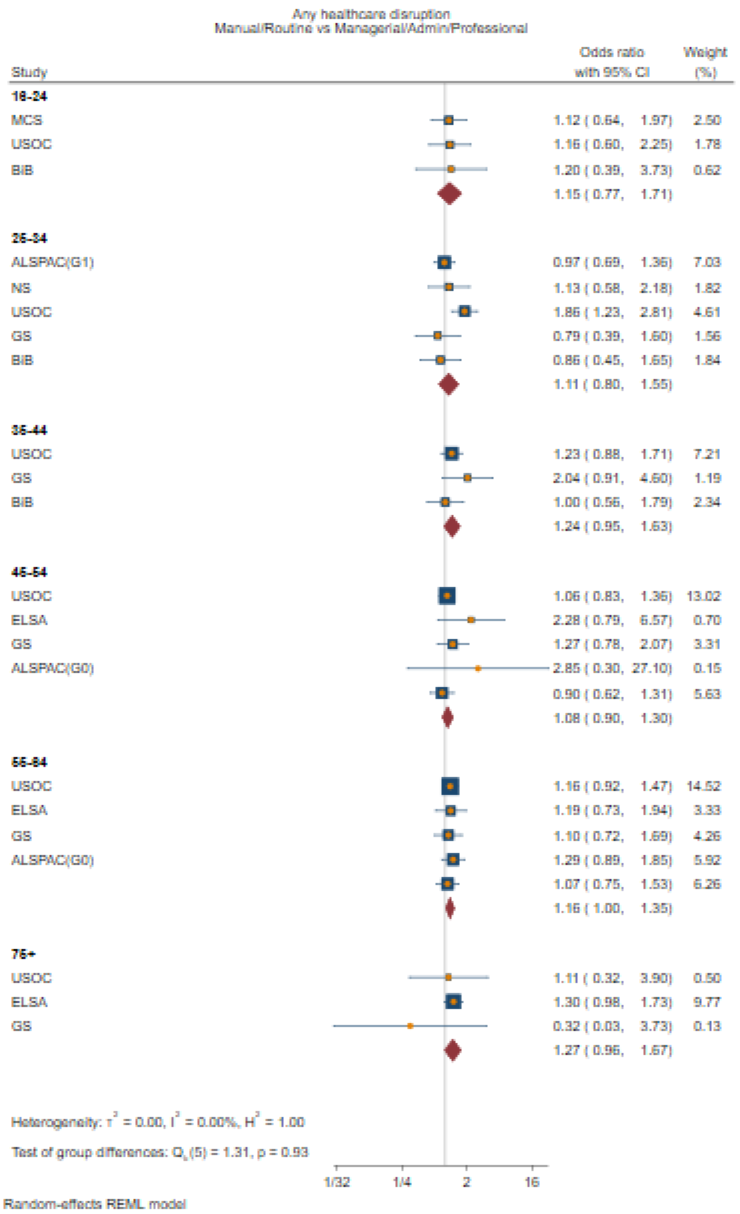
Any healthcare disruption
Manual/Routine vs Managerial/Admin/Professional
Study
Odds ratio with 95% CI
Weight (%)
18-24
MCS 1.12 (0.64, 1.97) 2.50
USOC 1.16 (0.60, 2.25) 1.78
BIB 1.20 (0.39, 3.73) 0.62
1.15 (0.77, 1.71)
25-34
ALSPAC(G1) 0.97 (0.69, 1.36) 7.03
NS 1.13 (0.58, 2.18) 1.82
USOC 1.86 (1.23, 2.81) 4.61
GS 0.79 (0.39, 1.60) 1.56
BIB 0.86 (0.45, 1.65) 1.84
1.11 (0.80, 1.55)
35-44
USOC 1.23 (0.88, 1.71) 7.21
GS 2.04 (0.91, 4.60) 1.19
BIB 1.00 (0.56, 1.79) 2.34
1.24 (0.95, 1.63)
45-54
USOC 1.06 (0.83, 1.36) 13.02
ELSA 2.28 (0.79, 6.57) 0.70
GS 1.27 (0.78, 2.07) 3.31
ALSPAC(G0) 2.85 (0.30, 27.10) 0.15
0.90 (0.62, 1.31) 5.63
1.08 (0.90, 1.30)
55-64
USOC 1.16 (0.92, 1.47) 14.52
ELSA 1.19 (0.73, 1.94) 3.33
GS 1.10 (0.72, 1.69) 4.26
ALSPAC(G0) 1.29 (0.89, 1.85) 5.92
1.07 (0.75, 1.53) 6.26
1.16 (1.00, 1.35)
75+
USOC 1.11 (0.32, 3.90) 0.50
ELSA 1.30 (0.98, 1.73) 9.77
GS 0.32 (0.03, 3.73) 0.13
1.27 (0.96, 1.67)
Heterogeneity: τ² = 0.00, I² = 0.00%, H² = 1.00
Test of group differences: Q_b(5) = 1.31, p = 0.93
1/32
1/4
2
16
Random-effects REML model

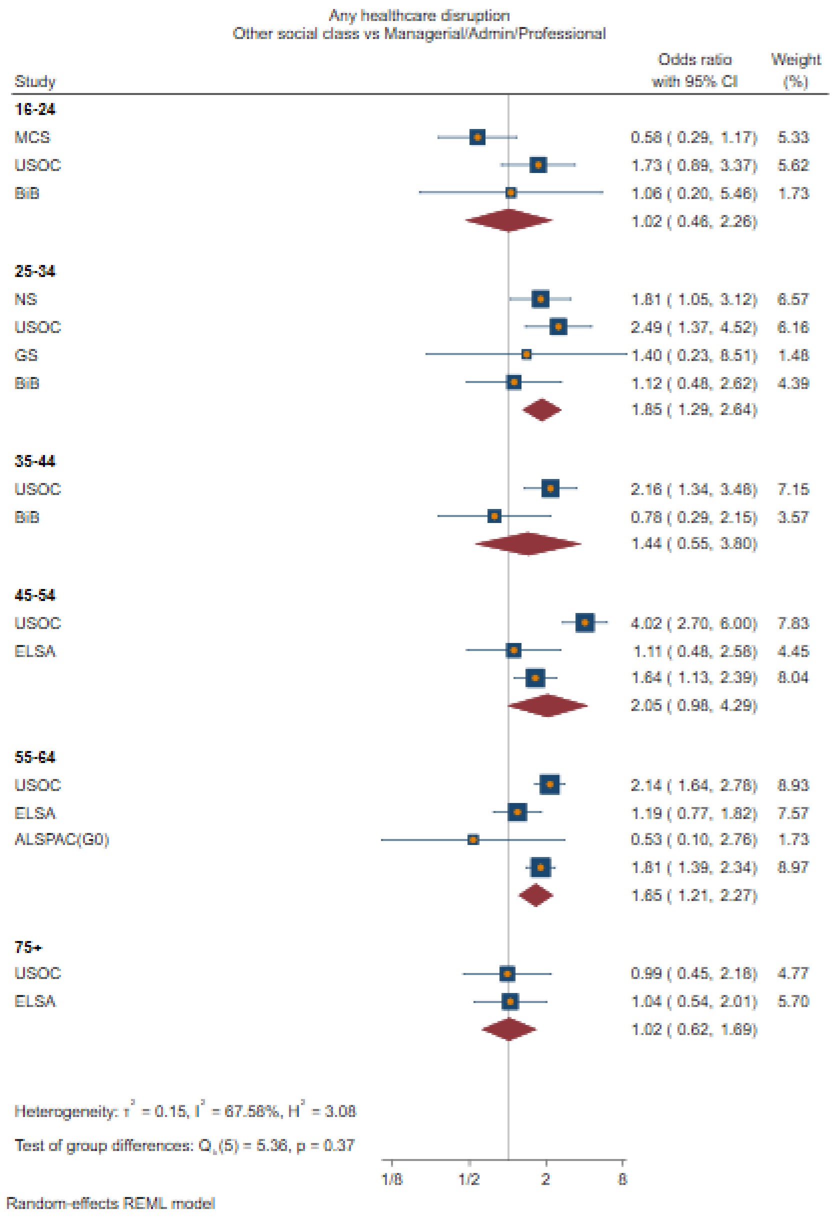
Any healthcare disruption
Other social class vs Managerial/Admin/Professional
Study
Odds ratio with 95% CI
Weight (%)
16-24
MCS 0.58 (0.29, 1.17) 5.33
USOC 1.73 (0.89, 3.37) 5.62
BiB 1.06 (0.20, 5.46) 1.73
1.02 (0.46, 2.26)
25-34
NS 1.81 (1.05, 3.12) 6.57
USOC 2.49 (1.37, 4.52) 6.16
GS 1.40 (0.23, 8.51) 1.48
BiB 1.12 (0.48, 2.62) 4.39
1.85 (1.29, 2.64)
35-44
USOC 2.16 (1.34, 3.48) 7.15
BiB 0.78 (0.29, 2.15) 3.57
1.44 (0.55, 3.80)
45-54
USOC 4.02 (2.70, 6.00) 7.83
ELSA 1.11 (0.48, 2.58) 4.45
1.64 (1.13, 2.39) 8.04
2.05 (0.98, 4.29)
55-64
USOC 2.14 (1.64, 2.78) 8.93
ELSA 1.19 (0.77, 1.82) 7.57
ALSPAC(G0) 0.53 (0.10, 2.76) 1.73
1.81 (1.39, 2.34) 8.97
1.65 (1.21, 2.27)
75+
USOC 0.99 (0.45, 2.18) 4.77
ELSA 1.04 (0.54, 2.01) 5.70
1.02 (0.62, 1.69)
Heterogeneity: τ² = 0.15, I² = 67.58%, H² = 3.08
Test of group differences: Qb(5) = 5.36, p = 0.37
1/8
1/2
2
8
Random-effects REML model

Education

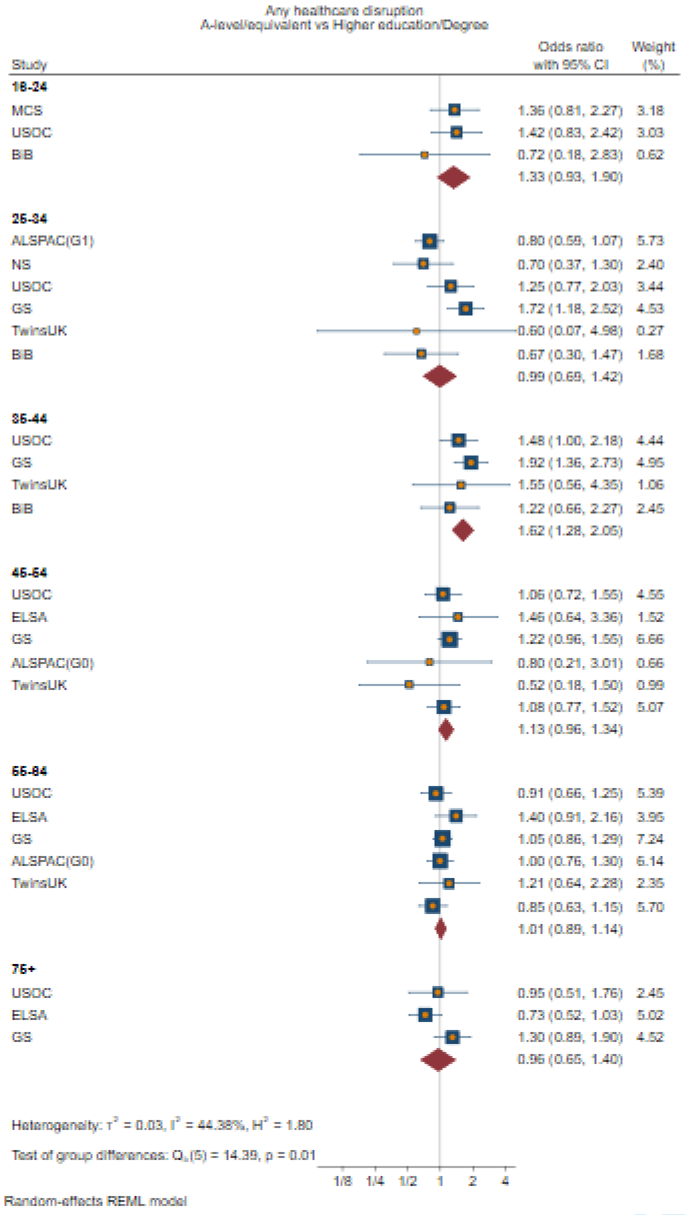
Any healthcare disruption
A-level/equivalent vs Higher education/Degree
Study
Odds ratio with 95% CI
Weight (%)
18-24
MCS 1.36 (0.81, 2.27) 3.18
USOC 1.42 (0.83, 2.42) 3.03
BIB 0.72 (0.18, 2.83) 0.62
1.33 (0.93, 1.90)
25-34
ALSPAC(G1) 0.80 (0.59, 1.07) 5.73
NS 0.70 (0.37, 1.30) 2.40
USOC 1.25 (0.77, 2.03) 3.44
GS 1.72 (1.18, 2.52) 4.53
TwinsUK 0.60 (0.07, 4.98) 0.27
BIB 0.67 (0.30, 1.47) 1.68
0.99 (0.69, 1.42)
35-44
USOC 1.48 (1.00, 2.18) 4.44
GS 1.92 (1.36, 2.73) 4.95
TwinsUK 1.55 (0.56, 4.35) 1.06
BIB 1.22 (0.66, 2.27) 2.45
1.62 (1.28, 2.05)
45-54
USOC 1.06 (0.72, 1.55) 4.55
ELSA 1.46 (0.64, 3.36) 1.52
GS 1.22 (0.96, 1.55) 6.66
ALSPAC(G0) 0.80 (0.21, 3.01) 0.66
TwinsUK 0.52 (0.18, 1.50) 0.99
1.08 (0.77, 1.52) 5.07
1.13 (0.96, 1.34)
55-64
USOC 0.91 (0.66, 1.25) 5.39
ELSA 1.40 (0.91, 2.16) 3.95
GS 1.05 (0.86, 1.29) 7.24
ALSPAC(G0) 1.00 (0.76, 1.30) 6.14
TwinsUK 1.21 (0.64, 2.28) 2.35
0.85 (0.63, 1.15) 5.70
1.01 (0.89, 1.14)
75+
USOC 0.95 (0.51, 1.76) 2.45
ELSA 0.73 (0.52, 1.03) 5.02
GS 1.30 (0.89, 1.90) 4.52
0.96 (0.65, 1.40)
Heterogeneity: τ² = 0.03, I² = 44.38%, H² = 1.80
Test of group differences: Q_b(5) = 14.39, p = 0.01
1/8 1/4 1/2 1 2 4
Random-effects REML model

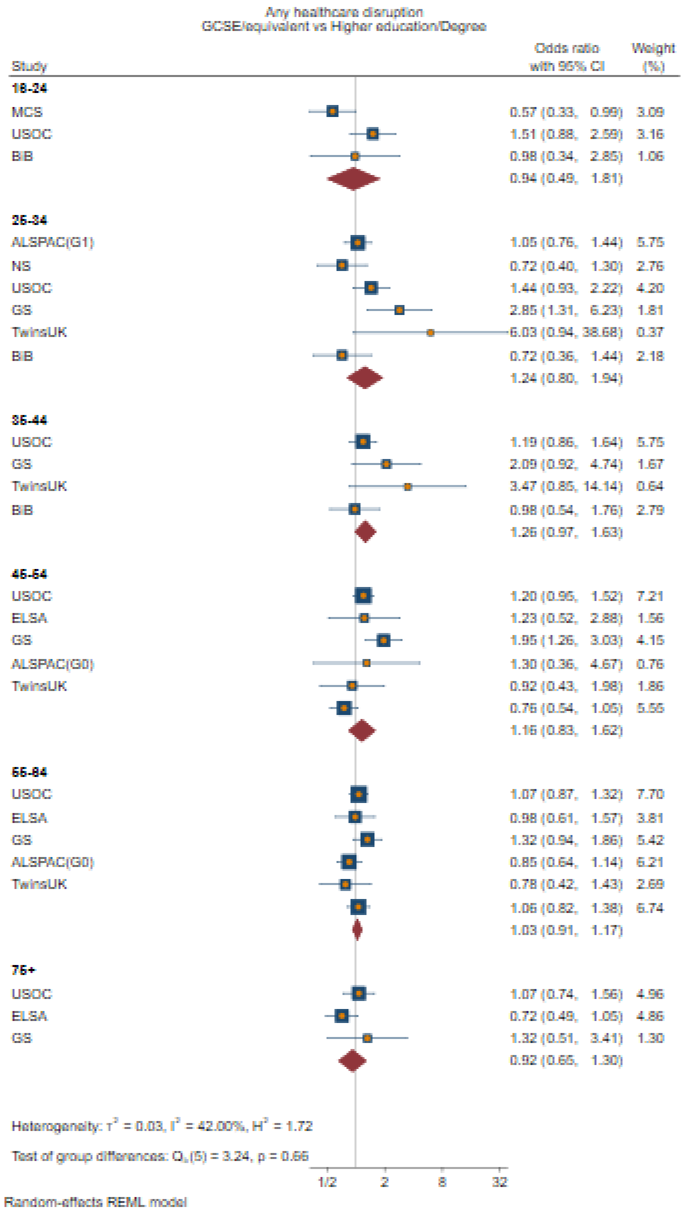
Any healthcare disruption
GCSE/equivalent vs Higher education/Degree
Study
Odds ratio with 95% CI
Weight (%)
18-24
MCS 0.57 (0.33, 0.99) 3.09
USOC 1.51 (0.88, 2.59) 3.16
BIB 0.98 (0.34, 2.85) 1.06
0.94 (0.49, 1.81)
25-34
ALSPAC(G1) 1.05 (0.76, 1.44) 5.75
NS 0.72 (0.40, 1.30) 2.76
USOC 1.44 (0.93, 2.22) 4.20
GS 2.85 (1.31, 6.23) 1.81
TwinsUK 6.03 (0.94, 38.68) 0.37
BIB 0.72 (0.36, 1.44) 2.18
1.24 (0.80, 1.94)
35-44
USOC 1.19 (0.86, 1.64) 5.75
GS 2.09 (0.92, 4.74) 1.67
TwinsUK 3.47 (0.85, 14.14) 0.64
BIB 0.98 (0.54, 1.76) 2.79
1.26 (0.97, 1.63)
45-54
USOC 1.20 (0.95, 1.52) 7.21
ELSA 1.23 (0.52, 2.88) 1.56
GS 1.95 (1.26, 3.03) 4.15
ALSPAC(G0) 1.30 (0.36, 4.67) 0.76
TwinsUK 0.92 (0.43, 1.98) 1.86
0.76 (0.54, 1.05) 5.55
1.16 (0.83, 1.62)
55-64
USOC 1.07 (0.87, 1.32) 7.70
ELSA 0.98 (0.61, 1.57) 3.81
GS 1.32 (0.94, 1.86) 5.42
ALSPAC(G0) 0.85 (0.64, 1.14) 6.21
TwinsUK 0.78 (0.42, 1.43) 2.69
1.06 (0.82, 1.38) 6.74
1.03 (0.91, 1.17)
75+
USOC 1.07 (0.74, 1.56) 4.96
ELSA 0.72 (0.49, 1.05) 4.86
GS 1.32 (0.51, 3.41) 1.30
0.92 (0.65, 1.30)
Heterogeneity: τ² = 0.03, I² = 42.00%, H² = 1.72
Test of group differences: Qb(5) = 3.24, p = 0.66
1/2 2 8 32
Random-effects REML model

153

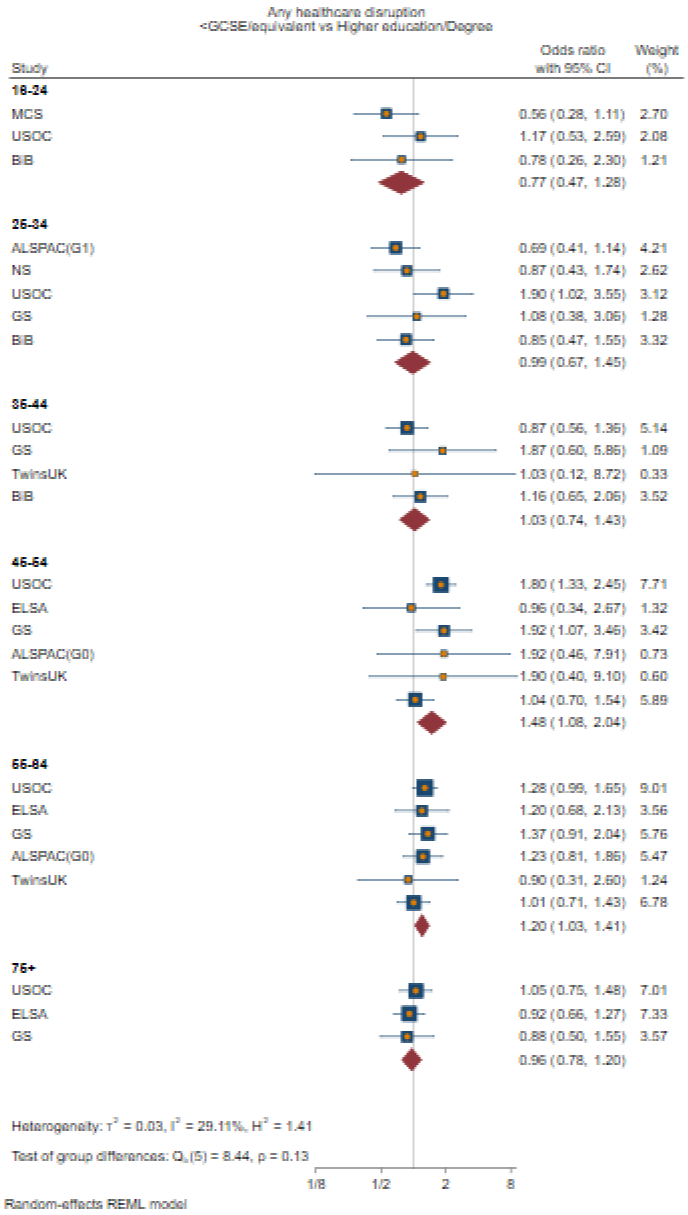

For peer review only - http://bmjopen.bmj.com/site/about/guidelines.xhtml

Ethnicity

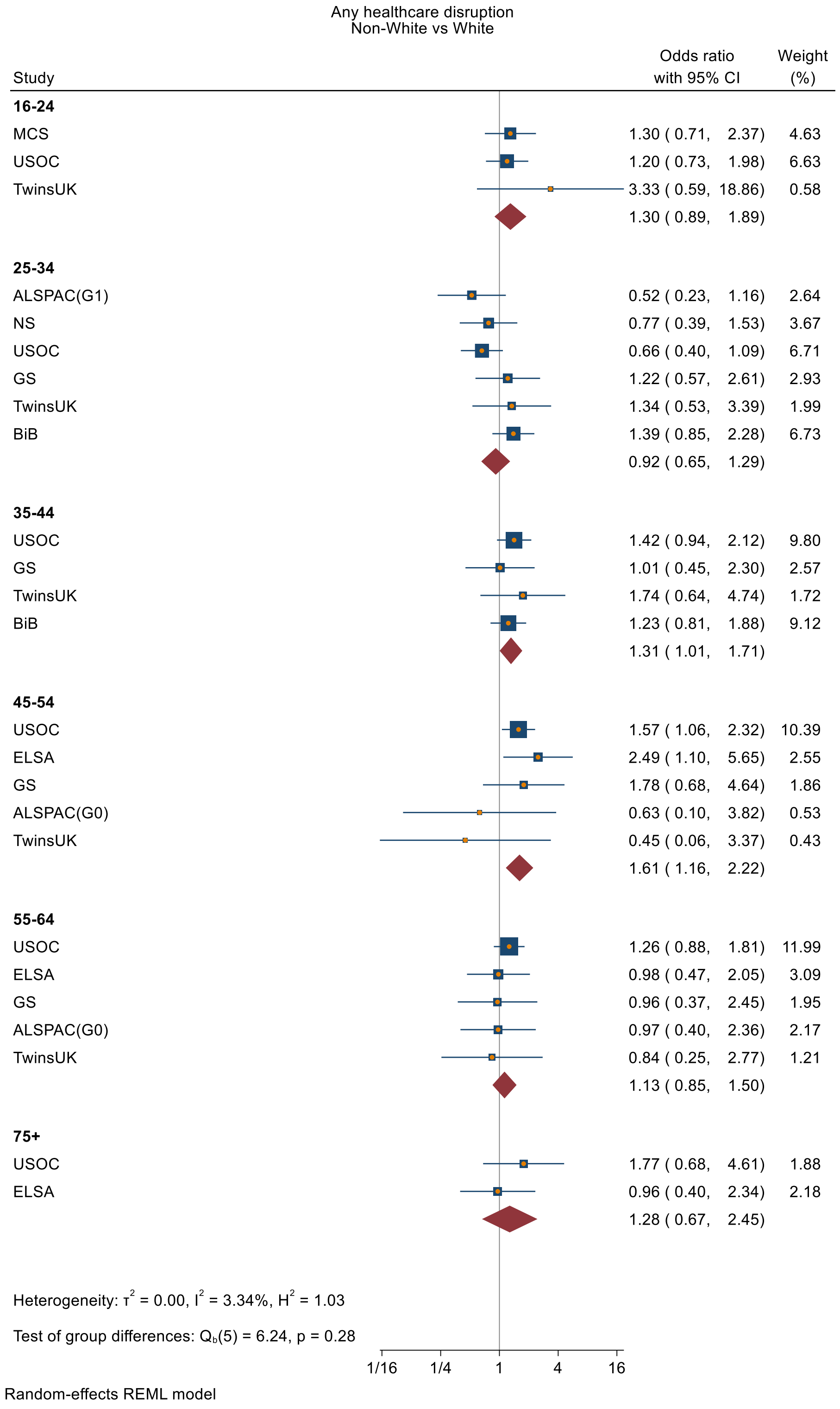
Any healthcare disruption
Non-White vs White
Study
Odds ratio with 95% CI
Weight (%)
16-24
MCS 1.30 (0.71, 2.37) 4.63
USOC 1.20 (0.73, 1.98) 6.63
TwinsUK 3.33 (0.59, 18.86) 0.58
1.30 (0.89, 1.89)
25-34
ALSPAC(G1) 0.52 (0.23, 1.16) 2.64
NS 0.77 (0.39, 1.53) 3.67
USOC 0.66 (0.40, 1.09) 6.71
GS 1.22 (0.57, 2.61) 2.93
TwinsUK 1.34 (0.53, 3.39) 1.99
BiB 1.39 (0.85, 2.28) 6.73
0.92 (0.65, 1.29)
35-44
USOC 1.42 (0.94, 2.12) 9.80
GS 1.01 (0.45, 2.30) 2.57
TwinsUK 1.74 (0.64, 4.74) 1.72
BiB 1.23 (0.81, 1.88) 9.12
1.31 (1.01, 1.71)
45-54
USOC 1.57 (1.06, 2.32) 10.39
ELSA 2.49 (1.10, 5.65) 2.55
GS 1.78 (0.68, 4.64) 1.86
ALSPAC(G0) 0.63 (0.10, 3.82) 0.53
TwinsUK 0.45 (0.06, 3.37) 0.43
1.61 (1.16, 2.22)
55-64
USOC 1.26 (0.88, 1.81) 11.99
ELSA 0.98 (0.47, 2.05) 3.09
GS 0.96 (0.37, 2.45) 1.95
ALSPAC(G0) 0.97 (0.40, 2.36) 2.17
TwinsUK 0.84 (0.25, 2.77) 1.21
1.13 (0.85, 1.50)
75+
USOC 1.77 (0.68, 4.61) 1.88
ELSA 0.96 (0.40, 2.34) 2.18
1.28 (0.67, 2.45)
Heterogeneity: τ² = 0.00, I² = 3.34%, H² = 1.03
Test of group differences: Q_b(5) = 6.24, p = 0.28
1/16
1/4
1
4
16
Random-effects REML model

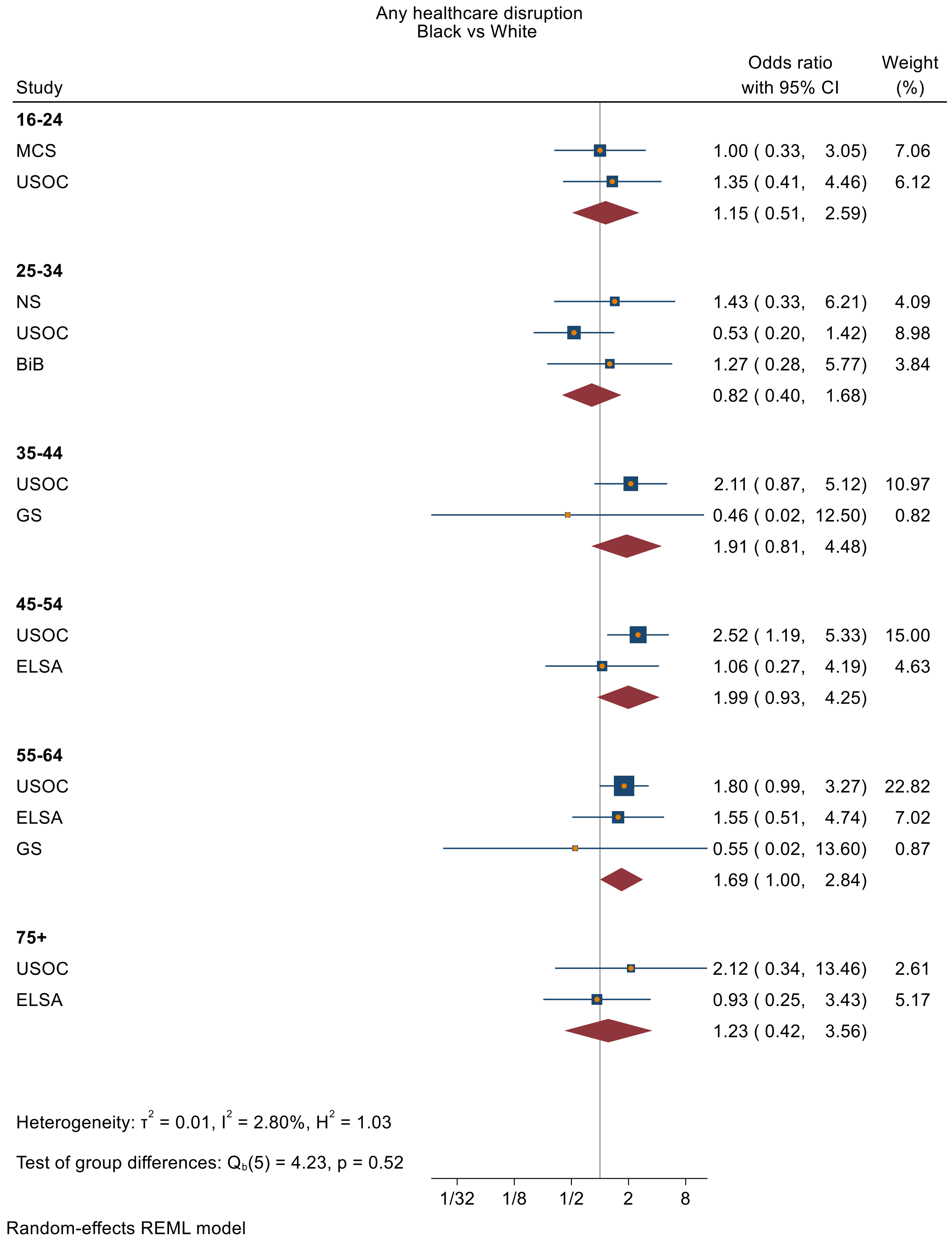

For peer review only - http://bmjopen.bmj.com/site/about/guidelines.xhtml

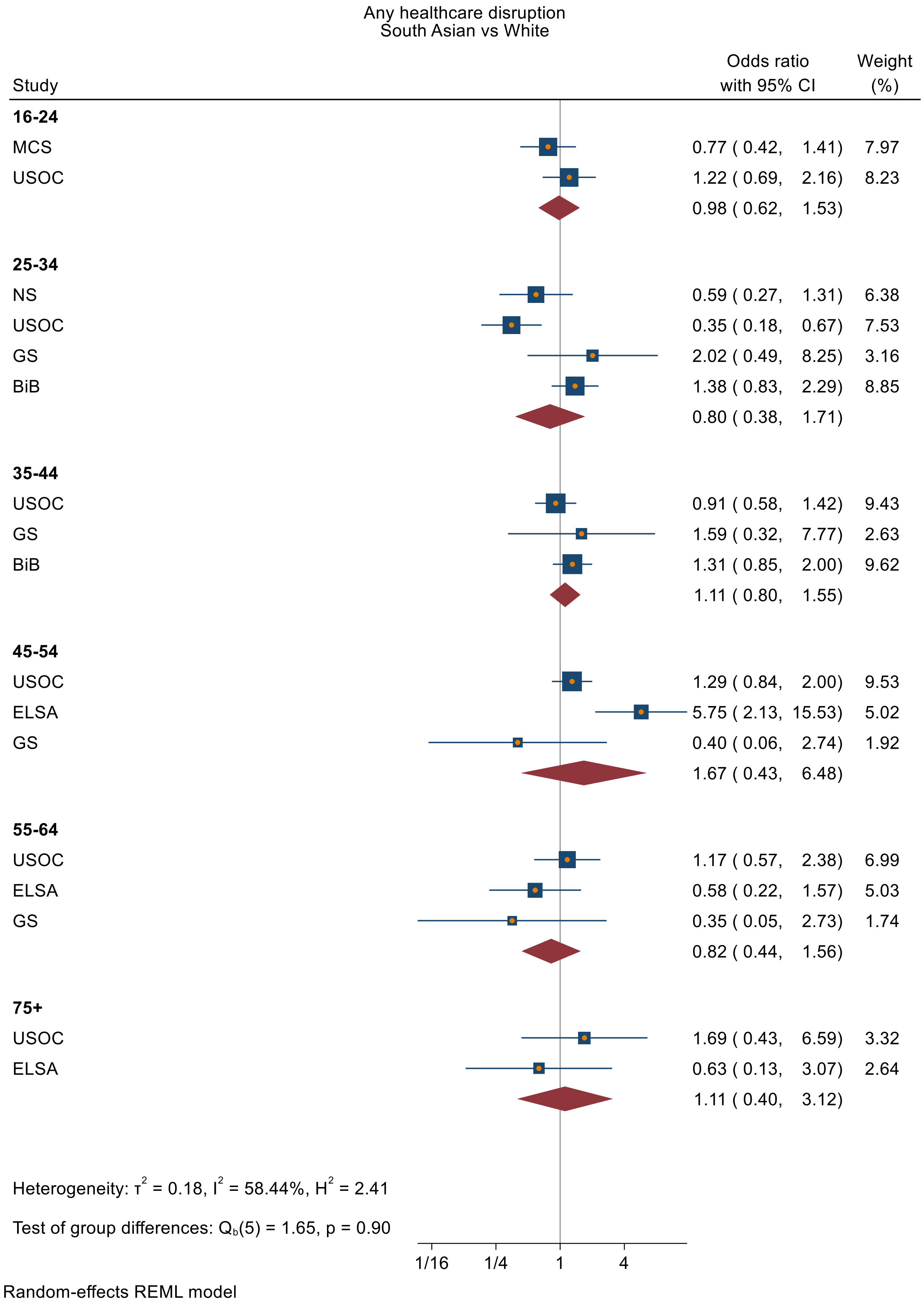
Any healthcare disruption
South Asian vs White
Study
Odds ratio with 95% CI
Weight (%)
16-24
MCS 0.77 (0.42, 1.41) 7.97
USOC 1.22 (0.69, 2.16) 8.23
0.98 (0.62, 1.53)
25-34
NS 0.59 (0.27, 1.31) 6.38
USOC 0.35 (0.18, 0.67) 7.53
GS 2.02 (0.49, 8.25) 3.16
BiB 1.38 (0.83, 2.29) 8.85
0.80 (0.38, 1.71)
35-44
USOC 0.91 (0.58, 1.42) 9.43
GS 1.59 (0.32, 7.77) 2.63
BiB 1.31 (0.85, 2.00) 9.62
1.11 (0.80, 1.55)
45-54
USOC 1.29 (0.84, 2.00) 9.53
ELSA 5.75 (2.13, 15.53) 5.02
GS 0.40 (0.06, 2.74) 1.92
1.67 (0.43, 6.48)
55-64
USOC 1.17 (0.57, 2.38) 6.99
ELSA 0.58 (0.22, 1.57) 5.03
GS 0.35 (0.05, 2.73) 1.74
0.82 (0.44, 1.56)
75+
USOC 1.69 (0.43, 6.59) 3.32
ELSA 0.63 (0.13, 3.07) 2.64
1.11 (0.40, 3.12)
Heterogeneity: τ² = 0.18, I² = 58.44%, H² = 2.41
Test of group differences: Q_b(5) = 1.65, p = 0.90
1/16
1/4
1
4
Random-effects REML model

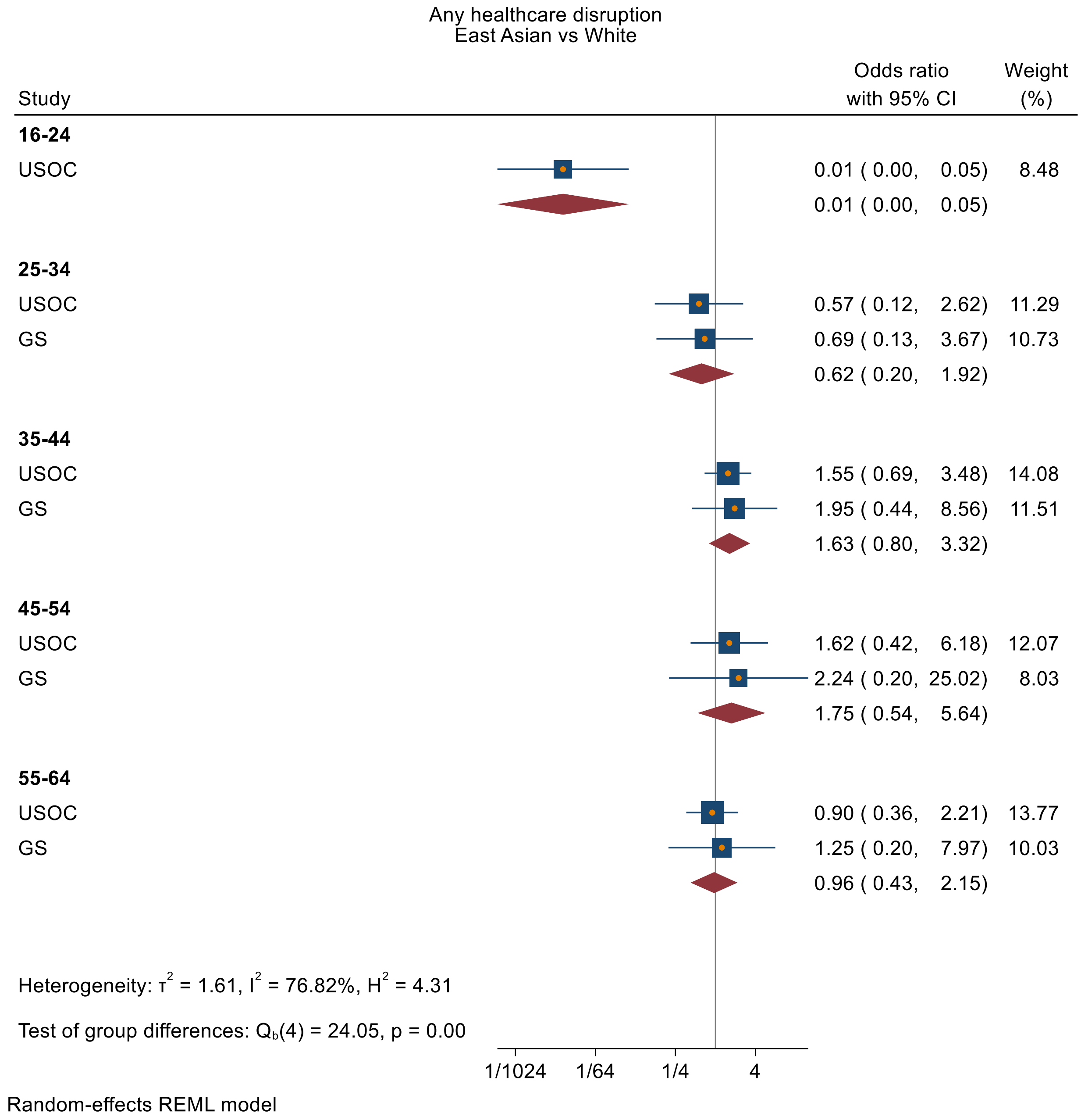
Any healthcare disruption
East Asian vs White
Study
Odds ratio with 95% CI
Weight (%)
16-24
USOC 0.01 (0.00, 0.05) 8.48
0.01 (0.00, 0.05)
25-34
USOC 0.57 (0.12, 2.62) 11.29
GS 0.69 (0.13, 3.67) 10.73
0.62 (0.20, 1.92)
35-44
USOC 1.55 (0.69, 3.48) 14.08
GS 1.95 (0.44, 8.56) 11.51
1.63 (0.80, 3.32)
45-54
USOC 1.62 (0.42, 6.18) 12.07
GS 2.24 (0.20, 25.02) 8.03
1.75 (0.54, 5.64)
55-64
USOC 0.90 (0.36, 2.21) 13.77
GS 1.25 (0.20, 7.97) 10.03
0.96 (0.43, 2.15)
Heterogeneity: τ² = 1.61, I² = 76.82%, H² = 4.31
Test of group differences: Q_b(4) = 24.05, p = 0.00
1/1024
1/64
1/4
4
Random-effects REML model

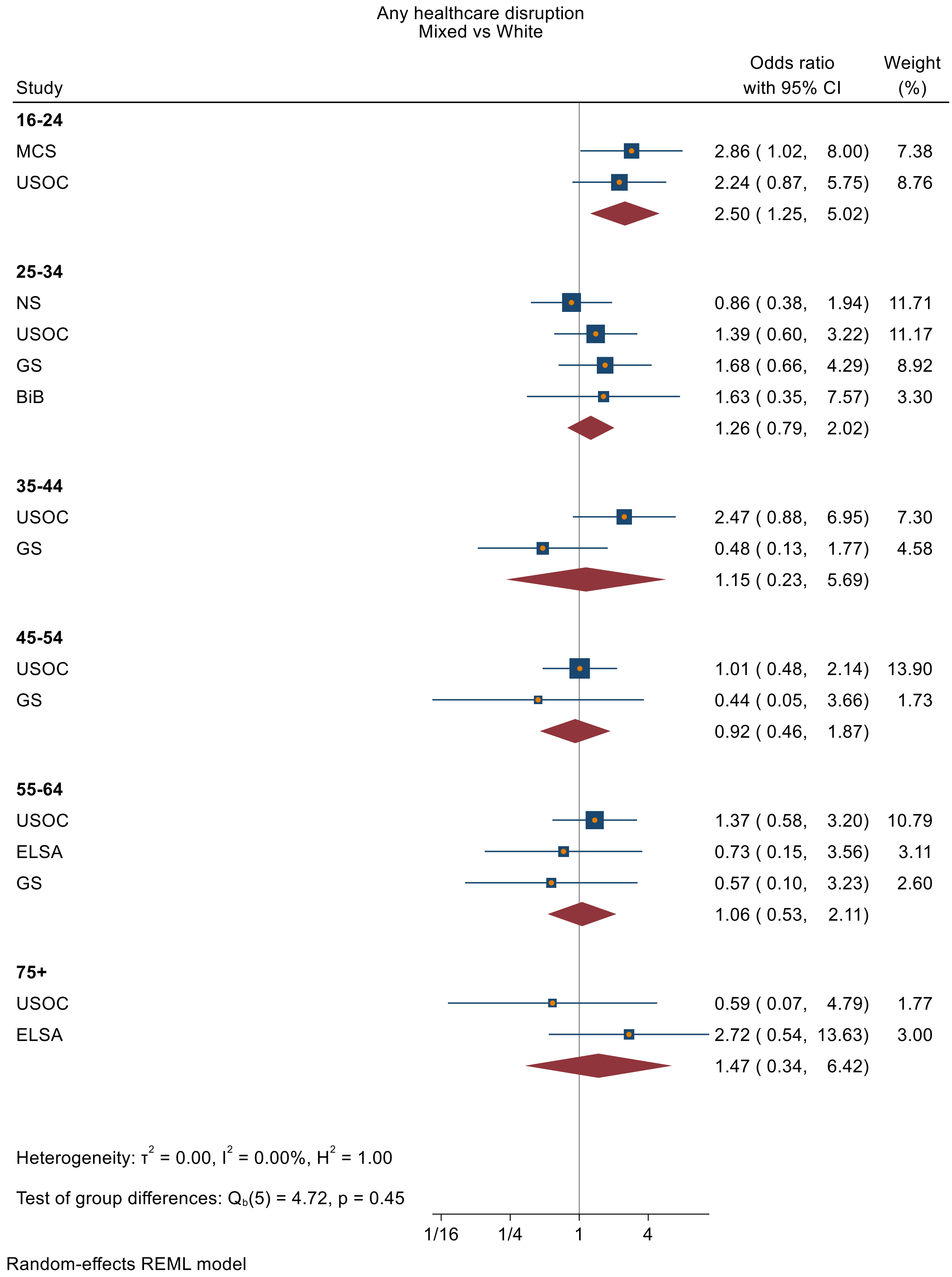

For peer review only - http://bmjopen.bmj.com/site/about/guidelines.xhtml

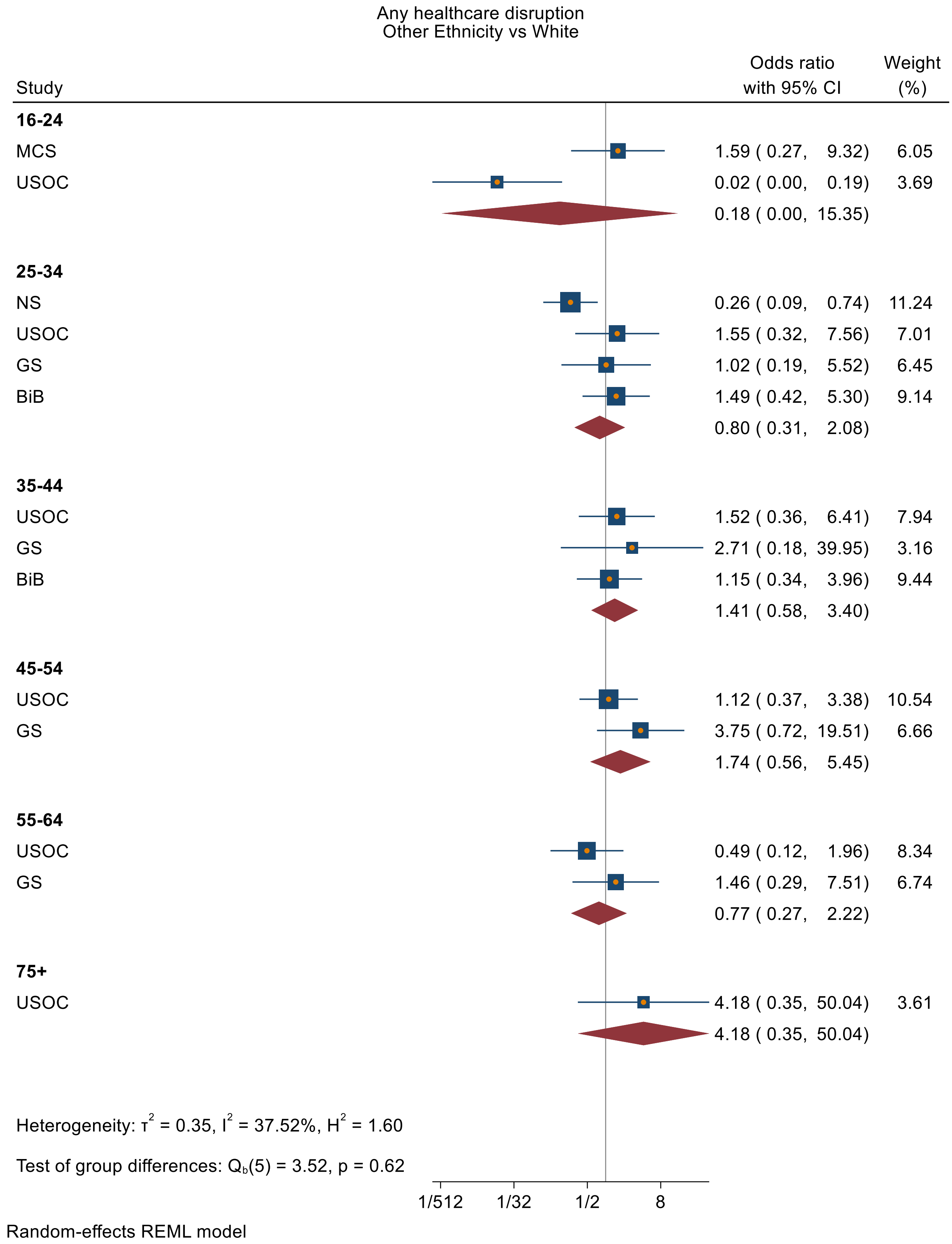
Any healthcare disruption
Other Ethnicity vs White
Study
Odds ratio with 95% CI
Weight (%)
16-24
MCS 1.59 (0.27, 9.32) 6.05
USOC 0.02 (0.00, 0.19) 3.69
0.18 (0.00, 15.35)
25-34
NS 0.26 (0.09, 0.74) 11.24
USOC 1.55 (0.32, 7.56) 7.01
GS 1.02 (0.19, 5.52) 6.45
BiB 1.49 (0.42, 5.30) 9.14
0.80 (0.31, 2.08)
35-44
USOC 1.52 (0.36, 6.41) 7.94
GS 2.71 (0.18, 39.95) 3.16
BiB 1.15 (0.34, 3.96) 9.44
1.41 (0.58, 3.40)
45-54
USOC 1.12 (0.37, 3.38) 10.54
GS 3.75 (0.72, 19.51) 6.66
1.74 (0.56, 5.45)
55-64
USOC 0.49 (0.12, 1.96) 8.34
GS 1.46 (0.29, 7.51) 6.74
0.77 (0.27, 2.22)
75+
USOC 4.18 (0.35, 50.04) 3.61
4.18 (0.35, 50.04)
Heterogeneity: τ² = 0.35, I² = 37.52%, H² = 1.60
Test of group differences: Q_b(5) = 3.52, p = 0.62
1/512
1/32
1/2
8
Random-effects REML model

STROBE Statement—Checklist of items that should be included in reports of ***cohort studies***

	Item No	Recommendation	Page No
**Title and abstract**	1	(*a*) Indicate the study's design with a commonly used term in the title or the abstract<br>(*b*) Provide in the abstract an informative and balanced summary of what was done and what was found	1-2
**Introduction**			
Background/rationale	2	Explain the scientific background and rationale for the investigation being reported	3-4
Objectives	3	State specific objectives, including any prespecified hypotheses	4
**Methods**			
Study design	4	Present key elements of study design early in the paper	4-5
Setting	5	Describe the setting, locations, and relevant dates, including periods of recruitment, exposure, follow-up, and data collection	4-5 & supplementary
Participants	6	(*a*) Give the eligibility criteria, and the sources and methods of selection of participants. Describe methods of follow-up<br>(*b*) For matched studies, give matching criteria and number of exposed and unexposed	4 & supplementary
Variables	7	Clearly define all outcomes, exposures, predictors, potential confounders, and effect modifiers. Give diagnostic criteria, if applicable	4-7 & supplementary
Data sources/ measurement	8*	For each variable of interest, give sources of data and details of methods of assessment (measurement). Describe comparability of assessment methods if there is more than one group	4-7 & supplementary
Bias	9	Describe any efforts to address potential sources of bias	4 & 8
Study size	10	Explain how the study size was arrived at	4 & supplementary
Quantitative variables	11	Explain how quantitative variables were handled in the analyses. If applicable, describe which groupings were chosen and why	7-8 & supplementary
Statistical methods	12	(*a*) Describe all statistical methods, including those used to control for confounding<br>(*b*) Describe any methods used to examine subgroups and interactions<br>(*c*) Explain how missing data were addressed<br>(*d*) If applicable, explain how loss to follow-up was addressed<br>(*e*) Describe any sensitivity analyses	7-8 & supplementary
**Results**			
Participants	13*	(a) Report numbers of individuals at each stage of study—eg numbers potentially eligible, examined for eligibility, confirmed eligible, included in the study, completing follow-up, and analysed<br>(b) Give reasons for non-participation at each stage<br>(c) Consider use of a flow diagram	9 & supplementary
Descriptive data	14*	(a) Give characteristics of study participants (eg demographic, clinical, social) and information on exposures and potential confounders<br>(b) Indicate number of participants with missing data for each variable of interest<br>(c) Summarise follow-up time (eg, average and total amount)	9 & supplementary
Outcome data	15*	Report numbers of outcome events or summary measures over time	9-12 & supplementary

For peer review only

Main results	16	(*a*) Give unadjusted estimates and, if applicable, confounder-adjusted estimates and their precision (eg, 95% confidence interval). Make clear which confounders were adjusted for and why they were included<br>(*b*) Report category boundaries when continuous variables were categorized<br>(*c*) If relevant, consider translating estimates of relative risk into absolute risk for a meaningful time period	9-12 & supplementary
Other analyses	17	Report other analyses done—eg analyses of subgroups and interactions, and sensitivity analyses	12& supplementary
**Discussion**			
Key results	18	Summarise key results with reference to study objectives	13
Limitations	19	Discuss limitations of the study, taking into account sources of potential bias or imprecision. Discuss both direction and magnitude of any potential bias	14-15
Interpretation	20	Give a cautious overall interpretation of results considering objectives, limitations, multiplicity of analyses, results from similar studies, and other relevant evidence	15-16
Generalisability	21	Discuss the generalisability (external validity) of the study results	14-15
**Other information**			
Funding	22	Give the source of funding and the role of the funders for the present study and, if applicable, for the original study on which the present article is based	17

*Give information separately for exposed and unexposed groups.

Note: An Explanation and Elaboration article discusses each checklist item and gives methodological background and published examples of transparent reporting. The STROBE checklist is best used in conjunction with this article (freely available on the Web sites of PLoS Medicine at http://www.plosmedicine.org/, Annals of Internal Medicine at http://www.annals.org/, and Epidemiology at http://www.epidem.com/). Information on the STROBE Initiative is available at http://www.strobe-statement.org.

For peer review only

For peer review only - http://bmjopen.bmj.com/site/about/guidelines.xhtml
